# Supplementation with small-extracellular vesicles from ovarian follicular fluid during in vitro production modulates bovine embryo development

**DOI:** 10.1371/journal.pone.0179451

**Published:** 2017-06-15

**Authors:** Juliano C. da Silveira, Gabriella M. Andrade, Maite del Collado, Rafael V. Sampaio, Juliano R. Sangalli, Luciano A. Silva, Fábio V. L. Pinaffi, Izabelle B. Jardim, Marcelo C. Cesar, Marcelo F. G. Nogueira, Aline S. M. Cesar, Luiz L. Coutinho, Rinaldo W. Pereira, Felipe Perecin, Flávio V. Meirelles

**Affiliations:** 1 Department of Veterinary Medicine, University of Sao Paulo, Pirassununga, Sao Paulo, Brazil; 2 Department of Biological Sciences, University of Sao Paulo State, Assis, Sao Paulo, Brazil; 3 Department of Animal Sciences, “Luiz de Queiroz” College of Agriculture, University of Sao Paulo, Piracicaba, São Paulo, Brazil; 4 Department of Biotechnology, Catholic University of Brasilia, Brasília, Brazil; University of Florida, UNITED STATES

## Abstract

Pregnancy success results from the interaction of multiple factors, among them are folliculogenesis and early embryonic development. Failure during these different processes can lead to difficulties in conception. Alternatives to overcome these problems are based on assisted reproductive techniques. Extracellular vesicles are cell-secreted vesicles present in different body fluids and contain bioactive materials, such as messenger RNA, microRNAs (miRNAs), and proteins. Thus, our hypothesis is that extracellular vesicles from follicular fluid from 3–6 mm ovarian follicles can modulate bovine embryo development in vitro. To test our hypothesis follicular fluid from bovine ovaries was aspirated and small-extracellular vesicles (<200 nm) were isolated for further analysis. Additionally, small-extracellular vesicles (EVs) were utilized for functional experiments investigating their role in modulating messenger RNA, microRNA as well as global DNA methylation and hydroxymethylation levels of bovine blastocysts. EVs from 3–6 mm follicles were used for RNA-seq and miRNA analysis. Functional annotation analysis of the EVs transcripts revealed messages related to chromatin remodeling and transcriptional regulation. EVs treatment during oocyte maturation and embryo development causes changes in blastocyst rates, as well as changes in the transcription levels of genes related to embryonic metabolism and development. Supplementation with EVs from 3–6 mm follicles during oocyte maturation and early embryo development (until the 4-cell stage) increased the levels of bta-miR-631 (enriched in EVs from 3–6 mm follicles) in embryos. Interestingly, the addition of EVs from 3–6 mm follicles induced changes in global DNA methylation and hydroxymethylation levels compared to embryos produced by the standard in vitro production system. Our results indicate that the supplementation of culture media with EVs isolated from the follicular fluid of 3–6 mm follicles during oocyte maturation and early embryo development can partially modify metabolic and developmental related genes as well as miRNA and global DNA methylation and hydroxymethylation, suggesting that EVs play an important role during oocyte maturation and early embryo development in vitro.

## Introduction

Pregnancy success depends on folliculogenesis, fertilization and an adequate uterine environment for embryonic development [1]. In general, early embryonic death can be related to perturbed RNA accumulation in the oocyte during follicular development, embryonic genome activation, and/or incorrect epigenetic reprogramming [2,3]. These problems also influence the success rate of embryo in vitro production (IVP), which are part of the assisted reproduction techniques (ART), leading to embryonic losses. ART approaches are continuously optimized to overcome many of these problems [4]. However, ART present their own problems, mainly because the in vitro environmental do not perfectly mimic conditions present in vivo [5].

Oocyte growth and follicle maturation are crucial for embryo production [3]. Bovine oocytes originating from small follicles (2–3 mm) and medium follicles (3–8 mm) are 110μm and 110–120μm in diameter; respectively, and have not completed their growth phase, which includes the accumulation of messenger RNA (mRNA), ribosomes, and polypeptides [3]. Interestingly, the oocyte continues to accumulate RNA transcripts until nine hours after the induction of meiosis resumption through RNA transfer from cumulus cells to the oocyte via transzonal projections [6,7]. Apart from accumulation of transcripts and proteins, the oocyte genome needs to acquire its imprinting signature, and to undergo DNA methylation, before it can be fertilized. Maternal acquisition of methylation appears to be gene specific, occurs at an oocyte size of ~110 μm, and the majority of the genes are fully methylated by the time its size reaches 120 μm [8]. Shortly after fertilization the bovine zygote genome undergoes DNA demethylation, followed by de novo DNA methylation, and then the embryo genome is activated at around the 8-16-cell stage [9]. However, during embryo production in vitro these processes are somehow defective, suggesting that factors necessary to proper embryo development are missing in the culture media.

Novel ART approaches are required in order to improve pregnancy development outcomes. Recently, much attention has been given to extracellular vesicles, which act as mediators of intercellular communication. Extracellular vesicles, called exosomes (50–150 nm) and microvesicles (100–1000 nm), are present in ovarian follicular fluid and oviductal fluids [10–12]. These cell-secreted vesicles contain bioactive components (i.e. proteins, lipids, mRNAs and miRNAs), which can be transferred among cells [13,14]. Extracellular vesicles secreted by donor cells can travel through body fluids without being degraded and deliver their contents to target cells leading to physiological responses in recipient cells. Extracellular vesicles described in equine, human, and bovine follicular fluid, are involved in follicle development and are associated with oocyte quality [10,15,16]. Additionally, a recent study suggested that extracellular vesicles from small antral follicles can modulate cumulus-oocyte-complex (COC) expansion in vitro, demonstrating that there is a physiological function for these vesicles during follicular growth and oocyte development [17]. Apart from the role of extracellular vesicles during follicle and oocyte development, these vesicles can also, originate from the oviduct and endometrium, mediating embryo-maternal communication, which illustrates their role during early embryo development [12,18]. Based on these observations and the in vitro production system, the extracellular vesicles from small ovarian follicles may be used as a supplement during bovine oocyte maturation and embryo development with the objective of improving embryo development.

Immature oocytes enclosed in growing ovarian follicles undergo mRNA accumulation and epigenetic maturation, and acquire the competence to carry on early embryo development until embryonic genome activation [3,8]. Furthermore, extracellular vesicles in follicular fluid can mediate intercellular communication and are possibly involved in regulating oocyte quality [10,16]. Our hypothesis is that small-extracellular vesicles from the follicular fluid of 3–6 mm ovarian follicles can modulate the development of bovine embryos produced in vitro. Therefore, aim of the present study was to determine the contents of small extracellular vesicles from 3-6mm follicles and the effects of EVs supplementation on blastocysts transcripts and global DNA methylation and Hydroxymethylation levels.

## Materials and methods

A series of experiments were carried out to identify and investigate the role of cell-secreted vesicles present in ovarian follicular fluid. To this end, follicular fluid and follicle contents were aspirated from bovine antral follicles, when their diameter was between 3–6 mm (post-mortem) as well as from pre-ovulatory follicles (in vivo), and used in different experiments. The experiments were performed using extracellular vesicles from 3-6mm (EVs3-6mm) and pre-ovulatory follicles (EVsPreOv) as well as extracellular vesicles-depleted Fetal Calf Serum (FCS) by ultracentrifugation at 100,000 xg for 12 h at 4°C and were termed EVsfree-FCS. Approval for this study was obtained from the University of São Paulo Research Ethics Committee (protocol number 14.1.688.74.1). All experiments were conducted in accordance with the International Guiding Principles for Biomedical Research Involving Animals (Society for the Study of Reproduction). Reagents and culture media used were purchased from Sigma-Aldrich Chemical Company (St. Louis, MO, USA) unless otherwise stated.

### Isolation of small-extracellular vesicles from bovine follicular fluid

Small-extracellular vesicles were isolated from follicular fluid collected from bovine ovarian follicles. To obtain follicular fluid from 3-6mm follicles ovaries from slaughterhouse were aspirated with an 18-gauge needle and fluid (without blood) was collected and pooled for extracellular vesicle isolation (EVs3-6mm). In addition to small follicles we collected follicular fluid from in vivo pre-ovulatory follicles (EVsPreOv), using cyclic Nellore cows, which had their ovarian follicular wave synchronized according to the following protocol. On the first day, Day 0, females received 50mg of cloprostenol (Sincrocio, OuroFino Saude Animal) and follicular wave synchronization was induced by follicular ablation, by follicle aspiration, associated with the administration of 2 mg of oestradiol benzoate (Sincrodiol, Ourofino Saude Animal) and intravaginal progesterone-releasing device (IPRD) (Sincrogest, OuroFino Saude Animal). On Day 8, females received 0.01 mg of buserelin acetate (GnRH analog, Sincroforte, OuroFino Saude Animal) and 25–26 hours later, the follicular fluids along with expanded COCs were recovered by ovum pick-up (OPU) of the pre-ovulatory follicle.

In order to isolate small-extracellular vesicles (<200 nm), we removed larger vesicles by standard differential centrifugation methods followed by ultracentrifugation with slight modifications [19]. Briefly, pooled follicular fluid (500–2000 μl) from ten ovarian pre-ovulatory follicles (from 5 cows) and follicles ranging between 3–6 mm in size (from 50 ovaries), were centrifuged at 300 xg for 10 min to remove live cells, at 2,000 xg for 10 min to remove residual cells and cell debris, and at 16,500 xg for 30 min to remove large microvesicles. The supernatant remaining was filtered through a 0.20 μm sterile syringe filter, to remove particles greater than 200 nm, and centrifuged at 100,000 xg for 70 min (Beckman 70TI rotor). Following centrifugation, the supernatant was discarded and phosphate buffered saline (PBS, pH 7.4) was added to wash the pellet, and the suspension was centrifuged one more time at 100,000 xg for 70 min to pellet the small-extracellular vesicles. All centrifugation steps were performed at 4°C. Follicular fluid EVs pellets were resuspended in 50 μL of PBS for characterization or mixed with EVsfree-FCS for embryo in vitro production experiments. For size determination experiments, samples were kept at 4°C until being analyzed using qNano (described below). Importantly, all the experiments were performed with follicular fluid obtained from the initial sample collection to minimize experimental variation. For protein and RNA isolation, samples were resuspended in 200 μL Trizol reagent (Molecular Research Center, Cincinnati, OH) and stored at -80°C until further use. For functional experiments, EVs were labeled with the green lipid dye PKH67 (described below), and used fresh without freezing.

### Small-extracellular vesicle size distribution and RNA contents

The isolated small-extracellular vesicles were analyzed using qNano (Izon Inc., Cambridge, MA). This technique allows for the assessment of vesicle size according to the pore of choice [20]. We utilized a pore size of 30–400 nm, since we filtered our samples in 0.2 μm sterile syringe filters, to allow the passage of vesicles of sizes within the pore range and to validate the presence of small-extracellular vesicles between 50–200 nm. For the analysis of EVs RNA yields and size, we used an Agilent 2100 Bioanalyzer with an RNA 6000 Nano and RNA 6000 Pico Kit. The protein concentrations were determined using the Bradford assay.

### Transmission electron microscopy

The small-extracellular vesicles obtained by differential centrifugation were processed for transmission electron microscopy (TEM) for morphological evaluation as described in Da Silveira et al. [10]. Briefly, the EVs (a total of three biological samples for each group) were fixed for 30 min at room temperature in 2.5% glutaraldehyde, 5% sucrose, and 0.1 M sodium cacodylate at pH 7.4. After fixation and staining pellets were embedded. Ultrathin 80nm sections were cut, mounted on 300-mesh nickel grids, stained with uranyl acetate and lead citrate, and examined using a MORGAGNI 268D ZEISS transmission electron microscope at 80 kV.

### PKH67 labeled small-extracellular vesicles and cell uptake

The EVs obtained by differential ultracentrifugation were collected from the follicular fluid of bovine ovarian follicles between 3–6 mm in size and were labeled with PKH67 (PKH67GL, Sigma-Aldrich, St. Louis, MO), a green fluorescent dye that labels lipid membranes as described in Da Silveira et al. [10]. This experiment was performed with granulosa cells, cumulus cells, cumulus-oocyte-complexes (24 h exposure), while embryos were exposed during IVC. Briefly, this preparation containing primarily EVs was incubated in 2 μL PKH67 (2 μM) for 30 min at room temperature, followed by incubation in 1% BSA for 10 min at room temperature, washed four times with medium to remove excess dye, and resuspended in cell culture medium depending on the experiment. For a negative control, sterile PBS was incubated with PKH67 and treated in the same manner as described above. The labeled EVs or PBS (negative control) were used to determine EVs uptake by granulosa or cumulus cells, cumulus-oocyte-complexes and embryos (described below).

Granulosa and COCs from bovine ovarian follicles between 3-6mm in size were separated from follicle contents using a stereomicroscope. A total of 5 x 10^5^ of granulosa cells and 15 COCs were cultured in 4-well plates in DMEM/F-12 (Gibco, Grand Island, US) without FCS according to Portela et al. [21]. After 24 h, the granulosa and COCs attached to the coverslip were fixed with 4% PFA, and the presence of fluorescent-labeled EVs in the cells was ascertained using a Zeiss Axioplan 2 fluorescence microscope with 100x or 40x objectives and Zeiss LSM710 with 63x objective. Experiments were replicated three times. In each replicate, labeled EVs isolated from follicular fluid of a different batch of ovaries were used. Labeled PBS was used as negative control.

### Functional experiments and in vitro production of blastocysts

Functional analysis of small-extracellular vesicles during oocyte maturation and embryo development were performed in vitro. The rationale behind this experiment was to investigate whether EVs containing messages involved in chromatin remodeling and mRNA translation control could modulate early embryo development. To this end, we supplemented COCs during in vitro maturation as well as early embryos with EVs. To perform EVs supplementation during oocyte maturation, small-extracellular vesicles were isolated from 1250 μL of the initially pooled follicular fluid from 3–6 mm or pre-ovulatory follicles using ultracentrifugation and diluted in 125 μL of extracellular vesicles-depleted FCS (EVsfree-FCS). Diluted vesicles were added to 1125 μL of B199 maturation media, thus maintaining the 10% concentration of FCS and 10 x concentrated EVs. To perform EVs supplementation during embryo development (days 0–7) small-extracellular vesicles were isolated from 1000μL of follicular fluid from 3–6 mm or pre-ovulatory follicles using ultracentrifugation and diluted in 25 μL of FCS depleted of extracellular vesicles. Diluted vesicles were added to 975 μL of SOFaac culture media, thus maintaining the 2.5% concentration of FCS and 10 x concentrated EVs.

Initially, we evaluated the blastocyst rates by comparing control (FCS) and EVsfree-FCS media. In this experiment, we performed five in vitro production routines and evaluated a total of 586 COCs. We did not observe difference in cleavage or blastocyst rates (p>0.05) between embryos produced in control or in EVsfree-FCS media, suggesting that removal of extracellular vesicles from FCS does not affect the production rates (S1 Fig). For bovine in vitro embryo production (IVP), oocytes were obtained by post mortem follicular aspiration. The IVP routines were composed by a single drop of each treatment containing a total of 20 to 25 grade I and II oocytes (good quality oocytes). Oocytes were matured in 90 μL drops of maturation medium (TCM-199; Gibco, Grand Island, US) buffered with bicarbonate and supplemented with 0.2 mM sodium pyruvate, 0.5 μg/mL of follicle stimulating hormone (FSH; Folltropin-V, Bioniche Animal Health Belleville, Canada), 5 U/mL of human Chorionic Gonadotropin (hCG), 50 μg/mL of gentamycin and 10% of exosomes-depleted fetal calf serum (EVsfree-FCS) under mineral oil at 38.5°C and in atmosphere with 5% CO_2_. For in vitro maturation experiments, oocytes were matured in media containing: EVs-depleted fetal calf serum (EVsfree-FCS), or EVsfree-FCS with the addition of EVs from 3–6 mm (EVs3-6mm), or pre-ovulatory follicles (EVsPreOv), obtained in vivo by OPU as previously described.

Following 22–24 h of in vitro maturation (IVM), matured oocytes from the different experimental groups were used for IVF in order to produce blastocysts from the different maturation media. For in vitro fertilization (IVF), semen from one collection batch of the same bull was used and distributed among treatment groups (EVsfree-FCS, EVs3-6mm and EVsPreOv). The IVF was performed in Tyrode/albumin/sodium lactate/sodium pyruvate (IVF-TALP) medium supplemented with 0.6% BSA. Subsequently, presumptive zygotes from all experimental groups were placed in in vitro cultures (IVCs) for 7 days in synthetic oviduct fluid with aminoacids, sodium citrate and myo-inositol (SOFaac) medium [22] supplemented with 6 mg/mL BSA-FAF, 0.2 mM sodium pyruvate, 50 μg/mL gentamicin, and 2.5% EVsfree-FCS at 38,5°C in a controlled atmosphere of 5% CO2. The FCS was supplemented according to the group analyzed and added to the IVC media: EVsfree-FCS, EVs3-6mm, and EVsPreOv groups. All the experiments involving EVs supplementation were performed using the described protocol. Samples were collected for RNA and immunofluorescence analysis always combining embryos from different IVP routines. Additionally, blastocysts as well as 4-cell embryos (day 2 after IVF) were pooled (n = 5) prior to RNA analysis.

### Western blot analysis

Proteins from follicular fluid, EVs and follicular cells were isolated using TRI Reagent^®^BD (Molecular Research Center, Cincinnati, OH) according to the manufacturer’s instructions and resuspended in 8 M urea. Briefly, the organic phase of the Tri Reagent lysis was mixed with 100% ethanol in a 1:1 ratio and centrifuged at 2,000 xg for 5min to precipitate the DNA. Next, the supernatant was mixed with isopropanol in a 1:2 ratio and incubated during 10 min at room temperature prior to centrifugation at 12,000 xg for 10 min. The protein pellet was washed three times with 0.3M guanidine hydrochloride in 95% ethanol. Next, protein pellet was dehydrated in 500 μL 100% Ethanol and centrifuged at 12,000 xg for 5 min. After air-drying, the pellet was resuspended in 20 μL of 8M urea. Protein concentrations were determined using the Bradford assay. A total of 15 μg of protein were loaded and resolved in 12% SDS-PAGE polyacrylamide gels (Bio-Rad, Hercules, CA, USA). Protein samples were run at 200 V for 45 min and transferred to nitrocellulose membranes (Biotrace NT, Pall life Sciences, Pensacolla, FL, USA) for 30 min at 25 V in a semidry transfer apparatus. The membranes were incubated in blocking buffer (5% BSA in TBST) for 1 h at room temperature. Subsequently, the presence of Programed cell death 6-interacting protein (ALIX) was investigated by exposing membranes to a goat polyclonal antibody raised against a peptide mapping at the N-terminus of ALIX of human origin (0.5 μg/mL, SC-49267, Santa Cruz, CA, USA) overnight at 4°C. The presence of CD63 was investigated by exposing membranes to a rabbit polyclonal antibody raised against a human CD63 (0.4 μg/mL, SC-15363, Santa Cruz, CA, USA) overnight at 4°C. The presence of Polyadenylate-binding protein (PABP) and Cytochrome C (CYT C) were investigated by exposing membranes to a goat polyclonal antibody raised against a human PABP (0.4 μg/mL, SC-18611, Santa Cruz, CA, USA) and human CYT C (0.5 μg/mL, SC-8385, Santa Cruz, CA, USA) overnight at 4°C. Membranes were washed three times in 1x TBST for 5 min, and incubated for 1 h at room temperature with a horseradish peroxidase conjugated anti-rabbit secondary antibody (1:3000, #7074S, Cell Signaling Technology, Danvers, MA, USA) and anti-goat secondary antibody (0.2 μg/mL, SC-2020, Santa Cruz, CA, USA). The presence of ACTB was assessed by exposing the membranes to a monoclonal anti-β-actin antibody (5^−^4 μg/mL, A3854, Sigma-Aldrich) for 3 h at room temperature. Membranes were washed three times in 1x TBST for 5 min and incubated for 5 min in ECL Plus Prime Western Blotting Detection System solution (Amersham^™^, Buckinghamshire, UK) for color development, and image and band analyses were performed using ChemiDoc MP Image System (Bio-Rad, Hercules, CA, USA).

### RNA extraction

Total RNA was extracted from EVs, 4-cell embryos and blastocysts (EVsfree-FCS, EVs3-6mm and EVsPreOv) using Trizol^®^ reagent (Molecular Research Center, Cincinnati, OH) according to the manufacturer’s instructions with modifications, such as the use of a small volume of Trizol (200 μL) and chloroform (128 μL), followed by purification of the aqueous phase in miRNeasy (Qiagen, #217084, Venlo, Limburg, Netherlands). RNA was dissolved in 10 μL of RNase free water and treated with DNaseI (Invitrogen, Carlsbad, CA) RNA concentration was measured with A260 measurements using NanoDrop 2000 (Thermo Scientific, Carlsbad, CA).

### RNA-seq library preparation and data analysis

A total of 100 ng of total RNA from a pool of EVs originating a single pool from 50 ovarian follicles between 3–6 mm in size was used for library preparation according to the protocol described in the TruSeq RNA Sample Preparation kit v2 guide (Illumina, San Diego, CA). It is important to mention that extracellular vesicles do not contain any ribosomal RNA, thus allowing us to skip the ribosomal depletion step. The libraries’ average sizes were estimated using the Agilent Bioanalyzer 2100 (Agilent, Santa Clara, CA, USA), and quantified using quantitative PCR with the KAPA Library Quantification kit (KAPA Biosystems, Foster City, CA, USA). A single lane of a sequencing flowcell, using the TruSeq PE Cluster kit v3-cBot-HS kit (Illumina, San Diego, CA, USA), was sequenced using HiScanSQ equipment (Illumina, San Diego, CA, USA) with a TruSeq SBS Kit v3-HS (200 cycles), according to the manufacturer’s instructions. The main goal of the application of the RNA-Seq approach in this study was to identify RNA molecules present within EVs, for this reason we did not apply trimming of low quality bases. This approach was performed due to important RNA-Seq analyses demonstrating that an incorrect trimming of quality-bases has a great impact on gene expression estimates from RNA-Seq data [23]. RNA-seq data was analyzed using the Discovery Environment platform on iPlant’s cyber infrastructure (http://www.iplantcollaborative.org) according to Tuxedo 2 pipeline [24]. Initially, the sequencing adaptors and low-complexity reads were removed in an initial data filtering step, and the quality of reads was estimated with the FASTQC program [25]. The alignment of the reads against UMD3.1 *Bos Taurus* reference genome (http://ensembl.org/Bos_taurus/Info/Index/) was performed by Tophat v2.0.9 software. Finally, the Cufflinks program was used to assemble the transcriptomes and to quantify the gene expression levels. Functional annotation was performed using the Database for Annotation, Visualization and Integrated Discovery (DAVID) v6.7 (https://david.ncifcrf.gov) [26]. Sequence data have been deposited in the GEO database (GSE96832).

### Messenger RNA and microRNA reverse transcription

Quantifiable, reverse-transcribed mRNA was generated using the High-Capacity cDNA Reverse Transcription Kit (Applied Biosystems, Foster City, CA, USA), according to the manufacturer’s protocol. Briefly, the reverse transcription reaction was carried out with approximately 15 ng of total RNA for each selected gene. RNA was incubated with 10x RT Buffer, 25x dNTP Mix (100mM), 10x RT random primers, nuclease-free water, MultiScribe^™^ reverse transcriptase at 25°C for 10 min, 37°C for 2 h followed by 5 min at 85°C.

Reverse transcription of miRNAs was generated using the miScript PCR System (Qiagen^®^ #218193, Venlo, Limburg, Netherlands) according to the manufacturer’s instructions. Briefly, the reverse transcription reaction was carried out with approximately 200 ng of total RNA. Total RNA, including the small RNA fraction, was incubated with 5x miScript HiFlex Buffer, 10x miScript Nucleic mix, RNase-free water, and miScript reverse transcriptase at 37°C for 60 min, followed by 5 min at 95°C.

### Real-time PCR expression analysis of mRNAs and miRNAs

Relative levels of mRNAs were examined in embryos at the blastocyst stage (n = 5 pools from five embryos from EVsfree-FCS, EVs3-6mm and EVsPreOv treatments) and EVs (n = 3 pools of follicular fluid retrieved from 10 follicles between 3–6 mm in size or from pre-ovulatory). RNAseq mRNA validation analysis were performed in polls of EVs3-6mm in size (n = 8). Each analysis was performed in 10 μL reactions containing Power SYBR Green PCR Master Mix (2x) (Thermo Fisher Scientific, Grand Island, NY, USA), 0.5 μM of forward and reverse primers, and 1 μL cDNA. Gene specific primers were designed using Primer3—BioTools software and are presented in Table 1. The PCR cycle conditions were as followed: 95°C for 10 min, 45 cycles of 95°C for 15 s and 60°C for 60 s followed by melt curve analysis to confirm amplification of single cDNA products. To identify differences in mRNA levels in pools of blastocysts treated with exosomes, or without exosomes, as well as EVs raw Ct values were normalized to the geometrical mean of two internal controls (*PPIA* and *GAPDH*) [27].

**Table 1:** Primer sequences of genes analyzed by real-time PCR.

**Gene symbol**	**Definition**	**Sequence 5’- 3’**	**Accession Number**	**Annealing Temperature °C**
PPIA	peptidylprolyl isomerase A (cyclophilin A)	F:GGTCCTGGCATCTTGTCCAT R:TGCCATCCAACCACTCAGTCT	XM_010804358.2	60
TET1	Ten-eleven translocation methylcytosine dioxygenase 1	F:TGCCTACTTGCAACTGTCTTGATC R:TCTATCCTTACTGCATTTCCTTTTTG	XM_015469834.1	60
DNMT1	DNA methylatransferase 1	F:GCAGTACCAGCCCATCCT R:GCGGGCAGCCACCAA	XM_015471997.1	60
DNMT3A	DNA methylatransferase 3A	F:GCTCATGTGTGGGAACAACAATT R:CACCAAGAGATCCACACATTCCA	NM_001206502.1	60
HDAC2	Histone deacetylase 2	F:AGTGTGGTGCAGACTCCCTA R:TTGTGTATCCACCTCCCCCA	NM_001075146.1	60
EHMT2	Histone-lysine N-methyltransferase 2	F:TCAGGCCCCAGTGAGTACAT R:GTGTCATTGGACACCCCAGC	NM_001206263.1	60
EIF4B	Eukariotic translation initiation factor 4B	F:ACGACTCCAGATCTGCACCTG R:TCTTCACCGTCAATGGCGAGA	XM_005206207.1	60
EIF4E	Eukariotic translation initiation factor 4E	F:TTAATGCCTGGCTGTGACTAC R:ACGATCGAGGTCACTTCGTCT	NM_174310.3	60
**Gene symbol**	**Definition**	**Function**	**TaqMan**	
PPIA	peptidylprolyl isomerase A (cyclophilin A)	Housekeeping	Bt03224615_g1	
CDH1	cadherin 1	Apoptosis	Bt03210097_m1	
ACSL6	acyl-CoA synthetase long-chain family member 6	Metabolism	Bt03231692_m1	
FADS2	fatty acid desaturase 2	Metabolism	Bt03256253_g1	
REST	RE1-silencing transcription factor	Pluripotency	Bt03264679_m1	

Microfluidic gene expression analysis in embryos (n = 5 pools from five embryos from EVsfree-FCS, EVs3-6mm and EVsPreOv treatments) was performed using custom TaqMan assays (20x, Applied Biosystems), specific for the *Bos taurus* species. The abundance of the mRNAs of four genes was analyzed, as indicated in Table 1, according to different functional categories. Prior to qPCR thermal cycling, each sample was submitted to a sequence-specific pre-amplification process as follows: 1.25 μL assay mix (TaqMan assay was pooled to a final concentration of 0.2x for each of the 47 assays), 2.5 μL TaqMan PreAmp Master Mix (Applied Biosystems) and 1.25 μL of blastocysts cDNA. The reactions were activated at 95°C for 10 min followed by denaturing at 95°C for 15 s, and annealing and amplification at 60°C for 4 min for 14 cycles. These pre-amplified products were diluted five-fold prior to RT-qPCR analysis with TaqMan Universal PCR Master Mix (2x, Applied Biosystems) and inventoried TaqMan assays (20x, Applied Biosystem) in the 96.96 Dynamic Array^™^ Integrated Fluidic Circuits on Biomark HD System (Fluidigm, South San Francisco, CA, USA) using the TaqMan GE 96x96 Standard protocol, according to the manufacturer’s instructions. Analysis was performed in duplicate and Ct values were calculated using the system’s software (Biomark Real-time PCR Analysis, Fluidigm). Cyclophilin-A (*PPIA*) was selected as the most stable housekeeping gene. The relative expression values for each gene were calculated using the ΔCt method [28].

Relative levels of 348 bovine mature miRNAs and one housekeeping (miR-99b) were examined in EVs3-6mm preparations isolated from pooled follicular fluid (n = 3) and in 4-cell embryos (n = 5, in EVsfree-FCS, EVs3-6mm), using “Bovine Profiler plates” designed using mature miRNA sequences downloaded from mirBase database (http://www.mirbase.org). Each analysis was performed in 10 μL reactions containing 2x Quantitec SYBR Green (Qiagen, Venlo, Limburg, Netherlands), 10 μM universal reverse primer (Qiagen, Venlo, Limburg, Netherlands) and miRNA specific forward primer, and 0.03 μL of 1:4 diluted cDNA. Real-time PCR was conducted using the QuantStudio 6 Real Time PCR (Life Technologies) in 96-well plates. Internal controls were established to evaluate changes within the plates. The PCR cycle conditions were as followed: 95°C for 15 min, 45 cycles of 94°C for 10 s, 55°C for 30 s, and 70°C for 30 s followed by a melt curve analysis to confirm amplification of single cDNA products. To identify the abundancy of miRNAs isolated from EVs3-6mm raw Ct values were normalized to miR-99b as previously used in extracellular vesicles from ovarian follicular fluid [29,30]. Selected miRNAs were analyzed using DIANA TOOLS pathways package as a group, since we were interested in pathways regulated by these miRNA groups.

### DNA methylation and DNA hydroxymethylation analysis

Changes in global DNA methylation and hydroxymethylation were evaluated in blastocysts by immunofluorescence to detect 5-methylcytosine (5mC) and 5-hydroxymethylcytosine (5hmC) according to the protocol previously described in mice [31]. Briefly, oocytes were collected and assigned to treatment groups (control, EVsfree-FCS and EVs3-6mm) upon maturation, and blastocysts (7dpi) were generated by IVF using semen from the same bull and distributed among treatment groups upon embryo development. Following blastocyst development, embryos were collected and washed with PBS to remove culture medium, then fixed in 4% paraformaldehyde for 20 min and stored in PBS with 0.1% PVP at 4°C for future analysis. Embryos were permeabilized by incubation in PBS with 1% Trition X-100 at room temperature for 15 min, incubated for 12 min in 4 N HCl for DNA denaturation, then neutralized in 100 mM Tris-HCl (pH 8.5) for 20 min. Embryos were blocked in PBS with 1% BSA for 30 min at room temperature. Next embryos were incubated with mouse-anti-5-mC specific antibodies (clone 33D3, # sc-56615, Santa Cruz, Santa Cruz, CA, USA) and rabbit-anti-5-hmC (polyclonal, # AP9160a; Abgent—San Diego, CA, USA), diluted at a concentration of 1:300 in PBS overnight at 4°C. After 2 h washing in 0.1% Triton X-100, embryos were incubated with secondary antibody goat/anti-Mouse IgG-AlexaFluor 488 (# 11008, Invitrogen—Foster City, CA, USA), and goat/anti-rabbit IgG-Texas Red (# 111 075 144, Jackson ImmunoResearch, West Grove, PA, USA), diluted at a concentration of 1:400 in PBS for 1 h at room temperature. Negative controls consisted of embryos incubated only with PBS and lacking a primary antibody.

Embryos were analyzed by confocal microscopy using a LSM 710 (Zeiss, Germany). All images were captured under the same parameters, performing sequential acquisitions. For the visualization of methylation excitation and emission, it was set to 488 nm and 516 nm, respectively. For hydroxymethylation excitation and emission, it was set at 543 nm and 574 nm, respectively. Confocal images of three embryos per treatment group were captured under a 63x objective with oil. Four images in different points of the embryo were used for analysis; five blastomeres were analyzed in each image with the ImageJ program (NIH; http://rsb.info.nih.gov/ij/), with software assignment of intensity values between 0 and 255 for each pixel.

### Statistical analysis

To assess statistical differences, blastocyst rates were arc-sin transformed and ANOVA was performed considering a 3 x 3 factorial arrangement. Variables considered in the model were IVM and IVC supplementation (each of them with three levels: EVsfree-FCS, EVs3-6mm or EVsPreOv), and interaction. Data from DNA methylation and hydroxymethylation were also analyzed by ANOVA considering a 3 x 3 factorial arrangement. Variables were IVM and IVC supplementation (with three levels each: Control, EVsfree-FCS or EVs3-6mm), and interaction. To assess changes in transcript levels ΔCt levels were analyzed by ANOVA, the same treatments were performed during IVM and IVC and the variables were EVsfree-FCS, EVs-3-6mm or EVsPreOv. Means were compared by Tukey’s test. The equality of the variances was tested using the Levene’s test and normality was assessed with the Shapiro-Wilk test. For mir-631 analysis and transcript levels in small extracellular vesicles we used Student’s T-test. A p-value of ≤ 0.05 was considered significant. Analyses were conducted using SAS software.

## Results

### Characterization of small-extracellular vesicles from 3–6 mm ovarian follicles

**Fig 1:**
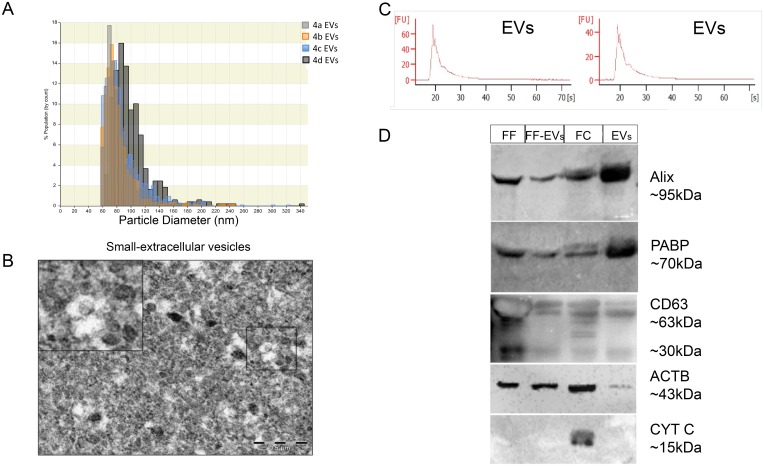
Characterization of small-extracellular vesicles (EVs) from 3–6 mm ovarian follicles. (A) The size of the EVs isolated from a pooled follicular fluid was evaluated by blockage of electrical current analysis, which demonstrated the presence of small size vesicles between 30-200nm. Different colors represent different replicates. (B) Transmission electron microscopy of isolated EVs demonstrated the presence of a homogenous population of small vesicles at 80kV (Scale bar 500nm). (C) EVs-RNA size distribution according to electropherogram of RNA molecules present in EVs from follicular fluid demonstrated the absence of 18s and 28s bands, as well as the presence of small RNA molecules. (D) Immunoblotting analysis of proteins in EVs and follicular cells. Herein we identified the presence of ALIX and CD63 two EVs markers, present in follicular fluid (FF), FF without EVs (FF-EV), follicular cells (FC), and EVs, isolated from 3-6mm ovarian follicles. We also identified the presence of PABP, an RNA binding protein, in FF, FF without EVs, follicular cells, and EVs. ACTB, a structural protein, was determined in the preparations, and demonstrated not to be enriched in EVs. Additionally, we verified the presence of Cytochrome C (CYT C), a mitochondrial protein, which serves as a negative control for cell contamination. EVs = extracellular vesicles <200 nm; ALIX = Programmed cell death 6-interacting protein; CD63 = CD63 molecule; PABP = Polyadenylate-binding protein 1; ACTB = Beta-actin; CYT C = Cytochrome c-1.

The EVs were isolated from follicular fluid and analyzed in different experiments to characterize and demonstrate the purity of the samples. Firstly, we qNano (Izon) analysis demonstrated the presence of small size extracellular vesicles derived from follicular fluid of 3-6mm bovine follicles, with diameters between 30–200 nm (Fig 1A). Secondly, TEM was used to identify precipitated vesicles from follicular fluid. This analysis revealed the presence of small-extracellular vesicles based on size (Fig 1B). Additionally, total RNA present in EVs exhibited an expected RNA size distribution (enriched in small non-coding RNAs) according to bioanalyzer histograms (Fig 1C). Lastly, we analyzed the presence of ALIX, CD63, ACTB, PABP and CYT C in follicular fluid, follicular fluid without EVs, EVs and follicular cells (Fig 1D). Based on Western blot analysis, we were able to demonstrate that follicular fluid, follicular fluid without EVs, EVs and follicular cells are positive for ALIX, CD63 and ACTB. Furthermore, we identified PABP, a mRNA stabilizing protein important for mRNA translation, within extracellular vesicles (Fig 1D). Additionally, the presence of CYT C, an indicator of cells presence, was only detected in follicular cells, indicating the absence of cells in follicular fluid and EVs.

### RNA content of small-extracellular vesicles isolated from bovine ovarian follicular fluid

**Fig 2:**
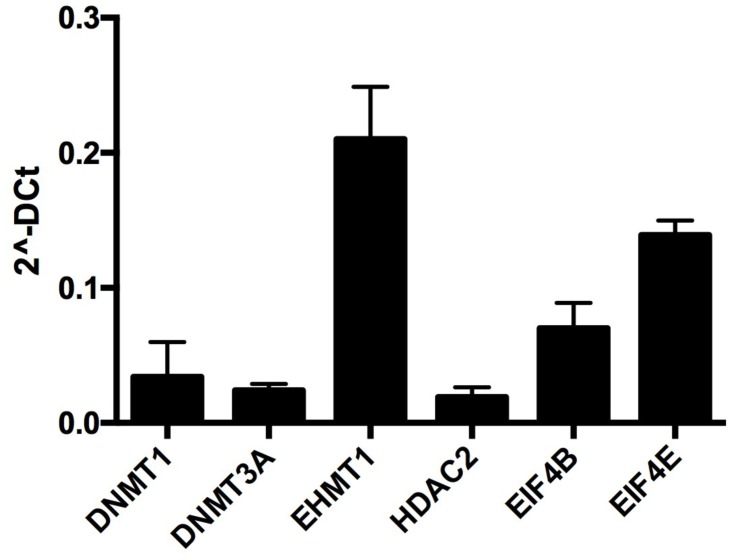
Presence of selected mRNAs involved in epigenetic chromatin modification and mRNA translation in small-extracellular vesicles from 3–6 mm ovarian follicles (EVs3-6mm). *DNMT1*, *DNMT3A*, *EHMT1* and *HDAC2* are genes involved in epigenetic chromatin modifications, while *EIF4B* and *EIF4E* are involved in mRNA stabilization during translation. Data are presented as mean of 2^^-ΔCt^ ± s.e.m.

In order to identify the transcripts, present in small-extracellular vesicles isolated from a pooled follicular fluid recovered from 3–6 mm bovine ovarian follicles (50 follicles), we performed a single set of RNA-seq analysis. RNA-seq results demonstrated a variety of coding and non-coding RNA molecules present in EVs (S1 Table). RNAseq analysis demonstrated a total of 11970875 reads and 8177304 (68.31%) pairs were aligned to the bovine genome. According to functional annotation analysis of the messages contained in EVs include nuclear reprogramming, mRNA translation, and chromatin remodeling, which is an important event occurring during this period of follicular growth (Table 2). To investigate the levels of mRNA levels of four major enzymes related to DNA methylation (*DNMT1*, *DNMT3A*, *EHMT1 and HDAC2)* and two mRNA binding proteins involved in transcript translation (*EIF4b* and *EIF4e*) in EVs (n = 8) isolated from the follicular fluid of ovarian follicles. Real-time PCR analysis revealed the presence of the investigated transcripts *DNMT1*, *DNMT3A*, *EHMT1*, *HDAC2*, *EIF4B* and *EIF4E* in EVs3-6mm (Fig 2).

**Table 2:** Functional annotation analysis of transcript from EVs3-6mm. Functional annotation demonstrated the presence of clusters involved in intracellular functions. FDR = false discovery rate.

Clusters	Functional Annotation	FDR
Cluster 1	nucleosome	6.90E-04
	protein-DNA complex	1.40E-03
	nucleosome core	1.60E-03
	chromosomal protein	6.60E-03
	chromatin	1.10E-02
	Histone core	8.50E-02
Cluster 2	DNA binding	2.10E-01
	dna-binding nucleus	5.00E+00
		8.90E+01
Cluster 3	Basal cell carcinoma	4.70E-01
	Wnt receptor signaling pathway, calcium modulating pathway	5.10E+00
	Wnt signaling pathway	5.30E+00
	Melanogenesis	6.70E+00
	Hedgehog signaling pathway	1.30E+01
	Wnt receptor signaling pathway	3.40E+01
Cluster 4	transcription regulator activity	3.40E+00
	sequence-specific DNA binding	4.20E+00
	regulation of transcription	2.10E+01
	transcription factor activity	4.90E+01
	regulation of RNA metabolic process	6.70E+01
	regulation of transcription, DNA-dependent	7.80E+01
Cluster 5	structural molecule activity	3.20E+00
	ribosome	8.00E+00
	ribosomal protein	1.10E+01
	ribonucleoprotein	1.80E+01
	ribosomal subunit	2.00E+01
	Ribosome	2.00E+01

### MiRNA profile of small-extracellular vesicles isolated from bovine ovarian follicular fluid

In addition to mRNA we identified the miRNA profile of EVs isolated from 3-6mm follicles (n = 3). In this experiment we identified 280 EVs miRNAs by qRT-PCR that exhibited a normal amplification curve and melting peak, and were detected under 35 Ct according to da Silveira [30] (S2 Table). We have identified 13 highly abundant miRNAs with Ct numbers between 10–25 Ct (bta-miR-615, bta-miR-27a-5p, bta-miR-631, bta-miR-323, bta-miR-574, bta-miR-767, bta-miR-320a, bta-miR-411b, bta-miR-421, bta-miR-99b, bta-miR-382, bta-miR-669 and bta-miR-760-5p) (Table 3). Based on the miRNA levels we selected the 13 highly abundant miRNAs and performed bioinformatics analysis using mirpath tool from DIANA DNA LAB software (http://diana.imis.athena-innovation.gr) [32]. Bioinformatics analysis predicted that the 13 highly abundant miRNAs were involved in the regulation of 25 cellular pathways (S3 Table). Among the 25 pathways, six are related to follicle development and function (Table 3), ECM-receptor interaction (12 genes), Pi3K-AKT signaling pathway (33 genes), focal adhesion (22 genes), ErbB signaling pathway (12 genes), gap junction (8 genes) and insulin signaling pathway (13 genes). Additionally, further analysis of the 13 highly abundant miRNAs, identified 13 validated target genes, according to mirTarbase (http://mirtarbase.mbc.nctu.edu.tw) [33] in humans or mice (Table 3).

**Table 3:** MiRNAs levels in small extracellular vesicles and bioinformatics analysis of predicted pathways. MiRNAs detected at high levels in small-extracellular vesicles (EVs) from 3–6 mm follicles and validated targets according to mirTarbase analysis. Bottom table presents top pathways predicted as regulated by selected miRNAs.

**miRNA**	**Average Ct Value**	**STDEV**	**Validated Target Genes**
bta-miR-615	11.62	±0.18	**IGF2**
bta-miR-27a-5p	15.90	±1.75	Only Predicted
bta-miR-631	21.12	±1.38	Only Predicted
bta-miR-323	22.01	±1.90	Only Predicted
bta-miR-574	22.79	±2.66	**FOXN3**
bta-miR-767	23.13	±7.35	**COL3A1, COL4A1, COL5A2, LOX**
bta-miR-320a	23.22	±0.78	**MCL1, NPR1, MAPK1**
bta-miR-411b	23.54	±13.35	Only Predicted
bta-miR-421	23.90	±5.51	**SMAD4**
bta-miR-99b	25.34	±0.71	**MTOR**, **RAVER2**
bta-miR-382	25.62	±5.21	**PTEN**
bta-miR-669	25.67	±4.00	Only Predicted
bta-miR-760-5p	26.00	±0.96	Only Predicted
**KEGG pathway**	**p-value**	**#genes**	**#miRNAs**
ECM-receptor interaction	1.95E-13	12	4
PI3K-Akt signaling pathway	1.37E-05	33	5
Focal adhesion	1.66E-04	22	5
ErbB signaling pathway	6.50E-04	12	3
Gap junction	0.0027	8	2
Insulin signaling pathway	0.0266	13	4

### Uptake of small-extracellular vesicles by granulosa cells, cumulus cells and embryos

**Fig 3:**
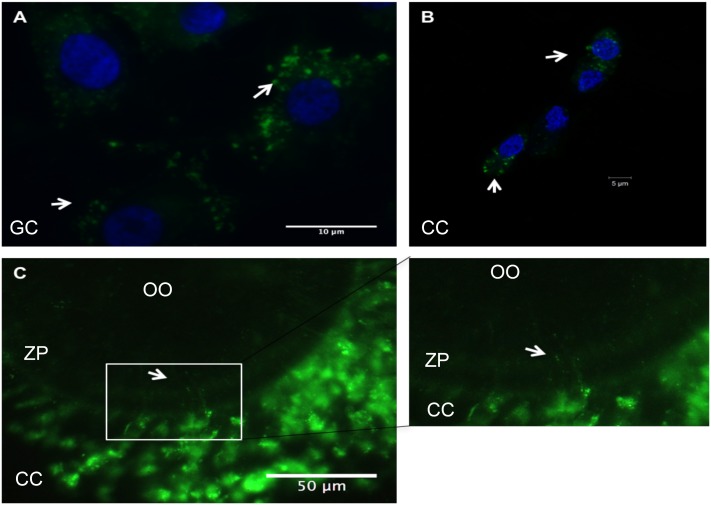
Uptake of PKH67 labeled small-extracellular vesicles (EVs) by follicular cells. (A) Granulosa cells exposed to EVs labeled with PKH67 (green) for 24h *in vitro*, granulosa cell nucleus (blue) with a 40x magnification. (B) Cumulus cells exposed to PKH67 labeled EVs for 9h during maturation in vitro with a 63X magnification. (C) Intact cumulus-oocyte-complex exposed to PKH67 labeled EVs for 9h during maturation in vitro with 100x magnification. Uptake of PKH67 labeled EVs by the different follicular cells (arrows). The enlarged white box shows the presence of EVs (green dots) inside the zona pellucida. GC = granulosa cells; CC = cumulus cells; OO = oocyte; ZP = zona pellucida.

**Fig 4:**
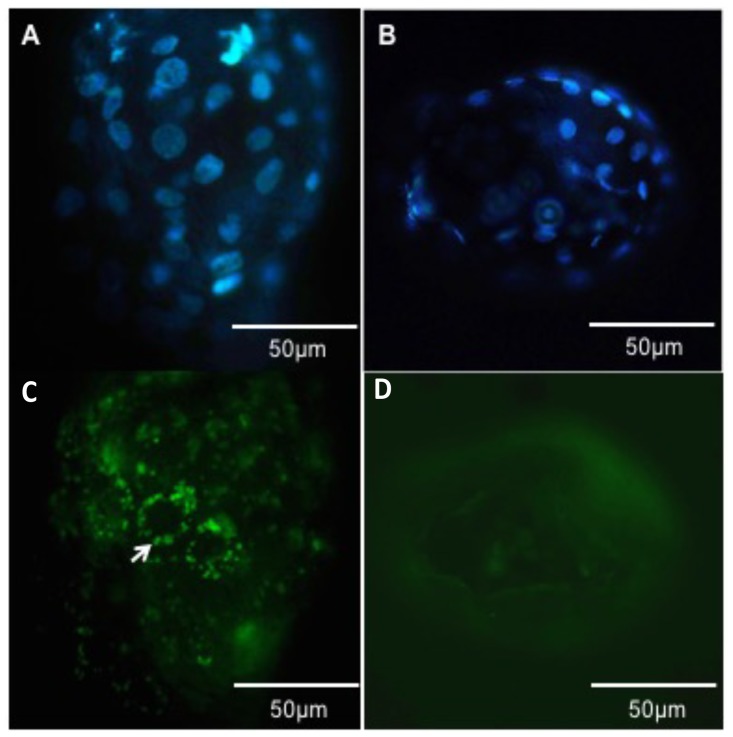
The small-extracellular vesicles (EVs) uptake by an embryo in in vitro culture. (A) Blastocysts stained with DAPI (nuclear staining) with a 40x magnification. (B) Blastocysts stained with DAPI (nuclear staining) with a 40x magnification. C) Blastocysts exposed to PKH-67 labeled follicular fluid EVs during embryo culture (D1-D7) presenting a punctuated staining due to labeled small-extracellular vesicles, with a 40x magnification. D) Blastocysts exposed to PKH67 labeled PBS 1x during embryo culture (D1-D7) presenting a non-specific green staining with a 40x magnification. Blue = DAPI (nuclear staining); Green dots = PKH67 labeled small-extracellular vesicles (arrow).

In order to further explore the role of EVs mediating oocyte and embryo development, we assessed EVs3-6mm uptake using fluorescence microscopy. The small-extracellular vesicles isolated from follicular fluid from groups of 50 follicles (n = 3) were labeled with PKH67. Following labeling EVs were added to granulosa cell cultures for 24 h. After fixation and microscopic analysis, we were able to visualize the presence of labeled EVs in the cytoplasm of granulosa cells (Fig 3A). We also observed the presence of labeled EVs in cumulus cells following nine hours of exposure to labeled EVs (Fig 4B). Interestingly, we detected labeled EVs within the transzonal projections of cumulus cells, but not into maturing oocytes (Fig 3C).

In addition to EVs uptake by follicular cells, we evaluated embryos treated with small-extracellular vesicles until day 7 in culture. Follicular fluid derived EVs3-6mm were labeled and added to SOF media. After seven days in culture we observed the presence of labeled EVs within embryonic cells suggesting, that there is uptake by embryonic cells (Fig 4).

### Transcript levels following small-extracellular vesicles treatment

The small-extracellular vesicles isolated from ovarian follicles between 3–6 mm (early antral) and pre-ovulatory were incubated with COCs during maturation and with embryos during in vitro culture. In order to evaluate the effects of EVs treatment, we determined the mRNA levels of epigenetic modifiers (*TET1*, *DNMT1*, *DNMT3A*, *HDAC2*, *EHMT1* and *EHMT2*) and genes involved in embryo development (*ACSL6*, *CDH1*, *REST* and *FADS2*).

**Fig 5:**
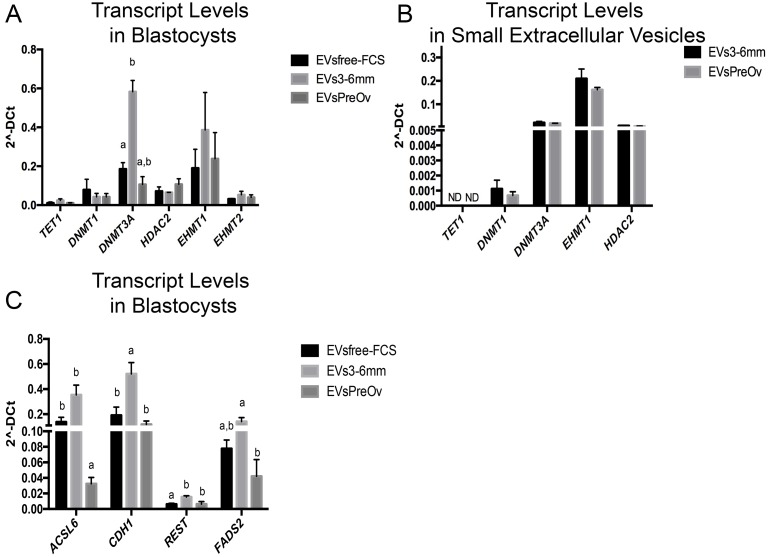
Transcription levels of genes involved in chromatin remodeling and embryo development. (A) Relative transcript levels in blastocysts treated during in vitro culture (IVC) with different EVs supplementation conditions. (B) Transcription levels of selected genes in extracellular vesicles (EVs) from 3–6 mm or pre-ovulatory follicles. (C) Transcription levels of *ACSL6*, *CDH1*, *REST* and *FADS2* in embryos treated during IVC with different EVs supplementation conditions. EVsfree-FCS (EVs-depleted FCS), EVs3-6mm (EVs from 3–6 mm in size follicles) and EVsPreOv (EVs from pre-ovulatory follicles). Different letters indicate p<0.05. ND = not detected. Data are presented as mean of 2^^-ΔCt^ ± s.e.m.

Relative gene expression analysis revealed no difference in levels of *TET1*, *DNMT1*, *HDAC2*, *EHMT1* and *EHMT2* after treatment with follicular EVs. However, we observed increased levels of *DNMT3A*, a gene encoding an enzyme involved in *de novo* methylation of DNA, in embryos following treatment with EVs from 3–6 mm follicles, compared to embryos produced in EVsfree-FCS media p = 0.001 (Fig 5A). Interestingly, *DNMT3A* mRNA levels, as well as the mRNA levels of the other epigenetic modifiers, were similar between EVs from 3–6 mm and pre-ovulatory follicles (Fig 5B). However, treatment with EVs from pre-ovulatory follicles did not change the levels of transcription of the analyzed genes. We also evaluated genes known to play an important role during early embryo development. EVs3-6mm treatment increased the transcription levels of *ASCL6* compared to EVsfree-FCS (p = 0.015) and EVsPreOv (p<0.002) in blastocysts (Fig 5C). Similarly, EVs3-6mm treatment increased the transcription levels of *CDH1* compared to embryos from EVsPreOv (p = 0.016) and EVsfree-FCS (p = 0.022) in Fig 5C. Also, EVs3-6mm treatment increased the transcription levels of *REST* compared to embryos from EVsPreOv (p = 0.049) and EVsfree-FCS (p = 0.027) in Fig 5C. Relative levels of *FADS2* were increased after treatment with EVs from 3-6mm follicles, compared to embryos produced in EVsPreOv (p = 0.021) in Fig 5C. Therefore, small-extracellular vesicles treatment during oocyte maturation and embryo culture modulates transcription levels compared to embryos treated with EVsfree-FCS.

**Fig 6:**
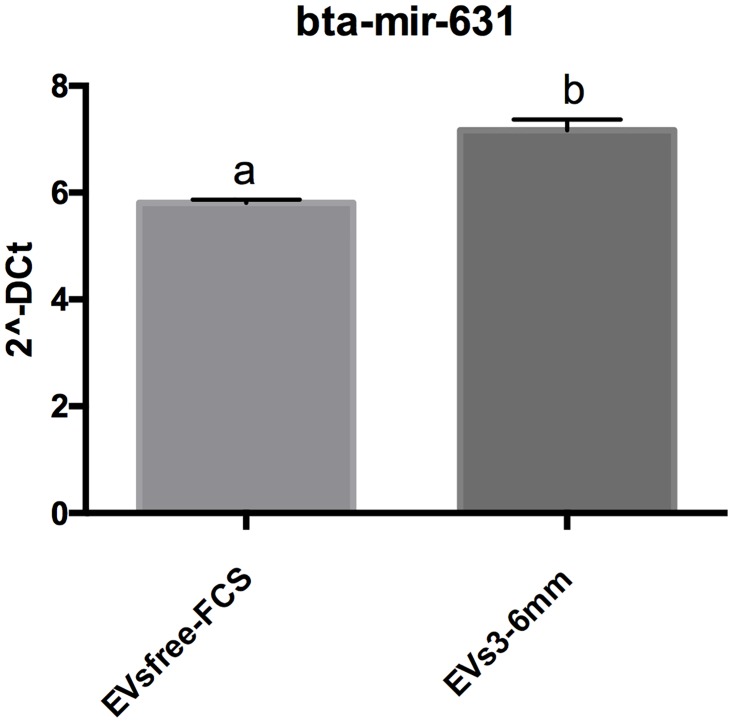
MiRNA-631 levels in in vitro produced 4-cell embryos. Levels of miR-631 in pooled 4-cell embryos (n = 3) after treatment with small-extracellular vesicles (EVs) during oocyte maturation and embryo culture. Different letters indicate p<0.05. EVsfree-FCS (EVs-depleted FCS), EVs3-6mm (EVs from 3–6 mm in size follicles). Data are presented as mean of 2^^-ΔCt^ ± s.e.m.

Furthermore, we investigated the levels of highly abundant EVs3-6mm miRNAs (miR-615, miR-27a-5p, miR-631, miR-323, miR-574, miR-767, miR-320a, miR-411b, miR-421, miR-382 and miR-669) in 4-cell embryos cultured in EVsfree-FCS and EVs3-6mm treatment groups. MiRNA levels following treatments during oocyte maturation and embryo culture were altered in 4-cell embryos on Day 3 after fertilization. Of the 13 miRNAs analyzed, the level of miRNA-631 was significantly increased in 4-cell embryos, following treatment with EVs from 3–6 mm follicles (p = 0.002) in Fig 6. Therefore, extracellular vesicles depletion from FCS and supplementation with EVs from 3–6 mm follicles during oocyte maturation and embryo culture can modulate the levels of miRNA-631 in bovine 4-cell embryos.

### Blastocyst rates after extracellular vesicles treatment

**Fig 7:**
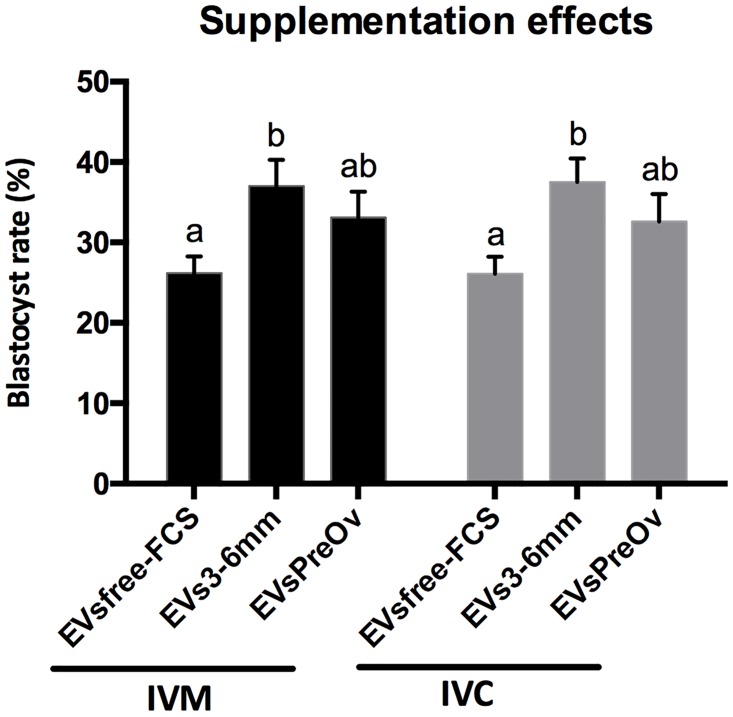
In vitro embryo developmental rates. (A) Blastocyst rates following small-extracellular vesicles (EVs) treatment during oocyte in vitro maturation (IVM) and embryo culture (IVC). Results are based on five independent in vitro production (IVP) routines in total ~979 cumulus-oocyte-complexes (COCs) (EVsfree-FCS = 318, EVs3-6mm = 318, EVsPreOv = 343). EVsfree-FCS (EVs-depleted FCS), EVs3-6mm (EVs from 3–6 mm in size follicles) and EVsPreOv (EVs from pre-ovulatory follicles). Different letters indicate statistical difference P<0.05.

Blastocyst rates were used to evaluate the different oocyte and embryo culture treatments. We evaluated the effects of supplementation with small-extracellular vesicles originating from pooled follicular fluid from early antral (3–6 mm) and pre-ovulatory follicles in in vitro production (IVP). In order to evaluate the role of EVs modulating IVP rates, we performed treatments during oocyte maturation and embryo development. Treatment with EVs3-6mm during oocyte maturation increased the embryo development rate (p = 0.014) to 37% compared to 26% obtained when embryos were maintained in maturation media with EVsfree-FCS (Fig 7A). The EVs from pre-ovulatory follicles did not change embryo development rates (p>0.05) when compared with EVsfree-FCS, and with treatment with EVs from 3–6 mm follicles (Fig 7A). Similarly, treatment with EVs3-6mm follicles during in vitro culture caused a significant increase in embryo development rates (p = 0.01) to 37.5% compared to 26%, when producing embryos in media with EVsfree-FCS (Fig 7A). Treatment with EVs from pre-ovulatory follicles during in vitro culture did not change blastocyst rates (p>0.05), when compared to treatment with EVsfree-FCS, and to treatment with EVs3-6mm follicles (Fig 7A).

### Global DNA methylation and hydroxymethylation analysis of blastocysts

**Fig 8:**
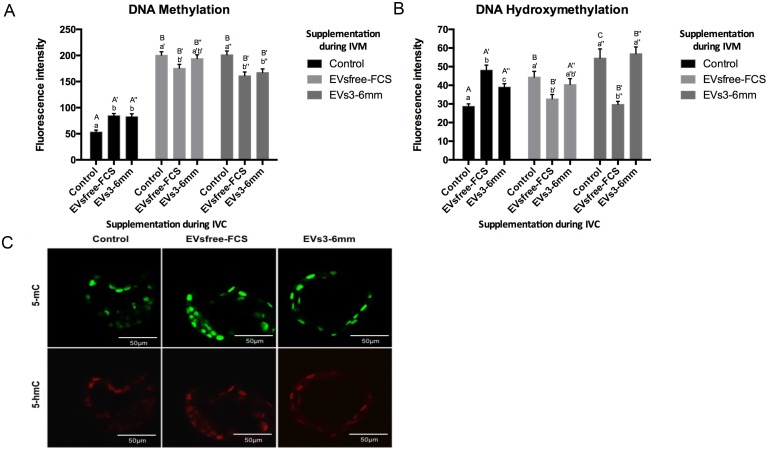
Global DNA methylation and hydroxymethylation levels in blastocysts after supplementation with small-extracellular vesicles (EVs). (A) Global DNA methylation levels in D7 blastocysts. (B) Global DNA hydroxymethylation levels in D7 blastocysts. (C) Confocal images of embryos that underwent different treatments during oocyte maturation and embryo development (40x objective). Different capital letters (A and B, A’ and B’ or A” and B”) indicate differences (p<0.05) among IVM groups within a given IVC group. Different small letters (a and b, a’ and b’, or a” and b”) indicate differences (p<0.05) among IVC groups within a given IVM group. Control (normal FCS), EVsfree-FCS (EVs-depleted FCS), EVs3-6mm (EVs from 3–6 mm in size follicles). Data are presented as mean ± s.e.m.

One of our objectives was to verify whether EVs might modulate changes in global DNA methylation and hydroxymethylation of in vitro produced bovine blastocysts. In order to evaluate such epigenetic modifications, we performed immunofluorescence with validated antibodies for 5mC and 5hmC in blastocysts. Since the above experiments suggested that EVs from 3–6 mm follicles contained epigenetic modifiers, and could be taken up by follicular cells and embryos, we decided to investigate their role in modulating global DNA methylation and hydroxymethylation of in vitro produced blastocysts. We compared IVF embryos (control) with embryos produced using EVs-depleted FCS (EVsfree-FCS) and small-extracellular vesicles (EVs3-6mm). Confocal microscopy analysis demonstrated that oocytes matured in control media and treated with EVsfree-FCS or with EVs3-6mm during IVC, produced embryos that presented higher methylation levels (p<0.05), respectively, compared to embryos cultured in control media (Fig 8A). Similarly, oocytes matured in control media, and treated with EVsfree-FCS and with EVs3-6mm during IVC, contained higher hydroxymethylation levels (p<0.05), respectively, compared to embryos cultured in control media (Fig 8B). Remarkably, the removal of extracellular vesicles from FCS, or the supplementation of EVs from 3–6 mm follicles during oocyte maturation, was capable of modulating epigenetic changes, according to confocal analysis. Withdrawal of EVs from FCS during maturation reduced DNA methylation and hydroxymethylation in embryos cultured during IVC in media containing EVsfree-FCS (p<0.05), compared to embryos cultured during IVC in control media (Fig 8A and 8B). Treatment with EVs3-6mm during oocyte maturation increased methylation levels in embryos cultured during IVC in control media (p<0.05) compared to embryos cultured in EVsfree-FCS and EVs3-6mm media (Fig 8A). Treatment with EVs3-6mm during oocyte maturation decreased levels of DNA hydroxymethylation in embryos cultured during IVC in EVsfree-FCS media (p<0.05) compared to embryos cultured in control and EVs3-6mm media (Fig 8B).

## Discussion

Here we identified the RNA content of small-extracellular vesicles isolated from follicular fluid and investigated the effects of these vesicles supplementation during bovine oocyte maturation and embryo development in vitro. Furthermore we demonstrated that EVs3-6mm supplementation induces changes in transcript levels, in blastocyst rates and in global DNA methylation and hydroxymethylation.

Nanoparticle analysis and TEM revealed the presence of small vesicles between 30–200 nm, resembling small-extracellular vesicles [19]. Western blot analysis demonstrated that EVs isolated herein are positive for ALIX, CD63 and negative for CYT C. The presence of ALIX and CD63 was still evident in follicular fluid without EVs, showing that ultracentrifugation does not remove the extracellular vesicles from follicular fluid in totality. Additionally, CD63 presented typical protein bands between 30-67kDa (highly glycosylated) as verified in other extracellular vesicles preparations confirming the isolation procedure [34]. Importantly, EVs preparations were negative for CYT C, demonstrating the absence of cell contamination/cell death, since CYT C is a mitochondrial protein and should not be present in the extracellular compartment [35]. In an attempt to identify a possible normalizer for EVs-protein input, we evaluated the levels of ACTB in these vesicles, as well as in follicular cells and in follicular fluid. The results suggest that ACTB is not a good normalizer for EVs due to its low abundance. Similar results were obtained in a complete proteomic analysis of different types of EVs, suggesting that ACTB is not a good small-extracellular vesicles marker, since it can also be present in other types of vesicles [34]. Based on the absence of CYT C and the presence of ALIX, and CD63, we demonstrated the purity of the EVs isolated and used in the experiments. Additionally, we determined the presence of PABP a mRNA stabilizing protein. The presence of PABP protein in extracellular vesicles was identified in human duodenal cancer cells, although the function is not clear [36]. The presence of PABP in EVs present in follicular cells may indicate that mRNAs present within EVs are stabilized and could possibly be translated, suggesting an important role for these molecules within these vesicles.

The biological activities of EVs are mediated by the ability of target cells to take up these vesicles. We determined the uptake of EVs by granulosa and cumulus cells, similar to what was previously described in horses and cattle, and demonstrated that EVs are indeed taken up by these cells [10,16]. Interestingly, we identified labeled EVs in cumulus cells and in the transzonal projections of the cumulus cells. These projections are formed by cytoplasmic extensions of cumulus cells, which are present during early oocyte maturation, and can mediate transfer of RNA molecules between cumulus cells and the oocyte [6]. Recently, small-extracellular vesicles resembling exosomes were identified outside of the transzonal projections, suggesting that there is secretion of these cell-secreted vesicles into the perivitelline space [6]. Based on our results is reasonable to speculate that these vesicles may mediate the transport of information from follicular fluid to the oocyte. Furthermore, we determined the uptake of EVs by embryonic cells during early development. Uterine fluid extracellular vesicles can transfer RNA molecules, such as endogenous retroviruses (enJSRVs), to the ovine conceptus [18]. Additionally, oviductal exosomes (oviductosomes) can transfer mRNA, lipids, and protein to the embryo during its passage through the oviduct [11]. Although we demonstrated that early bovine embryos can take up EVs from the follicular fluid of 3–6 mm follicles; however, the physiological mechanism is not yet clear.

RNA-seq analysis of small-extracellular vesicles isolated from 3–6 mm ovarian follicles demonstrated the presence of coding and non-coding RNA molecules in agreement with previous studies [17]. Interestingly, functional annotation indicated the presence of mRNAs involved in DNA-protein interactions, nucleosome assembly, and transcription regulation. To expand on this finding, we analyzed the levels of *DNMT1*, *DNMT3A*, *EHMT1*, *HDAC2*, *EIF4b* and *EIF4e* in EVs isolated from 3–6 mm follicles. Real-time PCR analysis revealed the presence of *DNMT1*, *DNMT3A*, *EHMT1*, *HDAC2*, *EIF4b* and *EIF4e* in EVs from 3–6 mm follicles. These transcripts were previously described in extracellular vesicles derived from mesenchymal stem cells and different types of cancers, such as glioblastoma and colorectal tumors [37]. In addition to RNA-seq, in the present study miRNA profile analysis identified a total of 280 miRNAs in EVs, and among them, 13 were present at high levels by real-time PCR. MiR-615 was the most abundantly expressed miRNA in small-extracellular vesicles. A validated target of miR-615 is IGF2, a well-known paternally imprinted gene involved in embryo development [38]. Furthermore, miR-323 was also detected in EVs from immature follicles. MiRNA-323 is known to cause down-regulation of embryonic ectoderm development (EED) mRNAs levels in mouse ES cells [39]. Thus, RNA-seq and miRNA analysis suggest that small-extracellular vesicles contents may modulate events occurring in small antral follicle development.

To investigate the roles of EVs in inducing changes in gene expression in bovine blastocysts, we supplemented the oocyte maturation and embryo culture media with EVs isolated from follicular fluid. In our analysis, we identified the presence of epigenetic modifiers, such as DNMTs and miRNAs in EVs3-6mm, suggesting that EVs content can induce changes in DNA methylation, and therefore gene expression. We investigated the transcript levels of epigenetic modifier genes (such as *TET1*, *DNMT1*, *DNMT3A*, *HDAC2*, *EHMT1* and *EHMT2*), but only *DNMT3A* was altered by EVs treatment during embryo culture. Interestingly, we found *DNMT3A* in EVs isolated from immature follicles, a time when the oocytes are finishing the acquisition of the epigenetic modifications [9]. Our analysis demonstrates that EVs treatment did not change *DNMT1* levels, but increased levels of *DNMT3A* in embryos after EVs3-6mm treatment. Additionally, it is possible that EVs carrying *DNMT3A* could modulate the “epigenetic maturation” of oocyte DNA during follicle growth impacting embryo development. In order to investigate whether EVs contents can induce changes related to embryo development, we evaluated levels of transcripts of genes involved with cell metabolism, cycle and fate. Among the genes whose transcripts were increased in blastocysts after treatment with EVs3-6mm vesicles, are *ASCL6*, *CDH1*, and *REST*. ASCL6 is involved in the embryo metabolism that modulated the catalysis of long chain fatty acid modulating membrane fluidity [40,41]. Also, we observed increased transcript levels of *CDH1* and *REST* after treatment with EVs3-6mm. These genes are involved in cell cycle, cell fate and neuronal development [42–44]. We also observed a negative effect of EVsPreOv treatment, which induced down-regulation of *FADS2* transcript levels. These results demonstrate that EVs treatment regulates genes involved in metabolism, suggesting a role in regulating embryo development. Similar to the effect on mRNA levels, supplementation with EVs from follicular fluid altered miRNA-631 levels in bovine 4-cell embryos. Interestingly, levels of the selected miRNA were higher in embryos from the EVs3-6mm group compared to EVsfree-FCS treatment, suggesting the transfer of miR-631 from EVs to embryonic cells. MiRNA-631 is predicted to target *CDR1as* a circular RNA involved in regulating mir-7 through its “miRNA-sponge” function [45]. Therefore, the observed changes in mRNAs and miRNAs levels after EVs supplementation suggest that these extracellular vesicles isolated from follicular fluid can be used as a new approach to modulate early embryonic development in the IVP system.

Furthermore, we performed functional experiments to evaluate the effects of EVs3-6mm on blastocysts rates compared to EVsfree-FCS and EVsPreOv, once we did not detected differences on IVP rates after EVs removal from FCS. Additionally, we compared the effects EVs3-6mm on global DNA methylation and hydroxymethylation compared to standard IVP and EVsfree-FCS. We observed that supplementation with EVs3-6mm during oocyte maturation and embryo development increased blastocyst rates. In a similar experiment, the treatment of mouse and bovine COCs with extracellular vesicles isolated from bovine follicular fluid of small follicles (3–5 mm) increased cumulus expansion rates compared to FBS and EV-free FBS [17]. In theory, EVs from pre-ovulatory follicles are present at the time of ovulation and of early embryo development events, such as fertilization and cleavage, however we did not observed changes in blastocyst rates. Similarly, the addition of extracellular vesicles from oviductal cells in bovine embryo cultures had mild effects on oocyte cleavage or blastocyst rates [46]. However, treatment with extracellular vesicles from oviductal cells, or removal of EVs from serum, improved embryo quality parameters, such as embryo survival after vitrification [46]. Importantly, in our experiments, supplementation with EVs from 3–6 mm follicles did not induce deleterious effects. The small-extracellular vesicles supplementation during oocyte IVM and embryo IVC induced changes in global DNA methylation and hydroxymethylation. This is the first report of EVs mediating epigenetic changes in in vitro-produced bovine embryos. Similarly, global DNA methylation was increased in bovine embryos produced at a high oxygen tension compared at a low oxygen tension [47]. These data demonstrate that global DNA methylation is modulated by environmental factors. Our study reveals that the removal of extracellular vesicles from FCS, and supplementation with EVs from 3–6 mm follicles during oocyte maturation, increases global DNA methylation and hydroxymethylation, except in the case of embryos cultured during IVC in EVsfree-FCS media. Additionally, removal of extracellular vesicles from FCS, and media supplementation with EVs3-6mm during embryo IVC, increased global DNA methylation and hydroxymethylation compared to embryos originating from oocytes matured in control media (FCS). The supplementation of media with EVs from 3–6 mm follicles during maturation and embryo development increased *DNMT3A* transcript levels in embryos in the blastocyst stage. Interestingly, global methylation analysis revealed that supplementation with EVs from 3–6 mm follicles, during embryo development, increases the levels of DNA methylation compared to supplementation with EVsfree-FCS and control media. Thus, supplementation of oocyte maturation media or SOF with EVs from 3–6 mm follicle can be used as a new approach to improve the in vitro production system.

In this study, we demonstrate that small-extracellular vesicles present in follicular fluid isolated from bovine ovarian follicles can modulate, mRNA and miRNA levels, blastocyst rates, as well as global DNA methylation and hydroxymethylation levels in in vitro-produced bovine embryos. Bioinformatics analysis demonstrated that messenger RNAs identified within small-extracellular vesicles may be involved in controlling DNA-protein interactions and translation machinery. Treatment with EVs induced changes in genes called epigenetic modifiers, which are associated with embryo development. In conclusion, EVs from 3–6 mm ovarian follicles can change transcription levels, as well as global DNA methylation and hydroxymethylation of in vitro produced bovine embryos, which may be carried by the small-extracellular vesicles present in follicular fluid.

## Supporting information

S1 Fig Blastocyst rates. Blastocyst rates demonstrating the effects of EVs removal from FCS.
(TIFF)

S1 Table RNA-seq results. RNA-seq results demonstrated a variety of coding and non-coding RNA molecules present in EVs.
(PDF)

S2 Table MicroRNAs analysis. MicroRNA profile of EVs.
(XLS)

S3 Table Bioinformatics analysis of selected miRNAs. Bioinformatics analysis predicted that the 13 highly abundant miRNAs.
(XLS)
