# Supplementary figures and images for: Supplementation with small-extracellular vesicles from ovarian follicular fluid during in vitro production modulates bovine embryo development
Source: PLoS One. 2017 Jun 15;12(6):e0179451. doi: 10.1371/journal.pone.0179451 (PMC5472319; doi:10.1371/journal.pone.0179451)

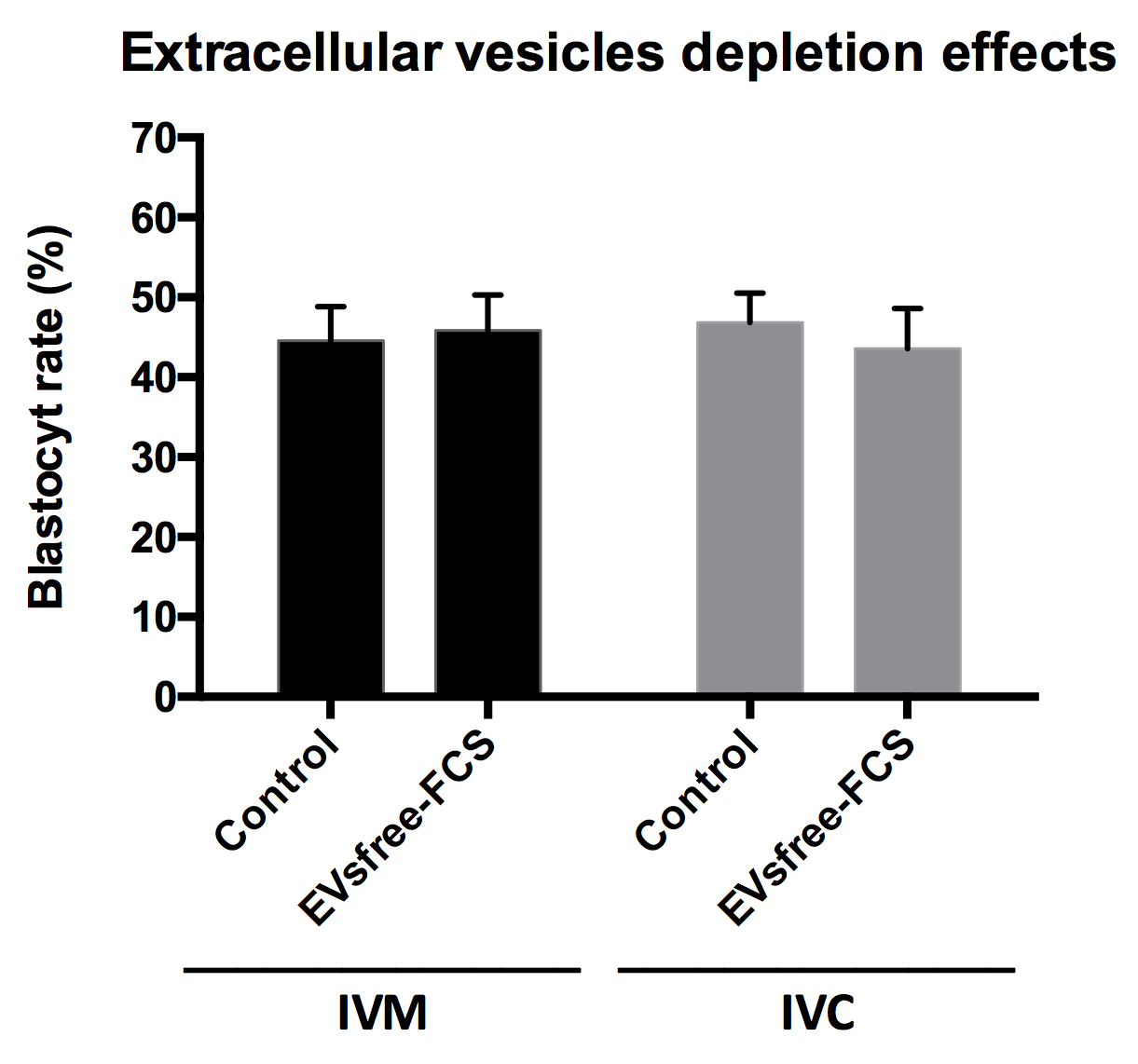

Supplement: S1 Fig — Blastocyst rates demonstrating the effects of EVs removal from FCS. (TIFF) [file pone.0179451.s001.tiff]
