# Supplementary material for: Supplementation with small-extracellular vesicles from ovarian follicular fluid during in vitro production modulates bovine embryo development
Source: PLoS One. 2017 Jun 15;12(6):e0179451. doi: 10.1371/journal.pone.0179451 (PMC5472319; doi:10.1371/journal.pone.0179451)
Supplement: S1 Table — RNA-seq results demonstrated a variety of coding and non-coding RNA molecules present in EVs. (PDF) [file pone.0179451.s002.pdf]

tracking_id	gene_id	locus	FPKM	FPKM_conf_l	FPKM_conf_l	FPKM_status
ENSBTAG00000037791	ENSBTAG00000037791	5:27642324-27643598	114.05	176.015	205.432	OK
ENSBTAG00000039523	ENSBTAG00000039523	7:15428920-15472604	61.259	413.175	452.475	OK
ENSBTAG00000044340	ENSBTAG00000044340	9:15520974-15521079	42.0077	0	13.8038	OK
ENSBTAG00000035785	ENSBTAG00000035785	10:24757321-24765544	35.9708	141.58	158.958	OK
ENSBTAG00000046685	ENSBTAG00000046685	2:31520691-31520798	29.9946	0	13.5458	OK
ENSBTAG00000044996	ENSBTAG00000044996	3:28540505-28540612	29.9942	0	13.5458	OK
ENSBTAG00000043819	ENSBTAG00000043819	8:83025390-83025497	29.9934	0	13.5458	OK
ENSBTAG00000042928	ENSBTAG00000042928	5:48996774-48996881	29.9919	0	13.5458	OK
ENSBTAG00000003282	ENSBTAG00000003282	5:89258892-89311142	28.8816	44.6732	54.0688	OK
ENSBTAG00000006050	ENSBTAG00000006050	3:20759080-20760404	26.3538	12.504	22.2528	OK
ENSBTAG00000042717	ENSBTAG00000042717	25:36563534-36563642	26.2586	0	13.4204	OK
ENSBTAG00000009772	ENSBTAG00000009772	7:98947945-99003794	23.0249	60.6782	69.5469	OK
ENSBTAG00000028219	ENSBTAG00000028219	2:16247240-16247360	21.1004	0	12.0783	OK
ENSBTAG00000045815	ENSBTAG00000045815	14:576755-576875	19.3864	0	12.0783	OK
ENSBTAG00000040129	ENSBTAG00000040129	8:109710619-109710738	17.6939	0	12.1798	OK
ENSBTAG00000044415	ENSBTAG00000044415	14:29074174-29074285	16.6651	0	13.0576	OK
ENSBTAG00000043711	ENSBTAG00000043711	7:77152336-77152455	15.4652	0	12.1798	OK
ENSBTAG00000030838	ENSBTAG00000030838	4:120643216-120644622	14.0351	2.50353	6.77427	OK
ENSBTAG00000042124	ENSBTAG00000042124	22:7693178-7693304	12.8902	0	11.5032	OK
ENSBTAG00000013910	ENSBTAG00000013910	3:21502102-21516918	12.414	9.53743	14.4882	OK
ENSBTAG00000043093	ENSBTAG00000043093	19:13159741-13159871	12.3849	0	11.1492	OK
ENSBTAG00000042477	ENSBTAG00000042477	7:7809393-7809519	12.148	0	11.5032	OK
ENSBTAG00000045007	ENSBTAG00000045007	15:39958517-39958663	12.0173	0	9.92739	OK
ENSBTAG00000042752	ENSBTAG00000042752	16:2043851-2043981	11.9709	0	11.1492	OK
ENSBTAG00000045764	ENSBTAG00000045764	5:12836041-12836171	11.7861	0	11.1492	OK
ENSBTAG00000035244	ENSBTAG00000035244	11:2775464-2782703	11.1999	5.14673	9.89756	OK
ENSBTAG00000045070	ENSBTAG00000045070	13:12039483-12039612	11.1391	0	11.2356	OK
ENSBTAG00000030892	ENSBTAG00000030892	3:121412143-121413236	11.0909	1.70495	5.68317	OK
ENSBTAG00000042648	ENSBTAG00000042648	X:67791939-67792068	11.0268	0	11.2356	OK

ENSBTAG00000047513	ENSBTAG00000047513	GJ059463.1:3684-3803	10.5218	0	12.1798	OK
ENSBTAG00000043125	ENSBTAG00000043125	22:7389768-7389898	10.4583	0	11.1492	OK
ENSBTAG00000039369	ENSBTAG00000039369	4:74047023-74047142	10.361	0	12.1798	OK
ENSBTAG00000047755	ENSBTAG00000047755	16:52297146-52298167	10.3124	0.284419	3.98186	OK
ENSBTAG00000039227	ENSBTAG00000039227	19:64048564-64048684	10.0251	0	12.0783	OK
ENSBTAG00000005491	ENSBTAG00000005491	20:55752613-55753240	9.10119	1.98141	7.59539	OK
ENSBTAG00000042678	ENSBTAG00000042678	13:68003659-68003794	8.66454	0	10.7363	OK
ENSBTAG00000009166	ENSBTAG00000009166	12:32445443-32446691	8.59139	0	2.9823	OK
ENSBTAG00000025182	ENSBTAG00000025182	7:48966619-48985225	8.18265	8.02817	12.3564	OK
ENSBTAG00000044198	ENSBTAG00000044198	17:74333155-74335433	8.13304	0	2.45199	OK
ENSBTAG00000003331	ENSBTAG00000003331	7:48620065-48620809	7.94478	0.278302	3.33963	OK
ENSBTAG00000038128	ENSBTAG00000038128	23:25404019-25411643	7.90857	6.98136	8.89062	OK
ENSBTAG00000029076	ENSBTAG00000029076	11:46773151-46773271	7.83899	0	12.0783	OK
ENSBTAG00000043193	ENSBTAG00000043193	6:99632945-99633080	7.75193	0	10.7363	OK
ENSBTAG00000029287	ENSBTAG00000029287	3:68946821-68946953	7.69295	0	10.9803	OK
ENSBTAG00000007070	ENSBTAG00000007070	18:53231598-53232201	7.5374	0	2.40365	OK
ENSBTAG00000042716	ENSBTAG00000042716	23:22191348-22191664	7.48022	0.655244	7.86292	OK
ENSBTAG00000045801	ENSBTAG00000045801	25:33028969-33029114	7.36036	0	9.99585	OK
ENSBTAG00000043271	ENSBTAG00000043271	1:88549111-88549241	7.25881	0	11.1492	OK
ENSBTAG00000047873	ENSBTAG00000047873	4:112048064-112048217	7.07271	0	9.47319	OK
ENSBTAG00000045711	ENSBTAG00000045711	11:105874694-105875699	6.93947	0.29163	3.49955	OK
ENSBTAG00000005748	ENSBTAG00000005748	14:23884841-23886642	6.93367	0.167929	2.01515	OK
ENSBTAG00000044472	ENSBTAG00000044472	3:115730325-115730459	6.19942	0	10.8164	OK
ENSBTAG00000043521	ENSBTAG00000043521	5:113334005-113334140	6.18341	0	10.7363	OK
ENSBTAG00000046046	ENSBTAG00000046046	24:40563770-40566586	6.09928	4.11761	7.4264	OK
ENSBTAG00000044941	ENSBTAG00000044941	4:91730656-91730781	5.84663	0	11.5952	OK
ENSBTAG00000030705	ENSBTAG00000030705	18:54208181-54218832	5.73566	0.136401	1.63681	OK
ENSBTAG00000044323	ENSBTAG00000044323	23:8744230-8744363	5.44964	0	10.8977	OK
ENSBTAG00000026779	ENSBTAG00000026779	5:44712168-44720976	5.11843	4.92684	10.2426	OK
ENSBTAG00000025391	ENSBTAG00000025391	23:31600422-31607791	5.04029	0.415777	4.98932	OK

ENSBTAG00000030166	ENSBTAG00000030166	19:57717494-57722442	4.99788	0.288179	2.44953	OK
ENSBTAG00000048007	ENSBTAG00000048007	8:8254876-8256175	4.80723	0	2.34911	OK
ENSBTAG00000030278	ENSBTAG00000030278	11:104130863-104131233	4.68652	0	6.11561	OK
ENSBTAG00000045882	ENSBTAG00000045882	8:119824-119943	4.61952	0	8.69987	OK
ENSBTAG00000045734	ENSBTAG00000045734	11:105920719-105921669	4.59103	0	1.66215	OK
ENSBTAG00000020203	ENSBTAG00000020203	29:44993274-44994609	4.57256	0	1.39589	OK
ENSBTAG00000019605	ENSBTAG00000019605	18:53545324-53612052	4.56638	0	22.2597	OK
ENSBTAG00000016573	ENSBTAG00000016573	11:30823434-30888003	4.54488	3.50971	5.57505	OK
ENSBTAG00000037523	ENSBTAG00000037523	16:52467803-52468793	4.41311	0	1.88234	OK
ENSBTAG00000040605	ENSBTAG00000040605	18:12433767-12434808	4.36308	0.198902	2.38682	OK
ENSBTAG00000030585	ENSBTAG00000030585	1:150439866-150440580	4.30602	0	2.02997	OK
ENSBTAG00000037902	ENSBTAG00000037902	2:122032488-122033438	4.17477	0	2.03282	OK
ENSBTAG00000042923	ENSBTAG00000042923	11:72613720-72613870	4.16732	0	8.28228	OK
ENSBTAG00000000124	ENSBTAG00000000124	5:30894555-30899509	4.15279	0.166713	2.00055	OK
ENSBTAG00000003207	ENSBTAG00000003207	2:25773542-25776191	4.14155	0	1.56861	OK
ENSBTAG00000047881	ENSBTAG00000047881	12:77227237-77228026	4.03494	0.26243	3.14916	OK
ENSBTAG00000040514	ENSBTAG00000040514	18:42358256-42358396	3.95352	0	7.39489	OK
ENSBTAG00000019303	ENSBTAG00000019303	25:41960756-41961974	3.91799	0	3.0578	OK
ENSBTAG00000040118	ENSBTAG00000040118	19:41980437-41981037	3.88987	0	3.10585	OK
ENSBTAG00000046703	ENSBTAG00000046703	25:1571168-1571725	3.81327	0	2.60215	OK
ENSBTAG00000047400	ENSBTAG00000047400	18:62561914-62564977	3.80149	0	2.3683	OK
ENSBTAG00000001765	ENSBTAG00000001765	7:3938569-3941465	3.74728	0	1.9291	OK
ENSBTAG00000032003	ENSBTAG00000032003	X:81941607-81942135	3.73509	0.392153	4.70584	OK
ENSBTAG00000046144	ENSBTAG00000046144	27:5219283-5223726	3.68013	0.483778	5.80534	OK
ENSBTAG00000008353	ENSBTAG00000008353	23:10560498-10568780	3.62517	1.67265	4.13762	OK
ENSBTAG00000000825	ENSBTAG00000000825	13:52590817-52593105	3.61721	0	2.53539	OK
ENSBTAG00000042649	ENSBTAG00000042649	15:55370966-55371116	3.60882	0	6.9019	OK
ENSBTAG00000042756	ENSBTAG00000042756	18:29354916-29355070	3.59763	0	6.72263	OK
ENSBTAG00000031768	ENSBTAG00000031768	23:31518524-31518917	3.59569	0	3.68804	OK
ENSBTAG00000013711	ENSBTAG00000013711	23:17893780-17895740	3.59562	0.155448	1.86538	OK

ENSBTAG00000014468	ENSBTAG00000014468	25:36180838-36185662	3.57958	1.81099	5.07078	OK
ENSBTAG00000017471	ENSBTAG00000017471	10:11967595-11968972	3.52165	0	1.35331	OK
ENSBTAG00000020568	ENSBTAG00000020568	20:4818456-4821534	3.50728	0.13222	1.58664	OK
ENSBTAG00000032200	ENSBTAG00000032200	15:62935665-63020362	3.50205	3.48972	7.56107	OK
ENSBTAG00000025666	ENSBTAG00000025666	10:26814898-26815069	3.50194	0	6.0543	OK
ENSBTAG00000047845	ENSBTAG00000047845	4:94432193-94432409	3.49954	0	6.71018	OK
ENSBTAG00000038034	ENSBTAG00000038034	18:62475321-62481823	3.48868	0.131674	1.05339	OK
ENSBTAG00000006649	ENSBTAG00000006649	7:3730629-3731451	3.4384	0.251894	3.02273	OK
ENSBTAG00000015753	ENSBTAG00000015753	4:43698579-43782568	3.40951	0	10.2945	OK
ENSBTAG00000007230	ENSBTAG00000007230	29:22456237-22457437	3.40806	0.345095	2.93331	OK
ENSBTAG00000044404	ENSBTAG00000044404	14:22605810-22605948	3.38507	0	6.00165	OK
ENSBTAG00000045661	ENSBTAG00000045661	10:42671323-42671620	3.37348	0	4.88013	OK
ENSBTAG00000023258	ENSBTAG00000023258	5:99102506-99103394	3.32322	3.49758	9.79323	OK
ENSBTAG00000004507	ENSBTAG00000004507	11:104509024-104510345	3.30125	0	2.96401	OK
ENSBTAG00000034506	ENSBTAG00000034506	19:12231222-12231584	3.29974	0.571981	5.71981	OK
ENSBTAG00000042435	ENSBTAG00000042435	2:120040447-120040581	3.23047	0	6.1808	OK
ENSBTAG00000032017	ENSBTAG00000032017	2:72523470-72527172	3.21573	0	1.51876	OK
ENSBTAG00000046336	ENSBTAG00000046336	7:17253184-17254000	3.15958	0.253746	3.04496	OK
ENSBTAG00000014287	ENSBTAG00000014287	X:58419390-58420170	3.15275	0.796373	5.0437	OK
ENSBTAG00000008881	ENSBTAG00000008881	7:52983070-52984039	3.13368	0.213681	2.56417	OK
ENSBTAG00000033884	ENSBTAG00000033884	5:11308519-11308714	3.11111	0	5.30915	OK
ENSBTAG00000007388	ENSBTAG00000007388	9:87840981-87862199	3.09577	0.26243	2.09944	OK
ENSBTAG00000005272	ENSBTAG00000005272	7:21649440-21654927	3.09306	0.151913	2.12678	OK
ENSBTAG00000046803	ENSBTAG00000046803	8:81511016-81512051	3.08118	0	1.40039	OK
ENSBTAG00000046966	ENSBTAG00000046966	19:35833320-35838743	3.07463	0	2.49801	OK
ENSBTAG00000047101	ENSBTAG00000047101	10:81675808-81677905	2.99802	0	0.888656	OK
ENSBTAG00000025441	ENSBTAG00000025441	23:27331772-27333698	2.98602	0.322519	2.04262	OK
ENSBTAG00000048136	ENSBTAG00000048136	1:74377808-74378084	2.95836	0	5.25144	OK
ENSBTAG00000020923	ENSBTAG00000020923	7:4264309-4266113	2.94648	0	1.22106	OK
ENSBTAG00000032453	ENSBTAG00000032453	3:20811442-20813500	2.94208	0	3.77229	OK

ENSBTAG00000037994	ENSBTAG00000037994	24:58601577-58602012	2.93565	0	4.28394	OK
ENSBTAG00000031327	ENSBTAG00000031327	21:69817754-69824185	2.9212	0	7.88142	OK
ENSBTAG00000019467	ENSBTAG00000019467	8:100138373-100139780	2.87347	0.294324	2.35459	OK
ENSBTAG00000000413	ENSBTAG00000000413	7:19743680-19747322	2.87011	0	1.97466	OK
ENSBTAG00000048033	ENSBTAG00000048033	7:67407604-67407826	2.86045	0	5.59613	OK
ENSBTAG00000039587	ENSBTAG00000039587	19:41998073-41998703	2.84415	0	2.95796	OK
ENSBTAG00000007035	ENSBTAG00000007035	5:20898143-20899948	2.83769	0.344139	2.17955	OK
ENSBTAG00000044213	ENSBTAG00000044213	14:17878463-17878752	2.83156	0	5.01522	OK
ENSBTAG00000040574	ENSBTAG00000040574	22:49566541-49568435	2.8279	0.401273	5.21655	OK
ENSBTAG00000018284	ENSBTAG00000018284	27:289080-291006	2.8252	0	0.773013	OK
ENSBTAG00000009762	ENSBTAG00000009762	18:34343974-34350507	2.79879	0	9.59843	OK
ENSBTAG00000045818	ENSBTAG00000045818	3:78439928-78440225	2.79859	0	4.88013	OK
ENSBTAG00000031771	ENSBTAG00000031771	23:31526581-31526974	2.75743	0	3.68804	OK
ENSBTAG00000016929	ENSBTAG00000016929	7:50814952-50815885	2.71055	0	1.99733	OK
ENSBTAG00000040580	ENSBTAG00000040580	21:20155589-20163194	2.70476	1.32569	4.39136	OK
ENSBTAG00000047206	ENSBTAG00000047206	23:31556335-31556992	2.70093	0	2.8364	OK
ENSBTAG00000046835	ENSBTAG00000046835	X:4379759-4381789	2.69965	0.256576	3.07891	OK
ENSBTAG00000046642	ENSBTAG00000046642	X:40837706-40838457	2.67628	0.275708	3.85992	OK
ENSBTAG00000036959	ENSBTAG00000036959	17:26438609-26438782	2.67264	0	4.78744	OK
ENSBTAG00000009752	ENSBTAG00000009752	7:19127027-19131655	2.66937	0	3.20191	OK
ENSBTAG00000016279	ENSBTAG00000016279	13:70685086-70688831	2.66162	0.0955942	1.33832	OK
ENSBTAG00000045835	ENSBTAG00000045835	19:38534839-38536253	2.65524	0	2.47478	OK
ENSBTAG00000020957	ENSBTAG00000020957	2:96470386-96472144	2.63395	0.11778	1.64892	OK
ENSBTAG00000047999	ENSBTAG00000047999	22:42296036-42296324	2.62572	0	5.03263	OK
ENSBTAG00000046684	ENSBTAG00000046684	10:102020146-102300271	2.61809	0	5.23619	OK
ENSBTAG00000001608	ENSBTAG00000001608	3:6938874-6939732	2.61037	0.241325	3.37855	OK
ENSBTAG00000002374	ENSBTAG00000002374	25:27750889-27758215	2.60184	0.146849	1.24822	OK
ENSBTAG00000040098	ENSBTAG00000040098	3:20770380-20771015	2.59684	0	12.9842	OK
ENSBTAG00000016450	ENSBTAG00000016450	13:29756746-29778409	2.59276	1.75782	4.39456	OK
ENSBTAG00000040600	ENSBTAG00000040600	19:43937801-43937992	2.58642	0	4.33627	OK

ENSBTAG00000022229	ENSBTAG00000022229	10:25040129-25040959	2.58498	0.495352	4.45816	OK
ENSBTAG00000007793	ENSBTAG00000007793	11:62992907-62993855	2.55381	0.218415	2.62097	OK
ENSBTAG00000047973	ENSBTAG00000047973	19:51499259-51515723	2.54354	0	7.63062	OK
ENSBTAG00000040103	ENSBTAG00000040103	29:50289093-50293879	2.53759	0.152697	1.83236	OK
ENSBTAG00000045034	ENSBTAG00000045034	23:25240215-25240487	2.49941	0	4.56743	OK
ENSBTAG00000045538	ENSBTAG00000045538	11:1666001-1667014	2.48383	0	3.50096	OK
ENSBTAG00000019597	ENSBTAG00000019597	4:115634874-115635149	2.45531	0	4.51761	OK
ENSBTAG00000004386	ENSBTAG00000004386	25:9975298-9975970	2.43541	0	2.15684	OK
ENSBTAG00000017911	ENSBTAG00000017911	26:43270655-43272421	2.4295	0	2.2588	OK
ENSBTAG00000024942	ENSBTAG00000024942	19:45889154-45889866	2.40658	0	2.03567	OK
ENSBTAG00000009274	ENSBTAG00000009274	10:21994852-21995149	2.40044	0	4.18297	OK
ENSBTAG00000046875	ENSBTAG00000046875	29:44563254-44566140	2.38762	0.148801	1.19041	OK
ENSBTAG00000032884	ENSBTAG00000032884	25:9988821-9990357	2.36641	0	7.8606	OK
ENSBTAG00000046516	ENSBTAG00000046516	11:106201874-106202759	2.36288	0	1.63774	OK
ENSBTAG00000008196	ENSBTAG00000008196	7:16995656-16997252	2.35005	0	7.27286	OK
ENSBTAG00000045621	ENSBTAG00000045621	19:14931962-14935460	2.34135	0	2.24018	OK
ENSBTAG00000045893	ENSBTAG00000045893	7:16593484-16594398	2.32605	0	1.72753	OK
ENSBTAG00000048103	ENSBTAG00000048103	3:102875743-102876031	2.32429	0	4.31369	OK
ENSBTAG00000047578	ENSBTAG00000047578	7:45715313-45733680	2.32398	0	2.41701	OK
ENSBTAG00000046888	ENSBTAG00000046888	6:31669691-31669966	2.30849	0	4.51761	OK
ENSBTAG00000024437	ENSBTAG00000024437	3:17137844-17140530	2.30494	0	4.08665	OK
ENSBTAG00000045717	ENSBTAG00000045717	28:19004886-19005699	2.30011	0	1.78278	OK
ENSBTAG00000026543	ENSBTAG00000026543	9:73661286-73661544	2.29216	0	4.01273	OK
ENSBTAG00000002664	ENSBTAG00000002664	19:45973040-45975469	2.28834	0	1.70651	OK
ENSBTAG00000007402	ENSBTAG00000007402	25:42221369-42229176	2.28406	0.35154	2.22642	OK
ENSBTAG00000019548	ENSBTAG00000019548	X:18793750-18794357	2.26661	0	2.38781	OK
ENSBTAG00000047272	ENSBTAG00000047272	19:8790001-8790463	2.26521	0	3.13723	OK
ENSBTAG00000016773	ENSBTAG00000016773	7:5585526-5590227	2.24506	0	2.20609	OK
ENSBTAG00000030648	ENSBTAG00000030648	5:75818029-75827375	2.23697	0.164201	1.97041	OK
ENSBTAG00000013881	ENSBTAG00000013881	3:111481701-111482589	2.23202	0	2.09855	OK

ENSBTAG00000046590	ENSBTAG00000046590	21:59119856-59120831	2.22466	0	1.91129	OK
ENSBTAG00000047961	ENSBTAG00000047961	15:40859002-40859597	2.21893	0	3.9565	OK
ENSBTAG00000002661	ENSBTAG00000002661	21:34950165-34952418	2.21015	0.0919028	1.28664	OK
ENSBTAG00000017221	ENSBTAG00000017221	19:5065785-5066986	2.20683	0.418297	3.76467	OK
ENSBTAG00000039708	ENSBTAG00000039708	11:106338333-106341261	2.18969	0	0.636446	OK
ENSBTAG00000023608	ENSBTAG00000023608	1:68577105-68577418	2.17958	0	3.96914	OK
ENSBTAG00000006121	ENSBTAG00000006121	18:62435401-62438857	2.15875	0	1.40855	OK
ENSBTAG00000018917	ENSBTAG00000018917	4:118002996-118007146	2.1536	0	3.33963	OK
ENSBTAG00000047076	ENSBTAG00000047076	1:45091052-45091460	2.15077	0	3.55245	OK
ENSBTAG00000039466	ENSBTAG00000039466	19:57053364-57064917	2.15069	0	1.33585	OK
ENSBTAG00000039951	ENSBTAG00000039951	3:36428457-36429816	2.15066	0.15236	1.82832	OK
ENSBTAG00000025401	ENSBTAG00000025401	7:40362435-40367515	2.14616	0	1.34744	OK
ENSBTAG00000011012	ENSBTAG00000011012	1:146028056-146029321	2.13633	0.163681	2.29154	OK
ENSBTAG00000034949	ENSBTAG00000034949	2:5345305-5345741	2.13222	0	3.32431	OK
ENSBTAG00000002604	ENSBTAG00000002604	4:29595418-29597808	2.13136	0	1.34162	OK
ENSBTAG00000014601	ENSBTAG00000014601	6:69526334-69553387	2.11681	2.25375	4.12388	OK
ENSBTAG00000020267	ENSBTAG00000020267	7:3070477-3074600	2.10996	0	1.59547	OK
ENSBTAG00000002216	ENSBTAG00000002216	15:35331400-35334046	2.10286	0.110726	1.55016	OK
ENSBTAG00000002027	ENSBTAG00000002027	2:122115659-122117248	2.10282	0.215012	2.58015	OK
ENSBTAG00000047885	ENSBTAG00000047885	13:63695916-63696654	2.09934	0	2.52508	OK
ENSBTAG00000045577	ENSBTAG00000045577	23:27576982-27578092	2.0934	0	3.40234	OK
ENSBTAG00000045608	ENSBTAG00000045608	28:3273925-3274777	2.08644	0	2.02148	OK
ENSBTAG00000007375	ENSBTAG00000007375	17:73273378-73274171	2.07473	0	2.53391	OK
ENSBTAG00000008441	ENSBTAG00000008441	19:54458855-54459555	2.0711	0	2.66216	OK
ENSBTAG00000014232	ENSBTAG00000014232	17:71660018-71678806	2.0613	0.12587	1.76219	OK
ENSBTAG00000014819	ENSBTAG00000014819	2:107655325-107658571	2.06119	0	2.67746	OK
ENSBTAG00000043728	ENSBTAG00000043728	10:95727125-95727458	2.05416	0	3.73076	OK
ENSBTAG00000031278	ENSBTAG00000031278	13:60355541-60362412	2.05119	0.14409	1.72908	OK
ENSBTAG00000031234	ENSBTAG00000031234	4:106517511-106518031	2.0483	0	4.03031	OK
ENSBTAG00000012333	ENSBTAG00000012333	18:62389054-62389957	2.04806	0	1.63221	OK

ENSBTAG00000032853	ENSBTAG00000032853	20:41491222-41492302	2.04128	0.241607	2.89928	OK
ENSBTAG00000048074	ENSBTAG00000048074	10:24049502-24050032	2.0255	0	3.63258	OK
ENSBTAG00000000478	ENSBTAG00000000478	22:50638074-50640896	2.02318	0	2.65458	OK
ENSBTAG00000010952	ENSBTAG00000010952	10:48071763-48072825	2.00421	0	1.36478	OK
ENSBTAG00000004130	ENSBTAG00000004130	15:19619433-19620030	2.00006	0.346829	3.46829	OK
ENSBTAG00000009433	ENSBTAG00000009433	3:14603347-14604401	1.98662	0	3.23526	OK
ENSBTAG00000012002	ENSBTAG00000012002	18:2613212-2625218	1.98529	0.077841	1.08977	OK
ENSBTAG00000007687	ENSBTAG00000007687	14:70399570-70416866	1.97643	0.122446	1.46936	OK
ENSBTAG00000003739	ENSBTAG00000003739	28:13411063-13411667	1.97189	0.34281	3.4281	OK
ENSBTAG00000019707	ENSBTAG00000019707	22:60016984-60024586	1.96514	0.137078	1.09663	OK
ENSBTAG00000010185	ENSBTAG00000010185	6:118330599-118331622	1.95504	0	1.82162	OK
ENSBTAG00000031279	ENSBTAG00000031279	17:72064669-72068000	1.93482	0	2.71423	OK
ENSBTAG00000011000	ENSBTAG00000011000	5:119814027-119819426	1.93269	0	6.34489	OK
ENSBTAG00000012407	ENSBTAG00000012407	13:41910590-41911910	1.93255	0.156861	1.88234	OK
ENSBTAG00000046848	ENSBTAG00000046848	26:19219742-19222118	1.92394	0	2.16652	OK
ENSBTAG00000042596	ENSBTAG00000042596	7:94200002-94200314	1.92186	0	3.31822	OK
ENSBTAG00000019764	ENSBTAG00000019764	15:27871671-27873811	1.91494	0	1.41497	OK
ENSBTAG00000007434	ENSBTAG00000007434	19:44356958-44368596	1.91484	1.30966	3.33963	OK
ENSBTAG00000030862	ENSBTAG00000030862	1:146010005-146010491	1.91375	0	3.33963	OK
ENSBTAG00000046879	ENSBTAG00000046879	18:34908885-34910089	1.9109	0	1.54777	OK
ENSBTAG00000020556	ENSBTAG00000020556	18:53054656-53057035	1.90806	0	3.32431	OK
ENSBTAG00000037984	ENSBTAG00000037984	25:27001813-27002052	1.90776	0	3.46539	OK
ENSBTAG00000014547	ENSBTAG00000014547	22:370971-373326	1.88776	0.243596	2.92316	OK
ENSBTAG00000018016	ENSBTAG00000018016	25:26340078-26341408	1.88727	0	3.2522	OK
ENSBTAG00000046012	ENSBTAG00000046012	X:3969067-3970030	1.87545	0	1.50509	OK
ENSBTAG00000046554	ENSBTAG00000046554	9:65961245-65962157	1.87292	0.231349	3.93293	OK
ENSBTAG00000025550	ENSBTAG00000025550	7:19771745-19773070	1.87024	0.159397	1.91277	OK
ENSBTAG00000019683	ENSBTAG00000019683	2:47068950-47099371	1.86418	0.552889	3.04089	OK
ENSBTAG00000045275	ENSBTAG00000045275	1:107819767-107820090	1.85873	0	3.20522	OK
ENSBTAG00000018749	ENSBTAG00000018749	1:140044680-140046241	1.853	0.449581	3.21902	OK

ENSBTAG00000033278	ENSBTAG00000033278	7:6097837-6099711	1.84822	0.130553	1.56664	OK
ENSBTAG00000040334	ENSBTAG00000040334	7:45556209-45557580	1.84526	0	9.2263	OK
ENSBTAG00000008551	ENSBTAG00000008551	8:18568933-18569798	1.84047	0	1.67561	OK
ENSBTAG00000031468	ENSBTAG00000031468	17:68596868-68597323	1.84024	0	3.18549	OK
ENSBTAG00000015913	ENSBTAG00000015913	27:24100832-24103670	1.83752	0.223845	1.41769	OK
ENSBTAG00000019221	ENSBTAG00000019221	X:22539301-22539972	1.82832	0.357611	3.2185	OK
ENSBTAG00000040378	ENSBTAG00000040378	23:31111554-31112327	1.82112	0	3.18549	OK
ENSBTAG00000016370	ENSBTAG00000016370	19:27784051-27785620	1.81141	0.395902	2.50738	OK
ENSBTAG00000030650	ENSBTAG00000030650	5:75810520-75817101	1.81039	0.169302	2.03163	OK
ENSBTAG00000002481	ENSBTAG00000002481	25:624817-627214	1.80746	0	1.77647	OK
ENSBTAG00000016630	ENSBTAG00000016630	21:24335502-24336999	1.80299	0	5.40898	OK
ENSBTAG00000027357	ENSBTAG00000027357	7:44876206-44878971	1.80244	0	2.30063	OK
ENSBTAG00000046066	ENSBTAG00000046066	7:41476353-41476823	1.8016	0	3.52437	OK
ENSBTAG00000020517	ENSBTAG00000020517	19:35427014-35432081	1.79988	0.309734	2.47787	OK
ENSBTAG00000032140	ENSBTAG00000032140	25:26077326-26081192	1.79946	0	6.16073	OK
ENSBTAG00000040555	ENSBTAG00000040555	25:27322440-27341960	1.78668	0.0993079	1.19169	OK
ENSBTAG00000022204	ENSBTAG00000022204	X:57664212-57664899	1.77966	0.301393	3.01393	OK
ENSBTAG00000006650	ENSBTAG00000006650	25:27431638-27440674	1.77714	0.169095	1.43731	OK
ENSBTAG00000027013	ENSBTAG00000027013	21:46199213-46200877	1.77308	0	1.1199	OK
ENSBTAG00000048156	ENSBTAG00000048156	21:19391573-19391762	1.7721	0	3.28662	OK
ENSBTAG00000016567	ENSBTAG00000016567	25:216921-217650	1.76713	0	2.92808	OK
ENSBTAG00000045910	ENSBTAG00000045910	17:74969196-74976403	1.74639	0	2.94593	OK
ENSBTAG00000046922	ENSBTAG00000046922	4:27854573-27855179	1.73077	0	2.39175	OK
ENSBTAG00000006368	ENSBTAG00000006368	7:40573897-40576306	1.72574	0	1.14186	OK
ENSBTAG00000018019	ENSBTAG00000018019	21:65841996-65938304	1.71809	0.0762641	0.915169	OK
ENSBTAG00000016354	ENSBTAG00000016354	7:13543663-13545435	1.71715	0.116849	1.40219	OK
ENSBTAG00000040313	ENSBTAG00000040313	3:92098623-92100051	1.71404	0.289996	2.31997	OK
ENSBTAG00000005572	ENSBTAG00000005572	25:2556669-2565296	1.70994	0.091295	1.27813	OK
ENSBTAG00000008684	ENSBTAG00000008684	29:24833895-24834951	1.70871	0.196077	2.35292	OK
ENSBTAG00000004246	ENSBTAG00000004246	16:45098448-45116962	1.70266	0.173415	1.38732	OK

ENSBTAG00000008500	ENSBTAG00000008500	18:2590735-2595390	1.70244	0	2.15684	OK
ENSBTAG00000022020	ENSBTAG00000022020	17:74749249-74750524	1.70093	0	1.13678	OK
ENSBTAG00000048083	ENSBTAG00000048083	19:41866874-41867171	1.6919	0	2.78865	OK
ENSBTAG00000034033	ENSBTAG00000034033	12:22819644-23026440	1.68359	0	2.89301	OK
ENSBTAG00000044853	ENSBTAG00000044853	5:104262199-104262521	1.67568	0	3.21517	OK
ENSBTAG00000018270	ENSBTAG00000018270	13:80015600-80114072	1.67442	0.150205	1.20164	OK
ENSBTAG00000017645	ENSBTAG00000017645	19:50609317-50609792	1.67439	0	3.05137	OK
ENSBTAG00000016189	ENSBTAG00000016189	16:55466078-55466692	1.66273	0	3.03504	OK
ENSBTAG00000037896	ENSBTAG00000037896	16:29717219-29721104	1.6569	0	1.43457	OK
ENSBTAG00000002738	ENSBTAG00000002738	7:15974815-15982578	1.65506	0.384435	1.84529	OK
ENSBTAG00000038854	ENSBTAG00000038854	4:103845269-103862938	1.65066	0.20936	2.51232	OK
ENSBTAG00000004119	ENSBTAG00000004119	19:40634438-40635587	1.64745	0	1.26144	OK
ENSBTAG00000011543	ENSBTAG00000011543	3:121284544-121294475	1.6452	0.123764	1.48517	OK
ENSBTAG00000023670	ENSBTAG00000023670	5:25408217-25408935	1.64116	0.309502	2.78552	OK
ENSBTAG00000019800	ENSBTAG00000019800	2:128629674-128656311	1.64069	0	1.49115	OK
ENSBTAG00000033690	ENSBTAG00000033690	2:103278271-103371020	1.64066	0.898445	2.41889	OK
ENSBTAG00000013574	ENSBTAG00000013574	13:58663304-58673817	1.63656	0	1.84872	OK
ENSBTAG00000010665	ENSBTAG00000010665	10:20579088-20580627	1.62754	0.218645	2.62374	OK
ENSBTAG00000039880	ENSBTAG00000039880	19:42004074-42004734	1.62641	0	2.30063	OK
ENSBTAG00000024204	ENSBTAG00000024204	4:114581779-114584311	1.62495	0	2.73885	OK
ENSBTAG00000047749	ENSBTAG00000047749	27:12356886-12383676	1.61512	0	5.6225	OK
ENSBTAG00000047214	ENSBTAG00000047214	19:28221789-28223283	1.61503	0.264103	2.90514	OK
ENSBTAG00000003556	ENSBTAG00000003556	21:69978022-69980890	1.60826	0	1.66025	OK
ENSBTAG00000004105	ENSBTAG00000004105	11:28771283-28775978	1.60685	0	2.64328	OK
ENSBTAG00000001672	ENSBTAG00000001672	19:42112690-42113449	1.60549	0	2.45522	OK
ENSBTAG00000004820	ENSBTAG00000004820	5:110249248-110257521	1.60543	0	3.19095	OK
ENSBTAG00000007246	ENSBTAG00000007246	25:1194759-1207350	1.60086	0	1.21719	OK
ENSBTAG00000001937	ENSBTAG00000001937	19:51604151-51609338	1.60018	0	2.16185	OK
ENSBTAG00000047342	ENSBTAG00000047342	25:31086699-31086912	1.59661	0	2.9163	OK
ENSBTAG00000000923	ENSBTAG00000000923	5:26245921-26252315	1.59479	0	2.00378	OK

ENSBTAG00000021858	ENSBTAG00000021858	15:58144414-58195706	1.58902	0.920568	2.26601	OK
ENSBTAG00000018268	ENSBTAG00000018268	11:72326390-72326953	1.58719	0	2.57442	OK
ENSBTAG00000003033	ENSBTAG00000003033	8:90017452-90018996	1.58681	0	1.72707	OK
ENSBTAG00000039176	ENSBTAG00000039176	1:122615297-122615492	1.58363	0	3.18549	OK
ENSBTAG00000004487	ENSBTAG00000004487	16:79921421-79921775	1.58009	0	2.92453	OK
ENSBTAG00000047718	ENSBTAG00000047718	17:73195724-73197071	1.57323	0	1.07602	OK
ENSBTAG00000034796	ENSBTAG00000034796	22:49687728-49717832	1.57151	0.125947	1.76326	OK
ENSBTAG00000012038	ENSBTAG00000012038	25:36233861-36236129	1.56443	0.0941596	1.12992	OK
ENSBTAG00000002419	ENSBTAG00000002419	3:9762000-9765432	1.56386	3.13723	5.7918	OK
ENSBTAG00000031993	ENSBTAG00000031993	17:65711148-65713710	1.55867	0	1.20182	OK
ENSBTAG00000018542	ENSBTAG00000018542	11:2916016-2918064	1.55728	0	3.0904	OK
ENSBTAG00000023318	ENSBTAG00000023318	11:61716756-61717260	1.55534	0	2.87579	OK
ENSBTAG00000045839	ENSBTAG00000045839	6:103192776-103193052	1.55186	0	3.00083	OK
ENSBTAG00000013766	ENSBTAG00000013766	18:52261041-52264944	1.54845	0	1.17424	OK
ENSBTAG00000001931	ENSBTAG00000001931	5:121181281-121188623	1.54032	0	1.33013	OK
ENSBTAG00000046322	ENSBTAG00000046322	17:75145210-75145929	1.53369	0	7.66844	OK
ENSBTAG00000016041	ENSBTAG00000016041	18:50838733-50840531	1.53004	0	1.27989	OK
ENSBTAG00000038584	ENSBTAG00000038584	11:106675879-106712955	1.51256	0.361485	2.6627	OK
ENSBTAG00000045656	ENSBTAG00000045656	18:46277660-46278152	1.50811	0	2.94593	OK
ENSBTAG00000001724	ENSBTAG00000001724	24:3761517-3762444	1.50735	0.223362	2.68035	OK
ENSBTAG00000002737	ENSBTAG00000002737	25:540853-543387	1.50053	0	0.876829	OK
ENSBTAG00000008501	ENSBTAG00000008501	3:82580747-82582193	1.49907	0	1.00235	OK
ENSBTAG00000009107	ENSBTAG00000009107	19:53360153-53361992	1.49794	0	1.7335	OK
ENSBTAG00000013242	ENSBTAG00000013242	11:104455974-104464046	1.49637	0.160385	1.92462	OK
ENSBTAG00000010003	ENSBTAG00000010003	20:3064509-3066676	1.49151	0	1.65456	OK
ENSBTAG00000031746	ENSBTAG00000031746	23:31298383-31299443	1.48887	0.58601	2.53938	OK
ENSBTAG00000039921	ENSBTAG00000039921	7:8175672-8176623	1.48257	0.435451	2.39498	OK
ENSBTAG00000014104	ENSBTAG00000014104	9:50515450-50515678	1.48027	0	2.72443	OK
ENSBTAG00000007483	ENSBTAG00000007483	17:65206691-65215132	1.47975	0	2.31205	OK
ENSBTAG00000045637	ENSBTAG00000045637	1:131169755-131170538	1.47543	0	2.49466	OK

ENSBTAG00000047078	ENSBTAG00000047078	18:56032584-56033542	1.47119	0	2.17627	OK
ENSBTAG00000047650	ENSBTAG00000047650	5:56548138-56556988	1.47011	0	1.39768	OK
ENSBTAG00000033890	ENSBTAG00000033890	17:73034039-73036903	1.46899	0	1.3763	OK
ENSBTAG00000020117	ENSBTAG00000020117	7:21189878-21196263	1.46167	0.118998	1.42798	OK
ENSBTAG00000016736	ENSBTAG00000016736	19:45194103-45196727	1.45519	0	2.76603	OK
ENSBTAG00000011580	ENSBTAG00000011580	22:51187153-51200326	1.45249	0.30812	1.61763	OK
ENSBTAG00000031849	ENSBTAG00000031849	17:66586627-66593115	1.44884	0.124658	1.74521	OK
ENSBTAG00000045977	ENSBTAG00000045977	17:65369276-65369579	1.44857	0	2.73343	OK
ENSBTAG00000006034	ENSBTAG00000006034	X:4067504-4068784	1.44822	0.161763	2.26469	OK
ENSBTAG00000001341	ENSBTAG00000001341	5:108637812-108649951	1.44811	0	1.32729	OK
ENSBTAG00000043818	ENSBTAG00000043818	3:22072710-22072995	1.44337	0	2.17955	OK
ENSBTAG00000022032	ENSBTAG00000022032	17:73206770-73208629	1.44299	0	2.13461	OK
ENSBTAG00000005741	ENSBTAG00000005741	2:24489073-24491347	1.43918	0	1.47781	OK
ENSBTAG00000032880	ENSBTAG00000032880	25:9995789-9996533	1.43692	0	2.69907	OK
ENSBTAG00000004232	ENSBTAG00000004232	22:53346357-53351626	1.43654	0	2.62097	OK
ENSBTAG00000016950	ENSBTAG00000016950	29:41657197-41661822	1.43519	0	1.30316	OK
ENSBTAG00000038488	ENSBTAG00000038488	X:140973701-140975767	1.43309	0.337226	2.69781	OK
ENSBTAG00000045594	ENSBTAG00000045594	21:71417178-71418135	1.43092	0	1.51452	OK
ENSBTAG00000000694	ENSBTAG00000000694	15:47073271-47074686	1.4295	0.271017	2.43915	OK
ENSBTAG00000046243	ENSBTAG00000046243	16:64648456-64650151	1.42875	0.122157	1.46589	OK
ENSBTAG00000017456	ENSBTAG00000017456	25:36603210-36609285	1.42622	0.0815827	1.14216	OK
ENSBTAG00000039477	ENSBTAG00000039477	9:22454646-22455909	1.42501	0	1.47547	OK
ENSBTAG00000015659	ENSBTAG00000015659	11:2335381-2342308	1.42211	0.882847	2.00112	OK
ENSBTAG00000047528	ENSBTAG00000047528	3:18983856-18984897	1.41664	0	1.39231	OK
ENSBTAG00000003713	ENSBTAG00000003713	29:44418399-44419658	1.41285	0	2.23328	OK
ENSBTAG00000005286	ENSBTAG00000005286	13:66334679-66351481	1.41	0	2.31205	OK
ENSBTAG00000032812	ENSBTAG00000032812	14:45039059-45039371	1.40667	0	2.65458	OK
ENSBTAG00000016257	ENSBTAG00000016257	17:74560554-74570229	1.40663	0	0.978222	OK
ENSBTAG00000005382	ENSBTAG00000005382	29:44671367-44672651	1.40585	0	1.12882	OK
ENSBTAG00000045499	ENSBTAG00000045499	23:16856944-16859269	1.40523	0	1.50041	OK

ENSBTAG00000047402	ENSBTAG00000047402	28:43774961-43778784	1.40184	0	1.95747	OK
ENSBTAG00000011760	ENSBTAG00000011760	23:17644486-17647839	1.40094	0.163037	2.28252	OK
ENSBTAG00000005922	ENSBTAG00000005922	22:59648571-59675093	1.39644	0	0.864739	OK
ENSBTAG00000007047	ENSBTAG00000007047	17:47935476-47937432	1.39167	0.211715	1.79957	OK
ENSBTAG00000030757	ENSBTAG00000030757	25:41817961-41820367	1.38531	0	0.774527	OK
ENSBTAG00000005351	ENSBTAG00000005351	12:24829485-24838271	1.38452	0.193692	2.32431	OK
ENSBTAG00000047413	ENSBTAG00000047413	8:23023862-23024450	1.38278	0	2.46496	OK
ENSBTAG00000007142	ENSBTAG00000007142	25:11494717-11602903	1.37738	0.473544	2.36772	OK
ENSBTAG00000017027	ENSBTAG00000017027	3:101167827-101172831	1.37712	0.311598	2.33699	OK
ENSBTAG00000017242	ENSBTAG00000017242	19:57141971-57157895	1.37608	0.154405	1.85286	OK
ENSBTAG00000039955	ENSBTAG00000039955	4:27890075-27890570	1.3745	0	2.50978	OK
ENSBTAG00000024995	ENSBTAG00000024995	19:41903869-41904340	1.37393	0	2.63767	OK
ENSBTAG00000012019	ENSBTAG00000012019	12:88664524-88669181	1.37353	0	0.524933	OK
ENSBTAG00000001794	ENSBTAG00000001794	7:19833363-19834615	1.37146	0	2.63049	OK
ENSBTAG00000047222	ENSBTAG00000047222	10:41943806-41944589	1.36713	0	2.37997	OK
ENSBTAG00000019745	ENSBTAG00000019745	25:724445-775899	1.36499	0.119617	1.67464	OK
ENSBTAG00000048160	ENSBTAG00000048160	14:2311789-2313733	1.36308	0	0.745575	OK
ENSBTAG00000012796	ENSBTAG00000012796	18:52165403-52174453	1.36298	0	1.80949	OK
ENSBTAG00000021015	ENSBTAG00000021015	26:46963426-47002009	1.36054	0	3.62811	OK
ENSBTAG00000001711	ENSBTAG00000001711	19:28443497-28445326	1.35995	0.113208	1.58491	OK
ENSBTAG00000047722	ENSBTAG00000047722	4:106477552-106478019	1.35564	0	2.17955	OK
ENSBTAG00000024552	ENSBTAG00000024552	4:118265404-118274566	1.35556	0	1.03455	OK
ENSBTAG00000031532	ENSBTAG00000031532	9:104124501-104134436	1.35523	0	0.671951	OK
ENSBTAG00000030504	ENSBTAG00000030504	5:95570689-95571095	1.35448	0	2.54996	OK
ENSBTAG00000011924	ENSBTAG00000011924	17:63439742-63448400	1.35303	0	1.63753	OK
ENSBTAG00000034064	ENSBTAG00000034064	X:118633279-118636998	1.35214	0.281902	2.39617	OK
ENSBTAG00000022580	ENSBTAG00000022580	3:31719423-31733085	1.34882	0.142307	1.70769	OK
ENSBTAG00000010493	ENSBTAG00000010493	18:50040252-50043146	1.34779	0.175919	2.11103	OK
ENSBTAG00000012149	ENSBTAG00000012149	5:26180270-26182515	1.34672	0.24331	2.4331	OK
ENSBTAG00000001262	ENSBTAG00000001262	18:52152025-52155244	1.34307	0	6.71533	OK

ENSBTAG00000024895	ENSBTAG00000024895	18:63766671-63767001	1.341	0	2.50978	OK
ENSBTAG00000048147	ENSBTAG00000048147	X:128040289-128040604	1.33967	0	2.62929	OK
ENSBTAG00000006657	ENSBTAG00000006657	17:75039313-75050946	1.33879	0	0.762173	OK
ENSBTAG00000006173	ENSBTAG00000006173	17:74110523-74113523	1.33844	0	0.948352	OK
ENSBTAG00000000397	ENSBTAG00000000397	4:118042854-118048886	1.33698	0	2.17955	OK
ENSBTAG00000012319	ENSBTAG00000012319	X:62455884-62457281	1.33692	0.148215	2.07502	OK
ENSBTAG00000046663	ENSBTAG00000046663	7:1560273-1562213	1.3348	0	2.00193	OK
ENSBTAG00000030322	ENSBTAG00000030322	2:134776471-134778862	1.33477	0.0865985	1.03918	OK
ENSBTAG00000046813	ENSBTAG00000046813	7:15690974-15692145	1.33435	0	6.67175	OK
ENSBTAG00000014162	ENSBTAG00000014162	22:15553513-15561007	1.33131	0.30904	2.3178	OK
ENSBTAG00000010148	ENSBTAG00000010148	9:42546052-42546652	1.33056	0	2.41566	OK
ENSBTAG00000002245	ENSBTAG00000002245	29:26158414-26159440	1.33029	0.20181	2.42172	OK
ENSBTAG00000009481	ENSBTAG00000009481	3:86260318-86342617	1.32458	0.841353	2.77646	OK
ENSBTAG00000020742	ENSBTAG00000020742	25:34245428-34247198	1.32429	0	1.05283	OK
ENSBTAG00000046109	ENSBTAG00000046109	26:423898-424648	1.32419	0	2.48468	OK
ENSBTAG00000046794	ENSBTAG00000046794	X:104334441-104334810	1.32102	0	2.80565	OK
ENSBTAG00000019600	ENSBTAG00000019600	9:12981372-12982550	1.32017	0	2.39372	OK
ENSBTAG00000016991	ENSBTAG00000016991	12:86242383-86263272	1.3195	0.322268	2.25587	OK
ENSBTAG00000044192	ENSBTAG00000044192	18:6551585-6552719	1.31932	0	1.43363	OK
ENSBTAG00000031866	ENSBTAG00000031866	4:93379462-93382232	1.31536	0	1.75048	OK
ENSBTAG00000022460	ENSBTAG00000022460	29:37575177-37575612	1.30836	0	2.37997	OK
ENSBTAG00000014967	ENSBTAG00000014967	2:134512628-134616219	1.30424	0	1.22761	OK
ENSBTAG00000020733	ENSBTAG00000020733	25:16531760-16537583	1.30407	0	2.70663	OK
ENSBTAG00000047705	ENSBTAG00000047705	9:58826-59069	1.30359	0	2.55626	OK
ENSBTAG00000045019	ENSBTAG00000045019	28:19729639-19729924	1.29863	0	2.17955	OK
ENSBTAG00000031962	ENSBTAG00000031962	12:89182099-89212365	1.29723	0	1.51611	OK
ENSBTAG00000004109	ENSBTAG00000004109	18:11076774-11077340	1.29639	0	2.56078	OK
ENSBTAG00000024641	ENSBTAG00000024641	X:140282267-140284361	1.29405	0.0988811	1.18657	OK
ENSBTAG00000010371	ENSBTAG00000010371	10:36598393-36600764	1.29368	0.13813	1.65756	OK
ENSBTAG00000016091	ENSBTAG00000016091	21:16946193-16960419	1.28874	0	0.925279	OK

ENSBTAG00000027407	ENSBTAG00000027407	13:54526442-54527951	1.28637	0	1.42377	OK
ENSBTAG00000004300	ENSBTAG00000004300	7:17877527-17884402	1.28631	0.168066	1.34453	OK
ENSBTAG00000013819	ENSBTAG00000013819	12:32300109-32309281	1.28207	0.224818	2.24818	OK
ENSBTAG00000019519	ENSBTAG00000019519	26:51657460-51674652	1.28112	0	1.10072	OK
ENSBTAG00000027172	ENSBTAG00000027172	3:30318708-30319287	1.28057	0	2.50328	OK
ENSBTAG00000011677	ENSBTAG00000011677	23:31636946-31637588	1.27956	0	2.25763	OK
ENSBTAG00000021111	ENSBTAG00000021111	23:27769892-27774392	1.27766	0	1.72069	OK
ENSBTAG00000046344	ENSBTAG00000046344	13:66538446-66538939	1.27609	0	2.51996	OK
ENSBTAG00000047321	ENSBTAG00000047321	3:17814054-17814288	1.2751	0	2.65458	OK
ENSBTAG00000001751	ENSBTAG00000001751	6:21984883-21985969	1.2731	0	6.36552	OK
ENSBTAG00000031905	ENSBTAG00000031905	23:27646133-27646571	1.2714	0	2.36366	OK
ENSBTAG00000045990	ENSBTAG00000045990	27:22394849-22395496	1.27049	0	2.31904	OK
ENSBTAG00000024926	ENSBTAG00000024926	19:54546913-54552469	1.27021	0	2.19606	OK
ENSBTAG00000006722	ENSBTAG00000006722	X:79946793-79954814	1.26951	0	2.32648	OK
ENSBTAG00000009972	ENSBTAG00000009972	2:108226857-108229830	1.26881	0	1.63897	OK
ENSBTAG00000020340	ENSBTAG00000020340	19:15446156-15447896	1.26862	0.356995	2.14197	OK
ENSBTAG00000031915	ENSBTAG00000031915	15:51529606-51530191	1.26522	0	2.4776	OK
ENSBTAG00000013929	ENSBTAG00000013929	18:34746054-34749250	1.26372	0	1.30134	OK
ENSBTAG00000030317	ENSBTAG00000030317	11:106462137-106465659	1.26368	0.0587896	0.705475	OK
ENSBTAG00000039302	ENSBTAG00000039302	7:15880797-15884598	1.26201	0	1.29952	OK
ENSBTAG00000011050	ENSBTAG00000011050	8:40114095-40115242	1.25673	0.18052	2.16625	OK
ENSBTAG00000024974	ENSBTAG00000024974	19:45452266-45459216	1.25584	0	1.47663	OK
ENSBTAG00000046621	ENSBTAG00000046621	23:31674089-31674743	1.25388	0	2.21621	OK
ENSBTAG00000046868	ENSBTAG00000046868	14:253709-254630	1.25227	0	2.78552	OK
ENSBTAG00000034498	ENSBTAG00000034498	14:2801382-2803020	1.25129	0	2.08547	OK
ENSBTAG00000018785	ENSBTAG00000018785	13:57874076-57879042	1.2509	0	1.37732	OK
ENSBTAG00000024701	ENSBTAG00000024701	15:82106826-82109417	1.24925	0	2.17955	OK
ENSBTAG00000019589	ENSBTAG00000019589	1:145801879-145813313	1.24767	0.108067	1.29681	OK
ENSBTAG00000005063	ENSBTAG00000005063	14:2839179-2842655	1.24425	0	0.878601	OK
ENSBTAG00000043714	ENSBTAG00000043714	7:78700288-78700591	1.24215	0	2.05007	OK

ENSBTAG00000046620	ENSBTAG00000046620	1:140578415-140578628	1.24165	0	2.9163	OK
ENSBTAG00000017412	ENSBTAG00000017412	24:7303046-7304704	1.24013	0	1.08326	OK
ENSBTAG00000005536	ENSBTAG00000005536	23:16625356-16626996	1.23995	0	3.30655	OK
ENSBTAG00000009795	ENSBTAG00000009795	9:82549525-82550198	1.23995	0	2.17193	OK
ENSBTAG00000045805	ENSBTAG00000045805	19:37319925-37325265	1.23877	0	3.30337	OK
ENSBTAG00000038958	ENSBTAG00000038958	8:22683542-22684089	1.23746	0	2.33085	OK
ENSBTAG00000027854	ENSBTAG00000027854	25:307202-309684	1.23617	0	1.70517	OK
ENSBTAG00000008494	ENSBTAG00000008494	20:8419596-8430125	1.23517	0	2.47034	OK
ENSBTAG00000025792	ENSBTAG00000025792	8:77332260-77338917	1.23351	0	4.2601	OK
ENSBTAG00000013030	ENSBTAG00000013030	11:99411392-99437846	1.23262	0.186538	1.18141	OK
ENSBTAG00000039388	ENSBTAG00000039388	X:20577361-20578873	1.23213	0.410827	1.91719	OK
ENSBTAG00000005010	ENSBTAG00000005010	7:40136163-40140496	1.23207	0	1.76636	OK
ENSBTAG00000047482	ENSBTAG00000047482	4:93350247-93358141	1.23185	0	3.28494	OK
ENSBTAG00000021928	ENSBTAG00000021928	11:104176567-104180741	1.23139	0	1.17265	OK
ENSBTAG00000038630	ENSBTAG00000038630	X:128884988-128886938	1.22986	0	0.743281	OK
ENSBTAG00000009634	ENSBTAG00000009634	5:26157430-26158878	1.2296	0	2.20567	OK
ENSBTAG00000001503	ENSBTAG00000001503	25:27552789-27561099	1.22898	0	1.25998	OK
ENSBTAG00000021995	ENSBTAG00000021995	5:108751107-108756773	1.22543	0	1.28152	OK
ENSBTAG00000020674	ENSBTAG00000020674	23:27529855-27531864	1.22441	0	1.58925	OK
ENSBTAG00000017191	ENSBTAG00000017191	13:44229669-44230470	1.22288	0	2.32648	OK
ENSBTAG00000001006	ENSBTAG00000001006	26:43257907-43259617	1.22187	0	1.47297	OK
ENSBTAG00000018881	ENSBTAG00000018881	3:29343181-29402185	1.22088	0.271194	2.16956	OK
ENSBTAG00000003079	ENSBTAG00000003079	29:44221928-44223344	1.2199	0	1.31604	OK
ENSBTAG00000039049	ENSBTAG00000039049	X:132948150-132949722	1.21949	0.131716	1.84402	OK
ENSBTAG00000001538	ENSBTAG00000001538	3:117506246-117506811	1.21848	0	2.19883	OK
ENSBTAG00000038127	ENSBTAG00000038127	4:106552208-106552634	1.21789	0	1.83236	OK
ENSBTAG00000045857	ENSBTAG00000045857	25:578684-583777	1.21723	0	1.64517	OK
ENSBTAG00000037933	ENSBTAG00000037933	1:4846977-4847475	1.21608	0	2.19806	OK
ENSBTAG00000018379	ENSBTAG00000018379	6:109036387-109056287	1.21475	0.115804	1.38964	OK
ENSBTAG00000010101	ENSBTAG00000010101	23:15696924-15706220	1.21468	0.108237	1.51531	OK

ENSBTAG00000019253	ENSBTAG00000019253	X:55209957-55213506	1.21365	0	6.06824	OK
ENSBTAG00000003476	ENSBTAG00000003476	7:20565300-20568880	1.21271	0.115674	0.925394	OK
ENSBTAG00000025283	ENSBTAG00000025283	18:14983395-14983970	1.211	0	2.16059	OK
ENSBTAG00000046396	ENSBTAG00000046396	24:39737548-39739323	1.21065	0	0.86583	OK
ENSBTAG00000009676	ENSBTAG00000009676	3:121065393-121074671	1.2103	0	4.09784	OK
ENSBTAG00000024340	ENSBTAG00000024340	4:67651906-67654339	1.20784	0	0.765932	OK
ENSBTAG00000008638	ENSBTAG00000008638	13:60798591-60812321	1.20598	0.119548	1.67367	OK
ENSBTAG00000048063	ENSBTAG00000048063	8:22700612-22701200	1.20562	0	2.11283	OK
ENSBTAG00000001187	ENSBTAG00000001187	17:70724860-70727762	1.20412	0.134017	1.60821	OK
ENSBTAG00000004563	ENSBTAG00000004563	X:118800901-118802822	1.20388	0.330234	1.98141	OK
ENSBTAG00000003102	ENSBTAG00000003102	4:92617108-92620507	1.20321	0.088942	1.24519	OK
ENSBTAG00000010731	ENSBTAG00000010731	7:16298107-16300588	1.20168	0.167522	2.01026	OK
ENSBTAG00000013133	ENSBTAG00000013133	29:45942346-45947879	1.19907	0	0.94787	OK
ENSBTAG00000017365	ENSBTAG00000017365	22:15431364-15437006	1.19731	0.121727	1.46072	OK
ENSBTAG00000039658	ENSBTAG00000039658	10:12021447-12030315	1.19625	0.120172	1.68241	OK
ENSBTAG00000011933	ENSBTAG00000011933	14:26115607-26116813	1.19426	0	1.88234	OK
ENSBTAG00000037469	ENSBTAG00000037469	X:123975692-123977484	1.19338	0.200637	2.00637	OK
ENSBTAG00000030503	ENSBTAG00000030503	5:95568129-95568806	1.19243	0	2.14091	OK
ENSBTAG00000046394	ENSBTAG00000046394	19:20703987-20706890	1.18947	0	2.21847	OK
ENSBTAG00000008462	ENSBTAG00000008462	1:73468657-73469929	1.18856	0	1.13946	OK
ENSBTAG00000046044	ENSBTAG00000046044	2:121974703-121975687	1.18448	0.224088	2.01679	OK
ENSBTAG00000004077	ENSBTAG00000004077	25:34884282-34906639	1.1823	0.146433	2.05007	OK
ENSBTAG00000045690	ENSBTAG00000045690	27:41055699-41056080	1.18218	0	2.17383	OK
ENSBTAG00000030199	ENSBTAG00000030199	5:114099571-114100123	1.18186	0	2.25062	OK
ENSBTAG00000002016	ENSBTAG00000002016	2:66585180-66586232	1.17937	0.199669	1.99669	OK
ENSBTAG00000047839	ENSBTAG00000047839	X:148804070-148805135	1.17933	0	1.36094	OK
ENSBTAG00000002389	ENSBTAG00000002389	15:82355402-82363432	1.1792	0.144391	1.73269	OK
ENSBTAG00000025788	ENSBTAG00000025788	7:4950068-4953210	1.17678	0	1.72707	OK
ENSBTAG00000047545	ENSBTAG00000047545	X:83676301-83678026	1.17626	0.240066	1.92053	OK
ENSBTAG00000002505	ENSBTAG00000002505	2:126478727-126479720	1.17588	0	1.87665	OK

ENSBTAG00000004620	ENSBTAG00000004620	13:38708010-38772942	1.17357	0	2.33566	OK
ENSBTAG00000046118	ENSBTAG00000046118	1:709514-710143	1.17208	0	2.30429	OK
ENSBTAG00000002060	ENSBTAG00000002060	19:40332946-40334703	1.16969	0	2.20665	OK
ENSBTAG00000018498	ENSBTAG00000018498	X:92092354-92098095	1.1695	0.148215	1.77859	OK
ENSBTAG00000017189	ENSBTAG00000017189	11:9683453-9694986	1.16792	0	2.14726	OK
ENSBTAG00000001812	ENSBTAG00000001812	22:56829077-56860068	1.1667	0	1.80573	OK
ENSBTAG00000002050	ENSBTAG00000002050	3:94230309-94249994	1.16655	0.226539	2.03885	OK
ENSBTAG00000007228	ENSBTAG00000007228	5:111106427-111123047	1.16632	0	1.91719	OK
ENSBTAG00000019928	ENSBTAG00000019928	29:18477127-18488713	1.16594	0.239649	1.91719	OK
ENSBTAG00000038900	ENSBTAG00000038900	1:10956250-10956659	1.1633	0	2.53126	OK
ENSBTAG00000006500	ENSBTAG00000006500	22:49941345-49944165	1.16308	0.0734245	0.881093	OK
ENSBTAG00000015006	ENSBTAG00000015006	29:45127204-45130554	1.1616	0	2.25334	OK
ENSBTAG00000040141	ENSBTAG00000040141	10:24832982-24833500	1.16032	0	2.28792	OK
ENSBTAG00000002248	ENSBTAG00000002248	29:43917754-43919976	1.15971	0	1.93451	OK
ENSBTAG00000005750	ENSBTAG00000005750	13:60991999-60997978	1.15967	0	1.38433	OK
ENSBTAG00000031688	ENSBTAG00000031688	29:45315573-45323300	1.1594	0.134803	1.88724	OK
ENSBTAG00000021308	ENSBTAG00000021308	2:115790539-115794253	1.15771	0.167251	1.05926	OK
ENSBTAG00000018507	ENSBTAG00000018507	18:13860760-13865260	1.15702	0.191188	2.10307	OK
ENSBTAG00000001727	ENSBTAG00000001727	16:51553540-51556655	1.15376	0	1.78717	OK
ENSBTAG00000047514	ENSBTAG00000047514	6:103522082-103528071	1.15035	0	1.98616	OK
ENSBTAG00000001150	ENSBTAG00000001150	1:401205-407700	1.15033	0	2.08359	OK
ENSBTAG00000009549	ENSBTAG00000009549	23:17138205-17151448	1.14992	0.225062	2.02556	OK
ENSBTAG00000010464	ENSBTAG00000010464	17:69431676-69478074	1.1473	0	3.9123	OK
ENSBTAG00000045584	ENSBTAG00000045584	4:52230673-52235915	1.14681	0	2.11715	OK
ENSBTAG00000007756	ENSBTAG00000007756	18:63455107-63462751	1.14614	0	1.70517	OK
ENSBTAG00000003445	ENSBTAG00000003445	14:64025334-64034795	1.14273	0.195521	1.95521	OK
ENSBTAG00000014799	ENSBTAG00000014799	19:56957746-56972759	1.142	0.111201	1.33442	OK
ENSBTAG00000046871	ENSBTAG00000046871	3:86767073-86767697	1.13934	0	1.99093	OK
ENSBTAG00000046339	ENSBTAG00000046339	25:3521519-3532204	1.13845	0.0740812	0.888974	OK
ENSBTAG00000003567	ENSBTAG00000003567	15:47185420-47186287	1.13839	0	2.15236	OK

ENSBTAG00000017396	ENSBTAG00000017396	10:239072-239764	1.1375	0	1.9306	OK
ENSBTAG00000013882	ENSBTAG00000013882	18:65960798-65963496	1.13721	0	2.03218	OK
ENSBTAG00000044177	ENSBTAG00000044177	15:21875711-21877553	1.135	0	1.99642	OK
ENSBTAG00000018545	ENSBTAG00000018545	18:57345386-57350243	1.13492	0	1.87261	OK
ENSBTAG00000038249	ENSBTAG00000038249	11:93960721-93961672	1.13192	0	1.95953	OK
ENSBTAG00000023730	ENSBTAG00000023730	18:14761259-14769914	1.12885	0.118386	1.6574	OK
ENSBTAG00000040432	ENSBTAG00000040432	25:34018455-34019669	1.12721	0	1.1939	OK
ENSBTAG00000011814	ENSBTAG00000011814	8:85382824-85420580	1.12564	0.26614	2.12912	OK
ENSBTAG00000026194	ENSBTAG00000026194	25:41183194-41190361	1.12496	0	0.966266	OK
ENSBTAG00000016731	ENSBTAG00000016731	22:54903387-54906875	1.12434	0	1.78156	OK
ENSBTAG00000046380	ENSBTAG00000046380	15:83123743-83125170	1.11951	0.21636	2.1636	OK
ENSBTAG00000013623	ENSBTAG00000013623	7:22448899-22450073	1.11894	0	1.58925	OK
ENSBTAG00000013700	ENSBTAG00000013700	16:35698744-35700058	1.11709	0.315155	1.89093	OK
ENSBTAG00000017886	ENSBTAG00000017886	17:71012185-71014256	1.11708	0	1.91719	OK
ENSBTAG00000048059	ENSBTAG00000048059	29:26321509-26322752	1.11387	0	2.11763	OK
ENSBTAG00000039397	ENSBTAG00000039397	7:2948733-2957539	1.11241	0	1.73511	OK
ENSBTAG00000012751	ENSBTAG00000012751	21:71204290-71206428	1.11094	0.118386	1.42063	OK
ENSBTAG00000030230	ENSBTAG00000030230	19:50732353-50732953	1.10814	0	2.07057	OK
ENSBTAG00000014949	ENSBTAG00000014949	2:22485429-22487883	1.10813	0	1.00235	OK
ENSBTAG00000005774	ENSBTAG00000005774	10:86073365-86075468	1.10736	0.0984579	1.18149	OK
ENSBTAG00000045625	ENSBTAG00000045625	25:1977341-1979953	1.10676	0	0.801659	OK
ENSBTAG00000047652	ENSBTAG00000047652	15:46287139-46288701	1.10477	0	1.19303	OK
ENSBTAG00000047559	ENSBTAG00000047559	16:68936717-68937098	1.10416	0	2.17383	OK
ENSBTAG00000001784	ENSBTAG00000001784	2:131589016-131597324	1.10383	0	1.76972	OK
ENSBTAG00000047752	ENSBTAG00000047752	13:24655213-24656659	1.10371	0	1.04348	OK
ENSBTAG00000039141	ENSBTAG00000039141	14:57784158-57785076	1.10157	0.225552	1.80442	OK
ENSBTAG00000016229	ENSBTAG00000016229	8:46883355-46907760	1.10142	0.155799	1.86959	OK
ENSBTAG00000001594	ENSBTAG00000001594	17:54802291-54804869	1.10113	0.17577	1.93347	OK
ENSBTAG00000019880	ENSBTAG00000019880	17:12705275-12706621	1.10023	0	5.50115	OK
ENSBTAG00000016580	ENSBTAG00000016580	5:113028011-113037944	1.1	0	1.99733	OK

ENSBTAG00000002415	ENSBTAG00000002415	21:32706741-32719331	1.09979	0.229808	1.83846	OK
ENSBTAG00000011441	ENSBTAG00000011441	19:39447332-39447800	1.09956	0	2.21215	OK
ENSBTAG00000024773	ENSBTAG00000024773	9:51596536-51601433	1.09841	0	1.9442	OK
ENSBTAG00000007194	ENSBTAG00000007194	3:104609130-104612391	1.09822	0	1.93964	OK
ENSBTAG00000047823	ENSBTAG00000047823	26:16719279-16720929	1.09783	0.125489	1.50587	OK
ENSBTAG00000037983	ENSBTAG00000037983	18:52882808-52889162	1.09721	0	1.46503	OK
ENSBTAG00000026657	ENSBTAG00000026657	5:75509438-75532725	1.09581	0	1.98378	OK
ENSBTAG00000000666	ENSBTAG00000000666	19:42946039-42949784	1.09507	0.101748	1.22097	OK
ENSBTAG00000003887	ENSBTAG00000003887	9:24240994-24273071	1.09449	0.88942	3.11297	OK
ENSBTAG00000006072	ENSBTAG00000006072	19:36915566-36927313	1.09397	0.144896	1.73876	OK
ENSBTAG00000044097	ENSBTAG00000044097	2:95781393-95903310	1.09388	0.148428	1.78114	OK
ENSBTAG00000036058	ENSBTAG00000036058	X:32630886-32634643	1.0927	0.28599	1.85894	OK
ENSBTAG00000003818	ENSBTAG00000003818	23:15560413-15568723	1.09213	0.213461	1.92115	OK
ENSBTAG00000047609	ENSBTAG00000047609	24:43231406-43232698	1.09165	0	2.16814	OK
ENSBTAG00000027524	ENSBTAG00000027524	15:82114215-82122724	1.09161	0	1.07363	OK
ENSBTAG00000000809	ENSBTAG00000000809	20:69472638-69477632	1.09112	0	1.00166	OK
ENSBTAG00000047524	ENSBTAG00000047524	2:125010195-125011119	1.09012	0	1.56861	OK
ENSBTAG00000000059	ENSBTAG00000000059	12:91026837-91027218	1.08913	0	2.98353	OK
ENSBTAG00000010666	ENSBTAG00000010666	10:20569773-20578425	1.08884	0.24657	1.29449	OK
ENSBTAG00000018579	ENSBTAG00000018579	11:10068211-10071090	1.08726	0	2.70329	OK
ENSBTAG00000001076	ENSBTAG00000001076	11:107214413-107220027	1.08557	0.0746151	0.895381	OK
ENSBTAG00000019904	ENSBTAG00000019904	3:100411037-100415225	1.08556	0	2.44251	OK
ENSBTAG00000020400	ENSBTAG00000020400	12:36373089-36374313	1.08109	0	1.18415	OK
ENSBTAG00000001321	ENSBTAG00000001321	11:46410276-46418787	1.08092	0.3572	1.90507	OK
ENSBTAG00000023628	ENSBTAG00000023628	23:27457505-27459147	1.07868	0.1261	1.76541	OK
ENSBTAG00000020185	ENSBTAG00000020185	X:39517280-39579616	1.07847	0.123469	1.48162	OK
ENSBTAG00000016785	ENSBTAG00000016785	2:69899472-69917270	1.07673	0	1.30576	OK
ENSBTAG00000014299	ENSBTAG00000014299	3:30769965-30776469	1.07634	0.192611	1.92611	OK
ENSBTAG00000021796	ENSBTAG00000021796	17:65435168-65445334	1.07614	0.118657	1.6612	OK
ENSBTAG00000005938	ENSBTAG00000005938	3:52691875-52696492	1.07531	0	1.63037	OK

ENSBTAG00000020590	ENSBTAG00000020590	19:44954981-44956760	1.07481	0	1.04751	OK
ENSBTAG00000046549	ENSBTAG00000046549	8:85692415-85743419	1.07459	0.0798523	1.11793	OK
ENSBTAG00000019145	ENSBTAG00000019145	14:24975949-24976948	1.07265	0	1.45085	OK
ENSBTAG00000010182	ENSBTAG00000010182	5:120222128-120229501	1.07112	0.189613	1.89613	OK
ENSBTAG00000016581	ENSBTAG00000016581	25:361739-364295	1.06962	0	1.70495	OK
ENSBTAG00000031250	ENSBTAG00000031250	8:104586634-104587023	1.06866	0	2.12912	OK
ENSBTAG00000008493	ENSBTAG00000008493	8:76525368-76531334	1.06781	0.117313	1.64238	OK
ENSBTAG00000035005	ENSBTAG00000035005	10:4842898-4843791	1.06699	0	2.0868	OK
ENSBTAG00000001138	ENSBTAG00000001138	25:36305261-36306273	1.06632	0	1.43221	OK
ENSBTAG00000005719	ENSBTAG00000005719	3:111519877-111522551	1.06494	0	1.63037	OK
ENSBTAG00000001448	ENSBTAG00000001448	4:66002071-66006203	1.06395	0	2.09148	OK
ENSBTAG00000006792	ENSBTAG00000006792	10:37412315-37496211	1.06235	0.189961	1.61466	OK
ENSBTAG00000047304	ENSBTAG00000047304	29:27048846-27050521	1.06176	0.219806	1.75845	OK
ENSBTAG00000046711	ENSBTAG00000046711	23:22381967-22387980	1.06169	0	2.32648	OK
ENSBTAG00000046486	ENSBTAG00000046486	8:39981322-40111016	1.06133	0	2.03396	OK
ENSBTAG00000021021	ENSBTAG00000021021	21:33934614-33939819	1.06132	0.12742	1.01936	OK
ENSBTAG00000013170	ENSBTAG00000013170	2:92490400-92495797	1.06088	0	3.55133	OK
ENSBTAG00000039292	ENSBTAG00000039292	26:31870224-31871586	1.05963	0	1.36822	OK
ENSBTAG00000040199	ENSBTAG00000040199	1:83437864-83442294	1.05792	0.109323	1.53052	OK
ENSBTAG00000014899	ENSBTAG00000014899	15:29720789-29725202	1.05746	0	2.10975	OK
ENSBTAG00000019891	ENSBTAG00000019891	17:74708564-74711079	1.05588	0	1.97735	OK
ENSBTAG00000048275	ENSBTAG00000048275	3:113943010-113944613	1.05485	0.233172	1.86538	OK
ENSBTAG00000000929	ENSBTAG00000000929	X:134309343-134309878	1.05472	0	2.00249	OK
ENSBTAG00000003396	ENSBTAG00000003396	13:70068209-70069181	1.05461	0	1.49115	OK
ENSBTAG00000001546	ENSBTAG00000001546	7:41337277-41354610	1.05388	0.418832	1.7916	OK
ENSBTAG00000017248	ENSBTAG00000017248	19:57139505-57139865	1.05345	0	2.87579	OK
ENSBTAG00000031757	ENSBTAG00000031757	23:31460913-31461294	1.05041	0	1.63037	OK
ENSBTAG00000020503	ENSBTAG00000020503	17:54568693-54571083	1.04554	0	1.87949	OK
ENSBTAG00000038690	ENSBTAG00000038690	4:106523230-106523700	1.04375	0	1.8005	OK
ENSBTAG00000011655	ENSBTAG00000011655	13:54345093-54348720	1.04338	0	1.72342	OK

ENSBTAG00000047195	ENSBTAG00000047195	8:22729309-22729897	1.04208	0	1.76069	OK
ENSBTAG00000008973	ENSBTAG00000008973	11:100052125-100057352	1.03938	0.0999792	1.19975	OK
ENSBTAG00000020684	ENSBTAG00000020684	12:56443649-56444762	1.03648	0	1.67432	OK
ENSBTAG00000001903	ENSBTAG00000001903	11:86614776-86620757	1.03625	0	5.18123	OK
ENSBTAG00000017981	ENSBTAG00000017981	27:32912524-32915668	1.03564	0	0.951257	OK
ENSBTAG00000008370	ENSBTAG00000008370	3:21555817-21560289	1.03502	0.208517	1.66813	OK
ENSBTAG00000000332	ENSBTAG00000000332	18:51350920-51357467	1.03411	0	1.05701	OK
ENSBTAG00000003705	ENSBTAG00000003705	19:34832960-34860869	1.03346	0.0843753	1.0125	OK
ENSBTAG00000001082	ENSBTAG00000001082	2:132522952-132529468	1.03287	0	1.12618	OK
ENSBTAG00000000437	ENSBTAG00000000437	26:14917016-14937522	1.03139	0.184543	1.84543	OK
ENSBTAG00000024539	ENSBTAG00000024539	16:44994736-45006429	1.0305	0.147005	1.24954	OK
ENSBTAG00000039357	ENSBTAG00000039357	3:18484768-18486100	1.03031	0.339995	1.86997	OK
ENSBTAG00000014847	ENSBTAG00000014847	15:58532711-58609229	1.03022	0.315315	1.89189	OK
ENSBTAG00000022775	ENSBTAG00000022775	21:70707407-70715785	1.03022	0.0851736	1.02208	OK
ENSBTAG00000046583	ENSBTAG00000046583	3:91919531-91925379	1.02954	0	1.96263	OK
ENSBTAG00000046110	ENSBTAG00000046110	29:45910396-45912701	1.02764	0	1.89093	OK
ENSBTAG00000010107	ENSBTAG00000010107	8:105331887-105422354	1.02746	0.0751023	0.901227	OK
ENSBTAG00000002550	ENSBTAG00000002550	6:62506124-62506524	1.0256	0	1.55293	OK
ENSBTAG00000025764	ENSBTAG00000025764	21:45767367-45767989	1.02383	0	1.99733	OK
ENSBTAG00000014497	ENSBTAG00000014497	9:103476874-103477813	1.02011	0	1.54356	OK
ENSBTAG00000017560	ENSBTAG00000017560	18:44005706-44012657	1.01518	0.222882	1.78305	OK
ENSBTAG00000045427	ENSBTAG00000045427	26:31577817-31578073	1.01483	0	2.42645	OK
ENSBTAG00000046542	ENSBTAG00000046542	7:45360211-45363064	1.01335	0	1.8897	OK
ENSBTAG00000046011	ENSBTAG00000046011	22:5649-9019	1.01332	0.220508	1.98457	OK
ENSBTAG00000033299	ENSBTAG00000033299	25:1366646-1368479	1.01191	0	0.790725	OK
ENSBTAG00000039673	ENSBTAG00000039673	15:27883637-27884824	1.01159	0.176972	1.76972	OK
ENSBTAG00000023947	ENSBTAG00000023947	26:18711831-18719926	1.01129	0	1.41282	OK
ENSBTAG00000026896	ENSBTAG00000026896	23:51549720-51553563	1.0103	0	1.37254	OK
ENSBTAG00000004994	ENSBTAG00000004994	18:43927221-43929798	1.01011	0.0974844	1.16981	OK
ENSBTAG00000021222	ENSBTAG00000021222	25:26840151-26848526	1.00916	0	1.12531	OK

ENSBTAG00000018181	ENSBTAG00000018181	18:54124478-54127099	1.0085	0.0789992	1.10599	OK
ENSBTAG00000010799	ENSBTAG00000010799	5:57486016-57489133	1.00763	0	5.03815	OK
ENSBTAG00000007199	ENSBTAG00000007199	13:51003520-51004117	1.00741	0	1.73415	OK
ENSBTAG00000037456	ENSBTAG00000037456	2:126350163-126354957	1.0045	0.0431909	0.604672	OK
ENSBTAG00000047357	ENSBTAG00000047357	11:105878863-105879664	1.0023	0	1.80949	OK
ENSBTAG00000014472	ENSBTAG00000014472	21:55600929-55602987	1.00131	0.201222	1.71038	OK
ENSBTAG00000009769	ENSBTAG00000009769	3:84711663-84712071	1.00038	0	1.52248	OK
ENSBTAG00000038090	ENSBTAG00000038090	X:116407338-116407980	0.9989	0	1.99093	OK
ENSBTAG00000046949	ENSBTAG00000046949	6:16676234-16676579	0.997799	0	1.8005	OK
ENSBTAG00000004420	ENSBTAG00000004420	10:36180772-36187722	0.993992	0.084238	1.17933	OK
ENSBTAG00000009868	ENSBTAG00000009868	25:36174073-36176834	0.991813	0	1.06652	OK
ENSBTAG00000046054	ENSBTAG00000046054	11:78464205-78466417	0.991569	0.187212	1.4977	OK
ENSBTAG00000014848	ENSBTAG00000014848	2:126892709-126894458	0.991478	0	1.85108	OK
ENSBTAG00000046942	ENSBTAG00000046942	12:44822976-44823477	0.990207	0	1.65315	OK
ENSBTAG00000046707	ENSBTAG00000046707	X:57209585-57209885	0.990064	0	2.07057	OK
ENSBTAG00000006195	ENSBTAG00000006195	29:44691681-44693901	0.987821	0.132729	1.59275	OK
ENSBTAG00000003162	ENSBTAG00000003162	3:70246970-70275573	0.986934	0.42745	2.13725	OK
ENSBTAG00000015985	ENSBTAG00000015985	14:3639631-3640717	0.984317	0	1.33462	OK
ENSBTAG00000007768	ENSBTAG00000007768	14:68030592-68032026	0.983645	0.144391	1.73269	OK
ENSBTAG00000047351	ENSBTAG00000047351	3:59174745-59178672	0.979815	0	2.61284	OK
ENSBTAG00000001301	ENSBTAG00000001301	15:56827091-56834190	0.976864	0.103736	1.24483	OK
ENSBTAG00000021759	ENSBTAG00000021759	4:104850910-104856526	0.974447	0	1.64331	OK
ENSBTAG00000002645	ENSBTAG00000002645	7:43938529-43943848	0.971904	0	1.41928	OK
ENSBTAG00000001465	ENSBTAG00000001465	1:115834617-115836268	0.971865	0.250826	1.63037	OK
ENSBTAG00000013932	ENSBTAG00000013932	22:51435768-51439453	0.968488	0.120803	1.44964	OK
ENSBTAG00000001844	ENSBTAG00000001844	11:10149023-10151887	0.968299	0.191188	1.52951	OK
ENSBTAG00000013666	ENSBTAG00000013666	21:66852436-66865682	0.966719	0.0836594	1.17123	OK
ENSBTAG00000024769	ENSBTAG00000024769	29:28422774-28423743	0.965974	0.213681	1.92313	OK
ENSBTAG00000010992	ENSBTAG00000010992	21:25675907-25696632	0.965953	0.148004	1.77604	OK
ENSBTAG00000021743	ENSBTAG00000021743	14:45995144-46040760	0.96504	0.164592	1.64592	OK

ENSBTAG00000009217	ENSBTAG00000009217	2:107564020-107576619	0.963663	0	1.16615	OK
ENSBTAG00000010626	ENSBTAG00000010626	11:99506998-99524044	0.961116	0	0.922988	OK
ENSBTAG00000006225	ENSBTAG00000006225	2:126030986-126055499	0.960652	0.354347	1.65362	OK
ENSBTAG00000010605	ENSBTAG00000010605	11:13492654-13520241	0.960089	0	1.68339	OK
ENSBTAG00000009440	ENSBTAG00000009440	15:47310396-47311224	0.959615	0	1.75048	OK
ENSBTAG00000008700	ENSBTAG00000008700	3:18222997-18224799	0.958577	0	1.70651	OK
ENSBTAG00000017007	ENSBTAG00000017007	13:61176217-61187210	0.957603	0.104998	1.46998	OK
ENSBTAG00000018320	ENSBTAG00000018320	10:16324340-16326492	0.957342	0	1.60509	OK
ENSBTAG00000013131	ENSBTAG00000013131	2:127530912-127533980	0.956251	0	1.1297	OK
ENSBTAG00000010566	ENSBTAG00000010566	19:35816341-35825642	0.955534	0	1.64331	OK
ENSBTAG00000010802	ENSBTAG00000010802	29:44439782-44442568	0.954772	0	1.33339	OK
ENSBTAG00000001074	ENSBTAG00000001074	23:12964406-12971106	0.954725	0	1.59625	OK
ENSBTAG00000012744	ENSBTAG00000012744	18:65964092-65967276	0.954462	0	1.28874	OK
ENSBTAG00000046936	ENSBTAG00000046936	6:117990817-117992917	0.951147	0.258983	1.5539	OK
ENSBTAG00000005186	ENSBTAG00000005186	13:74458056-74462461	0.951087	0	2.28617	OK
ENSBTAG00000047148	ENSBTAG00000047148	2:127502996-127504663	0.95094	0	1.75969	OK
ENSBTAG00000027841	ENSBTAG00000027841	X:147554231-147826321	0.950582	0.195891	1.56713	OK
ENSBTAG00000032617	ENSBTAG00000032617	16:42901487-42905541	0.94845	0.231219	1.61854	OK
ENSBTAG00000032777	ENSBTAG00000032777	8:62079194-62082501	0.948055	0	1.87212	OK
ENSBTAG00000012584	ENSBTAG00000012584	3:111493038-111498704	0.947515	0.102351	1.22822	OK
ENSBTAG00000005014	ENSBTAG00000005014	11:69204180-69321396	0.946954	0	4.00155	OK
ENSBTAG00000005038	ENSBTAG00000005038	21:70903808-70905041	0.946479	0	1.17551	OK
ENSBTAG00000004076	ENSBTAG00000004076	11:25183772-25184957	0.945366	0	1.57258	OK
ENSBTAG00000009574	ENSBTAG00000009574	15:78254746-78265239	0.945207	0	1.89041	OK
ENSBTAG00000015040	ENSBTAG00000015040	14:1966291-1968825	0.94483	0	4.72415	OK
ENSBTAG00000047925	ENSBTAG00000047925	19:27388082-27388880	0.944144	0	1.85108	OK
ENSBTAG00000046593	ENSBTAG00000046593	9:23792292-23793276	0.943741	0	2.83122	OK
ENSBTAG00000026428	ENSBTAG00000026428	25:2014228-2019603	0.943586	0.181788	1.6361	OK
ENSBTAG00000046481	ENSBTAG00000046481	5:26979338-26985686	0.943036	0	2.51476	OK
ENSBTAG00000013680	ENSBTAG00000013680	19:27973742-27978543	0.942445	0.145507	1.60058	OK

ENSBTAG00000003199	ENSBTAG00000003199	15:76725869-76731444	0.941974	0.165249	1.65249	OK
ENSBTAG00000022990	ENSBTAG00000022990	21:14561843-14562829	0.941739	0	1.84051	OK
ENSBTAG00000004037	ENSBTAG00000004037	3:87841041-87843087	0.941328	0.106183	1.2742	OK
ENSBTAG00000033562	ENSBTAG00000033562	19:13611603-13617403	0.940175	0.0973927	1.16871	OK
ENSBTAG00000013532	ENSBTAG00000013532	19:45239327-45240593	0.937837	0	1.47197	OK
ENSBTAG00000016348	ENSBTAG00000016348	13:62030344-62054881	0.937231	0	1.06914	OK
ENSBTAG00000003001	ENSBTAG00000003001	15:52508415-52514771	0.935304	0	1.76469	OK
ENSBTAG00000009562	ENSBTAG00000009562	2:127523709-127524790	0.935206	0	1.66088	OK
ENSBTAG00000016649	ENSBTAG00000016649	5:106253906-106276819	0.934681	0	1.70417	OK
ENSBTAG00000014133	ENSBTAG00000014133	1:67371545-67372112	0.934374	0	1.83236	OK
ENSBTAG00000001228	ENSBTAG00000001228	29:50840060-50843011	0.933951	0	1.11855	OK
ENSBTAG00000040042	ENSBTAG00000040042	19:57194106-57204958	0.932954	0.0555261	0.777366	OK
ENSBTAG00000017381	ENSBTAG00000017381	3:104091609-104096449	0.931224	0.124733	1.4968	OK
ENSBTAG00000032792	ENSBTAG00000032792	10:27201626-27202568	0.931193	0.219806	1.75845	OK
ENSBTAG00000043523	ENSBTAG00000043523	X:11449827-11450142	0.929633	0	1.97197	OK
ENSBTAG00000005951	ENSBTAG00000005951	17:74183419-74186418	0.929602	0	0.75845	OK
ENSBTAG00000000776	ENSBTAG00000000776	29:48988148-48989096	0.926784	0.218415	1.74732	OK
ENSBTAG00000009783	ENSBTAG00000009783	10:85463045-85463552	0.926753	0	1.63359	OK
ENSBTAG00000006618	ENSBTAG00000006618	19:5696797-5747138	0.924838	0	1.8005	OK
ENSBTAG00000013095	ENSBTAG00000013095	19:20548271-20561593	0.924067	0.103528	1.24234	OK
ENSBTAG00000040075	ENSBTAG00000040075	15:51455096-51456050	0.923835	0.217041	1.73633	OK
ENSBTAG00000015124	ENSBTAG00000015124	12:86311325-86323526	0.92356	0	1.591	OK
ENSBTAG00000005583	ENSBTAG00000005583	3:103156944-103159580	0.921883	0	1.70317	OK
ENSBTAG00000002772	ENSBTAG00000002772	29:44790175-44800852	0.921049	0	0.875711	OK
ENSBTAG00000021827	ENSBTAG00000021827	9:73063428-73066402	0.920539	0.166177	1.66177	OK
ENSBTAG00000015298	ENSBTAG00000015298	19:35711778-35721867	0.918964	0	1.76972	OK
ENSBTAG00000030890	ENSBTAG00000030890	3:77154430-77154946	0.918866	0	1.60509	OK
ENSBTAG00000005432	ENSBTAG00000005432	11:49423730-49438680	0.918582	0	1.50405	OK
ENSBTAG00000011689	ENSBTAG00000011689	18:63107488-63117295	0.916726	0.0824764	0.701049	OK
ENSBTAG00000017766	ENSBTAG00000017766	4:103726164-103727095	0.916418	0	2.06598	OK

ENSBTAG00000001704	ENSBTAG00000001704	15:15674940-15676443	0.914295	0.137762	1.65315	OK
ENSBTAG00000010711	ENSBTAG00000010711	5:25306647-25307206	0.913526	0	1.48162	OK
ENSBTAG00000045041	ENSBTAG00000045041	10:80471341-80471641	0.913435	0	3.45095	OK
ENSBTAG00000020539	ENSBTAG00000020539	2:108191932-108197089	0.912971	0	0.667686	OK
ENSBTAG00000002635	ENSBTAG00000002635	18:53946096-53948160	0.912318	0	1.80573	OK
ENSBTAG00000003676	ENSBTAG00000003676	2:125600539-125600953	0.910589	0	1.50041	OK
ENSBTAG00000013744	ENSBTAG00000013744	7:64020056-64027999	0.909489	0.0781642	1.0943	OK
ENSBTAG00000016065	ENSBTAG00000016065	7:43809452-43810403	0.909141	0.217726	1.7418	OK
ENSBTAG00000008076	ENSBTAG00000008076	23:17874862-17878559	0.906287	0.181231	1.44985	OK
ENSBTAG00000008289	ENSBTAG00000008289	19:27769638-27773345	0.906239	0.136536	1.09229	OK
ENSBTAG00000021730	ENSBTAG00000021730	10:30328464-30330417	0.905116	0.214345	1.71476	OK
ENSBTAG00000025968	ENSBTAG00000025968	13:23444707-23445437	0.904829	0	1.70184	OK
ENSBTAG00000001627	ENSBTAG00000001627	2:59143224-59144149	0.903778	0	1.71324	OK
ENSBTAG00000003656	ENSBTAG00000003656	9:27991133-27991829	0.903667	0	1.53149	OK
ENSBTAG00000045837	ENSBTAG00000045837	19:47118376-47118757	0.901536	0	1.63037	OK
ENSBTAG00000001139	ENSBTAG00000001139	25:36300531-36305041	0.901253	0	3.10332	OK
ENSBTAG00000045994	ENSBTAG00000045994	6:107010341-107011306	0.899071	0	1.50979	OK
ENSBTAG00000015598	ENSBTAG00000015598	23:8434515-8441522	0.89887	0	4.49435	OK
ENSBTAG00000046768	ENSBTAG00000046768	4:76720882-76725301	0.898381	0	1.58059	OK
ENSBTAG00000036019	ENSBTAG00000036019	1:150961577-150972224	0.898197	0	4.49098	OK
ENSBTAG00000002261	ENSBTAG00000002261	26:21894401-21896298	0.897692	0	1.20083	OK
ENSBTAG00000013873	ENSBTAG00000013873	20:6360599-6365489	0.896052	0.186875	1.495	OK
ENSBTAG00000018708	ENSBTAG00000018708	21:56883744-56886411	0.895662	0.388183	1.39746	OK
ENSBTAG00000017631	ENSBTAG00000017631	25:1540862-1543188	0.895618	0	1.25598	OK
ENSBTAG00000002412	ENSBTAG00000002412	18:36674603-36696376	0.892364	0	1.59978	OK
ENSBTAG00000021071	ENSBTAG00000021071	26:23536130-23548589	0.891818	0	1.12531	OK
ENSBTAG00000014278	ENSBTAG00000014278	19:11943184-11951411	0.891335	0	0.67571	OK
ENSBTAG00000024781	ENSBTAG00000024781	15:47004018-47005492	0.891255	0	1.74416	OK
ENSBTAG00000001206	ENSBTAG00000001206	11:106397536-106399302	0.891202	0	1.50041	OK
ENSBTAG00000047969	ENSBTAG00000047969	11:10875795-10876458	0.891083	0	1.61259	OK

ENSBTAG00000013442	ENSBTAG00000013442	22:49531472-49533582	0.890559	0	2.67168	OK
ENSBTAG00000025130	ENSBTAG00000025130	19:23655530-23659389	0.89022	0	0.64076	OK
ENSBTAG00000022902	ENSBTAG00000022902	24:49617410-49621972	0.890086	0	1.65646	OK
ENSBTAG00000013854	ENSBTAG00000013854	13:43507343-43508178	0.888459	0	1.73581	OK
ENSBTAG00000005530	ENSBTAG00000005530	23:16579605-16583028	0.88791	0	1.7465	OK
ENSBTAG00000015177	ENSBTAG00000015177	29:8788702-8797308	0.887499	0.128288	1.53946	OK
ENSBTAG00000011463	ENSBTAG00000011463	X:109949359-109951138	0.886974	0.11639	1.39667	OK
ENSBTAG00000014790	ENSBTAG00000014790	9:89576711-89603963	0.886881	0.121584	1.459	OK
ENSBTAG00000019822	ENSBTAG00000019822	18:35121242-35124820	0.886166	0	1.60821	OK
ENSBTAG00000014331	ENSBTAG00000014331	X:40458244-40460872	0.885758	0	4.42879	OK
ENSBTAG00000003544	ENSBTAG00000003544	7:16248164-16252264	0.88536	0.0891718	1.24841	OK
ENSBTAG00000000044	ENSBTAG00000000044	19:51562313-51565450	0.884683	0.13201	1.12208	OK
ENSBTAG00000012792	ENSBTAG00000012792	17:65008121-65014401	0.884597	0.204804	1.63843	OK
ENSBTAG00000045759	ENSBTAG00000045759	6:108806014-108806461	0.883674	0	1.38964	OK
ENSBTAG00000047842	ENSBTAG00000047842	26:39219422-39220535	0.882729	0	1.30225	OK
ENSBTAG00000003953	ENSBTAG00000003953	25:39006992-39014435	0.882136	0	0.488013	OK
ENSBTAG00000003380	ENSBTAG00000003380	13:73866813-73872277	0.881154	0	1.4379	OK
ENSBTAG00000013395	ENSBTAG00000013395	19:22656996-22658195	0.878917	0	1.66981	OK
ENSBTAG00000045954	ENSBTAG00000045954	20:71984462-71989365	0.878739	0	0.943619	OK
ENSBTAG00000038232	ENSBTAG00000038232	6:20415892-20416639	0.878474	0	1.66311	OK
ENSBTAG00000018418	ENSBTAG00000018418	13:60487256-60492005	0.87744	0	1.63221	OK
ENSBTAG00000007398	ENSBTAG00000007398	22:190317-212803	0.87713	0	1.55682	OK
ENSBTAG00000032896	ENSBTAG00000032896	X:118593431-118594445	0.876099	0.204198	1.63359	OK
ENSBTAG00000012171	ENSBTAG00000012171	2:86340777-86341872	0.87534	0.189093	1.51274	OK
ENSBTAG00000019675	ENSBTAG00000019675	23:40588547-40606204	0.874298	0.0781347	0.937616	OK
ENSBTAG00000004886	ENSBTAG00000004886	6:68820779-68824912	0.873891	0	3.78564	OK
ENSBTAG00000017616	ENSBTAG00000017616	21:70845970-70861972	0.873065	0	1.25658	OK
ENSBTAG00000030520	ENSBTAG00000030520	7:52290535-52293580	0.872439	0.067999	0.815988	OK
ENSBTAG00000016752	ENSBTAG00000016752	4:114549023-114559742	0.872345	0	1.039	OK
ENSBTAG00000047717	ENSBTAG00000047717	19:20734087-20786818	0.871979	0.118183	1.4182	OK

ENSBTAG00000037549	ENSBTAG00000037549	25:36868137-36869514	0.87053	0	1.35331	OK
ENSBTAG00000048034	ENSBTAG00000048034	19:27204702-27206013	0.869123	0	1.51452	OK
ENSBTAG00000046376	ENSBTAG00000046376	10:61503725-61504730	0.867018	0	1.69998	OK
ENSBTAG00000016169	ENSBTAG00000016169	13:61726124-61727283	0.866977	0	1.48049	OK
ENSBTAG00000008359	ENSBTAG00000008359	1:28611817-28612240	0.865933	0	1.46849	OK
ENSBTAG00000019386	ENSBTAG00000019386	23:28355360-28358332	0.865067	0	1.4031	OK
ENSBTAG00000040496	ENSBTAG00000040496	14:38021171-38454345	0.86477	0.227036	1.4379	OK
ENSBTAG00000038268	ENSBTAG00000038268	12:80236589-80244063	0.863822	0.156624	1.56624	OK
ENSBTAG00000005950	ENSBTAG00000005950	X:33906183-33910776	0.862967	0.236772	1.42063	OK
ENSBTAG00000004572	ENSBTAG00000004572	8:104244729-104247975	0.860474	0	1.45701	OK
ENSBTAG00000015176	ENSBTAG00000015176	23:49921819-49930811	0.860073	0	1.58867	OK
ENSBTAG00000033766	ENSBTAG00000033766	19:42463452-42465605	0.859838	0	1.56417	OK
ENSBTAG00000015188	ENSBTAG00000015188	13:44945067-44952151	0.859109	0.158908	1.35072	OK
ENSBTAG00000046022	ENSBTAG00000046022	19:44445640-44446881	0.856624	0	1.385	OK
ENSBTAG00000005797	ENSBTAG00000005797	3:120634568-120644626	0.855434	0	0.957331	OK
ENSBTAG00000012737	ENSBTAG00000012737	10:100807575-100816797	0.854729	0.188319	1.60071	OK
ENSBTAG00000023765	ENSBTAG00000023765	18:7779381-7781765	0.854593	0.175621	1.40497	OK
ENSBTAG00000016944	ENSBTAG00000016944	17:73576017-73589968	0.8544	0	0.974713	OK
ENSBTAG00000038045	ENSBTAG00000038045	18:56383542-56393411	0.8531	0	2.7851	OK
ENSBTAG00000016113	ENSBTAG00000016113	18:55278947-55287983	0.853007	0	1.5406	OK
ENSBTAG00000039275	ENSBTAG00000039275	7:98715724-98767729	0.852381	0.51201	1.79204	OK
ENSBTAG00000005229	ENSBTAG00000005229	20:20575855-20576238	0.850681	0	1.81629	OK
ENSBTAG00000045904	ENSBTAG00000045904	5:120970010-120973246	0.84942	0	1.14216	OK
ENSBTAG00000032503	ENSBTAG00000032503	25:16974399-16975998	0.848432	0	0.906441	OK
ENSBTAG00000027375	ENSBTAG00000027375	23:52099149-52135349	0.848157	0	1.5281	OK
ENSBTAG00000009214	ENSBTAG00000009214	1:152880453-152890252	0.847396	0.126408	1.5169	OK
ENSBTAG00000016650	ENSBTAG00000016650	5:106223070-106238040	0.846325	0.163037	1.46733	OK
ENSBTAG00000022378	ENSBTAG00000022378	29:28317437-28318367	0.846122	0	1.78114	OK
ENSBTAG00000015275	ENSBTAG00000015275	5:110365985-110370632	0.845916	0	1.01712	OK
ENSBTAG00000040450	ENSBTAG00000040450	5:101838622-101840389	0.845793	0.11718	1.52334	OK

ENSBTAG00000039937	ENSBTAG00000039937	16:42440210-42493773	0.845614	0.121512	1.45815	OK
ENSBTAG00000013203	ENSBTAG00000013203	1:73740502-73776574	0.845447	0	1.06365	OK
ENSBTAG00000021974	ENSBTAG00000021974	5:26015048-26019918	0.844842	0	1.3635	OK
ENSBTAG00000046433	ENSBTAG00000046433	4:106459665-106465829	0.844493	0	3.36559	OK
ENSBTAG00000001067	ENSBTAG00000001067	22:49674767-49683245	0.844136	0.0790595	1.10683	OK
ENSBTAG00000005030	ENSBTAG00000005030	25:26518231-26526783	0.84284	0.159397	1.51074	OK
ENSBTAG00000007049	ENSBTAG00000007049	26:6344780-6348912	0.842226	0.148004	1.48004	OK
ENSBTAG00000046655	ENSBTAG00000046655	16:18415805-18417530	0.84211	0	1.67657	OK
ENSBTAG00000021996	ENSBTAG00000021996	4:9613133-9620559	0.84143	0.197197	1.57758	OK
ENSBTAG00000023907	ENSBTAG00000023907	1:146989243-147040968	0.841057	0.0440266	0.528319	OK
ENSBTAG00000001834	ENSBTAG00000001834	18:56666459-56671418	0.841009	0	1.13146	OK
ENSBTAG00000018170	ENSBTAG00000018170	19:50814749-50815904	0.840595	0	1.25489	OK
ENSBTAG00000007166	ENSBTAG00000007166	19:25106723-25135095	0.840402	0.101648	1.21978	OK
ENSBTAG00000022598	ENSBTAG00000022598	22:53073617-53077228	0.839884	0	1.50283	OK
ENSBTAG00000013871	ENSBTAG00000013871	24:54470148-54474339	0.837557	0	1.28607	OK
ENSBTAG00000038684	ENSBTAG00000038684	26:42824586-42832080	0.836671	0	1.48962	OK
ENSBTAG00000000510	ENSBTAG00000000510	5:27962234-27968039	0.83666	0	1.27202	OK
ENSBTAG00000015115	ENSBTAG00000015115	18:57310722-57315855	0.836368	0	1.06495	OK
ENSBTAG00000014514	ENSBTAG00000014514	18:55753308-55764157	0.836237	0	1.19074	OK
ENSBTAG00000045812	ENSBTAG00000045812	6:71279031-71280632	0.835962	0	1.57887	OK
ENSBTAG00000006847	ENSBTAG00000006847	X:119338869-119339227	0.835358	0	1.73511	OK
ENSBTAG00000002518	ENSBTAG00000002518	6:101090296-101095292	0.83473	0	1.31644	OK
ENSBTAG00000003234	ENSBTAG00000003234	18:8636041-8729511	0.83369	0.177427	1.41941	OK
ENSBTAG00000005353	ENSBTAG00000005353	2:108075459-108082534	0.83354	0.0926015	1.11122	OK
ENSBTAG00000045086	ENSBTAG00000045086	7:41115462-41115731	0.833342	0	2.30919	OK
ENSBTAG00000010820	ENSBTAG00000010820	15:56384699-56404335	0.832128	0	0.902865	OK
ENSBTAG00000006582	ENSBTAG00000006582	21:48799459-48800704	0.831229	0	1.4968	OK
ENSBTAG00000011359	ENSBTAG00000011359	19:51152179-51155591	0.831171	0	1.48561	OK
ENSBTAG00000020746	ENSBTAG00000020746	15:79811631-79812585	0.830538	0	1.95337	OK
ENSBTAG00000015804	ENSBTAG00000015804	8:77345881-77348597	0.830341	0.133758	1.47133	OK

ENSBTAG00000019115	ENSBTAG00000019115	19:58620113-58622767	0.829848	0.0780169	1.09224	OK
ENSBTAG00000004742	ENSBTAG00000004742	1:148678709-148773773	0.829169	0	1.36822	OK
ENSBTAG00000031432	ENSBTAG00000031432	23:49282978-49374341	0.829141	0	1.51294	OK
ENSBTAG00000045446	ENSBTAG00000045446	1:110501344-110501623	0.828975	0	2.22642	OK
ENSBTAG00000018993	ENSBTAG00000018993	5:26958460-26973297	0.828219	0.158604	1.26883	OK
ENSBTAG00000032457	ENSBTAG00000032457	19:24801592-24802537	0.826997	0	1.53376	OK
ENSBTAG00000026365	ENSBTAG00000026365	25:13059741-13060996	0.826664	0.172836	1.38268	OK
ENSBTAG00000046323	ENSBTAG00000046323	18:25717807-25721329	0.82586	0	4.1293	OK
ENSBTAG00000044166	ENSBTAG00000044166	6:62295687-62298730	0.824981	0	1.55293	OK
ENSBTAG00000011255	ENSBTAG00000011255	7:18482458-18483897	0.824276	0	1.29501	OK
ENSBTAG00000002898	ENSBTAG00000002898	19:15233408-15265756	0.82406	0.19321	1.22366	OK
ENSBTAG00000040028	ENSBTAG00000040028	7:2600731-2618482	0.822355	0.0640647	0.896905	OK
ENSBTAG00000010674	ENSBTAG00000010674	19:3427682-3428230	0.82165	0	1.4379	OK
ENSBTAG00000009350	ENSBTAG00000009350	28:29106432-29125279	0.82097	0	1.48428	OK
ENSBTAG00000046682	ENSBTAG00000046682	10:22900140-22900718	0.820767	0	1.68339	OK
ENSBTAG00000032918	ENSBTAG00000032918	29:41501195-41504719	0.819967	0	1.38654	OK
ENSBTAG00000011668	ENSBTAG00000011668	29:18121097-18121808	0.819416	0	1.4561	OK
ENSBTAG00000047175	ENSBTAG00000047175	18:62935385-62945850	0.817889	0	4.08945	OK
ENSBTAG00000046819	ENSBTAG00000046819	10:24838411-24838993	0.816792	0	1.57658	OK
ENSBTAG00000020143	ENSBTAG00000020143	14:38745908-38775000	0.815262	0.159152	1.43237	OK
ENSBTAG00000031693	ENSBTAG00000031693	17:64491725-64494161	0.814831	0.254996	1.35998	OK
ENSBTAG00000046714	ENSBTAG00000046714	8:98271048-98271599	0.813942	0	1.50314	OK
ENSBTAG00000045708	ENSBTAG00000045708	18:53700345-53725742	0.813275	0	1.27476	OK
ENSBTAG00000039973	ENSBTAG00000039973	26:14447528-14455537	0.813258	0	0.888112	OK
ENSBTAG00000013888	ENSBTAG00000013888	21:48214178-48217962	0.813025	0	1.32446	OK
ENSBTAG00000017929	ENSBTAG00000017929	16:51277883-51279299	0.812708	0	1.31604	OK
ENSBTAG00000034106	ENSBTAG00000034106	22:15574311-15577582	0.812548	0.196263	1.5701	OK
ENSBTAG00000016125	ENSBTAG00000016125	18:56038882-56041594	0.812383	0.136671	1.36671	OK
ENSBTAG00000002363	ENSBTAG00000002363	2:125698159-125714128	0.81135	0	2.80272	OK
ENSBTAG00000043546	ENSBTAG00000043546	MT:13912-14440	0.811294	0	4.05647	OK

ENSBTAG00000002204	ENSBTAG00000002204	16:57642886-57643647	0.810929	0	1.63251	OK
ENSBTAG00000020524	ENSBTAG00000020524	25:35034016-35039183	0.810359	0	1.39365	OK
ENSBTAG00000006694	ENSBTAG00000006694	7:48661077-48669442	0.809345	0.252201	1.38711	OK
ENSBTAG00000032432	ENSBTAG00000032432	14:68938072-68938543	0.80893	0	1.31883	OK
ENSBTAG00000019966	ENSBTAG00000019966	7:20547963-20550264	0.807706	0.0908145	1.08977	OK
ENSBTAG00000019065	ENSBTAG00000019065	4:61354647-61476979	0.807608	0.0757341	1.06028	OK
ENSBTAG00000004688	ENSBTAG00000004688	3:91981618-92014282	0.806871	0.226209	1.35726	OK
ENSBTAG00000047218	ENSBTAG00000047218	X:63896593-63897112	0.806405	0	1.59581	OK
ENSBTAG00000005054	ENSBTAG00000005054	5:31859947-31860871	0.804944	0	2.01679	OK
ENSBTAG00000011330	ENSBTAG00000011330	9:64879263-64929935	0.80453	0	1.59684	OK
ENSBTAG00000031031	ENSBTAG00000031031	5:57952734-57953673	0.804081	0	1.54356	OK
ENSBTAG00000023415	ENSBTAG00000023415	18:56081110-56084735	0.803113	0	1.55682	OK
ENSBTAG00000039652	ENSBTAG00000039652	29:37761270-37762491	0.802753	0	1.18706	OK
ENSBTAG00000009824	ENSBTAG00000009824	17:29777298-29777556	0.802348	0	2.40764	OK
ENSBTAG00000021603	ENSBTAG00000021603	X:124027196-124028169	0.802318	0	1.50822	OK
ENSBTAG00000048016	ENSBTAG00000048016	4:106455791-106456263	0.801712	0	1.83236	OK
ENSBTAG00000008313	ENSBTAG00000008313	22:61283171-61292866	0.801471	0	0.831183	OK
ENSBTAG00000037964	ENSBTAG00000037964	7:2485680-2487189	0.800444	0.137215	1.37215	OK
ENSBTAG00000018398	ENSBTAG00000018398	18:53615948-53617690	0.800193	0.118862	1.42634	OK
ENSBTAG00000014506	ENSBTAG00000014506	28:31317208-31320375	0.799356	0	0.759643	OK
ENSBTAG00000046171	ENSBTAG00000046171	X:124082650-124083682	0.798079	0	1.40446	OK
ENSBTAG00000038970	ENSBTAG00000038970	24:39250141-39251488	0.798067	0	1.38345	OK
ENSBTAG00000019051	ENSBTAG00000019051	18:62472029-62474435	0.795968	0	0.873658	OK
ENSBTAG00000011358	ENSBTAG00000011358	23:28088455-28089722	0.795959	0	1.41941	OK
ENSBTAG00000011991	ENSBTAG00000011991	28:43961639-43993582	0.794909	0	1.48828	OK
ENSBTAG00000025531	ENSBTAG00000025531	23:27793272-27794712	0.794448	0	1.53186	OK
ENSBTAG00000048086	ENSBTAG00000048086	X:60849982-60851564	0.79431	0.177884	1.42307	OK
ENSBTAG00000046408	ENSBTAG00000046408	12:20704475-20778873	0.794205	0	1.21322	OK
ENSBTAG00000005349	ENSBTAG00000005349	15:44469326-44472127	0.794118	0	3.17647	OK
ENSBTAG00000006902	ENSBTAG00000006902	29:32823874-32829939	0.792846	0.162398	1.29918	OK

ENSBTAG00000032031	ENSBTAG00000032031	23:27274033-27275413	0.792298	0	1.35037	OK
ENSBTAG00000046512	ENSBTAG00000046512	22:12549675-12558895	0.792027	0.165065	0.79231	OK
ENSBTAG00000047996	ENSBTAG00000047996	29:50450723-50460449	0.791662	0	1.45668	OK
ENSBTAG00000006111	ENSBTAG00000006111	28:3358380-3360841	0.790504	0.0841353	1.17789	OK
ENSBTAG00000004595	ENSBTAG00000004595	14:2715415-2742638	0.789773	0	1.38407	OK
ENSBTAG00000015113	ENSBTAG00000015113	3:105949583-105950924	0.788215	0	1.08083	OK
ENSBTAG00000003776	ENSBTAG00000003776	6:107658058-107663981	0.787422	0	1.45815	OK
ENSBTAG00000018406	ENSBTAG00000018406	26:7475455-7477851	0.787158	0.175027	1.3127	OK
ENSBTAG00000008922	ENSBTAG00000008922	5:74226737-74228722	0.785683	0	1.40039	OK
ENSBTAG00000019280	ENSBTAG00000019280	17:68506966-68519007	0.784995	0	3.92498	OK
ENSBTAG00000014457	ENSBTAG00000014457	X:118995027-118997078	0.784841	0.191188	1.52951	OK
ENSBTAG00000044542	ENSBTAG00000044542	14:31274130-31274386	0.784304	0	2.42645	OK
ENSBTAG00000016561	ENSBTAG00000016561	25:1360578-1365802	0.784281	0	1.15398	OK
ENSBTAG00000014597	ENSBTAG00000014597	18:55336319-55358014	0.783361	0	0.766878	OK
ENSBTAG00000022575	ENSBTAG00000022575	22:60590208-60598690	0.783045	0	1.14577	OK
ENSBTAG00000018723	ENSBTAG00000018723	16:53069567-53074055	0.782873	0.15574	1.32379	OK
ENSBTAG00000014642	ENSBTAG00000014642	14:2327869-2331019	0.782419	0	0.863766	OK
ENSBTAG00000024852	ENSBTAG00000024852	22:52189556-52191061	0.782162	0	1.2587	OK
ENSBTAG00000034995	ENSBTAG00000034995	11:2394758-2396426	0.781333	0.124135	1.36548	OK
ENSBTAG00000014798	ENSBTAG00000014798	X:84354014-84356815	0.780595	0.127341	1.40075	OK
ENSBTAG00000039343	ENSBTAG00000039343	11:97651077-97652610	0.779694	0.135067	1.35067	OK
ENSBTAG00000003491	ENSBTAG00000003491	7:19235825-19258805	0.779379	0	1.29952	OK
ENSBTAG00000045939	ENSBTAG00000045939	15:83332007-83334140	0.778786	0	0.700193	OK
ENSBTAG00000026963	ENSBTAG00000026963	14:64875134-64898581	0.778693	0.225921	1.24257	OK
ENSBTAG00000017890	ENSBTAG00000017890	17:71015442-71038221	0.778396	0	1.35184	OK
ENSBTAG00000007992	ENSBTAG00000007992	18:36449382-36455306	0.777754	0.184051	1.28835	OK
ENSBTAG00000047958	ENSBTAG00000047958	GJ059509.1:3534-6470	0.776707	0	1.1717	OK
ENSBTAG00000009297	ENSBTAG00000009297	5:121170972-121178483	0.776652	0	0.655474	OK
ENSBTAG00000005138	ENSBTAG00000005138	15:30023046-30033583	0.77538	0.0922714	0.738171	OK
ENSBTAG00000038526	ENSBTAG00000038526	18:57748092-57753897	0.77464	0.185203	1.48162	OK

ENSBTAG00000018108	ENSBTAG00000018108	17:64818099-64833437	0.774451	0	1.04751	OK
ENSBTAG00000011461	ENSBTAG00000011461	16:718145-759737	0.772884	0.144947	1.23205	OK
ENSBTAG00000000995	ENSBTAG00000000995	9:21763777-21766208	0.772483	0.158664	1.26931	OK
ENSBTAG00000011241	ENSBTAG00000011241	3:101333429-101335081	0.772419	0	1.12803	OK
ENSBTAG00000039797	ENSBTAG00000039797	7:42678162-42679104	0.771979	0	1.53864	OK
ENSBTAG00000047613	ENSBTAG00000047613	6:119387683-119418513	0.771738	0	1.16034	OK
ENSBTAG00000048015	ENSBTAG00000048015	7:9655748-9656714	0.770486	0	1.71476	OK
ENSBTAG00000038600	ENSBTAG00000038600	1:73785483-73787238	0.770323	0.235962	1.29779	OK
ENSBTAG00000033331	ENSBTAG00000033331	18:14592687-14599518	0.770011	0	2.60437	OK
ENSBTAG00000047654	ENSBTAG00000047654	18:34471578-34475671	0.769969	0	1.48428	OK
ENSBTAG00000014208	ENSBTAG00000014208	1:70788696-70792140	0.769009	0	1.39589	OK
ENSBTAG00000009199	ENSBTAG00000009199	25:3492817-3497250	0.76795	0	0.920253	OK
ENSBTAG00000001809	ENSBTAG00000001809	X:104278050-104278641	0.766851	0	1.4014	OK
ENSBTAG00000003721	ENSBTAG00000003721	15:76573747-76577430	0.766712	0.0925187	1.29526	OK
ENSBTAG00000023744	ENSBTAG00000023744	11:69688036-69702229	0.766228	0	1.56073	OK
ENSBTAG00000022368	ENSBTAG00000022368	15:50510481-50511429	0.766135	0	1.96573	OK
ENSBTAG00000017951	ENSBTAG00000017951	29:45899599-45901384	0.764582	0	1.47747	OK
ENSBTAG00000017361	ENSBTAG00000017361	21:69843887-69846777	0.764188	0.230833	1.26958	OK
ENSBTAG00000011290	ENSBTAG00000011290	14:68986918-68987671	0.763977	0	2.19981	OK
ENSBTAG00000003219	ENSBTAG00000003219	20:57300937-57311455	0.76346	0	1.38038	OK
ENSBTAG00000045776	ENSBTAG00000045776	3:108695599-108696665	0.762538	0	1.44998	OK
ENSBTAG00000030367	ENSBTAG00000030367	2:133473126-133474216	0.761965	0	1.32972	OK
ENSBTAG00000006059	ENSBTAG00000006059	2:73827081-73833492	0.761722	0	1.46998	OK
ENSBTAG00000011659	ENSBTAG00000011659	1:72024794-72028847	0.761438	0.120172	1.3219	OK
ENSBTAG00000015721	ENSBTAG00000015721	7:16073296-16086918	0.760002	0.122049	0.976396	OK
ENSBTAG00000010455	ENSBTAG00000010455	29:41679913-41684529	0.759862	0.127146	1.01717	OK
ENSBTAG00000014554	ENSBTAG00000014554	13:78766067-78772194	0.758009	0	1.09169	OK
ENSBTAG00000033835	ENSBTAG00000033835	3:8229235-8234089	0.757817	0.214678	1.28807	OK
ENSBTAG00000013702	ENSBTAG00000013702	18:53154420-53158137	0.757396	0	0.994101	OK
ENSBTAG00000019468	ENSBTAG00000019468	8:100142636-100144156	0.756828	0.136222	1.36222	OK

ENSBTAG00000012289	ENSBTAG00000012289	2:133953058-133956669	0.756341	0	1.41405	OK
ENSBTAG00000020819	ENSBTAG00000020819	21:66817738-66842006	0.755795	0.175027	1.40022	OK
ENSBTAG00000007644	ENSBTAG00000007644	7:22200649-22338566	0.754932	0.115288	0.979947	OK
ENSBTAG00000040596	ENSBTAG00000040596	3:19020284-19022096	0.754473	0	1.02843	OK
ENSBTAG00000045128	ENSBTAG00000045128	1:14608518-14608853	0.75351	0	1.85424	OK
ENSBTAG00000044686	ENSBTAG00000044686	3:23148104-23148345	0.752743	0	2.57747	OK
ENSBTAG00000040106	ENSBTAG00000040106	5:112904447-112911243	0.751519	0	0.608991	OK
ENSBTAG00000020751	ENSBTAG00000020751	14:1806080-1825793	0.751445	0	0.922075	OK
ENSBTAG00000012211	ENSBTAG00000012211	4:69353605-69356273	0.750461	0.125413	1.25413	OK
ENSBTAG00000013450	ENSBTAG00000013450	7:70889713-70892378	0.750183	0.222762	1.22519	OK
ENSBTAG00000046941	ENSBTAG00000046941	X:74782935-74783327	0.749594	0	1.58462	OK
ENSBTAG00000004838	ENSBTAG00000004838	18:23309539-23312757	0.749274	0.103219	1.34184	OK
ENSBTAG00000018805	ENSBTAG00000018805	13:43407523-43422283	0.749039	0	1.31286	OK
ENSBTAG00000001078	ENSBTAG00000001078	25:3392667-3404630	0.748684	0.157818	1.26254	OK
ENSBTAG00000033594	ENSBTAG00000033594	5:22719222-22720382	0.748485	0	1.4014	OK
ENSBTAG00000046727	ENSBTAG00000046727	26:25763969-25764923	0.748475	0	1.30225	OK
ENSBTAG00000002098	ENSBTAG00000002098	7:44789107-44794947	0.746129	0	1.31049	OK
ENSBTAG00000037822	ENSBTAG00000037822	11:93866952-93868392	0.745321	0	1.57908	OK
ENSBTAG00000004457	ENSBTAG00000004457	17:55858842-55873788	0.745057	0	1.19994	OK
ENSBTAG00000046952	ENSBTAG00000046952	10:84683742-84684195	0.74448	0	2.2854	OK
ENSBTAG00000046934	ENSBTAG00000046934	23:28984378-28985359	0.742898	0	1.68854	OK
ENSBTAG00000037704	ENSBTAG00000037704	X:119373318-119374578	0.742891	0.164331	1.31465	OK
ENSBTAG00000005184	ENSBTAG00000005184	13:74440208-74443091	0.742022	0	1.39151	OK
ENSBTAG00000009502	ENSBTAG00000009502	2:130360071-130363164	0.741964	0.267775	1.20499	OK
ENSBTAG00000046777	ENSBTAG00000046777	23:29280760-29282962	0.74195	0	1.73088	OK
ENSBTAG00000001338	ENSBTAG00000001338	22:53761588-53762665	0.74171	0	1.34577	OK
ENSBTAG00000021696	ENSBTAG00000021696	8:60821684-60823630	0.740745	0.212803	1.17041	OK
ENSBTAG00000015857	ENSBTAG00000015857	16:50687997-50690794	0.740626	0	1.32244	OK
ENSBTAG00000019530	ENSBTAG00000019530	25:25072847-25082574	0.739896	0	1.34744	OK
ENSBTAG00000025564	ENSBTAG00000025564	10:42699003-42700655	0.739333	0	1.29142	OK

ENSBTAG00000023377	ENSBTAG00000023377	1:154024456-154103515	0.739211	0	1.06339	OK
ENSBTAG00000014353	ENSBTAG00000014353	10:35805062-35808329	0.739138	0	1.44795	OK
ENSBTAG00000013536	ENSBTAG00000013536	7:15894541-15896884	0.738809	0	1.20985	OK
ENSBTAG00000007841	ENSBTAG00000007841	18:45075879-45101465	0.738793	0	0.716814	OK
ENSBTAG00000046217	ENSBTAG00000046217	19:24749222-24750164	0.73862	0	1.31883	OK
ENSBTAG00000010910	ENSBTAG00000010910	3:120123113-120127712	0.738427	0	1.33013	OK
ENSBTAG00000015499	ENSBTAG00000015499	29:43968701-43981536	0.737359	0	0.829334	OK
ENSBTAG00000025928	ENSBTAG00000025928	8:22650207-22650777	0.737127	0	1.81629	OK
ENSBTAG00000012465	ENSBTAG00000012465	18:12422845-12425239	0.736476	0.0878104	1.05372	OK
ENSBTAG00000001601	ENSBTAG00000001601	10:18965983-18992445	0.734694	0.182188	1.27532	OK
ENSBTAG00000015565	ENSBTAG00000015565	23:6975319-6990903	0.734531	0	1.36401	OK
ENSBTAG00000020357	ENSBTAG00000020357	3:20082068-20126434	0.733945	0.151579	1.36421	OK
ENSBTAG00000039055	ENSBTAG00000039055	16:52376215-52379152	0.733816	0	0.960079	OK
ENSBTAG00000033608	ENSBTAG00000033608	5:19459448-19461191	0.733667	0.123248	1.23248	OK
ENSBTAG00000003551	ENSBTAG00000003551	7:43466455-43467376	0.7335	0	1.79854	OK
ENSBTAG00000003845	ENSBTAG00000003845	22:12520392-12532806	0.732795	0	0.895059	OK
ENSBTAG00000007512	ENSBTAG00000007512	18:35747166-35760966	0.73224	0.151579	1.21263	OK
ENSBTAG00000018012	ENSBTAG00000018012	11:46636521-46639761	0.731543	0	1.35331	OK
ENSBTAG00000003304	ENSBTAG00000003304	5:26136685-26138815	0.731239	0.125794	1.25794	OK
ENSBTAG00000040435	ENSBTAG00000040435	3:101348533-101349004	0.730005	0	1.31883	OK
ENSBTAG00000004727	ENSBTAG00000004727	25:2378552-2382039	0.729955	0	0.944507	OK
ENSBTAG00000030305	ENSBTAG00000030305	19:43828734-43829232	0.729799	0	2.07889	OK
ENSBTAG00000046650	ENSBTAG00000046650	3:11104356-11105301	0.729781	0	1.31465	OK
ENSBTAG00000047697	ENSBTAG00000047697	29:51208025-51216725	0.729094	0	1.34891	OK
ENSBTAG00000046545	ENSBTAG00000046545	3:14380071-14413740	0.72887	0.0787888	1.10304	OK
ENSBTAG00000044964	ENSBTAG00000044964	10:101695183-101695454	0.728277	0	2.29214	OK
ENSBTAG00000016319	ENSBTAG00000016319	19:25323255-25329033	0.72803	0	1.24135	OK
ENSBTAG00000005107	ENSBTAG00000005107	5:23629704-23632125	0.72691	0.170839	1.36671	OK
ENSBTAG00000047822	ENSBTAG00000047822	11:67802266-67803322	0.726892	0	1.37254	OK
ENSBTAG00000000154	ENSBTAG00000000154	X:11966613-11968272	0.725717	0	0.873658	OK

ENSBTAG00000011491	ENSBTAG00000011491	19:23393585-23402810	0.725513	0.0986925	0.78954	OK
ENSBTAG00000019754	ENSBTAG00000019754	15:47329715-47331334	0.72478	0	1.39499	OK
ENSBTAG00000023611	ENSBTAG00000023611	18:46616258-46619888	0.724342	0.109207	1.31049	OK
ENSBTAG00000018346	ENSBTAG00000018346	1:129262594-129292281	0.723896	0.190135	1.23588	OK
ENSBTAG00000022036	ENSBTAG00000022036	17:70526961-70534280	0.723824	0	0.77906	OK
ENSBTAG00000046484	ENSBTAG00000046484	11:101335451-101343787	0.723646	0	1.61763	OK
ENSBTAG00000040575	ENSBTAG00000040575	25:2102772-2123806	0.723345	0.092934	1.11521	OK
ENSBTAG00000046837	ENSBTAG00000046837	7:17610090-17611773	0.723047	0	1.10726	OK
ENSBTAG00000021715	ENSBTAG00000021715	3:110305530-110311199	0.723005	0	1.14186	OK
ENSBTAG00000036060	ENSBTAG00000036060	X:32978866-32979646	0.722521	0	1.32729	OK
ENSBTAG00000026156	ENSBTAG00000026156	1:34683615-34707256	0.722413	0	1.44219	OK
ENSBTAG00000022084	ENSBTAG00000022084	17:46028479-46029470	0.722247	0	1.34162	OK
ENSBTAG00000025663	ENSBTAG00000025663	10:26255988-26257533	0.721663	0	1.21942	OK
ENSBTAG00000019676	ENSBTAG00000019676	24:59670135-59671874	0.720172	0.238133	1.30973	OK
ENSBTAG00000000354	ENSBTAG00000000354	19:51768183-51769908	0.719392	0	1.24433	OK
ENSBTAG00000001450	ENSBTAG00000001450	7:53414892-53421127	0.718401	0.100318	1.20382	OK
ENSBTAG00000008633	ENSBTAG00000008633	25:26385987-26389337	0.7184	0	1.24412	OK
ENSBTAG00000006152	ENSBTAG00000006152	4:84628121-84628559	0.718303	0	1.4182	OK
ENSBTAG00000004950	ENSBTAG00000004950	10:26288222-26289852	0.717386	0	1.22664	OK
ENSBTAG00000000226	ENSBTAG00000000226	27:36903866-36907355	0.717084	0	1.37885	OK
ENSBTAG00000017488	ENSBTAG00000017488	6:59593803-59610697	0.716858	0	1.35966	OK
ENSBTAG00000035144	ENSBTAG00000035144	GJ060118.1:1941-8662	0.716229	0	1.15859	OK
ENSBTAG00000047418	ENSBTAG00000047418	GJ060129.1:1866-6353	0.716147	0	1.07522	OK
ENSBTAG00000002100	ENSBTAG00000002100	7:44797941-44803445	0.716015	0	1.28767	OK
ENSBTAG00000008645	ENSBTAG00000008645	29:43221838-43228308	0.714876	0.100124	1.10137	OK
ENSBTAG00000008468	ENSBTAG00000008468	4:114461158-114517394	0.714729	0	0.690958	OK
ENSBTAG00000020458	ENSBTAG00000020458	16:63581737-63628883	0.714526	0.166044	1.24533	OK
ENSBTAG00000044038	ENSBTAG00000044038	19:56279921-56300266	0.713935	0	1.41047	OK
ENSBTAG00000007153	ENSBTAG00000007153	2:130792854-130795743	0.713036	0	2.4814	OK
ENSBTAG00000006135	ENSBTAG00000006135	13:64497253-64498905	0.711993	0	1.30225	OK

ENSBTAG00000016079	ENSBTAG00000016079	18:11799174-11807341	0.711281	0	3.5564	OK
ENSBTAG00000018455	ENSBTAG00000018455	14:1531490-1533526	0.710405	0	1.20582	OK
ENSBTAG00000019114	ENSBTAG00000019114	10:50282170-50283148	0.710391	0	1.27029	OK
ENSBTAG00000006374	ENSBTAG00000006374	7:40601548-40605473	0.70994	0	1.40664	OK
ENSBTAG00000008335	ENSBTAG00000008335	7:8237779-8238724	0.708895	0	1.97197	OK
ENSBTAG00000031789	ENSBTAG00000031789	25:27048342-27051971	0.708698	0	1.05881	OK
ENSBTAG00000018872	ENSBTAG00000018872	7:40258128-40266423	0.708496	0	0.923445	OK
ENSBTAG00000003161	ENSBTAG00000003161	1:147707849-147712115	0.708269	0	1.18183	OK
ENSBTAG00000046305	ENSBTAG00000046305	10:24120161-24120630	0.708124	0	1.72547	OK
ENSBTAG00000046249	ENSBTAG00000046249	7:15204471-15205428	0.707955	0	1.73088	OK
ENSBTAG00000018580	ENSBTAG00000018580	12:15818916-15832440	0.707501	0	3.5375	OK
ENSBTAG00000017716	ENSBTAG00000017716	21:67072574-67100143	0.706832	0	0.7429	OK
ENSBTAG00000000066	ENSBTAG00000000066	13:48578734-48593922	0.706588	0.0947196	1.23135	OK
ENSBTAG00000017424	ENSBTAG00000017424	13:58463227-58510710	0.706565	0.18348	1.19262	OK
ENSBTAG00000033395	ENSBTAG00000033395	25:1116676-1117637	0.70563	0	1.40378	OK
ENSBTAG00000015828	ENSBTAG00000015828	5:31048545-31052006	0.70405	0	1.16161	OK
ENSBTAG00000006108	ENSBTAG00000006108	17:73216821-73220883	0.70365	0	0.855607	OK
ENSBTAG00000036082	ENSBTAG00000036082	21:20611312-20612780	0.703036	0	1.31883	OK
ENSBTAG00000000715	ENSBTAG00000000715	4:114148310-114155451	0.702919	0	3.5146	OK
ENSBTAG00000026248	ENSBTAG00000026248	25:36421451-36422572	0.701253	0	1.19272	OK
ENSBTAG00000007106	ENSBTAG00000007106	1:146859200-146867963	0.700955	0	1.15859	OK
ENSBTAG00000035726	ENSBTAG00000035726	6:85900616-85901870	0.700777	0.165117	1.32094	OK
ENSBTAG00000013085	ENSBTAG00000013085	2:131425506-131432711	0.700514	0	1.37579	OK
ENSBTAG00000010060	ENSBTAG00000010060	15:81978439-81996848	0.700259	0.0809449	1.13323	OK
ENSBTAG00000031769	ENSBTAG00000031769	23:31519197-31519765	0.69966	0	1.09361	OK
ENSBTAG00000000335	ENSBTAG00000000335	3:112536430-112537155	0.699304	0	1.42798	OK
ENSBTAG00000006261	ENSBTAG00000006261	2:4805766-4811802	0.698523	0.0882972	1.05957	OK
ENSBTAG00000000528	ENSBTAG00000000528	13:66899154-66926952	0.698167	0	1.16161	OK
ENSBTAG00000032709	ENSBTAG00000032709	10:21379412-21381885	0.697953	0	1.3222	OK
ENSBTAG00000039807	ENSBTAG00000039807	10:23933879-23934477	0.697492	0	1.60925	OK

ENSBTAG00000032534	ENSBTAG00000032534	17:55759605-55773011	0.697401	0.100076	1.20091	OK
ENSBTAG00000010758	ENSBTAG00000010758	19:56566598-56573547	0.696052	0	1.25598	OK
ENSBTAG00000021100	ENSBTAG00000021100	X:92387160-92403412	0.69489	0.0714482	1.00028	OK
ENSBTAG00000018345	ENSBTAG00000018345	19:25750464-25758746	0.694616	0.121015	1.21015	OK
ENSBTAG00000004909	ENSBTAG00000004909	19:27094264-27096217	0.694085	0.21204	1.16622	OK
ENSBTAG00000021432	ENSBTAG00000021432	16:64965974-64966877	0.693386	0	1.37579	OK
ENSBTAG00000012823	ENSBTAG00000012823	23:15901125-15908139	0.693008	0	1.32587	OK
ENSBTAG00000016834	ENSBTAG00000016834	23:9733330-9739310	0.692059	0	1.26062	OK
ENSBTAG00000003039	ENSBTAG00000003039	23:7113303-7116676	0.691912	0	1.34079	OK
ENSBTAG00000038551	ENSBTAG00000038551	11:93731041-93731977	0.691794	0	1.32729	OK
ENSBTAG00000006185	ENSBTAG00000006185	7:16811451-16817517	0.691628	0	1.17479	OK
ENSBTAG00000014101	ENSBTAG00000014101	25:1067757-1070205	0.691008	0	1.20083	OK
ENSBTAG00000018925	ENSBTAG00000018925	8:62341238-62345923	0.690441	0	1.32486	OK
ENSBTAG00000017674	ENSBTAG00000017674	16:52595745-52599094	0.689715	0	1.09361	OK
ENSBTAG00000043974	ENSBTAG00000043974	25:26968646-26970732	0.689566	0.119479	1.19479	OK
ENSBTAG00000011403	ENSBTAG00000011403	8:60134671-60196453	0.689359	0.115674	0.771162	OK
ENSBTAG00000016299	ENSBTAG00000016299	18:62371837-62374877	0.688765	0	1.00135	OK
ENSBTAG00000007759	ENSBTAG00000007759	4:114404771-114422140	0.688578	0	1.69105	OK
ENSBTAG00000039789	ENSBTAG00000039789	10:38105763-38108620	0.688204	0.260777	1.13003	OK
ENSBTAG00000017228	ENSBTAG00000017228	15:75870184-75884870	0.687867	0	1.34978	OK
ENSBTAG00000027557	ENSBTAG00000027557	15:77516534-77521982	0.687667	0	0.62618	OK
ENSBTAG00000026451	ENSBTAG00000026451	X:32611006-32611744	0.686519	0	1.40282	OK
ENSBTAG00000039372	ENSBTAG00000039372	7:44755703-44760148	0.686169	0	1.24135	OK
ENSBTAG00000001260	ENSBTAG00000001260	18:52120386-52124418	0.686057	0	1.33413	OK
ENSBTAG00000021912	ENSBTAG00000021912	22:49379907-49385352	0.685696	0	1.24265	OK
ENSBTAG00000046271	ENSBTAG00000046271	12:80714188-80719236	0.685691	0	1.71689	OK
ENSBTAG00000005228	ENSBTAG00000005228	15:47410764-47415608	0.685571	0.119825	1.19825	OK
ENSBTAG00000003479	ENSBTAG00000003479	2:123392014-123401324	0.685001	0	1.2085	OK
ENSBTAG00000003370	ENSBTAG00000003370	10:71986325-71987819	0.684756	0	1.01712	OK
ENSBTAG00000018316	ENSBTAG00000018316	19:27243299-27248391	0.684348	0.0886374	1.06365	OK

ENSBTAG00000021427	ENSBTAG00000021427	19:38573224-38576915	0.684306	0.145712	1.1657	OK
ENSBTAG00000003671	ENSBTAG00000003671	7:46023821-46025519	0.683423	0	0.853592	OK
ENSBTAG00000008257	ENSBTAG00000008257	14:59100800-59101904	0.68318	0	1.34577	OK
ENSBTAG00000019069	ENSBTAG00000019069	19:54611055-54614829	0.682829	0	1.249	OK
ENSBTAG00000018817	ENSBTAG00000018817	7:59524544-59525876	0.682452	0	1.22157	OK
ENSBTAG00000003202	ENSBTAG00000003202	2:125213836-125255802	0.682252	0	1.29526	OK
ENSBTAG00000026199	ENSBTAG00000026199	25:39343632-39347044	0.681677	0.113146	1.2446	OK
ENSBTAG00000013314	ENSBTAG00000013314	11:96719399-96772227	0.680448	0	1.81453	OK
ENSBTAG00000000961	ENSBTAG00000000961	13:75365122-75367907	0.680353	0.129168	1.16252	OK
ENSBTAG00000000039	ENSBTAG00000000039	19:51585608-51591804	0.67993	0	1.09618	OK
ENSBTAG00000033506	ENSBTAG00000033506	15:84088595-84089531	0.679845	0	1.32729	OK
ENSBTAG00000007603	ENSBTAG00000007603	19:38594901-38596261	0.679525	0	1.13893	OK
ENSBTAG00000038173	ENSBTAG00000038173	23:31112602-31113277	0.678934	0	3.39467	OK
ENSBTAG00000004022	ENSBTAG00000004022	14:25007290-25009296	0.677548	0.138038	1.1043	OK
ENSBTAG00000013585	ENSBTAG00000013585	28:31244062-31244908	0.67735	0	1.22374	OK
ENSBTAG00000025320	ENSBTAG00000025320	19:8181242-8192864	0.676915	0	1.80511	OK
ENSBTAG00000047404	ENSBTAG00000047404	X:138379923-138380874	0.676502	0	1.30635	OK
ENSBTAG00000048006	ENSBTAG00000048006	3:100068431-100068834	0.676485	0	1.54137	OK
ENSBTAG00000009143	ENSBTAG00000009143	24:43983879-43984857	0.675802	0	1.27029	OK
ENSBTAG00000047701	ENSBTAG00000047701	X:12158375-12159509	0.675802	0	1.46072	OK
ENSBTAG00000046326	ENSBTAG00000046326	25:2646520-2647465	0.675345	0	1.31465	OK
ENSBTAG00000007446	ENSBTAG00000007446	3:28124614-28125503	0.674644	0	1.16455	OK
ENSBTAG00000016633	ENSBTAG00000016633	17:2059568-2063780	0.674439	0.139998	1.25998	OK
ENSBTAG00000048268	ENSBTAG00000048268	21:71530012-71531441	0.674397	0	3.37198	OK
ENSBTAG00000047748	ENSBTAG00000047748	8:22674398-22674986	0.672758	0	1.05641	OK
ENSBTAG00000016741	ENSBTAG00000016741	13:73188594-73196443	0.67203	0	1.29591	OK
ENSBTAG00000001287	ENSBTAG00000001287	18:34961685-34974863	0.671854	0	0.971447	OK
ENSBTAG00000010392	ENSBTAG00000010392	16:20595767-20789340	0.67181	0.151026	1.20821	OK
ENSBTAG00000005916	ENSBTAG00000005916	24:680618-701258	0.671804	0.240694	1.08312	OK
ENSBTAG00000024899	ENSBTAG00000024899	19:62207473-62223856	0.671509	0	1.25947	OK

ENSBTAG00000027263	ENSBTAG00000027263	3:21112093-21112565	0.671254	0	1.31604	OK
ENSBTAG00000016478	ENSBTAG00000016478	7:22560007-22561849	0.670949	0	0.786861	OK
ENSBTAG00000046384	ENSBTAG00000046384	1:4955535-4956408	0.670911	0	1.18589	OK
ENSBTAG00000018882	ENSBTAG00000018882	18:51926742-51931019	0.670666	0	1.45815	OK
ENSBTAG00000018098	ENSBTAG00000018098	X:24737904-24739261	0.669922	0	1.22067	OK
ENSBTAG00000008832	ENSBTAG00000008832	19:16110979-16114069	0.669853	0	1.7577	OK
ENSBTAG00000046967	ENSBTAG00000046967	8:23253527-23254085	0.669634	0	1.11321	OK
ENSBTAG00000048123	ENSBTAG00000048123	17:74612300-74613377	0.669539	0	1.15352	OK
ENSBTAG00000025775	ENSBTAG00000025775	7:5252671-5254252	0.669524	0	1.08265	OK
ENSBTAG00000018193	ENSBTAG00000018193	11:72831968-72868413	0.669165	0	1.19884	OK
ENSBTAG00000009223	ENSBTAG00000009223	2:126929534-126930878	0.667983	0	1.23248	OK
ENSBTAG00000007121	ENSBTAG00000007121	19:54596646-54610033	0.667924	0	1.22438	OK
ENSBTAG00000006233	ENSBTAG00000006233	20:31484953-31510630	0.667915	0.157938	1.26351	OK
ENSBTAG00000045978	ENSBTAG00000045978	4:106481300-106481799	0.667791	0	1.68796	OK
ENSBTAG00000037393	ENSBTAG00000037393	1:88405654-88410920	0.667431	0.0990229	1.58437	OK
ENSBTAG00000005174	ENSBTAG00000005174	21:22046769-22056010	0.667421	0.118862	1.18862	OK
ENSBTAG00000020704	ENSBTAG00000020704	4:77171036-77193849	0.666937	0.144291	1.15432	OK
ENSBTAG00000012728	ENSBTAG00000012728	10:21652616-21653790	0.666286	0	1.23458	OK
ENSBTAG00000033034	ENSBTAG00000033034	10:25357238-25357888	0.666117	0	1.34744	OK
ENSBTAG00000001744	ENSBTAG00000001744	11:107155090-107159421	0.665572	0.220979	1.10489	OK
ENSBTAG00000018053	ENSBTAG00000018053	13:58537700-58585721	0.665494	0.172547	1.12156	OK
ENSBTAG00000009436	ENSBTAG00000009436	3:14561666-14567714	0.66508	0.0987396	1.18488	OK
ENSBTAG00000000534	ENSBTAG00000000534	8:10251631-10280827	0.66492	0	1.19884	OK
ENSBTAG00000046178	ENSBTAG00000046178	5:91945425-91946169	0.664891	0	1.66981	OK
ENSBTAG00000005252	ENSBTAG00000005252	X:94214373-94219492	0.66445	0.151247	1.20997	OK
ENSBTAG00000038810	ENSBTAG00000038810	23:27945516-27946340	0.66313	0	1.34162	OK
ENSBTAG00000008981	ENSBTAG00000008981	23:15616806-15624462	0.662701	0.100905	1.21086	OK
ENSBTAG00000014449	ENSBTAG00000014449	1:84033059-84033889	0.662619	0	1.24733	OK
ENSBTAG00000021008	ENSBTAG00000021008	10:26053956-26056906	0.662078	0	0.673826	OK
ENSBTAG00000011508	ENSBTAG00000011508	16:31417941-31421544	0.661906	0	1.04574	OK

ENSBTAG00000013240	ENSBTAG00000013240	29:41855870-41896760	0.661895	0.101499	1.21798	OK
ENSBTAG00000007820	ENSBTAG00000007820	7:2568115-2568744	0.661845	0	1.64592	OK
ENSBTAG00000047035	ENSBTAG00000047035	18:56681332-56691335	0.661841	0.0818731	0.982477	OK
ENSBTAG00000007834	ENSBTAG00000007834	14:1628813-1633988	0.660713	0	0.579065	OK
ENSBTAG00000010015	ENSBTAG00000010015	25:3603662-3609530	0.659845	0	1.31883	OK
ENSBTAG00000038059	ENSBTAG00000038059	19:24711368-24712313	0.659435	0	1.31465	OK
ENSBTAG00000015646	ENSBTAG00000015646	5:49348493-49352530	0.659377	0	1.38964	OK
ENSBTAG00000021787	ENSBTAG00000021787	2:127813022-127818513	0.659115	0	1.27714	OK
ENSBTAG00000016170	ENSBTAG00000016170	15:35650714-35653362	0.659017	0.0781937	1.09471	OK
ENSBTAG00000047399	ENSBTAG00000047399	18:1573209-1574133	0.658989	0	1.12653	OK
ENSBTAG00000013113	ENSBTAG00000013113	7:47247743-47273458	0.65885	0	1.3177	OK
ENSBTAG00000030930	ENSBTAG00000030930	22:59818308-59832821	0.658736	0	1.03701	OK
ENSBTAG00000021835	ENSBTAG00000021835	22:37346724-37439282	0.658318	0.0803481	0.964177	OK
ENSBTAG00000033429	ENSBTAG00000033429	9:38816217-38821062	0.658117	0	1.52248	OK
ENSBTAG00000014176	ENSBTAG00000014176	13:61619568-61628687	0.657455	0	1.22923	OK
ENSBTAG00000005434	ENSBTAG00000005434	7:5685230-5704120	0.657217	0.101898	1.12088	OK
ENSBTAG00000006872	ENSBTAG00000006872	1:80940146-80940854	0.655894	0	1.46227	OK
ENSBTAG00000006982	ENSBTAG00000006982	19:42898001-42903081	0.655815	0.134017	1.07214	OK
ENSBTAG00000019370	ENSBTAG00000019370	X:97425376-97426021	0.654882	0	1.60509	OK
ENSBTAG00000020161	ENSBTAG00000020161	17:74498663-74519770	0.654307	0	0.796033	OK
ENSBTAG00000019658	ENSBTAG00000019658	19:44614051-44621587	0.654016	0.158604	1.11022	OK
ENSBTAG00000026748	ENSBTAG00000026748	8:75984695-76010699	0.653973	0.099451	1.19341	OK
ENSBTAG00000006910	ENSBTAG00000006910	29:41454919-41473601	0.653418	0	1.26918	OK
ENSBTAG00000020186	ENSBTAG00000020186	29:44756083-44759164	0.652043	0	1.17313	OK
ENSBTAG00000016413	ENSBTAG00000016413	27:28963364-28969954	0.651991	0	0.940557	OK
ENSBTAG00000031249	ENSBTAG00000031249	23:50327385-50331704	0.651861	0	1.15032	OK
ENSBTAG00000007513	ENSBTAG00000007513	17:45491536-45499180	0.651804	0.11358	1.1358	OK
ENSBTAG00000038222	ENSBTAG00000038222	16:55768188-55770872	0.650337	0.148109	1.18488	OK
ENSBTAG00000005029	ENSBTAG00000005029	3:99610186-99618464	0.65014	0.100807	1.10887	OK
ENSBTAG00000009115	ENSBTAG00000009115	1:128830907-128901497	0.650033	0.0949803	1.13976	OK

ENSBTAG00000032895	ENSBTAG00000032895	X:119050688-119052955	0.649613	0	1.2714	OK
ENSBTAG00000013393	ENSBTAG00000013393	19:22661935-22665115	0.648936	0.195337	1.0418	OK
ENSBTAG00000000357	ENSBTAG00000000357	27:23463554-23505183	0.648769	0.0489844	0.685781	OK
ENSBTAG00000047317	ENSBTAG00000047317	28:35714666-35748478	0.648598	0	1.27588	OK
ENSBTAG00000004738	ENSBTAG00000004738	16:50540188-50551301	0.648406	0.0621047	0.745256	OK
ENSBTAG00000026192	ENSBTAG00000026192	25:42245835-42303133	0.64797	0	0.771002	OK
ENSBTAG00000010769	ENSBTAG00000010769	12:33114058-33115063	0.647164	0	1.23616	OK
ENSBTAG00000018919	ENSBTAG00000018919	10:26005890-26006826	0.64694	0	1.32729	OK
ENSBTAG00000031806	ENSBTAG00000031806	25:26831622-26835977	0.646335	0.0721704	0.866045	OK
ENSBTAG00000035399	ENSBTAG00000035399	7:14527359-14528301	0.646035	0	1.31883	OK
ENSBTAG00000045937	ENSBTAG00000045937	4:17767924-17769016	0.645957	0	1.13768	OK
ENSBTAG00000023820	ENSBTAG00000023820	X:39474622-39476650	0.645644	0.204198	1.12309	OK
ENSBTAG00000012097	ENSBTAG00000012097	18:53779850-53800006	0.64478	0	0.867557	OK
ENSBTAG00000003018	ENSBTAG00000003018	7:44896886-44903223	0.644432	0	0.953203	OK
ENSBTAG00000021833	ENSBTAG00000021833	3:21859234-21860557	0.643597	0	1.25205	OK
ENSBTAG00000002758	ENSBTAG00000002758	13:42217370-42221004	0.643454	0.113955	0.968621	OK
ENSBTAG00000007080	ENSBTAG00000007080	11:72493260-72497358	0.642776	0	1.71407	OK
ENSBTAG00000006130	ENSBTAG00000006130	21:48843728-48845622	0.642433	0	0.983903	OK
ENSBTAG00000034964	ENSBTAG00000034964	29:4829191-4829806	0.642224	0	1.68339	OK
ENSBTAG00000033334	ENSBTAG00000033334	18:14617871-14621516	0.641544	0	0.833946	OK
ENSBTAG00000006599	ENSBTAG00000006599	5:56334243-56335809	0.641389	0	1.25584	OK
ENSBTAG00000004796	ENSBTAG00000004796	24:58710083-58722719	0.641244	0	1.19513	OK
ENSBTAG00000033643	ENSBTAG00000033643	6:20812850-20861945	0.641203	0	1.31883	OK
ENSBTAG00000017834	ENSBTAG00000017834	16:1041922-1055957	0.640474	0.222343	1.05613	OK
ENSBTAG00000020672	ENSBTAG00000020672	3:113369454-113371967	0.64002	0	1.2714	OK
ENSBTAG00000022039	ENSBTAG00000022039	17:66827050-66828139	0.639355	0	1.14081	OK
ENSBTAG00000039529	ENSBTAG00000039529	3:101784089-101789519	0.638465	0.0980383	1.17646	OK
ENSBTAG00000008333	ENSBTAG00000008333	19:44068483-44083638	0.638142	0.103736	1.1411	OK
ENSBTAG00000023648	ENSBTAG00000023648	15:52255759-52259540	0.637896	0.131548	1.05239	OK
ENSBTAG00000037458	ENSBTAG00000037458	7:8193570-8194554	0.636103	0	1.26254	OK

ENSBTAG00000001854	ENSBTAG00000001854	18:25512316-25525079	0.635995	0.1308	1.0464	OK
ENSBTAG00000008588	ENSBTAG00000008588	23:24489540-24490044	0.635724	0	1.23248	OK
ENSBTAG00000020195	ENSBTAG00000020195	21:25976048-25977656	0.63537	0.128767	1.03013	OK
ENSBTAG00000008395	ENSBTAG00000008395	7:21440492-21447907	0.635255	0	0.933156	OK
ENSBTAG00000023186	ENSBTAG00000023186	10:3586113-3586506	0.634352	0	1.58059	OK
ENSBTAG00000047336	ENSBTAG00000047336	13:75950888-75955042	0.634196	0	1.11923	OK
ENSBTAG00000012968	ENSBTAG00000012968	4:78141986-78147625	0.633995	0	1.06841	OK
ENSBTAG00000037529	ENSBTAG00000037529	19:24707450-24708485	0.632966	0	1.20033	OK
ENSBTAG00000012503	ENSBTAG00000012503	5:111423286-111424897	0.632869	0	0.899689	OK
ENSBTAG00000025329	ENSBTAG00000025329	10:89245838-89248163	0.632803	0	0.623397	OK
ENSBTAG00000039363	ENSBTAG00000039363	24:49176825-49177310	0.632082	0	1.28076	OK
ENSBTAG00000024595	ENSBTAG00000024595	9:77893556-77907314	0.631566	0	1.22438	OK
ENSBTAG00000018094	ENSBTAG00000018094	25:27014539-27018793	0.63147	0	0.901118	OK
ENSBTAG00000016783	ENSBTAG00000016783	7:5517842-5526391	0.63144	0	1.19227	OK
ENSBTAG00000038345	ENSBTAG00000038345	29:28175521-28176469	0.631219	0	1.74732	OK
ENSBTAG00000016852	ENSBTAG00000016852	3:108926198-108941387	0.631211	0	1.17076	OK
ENSBTAG00000002445	ENSBTAG00000002445	4:113141787-113150955	0.631186	0	0.856619	OK
ENSBTAG00000015708	ENSBTAG00000015708	22:53998566-54003908	0.630862	0.105911	1.05911	OK
ENSBTAG00000007193	ENSBTAG00000007193	19:14782700-14786690	0.630784	0	1.34978	OK
ENSBTAG00000007362	ENSBTAG00000007362	22:58702012-58724435	0.630715	0	0.663172	OK
ENSBTAG00000033545	ENSBTAG00000033545	27:5483407-5539158	0.629603	0	1.0673	OK
ENSBTAG00000015437	ENSBTAG00000015437	11:100042201-100049863	0.629472	0	1.23986	OK
ENSBTAG00000019965	ENSBTAG00000019965	12:90796150-90813125	0.62899	0	1.21798	OK
ENSBTAG00000017922	ENSBTAG00000017922	11:99563894-99573250	0.628735	0.0954619	1.14554	OK
ENSBTAG00000007642	ENSBTAG00000007642	3:31967840-31971684	0.628653	0	1.47371	OK
ENSBTAG00000010297	ENSBTAG00000010297	19:13237730-13247622	0.628449	0	1.16981	OK
ENSBTAG00000001823	ENSBTAG00000001823	20:4908291-4918945	0.628295	0	1.13893	OK
ENSBTAG00000018736	ENSBTAG00000018736	7:8133742-8134672	0.628172	0	1.11321	OK
ENSBTAG00000039077	ENSBTAG00000039077	10:22836232-22836789	0.628042	0	1.81629	OK
ENSBTAG00000015794	ENSBTAG00000015794	3:14208424-14215064	0.62768	0.224088	1.0308	OK

ENSBTAG00000030677	ENSBTAG00000030677	11:93935689-93936631	0.626974	0	1.75845	OK
ENSBTAG00000010957	ENSBTAG00000010957	26:44431213-44558731	0.626035	0	1.16981	OK
ENSBTAG00000030676	ENSBTAG00000030676	11:93969255-93970206	0.625585	0	1.08863	OK
ENSBTAG00000046273	ENSBTAG00000046273	19:22667522-22675073	0.624768	0	1.0852	OK
ENSBTAG00000047433	ENSBTAG00000047433	7:11339251-11340452	0.623207	0	1.66311	OK
ENSBTAG00000002380	ENSBTAG00000002380	18:45830183-45831279	0.622811	0	1.14713	OK
ENSBTAG00000047786	ENSBTAG00000047786	29:40674001-40676155	0.622675	0	0.86514	OK
ENSBTAG00000040130	ENSBTAG00000040130	4:108022063-108023023	0.621698	0	1.29411	OK
ENSBTAG00000005043	ENSBTAG00000005043	X:91232234-91236073	0.621663	0	1.02503	OK
ENSBTAG00000026250	ENSBTAG00000026250	6:30063479-30064806	0.620768	0	1.09224	OK
ENSBTAG00000030507	ENSBTAG00000030507	X:17711396-17712557	0.620427	0	1.16981	OK
ENSBTAG00000004170	ENSBTAG00000004170	8:68113366-68114189	0.619595	0	1.02124	OK
ENSBTAG00000013943	ENSBTAG00000013943	27:32683064-32685824	0.618868	0	0.821189	OK
ENSBTAG00000020979	ENSBTAG00000020979	19:37725130-37744573	0.618855	0.0621979	0.870771	OK
ENSBTAG00000005972	ENSBTAG00000005972	8:38400858-38401511	0.618795	0	0.951257	OK
ENSBTAG00000034711	ENSBTAG00000034711	15:50964145-50965099	0.618444	0	1.73633	OK
ENSBTAG00000002676	ENSBTAG00000002676	18:54608485-54670718	0.618385	0.103944	1.03944	OK
ENSBTAG00000026580	ENSBTAG00000026580	9:63762921-63764019	0.618186	0	1.13146	OK
ENSBTAG00000015521	ENSBTAG00000015521	11:21147965-21151578	0.617832	0	1.0673	OK
ENSBTAG00000011381	ENSBTAG00000011381	11:72364681-72373190	0.617451	0	0.942596	OK
ENSBTAG00000008573	ENSBTAG00000008573	18:49361493-49375317	0.616538	0	2.07088	OK
ENSBTAG00000013353	ENSBTAG00000013353	18:65732472-65755773	0.616499	0.0787289	0.944747	OK
ENSBTAG00000008035	ENSBTAG00000008035	11:11283272-11283761	0.616035	0	2.11715	OK
ENSBTAG00000046347	ENSBTAG00000046347	2:29428423-29428879	0.615751	0	1.36222	OK
ENSBTAG00000016225	ENSBTAG00000016225	8:112869670-112892968	0.615652	0	1.11407	OK
ENSBTAG00000040512	ENSBTAG00000040512	3:8249980-8276724	0.614871	0.153035	1.22428	OK
ENSBTAG00000038084	ENSBTAG00000038084	22:50604476-50605424	0.614698	0	1.09207	OK
ENSBTAG00000021393	ENSBTAG00000021393	10:98332058-98334041	0.614109	0.104416	1.04416	OK
ENSBTAG00000040490	ENSBTAG00000040490	13:11543627-11636251	0.613503	0.159336	1.03568	OK
ENSBTAG00000039012	ENSBTAG00000039012	19:28908995-28911151	0.613381	0	1.14306	OK

ENSBTAG00000000524	ENSBTAG00000000524	1:146555392-146558844	0.613345	0	1.01168	OK
ENSBTAG00000009292	ENSBTAG00000009292	19:6237390-6239838	0.612932	0	1.13768	OK
ENSBTAG00000015205	ENSBTAG00000015205	11:45780702-45829966	0.612664	0.0917399	1.10088	OK
ENSBTAG00000000546	ENSBTAG00000000546	19:36512063-36515184	0.612415	0.141723	1.13378	OK
ENSBTAG00000032656	ENSBTAG00000032656	1:73792743-73802039	0.612325	0.101748	1.11923	OK
ENSBTAG00000012581	ENSBTAG00000012581	1:64870748-64891029	0.612038	0	1.16107	OK
ENSBTAG00000004161	ENSBTAG00000004161	7:52414546-52457128	0.611719	0.11427	1.02843	OK
ENSBTAG00000010993	ENSBTAG00000010993	21:68778064-68786417	0.611509	0	1.74138	OK
ENSBTAG00000013071	ENSBTAG00000013071	16:47942385-47947791	0.611218	0	0.760839	OK
ENSBTAG00000010192	ENSBTAG00000010192	21:7011496-7012012	0.610946	0	2.00637	OK
ENSBTAG00000048052	ENSBTAG00000048052	15:51054145-51055267	0.610032	0	1.19341	OK
ENSBTAG00000031287	ENSBTAG00000031287	22:54635350-54636965	0.60959	0	0.89746	OK
ENSBTAG00000027015	ENSBTAG00000027015	11:30962349-30963034	0.609153	0	1.78806	OK
ENSBTAG00000001675	ENSBTAG00000001675	10:37035179-37043518	0.607666	0	0.808365	OK
ENSBTAG00000010532	ENSBTAG00000010532	19:27685378-27688137	0.607248	0.150096	1.05067	OK
ENSBTAG00000018262	ENSBTAG00000018262	11:72350277-72353477	0.607072	0	1.28129	OK
ENSBTAG00000007001	ENSBTAG00000007001	6:109090377-109092922	0.606786	0	0.683356	OK
ENSBTAG00000020850	ENSBTAG00000020850	2:132731028-132738645	0.605934	0	0.768776	OK
ENSBTAG00000002710	ENSBTAG00000002710	10:20786858-20789307	0.605724	0	1.1115	OK
ENSBTAG00000021394	ENSBTAG00000021394	10:20851382-20853174	0.605612	0	1.16324	OK
ENSBTAG00000000198	ENSBTAG00000000198	5:44443586-44448198	0.605496	0	1.39903	OK
ENSBTAG00000020663	ENSBTAG00000020663	5:57770768-57774153	0.604955	0	1.18998	OK
ENSBTAG00000018031	ENSBTAG00000018031	22:60507745-60556978	0.604943	0	0.788574	OK
ENSBTAG00000015130	ENSBTAG00000015130	23:35437309-35439298	0.604359	0.104101	1.04101	OK
ENSBTAG00000001357	ENSBTAG00000001357	2:120002010-120003207	0.604358	0	1.00905	OK
ENSBTAG00000025669	ENSBTAG00000025669	23:7394228-7395863	0.604202	0	0.971447	OK
ENSBTAG00000047556	ENSBTAG00000047556	15:51066336-51067463	0.60419	0	1.18998	OK
ENSBTAG00000004989	ENSBTAG00000004989	4:93706153-93717881	0.604057	0.080787	1.05023	OK
ENSBTAG00000037764	ENSBTAG00000037764	7:20040117-20041620	0.603425	0	0.964337	OK
ENSBTAG00000010977	ENSBTAG00000010977	2:13724667-13761787	0.603389	0.176219	1.05731	OK

ENSBTAG00000001079	ENSBTAG00000001079	5:39642690-39643783	0.603212	0	1.13663	OK
ENSBTAG00000008020	ENSBTAG00000008020	5:65648254-65648799	0.602801	0	1.13976	OK
ENSBTAG00000039052	ENSBTAG00000039052	23:29581528-29582479	0.602773	0	1.30635	OK
ENSBTAG00000031524	ENSBTAG00000031524	13:59023079-59076365	0.602359	0	1.14186	OK
ENSBTAG00000003037	ENSBTAG00000003037	23:7082808-7086786	0.602352	0	1.07922	OK
ENSBTAG00000020019	ENSBTAG00000020019	22:53944134-53945136	0.602337	0	1.03322	OK
ENSBTAG00000002943	ENSBTAG00000002943	11:36063964-36064471	0.601961	0	1.22519	OK
ENSBTAG00000011982	ENSBTAG00000011982	16:72740450-72753761	0.601899	0	0.978993	OK
ENSBTAG00000003107	ENSBTAG00000003107	17:63254161-63263364	0.601331	0	0.912592	OK
ENSBTAG00000005261	ENSBTAG00000005261	18:49726102-49751466	0.601054	0.0360036	0.504051	OK
ENSBTAG00000003779	ENSBTAG00000003779	X:101028238-101103881	0.600642	0.100562	1.00562	OK
ENSBTAG00000016541	ENSBTAG00000016541	16:75611799-75642910	0.60017	0.0874396	1.04928	OK
ENSBTAG00000045509	ENSBTAG00000045509	13:57115095-57116235	0.599052	0	1.08977	OK
ENSBTAG00000003791	ENSBTAG00000003791	3:59403908-59459794	0.599021	0.102758	1.02758	OK
ENSBTAG00000008853	ENSBTAG00000008853	28:13902310-13920905	0.598838	0.198235	1.0903	OK
ENSBTAG00000044118	ENSBTAG00000044118	5:48053845-48199963	0.59881	0	0.898945	OK
ENSBTAG00000010155	ENSBTAG00000010155	19:14735717-14741999	0.598495	0	1.21086	OK
ENSBTAG00000027213	ENSBTAG00000027213	1:105171798-105172518	0.597967	0	1.4379	OK
ENSBTAG00000034325	ENSBTAG00000034325	8:22909808-22910378	0.597637	0	1.08977	OK
ENSBTAG00000046585	ENSBTAG00000046585	21:67427513-67431509	0.59743	0	1.19486	OK
ENSBTAG00000038149	ENSBTAG00000038149	18:61091347-61143063	0.596579	0	1.47294	OK
ENSBTAG00000004004	ENSBTAG00000004004	5:113160818-113174721	0.596351	0.0846167	1.0154	OK
ENSBTAG00000004248	ENSBTAG00000004248	18:10250179-10263073	0.596158	0	0.885399	OK
ENSBTAG00000023002	ENSBTAG00000023002	13:73303265-73375173	0.595849	0	0.691177	OK
ENSBTAG00000006095	ENSBTAG00000006095	9:50873668-50881408	0.595735	0	0.682391	OK
ENSBTAG00000048142	ENSBTAG00000048142	11:9193843-9194965	0.595641	0	1.10726	OK
ENSBTAG00000031829	ENSBTAG00000031829	X:91678172-91687290	0.59559	0.0677099	1.15107	OK
ENSBTAG00000031590	ENSBTAG00000031590	29:45870039-45877738	0.595468	0.0775204	0.930245	OK
ENSBTAG00000010521	ENSBTAG00000010521	25:36728416-36729808	0.595221	0	1.01201	OK
ENSBTAG00000046849	ENSBTAG00000046849	8:23177938-23178499	0.595096	0	1.84543	OK

ENSBTAG00000034338	ENSBTAG00000034338	15:22136720-22171026	0.594717	0	1.60013	OK
ENSBTAG00000011196	ENSBTAG00000011196	2:130769171-130775911	0.594478	0	1.09207	OK
ENSBTAG00000046728	ENSBTAG00000046728	29:45985246-45986608	0.594003	0	1.06417	OK
ENSBTAG00000024772	ENSBTAG00000024772	25:36321719-36325556	0.593671	0	0.862224	OK
ENSBTAG00000010885	ENSBTAG00000010885	9:34872095-34875354	0.593029	0.190602	1.01654	OK
ENSBTAG00000047876	ENSBTAG00000047876	6:119323445-119356880	0.592877	0	0.459786	OK
ENSBTAG00000027516	ENSBTAG00000027516	15:81734844-81738412	0.592695	0.116063	0.928507	OK
ENSBTAG00000001660	ENSBTAG00000001660	29:26356892-26373003	0.592603	0.18637	1.02503	OK
ENSBTAG00000021205	ENSBTAG00000021205	11:101502264-101517588	0.591987	0	0.956138	OK
ENSBTAG00000008409	ENSBTAG00000008409	14:13769243-13774438	0.59197	0.0938608	1.03247	OK
ENSBTAG00000014060	ENSBTAG00000014060	17:12322225-12340189	0.591846	0	1.57338	OK
ENSBTAG00000019812	ENSBTAG00000019812	19:51665457-51667844	0.591066	0	1.17646	OK
ENSBTAG00000013076	ENSBTAG00000013076	29:37526200-37555971	0.590584	0.123912	1.05325	OK
ENSBTAG00000011284	ENSBTAG00000011284	3:16530783-16534816	0.589657	0.100905	1.00905	OK
ENSBTAG00000024952	ENSBTAG00000024952	15:81464786-81465731	0.58932	0	1.75286	OK
ENSBTAG00000002817	ENSBTAG00000002817	19:20442060-20449646	0.589252	0.138268	1.03701	OK
ENSBTAG00000005312	ENSBTAG00000005312	7:6342025-6382668	0.589102	0.0667926	0.935096	OK
ENSBTAG00000030941	ENSBTAG00000030941	22:59916189-59916744	0.5889	0	1.11923	OK
ENSBTAG00000002206	ENSBTAG00000002206	X:100376965-100431416	0.588654	0.167116	1.08626	OK
ENSBTAG00000026633	ENSBTAG00000026633	9:43523813-43524276	0.588575	0	1.34162	OK
ENSBTAG00000011787	ENSBTAG00000011787	17:66685451-66691553	0.588209	0	1.05534	OK
ENSBTAG00000046741	ENSBTAG00000046741	15:48648061-48649006	0.587523	0	1.31465	OK
ENSBTAG00000005788	ENSBTAG00000005788	3:27742478-27783993	0.587508	0	1.05172	OK
ENSBTAG00000021557	ENSBTAG00000021557	18:55786903-55787938	0.587414	0	1.00028	OK
ENSBTAG00000005971	ENSBTAG00000005971	23:16376205-16390351	0.587413	0	1.10388	OK
ENSBTAG00000002670	ENSBTAG00000002670	28:44952354-44953778	0.586576	0	0.9401	OK
ENSBTAG00000010106	ENSBTAG00000010106	23:15707555-15714372	0.586084	0	0.940218	OK
ENSBTAG00000003212	ENSBTAG00000003212	13:67118489-67120879	0.585933	0	2.03274	OK
ENSBTAG00000012775	ENSBTAG00000012775	20:8803855-8804333	0.58593	0	1.29952	OK
ENSBTAG00000004259	ENSBTAG00000004259	11:87191214-87307998	0.58562	0	1.05402	OK

ENSBTAG00000023039	ENSBTAG00000023039	28:31044241-31057065	0.583903	0	2.91951	OK
ENSBTAG00000017659	ENSBTAG00000017659	1:137030484-137107221	0.583841	0	1.09772	OK
ENSBTAG00000033021	ENSBTAG00000033021	26:20914836-20916212	0.583091	0	1.43457	OK
ENSBTAG00000014897	ENSBTAG00000014897	7:39677303-39692612	0.58259	0.0682006	0.954808	OK
ENSBTAG00000016742	ENSBTAG00000016742	13:75267858-75284423	0.582386	0	1.10844	OK
ENSBTAG00000048020	ENSBTAG00000048020	17:12206072-12250205	0.582058	0	1.26254	OK
ENSBTAG00000000422	ENSBTAG00000000422	24:3674812-3678046	0.581812	0.064025	0.89635	OK
ENSBTAG00000046017	ENSBTAG00000046017	9:45452890-45455659	0.581533	0	1.18183	OK
ENSBTAG00000010606	ENSBTAG00000010606	27:24316622-24317477	0.581509	0	0.968688	OK
ENSBTAG00000046488	ENSBTAG00000046488	10:27523854-27524781	0.580453	0	1.34017	OK
ENSBTAG00000020371	ENSBTAG00000020371	13:75327438-75339408	0.580194	0	1.06822	OK
ENSBTAG00000008060	ENSBTAG00000008060	17:54404986-54408726	0.579475	0	1.10254	OK
ENSBTAG00000021443	ENSBTAG00000021443	3:17208989-17224318	0.57903	0	1.0125	OK
ENSBTAG00000000457	ENSBTAG00000000457	29:9934144-9935558	0.578835	0	1.02503	OK
ENSBTAG00000007686	ENSBTAG00000007686	9:105681989-105684410	0.577964	0	0.949803	OK
ENSBTAG00000001385	ENSBTAG00000001385	21:22702079-22714918	0.576968	0.0624795	0.874713	OK
ENSBTAG00000000359	ENSBTAG00000000359	10:103984740-104002866	0.576215	0	1.13643	OK
ENSBTAG00000038292	ENSBTAG00000038292	3:110649044-110649921	0.576168	0	0.944387	OK
ENSBTAG00000026859	ENSBTAG00000026859	19:24654313-24655261	0.575992	0	1.09207	OK
ENSBTAG00000010553	ENSBTAG00000010553	8:90432692-90433162	0.575951	0	1.32164	OK
ENSBTAG00000019506	ENSBTAG00000019506	21:33559734-33563692	0.575112	0	1.16652	OK
ENSBTAG00000020393	ENSBTAG00000020393	12:82907166-82916245	0.574831	0	1.07204	OK
ENSBTAG00000014918	ENSBTAG00000014918	7:2869389-2879155	0.57466	0	1.08083	OK
ENSBTAG00000006969	ENSBTAG00000006969	23:28101726-28105320	0.57352	0.123395	0.987161	OK
ENSBTAG00000010348	ENSBTAG00000010348	29:50849496-50854698	0.573268	0	0.847106	OK
ENSBTAG00000019667	ENSBTAG00000019667	7:2453802-2457284	0.572774	0	1.00028	OK
ENSBTAG00000021166	ENSBTAG00000021166	16:72811910-72814133	0.572545	0.093143	1.02457	OK
ENSBTAG00000015085	ENSBTAG00000015085	11:106229011-106233455	0.572241	0	1.71672	OK
ENSBTAG00000038227	ENSBTAG00000038227	10:27026975-27027914	0.572193	0	1.32305	OK
ENSBTAG00000009058	ENSBTAG00000009058	2:71893617-71894865	0.572024	0	0.995466	OK

ENSBTAG00000044115	ENSBTAG00000044115	X:60165466-60168537	0.571431	0	2.88411	OK
ENSBTAG00000003697	ENSBTAG00000003697	16:43475059-43486514	0.571406	0	1.13612	OK
ENSBTAG00000046565	ENSBTAG00000046565	X:32834121-32835672	0.571376	0	0.869987	OK
ENSBTAG00000011193	ENSBTAG00000011193	2:130783986-130788357	0.570837	0	0.967556	OK
ENSBTAG00000003967	ENSBTAG00000003967	29:42882592-42889928	0.570055	0	1.04469	OK
ENSBTAG00000048214	ENSBTAG00000048214	15:48635494-48636412	0.570021	0	1.35331	OK
ENSBTAG00000039581	ENSBTAG00000039581	2:20794854-20797028	0.569251	0	1.01623	OK
ENSBTAG00000040131	ENSBTAG00000040131	3:26825752-26889208	0.569053	0.0883349	1.06002	OK
ENSBTAG00000002981	ENSBTAG00000002981	19:26021978-26026009	0.569045	0	1.00374	OK
ENSBTAG00000031503	ENSBTAG00000031503	5:56537020-56539323	0.569044	0	1.04469	OK
ENSBTAG00000021777	ENSBTAG00000021777	29:46114977-46119340	0.568507	0	1.09637	OK
ENSBTAG00000024653	ENSBTAG00000024653	7:43401908-43403009	0.567945	0	1.12838	OK
ENSBTAG00000024903	ENSBTAG00000024903	19:61226689-61229571	0.567621	0.0988811	0.988811	OK
ENSBTAG00000045572	ENSBTAG00000045572	18:25984797-25986558	0.567487	0	0.823054	OK
ENSBTAG00000046638	ENSBTAG00000046638	8:23073253-23073823	0.567044	0	1.08977	OK
ENSBTAG00000005255	ENSBTAG00000005255	15:83845501-83846449	0.56637	0	1.31049	OK
ENSBTAG00000003140	ENSBTAG00000003140	1:81281007-81286776	0.565769	0	1.00189	OK
ENSBTAG00000017537	ENSBTAG00000017537	13:78319019-78368752	0.565553	0.0938608	1.03247	OK
ENSBTAG00000013149	ENSBTAG00000013149	6:95805272-95805962	0.565466	0	0.900248	OK
ENSBTAG00000035686	ENSBTAG00000035686	15:51778326-51779310	0.565442	0	1.05212	OK
ENSBTAG00000046840	ENSBTAG00000046840	25:9666053-9680637	0.565324	0	1.06495	OK
ENSBTAG00000013157	ENSBTAG00000013157	18:53874462-53889874	0.564706	0	1.039	OK
ENSBTAG00000008624	ENSBTAG00000008624	21:27346490-27348447	0.564664	0	0.952229	OK
ENSBTAG00000024620	ENSBTAG00000024620	X:113784792-113785869	0.56435	0	1.00611	OK
ENSBTAG00000019252	ENSBTAG00000019252	2:126844576-126857428	0.564217	0	1.01554	OK
ENSBTAG00000046279	ENSBTAG00000046279	1:93990986-93992443	0.563899	0	1.13689	OK
ENSBTAG00000046281	ENSBTAG00000046281	2:68534301-68535069	0.563193	0	0.862737	OK
ENSBTAG00000047298	ENSBTAG00000047298	15:77719052-77720542	0.563173	0	1.30498	OK
ENSBTAG00000017051	ENSBTAG00000017051	3:22064165-22085071	0.56308	0.114459	0.915675	OK
ENSBTAG00000045814	ENSBTAG00000045814	18:65370606-65371166	0.562941	0	1.10923	OK

ENSBTAG00000008428	ENSBTAG00000008428	4:7528099-7556112	0.562691	0	0.973927	OK
ENSBTAG00000010264	ENSBTAG00000010264	25:39359289-39376730	0.562502	0	2.81251	OK
ENSBTAG00000024507	ENSBTAG00000024507	3:9705463-9709562	0.561875	0	1.01999	OK
ENSBTAG00000005892	ENSBTAG00000005892	11:3016324-3056118	0.561837	0.105025	0.840201	OK
ENSBTAG00000008065	ENSBTAG00000008065	5:117240032-117248391	0.561353	0	1.11521	OK
ENSBTAG00000039706	ENSBTAG00000039706	7:41588233-41590604	0.561193	0	1.33156	OK
ENSBTAG00000023147	ENSBTAG00000023147	21:33292503-33294348	0.560719	0	1.01003	OK
ENSBTAG00000001618	ENSBTAG00000001618	21:22883304-22935573	0.560515	0.0756925	0.643387	OK
ENSBTAG00000014006	ENSBTAG00000014006	16:61858194-61886167	0.560422	0	1.01887	OK
ENSBTAG00000026829	ENSBTAG00000026829	2:72355936-72358049	0.559285	0.0979919	0.979919	OK
ENSBTAG00000017220	ENSBTAG00000017220	19:48768617-48772014	0.558817	0	1.01374	OK
ENSBTAG00000011567	ENSBTAG00000011567	10:36546257-36554962	0.55881	0.0540901	0.649081	OK
ENSBTAG00000015461	ENSBTAG00000015461	4:43994071-43994562	0.557675	0	1.26511	OK
ENSBTAG00000026796	ENSBTAG00000026796	15:52287688-52290947	0.557585	0	0.940313	OK
ENSBTAG00000016818	ENSBTAG00000016818	2:122784557-122889605	0.55752	0.127341	1.01873	OK
ENSBTAG00000021430	ENSBTAG00000021430	25:35018573-35023604	0.556819	0	0.815185	OK
ENSBTAG00000038787	ENSBTAG00000038787	15:50601201-50602143	0.55622	0	1.31883	OK
ENSBTAG00000015779	ENSBTAG00000015779	21:34970952-35025051	0.554832	0.0776658	0.931989	OK
ENSBTAG00000002009	ENSBTAG00000002009	2:20918859-20919297	0.554276	0	1.46849	OK
ENSBTAG00000013631	ENSBTAG00000013631	16:64948331-64958885	0.554188	0.15539	0.932339	OK
ENSBTAG00000006535	ENSBTAG00000006535	X:61853457-61857225	0.553791	0.109903	0.934174	OK
ENSBTAG00000019144	ENSBTAG00000019144	15:46526625-46527585	0.553359	0	1.07842	OK
ENSBTAG00000005033	ENSBTAG00000005033	25:26498126-26503844	0.55324	0	1.40061	OK
ENSBTAG00000017627	ENSBTAG00000017627	8:75636007-75659670	0.552458	0	1.01416	OK
ENSBTAG00000000662	ENSBTAG00000000662	5:113634003-113683472	0.552426	0.200701	0.769355	OK
ENSBTAG00000004043	ENSBTAG00000004043	18:63174841-63177907	0.551919	0	1.05038	OK
ENSBTAG00000014305	ENSBTAG00000014305	29:28119644-28120592	0.551811	0	1.74732	OK
ENSBTAG00000005647	ENSBTAG00000005647	21:71077799-71078924	0.551731	0	0.920253	OK
ENSBTAG00000047784	ENSBTAG00000047784	2:120145263-120146286	0.551514	0	1.01201	OK
ENSBTAG00000030172	ENSBTAG00000030172	19:56015759-56041651	0.551306	0.104945	0.944507	OK

ENSBTAG00000008336	ENSBTAG00000008336	2:121610858-121616754	0.551158	0	1.65347	OK
ENSBTAG00000008182	ENSBTAG00000008182	18:53501994-53508395	0.549939	0.0643434	0.900807	OK
ENSBTAG00000032227	ENSBTAG00000032227	14:79753909-79759169	0.549893	0	1.06365	OK
ENSBTAG00000009042	ENSBTAG00000009042	3:34070246-34078942	0.549419	0.0892102	0.981313	OK
ENSBTAG00000033080	ENSBTAG00000033080	27:41930645-41934590	0.549261	0.0943742	0.943742	OK
ENSBTAG00000002219	ENSBTAG00000002219	16:52336170-52352541	0.548119	0	0.764675	OK
ENSBTAG00000012927	ENSBTAG00000012927	25:26470487-26475202	0.547892	0	1.04442	OK
ENSBTAG00000008116	ENSBTAG00000008116	19:20391105-20398885	0.547414	0	0.988676	OK
ENSBTAG00000046148	ENSBTAG00000046148	19:22856387-22859271	0.547158	0	1.08407	OK
ENSBTAG00000012772	ENSBTAG00000012772	11:103039730-103051510	0.547129	0	0.941168	OK
ENSBTAG00000015984	ENSBTAG00000015984	18:52178834-52183405	0.547029	0	1.08003	OK
ENSBTAG00000047191	ENSBTAG00000047191	5:105762454-105764041	0.546338	0	1.04377	OK
ENSBTAG00000004690	ENSBTAG00000004690	19:57437657-57439771	0.545709	0	1.49392	OK
ENSBTAG00000015563	ENSBTAG00000015563	22:44179969-44183679	0.545408	0.0947629	0.947629	OK
ENSBTAG00000010582	ENSBTAG00000010582	11:104860031-104880077	0.545349	0	0.61421	OK
ENSBTAG00000038042	ENSBTAG00000038042	2:106906772-106914272	0.544917	0	1.06002	OK
ENSBTAG00000004894	ENSBTAG00000004894	4:114127187-114137744	0.544702	0	0.963056	OK
ENSBTAG00000001198	ENSBTAG00000001198	4:113780810-113781704	0.54433	0	1.38964	OK
ENSBTAG00000003260	ENSBTAG00000003260	7:59688670-59689933	0.543699	0	1.31152	OK
ENSBTAG00000039720	ENSBTAG00000039720	25:37354258-37362187	0.54351	0	1.18318	OK
ENSBTAG00000011813	ENSBTAG00000011813	29:6351876-6462240	0.543168	0	1.74101	OK
ENSBTAG00000047364	ENSBTAG00000047364	3:57390506-57391002	0.543102	0	1.25236	OK
ENSBTAG00000013125	ENSBTAG00000013125	18:52202331-52215619	0.543088	0	0.946186	OK
ENSBTAG00000001325	ENSBTAG00000001325	17:73500518-73527334	0.542976	0	0.975369	OK
ENSBTAG00000008330	ENSBTAG00000008330	2:121552617-121576145	0.542227	0.1617	0.88935	OK
ENSBTAG00000015636	ENSBTAG00000015636	7:15876345-15880691	0.542077	0	0.989756	OK
ENSBTAG00000015490	ENSBTAG00000015490	11:78292605-78322321	0.542043	0	0.882032	OK
ENSBTAG00000016646	ENSBTAG00000016646	5:105663224-105664712	0.541674	0	0.974058	OK
ENSBTAG00000000773	ENSBTAG00000000773	29:41741320-41750148	0.541625	0.0837609	1.17265	OK
ENSBTAG00000044087	ENSBTAG00000044087	24:20313070-20313992	0.541433	0	1.88576	OK

ENSBTAG00000010846	ENSBTAG00000010846	11:2730997-2762524	0.540845	0	0.768142	OK
ENSBTAG00000014526	ENSBTAG00000014526	7:22748791-22767365	0.540784	0.0964851	0.964851	OK
ENSBTAG00000046509	ENSBTAG00000046509	3:25703049-25705109	0.540623	0	1.05911	OK
ENSBTAG00000018077	ENSBTAG00000018077	18:51991409-51995823	0.54059	0	1.03013	OK
ENSBTAG00000046189	ENSBTAG00000046189	10:26126016-26126478	0.540237	0	1.34453	OK
ENSBTAG00000038098	ENSBTAG00000038098	10:23099396-23099928	0.539838	0	1.72547	OK
ENSBTAG00000046510	ENSBTAG00000046510	18:58589151-58590096	0.539417	0	1.31465	OK
ENSBTAG00000010420	ENSBTAG00000010420	12:5086498-5200843	0.53901	0.0593456	0.830839	OK
ENSBTAG00000044908	ENSBTAG00000044908	2:118063791-118064078	0.538984	0	2.16436	OK
ENSBTAG00000006346	ENSBTAG00000006346	20:62630505-62697437	0.53869	0.0952424	0.952424	OK
ENSBTAG00000002687	ENSBTAG00000002687	18:41991379-41994613	0.538601	0.12805	0.89635	OK
ENSBTAG00000000009	ENSBTAG00000000009	18:12379749-12382722	0.538473	0	0.939338	OK
ENSBTAG00000011752	ENSBTAG00000011752	21:7768428-7793372	0.538455	0.0806768	0.685753	OK
ENSBTAG00000038662	ENSBTAG00000038662	12:36310778-36318420	0.537758	0	0.948559	OK
ENSBTAG00000034360	ENSBTAG00000034360	20:10018766-10022546	0.537748	0	1.21322	OK
ENSBTAG00000012975	ENSBTAG00000012975	28:31154551-31168822	0.537627	0	1.12455	OK
ENSBTAG00000037885	ENSBTAG00000037885	7:53853038-53855495	0.537406	0.168545	0.926995	OK
ENSBTAG00000008032	ENSBTAG00000008032	4:115901832-115951987	0.537335	0	1.03234	OK
ENSBTAG00000039530	ENSBTAG00000039530	8:73147852-73152844	0.537009	0.147057	0.882345	OK
ENSBTAG00000001397	ENSBTAG00000001397	3:103537255-103538203	0.53695	0	1.74732	OK
ENSBTAG00000015912	ENSBTAG00000015912	18:46370902-46381860	0.536537	0	1.0263	OK
ENSBTAG00000009850	ENSBTAG00000009850	4:113720918-113729756	0.536475	0	1.31604	OK
ENSBTAG00000018334	ENSBTAG00000018334	13:62226993-62236866	0.536224	0	1.00452	OK
ENSBTAG00000018489	ENSBTAG00000018489	1:106700431-106731202	0.535748	0.12406	0.992484	OK
ENSBTAG00000039901	ENSBTAG00000039901	23:29176695-29177619	0.535628	0	1.34453	OK
ENSBTAG00000013052	ENSBTAG00000013052	25:20058141-20076660	0.535548	0	1.05911	OK
ENSBTAG00000045507	ENSBTAG00000045507	18:13740621-13753849	0.535353	0.0589738	0.373501	OK
ENSBTAG00000047081	ENSBTAG00000047081	28:25049573-25050205	0.535208	0	1.00839	OK
ENSBTAG00000045591	ENSBTAG00000045591	10:60202861-60203343	0.534987	0	1.75472	OK
ENSBTAG00000025931	ENSBTAG00000025931	15:55029089-55043623	0.533906	0.206781	0.896052	OK

ENSBTAG00000005311	ENSBTAG00000005311	5:113138059-113150601	0.533838	0	1.60151	OK
ENSBTAG00000046283	ENSBTAG00000046283	19:9891231-9892158	0.533596	0	1.21798	OK
ENSBTAG00000037429	ENSBTAG00000037429	9:17302417-17303587	0.533316	0	1.06183	OK
ENSBTAG00000012081	ENSBTAG00000012081	4:62080987-62082357	0.532044	0	1.05796	OK
ENSBTAG00000014505	ENSBTAG00000014505	21:22060137-22066205	0.532028	0	1.05873	OK
ENSBTAG00000006434	ENSBTAG00000006434	6:7388727-7590933	0.531956	0.244113	0.829985	OK
ENSBTAG00000039664	ENSBTAG00000039664	22:49660684-49661474	0.531285	0	1.04839	OK
ENSBTAG00000018501	ENSBTAG00000018501	20:67789365-67851470	0.530628	0.124321	0.652686	OK
ENSBTAG00000037597	ENSBTAG00000037597	23:31235364-31236309	0.530586	0	1.31465	OK
ENSBTAG00000020523	ENSBTAG00000020523	14:83268539-83269856	0.530236	0	0.943312	OK
ENSBTAG00000007772	ENSBTAG00000007772	25:39515687-39526238	0.529915	0	0.918504	OK
ENSBTAG00000046939	ENSBTAG00000046939	11:103668230-103672322	0.52953	0	0.797054	OK
ENSBTAG00000014881	ENSBTAG00000014881	19:27582169-27585794	0.529518	0	1.05903	OK
ENSBTAG00000015772	ENSBTAG00000015772	21:35027785-35036053	0.5295	0	1.02673	OK
ENSBTAG00000016667	ENSBTAG00000016667	13:54471016-54499223	0.529326	0	0.774665	OK
ENSBTAG00000019698	ENSBTAG00000019698	3:106486405-106489042	0.529123	0	1.48109	OK
ENSBTAG00000024563	ENSBTAG00000024563	17:7050399-7050956	0.528985	0	1.14607	OK
ENSBTAG00000011399	ENSBTAG00000011399	5:77502139-77528802	0.528852	0.17023	1.13487	OK
ENSBTAG00000015266	ENSBTAG00000015266	29:40616948-40626477	0.528791	0	1.04574	OK
ENSBTAG00000015742	ENSBTAG00000015742	7:16087158-16094419	0.528768	0	1.02786	OK
ENSBTAG00000021126	ENSBTAG00000021126	X:134582940-134584743	0.528318	0.117579	0.940634	OK
ENSBTAG00000007971	ENSBTAG00000007971	15:82560716-82561670	0.528021	0	0.868163	OK
ENSBTAG00000027017	ENSBTAG00000027017	11:27200796-27204850	0.527651	0.0921892	0.921892	OK
ENSBTAG00000021329	ENSBTAG00000021329	29:41754016-41755558	0.527409	0	0.939947	OK
ENSBTAG00000015341	ENSBTAG00000015341	14:83664778-83783770	0.526993	0	1.0157	OK
ENSBTAG00000010644	ENSBTAG00000010644	5:68385644-68645774	0.525279	0	0.955438	OK
ENSBTAG00000025410	ENSBTAG00000025410	23:27024476-27027205	0.525174	0	2.62587	OK
ENSBTAG00000010865	ENSBTAG00000010865	5:75343293-75464712	0.524817	0	0.965622	OK
ENSBTAG00000021191	ENSBTAG00000021191	18:55071101-55087454	0.524016	0.06895	0.8274	OK
ENSBTAG00000000459	ENSBTAG00000000459	22:15688176-15708173	0.523603	0	0.749	OK

ENSBTAG00000019618	ENSBTAG00000019618	3:10399266-10400202	0.523195	0	1.99093	OK
ENSBTAG00000030908	ENSBTAG00000030908	15:82728160-82729105	0.523052	0	0.876432	OK
ENSBTAG00000016218	ENSBTAG00000016218	11:98770574-98775940	0.522815	0	0.984646	OK
ENSBTAG00000037470	ENSBTAG00000037470	14:38614592-38615161	0.522547	0	1.09169	OK
ENSBTAG00000046374	ENSBTAG00000046374	29:9175978-9176500	0.522528	0	1.18998	OK
ENSBTAG00000017322	ENSBTAG00000017322	1:107687595-107688174	0.522315	0	1.07283	OK
ENSBTAG00000046282	ENSBTAG00000046282	9:33941838-33947630	0.520893	0	0.914447	OK
ENSBTAG00000030461	ENSBTAG00000030461	5:99242114-99243044	0.520588	0	1.33585	OK
ENSBTAG00000005096	ENSBTAG00000005096	9:15041835-15046972	0.520499	0	1.25236	OK
ENSBTAG00000013727	ENSBTAG00000013727	1:69732777-69782823	0.520399	0.113084	0.904673	OK
ENSBTAG00000043568	ENSBTAG00000043568	MT:9822-10168	0.520252	0	1.79529	OK
ENSBTAG00000011034	ENSBTAG00000011034	27:4422699-4481033	0.520083	0	1.63789	OK
ENSBTAG00000036297	ENSBTAG00000036297	24:729250-741978	0.519776	0	0.994868	OK
ENSBTAG00000038534	ENSBTAG00000038534	18:65906272-65910529	0.519718	0	0.906441	OK
ENSBTAG00000040147	ENSBTAG00000040147	13:42791821-42894170	0.519693	0	0.86877	OK
ENSBTAG00000047582	ENSBTAG00000047582	5:110570717-110582589	0.51961	0	1.05821	OK
ENSBTAG00000021141	ENSBTAG00000021141	11:47988168-47995400	0.519578	0.0963504	0.867153	OK
ENSBTAG00000004009	ENSBTAG00000004009	5:113174948-113185273	0.519515	0	0.929102	OK
ENSBTAG00000012057	ENSBTAG00000012057	15:84361363-84380392	0.519409	0	1.01999	OK
ENSBTAG00000018374	ENSBTAG00000018374	2:126399311-126469335	0.519311	0.204956	0.819825	OK
ENSBTAG00000037462	ENSBTAG00000037462	25:27836992-27837967	0.518584	0	1.2742	OK
ENSBTAG00000020425	ENSBTAG00000020425	23:22997915-23057661	0.517863	0.101349	0.912145	OK
ENSBTAG00000000950	ENSBTAG00000000950	28:29460097-29461928	0.517736	0	0.909474	OK
ENSBTAG00000006550	ENSBTAG00000006550	18:2907579-2917649	0.517346	0	1.09786	OK
ENSBTAG00000020600	ENSBTAG00000020600	29:45685401-45694819	0.51719	0	0.985986	OK
ENSBTAG00000038479	ENSBTAG00000038479	15:80933344-80935697	0.517124	0	1.20382	OK
ENSBTAG00000009982	ENSBTAG00000009982	3:9695128-9702141	0.517048	0	0.66334	OK
ENSBTAG00000007215	ENSBTAG00000007215	3:120320960-120324657	0.516953	0	0.918892	OK
ENSBTAG00000012223	ENSBTAG00000012223	7:19076009-19080237	0.516821	0	0.865623	OK
ENSBTAG00000012139	ENSBTAG00000012139	10:73068705-73072802	0.516668	0.0994032	0.894629	OK

ENSBTAG00000002980	ENSBTAG00000002980	18:15370926-15405215	0.516582	0.0947629	0.852866	OK
ENSBTAG00000015109	ENSBTAG00000015109	5:113060939-113070989	0.516545	0.142995	0.857971	OK
ENSBTAG00000044056	ENSBTAG00000044056	19:40068314-40071728	0.516468	0	1.04223	OK
ENSBTAG00000038911	ENSBTAG00000038911	18:64136160-64137099	0.515791	0	0.882032	OK
ENSBTAG00000012320	ENSBTAG00000012320	24:35870000-35870886	0.515306	0	0.934794	OK
ENSBTAG00000003189	ENSBTAG00000003189	28:15582653-15636803	0.515281	0	1.08312	OK
ENSBTAG00000017851	ENSBTAG00000017851	11:105990343-106015000	0.515264	0	0.94178	OK
ENSBTAG00000026586	ENSBTAG00000026586	9:88384682-88500749	0.515217	0.097853	0.880677	OK
ENSBTAG00000020409	ENSBTAG00000020409	1:147600281-147615974	0.515031	0	0.960821	OK
ENSBTAG00000014768	ENSBTAG00000014768	4:113021968-113030533	0.514191	0	0.879845	OK
ENSBTAG00000018938	ENSBTAG00000018938	25:3650393-3673924	0.51392	0	0.87284	OK
ENSBTAG00000019588	ENSBTAG00000019588	23:25855145-25863045	0.513889	0	0.874396	OK
ENSBTAG00000007553	ENSBTAG00000007553	5:105538143-105540341	0.513795	0	0.847822	OK
ENSBTAG00000047871	ENSBTAG00000047871	3:102526630-102541169	0.513693	0	1.36985	OK
ENSBTAG00000047139	ENSBTAG00000047139	17:60505859-60521906	0.513679	0	0.907008	OK
ENSBTAG00000014926	ENSBTAG00000014926	7:17900966-17903800	0.513322	0	0.899596	OK
ENSBTAG00000047810	ENSBTAG00000047810	6:118316992-118318272	0.512689	0	0.878104	OK
ENSBTAG00000007417	ENSBTAG00000007417	5:57830914-57837708	0.512668	0	0.915371	OK
ENSBTAG00000031918	ENSBTAG00000031918	2:74646309-74649158	0.512112	0	1.81629	OK
ENSBTAG00000014810	ENSBTAG00000014810	25:33956423-33958067	0.511937	0	0.988339	OK
ENSBTAG00000015062	ENSBTAG00000015062	26:37857985-37870236	0.511534	0	0.901031	OK
ENSBTAG00000038316	ENSBTAG00000038316	19:44817718-44889648	0.511422	0.22854	0.777035	OK
ENSBTAG00000018424	ENSBTAG00000018424	3:116650277-116662501	0.510043	0	0.958043	OK
ENSBTAG00000030297	ENSBTAG00000030297	7:70213685-70224203	0.510001	0	0.951257	OK
ENSBTAG00000033509	ENSBTAG00000033509	15:84074992-84075928	0.509995	0	1.10607	OK
ENSBTAG00000008477	ENSBTAG00000008477	29:48068208-48070209	0.509659	0	0.920253	OK
ENSBTAG00000019045	ENSBTAG00000019045	15:67563702-67576723	0.509592	0	0.987553	OK
ENSBTAG00000009101	ENSBTAG00000009101	17:57398013-57422729	0.50954	0.0895963	0.895963	OK
ENSBTAG00000005953	ENSBTAG00000005953	X:58699811-58706139	0.50943	0	0.974844	OK
ENSBTAG00000019099	ENSBTAG00000019099	8:71542980-71588259	0.509334	0	0.901031	OK

ENSBTAG00000011660	ENSBTAG00000011660	28:44443909-44454333	0.509256	0	2.54628	OK
ENSBTAG00000003278	ENSBTAG00000003278	5:26202175-26206357	0.509071	0	0.911142	OK
ENSBTAG00000045850	ENSBTAG00000045850	15:50479197-50480142	0.508873	0	1.09554	OK
ENSBTAG00000046717	ENSBTAG00000046717	7:53618848-53621218	0.508456	0	0.786292	OK
ENSBTAG00000016590	ENSBTAG00000016590	25:383467-386626	0.508117	0	0.963056	OK
ENSBTAG00000016502	ENSBTAG00000016502	29:36806923-36809433	0.507944	0.089635	0.89635	OK
ENSBTAG00000003101	ENSBTAG00000003101	13:80297261-80313158	0.507705	0	0.551335	OK
ENSBTAG00000030468	ENSBTAG00000030468	5:99140567-99141497	0.507559	0	1.33585	OK
ENSBTAG00000000372	ENSBTAG00000000372	10:13195493-13212891	0.507192	0	1.11788	OK
ENSBTAG00000039794	ENSBTAG00000039794	X:91543876-91546246	0.506491	0.110607	0.884859	OK
ENSBTAG00000015089	ENSBTAG00000015089	5:110014542-110017878	0.506272	0	1.22519	OK
ENSBTAG00000032233	ENSBTAG00000032233	14:78605261-78605673	0.506239	0	1.61763	OK
ENSBTAG00000039099	ENSBTAG00000039099	24:421050-422220	0.505776	0	1.31187	OK
ENSBTAG00000012956	ENSBTAG00000012956	23:28955605-28956565	0.505398	0	1.29411	OK
ENSBTAG00000016485	ENSBTAG00000016485	4:93557350-93560340	0.505125	0	0.985986	OK
ENSBTAG00000003550	ENSBTAG00000003550	29:1032167-1056464	0.504956	0.092809	1.20652	OK
ENSBTAG00000039391	ENSBTAG00000039391	X:92647395-92650330	0.504821	0.141095	0.84657	OK
ENSBTAG00000039491	ENSBTAG00000039491	18:58067006-58068879	0.504788	0	1.4182	OK
ENSBTAG00000003741	ENSBTAG00000003741	26:24330860-24403159	0.504636	0	0.799105	OK
ENSBTAG00000031843	ENSBTAG00000031843	23:29223820-29224756	0.504468	0	1.10607	OK
ENSBTAG00000039035	ENSBTAG00000039035	3:8027844-8029776	0.504395	0	0.964551	OK
ENSBTAG00000007964	ENSBTAG00000007964	22:16958694-16967839	0.504188	0	1.16455	OK
ENSBTAG00000040017	ENSBTAG00000040017	15:49372982-49373927	0.503993	0	1.31465	OK
ENSBTAG00000027275	ENSBTAG00000027275	15:79573190-79574117	0.503539	0	1.11681	OK
ENSBTAG00000015604	ENSBTAG00000015604	5:25807925-25823618	0.503514	0.0873658	0.873658	OK
ENSBTAG00000030719	ENSBTAG00000030719	7:43432444-43433410	0.503388	0	1.30635	OK
ENSBTAG00000045655	ENSBTAG00000045655	9:43459248-43460942	0.503097	0	1.27029	OK
ENSBTAG00000002330	ENSBTAG00000002330	6:73629777-73630251	0.502711	0	1.31049	OK
ENSBTAG00000019539	ENSBTAG00000019539	8:1697552-1712467	0.502173	0	0.860586	OK
ENSBTAG00000019162	ENSBTAG00000019162	19:22628975-22633394	0.502149	0	0.993079	OK

ENSBTAG00000030425	ENSBTAG00000030425	2:129895412-129897012	0.501828	0	0.892487	OK
ENSBTAG00000036113	ENSBTAG00000036113	17:73799587-73847910	0.501014	0	2.50507	OK
ENSBTAG00000010913	ENSBTAG00000010913	13:60944182-60949364	0.500667	0	0.970959	OK
ENSBTAG00000019428	ENSBTAG00000019428	22:53851968-53858019	0.500119	0.155975	0.857863	OK
ENSBTAG00000014813	ENSBTAG00000014813	11:72470283-72474442	0.500062	0	0.909855	OK
ENSBTAG00000038578	ENSBTAG00000038578	15:49516134-49517095	0.500027	0	1.29276	OK
ENSBTAG00000021942	ENSBTAG00000021942	26:22623886-22626506	0.499917	0	0.711264	OK
ENSBTAG00000009719	ENSBTAG00000009719	26:24275662-24281013	0.499345	0.102049	0.81639	OK
ENSBTAG00000011939	ENSBTAG00000011939	14:2563159-2565613	0.499231	0	0.871819	OK
ENSBTAG00000032947	ENSBTAG00000032947	6:63347074-63484980	0.499203	0	1.18998	OK
ENSBTAG00000039593	ENSBTAG00000039593	18:48475926-48479868	0.49895	0	2.49475	OK
ENSBTAG00000019535	ENSBTAG00000019535	1:132449821-132451035	0.498831	0	0.856315	OK
ENSBTAG00000046424	ENSBTAG00000046424	7:41444282-41445695	0.498626	0	1.11681	OK
ENSBTAG00000017827	ENSBTAG00000017827	3:17882973-17884629	0.498468	0	0.898017	OK
ENSBTAG00000003244	ENSBTAG00000003244	22:59609593-59620828	0.498193	0	0.853592	OK
ENSBTAG00000003078	ENSBTAG00000003078	9:34986665-34993308	0.497905	0	0.885279	OK
ENSBTAG00000038640	ENSBTAG00000038640	22:15511914-15518283	0.497884	0	1.24284	OK
ENSBTAG00000021774	ENSBTAG00000021774	7:21813143-21839351	0.497725	0	0.985986	OK
ENSBTAG00000005855	ENSBTAG00000005855	14:67463905-67464413	0.497607	0	1.22278	OK
ENSBTAG00000025220	ENSBTAG00000025220	12:86812378-87425485	0.497356	0	0.904179	OK
ENSBTAG00000039534	ENSBTAG00000039534	23:29241754-29242690	0.497281	0	1.10607	OK
ENSBTAG00000013820	ENSBTAG00000013820	26:35435895-35436976	0.497182	0	1.02402	OK
ENSBTAG00000047000	ENSBTAG00000047000	10:73628026-73629463	0.496842	0	0.882972	OK
ENSBTAG00000006295	ENSBTAG00000006295	3:28419562-28424798	0.495812	0	1.16981	OK
ENSBTAG00000005198	ENSBTAG00000005198	7:55508006-55601836	0.495692	0	0.931291	OK
ENSBTAG00000010828	ENSBTAG00000010828	21:35206044-35208959	0.495475	0	0.79231	OK
ENSBTAG00000024230	ENSBTAG00000024230	4:107184503-107185448	0.495368	0	1.31465	OK
ENSBTAG00000008923	ENSBTAG00000008923	15:1596708-1609925	0.495116	0	0.988339	OK
ENSBTAG00000010450	ENSBTAG00000010450	7:8742335-8750651	0.495068	0	0.580353	OK
ENSBTAG00000013950	ENSBTAG00000013950	13:74829024-74836849	0.49475	0	2.47375	OK

ENSBTAG00000020552	ENSBTAG00000020552	16:55096210-55154550	0.494671	0.246496	0.780572	OK
ENSBTAG00000016934	ENSBTAG00000016934	11:70746579-70755170	0.494238	0	0.988476	OK
ENSBTAG00000034712	ENSBTAG00000034712	X:14002640-14013045	0.494061	0	0.968202	OK
ENSBTAG00000005696	ENSBTAG00000005696	19:45074946-45103998	0.494057	0	0.865313	OK
ENSBTAG00000013311	ENSBTAG00000013311	X:94378600-94380496	0.49405	0.109207	0.982865	OK
ENSBTAG00000008176	ENSBTAG00000008176	12:19557640-19563107	0.493969	0	1.24827	OK
ENSBTAG00000021831	ENSBTAG00000021831	X:104607204-104607732	0.492964	0	1.17646	OK
ENSBTAG00000006213	ENSBTAG00000006213	14:58850047-58851086	0.492857	0	0.797139	OK
ENSBTAG00000038418	ENSBTAG00000038418	28:2733613-2735422	0.492445	0	1.33013	OK
ENSBTAG00000005429	ENSBTAG00000005429	10:58336605-58340848	0.492302	0	0.757525	OK
ENSBTAG00000032280	ENSBTAG00000032280	15:47719255-47720203	0.492298	0	1.31049	OK
ENSBTAG00000046139	ENSBTAG00000046139	7:15224286-15225234	0.491675	0	1.31049	OK
ENSBTAG00000016385	ENSBTAG00000016385	12:90516720-90530178	0.491628	0	0.907008	OK
ENSBTAG00000025762	ENSBTAG00000025762	19:42774248-42780091	0.49126	0	0.815899	OK
ENSBTAG00000001368	ENSBTAG00000001368	19:54035258-54045168	0.491059	0.090339	0.813051	OK
ENSBTAG00000009238	ENSBTAG00000009238	5:26161891-26163475	0.490858	0	0.963056	OK
ENSBTAG00000020638	ENSBTAG00000020638	5:71751414-71809052	0.490792	0.0970731	0.873658	OK
ENSBTAG00000039088	ENSBTAG00000039088	15:48852958-48853909	0.49046	0	1.08863	OK
ENSBTAG00000013501	ENSBTAG00000013501	19:20828076-20832961	0.490417	0	0.933891	OK
ENSBTAG00000005735	ENSBTAG00000005735	5:26535463-26539249	0.489832	0	1.04049	OK
ENSBTAG00000030325	ENSBTAG00000030325	5:107398921-107402337	0.489355	0	0.930175	OK
ENSBTAG00000000418	ENSBTAG00000000418	18:21000512-21120358	0.489258	0	0.987161	OK
ENSBTAG00000010931	ENSBTAG00000010931	4:105842747-105853468	0.489063	0	0.978222	OK
ENSBTAG00000006386	ENSBTAG00000006386	29:43008118-43008581	0.488743	0	1.56861	OK
ENSBTAG00000020533	ENSBTAG00000020533	10:68063634-68065776	0.488537	0.0966652	0.869987	OK
ENSBTAG00000014138	ENSBTAG00000014138	X:33460692-33551321	0.488401	0	1.00028	OK
ENSBTAG00000030548	ENSBTAG00000030548	19:41631641-41637275	0.488237	0	0.905874	OK
ENSBTAG00000024806	ENSBTAG00000024806	1:47006455-47007062	0.488191	0	1.32729	OK
ENSBTAG00000045564	ENSBTAG00000045564	X:126641462-126641967	0.488164	0	1.25743	OK
ENSBTAG00000007786	ENSBTAG00000007786	5:108610753-108613565	0.488135	0.0736333	0.8836	OK

ENSBTAG00000004624	ENSBTAG00000004624	28:3159663-3160998	0.487721	0	0.930593	OK
ENSBTAG00000018387	ENSBTAG00000018387	8:113001324-113068623	0.487537	0	2.43769	OK
ENSBTAG00000015004	ENSBTAG00000015004	18:46963843-46972220	0.48748	0	0.887704	OK
ENSBTAG00000016710	ENSBTAG00000016710	19:10894827-10903658	0.487385	0	0.966427	OK
ENSBTAG00000011584	ENSBTAG00000011584	1:126870799-126874541	0.48696	0.166	0.774665	OK
ENSBTAG00000020520	ENSBTAG00000020520	19:35423866-35425459	0.486784	0	0.905497	OK
ENSBTAG00000023037	ENSBTAG00000023037	28:30766406-30766924	0.486338	0	1.19917	OK
ENSBTAG00000007470	ENSBTAG00000007470	7:42623075-42637851	0.486197	0	0.848594	OK
ENSBTAG00000018603	ENSBTAG00000018603	21:14349792-14376704	0.485999	0	0.858564	OK
ENSBTAG00000001548	ENSBTAG00000001548	3:14767914-14781668	0.485921	0.111561	0.892487	OK
ENSBTAG00000048056	ENSBTAG00000048056	7:9576529-9577516	0.485555	0	1.2587	OK
ENSBTAG00000038498	ENSBTAG00000038498	5:56764458-56766582	0.485359	0.0974844	0.779876	OK
ENSBTAG00000017462	ENSBTAG00000017462	5:111462844-111464936	0.485202	0	0.880469	OK
ENSBTAG00000006725	ENSBTAG00000006725	13:64603585-64604291	0.485168	0	0.895059	OK
ENSBTAG00000016265	ENSBTAG00000016265	8:76107823-76118491	0.484864	0	1.29297	OK
ENSBTAG00000004611	ENSBTAG00000004611	18:57537669-57542716	0.484086	0	1.01201	OK
ENSBTAG00000008365	ENSBTAG00000008365	3:21519978-21521463	0.483892	0	0.836594	OK
ENSBTAG00000037927	ENSBTAG00000037927	4:108125472-108126405	0.483833	0	1.33156	OK
ENSBTAG00000008652	ENSBTAG00000008652	3:1656684-1723930	0.483692	0.149356	0.796564	OK
ENSBTAG00000048109	ENSBTAG00000048109	10:27071791-27072736	0.483508	0	1.09554	OK
ENSBTAG00000021113	ENSBTAG00000021113	19:34483123-34489156	0.483407	0	0.965622	OK
ENSBTAG00000012940	ENSBTAG00000012940	15:29725505-29729852	0.483122	0.0811352	0.811352	OK
ENSBTAG00000008374	ENSBTAG00000008374	5:30097667-30103142	0.482478	0	0.886121	OK
ENSBTAG00000047391	ENSBTAG00000047391	12:57877140-57877506	0.482338	0	1.69719	OK
ENSBTAG00000018050	ENSBTAG00000018050	25:20218557-20247294	0.482115	0	1.08476	OK
ENSBTAG00000000973	ENSBTAG00000000973	1:152301467-152306134	0.481883	0.10602	0.84816	OK
ENSBTAG00000015463	ENSBTAG00000015463	27:28415996-28427209	0.481849	0	1.28493	OK
ENSBTAG00000017116	ENSBTAG00000017116	5:74106328-74117622	0.481813	0.0670304	0.804365	OK
ENSBTAG00000008544	ENSBTAG00000008544	18:19640446-19654562	0.481623	0.157219	0.786093	OK
ENSBTAG00000008074	ENSBTAG00000008074	5:75992621-75999893	0.481556	0.0765743	0.842318	OK

ENSBTAG00000023078	ENSBTAG00000023078	8:71147229-71148267	0.481467	0	0.809607	OK
ENSBTAG00000010465	ENSBTAG00000010465	29:41690245-41693633	0.48144	0	1.05966	OK
ENSBTAG00000010681	ENSBTAG00000010681	15:78323083-78330233	0.481268	0	0.864796	OK
ENSBTAG00000002107	ENSBTAG00000002107	4:8490675-8494829	0.480949	0.0996904	0.797523	OK
ENSBTAG00000030933	ENSBTAG00000030933	18:52156626-52159132	0.480331	0	0.920253	OK
ENSBTAG00000018198	ENSBTAG00000018198	22:49229609-49233939	0.480163	0.063612	0.763344	OK
ENSBTAG00000011894	ENSBTAG00000011894	22:51375766-51379188	0.480001	0	0.954913	OK
ENSBTAG00000018088	ENSBTAG00000018088	24:45025455-45141793	0.47974	0.194831	0.730617	OK
ENSBTAG00000009796	ENSBTAG00000009796	2:126983129-126992474	0.479202	0.10027	0.802158	OK
ENSBTAG00000040439	ENSBTAG00000040439	20:15558580-15559849	0.479173	0	0.815827	OK
ENSBTAG00000010475	ENSBTAG00000010475	12:18953336-18955344	0.479152	0	0.854195	OK
ENSBTAG00000045924	ENSBTAG00000045924	X:121978462-121979509	0.479024	0	1.18657	OK
ENSBTAG00000040051	ENSBTAG00000040051	15:32148574-32149038	0.478712	0	1.33873	OK
ENSBTAG00000006273	ENSBTAG00000006273	5:115084015-115117266	0.4786	0	0.824563	OK
ENSBTAG00000044073	ENSBTAG00000044073	29:45016283-45018614	0.478309	0	0.815185	OK
ENSBTAG00000013249	ENSBTAG00000013249	10:25704581-25709754	0.477938	0.111381	0.891047	OK
ENSBTAG00000030939	ENSBTAG00000030939	18:52064956-52074914	0.477759	0	2.38879	OK
ENSBTAG00000044829	ENSBTAG00000044829	10:70394036-70394330	0.477538	0	2.11283	OK
ENSBTAG00000009614	ENSBTAG00000009614	1:71765080-71791874	0.477536	0.0846859	0.846859	OK
ENSBTAG00000010515	ENSBTAG00000010515	11:106287253-106291495	0.47745	0	0.632925	OK
ENSBTAG00000000232	ENSBTAG00000000232	15:51023571-51024546	0.477223	0	1.06183	OK
ENSBTAG00000047213	ENSBTAG00000047213	X:50499827-50502731	0.476521	0.0734245	0.881093	OK
ENSBTAG00000000703	ENSBTAG00000000703	11:45414101-45469589	0.476321	0.0778702	0.856573	OK
ENSBTAG00000009753	ENSBTAG00000009753	X:25009015-25009992	0.475911	0	0.928507	OK
ENSBTAG00000014669	ENSBTAG00000014669	5:24846224-24873123	0.475564	0	0.978222	OK
ENSBTAG00000038111	ENSBTAG00000038111	1:127128894-127129613	0.475506	0	0.904179	OK
ENSBTAG00000048176	ENSBTAG00000048176	15:45378868-45379801	0.475122	0	1.33156	OK
ENSBTAG00000001565	ENSBTAG00000001565	21:27322044-27330423	0.475054	0	0.885399	OK
ENSBTAG00000025250	ENSBTAG00000025250	19:14673889-14675910	0.474836	0	0.718948	OK
ENSBTAG00000013983	ENSBTAG00000013983	10:86714252-86722247	0.474689	0	1.03983	OK

ENSBTAG00000045500	ENSBTAG00000045500	19:23888013-23924563	0.474597	0.0598431	0.718117	OK
ENSBTAG00000003877	ENSBTAG00000003877	28:35189621-35251005	0.474526	0	0.885616	OK
ENSBTAG00000017855	ENSBTAG00000017855	2:119476495-119490380	0.474363	0	0.904179	OK
ENSBTAG00000012485	ENSBTAG00000012485	11:99318414-99327141	0.474305	0	0.907349	OK
ENSBTAG00000012332	ENSBTAG00000012332	11:9240934-9251371	0.474201	0.119272	0.834907	OK
ENSBTAG00000007145	ENSBTAG00000007145	5:27659716-27672880	0.473659	0.0805669	0.805669	OK
ENSBTAG00000003885	ENSBTAG00000003885	14:42315040-42330314	0.473042	0	0.849755	OK
ENSBTAG00000003578	ENSBTAG00000003578	26:22205906-22227992	0.473023	0	0.726516	OK
ENSBTAG00000047984	ENSBTAG00000047984	10:27641547-27642453	0.472947	0	1.1427	OK
ENSBTAG00000037625	ENSBTAG00000037625	10:78463268-78464048	0.472909	0	1.32729	OK
ENSBTAG00000020305	ENSBTAG00000020305	11:100219225-100227150	0.472803	0	0.87798	OK
ENSBTAG00000014406	ENSBTAG00000014406	12:35919545-35942309	0.472755	0	0.537035	OK
ENSBTAG00000000861	ENSBTAG00000000861	3:13778326-13784728	0.472362	0	0.769014	OK
ENSBTAG00000005419	ENSBTAG00000005419	4:114249781-114262317	0.47187	0.0669437	0.870268	OK
ENSBTAG00000038093	ENSBTAG00000038093	4:11912008-11921418	0.471543	0.0646851	0.840906	OK
ENSBTAG00000040360	ENSBTAG00000040360	14:2253176-2254950	0.471491	0	0.862224	OK
ENSBTAG00000011843	ENSBTAG00000011843	29:41614570-41624167	0.471387	0	0.874273	OK
ENSBTAG00000045884	ENSBTAG00000045884	23:42467106-42467856	0.471387	0	0.828228	OK
ENSBTAG00000019198	ENSBTAG00000019198	3:92809553-92830529	0.471191	0	0.906441	OK
ENSBTAG00000002542	ENSBTAG00000002542	11:47874691-47879023	0.470805	0	1.16324	OK
ENSBTAG00000019627	ENSBTAG00000019627	15:30509503-30515399	0.4703	0.107618	0.860944	OK
ENSBTAG00000021512	ENSBTAG00000021512	3:15634914-15648928	0.469662	0	2.34831	OK
ENSBTAG00000017438	ENSBTAG00000017438	17:71755942-71760583	0.469534	0	0.797395	OK
ENSBTAG00000047966	ENSBTAG00000047966	16:6155902-6156793	0.469502	0	0.929549	OK
ENSBTAG00000038161	ENSBTAG00000038161	15:49771680-49772631	0.469474	0	1.30635	OK
ENSBTAG00000012748	ENSBTAG00000012748	X:34800751-34801780	0.469082	0	1.00611	OK
ENSBTAG00000011103	ENSBTAG00000011103	17:63561391-63591370	0.469041	0.0627636	0.753163	OK
ENSBTAG00000006795	ENSBTAG00000006795	18:7793105-7805806	0.468765	0	1.15138	OK
ENSBTAG00000012439	ENSBTAG00000012439	23:15910248-15921069	0.468743	0	1.6406	OK
ENSBTAG00000019679	ENSBTAG00000019679	18:46864523-46869490	0.468601	0	1.66476	OK

ENSBTAG00000011864	ENSBTAG00000011864	7:100425876-100446422	0.468599	0	0.825586	OK
ENSBTAG00000040093	ENSBTAG00000040093	11:47479961-47484355	0.468344	0	0.823616	OK
ENSBTAG00000007499	ENSBTAG00000007499	X:55234947-55241418	0.46816	0.0880344	0.880344	OK
ENSBTAG00000014387	ENSBTAG00000014387	3:22635093-22646401	0.467924	0	1.22374	OK
ENSBTAG00000005070	ENSBTAG00000005070	15:51796619-51797639	0.467471	0	1.01499	OK
ENSBTAG00000016716	ENSBTAG00000016716	14:32699359-32701121	0.467201	0	0.822587	OK
ENSBTAG00000010668	ENSBTAG00000010668	18:53160658-53167519	0.467164	0	0.88993	OK
ENSBTAG00000012989	ENSBTAG00000012989	15:82124875-82138170	0.467117	0	0.806454	OK
ENSBTAG00000032089	ENSBTAG00000032089	29:44189277-44195090	0.46709	0	0.851502	OK
ENSBTAG00000048021	ENSBTAG00000048021	18:54129779-54131732	0.466947	0	0.863766	OK
ENSBTAG00000040547	ENSBTAG00000040547	2:133067840-133068686	0.46672	0	0.734245	OK
ENSBTAG00000009943	ENSBTAG00000009943	16:36963320-36966885	0.466632	0	1.11923	OK
ENSBTAG00000045529	ENSBTAG00000045529	18:62860198-62864521	0.466574	0	0.952424	OK
ENSBTAG00000038132	ENSBTAG00000038132	17:74307985-74318794	0.464841	0	0.674767	OK
ENSBTAG00000002900	ENSBTAG00000002900	10:99351221-99352544	0.464635	0	0.782528	OK
ENSBTAG00000006538	ENSBTAG00000006538	5:82246521-82258858	0.464411	0	2.32205	OK
ENSBTAG00000019709	ENSBTAG00000019709	3:19584960-19585461	0.46424	0	1.32446	OK
ENSBTAG00000006349	ENSBTAG00000006349	2:127647570-127665188	0.464192	0.0581784	0.814498	OK
ENSBTAG00000047176	ENSBTAG00000047176	15:47438973-47440304	0.464183	0	1.29816	OK
ENSBTAG00000000368	ENSBTAG00000000368	15:47577989-47578946	0.464134	0	1.29816	OK
ENSBTAG00000021284	ENSBTAG00000021284	1:144320105-144333299	0.46369	0	0.843409	OK
ENSBTAG00000013253	ENSBTAG00000013253	3:121118652-121153701	0.463675	0	2.44471	OK
ENSBTAG00000009861	ENSBTAG00000009861	23:15592218-15601796	0.463487	0	0.883181	OK
ENSBTAG00000046902	ENSBTAG00000046902	17:24418841-24419954	0.463423	0	0.930175	OK
ENSBTAG00000018600	ENSBTAG00000018600	22:56220083-56290873	0.46258	0	0.794844	OK
ENSBTAG00000018063	ENSBTAG00000018063	13:78712287-78722796	0.462319	0.107034	0.802754	OK
ENSBTAG00000020426	ENSBTAG00000020426	23:23097250-23122686	0.461565	0	0.856113	OK
ENSBTAG00000004542	ENSBTAG00000004542	11:101477075-101489330	0.461486	0	1.58638	OK
ENSBTAG00000030704	ENSBTAG00000030704	7:41214993-41216392	0.461151	0	0.908809	OK
ENSBTAG00000038204	ENSBTAG00000038204	X:55346313-55347499	0.461112	0	1.01499	OK

ENSBTAG00000003865	ENSBTAG00000003865	1:67255523-67344655	0.46107	0.197272	0.71018	OK
ENSBTAG00000048313	ENSBTAG00000048313	9:71468911-71469958	0.460836	0	0.988811	OK
ENSBTAG00000019469	ENSBTAG00000019469	9:62754533-62784964	0.460753	0	1.03736	OK
ENSBTAG00000014738	ENSBTAG00000014738	4:69311986-69314613	0.460133	0	0.854901	OK
ENSBTAG00000040295	ENSBTAG00000040295	25:28016898-28022616	0.458481	0	0.757525	OK
ENSBTAG00000009661	ENSBTAG00000009661	7:13837388-13844314	0.458304	0	0.885941	OK
ENSBTAG00000030834	ENSBTAG00000030834	11:77669173-77669687	0.458131	0	1.2085	OK
ENSBTAG00000008533	ENSBTAG00000008533	4:17067021-17141147	0.458022	0	0.873658	OK
ENSBTAG00000001104	ENSBTAG00000001104	18:34476951-34556068	0.457713	0.0792108	0.792108	OK
ENSBTAG00000008786	ENSBTAG00000008786	18:26593474-26593959	0.457675	0	1.28076	OK
ENSBTAG00000023967	ENSBTAG00000023967	26:11056271-11057005	0.457536	0	0.846282	OK
ENSBTAG00000045996	ENSBTAG00000045996	29:8121686-8122321	0.456666	0	0.978222	OK
ENSBTAG00000016551	ENSBTAG00000016551	29:43152059-43154681	0.45654	0	2.2827	OK
ENSBTAG00000000356	ENSBTAG00000000356	19:51757840-51763491	0.456448	0	0.911253	OK
ENSBTAG00000003088	ENSBTAG00000003088	X:111214350-111325716	0.456026	0	0.819703	OK
ENSBTAG00000003209	ENSBTAG00000003209	13:67114712-67125217	0.456004	0.102758	0.822062	OK
ENSBTAG00000047644	ENSBTAG00000047644	23:29018638-29019726	0.455743	0	1.33156	OK
ENSBTAG00000033954	ENSBTAG00000033954	4:26540376-26541499	0.455498	0	0.921892	OK
ENSBTAG00000021276	ENSBTAG00000021276	1:144067290-144071071	0.455246	0	0.753848	OK
ENSBTAG00000038169	ENSBTAG00000038169	29:5845678-5851558	0.454603	0	0.922303	OK
ENSBTAG00000014836	ENSBTAG00000014836	11:25334291-25335328	0.454249	0	1.23863	OK
ENSBTAG00000009942	ENSBTAG00000009942	1:155618261-155833445	0.453811	0.16104	0.724679	OK
ENSBTAG00000015097	ENSBTAG00000015097	11:104408231-104417213	0.45298	0	0.714341	OK
ENSBTAG00000013956	ENSBTAG00000013956	5:109584776-109635907	0.452909	0	0.803931	OK
ENSBTAG00000032265	ENSBTAG00000032265	1:80502600-80506904	0.452311	0	0.839258	OK
ENSBTAG00000011758	ENSBTAG00000011758	5:104740722-104741272	0.452152	0	1.1294	OK
ENSBTAG00000019129	ENSBTAG00000019129	1:154375850-154416952	0.451667	0	1.355	OK
ENSBTAG00000013641	ENSBTAG00000013641	26:40118555-40141989	0.451379	0.0836256	0.75263	OK
ENSBTAG00000006486	ENSBTAG00000006486	18:63393522-63397442	0.451264	0	0.874889	OK
ENSBTAG00000018506	ENSBTAG00000018506	5:58013469-58014035	0.451222	0	1.09748	OK

ENSBTAG00000003312	ENSBTAG00000003312	26:44047736-44081511	0.450791	0.0984579	0.787663	OK
ENSBTAG00000002879	ENSBTAG00000002879	29:30023033-30065785	0.45068	0.127813	0.766878	OK
ENSBTAG00000002768	ENSBTAG00000002768	X:92098161-92105518	0.450531	0	1.20142	OK
ENSBTAG00000045715	ENSBTAG00000045715	X:8120114-8121548	0.450513	0	1.15513	OK
ENSBTAG00000006525	ENSBTAG00000006525	19:57164030-57175524	0.450217	0	2.25109	OK
ENSBTAG00000023963	ENSBTAG00000023963	2:115826527-115960780	0.449991	0.0829768	0.888255	OK
ENSBTAG00000037436	ENSBTAG00000037436	5:99748858-99749792	0.44977	0	1.12653	OK
ENSBTAG00000021949	ENSBTAG00000021949	8:73180099-73184369	0.44962	0.0950238	0.760191	OK
ENSBTAG00000003971	ENSBTAG00000003971	13:63705709-63714008	0.448668	0	0.784307	OK
ENSBTAG00000016954	ENSBTAG00000016954	2:122194338-122213700	0.448638	0	0.846611	OK
ENSBTAG00000011327	ENSBTAG00000011327	3:29506171-29509176	0.448563	0	0.82026	OK
ENSBTAG00000004200	ENSBTAG00000004200	7:5709941-5713187	0.448507	0	0.759643	OK
ENSBTAG00000019814	ENSBTAG00000019814	7:44065139-44066921	0.448148	0	0.813355	OK
ENSBTAG00000013921	ENSBTAG00000013921	18:53383533-53392948	0.447955	0	0.79231	OK
ENSBTAG00000001517	ENSBTAG00000001517	5:27171924-27175575	0.447715	0	0.846859	OK
ENSBTAG00000004421	ENSBTAG00000004421	10:36191060-36193015	0.447692	0	0.847292	OK
ENSBTAG00000014670	ENSBTAG00000014670	X:55856790-55859078	0.447663	0	1.01168	OK
ENSBTAG00000030725	ENSBTAG00000030725	7:43101692-43102628	0.44759	0	1.32729	OK
ENSBTAG00000007946	ENSBTAG00000007946	19:20964784-20978420	0.447445	0.0761519	0.761519	OK
ENSBTAG00000045925	ENSBTAG00000045925	X:83495861-83501893	0.446961	0	2.23481	OK
ENSBTAG00000021445	ENSBTAG00000021445	7:102456174-102555855	0.44673	0	1.19917	OK
ENSBTAG00000046035	ENSBTAG00000046035	X:32898971-32900393	0.446304	0	0.977606	OK
ENSBTAG00000024086	ENSBTAG00000024086	3:111277767-111280979	0.446265	0	1.27971	OK
ENSBTAG00000017112	ENSBTAG00000017112	18:7421961-7474074	0.446233	0	0.829888	OK
ENSBTAG00000040482	ENSBTAG00000040482	7:14969549-14970491	0.446215	0	1.31883	OK
ENSBTAG00000011579	ENSBTAG00000011579	29:41919873-41921707	0.445332	0	0.746959	OK
ENSBTAG00000025109	ENSBTAG00000025109	7:71621005-71621548	0.444842	0	1.14396	OK
ENSBTAG00000006026	ENSBTAG00000006026	X:57269279-57270782	0.44483	0	0.807764	OK
ENSBTAG00000046470	ENSBTAG00000046470	10:57712107-57744837	0.444796	0	0.815644	OK
ENSBTAG00000004241	ENSBTAG00000004241	2:27274584-27281293	0.444701	0	1.16324	OK

ENSBTAG00000004110	ENSBTAG00000004110	X:84590184-84590760	0.444619	0	1.07842	OK
ENSBTAG00000034871	ENSBTAG00000034871	22:7523815-7526444	0.444393	0	1.01399	OK
ENSBTAG00000003483	ENSBTAG00000003483	8:69922730-69925053	0.444304	0	0.776221	OK
ENSBTAG00000038407	ENSBTAG00000038407	5:100719637-100720427	0.444237	0	0.786292	OK
ENSBTAG00000018903	ENSBTAG00000018903	18:35327417-35333286	0.444198	0	1.33259	OK
ENSBTAG00000008351	ENSBTAG00000008351	5:95516923-95518003	0.444136	0	2.31026	OK
ENSBTAG00000012555	ENSBTAG00000012555	3:33200770-33203515	0.443941	0.0754306	0.754306	OK
ENSBTAG00000016829	ENSBTAG00000016829	23:7426486-7428391	0.44394	0	0.869531	OK
ENSBTAG00000009099	ENSBTAG00000009099	25:27034799-27038835	0.443401	0	0.864087	OK
ENSBTAG00000021462	ENSBTAG00000021462	19:40981689-40983998	0.443359	0	0.862737	OK
ENSBTAG00000009895	ENSBTAG00000009895	18:45731749-45746037	0.443122	0.0974386	0.779509	OK
ENSBTAG00000021434	ENSBTAG00000021434	3:105299334-105305259	0.442833	0	0.828891	OK
ENSBTAG00000014390	ENSBTAG00000014390	2:122121402-122128496	0.442424	0	0.780926	OK
ENSBTAG00000020717	ENSBTAG00000020717	X:103969314-104013030	0.442343	0.0796986	0.796986	OK
ENSBTAG00000003502	ENSBTAG00000003502	7:49961404-49995991	0.44206	0	1.02301	OK
ENSBTAG00000044171	ENSBTAG00000044171	16:28441399-28580230	0.441818	0	0.721453	OK
ENSBTAG00000009818	ENSBTAG00000009818	13:74446433-74446924	0.441383	0	1.26511	OK
ENSBTAG00000018084	ENSBTAG00000018084	17:71452536-71457053	0.44134	0	0.800478	OK
ENSBTAG00000014149	ENSBTAG00000014149	11:98793762-98797809	0.441281	0	0.767826	OK
ENSBTAG00000019212	ENSBTAG00000019212	11:80842560-80844195	0.441058	0	0.759842	OK
ENSBTAG00000009285	ENSBTAG00000009285	7:41551824-41552772	0.440811	0	1.09207	OK
ENSBTAG00000003177	ENSBTAG00000003177	16:44801769-44831904	0.440638	0	0.858564	OK
ENSBTAG00000037962	ENSBTAG00000037962	15:14426512-14556383	0.440493	0.144391	0.794152	OK
ENSBTAG00000018402	ENSBTAG00000018402	1:118612163-118657811	0.440415	0.0936062	0.74885	OK
ENSBTAG00000038395	ENSBTAG00000038395	1:109550309-109551293	0.440264	0	0.841695	OK
ENSBTAG00000005267	ENSBTAG00000005267	26:50573880-50574822	0.440231	0	0.879223	OK
ENSBTAG00000001873	ENSBTAG00000001873	3:56177925-56180722	0.440041	0	0.788788	OK
ENSBTAG00000019070	ENSBTAG00000019070	19:33357140-33382707	0.440006	0	0.805669	OK
ENSBTAG00000009405	ENSBTAG00000009405	12:24049604-24194475	0.439996	0	0.879991	OK
ENSBTAG00000016315	ENSBTAG00000016315	18:10781075-10825733	0.439743	0	0.827755	OK

ENSBTAG00000031497	ENSBTAG00000031497	6:115940218-115943190	0.439498	0	0.920253	OK
ENSBTAG00000007007	ENSBTAG00000007007	21:68639657-68696036	0.439487	0.087885	0.790965	OK
ENSBTAG00000030744	ENSBTAG00000030744	5:68109724-68127878	0.438985	0	1.04928	OK
ENSBTAG00000004915	ENSBTAG00000004915	19:27081318-27084643	0.438878	0	0.762173	OK
ENSBTAG00000046129	ENSBTAG00000046129	19:20698683-20702070	0.438552	0	0.840329	OK
ENSBTAG00000031891	ENSBTAG00000031891	1:93626218-93632229	0.438494	0	1.13332	OK
ENSBTAG00000020385	ENSBTAG00000020385	8:62891040-62892603	0.438395	0	0.794844	OK
ENSBTAG00000033143	ENSBTAG00000033143	2:43529728-43530624	0.437851	0	0.705876	OK
ENSBTAG00000020366	ENSBTAG00000020366	25:1529746-1535805	0.43773	0	0.57699	OK
ENSBTAG00000027326	ENSBTAG00000027326	13:76083308-76087372	0.437547	0	0.75075	OK
ENSBTAG00000000647	ENSBTAG00000000647	5:119771355-119791795	0.437428	0	0.800478	OK
ENSBTAG00000005670	ENSBTAG00000005670	2:136480736-136491543	0.437201	0	0.651578	OK
ENSBTAG00000000470	ENSBTAG00000000470	6:38309554-38323305	0.437106	0	1.00028	OK
ENSBTAG00000000040	ENSBTAG00000000040	19:51576539-51582086	0.437052	0	0.842184	OK
ENSBTAG00000006879	ENSBTAG00000006879	5:26215736-26218209	0.436091	0	0.863457	OK
ENSBTAG00000037457	ENSBTAG00000037457	24:54044362-54093578	0.435739	0	1.22519	OK
ENSBTAG00000015192	ENSBTAG00000015192	7:40378359-40409143	0.435656	0	1.30697	OK
ENSBTAG00000033477	ENSBTAG00000033477	29:28329962-28330937	0.435568	0	1.06183	OK
ENSBTAG00000011019	ENSBTAG00000011019	29:42478122-42495303	0.435559	0	0.859157	OK
ENSBTAG00000038148	ENSBTAG00000038148	6:93486377-93488711	0.435382	0	0.79842	OK
ENSBTAG00000033248	ENSBTAG00000033248	18:36095733-36140923	0.435137	0.0664709	0.797651	OK
ENSBTAG00000012212	ENSBTAG00000012212	11:12371179-12387041	0.434914	0	0.802028	OK
ENSBTAG00000026929	ENSBTAG00000026929	15:48116742-48117711	0.434863	0	1.06841	OK
ENSBTAG00000014083	ENSBTAG00000014083	3:34054794-34060289	0.434656	0.206355	0.701608	OK
ENSBTAG00000037907	ENSBTAG00000037907	24:9741023-9744662	0.434424	0.170698	0.739692	OK
ENSBTAG00000011328	ENSBTAG00000011328	11:72521481-72524704	0.434013	0	0.843982	OK
ENSBTAG00000008193	ENSBTAG00000008193	15:40918450-40981652	0.433808	0	0.867614	OK
ENSBTAG00000037398	ENSBTAG00000037398	15:49603715-49604723	0.433759	0	1.23248	OK
ENSBTAG00000000541	ENSBTAG00000000541	21:47130287-47134065	0.433549	0.0727537	0.727537	OK
ENSBTAG00000045998	ENSBTAG00000045998	21:781267-784819	0.433415	0.116586	0.699517	OK

ENSBTAG00000030792	ENSBTAG00000030792	10:74517548-74530270	0.433339	0	0.958597	OK
ENSBTAG00000039695	ENSBTAG00000039695	23:28128193-28137021	0.433227	0.0694123	0.763536	OK
ENSBTAG00000008766	ENSBTAG00000008766	2:126676549-126727482	0.433163	0.111591	0.725341	OK
ENSBTAG00000001315	ENSBTAG00000001315	8:21763505-21768460	0.432867	0	0.818869	OK
ENSBTAG00000031647	ENSBTAG00000031647	7:20812033-20814992	0.432519	0	0.855607	OK
ENSBTAG00000001734	ENSBTAG00000001734	29:37718325-37725179	0.432504	0	0.804625	OK
ENSBTAG00000046333	ENSBTAG00000046333	6:26345280-26350653	0.43238	0.0770731	0.616585	OK
ENSBTAG00000031146	ENSBTAG00000031146	5:57703546-57724145	0.432101	0	0.950675	OK
ENSBTAG00000006634	ENSBTAG00000006634	29:26844006-26844945	0.430808	0	1.10254	OK
ENSBTAG00000024884	ENSBTAG00000024884	25:1968198-1972458	0.430796	0	0.846117	OK
ENSBTAG00000045612	ENSBTAG00000045612	15:49788913-49789867	0.430689	0	1.30225	OK
ENSBTAG00000008343	ENSBTAG00000008343	1:83316636-83318468	0.430632	0	0.791156	OK
ENSBTAG00000019124	ENSBTAG00000019124	28:26764784-26786028	0.430433	0	1.04928	OK
ENSBTAG00000047471	ENSBTAG00000047471	18:36364155-36365385	0.430204	0	0.920253	OK
ENSBTAG00000045911	ENSBTAG00000045911	3:119737524-119738454	0.430123	0	1.11321	OK
ENSBTAG00000010559	ENSBTAG00000010559	19:35768849-35815316	0.430059	0	0.836932	OK
ENSBTAG00000039691	ENSBTAG00000039691	18:63313173-63319123	0.430057	0	0.687136	OK
ENSBTAG00000021914	ENSBTAG00000021914	19:51700264-51700886	0.430016	0	1.01665	OK
ENSBTAG00000046332	ENSBTAG00000046332	28:419153-421910	0.429928	0	2.14964	OK
ENSBTAG00000001987	ENSBTAG00000001987	15:43509669-43585976	0.429914	0.14014	0.77077	OK
ENSBTAG00000048014	ENSBTAG00000048014	17:56084029-56084726	0.429878	0	0.922988	OK
ENSBTAG00000035265	ENSBTAG00000035265	17:159888-179634	0.429817	0	1.12409	OK
ENSBTAG00000003298	ENSBTAG00000003298	26:21703369-21710503	0.429738	0	0.668645	OK
ENSBTAG00000036242	ENSBTAG00000036242	7:97470513-97473035	0.429482	0	1.30225	OK
ENSBTAG00000014583	ENSBTAG00000014583	18:54169312-54178928	0.429309	0	0.798292	OK
ENSBTAG00000006367	ENSBTAG00000006367	9:70873220-70876455	0.428893	0.0898685	0.718948	OK
ENSBTAG00000020609	ENSBTAG00000020609	28:39619129-39619833	0.428687	0	0.882345	OK
ENSBTAG00000015253	ENSBTAG00000015253	25:42316374-42321964	0.428673	0	0.706679	OK
ENSBTAG00000020454	ENSBTAG00000020454	13:52494960-52500593	0.428175	0.16688	0.667522	OK
ENSBTAG00000010522	ENSBTAG00000010522	26:25714850-25734311	0.42805	0	0.457079	OK

ENSBTAG00000017068	ENSBTAG00000017068	7:5729333-5739114	0.42781	0	0.820028	OK
ENSBTAG00000032711	ENSBTAG00000032711	12:61535619-61537911	0.427547	0	0.91295	OK
ENSBTAG00000007241	ENSBTAG00000007241	3:9848245-9851355	0.427035	0	0.997384	OK
ENSBTAG00000012079	ENSBTAG00000012079	23:50735296-50800744	0.426966	0	0.774045	OK
ENSBTAG00000046080	ENSBTAG00000046080	7:80188340-80188849	0.426889	0	1.22037	OK
ENSBTAG00000039050	ENSBTAG00000039050	15:53492849-53499415	0.426888	0	0.766168	OK
ENSBTAG00000000564	ENSBTAG00000000564	20:5656109-5727730	0.426647	0.088942	0.711536	OK
ENSBTAG00000026922	ENSBTAG00000026922	15:48225176-48226235	0.426532	0	1.20733	OK
ENSBTAG00000045791	ENSBTAG00000045791	14:2266061-2267715	0.426409	0	0.808816	OK
ENSBTAG00000005626	ENSBTAG00000005626	5:60225837-60226830	0.42608	0	1.04258	OK
ENSBTAG00000039839	ENSBTAG00000039839	11:46621378-46625624	0.426058	0	0.805147	OK
ENSBTAG00000045537	ENSBTAG00000045537	X:118804870-118805932	0.42587	0	1.01798	OK
ENSBTAG00000043561	ENSBTAG00000043561	MT:5686-7231	0.425235	0	0.95678	OK
ENSBTAG00000022632	ENSBTAG00000022632	22:51254438-51257322	0.425029	0	0.794336	OK
ENSBTAG00000004747	ENSBTAG00000004747	22:48710804-48712454	0.424975	0	1.28767	OK
ENSBTAG00000046223	ENSBTAG00000046223	11:105907057-105913137	0.424878	0	0.752934	OK
ENSBTAG00000040329	ENSBTAG00000040329	2:130245901-130247035	0.424592	0	0.91295	OK
ENSBTAG00000016294	ENSBTAG00000016294	13:34524931-34807031	0.424543	0.0675333	0.742866	OK
ENSBTAG00000008350	ENSBTAG00000008350	16:47645899-47659012	0.4245	0	0.598431	OK
ENSBTAG00000047612	ENSBTAG00000047612	7:17264371-17267661	0.424338	0	0.990703	OK
ENSBTAG00000020364	ENSBTAG00000020364	4:108377643-108378588	0.424075	0	1.09554	OK
ENSBTAG00000002157	ENSBTAG00000002157	4:88746218-88755270	0.42386	0.0997384	0.797907	OK
ENSBTAG00000002058	ENSBTAG00000002058	9:32549920-32562773	0.423527	0	1.01698	OK
ENSBTAG00000021709	ENSBTAG00000021709	15:78402904-78419772	0.423434	0	0.743917	OK
ENSBTAG00000026924	ENSBTAG00000026924	15:48164655-48165627	0.42337	0	1.06511	OK
ENSBTAG00000040358	ENSBTAG00000040358	18:64856653-64891741	0.423296	0	1.65731	OK
ENSBTAG00000007444	ENSBTAG00000007444	1:6366368-6414544	0.423239	0	1.12838	OK
ENSBTAG00000018530	ENSBTAG00000018530	5:109876441-109894993	0.423055	0	0.821655	OK
ENSBTAG00000040419	ENSBTAG00000040419	16:56022843-56025833	0.422981	0	0.841695	OK
ENSBTAG00000017649	ENSBTAG00000017649	X:132976589-132992889	0.4229	0	1.04839	OK

ENSBTAG00000035659	ENSBTAG00000035659	16:297299-298235	0.422794	0	1.32729	OK
ENSBTAG00000037623	ENSBTAG00000037623	29:27770677-27771721	0.422647	0	1.31883	OK
ENSBTAG00000016768	ENSBTAG00000016768	15:34769328-34791462	0.422645	0	0.667208	OK
ENSBTAG00000031084	ENSBTAG00000031084	19:33524874-33548224	0.422616	0	0.759378	OK
ENSBTAG00000000321	ENSBTAG00000000321	25:34151409-34156679	0.422547	0.105052	0.840414	OK
ENSBTAG00000020051	ENSBTAG00000020051	25:24935875-24937932	0.422384	0.10066	0.805277	OK
ENSBTAG00000002849	ENSBTAG00000002849	16:75401220-75417466	0.422034	0	1.04084	OK
ENSBTAG00000030962	ENSBTAG00000030962	22:60063339-60172924	0.42202	0	0.729808	OK
ENSBTAG00000011106	ENSBTAG00000011106	9:99649025-100180707	0.421798	0	2.10899	OK
ENSBTAG00000023247	ENSBTAG00000023247	3:11412356-11413325	0.421642	0	1.28209	OK
ENSBTAG00000014788	ENSBTAG00000014788	9:89558648-89563595	0.421456	0.125565	0.711536	OK
ENSBTAG00000046472	ENSBTAG00000046472	18:58529772-58530702	0.421049	0	1.11321	OK
ENSBTAG00000025335	ENSBTAG00000025335	11:73529505-73529964	0.420874	0	1.35331	OK
ENSBTAG00000013306	ENSBTAG00000013306	24:48164178-48177831	0.420699	0	0.752543	OK
ENSBTAG00000003398	ENSBTAG00000003398	13:79646520-79651314	0.420663	0	0.804105	OK
ENSBTAG00000037985	ENSBTAG00000037985	10:28577030-28578629	0.420473	0	0.776949	OK
ENSBTAG00000037650	ENSBTAG00000037650	X:63334009-63338678	0.420321	0.15281	0.713111	OK
ENSBTAG00000038643	ENSBTAG00000038643	10:26880574-26881549	0.42031	0	1.2742	OK
ENSBTAG00000006384	ENSBTAG00000006384	X:126010549-126012970	0.420288	0.0894028	0.715223	OK
ENSBTAG00000011234	ENSBTAG00000011234	9:42008605-42118673	0.420112	0	0.820434	OK
ENSBTAG00000013309	ENSBTAG00000013309	2:124956593-124982238	0.419976	0.0912547	0.730038	OK
ENSBTAG00000006581	ENSBTAG00000006581	15:14120304-14151878	0.419972	0	0.877773	OK
ENSBTAG00000031320	ENSBTAG00000031320	X:121951934-121952981	0.419564	0	0.988811	OK
ENSBTAG00000025809	ENSBTAG00000025809	7:5744756-5751713	0.419401	0	0.759245	OK
ENSBTAG00000017373	ENSBTAG00000017373	10:46962110-46979997	0.419176	0.10286	0.822879	OK
ENSBTAG00000025494	ENSBTAG00000025494	23:24528154-24571511	0.419175	0.166444	0.721259	OK
ENSBTAG00000039630	ENSBTAG00000039630	17:54330420-54338333	0.418926	0	1.02503	OK
ENSBTAG00000012010	ENSBTAG00000012010	29:590731-644582	0.418511	0	0.974386	OK
ENSBTAG00000004379	ENSBTAG00000004379	18:52048412-52065702	0.418441	0	1.07172	OK
ENSBTAG00000002101	ENSBTAG00000002101	25:17210000-17237789	0.418209	0	0.804625	OK

ENSBTAG00000002478	ENSBTAG00000002478	16:53199432-53208249	0.418157	0	0.688469	OK
ENSBTAG00000009086	ENSBTAG00000009086	21:35057677-35082119	0.417664	0	0.597444	OK
ENSBTAG00000004647	ENSBTAG00000004647	17:17467449-17473822	0.41749	0	0.813264	OK
ENSBTAG00000009353	ENSBTAG00000009353	19:19027997-19036601	0.41747	0.11043	1.43559	OK
ENSBTAG00000016896	ENSBTAG00000016896	18:25048852-25059249	0.417276	0	0.76236	OK
ENSBTAG00000003966	ENSBTAG00000003966	19:44281112-44294691	0.417042	0.0595774	0.833583	OK
ENSBTAG00000004936	ENSBTAG00000004936	7:20992766-21001767	0.41701	0	0.819216	OK
ENSBTAG00000026666	ENSBTAG00000026666	5:72077085-72082472	0.417006	0	0.797395	OK
ENSBTAG00000047541	ENSBTAG00000047541	3:11293910-11294927	0.416926	0	1.26254	OK
ENSBTAG00000043993	ENSBTAG00000043993	1:2029405-2048813	0.416719	0	0.793322	OK
ENSBTAG00000025307	ENSBTAG00000025307	12:68076713-68077964	0.416458	0	0.846512	OK
ENSBTAG00000013039	ENSBTAG00000013039	2:133319862-133323729	0.416143	0	0.814543	OK
ENSBTAG00000039260	ENSBTAG00000039260	3:18623658-18628095	0.416046	0.0656698	0.853708	OK
ENSBTAG00000018309	ENSBTAG00000018309	23:49302302-49303199	0.415715	0	1.16063	OK
ENSBTAG00000039341	ENSBTAG00000039341	18:64743055-64744108	0.415481	0	0.983176	OK
ENSBTAG00000018311	ENSBTAG00000018311	X:32020737-32021265	0.415335	0	1.17646	OK
ENSBTAG00000047596	ENSBTAG00000047596	11:72490793-72491990	0.415316	0	0.8649	OK
ENSBTAG00000047300	ENSBTAG00000047300	7:41568975-41582156	0.415313	0	1.29816	OK
ENSBTAG00000008545	ENSBTAG00000008545	16:72820025-72832974	0.415232	0	0.72688	OK
ENSBTAG00000047957	ENSBTAG00000047957	26:21132750-21133969	0.41521	0	0.8836	OK
ENSBTAG00000006951	ENSBTAG00000006951	15:65394510-65407118	0.415095	0	0.729502	OK
ENSBTAG00000003644	ENSBTAG00000003644	18:62643395-62653550	0.415004	0	0.767351	OK
ENSBTAG00000039328	ENSBTAG00000039328	27:26260212-26261265	0.41479	0	0.786541	OK
ENSBTAG00000001827	ENSBTAG00000001827	13:54936108-54942283	0.414771	0	0.74214	OK
ENSBTAG00000022528	ENSBTAG00000022528	3:10575891-10576857	0.414043	0	1.07172	OK
ENSBTAG00000031772	ENSBTAG00000031772	18:46696419-46711973	0.41395	0	0.807764	OK
ENSBTAG00000019997	ENSBTAG00000019997	14:57479101-57524004	0.413776	0	1.03788	OK
ENSBTAG00000035696	ENSBTAG00000035696	13:43605578-43610260	0.413616	0	0.667926	OK
ENSBTAG00000003992	ENSBTAG00000003992	16:62868731-62915247	0.413171	0	0.706077	OK
ENSBTAG00000032136	ENSBTAG00000032136	25:26099147-26099910	0.413148	0	0.814116	OK

ENSBTAG00000015969	ENSBTAG00000015969	3:110074347-110109946	0.412626	0	0.722292	OK
ENSBTAG00000010256	ENSBTAG00000010256	23:44660174-44674040	0.412564	0	1.01999	OK
ENSBTAG00000008685	ENSBTAG00000008685	8:112962795-112991630	0.412325	0	0.631913	OK
ENSBTAG00000009111	ENSBTAG00000009111	16:2184533-2187560	0.412306	0	0.755682	OK
ENSBTAG00000047985	ENSBTAG00000047985	9:71749777-71750791	0.412292	0	1.02099	OK
ENSBTAG00000034656	ENSBTAG00000034656	24:12798434-12798827	0.412171	0	1.58059	OK
ENSBTAG00000038209	ENSBTAG00000038209	29:5748316-5757809	0.411937	0	0.768586	OK
ENSBTAG00000000199	ENSBTAG00000000199	14:72678593-72686976	0.41124	0	0.959577	OK
ENSBTAG00000031669	ENSBTAG00000031669	11:54723189-55906462	0.410975	0	0.770301	OK
ENSBTAG00000008098	ENSBTAG00000008098	17:7559055-7741485	0.410792	0.0977606	0.782085	OK
ENSBTAG00000007367	ENSBTAG00000007367	25:38576191-38584314	0.410484	0	0.703677	OK
ENSBTAG00000033335	ENSBTAG00000033335	21:13421642-13422116	0.410342	0	1.31049	OK
ENSBTAG00000013791	ENSBTAG00000013791	18:46244899-46249752	0.409789	0	2.04894	OK
ENSBTAG00000047712	ENSBTAG00000047712	18:59365712-59366378	0.409671	0	0.932689	OK
ENSBTAG00000015032	ENSBTAG00000015032	7:53446105-53448979	0.409653	0.074214	0.74214	OK
ENSBTAG00000002039	ENSBTAG00000002039	29:44248434-44269691	0.409319	0.0669437	0.736381	OK
ENSBTAG00000021780	ENSBTAG00000021780	19:30276155-30296404	0.409242	0.109178	0.655071	OK
ENSBTAG00000035855	ENSBTAG00000035855	9:7903534-7904947	0.408957	0	0.732686	OK
ENSBTAG00000045785	ENSBTAG00000045785	5:73905662-73946203	0.408887	0	0.807414	OK
ENSBTAG00000011593	ENSBTAG00000011593	9:100370036-100480357	0.408856	0	0.694356	OK
ENSBTAG00000001817	ENSBTAG00000001817	7:4032540-4046719	0.408789	0.0671827	0.739009	OK
ENSBTAG00000002335	ENSBTAG00000002335	7:67715958-67719274	0.408459	0	0.703876	OK
ENSBTAG00000009789	ENSBTAG00000009789	8:53970971-54280697	0.407806	0	0.925187	OK
ENSBTAG00000020467	ENSBTAG00000020467	24:32570074-32583708	0.40759	0	1.06183	OK
ENSBTAG00000012302	ENSBTAG00000012302	19:23559325-23560666	0.407473	0	0.772024	OK
ENSBTAG00000047893	ENSBTAG00000047893	10:21958924-21960710	0.407038	0	1.10254	OK
ENSBTAG00000027782	ENSBTAG00000027782	1:42420005-42420968	0.40664	0	1.29007	OK
ENSBTAG00000004219	ENSBTAG00000004219	23:10446345-10446756	0.406594	0	1.51136	OK
ENSBTAG00000025462	ENSBTAG00000025462	7:22411967-22414079	0.406338	0	0.77841	OK
ENSBTAG00000001663	ENSBTAG00000001663	18:36598367-36600210	0.4061	0	0.785795	OK

ENSBTAG00000019604	ENSBTAG00000019604	18:53531932-53544919	0.405944	0.0921072	0.736857	OK
ENSBTAG00000011925	ENSBTAG00000011925	10:25589274-25590216	0.405694	0	1.09903	OK
ENSBTAG00000020017	ENSBTAG00000020017	7:19251322-19258151	0.40568	0	0.780365	OK
ENSBTAG00000005004	ENSBTAG00000005004	28:42612011-42616128	0.405463	0	0.958597	OK
ENSBTAG00000046708	ENSBTAG00000046708	18:34709888-34711484	0.405324	0	0.77841	OK
ENSBTAG00000001120	ENSBTAG00000001120	19:21426532-21434772	0.40514	0	0.717564	OK
ENSBTAG00000007970	ENSBTAG00000007970	1:35753285-35759257	0.40513	0	0.920253	OK
ENSBTAG00000012003	ENSBTAG00000012003	4:77409432-77413143	0.404885	0	1.19686	OK
ENSBTAG00000037832	ENSBTAG00000037832	17:68354299-68362110	0.40469	0	0.702364	OK
ENSBTAG00000015464	ENSBTAG00000015464	6:29353893-29354730	0.404438	0	0.74214	OK
ENSBTAG00000047955	ENSBTAG00000047955	21:22661291-22669239	0.404287	0	0.734245	OK
ENSBTAG00000021426	ENSBTAG00000021426	21:34242438-34247229	0.404149	0	0.739112	OK
ENSBTAG00000003442	ENSBTAG00000003442	28:39516529-39527943	0.404122	0	0.775494	OK
ENSBTAG00000013503	ENSBTAG00000013503	15:83005111-83006056	0.403989	0	1.09554	OK
ENSBTAG00000017731	ENSBTAG00000017731	14:8166448-8304533	0.403823	0.0557504	0.724755	OK
ENSBTAG00000039566	ENSBTAG00000039566	3:119869624-119870584	0.403817	0	1.07842	OK
ENSBTAG00000018143	ENSBTAG00000018143	16:4686517-4697039	0.403648	0	0.719662	OK
ENSBTAG00000009768	ENSBTAG00000009768	26:11083266-11090209	0.403372	0	0.83744	OK
ENSBTAG00000009047	ENSBTAG00000009047	25:26451071-26454431	0.403349	0	0.636446	OK
ENSBTAG00000007394	ENSBTAG00000007394	1:97339618-97343997	0.403334	0	1.06639	OK
ENSBTAG00000009048	ENSBTAG00000009048	16:53306775-53324889	0.403204	0	0.771002	OK
ENSBTAG00000036098	ENSBTAG00000036098	14:1136303-1137497	0.402914	0	1.14144	OK
ENSBTAG00000045773	ENSBTAG00000045773	X:29476964-29477684	0.402769	0	1.22278	OK
ENSBTAG00000027889	ENSBTAG00000027889	15:48132341-48133313	0.402691	0	1.06511	OK
ENSBTAG00000015841	ENSBTAG00000015841	2:1243722-1247799	0.402683	0	0.925911	OK
ENSBTAG00000005604	ENSBTAG00000005604	1:65002247-65014687	0.401614	0	0.698337	OK
ENSBTAG00000005385	ENSBTAG00000005385	17:65102637-65104915	0.401359	0	0.747498	OK
ENSBTAG00000003238	ENSBTAG00000003238	4:23943519-24019359	0.401265	0.0901816	0.721453	OK
ENSBTAG00000003474	ENSBTAG00000003474	10:77387905-77408370	0.401206	0.063867	0.702537	OK
ENSBTAG00000005301	ENSBTAG00000005301	9:53360138-53421408	0.401042	0	0.737733	OK

ENSBTAG00000008327	ENSBTAG00000008327	19:57567605-57577938	0.40084	0	0.665778	OK
ENSBTAG00000035975	ENSBTAG00000035975	19:26916674-26922987	0.400784	0	1.13768	OK
ENSBTAG00000018022	ENSBTAG00000018022	29:42781094-42791900	0.40041	0	0.714606	OK
ENSBTAG00000033339	ENSBTAG00000033339	17:45465975-45489004	0.400169	0.0610607	0.732729	OK
ENSBTAG00000046482	ENSBTAG00000046482	10:26113953-26114424	0.400111	0	1.31883	OK
ENSBTAG00000031362	ENSBTAG00000031362	15:25515541-25524857	0.400106	0	0.787289	OK
ENSBTAG00000001948	ENSBTAG00000001948	10:80113760-80131964	0.39998	0	1.9999	OK
ENSBTAG00000031885	ENSBTAG00000031885	21:58404956-58407259	0.399907	0	0.997064	OK
ENSBTAG00000024548	ENSBTAG00000024548	16:37865242-37865936	0.39983	0	0.910808	OK
ENSBTAG00000018391	ENSBTAG00000018391	19:12822572-12833792	0.399723	0	0.704275	OK
ENSBTAG00000015712	ENSBTAG00000015712	X:73224660-73225752	0.399477	0	0.75845	OK
ENSBTAG00000047190	ENSBTAG00000047190	7:34126022-34127202	0.39919	0	0.920253	OK
ENSBTAG00000011038	ENSBTAG00000011038	24:44006263-44024553	0.399143	0	0.853843	OK
ENSBTAG00000032247	ENSBTAG00000032247	23:28707263-28710379	0.399114	0	0.769728	OK
ENSBTAG00000004100	ENSBTAG00000004100	13:67528733-67570054	0.398769	0.106292	0.690898	OK
ENSBTAG00000008597	ENSBTAG00000008597	14:3711836-3739970	0.398691	0	0.572659	OK
ENSBTAG00000004356	ENSBTAG00000004356	29:28719988-28734705	0.398563	0.0561282	0.673538	OK
ENSBTAG00000046440	ENSBTAG00000046440	6:9455205-9456342	0.398176	0	0.910541	OK
ENSBTAG00000037903	ENSBTAG00000037903	19:77880-78836	0.397715	0	1.08293	OK
ENSBTAG00000008379	ENSBTAG00000008379	15:79284950-79285849	0.397642	0	1.1516	OK
ENSBTAG00000010193	ENSBTAG00000010193	22:59862213-59877343	0.397493	0	0.657846	OK
ENSBTAG00000046088	ENSBTAG00000046088	15:84808300-84809334	0.397472	0	1.00124	OK
ENSBTAG00000045838	ENSBTAG00000045838	2:15872135-15872832	0.397244	0	0.891206	OK
ENSBTAG00000009474	ENSBTAG00000009474	14:20596073-20606765	0.396954	0	0.836256	OK
ENSBTAG00000002255	ENSBTAG00000002255	10:10127780-10151271	0.396804	0	0.789433	OK
ENSBTAG00000005305	ENSBTAG00000005305	5:15609694-15621365	0.396752	0	1.03185	OK
ENSBTAG00000009667	ENSBTAG00000009667	7:112332876-112355108	0.396717	0	0.657324	OK
ENSBTAG00000024964	ENSBTAG00000024964	12:18061075-18062037	0.396672	0	1.07618	OK
ENSBTAG00000047548	ENSBTAG00000047548	20:22138977-22139421	0.396584	0	1.39903	OK
ENSBTAG00000033529	ENSBTAG00000033529	15:29291351-29303246	0.396429	0	0.640382	OK

ENSBTAG00000047883	ENSBTAG00000047883	22:15165946-15177342	0.396328	0	0.643034	OK
ENSBTAG00000015879	ENSBTAG00000015879	5:107343178-107373637	0.396036	0.0637294	0.701024	OK
ENSBTAG00000001500	ENSBTAG00000001500	4:5374834-5376886	0.396003	0	0.80724	OK
ENSBTAG00000045727	ENSBTAG00000045727	14:2133424-2146844	0.395625	0.0311013	0.24881	OK
ENSBTAG00000048265	ENSBTAG00000048265	X:50718613-50719778	0.395602	0	1.18657	OK
ENSBTAG00000004207	ENSBTAG00000004207	13:42244267-42247239	0.395468	0	0.684863	OK
ENSBTAG00000021026	ENSBTAG00000021026	X:86605968-86606883	0.395437	0	0.678875	OK
ENSBTAG00000006704	ENSBTAG00000006704	16:27547514-27549591	0.39534	0	0.697833	OK
ENSBTAG00000015315	ENSBTAG00000015315	24:22131077-22132428	0.394803	0	1.04049	OK
ENSBTAG00000026429	ENSBTAG00000026429	25:387896-395807	0.394783	0	0.747884	OK
ENSBTAG00000023986	ENSBTAG00000023986	3:103893618-103899030	0.394768	0	0.733378	OK
ENSBTAG00000046461	ENSBTAG00000046461	4:114910413-114911389	0.394729	0	1.11681	OK
ENSBTAG00000018115	ENSBTAG00000018115	17:59333428-59337958	0.394536	0	0.738434	OK
ENSBTAG00000026139	ENSBTAG00000026139	X:55368950-55370786	0.39452	0	0.902209	OK
ENSBTAG00000007309	ENSBTAG00000007309	3:67863092-68073051	0.394451	0	0.717702	OK
ENSBTAG00000046792	ENSBTAG00000046792	3:119698506-119699475	0.394026	0	1.06841	OK
ENSBTAG00000008125	ENSBTAG00000008125	16:48081091-48137067	0.393919	0	1.96959	OK
ENSBTAG00000009761	ENSBTAG00000009761	11:63552996-63591980	0.393788	0.0772601	0.695341	OK
ENSBTAG00000004706	ENSBTAG00000004706	X:91856089-91868994	0.393632	0.0742938	0.668645	OK
ENSBTAG00000024542	ENSBTAG00000024542	16:40785952-40788409	0.393563	0	0.867557	OK
ENSBTAG00000018610	ENSBTAG00000018610	1:145996974-145997666	0.393357	0	0.897646	OK
ENSBTAG00000011160	ENSBTAG00000011160	8:38691667-38692228	0.393193	0	1.10726	OK
ENSBTAG00000011598	ENSBTAG00000011598	1:85933322-85934782	0.393096	0	0.709099	OK
ENSBTAG00000018033	ENSBTAG00000018033	13:23900872-24085889	0.393028	0	0.687897	OK
ENSBTAG00000001882	ENSBTAG00000001882	18:51679283-51682971	0.392902	0	1.96451	OK
ENSBTAG00000001383	ENSBTAG00000001383	15:80162249-80163970	0.392628	0	1.10489	OK
ENSBTAG00000025233	ENSBTAG00000025233	7:45290573-45299405	0.392601	0	0.622861	OK
ENSBTAG00000038670	ENSBTAG00000038670	5:31633256-31634195	0.39246	0	1.10254	OK
ENSBTAG00000002705	ENSBTAG00000002705	10:20810612-20816425	0.392445	0	0.751416	OK
ENSBTAG00000008693	ENSBTAG00000008693	14:21456451-21464699	0.392358	0	0.802546	OK

ENSBTAG00000009472	ENSBTAG00000009472	4:78839307-78841975	0.392097	0	0.715223	OK
ENSBTAG00000015730	ENSBTAG00000015730	23:7204349-7207052	0.392048	0	0.860347	OK
ENSBTAG00000027373	ENSBTAG00000027373	29:26060989-26062145	0.391901	0	1.36222	OK
ENSBTAG00000005628	ENSBTAG00000005628	2:127396372-127397972	0.391704	0	1.28607	OK
ENSBTAG00000015293	ENSBTAG00000015293	19:37671530-37672235	0.391552	0	0.881093	OK
ENSBTAG00000011224	ENSBTAG00000011224	9:78071005-78073397	0.391145	0	0.752543	OK
ENSBTAG00000047312	ENSBTAG00000047312	10:23342457-23343360	0.391082	0	1.0673	OK
ENSBTAG00000039151	ENSBTAG00000039151	1:35603048-35604149	0.391052	0	1.12838	OK
ENSBTAG00000034691	ENSBTAG00000034691	14:2288554-2293395	0.390881	0	0.628397	OK
ENSBTAG00000004129	ENSBTAG00000004129	19:16196625-16199525	0.390843	0	1.26737	OK
ENSBTAG00000013478	ENSBTAG00000013478	26:18732856-18740515	0.39056	0.0728561	0.655705	OK
ENSBTAG00000040021	ENSBTAG00000040021	14:32532903-32536287	0.390269	0	0.694356	OK
ENSBTAG00000017439	ENSBTAG00000017439	5:111444866-111459411	0.390147	0.139652	0.605157	OK
ENSBTAG00000000158	ENSBTAG00000000158	14:2755205-2762197	0.390051	0	0.961565	OK
ENSBTAG00000005290	ENSBTAG00000005290	X:60134286-60136956	0.390022	0.0775494	0.697945	OK
ENSBTAG00000039711	ENSBTAG00000039711	7:41404241-41406959	0.389955	0.0761799	1.06652	OK
ENSBTAG00000006364	ENSBTAG00000006364	3:14558546-14561397	0.389929	0	0.666635	OK
ENSBTAG00000035241	ENSBTAG00000035241	7:10974873-10975884	0.389574	0	1.22882	OK
ENSBTAG00000003205	ENSBTAG00000003205	11:95850115-95854734	0.389313	0	1.94657	OK
ENSBTAG00000001549	ENSBTAG00000001549	11:93797495-93798488	0.38913	0	1.2511	OK
ENSBTAG00000004662	ENSBTAG00000004662	26:11141010-11161385	0.388849	0	0.737733	OK
ENSBTAG00000044043	ENSBTAG00000044043	27:19273620-19283327	0.38884	0	0.820028	OK
ENSBTAG00000020302	ENSBTAG00000020302	22:39068358-39073040	0.388792	0	0.719305	OK
ENSBTAG00000004777	ENSBTAG00000004777	1:148009650-148016981	0.388648	0	0.882345	OK
ENSBTAG00000002240	ENSBTAG00000002240	12:80095127-80098875	0.388354	0	0.749663	OK
ENSBTAG00000013732	ENSBTAG00000013732	4:89805490-89823611	0.388262	0.0758173	0.682355	OK
ENSBTAG00000000763	ENSBTAG00000000763	15:49740026-49740968	0.387519	0	1.09903	OK
ENSBTAG00000035654	ENSBTAG00000035654	1:137783194-137783923	0.387375	0	0.852086	OK
ENSBTAG00000026966	ENSBTAG00000026966	5:15546052-15579641	0.387237	0	0.949803	OK
ENSBTAG00000009743	ENSBTAG00000009743	13:62007730-62014961	0.387151	0	0.72688	OK

ENSBTAG00000033894	ENSBTAG00000033894	X:124040482-124042203	0.387046	0	1.25034	OK
ENSBTAG00000009033	ENSBTAG00000009033	11:47409405-47414102	0.386796	0	0.666492	OK
ENSBTAG00000038025	ENSBTAG00000038025	3:20771299-20773622	0.386497	0	0.802201	OK
ENSBTAG00000017570	ENSBTAG00000017570	27:25555639-25556381	0.386437	0	0.837158	OK
ENSBTAG00000027930	ENSBTAG00000027930	2:122169404-122170058	0.386367	0	0.949803	OK
ENSBTAG00000014339	ENSBTAG00000014339	29:29619275-29621748	0.386335	0	0.729502	OK
ENSBTAG00000045633	ENSBTAG00000045633	1:120403008-120404088	0.386087	0	0.958597	OK
ENSBTAG00000039110	ENSBTAG00000039110	15:80949243-80950203	0.38582	0	1.07842	OK
ENSBTAG00000027059	ENSBTAG00000027059	21:21724261-21732701	0.385774	0	0.679321	OK
ENSBTAG00000047161	ENSBTAG00000047161	X:138986760-138999328	0.385685	0	0.74214	OK
ENSBTAG00000000750	ENSBTAG00000000750	1:134190684-134220225	0.385585	0	0.822879	OK
ENSBTAG00000019362	ENSBTAG00000019362	6:74016233-74026536	0.385273	0.0880719	0.704575	OK
ENSBTAG00000047994	ENSBTAG00000047994	X:15127647-15128637	0.384896	0	1.04574	OK
ENSBTAG00000046045	ENSBTAG00000046045	X:35334720-35335629	0.384816	0	0.683356	OK
ENSBTAG00000002810	ENSBTAG00000002810	4:113238977-113261531	0.384759	0	0.649081	OK
ENSBTAG00000019719	ENSBTAG00000019719	9:92152081-92209694	0.384615	0	0.878601	OK
ENSBTAG00000012626	ENSBTAG00000012626	13:78464293-78484987	0.38452	0	0.798215	OK
ENSBTAG00000045493	ENSBTAG00000045493	28:23733533-23734721	0.384123	0	1.2987	OK
ENSBTAG00000016167	ENSBTAG00000016167	11:20691385-20758963	0.383812	0	0.944926	OK
ENSBTAG00000011459	ENSBTAG00000011459	19:42666503-42679044	0.383646	0	0.73202	OK
ENSBTAG00000010502	ENSBTAG00000010502	18:50050182-50053543	0.382606	0	0.786691	OK
ENSBTAG00000007584	ENSBTAG00000007584	2:5867811-5903148	0.382572	0	0.79842	OK
ENSBTAG00000013567	ENSBTAG00000013567	21:1852997-1854799	0.382323	0	1.1002	OK
ENSBTAG00000047488	ENSBTAG00000047488	14:4644331-4741649	0.382293	0	0.738171	OK
ENSBTAG00000005211	ENSBTAG00000005211	10:13318874-13323526	0.382069	0	0.745345	OK
ENSBTAG00000040061	ENSBTAG00000040061	1:128532756-128536371	0.381718	0.114554	0.630049	OK
ENSBTAG00000010366	ENSBTAG00000010366	2:122641986-122649721	0.381636	0	0.648066	OK
ENSBTAG00000011310	ENSBTAG00000011310	X:86163689-86189860	0.381593	0	0.728048	OK
ENSBTAG00000020342	ENSBTAG00000020342	16:39957136-39971282	0.381581	0	0.670707	OK
ENSBTAG00000006758	ENSBTAG00000006758	17:45268622-45269814	0.380828	0	0.868528	OK

ENSBTAG00000000806	ENSBTAG00000000806	26:9411264-9448494	0.380415	0	0.788188	OK
ENSBTAG00000002934	ENSBTAG00000002934	23:20745535-20758331	0.38011	0	0.897646	OK
ENSBTAG00000048224	ENSBTAG00000048224	5:58928010-58928949	0.379966	0	1.10254	OK
ENSBTAG00000011995	ENSBTAG00000011995	18:2604151-2607410	0.379861	0	0.757525	OK
ENSBTAG00000013708	ENSBTAG00000013708	25:41480284-41519854	0.37978	0	0.723975	OK
ENSBTAG00000004339	ENSBTAG00000004339	21:20422126-20429481	0.379734	0.0880344	0.704275	OK
ENSBTAG00000002186	ENSBTAG00000002186	21:736182-737792	0.379661	0	0.643034	OK
ENSBTAG00000007449	ENSBTAG00000007449	6:111369913-111406680	0.379415	0	0.667495	OK
ENSBTAG00000011195	ENSBTAG00000011195	2:130781187-130782246	0.3789	0	0.586564	OK
ENSBTAG00000047112	ENSBTAG00000047112	11:93655027-93655969	0.378885	0	1.09903	OK
ENSBTAG00000038665	ENSBTAG00000038665	11:93885138-93886080	0.378826	0	1.09903	OK
ENSBTAG00000046836	ENSBTAG00000046836	14:76820429-76821647	0.378799	0	0.99451	OK
ENSBTAG00000008646	ENSBTAG00000008646	29:43228262-43229598	0.378357	0	1.89178	OK
ENSBTAG00000020453	ENSBTAG00000020453	4:106059819-106070381	0.377896	0	1.11321	OK
ENSBTAG00000046921	ENSBTAG00000046921	15:49419950-49420895	0.377522	0	1.09554	OK
ENSBTAG00000040610	ENSBTAG00000040610	7:187295-188234	0.377503	0	1.32305	OK
ENSBTAG00000015592	ENSBTAG00000015592	5:25825585-25827510	0.377464	0	0.697161	OK
ENSBTAG00000017363	ENSBTAG00000017363	X:126467441-126470489	0.377188	0	0.985986	OK
ENSBTAG00000008576	ENSBTAG00000008576	10:81593973-81624744	0.377025	0	0.664354	OK
ENSBTAG00000024526	ENSBTAG00000024526	27:22570242-22576884	0.376996	0	1.03013	OK
ENSBTAG00000017725	ENSBTAG00000017725	7:42597222-42598194	0.376814	0	1.06511	OK
ENSBTAG00000002566	ENSBTAG00000002566	7:4760643-4798974	0.376734	0.105292	0.631753	OK
ENSBTAG00000016378	ENSBTAG00000016378	10:21786856-21792534	0.376375	0	0.600552	OK
ENSBTAG00000000672	ENSBTAG00000000672	20:58634393-58664249	0.376154	0.0774914	0.619931	OK
ENSBTAG00000047155	ENSBTAG00000047155	28:43930427-43934735	0.37614	0.0961267	0.624824	OK
ENSBTAG00000001117	ENSBTAG00000001117	17:33496004-33529948	0.375893	0.144795	0.627445	OK
ENSBTAG00000025803	ENSBTAG00000025803	10:20379754-20392155	0.375445	0	0.663219	OK
ENSBTAG00000008911	ENSBTAG00000008911	X:102429174-102431592	0.375387	0.0856315	0.685052	OK
ENSBTAG00000019557	ENSBTAG00000019557	1:65205810-65238596	0.375169	0.0890951	0.712761	OK
ENSBTAG00000038608	ENSBTAG00000038608	23:29314809-29315824	0.374972	0	1.0852	OK

ENSBTAG00000010100	ENSBTAG00000010100	23:15685886-15696735	0.374851	0.0839647	0.671718	OK
ENSBTAG00000018046	ENSBTAG00000018046	11:60654633-60687730	0.374834	0.0702602	0.702602	OK
ENSBTAG00000021615	ENSBTAG00000021615	5:109933972-109940236	0.374821	0	0.86514	OK
ENSBTAG00000002311	ENSBTAG00000002311	7:45515184-45520639	0.37413	0	0.671391	OK
ENSBTAG00000008437	ENSBTAG00000008437	7:54690460-54702923	0.37407	0.103296	0.619776	OK
ENSBTAG00000046379	ENSBTAG00000046379	11:105866117-105869982	0.37399	0	0.715223	OK
ENSBTAG00000003174	ENSBTAG00000003174	9:53673440-53682097	0.373807	0	1.24433	OK
ENSBTAG00000032846	ENSBTAG00000032846	2:25705588-25720829	0.373705	0	1.10332	OK
ENSBTAG00000016859	ENSBTAG00000016859	14:79936391-79937019	0.373637	0	1.01333	OK
ENSBTAG00000046950	ENSBTAG00000046950	7:43593813-43594764	0.373431	0	1.08863	OK
ENSBTAG00000048184	ENSBTAG00000048184	18:62910387-62916510	0.372841	0	0.813264	OK
ENSBTAG00000025302	ENSBTAG00000025302	23:34479661-34491996	0.372806	0	1.10137	OK
ENSBTAG00000019931	ENSBTAG00000019931	16:55565386-55567903	0.372757	0	0.641043	OK
ENSBTAG00000020544	ENSBTAG00000020544	25:34163443-34181192	0.372715	0	0.647863	OK
ENSBTAG00000017477	ENSBTAG00000017477	10:81017715-81018344	0.372469	0	0.987553	OK
ENSBTAG00000018773	ENSBTAG00000018773	5:31090624-31097555	0.372153	0	0.63867	OK
ENSBTAG00000016239	ENSBTAG00000016239	10:65536978-65540482	0.371745	0	1.85872	OK
ENSBTAG00000020376	ENSBTAG00000020376	20:31284381-31311777	0.37174	0	0.735113	OK
ENSBTAG00000031802	ENSBTAG00000031802	1:94965197-95364350	0.371641	0	0.916181	OK
ENSBTAG00000003601	ENSBTAG00000003601	X:55653715-55694963	0.371368	0	0.872023	OK
ENSBTAG00000006716	ENSBTAG00000006716	11:93219286-93245045	0.371291	0.0794235	0.635388	OK
ENSBTAG00000008656	ENSBTAG00000008656	12:11363647-11365765	0.371259	0	0.715752	OK
ENSBTAG00000022915	ENSBTAG00000022915	10:27480391-27481303	0.371181	0	1.13518	OK
ENSBTAG00000037807	ENSBTAG00000037807	3:11676780-11678645	0.371081	0	1.03013	OK
ENSBTAG00000016411	ENSBTAG00000016411	27:28450184-28459083	0.370427	0	0.679618	OK
ENSBTAG00000046796	ENSBTAG00000046796	X:9329627-9331874	0.370337	0	0.6501	OK
ENSBTAG00000006643	ENSBTAG00000006643	6:58486549-58565108	0.370329	0	0.647728	OK
ENSBTAG00000004849	ENSBTAG00000004849	3:26735792-26817441	0.370029	0.0559765	0.671718	OK
ENSBTAG00000015771	ENSBTAG00000015771	2:125294727-125324175	0.369576	0	0.791049	OK
ENSBTAG00000014748	ENSBTAG00000014748	5:31898805-31899735	0.369552	0	1.11321	OK

ENSBTAG00000039540	ENSBTAG00000039540	3:93374368-93376742	0.369337	0.0872186	0.697749	OK
ENSBTAG00000015336	ENSBTAG00000015336	3:19788205-19806629	0.369237	0.0740812	0.666731	OK
ENSBTAG00000020236	ENSBTAG00000020236	18:10316498-10350461	0.369147	0	0.608544	OK
ENSBTAG00000039149	ENSBTAG00000039149	15:24660851-24661502	0.368661	0	0.95418	OK
ENSBTAG00000046257	ENSBTAG00000046257	4:113866799-113874303	0.368234	0	0.915169	OK
ENSBTAG00000000014	ENSBTAG00000000014	5:75258028-75267776	0.368184	0	0.594991	OK
ENSBTAG00000020272	ENSBTAG00000020272	15:22246390-22372643	0.36799	0.0841182	0.630887	OK
ENSBTAG00000014944	ENSBTAG00000014944	7:21561178-21569885	0.367888	0	0.73313	OK
ENSBTAG00000013740	ENSBTAG00000013740	19:39194569-39296546	0.367731	0	0.980616	OK
ENSBTAG00000003249	ENSBTAG00000003249	3:95217-96237	0.367638	0	1.14904	OK
ENSBTAG00000022275	ENSBTAG00000022275	1:54649605-54650250	0.367473	0	0.963056	OK
ENSBTAG00000009418	ENSBTAG00000009418	4:107560494-107567847	0.367351	0	0.683356	OK
ENSBTAG00000011973	ENSBTAG00000011973	6:38754414-38754897	0.367244	0	1.28607	OK
ENSBTAG00000009132	ENSBTAG00000009132	1:143211538-143246217	0.367197	0	0.696868	OK
ENSBTAG00000009148	ENSBTAG00000009148	3:1726503-1779667	0.367149	0	0.69999	OK
ENSBTAG00000047499	ENSBTAG00000047499	20:70187677-70191093	0.366969	0	0.733205	OK
ENSBTAG00000008579	ENSBTAG00000008579	2:135784597-135809900	0.366946	0	1.52984	OK
ENSBTAG00000021576	ENSBTAG00000021576	16:49624531-49651879	0.366308	0	0.581387	OK
ENSBTAG00000019267	ENSBTAG00000019267	18:23828637-23855657	0.366239	0.0701412	0.631271	OK
ENSBTAG00000008908	ENSBTAG00000008908	13:42407300-42413062	0.366077	0	0.656542	OK
ENSBTAG00000012757	ENSBTAG00000012757	8:52848303-52850731	0.365937	0	0.767509	OK
ENSBTAG00000030722	ENSBTAG00000030722	7:43278133-43280173	0.365658	0	1.05212	OK
ENSBTAG00000007196	ENSBTAG00000007196	15:28318410-28323666	0.365157	0	0.556605	OK
ENSBTAG00000008719	ENSBTAG00000008719	22:48307243-48337780	0.364998	0.069599	0.626391	OK
ENSBTAG00000018908	ENSBTAG00000018908	18:35344407-35383712	0.364531	0	0.718591	OK
ENSBTAG00000030976	ENSBTAG00000030976	13:65424261-65426839	0.364473	0	0.754581	OK
ENSBTAG00000017941	ENSBTAG00000017941	25:34332033-34337702	0.363851	0	0.646245	OK
ENSBTAG00000048032	ENSBTAG00000048032	15:84278444-84279377	0.362848	0	0.665778	OK
ENSBTAG00000048054	ENSBTAG00000048054	11:106156868-106161862	0.362633	0	0.605076	OK
ENSBTAG00000005103	ENSBTAG00000005103	4:87501283-87503583	0.362527	0	0.723469	OK

ENSBTAG00000018809	ENSBTAG00000018809	19:28519718-28521133	0.362085	0	1.25034	OK
ENSBTAG00000007530	ENSBTAG00000007530	23:28613832-28622421	0.362017	0	0.714694	OK
ENSBTAG00000045685	ENSBTAG00000045685	3:118208053-118223089	0.361848	0	0.633202	OK
ENSBTAG00000037965	ENSBTAG00000037965	1:95203731-95206770	0.361808	0	0.800684	OK
ENSBTAG00000001405	ENSBTAG00000001405	10:16108198-16117541	0.361772	0.0585734	0.702881	OK
ENSBTAG00000015711	ENSBTAG00000015711	16:890071-892370	0.361465	0	0.700305	OK
ENSBTAG00000037826	ENSBTAG00000037826	15:52268974-52271899	0.361443	0	1.80721	OK
ENSBTAG00000034449	ENSBTAG00000034449	3:5454660-5455256	0.361399	0	1.04223	OK
ENSBTAG00000047119	ENSBTAG00000047119	28:27613177-27647456	0.361395	0	0.921619	OK
ENSBTAG00000011512	ENSBTAG00000011512	1:151008562-151021272	0.361392	0	1.09942	OK
ENSBTAG00000018978	ENSBTAG00000018978	13:38638356-38661300	0.361254	0	0.648791	OK
ENSBTAG00000022858	ENSBTAG00000022858	15:80555498-80556455	0.361083	0	1.0818	OK
ENSBTAG00000016826	ENSBTAG00000016826	10:35566118-35695711	0.361067	0	0.767233	OK
ENSBTAG00000015876	ENSBTAG00000015876	15:79824623-79825547	0.360889	0	1.12044	OK
ENSBTAG00000000082	ENSBTAG00000000082	18:55584938-55588381	0.360697	0	0.707162	OK
ENSBTAG00000011268	ENSBTAG00000011268	14:75870562-75904774	0.360523	0	0.696575	OK
ENSBTAG00000046318	ENSBTAG00000046318	7:42747368-42748307	0.36035	0	1.10254	OK
ENSBTAG00000006372	ENSBTAG00000006372	5:82452436-82468701	0.359985	0	0.654829	OK
ENSBTAG00000006373	ENSBTAG00000006373	3:14531699-14538480	0.359777	0	1.23248	OK
ENSBTAG00000046764	ENSBTAG00000046764	15:48252516-48253458	0.359769	0	1.09903	OK
ENSBTAG00000014229	ENSBTAG00000014229	25:16821398-16908660	0.359721	0	0.600165	OK
ENSBTAG00000007917	ENSBTAG00000007917	13:81601020-81604089	0.359659	0.0674672	0.607205	OK
ENSBTAG00000001491	ENSBTAG00000001491	6:58461963-58480538	0.359635	0.127498	0.552492	OK
ENSBTAG00000046679	ENSBTAG00000046679	11:11874422-11874812	0.359402	0	1.65646	OK
ENSBTAG00000038126	ENSBTAG00000038126	19:41162357-41177893	0.359391	0	0.657741	OK
ENSBTAG00000018436	ENSBTAG00000018436	9:61766992-61782483	0.359365	0	0.8649	OK
ENSBTAG00000034302	ENSBTAG00000034302	18:63705512-63706484	0.359363	0	0.639065	OK
ENSBTAG00000040293	ENSBTAG00000040293	15:67827232-67830364	0.359212	0.0661101	0.594991	OK
ENSBTAG00000017769	ENSBTAG00000017769	X:96253140-96266985	0.359094	0	0.708614	OK
ENSBTAG00000025812	ENSBTAG00000025812	5:63238916-63239757	0.358868	0	0.73861	OK

ENSBTAG00000013413	ENSBTAG00000013413	22:51994223-52003466	0.358812	0	0.653177	OK
ENSBTAG00000022796	ENSBTAG00000022796	8:48953077-48954464	0.358374	0	0.74642	OK
ENSBTAG00000038916	ENSBTAG00000038916	23:15604080-15608831	0.35831	0	0.714482	OK
ENSBTAG00000015379	ENSBTAG00000015379	13:52501258-52548991	0.357919	0.0872554	0.698043	OK
ENSBTAG00000002357	ENSBTAG00000002357	10:4374428-4391820	0.357679	0	0.76631	OK
ENSBTAG00000026375	ENSBTAG00000026375	25:10052223-10057594	0.357491	0	0.714482	OK
ENSBTAG00000018211	ENSBTAG00000018211	16:67881454-67882024	0.357245	0	1.08977	OK
ENSBTAG00000047239	ENSBTAG00000047239	10:66692833-66693863	0.357063	0	1.07172	OK
ENSBTAG00000006812	ENSBTAG00000006812	29:13003057-13004907	0.356868	0	0.671536	OK
ENSBTAG00000026826	ENSBTAG00000026826	15:45497676-45498609	0.356796	0	1.10963	OK
ENSBTAG00000014354	ENSBTAG00000014354	15:28926759-28963808	0.356704	0	0.6727	OK
ENSBTAG00000002390	ENSBTAG00000002390	5:32277007-32278883	0.356612	0	0.662229	OK
ENSBTAG00000009359	ENSBTAG00000009359	15:38876121-38876667	0.356443	0	1.13768	OK
ENSBTAG00000047507	ENSBTAG00000047507	7:15647683-15649879	0.356067	0	0.754306	OK
ENSBTAG00000018598	ENSBTAG00000018598	18:46654242-46656762	0.356014	0	0.887387	OK
ENSBTAG00000018249	ENSBTAG00000018249	3:15781067-15948350	0.355999	0	0.66334	OK
ENSBTAG00000010065	ENSBTAG00000010065	X:65246770-65449919	0.355954	0.0707887	0.707887	OK
ENSBTAG00000014424	ENSBTAG00000014424	5:30984169-30989090	0.355486	0.0597912	0.597912	OK
ENSBTAG00000002377	ENSBTAG00000002377	3:110720282-110756534	0.355485	0	0.771641	OK
ENSBTAG00000001440	ENSBTAG00000001440	25:7700703-7730822	0.355381	0	0.694489	OK
ENSBTAG00000021861	ENSBTAG00000021861	11:105843376-105850401	0.355078	0	0.7007	OK
ENSBTAG00000014412	ENSBTAG00000014412	7:17051781-17059767	0.355069	0	0.56113	OK
ENSBTAG00000019235	ENSBTAG00000019235	5:99447174-99457656	0.355009	0	0.871452	OK
ENSBTAG00000040361	ENSBTAG00000040361	25:41304500-41313035	0.354998	0	0.632925	OK
ENSBTAG00000006709	ENSBTAG00000006709	16:42712043-42713502	0.35487	0	0.74214	OK
ENSBTAG00000017613	ENSBTAG00000017613	18:64870964-64878298	0.354763	0	0.678875	OK
ENSBTAG00000021127	ENSBTAG00000021127	8:53686441-53949026	0.354704	0	0.621047	OK
ENSBTAG00000021417	ENSBTAG00000021417	18:53770875-53777767	0.354014	0	0.582556	OK
ENSBTAG00000018199	ENSBTAG00000018199	2:121847011-121901991	0.353894	0	0.857592	OK
ENSBTAG00000016770	ENSBTAG00000016770	20:20811836-20816932	0.353626	0	1.01887	OK

ENSBTAG00000040208	ENSBTAG00000040208	10:17720047-17721650	0.353522	0	0.77501	OK
ENSBTAG00000008134	ENSBTAG00000008134	15:59164540-59200908	0.353474	0	0.7007	OK
ENSBTAG00000000462	ENSBTAG00000000462	18:25580586-25602166	0.353055	0	0.60746	OK
ENSBTAG00000005424	ENSBTAG00000005424	15:35272153-35317245	0.352863	0.0653177	0.587859	OK
ENSBTAG00000009887	ENSBTAG00000009887	5:110675823-110691542	0.352616	0	0.640194	OK
ENSBTAG00000009137	ENSBTAG00000009137	18:57812528-57815281	0.352094	0	0.743739	OK
ENSBTAG00000045589	ENSBTAG00000045589	7:44464559-44465483	0.352059	0	1.12044	OK
ENSBTAG00000015150	ENSBTAG00000015150	1:108285282-108291983	0.351981	0	0.900248	OK
ENSBTAG00000014471	ENSBTAG00000014471	8:101361500-101406330	0.351972	0.156293	0.562655	OK
ENSBTAG00000031026	ENSBTAG00000031026	15:79187284-79188214	0.351886	0	0.667926	OK
ENSBTAG00000005098	ENSBTAG00000005098	X:96541497-96542079	0.351336	0	1.0673	OK
ENSBTAG00000031519	ENSBTAG00000031519	22:52858271-52862341	0.35127	0	0.560624	OK
ENSBTAG00000046255	ENSBTAG00000046255	18:39376390-39378454	0.351088	0	0.60191	OK
ENSBTAG00000006485	ENSBTAG00000006485	23:15081866-15086276	0.351036	0	0.631913	OK
ENSBTAG00000031852	ENSBTAG00000031852	25:26801395-26811293	0.350765	0	1.75383	OK
ENSBTAG00000040478	ENSBTAG00000040478	15:50463259-50464198	0.350713	0	1.10254	OK
ENSBTAG00000000224	ENSBTAG00000000224	28:25960050-26012583	0.350226	0	0.651532	OK
ENSBTAG00000020997	ENSBTAG00000020997	1:42468326-42469403	0.349807	0	0.961267	OK
ENSBTAG00000022382	ENSBTAG00000022382	29:28903055-28918088	0.349551	0	0.814116	OK
ENSBTAG00000004587	ENSBTAG00000004587	5:19276347-19280028	0.349524	0	0.604546	OK
ENSBTAG00000020515	ENSBTAG00000020515	15:47441672-47455959	0.349111	0	0.612336	OK
ENSBTAG00000030714	ENSBTAG00000030714	7:43686274-43687228	0.348778	0	1.0852	OK
ENSBTAG00000000277	ENSBTAG00000000277	15:22800426-22826698	0.348542	0	1.20558	OK
ENSBTAG00000009187	ENSBTAG00000009187	1:1308526-1310072	0.348397	0	1.06456	OK
ENSBTAG00000008641	ENSBTAG00000008641	4:40775884-40830619	0.348352	0	1.16652	OK
ENSBTAG00000013625	ENSBTAG00000013625	X:129541463-129542201	0.347746	0	0.841695	OK
ENSBTAG00000037489	ENSBTAG00000037489	25:28255763-28294982	0.347063	0	0.64144	OK
ENSBTAG00000007052	ENSBTAG00000007052	21:62760702-62772148	0.347055	0	0.549223	OK
ENSBTAG00000018349	ENSBTAG00000018349	7:4981194-4984553	0.346989	0	0.643034	OK
ENSBTAG00000010860	ENSBTAG00000010860	15:24303203-24316174	0.346984	0	1.20587	OK

ENSBTAG00000015945	ENSBTAG00000015945	16:2690257-2692411	0.346929	0	0.672887	OK
ENSBTAG00000011190	ENSBTAG00000011190	X:40310756-40332714	0.346926	0.0502626	0.402101	OK
ENSBTAG00000024096	ENSBTAG00000024096	3:110065580-110068037	0.346854	0	0.612595	OK
ENSBTAG00000003027	ENSBTAG00000003027	26:38163824-38170070	0.346766	0	0.670707	OK
ENSBTAG00000008959	ENSBTAG00000008959	23:27600054-27604052	0.346741	0	0.582849	OK
ENSBTAG00000017321	ENSBTAG00000017321	1:145031788-145041320	0.346617	0	0.646245	OK
ENSBTAG00000007067	ENSBTAG00000007067	12:16084410-16183956	0.346604	0.117512	0.548391	OK
ENSBTAG00000016807	ENSBTAG00000016807	15:968399-969332	0.346132	0	1.10963	OK
ENSBTAG00000030334	ENSBTAG00000030334	19:43508613-43513367	0.345919	0	0.630722	OK
ENSBTAG00000021122	ENSBTAG00000021122	5:58067220-58068156	0.345832	0	1.10607	OK
ENSBTAG00000046023	ENSBTAG00000046023	23:29299941-29300874	0.345756	0	1.10963	OK
ENSBTAG00000002008	ENSBTAG00000002008	4:96266936-96267801	0.345263	0	0.732513	OK
ENSBTAG00000019504	ENSBTAG00000019504	13:51370889-51392113	0.345037	0	0.607561	OK
ENSBTAG00000004392	ENSBTAG00000004392	29:46407486-46417346	0.345031	0	0.674453	OK
ENSBTAG00000008062	ENSBTAG00000008062	8:43780985-43787918	0.345018	0	0.608224	OK
ENSBTAG00000038104	ENSBTAG00000038104	2:127251766-127255331	0.344921	0	0.858445	OK
ENSBTAG00000001151	ENSBTAG00000001151	18:46754201-46764958	0.344849	0	0.62582	OK
ENSBTAG00000004893	ENSBTAG00000004893	6:106492147-106531843	0.344749	0	0.654208	OK
ENSBTAG00000000477	ENSBTAG00000000477	16:27303740-27306317	0.344691	0	0.964177	OK
ENSBTAG00000039634	ENSBTAG00000039634	21:28691424-28692943	0.344544	0	0.681557	OK
ENSBTAG00000032848	ENSBTAG00000032848	20:41498516-41550461	0.344428	0.0770301	0.616241	OK
ENSBTAG00000017996	ENSBTAG00000017996	18:50326697-50334391	0.344413	0	0.583588	OK
ENSBTAG00000014840	ENSBTAG00000014840	3:15464425-15468964	0.344357	0	0.567798	OK
ENSBTAG00000019732	ENSBTAG00000019732	8:6818690-6913336	0.344247	0	0.917806	OK
ENSBTAG00000032808	ENSBTAG00000032808	16:41027787-41036681	0.344134	0	0.671173	OK
ENSBTAG00000020355	ENSBTAG00000020355	8:98843683-98847740	0.34383	0	0.618608	OK
ENSBTAG00000034531	ENSBTAG00000034531	9:24273356-24300611	0.343796	0	0.664709	OK
ENSBTAG00000017993	ENSBTAG00000017993	18:60384486-60386112	0.34376	0	0.828228	OK
ENSBTAG00000047709	ENSBTAG00000047709	X:123603171-123605485	0.34325	0	0.948063	OK
ENSBTAG00000045966	ENSBTAG00000045966	6:36989138-36991481	0.343243	0	0.618608	OK

ENSBTAG00000012349	ENSBTAG00000012349	16:1327007-1335059	0.343218	0	0.601327	OK
ENSBTAG00000019845	ENSBTAG00000019845	17:66239944-66263958	0.343094	0.06895	0.5516	OK
ENSBTAG00000002144	ENSBTAG00000002144	7:62229185-62230442	0.343054	0	0.823616	OK
ENSBTAG00000014123	ENSBTAG00000014123	18:43038545-43068295	0.342596	0	0.867254	OK
ENSBTAG00000017015	ENSBTAG00000017015	25:9423861-9474899	0.342469	0	0.650713	OK
ENSBTAG00000030258	ENSBTAG00000030258	5:109925708-109933537	0.342294	0	0.59272	OK
ENSBTAG00000004342	ENSBTAG00000004342	29:45271647-45274221	0.342227	0.0804417	0.643534	OK
ENSBTAG00000008287	ENSBTAG00000008287	10:86425790-86479573	0.341467	0.11663	0.544273	OK
ENSBTAG00000022684	ENSBTAG00000022684	20:3004313-3005537	0.341322	0	1.0818	OK
ENSBTAG00000016624	ENSBTAG00000016624	1:83676970-83683796	0.341217	0	0.770301	OK
ENSBTAG00000005744	ENSBTAG00000005744	6:99812237-99839387	0.341168	0	0.669654	OK
ENSBTAG00000046760	ENSBTAG00000046760	5:120826687-120858589	0.341076	0	0.524638	OK
ENSBTAG00000019665	ENSBTAG00000019665	11:46699165-46706152	0.340932	0	0.663644	OK
ENSBTAG00000015806	ENSBTAG00000015806	10:45428931-45433129	0.340341	0	0.637098	OK
ENSBTAG00000047619	ENSBTAG00000047619	5:59672294-59673287	0.340313	0	1.04258	OK
ENSBTAG00000020103	ENSBTAG00000020103	X:64976876-64977519	0.340223	0	0.982865	OK
ENSBTAG00000000056	ENSBTAG00000000056	19:48628882-48656396	0.340009	0	0.95833	OK
ENSBTAG00000007392	ENSBTAG00000007392	21:59667404-59678093	0.339991	0	0.848594	OK
ENSBTAG00000000385	ENSBTAG00000000385	16:33913962-33918059	0.339757	0	0.593286	OK
ENSBTAG00000001728	ENSBTAG00000001728	1:117532036-117544618	0.339647	0.116243	0.639334	OK
ENSBTAG00000046138	ENSBTAG00000046138	3:18108306-18109095	0.339256	0	0.828228	OK
ENSBTAG00000008063	ENSBTAG00000008063	5:117151548-117233112	0.339133	0	0.638736	OK
ENSBTAG00000038444	ENSBTAG00000038444	23:29552974-29554181	0.338814	0	1.07172	OK
ENSBTAG00000037781	ENSBTAG00000037781	23:27055777-27057357	0.338531	0	0.549223	OK
ENSBTAG00000017425	ENSBTAG00000017425	12:36335915-36340957	0.338435	0	0.643605	OK
ENSBTAG00000020300	ENSBTAG00000020300	X:40247365-40254767	0.337989	0	1.68994	OK
ENSBTAG00000012725	ENSBTAG00000012725	10:21678736-21695268	0.337913	0	0.634588	OK
ENSBTAG00000017313	ENSBTAG00000017313	5:109436266-109454513	0.337733	0	0.645439	OK
ENSBTAG00000000374	ENSBTAG00000000374	11:58433586-58435146	0.337679	0	1.06183	OK
ENSBTAG00000027760	ENSBTAG00000027760	14:21890882-21891976	0.337319	0	0.567798	OK

ENSBTAG00000008839	ENSBTAG00000008839	X:101529563-101532923	0.337294	0.0616241	0.554617	OK
ENSBTAG00000006566	ENSBTAG00000006566	18:46312177-46314341	0.337202	0	0.553135	OK
ENSBTAG00000047069	ENSBTAG00000047069	9:99863455-99864100	0.337126	0	0.970579	OK
ENSBTAG00000009431	ENSBTAG00000009431	3:14627938-14644796	0.337046	0	0.691535	OK
ENSBTAG00000039983	ENSBTAG00000039983	7:7972553-7973501	0.336982	0	0.655244	OK
ENSBTAG00000030559	ENSBTAG00000030559	19:41908535-41909488	0.336861	0	1.08634	OK
ENSBTAG00000010126	ENSBTAG00000010126	13:75404992-75416988	0.33668	0.0607561	0.607561	OK
ENSBTAG00000035018	ENSBTAG00000035018	X:3126451-3129071	0.336338	0	0.608991	OK
ENSBTAG00000005841	ENSBTAG00000005841	18:63349486-63355736	0.336188	0	0.639394	OK
ENSBTAG00000003425	ENSBTAG00000003425	13:38546388-38554838	0.336072	0	0.585458	OK
ENSBTAG00000020668	ENSBTAG00000020668	13:47904559-47905533	0.33599	0	1.06292	OK
ENSBTAG00000005285	ENSBTAG00000005285	4:105101879-105110529	0.335986	0	0.895059	OK
ENSBTAG00000011079	ENSBTAG00000011079	18:57317722-57324156	0.335982	0	0.600862	OK
ENSBTAG00000018037	ENSBTAG00000018037	18:35521164-35534184	0.335962	0	0.636609	OK
ENSBTAG00000003132	ENSBTAG00000003132	21:31518369-31537227	0.335946	0	0.694356	OK
ENSBTAG00000025126	ENSBTAG00000025126	19:26675244-26676864	0.335928	0	0.671391	OK
ENSBTAG00000045683	ENSBTAG00000045683	7:54130984-54133372	0.335821	0	0.606951	OK
ENSBTAG00000046508	ENSBTAG00000046508	6:69242940-69243912	0.335682	0	1.35924	OK
ENSBTAG00000048159	ENSBTAG00000048159	X:117948889-117980661	0.335478	0	0.922303	OK
ENSBTAG00000007020	ENSBTAG00000007020	22:16514155-16523810	0.335285	0	0.732729	OK
ENSBTAG00000001243	ENSBTAG00000001243	27:36716851-36734481	0.335283	0	0.669468	OK
ENSBTAG00000018768	ENSBTAG00000018768	1:140906954-140913324	0.334935	0	0.864178	OK
ENSBTAG00000013869	ENSBTAG00000013869	3:115210339-115232304	0.334342	0.0899856	0.539914	OK
ENSBTAG00000033092	ENSBTAG00000033092	7:8067705-8068653	0.33431	0	0.655244	OK
ENSBTAG00000010129	ENSBTAG00000010129	12:62666723-62669717	0.334237	0	0.612142	OK
ENSBTAG00000045995	ENSBTAG00000045995	3:759065-759496	0.333888	0	1.52998	OK
ENSBTAG00000004432	ENSBTAG00000004432	X:63497120-63590236	0.333776	0	0.740812	OK
ENSBTAG00000005581	ENSBTAG00000005581	7:18000483-18003965	0.333764	0	0.655935	OK
ENSBTAG00000002180	ENSBTAG00000002180	2:97070143-97071259	0.333657	0	0.927675	OK
ENSBTAG00000014981	ENSBTAG00000014981	21:59231721-59254106	0.333313	0.0680661	0.544528	OK

ENSBTAG00000005003	ENSBTAG00000005003	28:42594372-42603915	0.333255	0	0.48265	OK
ENSBTAG00000015878	ENSBTAG00000015878	5:107374653-107377720	0.333234	0	0.698102	OK
ENSBTAG00000005475	ENSBTAG00000005475	4:107804451-107821622	0.33315	0.0556007	0.556007	OK
ENSBTAG00000001470	ENSBTAG00000001470	25:34979328-35015686	0.333019	0	0.665472	OK
ENSBTAG00000045743	ENSBTAG00000045743	7:53990708-53993078	0.332814	0	0.611561	OK
ENSBTAG00000014821	ENSBTAG00000014821	10:21832583-21864021	0.332638	0	0.60602	OK
ENSBTAG00000007170	ENSBTAG00000007170	18:26514590-26525996	0.332527	0.0584741	0.584741	OK
ENSBTAG00000045803	ENSBTAG00000045803	18:63769584-63772329	0.332427	0	0.542034	OK
ENSBTAG00000014136	ENSBTAG00000014136	16:51841818-51846448	0.332104	0	0.575558	OK
ENSBTAG00000013009	ENSBTAG00000013009	13:60092042-60107177	0.332009	0	0.643034	OK
ENSBTAG00000040043	ENSBTAG00000040043	X:55413912-55415816	0.331983	0	0.65249	OK
ENSBTAG00000009310	ENSBTAG00000009310	6:87798135-87799560	0.331903	0	0.726516	OK
ENSBTAG00000022663	ENSBTAG00000022663	7:10193997-10194954	0.331729	0	1.0818	OK
ENSBTAG00000011530	ENSBTAG00000011530	18:14302695-14321678	0.331728	0	0.491655	OK
ENSBTAG00000013279	ENSBTAG00000013279	X:92369103-92375461	0.331583	0	0.592014	OK
ENSBTAG00000016835	ENSBTAG00000016835	23:24390406-24397956	0.331453	0	0.763484	OK
ENSBTAG00000026063	ENSBTAG00000026063	X:17028729-17030114	0.331296	0	0.826025	OK
ENSBTAG00000014376	ENSBTAG00000014376	17:65519356-65552128	0.331062	0	0.654947	OK
ENSBTAG00000039735	ENSBTAG00000039735	15:50235978-50236920	0.330597	0	1.09903	OK
ENSBTAG00000026111	ENSBTAG00000026111	11:69592078-69618485	0.330563	0	0.654061	OK
ENSBTAG00000047478	ENSBTAG00000047478	7:53933783-53936171	0.330504	0	0.606951	OK
ENSBTAG00000012043	ENSBTAG00000012043	2:135891756-135920584	0.330455	0	0.619931	OK
ENSBTAG00000007372	ENSBTAG00000007372	25:38589843-38593638	0.330327	0	0.545605	OK
ENSBTAG00000000441	ENSBTAG00000000441	X:58542824-58551785	0.330106	0	0.555609	OK
ENSBTAG00000047323	ENSBTAG00000047323	28:29087620-29095422	0.329904	0	1.64348	OK
ENSBTAG00000033268	ENSBTAG00000033268	14:41988046-42189775	0.329861	0.170761	0.474336	OK
ENSBTAG00000046016	ENSBTAG00000046016	5:59243491-59244427	0.329826	0	1.10607	OK
ENSBTAG00000039443	ENSBTAG00000039443	8:60662374-60663331	0.329742	0	1.0818	OK
ENSBTAG00000046409	ENSBTAG00000046409	28:19012869-19015927	0.32956	0	0.595333	OK
ENSBTAG00000031687	ENSBTAG00000031687	18:47062612-47063491	0.329397	0	1.1778	OK

ENSBTAG00000002012	ENSBTAG00000002012	10:45936705-45952110	0.329275	0	0.714811	OK
ENSBTAG00000010370	ENSBTAG00000010370	21:71041899-71047128	0.329264	0	0.594991	OK
ENSBTAG00000038595	ENSBTAG00000038595	X:112749383-112760963	0.329036	0	0.803065	OK
ENSBTAG00000045654	ENSBTAG00000045654	14:1127006-1127948	0.32884	0	1.10489	OK
ENSBTAG00000046982	ENSBTAG00000046982	7:14999355-15000312	0.328668	0	1.0818	OK
ENSBTAG00000031355	ENSBTAG00000031355	22:53826231-53832796	0.328615	0	0.75075	OK
ENSBTAG00000007794	ENSBTAG00000007794	19:41720558-41731744	0.328594	0	0.739335	OK
ENSBTAG00000001049	ENSBTAG00000001049	X:145362861-145568526	0.328194	0	1.64097	OK
ENSBTAG00000024593	ENSBTAG00000024593	20:153401-155113	0.328086	0	1.0885	OK
ENSBTAG00000008523	ENSBTAG00000008523	X:92189577-92201107	0.327928	0	0.901803	OK
ENSBTAG00000046948	ENSBTAG00000046948	X:104775258-104777775	0.327745	0	0.869531	OK
ENSBTAG00000033220	ENSBTAG00000033220	3:16367443-16370673	0.32771	0	0.89635	OK
ENSBTAG00000007092	ENSBTAG00000007092	X:134606819-134643886	0.327688	0	0.89635	OK
ENSBTAG00000007484	ENSBTAG00000007484	17:65220604-65237484	0.327687	0	0.634819	OK
ENSBTAG00000032859	ENSBTAG00000032859	15:35836625-35837711	0.327605	0	1.13146	OK
ENSBTAG00000013683	ENSBTAG00000013683	15:51434157-51435105	0.327449	0	0.655244	OK
ENSBTAG00000048158	ENSBTAG00000048158	21:7195110-7196130	0.327084	0	1.04469	OK
ENSBTAG00000037883	ENSBTAG00000037883	X:106264819-106265721	0.326943	0	0.68866	OK
ENSBTAG00000024081	ENSBTAG00000024081	8:85540500-85579683	0.326877	0	0.852476	OK
ENSBTAG00000005219	ENSBTAG00000005219	X:1383594-1454584	0.326802	0	0.806846	OK
ENSBTAG00000048264	ENSBTAG00000048264	29:43048587-43050621	0.326774	0	0.610787	OK
ENSBTAG00000016905	ENSBTAG00000016905	5:31606427-31607357	0.326667	0	1.11321	OK
ENSBTAG00000008844	ENSBTAG00000008844	8:9832643-9870025	0.326631	0	0.550683	OK
ENSBTAG00000005562	ENSBTAG00000005562	2:41791845-42371758	0.326627	0	0.743472	OK
ENSBTAG00000013227	ENSBTAG00000013227	14:21577308-21580910	0.326245	0	0.62492	OK
ENSBTAG00000019422	ENSBTAG00000019422	11:22366048-22367102	0.326167	0	1.02099	OK
ENSBTAG00000008877	ENSBTAG00000008877	8:45865187-45949526	0.326131	0	0.57791	OK
ENSBTAG00000031871	ENSBTAG00000031871	23:30175392-30179010	0.32606	0.0624983	0.624983	OK
ENSBTAG00000032217	ENSBTAG00000032217	1:84324176-84325181	0.32598	0	1.23616	OK
ENSBTAG00000009840	ENSBTAG00000009840	25:37071830-37072784	0.32543	0	1.0852	OK

ENSBTAG00000003438	ENSBTAG00000003438	11:97582041-97608142	0.325364	0.07182	0.57456	OK
ENSBTAG00000035282	ENSBTAG00000035282	26:401624-402566	0.325306	0	1.09903	OK
ENSBTAG00000032923	ENSBTAG00000032923	X:49737022-49737868	0.325272	0	0.734245	OK
ENSBTAG00000010850	ENSBTAG00000010850	16:74993031-75000062	0.325081	0.0558557	0.558557	OK
ENSBTAG00000043990	ENSBTAG00000043990	23:184672-864355	0.324847	0	0.787888	OK
ENSBTAG00000007827	ENSBTAG00000007827	29:42868377-42872337	0.324818	0	0.629735	OK
ENSBTAG00000046018	ENSBTAG00000046018	11:93304996-93305947	0.324522	0	1.08863	OK
ENSBTAG00000006802	ENSBTAG00000006802	13:63227592-63239140	0.324228	0	1.18792	OK
ENSBTAG00000007954	ENSBTAG00000007954	25:26646274-26649700	0.323937	0	0.53851	OK
ENSBTAG00000025442	ENSBTAG00000025442	23:27334343-27338328	0.323895	0	0.712761	OK
ENSBTAG00000021601	ENSBTAG00000021601	13:79433477-79448501	0.323834	0	0.570798	OK
ENSBTAG00000020227	ENSBTAG00000020227	16:3836699-3849134	0.323595	0	0.79599	OK
ENSBTAG00000006831	ENSBTAG00000006831	8:48456363-48516656	0.323584	0	1.1356	OK
ENSBTAG00000040168	ENSBTAG00000040168	23:30206788-30211745	0.323298	0	0.784307	OK
ENSBTAG00000004835	ENSBTAG00000004835	2:20776407-20780505	0.322995	0	0.576359	OK
ENSBTAG00000000312	ENSBTAG00000000312	14:2018558-2021709	0.322813	0	0.58957	OK
ENSBTAG00000001495	ENSBTAG00000001495	13:75122285-75129707	0.322746	0	1.01598	OK
ENSBTAG00000011278	ENSBTAG00000011278	22:11813890-11849423	0.322378	0	0.618696	OK
ENSBTAG00000001714	ENSBTAG00000001714	11:7422150-7432712	0.322018	0	0.570405	OK
ENSBTAG00000013366	ENSBTAG00000013366	15:44175365-44203114	0.322006	0	0.659067	OK
ENSBTAG00000023748	ENSBTAG00000023748	3:103596243-103597188	0.322001	0	1.09554	OK
ENSBTAG00000009384	ENSBTAG00000009384	16:42806429-42825905	0.321844	0	0.567798	OK
ENSBTAG00000047332	ENSBTAG00000047332	X:1957142-1957953	0.321785	0	0.787289	OK
ENSBTAG00000010233	ENSBTAG00000010233	29:44092126-44114038	0.321766	0	0.61956	OK
ENSBTAG00000038321	ENSBTAG00000038321	18:51216018-51227395	0.321481	0	0.547563	OK
ENSBTAG00000002764	ENSBTAG00000002764	18:46641557-46644180	0.321366	0	0.610337	OK
ENSBTAG00000038267	ENSBTAG00000038267	15:82660717-82661671	0.321153	0	0.651123	OK
ENSBTAG00000019963	ENSBTAG00000019963	12:90769073-90783057	0.32078	0	0.499793	OK
ENSBTAG00000015149	ENSBTAG00000015149	24:36277658-36279084	0.320766	0	0.728561	OK
ENSBTAG00000047539	ENSBTAG00000047539	7:10273564-10274491	0.320541	0	1.11681	OK

ENSBTAG00000011213	ENSBTAG00000011213	21:19223847-19283222	0.320451	0	0.655935	OK
ENSBTAG00000012182	ENSBTAG00000012182	3:77571198-77573843	0.320429	0	0.654829	OK
ENSBTAG00000016203	ENSBTAG00000016203	14:1445333-1456750	0.320392	0	0.53893	OK
ENSBTAG00000008895	ENSBTAG00000008895	4:99223159-99254538	0.320354	0	0.754031	OK
ENSBTAG00000034869	ENSBTAG00000034869	1:3972169-3973049	0.320015	0	0.705876	OK
ENSBTAG00000019072	ENSBTAG00000019072	11:46753577-46769867	0.31991	0	0.600165	OK
ENSBTAG00000032798	ENSBTAG00000032798	10:26948543-26949581	0.319711	0	1.19686	OK
ENSBTAG00000000608	ENSBTAG00000000608	3:14090126-14117838	0.319559	0	0.550683	OK
ENSBTAG00000010152	ENSBTAG00000010152	X:139791004-139928580	0.31946	0.0416697	0.666716	OK
ENSBTAG00000001606	ENSBTAG00000001606	25:2815161-2816100	0.319365	0	0.661524	OK
ENSBTAG00000038589	ENSBTAG00000038589	X:90997108-91000794	0.31926	0	0.900248	OK
ENSBTAG00000016081	ENSBTAG00000016081	2:47998613-47999416	0.318989	0	0.773563	OK
ENSBTAG00000005974	ENSBTAG00000005974	23:14985520-14998187	0.318541	0	0.552643	OK
ENSBTAG00000047666	ENSBTAG00000047666	28:45002033-45003218	0.318284	0	1.10254	OK
ENSBTAG00000001099	ENSBTAG00000001099	5:104114215-104117626	0.318206	0	0.591591	OK
ENSBTAG00000010634	ENSBTAG00000010634	6:4372936-4376146	0.318028	0	0.675773	OK
ENSBTAG00000039136	ENSBTAG00000039136	15:1198238-1199173	0.318002	0	1.10726	OK
ENSBTAG00000008491	ENSBTAG00000008491	23:32385305-32386038	0.317824	0	0.847436	OK
ENSBTAG00000017884	ENSBTAG00000017884	8:42030854-42044319	0.317659	0	0.608633	OK
ENSBTAG00000040543	ENSBTAG00000040543	9:69513252-69514728	0.317597	0	0.841695	OK
ENSBTAG00000010113	ENSBTAG00000010113	9:41168309-41176127	0.317591	0	0.741593	OK
ENSBTAG00000010859	ENSBTAG00000010859	29:29615507-29627607	0.317558	0	0.795353	OK
ENSBTAG00000039662	ENSBTAG00000039662	16:53131204-53133055	0.317514	0	0.894898	OK
ENSBTAG00000015811	ENSBTAG00000015811	17:18759163-18760093	0.317243	0	0.685619	OK
ENSBTAG00000026278	ENSBTAG00000026278	25:33988116-33989662	0.317114	0	0.535723	OK
ENSBTAG00000035641	ENSBTAG00000035641	X:118603965-118605128	0.316976	0	0.536417	OK
ENSBTAG00000046316	ENSBTAG00000046316	21:57341358-57341889	0.316871	0	1.16981	OK
ENSBTAG00000015681	ENSBTAG00000015681	28:39531570-39542179	0.316411	0	0.587396	OK
ENSBTAG00000020375	ENSBTAG00000020375	26:16130564-16159314	0.316286	0	0.67999	OK
ENSBTAG00000025068	ENSBTAG00000025068	10:27142658-27143630	0.315885	0	1.06511	OK

ENSBTAG00000007269	ENSBTAG00000007269	17:54297405-54308318	0.315854	0	0.706277	OK
ENSBTAG00000006695	ENSBTAG00000006695	14:32884898-32908633	0.315752	0.056342	0.56342	OK
ENSBTAG00000011600	ENSBTAG00000011600	13:35600312-35626614	0.315472	0	0.660206	OK
ENSBTAG00000035147	ENSBTAG00000035147	19:7830733-7860147	0.315407	0	0.794336	OK
ENSBTAG00000030675	ENSBTAG00000030675	11:93984122-93991685	0.315325	0	0.545748	OK
ENSBTAG00000011233	ENSBTAG00000011233	13:72984199-72985097	0.315099	0	0.691727	OK
ENSBTAG00000020441	ENSBTAG00000020441	21:33083548-33160047	0.314958	0.0573406	0.573406	OK
ENSBTAG00000022890	ENSBTAG00000022890	24:2558225-2590450	0.314844	0	0.611148	OK
ENSBTAG00000021721	ENSBTAG00000021721	23:27808495-27812710	0.314712	0	0.578102	OK
ENSBTAG00000027223	ENSBTAG00000027223	9:71557681-71558755	0.314661	0	0.963952	OK
ENSBTAG00000008466	ENSBTAG00000008466	10:26830646-26838482	0.314402	0	0.710722	OK
ENSBTAG00000032951	ENSBTAG00000032951	21:27054891-27108980	0.314351	0	0.543219	OK
ENSBTAG00000002582	ENSBTAG00000002582	11:4335800-4346724	0.314248	0.0695055	0.62555	OK
ENSBTAG00000009069	ENSBTAG00000009069	7:8094995-8095964	0.314221	0	0.641043	OK
ENSBTAG00000034918	ENSBTAG00000034918	26:11069933-11071316	0.313961	0	0.748579	OK
ENSBTAG00000012451	ENSBTAG00000012451	23:7186800-7193645	0.313942	0	0.652353	OK
ENSBTAG00000018834	ENSBTAG00000018834	18:53169568-53207223	0.313822	0	0.479616	OK
ENSBTAG00000034659	ENSBTAG00000034659	20:3903959-3968828	0.313712	0	0.658718	OK
ENSBTAG00000045760	ENSBTAG00000045760	10:21975579-21976545	0.313532	0	1.07172	OK
ENSBTAG00000023563	ENSBTAG00000023563	23:26916510-26927031	0.313439	0	0.579991	OK
ENSBTAG00000037575	ENSBTAG00000037575	3:11642692-11644109	0.313331	0	1.75845	OK
ENSBTAG00000011315	ENSBTAG00000011315	9:61238503-61240861	0.313265	0	0.774045	OK
ENSBTAG00000039253	ENSBTAG00000039253	15:49622593-49623553	0.313195	0	1.07842	OK
ENSBTAG00000002680	ENSBTAG00000002680	19:15792360-15810652	0.31302	0	0.485561	OK
ENSBTAG00000005018	ENSBTAG00000005018	5:113843750-113862537	0.312612	0	0.591591	OK
ENSBTAG00000005868	ENSBTAG00000005868	5:97348104-97385488	0.312461	0	0.603079	OK
ENSBTAG00000006296	ENSBTAG00000006296	X:34102419-34112798	0.312223	0.0476541	0.762465	OK
ENSBTAG00000006004	ENSBTAG00000006004	14:75577518-75651194	0.312217	0	0.944029	OK
ENSBTAG00000001740	ENSBTAG00000001740	21:20871464-20886141	0.311813	0	0.555708	OK
ENSBTAG00000046463	ENSBTAG00000046463	X:71676361-71677651	0.311598	0	0.802546	OK

ENSBTAG00000021204	ENSBTAG00000021204	4:52477240-52532254	0.311596	0	0.687897	OK
ENSBTAG00000010620	ENSBTAG00000010620	11:13463413-13490532	0.31158	0	0.565288	OK
ENSBTAG00000039486	ENSBTAG00000039486	20:20088694-20200206	0.311508	0	0.992286	OK
ENSBTAG00000000355	ENSBTAG00000000355	19:51763603-51765917	0.311362	0	0.686528	OK
ENSBTAG00000027637	ENSBTAG00000027637	15:50991934-50992888	0.311134	0	1.0852	OK
ENSBTAG00000017435	ENSBTAG00000017435	25:28461991-28472461	0.311111	0.0938183	0.516001	OK
ENSBTAG00000006078	ENSBTAG00000006078	13:17623241-17639994	0.311076	0	0.822743	OK
ENSBTAG00000003074	ENSBTAG00000003074	19:62486091-62496100	0.310942	0	0.545174	OK
ENSBTAG00000001965	ENSBTAG00000001965	19:53412855-53418596	0.310871	0	0.600165	OK
ENSBTAG00000040143	ENSBTAG00000040143	28:44819688-44820675	0.310839	0	0.629352	OK
ENSBTAG00000039855	ENSBTAG00000039855	22:21994944-22088653	0.310592	0	0.58601	OK
ENSBTAG00000016874	ENSBTAG00000016874	7:12366855-12370535	0.310567	0	0.555609	OK
ENSBTAG00000047566	ENSBTAG00000047566	10:27827278-27828217	0.310517	0	1.10254	OK
ENSBTAG00000008947	ENSBTAG00000008947	6:2146496-2149069	0.31034	0	0.643784	OK
ENSBTAG00000006605	ENSBTAG00000006605	5:57934909-57935872	0.310229	0	0.645037	OK
ENSBTAG00000008322	ENSBTAG00000008322	3:33033624-33035163	0.310137	0	0.80724	OK
ENSBTAG00000040305	ENSBTAG00000040305	7:8999055-9000628	0.30978	0	1.23912	OK
ENSBTAG00000003443	ENSBTAG00000003443	6:69952425-70003063	0.309771	0.0415027	0.581038	OK
ENSBTAG00000031658	ENSBTAG00000031658	7:20787832-20800309	0.309743	0	0.560696	OK
ENSBTAG00000037770	ENSBTAG00000037770	3:103607880-103608828	0.309417	0	1.09207	OK
ENSBTAG00000014088	ENSBTAG00000014088	X:16113421-16173828	0.309386	0	0.608991	OK
ENSBTAG00000009478	ENSBTAG00000009478	7:46064179-46067119	0.309337	0	0.694046	OK
ENSBTAG00000014024	ENSBTAG00000014024	12:31492829-31503972	0.309302	0	0.993079	OK
ENSBTAG00000040072	ENSBTAG00000040072	17:45107092-45116312	0.309217	0	0.900248	OK
ENSBTAG00000012688	ENSBTAG00000012688	20:38272476-38300277	0.309089	0	0.739489	OK
ENSBTAG00000003361	ENSBTAG00000003361	15:59585679-59800570	0.309001	0	0.682605	OK
ENSBTAG00000014599	ENSBTAG00000014599	6:69501919-69523156	0.30871	0	0.643534	OK
ENSBTAG00000001449	ENSBTAG00000001449	7:53408162-53414128	0.308636	0	0.689424	OK
ENSBTAG00000047874	ENSBTAG00000047874	3:34785791-34793589	0.308563	0	0.57918	OK
ENSBTAG00000026813	ENSBTAG00000026813	5:30080720-30084591	0.308525	0	0.556605	OK

ENSBTAG00000016048	ENSBTAG00000016048	5:120098391-120106718	0.308127	0.0697631	0.558105	OK
ENSBTAG00000013105	ENSBTAG00000013105	18:57171630-57183964	0.307873	0	0.546565	OK
ENSBTAG00000046579	ENSBTAG00000046579	11:7006511-7007310	0.307697	0	0.795353	OK
ENSBTAG00000012197	ENSBTAG00000012197	25:39736449-39742348	0.307544	0	0.553628	OK
ENSBTAG00000038318	ENSBTAG00000038318	2:1404183-1406607	0.307538	0	0.597937	OK
ENSBTAG00000032670	ENSBTAG00000032670	8:60634514-60635474	0.307477	0	1.07842	OK
ENSBTAG00000025953	ENSBTAG00000025953	12:9843842-9845040	0.307394	0	0.872186	OK
ENSBTAG00000038928	ENSBTAG00000038928	23:29540532-29541693	0.307366	0	1.10254	OK
ENSBTAG00000026953	ENSBTAG00000026953	21:57300673-57331304	0.307351	0	0.857182	OK
ENSBTAG00000039377	ENSBTAG00000039377	X:28706983-28709518	0.307326	0	0.571755	OK
ENSBTAG00000039425	ENSBTAG00000039425	5:27558901-27564151	0.307189	0	0.574626	OK
ENSBTAG00000005749	ENSBTAG00000005749	X:111801657-111805157	0.307155	0	1.00028	OK
ENSBTAG00000014862	ENSBTAG00000014862	16:11057882-11058607	0.307069	0	0.856787	OK
ENSBTAG00000026326	ENSBTAG00000026326	25:26765630-26769345	0.306986	0.0619375	0.557437	OK
ENSBTAG00000020643	ENSBTAG00000020643	29:33240587-33255647	0.306955	0	0.714811	OK
ENSBTAG00000012860	ENSBTAG00000012860	X:85450102-85453009	0.30664	0	0.611389	OK
ENSBTAG00000016295	ENSBTAG00000016295	8:77318254-77320448	0.306572	0	0.702841	OK
ENSBTAG00000038669	ENSBTAG00000038669	3:11320726-11321701	0.30648	0	1.06183	OK
ENSBTAG00000013763	ENSBTAG00000013763	26:37385336-37393063	0.306454	0	0.647053	OK
ENSBTAG00000040244	ENSBTAG00000040244	5:74974806-74986755	0.306177	0.0705475	1.19931	OK
ENSBTAG00000021130	ENSBTAG00000021130	6:107924383-107928256	0.306141	0	0.599875	OK
ENSBTAG00000015181	ENSBTAG00000015181	9:84028747-84136430	0.306015	0.0658369	0.526695	OK
ENSBTAG00000008504	ENSBTAG00000008504	25:1545799-1549999	0.306008	0	0.600165	OK
ENSBTAG00000033979	ENSBTAG00000033979	23:25966733-25980368	0.305689	0	0.775494	OK
ENSBTAG00000009159	ENSBTAG00000009159	22:60913458-60946157	0.305678	0	0.326245	OK
ENSBTAG00000016323	ENSBTAG00000016323	1:97650782-97762867	0.305656	0.0619189	0.557271	OK
ENSBTAG00000001432	ENSBTAG00000001432	15:84007597-84008311	0.305588	0	0.869987	OK
ENSBTAG00000032926	ENSBTAG00000032926	X:89987695-89988761	0.305429	0	0.591028	OK
ENSBTAG00000032508	ENSBTAG00000032508	20:63676556-63677447	0.30535	0	1.16194	OK
ENSBTAG00000012178	ENSBTAG00000012178	19:41040925-41048228	0.305172	0	0.915515	OK

ENSBTAG00000025445	ENSBTAG00000025445	7:22831358-22831913	0.305045	0	1.11923	OK
ENSBTAG00000013081	ENSBTAG00000013081	25:27955044-27977149	0.304965	0	0.774334	OK
ENSBTAG00000004290	ENSBTAG00000004290	18:35600676-35602509	0.304867	0	0.564803	OK
ENSBTAG00000046068	ENSBTAG00000046068	9:29703281-29703781	0.304623	0	1.24234	OK
ENSBTAG00000021245	ENSBTAG00000021245	17:34749101-34753222	0.304614	0	0.706679	OK
ENSBTAG00000020784	ENSBTAG00000020784	5:17207295-17208334	0.304387	0	0.996424	OK
ENSBTAG00000021685	ENSBTAG00000021685	13:54623916-54631595	0.304378	0	0.584246	OK
ENSBTAG00000020854	ENSBTAG00000020854	19:27447121-27451108	0.304331	0	0.558105	OK
ENSBTAG00000022381	ENSBTAG00000022381	11:10546063-10646706	0.304142	0.0411562	0.493875	OK
ENSBTAG00000021880	ENSBTAG00000021880	16:30656000-30703396	0.304122	0	0.578371	OK
ENSBTAG00000031544	ENSBTAG00000031544	5:56285007-56289214	0.304083	0	1.52041	OK
ENSBTAG00000013741	ENSBTAG00000013741	10:5215473-5217493	0.30372	0	0.551172	OK
ENSBTAG00000033803	ENSBTAG00000033803	9:28834077-28837863	0.303584	0	0.763109	OK
ENSBTAG00000011406	ENSBTAG00000011406	8:60207471-60212368	0.303571	0	0.567946	OK
ENSBTAG00000043959	ENSBTAG00000043959	17:43348701-43457620	0.303563	0	0.662229	OK
ENSBTAG00000047633	ENSBTAG00000047633	7:1446911-1450876	0.303517	0	0.471656	OK
ENSBTAG00000020399	ENSBTAG00000020399	14:17172452-17186547	0.303434	0	0.619004	OK
ENSBTAG00000018202	ENSBTAG00000018202	29:40551222-40562584	0.303165	0	0.554221	OK
ENSBTAG00000006573	ENSBTAG00000006573	22:52871074-52922306	0.303104	0.0445859	0.535031	OK
ENSBTAG00000015759	ENSBTAG00000015759	3:43137017-43137640	0.302787	0	0.997064	OK
ENSBTAG00000022360	ENSBTAG00000022360	5:86571272-87036285	0.30276	0.075075	0.6006	OK
ENSBTAG00000046864	ENSBTAG00000046864	18:58485272-58486123	0.302102	0	1.21655	OK
ENSBTAG00000047215	ENSBTAG00000047215	7:8160325-8161273	0.302064	0	0.655244	OK
ENSBTAG00000020939	ENSBTAG00000020939	28:35366024-35378318	0.301846	0	0.55911	OK
ENSBTAG00000047696	ENSBTAG00000047696	7:10938910-10939846	0.301809	0	1.10607	OK
ENSBTAG00000047415	ENSBTAG00000047415	7:43706042-43706999	0.301455	0	1.0818	OK
ENSBTAG00000019325	ENSBTAG00000019325	7:13572877-13581965	0.301328	0	0.591873	OK
ENSBTAG00000039069	ENSBTAG00000039069	8:22563336-22563918	0.301295	0	1.0673	OK
ENSBTAG00000047935	ENSBTAG00000047935	21:26181930-26182658	0.301286	0	1.04751	OK
ENSBTAG00000014538	ENSBTAG00000014538	3:106976316-106988248	0.30123	0.0448369	0.58288	OK

ENSBTAG00000001084	ENSBTAG00000001084	7:8270705-8271653	0.301044	0	0.655244	OK
ENSBTAG00000015775	ENSBTAG00000015775	21:30079542-30102510	0.300868	0	1.50434	OK
ENSBTAG00000011042	ENSBTAG00000011042	6:109631168-109635557	0.300864	0	0.595236	OK
ENSBTAG00000047721	ENSBTAG00000047721	15:80882783-80883710	0.300686	0	1.11681	OK
ENSBTAG00000039494	ENSBTAG00000039494	3:11340118-11341072	0.300616	0	1.0852	OK
ENSBTAG00000013726	ENSBTAG00000013726	16:70815222-70823936	0.30028	0	0.500138	OK
ENSBTAG00000018955	ENSBTAG00000018955	12:25231842-25324738	0.300064	0	0.736333	OK
ENSBTAG00000006112	ENSBTAG00000006112	28:3346616-3358272	0.300045	0	0.542602	OK
ENSBTAG00000011115	ENSBTAG00000011115	26:10976194-10978251	0.300006	0	0.503298	OK
ENSBTAG00000012562	ENSBTAG00000012562	15:30444351-30445653	0.299898	0	0.47709	OK
ENSBTAG00000004269	ENSBTAG00000004269	9:73305312-73310869	0.29978	0	0.703378	OK
ENSBTAG00000040485	ENSBTAG00000040485	7:44639890-44650050	0.299723	0	1.49861	OK
ENSBTAG00000026249	ENSBTAG00000026249	5:35174129-35174846	0.299604	0	0.945466	OK
ENSBTAG00000014488	ENSBTAG00000014488	24:11082495-11214011	0.299588	0	0.516996	OK
ENSBTAG00000047380	ENSBTAG00000047380	15:38041579-38044615	0.299414	0	0.79231	OK
ENSBTAG00000008688	ENSBTAG00000008688	3:107032467-107047976	0.299303	0.062897	0.503176	OK
ENSBTAG00000017115	ENSBTAG00000017115	1:82285912-82294596	0.299262	0	0.659417	OK
ENSBTAG00000004036	ENSBTAG00000004036	19:45154678-45186611	0.299167	0	0.549514	OK
ENSBTAG00000013533	ENSBTAG00000013533	23:27393341-27398934	0.29915	0	0.550196	OK
ENSBTAG00000039152	ENSBTAG00000039152	5:31775720-31776650	0.29885	0	1.11321	OK
ENSBTAG00000047246	ENSBTAG00000047246	15:47683170-47684118	0.298847	0	1.09207	OK
ENSBTAG00000017188	ENSBTAG00000017188	13:7018842-7051645	0.298555	0	0.67638	OK
ENSBTAG00000009006	ENSBTAG00000009006	17:72467983-72495691	0.29849	0.097853	0.489265	OK
ENSBTAG00000009813	ENSBTAG00000009813	3:103233546-103238303	0.298446	0	0.718948	OK
ENSBTAG00000011186	ENSBTAG00000011186	29:41162551-41172395	0.298431	0.0553184	0.497866	OK
ENSBTAG00000019930	ENSBTAG00000019930	29:18444586-18465087	0.298379	0	0.654415	OK
ENSBTAG00000001868	ENSBTAG00000001868	19:51592127-51599282	0.298366	0	0.564803	OK
ENSBTAG00000020731	ENSBTAG00000020731	15:64311997-64326851	0.298342	0	0.834907	OK
ENSBTAG00000046086	ENSBTAG00000046086	29:27869818-27870748	0.298192	0	1.11321	OK
ENSBTAG00000039384	ENSBTAG00000039384	6:26043474-26064216	0.29819	0	0.839647	OK

ENSBTAG00000009722	ENSBTAG00000009722	17:24458072-24459866	0.297935	0	1.06292	OK
ENSBTAG00000009841	ENSBTAG00000009841	24:4267544-4278276	0.297817	0	0.519461	OK
ENSBTAG00000002689	ENSBTAG00000002689	16:37593774-37838172	0.297333	0	1.48667	OK
ENSBTAG00000046190	ENSBTAG00000046190	7:53585215-53588056	0.297101	0	0.515708	OK
ENSBTAG00000037467	ENSBTAG00000037467	29:28077912-28078906	0.297056	0	1.11321	OK
ENSBTAG00000024934	ENSBTAG00000024934	19:50915618-50922806	0.297052	0	0.752934	OK
ENSBTAG00000012071	ENSBTAG00000012071	19:27529096-27532634	0.296772	0	0.522871	OK
ENSBTAG00000003950	ENSBTAG00000003950	2:5034438-5044966	0.296685	0	0.529896	OK
ENSBTAG00000021416	ENSBTAG00000021416	28:36008546-36026991	0.296685	0	0.674892	OK
ENSBTAG00000000841	ENSBTAG00000000841	8:67861050-67867745	0.296131	0	0.537531	OK
ENSBTAG00000006313	ENSBTAG00000006313	5:58849294-58850254	0.296115	0	1.07842	OK
ENSBTAG00000000527	ENSBTAG00000000527	23:11712801-12040961	0.296055	0	0.578776	OK
ENSBTAG00000015891	ENSBTAG00000015891	3:100351394-100374287	0.296036	0	0.704275	OK
ENSBTAG00000006720	ENSBTAG00000006720	3:21983347-21999397	0.295984	0	0.570143	OK
ENSBTAG00000015154	ENSBTAG00000015154	3:20172324-20176960	0.295934	0.0551564	0.496407	OK
ENSBTAG00000003866	ENSBTAG00000003866	2:126111730-126129768	0.295858	0	0.576225	OK
ENSBTAG00000008190	ENSBTAG00000008190	25:27995065-28005720	0.295619	0	0.886857	OK
ENSBTAG00000005667	ENSBTAG00000005667	8:58234603-58240521	0.295004	0.0472733	0.756372	OK
ENSBTAG00000017652	ENSBTAG00000017652	5:116316366-116335303	0.294967	0	0.519461	OK
ENSBTAG00000015591	ENSBTAG00000015591	7:1334565-1346051	0.294903	0	0.884709	OK
ENSBTAG00000013255	ENSBTAG00000013255	10:27849078-27850059	0.294678	0	1.05534	OK
ENSBTAG00000004218	ENSBTAG00000004218	10:86105103-86110121	0.294595	0	0.535954	OK
ENSBTAG00000010376	ENSBTAG00000010376	17:10643736-10672839	0.294594	0	0.626812	OK
ENSBTAG00000006588	ENSBTAG00000006588	5:110806462-110815850	0.294316	0	0.627445	OK
ENSBTAG00000040242	ENSBTAG00000040242	17:30095168-30095748	0.294249	0	1.07098	OK
ENSBTAG00000046256	ENSBTAG00000046256	17:49445323-49739129	0.29404	0	0.567798	OK
ENSBTAG00000038682	ENSBTAG00000038682	14:2227349-2232020	0.294035	0	0.408742	OK
ENSBTAG00000045678	ENSBTAG00000045678	X:78134339-78135016	0.29399	0	0.917535	OK
ENSBTAG00000017971	ENSBTAG00000017971	23:14309127-14351087	0.293509	0	0.525525	OK
ENSBTAG00000046239	ENSBTAG00000046239	26:18116430-18120174	0.292933	0.0553037	0.497733	OK

ENSBTAG00000008793	ENSBTAG00000008793	10:26388179-26389761	0.292879	0	0.733378	OK
ENSBTAG00000013109	ENSBTAG00000013109	15:51845121-52049569	0.292874	0.0516352	0.516352	OK
ENSBTAG00000038737	ENSBTAG00000038737	15:53559421-53564982	0.292571	0	0.503788	OK
ENSBTAG00000016738	ENSBTAG00000016738	17:63358581-63396162	0.292401	0	0.540619	OK
ENSBTAG00000021508	ENSBTAG00000021508	22:32534125-32550296	0.292242	0	0.750546	OK
ENSBTAG00000048043	ENSBTAG00000048043	29:27234897-27235833	0.291173	0	0.663644	OK
ENSBTAG00000002116	ENSBTAG00000002116	13:61254417-61257121	0.291053	0	0.53602	OK
ENSBTAG00000045982	ENSBTAG00000045982	5:60120923-60121865	0.290805	0	1.09903	OK
ENSBTAG00000006472	ENSBTAG00000006472	X:143861797-143891357	0.290706	0	0.525192	OK
ENSBTAG00000006587	ENSBTAG00000006587	8:84514065-84537863	0.290569	0	0.43837	OK
ENSBTAG00000039225	ENSBTAG00000039225	11:93459895-93460798	0.290564	0	1.14649	OK
ENSBTAG00000010317	ENSBTAG00000010317	19:46492709-46494782	0.290534	0	0.499414	OK
ENSBTAG00000038980	ENSBTAG00000038980	7:8215663-8216614	0.290477	0	0.653177	OK
ENSBTAG00000009117	ENSBTAG00000009117	13:26122535-26124764	0.290411	0	0.743138	OK
ENSBTAG00000010875	ENSBTAG00000010875	6:106061462-106065759	0.29036	0	0.545174	OK
ENSBTAG00000017155	ENSBTAG00000017155	8:107645158-107656813	0.290051	0	0.530915	OK
ENSBTAG00000011476	ENSBTAG00000011476	4:69331791-69334928	0.289975	0.0660048	0.528038	OK
ENSBTAG00000007094	ENSBTAG00000007094	21:26109823-26119769	0.289687	0.0683131	0.546505	OK
ENSBTAG00000010265	ENSBTAG00000010265	3:3296281-3319165	0.28967	0	0.719781	OK
ENSBTAG00000011780	ENSBTAG00000011780	X:26731210-26733724	0.28917	0	0.658892	OK
ENSBTAG00000030393	ENSBTAG00000030393	18:62787902-62804872	0.288971	0.0706438	0.56515	OK
ENSBTAG00000023885	ENSBTAG00000023885	11:98174565-98189943	0.288905	0	0.983633	OK
ENSBTAG00000007352	ENSBTAG00000007352	3:33302948-33325104	0.288396	0	0.549223	OK
ENSBTAG00000004115	ENSBTAG00000004115	23:40770314-40790973	0.288265	0.0704755	0.563804	OK
ENSBTAG00000012518	ENSBTAG00000012518	7:54624664-54639999	0.288209	0	0.484029	OK
ENSBTAG00000019352	ENSBTAG00000019352	28:2710493-2711438	0.288205	0	1.09554	OK
ENSBTAG00000030974	ENSBTAG00000030974	2:107921713-107925511	0.287984	0	0.56342	OK
ENSBTAG00000008259	ENSBTAG00000008259	12:52417588-52424159	0.287957	0	0.734245	OK
ENSBTAG00000020107	ENSBTAG00000020107	25:2992451-2995606	0.287859	0	0.903638	OK
ENSBTAG00000009510	ENSBTAG00000009510	11:2221055-2222399	0.287726	0	0.462181	OK

ENSBTAG00000045590	ENSBTAG00000045590	1:36089412-36090222	0.287709	0	1.27813	OK
ENSBTAG00000004652	ENSBTAG00000004652	7:3624278-3627734	0.287682	0	0.557204	OK
ENSBTAG00000046630	ENSBTAG00000046630	16:56114382-56118319	0.287398	0	0.697161	OK
ENSBTAG00000032781	ENSBTAG00000032781	10:27354030-27354967	0.287249	0	1.10489	OK
ENSBTAG00000005454	ENSBTAG00000005454	27:28320660-28398005	0.287165	0.0539492	0.485543	OK
ENSBTAG00000010598	ENSBTAG00000010598	2:120995643-121025344	0.287029	0	0.595562	OK
ENSBTAG00000008683	ENSBTAG00000008683	29:26543860-26554318	0.286835	0	0.764893	OK
ENSBTAG00000012104	ENSBTAG00000012104	18:53834506-53841046	0.28668	0	0.46819	OK
ENSBTAG00000015919	ENSBTAG00000015919	6:91597121-91723390	0.286447	0	0.462181	OK
ENSBTAG00000019379	ENSBTAG00000019379	19:22167562-22186258	0.286042	0.0595505	0.476404	OK
ENSBTAG00000018207	ENSBTAG00000018207	5:101439876-101452407	0.286038	0	0.513152	OK
ENSBTAG00000037602	ENSBTAG00000037602	7:253489-265612	0.286018	0	0.695289	OK
ENSBTAG00000013705	ENSBTAG00000013705	23:17879934-17887291	0.28579	0	0.552741	OK
ENSBTAG00000013947	ENSBTAG00000013947	8:76066457-76082190	0.28548	0	0.999179	OK
ENSBTAG00000013213	ENSBTAG00000013213	14:67533221-67540870	0.285421	0	0.473003	OK
ENSBTAG00000015983	ENSBTAG00000015983	10:26902107-26903076	0.2854	0	1.06841	OK
ENSBTAG00000046242	ENSBTAG00000046242	3:22310553-22311489	0.285341	0	1.10607	OK
ENSBTAG00000004013	ENSBTAG00000004013	10:61005652-61068184	0.285328	0	0.97853	OK
ENSBTAG00000008314	ENSBTAG00000008314	2:136125303-136154362	0.285248	0	0.641705	OK
ENSBTAG00000025219	ENSBTAG00000025219	19:15183250-15184294	0.285209	0	0.991652	OK
ENSBTAG00000010591	ENSBTAG00000010591	28:18549462-18572813	0.28502	0	0.509993	OK
ENSBTAG00000012273	ENSBTAG00000012273	1:83457840-83464244	0.284984	0	0.488918	OK
ENSBTAG00000047054	ENSBTAG00000047054	26:47635450-47651856	0.284843	0	0.575691	OK
ENSBTAG00000008989	ENSBTAG00000008989	5:97814580-97911805	0.284748	0	0.459106	OK
ENSBTAG00000039893	ENSBTAG00000039893	15:82635705-82636650	0.284697	0	0.657324	OK
ENSBTAG00000000634	ENSBTAG00000000634	3:117584072-117637812	0.284659	0	0.547126	OK
ENSBTAG00000044135	ENSBTAG00000044135	8:59755656-59762312	0.284633	0.0590747	0.472598	OK
ENSBTAG00000038674	ENSBTAG00000038674	18:60330958-60344819	0.284423	0	0.643034	OK
ENSBTAG00000010188	ENSBTAG00000010188	7:15815878-15822349	0.284334	0	0.573565	OK
ENSBTAG00000011514	ENSBTAG00000011514	2:112851019-112858967	0.28401	0	0.744809	OK

ENSBTAG00000040111	ENSBTAG00000040111	19:57097871-57105114	0.283878	0	0.475337	OK
ENSBTAG00000014728	ENSBTAG00000014728	5:104292853-104300410	0.283629	0	0.850886	OK
ENSBTAG00000003237	ENSBTAG00000003237	22:59460405-59549010	0.283518	0	0.41542	OK
ENSBTAG00000025099	ENSBTAG00000025099	7:72843639-72906344	0.283413	0.0559613	0.503652	OK
ENSBTAG00000022954	ENSBTAG00000022954	3:2630818-2631902	0.283254	0	0.61808	OK
ENSBTAG00000000977	ENSBTAG00000000977	15:26101410-26451509	0.282981	0	0.495352	OK
ENSBTAG00000048087	ENSBTAG00000048087	13:53816738-53830302	0.28296	0	0.748579	OK
ENSBTAG00000019066	ENSBTAG00000019066	8:7734138-7765335	0.282674	0.051239	0.51239	OK
ENSBTAG00000002242	ENSBTAG00000002242	18:25254471-25294908	0.282168	0	0.683356	OK
ENSBTAG00000012969	ENSBTAG00000012969	6:19381412-19511148	0.281925	0	0.493228	OK
ENSBTAG00000039823	ENSBTAG00000039823	12:11441774-11443817	0.281901	0	0.506747	OK
ENSBTAG00000010245	ENSBTAG00000010245	X:38260923-38261790	0.281797	0	0.71646	OK
ENSBTAG00000046665	ENSBTAG00000046665	29:27716011-27718456	0.281731	0	1.0818	OK
ENSBTAG00000008410	ENSBTAG00000008410	13:38575830-38604712	0.281242	0	1.12682	OK
ENSBTAG00000002080	ENSBTAG00000002080	14:47005559-47013935	0.281234	0	0.703976	OK
ENSBTAG00000018221	ENSBTAG00000018221	19:24668410-24669376	0.281187	0	0.643034	OK
ENSBTAG00000030910	ENSBTAG00000030910	16:77741102-77779745	0.281121	0	0.869257	OK
ENSBTAG00000017495	ENSBTAG00000017495	29:27693139-27694078	0.280905	0	1.10254	OK
ENSBTAG00000016629	ENSBTAG00000016629	25:3242403-3344129	0.28088	0.0509365	0.509365	OK
ENSBTAG00000037603	ENSBTAG00000037603	15:46603201-46604152	0.280486	0	1.08863	OK
ENSBTAG00000008075	ENSBTAG00000008075	15:30117754-30121430	0.280448	0	0.487958	OK
ENSBTAG00000038186	ENSBTAG00000038186	7:97531725-97541600	0.280328	0	0.71646	OK
ENSBTAG00000039257	ENSBTAG00000039257	1:69694-71121	0.280158	0	1.08863	OK
ENSBTAG00000012458	ENSBTAG00000012458	6:118651434-118653913	0.280099	0	0.501146	OK
ENSBTAG00000048278	ENSBTAG00000048278	19:9718925-9721230	0.280062	0	0.622915	OK
ENSBTAG00000019741	ENSBTAG00000019741	5:102025880-102035208	0.280058	0.0601735	0.782255	OK
ENSBTAG00000046218	ENSBTAG00000046218	11:87525613-87536326	0.280051	0.0506995	0.506995	OK
ENSBTAG00000012352	ENSBTAG00000012352	10:12151280-12176608	0.280015	0	0.505017	OK
ENSBTAG00000018625	ENSBTAG00000018625	25:2733864-2740470	0.279964	0.0658369	0.526695	OK
ENSBTAG00000031718	ENSBTAG00000031718	13:55043086-55050690	0.279814	0	0.532007	OK

ENSBTAG00000033140	ENSBTAG00000033140	27:39139084-39139864	0.279759	0	0.796373	OK
ENSBTAG00000006597	ENSBTAG00000006597	14:1288667-1289627	0.279669	0	1.10372	OK
ENSBTAG00000038888	ENSBTAG00000038888	23:7568875-7571700	0.279663	0	0.483401	OK
ENSBTAG00000046750	ENSBTAG00000046750	7:5271364-5291295	0.27946	0	0.473165	OK
ENSBTAG00000017173	ENSBTAG00000017173	22:58782200-58783528	0.279385	0	1.06183	OK
ENSBTAG00000013263	ENSBTAG00000013263	4:69401000-69402503	0.279359	0	0.619931	OK
ENSBTAG00000014096	ENSBTAG00000014096	10:72577419-72602467	0.279324	0	0.6617	OK
ENSBTAG00000001468	ENSBTAG00000001468	10:67430990-67516426	0.279291	0	0.481528	OK
ENSBTAG00000002842	ENSBTAG00000002842	10:85310598-85350659	0.279273	0	0.62304	OK
ENSBTAG00000038649	ENSBTAG00000038649	4:107659927-107661137	0.279223	0	0.700305	OK
ENSBTAG00000045624	ENSBTAG00000045624	9:49837662-49839034	0.279137	0	0.993555	OK
ENSBTAG00000004860	ENSBTAG00000004860	7:26237928-26329594	0.278769	0	0.721139	OK
ENSBTAG00000014572	ENSBTAG00000014572	15:82916358-82917300	0.278667	0	0.659417	OK
ENSBTAG00000033423	ENSBTAG00000033423	3:15567317-15576042	0.278644	0	0.474901	OK
ENSBTAG00000000593	ENSBTAG00000000593	5:88287351-88453869	0.278612	0	0.579991	OK
ENSBTAG00000011287	ENSBTAG00000011287	22:37587793-37675105	0.278601	0	1.59579	OK
ENSBTAG00000047739	ENSBTAG00000047739	12:14722212-14725630	0.278576	0	0.560522	OK
ENSBTAG00000046746	ENSBTAG00000046746	19:43279034-43283109	0.278468	0	0.489265	OK
ENSBTAG00000018566	ENSBTAG00000018566	26:18782563-18787376	0.278443	0	0.490076	OK
ENSBTAG00000011373	ENSBTAG00000011373	17:18144414-18325277	0.27837	0	0.51894	OK
ENSBTAG00000016928	ENSBTAG00000016928	26:21713121-21730353	0.278173	0	0.463993	OK
ENSBTAG00000046652	ENSBTAG00000046652	19:24677021-24677969	0.278146	0	0.655244	OK
ENSBTAG00000005994	ENSBTAG00000005994	10:15743108-15770306	0.278054	0	0.770875	OK
ENSBTAG00000003743	ENSBTAG00000003743	9:57516042-57517306	0.277932	0	0.695573	OK
ENSBTAG00000027490	ENSBTAG00000027490	15:83981763-83982711	0.277921	0	0.655244	OK
ENSBTAG00000005143	ENSBTAG00000005143	3:102575256-102576762	0.277887	0	0.747498	OK
ENSBTAG00000046745	ENSBTAG00000046745	29:26921866-26922802	0.277443	0	0.663644	OK
ENSBTAG00000014634	ENSBTAG00000014634	X:47028834-47029865	0.277328	0	0.602494	OK
ENSBTAG00000021458	ENSBTAG00000021458	4:14276356-14281435	0.277236	0	0.559613	OK
ENSBTAG00000007031	ENSBTAG00000007031	27:39861722-39865112	0.277127	0	0.539211	OK

ENSBTAG00000005793	ENSBTAG00000005793	3:9578374-9588605	0.276949	0	0.489496	OK
ENSBTAG00000035844	ENSBTAG00000035844	1:74777084-74793631	0.276869	0	0.757618	OK
ENSBTAG00000047647	ENSBTAG00000047647	8:66161957-66162749	0.276809	0	0.784307	OK
ENSBTAG00000048049	ENSBTAG00000048049	21:71488885-71492946	0.27678	0	0.465297	OK
ENSBTAG00000015543	ENSBTAG00000015543	18:54425389-54497692	0.276772	0.0791653	0.435409	OK
ENSBTAG00000018258	ENSBTAG00000018258	19:55666049-55669890	0.276485	0	0.55297	OK
ENSBTAG00000046167	ENSBTAG00000046167	15:80827950-80829405	0.276471	0	1.09554	OK
ENSBTAG00000047974	ENSBTAG00000047974	10:32531489-32537714	0.276321	0	0.6437	OK
ENSBTAG00000005685	ENSBTAG00000005685	18:35159765-35165470	0.27629	0	0.443852	OK
ENSBTAG00000046459	ENSBTAG00000046459	29:26987136-26988069	0.276156	0	0.665778	OK
ENSBTAG00000045704	ENSBTAG00000045704	1:64357121-64488105	0.276056	0	0.540337	OK
ENSBTAG00000009178	ENSBTAG00000009178	7:17706869-17715987	0.276034	0	0.443377	OK
ENSBTAG00000009022	ENSBTAG00000009022	25:34084147-34085617	0.275964	0	0.422565	OK
ENSBTAG00000017647	ENSBTAG00000017647	15:48731977-48733880	0.275855	0	0.544028	OK
ENSBTAG00000012648	ENSBTAG00000012648	3:101566201-101614105	0.275778	0	0.735408	OK
ENSBTAG00000004863	ENSBTAG00000004863	15:44962851-45024904	0.275771	0	0.56151	OK
ENSBTAG00000005535	ENSBTAG00000005535	18:18468647-18470478	0.275713	0	0.573565	OK
ENSBTAG00000046135	ENSBTAG00000046135	14:69160053-69161571	0.27543	0	0.831554	OK
ENSBTAG00000038497	ENSBTAG00000038497	X:4355492-4387239	0.275362	0	0.645439	OK
ENSBTAG00000038414	ENSBTAG00000038414	9:71780662-71784702	0.275154	0	1.01499	OK
ENSBTAG00000018340	ENSBTAG00000018340	8:15978602-15979256	0.275079	0	0.949803	OK
ENSBTAG00000038064	ENSBTAG00000038064	2:11700813-11701755	0.274997	0	0.659417	OK
ENSBTAG00000014770	ENSBTAG00000014770	X:14260384-14261392	0.27497	0	0.616241	OK
ENSBTAG00000006947	ENSBTAG00000006947	1:68954524-68989623	0.274866	0	0.606612	OK
ENSBTAG00000048170	ENSBTAG00000048170	7:53650654-53653078	0.274757	0	0.512517	OK
ENSBTAG00000039223	ENSBTAG00000039223	11:95846029-95850595	0.274684	0	0.859871	OK
ENSBTAG00000000390	ENSBTAG00000000390	25:28339034-28445471	0.274523	0	0.632557	OK
ENSBTAG00000007497	ENSBTAG00000007497	13:54519581-54525754	0.274191	0	0.516674	OK
ENSBTAG00000019593	ENSBTAG00000019593	25:17411488-17424291	0.273971	0	0.51894	OK
ENSBTAG00000047681	ENSBTAG00000047681	2:75406353-75407601	0.273806	0	0.61808	OK

ENSBTAG00000011184	ENSBTAG00000011184	29:41172508-41175112	0.2737	0	0.719781	OK
ENSBTAG00000038564	ENSBTAG00000038564	7:43333925-43334888	0.273635	0	1.07506	OK
ENSBTAG00000023851	ENSBTAG00000023851	11:98621302-98654751	0.273403	0	0.482932	OK
ENSBTAG00000000811	ENSBTAG00000000811	7:4216054-4223818	0.272999	0	0.480132	OK
ENSBTAG00000000008	ENSBTAG00000000008	29:32757308-32787597	0.272766	0	0.685165	OK
ENSBTAG00000047726	ENSBTAG00000047726	5:113269490-113270377	0.272566	0	0.704275	OK
ENSBTAG00000033304	ENSBTAG00000033304	27:34893818-34895119	0.272341	0	0.795761	OK
ENSBTAG00000001994	ENSBTAG00000001994	18:46125198-46130449	0.272296	0	0.942882	OK
ENSBTAG00000004903	ENSBTAG00000004903	19:42356579-42361365	0.27223	0	0.485764	OK
ENSBTAG00000047699	ENSBTAG00000047699	X:130065241-130067460	0.272208	0	0.572244	OK
ENSBTAG00000048004	ENSBTAG00000048004	22:53923880-53924433	0.271959	0	1.12327	OK
ENSBTAG00000047538	ENSBTAG00000047538	17:74154045-74158785	0.27189	0	0.321019	OK
ENSBTAG00000015789	ENSBTAG00000015789	3:14223568-14241533	0.271826	0	0.513152	OK
ENSBTAG00000002224	ENSBTAG00000002224	7:20436669-20469912	0.271718	0.0530643	0.477579	OK
ENSBTAG00000032668	ENSBTAG00000032668	8:60562018-60563035	0.271624	0	0.643034	OK
ENSBTAG00000003634	ENSBTAG00000003634	26:742616-760794	0.271329	0	0.827125	OK
ENSBTAG00000000242	ENSBTAG00000000242	1:58243448-58244153	0.271328	0	0.881093	OK
ENSBTAG00000004590	ENSBTAG00000004590	3:104140102-104159895	0.271135	0	0.806715	OK
ENSBTAG00000017376	ENSBTAG00000017376	2:38796688-38838127	0.27113	0	0.660118	OK
ENSBTAG00000012624	ENSBTAG00000012624	5:39422869-39442801	0.27097	0	0.648066	OK
ENSBTAG00000027051	ENSBTAG00000027051	2:125784496-125811602	0.270947	0.0606139	0.484911	OK
ENSBTAG00000047557	ENSBTAG00000047557	29:27059115-27061111	0.270549	0	0.637098	OK
ENSBTAG00000043563	ENSBTAG00000043563	MT:12108-13929	0.270408	0	1.02335	OK
ENSBTAG00000008139	ENSBTAG00000008139	4:69357160-69389905	0.26988	0	0.495945	OK
ENSBTAG00000047753	ENSBTAG00000047753	29:27880231-27883328	0.269644	0	1.00318	OK
ENSBTAG00000046370	ENSBTAG00000046370	10:27726091-27727613	0.269393	0	1.13146	OK
ENSBTAG00000033731	ENSBTAG00000033731	29:12820129-12908442	0.269277	0	0.594422	OK
ENSBTAG00000015453	ENSBTAG00000015453	7:53986018-53988346	0.269261	0	0.533652	OK
ENSBTAG00000047846	ENSBTAG00000047846	7:42171047-42171970	0.269228	0	0.672991	OK
ENSBTAG00000018363	ENSBTAG00000018363	4:9723790-9730052	0.269056	0	0.774914	OK

ENSBTAG00000004924	ENSBTAG00000004924	14:25218585-25222991	0.269013	0	0.483401	OK
ENSBTAG00000023179	ENSBTAG00000023179	14:16353387-16361545	0.268873	0.0593967	0.475174	OK
ENSBTAG00000013673	ENSBTAG00000013673	4:108113407-108114340	0.268546	0	1.10963	OK
ENSBTAG00000030255	ENSBTAG00000030255	5:110000367-110006138	0.268545	0	0.53146	OK
ENSBTAG00000012854	ENSBTAG00000012854	19:40894256-40905176	0.268448	0	0.756234	OK
ENSBTAG00000043954	ENSBTAG00000043954	9:80611656-80619872	0.268437	0	0.738961	OK
ENSBTAG00000009287	ENSBTAG00000009287	X:91735332-91739378	0.26843	0	0.617466	OK
ENSBTAG00000039594	ENSBTAG00000039594	4:113495069-113525384	0.268292	0	0.531369	OK
ENSBTAG00000039683	ENSBTAG00000039683	23:29122353-29123280	0.26826	0	1.11681	OK
ENSBTAG00000003407	ENSBTAG00000003407	11:75039680-75047800	0.268237	0	0.804625	OK
ENSBTAG00000001408	ENSBTAG00000001408	11:66679942-66704192	0.268222	0	0.610427	OK
ENSBTAG00000035349	ENSBTAG00000035349	1:83322-84281	0.26821	0	1.09903	OK
ENSBTAG00000048125	ENSBTAG00000048125	11:106183597-106189094	0.267837	0	0.43868	OK
ENSBTAG00000048096	ENSBTAG00000048096	21:20684173-20685028	0.267806	0	0.726516	OK
ENSBTAG00000006216	ENSBTAG00000006216	7:8329685-8330618	0.267755	0	0.665778	OK
ENSBTAG00000030683	ENSBTAG00000030683	5:72049627-72052680	0.267653	0	0.677395	OK
ENSBTAG00000001726	ENSBTAG00000001726	X:82550389-82559474	0.267623	0	0.726516	OK
ENSBTAG00000025297	ENSBTAG00000025297	X:19576178-19585346	0.267617	0	0.672991	OK
ENSBTAG00000012216	ENSBTAG00000012216	18:2160855-2177621	0.267524	0	0.709585	OK
ENSBTAG00000037580	ENSBTAG00000037580	15:72466674-72475397	0.267321	0	1.33661	OK
ENSBTAG00000038606	ENSBTAG00000038606	6:107575517-107576939	0.267198	0	0.436829	OK
ENSBTAG00000017316	ENSBTAG00000017316	3:22880134-22899870	0.267102	0	0.632042	OK
ENSBTAG00000006729	ENSBTAG00000006729	28:18003735-18191994	0.267007	0.0608991	0.487193	OK
ENSBTAG00000007564	ENSBTAG00000007564	17:35334853-35336980	0.266875	0	0.584082	OK
ENSBTAG00000016912	ENSBTAG00000016912	5:56003066-56011109	0.266832	0	0.508323	OK
ENSBTAG00000047167	ENSBTAG00000047167	11:54764950-55025216	0.266658	0	0.587673	OK
ENSBTAG00000009391	ENSBTAG00000009391	1:64933573-64937122	0.266541	0	0.584907	OK
ENSBTAG00000009049	ENSBTAG00000009049	5:57758049-57762409	0.266439	0	0.45507	OK
ENSBTAG00000032623	ENSBTAG00000032623	11:24909397-24962486	0.266423	0	0.51894	OK
ENSBTAG00000026266	ENSBTAG00000026266	24:37834592-37910130	0.266412	0	0.647728	OK

ENSBTAG00000020570	ENSBTAG00000020570	6:96650284-96657180	0.266404	0	0.50899	OK
ENSBTAG00000011204	ENSBTAG00000011204	8:85936414-85947675	0.266349	0	0.552152	OK
ENSBTAG00000046520	ENSBTAG00000046520	13:55002989-55019058	0.266242	0	0.610787	OK
ENSBTAG00000046474	ENSBTAG00000046474	7:42913831-42914770	0.266225	0	1.10254	OK
ENSBTAG00000008937	ENSBTAG00000008937	13:12934867-13000042	0.266171	0	0.43899	OK
ENSBTAG00000035083	ENSBTAG00000035083	5:4048887-4052625	0.265948	0.0553924	0.498532	OK
ENSBTAG00000012996	ENSBTAG00000012996	3:95599736-95601983	0.265839	0	0.77841	OK
ENSBTAG00000018161	ENSBTAG00000018161	9:65579317-65608088	0.265761	0	0.511462	OK
ENSBTAG00000045588	ENSBTAG00000045588	7:5655108-5659111	0.265536	0	0.50338	OK
ENSBTAG00000034586	ENSBTAG00000034586	11:1821485-1846701	0.265505	0	0.60602	OK
ENSBTAG00000045528	ENSBTAG00000045528	11:93765537-93766494	0.265437	0	0.649081	OK
ENSBTAG00000027425	ENSBTAG00000027425	3:6560554-6569286	0.265268	0	0.73993	OK
ENSBTAG00000005694	ENSBTAG00000005694	10:86770120-86802293	0.265238	0	0.78312	OK
ENSBTAG00000012973	ENSBTAG00000012973	23:4980259-5004773	0.265126	0	0.637098	OK
ENSBTAG00000034332	ENSBTAG00000034332	3:106190339-106191986	0.265079	0	0.557604	OK
ENSBTAG00000009453	ENSBTAG00000009453	22:220367-251230	0.264972	0	0.706593	OK
ENSBTAG00000026400	ENSBTAG00000026400	25:3373124-3374097	0.264957	0	0.638408	OK
ENSBTAG00000002701	ENSBTAG00000002701	20:38182069-38206820	0.264896	0	0.73993	OK
ENSBTAG00000013283	ENSBTAG00000013283	18:51304729-51307867	0.264891	0	0.484061	OK
ENSBTAG00000010752	ENSBTAG00000010752	29:27015864-27016853	0.264889	0	0.62808	OK
ENSBTAG00000010008	ENSBTAG00000010008	15:24209209-24254092	0.264855	0	0.484684	OK
ENSBTAG00000002545	ENSBTAG00000002545	11:47884973-47885885	0.264648	0	0.681108	OK
ENSBTAG00000039654	ENSBTAG00000039654	15:50679503-50680444	0.264618	0	1.1002	OK
ENSBTAG00000019958	ENSBTAG00000019958	8:91124598-91132281	0.264536	0	0.511568	OK
ENSBTAG00000001042	ENSBTAG00000001042	11:68232861-68255606	0.264533	0	0.562146	OK
ENSBTAG00000025148	ENSBTAG00000025148	7:15511710-15518457	0.26452	0	0.449635	OK
ENSBTAG00000044071	ENSBTAG00000044071	10:5409680-5410760	0.264267	0	0.575158	OK
ENSBTAG00000038597	ENSBTAG00000038597	23:30607404-30608346	0.264235	0	0.659417	OK
ENSBTAG00000038263	ENSBTAG00000038263	3:11993522-11997024	0.264227	0	0.912706	OK
ENSBTAG00000006423	ENSBTAG00000006423	5:113079626-113088634	0.264076	0	0.687136	OK

ENSBTAG00000008718	ENSBTAG00000008718	25:29391799-29824281	0.263956	0	0.515352	OK
ENSBTAG00000014372	ENSBTAG00000014372	4:114615443-114680160	0.263643	0	0.495055	OK
ENSBTAG00000032340	ENSBTAG00000032340	10:36117897-36127741	0.26358	0	0.917137	OK
ENSBTAG00000031673	ENSBTAG00000031673	7:20473897-20486894	0.263192	0	0.588788	OK
ENSBTAG00000016412	ENSBTAG00000016412	8:22114511-22119419	0.26299	0	0.46582	OK
ENSBTAG00000017404	ENSBTAG00000017404	17:71551241-71572738	0.262976	0	0.422853	OK
ENSBTAG00000001662	ENSBTAG00000001662	11:68632155-68661110	0.262786	0.0608097	0.486477	OK
ENSBTAG00000027694	ENSBTAG00000027694	2:19257061-19259992	0.262663	0	0.56515	OK
ENSBTAG00000016121	ENSBTAG00000016121	5:27630614-27640849	0.262577	0	0.483778	OK
ENSBTAG00000003268	ENSBTAG00000003268	20:26278616-26280571	0.2624	0	0.500138	OK
ENSBTAG00000019739	ENSBTAG00000019739	7:57828457-57829687	0.26238	0	0.507078	OK
ENSBTAG00000006703	ENSBTAG00000006703	10:45205472-45212956	0.262282	0	0.53607	OK
ENSBTAG00000006534	ENSBTAG00000006534	1:58152816-58166584	0.26222	0	0.635144	OK
ENSBTAG00000007184	ENSBTAG00000007184	18:4990462-5003016	0.262116	0.0469837	0.469837	OK
ENSBTAG00000026753	ENSBTAG00000026753	2:106936886-106940905	0.262104	0	0.575158	OK
ENSBTAG00000032436	ENSBTAG00000032436	17:53142510-53143425	0.262003	0	0.678875	OK
ENSBTAG00000015470	ENSBTAG00000015470	3:34082542-34095369	0.261976	0	0.48265	OK
ENSBTAG00000003113	ENSBTAG00000003113	24:25452968-25496186	0.261947	0	0.711536	OK
ENSBTAG00000046009	ENSBTAG00000046009	10:27716034-27717346	0.26136	0	1.0852	OK
ENSBTAG00000048079	ENSBTAG00000048079	13:37375144-37377820	0.261243	0	0.570405	OK
ENSBTAG00000002362	ENSBTAG00000002362	5:97520909-97524789	0.26121	0.0533652	0.426922	OK
ENSBTAG00000047412	ENSBTAG00000047412	16:41541498-41552708	0.261188	0	0.718324	OK
ENSBTAG00000004852	ENSBTAG00000004852	3:20519725-20528784	0.261181	0	0.436522	OK
ENSBTAG00000027665	ENSBTAG00000027665	27:32799189-32803514	0.261155	0	0.440313	OK
ENSBTAG00000021586	ENSBTAG00000021586	8:45177228-45178867	0.261126	0	0.631656	OK
ENSBTAG00000004875	ENSBTAG00000004875	25:2867519-2884577	0.261058	0	0.443693	OK
ENSBTAG00000019651	ENSBTAG00000019651	7:41775016-41780369	0.260834	0	0.763344	OK
ENSBTAG00000025088	ENSBTAG00000025088	19:29653156-29661163	0.260832	0	0.597855	OK
ENSBTAG00000035709	ENSBTAG00000035709	8:66018696-66030285	0.26075	0	1.30375	OK
ENSBTAG00000015345	ENSBTAG00000015345	3:19779459-19787707	0.260719	0	0.592607	OK

ENSBTAG00000013402	ENSBTAG00000013402	22:11390435-11468069	0.260702	0	0.529557	OK
ENSBTAG00000023462	ENSBTAG00000023462	8:24927467-24930788	0.260595	0	1.11923	OK
ENSBTAG00000047324	ENSBTAG00000047324	26:16498732-16538258	0.260238	0	0.884859	OK
ENSBTAG00000017459	ENSBTAG00000017459	17:71750226-71755793	0.260234	0	0.470126	OK
ENSBTAG00000033381	ENSBTAG00000033381	27:32417550-32418888	0.26003	0	0.784307	OK
ENSBTAG00000010601	ENSBTAG00000010601	11:99460813-99462903	0.259868	0	0.495352	OK
ENSBTAG00000033508	ENSBTAG00000033508	29:27001839-27002766	0.259535	0	0.670087	OK
ENSBTAG00000019104	ENSBTAG00000019104	19:51833820-51842965	0.259474	0	0.450124	OK
ENSBTAG00000048292	ENSBTAG00000048292	3:10991141-10992086	0.259198	0	0.657324	OK
ENSBTAG00000048225	ENSBTAG00000048225	1:72476819-72477521	0.259108	0	0.884859	OK
ENSBTAG00000000919	ENSBTAG00000000919	9:25873434-25884617	0.259034	0	0.509365	OK
ENSBTAG00000013341	ENSBTAG00000013341	X:140120346-140138151	0.258804	0	0.508427	OK
ENSBTAG00000007837	ENSBTAG00000007837	11:73134171-73173557	0.258727	0	0.697631	OK
ENSBTAG00000019391	ENSBTAG00000019391	19:24569281-24570235	0.258718	0	0.651123	OK
ENSBTAG00000016961	ENSBTAG00000016961	1:115476358-115480295	0.258578	0.0525926	0.420741	OK
ENSBTAG00000002096	ENSBTAG00000002096	3:7996518-8005228	0.258566	0	0.641705	OK
ENSBTAG00000014659	ENSBTAG00000014659	5:56697177-56705119	0.258474	0	0.713497	OK
ENSBTAG00000014345	ENSBTAG00000014345	13:74526198-74535935	0.258235	0	0.712352	OK
ENSBTAG00000048238	ENSBTAG00000048238	29:26937411-26938343	0.258131	0	0.666492	OK
ENSBTAG00000003594	ENSBTAG00000003594	5:116546018-116556849	0.25804	0	0.437136	OK
ENSBTAG00000000617	ENSBTAG00000000617	20:67608-68945	0.258038	0	1.11082	OK
ENSBTAG00000045748	ENSBTAG00000045748	22:57156995-57161484	0.258031	0	0.55911	OK
ENSBTAG00000019684	ENSBTAG00000019684	23:8297777-8414740	0.258019	0	0.432811	OK
ENSBTAG00000040150	ENSBTAG00000040150	15:82740312-82741287	0.25791	0	0.637098	OK
ENSBTAG00000005507	ENSBTAG00000005507	28:14234515-14235496	0.257888	0	0.633202	OK
ENSBTAG00000005017	ENSBTAG00000005017	8:86955748-86974921	0.257867	0	0.589906	OK
ENSBTAG00000008964	ENSBTAG00000008964	13:38909549-38989758	0.25785	0	0.484533	OK
ENSBTAG00000015218	ENSBTAG00000015218	8:86491136-86491828	0.257785	0	0.904179	OK
ENSBTAG00000004165	ENSBTAG00000004165	12:80409750-80638113	0.257355	0	1.33954	OK
ENSBTAG00000047362	ENSBTAG00000047362	15:63656458-63663593	0.256944	0	0.607799	OK

ENSBTAG00000007493	ENSBTAG00000007493	X:96027032-96028213	0.256943	0	0.701888	OK
ENSBTAG00000047107	ENSBTAG00000047107	17:40818490-40848672	0.256943	0	0.692498	OK
ENSBTAG00000005066	ENSBTAG00000005066	1:67664798-67720894	0.256825	0	0.64976	OK
ENSBTAG00000027513	ENSBTAG00000027513	6:90695493-90697557	0.256817	0	0.558105	OK
ENSBTAG00000002019	ENSBTAG00000002019	24:62248908-62271054	0.256777	0	0.710072	OK
ENSBTAG00000003016	ENSBTAG00000003016	16:70902205-70917245	0.256735	0	0.506458	OK
ENSBTAG00000047027	ENSBTAG00000047027	10:21596541-21598722	0.256732	0	0.767826	OK
ENSBTAG00000027430	ENSBTAG00000027430	15:47991244-47992231	0.256444	0	1.04892	OK
ENSBTAG00000008255	ENSBTAG00000008255	6:4093247-4152761	0.256382	0	0.625928	OK
ENSBTAG00000008177	ENSBTAG00000008177	13:176430-177363	0.256376	0	1.10963	OK
ENSBTAG00000010856	ENSBTAG00000010856	18:54853502-54881796	0.256325	0	0.448661	OK
ENSBTAG00000024044	ENSBTAG00000024044	11:21526905-21574655	0.256281	0	0.863457	OK
ENSBTAG00000001515	ENSBTAG00000001515	15:992063-992999	0.256266	0	1.10607	OK
ENSBTAG00000002169	ENSBTAG00000002169	7:1590816-1592152	0.256241	0	0.777825	OK
ENSBTAG00000001537	ENSBTAG00000001537	15:56961319-56974713	0.256017	0	0.490463	OK
ENSBTAG00000038201	ENSBTAG00000038201	4:108082734-108083676	0.255948	0	1.09903	OK
ENSBTAG00000018090	ENSBTAG00000018090	X:18910528-18918493	0.255922	0	0.645037	OK
ENSBTAG00000022647	ENSBTAG00000022647	9:46186208-46187168	0.255764	0	0.647053	OK
ENSBTAG00000046784	ENSBTAG00000046784	4:90056286-90057118	0.255731	0	0.768776	OK
ENSBTAG00000007424	ENSBTAG00000007424	17:71413854-71418166	0.25559	0	0.451843	OK
ENSBTAG00000007262	ENSBTAG00000007262	18:41116112-41214205	0.255427	0	0.477456	OK
ENSBTAG00000002184	ENSBTAG00000002184	2:121636111-121638403	0.255407	0	0.401273	OK
ENSBTAG00000009784	ENSBTAG00000009784	9:84937828-84969252	0.255212	0	0.606612	OK
ENSBTAG00000035182	ENSBTAG00000035182	24:62340434-62350499	0.254861	0	0.539211	OK
ENSBTAG00000000801	ENSBTAG00000000801	18:63903403-63910967	0.254788	0	1.14348	OK
ENSBTAG00000047569	ENSBTAG00000047569	24:60351646-60352993	0.254514	0	0.480039	OK
ENSBTAG00000024379	ENSBTAG00000024379	4:44481886-44517455	0.254503	0	0.678675	OK
ENSBTAG00000010456	ENSBTAG00000010456	3:2089526-2105290	0.254344	0	0.545365	OK
ENSBTAG00000016151	ENSBTAG00000016151	19:20413682-20416614	0.254289	0	0.38606	OK
ENSBTAG00000019143	ENSBTAG00000019143	8:64998046-65000257	0.254167	0	0.468243	OK

ENSBTAG00000047463	ENSBTAG00000047463	29:27079828-27081153	0.254093	0	0.655244	OK
ENSBTAG00000047182	ENSBTAG00000047182	7:41516959-41517898	0.254034	0	0.661524	OK
ENSBTAG00000026123	ENSBTAG00000026123	8:60483505-60484453	0.254017	0	0.655244	OK
ENSBTAG00000034349	ENSBTAG00000034349	3:66282836-66297668	0.253778	0	0.74642	OK
ENSBTAG00000012868	ENSBTAG00000012868	7:22051620-22085201	0.253675	0	0.598431	OK
ENSBTAG00000038798	ENSBTAG00000038798	14:79879897-79896055	0.253637	0	0.66068	OK
ENSBTAG00000034776	ENSBTAG00000034776	3:58808969-58965541	0.253618	0	0.533102	OK
ENSBTAG00000038968	ENSBTAG00000038968	15:43948285-43949549	0.253427	0	0.83022	OK
ENSBTAG00000006411	ENSBTAG00000006411	18:35472715-35474389	0.253386	0	0.49476	OK
ENSBTAG00000013384	ENSBTAG00000013384	23:30349198-30350140	0.253333	0	1.09903	OK
ENSBTAG00000006499	ENSBTAG00000006499	19:39995183-40017502	0.253287	0	0.484155	OK
ENSBTAG00000048209	ENSBTAG00000048209	2:72106413-72107566	0.253269	0	0.936062	OK
ENSBTAG00000047934	ENSBTAG00000047934	29:27172355-27173288	0.253212	0	0.665778	OK
ENSBTAG00000014517	ENSBTAG00000014517	6:60173833-60206257	0.253099	0	0.528375	OK
ENSBTAG00000016139	ENSBTAG00000016139	25:34726228-34731680	0.253068	0	0.475993	OK
ENSBTAG00000031135	ENSBTAG00000031135	7:27491081-27503407	0.252913	0	0.712025	OK
ENSBTAG00000020030	ENSBTAG00000020030	13:73467644-73473671	0.252849	0.0582603	0.466082	OK
ENSBTAG00000006015	ENSBTAG00000006015	23:17092571-17115947	0.252791	0	0.581893	OK
ENSBTAG00000009757	ENSBTAG00000009757	22:12729702-12741653	0.252578	0	0.62304	OK
ENSBTAG00000026275	ENSBTAG00000026275	25:34578755-34582072	0.252438	0	0.456073	OK
ENSBTAG00000039218	ENSBTAG00000039218	11:60268165-60268762	0.252188	0	1.0673	OK
ENSBTAG00000007259	ENSBTAG00000007259	5:76173494-76175969	0.252134	0	0.501956	OK
ENSBTAG00000001767	ENSBTAG00000001767	7:40482364-40490836	0.251969	0	1.25985	OK
ENSBTAG00000008113	ENSBTAG00000008113	11:79487138-79494238	0.251885	0	0.430696	OK
ENSBTAG00000038221	ENSBTAG00000038221	7:45308773-45323334	0.251867	0	0.415568	OK
ENSBTAG00000011539	ENSBTAG00000011539	19:39964764-39969793	0.251844	0	0.442052	OK
ENSBTAG00000005961	ENSBTAG00000005961	7:24318563-24436629	0.251773	0	0.563676	OK
ENSBTAG00000017461	ENSBTAG00000017461	19:51243971-51255385	0.251346	0	0.436093	OK
ENSBTAG00000020327	ENSBTAG00000020327	10:85669278-85711371	0.25133	0	0.670213	OK
ENSBTAG00000003925	ENSBTAG00000003925	9:105073005-105075610	0.251326	0	0.670204	OK

ENSBTAG00000020099	ENSBTAG00000020099	14:44969001-45027081	0.251281	0	0.643034	OK
ENSBTAG00000039509	ENSBTAG00000039509	5:97433254-97453094	0.2512	0	0.504033	OK
ENSBTAG00000026523	ENSBTAG00000026523	9:82419234-82420458	0.251072	0	0.507493	OK
ENSBTAG00000024169	ENSBTAG00000024169	23:31040773-31044230	0.25092	0	0.59895	OK
ENSBTAG00000021643	ENSBTAG00000021643	22:56965858-56983710	0.250917	0	0.471119	OK
ENSBTAG00000007601	ENSBTAG00000007601	19:38611845-38613064	0.250809	0	0.84929	OK
ENSBTAG00000020522	ENSBTAG00000020522	X:56005900-56020926	0.250789	0	0.713579	OK
ENSBTAG00000003365	ENSBTAG00000003365	19:10390869-10395903	0.250758	0	0.488067	OK
ENSBTAG00000022809	ENSBTAG00000022809	20:63004483-63005503	0.25075	0	1.06511	OK
ENSBTAG00000021435	ENSBTAG00000021435	5:110475769-110484826	0.25069	0	0.495589	OK
ENSBTAG00000010795	ENSBTAG00000010795	10:68073406-68110299	0.250556	0.0490656	0.441591	OK
ENSBTAG00000009600	ENSBTAG00000009600	11:10771665-10795813	0.250497	0	0.537531	OK
ENSBTAG00000034850	ENSBTAG00000034850	29:7723700-7725004	0.250318	0	0.79393	OK
ENSBTAG00000037765	ENSBTAG00000037765	7:20697580-20716655	0.250271	0	0.474176	OK
ENSBTAG00000008059	ENSBTAG00000008059	28:12810766-12812539	0.25024	0	0.583917	OK
ENSBTAG00000019509	ENSBTAG00000019509	13:12246302-12266216	0.250216	0	0.502565	OK
ENSBTAG00000012491	ENSBTAG00000012491	X:148404251-148409530	0.250073	0	0.879845	OK
ENSBTAG00000046945	ENSBTAG00000046945	7:9986650-9987589	0.249946	0	1.10254	OK
ENSBTAG00000037546	ENSBTAG00000037546	3:11610518-11611472	0.249863	0	1.0852	OK
ENSBTAG00000046570	ENSBTAG00000046570	3:99695132-99696623	0.249771	0	0.700463	OK
ENSBTAG00000018863	ENSBTAG00000018863	27:14110850-14163989	0.249752	0	0.595562	OK
ENSBTAG00000008423	ENSBTAG00000008423	19:22432981-22439070	0.249739	0	0.390919	OK
ENSBTAG00000039347	ENSBTAG00000039347	5:102144409-102152592	0.249729	0	0.745703	OK
ENSBTAG00000007235	ENSBTAG00000007235	11:102286503-102356914	0.249687	0	1.24844	OK
ENSBTAG00000009527	ENSBTAG00000009527	3:92701748-92745663	0.249534	0	0.614412	OK
ENSBTAG00000015761	ENSBTAG00000015761	17:65047402-65064488	0.249275	0	0.682906	OK
ENSBTAG00000002076	ENSBTAG00000002076	14:38927060-39070664	0.249262	0	0.691727	OK
ENSBTAG00000019417	ENSBTAG00000019417	X:55447560-55451996	0.249228	0	0.495747	OK
ENSBTAG00000019949	ENSBTAG00000019949	1:132002920-132014620	0.249116	0	0.861073	OK
ENSBTAG00000012912	ENSBTAG00000012912	2:38780332-38785787	0.249041	0	0.658159	OK

ENSBTAG00000027713	ENSBTAG00000027713	7:92440984-92446470	0.248881	0	0.884859	OK
ENSBTAG00000000619	ENSBTAG00000000619	5:27487719-27496237	0.248585	0	0.866813	OK
ENSBTAG00000004941	ENSBTAG00000004941	16:61350200-61360520	0.248476	0	0.69019	OK
ENSBTAG00000036111	ENSBTAG00000036111	28:21867758-21868711	0.247894	0	0.651806	OK
ENSBTAG00000000693	ENSBTAG00000000693	8:76567995-76663653	0.247893	0	0.725667	OK
ENSBTAG00000046252	ENSBTAG00000046252	X:118840822-118842716	0.24781	0	0.610787	OK
ENSBTAG00000006198	ENSBTAG00000006198	10:26963200-26964130	0.247596	0	1.11321	OK
ENSBTAG00000003193	ENSBTAG00000003193	10:36280970-36284979	0.247401	0	0.374425	OK
ENSBTAG00000004428	ENSBTAG00000004428	6:26103021-26104344	0.247206	0	0.863937	OK
ENSBTAG00000019132	ENSBTAG00000019132	6:104304074-104320863	0.24719	0	0.681781	OK
ENSBTAG00000010688	ENSBTAG00000010688	1:67373083-67383963	0.24711	0	1.06365	OK
ENSBTAG00000020961	ENSBTAG00000020961	2:13503489-13519946	0.247096	0	0.698926	OK
ENSBTAG00000005859	ENSBTAG00000005859	5:31347860-31349882	0.24693	0	0.857971	OK
ENSBTAG00000021678	ENSBTAG00000021678	8:104172061-104206275	0.246872	0	0.550391	OK
ENSBTAG00000016847	ENSBTAG00000016847	19:43945991-43947907	0.246738	0	0.461494	OK
ENSBTAG00000002272	ENSBTAG00000002272	2:89450806-89471623	0.24667	0	0.480782	OK
ENSBTAG00000013995	ENSBTAG00000013995	X:119253544-119254582	0.246525	0	0.598431	OK
ENSBTAG00000005362	ENSBTAG00000005362	23:2936080-2953601	0.246334	0	0.869987	OK
ENSBTAG00000021157	ENSBTAG00000021157	18:62665593-62676998	0.245733	0	2.38051	OK
ENSBTAG00000001755	ENSBTAG00000001755	2:14953995-14956356	0.245634	0	0.526193	OK
ENSBTAG00000017090	ENSBTAG00000017090	19:34932549-34960757	0.24558	0	0.441016	OK
ENSBTAG00000003273	ENSBTAG00000003273	14:1259892-1260826	0.245498	0	1.10844	OK
ENSBTAG00000017275	ENSBTAG00000017275	19:7988037-8010218	0.245473	0	0.629352	OK
ENSBTAG00000014838	ENSBTAG00000014838	15:84838923-84855877	0.245365	0	1.22683	OK
ENSBTAG00000022779	ENSBTAG00000022779	12:10667342-10690088	0.245318	0	0.658578	OK
ENSBTAG00000014411	ENSBTAG00000014411	5:70573523-70593134	0.245072	0	0.554518	OK
ENSBTAG00000021415	ENSBTAG00000021415	23:20893700-21091299	0.244948	0	0.457584	OK
ENSBTAG00000009271	ENSBTAG00000009271	10:85511320-85513973	0.24489	0	0.468278	OK
ENSBTAG00000024381	ENSBTAG00000024381	9:32093590-32262873	0.244793	0	0.453674	OK
ENSBTAG00000048273	ENSBTAG00000048273	6:7145413-7150482	0.244763	0	0.613684	OK

ENSBTAG00000015406	ENSBTAG00000015406	19:50475195-50483968	0.244756	0	0.468758	OK
ENSBTAG00000005945	ENSBTAG00000005945	15:25419218-25434051	0.244558	0	0.705475	OK
ENSBTAG00000003462	ENSBTAG00000003462	18:65676641-65689192	0.24453	0	0.455404	OK
ENSBTAG00000005212	ENSBTAG00000005212	1:82573989-82694776	0.244445	0	0.473815	OK
ENSBTAG00000016647	ENSBTAG00000016647	29:27265035-27265974	0.244077	0	0.661524	OK
ENSBTAG00000045586	ENSBTAG00000045586	23:28950034-28951009	0.244009	0	0.637098	OK
ENSBTAG00000014724	ENSBTAG00000014724	1:81057778-81064090	0.243902	0	0.657324	OK
ENSBTAG00000031654	ENSBTAG00000031654	3:31464989-31684943	0.243852	0	0.526059	OK
ENSBTAG00000005380	ENSBTAG00000005380	20:25965174-25975522	0.243731	0	0.66082	OK
ENSBTAG00000010612	ENSBTAG00000010612	4:77540573-77543351	0.243612	0	0.640647	OK
ENSBTAG00000035442	ENSBTAG00000035442	X:28811969-28812350	0.24347	0	1.63037	OK
ENSBTAG00000027387	ENSBTAG00000027387	13:61211661-61218035	0.243178	0	0.777314	OK
ENSBTAG00000009764	ENSBTAG00000009764	8:85141063-85150512	0.24303	0.0502687	0.653494	OK
ENSBTAG00000047579	ENSBTAG00000047579	3:58428133-58428880	0.242926	0	0.831554	OK
ENSBTAG00000045505	ENSBTAG00000045505	27:28343936-28345036	0.24292	0	1.00189	OK
ENSBTAG00000039522	ENSBTAG00000039522	2:18780935-18799857	0.242785	0	1.05283	OK
ENSBTAG00000007353	ENSBTAG00000007353	19:37314776-37320363	0.242384	0	0.662582	OK
ENSBTAG00000037616	ENSBTAG00000037616	1:151610044-151610971	0.242269	0	0.670087	OK
ENSBTAG00000016720	ENSBTAG00000016720	11:63429724-63458565	0.242144	0	0.835257	OK
ENSBTAG00000009141	ENSBTAG00000009141	24:43927643-43941907	0.242	0	0.621047	OK
ENSBTAG00000004140	ENSBTAG00000004140	11:104341437-104358922	0.241675	0	0.428689	OK
ENSBTAG00000047371	ENSBTAG00000047371	1:42308633-42309643	0.241126	0	1.07062	OK
ENSBTAG00000046101	ENSBTAG00000046101	18:64014664-64032839	0.241124	0	0.478634	OK
ENSBTAG00000007704	ENSBTAG00000007704	20:18516520-18532377	0.24101	0	0.793322	OK
ENSBTAG00000004721	ENSBTAG00000004721	5:97239156-97258621	0.240908	0	0.461151	OK
ENSBTAG00000005807	ENSBTAG00000005807	28:28562367-28777941	0.240745	0	0.529332	OK
ENSBTAG00000007123	ENSBTAG00000007123	3:20141693-20146997	0.240576	0	0.510621	OK
ENSBTAG00000000562	ENSBTAG00000000562	28:7926959-7941655	0.240573	0	0.615142	OK
ENSBTAG00000046002	ENSBTAG00000046002	3:119687613-119688555	0.240234	0	0.659417	OK
ENSBTAG00000012595	ENSBTAG00000012595	14:68412746-68420944	0.240201	0	0.60191	OK

ENSBTAG00000001166	ENSBTAG00000001166	19:9645918-9670385	0.240179	0	0.839477	OK
ENSBTAG00000011994	ENSBTAG00000011994	19:28637770-28672316	0.240173	0	0.829399	OK
ENSBTAG00000010734	ENSBTAG00000010734	7:39202865-39207621	0.240105	0	0.717287	OK
ENSBTAG00000038982	ENSBTAG00000038982	8:59542275-59548164	0.240089	0.0517772	0.465995	OK
ENSBTAG00000001992	ENSBTAG00000001992	4:9306413-9323252	0.239937	0	0.625078	OK
ENSBTAG00000004063	ENSBTAG00000004063	21:59782813-59795808	0.239838	0	0.488342	OK
ENSBTAG00000009643	ENSBTAG00000009643	10:76943980-76960818	0.239621	0	0.608991	OK
ENSBTAG00000014227	ENSBTAG00000014227	12:54923988-54993756	0.239449	0	0.676657	OK
ENSBTAG00000023666	ENSBTAG00000023666	1:42566822-42571545	0.239403	0	1.19702	OK
ENSBTAG00000003543	ENSBTAG00000003543	7:16235814-16244046	0.239366	0	0.407272	OK
ENSBTAG00000007807	ENSBTAG00000007807	10:76992201-76994112	0.238945	0	0.436829	OK
ENSBTAG00000014080	ENSBTAG00000014080	6:7797200-7837774	0.238662	0	0.621093	OK
ENSBTAG00000008310	ENSBTAG00000008310	15:44215627-44234314	0.23856	0	0.537531	OK
ENSBTAG00000025293	ENSBTAG00000025293	7:42651610-42652555	0.23794	0	0.657324	OK
ENSBTAG00000031115	ENSBTAG00000031115	22:57789250-57823760	0.23778	0	0.873658	OK
ENSBTAG00000005779	ENSBTAG00000005779	7:33311978-33313090	0.237594	0	0.558607	OK
ENSBTAG00000018201	ENSBTAG00000018201	29:40537652-40549701	0.237432	0	0.41099	OK
ENSBTAG00000040585	ENSBTAG00000040585	11:95551662-95586813	0.237305	0	1.18652	OK
ENSBTAG00000047082	ENSBTAG00000047082	7:41684971-41685919	0.237274	0	1.09207	OK
ENSBTAG00000038168	ENSBTAG00000038168	12:59144542-59146633	0.237098	0	0.495115	OK
ENSBTAG00000007594	ENSBTAG00000007594	26:21010621-21035035	0.237075	0	0.631592	OK
ENSBTAG00000003446	ENSBTAG00000003446	25:36347293-36362877	0.236992	0	0.408665	OK
ENSBTAG00000013159	ENSBTAG00000013159	12:19150793-19184692	0.236714	0	0.569777	OK
ENSBTAG00000008308	ENSBTAG00000008308	11:105140965-105142767	0.2367	0	1.12899	OK
ENSBTAG00000001274	ENSBTAG00000001274	1:107241226-107576086	0.236655	0	0.573565	OK
ENSBTAG00000035882	ENSBTAG00000035882	X:79134280-79135105	0.236655	0	0.752934	OK
ENSBTAG00000001698	ENSBTAG00000001698	18:36458138-36469860	0.236588	0	0.426922	OK
ENSBTAG00000013130	ENSBTAG00000013130	4:107240159-107241118	0.236559	0	0.647728	OK
ENSBTAG00000023550	ENSBTAG00000023550	1:58996572-58997512	0.23641	0	0.66082	OK
ENSBTAG00000005679	ENSBTAG00000005679	25:37902688-37918783	0.236196	0	0.412628	OK

ENSBTAG00000036273	ENSBTAG00000036273	9:274515-276372	0.236046	0	1.05212	OK
ENSBTAG00000017483	ENSBTAG00000017483	23:50515221-50529891	0.235737	0	0.500944	OK
ENSBTAG00000013408	ENSBTAG00000013408	14:72874864-72877819	0.235688	0	0.42042	OK
ENSBTAG00000038738	ENSBTAG00000038738	13:63726557-63743492	0.235613	0	0.41439	OK
ENSBTAG00000021961	ENSBTAG00000021961	11:11540746-11548873	0.235607	0	0.427215	OK
ENSBTAG00000002082	ENSBTAG00000002082	9:37022763-37026932	0.235386	0	0.912802	OK
ENSBTAG00000003856	ENSBTAG00000003856	18:42749251-42750804	0.235347	0	0.489111	OK
ENSBTAG00000040119	ENSBTAG00000040119	15:50576451-50577620	0.235323	0	0.653177	OK
ENSBTAG00000047719	ENSBTAG00000047719	10:5653452-5655684	0.235242	0	0.463837	OK
ENSBTAG00000003965	ENSBTAG00000003965	11:4280892-4283779	0.235228	0	0.810716	OK
ENSBTAG00000003035	ENSBTAG00000003035	24:32611657-32646496	0.235016	0	0.558707	OK
ENSBTAG00000007477	ENSBTAG00000007477	27:4788446-4815238	0.234924	0	0.664354	OK
ENSBTAG00000009911	ENSBTAG00000009911	10:60140537-60162731	0.234924	0	0.445667	OK
ENSBTAG00000004643	ENSBTAG00000004643	4:88776839-88856547	0.234876	0	0.565536	OK
ENSBTAG00000022762	ENSBTAG00000022762	7:89654003-89655145	0.234392	0	0.543932	OK
ENSBTAG00000035643	ENSBTAG00000035643	13:41140042-41141508	0.234239	0	0.725497	OK
ENSBTAG00000013169	ENSBTAG00000013169	25:22964773-23034012	0.234216	0	0.489496	OK
ENSBTAG00000022450	ENSBTAG00000022450	6:99498239-99519280	0.233746	0	0.589906	OK
ENSBTAG00000005150	ENSBTAG00000005150	X:33794433-33801266	0.233668	0	1.30204	OK
ENSBTAG00000002976	ENSBTAG00000002976	18:51877253-51888120	0.233507	0	0.381789	OK
ENSBTAG00000012217	ENSBTAG00000012217	2:133111282-133124161	0.233175	0.046666	0.373328	OK
ENSBTAG00000011632	ENSBTAG00000011632	18:13017743-13031053	0.233162	0	0.671899	OK
ENSBTAG00000013008	ENSBTAG00000013008	5:66359937-66361103	0.233122	0	1.16324	OK
ENSBTAG00000008678	ENSBTAG00000008678	8:7143930-7145796	0.232813	0	0.568525	OK
ENSBTAG00000024869	ENSBTAG00000024869	18:25470740-25474347	0.232785	0	0.550683	OK
ENSBTAG00000032764	ENSBTAG00000032764	6:323370-323914	0.232665	0	1.14186	OK
ENSBTAG00000038327	ENSBTAG00000038327	10:65655783-65657656	0.232574	0	0.561739	OK
ENSBTAG00000007880	ENSBTAG00000007880	25:517558-539514	0.232388	0	0.417959	OK
ENSBTAG00000008650	ENSBTAG00000008650	13:11729422-11787953	0.23234	0	0.53457	OK
ENSBTAG00000035701	ENSBTAG00000035701	1:140442787-140444211	0.232303	0	1.04469	OK

ENSBTAG00000010002	ENSBTAG00000010002	27:13909932-13950319	0.232139	0	0.591591	OK
ENSBTAG00000025593	ENSBTAG00000025593	10:36264748-36274189	0.232122	0	0.639855	OK
ENSBTAG00000002286	ENSBTAG00000002286	7:52305243-52337268	0.232116	0.0478192	0.430372	OK
ENSBTAG00000047080	ENSBTAG00000047080	5:121141235-121142831	0.232087	0	0.688354	OK
ENSBTAG00000014576	ENSBTAG00000014576	17:44600508-44665571	0.231996	0	0.508115	OK
ENSBTAG00000047159	ENSBTAG00000047159	27:17502163-17503078	0.231974	0	0.701888	OK
ENSBTAG00000032944	ENSBTAG00000032944	14:39504411-39505564	0.231897	0	0.546805	OK
ENSBTAG00000040062	ENSBTAG00000040062	26:50473056-50473924	0.231737	0	0.715635	OK
ENSBTAG00000012798	ENSBTAG00000012798	1:138455303-138676649	0.231656	0	0.432269	OK
ENSBTAG00000011820	ENSBTAG00000011820	10:46006653-46130505	0.231604	0	0.391906	OK
ENSBTAG00000008485	ENSBTAG00000008485	X:57713805-57715859	0.231478	0	0.566245	OK
ENSBTAG00000016744	ENSBTAG00000016744	15:65457709-65497385	0.231161	0	0.499534	OK
ENSBTAG00000010184	ENSBTAG00000010184	19:43590845-43601632	0.230387	0	0.628636	OK
ENSBTAG00000048222	ENSBTAG00000048222	29:26960625-26961561	0.230387	0	0.663644	OK
ENSBTAG00000040038	ENSBTAG00000040038	8:8621812-8633235	0.230352	0	0.565729	OK
ENSBTAG00000037758	ENSBTAG00000037758	5:58114642-58115581	0.230165	0	0.661524	OK
ENSBTAG00000018147	ENSBTAG00000018147	2:58582460-58712391	0.229967	0	0.757895	OK
ENSBTAG00000021632	ENSBTAG00000021632	8:8635549-9019576	0.229809	0	0.454404	OK
ENSBTAG00000047879	ENSBTAG00000047879	3:32851232-32852963	0.229519	0	0.358851	OK
ENSBTAG00000035631	ENSBTAG00000035631	29:27820501-27821434	0.229489	0	0.665778	OK
ENSBTAG00000046452	ENSBTAG00000046452	X:4247932-4250133	0.229457	0	0.645708	OK
ENSBTAG00000046395	ENSBTAG00000046395	X:13761203-13761845	0.229251	0	0.967556	OK
ENSBTAG00000019014	ENSBTAG00000019014	1:83090389-83091984	0.229224	0	0.389449	OK
ENSBTAG00000005251	ENSBTAG00000005251	15:48506849-48521081	0.228932	0	0.692962	OK
ENSBTAG00000005106	ENSBTAG00000005106	26:15126482-15134920	0.228895	0	0.823616	OK
ENSBTAG00000048272	ENSBTAG00000048272	8:31463623-31464959	0.228874	0	0.733378	OK
ENSBTAG00000020124	ENSBTAG00000020124	11:10089785-10093086	0.228654	0	1.14327	OK
ENSBTAG00000039162	ENSBTAG00000039162	7:54106880-54110067	0.22837	0	0.454785	OK
ENSBTAG00000012206	ENSBTAG00000012206	21:33625676-33633910	0.228357	0	0.361988	OK
ENSBTAG00000003178	ENSBTAG00000003178	26:42041628-42198853	0.228332	0	0.582931	OK

ENSBTAG00000016030	ENSBTAG00000016030	2:20828933-20832040	0.228208	0	0.591253	OK
ENSBTAG00000012793	ENSBTAG00000012793	5:66127272-66128521	0.228179	0	0.497335	OK
ENSBTAG00000040602	ENSBTAG00000040602	2:131410547-131414758	0.228178	0	0.593854	OK
ENSBTAG00000015116	ENSBTAG00000015116	7:13724191-13730226	0.22809	0	0.568838	OK
ENSBTAG00000006380	ENSBTAG00000006380	X:126050903-126054020	0.228001	0	0.786203	OK
ENSBTAG00000007349	ENSBTAG00000007349	7:54012328-54014719	0.227941	0	0.432992	OK
ENSBTAG00000006278	ENSBTAG00000006278	13:65214403-65220014	0.227858	0	0.369085	OK
ENSBTAG00000007714	ENSBTAG00000007714	18:52550187-52559972	0.227856	0	1.13928	OK
ENSBTAG00000047919	ENSBTAG00000047919	4:106869242-106883852	0.227675	0	0.865743	OK
ENSBTAG00000045574	ENSBTAG00000045574	5:37326838-37328199	0.227668	0	0.456408	OK
ENSBTAG00000009679	ENSBTAG00000009679	9:88955699-89122287	0.227653	0.0475665	0.380532	OK
ENSBTAG00000030564	ENSBTAG00000030564	13:80427989-80429863	0.227613	0	0.436829	OK
ENSBTAG00000047837	ENSBTAG00000047837	2:136338528-136351001	0.227578	0	0.500894	OK
ENSBTAG00000006846	ENSBTAG00000006846	19:19738611-19757359	0.22746	0	1.1373	OK
ENSBTAG00000014309	ENSBTAG00000014309	15:80314081-80314987	0.227432	0	0.685619	OK
ENSBTAG00000001020	ENSBTAG00000001020	5:105896015-105941353	0.227396	0	0.413287	OK
ENSBTAG00000018015	ENSBTAG00000018015	25:26319021-26324595	0.227112	0	0.518507	OK
ENSBTAG00000038335	ENSBTAG00000038335	8:103392506-103426196	0.227005	0	0.747498	OK
ENSBTAG00000020963	ENSBTAG00000020963	22:50580393-50586504	0.226972	0	0.366472	OK
ENSBTAG00000015911	ENSBTAG00000015911	9:23384928-23387921	0.226951	0	0.635144	OK
ENSBTAG00000012249	ENSBTAG00000012249	19:39171883-39177343	0.226703	0	0.447529	OK
ENSBTAG00000048025	ENSBTAG00000048025	7:43187984-43188914	0.226695	0	0.667926	OK
ENSBTAG00000016388	ENSBTAG00000016388	13:55149363-55172423	0.226626	0	0.444138	OK
ENSBTAG00000023073	ENSBTAG00000023073	28:3656712-3676337	0.226565	0	0.442745	OK
ENSBTAG00000000019	ENSBTAG00000000019	9:29081412-29113549	0.226561	0	0.743739	OK
ENSBTAG00000008924	ENSBTAG00000008924	12:12741068-12782474	0.2265	0	0.653177	OK
ENSBTAG00000047021	ENSBTAG00000047021	15:50403622-50404606	0.226164	0	0.631271	OK
ENSBTAG00000047267	ENSBTAG00000047267	29:27100822-27101755	0.226153	0	0.665778	OK
ENSBTAG00000000911	ENSBTAG00000000911	4:94467622-94565768	0.226131	0	0.555609	OK
ENSBTAG00000010564	ENSBTAG00000010564	6:16376641-16510240	0.226117	0	0.589486	OK

ENSBTAG00000033747	ENSBTAG00000033747	7:51888629-52144488	0.225643	0	0.371736	OK
ENSBTAG00000001290	ENSBTAG00000001290	19:11046573-11070490	0.225626	0	0.596993	OK
ENSBTAG00000002025	ENSBTAG00000002025	5:33340317-33341883	0.225498	0	0.661101	OK
ENSBTAG00000007537	ENSBTAG00000007537	3:9517704-9523145	0.225377	0	0.53146	OK
ENSBTAG00000037577	ENSBTAG00000037577	23:29622275-29623220	0.224401	0	0.657324	OK
ENSBTAG00000007104	ENSBTAG00000007104	10:21206753-21214476	0.224384	0	0.367774	OK
ENSBTAG00000047355	ENSBTAG00000047355	14:42269143-42270032	0.224369	0	0.717287	OK
ENSBTAG00000047591	ENSBTAG00000047591	23:22040480-22044849	0.224299	0	0.448175	OK
ENSBTAG00000016873	ENSBTAG00000016873	13:73391553-73401614	0.22426	0	0.681108	OK
ENSBTAG00000009647	ENSBTAG00000009647	7:28555706-28568143	0.224142	0	0.489496	OK
ENSBTAG00000004575	ENSBTAG00000004575	4:31742335-31791212	0.224087	0	0.661101	OK
ENSBTAG00000040438	ENSBTAG00000040438	25:2658918-2661149	0.22408	0	0.643034	OK
ENSBTAG00000046346	ENSBTAG00000046346	21:71291845-71316837	0.224021	0	0.403148	OK
ENSBTAG00000008842	ENSBTAG00000008842	14:39633188-39725607	0.223982	0.0465506	0.372405	OK
ENSBTAG00000021180	ENSBTAG00000021180	28:27096454-27111015	0.223928	0	0.498932	OK
ENSBTAG00000048037	ENSBTAG00000048037	15:79467385-79468306	0.223662	0	0.674453	OK
ENSBTAG00000006665	ENSBTAG00000006665	17:64419026-64426503	0.22361	0	0.50338	OK
ENSBTAG00000006779	ENSBTAG00000006779	18:2486595-2491819	0.223553	0	0.437568	OK
ENSBTAG00000004712	ENSBTAG00000004712	13:60185128-60186103	0.223538	0	0.637098	OK
ENSBTAG00000019092	ENSBTAG00000019092	19:15284155-15295984	0.22352	0	0.476724	OK
ENSBTAG00000013664	ENSBTAG00000013664	1:43756829-43760080	0.223506	0	0.664354	OK
ENSBTAG00000039370	ENSBTAG00000039370	10:27362584-27364366	0.223421	0	0.674453	OK
ENSBTAG00000048221	ENSBTAG00000048221	15:415570-416485	0.223409	0	0.678875	OK
ENSBTAG00000003321	ENSBTAG00000003321	X:60442347-60489231	0.223234	0	0.743917	OK
ENSBTAG00000006672	ENSBTAG00000006672	9:64722281-64744803	0.223148	0	0.519461	OK
ENSBTAG00000012176	ENSBTAG00000012176	18:53684729-53690649	0.223143	0	0.417454	OK
ENSBTAG00000027937	ENSBTAG00000027937	13:47400412-47418507	0.223142	0	0.58601	OK
ENSBTAG00000017180	ENSBTAG00000017180	1:103001087-103004030	0.223117	0	0.703557	OK
ENSBTAG00000031028	ENSBTAG00000031028	15:79232754-79233684	0.223021	0	0.667926	OK
ENSBTAG00000005077	ENSBTAG00000005077	28:45410675-45418793	0.22298	0	0.60261	OK

ENSBTAG00000008446	ENSBTAG00000008446	25:28638037-28780415	0.222913	0	0.444138	OK
ENSBTAG00000016061	ENSBTAG00000016061	11:90012629-90029927	0.222859	0	0.484911	OK
ENSBTAG00000010048	ENSBTAG00000010048	2:27296840-27309226	0.222819	0	0.607799	OK
ENSBTAG00000011379	ENSBTAG00000011379	10:75235433-75637242	0.222637	0	0.558293	OK
ENSBTAG00000018771	ENSBTAG00000018771	18:10451368-10472288	0.222423	0	0.363099	OK
ENSBTAG00000017492	ENSBTAG00000017492	14:22669362-22717576	0.222422	0	0.460638	OK
ENSBTAG00000026369	ENSBTAG00000026369	20:6836624-6848939	0.222361	0.0463941	0.371153	OK
ENSBTAG00000005427	ENSBTAG00000005427	10:102933983-102966398	0.222265	0	0.438267	OK
ENSBTAG00000003838	ENSBTAG00000003838	14:10787394-10788143	0.222172	0	0.834907	OK
ENSBTAG00000010389	ENSBTAG00000010389	6:92967766-92971371	0.222045	0	0.555112	OK
ENSBTAG00000039503	ENSBTAG00000039503	15:50648224-50650344	0.222008	0	0.709099	OK
ENSBTAG00000003419	ENSBTAG00000003419	21:45354861-45371985	0.221955	0	0.533805	OK
ENSBTAG00000007989	ENSBTAG00000007989	9:54432729-54433809	0.221883	0	0.958597	OK
ENSBTAG00000019340	ENSBTAG00000019340	12:40887254-40890353	0.221714	0	0.601327	OK
ENSBTAG00000016957	ENSBTAG00000016957	8:112124218-112138177	0.221666	0	0.528207	OK
ENSBTAG00000015459	ENSBTAG00000015459	3:32942121-32945611	0.221572	0	0.544171	OK
ENSBTAG00000019987	ENSBTAG00000019987	22:43553716-43561145	0.221551	0	0.525525	OK
ENSBTAG00000045833	ENSBTAG00000045833	3:119708654-119709602	0.22152	0	0.655244	OK
ENSBTAG00000044075	ENSBTAG00000044075	1:136636525-136676796	0.221353	0	0.57452	OK
ENSBTAG00000039599	ENSBTAG00000039599	19:38561273-38564096	0.221192	0	0.413287	OK
ENSBTAG00000046915	ENSBTAG00000046915	3:91541032-91541967	0.221186	0	1.10593	OK
ENSBTAG00000004580	ENSBTAG00000004580	15:84615939-84630983	0.221083	0	0.567798	OK
ENSBTAG00000012904	ENSBTAG00000012904	22:21268890-21295366	0.220954	0	0.398296	OK
ENSBTAG00000007633	ENSBTAG00000007633	7:82970045-82971584	0.220777	0	0.6727	OK
ENSBTAG00000038619	ENSBTAG00000038619	23:28299436-28311309	0.22073	0	0.565729	OK
ENSBTAG00000013192	ENSBTAG00000013192	1:71834224-71839862	0.220577	0	0.60261	OK
ENSBTAG00000048084	ENSBTAG00000048084	3:119906879-119907812	0.22038	0	0.665778	OK
ENSBTAG00000014246	ENSBTAG00000014246	20:10407094-10425913	0.220369	0	0.832222	OK
ENSBTAG00000033389	ENSBTAG00000033389	25:1167350-1168312	0.220321	0	0.645708	OK
ENSBTAG00000047550	ENSBTAG00000047550	8:39037129-39038896	0.220119	0	0.70308	OK

ENSBTAG00000026610	ENSBTAG00000026610	5:95530630-95531308	0.220079	0	0.916181	OK
ENSBTAG00000000250	ENSBTAG00000000250	8:104331799-104334927	0.219693	0	0.383913	OK
ENSBTAG00000000313	ENSBTAG00000000313	28:29352463-29369144	0.219669	0	0.596362	OK
ENSBTAG00000003047	ENSBTAG00000003047	7:35574934-35578877	0.219593	0	0.520244	OK
ENSBTAG00000047817	ENSBTAG00000047817	7:10454412-10455333	0.219408	0	0.674453	OK
ENSBTAG00000001014	ENSBTAG00000001014	19:34687987-34693036	0.219364	0	0.409338	OK
ENSBTAG00000044027	ENSBTAG00000044027	12:81980947-82108529	0.219346	0	0.457079	OK
ENSBTAG00000015101	ENSBTAG00000015101	8:5583683-5586018	0.219065	0	0.540619	OK
ENSBTAG00000001324	ENSBTAG00000001324	1:136435601-136528516	0.218995	0	0.533927	OK
ENSBTAG00000002130	ENSBTAG00000002130	17:74397214-74412807	0.218984	0	0.422812	OK
ENSBTAG00000046160	ENSBTAG00000046160	23:45020473-45041768	0.218973	0.0457281	0.411553	OK
ENSBTAG00000038755	ENSBTAG00000038755	18:60952726-60988020	0.218962	0	0.496539	OK
ENSBTAG00000010793	ENSBTAG00000010793	1:57819992-57855630	0.218954	0	0.394287	OK
ENSBTAG00000040307	ENSBTAG00000040307	23:30553845-30554778	0.218926	0	0.665778	OK
ENSBTAG00000015516	ENSBTAG00000015516	9:77563946-77580398	0.218835	0	0.694046	OK
ENSBTAG00000008431	ENSBTAG00000008431	2:111602254-111631564	0.218777	0	0.555609	OK
ENSBTAG00000005970	ENSBTAG00000005970	17:70344598-70349566	0.218683	0	0.588564	OK
ENSBTAG00000017169	ENSBTAG00000017169	14:43705876-43706620	0.218507	0	0.87736	OK
ENSBTAG00000040351	ENSBTAG00000040351	14:20717784-20720429	0.218461	0	1.0923	OK
ENSBTAG00000009309	ENSBTAG00000009309	16:51683115-51688667	0.218425	0	0.387022	OK
ENSBTAG00000002625	ENSBTAG00000002625	9:60555708-60620133	0.218421	0	0.554617	OK
ENSBTAG00000024713	ENSBTAG00000024713	7:41463092-41464070	0.218277	0	1.05857	OK
ENSBTAG00000015146	ENSBTAG00000015146	10:25731941-25747290	0.218202	0	0.478744	OK
ENSBTAG00000015228	ENSBTAG00000015228	7:63748884-63756646	0.218181	0	0.452419	OK
ENSBTAG00000005294	ENSBTAG00000005294	X:60106180-60121827	0.217693	0	0.5733	OK
ENSBTAG00000016389	ENSBTAG00000016389	3:33424620-33426233	0.217656	0	0.385103	OK
ENSBTAG00000030584	ENSBTAG00000030584	7:53424461-53426334	0.217656	0	0.331645	OK
ENSBTAG00000046028	ENSBTAG00000046028	15:811485-812385	0.21755	0	0.69019	OK
ENSBTAG00000015263	ENSBTAG00000015263	29:40600719-40616875	0.21752	0	0.409203	OK
ENSBTAG00000040047	ENSBTAG00000040047	11:93617051-93618040	0.21745	0	0.666492	OK

ENSBTAG00000000197	ENSBTAG00000000197	6:26444868-26470795	0.217114	0	0.53886	OK
ENSBTAG00000040083	ENSBTAG00000040083	22:50950282-50953897	0.217024	0	0.40094	OK
ENSBTAG00000009127	ENSBTAG00000009127	9:34889675-34893768	0.217009	0	0.455293	OK
ENSBTAG00000031895	ENSBTAG00000031895	9:93240603-93307575	0.216507	0	0.661065	OK
ENSBTAG00000006815	ENSBTAG00000006815	15:48250682-48251648	0.2165	0	0.643034	OK
ENSBTAG00000003378	ENSBTAG00000003378	1:72758743-72926385	0.216417	0	0.796676	OK
ENSBTAG00000010852	ENSBTAG00000010852	23:31343550-31373121	0.216326	0	0.692962	OK
ENSBTAG00000009409	ENSBTAG00000009409	29:28458904-28466435	0.216319	0	0.526862	OK
ENSBTAG00000048274	ENSBTAG00000048274	X:82352349-82353434	0.216244	0	0.57945	OK
ENSBTAG00000012507	ENSBTAG00000012507	15:30297041-30302445	0.216192	0	0.387143	OK
ENSBTAG00000016252	ENSBTAG00000016252	19:44509433-44529146	0.216061	0	0.627762	OK
ENSBTAG00000004995	ENSBTAG00000004995	4:15183825-15227662	0.215975	0	0.670521	OK
ENSBTAG00000006383	ENSBTAG00000006383	X:126048948-126049743	0.215938	0	0.781347	OK
ENSBTAG00000040582	ENSBTAG00000040582	23:29462781-29463723	0.215894	0	0.659417	OK
ENSBTAG00000037996	ENSBTAG00000037996	X:55421777-55426607	0.215763	0	0.535492	OK
ENSBTAG00000030925	ENSBTAG00000030925	15:80177812-80178769	0.215448	0	0.649081	OK
ENSBTAG00000012375	ENSBTAG00000012375	8:22876231-22878736	0.215389	0	0.495945	OK
ENSBTAG00000011590	ENSBTAG00000011590	5:4974614-5021025	0.215251	0	1.10963	OK
ENSBTAG00000017367	ENSBTAG00000017367	26:11117505-11127464	0.215161	0	0.591967	OK
ENSBTAG00000006626	ENSBTAG00000006626	17:52565555-52785519	0.215131	0	0.389571	OK
ENSBTAG00000016377	ENSBTAG00000016377	3:100935636-100972708	0.215007	0	0.527758	OK
ENSBTAG00000048303	ENSBTAG00000048303	24:24396857-24401904	0.214909	0	1.04892	OK
ENSBTAG00000048286	ENSBTAG00000048286	11:86346273-86368606	0.214875	0	0.403227	OK
ENSBTAG00000001623	ENSBTAG00000001623	15:68996827-69000079	0.214694	0	0.699517	OK
ENSBTAG00000024065	ENSBTAG00000024065	4:106103109-106104018	0.214461	0	0.683356	OK
ENSBTAG00000045790	ENSBTAG00000045790	15:771398-773269	0.21412	0	0.670087	OK
ENSBTAG00000039812	ENSBTAG00000039812	23:31665561-31667068	0.213659	0	0.41219	OK
ENSBTAG00000037968	ENSBTAG00000037968	7:54125767-54128330	0.21342	0	0.426043	OK
ENSBTAG00000003588	ENSBTAG00000003588	14:83241746-83267308	0.21328	0	0.51829	OK
ENSBTAG00000037570	ENSBTAG00000037570	29:42120555-42155545	0.213249	0	0.624041	OK

ENSBTAG00000004361	ENSBTAG00000004361	16:56696959-56706787	0.213203	0	0.409203	OK
ENSBTAG00000001966	ENSBTAG00000001966	27:18701955-18775559	0.213176	0	0.402052	OK
ENSBTAG00000003172	ENSBTAG00000003172	10:32643647-32866661	0.213151	0	0.482463	OK
ENSBTAG00000009964	ENSBTAG00000009964	25:1763776-1766831	0.21311	0	0.412464	OK
ENSBTAG00000020214	ENSBTAG00000020214	3:53852739-53862657	0.213075	0	0.480411	OK
ENSBTAG00000020516	ENSBTAG00000020516	8:80779900-80785155	0.212981	0	0.752934	OK
ENSBTAG00000017845	ENSBTAG00000017845	8:76352437-76363634	0.212953	0	0.606849	OK
ENSBTAG00000010604	ENSBTAG00000010604	11:66566346-66575632	0.212807	0	0.532737	OK
ENSBTAG00000005754	ENSBTAG00000005754	6:37876469-37898522	0.212759	0	0.471537	OK
ENSBTAG00000008704	ENSBTAG00000008704	2:32850154-32852428	0.212641	0	0.45527	OK
ENSBTAG00000002690	ENSBTAG00000002690	16:37838603-37860891	0.212634	0	0.660258	OK
ENSBTAG00000005099	ENSBTAG00000005099	3:73263143-73263914	0.212593	0	0.805669	OK
ENSBTAG00000003897	ENSBTAG00000003897	6:46975913-46988666	0.212557	0	0.660118	OK
ENSBTAG00000020849	ENSBTAG00000020849	7:43551299-43552134	0.212538	0	0.754764	OK
ENSBTAG00000015086	ENSBTAG00000015086	16:75463611-75508770	0.212335	0	0.622416	OK
ENSBTAG00000020034	ENSBTAG00000020034	14:24847256-24920713	0.212121	0	0.436093	OK
ENSBTAG00000006676	ENSBTAG00000006676	15:58477426-58481142	0.211819	0	0.501483	OK
ENSBTAG00000021795	ENSBTAG00000021795	17:65417478-65434194	0.211693	0	0.418297	OK
ENSBTAG00000040217	ENSBTAG00000040217	3:120004689-120006101	0.21157	0	0.663644	OK
ENSBTAG00000010350	ENSBTAG00000010350	6:7151717-7207800	0.211455	0.0391708	0.548391	OK
ENSBTAG00000005240	ENSBTAG00000005240	17:44966896-44973509	0.211452	0	0.613502	OK
ENSBTAG00000037721	ENSBTAG00000037721	18:64708128-64708966	0.211395	0	0.816256	OK
ENSBTAG00000014913	ENSBTAG00000014913	6:72468364-72518226	0.211347	0	0.62055	OK
ENSBTAG00000018152	ENSBTAG00000018152	18:62018418-62024004	0.211046	0	0.369305	OK
ENSBTAG00000006200	ENSBTAG00000006200	5:112564969-112607538	0.210983	0	0.614048	OK
ENSBTAG00000003581	ENSBTAG00000003581	17:18463313-18515964	0.210956	0	0.543457	OK
ENSBTAG00000003523	ENSBTAG00000003523	6:86334722-86353208	0.210823	0	0.646245	OK
ENSBTAG00000000469	ENSBTAG00000000469	7:47425979-47450747	0.210797	0	0.63239	OK
ENSBTAG00000014215	ENSBTAG00000014215	29:37751107-37759830	0.210782	0	0.32901	OK
ENSBTAG00000024752	ENSBTAG00000024752	10:25653299-25654241	0.210768	0	0.659417	OK

ENSBTAG00000011384	ENSBTAG00000011384	17:62532617-62581166	0.210698	0	0.398186	OK
ENSBTAG00000037640	ENSBTAG00000037640	2:117836411-118104185	0.210658	0	0.492797	OK
ENSBTAG00000021410	ENSBTAG00000021410	X:4694746-4698796	0.210529	0	0.655935	OK
ENSBTAG00000019616	ENSBTAG00000019616	3:10245765-10246803	0.210508	0	0.669365	OK
ENSBTAG00000031777	ENSBTAG00000031777	15:55392274-55483132	0.210496	0	0.41798	OK
ENSBTAG00000019426	ENSBTAG00000019426	17:3880447-3946699	0.210322	0	0.397168	OK
ENSBTAG00000006354	ENSBTAG00000006354	18:39173134-39179293	0.210319	0	0.457753	OK
ENSBTAG00000023912	ENSBTAG00000023912	15:48073492-48074464	0.210278	0	0.639065	OK
ENSBTAG00000030587	ENSBTAG00000030587	19:40090994-40131373	0.210266	0	0.378829	OK
ENSBTAG00000020361	ENSBTAG00000020361	28:6762321-7195661	0.210235	0	0.421705	OK
ENSBTAG00000005732	ENSBTAG00000005732	3:110260237-110275243	0.210034	0	0.515067	OK
ENSBTAG00000011180	ENSBTAG00000011180	22:47591903-47607214	0.209814	0	0.471871	OK
ENSBTAG00000014877	ENSBTAG00000014877	11:3793439-3802385	0.209743	0	0.543457	OK
ENSBTAG00000001100	ENSBTAG00000001100	2:129351697-129378160	0.209687	0	0.373076	OK
ENSBTAG00000037384	ENSBTAG00000037384	15:45478429-45479365	0.209673	0	0.663644	OK
ENSBTAG00000015942	ENSBTAG00000015942	21:31177693-31192961	0.209672	0	0.460809	OK
ENSBTAG00000019156	ENSBTAG00000019156	5:44100434-44116239	0.209665	0	0.536973	OK
ENSBTAG00000027241	ENSBTAG00000027241	7:42868128-42869064	0.20957	0	0.663644	OK
ENSBTAG00000010562	ENSBTAG00000010562	16:77367501-77389361	0.209346	0	0.510412	OK
ENSBTAG00000021077	ENSBTAG00000021077	23:25375269-25388620	0.209326	0	0.449798	OK
ENSBTAG00000039513	ENSBTAG00000039513	23:45370138-45372316	0.209079	0	0.475337	OK
ENSBTAG00000004891	ENSBTAG00000004891	1:155017756-155158936	0.209045	0	0.445603	OK
ENSBTAG00000046618	ENSBTAG00000046618	15:48191233-48192157	0.208854	0	0.672263	OK
ENSBTAG00000048218	ENSBTAG00000048218	11:93808259-93809237	0.208848	0	0.635144	OK
ENSBTAG00000046910	ENSBTAG00000046910	4:108156936-108157815	0.208749	0	0.706679	OK
ENSBTAG00000002154	ENSBTAG00000002154	4:60030152-60031016	0.208731	0	0.718948	OK
ENSBTAG00000002011	ENSBTAG00000002011	4:61273628-61319128	0.20867	0	0.546036	OK
ENSBTAG00000008439	ENSBTAG00000008439	13:51837800-51842049	0.20857	0	0.410827	OK
ENSBTAG00000010223	ENSBTAG00000010223	5:105140258-105217724	0.208342	0	0.432269	OK
ENSBTAG00000047020	ENSBTAG00000047020	29:27903818-27904742	0.208276	0	0.672263	OK

ENSBTAG00000023652	ENSBTAG00000023652	1:37803107-37866950	0.208271	0	0.521554	OK
ENSBTAG00000001656	ENSBTAG00000001656	28:35425159-35433904	0.208205	0	0.555215	OK
ENSBTAG00000039801	ENSBTAG00000039801	15:81169227-81170247	0.208143	0	0.665778	OK
ENSBTAG00000036215	ENSBTAG00000036215	18:63602390-63604895	0.207902	0	0.647053	OK
ENSBTAG00000045565	ENSBTAG00000045565	X:83663265-83668629	0.207767	0	0.480411	OK
ENSBTAG00000039651	ENSBTAG00000039651	15:49638763-49639732	0.207562	0	0.641043	OK
ENSBTAG00000005145	ENSBTAG00000005145	21:32773321-32839248	0.207509	0.0394845	0.355361	OK
ENSBTAG00000040463	ENSBTAG00000040463	15:81218858-81220800	0.20749	0	0.663644	OK
ENSBTAG00000023106	ENSBTAG00000023106	1:140923413-140933074	0.207437	0	0.496937	OK
ENSBTAG00000014508	ENSBTAG00000014508	1:66712456-66717255	0.20742	0	0.484684	OK
ENSBTAG00000005049	ENSBTAG00000005049	8:7658876-7710709	0.207356	0	0.406592	OK
ENSBTAG00000018971	ENSBTAG00000018971	10:80248848-80631540	0.207348	0	0.548641	OK
ENSBTAG00000021755	ENSBTAG00000021755	2:60615827-60616790	0.207235	0	0.645037	OK
ENSBTAG00000032209	ENSBTAG00000032209	X:84820229-84822311	0.207143	0	0.842837	OK
ENSBTAG00000002843	ENSBTAG00000002843	10:85375285-85380533	0.207022	0	0.386541	OK
ENSBTAG00000031923	ENSBTAG00000031923	15:51375975-51376920	0.206879	0	0.657324	OK
ENSBTAG00000010368	ENSBTAG00000010368	17:68445521-68499477	0.206846	0	0.357817	OK
ENSBTAG00000047458	ENSBTAG00000047458	19:24755703-24756645	0.206715	0	0.659417	OK
ENSBTAG00000046220	ENSBTAG00000046220	3:103318856-103324197	0.206696	0	0.651123	OK
ENSBTAG00000039554	ENSBTAG00000039554	10:28047934-28049148	0.20663	0	1.0818	OK
ENSBTAG00000047662	ENSBTAG00000047662	15:49534859-49535828	0.206451	0	0.641043	OK
ENSBTAG00000020669	ENSBTAG00000020669	11:66986759-66998537	0.20645	0	0.525525	OK
ENSBTAG00000034182	ENSBTAG00000034182	1:40580692-40582048	0.206306	0	0.458091	OK
ENSBTAG00000009406	ENSBTAG00000009406	27:34243801-34245233	0.206183	0	0.745882	OK
ENSBTAG00000014127	ENSBTAG00000014127	16:69263775-69271399	0.206096	0	0.474765	OK
ENSBTAG00000011733	ENSBTAG00000011733	3:66795566-66895377	0.206059	0	0.670087	OK
ENSBTAG00000047806	ENSBTAG00000047806	2:98260689-98269171	0.205654	0	0.73165	OK
ENSBTAG00000016060	ENSBTAG00000016060	13:18496783-18563503	0.205286	0	1.02643	OK
ENSBTAG00000003751	ENSBTAG00000003751	4:28835991-28861721	0.205231	0	0.60543	OK
ENSBTAG00000004040	ENSBTAG00000004040	6:86810495-86835733	0.205134	0	0.591803	OK

ENSBTAG00000001592	ENSBTAG00000001592	4:117906797-117920021	0.205082	0	0.374832	OK
ENSBTAG00000017525	ENSBTAG00000017525	10:4619382-4799774	0.205039	0	0.679618	OK
ENSBTAG00000014396	ENSBTAG00000014396	14:63951757-63957275	0.204975	0	0.437598	OK
ENSBTAG00000012168	ENSBTAG00000012168	8:38922906-38926224	0.204836	0	0.561637	OK
ENSBTAG00000017594	ENSBTAG00000017594	13:70536646-70539478	0.204677	0	0.365567	OK
ENSBTAG00000024561	ENSBTAG00000024561	16:29832590-29840787	0.204666	0	0.592155	OK
ENSBTAG00000033476	ENSBTAG00000033476	29:28348860-28350510	0.204582	0	0.637098	OK
ENSBTAG00000014596	ENSBTAG00000014596	3:113006124-113050192	0.204575	0	0.550683	OK
ENSBTAG00000014523	ENSBTAG00000014523	8:88673820-88708956	0.20456	0	0.461563	OK
ENSBTAG00000022613	ENSBTAG00000022613	4:71186305-71189107	0.204477	0	0.639723	OK
ENSBTAG00000010555	ENSBTAG00000010555	4:55858086-55868557	0.204438	0	0.515494	OK
ENSBTAG00000023375	ENSBTAG00000023375	1:154171376-154187728	0.204327	0	0.816471	OK
ENSBTAG00000006282	ENSBTAG00000006282	15:21951909-21976900	0.204177	0	0.382447	OK
ENSBTAG00000047927	ENSBTAG00000047927	7:41662022-41662970	0.204152	0	1.09207	OK
ENSBTAG00000015226	ENSBTAG00000015226	18:62616006-62638443	0.204142	0	0.335315	OK
ENSBTAG00000013907	ENSBTAG00000013907	3:10100405-10104524	0.204112	0	0.426702	OK
ENSBTAG00000007725	ENSBTAG00000007725	7:2913859-2931918	0.204057	0	0.345671	OK
ENSBTAG00000010046	ENSBTAG00000010046	18:58212121-58220103	0.204057	0	0.517642	OK
ENSBTAG00000046229	ENSBTAG00000046229	X:50503408-50504136	0.204021	0	0.853257	OK
ENSBTAG00000003115	ENSBTAG00000003115	4:64321837-64324408	0.203694	0	0.483213	OK
ENSBTAG00000008600	ENSBTAG00000008600	9:39309719-39341546	0.203685	0	0.492758	OK
ENSBTAG00000017588	ENSBTAG00000017588	7:43654026-43655001	0.203626	0	1.07842	OK
ENSBTAG00000020958	ENSBTAG00000020958	3:53475695-53606716	0.203535	0	0.548495	OK
ENSBTAG00000002454	ENSBTAG00000002454	8:64718465-64722833	0.203336	0	0.564496	OK
ENSBTAG00000015107	ENSBTAG00000015107	3:30533844-30563298	0.203313	0	0.525859	OK
ENSBTAG00000007400	ENSBTAG00000007400	4:108052701-108053634	0.203024	0	0.665778	OK
ENSBTAG00000008873	ENSBTAG00000008873	1:72014191-72020012	0.203023	0	0.59728	OK
ENSBTAG00000046801	ENSBTAG00000046801	15:80920111-80921056	0.203008	0	0.657324	OK
ENSBTAG00000000532	ENSBTAG00000000532	3:106234353-106246150	0.202999	0	0.604546	OK
ENSBTAG00000018523	ENSBTAG00000018523	23:31693731-31708621	0.202817	0	0.58823	OK

ENSBTAG00000007581	ENSBTAG00000007581	19:34084459-34104983	0.202745	0	0.348385	OK
ENSBTAG00000026008	ENSBTAG00000026008	2:26582628-26592944	0.202706	0	1.01353	OK
ENSBTAG00000031309	ENSBTAG00000031309	15:67841772-67849540	0.202657	0	0.653692	OK
ENSBTAG00000032518	ENSBTAG00000032518	10:33950023-34069008	0.202631	0	0.523532	OK
ENSBTAG00000008639	ENSBTAG00000008639	27:16733402-16740345	0.202625	0	0.440547	OK
ENSBTAG00000013589	ENSBTAG00000013589	11:48195760-48238673	0.202598	0	0.477456	OK
ENSBTAG00000033519	ENSBTAG00000033519	15:84019178-84020268	0.202537	0	0.665778	OK
ENSBTAG00000031679	ENSBTAG00000031679	6:103971536-103991240	0.20248	0	0.712761	OK
ENSBTAG00000010149	ENSBTAG00000010149	5:70606114-70701030	0.202271	0	0.584576	OK
ENSBTAG00000006478	ENSBTAG00000006478	8:78544395-78556152	0.202148	0	0.56759	OK
ENSBTAG00000013612	ENSBTAG00000013612	7:92366906-92391409	0.202043	0	0.45187	OK
ENSBTAG00000022109	ENSBTAG00000022109	17:17627246-17639129	0.202025	0	0.65802	OK
ENSBTAG00000047672	ENSBTAG00000047672	10:21934924-21935845	0.202019	0	0.674453	OK
ENSBTAG00000006393	ENSBTAG00000006393	24:57298433-57333273	0.201843	0	0.355564	OK
ENSBTAG00000046130	ENSBTAG00000046130	29:27939102-27940218	0.201748	0	0.66082	OK
ENSBTAG00000031595	ENSBTAG00000031595	2:88810053-88850219	0.201587	0	0.62055	OK
ENSBTAG00000006549	ENSBTAG00000006549	9:34687291-34689575	0.20153	0	0.603079	OK
ENSBTAG00000011650	ENSBTAG00000011650	16:46124224-46125720	0.201471	0	0.634495	OK
ENSBTAG00000002427	ENSBTAG00000002427	X:23837350-23838406	0.201461	0	0.58823	OK
ENSBTAG00000018130	ENSBTAG00000018130	2:97843886-97988140	0.201136	0	0.426848	OK
ENSBTAG00000011389	ENSBTAG00000011389	19:57238647-57263608	0.201119	0	0.362368	OK
ENSBTAG00000038824	ENSBTAG00000038824	15:49734991-49735878	0.201118	0	1.16718	OK
ENSBTAG00000038616	ENSBTAG00000038616	22:53315512-53337107	0.201013	0	0.584356	OK
ENSBTAG00000023847	ENSBTAG00000023847	26:43118089-43134286	0.200746	0	0.586979	OK
ENSBTAG00000017682	ENSBTAG00000017682	6:21176965-21218829	0.20072	0.0351063	0.456382	OK
ENSBTAG00000010328	ENSBTAG00000010328	23:13160209-13172869	0.200626	0	0.425168	OK
ENSBTAG00000017160	ENSBTAG00000017160	23:43001021-43024073	0.20053	0	0.491433	OK
ENSBTAG00000010640	ENSBTAG00000010640	23:48923747-48924629	0.200519	0	0.704275	OK
ENSBTAG00000038075	ENSBTAG00000038075	15:50535335-50537104	0.200364	0	0.647053	OK
ENSBTAG00000038732	ENSBTAG00000038732	12:91148972-91150835	0.200249	0	0.637098	OK

ENSBTAG00000046664	ENSBTAG00000046664	12:14843775-14846181	0.200192	0	0.338882	OK
ENSBTAG00000019256	ENSBTAG00000019256	X:55224013-55234673	0.199886	0	0.959584	OK
ENSBTAG00000018400	ENSBTAG00000018400	21:46441408-46485539	0.199836	0	0.500541	OK
ENSBTAG00000009237	ENSBTAG00000009237	25:38871559-38886919	0.199809	0	0.566762	OK
ENSBTAG00000039422	ENSBTAG00000039422	7:42117711-42118719	0.199763	0	0.616241	OK
ENSBTAG00000006860	ENSBTAG00000006860	26:35006214-35047805	0.1997	0	0.389059	OK
ENSBTAG00000001027	ENSBTAG00000001027	15:55514944-55525175	0.199667	0	0.309656	OK
ENSBTAG00000000953	ENSBTAG00000000953	15:48372075-48372968	0.199635	0	0.6956	OK
ENSBTAG00000040090	ENSBTAG00000040090	29:28404692-28405661	0.199478	0	0.641043	OK
ENSBTAG00000001666	ENSBTAG00000001666	18:36607627-36610400	0.199432	0	0.624041	OK
ENSBTAG00000047235	ENSBTAG00000047235	9:104037186-104038104	0.199343	0	0.676657	OK
ENSBTAG00000008008	ENSBTAG00000008008	17:3949709-3963032	0.199082	0	0.51578	OK
ENSBTAG00000005865	ENSBTAG00000005865	10:58437864-58449901	0.199011	0	0.624842	OK
ENSBTAG00000013586	ENSBTAG00000013586	22:53613210-53614323	0.198934	0	0.558105	OK
ENSBTAG00000031632	ENSBTAG00000031632	8:75257160-75364423	0.19886	0	0.420676	OK
ENSBTAG00000016405	ENSBTAG00000016405	9:91291249-91292179	0.198837	0	0.667926	OK
ENSBTAG00000020790	ENSBTAG00000020790	26:11812675-11813475	0.198731	0	0.776464	OK
ENSBTAG00000004549	ENSBTAG00000004549	X:80939905-80970032	0.198696	0	0.549223	OK
ENSBTAG00000018067	ENSBTAG00000018067	2:121648042-121679666	0.198668	0	0.612897	OK
ENSBTAG00000015549	ENSBTAG00000015549	17:20602271-20616261	0.198571	0	0.490656	OK
ENSBTAG00000006958	ENSBTAG00000006958	23:28152664-28156380	0.19857	0	0.506252	OK
ENSBTAG00000004328	ENSBTAG00000004328	11:5469349-5488597	0.198394	0	0.423083	OK
ENSBTAG00000044105	ENSBTAG00000044105	12:21915746-22005338	0.198327	0	0.331291	OK
ENSBTAG00000003766	ENSBTAG00000003766	6:118023636-118025125	0.198269	0	0.417173	OK
ENSBTAG00000018622	ENSBTAG00000018622	22:49521551-49531507	0.197931	0	0.526594	OK
ENSBTAG00000000139	ENSBTAG00000000139	17:54457611-54476577	0.197888	0	0.791575	OK
ENSBTAG00000047489	ENSBTAG00000047489	15:50500807-50501752	0.197816	0	0.657324	OK
ENSBTAG00000035319	ENSBTAG00000035319	6:6013171-6020467	0.197739	0	0.735287	OK
ENSBTAG00000046493	ENSBTAG00000046493	20:71916824-71932650	0.19755	0	0.496937	OK
ENSBTAG00000005844	ENSBTAG00000005844	21:34205437-34230892	0.197398	0	0.417959	OK

ENSBTAG00000032393	ENSBTAG00000032393	4:73367761-73368520	0.197311	0	0.818407	OK
ENSBTAG00000038054	ENSBTAG00000038054	29:46080001-46081847	0.19731	0	0.623666	OK
ENSBTAG00000024326	ENSBTAG00000024326	X:61427737-61428592	0.197196	0	0.726516	OK
ENSBTAG00000019157	ENSBTAG00000019157	5:44094623-44097706	0.197191	0	0.537287	OK
ENSBTAG00000009621	ENSBTAG00000009621	16:41507400-41508463	0.197088	0	0.584356	OK
ENSBTAG00000038049	ENSBTAG00000038049	15:80695335-80696395	0.196995	0	0.651123	OK
ENSBTAG00000002823	ENSBTAG00000002823	3:930875-1012734	0.196968	0	0.369195	OK
ENSBTAG00000021906	ENSBTAG00000021906	1:153531716-153547327	0.196966	0	0.718449	OK
ENSBTAG00000003762	ENSBTAG00000003762	10:58291302-58324001	0.19673	0	0.507493	OK
ENSBTAG00000014766	ENSBTAG00000014766	19:9038199-9041927	0.196722	0	0.451432	OK
ENSBTAG00000013454	ENSBTAG00000013454	9:87759387-87798966	0.196682	0	0.629831	OK
ENSBTAG00000033837	ENSBTAG00000033837	3:120109577-120111016	0.196639	0	0.641043	OK
ENSBTAG00000017136	ENSBTAG00000017136	19:58718133-58719240	0.196478	0	0.56113	OK
ENSBTAG00000037942	ENSBTAG00000037942	13:74496557-74498471	0.196346	0	0.540901	OK
ENSBTAG00000000784	ENSBTAG00000000784	3:120809686-120815327	0.196109	0	0.393395	OK
ENSBTAG00000047580	ENSBTAG00000047580	X:100868720-100869764	0.196082	0	0.626812	OK
ENSBTAG00000020512	ENSBTAG00000020512	X:84715935-84723342	0.196003	0	0.390674	OK
ENSBTAG00000039772	ENSBTAG00000039772	2:119704775-119719894	0.195946	0	0.676215	OK
ENSBTAG00000010071	ENSBTAG00000010071	15:65186336-65219692	0.19585	0	0.452089	OK
ENSBTAG00000046387	ENSBTAG00000046387	15:1295524-1296403	0.195841	0	0.706679	OK
ENSBTAG00000010242	ENSBTAG00000010242	12:90609709-90621680	0.195779	0	0.303158	OK
ENSBTAG00000015868	ENSBTAG00000015868	12:87606117-87613800	0.195772	0	0.433779	OK
ENSBTAG00000039416	ENSBTAG00000039416	10:27153517-27154462	0.195605	0	0.657324	OK
ENSBTAG00000021590	ENSBTAG00000021590	15:47491601-47492582	0.195403	0	0.633202	OK
ENSBTAG00000038291	ENSBTAG00000038291	8:8603816-8614097	0.195121	0	0.707484	OK
ENSBTAG00000011094	ENSBTAG00000011094	24:18145196-18146402	0.195045	0	0.515067	OK
ENSBTAG00000021741	ENSBTAG00000021741	9:102918981-103074109	0.194999	0	0.367774	OK
ENSBTAG00000002279	ENSBTAG00000002279	19:36622183-36647084	0.194896	0	0.555112	OK
ENSBTAG00000011239	ENSBTAG00000011239	21:22754374-22787431	0.194859	0	0.566762	OK
ENSBTAG00000001855	ENSBTAG00000001855	18:25525199-25537104	0.194775	0	0.387505	OK

ENSBTAG00000000257	ENSBTAG00000000257	9:66223879-66287509	0.194717	0	0.564803	OK
ENSBTAG00000032256	ENSBTAG00000032256	15:48048942-48049743	0.194592	0	0.775494	OK
ENSBTAG00000007953	ENSBTAG00000007953	17:5298963-5332288	0.194474	0	0.482425	OK
ENSBTAG00000045899	ENSBTAG00000045899	19:24762295-24763240	0.194474	0	0.657324	OK
ENSBTAG00000007190	ENSBTAG00000007190	6:92076749-92104346	0.194376	0	0.388752	OK
ENSBTAG00000020765	ENSBTAG00000020765	3:10461144-10462662	0.194371	0	0.62555	OK
ENSBTAG00000038894	ENSBTAG00000038894	3:11354961-11355939	0.194284	0	0.635144	OK
ENSBTAG00000002092	ENSBTAG00000002092	23:10836789-10848749	0.194253	0	0.679884	OK
ENSBTAG00000046720	ENSBTAG00000046720	3:84125368-84127017	0.194214	0	0.809871	OK
ENSBTAG00000020053	ENSBTAG00000020053	13:34063174-34261299	0.194201	0	0.367339	OK
ENSBTAG00000017225	ENSBTAG00000017225	19:18330387-18343193	0.194179	0	0.441674	OK
ENSBTAG00000014011	ENSBTAG00000014011	10:58662511-58711813	0.194047	0	0.579586	OK
ENSBTAG00000031088	ENSBTAG00000031088	3:61240416-61241633	0.193954	0	0.734245	OK
ENSBTAG00000016026	ENSBTAG00000016026	1:127132776-127229651	0.193916	0	0.32488	OK
ENSBTAG00000019762	ENSBTAG00000019762	23:22220796-22222422	0.193787	0	0.764048	OK
ENSBTAG00000024915	ENSBTAG00000024915	19:50942747-50968699	0.193666	0	0.96833	OK
ENSBTAG00000008826	ENSBTAG00000008826	13:38855822-38863391	0.19357	0	0.448175	OK
ENSBTAG00000045976	ENSBTAG00000045976	9:64859551-64870020	0.193423	0	0.608991	OK
ENSBTAG00000047616	ENSBTAG00000047616	18:55439835-55444508	0.19339	0	0.496539	OK
ENSBTAG00000016547	ENSBTAG00000016547	15:14717410-14757469	0.193199	0	0.69019	OK
ENSBTAG00000010686	ENSBTAG00000010686	8:102489019-102489970	0.193151	0	0.653177	OK
ENSBTAG00000010593	ENSBTAG00000010593	6:73511905-73537572	0.193137	0	0.484722	OK
ENSBTAG00000020825	ENSBTAG00000020825	9:18690419-18703194	0.193084	0	0.664354	OK
ENSBTAG00000034676	ENSBTAG00000034676	24:8845812-8847179	0.192695	0	0.678875	OK
ENSBTAG00000001706	ENSBTAG00000001706	11:7324935-7414636	0.1926	0	0.406792	OK
ENSBTAG00000014113	ENSBTAG00000014113	19:16137673-16139707	0.192486	0	0.751114	OK
ENSBTAG00000017486	ENSBTAG00000017486	1:145518050-145555780	0.192441	0	0.382377	OK
ENSBTAG00000006243	ENSBTAG00000006243	13:59998609-60002281	0.19243	0	0.803169	OK
ENSBTAG00000012259	ENSBTAG00000012259	19:39181603-39188148	0.192289	0	0.419144	OK
ENSBTAG00000014328	ENSBTAG00000014328	18:61608936-61623944	0.192134	0	0.413013	OK

ENSBTAG00000000628	ENSBTAG00000000628	15:55850825-55853143	0.192118	0	0.457888	OK
ENSBTAG00000017138	ENSBTAG00000017138	14:9615579-9639486	0.192098	0	0.701888	OK
ENSBTAG00000008985	ENSBTAG00000008985	11:68383792-68385266	0.191982	0	0.421419	OK
ENSBTAG00000010210	ENSBTAG00000010210	13:29813364-29835882	0.191944	0	0.582275	OK
ENSBTAG00000034501	ENSBTAG00000034501	6:16765033-16815352	0.191785	0	0.493228	OK
ENSBTAG00000026624	ENSBTAG00000026624	5:85210769-85217660	0.19175	0	0.476724	OK
ENSBTAG00000046786	ENSBTAG00000046786	18:1021247-1026029	0.191588	0	0.555112	OK
ENSBTAG00000019231	ENSBTAG00000019231	2:88935943-88947500	0.191576	0	0.556605	OK
ENSBTAG00000045702	ENSBTAG00000045702	16:1400456-1403399	0.191453	0	0.562846	OK
ENSBTAG00000002827	ENSBTAG00000002827	9:97466404-97481898	0.191415	0	0.701888	OK
ENSBTAG00000007873	ENSBTAG00000007873	5:58618445-58619384	0.191264	0	0.661524	OK
ENSBTAG00000023938	ENSBTAG00000023938	11:72781246-72787461	0.191067	0	0.625172	OK
ENSBTAG00000018493	ENSBTAG00000018493	14:68428889-68454310	0.190915	0	0.41439	OK
ENSBTAG00000048220	ENSBTAG00000048220	15:49632146-49633099	0.190835	0	0.651806	OK
ENSBTAG00000005492	ENSBTAG00000005492	13:18359844-18395890	0.190764	0	0.421133	OK
ENSBTAG00000005928	ENSBTAG00000005928	26:9170250-9234323	0.190669	0	0.734403	OK
ENSBTAG00000046460	ENSBTAG00000046460	15:83156499-83157432	0.190597	0	0.665778	OK
ENSBTAG00000005682	ENSBTAG00000005682	12:80056875-80227030	0.190557	0	0.481752	OK
ENSBTAG00000045520	ENSBTAG00000045520	13:53605291-53619401	0.19046	0	0.49476	OK
ENSBTAG00000005905	ENSBTAG00000005905	8:11391202-11401136	0.1904	0	0.474297	OK
ENSBTAG00000009476	ENSBTAG00000009476	X:135366418-135410968	0.19028	0	0.511757	OK
ENSBTAG00000014082	ENSBTAG00000014082	24:30037088-30315707	0.190197	0	0.62897	OK
ENSBTAG00000040016	ENSBTAG00000040016	15:206551-207487	0.190171	0	1.12409	OK
ENSBTAG00000009980	ENSBTAG00000009980	21:50392542-50393631	0.190002	0	0.610787	OK
ENSBTAG00000006876	ENSBTAG00000006876	4:44755330-44769491	0.189954	0	0.643434	OK
ENSBTAG00000046315	ENSBTAG00000046315	3:23537154-23539207	0.189789	0	0.509741	OK
ENSBTAG00000021345	ENSBTAG00000021345	12:78063843-78064857	0.189787	0	0.612595	OK
ENSBTAG00000020762	ENSBTAG00000020762	1:2781251-2919429	0.189561	0	0.29081	OK
ENSBTAG00000039329	ENSBTAG00000039329	9:88232043-88402262	0.189484	0	0.62492	OK
ENSBTAG00000024139	ENSBTAG00000024139	13:216568-217741	0.189392	0	0.655244	OK

ENSBTAG00000004176	ENSBTAG00000004176	16:69009429-69013701	0.189089	0	0.62055	OK
ENSBTAG00000046634	ENSBTAG00000046634	22:23847-26284	0.189015	0	0.634495	OK
ENSBTAG00000046277	ENSBTAG00000046277	3:6287011-6292402	0.188894	0	0.602494	OK
ENSBTAG00000012437	ENSBTAG00000012437	3:11009681-11010572	0.188874	0	0.697161	OK
ENSBTAG00000010552	ENSBTAG00000010552	24:43515582-43525527	0.188829	0	0.637752	OK
ENSBTAG00000025266	ENSBTAG00000025266	15:48706647-48709290	0.188825	0	0.437013	OK
ENSBTAG00000019553	ENSBTAG00000019553	X:3481954-3508062	0.188722	0	0.543278	OK
ENSBTAG00000034991	ENSBTAG00000034991	13:3737181-3815751	0.188717	0	0.514214	OK
ENSBTAG00000040019	ENSBTAG00000040019	5:27594460-27599306	0.188697	0	0.371514	OK
ENSBTAG00000009566	ENSBTAG00000009566	7:89393227-89423692	0.188671	0	0.525081	OK
ENSBTAG00000010341	ENSBTAG00000010341	7:73779311-73797972	0.188565	0	0.456878	OK
ENSBTAG00000038822	ENSBTAG00000038822	11:93835431-93836470	0.188492	0	0.597855	OK
ENSBTAG00000011908	ENSBTAG00000011908	14:69287301-69893052	0.188472	0	0.528476	OK
ENSBTAG00000005284	ENSBTAG00000005284	12:20856478-20887977	0.188365	0	0.404672	OK
ENSBTAG00000010385	ENSBTAG00000010385	1:117252851-117253808	0.188362	0	0.649081	OK
ENSBTAG00000038579	ENSBTAG00000038579	15:50562716-50563915	0.188103	0	0.651123	OK
ENSBTAG00000045782	ENSBTAG00000045782	X:93971183-93977809	0.188096	0	0.524195	OK
ENSBTAG00000001961	ENSBTAG00000001961	20:9330174-9419040	0.188024	0.0277966	0.389153	OK
ENSBTAG00000048310	ENSBTAG00000048310	15:49330069-49331031	0.187947	0	0.645708	OK
ENSBTAG00000008307	ENSBTAG00000008307	1:112962333-113071514	0.187814	0	0.375629	OK
ENSBTAG00000045539	ENSBTAG00000045539	7:53603718-53606070	0.187795	0	0.352138	OK
ENSBTAG00000002167	ENSBTAG00000002167	16:62225041-62244587	0.18776	0	0.526862	OK
ENSBTAG00000004760	ENSBTAG00000004760	28:42248869-42250003	0.187686	0	0.54777	OK
ENSBTAG00000030960	ENSBTAG00000030960	8:102759007-102776534	0.187573	0	0.429282	OK
ENSBTAG00000000985	ENSBTAG00000000985	11:73335435-73400455	0.187563	0	0.546324	OK
ENSBTAG00000027316	ENSBTAG00000027316	13:78845235-78873171	0.187543	0	0.567902	OK
ENSBTAG00000006738	ENSBTAG00000006738	21:56588075-56591240	0.187538	0	0.327104	OK
ENSBTAG00000020434	ENSBTAG00000020434	18:40455365-40467458	0.187522	0	0.423256	OK
ENSBTAG00000017043	ENSBTAG00000017043	2:94940391-94941459	0.18742	0	0.581621	OK
ENSBTAG00000025314	ENSBTAG00000025314	23:34431088-34440610	0.187393	0	0.697945	OK

ENSBTAG00000040064	ENSBTAG00000040064	23:29593122-29594291	0.187251	0	1.09554	OK
ENSBTAG00000037992	ENSBTAG00000037992	15:48863185-48864151	0.187214	0	0.643034	OK
ENSBTAG00000013468	ENSBTAG00000013468	29:41576804-41605018	0.18715	0.0616645	0.320655	OK
ENSBTAG00000033504	ENSBTAG00000033504	27:28406556-28416649	0.187131	0	0.607502	OK
ENSBTAG00000021543	ENSBTAG00000021543	4:53756423-53854749	0.187066	0	0.452584	OK
ENSBTAG00000047850	ENSBTAG00000047850	15:45558537-45559476	0.187036	0	0.661524	OK
ENSBTAG00000014620	ENSBTAG00000014620	20:1903853-1907281	0.187024	0	0.340928	OK
ENSBTAG00000045959	ENSBTAG00000045959	15:49383156-49384116	0.186976	0	0.647053	OK
ENSBTAG00000000872	ENSBTAG00000000872	6:113329381-113331129	0.186855	0	1.02809	OK
ENSBTAG00000004098	ENSBTAG00000004098	9:89649144-89662655	0.186767	0	0.551172	OK
ENSBTAG00000047756	ENSBTAG00000047756	19:28224424-28238427	0.186605	0	0.340891	OK
ENSBTAG00000036013	ENSBTAG00000036013	8:96092373-96093327	0.186269	0	0.651123	OK
ENSBTAG00000021596	ENSBTAG00000021596	2:92564233-92587607	0.18597	0	0.898945	OK
ENSBTAG00000010291	ENSBTAG00000010291	25:39080279-39089918	0.185906	0	0.429876	OK
ENSBTAG00000001696	ENSBTAG00000001696	17:2523017-2567620	0.185846	0	0.529783	OK
ENSBTAG00000000252	ENSBTAG00000000252	8:104356461-104359086	0.185782	0	0.543742	OK
ENSBTAG00000046187	ENSBTAG00000046187	15:81119008-81119949	0.185739	0	0.660118	OK
ENSBTAG00000009873	ENSBTAG00000009873	23:48868150-48877436	0.185342	0	0.402313	OK
ENSBTAG00000005914	ENSBTAG00000005914	15:79135785-79136715	0.18524	0	0.667926	OK
ENSBTAG00000015517	ENSBTAG00000015517	16:353115-354883	0.185197	0	0.641043	OK
ENSBTAG00000025009	ENSBTAG00000025009	19:38503101-38507053	0.184787	0	0.472014	OK
ENSBTAG00000048208	ENSBTAG00000048208	7:10012564-10013512	0.184695	0	0.655244	OK
ENSBTAG00000046441	ENSBTAG00000046441	15:48747045-48748005	0.18468	0	0.647053	OK
ENSBTAG00000034601	ENSBTAG00000034601	3:11029724-11030663	0.18466	0	0.661524	OK
ENSBTAG00000007256	ENSBTAG00000007256	5:46323522-46333943	0.184565	0	0.345095	OK
ENSBTAG00000010397	ENSBTAG00000010397	1:84366237-84428658	0.184444	0	0.570667	OK
ENSBTAG00000009050	ENSBTAG00000009050	5:108705364-108724898	0.184438	0	0.374651	OK
ENSBTAG00000013593	ENSBTAG00000013593	21:29683788-29696818	0.184379	0	0.545844	OK
ENSBTAG00000002792	ENSBTAG00000002792	28:29833270-29841130	0.184284	0	0.348776	OK
ENSBTAG00000047802	ENSBTAG00000047802	10:27462668-27463598	0.184244	0	0.667926	OK

ENSBTAG00000007029	ENSBTAG00000007029	10:25594156-25595098	0.184113	0	0.659417	OK
ENSBTAG00000019093	ENSBTAG00000019093	3:39926297-39948945	0.184103	0	0.54748	OK
ENSBTAG00000019549	ENSBTAG00000019549	15:48900172-48901135	0.1841	0	0.645037	OK
ENSBTAG00000027547	ENSBTAG00000027547	23:31250285-31252079	0.18409	0	0.920449	OK
ENSBTAG00000046953	ENSBTAG00000046953	7:44130118-44131060	0.184067	0	0.659417	OK
ENSBTAG00000027080	ENSBTAG00000027080	10:82128378-82273227	0.184018	0	0.37187	OK
ENSBTAG00000046006	ENSBTAG00000046006	9:62659095-62660514	0.183983	0	0.437753	OK
ENSBTAG00000022384	ENSBTAG00000022384	29:27150151-27151084	0.183759	0	0.665778	OK
ENSBTAG00000044035	ENSBTAG00000044035	7:39423155-39426047	0.183644	0	0.357982	OK
ENSBTAG00000006562	ENSBTAG00000006562	27:24801787-24804337	0.183544	0	0.324795	OK
ENSBTAG00000030845	ENSBTAG00000030845	15:83667091-83671187	0.183347	0	0.454959	OK
ENSBTAG00000003506	ENSBTAG00000003506	4:74886595-74911776	0.183263	0	0.51894	OK
ENSBTAG00000003625	ENSBTAG00000003625	4:407816-432247	0.183189	0	0.506995	OK
ENSBTAG00000002125	ENSBTAG00000002125	24:43111843-43163936	0.182941	0	0.391535	OK
ENSBTAG00000039354	ENSBTAG00000039354	10:27944301-27945240	0.182919	0	0.661524	OK
ENSBTAG00000009044	ENSBTAG00000009044	15:47981947-47982913	0.182794	0	0.643034	OK
ENSBTAG00000012184	ENSBTAG00000012184	7:74119594-74127285	0.18276	0	0.776464	OK
ENSBTAG00000007122	ENSBTAG00000007122	7:2371312-2394402	0.182636	0	0.536881	OK
ENSBTAG00000017905	ENSBTAG00000017905	4:99724861-99750236	0.182625	0	0.317694	OK
ENSBTAG00000047716	ENSBTAG00000047716	29:28207496-28208432	0.182598	0	0.663644	OK
ENSBTAG00000001939	ENSBTAG00000001939	8:90264886-90298568	0.182475	0	0.454904	OK
ENSBTAG00000033874	ENSBTAG00000033874	X:32590618-32592442	0.182376	0	0.613805	OK
ENSBTAG00000031027	ENSBTAG00000031027	15:79238961-79239891	0.182283	0	0.667926	OK
ENSBTAG00000036042	ENSBTAG00000036042	15:83036767-83037703	0.182267	0	0.663644	OK
ENSBTAG00000005996	ENSBTAG00000005996	28:43603726-43620831	0.182179	0	0.521554	OK
ENSBTAG00000040349	ENSBTAG00000040349	4:89053953-89070650	0.182137	0	0.715965	OK
ENSBTAG00000006775	ENSBTAG00000006775	20:71395141-71420351	0.182072	0	0.360816	OK
ENSBTAG00000013662	ENSBTAG00000013662	1:43541935-43717619	0.18204	0	0.364894	OK
ENSBTAG00000017368	ENSBTAG00000017368	3:104112933-104133708	0.181887	0	0.407058	OK
ENSBTAG00000037464	ENSBTAG00000037464	13:53169425-53196424	0.181862	0	0.291219	OK

ENSBTAG00000039157	ENSBTAG00000039157	5:56773335-56775425	0.18182	0	0.393644	OK
ENSBTAG00000047611	ENSBTAG00000047611	22:16612730-16613704	0.181602	0	0.706679	OK
ENSBTAG00000016421	ENSBTAG00000016421	9:101442937-101443961	0.181583	0	0.606612	OK
ENSBTAG00000001265	ENSBTAG00000001265	1:81127290-81135756	0.181513	0	0.626686	OK
ENSBTAG00000019009	ENSBTAG00000019009	X:28318645-28319668	0.181431	0	0.607205	OK
ENSBTAG00000010458	ENSBTAG00000010458	3:8879269-8892720	0.181364	0	0.437136	OK
ENSBTAG00000010843	ENSBTAG00000010843	17:29872405-29890867	0.180986	0	0.565112	OK
ENSBTAG00000048307	ENSBTAG00000048307	15:49833388-49834463	0.180916	0	0.638408	OK
ENSBTAG00000046137	ENSBTAG00000046137	11:93313594-93314530	0.180879	0	0.663644	OK
ENSBTAG00000031941	ENSBTAG00000031941	18:44018757-44137173	0.180842	0	0.327104	OK
ENSBTAG00000031247	ENSBTAG00000031247	25:36267194-36276308	0.180715	0	0.542144	OK
ENSBTAG00000001949	ENSBTAG00000001949	11:1953346-1958756	0.180575	0	1.07269	OK
ENSBTAG00000033480	ENSBTAG00000033480	29:28157850-28158795	0.18055	0	0.657324	OK
ENSBTAG00000024219	ENSBTAG00000024219	4:106996939-107013515	0.180548	0	0.326717	OK
ENSBTAG00000037434	ENSBTAG00000037434	7:88995195-88995993	0.180467	0	0.77841	OK
ENSBTAG00000045744	ENSBTAG00000045744	X:3791620-3806381	0.180329	0	0.40574	OK
ENSBTAG00000046126	ENSBTAG00000046126	11:93609987-93610929	0.180198	0	0.659417	OK
ENSBTAG00000003758	ENSBTAG00000003758	22:48264510-48288061	0.180071	0	0.900355	OK
ENSBTAG00000045714	ENSBTAG00000045714	6:104273699-104278722	0.179897	0	0.506004	OK
ENSBTAG00000039602	ENSBTAG00000039602	1:4775169-4776014	0.179313	0	0.735113	OK
ENSBTAG00000002043	ENSBTAG00000002043	15:48405342-48406284	0.179254	0	0.659417	OK
ENSBTAG00000007605	ENSBTAG00000007605	5:107445595-107453517	0.179178	0	0.296502	OK
ENSBTAG00000024607	ENSBTAG00000024607	5:58883729-58884668	0.17903	0	0.661524	OK
ENSBTAG00000004331	ENSBTAG00000004331	3:15682354-15686554	0.179003	0	0.332996	OK
ENSBTAG00000047825	ENSBTAG00000047825	5:58419456-58420410	0.178781	0	0.651123	OK
ENSBTAG00000039116	ENSBTAG00000039116	23:29143511-29144489	0.17861	0	0.635144	OK
ENSBTAG00000032224	ENSBTAG00000032224	15:48738467-48739424	0.178439	0	0.649081	OK
ENSBTAG00000005712	ENSBTAG00000005712	6:106365303-106482788	0.178434	0	0.409689	OK
ENSBTAG00000008792	ENSBTAG00000008792	10:26402506-26404000	0.178365	0	0.644368	OK
ENSBTAG00000027625	ENSBTAG00000027625	27:35900361-35943920	0.17821	0	0.342972	OK

ENSBTAG00000046645	ENSBTAG00000046645	5:58976108-58977044	0.17821	0	0.663644	OK
ENSBTAG00000012339	ENSBTAG00000012339	7:54986240-54988121	0.178129	0	0.667208	OK
ENSBTAG00000000300	ENSBTAG00000000300	X:81474206-81480046	0.178073	0	0.546685	OK
ENSBTAG00000012676	ENSBTAG00000012676	5:27271879-27282427	0.177861	0	0.298496	OK
ENSBTAG00000016943	ENSBTAG00000016943	16:79057372-79136394	0.177828	0	0.64976	OK
ENSBTAG00000031030	ENSBTAG00000031030	15:79267159-79268059	0.177825	0	0.69019	OK
ENSBTAG00000038490	ENSBTAG00000038490	5:102076230-102089108	0.17775	0	0.621419	OK
ENSBTAG00000004409	ENSBTAG00000004409	10:36140625-36152399	0.177691	0	0.360726	OK
ENSBTAG00000046462	ENSBTAG00000046462	8:56547893-56552112	0.177673	0	0.330234	OK
ENSBTAG00000005376	ENSBTAG00000005376	9:84176751-84182674	0.177655	0	0.487575	OK
ENSBTAG00000010279	ENSBTAG00000010279	19:14096512-14122123	0.177642	0	0.421133	OK
ENSBTAG00000007110	ENSBTAG00000007110	16:73955569-74006866	0.177551	0	0.524726	OK
ENSBTAG00000013412	ENSBTAG00000013412	18:36847729-36898690	0.177519	0	0.549952	OK
ENSBTAG00000010114	ENSBTAG00000010114	X:50449122-50450786	0.177416	0	0.633589	OK
ENSBTAG00000045511	ENSBTAG00000045511	24:314606-317002	0.177379	0	0.647728	OK
ENSBTAG00000013654	ENSBTAG00000013654	23:29206926-29207889	0.177084	0	0.645037	OK
ENSBTAG00000045527	ENSBTAG00000045527	11:93678032-93678974	0.177044	0	0.659417	OK
ENSBTAG00000026673	ENSBTAG00000026673	9:34316844-34358840	0.177041	0	0.605076	OK
ENSBTAG00000025165	ENSBTAG00000025165	19:46947230-46949138	0.17701	0	0.550391	OK
ENSBTAG00000033041	ENSBTAG00000033041	11:14919595-14937163	0.17696	0	0.659417	OK
ENSBTAG00000010597	ENSBTAG00000010597	4:66204845-66214292	0.176887	0	0.515494	OK
ENSBTAG00000045558	ENSBTAG00000045558	15:248024-248981	0.176839	0	0.649081	OK
ENSBTAG00000033486	ENSBTAG00000033486	17:41307377-41310601	0.176717	0	0.532007	OK
ENSBTAG00000032799	ENSBTAG00000032799	10:26932004-26932931	0.17662	0	0.670087	OK
ENSBTAG00000010526	ENSBTAG00000010526	20:23658801-23793336	0.176416	0	0.408934	OK
ENSBTAG00000026548	ENSBTAG00000026548	9:71628868-71629939	0.176408	0	0.579991	OK
ENSBTAG00000014579	ENSBTAG00000014579	7:5741607-5742859	0.176286	0	0.595562	OK
ENSBTAG00000047448	ENSBTAG00000047448	25:26734079-26736769	0.176151	0	0.306751	OK
ENSBTAG00000005300	ENSBTAG00000005300	16:53534181-53538875	0.175974	0	0.392649	OK
ENSBTAG00000013750	ENSBTAG00000013750	28:8232078-8297594	0.175942	0	0.879709	OK

ENSBTAG00000036025	ENSBTAG00000036025	15:1207714-1208639	0.175894	0	0.692498	OK
ENSBTAG00000025379	ENSBTAG00000025379	22:14972008-14972887	0.175877	0	0.718948	OK
ENSBTAG00000047396	ENSBTAG00000047396	15:82511486-82512419	0.175657	0	0.665778	OK
ENSBTAG00000040252	ENSBTAG00000040252	15:45198362-45199307	0.175601	0	0.657324	OK
ENSBTAG00000020264	ENSBTAG00000020264	16:37875177-37890412	0.175494	0	0.753482	OK
ENSBTAG00000003910	ENSBTAG00000003910	28:36127030-36212116	0.175472	0	0.877359	OK
ENSBTAG00000038903	ENSBTAG00000038903	18:58680350-58682409	0.17541	0	0.61808	OK
ENSBTAG00000006753	ENSBTAG00000006753	17:45254529-45256157	0.175364	0	0.587118	OK
ENSBTAG00000019892	ENSBTAG00000019892	14:19703610-19733798	0.175312	0	0.426775	OK
ENSBTAG00000018873	ENSBTAG00000018873	13:73617658-73627421	0.175204	0	0.424123	OK
ENSBTAG00000047244	ENSBTAG00000047244	7:10687059-10688016	0.175026	0	0.649081	OK
ENSBTAG00000046034	ENSBTAG00000046034	7:40467798-40469181	0.175012	0	0.449147	OK
ENSBTAG00000038537	ENSBTAG00000038537	20:13233-15549	0.174938	0	0.638408	OK
ENSBTAG00000007323	ENSBTAG00000007323	5:44765460-44793485	0.174894	0	0.487652	OK
ENSBTAG00000047016	ENSBTAG00000047016	7:42811930-42813032	0.174893	0	0.665778	OK
ENSBTAG00000043558	ENSBTAG00000043558	MT:3100-4056	0.174823	0	0.64976	OK
ENSBTAG00000040236	ENSBTAG00000040236	15:49562174-49563131	0.174822	0	0.649081	OK
ENSBTAG00000019983	ENSBTAG00000019983	11:73602729-73638589	0.174659	0	0.393271	OK
ENSBTAG00000001803	ENSBTAG00000001803	9:53977188-54030371	0.174636	0	0.577296	OK
ENSBTAG00000020916	ENSBTAG00000020916	5:101871072-101876226	0.174601	0	0.63867	OK
ENSBTAG00000038319	ENSBTAG00000038319	15:212768-213701	0.174559	0	0.665778	OK
ENSBTAG00000021519	ENSBTAG00000021519	7:47837917-47845672	0.174493	0	0.457079	OK
ENSBTAG00000039785	ENSBTAG00000039785	10:26924386-26925352	0.174487	0	0.643034	OK
ENSBTAG00000021938	ENSBTAG00000021938	19:31913650-31965004	0.174396	0	0.338743	OK
ENSBTAG00000013007	ENSBTAG00000013007	13:60109035-60119697	0.174332	0	0.429727	OK
ENSBTAG00000048094	ENSBTAG00000048094	21:61687615-61694371	0.174323	0	0.434082	OK
ENSBTAG00000021773	ENSBTAG00000021773	25:12923481-12961007	0.174178	0	0.37633	OK
ENSBTAG00000037657	ENSBTAG00000037657	15:27816255-27827640	0.174176	0	0.473453	OK
ENSBTAG00000004059	ENSBTAG00000004059	16:62068138-62128359	0.174145	0	0.465297	OK
ENSBTAG00000037517	ENSBTAG00000037517	22:63639-65183	0.174113	0	0.660118	OK

ENSBTAG00000006601	ENSBTAG00000006601	19:19548866-19618012	0.174093	0	0.315997	OK
ENSBTAG00000020861	ENSBTAG00000020861	12:91100866-91103281	0.174084	0	0.514427	OK
ENSBTAG00000015716	ENSBTAG00000015716	10:11438272-11488427	0.174022	0	0.519461	OK
ENSBTAG00000021731	ENSBTAG00000021731	11:9200409-9227574	0.17393	0	0.33403	OK
ENSBTAG00000006745	ENSBTAG00000006745	17:2823020-2830960	0.173791	0	0.601315	OK
ENSBTAG00000039321	ENSBTAG00000039321	19:43562850-43571700	0.173551	0	0.329184	OK
ENSBTAG00000038629	ENSBTAG00000038629	7:41307714-41308653	0.173513	0	0.661524	OK
ENSBTAG00000017060	ENSBTAG00000017060	1:145104445-145133581	0.173133	0	0.296219	OK
ENSBTAG00000013042	ENSBTAG00000013042	10:69862216-69914486	0.172931	0	0.448823	OK
ENSBTAG00000039986	ENSBTAG00000039986	7:42084485-42085424	0.172914	0	0.661524	OK
ENSBTAG00000008787	ENSBTAG00000008787	13:30069066-30123389	0.172893	0	0.34196	OK
ENSBTAG00000047914	ENSBTAG00000047914	29:50896191-50902677	0.172831	0	0.330103	OK
ENSBTAG00000007306	ENSBTAG00000007306	20:20448539-20741101	0.172819	0	0.915259	OK
ENSBTAG00000000845	ENSBTAG00000000845	3:41420908-41642403	0.172786	0	0.534203	OK
ENSBTAG00000032276	ENSBTAG00000032276	15:47629005-47629947	0.172652	0	0.659417	OK
ENSBTAG00000026972	ENSBTAG00000026972	5:10339424-10342660	0.172576	0	0.522871	OK
ENSBTAG00000031913	ENSBTAG00000031913	23:27614102-27615194	0.17255	0	0.568838	OK
ENSBTAG00000046489	ENSBTAG00000046489	15:48658918-48659848	0.172529	0	0.667926	OK
ENSBTAG00000046104	ENSBTAG00000046104	1:107212960-107213956	0.172421	0	0.623666	OK
ENSBTAG00000047728	ENSBTAG00000047728	11:93646063-93647008	0.17241	0	0.657324	OK
ENSBTAG00000047689	ENSBTAG00000047689	19:9276038-9276974	0.17239	0	0.663644	OK
ENSBTAG00000018519	ENSBTAG00000018519	19:14939571-14946571	0.1723	0	0.315155	OK
ENSBTAG00000003056	ENSBTAG00000003056	15:79864247-79865213	0.172222	0	0.643034	OK
ENSBTAG00000035007	ENSBTAG00000035007	8:3816703-5330369	0.172098	0	0.343948	OK
ENSBTAG00000020987	ENSBTAG00000020987	X:126856699-126939882	0.172093	0	0.388183	OK
ENSBTAG00000020660	ENSBTAG00000020660	11:93702913-93703858	0.172091	0	0.657324	OK
ENSBTAG00000001649	ENSBTAG00000001649	14:60994138-61520208	0.17209	0	0.358955	OK
ENSBTAG00000039878	ENSBTAG00000039878	11:10812245-10825732	0.172043	0	0.480969	OK
ENSBTAG00000015319	ENSBTAG00000015319	14:18158509-18173288	0.172027	0	0.495747	OK
ENSBTAG00000017781	ENSBTAG00000017781	5:30211621-30217104	0.172002	0	0.402573	OK

ENSBTAG00000016063	ENSBTAG00000016063	2:118194130-118436930	0.171941	0	0.295796	OK
ENSBTAG00000000926	ENSBTAG00000000926	15:79150672-79151599	0.171914	0	0.670087	OK
ENSBTAG00000039405	ENSBTAG00000039405	15:45175107-45176052	0.171911	0	0.657324	OK
ENSBTAG00000021398	ENSBTAG00000021398	9:43435673-43446267	0.171883	0	0.436522	OK
ENSBTAG00000019341	ENSBTAG00000019341	19:21799219-21844511	0.171846	0	0.512771	OK
ENSBTAG00000017824	ENSBTAG00000017824	18:11883375-11905043	0.171845	0	0.284289	OK
ENSBTAG00000046610	ENSBTAG00000046610	5:59971693-59972920	0.171506	0	0.647053	OK
ENSBTAG00000045733	ENSBTAG00000045733	7:42890344-42891283	0.171421	0	0.661524	OK
ENSBTAG00000047625	ENSBTAG00000047625	7:64773692-64778702	0.171236	0	0.348581	OK
ENSBTAG00000009243	ENSBTAG00000009243	9:39618966-39627196	0.171165	0	0.424297	OK
ENSBTAG00000016446	ENSBTAG00000016446	27:32885369-32890975	0.170991	0	0.450777	OK
ENSBTAG00000037474	ENSBTAG00000037474	15:50741420-50742362	0.170589	0	0.659417	OK
ENSBTAG00000020090	ENSBTAG00000020090	7:8911329-8916078	0.170446	0	0.354145	OK
ENSBTAG00000003595	ENSBTAG00000003595	12:12964734-12975239	0.170429	0	0.43868	OK
ENSBTAG00000021588	ENSBTAG00000021588	2:112549868-112555375	0.170399	0	0.52597	OK
ENSBTAG00000021135	ENSBTAG00000021135	8:78599029-78600903	0.17035	0	0.662936	OK
ENSBTAG00000019345	ENSBTAG00000019345	8:52737521-52743129	0.170183	0	0.550683	OK
ENSBTAG00000040384	ENSBTAG00000040384	10:52648526-52655730	0.170037	0	0.422853	OK
ENSBTAG00000039274	ENSBTAG00000039274	23:30695955-30696900	0.169996	0	0.657324	OK
ENSBTAG00000004352	ENSBTAG00000004352	11:44876760-44897731	0.169748	0	0.296502	OK
ENSBTAG00000014249	ENSBTAG00000014249	16:67765815-67785616	0.169632	0	0.848161	OK
ENSBTAG00000037875	ENSBTAG00000037875	15:50185532-50186486	0.169624	0	0.651123	OK
ENSBTAG00000013156	ENSBTAG00000013156	15:1123538-1124441	0.169415	0	0.687897	OK
ENSBTAG00000006733	ENSBTAG00000006733	4:54866420-54906096	0.169211	0	0.557771	OK
ENSBTAG00000033460	ENSBTAG00000033460	9:34300227-34313235	0.169114	0	0.489496	OK
ENSBTAG00000019660	ENSBTAG00000019660	19:44629958-44633906	0.169105	0	0.332533	OK
ENSBTAG00000024210	ENSBTAG00000024210	X:15389373-15390372	0.169026	0	0.621793	OK
ENSBTAG00000012272	ENSBTAG00000012272	5:1303244-1321239	0.169005	0	0.404014	OK
ENSBTAG00000009654	ENSBTAG00000009654	8:102051153-102141484	0.168948	0	0.401014	OK
ENSBTAG00000002913	ENSBTAG00000002913	5:59432557-59433496	0.168839	0	0.661524	OK

ENSBTAG00000046251	ENSBTAG00000046251	15:79972833-79974879	0.168715	0	0.682605	OK
ENSBTAG00000026994	ENSBTAG00000026994	2:6038402-6113008	0.168369	0	1.07098	OK
ENSBTAG00000038761	ENSBTAG00000038761	9:71719615-71720955	0.168069	0	0.840346	OK
ENSBTAG00000047886	ENSBTAG00000047886	5:59852495-59853470	0.167997	0	0.637098	OK
ENSBTAG00000000162	ENSBTAG00000000162	28:28446890-28457153	0.167995	0	0.508323	OK
ENSBTAG00000014726	ENSBTAG00000014726	16:46455182-46464710	0.167855	0	0.479299	OK
ENSBTAG00000040020	ENSBTAG00000040020	29:26774295-26775286	0.167765	0	0.639065	OK
ENSBTAG00000040040	ENSBTAG00000040040	4:107447956-107449330	0.167723	0	0.639723	OK
ENSBTAG00000037611	ENSBTAG00000037611	7:42365707-42366655	0.167581	0	0.655244	OK
ENSBTAG00000038362	ENSBTAG00000038362	3:11276411-11277356	0.167466	0	0.657324	OK
ENSBTAG00000016599	ENSBTAG00000016599	11:10141775-10144861	0.167383	0	0.428246	OK
ENSBTAG00000039120	ENSBTAG00000039120	10:27178465-27179998	0.167333	0	0.649081	OK
ENSBTAG00000013841	ENSBTAG00000013841	1:459811-460830	0.167074	0	0.609589	OK
ENSBTAG00000001044	ENSBTAG00000001044	28:25712750-25737804	0.167035	0	0.435176	OK
ENSBTAG00000047327	ENSBTAG00000047327	9:71483874-71484900	0.166944	0	0.60543	OK
ENSBTAG00000046527	ENSBTAG00000046527	15:80244343-80245270	0.166886	0	0.670087	OK
ENSBTAG00000000351	ENSBTAG00000000351	15:47753978-47754920	0.166839	0	0.659417	OK
ENSBTAG00000002333	ENSBTAG00000002333	6:73639369-73648429	0.166785	0	0.642369	OK
ENSBTAG00000008236	ENSBTAG00000008236	15:50940887-50941850	0.166658	0	0.645037	OK
ENSBTAG00000015366	ENSBTAG00000015366	4:50009881-50020466	0.166645	0	0.377611	OK
ENSBTAG00000021602	ENSBTAG00000021602	3:30987501-31026962	0.166613	0	0.393769	OK
ENSBTAG00000019703	ENSBTAG00000019703	X:59804942-59827385	0.166406	0	0.498212	OK
ENSBTAG00000012545	ENSBTAG00000012545	10:103802802-103891475	0.166206	0	0.512042	OK
ENSBTAG00000026611	ENSBTAG00000026611	5:88504327-88530297	0.166194	0	0.723975	OK
ENSBTAG00000000551	ENSBTAG00000000551	26:31141808-31152181	0.16617	0	0.557104	OK
ENSBTAG00000012117	ENSBTAG00000012117	X:40402700-40410481	0.166164	0	0.295374	OK
ENSBTAG00000005087	ENSBTAG00000005087	15:79852684-79853677	0.166144	0	0.62555	OK
ENSBTAG00000048072	ENSBTAG00000048072	7:10355258-10356194	0.166137	0	0.663644	OK
ENSBTAG00000036271	ENSBTAG00000036271	28:2500073-2501037	0.165985	0	0.65249	OK
ENSBTAG00000003504	ENSBTAG00000003504	13:64842137-64867355	0.165957	0	0.321517	OK

ENSBTAG00000016004	ENSBTAG00000016004	6:26925348-26979063	0.165892	0	0.385581	OK
ENSBTAG00000009169	ENSBTAG00000009169	16:63299969-63502107	0.165856	0	0.446821	OK
ENSBTAG00000011010	ENSBTAG00000011010	13:47444351-47449390	0.165824	0	0.500742	OK
ENSBTAG00000038174	ENSBTAG00000038174	15:42515027-42516593	0.165752	0	0.661101	OK
ENSBTAG00000001861	ENSBTAG00000001861	2:17708889-17730680	0.165671	0	0.480411	OK
ENSBTAG00000031354	ENSBTAG00000031354	13:63346797-63361747	0.165654	0	0.603313	OK
ENSBTAG00000003906	ENSBTAG00000003906	11:72021272-72033985	0.165571	0	0.398059	OK
ENSBTAG00000007131	ENSBTAG00000007131	22:5258462-5452369	0.165471	0	0.305935	OK
ENSBTAG00000005493	ENSBTAG00000005493	6:118212843-118312151	0.165434	0	0.322089	OK
ENSBTAG00000002594	ENSBTAG00000002594	2:130078396-130084459	0.165393	0	0.413562	OK
ENSBTAG00000032548	ENSBTAG00000032548	14:56909761-56997967	0.165199	0	0.825995	OK
ENSBTAG00000047945	ENSBTAG00000047945	15:50213808-50214759	0.165182	0	0.653177	OK
ENSBTAG00000011131	ENSBTAG00000011131	7:65631968-65649628	0.165134	0	0.491822	OK
ENSBTAG00000006677	ENSBTAG00000006677	27:41956645-41957517	0.165021	0	0.71646	OK
ENSBTAG00000004720	ENSBTAG00000004720	14:35476251-35558292	0.164988	0	0.395298	OK
ENSBTAG00000013675	ENSBTAG00000013675	11:46481774-46490168	0.164988	0	0.50849	OK
ENSBTAG00000007577	ENSBTAG00000007577	1:71542375-71573340	0.164867	0	0.546902	OK
ENSBTAG00000031025	ENSBTAG00000031025	15:79166474-79167404	0.164813	0	0.667926	OK
ENSBTAG00000016276	ENSBTAG00000016276	5:60618662-60641990	0.164762	0	0.35455	OK
ENSBTAG00000017964	ENSBTAG00000017964	15:52492120-52508047	0.164607	0	0.479299	OK
ENSBTAG00000016721	ENSBTAG00000016721	7:14012313-14014696	0.164547	0	0.566658	OK
ENSBTAG00000021801	ENSBTAG00000021801	26:40283526-40290147	0.164442	0	0.657324	OK
ENSBTAG00000000892	ENSBTAG00000000892	9:13154612-13177012	0.164334	0	0.414944	OK
ENSBTAG00000011808	ENSBTAG00000011808	2:6213565-6220196	0.16431	0	0.448823	OK
ENSBTAG00000015067	ENSBTAG00000015067	3:27593571-27596691	0.164309	0	0.471656	OK
ENSBTAG00000046221	ENSBTAG00000046221	11:93742433-93743378	0.164246	0	0.657324	OK
ENSBTAG00000009497	ENSBTAG00000009497	7:43264087-43265023	0.164194	0	0.663644	OK
ENSBTAG00000014192	ENSBTAG00000014192	7:51803644-51806667	0.16418	0	0.463907	OK
ENSBTAG00000019464	ENSBTAG00000019464	2:72589719-72592009	0.164125	0	0.542034	OK
ENSBTAG00000032142	ENSBTAG00000032142	7:14560280-14561198	0.164102	0	0.676657	OK

ENSBTAG00000021357	ENSBTAG00000021357	26:17367944-17372393	0.164055	0	0.465401	OK
ENSBTAG00000001181	ENSBTAG00000001181	16:72241104-72445379	0.163983	0	0.375188	OK
ENSBTAG00000046103	ENSBTAG00000046103	7:54117004-54119380	0.163563	0	0.304048	OK
ENSBTAG00000007718	ENSBTAG00000007718	24:37940877-37948732	0.163553	0	0.407325	OK
ENSBTAG00000005893	ENSBTAG00000005893	14:24711326-24747118	0.16342	0	0.434689	OK
ENSBTAG00000015230	ENSBTAG00000015230	6:16828641-16843001	0.163398	0	0.43568	OK
ENSBTAG00000039231	ENSBTAG00000039231	4:66610954-66643765	0.163377	0.0358913	0.323022	OK
ENSBTAG00000025720	ENSBTAG00000025720	13:422888-424810	0.16318	0	0.577296	OK
ENSBTAG00000000305	ENSBTAG00000000305	3:56832932-56846708	0.163174	0	0.474176	OK
ENSBTAG00000011648	ENSBTAG00000011648	X:38362069-38383556	0.16308	0	0.552152	OK
ENSBTAG00000040456	ENSBTAG00000040456	1:42035718-42036686	0.163073	0	0.655244	OK
ENSBTAG00000038510	ENSBTAG00000038510	7:14760181-14761114	0.163061	0	0.665778	OK
ENSBTAG00000011926	ENSBTAG00000011926	18:58994579-59004703	0.163005	0	0.492407	OK
ENSBTAG00000038088	ENSBTAG00000038088	18:65429748-65445944	0.162967	0	0.462387	OK
ENSBTAG00000007201	ENSBTAG00000007201	9:95353541-95394421	0.162964	0	0.398442	OK
ENSBTAG00000010204	ENSBTAG00000010204	9:88068717-88101696	0.162925	0	0.980595	OK
ENSBTAG00000000195	ENSBTAG00000000195	18:64807706-64812263	0.16271	0	0.437444	OK
ENSBTAG00000008543	ENSBTAG00000008543	9:96153559-96167611	0.1627	0	0.752023	OK
ENSBTAG00000047890	ENSBTAG00000047890	5:59473205-59474141	0.162625	0	0.663644	OK
ENSBTAG00000035985	ENSBTAG00000035985	15:80484653-80485613	0.162467	0	0.647053	OK
ENSBTAG00000045684	ENSBTAG00000045684	5:58951874-58952810	0.162427	0	0.663644	OK
ENSBTAG00000012656	ENSBTAG00000012656	29:44574559-44585598	0.162314	0	0.811568	OK
ENSBTAG00000038188	ENSBTAG00000038188	10:27842827-27843766	0.162275	0	0.661524	OK
ENSBTAG00000005377	ENSBTAG00000005377	4:24927682-24931927	0.162225	0	0.595676	OK
ENSBTAG00000047109	ENSBTAG00000047109	5:59178070-59178994	0.161597	0	0.672263	OK
ENSBTAG00000013781	ENSBTAG00000013781	4:15543252-15580346	0.161528	0	0.424587	OK
ENSBTAG00000001439	ENSBTAG00000001439	22:44449108-44450787	0.161425	0	0.794336	OK
ENSBTAG00000003629	ENSBTAG00000003629	19:24825865-24847108	0.161375	0	0.62254	OK
ENSBTAG00000014795	ENSBTAG00000014795	4:49945496-49946378	0.161353	0	0.704275	OK
ENSBTAG00000039804	ENSBTAG00000039804	7:42326727-42327651	0.161267	0	0.672263	OK

ENSBTAG00000039653	ENSBTAG00000039653	3:11238942-11240026	0.161229	0	0.631271	OK
ENSBTAG00000039348	ENSBTAG00000039348	15:80685808-80686821	0.161119	0	0.651123	OK
ENSBTAG00000012618	ENSBTAG00000012618	2:107836178-107842357	0.161067	0	0.334502	OK
ENSBTAG00000002202	ENSBTAG00000002202	25:1245668-1284542	0.161062	0	0.270663	OK
ENSBTAG00000010487	ENSBTAG00000010487	7:64133811-64153713	0.160812	0	0.395902	OK
ENSBTAG00000019726	ENSBTAG00000019726	13:3147663-3170170	0.160772	0	0.444138	OK
ENSBTAG00000013417	ENSBTAG00000013417	5:25595261-25598162	0.160671	0	0.973622	OK
ENSBTAG00000021869	ENSBTAG00000021869	4:59975918-59982000	0.160625	0	0.55911	OK
ENSBTAG00000040285	ENSBTAG00000040285	15:45347107-45352210	0.160588	0	0.62555	OK
ENSBTAG00000007647	ENSBTAG00000007647	X:15453981-15454970	0.160562	0	0.649081	OK
ENSBTAG00000024798	ENSBTAG00000024798	5:58450944-58451880	0.160561	0	0.663644	OK
ENSBTAG00000000207	ENSBTAG00000000207	X:146775606-146843115	0.160448	0	0.309502	OK
ENSBTAG00000003754	ENSBTAG00000003754	10:38111922-38135944	0.160368	0	0.307597	OK
ENSBTAG00000021832	ENSBTAG00000021832	2:129687546-129705679	0.1603	0	0.395398	OK
ENSBTAG00000047333	ENSBTAG00000047333	20:132122-133868	0.160255	0	0.627445	OK
ENSBTAG00000040188	ENSBTAG00000040188	23:29106707-29110560	0.160233	0	0.529332	OK
ENSBTAG00000007705	ENSBTAG00000007705	24:35630928-35816269	0.160216	0	0.328228	OK
ENSBTAG00000004713	ENSBTAG00000004713	1:106967259-106968738	0.160153	0	0.424297	OK
ENSBTAG00000044099	ENSBTAG00000044099	19:34074735-34084350	0.160132	0	0.410556	OK
ENSBTAG00000004124	ENSBTAG00000004124	1:42518055-42531538	0.16012	0	0.294533	OK
ENSBTAG00000040082	ENSBTAG00000040082	4:69322974-69326738	0.160118	0	0.800592	OK
ENSBTAG00000020199	ENSBTAG00000020199	10:7879495-7897150	0.160114	0	0.354651	OK
ENSBTAG00000038893	ENSBTAG00000038893	5:29175925-29177488	0.159994	0	0.397422	OK
ENSBTAG00000038308	ENSBTAG00000038308	8:60653091-60654051	0.159992	0	0.647053	OK
ENSBTAG00000022044	ENSBTAG00000022044	17:64724243-64742928	0.15989	0	0.300191	OK
ENSBTAG00000020981	ENSBTAG00000020981	26:13831826-13865056	0.159859	0	0.54109	OK
ENSBTAG00000003577	ENSBTAG00000003577	25:10622208-10652097	0.159802	0	0.409338	OK
ENSBTAG00000015872	ENSBTAG00000015872	13:47908388-47915709	0.159785	0	0.53781	OK
ENSBTAG00000009188	ENSBTAG00000009188	1:1265742-1292028	0.159744	0	0.334322	OK
ENSBTAG00000045699	ENSBTAG00000045699	28:22419202-24270401	0.159684	0	0.462181	OK

ENSBTAG00000038452	ENSBTAG00000038452	15:47809649-47810585	0.159614	0	0.663644	OK
ENSBTAG00000018185	ENSBTAG00000018185	4:12597837-12699701	0.159552	0	0.480782	OK
ENSBTAG00000021041	ENSBTAG00000021041	7:18331127-18339203	0.159496	0	0.280312	OK
ENSBTAG00000011070	ENSBTAG00000011070	5:71366685-71388046	0.159447	0	0.405994	OK
ENSBTAG00000019125	ENSBTAG00000019125	8:40178393-40253322	0.159431	0	0.347411	OK
ENSBTAG00000001019	ENSBTAG00000001019	28:42628170-42640763	0.159388	0	0.250371	OK
ENSBTAG00000009412	ENSBTAG00000009412	29:28485871-28523643	0.159354	0	0.301101	OK
ENSBTAG00000023556	ENSBTAG00000023556	3:11458291-11459230	0.159262	0	0.661524	OK
ENSBTAG00000016880	ENSBTAG00000016880	4:105874499-105875450	0.159232	0	0.653177	OK
ENSBTAG00000014912	ENSBTAG00000014912	16:925080-935131	0.159228	0	0.398116	OK
ENSBTAG00000003208	ENSBTAG00000003208	17:18553786-18573502	0.159161	0	0.40786	OK
ENSBTAG00000047024	ENSBTAG00000047024	9:41357861-41386980	0.159035	0	0.424297	OK
ENSBTAG00000040157	ENSBTAG00000040157	7:14692182-14699346	0.159033	0	0.657324	OK
ENSBTAG00000009978	ENSBTAG00000009978	23:19892714-19904104	0.158971	0.0304138	0.304138	OK
ENSBTAG00000047829	ENSBTAG00000047829	23:29071804-29072731	0.158927	0	0.670087	OK
ENSBTAG00000046740	ENSBTAG00000046740	29:5214443-5215601	0.158888	0	0.536417	OK
ENSBTAG00000003916	ENSBTAG00000003916	1:155004506-155009732	0.158879	0	0.511504	OK
ENSBTAG00000023546	ENSBTAG00000023546	5:29713133-29752153	0.158522	0	0.37024	OK
ENSBTAG00000013769	ENSBTAG00000013769	1:56353744-56378308	0.158496	0	0.675333	OK
ENSBTAG00000000548	ENSBTAG00000000548	4:94676715-94737325	0.158228	0	0.356176	OK
ENSBTAG00000046872	ENSBTAG00000046872	3:22348929-22349850	0.158228	0	0.674453	OK
ENSBTAG00000005478	ENSBTAG00000005478	17:72229522-72247004	0.158203	0	0.261683	OK
ENSBTAG00000046594	ENSBTAG00000046594	8:73804507-73816226	0.158193	0	0.594991	OK
ENSBTAG00000014931	ENSBTAG00000014931	5:104058658-104063530	0.157786	0	0.426336	OK
ENSBTAG00000046228	ENSBTAG00000046228	15:182644-183853	0.15772	0	0.694822	OK
ENSBTAG00000002915	ENSBTAG00000002915	9:53812417-53813749	0.157632	0	0.466344	OK
ENSBTAG00000038237	ENSBTAG00000038237	3:18061245-18062976	0.157522	0	0.367122	OK
ENSBTAG00000005112	ENSBTAG00000005112	29:27301298-27302243	0.1575	0	0.657324	OK
ENSBTAG00000001057	ENSBTAG00000001057	5:114291726-114341661	0.157485	0	0.387457	OK
ENSBTAG00000014031	ENSBTAG00000014031	6:59687275-59706713	0.157473	0	0.695405	OK

ENSBTAG00000021069	ENSBTAG00000021069	8:10819982-10852941	0.157362	0	0.529287	OK
ENSBTAG00000005923	ENSBTAG00000005923	15:65548290-65735623	0.15729	0	0.268556	OK
ENSBTAG00000016582	ENSBTAG00000016582	9:39846061-39856358	0.157158	0	0.745703	OK
ENSBTAG00000002851	ENSBTAG00000002851	14:79296712-79298474	0.157087	0	0.663644	OK
ENSBTAG00000035485	ENSBTAG00000035485	15:79681638-79682589	0.156962	0	0.653177	OK
ENSBTAG00000035027	ENSBTAG00000035027	15:50080175-50081135	0.156952	0	0.647053	OK
ENSBTAG00000011025	ENSBTAG00000011025	3:94837404-94859216	0.156918	0	0.893752	OK
ENSBTAG00000002448	ENSBTAG00000002448	14:22212867-22350058	0.156857	0	0.32901	OK
ENSBTAG00000002575	ENSBTAG00000002575	16:52310819-52325725	0.156737	0	0.68866	OK
ENSBTAG00000021187	ENSBTAG00000021187	11:88584861-88587166	0.156534	0	0.526862	OK
ENSBTAG00000025644	ENSBTAG00000025644	10:103059963-103067071	0.156487	0	0.421705	OK
ENSBTAG00000037485	ENSBTAG00000037485	29:26802125-26804198	0.156423	0	0.623666	OK
ENSBTAG00000039944	ENSBTAG00000039944	16:382696-383632	0.156379	0	0.663644	OK
ENSBTAG00000023929	ENSBTAG00000023929	11:71329771-71349193	0.15631	0	0.25292	OK
ENSBTAG00000039757	ENSBTAG00000039757	29:27175705-27176754	0.156296	0	0.663644	OK
ENSBTAG00000038802	ENSBTAG00000038802	29:26828029-26828998	0.156213	0	0.641043	OK
ENSBTAG00000003546	ENSBTAG00000003546	28:46067522-46079446	0.156204	0	0.496937	OK
ENSBTAG00000002835	ENSBTAG00000002835	17:12906375-12953417	0.156128	0	0.371736	OK
ENSBTAG00000001258	ENSBTAG00000001258	1:1324543-1361378	0.156099	0	0.505263	OK
ENSBTAG00000040551	ENSBTAG00000040551	10:76961606-76979552	0.155972	0	0.347217	OK
ENSBTAG00000039825	ENSBTAG00000039825	29:5883516-5892565	0.155686	0	0.454072	OK
ENSBTAG00000047921	ENSBTAG00000047921	7:15028920-15029853	0.155561	0	0.665778	OK
ENSBTAG00000025954	ENSBTAG00000025954	8:7133794-7135969	0.155542	0	0.475993	OK
ENSBTAG00000017778	ENSBTAG00000017778	3:11482670-11485878	0.155505	0	0.631271	OK
ENSBTAG00000040339	ENSBTAG00000040339	15:50265666-50266623	0.155386	0	0.649081	OK
ENSBTAG00000010283	ENSBTAG00000010283	7:57235827-57236969	0.155072	0	0.577296	OK
ENSBTAG00000002477	ENSBTAG00000002477	25:41527725-41533975	0.155071	0	0.347605	OK
ENSBTAG00000034323	ENSBTAG00000034323	16:45252620-45255676	0.155029	0	0.458429	OK
ENSBTAG00000016940	ENSBTAG00000016940	X:29683761-29687322	0.154958	0	0.540901	OK
ENSBTAG00000035059	ENSBTAG00000035059	15:80515013-80515961	0.154944	0	0.655244	OK

ENSBTAG00000018162	ENSBTAG00000018162	18:58494236-58495142	0.154899	0	0.685619	OK
ENSBTAG00000035396	ENSBTAG00000035396	7:14773568-14774507	0.154835	0	0.661524	OK
ENSBTAG00000037735	ENSBTAG00000037735	18:54777174-54781917	0.15483	0	0.433779	OK
ENSBTAG00000001395	ENSBTAG00000001395	1:18719634-18748457	0.154762	0	0.535954	OK
ENSBTAG00000010980	ENSBTAG00000010980	4:25057922-25097468	0.154706	0	0.407112	OK
ENSBTAG00000003397	ENSBTAG00000003397	26:44888007-44936346	0.154682	0	0.533376	OK
ENSBTAG00000033603	ENSBTAG00000033603	23:7758143-7820913	0.15453	0	1.46144	OK
ENSBTAG00000006977	ENSBTAG00000006977	X:57865110-57880915	0.154447	0	0.492056	OK
ENSBTAG00000035572	ENSBTAG00000035572	13:43280031-43281472	0.15437	0	0.431069	OK
ENSBTAG00000017753	ENSBTAG00000017753	1:9607381-9921004	0.154338	0	0.334827	OK
ENSBTAG00000006276	ENSBTAG00000006276	19:53978216-53984772	0.15432	0	0.247478	OK
ENSBTAG00000047428	ENSBTAG00000047428	16:81040750-81042142	0.15429	0	0.446243	OK
ENSBTAG00000039981	ENSBTAG00000039981	11:49070189-49071859	0.154265	0	0.371959	OK
ENSBTAG00000000565	ENSBTAG00000000565	10:54281615-54422091	0.154025	0.0284654	0.37005	OK
ENSBTAG00000010832	ENSBTAG00000010832	22:16901719-16904521	0.154019	0	0.575158	OK
ENSBTAG00000048233	ENSBTAG00000048233	5:59106641-59107701	0.15398	0	0.647053	OK
ENSBTAG00000040489	ENSBTAG00000040489	9:71658063-71659116	0.153939	0	0.589906	OK
ENSBTAG00000023026	ENSBTAG00000023026	24:12747968-12765314	0.15383	0	0.493934	OK
ENSBTAG00000002562	ENSBTAG00000002562	X:92231883-92234517	0.153794	0	0.507078	OK
ENSBTAG00000040008	ENSBTAG00000040008	15:362683-363613	0.153762	0	0.667926	OK
ENSBTAG00000039837	ENSBTAG00000039837	28:2592872-2594981	0.15374	0	0.639065	OK
ENSBTAG00000000940	ENSBTAG00000000940	23:16404967-16407589	0.153648	0	0.394845	OK
ENSBTAG00000015534	ENSBTAG00000015534	16:23350278-23382370	0.153597	0	0.484533	OK
ENSBTAG00000005548	ENSBTAG00000005548	4:88965020-88977481	0.153363	0	0.429579	OK
ENSBTAG00000032704	ENSBTAG00000032704	13:42416037-42453614	0.153311	0	0.303959	OK
ENSBTAG00000016839	ENSBTAG00000016839	9:13189167-13209725	0.153065	0	0.453079	OK
ENSBTAG00000039484	ENSBTAG00000039484	7:42281520-42282450	0.152935	0	0.667926	OK
ENSBTAG00000002488	ENSBTAG00000002488	29:26506379-26539353	0.152922	0	0.764609	OK
ENSBTAG00000034945	ENSBTAG00000034945	23:25634654-25643394	0.152887	0	0.457483	OK
ENSBTAG00000001774	ENSBTAG00000001774	12:55684000-55685894	0.152877	0	0.327968	OK

ENSBTAG00000004048	ENSBTAG00000004048	5:112510410-112513883	0.152822	0	0.274854	OK
ENSBTAG00000032050	ENSBTAG00000032050	15:46545528-46546503	0.152751	0	0.637098	OK
ENSBTAG00000039782	ENSBTAG00000039782	25:39051001-39055715	0.15274	0	0.458219	OK
ENSBTAG00000015043	ENSBTAG00000015043	5:76266276-76282792	0.15263	0	0.305996	OK
ENSBTAG00000008357	ENSBTAG00000008357	20:3535970-3537097	0.152622	0	0.557604	OK
ENSBTAG00000008416	ENSBTAG00000008416	22:14451241-14492710	0.152611	0	0.521029	OK
ENSBTAG00000015220	ENSBTAG00000015220	3:110530153-110558237	0.152569	0	0.353098	OK
ENSBTAG00000003116	ENSBTAG00000003116	12:34177964-34197920	0.152537	0	0.362516	OK
ENSBTAG00000019486	ENSBTAG00000019486	4:7666827-7681085	0.152533	0	0.360517	OK
ENSBTAG00000026263	ENSBTAG00000026263	18:52429557-52446106	0.152459	0	0.398954	OK
ENSBTAG00000008499	ENSBTAG00000008499	5:30658594-30665306	0.152417	0	0.241795	OK
ENSBTAG00000000686	ENSBTAG00000000686	13:10871596-10879769	0.15237	0	0.434689	OK
ENSBTAG00000008967	ENSBTAG00000008967	5:45355307-45399175	0.152307	0	0.514555	OK
ENSBTAG00000015962	ENSBTAG00000015962	2:46648516-46664675	0.152162	0	0.475629	OK
ENSBTAG00000004722	ENSBTAG00000004722	11:22066099-22109204	0.152128	0	0.483552	OK
ENSBTAG00000046778	ENSBTAG00000046778	5:58512429-58513368	0.152078	0	0.661524	OK
ENSBTAG00000023218	ENSBTAG00000023218	14:21225302-21245522	0.152036	0	0.528207	OK
ENSBTAG00000038518	ENSBTAG00000038518	15:46417937-46418998	0.152014	0	0.643034	OK
ENSBTAG00000000566	ENSBTAG00000000566	1:68895326-68937081	0.151979	0	0.449876	OK
ENSBTAG00000012397	ENSBTAG00000012397	6:88049497-88077488	0.151969	0	0.442429	OK
ENSBTAG00000021762	ENSBTAG00000021762	2:9849226-9870250	0.151933	0	0.515323	OK
ENSBTAG00000002290	ENSBTAG00000002290	18:61680431-61686814	0.151892	0	0.377841	OK
ENSBTAG00000007567	ENSBTAG00000007567	24:8748240-8785474	0.151742	0	0.366364	OK
ENSBTAG00000000957	ENSBTAG00000000957	27:13295339-13298891	0.151707	0	0.390969	OK
ENSBTAG00000035758	ENSBTAG00000035758	10:27407173-27408109	0.151674	0	0.663644	OK
ENSBTAG00000020471	ENSBTAG00000020471	10:89648590-89658522	0.151454	0	0.596546	OK
ENSBTAG00000021752	ENSBTAG00000021752	3:66922999-66931737	0.151374	0	0.484155	OK
ENSBTAG00000039964	ENSBTAG00000039964	29:27444677-27445628	0.151338	0	0.653177	OK
ENSBTAG00000040330	ENSBTAG00000040330	15:81067187-81068141	0.151305	0	0.651123	OK
ENSBTAG00000039991	ENSBTAG00000039991	6:86169556-86190786	0.151214	0	0.56542	OK

ENSBTAG00000021437	ENSBTAG00000021437	X:78187377-78188379	0.15097	0	0.619931	OK
ENSBTAG00000021000	ENSBTAG00000021000	1:58790853-58811091	0.150935	0	0.310616	OK
ENSBTAG00000003496	ENSBTAG00000003496	9:81550366-81829531	0.150918	0	0.54109	OK
ENSBTAG00000047282	ENSBTAG00000047282	9:29145020-29146253	0.150839	0	0.670087	OK
ENSBTAG00000040341	ENSBTAG00000040341	15:79725812-79726742	0.1508	0	0.667926	OK
ENSBTAG00000021849	ENSBTAG00000021849	4:48851697-48868187	0.150498	0	0.487767	OK
ENSBTAG00000045651	ENSBTAG00000045651	7:55040491-55041418	0.150379	0	0.670087	OK
ENSBTAG00000015700	ENSBTAG00000015700	26:22742694-22745622	0.150369	0	0.540619	OK
ENSBTAG00000010937	ENSBTAG00000010937	X:126397964-126427158	0.15032	0	0.706679	OK
ENSBTAG00000003068	ENSBTAG00000003068	17:489023-505713	0.150233	0	0.571349	OK
ENSBTAG00000047916	ENSBTAG00000047916	29:27648732-27649656	0.150192	0	0.672263	OK
ENSBTAG00000019502	ENSBTAG00000019502	12:17973730-17988210	0.150046	0	0.468455	OK
ENSBTAG00000048078	ENSBTAG00000048078	14:5674860-5688668	0.150039	0	0.353541	OK
ENSBTAG00000014171	ENSBTAG00000014171	4:44665682-44720623	0.149934	0	0.407592	OK
ENSBTAG00000010051	ENSBTAG00000010051	2:238092-305169	0.149901	0	0.526327	OK
ENSBTAG00000011976	ENSBTAG00000011976	3:99937184-99957408	0.149852	0	0.350548	OK
ENSBTAG00000033535	ENSBTAG00000033535	9:34605303-34613344	0.149772	0	0.449147	OK
ENSBTAG00000037424	ENSBTAG00000037424	19:9224989-9226905	0.149675	0	0.637098	OK
ENSBTAG00000017527	ENSBTAG00000017527	9:43886255-43937667	0.149612	0	0.333843	OK
ENSBTAG00000047165	ENSBTAG00000047165	19:42413412-42418215	0.149566	0	0.399724	OK
ENSBTAG00000031609	ENSBTAG00000031609	15:56543032-56571783	0.149537	0	0.438123	OK
ENSBTAG00000013802	ENSBTAG00000013802	3:89639196-89699453	0.149524	0	0.425168	OK
ENSBTAG00000048138	ENSBTAG00000048138	14:36480272-36500327	0.149458	0	0.564188	OK
ENSBTAG00000026242	ENSBTAG00000026242	14:63446265-63534092	0.149322	0	0.38582	OK
ENSBTAG00000039615	ENSBTAG00000039615	9:71759483-71761088	0.149202	0	0.746008	OK
ENSBTAG00000039868	ENSBTAG00000039868	8:96152907-96153861	0.149114	0	0.651123	OK
ENSBTAG00000015874	ENSBTAG00000015874	27:14084621-14110610	0.149049	0	0.408396	OK
ENSBTAG00000030710	ENSBTAG00000030710	3:82389601-82495202	0.149043	0	0.71399	OK
ENSBTAG00000045580	ENSBTAG00000045580	17:8880523-8891738	0.149014	0	0.510412	OK
ENSBTAG00000039316	ENSBTAG00000039316	17:45221484-45223845	0.14872	0	0.470157	OK

ENSBTAG00000019836	ENSBTAG00000019836	21:62870562-62892534	0.148567	0	0.478413	OK
ENSBTAG00000000959	ENSBTAG00000000959	13:75359805-75362820	0.1485	0	0.298353	OK
ENSBTAG00000021011	ENSBTAG00000021011	17:28834267-28835511	0.14834	0	0.499333	OK
ENSBTAG00000046671	ENSBTAG00000046671	6:104673700-104696495	0.147978	0	0.23291	OK
ENSBTAG00000038672	ENSBTAG00000038672	10:27189546-27190857	0.147897	0	0.649081	OK
ENSBTAG00000013439	ENSBTAG00000013439	1:114292425-114431455	0.147852	0	0.351182	OK
ENSBTAG00000003330	ENSBTAG00000003330	29:42617153-42658661	0.147811	0	0.313881	OK
ENSBTAG00000010059	ENSBTAG00000010059	8:16694706-16714705	0.147757	0	0.397041	OK
ENSBTAG00000045689	ENSBTAG00000045689	12:15274613-15276029	0.147697	0	0.43868	OK
ENSBTAG00000046163	ENSBTAG00000046163	7:11141264-11142224	0.147562	0	0.647053	OK
ENSBTAG00000039331	ENSBTAG00000039331	15:79980016-79981032	0.14756	0	0.645708	OK
ENSBTAG00000016519	ENSBTAG00000016519	23:37498571-37608778	0.147525	0	0.444328	OK
ENSBTAG00000046892	ENSBTAG00000046892	23:29061845-29062781	0.147472	0	0.663644	OK
ENSBTAG00000047098	ENSBTAG00000047098	X:4062425-4063594	0.147425	0	0.531369	OK
ENSBTAG00000002315	ENSBTAG00000002315	17:56021829-56042534	0.147292	0	0.317897	OK
ENSBTAG00000018691	ENSBTAG00000018691	28:2167763-2177307	0.147228	0	0.376604	OK
ENSBTAG00000005786	ENSBTAG00000005786	21:57347864-57381142	0.147136	0	0.553628	OK
ENSBTAG00000040509	ENSBTAG00000040509	9:71635793-71636950	0.146882	0	0.593286	OK
ENSBTAG00000017651	ENSBTAG00000017651	18:58866567-58876003	0.146862	0	0.554815	OK
ENSBTAG00000005607	ENSBTAG00000005607	X:83572298-83601869	0.146834	0	0.4418	OK
ENSBTAG00000039533	ENSBTAG00000039533	7:9244569-9245508	0.146819	0	0.661524	OK
ENSBTAG00000040023	ENSBTAG00000040023	15:83886612-83887661	0.146756	0	0.665778	OK
ENSBTAG00000012697	ENSBTAG00000012697	26:18514078-18520590	0.146737	0	0.363896	OK
ENSBTAG00000009176	ENSBTAG00000009176	15:1732076-1734884	0.146643	0	0.493934	OK
ENSBTAG00000011601	ENSBTAG00000011601	11:48848623-48905318	0.146465	0	0.40786	OK
ENSBTAG00000010801	ENSBTAG00000010801	20:63065547-63090743	0.146301	0	0.417734	OK
ENSBTAG00000006769	ENSBTAG00000006769	3:23813911-23823807	0.146229	0	0.380853	OK
ENSBTAG00000006202	ENSBTAG00000006202	5:112612770-112636488	0.14621	0	0.366364	OK
ENSBTAG00000043550	ENSBTAG00000043550	MT:14513-15653	0.146207	0	0.544887	OK
ENSBTAG00000048193	ENSBTAG00000048193	7:10695138-10696824	0.146188	0	0.623666	OK

ENSBTAG00000002912	ENSBTAG00000002912	4:79986253-79997754	0.14613	0	0.376467	OK
ENSBTAG00000031099	ENSBTAG00000031099	5:59518268-59519207	0.146043	0	0.661524	OK
ENSBTAG00000015644	ENSBTAG00000015644	18:49480064-49481558	0.145879	0	0.415777	OK
ENSBTAG00000038961	ENSBTAG00000038961	3:11055064-11056394	0.145859	0	0.657324	OK
ENSBTAG00000015958	ENSBTAG00000015958	19:45914505-45935073	0.145844	0	0.31268	OK
ENSBTAG00000019061	ENSBTAG00000019061	X:13915386-13926276	0.145814	0	0.258713	OK
ENSBTAG00000005199	ENSBTAG00000005199	29:28072193-28073453	0.145465	0	0.657324	OK
ENSBTAG00000002891	ENSBTAG00000002891	4:117713084-117730633	0.145444	0	0.276199	OK
ENSBTAG00000010868	ENSBTAG00000010868	18:16836725-16841714	0.145403	0	0.412628	OK
ENSBTAG00000006473	ENSBTAG00000006473	3:71025064-71025924	0.145372	0	0.732513	OK
ENSBTAG00000009103	ENSBTAG00000009103	7:72960643-72980667	0.145367	0	0.493463	OK
ENSBTAG00000006708	ENSBTAG00000006708	10:19197513-19300694	0.145305	0	0.383155	OK
ENSBTAG00000012501	ENSBTAG00000012501	18:22118200-22541532	0.145226	0	0.330234	OK
ENSBTAG00000035029	ENSBTAG00000035029	15:80856240-80857182	0.145212	0	0.659417	OK
ENSBTAG00000046963	ENSBTAG00000046963	7:41276394-41277330	0.145159	0	0.663644	OK
ENSBTAG00000023928	ENSBTAG00000023928	11:80261888-80266800	0.145141	0	0.415499	OK
ENSBTAG00000001659	ENSBTAG00000001659	18:36584632-36595360	0.14514	0	0.725702	OK
ENSBTAG00000001697	ENSBTAG00000001697	1:81878690-81898384	0.145123	0	0.441172	OK
ENSBTAG00000015776	ENSBTAG00000015776	3:43332800-43386388	0.145111	0	0.40362	OK
ENSBTAG00000005671	ENSBTAG00000005671	15:48700000-48701806	0.145024	0	0.573247	OK
ENSBTAG00000013579	ENSBTAG00000013579	27:25391026-25416076	0.144986	0	0.32488	OK
ENSBTAG00000017934	ENSBTAG00000017934	15:50412562-50413522	0.144922	0	0.647053	OK
ENSBTAG00000019232	ENSBTAG00000019232	2:88915907-88935670	0.144711	0	0.638408	OK
ENSBTAG00000006817	ENSBTAG00000006817	15:30314910-30406073	0.14441	0	0.383013	OK
ENSBTAG00000047812	ENSBTAG00000047812	23:52388043-52389508	0.144367	0	0.657324	OK
ENSBTAG00000046299	ENSBTAG00000046299	29:27380843-27382242	0.144351	0	0.61808	OK
ENSBTAG00000046216	ENSBTAG00000046216	25:11449826-11450815	0.144334	0	0.665778	OK
ENSBTAG00000018650	ENSBTAG00000018650	29:28751163-28767534	0.144219	0	0.25027	OK
ENSBTAG00000015485	ENSBTAG00000015485	9:101942116-101966174	0.144076	0	0.381789	OK
ENSBTAG00000009507	ENSBTAG00000009507	5:113565316-113569978	0.143988	0	0.257747	OK

ENSBTAG00000025213	ENSBTAG00000025213	19:19044189-19046816	0.143913	0	0.57452	OK
ENSBTAG00000010884	ENSBTAG00000010884	X:4098499-4114350	0.143853	0	0.457753	OK
ENSBTAG00000047643	ENSBTAG00000047643	27:22499289-22500766	0.143628	0	0.420563	OK
ENSBTAG00000008324	ENSBTAG00000008324	19:24480296-24481721	0.143416	0	0.717081	OK
ENSBTAG00000038947	ENSBTAG00000038947	18:65278552-65280012	0.143367	0	0.498932	OK
ENSBTAG00000022535	ENSBTAG00000022535	25:17757360-17799905	0.14322	0	0.586564	OK
ENSBTAG00000003325	ENSBTAG00000003325	22:15877257-15923665	0.143213	0	0.298353	OK
ENSBTAG00000020938	ENSBTAG00000020938	5:36847868-36859266	0.143157	0	0.500944	OK
ENSBTAG00000008887	ENSBTAG00000008887	6:95409077-95441932	0.14313	0	0.429391	OK
ENSBTAG00000046670	ENSBTAG00000046670	19:43283854-43290953	0.143014	0	0.564188	OK
ENSBTAG00000038521	ENSBTAG00000038521	5:58781760-58782696	0.142829	0	0.663644	OK
ENSBTAG00000007374	ENSBTAG00000007374	10:9500428-9527328	0.142641	0	0.260722	OK
ENSBTAG00000000375	ENSBTAG00000000375	15:81817412-81826972	0.14264	0	0.236998	OK
ENSBTAG00000009210	ENSBTAG00000009210	29:36988681-37011135	0.142409	0	0.368757	OK
ENSBTAG00000030929	ENSBTAG00000030929	7:17218710-17230346	0.142393	0	0.500138	OK
ENSBTAG00000001093	ENSBTAG00000001093	2:26619584-26636478	0.142381	0	0.616241	OK
ENSBTAG00000045488	ENSBTAG00000045488	15:50018820-50019780	0.142342	0	0.647053	OK
ENSBTAG00000013141	ENSBTAG00000013141	1:98396411-98398345	0.142293	0	0.457416	OK
ENSBTAG00000004833	ENSBTAG00000004833	1:44261476-44281308	0.142093	0	0.532737	OK
ENSBTAG00000013207	ENSBTAG00000013207	5:91770302-91771388	0.142043	0	0.571981	OK
ENSBTAG00000002451	ENSBTAG00000002451	3:95558169-95589486	0.141859	0	0.345864	OK
ENSBTAG00000014217	ENSBTAG00000014217	26:14120257-14126068	0.141655	0	0.364323	OK
ENSBTAG00000039046	ENSBTAG00000039046	2:118596200-118598002	0.1416	0	0.57452	OK
ENSBTAG00000009962	ENSBTAG00000009962	11:5550083-5561240	0.14157	0	0.414114	OK
ENSBTAG00000014513	ENSBTAG00000014513	15:80715052-80715995	0.141566	0	0.658718	OK
ENSBTAG00000018196	ENSBTAG00000018196	11:70851864-70890941	0.141558	0	0.360936	OK
ENSBTAG00000047464	ENSBTAG00000047464	15:83053427-83054384	0.141518	0	0.649081	OK
ENSBTAG00000039021	ENSBTAG00000039021	8:77451685-77457766	0.141507	0	0.310198	OK
ENSBTAG00000002940	ENSBTAG00000002940	4:86598083-86609355	0.141506	0	0.396205	OK
ENSBTAG00000001178	ENSBTAG00000001178	12:30210163-30231502	0.141499	0	0.377841	OK

ENSBTAG00000045929	ENSBTAG00000045929	21:20128500-20132903	0.141483	0	0.59193	OK
ENSBTAG00000015357	ENSBTAG00000015357	15:51516635-51517716	0.141429	0	0.645037	OK
ENSBTAG00000039091	ENSBTAG00000039091	2:30716353-30808741	0.14139	0	0.383913	OK
ENSBTAG00000002775	ENSBTAG00000002775	5:82653405-82656449	0.141068	0	0.542507	OK
ENSBTAG00000004181	ENSBTAG00000004181	5:32004194-32005115	0.140827	0	0.674453	OK
ENSBTAG00000034743	ENSBTAG00000034743	7:43446064-43447039	0.140703	0	0.637098	OK
ENSBTAG00000003549	ENSBTAG00000003549	10:27989291-27990230	0.140691	0	0.661524	OK
ENSBTAG00000019123	ENSBTAG00000019123	25:2691853-2704324	0.140659	0	0.264553	OK
ENSBTAG00000019184	ENSBTAG00000019184	18:14079699-14083634	0.140571	0	0.312146	OK
ENSBTAG00000046505	ENSBTAG00000046505	24:392054-392994	0.140545	0	0.670087	OK
ENSBTAG00000015277	ENSBTAG00000015277	29:12555853-12580195	0.140393	0	0.355632	OK
ENSBTAG00000025434	ENSBTAG00000025434	10:80965363-80970846	0.140388	0	0.34372	OK
ENSBTAG00000021652	ENSBTAG00000021652	25:19881633-19900610	0.140248	0	0.701242	OK
ENSBTAG00000006197	ENSBTAG00000006197	8:29553186-29609481	0.140019	0	0.464462	OK
ENSBTAG00000012342	ENSBTAG00000012342	5:29804688-29898282	0.140019	0	0.334863	OK
ENSBTAG00000019060	ENSBTAG00000019060	1:97788088-97802992	0.139993	0	0.331468	OK
ENSBTAG00000004168	ENSBTAG00000004168	11:30722155-30739321	0.139929	0	0.401507	OK
ENSBTAG00000046042	ENSBTAG00000046042	7:42947454-42948392	0.13989	0	0.662229	OK
ENSBTAG00000047174	ENSBTAG00000047174	22:52425503-52428832	0.139771	0	0.378301	OK
ENSBTAG00000002056	ENSBTAG00000002056	2:86593546-86595781	0.139678	0	0.277929	OK
ENSBTAG00000036331	ENSBTAG00000036331	7:9595047-9595989	0.13966	0	0.659417	OK
ENSBTAG00000030897	ENSBTAG00000030897	4:115796822-115821949	0.139505	0	0.376057	OK
ENSBTAG00000000061	ENSBTAG00000000061	6:51536862-52007783	0.139406	0	0.286624	OK
ENSBTAG00000047771	ENSBTAG00000047771	22:41429737-41430712	0.139321	0	0.690958	OK
ENSBTAG00000020647	ENSBTAG00000020647	6:70179361-70183823	0.139297	0	0.323358	OK
ENSBTAG00000018739	ENSBTAG00000018739	3:51195434-51281129	0.139267	0	0.524416	OK
ENSBTAG00000010023	ENSBTAG00000010023	13:29501667-29578090	0.139246	0	0.426628	OK
ENSBTAG00000009427	ENSBTAG00000009427	19:12602356-12662178	0.139245	0	0.321684	OK
ENSBTAG00000000804	ENSBTAG00000000804	8:16745166-16961930	0.139198	0	0.25437	OK
ENSBTAG00000047610	ENSBTAG00000047610	11:93268703-93269738	0.139183	0	0.600165	OK

ENSBTAG00000005205	ENSBTAG00000005205	9:34718088-34766026	0.139098	0	0.327708	OK
ENSBTAG00000015238	ENSBTAG00000015238	24:26396991-26448151	0.139072	0	0.548916	OK
ENSBTAG00000002115	ENSBTAG00000002115	13:61265882-61285459	0.138853	0	0.448823	OK
ENSBTAG00000004383	ENSBTAG00000004383	3:50082609-50146984	0.138831	0	0.319286	OK
ENSBTAG00000016987	ENSBTAG00000016987	11:75071678-75078888	0.138652	0	0.51201	OK
ENSBTAG00000008788	ENSBTAG00000008788	19:20501230-20525353	0.138579	0	0.260996	OK
ENSBTAG00000046932	ENSBTAG00000046932	14:1301786-1304027	0.138507	0	0.667926	OK
ENSBTAG00000046274	ENSBTAG00000046274	15:80507649-80508576	0.138492	0	0.670087	OK
ENSBTAG00000031412	ENSBTAG00000031412	7:42479679-42480633	0.138483	0	0.651123	OK
ENSBTAG00000002779	ENSBTAG00000002779	20:2583485-2610136	0.138373	0	0.404145	OK
ENSBTAG00000007557	ENSBTAG00000007557	7:42787985-42788915	0.138275	0	0.667926	OK
ENSBTAG00000032425	ENSBTAG00000032425	6:90664367-90665675	0.138274	0	0.739489	OK
ENSBTAG00000005828	ENSBTAG00000005828	11:332457-427685	0.13801	0	0.298267	OK
ENSBTAG00000013578	ENSBTAG00000013578	3:32269407-32284496	0.137895	0	0.524195	OK
ENSBTAG00000038485	ENSBTAG00000038485	10:27085889-27086834	0.137763	0	0.657324	OK
ENSBTAG00000017287	ENSBTAG00000017287	5:66043814-66079316	0.137754	0	0.393794	OK
ENSBTAG00000024691	ENSBTAG00000024691	5:59169748-59170684	0.137747	0	0.663644	OK
ENSBTAG00000048141	ENSBTAG00000048141	15:79933395-79935009	0.137628	0	0.645037	OK
ENSBTAG00000005273	ENSBTAG00000005273	11:6876947-6929909	0.137391	0	0.361988	OK
ENSBTAG00000008613	ENSBTAG00000008613	15:64480785-64510275	0.137358	0	0.363258	OK
ENSBTAG00000039433	ENSBTAG00000039433	7:53946907-53949301	0.137349	0	0.25947	OK
ENSBTAG00000009281	ENSBTAG00000009281	5:57253562-57255634	0.13727	0	0.686352	OK
ENSBTAG00000037511	ENSBTAG00000037511	10:27289004-27289943	0.137257	0	0.661524	OK
ENSBTAG00000035309	ENSBTAG00000035309	10:28022292-28023255	0.137234	0	0.645037	OK
ENSBTAG00000010649	ENSBTAG00000010649	21:7045097-7225412	0.137233	0	0.298353	OK
ENSBTAG00000024803	ENSBTAG00000024803	15:15585022-15619671	0.137156	0	0.272742	OK
ENSBTAG00000008603	ENSBTAG00000008603	X:118258768-118271528	0.137122	0	0.464948	OK
ENSBTAG00000023726	ENSBTAG00000023726	18:15078492-15085787	0.13701	0	0.654553	OK
ENSBTAG00000018313	ENSBTAG00000018313	12:78283459-78445751	0.136908	0	0.355158	OK
ENSBTAG00000046471	ENSBTAG00000046471	15:79895418-79896363	0.136718	0	0.657324	OK

ENSBTAG00000047485	ENSBTAG00000047485	23:52444547-52445489	0.136188	0	0.659417	OK
ENSBTAG00000001303	ENSBTAG00000001303	17:58405436-58418688	0.135976	0	0.325903	OK
ENSBTAG00000005665	ENSBTAG00000005665	19:23139101-23159120	0.135913	0	0.347217	OK
ENSBTAG00000002450	ENSBTAG00000002450	15:83602862-83605727	0.135788	0	0.35679	OK
ENSBTAG00000047294	ENSBTAG00000047294	4:41064512-41066816	0.135783	0	0.449342	OK
ENSBTAG00000033543	ENSBTAG00000033543	8:25197703-25199286	0.135764	0	0.392401	OK
ENSBTAG00000046612	ENSBTAG00000046612	9:63520826-63622497	0.135762	0.0234519	0.328327	OK
ENSBTAG00000038264	ENSBTAG00000038264	16:55803156-55806065	0.135739	0	0.423429	OK
ENSBTAG00000037710	ENSBTAG00000037710	18:57630985-57632950	0.135611	0	0.459106	OK
ENSBTAG00000012250	ENSBTAG00000012250	10:65111942-65126559	0.135489	0	0.43868	OK
ENSBTAG00000000095	ENSBTAG00000000095	8:39497222-39514616	0.135438	0	0.465645	OK
ENSBTAG00000023840	ENSBTAG00000023840	26:44719078-44746207	0.135342	0	0.490269	OK
ENSBTAG00000007913	ENSBTAG00000007913	15:15705459-15713952	0.135337	0	0.525081	OK
ENSBTAG00000008148	ENSBTAG00000008148	23:25066914-25087445	0.135278	0	0.413287	OK
ENSBTAG00000011516	ENSBTAG00000011516	14:62215197-62305653	0.135218	0	0.273222	OK
ENSBTAG00000037820	ENSBTAG00000037820	1:42358222-42359149	0.135205	0	0.670087	OK
ENSBTAG00000045603	ENSBTAG00000045603	7:53908353-53910744	0.135083	0	0.259795	OK
ENSBTAG00000001291	ENSBTAG00000001291	15:80288367-80289306	0.135055	0	0.661524	OK
ENSBTAG00000026672	ENSBTAG00000026672	9:34516781-34548809	0.135029	0	0.966978	OK
ENSBTAG00000018352	ENSBTAG00000018352	14:59974385-59985200	0.134997	0	0.371736	OK
ENSBTAG00000015902	ENSBTAG00000015902	3:66382017-66445670	0.1349	0	0.479098	OK
ENSBTAG00000007636	ENSBTAG00000007636	5:47796255-47839675	0.134871	0	0.463881	OK
ENSBTAG00000020120	ENSBTAG00000020120	13:7570430-7648329	0.134866	0	0.499414	OK
ENSBTAG00000008527	ENSBTAG00000008527	13:72804659-72810323	0.13486	0	0.361882	OK
ENSBTAG00000002863	ENSBTAG00000002863	24:49908125-49942827	0.13484	0	0.393395	OK
ENSBTAG00000027923	ENSBTAG00000027923	3:27976838-27978841	0.13476	0	0.396914	OK
ENSBTAG00000037684	ENSBTAG00000037684	6:5795-6744	0.134697	0	0.654553	OK
ENSBTAG00000017597	ENSBTAG00000017597	24:35021779-35047784	0.134429	0	0.464948	OK
ENSBTAG00000027534	ENSBTAG00000027534	6:90636201-90637197	0.134386	0	0.623666	OK
ENSBTAG00000011227	ENSBTAG00000011227	15:7652987-7714074	0.134382	0	0.411371	OK

ENSBTAG00000008097	ENSBTAG00000008097	4:51398055-51441973	0.134265	0	0.275953	OK
ENSBTAG00000006726	ENSBTAG00000006726	24:8587477-8645504	0.134246	0	0.452419	OK
ENSBTAG00000024070	ENSBTAG00000024070	3:4647-5585	0.134061	0	0.662229	OK
ENSBTAG00000002921	ENSBTAG00000002921	10:36404113-36419370	0.133713	0	0.291083	OK
ENSBTAG00000016709	ENSBTAG00000016709	4:64185798-64205027	0.133628	0	0.59193	OK
ENSBTAG00000017735	ENSBTAG00000017735	29:12170163-12171433	0.133488	0	0.631913	OK
ENSBTAG00000047151	ENSBTAG00000047151	29:27637610-27638570	0.133451	0	0.647053	OK
ENSBTAG00000020935	ENSBTAG00000020935	10:74095880-74139364	0.133381	0	0.487002	OK
ENSBTAG00000030801	ENSBTAG00000030801	10:73153516-73363530	0.133345	0	0.447852	OK
ENSBTAG00000014267	ENSBTAG00000014267	18:13036325-13079896	0.133028	0	0.218415	OK
ENSBTAG00000013138	ENSBTAG00000013138	10:102697552-102766261	0.133022	0	0.294673	OK
ENSBTAG00000018080	ENSBTAG00000018080	18:45161779-45162954	0.132917	0	0.534111	OK
ENSBTAG00000011203	ENSBTAG00000011203	14:23990192-23999338	0.132857	0	0.33321	OK
ENSBTAG00000013919	ENSBTAG00000013919	23:25458593-25476944	0.132822	0	0.489111	OK
ENSBTAG00000046503	ENSBTAG00000046503	9:25723681-25766095	0.132764	0	0.512517	OK
ENSBTAG00000039493	ENSBTAG00000039493	7:15324726-15346774	0.132758	0	0.377611	OK
ENSBTAG00000047595	ENSBTAG00000047595	17:37860400-38027113	0.132731	0	0.554122	OK
ENSBTAG00000004353	ENSBTAG00000004353	6:94177120-94217673	0.132489	0	0.402835	OK
ENSBTAG00000020953	ENSBTAG00000020953	16:9512152-9514110	0.132467	0	0.317248	OK
ENSBTAG00000019160	ENSBTAG00000019160	19:22633514-22653270	0.132391	0	0.385342	OK
ENSBTAG00000038190	ENSBTAG00000038190	9:10012308-10201631	0.13216	0	0.400239	OK
ENSBTAG00000038377	ENSBTAG00000038377	10:82529905-82532119	0.131985	0	0.486049	OK
ENSBTAG00000038590	ENSBTAG00000038590	29:26859305-26860750	0.131772	0	0.631271	OK
ENSBTAG00000011862	ENSBTAG00000011862	12:398726-402597	0.131665	0	0.362495	OK
ENSBTAG00000040570	ENSBTAG00000040570	16:55744283-55747093	0.131578	0	0.453079	OK
ENSBTAG00000008172	ENSBTAG00000008172	21:44805297-44834310	0.131278	0	0.305996	OK
ENSBTAG00000011796	ENSBTAG00000011796	10:73369416-73380029	0.131239	0	0.444901	OK
ENSBTAG00000007944	ENSBTAG00000007944	15:79774519-79775464	0.131198	0	0.657324	OK
ENSBTAG00000018557	ENSBTAG00000018557	14:29830348-29863766	0.131161	0	0.325561	OK
ENSBTAG00000021193	ENSBTAG00000021193	9:91056938-91067416	0.13097	0	0.533652	OK

ENSBTAG00000033563	ENSBTAG00000033563	18:47193773-47226339	0.130956	0	0.56759	OK
ENSBTAG00000040248	ENSBTAG00000040248	3:51490100-51491654	0.130808	0	0.399724	OK
ENSBTAG00000033096	ENSBTAG00000033096	29:38155710-38165208	0.130693	0	0.740366	OK
ENSBTAG00000002950	ENSBTAG00000002950	17:49075826-49090245	0.13051	0	0.333963	OK
ENSBTAG00000019197	ENSBTAG00000019197	13:43333397-43366338	0.130507	0.0276962	0.249266	OK
ENSBTAG00000046297	ENSBTAG00000046297	7:9911859-9912813	0.130373	0	0.651123	OK
ENSBTAG00000001092	ENSBTAG00000001092	2:26660799-26665974	0.130353	0	0.531824	OK
ENSBTAG00000006182	ENSBTAG00000006182	8:88081847-88083087	0.130269	0	0.500944	OK
ENSBTAG00000006413	ENSBTAG00000006413	10:28459344-28509712	0.130246	0	0.473453	OK
ENSBTAG00000004836	ENSBTAG00000004836	X:53481284-53508483	0.129962	0	0.413287	OK
ENSBTAG00000005215	ENSBTAG00000005215	15:29093383-29102483	0.129901	0	0.360099	OK
ENSBTAG00000002455	ENSBTAG00000002455	6:103844032-103871753	0.129844	0	0.414445	OK
ENSBTAG00000003666	ENSBTAG00000003666	1:47253804-47305082	0.12945	0	0.497733	OK
ENSBTAG00000031458	ENSBTAG00000031458	4:101019787-101021837	0.129229	0	0.50899	OK
ENSBTAG00000010085	ENSBTAG00000010085	27:18785637-18810610	0.129155	0	0.313248	OK
ENSBTAG00000003770	ENSBTAG00000003770	15:22912246-22920397	0.129024	0	0.65802	OK
ENSBTAG00000017362	ENSBTAG00000017362	7:493449-574753	0.12901	0	0.353038	OK
ENSBTAG00000013455	ENSBTAG00000013455	2:58798301-58846989	0.128922	0	0.444011	OK
ENSBTAG00000035395	ENSBTAG00000035395	7:14857369-14858308	0.12873	0	0.661524	OK
ENSBTAG00000046280	ENSBTAG00000046280	11:72097721-72102932	0.128633	0	0.397346	OK
ENSBTAG00000001343	ENSBTAG00000001343	3:77093221-77113283	0.12861	0	0.510831	OK
ENSBTAG00000001513	ENSBTAG00000001513	2:127561536-127569228	0.128519	0	0.448823	OK
ENSBTAG00000006360	ENSBTAG00000006360	20:12352170-12366624	0.12835	0	0.481528	OK
ENSBTAG00000005338	ENSBTAG00000005338	15:64095494-64112928	0.128273	0	0.484533	OK
ENSBTAG00000007457	ENSBTAG00000007457	12:20512076-20568813	0.128248	0	0.278302	OK
ENSBTAG00000048129	ENSBTAG00000048129	17:58884-60218	0.128045	0	0.668645	OK
ENSBTAG00000036130	ENSBTAG00000036130	15:81147756-81148686	0.127985	0	0.667926	OK
ENSBTAG00000000455	ENSBTAG00000000455	29:9952632-9958039	0.127935	0	0.287646	OK
ENSBTAG00000009717	ENSBTAG00000009717	4:44236253-44240215	0.127685	0	0.413838	OK
ENSBTAG00000013260	ENSBTAG00000013260	X:99440293-99442061	0.127587	0	0.563676	OK

ENSBTAG00000001013	ENSBTAG00000001013	20:10455655-10487640	0.12727	0	0.371469	OK
ENSBTAG00000001336	ENSBTAG00000001336	5:99072338-99073256	0.127209	0	0.676657	OK
ENSBTAG00000007881	ENSBTAG00000007881	26:11108662-11110084	0.127136	0	0.436829	OK
ENSBTAG00000030435	ENSBTAG00000030435	2:129536932-129540976	0.127023	0	0.426922	OK
ENSBTAG00000014289	ENSBTAG00000014289	3:94600423-94713143	0.126907	0	0.411009	OK
ENSBTAG00000016309	ENSBTAG00000016309	10:79584174-79599910	0.126897	0	0.577347	OK
ENSBTAG00000019881	ENSBTAG00000019881	7:32973282-33031108	0.126831	0	1.33029	OK
ENSBTAG00000009337	ENSBTAG00000009337	18:54079701-54083589	0.126808	0	0.276592	OK
ENSBTAG00000005852	ENSBTAG00000005852	X:98764051-98853465	0.126729	0	0.534111	OK
ENSBTAG00000000943	ENSBTAG00000000943	19:33673395-33688126	0.126728	0	0.351018	OK
ENSBTAG00000022556	ENSBTAG00000022556	3:11435547-11436489	0.126627	0	0.659417	OK
ENSBTAG00000001102	ENSBTAG00000001102	24:62615490-62638833	0.1265	0	0.630119	OK
ENSBTAG00000009470	ENSBTAG00000009470	2:128710452-128783921	0.126323	0	0.387505	OK
ENSBTAG00000008131	ENSBTAG00000008131	3:21639997-21705577	0.126236	0	0.464253	OK
ENSBTAG00000017743	ENSBTAG00000017743	2:29080499-29131828	0.126228	0.0548931	0.347657	OK
ENSBTAG00000009939	ENSBTAG00000009939	5:46719649-46753948	0.125896	0	0.377927	OK
ENSBTAG00000003218	ENSBTAG00000003218	6:113469761-113567507	0.125869	0	0.361146	OK
ENSBTAG00000046564	ENSBTAG00000046564	7:15091726-15092665	0.125846	0	0.661524	OK
ENSBTAG00000001317	ENSBTAG00000001317	24:402200-403135	0.125844	0	0.675186	OK
ENSBTAG00000010947	ENSBTAG00000010947	28:14904832-14999860	0.125801	0	0.751841	OK
ENSBTAG00000015931	ENSBTAG00000015931	3:92106926-92132082	0.125744	0	0.258391	OK
ENSBTAG00000020398	ENSBTAG00000020398	14:17195630-17197461	0.125716	0	0.339252	OK
ENSBTAG00000013045	ENSBTAG00000013045	X:39037543-39049378	0.125711	0	0.266941	OK
ENSBTAG00000047519	ENSBTAG00000047519	7:43739514-43740444	0.125624	0	0.667926	OK
ENSBTAG00000024578	ENSBTAG00000024578	6:27328571-27329745	0.125582	0	0.529106	OK
ENSBTAG00000038083	ENSBTAG00000038083	10:28137445-28138571	0.125567	0	0.649081	OK
ENSBTAG00000018131	ENSBTAG00000018131	5:29272371-29336696	0.125532	0	0.419653	OK
ENSBTAG00000019690	ENSBTAG00000019690	26:37192555-37223786	0.125167	0	0.442429	OK
ENSBTAG00000018440	ENSBTAG00000018440	4:105887870-105888761	0.125053	0	0.697161	OK
ENSBTAG00000036012	ENSBTAG00000036012	8:96041003-96042035	0.124963	0	0.60191	OK

ENSBTAG00000003724	ENSBTAG00000003724	3:51546135-51565701	0.12494	0	0.59272	OK
ENSBTAG00000018935	ENSBTAG00000018935	22:22108900-22119730	0.124687	0	0.393457	OK
ENSBTAG00000030940	ENSBTAG00000030940	4:113910741-113941457	0.124576	0	0.400497	OK
ENSBTAG00000017158	ENSBTAG00000017158	11:81086719-81209374	0.1245	0	0.389693	OK
ENSBTAG00000002714	ENSBTAG00000002714	4:41160157-41266152	0.124366	0	0.334071	OK
ENSBTAG00000003424	ENSBTAG00000003424	4:11162354-11176872	0.124294	0	0.418015	OK
ENSBTAG00000012885	ENSBTAG00000012885	15:17999931-18028984	0.124199	0	0.401792	OK
ENSBTAG00000047188	ENSBTAG00000047188	4:107971283-107972237	0.124184	0	0.651123	OK
ENSBTAG00000019606	ENSBTAG00000019606	13:72886682-72924464	0.124151	0	0.421705	OK
ENSBTAG00000046083	ENSBTAG00000046083	17:73020121-73021815	0.124011	0	0.384388	OK
ENSBTAG00000017863	ENSBTAG00000017863	28:25631916-25647735	0.123776	0	0.531369	OK
ENSBTAG00000009012	ENSBTAG00000009012	1:111027803-111033868	0.123754	0	0.337226	OK
ENSBTAG00000018667	ENSBTAG00000018667	8:39930089-39994840	0.123695	0	0.33595	OK
ENSBTAG00000006114	ENSBTAG00000006114	17:55192960-55213770	0.123577	0	0.287446	OK
ENSBTAG00000039809	ENSBTAG00000039809	7:14888995-14895879	0.123573	0	0.653177	OK
ENSBTAG00000012994	ENSBTAG00000012994	7:33089539-33101644	0.123498	0	0.313406	OK
ENSBTAG00000027446	ENSBTAG00000027446	13:31347073-31554426	0.123338	0	0.43899	OK
ENSBTAG00000019110	ENSBTAG00000019110	9:61921604-61925635	0.123263	0	0.351938	OK
ENSBTAG00000014069	ENSBTAG00000014069	4:12754201-12767677	0.12321	0	0.328662	OK
ENSBTAG00000013464	ENSBTAG00000013464	1:46431093-46452391	0.123188	0	0.371625	OK
ENSBTAG00000033218	ENSBTAG00000033218	9:45445931-45447231	0.123058	0	0.477824	OK
ENSBTAG00000005547	ENSBTAG00000005547	10:43089377-43094843	0.12274	0	0.488726	OK
ENSBTAG00000047013	ENSBTAG00000047013	24:510107-511067	0.122696	0	0.647053	OK
ENSBTAG00000038156	ENSBTAG00000038156	X:111023273-111034498	0.122667	0	0.490192	OK
ENSBTAG00000008629	ENSBTAG00000008629	14:31735267-31794502	0.12266	0	0.335405	OK
ENSBTAG00000001060	ENSBTAG00000001060	2:61582124-61585838	0.122528	0	0.371292	OK
ENSBTAG00000040169	ENSBTAG00000040169	18:65197725-65211381	0.122492	0	0.388596	OK
ENSBTAG00000012921	ENSBTAG00000012921	8:76990451-77062826	0.122482	0	0.253384	OK
ENSBTAG00000040350	ENSBTAG00000040350	X:7830562-7845821	0.122456	0	0.415777	OK
ENSBTAG00000011510	ENSBTAG00000011510	10:27277493-27278453	0.122356	0	0.647053	OK

ENSBTAG00000006564	ENSBTAG00000006564	15:38745760-38758646	0.122282	0	0.53781	OK
ENSBTAG00000020261	ENSBTAG00000020261	13:57056796-57091107	0.122222	0	0.277556	OK
ENSBTAG00000024643	ENSBTAG00000024643	1:110986516-111211551	0.122156	0	0.435726	OK
ENSBTAG00000033170	ENSBTAG00000033170	1:65680765-65700685	0.121971	0	0.39921	OK
ENSBTAG00000011591	ENSBTAG00000011591	5:5020414-5034909	0.121902	0	0.365705	OK
ENSBTAG00000047888	ENSBTAG00000047888	7:9495368-9496334	0.121874	0	0.643034	OK
ENSBTAG00000014746	ENSBTAG00000014746	7:48012506-48043848	0.121583	0	0.516352	OK
ENSBTAG00000001852	ENSBTAG00000001852	18:24827699-24854528	0.121437	0	0.607184	OK
ENSBTAG00000046608	ENSBTAG00000046608	X:50551257-50552964	0.121371	0	0.372405	OK
ENSBTAG00000047077	ENSBTAG00000047077	26:20494068-20524328	0.121351	0	0.317572	OK
ENSBTAG00000009514	ENSBTAG00000009514	20:13929991-13952132	0.121208	0	0.492758	OK
ENSBTAG00000015637	ENSBTAG00000015637	14:25544906-25560879	0.121185	0	0.259253	OK
ENSBTAG00000004564	ENSBTAG00000004564	1:116238830-116394390	0.121157	0	0.404145	OK
ENSBTAG00000012417	ENSBTAG00000012417	15:61834228-61847775	0.121068	0	0.475993	OK
ENSBTAG00000008511	ENSBTAG00000008511	X:122325330-122327649	0.121041	0	0.446436	OK
ENSBTAG00000020202	ENSBTAG00000020202	10:8038069-8052118	0.121007	0	0.392649	OK
ENSBTAG00000032961	ENSBTAG00000032961	24:47304920-47322103	0.120982	0	0.447852	OK
ENSBTAG00000008674	ENSBTAG00000008674	11:14162022-14166468	0.12086	0	0.352337	OK
ENSBTAG00000011826	ENSBTAG00000011826	5:109855400-109860640	0.120795	0	0.313248	OK
ENSBTAG00000008534	ENSBTAG00000008534	22:36684970-36686398	0.120788	0	0.650441	OK
ENSBTAG00000012982	ENSBTAG00000012982	3:104311411-104315126	0.120743	0	0.3478	OK
ENSBTAG00000004324	ENSBTAG00000004324	X:114437599-114441135	0.120478	0	0.593967	OK
ENSBTAG00000019209	ENSBTAG00000019209	20:39265412-39274082	0.120402	0	0.346829	OK
ENSBTAG00000020792	ENSBTAG00000020792	5:1524730-1628118	0.120323	0	0.411644	OK
ENSBTAG00000000472	ENSBTAG00000000472	18:47656763-47675770	0.120311	0	0.506871	OK
ENSBTAG00000001725	ENSBTAG00000001725	6:92623586-92625937	0.120244	0	0.52731	OK
ENSBTAG00000011024	ENSBTAG00000011024	3:94860379-94895914	0.120111	0	0.444011	OK
ENSBTAG00000007014	ENSBTAG00000007014	1:135871799-135935191	0.120075	0	0.439611	OK
ENSBTAG00000005355	ENSBTAG00000005355	23:2956935-2966980	0.120068	0	0.341115	OK
ENSBTAG00000034430	ENSBTAG00000034430	27:14732023-14745342	0.119774	0	0.413838	OK

ENSBTAG00000040368	ENSBTAG00000040368	10:13992196-14074945	0.119565	0	0.391166	OK
ENSBTAG00000038364	ENSBTAG00000038364	20:56835-57927	0.119257	0	0.65249	OK
ENSBTAG00000047007	ENSBTAG00000047007	15:1174968-1175889	0.119254	0	0.674453	OK
ENSBTAG00000002717	ENSBTAG00000002717	26:24131989-24144138	0.119091	0	0.321317	OK
ENSBTAG00000003257	ENSBTAG00000003257	13:74092648-74179111	0.1189	0	0.32505	OK
ENSBTAG00000019211	ENSBTAG00000019211	20:39256326-39265218	0.118882	0	0.483026	OK
ENSBTAG00000047799	ENSBTAG00000047799	7:15151348-15153336	0.118838	0	0.612595	OK
ENSBTAG00000045800	ENSBTAG00000045800	7:92280133-92281108	0.118668	0	0.663644	OK
ENSBTAG00000005709	ENSBTAG00000005709	24:34929109-34977410	0.118615	0	0.458429	OK
ENSBTAG00000022783	ENSBTAG00000022783	18:29542417-29847569	0.118543	0	0.592716	OK
ENSBTAG00000016070	ENSBTAG00000016070	8:16269364-16271185	0.118433	0	0.341115	OK
ENSBTAG00000021747	ENSBTAG00000021747	14:56787217-56790187	0.118403	0	0.348581	OK
ENSBTAG00000038610	ENSBTAG00000038610	11:94081311-94082670	0.118169	0	0.457079	OK
ENSBTAG00000015244	ENSBTAG00000015244	15:35818588-35819979	0.118148	0	0.637098	OK
ENSBTAG00000003572	ENSBTAG00000003572	27:15350930-15370080	0.118129	0	0.360224	OK
ENSBTAG00000038648	ENSBTAG00000038648	6:85177920-85245141	0.118104	0	0.590519	OK
ENSBTAG00000040279	ENSBTAG00000040279	19:41557544-41561796	0.11805	0	0.367122	OK
ENSBTAG00000000266	ENSBTAG00000000266	2:5567017-5603683	0.118037	0	0.325903	OK
ENSBTAG00000021092	ENSBTAG00000021092	14:78908051-78964199	0.117996	0	0.317248	OK
ENSBTAG00000009673	ENSBTAG00000009673	6:23187623-23254634	0.117914	0	0.377153	OK
ENSBTAG00000048295	ENSBTAG00000048295	5:58540316-58541252	0.117875	0	0.663644	OK
ENSBTAG00000018386	ENSBTAG00000018386	19:18400705-18408200	0.117844	0	0.671391	OK
ENSBTAG00000019255	ENSBTAG00000019255	2:19659539-19692048	0.117742	0	0.430114	OK
ENSBTAG00000011238	ENSBTAG00000011238	3:9022903-9053379	0.117712	0	0.662229	OK
ENSBTAG00000015901	ENSBTAG00000015901	14:31695683-31720448	0.117682	0	0.33966	OK
ENSBTAG00000001233	ENSBTAG00000001233	15:29345978-29376614	0.117672	0	0.35035	OK
ENSBTAG00000039485	ENSBTAG00000039485	19:45036708-45050178	0.117648	0	0.424587	OK
ENSBTAG00000011798	ENSBTAG00000011798	5:82972062-83055263	0.117602	0	0.29636	OK
ENSBTAG00000001788	ENSBTAG00000001788	16:55407770-55442826	0.117316	0	0.410014	OK
ENSBTAG00000011247	ENSBTAG00000011247	4:94634224-94650468	0.117248	0	0.288648	OK

ENSBTAG00000003649	ENSBTAG00000003649	27:37422652-37437162	0.117199	0	0.187325	OK
ENSBTAG00000019028	ENSBTAG00000019028	28:25268531-25328274	0.117095	0	0.344177	OK
ENSBTAG00000013515	ENSBTAG00000013515	5:83279481-83310427	0.116999	0	0.395298	OK
ENSBTAG00000032894	ENSBTAG00000032894	10:40160276-40161181	0.116968	0	0.707484	OK
ENSBTAG00000011531	ENSBTAG00000011531	24:31020508-31096441	0.116908	0	0.355158	OK
ENSBTAG00000004399	ENSBTAG00000004399	4:57586910-57589037	0.116907	0	0.486735	OK
ENSBTAG00000002340	ENSBTAG00000002340	4:72833496-72862043	0.116811	0	0.421419	OK
ENSBTAG00000005609	ENSBTAG00000005609	15:48323708-48332456	0.116655	0	0.440234	OK
ENSBTAG00000046748	ENSBTAG00000046748	3:10508393-10509317	0.116636	0	0.672263	OK
ENSBTAG00000021691	ENSBTAG00000021691	2:35076259-35186366	0.11658	0	0.404145	OK
ENSBTAG00000021992	ENSBTAG00000021992	8:91916164-91928339	0.116313	0	0.359724	OK
ENSBTAG00000011587	ENSBTAG00000011587	26:46273595-46356752	0.116224	0	0.282479	OK
ENSBTAG00000001096	ENSBTAG00000001096	2:18433085-18449877	0.116008	0	0.448499	OK
ENSBTAG00000000754	ENSBTAG00000000754	16:73077107-73151753	0.115953	0	0.340442	OK
ENSBTAG00000008886	ENSBTAG00000008886	27:13499303-13690638	0.115873	0	0.23547	OK
ENSBTAG00000019645	ENSBTAG00000019645	12:90738264-90754997	0.115682	0	0.274006	OK
ENSBTAG00000005100	ENSBTAG00000005100	9:15058057-15102252	0.115681	0	0.371514	OK
ENSBTAG00000040422	ENSBTAG00000040422	14:49706794-49714027	0.115626	0	0.444487	OK
ENSBTAG00000014399	ENSBTAG00000014399	15:64386477-64427635	0.115572	0	0.390011	OK
ENSBTAG00000033671	ENSBTAG00000033671	5:99753915-99756298	0.115293	0	0.64976	OK
ENSBTAG00000025477	ENSBTAG00000025477	7:22897110-22984690	0.115278	0	0.39991	OK
ENSBTAG00000003499	ENSBTAG00000003499	23:19369191-19384102	0.115099	0	0.273282	OK
ENSBTAG00000006191	ENSBTAG00000006191	21:59708511-59723931	0.114892	0	0.358644	OK
ENSBTAG00000021843	ENSBTAG00000021843	X:98958217-98960092	0.114756	0	0.331291	OK
ENSBTAG00000008987	ENSBTAG00000008987	11:28985137-29053248	0.114671	0	0.311833	OK
ENSBTAG00000019672	ENSBTAG00000019672	23:40609759-40611238	0.114646	0	0.419994	OK
ENSBTAG00000010736	ENSBTAG00000010736	2:26800660-26821659	0.114517	0	0.453079	OK
ENSBTAG00000034577	ENSBTAG00000034577	20:72010569-72012136	0.114481	0	0.670811	OK
ENSBTAG00000039731	ENSBTAG00000039731	2:45800465-45822011	0.114297	0	0.376878	OK
ENSBTAG00000002041	ENSBTAG00000002041	X:13681410-13695769	0.114017	0	0.230833	OK

ENSBTAG00000022741	ENSBTAG00000022741	3:20961927-20975445	0.113795	0	0.468455	OK
ENSBTAG00000014319	ENSBTAG00000014319	16:40118744-40472474	0.113747	0	0.336131	OK
ENSBTAG00000004484	ENSBTAG00000004484	5:24226911-24374720	0.113691	0	0.429757	OK
ENSBTAG00000011779	ENSBTAG00000011779	14:47140707-47173966	0.113657	0	0.366732	OK
ENSBTAG00000010778	ENSBTAG00000010778	10:36938123-36948842	0.11358	0	0.498132	OK
ENSBTAG00000004167	ENSBTAG00000004167	17:11479936-11751802	0.11357	0	0.373749	OK
ENSBTAG00000002845	ENSBTAG00000002845	20:48874611-49060950	0.113488	0	0.321417	OK
ENSBTAG00000007923	ENSBTAG00000007923	6:26152252-26163651	0.113446	0	0.4393	OK
ENSBTAG00000018214	ENSBTAG00000018214	12:33578133-33583714	0.113366	0	0.234936	OK
ENSBTAG00000018261	ENSBTAG00000018261	15:66231836-66307403	0.11324	0	0.272085	OK
ENSBTAG00000019387	ENSBTAG00000019387	2:73804849-73815299	0.113174	0	0.387747	OK
ENSBTAG00000010932	ENSBTAG00000010932	28:24397287-24447263	0.113092	0	0.565461	OK
ENSBTAG00000003602	ENSBTAG00000003602	6:47318339-47429147	0.113089	0	0.405994	OK
ENSBTAG00000040473	ENSBTAG00000040473	7:54111954-54114015	0.113014	0	0.301393	OK
ENSBTAG00000048317	ENSBTAG00000048317	14:77383921-77387359	0.112978	0	0.375557	OK
ENSBTAG00000033961	ENSBTAG00000033961	9:26441434-26484894	0.11293	0	0.493777	OK
ENSBTAG00000030956	ENSBTAG00000030956	25:36924065-36931834	0.112895	0	0.319697	OK
ENSBTAG00000013074	ENSBTAG00000013074	16:1966720-1972162	0.112606	0	0.262097	OK
ENSBTAG00000047048	ENSBTAG00000047048	5:91880700-91882214	0.112478	0	1.05019	OK
ENSBTAG00000047350	ENSBTAG00000047350	8:95550068-95552238	0.112179	0	0.490192	OK
ENSBTAG00000000526	ENSBTAG00000000526	1:136833121-136903149	0.112152	0	0.346902	OK
ENSBTAG00000001160	ENSBTAG00000001160	2:125343514-125370269	0.112129	0	0.327104	OK
ENSBTAG00000010386	ENSBTAG00000010386	2:106978525-107006143	0.112092	0	0.453079	OK
ENSBTAG00000015509	ENSBTAG00000015509	4:47597859-47635332	0.112087	0	0.342432	OK
ENSBTAG00000014161	ENSBTAG00000014161	4:44601611-44662241	0.11207	0	0.385342	OK
ENSBTAG00000021307	ENSBTAG00000021307	8:74924183-74947549	0.111774	0	0.478929	OK
ENSBTAG00000013070	ENSBTAG00000013070	1:66900638-66956302	0.111713	0	0.512095	OK
ENSBTAG00000019859	ENSBTAG00000019859	16:12680818-12688442	0.111486	0	0.322418	OK
ENSBTAG00000024361	ENSBTAG00000024361	4:56547058-56548447	0.111421	0	0.467749	OK
ENSBTAG00000026309	ENSBTAG00000026309	14:18386656-18389173	0.111261	0	0.24679	OK

ENSBTAG00000018706	ENSBTAG00000018706	19:6439282-6464109	0.11121	0	0.324541	OK
ENSBTAG00000038986	ENSBTAG00000038986	18:55389247-55390198	0.111123	0	0.653177	OK
ENSBTAG00000014389	ENSBTAG00000014389	4:30321663-30400544	0.111035	0	0.416781	OK
ENSBTAG00000002908	ENSBTAG00000002908	28:8340142-8408741	0.111014	0	0.300155	OK
ENSBTAG00000047224	ENSBTAG00000047224	9:85809353-85810860	0.110849	0	0.464253	OK
ENSBTAG00000019581	ENSBTAG00000019581	14:71515116-71535602	0.110813	0	0.375511	OK
ENSBTAG00000018036	ENSBTAG00000018036	27:19052443-19067489	0.110787	0	0.394394	OK
ENSBTAG00000014529	ENSBTAG00000014529	3:54643138-54683050	0.11078	0	0.364794	OK
ENSBTAG00000008129	ENSBTAG00000008129	5:103581387-103611327	0.11069	0	0.553449	OK
ENSBTAG00000046264	ENSBTAG00000046264	19:43527202-43531702	0.110562	0	0.245619	OK
ENSBTAG00000018218	ENSBTAG00000018218	14:5423555-5657244	0.11045	0	0.296927	OK
ENSBTAG00000037937	ENSBTAG00000037937	15:46854182-46858574	0.110318	0	0.384507	OK
ENSBTAG00000037717	ENSBTAG00000037717	7:54147731-54150161	0.110286	0	0.255626	OK
ENSBTAG00000016522	ENSBTAG00000016522	21:23721988-23728300	0.110264	0	0.289725	OK
ENSBTAG00000020567	ENSBTAG00000020567	11:20929507-20967132	0.110201	0	0.361608	OK
ENSBTAG00000009072	ENSBTAG00000009072	14:16759638-16763916	0.110172	0	0.372628	OK
ENSBTAG00000000885	ENSBTAG00000000885	8:99849409-99850892	0.110061	0	0.435909	OK
ENSBTAG00000020282	ENSBTAG00000020282	27:12916140-12938962	0.109785	0	0.351142	OK
ENSBTAG00000015625	ENSBTAG00000015625	7:64076577-64107651	0.109593	0	0.327795	OK
ENSBTAG00000000275	ENSBTAG00000000275	5:106604204-106638053	0.109365	0	0.256364	OK
ENSBTAG00000004705	ENSBTAG00000004705	1:67082114-67116669	0.10928	0	0.249466	OK
ENSBTAG00000033397	ENSBTAG00000033397	13:60208923-60219601	0.10915	0	0.399467	OK
ENSBTAG00000015180	ENSBTAG00000015180	3:39113551-39114954	0.109097	0	0.599586	OK
ENSBTAG00000018947	ENSBTAG00000018947	10:74384704-74497702	0.109075	0	0.322184	OK
ENSBTAG00000002632	ENSBTAG00000002632	6:87179501-87188025	0.109022	0	0.568317	OK
ENSBTAG00000046729	ENSBTAG00000046729	8:109802370-109803472	0.109003	0	0.664354	OK
ENSBTAG00000002858	ENSBTAG00000002858	13:29940128-29943624	0.108923	0	0.3863	OK
ENSBTAG00000018433	ENSBTAG00000018433	22:44077869-44121649	0.108909	0	0.330938	OK
ENSBTAG00000018694	ENSBTAG00000018694	5:10731240-10905490	0.108798	0	0.318942	OK
ENSBTAG00000004942	ENSBTAG00000004942	6:3419546-3432299	0.108768	0	0.427804	OK

ENSBTAG00000013984	ENSBTAG00000013984	12:28073217-28110415	0.108713	0	0.2044	OK
ENSBTAG00000015505	ENSBTAG00000015505	29:41045231-41082248	0.10835	0	0.210139	OK
ENSBTAG00000018746	ENSBTAG00000018746	2:91475504-91548842	0.108315	0	0.254006	OK
ENSBTAG00000039707	ENSBTAG00000039707	10:28065336-28067016	0.108163	0	0.647053	OK
ENSBTAG00000008721	ENSBTAG00000008721	5:97925814-97949862	0.107997	0	0.331822	OK
ENSBTAG00000000183	ENSBTAG00000000183	1:53995475-54043198	0.107561	0	0.41148	OK
ENSBTAG00000031747	ENSBTAG00000031747	23:31322595-31330574	0.10754	0	0.443377	OK
ENSBTAG00000013294	ENSBTAG00000013294	16:4274966-4284961	0.107524	0	0.292729	OK
ENSBTAG00000019872	ENSBTAG00000019872	26:17781385-17843976	0.107501	0	0.26243	OK
ENSBTAG00000046917	ENSBTAG00000046917	4:48785303-48789698	0.107185	0	0.43868	OK
ENSBTAG00000010944	ENSBTAG00000010944	10:17722004-17875574	0.107132	0	0.405994	OK
ENSBTAG00000009941	ENSBTAG00000009941	21:46916936-46935846	0.107088	0	0.423141	OK
ENSBTAG00000014741	ENSBTAG00000014741	10:69458817-69462865	0.107056	0	0.471656	OK
ENSBTAG00000012762	ENSBTAG00000012762	7:47910524-47917813	0.106937	0	0.31043	OK
ENSBTAG00000014588	ENSBTAG00000014588	25:10596930-10609096	0.106935	0	0.342999	OK
ENSBTAG00000047417	ENSBTAG00000047417	X:69903616-69933552	0.106568	0	0.62086	OK
ENSBTAG00000000240	ENSBTAG00000000240	9:70181053-70304090	0.106566	0	0.511504	OK
ENSBTAG00000026708	ENSBTAG00000026708	9:23710553-23729764	0.106417	0	0.328453	OK
ENSBTAG00000023989	ENSBTAG00000023989	15:1991264-2001671	0.106341	0	0.3591	OK
ENSBTAG00000014374	ENSBTAG00000014374	20:33638643-33653345	0.106204	0	0.307815	OK
ENSBTAG00000013718	ENSBTAG00000013718	6:88695939-88739180	0.10615	0	0.369745	OK
ENSBTAG00000001017	ENSBTAG00000001017	26:24786359-24844579	0.10612	0	0.320522	OK
ENSBTAG00000010664	ENSBTAG00000010664	13:17093613-17185063	0.106107	0	0.325254	OK
ENSBTAG00000037488	ENSBTAG00000037488	15:14118037-14119612	0.105942	0	0.411644	OK
ENSBTAG00000009161	ENSBTAG00000009161	17:4875453-4876992	0.105805	0	0.40362	OK
ENSBTAG00000021819	ENSBTAG00000021819	1:1467703-1496151	0.105763	0	0.314485	OK
ENSBTAG00000030416	ENSBTAG00000030416	18:61931164-61932568	0.105638	0	0.442429	OK
ENSBTAG00000019285	ENSBTAG00000019285	7:109976941-110095864	0.105618	0	0.354145	OK
ENSBTAG00000021372	ENSBTAG00000021372	6:93528979-93628589	0.10554	0	0.335224	OK
ENSBTAG00000015786	ENSBTAG00000015786	3:43251250-43272503	0.105534	0	0.776076	OK

ENSBTAG00000019039	ENSBTAG00000019039	28:46050992-46053236	0.105399	0	0.453079	OK
ENSBTAG00000022656	ENSBTAG00000022656	22:43413165-43416190	0.105343	0	0.376467	OK
ENSBTAG00000017069	ENSBTAG00000017069	17:41769526-41784176	0.105292	0	0.275342	OK
ENSBTAG00000020395	ENSBTAG00000020395	3:19687359-19695881	0.105113	0	0.525567	OK
ENSBTAG00000019960	ENSBTAG00000019960	1:119800139-119823457	0.105062	0	0.530462	OK
ENSBTAG00000015432	ENSBTAG00000015432	18:2882155-2903093	0.105027	0	0.232561	OK
ENSBTAG00000013378	ENSBTAG00000013378	1:117142488-117168355	0.104948	0	0.513789	OK
ENSBTAG00000001250	ENSBTAG00000001250	23:45480545-45499034	0.104899	0	0.22498	OK
ENSBTAG00000015630	ENSBTAG00000015630	X:81551754-81571039	0.104877	0	0.352978	OK
ENSBTAG00000039442	ENSBTAG00000039442	1:98180182-98202494	0.104848	0	0.505017	OK
ENSBTAG00000021287	ENSBTAG00000021287	5:53987908-54214799	0.10483	0	0.42657	OK
ENSBTAG00000045523	ENSBTAG00000045523	6:35008594-35010340	0.104601	0	0.355768	OK
ENSBTAG00000001299	ENSBTAG00000001299	14:30982607-31154016	0.104552	0	0.371514	OK
ENSBTAG00000019295	ENSBTAG00000019295	11:61970466-61994479	0.104533	0	0.489496	OK
ENSBTAG00000000072	ENSBTAG00000000072	16:31552369-31570749	0.104441	0	0.328662	OK
ENSBTAG00000010571	ENSBTAG00000010571	6:73448406-73487539	0.104344	0	0.332889	OK
ENSBTAG00000045787	ENSBTAG00000045787	6:101195830-101270493	0.104339	0	0.448499	OK
ENSBTAG00000030674	ENSBTAG00000030674	3:80491323-80582219	0.104207	0	0.296927	OK
ENSBTAG00000032674	ENSBTAG00000032674	1:71980543-72010052	0.104193	0	0.388912	OK
ENSBTAG00000031020	ENSBTAG00000031020	X:141003991-141007093	0.103828	0	0.336678	OK
ENSBTAG00000019652	ENSBTAG00000019652	X:15811929-15878198	0.103651	0	0.322418	OK
ENSBTAG00000015025	ENSBTAG00000015025	5:38674058-38753871	0.103585	0	0.414761	LOWDATA
ENSBTAG00000002289	ENSBTAG00000002289	15:46301833-46339682	0.103568	0	0.314485	OK
ENSBTAG00000015023	ENSBTAG00000015023	21:49597595-49608630	0.103377	0	0.355768	OK
ENSBTAG00000016867	ENSBTAG00000016867	8:84382335-84436927	0.103323	0	0.383676	OK
ENSBTAG00000007700	ENSBTAG00000007700	13:28254813-28275573	0.103227	0	0.436522	OK
ENSBTAG00000031886	ENSBTAG00000031886	23:30737492-30741740	0.103145	0	0.443377	OK
ENSBTAG00000003128	ENSBTAG00000003128	3:98370382-98536867	0.103125	0	0.413041	OK
ENSBTAG00000010359	ENSBTAG00000010359	7:71361663-71368566	0.103037	0	0.2812	OK
ENSBTAG00000003469	ENSBTAG00000003469	10:77359721-77378295	0.102982	0	0.375511	OK

ENSBTAG00000038533	ENSBTAG00000038533	7:9704189-9705146	0.102856	0	0.649081	OK
ENSBTAG00000004901	ENSBTAG00000004901	15:35867459-35912334	0.102768	0	0.417285	OK
ENSBTAG00000020406	ENSBTAG00000020406	X:17305873-17770661	0.102748	0	0.35638	OK
ENSBTAG00000018432	ENSBTAG00000018432	5:14572185-14604741	0.102674	0	0.321817	OK
ENSBTAG00000001460	ENSBTAG00000001460	15:58994223-59004197	0.102621	0	0.324625	OK
ENSBTAG00000021614	ENSBTAG00000021614	13:7131226-7383858	0.102597	0	0.512983	OK
ENSBTAG00000007270	ENSBTAG00000007270	17:54276967-54293683	0.102501	0	0.442429	OK
ENSBTAG00000025632	ENSBTAG00000025632	10:28351839-28355349	0.102428	0	0.352257	OK
ENSBTAG00000018713	ENSBTAG00000018713	27:34913396-34915034	0.10227	0	0.492993	OK
ENSBTAG00000006147	ENSBTAG00000006147	1:114144238-114229402	0.1022	0	0.277185	OK
ENSBTAG00000011839	ENSBTAG00000011839	20:31451385-31474239	0.102171	0	0.292591	OK
ENSBTAG00000021521	ENSBTAG00000021521	25:13640453-13672230	0.102133	0	0.23204	OK
ENSBTAG00000019037	ENSBTAG00000019037	24:30362737-30377272	0.10203	0	0.290759	OK
ENSBTAG00000025856	ENSBTAG00000025856	10:10242750-10313028	0.101923	0	0.192194	OK
ENSBTAG00000039245	ENSBTAG00000039245	6:95882917-95884576	0.101862	0	0.374425	OK
ENSBTAG00000000545	ENSBTAG00000000545	11:38099215-38150591	0.101851	0	0.376194	OK
ENSBTAG00000031835	ENSBTAG00000031835	23:29038752-29039709	0.10185	0	0.649081	OK
ENSBTAG00000027074	ENSBTAG00000027074	21:16214669-16460490	0.101758	0	0.228414	OK
ENSBTAG00000002805	ENSBTAG00000002805	X:34629930-34631070	0.101659	0	0.908145	OK
ENSBTAG00000005519	ENSBTAG00000005519	8:60970863-61021657	0.101609	0	0.369525	OK
ENSBTAG00000021600	ENSBTAG00000021600	2:98279694-98378538	0.101608	0	0.352241	OK
ENSBTAG00000017135	ENSBTAG00000017135	3:20024301-20047228	0.101604	0	0.368866	OK
ENSBTAG00000030824	ENSBTAG00000030824	10:71992844-72005431	0.101436	0	0.28959	OK
ENSBTAG00000037490	ENSBTAG00000037490	3:54505004-54525762	0.101137	0	0.326588	OK
ENSBTAG00000013809	ENSBTAG00000013809	24:214967-221212	0.101069	0	0.450124	OK
ENSBTAG00000016275	ENSBTAG00000016275	5:60598347-60613063	0.10104	0	0.344903	OK
ENSBTAG00000000898	ENSBTAG00000000898	10:7777599-7788321	0.100582	0	0.361735	OK
ENSBTAG00000008550	ENSBTAG00000008550	4:114075523-114086267	0.100543	0	0.349955	OK
ENSBTAG00000033648	ENSBTAG00000033648	6:20728485-20752923	0.100465	0	0.400755	OK
ENSBTAG00000022205	ENSBTAG00000022205	6:86223187-86239206	0.100134	0	0.383439	OK

ENSBTAG00000003314	ENSBTAG00000003314	12:35841562-35854986	0.100045	0	0.436185	OK
ENSBTAG00000031682	ENSBTAG00000031682	8:85224606-85232666	0.100009	0	0.436277	OK
ENSBTAG00000027764	ENSBTAG00000027764	10:61591975-61593731	0.0999374	0	0.353742	OK
ENSBTAG00000012790	ENSBTAG00000012790	17:64998265-65000579	0.0998986	0	0.499493	OK
ENSBTAG00000046808	ENSBTAG00000046808	7:14947959-14949330	0.0997803	0	0.453079	OK
ENSBTAG00000006036	ENSBTAG00000006036	5:95397670-95422809	0.09975	0	0.453079	OK
ENSBTAG00000017655	ENSBTAG00000017655	3:43688102-43748131	0.0997014	0	0.490656	OK
ENSBTAG00000007130	ENSBTAG00000007130	13:7466617-7532561	0.0995524	0	0.37207	OK
ENSBTAG00000039873	ENSBTAG00000039873	8:7160380-7162249	0.0993336	0	0.340554	OK
ENSBTAG00000039028	ENSBTAG00000039028	13:74272868-74274940	0.0992851	0	0.879845	OK
ENSBTAG00000016332	ENSBTAG00000016332	4:86211496-86237574	0.099261	0	0.450124	OK
ENSBTAG00000047586	ENSBTAG00000047586	6:2415743-2423445	0.0990606	0	0.358106	OK
ENSBTAG00000007730	ENSBTAG00000007730	X:126146066-126168149	0.0988678	0	0.23326	OK
ENSBTAG00000002291	ENSBTAG00000002291	16:78008530-78041752	0.0984979	0	0.379255	OK
ENSBTAG00000015854	ENSBTAG00000015854	3:18686494-18690937	0.0983161	0	0.332978	OK
ENSBTAG00000032515	ENSBTAG00000032515	2:36373938-36508295	0.0982604	0	0.279523	OK
ENSBTAG00000018653	ENSBTAG00000018653	2:80641458-80650245	0.0981955	0	0.337226	OK
ENSBTAG00000008737	ENSBTAG00000008737	3:50242938-50265059	0.0981921	0	0.294603	OK
ENSBTAG00000008099	ENSBTAG00000008099	22:53598838-53602003	0.0980527	0	0.433779	OK
ENSBTAG00000046609	ENSBTAG00000046609	5:101791823-101799616	0.0977413	0	0.602264	OK
ENSBTAG00000017184	ENSBTAG00000017184	X:5043333-5050947	0.0977387	0	0.315215	OK
ENSBTAG00000008120	ENSBTAG00000008120	19:20399048-20403876	0.0973972	0	0.332711	OK
ENSBTAG00000017141	ENSBTAG00000017141	10:67244516-67272546	0.0972882	0	0.393894	OK
ENSBTAG00000020035	ENSBTAG00000020035	1:351707-362907	0.0972788	0	0.273282	OK
ENSBTAG00000003319	ENSBTAG00000003319	13:8041872-8046999	0.0968877	0	0.301759	OK
ENSBTAG00000013262	ENSBTAG00000013262	7:75522539-75539456	0.0968136	0	0.456073	OK
ENSBTAG00000004949	ENSBTAG00000004949	15:22543097-22547643	0.0967843	0	0.331291	OK
ENSBTAG00000009363	ENSBTAG00000009363	18:26282978-26289446	0.0967148	0	0.301686	OK
ENSBTAG00000018360	ENSBTAG00000018360	6:94337923-94408905	0.0966673	0	0.404145	OK
ENSBTAG00000013842	ENSBTAG00000013842	5:64510373-64529644	0.0966057	0	0.40362	OK

ENSBTAG00000019849	ENSBTAG00000019849	6:87469515-87471608	0.0962628	0	0.426043	OK
ENSBTAG00000047635	ENSBTAG00000047635	24:50785277-50810025	0.0962623	0	0.296078	OK
ENSBTAG00000018482	ENSBTAG00000018482	15:78577182-78580931	0.0962283	0	0.26332	OK
ENSBTAG00000012630	ENSBTAG00000012630	15:66754733-66832246	0.096054	0	0.22498	OK
ENSBTAG00000009637	ENSBTAG00000009637	7:26973461-27064924	0.095851	0	0.252879	OK
ENSBTAG00000037932	ENSBTAG00000037932	10:82467737-82473614	0.0957953	0	0.280565	OK
ENSBTAG00000039186	ENSBTAG00000039186	11:92261631-92279410	0.0957369	0	0.421419	OK
ENSBTAG00000010134	ENSBTAG00000010134	9:42551624-42594524	0.0956771	0	0.331503	OK
ENSBTAG00000004388	ENSBTAG00000004388	12:61064672-61067201	0.0955923	0	0.409365	OK
ENSBTAG00000003833	ENSBTAG00000003833	1:133401648-133439465	0.0955428	0	0.396155	OK
ENSBTAG00000037812	ENSBTAG00000037812	11:81239898-81246422	0.0955183	0	0.367122	OK
ENSBTAG00000014674	ENSBTAG00000014674	4:101552611-101554085	0.095439	0	0.421419	OK
ENSBTAG00000006801	ENSBTAG00000006801	19:43813980-43820826	0.0953969	0	0.279807	OK
ENSBTAG00000015569	ENSBTAG00000015569	13:26078956-26115031	0.0952697	0	0.351739	OK
ENSBTAG00000020073	ENSBTAG00000020073	1:95585588-95649520	0.0949748	0	0.267804	OK
ENSBTAG00000003807	ENSBTAG00000003807	2:107256012-107289222	0.0949474	0	0.325254	OK
ENSBTAG00000032893	ENSBTAG00000032893	13:39657080-39658685	0.0949005	0	0.398954	OK
ENSBTAG00000009151	ENSBTAG00000009151	10:54865901-54887753	0.0948599	0	0.409473	OK
ENSBTAG00000037559	ENSBTAG00000037559	26:15931984-15973325	0.0948345	0	0.331822	OK
ENSBTAG00000019026	ENSBTAG00000019026	3:42560282-42578055	0.0945457	0	0.354914	OK
ENSBTAG00000016010	ENSBTAG00000016010	4:31900914-31956645	0.0944752	0	0.333532	OK
ENSBTAG00000019925	ENSBTAG00000019925	7:43291919-43293589	0.0941858	0	0.593286	OK
ENSBTAG00000013451	ENSBTAG00000013451	6:60848685-60896785	0.0938868	0	0.332177	OK
ENSBTAG00000025246	ENSBTAG00000025246	12:80718631-80724166	0.0938306	0	0.469153	OK
ENSBTAG00000039732	ENSBTAG00000039732	23:47244997-47269543	0.0935392	0	0.32185	OK
ENSBTAG00000012894	ENSBTAG00000012894	4:10302666-10307412	0.0934322	0	0.349021	OK
ENSBTAG00000007357	ENSBTAG00000007357	21:5435701-5525361	0.0933743	0	0.271942	OK
ENSBTAG00000038189	ENSBTAG00000038189	16:57209959-57214299	0.0933002	0	0.320026	OK
ENSBTAG00000014166	ENSBTAG00000014166	11:46117226-46139199	0.0932563	0	0.261106	OK
ENSBTAG00000032277	ENSBTAG00000032277	2:53844154-53964125	0.0931932	0	0.391906	OK

ENSBTAG00000006943	ENSBTAG00000006943	19:47474734-47507698	0.0931281	0	0.369745	OK
ENSBTAG00000003769	ENSBTAG00000003769	10:26332079-26333873	0.0930912	0	0.346249	OK
ENSBTAG00000039980	ENSBTAG00000039980	5:100762264-100779160	0.0928902	0	0.575158	OK
ENSBTAG00000000333	ENSBTAG00000000333	15:20909250-21004834	0.0928198	0	0.307024	OK
ENSBTAG00000013498	ENSBTAG00000013498	12:16823523-16889281	0.0927542	0	0.290565	OK
ENSBTAG00000012476	ENSBTAG00000012476	28:19352304-19381439	0.092681	0	0.336313	OK
ENSBTAG00000007386	ENSBTAG00000007386	14:57249476-57342028	0.0924469	0	0.336788	OK
ENSBTAG00000010782	ENSBTAG00000010782	X:3322632-3365424	0.0924251	0	0.264272	OK
ENSBTAG00000002382	ENSBTAG00000002382	15:18644691-18952411	0.0923485	0	0.320522	OK
ENSBTAG00000012818	ENSBTAG00000012818	6:31337011-31567339	0.0922072	0	0.513697	OK
ENSBTAG00000005169	ENSBTAG00000005169	20:14836401-14941022	0.0921243	0	0.286122	OK
ENSBTAG00000011153	ENSBTAG00000011153	5:15741025-15751868	0.0921038	0	0.429579	OK
ENSBTAG00000020650	ENSBTAG00000020650	24:36114520-36121103	0.0920632	0	0.235292	OK
ENSBTAG00000046294	ENSBTAG00000046294	3:54179413-54190203	0.0920058	0	0.411644	OK
ENSBTAG00000016398	ENSBTAG00000016398	5:30641008-30644034	0.0918682	0	0.587203	OK
ENSBTAG00000002151	ENSBTAG00000002151	4:88697391-88729365	0.0917774	0	0.367905	OK
ENSBTAG00000018848	ENSBTAG00000018848	12:52466039-52477380	0.0917524	0	0.341003	OK
ENSBTAG00000003715	ENSBTAG00000003715	10:82506800-82509044	0.0913564	0	0.276814	OK
ENSBTAG00000016002	ENSBTAG00000016002	20:6595533-6657251	0.0912897	0	0.289725	OK
ENSBTAG00000015334	ENSBTAG00000015334	14:18126450-18149245	0.0912005	0	0.26844	OK
ENSBTAG00000037969	ENSBTAG00000037969	13:20930713-20975352	0.0911634	0	0.335224	OK
ENSBTAG00000016550	ENSBTAG00000016550	3:1881238-1937675	0.0910364	0	0.366041	OK
ENSBTAG00000011258	ENSBTAG00000011258	X:126510125-126539314	0.0909146	0	0.376011	OK
ENSBTAG00000013177	ENSBTAG00000013177	X:97899666-97907461	0.0908968	0	0.319368	OK
ENSBTAG00000022306	ENSBTAG00000022306	X:46306537-46308193	0.0907431	0	0.38582	OK
ENSBTAG00000003267	ENSBTAG00000003267	18:65878288-65880106	0.0906897	0	0.341678	OK
ENSBTAG00000013671	ENSBTAG00000013671	X:13790536-13807198	0.0906527	0	0.246496	OK
ENSBTAG00000007662	ENSBTAG00000007662	11:96115571-96119306	0.0905392	0	0.315155	OK
ENSBTAG00000014302	ENSBTAG00000014302	1:44294567-44331743	0.0904466	0	0.286254	OK
ENSBTAG00000003371	ENSBTAG00000003371	10:72005596-72027740	0.0903015	0	0.590242	OK

ENSBTAG00000047655	ENSBTAG00000047655	X:53582626-53588080	0.0902286	0	0.457416	OK
ENSBTAG00000000070	ENSBTAG00000000070	16:77848140-77869023	0.0901194	0	0.284289	OK
ENSBTAG00000018703	ENSBTAG00000018703	1:76685702-76721920	0.0901084	0	0.43257	OK
ENSBTAG00000020892	ENSBTAG00000020892	13:17540960-17586321	0.0900186	0	0.360785	OK
ENSBTAG00000012857	ENSBTAG00000012857	26:23795435-23972895	0.0897669	0	0.18812	OK
ENSBTAG00000005195	ENSBTAG00000005195	25:2675125-2686354	0.0897002	0	0.343948	OK
ENSBTAG00000021965	ENSBTAG00000021965	20:41122022-41143914	0.0896841	0	0.308212	OK
ENSBTAG00000031461	ENSBTAG00000031461	3:44657521-44775833	0.0895904	0	0.353541	OK
ENSBTAG00000004131	ENSBTAG00000004131	X:15973187-15985955	0.08954	0	0.325459	OK
ENSBTAG00000000053	ENSBTAG00000000053	9:15140840-15249645	0.0893688	0	0.242797	OK
ENSBTAG00000003541	ENSBTAG00000003541	18:58180499-58183055	0.0891942	0	0.358851	OK
ENSBTAG00000014181	ENSBTAG00000014181	10:51288676-51380159	0.0890938	0	0.256894	OK
ENSBTAG00000031614	ENSBTAG00000031614	2:86630194-86693857	0.0889588	0	0.444794	OK
ENSBTAG00000007974	ENSBTAG00000007974	9:97434235-97460620	0.0889406	0	0.304645	OK
ENSBTAG00000025062	ENSBTAG00000025062	X:80601976-80604221	0.0889028	0	0.276691	OK
ENSBTAG00000044190	ENSBTAG00000044190	14:72788486-72816854	0.0888416	0	0.33796	OK
ENSBTAG00000011146	ENSBTAG00000011146	10:46837291-46907334	0.0885989	0	0.257427	OK
ENSBTAG00000002166	ENSBTAG00000002166	23:22130934-22156052	0.088522	0	0.462525	OK
ENSBTAG00000007651	ENSBTAG00000007651	21:29296000-29340066	0.0883555	0	0.233172	OK
ENSBTAG00000003959	ENSBTAG00000003959	6:102500169-102666713	0.0883416	0	0.234847	OK
ENSBTAG00000021252	ENSBTAG00000021252	X:54954894-54967415	0.0882437	0	0.302862	OK
ENSBTAG00000038655	ENSBTAG00000038655	29:42247002-42271225	0.0880707	0	0.348385	OK
ENSBTAG00000020824	ENSBTAG00000020824	19:41663604-41667960	0.0879015	0	0.318549	OK
ENSBTAG00000000494	ENSBTAG00000000494	20:20268720-20321935	0.0878711	0	0.353944	OK
ENSBTAG00000039276	ENSBTAG00000039276	2:127471497-127483325	0.087756	0	0.301393	OK
ENSBTAG00000011586	ENSBTAG00000011586	8:84579582-84615685	0.0876825	0	0.231262	OK
ENSBTAG00000011986	ENSBTAG00000011986	1:123231175-123273104	0.0876271	0	0.332355	OK
ENSBTAG00000001839	ENSBTAG00000001839	6:69175253-69187082	0.0874878	0	0.382494	OK
ENSBTAG00000011789	ENSBTAG00000011789	6:73838853-73863778	0.0873075	0	0.211426	OK
ENSBTAG00000006901	ENSBTAG00000006901	8:96238914-96252149	0.0872699	0	0.387747	OK

ENSBTAG00000026172	ENSBTAG00000026172	14:15464447-15469509	0.0870397	0	0.232179	OK
ENSBTAG00000000071	ENSBTAG00000000071	14:46801774-46806914	0.0870131	0	0.419427	OK
ENSBTAG00000006201	ENSBTAG00000006201	10:60913314-60924152	0.0869563	0	0.378763	OK
ENSBTAG00000004745	ENSBTAG00000004745	17:18613424-18687720	0.0868505	0	0.220156	OK
ENSBTAG00000015615	ENSBTAG00000015615	9:66683789-66885925	0.0868462	0	0.309348	OK
ENSBTAG00000037856	ENSBTAG00000037856	11:10196558-10203502	0.0866723	0	0.307358	OK
ENSBTAG00000022504	ENSBTAG00000022504	3:2369383-2385851	0.0866066	0	0.386541	OK
ENSBTAG00000008150	ENSBTAG00000008150	14:43880468-43978277	0.0865788	0	0.330234	OK
ENSBTAG00000027024	ENSBTAG00000027024	21:42937759-43000529	0.0862799	0	0.291288	OK
ENSBTAG00000014923	ENSBTAG00000014923	18:24205241-24231176	0.0860279	0	0.231262	OK
ENSBTAG00000015303	ENSBTAG00000015303	4:71625583-71690219	0.0858003	0	0.369965	OK
ENSBTAG00000019287	ENSBTAG00000019287	7:97094358-97151941	0.0855731	0	0.408934	OK
ENSBTAG00000010587	ENSBTAG00000010587	X:70399115-70527059	0.085569	0	0.378532	OK
ENSBTAG00000001578	ENSBTAG00000001578	10:19377401-19409943	0.0850764	0	0.238912	OK
ENSBTAG00000020367	ENSBTAG00000020367	6:62509247-62594616	0.0850399	0	0.340554	OK
ENSBTAG00000020947	ENSBTAG00000020947	11:48149254-48171946	0.0849109	0	0.294115	OK
ENSBTAG00000018989	ENSBTAG00000018989	4:33769688-34021297	0.0848846	0	0.181682	OK
ENSBTAG00000005257	ENSBTAG00000005257	11:43608811-43640302	0.0848839	0	0.293976	OK
ENSBTAG00000000326	ENSBTAG00000000326	5:111671082-111766560	0.0848417	0	0.338882	OK
ENSBTAG00000011785	ENSBTAG00000011785	16:28258332-28291746	0.0846532	0	0.276938	OK
ENSBTAG00000040567	ENSBTAG00000040567	15:6195377-6209163	0.0846462	0	0.336678	OK
ENSBTAG00000016000	ENSBTAG00000016000	2:91658158-91726299	0.084629	0	0.343948	OK
ENSBTAG00000046566	ENSBTAG00000046566	3:27428792-27430601	0.0845811	0	0.346249	OK
ENSBTAG00000009681	ENSBTAG00000009681	21:45794780-45820504	0.0843975	0	0.411917	OK
ENSBTAG00000019950	ENSBTAG00000019950	7:62740773-62749742	0.0842716	0	0.293781	OK
ENSBTAG00000003045	ENSBTAG00000003045	13:36326193-36333080	0.0842696	0	0.23547	OK
ENSBTAG00000009709	ENSBTAG00000009709	26:24211947-24225837	0.0841535	0	0.257214	OK
ENSBTAG00000034875	ENSBTAG00000034875	6:14280382-14342391	0.0838898	0	0.704361	OK
ENSBTAG00000046914	ENSBTAG00000046914	7:34103728-34117120	0.0836806	0	0.572508	OK
ENSBTAG00000007632	ENSBTAG00000007632	7:82923269-82957971	0.0836065	0	0.372539	OK

ENSBTAG00000039448	ENSBTAG00000039448	15:81500021-81500951	0.0834853	0	0.667926	OK
ENSBTAG00000001192	ENSBTAG00000001192	2:126134950-126170517	0.0832572	0	0.297638	OK
ENSBTAG00000020042	ENSBTAG00000020042	21:55179304-55207518	0.0832142	0	0.315796	OK
ENSBTAG00000039820	ENSBTAG00000039820	1:5035869-5037857	0.0831501	0	0.31246	OK
ENSBTAG00000017170	ENSBTAG00000017170	9:52921131-52922729	0.0828551	0	0.419427	OK
ENSBTAG00000027696	ENSBTAG00000027696	2:19323589-19326224	0.0828461	0	0.235738	OK
ENSBTAG00000048173	ENSBTAG00000048173	X:18627967-18629813	0.0828212	0	0.336496	OK
ENSBTAG00000025005	ENSBTAG00000025005	5:29161682-29172757	0.0826874	0	0.334322	OK
ENSBTAG00000004869	ENSBTAG00000004869	1:18950611-18993431	0.0826007	0	0.298927	OK
ENSBTAG00000011150	ENSBTAG00000011150	1:119151122-119157806	0.082295	0	0.306297	OK
ENSBTAG00000030474	ENSBTAG00000030474	7:54021984-54024905	0.0822741	0	0.212657	OK
ENSBTAG00000030523	ENSBTAG00000030523	19:40868078-40877769	0.082265	0	0.328141	OK
ENSBTAG00000002281	ENSBTAG00000002281	7:2184136-2190127	0.0822369	0	0.377841	OK
ENSBTAG00000021859	ENSBTAG00000021859	9:43099803-43363802	0.0816469	0	0.298927	OK
ENSBTAG00000007214	ENSBTAG00000007214	7:97623183-97650531	0.0814867	0	0.333425	OK
ENSBTAG00000001068	ENSBTAG00000001068	25:36650860-36674762	0.0813083	0	0.475337	OK
ENSBTAG00000032084	ENSBTAG00000032084	12:80142500-80157134	0.0812974	0	0.413013	OK
ENSBTAG00000031435	ENSBTAG00000031435	1:118445767-118476097	0.0811131	0	0.315925	OK
ENSBTAG00000011072	ENSBTAG00000011072	28:30215524-30732444	0.0810767	0	0.342432	OK
ENSBTAG00000002179	ENSBTAG00000002179	10:32328991-32570684	0.0810594	0	0.276199	OK
ENSBTAG00000011844	ENSBTAG00000011844	18:60069202-60077406	0.0810022	0	0.327725	OK
ENSBTAG00000019298	ENSBTAG00000019298	2:90432215-90456178	0.080898	0	0.358437	OK
ENSBTAG00000001796	ENSBTAG00000001796	7:19777110-19813926	0.0808822	0	0.256047	OK
ENSBTAG00000003168	ENSBTAG00000003168	18:50718844-50752307	0.0808798	0	0.170792	OK
ENSBTAG00000032650	ENSBTAG00000032650	4:62259289-62349341	0.0807847	0	0.251792	OK
ENSBTAG00000004072	ENSBTAG00000004072	4:51781848-51836823	0.0806972	0	0.392401	OK
ENSBTAG00000013224	ENSBTAG00000013224	9:89612881-89648757	0.0806208	0	0.319335	OK
ENSBTAG00000017508	ENSBTAG00000017508	12:18525479-18531621	0.0805149	0	0.307663	OK
ENSBTAG00000010419	ENSBTAG00000010419	15:38420925-38445583	0.0803405	0	0.401703	OK
ENSBTAG00000038480	ENSBTAG00000038480	3:70878976-70887093	0.0802666	0	0.305214	OK

ENSBTAG00000016885	ENSBTAG00000016885	28:23510347-23719897	0.0799002	0	0.286122	OK
ENSBTAG00000002135	ENSBTAG00000002135	5:100740573-100747672	0.0797246	0	0.364751	OK
ENSBTAG00000006186	ENSBTAG00000006186	16:19500370-19567831	0.0796117	0	0.249868	OK
ENSBTAG00000005497	ENSBTAG00000005497	24:21361883-21427671	0.0789046	0	0.662757	OK
ENSBTAG00000046773	ENSBTAG00000046773	3:59303475-59351124	0.0788804	0	0.296927	OK
ENSBTAG00000011709	ENSBTAG00000011709	14:83277474-83299881	0.0787464	0	0.265912	OK
ENSBTAG00000019500	ENSBTAG00000019500	10:67176768-67191630	0.0787286	0	0.393643	OK
ENSBTAG00000008226	ENSBTAG00000008226	X:25262322-25265111	0.0786746	0	0.573036	OK
ENSBTAG00000000287	ENSBTAG00000000287	6:64961797-64992219	0.0785859	0	0.342999	OK
ENSBTAG00000010803	ENSBTAG00000010803	16:35948786-35950742	0.0785538	0	0.317572	OK
ENSBTAG00000023202	ENSBTAG00000023202	15:81448318-81451166	0.0784865	0	0.639065	OK
ENSBTAG00000005308	ENSBTAG00000005308	15:7148660-7165793	0.0784649	0	0.373076	OK
ENSBTAG00000000492	ENSBTAG00000000492	2:6605443-6616484	0.0784027	0	0.260558	OK
ENSBTAG00000013112	ENSBTAG00000013112	7:47231928-47244897	0.0783198	0	0.288917	OK
ENSBTAG00000008931	ENSBTAG00000008931	3:1196981-1208368	0.0783089	0	0.312775	OK
ENSBTAG00000011494	ENSBTAG00000011494	10:43800151-43840994	0.0782782	0	0.222323	OK
ENSBTAG00000015047	ENSBTAG00000015047	6:86676446-86713666	0.078063	0	0.327968	OK
ENSBTAG00000045653	ENSBTAG00000045653	X:75958543-75982689	0.0779491	0	0.353098	OK
ENSBTAG00000016355	ENSBTAG00000016355	15:55992136-56317353	0.0779034	0	0.277185	OK
ENSBTAG00000012708	ENSBTAG00000012708	1:100456487-100539394	0.0776917	0	0.392153	OK
ENSBTAG00000002163	ENSBTAG00000002163	10:11773705-11786080	0.0775502	0	0.250371	OK
ENSBTAG00000039709	ENSBTAG00000039709	5:66293050-66359522	0.0772321	0	0.342054	OK
ENSBTAG00000007116	ENSBTAG00000007116	7:93240418-93253094	0.0770145	0	0.271847	OK
ENSBTAG00000047780	ENSBTAG00000047780	20:71964290-71979896	0.0769005	0	0.227535	OK
ENSBTAG00000031397	ENSBTAG00000031397	1:117692320-117695064	0.0766538	0	0.37729	OK
ENSBTAG00000001435	ENSBTAG00000001435	2:43543015-43584232	0.076568	0	0.305996	OK
ENSBTAG00000019353	ENSBTAG00000019353	6:47941558-48006406	0.0765474	0	0.283899	OK
ENSBTAG00000024826	ENSBTAG00000024826	6:81511553-81653990	0.0764026	0	0.381086	OK
ENSBTAG00000006734	ENSBTAG00000006734	5:77857447-77924511	0.0763961	0	0.427519	OK
ENSBTAG00000038195	ENSBTAG00000038195	2:36656332-36681937	0.0763572	0	0.324337	OK

ENSBTAG00000040187	ENSBTAG00000040187	23:29636420-29637531	0.0763493	0	0.653177	OK
ENSBTAG00000013851	ENSBTAG00000013851	3:59010763-59020234	0.0763444	0	0.32185	OK
ENSBTAG00000012931	ENSBTAG00000012931	9:32823611-32834377	0.0762519	0	0.395751	OK
ENSBTAG00000024534	ENSBTAG00000024534	3:1328185-1415265	0.0761644	0	0.23109	OK
ENSBTAG00000013400	ENSBTAG00000013400	9:90889141-90897698	0.075931	0	0.392897	OK
ENSBTAG00000046561	ENSBTAG00000046561	6:38840893-38992112	0.0758981	0	0.247577	OK
ENSBTAG00000024027	ENSBTAG00000024027	1:46339131-46343357	0.0756201	0	0.369965	OK
ENSBTAG00000005723	ENSBTAG00000005723	1:58170477-58185755	0.0755379	0	0.366472	OK
ENSBTAG00000009354	ENSBTAG00000009354	19:19020656-19024736	0.0754593	0	0.408128	OK
ENSBTAG00000012553	ENSBTAG00000012553	9:91531128-91533874	0.0753789	0	0.317897	OK
ENSBTAG00000021577	ENSBTAG00000021577	7:82875304-82908670	0.0752402	0	0.244123	OK
ENSBTAG00000010387	ENSBTAG00000010387	13:30604488-30684245	0.0752391	0	0.271847	OK
ENSBTAG00000007681	ENSBTAG00000007681	7:83024157-83040758	0.0750234	0	0.28838	OK
ENSBTAG00000019214	ENSBTAG00000019214	24:35529295-35564664	0.0748739	0	0.255731	OK
ENSBTAG00000020054	ENSBTAG00000020054	23:20410838-20474989	0.0747657	0	0.174732	OK
ENSBTAG00000030286	ENSBTAG00000030286	7:75703665-75759741	0.0746973	0	0.264103	OK
ENSBTAG00000021587	ENSBTAG00000021587	9:28811577-28828968	0.0746968	0	0.317086	OK
ENSBTAG00000013974	ENSBTAG00000013974	8:11198677-11204065	0.0744784	0	0.241185	OK
ENSBTAG00000033313	ENSBTAG00000033313	20:23461325-23565673	0.074326	0	0.253332	OK
ENSBTAG00000001977	ENSBTAG00000001977	X:87205698-87231196	0.0742307	0	0.27374	OK
ENSBTAG00000001911	ENSBTAG00000001911	X:42583761-42621146	0.0741181	0	0.342696	OK
ENSBTAG00000002781	ENSBTAG00000002781	10:6432187-6433552	0.0740044	0	0.45507	OK
ENSBTAG00000013147	ENSBTAG00000013147	17:70821077-70828226	0.0732197	0	0.191188	OK
ENSBTAG00000014752	ENSBTAG00000014752	12:12459629-12495973	0.0732102	0	0.209713	OK
ENSBTAG00000014561	ENSBTAG00000014561	28:25404675-25459344	0.0731983	0	0.251894	OK
ENSBTAG00000013985	ENSBTAG00000013985	18:54288965-54297589	0.0731479	0	0.154983	OK
ENSBTAG00000008260	ENSBTAG00000008260	14:39233173-39295068	0.0731468	0	0.326417	OK
ENSBTAG00000002267	ENSBTAG00000002267	19:36655573-36675085	0.0730538	0	0.170511	OK
ENSBTAG00000016077	ENSBTAG00000016077	7:14734732-14747553	0.0729424	0	0.23049	OK
ENSBTAG00000017157	ENSBTAG00000017157	27:14748842-14760880	0.0728406	0	0.412793	OK

ENSBTAG00000015019	ENSBTAG00000015019	26:24700353-24737868	0.072835	0	0.297924	OK
ENSBTAG00000008326	ENSBTAG00000008326	X:78972754-78974555	0.0727781	0	0.428098	OK
ENSBTAG00000004997	ENSBTAG00000004997	8:100221979-100277722	0.0727491	0	0.276322	OK
ENSBTAG00000000687	ENSBTAG00000000687	5:19357950-19462573	0.0727489	0	0.246301	OK
ENSBTAG00000009694	ENSBTAG00000009694	11:43541230-43603979	0.0723428	0	0.345479	OK
ENSBTAG00000009381	ENSBTAG00000009381	20:2032972-2082267	0.0723306	0	0.259578	OK
ENSBTAG00000018317	ENSBTAG00000018317	8:74834627-74913332	0.0721481	0	0.294533	OK
ENSBTAG00000003684	ENSBTAG00000003684	21:51772759-52095083	0.0721419	0	0.294115	OK
ENSBTAG00000009290	ENSBTAG00000009290	11:60352575-60381305	0.0719707	0	0.256364	OK
ENSBTAG00000003144	ENSBTAG00000003144	7:96124691-96354407	0.0719097	0	0.257854	OK
ENSBTAG00000015129	ENSBTAG00000015129	18:57448224-57454197	0.0718765	0	0.2674	OK
ENSBTAG00000006065	ENSBTAG00000006065	13:47788529-47794602	0.0718679	0	0.31246	OK
ENSBTAG00000032640	ENSBTAG00000032640	25:14350017-14368763	0.0715812	0	0.260558	OK
ENSBTAG00000014661	ENSBTAG00000014661	7:25178591-25466387	0.0715759	0	0.234493	OK
ENSBTAG00000034827	ENSBTAG00000034827	15:4501434-4788300	0.0715545	0	0.302273	OK
ENSBTAG00000047147	ENSBTAG00000047147	1:41700078-41756925	0.0713555	0	0.257341	OK
ENSBTAG00000005825	ENSBTAG00000005825	27:7557040-7603267	0.0711632	0	0.302715	OK
ENSBTAG00000011952	ENSBTAG00000011952	6:87035925-87094952	0.0706042	0	0.343378	OK
ENSBTAG00000012858	ENSBTAG00000012858	26:23983205-24080557	0.0705921	0	0.209431	OK
ENSBTAG00000020194	ENSBTAG00000020194	X:22043209-22118277	0.0703559	0	0.264441	OK
ENSBTAG00000040255	ENSBTAG00000040255	X:50811121-50813176	0.07012	0	0.394645	OK
ENSBTAG00000003878	ENSBTAG00000003878	9:34364850-34389669	0.0701149	0	0.282094	OK
ENSBTAG00000004411	ENSBTAG00000004411	14:40420995-40471464	0.0701115	0	0.213608	OK
ENSBTAG00000021083	ENSBTAG00000021083	5:89116788-89149899	0.0700262	0	0.350131	OK
ENSBTAG00000019984	ENSBTAG00000019984	13:54063353-54079411	0.0695852	0	0.229638	OK
ENSBTAG00000020806	ENSBTAG00000020806	5:47713519-47751430	0.0695455	0	0.242758	OK
ENSBTAG00000005498	ENSBTAG00000005498	14:16723392-16745626	0.0695343	0	0.288514	OK
ENSBTAG00000010956	ENSBTAG00000010956	6:92797819-92879565	0.0695285	0	0.233857	OK
ENSBTAG00000016006	ENSBTAG00000016006	18:14371891-14436368	0.069468	0	0.131348	OK
ENSBTAG00000021988	ENSBTAG00000021988	10:67735432-67749358	0.069393	0	0.280692	OK

ENSBTAG00000021214	ENSBTAG00000021214	6:115573480-115610298	0.0693273	0	0.282736	OK
ENSBTAG00000007703	ENSBTAG00000007703	13:29967416-30027451	0.0691835	0	0.345917	OK
ENSBTAG00000017426	ENSBTAG00000017426	22:8092170-8160760	0.0691142	0	0.214641	OK
ENSBTAG00000016691	ENSBTAG00000016691	12:14136841-14145893	0.0690207	0	0.396155	OK
ENSBTAG00000011981	ENSBTAG00000011981	14:32840048-32862669	0.0690201	0	0.281966	OK
ENSBTAG00000046358	ENSBTAG00000046358	14:65816005-65833756	0.0687966	0	0.218031	OK
ENSBTAG00000012827	ENSBTAG00000012827	11:92737268-92903570	0.0687849	0	0.203037	OK
ENSBTAG00000039815	ENSBTAG00000039815	11:94072876-94074965	0.068713	0	0.297353	OK
ENSBTAG00000019371	ENSBTAG00000019371	2:129597585-129634311	0.0684948	0	0.249566	OK
ENSBTAG00000034347	ENSBTAG00000034347	1:22455499-22474290	0.0683474	0	0.341737	OK
ENSBTAG00000006607	ENSBTAG00000006607	7:77249996-77257261	0.0682404	0	0.268208	OK
ENSBTAG00000002296	ENSBTAG00000002296	7:52361792-52368150	0.0680338	0	0.247085	OK
ENSBTAG00000002642	ENSBTAG00000002642	23:31721013-31739244	0.0675485	0	0.312932	OK
ENSBTAG00000038715	ENSBTAG00000038715	18:65496393-65506427	0.0675331	0	0.309966	OK
ENSBTAG00000003871	ENSBTAG00000003871	18:50564355-50581387	0.0675114	0	0.276322	OK
ENSBTAG00000004547	ENSBTAG00000004547	5:100244331-100255640	0.0673554	0	0.328488	OK
ENSBTAG00000008079	ENSBTAG00000008079	14:2152611-2159648	0.0673322	0	0.188921	OK
ENSBTAG00000002232	ENSBTAG00000002232	1:127257266-127318221	0.0671087	0	0.402652	OK
ENSBTAG00000019021	ENSBTAG00000019021	17:36566053-36616303	0.0668331	0	0.267332	OK
ENSBTAG00000002613	ENSBTAG00000002613	21:55398773-55477656	0.0668088	0	0.324948	OK
ENSBTAG00000015337	ENSBTAG00000015337	4:86039504-86111099	0.0667857	0	0.268905	OK
ENSBTAG00000015010	ENSBTAG00000015010	1:44199314-44254410	0.0667368	0	0.294394	OK
ENSBTAG00000008682	ENSBTAG00000008682	27:15232458-15243399	0.06672	0	0.342243	OK
ENSBTAG00000040132	ENSBTAG00000040132	X:53703693-53705778	0.0666557	0	0.297924	OK
ENSBTAG00000019794	ENSBTAG00000019794	4:47423618-47453580	0.0666454	0	0.279555	OK
ENSBTAG00000014911	ENSBTAG00000014911	4:93249873-93266624	0.066474	0	0.211931	OK
ENSBTAG00000001254	ENSBTAG00000001254	14:65992658-66033894	0.0663443	0	0.227036	OK
ENSBTAG00000023279	ENSBTAG00000023279	12:28545677-28560619	0.0663399	0	0.322686	OK
ENSBTAG00000004425	ENSBTAG00000004425	5:5813256-5816003	0.066169	0	0.28932	OK
ENSBTAG00000007773	ENSBTAG00000007773	3:42703655-42725971	0.0661034	0	0.330517	OK

ENSBTAG00000014551	ENSBTAG00000014551	7:35812576-35858617	0.0659677	0	0.341115	OK
ENSBTAG00000026286	ENSBTAG00000026286	25:33212216-33259588	0.0653739	0	0.191838	OK
ENSBTAG00000012984	ENSBTAG00000012984	11:21130361-21132147	0.0653035	0	0.355768	OK
ENSBTAG00000008280	ENSBTAG00000008280	14:40830953-40965775	0.0649004	0	0.226691	OK
ENSBTAG00000015800	ENSBTAG00000015800	4:73609245-73611951	0.0648761	0	0.342054	OK
ENSBTAG00000014261	ENSBTAG00000014261	20:40349624-40373064	0.0647856	0	0.222085	OK
ENSBTAG00000006714	ENSBTAG00000006714	26:33254109-33335575	0.0642787	0	0.308426	OK
ENSBTAG00000014158	ENSBTAG00000014158	2:62649167-62681201	0.0641913	0	0.207597	OK
ENSBTAG00000013569	ENSBTAG00000013569	6:115802868-115858942	0.0638129	0	0.260996	OK
ENSBTAG00000003358	ENSBTAG00000003358	11:29200742-29210371	0.0634029	0	0.32505	OK
ENSBTAG00000017244	ENSBTAG00000017244	13:28565186-28620268	0.0628576	0	0.262319	OK
ENSBTAG00000010822	ENSBTAG00000010822	20:33030482-33230694	0.0626224	0	0.287048	OK
ENSBTAG00000012741	ENSBTAG00000012741	10:55001791-55028162	0.0622792	0	0.265912	OK
ENSBTAG00000022161	ENSBTAG00000022161	X:141044354-141063596	0.0622134	0	0.304227	OK
ENSBTAG00000017741	ENSBTAG00000017741	8:23339674-23369117	0.0621748	0	0.308426	OK
ENSBTAG00000000013	ENSBTAG00000000013	20:33688290-33716677	0.0621546	0	0.216761	OK
ENSBTAG00000004964	ENSBTAG00000004964	26:12879193-12931310	0.0620617	0	0.245425	OK
ENSBTAG00000014432	ENSBTAG00000014432	2:59376268-59417330	0.0619288	0	0.330762	OK
ENSBTAG00000043950	ENSBTAG00000043950	9:19327495-19368400	0.0617667	0	0.302273	OK
ENSBTAG00000020004	ENSBTAG00000020004	4:92424190-92444252	0.0616366	0	0.302126	OK
ENSBTAG00000007921	ENSBTAG00000007921	6:26167968-26224688	0.0613108	0	0.233787	OK
ENSBTAG00000009106	ENSBTAG00000009106	1:118495011-118536744	0.0612591	0	0.245814	OK
ENSBTAG00000024387	ENSBTAG00000024387	4:49867701-49907595	0.0609336	0	0.258176	OK
ENSBTAG00000021323	ENSBTAG00000021323	2:116441986-116516520	0.0607806	0	0.245522	OK
ENSBTAG00000012554	ENSBTAG00000012554	2:6544588-6596238	0.0607146	0	0.211996	OK
ENSBTAG00000033413	ENSBTAG00000033413	28:25335073-25368153	0.0606716	0	0.257214	OK
ENSBTAG00000011028	ENSBTAG00000011028	X:81008165-81167688	0.0605006	0	0.255626	OK
ENSBTAG00000002020	ENSBTAG00000002020	20:4684743-4740893	0.0594293	0	0.297146	OK
ENSBTAG00000008243	ENSBTAG00000008243	3:75294025-75332667	0.0593712	0	0.339995	OK
ENSBTAG00000006140	ENSBTAG00000006140	21:21523626-21530708	0.0591774	0	0.23291	OK

ENSBTAG00000003143	ENSBTAG00000003143	1:81256662-81279171	0.0591424	0	0.254787	OK
ENSBTAG00000010422	ENSBTAG00000010422	5:45178134-45203567	0.0589977	0	0.232997	OK
ENSBTAG00000022731	ENSBTAG00000022731	23:47853792-47871932	0.0588612	0	0.198077	OK
ENSBTAG00000001571	ENSBTAG00000001571	4:9334913-9351034	0.0587302	0	0.399415	OK
ENSBTAG00000038806	ENSBTAG00000038806	17:45140913-45151805	0.0581912	0	0.302273	OK
ENSBTAG00000000176	ENSBTAG00000000176	2:22841313-22888725	0.0579638	0	0.273644	OK
ENSBTAG00000005293	ENSBTAG00000005293	7:51313473-51344138	0.0578536	0	0.257107	OK
ENSBTAG00000017354	ENSBTAG00000017354	13:36610251-36833259	0.057803	0	0.190251	OK
ENSBTAG00000000900	ENSBTAG00000000900	3:53717831-53810701	0.0575005	0	0.222722	OK
ENSBTAG00000009834	ENSBTAG00000009834	11:3553256-3590989	0.0570462	0	0.157219	OK
ENSBTAG00000026234	ENSBTAG00000026234	14:67677675-67987801	0.0570433	0	0.221768	OK
ENSBTAG00000022588	ENSBTAG00000022588	14:34427578-34830956	0.0567221	0	0.222482	OK
ENSBTAG00000031707	ENSBTAG00000031707	10:44342322-44615289	0.0566602	0	0.206561	OK
ENSBTAG00000006494	ENSBTAG00000006494	1:77469356-77492294	0.0566	0	0.214419	OK
ENSBTAG00000039904	ENSBTAG00000039904	X:116418808-116422639	0.0559267	0	0.306448	OK
ENSBTAG00000008371	ENSBTAG00000008371	19:40379411-40478817	0.0558638	0	0.199862	OK
ENSBTAG00000021471	ENSBTAG00000021471	7:104530076-104559401	0.0556332	0	0.523556	OK
ENSBTAG00000034883	ENSBTAG00000034883	22:7383751-7388696	0.0553663	0	0.237905	OK
ENSBTAG00000039163	ENSBTAG00000039163	23:31490461-31494121	0.0551944	0	0.169719	OK
ENSBTAG00000007648	ENSBTAG00000007648	13:43526441-43538233	0.0551084	0	0.196698	OK
ENSBTAG00000006755	ENSBTAG00000006755	15:36224847-36251371	0.0546819	0	0.215909	OK
ENSBTAG00000005392	ENSBTAG00000005392	15:1802548-1889711	0.0541771	0	0.321185	OK
ENSBTAG00000040357	ENSBTAG00000040357	X:114144058-114148031	0.0541269	0	0.273644	OK
ENSBTAG00000015311	ENSBTAG00000015311	5:42837596-43112731	0.0540936	0	0.19279	OK
ENSBTAG00000001572	ENSBTAG00000001572	6:61311621-61357185	0.0537978	0	0.262985	OK
ENSBTAG00000012074	ENSBTAG00000012074	9:74222881-74258203	0.0534686	0	0.267343	OK
ENSBTAG00000012371	ENSBTAG00000012371	19:22025795-22086708	0.0533544	0	0.186762	OK
ENSBTAG00000016622	ENSBTAG00000016622	22:16310517-16348300	0.0533472	0	0.266736	OK
ENSBTAG00000006816	ENSBTAG00000006816	8:48407616-48455649	0.053321	0	0.247972	OK
ENSBTAG00000045794	ENSBTAG00000045794	4:100261071-100277950	0.0532902	0	0.306903	OK

ENSBTAG00000040063	ENSBTAG00000040063	18:47466373-47473766	0.053262	0	0.279429	OK
ENSBTAG00000002972	ENSBTAG00000002972	5:85217885-85310153	0.0526402	0	0.280059	OK
ENSBTAG00000030539	ENSBTAG00000030539	19:41515534-41524688	0.0525866	0	0.285333	OK
ENSBTAG00000022920	ENSBTAG00000022920	X:56840832-56905852	0.0523176	0	0.221768	OK
ENSBTAG00000017811	ENSBTAG00000017811	10:81533906-81577272	0.0520785	0	0.176319	OK
ENSBTAG00000019458	ENSBTAG00000019458	11:36254029-36371025	0.0520457	0	0.184378	OK
ENSBTAG00000012454	ENSBTAG00000012454	3:43400345-43444844	0.0516338	0	0.209064	OK
ENSBTAG00000039671	ENSBTAG00000039671	26:37282718-37285100	0.0513747	0	0.260777	OK
ENSBTAG00000046056	ENSBTAG00000046056	9:50574573-50577379	0.0511311	0	0.223042	OK
ENSBTAG00000016882	ENSBTAG00000016882	28:25683638-25708826	0.0508941	0	0.23326	OK
ENSBTAG00000021342	ENSBTAG00000021342	15:31440212-31446969	0.0500654	0	0.170745	OK
ENSBTAG00000012170	ENSBTAG00000012170	12:30868718-30881788	0.0498789	0	0.193874	OK
ENSBTAG00000031934	ENSBTAG00000031934	21:54748362-54751240	0.0497346	0	0.215834	OK
ENSBTAG00000000501	ENSBTAG00000000501	X:1975397-2038024	0.0496242	0	0.188806	OK
ENSBTAG00000017271	ENSBTAG00000017271	13:18015908-18038511	0.0495248	0	0.227036	OK
ENSBTAG00000015348	ENSBTAG00000015348	8:92751542-92762917	0.0494408	0	0.208657	OK
ENSBTAG00000010428	ENSBTAG00000010428	5:51380095-51510100	0.0494358	0	0.212876	OK
ENSBTAG00000007870	ENSBTAG00000007870	4:66758678-66770727	0.0493847	0	0.211354	OK
ENSBTAG00000016217	ENSBTAG00000016217	2:44961706-44973211	0.0487326	0	0.230747	OK
ENSBTAG00000000502	ENSBTAG00000000502	1:155383457-155406442	0.048252	0	0.211498	OK
ENSBTAG00000012566	ENSBTAG00000012566	28:5711872-5714658	0.0482135	0	0.222962	OK
ENSBTAG00000007442	ENSBTAG00000007442	4:9162748-9303751	0.0481809	0	0.141083	OK
ENSBTAG00000004939	ENSBTAG00000004939	18:47619375-47630372	0.0480568	0	0.301832	OK
ENSBTAG00000002974	ENSBTAG00000002974	16:39612585-39656811	0.0478868	0	0.210638	OK
ENSBTAG00000040055	ENSBTAG00000040055	2:119340635-119443035	0.0476866	0	0.161134	OK
ENSBTAG00000016433	ENSBTAG00000016433	X:135904188-135921413	0.0476131	0	0.240484	OK
ENSBTAG00000015200	ENSBTAG00000015200	10:43211977-43265008	0.0469346	0	0.190368	OK
ENSBTAG00000021344	ENSBTAG00000021344	X:16857811-16860502	0.0469125	0	0.230833	OK
ENSBTAG00000010299	ENSBTAG00000010299	24:23166233-23627792	0.0463414	0	0.278048	OK
ENSBTAG00000021742	ENSBTAG00000021742	18:47384123-47406577	0.0457993	0	0.281073	OK

ENSBTAG00000002292	ENSBTAG00000002292	X:13358798-13433781	0.0455819	0	0.170792	OK
ENSBTAG00000040206	ENSBTAG00000040206	10:30525209-30527276	0.0455685	0	0.300518	OK
ENSBTAG00000001607	ENSBTAG00000001607	15:43901751-43929481	0.0453146	0	0.191779	OK
ENSBTAG00000009085	ENSBTAG00000009085	1:57786941-57807544	0.0452292	0	0.200766	OK
ENSBTAG00000016473	ENSBTAG00000016473	27:38862139-38868825	0.0450512	0	0.226622	OK
ENSBTAG00000021582	ENSBTAG00000021582	6:38765968-38812051	0.0449215	0	0.193391	OK
ENSBTAG00000012271	ENSBTAG00000012271	5:1278216-1293311	0.0443835	0	0.207403	OK
ENSBTAG00000015778	ENSBTAG00000015778	3:43291332-43331499	0.043955	0	0.214715	OK
ENSBTAG00000010719	ENSBTAG00000010719	16:61657544-61681733	0.0439021	0	0.243596	OK
ENSBTAG00000006639	ENSBTAG00000006639	13:60077490-60091145	0.0438918	0	0.186426	OK
ENSBTAG00000034090	ENSBTAG00000034090	18:60599036-60640035	0.0436429	0	0.178806	OK
ENSBTAG00000003690	ENSBTAG00000003690	26:34584584-34640028	0.042609	0	0.163251	OK
ENSBTAG00000009232	ENSBTAG00000009232	1:88504289-88533061	0.0425497	0	0.193632	OK
ENSBTAG00000010196	ENSBTAG00000010196	9:88051511-88066828	0.0422848	0	0.194298	OK
ENSBTAG00000025920	ENSBTAG00000025920	6:85410240-85452310	0.0411882	0	0.228961	OK
ENSBTAG00000001805	ENSBTAG00000001805	24:41986505-42008147	0.0408567	0	0.176569	OK
ENSBTAG00000001533	ENSBTAG00000001533	9:45735181-45855545	0.0407316	0	0.176569	OK
ENSBTAG00000009140	ENSBTAG00000009140	X:112565064-112621983	0.0405636	0	0.215012	OK
ENSBTAG00000008142	ENSBTAG00000008142	2:34217172-34272473	0.0400401	0	0.186426	OK
ENSBTAG00000007237	ENSBTAG00000007237	10:35903020-35958915	0.0399933	0	0.166937	OK
ENSBTAG00000004581	ENSBTAG00000004581	15:84546972-84574455	0.0394516	0	0.206438	OK
ENSBTAG00000038700	ENSBTAG00000038700	2:113274594-113287683	0.0391372	0	0.164549	OK
ENSBTAG00000030470	ENSBTAG00000030470	18:58688853-58765602	0.0390552	0	0.207889	OK
ENSBTAG00000008699	ENSBTAG00000008699	10:4305631-4334954	0.0388316	0	0.164679	OK
ENSBTAG00000012918	ENSBTAG00000012918	23:22162641-22189911	0.0388037	0	0.236276	OK
ENSBTAG00000017083	ENSBTAG00000017083	27:26078604-26094196	0.0384713	0	0.150168	OK
ENSBTAG00000038347	ENSBTAG00000038347	29:22698848-22739291	0.0382542	0	0.178395	OK
ENSBTAG00000018245	ENSBTAG00000018245	20:37596603-37679976	0.0373898	0	0.160261	OK
ENSBTAG00000027728	ENSBTAG00000027728	7:105030975-105045004	0.0371482	0	0.180259	OK
ENSBTAG00000002002	ENSBTAG00000002002	14:18046446-18108338	0.0365357	0	0.440369	OK

ENSBTAG00000014884	ENSBTAG00000014884	15:64704078-64758178	0.0361719	0	0.160468	OK
ENSBTAG00000040031	ENSBTAG00000040031	1:70287256-70382437	0.035495	0	0.136114	OK
ENSBTAG00000021967	ENSBTAG00000021967	4:56459730-56517535	0.0352318	0	0.155176	OK
ENSBTAG00000002956	ENSBTAG00000002956	X:102851710-102885917	0.0350951	0	0.180941	OK
ENSBTAG00000037783	ENSBTAG00000037783	5:40638740-40667231	0.0350809	0	0.17298	OK
ENSBTAG00000010227	ENSBTAG00000010227	21:57395845-57429002	0.0346145	0	0.16407	OK
ENSBTAG00000003221	ENSBTAG00000003221	5:95800987-95858963	0.0340024	0	0.166534	OK
ENSBTAG00000006555	ENSBTAG00000006555	3:111180175-111205859	0.0339792	0	0.170745	OK
ENSBTAG00000026122	ENSBTAG00000026122	2:11593229-11598298	0.0323886	0	0.184269	OK
ENSBTAG00000025311	ENSBTAG00000025311	10:67212807-67226749	0.0321131	0	0.19166	OK
ENSBTAG00000001523	ENSBTAG00000001523	24:35973658-36038238	0.0308626	0	0.125009	OK
ENSBTAG00000024632	ENSBTAG00000024632	2:56711719-57011911	0.0298733	0	0.119894	OK
ENSBTAG00000007709	ENSBTAG00000007709	26:31762791-31849699	0.0280345	0	0.15826	OK
ENSBTAG00000044004	ENSBTAG00000044004	8:102796660-102872318	0.0228585	0	0.143094	OK
ENSBTAG00000018322	ENSBTAG00000018322	7:47754926-47832191	0.0200052	0	0.0968914	OK
ENSBTAG00000000027	ENSBTAG00000000027	18:53961669-53964590	0	0	0	OK
ENSBTAG00000000030	ENSBTAG00000000030	19:45846845-45857796	0	0	0	OK
ENSBTAG00000000067	ENSBTAG00000000067	17:74104344-74106285	0	0	0	OK
ENSBTAG00000000078	ENSBTAG00000000078	8:60797958-60816816	0	0	0	OK
ENSBTAG00000000079	ENSBTAG00000000079	28:281479-297049	0	0	0	OK
ENSBTAG00000000100	ENSBTAG00000000100	4:47306856-47307501	0	0	0	OK
ENSBTAG00000000103	ENSBTAG00000000103	29:50182528-50187374	0	0	0	OK
ENSBTAG00000000106	ENSBTAG00000000106	24:4308176-4325743	0	0	0	OK
ENSBTAG00000000109	ENSBTAG00000000109	5:110939443-110956819	0	0	0	OK
ENSBTAG00000000111	ENSBTAG00000000111	11:62244353-62299938	0	0	0	OK
ENSBTAG00000000115	ENSBTAG00000000115	11:62301308-62377281	0	0	0	OK
ENSBTAG00000000118	ENSBTAG00000000118	15:47796518-47798110	0	0	0	OK
ENSBTAG00000000119	ENSBTAG00000000119	6:10223758-10224427	0	0	0	OK
ENSBTAG00000000128	ENSBTAG00000000128	20:3132654-3195021	0	0	0	OK
ENSBTAG00000000141	ENSBTAG00000000141	11:67752715-67781632	0	0	0	OK

ENSBTAG00000000153	ENSBTAG00000000153	18:46818877-46825746	0	0	0	OK
ENSBTAG00000000156	ENSBTAG00000000156	11:62812718-62817829	0	0	0	OK
ENSBTAG00000000164	ENSBTAG00000000164	8:73698975-73702769	0	0	0	OK
ENSBTAG00000000168	ENSBTAG00000000168	11:56969033-56969285	0	0	0	OK
ENSBTAG00000000171	ENSBTAG00000000171	18:23592717-23593060	0	0	0	OK
ENSBTAG00000000189	ENSBTAG00000000189	4:45806018-46233536	0	0	0	OK
ENSBTAG00000000203	ENSBTAG00000000203	8:105493586-105502576	0	0	0	OK
ENSBTAG00000000233	ENSBTAG00000000233	15:51007542-51008496	0	0	0	OK
ENSBTAG00000000236	ENSBTAG00000000236	14:65584486-65617329	0	0	0	OK
ENSBTAG00000000243	ENSBTAG00000000243	9:77215759-77222555	0	0	0	OK
ENSBTAG00000000245	ENSBTAG00000000245	9:77233381-77296724	0	0	0	OK
ENSBTAG00000000261	ENSBTAG00000000261	11:74984267-74996342	0	0	0	OK
ENSBTAG00000000271	ENSBTAG00000000271	10:53958911-54043420	0	0	0	OK
ENSBTAG00000000273	ENSBTAG00000000273	22:23308620-23340998	0	0	0	OK
ENSBTAG00000000280	ENSBTAG00000000280	5:31553987-31554917	0	0	0	OK
ENSBTAG00000000281	ENSBTAG00000000281	17:4212633-4296899	0	0	0	OK
ENSBTAG00000000285	ENSBTAG00000000285	6:64934476-64959027	0	0	0	OK
ENSBTAG00000000297	ENSBTAG00000000297	17:16835047-16854199	0	0	0	OK
ENSBTAG00000000301	ENSBTAG00000000301	2:111598715-111600550	0	0	0	OK
ENSBTAG00000000310	ENSBTAG00000000310	5:101647628-101659377	0	0	0	OK
ENSBTAG00000000341	ENSBTAG00000000341	7:26174297-26197424	0	0	0	OK
ENSBTAG00000000344	ENSBTAG00000000344	19:17572478-17575473	0	0	0	OK
ENSBTAG00000000347	ENSBTAG00000000347	15:52061682-52075146	0	0	0	OK
ENSBTAG00000000360	ENSBTAG00000000360	7:107163203-107164593	0	0	0	OK
ENSBTAG00000000379	ENSBTAG00000000379	5:89102687-89116625	0	0	0	OK
ENSBTAG00000000381	ENSBTAG00000000381	7:21889525-21890683	0	0	0	OK
ENSBTAG00000000386	ENSBTAG00000000386	19:28422864-28423634	0	0	0	OK
ENSBTAG00000000389	ENSBTAG00000000389	4:113127974-113131240	0	0	0	OK
ENSBTAG00000000393	ENSBTAG00000000393	5:84096323-84138430	0	0	0	OK
ENSBTAG00000000394	ENSBTAG00000000394	1:142875108-142980623	0	0	0	OK

ENSBTAG00000000399	ENSBTAG00000000399	7:12916683-12919313	0	0	0	OK
ENSBTAG00000000423	ENSBTAG00000000423	29:45338336-45347624	0	0	0	OK
ENSBTAG00000000425	ENSBTAG00000000425	21:55609536-55610119	0	0	0	OK
ENSBTAG00000000426	ENSBTAG00000000426	15:43633306-43633857	0	0	0	OK
ENSBTAG00000000435	ENSBTAG00000000435	5:26681521-26686845	0	0	0	OK
ENSBTAG00000000440	ENSBTAG00000000440	15:22571244-22572575	0	0	0	OK
ENSBTAG00000000483	ENSBTAG00000000483	22:50600488-50601799	0	0	0	OK
ENSBTAG00000000487	ENSBTAG00000000487	15:73389837-73390856	0	0	0	OK
ENSBTAG00000000512	ENSBTAG00000000512	13:63408785-63490256	0	0	0	OK
ENSBTAG00000000516	ENSBTAG00000000516	13:10760672-10763286	0	0	0	OK
ENSBTAG00000000539	ENSBTAG00000000539	21:7411026-7600816	0	0	0	OK
ENSBTAG00000000542	ENSBTAG00000000542	11:38066010-38095813	0	0	0	OK
ENSBTAG00000000550	ENSBTAG00000000550	7:45302251-45304424	0	0	0	OK
ENSBTAG00000000559	ENSBTAG00000000559	13:75374574-75384705	0	0	0	OK
ENSBTAG00000000560	ENSBTAG00000000560	19:13525431-13526742	0	0	0	OK
ENSBTAG00000000563	ENSBTAG00000000563	22:11646790-11651003	0	0	0	OK
ENSBTAG00000000567	ENSBTAG00000000567	X:22939074-22939650	0	0	0	OK
ENSBTAG00000000589	ENSBTAG00000000589	19:63675099-63725656	0	0	0	OK
ENSBTAG00000000598	ENSBTAG00000000598	13:42562166-42566091	0	0	0	OK
ENSBTAG00000000611	ENSBTAG00000000611	25:2885983-2887757	0	0	0	OK
ENSBTAG00000000620	ENSBTAG00000000620	14:33074313-33113841	0	0	0	OK
ENSBTAG00000000638	ENSBTAG00000000638	18:14053450-14057708	0	0	0	OK
ENSBTAG00000000639	ENSBTAG00000000639	18:14057702-14060472	0	0	0	OK
ENSBTAG00000000644	ENSBTAG00000000644	3:16891317-16894701	0	0	0	OK
ENSBTAG00000000655	ENSBTAG00000000655	21:47852814-48169316	0	0	0	OK
ENSBTAG00000000679	ENSBTAG00000000679	9:23358370-23384805	0	0	0	OK
ENSBTAG00000000707	ENSBTAG00000000707	14:9176078-9187850	0	0	0	OK
ENSBTAG00000000720	ENSBTAG00000000720	8:82365268-82371037	0	0	0	OK
ENSBTAG00000000726	ENSBTAG00000000726	11:93593229-93594171	0	0	0	OK
ENSBTAG00000000727	ENSBTAG00000000727	15:54840265-54923335	0	0	0	OK

ENSBTAG00000000735	ENSBTAG00000000735	21:26481214-26490869	0	0	0	OK
ENSBTAG00000000743	ENSBTAG00000000743	27:25448499-25457512	0	0	0	OK
ENSBTAG00000000744	ENSBTAG00000000744	27:25462499-25484164	0	0	0	OK
ENSBTAG00000000772	ENSBTAG00000000772	16:65014451-65015946	0	0	0	OK
ENSBTAG00000000783	ENSBTAG00000000783	11:13969791-13984394	0	0	0	OK
ENSBTAG00000000802	ENSBTAG00000000802	15:42678183-42692464	0	0	0	OK
ENSBTAG00000000805	ENSBTAG00000000805	22:49557991-49560687	0	0	0	OK
ENSBTAG00000000807	ENSBTAG00000000807	21:25993164-26004025	0	0	0	OK
ENSBTAG00000000808	ENSBTAG00000000808	5:29952086-29956382	0	0	0	OK
ENSBTAG00000000820	ENSBTAG00000000820	4:11074736-11079756	0	0	0	OK
ENSBTAG00000000821	ENSBTAG00000000821	10:11495133-11555270	0	0	0	OK
ENSBTAG00000000828	ENSBTAG00000000828	X:64684213-64708869	0	0	0	OK
ENSBTAG00000000833	ENSBTAG00000000833	19:24971931-24977062	0	0	0.329446	OK
ENSBTAG00000000836	ENSBTAG00000000836	5:27213749-27221177	0	0	0	OK
ENSBTAG00000000853	ENSBTAG00000000853	29:20913362-20914375	0	0	0	OK
ENSBTAG00000000856	ENSBTAG00000000856	14:1766766-1769754	0	0	0	OK
ENSBTAG00000000875	ENSBTAG00000000875	19:52110944-52124532	0	0	0	OK
ENSBTAG00000000879	ENSBTAG00000000879	14:1487365-1489409	0	0	0	OK
ENSBTAG00000000880	ENSBTAG00000000880	9:87948483-87963094	0	0	0	OK
ENSBTAG00000000893	ENSBTAG00000000893	11:103251566-103254248	0	0	0	OK
ENSBTAG00000000899	ENSBTAG00000000899	11:60934734-60942371	0	0	0	OK
ENSBTAG00000000908	ENSBTAG00000000908	17:55035079-55036087	0	0	0	OK
ENSBTAG00000000913	ENSBTAG00000000913	17:71038497-71040693	0	0	0	OK
ENSBTAG00000000924	ENSBTAG00000000924	5:26234885-26236477	0	0	0	OK
ENSBTAG00000000948	ENSBTAG00000000948	14:27864734-27937015	0	0	0	OK
ENSBTAG00000000949	ENSBTAG00000000949	28:29452334-29456540	0	0	0	OK
ENSBTAG00000000951	ENSBTAG00000000951	26:51450394-51504242	0	0	0	OK
ENSBTAG00000000954	ENSBTAG00000000954	15:62869596-62935638	0	0	0	OK
ENSBTAG00000000960	ENSBTAG00000000960	13:75363561-75364594	0	0	0	OK
ENSBTAG00000000966	ENSBTAG00000000966	5:100112514-100118909	0	0	0	OK

ENSBTAG00000000972	ENSBTAG00000000972	1:146076329-146077070	0	0	0	OK
ENSBTAG00000000982	ENSBTAG00000000982	22:51418819-51426814	0	0	0	OK
ENSBTAG00000000991	ENSBTAG00000000991	4:78816511-78821610	0	0	0	OK
ENSBTAG00000000992	ENSBTAG00000000992	11:67187148-67193866	0	0	0	OK
ENSBTAG00000000997	ENSBTAG00000000997	X:28241960-28242450	0	0	0	OK
ENSBTAG00000001005	ENSBTAG00000001005	17:73002287-73010751	0	0	0	OK
ENSBTAG00000001007	ENSBTAG00000001007	X:3646866-3687086	0	0	0	OK
ENSBTAG00000001038	ENSBTAG00000001038	13:53134612-53143629	0	0	0	OK
ENSBTAG00000001043	ENSBTAG00000001043	29:20259768-20557376	0	0	0	OK
ENSBTAG00000001062	ENSBTAG00000001062	18:56336879-56337677	0	0	0	OK
ENSBTAG00000001090	ENSBTAG00000001090	2:26666109-26690420	0	0	0	OK
ENSBTAG00000001097	ENSBTAG00000001097	2:18413883-18421599	0	0	0	OK
ENSBTAG00000001098	ENSBTAG00000001098	2:18382407-18410532	0	0	0	OK
ENSBTAG00000001107	ENSBTAG00000001107	16:57694738-57705495	0	0	0	OK
ENSBTAG00000001109	ENSBTAG00000001109	16:57711733-57731147	0	0	0	OK
ENSBTAG00000001113	ENSBTAG00000001113	11:19683130-19699850	0	0	0	OK
ENSBTAG00000001123	ENSBTAG00000001123	23:47647767-47663909	0	0	0	OK
ENSBTAG00000001131	ENSBTAG00000001131	25:36326327-36335481	0	0	0	OK
ENSBTAG00000001144	ENSBTAG00000001144	13:39883239-39901761	0	0	0	OK
ENSBTAG00000001152	ENSBTAG00000001152	7:22019473-22023366	0	0	0	OK
ENSBTAG00000001156	ENSBTAG00000001156	14:8970644-8985785	0	0	0	OK
ENSBTAG00000001176	ENSBTAG00000001176	22:22576209-22578360	0	0	0	OK
ENSBTAG00000001210	ENSBTAG00000001210	16:56095250-56098973	0	0	0	OK
ENSBTAG00000001213	ENSBTAG00000001213	21:71548490-71550674	0	0	0	OK
ENSBTAG00000001221	ENSBTAG00000001221	8:71663194-71666501	0	0	0	OK
ENSBTAG00000001242	ENSBTAG00000001242	26:34805080-34806484	0	0	0	OK
ENSBTAG00000001247	ENSBTAG00000001247	7:48987177-48998964	0	0	0	OK
ENSBTAG00000001289	ENSBTAG00000001289	9:97493687-97499407	0	0	0	OK
ENSBTAG00000001302	ENSBTAG00000001302	14:45279167-45491347	0	0	0	OK
ENSBTAG00000001310	ENSBTAG00000001310	22:56867886-56872817	0	0	0	OK

ENSBTAG00000001344	ENSBTAG00000001344	12:83099333-83115415	0	0	0	OK
ENSBTAG00000001348	ENSBTAG00000001348	6:67746951-67761215	0	0	0	OK
ENSBTAG00000001350	ENSBTAG00000001350	18:50343554-50344106	0	0	0	OK
ENSBTAG00000001359	ENSBTAG00000001359	2:119989627-119990869	0	0	0	OK
ENSBTAG00000001360	ENSBTAG00000001360	9:71974859-71978200	0	0	0	OK
ENSBTAG00000001361	ENSBTAG00000001361	16:44457294-44462940	0	0	0	OK
ENSBTAG00000001365	ENSBTAG00000001365	12:34540960-34548854	0	0	0	OK
ENSBTAG00000001367	ENSBTAG00000001367	7:74035697-74057799	0	0	0	OK
ENSBTAG00000001376	ENSBTAG00000001376	13:60407803-60446557	0	0	0	OK
ENSBTAG00000001387	ENSBTAG00000001387	21:22728672-22740461	0	0	0	OK
ENSBTAG00000001414	ENSBTAG00000001414	12:52351170-52352151	0	0	0	OK
ENSBTAG00000001416	ENSBTAG00000001416	1:41870256-41915644	0	0	0	OK
ENSBTAG00000001420	ENSBTAG00000001420	13:43187468-43240717	0	0	0	OK
ENSBTAG00000001422	ENSBTAG00000001422	9:19000363-19035860	0	0	0	OK
ENSBTAG00000001423	ENSBTAG00000001423	10:45166944-45169879	0	0	0	OK
ENSBTAG00000001429	ENSBTAG00000001429	20:4337738-4356155	0	0	0	OK
ENSBTAG00000001442	ENSBTAG00000001442	1:67036690-67046927	0	0	0	OK
ENSBTAG00000001446	ENSBTAG00000001446	9:77787554-77802592	0	0	0	OK
ENSBTAG00000001447	ENSBTAG00000001447	22:55929987-55931463	0	0	0	OK
ENSBTAG00000001451	ENSBTAG00000001451	1:132063621-132068263	0	0	0	OK
ENSBTAG00000001455	ENSBTAG00000001455	4:69340737-69342484	0	0	0	OK
ENSBTAG00000001475	ENSBTAG00000001475	23:42547987-42567105	0	0	0	OK
ENSBTAG00000001476	ENSBTAG00000001476	23:27715022-27718202	0	0	0	OK
ENSBTAG00000001477	ENSBTAG00000001477	23:28298188-28298628	0	0	0	OK
ENSBTAG00000001478	ENSBTAG00000001478	16:33290069-33331173	0	0	0	OK
ENSBTAG00000001502	ENSBTAG00000001502	4:5369373-5370370	0	0	0	OK
ENSBTAG00000001511	ENSBTAG00000001511	1:80179481-80202388	0	0	0	OK
ENSBTAG00000001514	ENSBTAG00000001514	X:135436137-135465103	0	0	0	OK
ENSBTAG00000001516	ENSBTAG00000001516	15:38336936-38340230	0	0	0	OK
ENSBTAG00000001519	ENSBTAG00000001519	3:14146762-14153732	0	0	0	OK

ENSBTAG00000001526	ENSBTAG00000001526	21:20502553-20503402	0	0	0	OK
ENSBTAG00000001543	ENSBTAG00000001543	17:53067558-53088676	0	0	0	OK
ENSBTAG00000001568	ENSBTAG00000001568	7:32114444-32128245	0	0	0	OK
ENSBTAG00000001605	ENSBTAG00000001605	10:92624409-92632934	0	0	0	OK
ENSBTAG00000001609	ENSBTAG00000001609	19:61708768-61814973	0	0	0	OK
ENSBTAG00000001635	ENSBTAG00000001635	23:7534223-7535613	0	0	0	OK
ENSBTAG00000001668	ENSBTAG00000001668	22:58809371-58873170	0	0	0	OK
ENSBTAG00000001681	ENSBTAG00000001681	8:62646416-62647032	0	0	0	OK
ENSBTAG00000001686	ENSBTAG00000001686	3:3425058-3446915	0	0	0	OK
ENSBTAG00000001695	ENSBTAG00000001695	15:15672402-15672869	0	0	0	OK
ENSBTAG00000001710	ENSBTAG00000001710	22:6789384-6885301	0	0	0	OK
ENSBTAG00000001730	ENSBTAG00000001730	11:107150454-107154002	0	0	0	OK
ENSBTAG00000001745	ENSBTAG00000001745	5:21037442-21044658	0	0	0	OK
ENSBTAG00000001747	ENSBTAG00000001747	9:81967632-81999683	0	0	0	OK
ENSBTAG00000001752	ENSBTAG00000001752	9:82007664-82027058	0	0	0	OK
ENSBTAG00000001761	ENSBTAG00000001761	7:42532984-42533975	0	0	0	OK
ENSBTAG00000001782	ENSBTAG00000001782	18:48905797-48907691	0	0	0	OK
ENSBTAG00000001792	ENSBTAG00000001792	7:19841556-19843106	0	0	0	OK
ENSBTAG00000001801	ENSBTAG00000001801	24:12950390-12960186	0	0	0	OK
ENSBTAG00000001806	ENSBTAG00000001806	17:63474992-63497850	0	0	0	OK
ENSBTAG00000001808	ENSBTAG00000001808	14:36438471-36470764	0	0	0	OK
ENSBTAG00000001816	ENSBTAG00000001816	7:4052317-4062831	0	0	0	OK
ENSBTAG00000001825	ENSBTAG00000001825	19:39229248-39230376	0	0	0	OK
ENSBTAG00000001836	ENSBTAG00000001836	19:19937601-19947884	0	0	0	OK
ENSBTAG00000001857	ENSBTAG00000001857	18:25547364-25555198	0	0	0.162398	OK
ENSBTAG00000001858	ENSBTAG00000001858	5:70910365-70911285	0	0	0	OK
ENSBTAG00000001862	ENSBTAG00000001862	7:59782767-60099412	0	0	0	OK
ENSBTAG00000001880	ENSBTAG00000001880	X:20126975-20143499	0	0	0	OK
ENSBTAG00000001885	ENSBTAG00000001885	19:18604658-18609323	0	0	0	OK
ENSBTAG00000001886	ENSBTAG00000001886	X:18407390-18436265	0	0	0	OK

ENSBTAG00000001901	ENSBTAG00000001901	7:43580137-43581051	0	0	0	OK
ENSBTAG00000001915	ENSBTAG00000001915	19:63230260-63243064	0	0	0	OK
ENSBTAG00000001919	ENSBTAG00000001919	19:37919481-37932791	0	0	0	OK
ENSBTAG00000001922	ENSBTAG00000001922	15:44260211-44267703	0	0	0	OK
ENSBTAG00000001925	ENSBTAG00000001925	6:85255405-85318173	0	0	0	OK
ENSBTAG00000001926	ENSBTAG00000001926	8:98681863-98726100	0	0	0	OK
ENSBTAG00000001932	ENSBTAG00000001932	11:99576793-99589668	0	0	0	OK
ENSBTAG00000001938	ENSBTAG00000001938	8:90300716-90305611	0	0	0	OK
ENSBTAG00000001957	ENSBTAG00000001957	9:25578681-25640399	0	0	0	OK
ENSBTAG00000001976	ENSBTAG00000001976	4:65155643-65175535	0	0	0	OK
ENSBTAG00000001985	ENSBTAG00000001985	13:54591298-54597058	0	0	0	OK
ENSBTAG00000002000	ENSBTAG00000002000	15:22720232-22722218	0	0	0	OK
ENSBTAG00000002001	ENSBTAG00000002001	X:72300237-72316894	0	0	0	OK
ENSBTAG00000002034	ENSBTAG00000002034	11:75084558-75097310	0	0	0	OK
ENSBTAG00000002046	ENSBTAG00000002046	11:107244219-107246853	0	0	0	OK
ENSBTAG00000002065	ENSBTAG00000002065	13:59903159-59912715	0	0	0	OK
ENSBTAG00000002069	ENSBTAG00000002069	23:28502523-28506312	0	0	0	OK
ENSBTAG00000002088	ENSBTAG00000002088	11:46575177-46584984	0	0	0	OK
ENSBTAG00000002090	ENSBTAG00000002090	25:37500101-37512370	0	0	0	OK
ENSBTAG00000002094	ENSBTAG00000002094	25:37494181-37499276	0	0	0	OK
ENSBTAG00000002112	ENSBTAG00000002112	2:70121999-70142602	0	0	0	OK
ENSBTAG00000002121	ENSBTAG00000002121	29:46033673-46035477	0	0	0	OK
ENSBTAG00000002122	ENSBTAG00000002122	17:57352624-57353599	0	0	0	OK
ENSBTAG00000002137	ENSBTAG00000002137	12:11131392-11172149	0	0	0	OK
ENSBTAG00000002140	ENSBTAG00000002140	7:23709466-23711664	0	0	0	OK
ENSBTAG00000002143	ENSBTAG00000002143	7:36197352-36269226	0	0	0	OK
ENSBTAG00000002150	ENSBTAG00000002150	23:24346987-24350509	0	0	0	OK
ENSBTAG00000002181	ENSBTAG00000002181	14:7440522-7582740	0	0	0	OK
ENSBTAG00000002182	ENSBTAG00000002182	1:65024152-65031968	0	0	0	OK
ENSBTAG00000002188	ENSBTAG00000002188	25:4088164-4102526	0	0	0	OK

ENSBTAG00000002194	ENSBTAG00000002194	10:52673545-52771273	0	0	0	OK
ENSBTAG00000002196	ENSBTAG00000002196	14:39328604-39348337	0	0	0	OK
ENSBTAG00000002256	ENSBTAG00000002256	5:44318356-44342854	0	0	0	OK
ENSBTAG00000002275	ENSBTAG00000002275	9:512138-516069	0	0	0	OK
ENSBTAG00000002283	ENSBTAG00000002283	2:90987007-90989214	0	0	0	OK
ENSBTAG00000002284	ENSBTAG00000002284	7:18508982-18510041	0	0	0	OK
ENSBTAG00000002300	ENSBTAG00000002300	12:69502940-69544658	0	0	0	OK
ENSBTAG00000002316	ENSBTAG00000002316	2:916139-963744	0	0	0	OK
ENSBTAG00000002326	ENSBTAG00000002326	10:67843327-67861113	0	0	0	OK
ENSBTAG00000002332	ENSBTAG00000002332	4:64411818-64415413	0	0	0	OK
ENSBTAG00000002344	ENSBTAG00000002344	25:27343999-27349045	0	0	0	OK
ENSBTAG00000002354	ENSBTAG00000002354	10:4360165-4362131	0	0	0	OK
ENSBTAG00000002355	ENSBTAG00000002355	5:29102540-29120314	0	0	0	OK
ENSBTAG00000002378	ENSBTAG00000002378	18:55939559-55955606	0	0	0	OK
ENSBTAG00000002385	ENSBTAG00000002385	17:71733168-71739085	0	0	0	OK
ENSBTAG00000002401	ENSBTAG00000002401	5:26679273-26680063	0	0	0	OK
ENSBTAG00000002413	ENSBTAG00000002413	5:114576426-114588012	0	0	0	OK
ENSBTAG00000002423	ENSBTAG00000002423	19:50114344-50134716	0	0	0	OK
ENSBTAG00000002432	ENSBTAG00000002432	12:87630496-87631510	0	0	0	OK
ENSBTAG00000002441	ENSBTAG00000002441	19:44437198-44437928	0	0	0	OK
ENSBTAG00000002446	ENSBTAG00000002446	13:43298762-43299144	0	0	0	OK
ENSBTAG00000002447	ENSBTAG00000002447	7:6428154-6441115	0	0	0	OK
ENSBTAG00000002462	ENSBTAG00000002462	1:88111911-88129274	0	0	0	OK
ENSBTAG00000002463	ENSBTAG00000002463	1:88096666-88111780	0	0	0	OK
ENSBTAG00000002467	ENSBTAG00000002467	25:618417-619923	0	0	0	OK
ENSBTAG00000002468	ENSBTAG00000002468	7:18198991-18200124	0	0	0	OK
ENSBTAG00000002469	ENSBTAG00000002469	7:18200290-18210847	0	0	0	OK
ENSBTAG00000002470	ENSBTAG00000002470	25:620321-623768	0	0	0	OK
ENSBTAG00000002474	ENSBTAG00000002474	25:41535262-41778055	0	0	0	OK
ENSBTAG00000002480	ENSBTAG00000002480	25:41522213-41527685	0	0	0	OK

ENSBTAG00000002483	ENSBTAG00000002483	16:51585627-51587187	0	0	0	OK
ENSBTAG00000002484	ENSBTAG00000002484	25:627455-633977	0	0	0	OK
ENSBTAG00000002492	ENSBTAG00000002492	5:65881779-65888886	0	0	0	OK
ENSBTAG00000002497	ENSBTAG00000002497	23:45093667-45111322	0	0	0	OK
ENSBTAG00000002501	ENSBTAG00000002501	26:22919817-22921523	0	0	0	OK
ENSBTAG00000002508	ENSBTAG00000002508	29:41722353-41724967	0	0	0.435909	OK
ENSBTAG00000002516	ENSBTAG00000002516	7:12929337-12933843	0	0	0	OK
ENSBTAG00000002524	ENSBTAG00000002524	7:12934834-12941251	0	0	0	OK
ENSBTAG00000002525	ENSBTAG00000002525	7:12942937-12948365	0	0	0	OK
ENSBTAG00000002534	ENSBTAG00000002534	23:47219513-47235324	0	0	0	OK
ENSBTAG00000002555	ENSBTAG00000002555	3:99641130-99646599	0	0	0	OK
ENSBTAG00000002557	ENSBTAG00000002557	11:43885453-43896122	0	0	0	OK
ENSBTAG00000002559	ENSBTAG00000002559	5:120002246-120004065	0	0	0	OK
ENSBTAG00000002565	ENSBTAG00000002565	X:92253729-92254047	0	0	0	OK
ENSBTAG00000002574	ENSBTAG00000002574	6:7251252-7289661	0	0	0	OK
ENSBTAG00000002599	ENSBTAG00000002599	17:18709902-18721278	0	0	0	OK
ENSBTAG00000002609	ENSBTAG00000002609	19:21960824-21973891	0	0	0	OK
ENSBTAG00000002612	ENSBTAG00000002612	21:55385792-55395266	0	0	0	OK
ENSBTAG00000002614	ENSBTAG00000002614	28:24917631-24955300	0	0	0	OK
ENSBTAG00000002616	ENSBTAG00000002616	21:55487969-55488586	0	0	0	OK
ENSBTAG00000002623	ENSBTAG00000002623	1:22123654-22181827	0	0	0	OK
ENSBTAG00000002627	ENSBTAG00000002627	3:74448728-74464316	0	0	0	OK
ENSBTAG00000002633	ENSBTAG00000002633	19:55118591-55153750	0	0	0	OK
ENSBTAG00000002634	ENSBTAG00000002634	22:50559653-50564391	0	0	0	OK
ENSBTAG00000002639	ENSBTAG00000002639	18:11116412-11133102	0	0	0	OK
ENSBTAG00000002641	ENSBTAG00000002641	23:30236773-30241973	0	0	0	OK
ENSBTAG00000002646	ENSBTAG00000002646	2:24807356-24860776	0	0	0	OK
ENSBTAG00000002673	ENSBTAG00000002673	5:28197780-28199954	0	0	0	OK
ENSBTAG00000002674	ENSBTAG00000002674	4:11060375-11067420	0	0	0	OK
ENSBTAG00000002675	ENSBTAG00000002675	13:20534889-20581215	0	0	0	OK

ENSBTAG00000002708	ENSBTAG00000002708	5:23426160-23456891	0	0	0	OK
ENSBTAG00000002712	ENSBTAG00000002712	10:20780357-20781857	0	0	0	OK
ENSBTAG00000002724	ENSBTAG00000002724	3:20059125-20078990	0	0	0	OK
ENSBTAG00000002735	ENSBTAG00000002735	25:543805-545583	0	0	0	OK
ENSBTAG00000002739	ENSBTAG00000002739	4:64985736-65050206	0	0	0	OK
ENSBTAG00000002741	ENSBTAG00000002741	5:44886445-44886802	0	0	0	OK
ENSBTAG00000002746	ENSBTAG00000002746	8:51440386-51501432	0	0	0	OK
ENSBTAG00000002751	ENSBTAG00000002751	29:978635-979365	0	0	0	OK
ENSBTAG00000002773	ENSBTAG00000002773	19:57328130-57334739	0	0	0	OK
ENSBTAG00000002788	ENSBTAG00000002788	24:54544870-54715264	0	0	0	OK
ENSBTAG00000002808	ENSBTAG00000002808	10:70797327-70822594	0	0	0	OK
ENSBTAG00000002809	ENSBTAG00000002809	3:120332022-120339480	0	0	0	OK
ENSBTAG00000002856	ENSBTAG00000002856	13:29882065-29898674	0	0	0	OK
ENSBTAG00000002882	ENSBTAG00000002882	7:28414755-28465352	0	0	0	OK
ENSBTAG00000002894	ENSBTAG00000002894	16:41689562-41709076	0	0	0	OK
ENSBTAG00000002895	ENSBTAG00000002895	6:32136546-32138712	0	0	0	OK
ENSBTAG00000002904	ENSBTAG00000002904	18:63687552-63695160	0	0	0	OK
ENSBTAG00000002917	ENSBTAG00000002917	4:114885500-114925565	0	0	0	OK
ENSBTAG00000002919	ENSBTAG00000002919	23:14959071-14965828	0	0	0	OK
ENSBTAG00000002928	ENSBTAG00000002928	6:87608793-87621025	0	0	0	OK
ENSBTAG00000002931	ENSBTAG00000002931	5:43995904-44048333	0	0	0	OK
ENSBTAG00000002936	ENSBTAG00000002936	11:100080557-100132328	0	0	0	OK
ENSBTAG00000002942	ENSBTAG00000002942	13:76091991-76103853	0	0	0	OK
ENSBTAG00000002951	ENSBTAG00000002951	3:8794792-8831787	0	0	0	OK
ENSBTAG00000002955	ENSBTAG00000002955	28:13166932-13179478	0	0	0	OK
ENSBTAG00000002983	ENSBTAG00000002983	3:106993388-107008708	0	0	0	OK
ENSBTAG00000003000	ENSBTAG00000003000	15:52518371-52520710	0	0	0	OK
ENSBTAG00000003006	ENSBTAG00000003006	10:26574076-26574619	0	0	0	OK
ENSBTAG00000003019	ENSBTAG00000003019	4:114773448-114779653	0	0	0	OK
ENSBTAG00000003022	ENSBTAG00000003022	22:52143305-52145266	0	0	0	OK

ENSBTAG00000003023	ENSBTAG00000003023	15:1138764-1139715	0	0	0	OK
ENSBTAG00000003043	ENSBTAG00000003043	10:44711723-44842190	0	0	0	OK
ENSBTAG00000003073	ENSBTAG00000003073	29:43032946-43044726	0	0	0	OK
ENSBTAG00000003081	ENSBTAG00000003081	27:13404608-13412552	0	0	0	OK
ENSBTAG00000003086	ENSBTAG00000003086	7:33638715-33639158	0	0	0	OK
ENSBTAG00000003096	ENSBTAG00000003096	3:11068657-11069608	0	0	0	OK
ENSBTAG00000003103	ENSBTAG00000003103	7:39467951-39470752	0	0	0	OK
ENSBTAG00000003109	ENSBTAG00000003109	12:18114552-18139506	0	0	0	OK
ENSBTAG00000003112	ENSBTAG00000003112	22:17264152-17283523	0	0	0	OK
ENSBTAG00000003114	ENSBTAG00000003114	4:64353893-64387651	0	0	0	OK
ENSBTAG00000003118	ENSBTAG00000003118	7:98896054-98896522	0	0	0	OK
ENSBTAG00000003134	ENSBTAG00000003134	1:144887618-144890448	0	0	0	OK
ENSBTAG00000003135	ENSBTAG00000003135	1:109080710-109102233	0	0	0	OK
ENSBTAG00000003155	ENSBTAG00000003155	21:59346452-59348581	0	0	0	OK
ENSBTAG00000003164	ENSBTAG00000003164	2:28400754-28401735	0	0	0	OK
ENSBTAG00000003179	ENSBTAG00000003179	1:80221897-80225631	0	0	0	OK
ENSBTAG00000003196	ENSBTAG00000003196	26:9365643-9402403	0	0	0	OK
ENSBTAG00000003197	ENSBTAG00000003197	5:102100647-102101142	0	0	0	OK
ENSBTAG00000003220	ENSBTAG00000003220	3:10647426-10648940	0	0	0	OK
ENSBTAG00000003228	ENSBTAG00000003228	5:111261437-111267705	0	0	0	OK
ENSBTAG00000003231	ENSBTAG00000003231	18:57819482-57826637	0	0	0	OK
ENSBTAG00000003236	ENSBTAG00000003236	29:39889073-39918516	0	0	0	OK
ENSBTAG00000003240	ENSBTAG00000003240	8:101433013-101438257	0	0	0	OK
ENSBTAG00000003255	ENSBTAG00000003255	5:103995315-104017746	0	0	0	OK
ENSBTAG00000003258	ENSBTAG00000003258	7:90900124-90900797	0	0	0	OK
ENSBTAG00000003263	ENSBTAG00000003263	10:8737159-8822375	0	0	0	OK
ENSBTAG00000003264	ENSBTAG00000003264	18:56998859-57005507	0	0	0	OK
ENSBTAG00000003289	ENSBTAG00000003289	19:14702489-14703961	0	0	0	OK
ENSBTAG00000003295	ENSBTAG00000003295	16:38902322-38924937	0	0	0	OK
ENSBTAG00000003301	ENSBTAG00000003301	1:14791089-15026555	0	0	0	OK

ENSBTAG00000003305	ENSBTAG00000003305	25:33267457-33282335	0	0	0	OK
ENSBTAG00000003315	ENSBTAG00000003315	12:35840399-35841483	0	0	0	OK
ENSBTAG00000003352	ENSBTAG00000003352	23:25423550-25424597	0	0	0	OK
ENSBTAG00000003364	ENSBTAG00000003364	13:61523658-61533444	0	0	0	OK
ENSBTAG00000003369	ENSBTAG00000003369	29:45655688-45676864	0	0	0	OK
ENSBTAG00000003383	ENSBTAG00000003383	1:70697539-70698145	0	0	0	OK
ENSBTAG00000003387	ENSBTAG00000003387	11:98601202-98609975	0	0	0	OK
ENSBTAG00000003388	ENSBTAG00000003388	11:98612579-98615633	0	0	0	OK
ENSBTAG00000003389	ENSBTAG00000003389	3:120451717-120488810	0	0	0	OK
ENSBTAG00000003484	ENSBTAG00000003484	3:93583351-93590342	0	0	0	OK
ENSBTAG00000003493	ENSBTAG00000003493	10:353755-361515	0	0	0	OK
ENSBTAG00000003497	ENSBTAG00000003497	4:104416410-104417026	0	0	0	OK
ENSBTAG00000003500	ENSBTAG00000003500	19:48516497-48543240	0	0	0	OK
ENSBTAG00000003503	ENSBTAG00000003503	18:15186659-15219204	0	0	0	OK
ENSBTAG00000003505	ENSBTAG00000003505	5:21080012-21119087	0	0	0	OK
ENSBTAG00000003509	ENSBTAG00000003509	27:33747817-33776271	0	0	0	OK
ENSBTAG00000003535	ENSBTAG00000003535	3:15517157-15517733	0	0	0	OK
ENSBTAG00000003545	ENSBTAG00000003545	29:1056626-1062580	0	0	0	OK
ENSBTAG00000003548	ENSBTAG00000003548	29:46087141-46090005	0	0	0	OK
ENSBTAG00000003553	ENSBTAG00000003553	11:25585492-25587785	0	0	0	OK
ENSBTAG00000003560	ENSBTAG00000003560	12:18173184-18173958	0	0	0	OK
ENSBTAG00000003561	ENSBTAG00000003561	13:53482006-53494820	0	0	0	OK
ENSBTAG00000003574	ENSBTAG00000003574	5:115683471-115697601	0	0	0	OK
ENSBTAG00000003575	ENSBTAG00000003575	23:30186450-30193866	0	0	0	OK
ENSBTAG00000003582	ENSBTAG00000003582	15:48844272-48847388	0	0	0	OK
ENSBTAG00000003589	ENSBTAG00000003589	13:23741426-23743812	0	0	0	OK
ENSBTAG00000003624	ENSBTAG00000003624	15:83291642-83293465	0	0	0	OK
ENSBTAG00000003633	ENSBTAG00000003633	4:106358114-106362513	0	0	0	OK
ENSBTAG00000003642	ENSBTAG00000003642	3:101152891-101166725	0	0	0	OK
ENSBTAG00000003652	ENSBTAG00000003652	6:107477961-107493240	0	0	0	OK

ENSBTAG00000003661	ENSBTAG00000003661	15:17642141-17655900	0	0	0	OK
ENSBTAG00000003664	ENSBTAG00000003664	4:106050917-106051862	0	0	0	OK
ENSBTAG00000003691	ENSBTAG00000003691	11:86183810-86191260	0	0	0	OK
ENSBTAG00000003701	ENSBTAG00000003701	16:72494426-72531856	0	0	0	OK
ENSBTAG00000003707	ENSBTAG00000003707	19:34796321-34817309	0	0	0	OK
ENSBTAG00000003712	ENSBTAG00000003712	29:44420405-44422038	0	0	0	OK
ENSBTAG00000003731	ENSBTAG00000003731	2:90214944-90219273	0	0	0	OK
ENSBTAG00000003733	ENSBTAG00000003733	19:27210647-27216974	0	0	0	OK
ENSBTAG00000003760	ENSBTAG00000003760	29:30021530-30022049	0	0	0	OK
ENSBTAG00000003764	ENSBTAG00000003764	9:63127338-63150180	0	0	0	OK
ENSBTAG00000003771	ENSBTAG00000003771	2:71283903-71285350	0	0	0	OK
ENSBTAG00000003802	ENSBTAG00000003802	19:33418199-33451244	0	0	0	OK
ENSBTAG00000003832	ENSBTAG00000003832	2:136187692-136192151	0	0	0	OK
ENSBTAG00000003850	ENSBTAG00000003850	3:78846969-78852272	0	0	0	OK
ENSBTAG00000003857	ENSBTAG00000003857	10:81751603-81800132	0	0	0	OK
ENSBTAG00000003872	ENSBTAG00000003872	13:58237975-58260726	0	0	0	OK
ENSBTAG00000003882	ENSBTAG00000003882	22:16833932-16848259	0	0	0	OK
ENSBTAG00000003893	ENSBTAG00000003893	X:110433907-110444196	0	0	0	OK
ENSBTAG00000003895	ENSBTAG00000003895	18:13931106-13938075	0	0	0	OK
ENSBTAG00000003912	ENSBTAG00000003912	4:46669594-46670063	0	0	0	OK
ENSBTAG00000003914	ENSBTAG00000003914	7:5611161-5615225	0	0	0	OK
ENSBTAG00000003920	ENSBTAG00000003920	10:20735502-20750260	0	0	0	OK
ENSBTAG00000003923	ENSBTAG00000003923	2:107011784-107014997	0	0	0	OK
ENSBTAG00000003930	ENSBTAG00000003930	15:905559-907503	0	0	0	OK
ENSBTAG00000003937	ENSBTAG00000003937	13:22813810-22814335	0	0	0	OK
ENSBTAG00000003942	ENSBTAG00000003942	5:56190024-56197400	0	0	0	OK
ENSBTAG00000003952	ENSBTAG00000003952	22:7645492-7720326	0	0	0	OK
ENSBTAG00000003956	ENSBTAG00000003956	19:37113476-37122446	0	0	0	OK
ENSBTAG00000003957	ENSBTAG00000003957	21:29762147-29766253	0	0	0	OK
ENSBTAG00000003958	ENSBTAG00000003958	19:30304817-30322098	0	0	0	OK

ENSBTAG00000003962	ENSBTAG00000003962	13:63690852-63692393	0	0	0	OK
ENSBTAG00000003986	ENSBTAG00000003986	7:52505096-52513059	0	0	0	OK
ENSBTAG00000003987	ENSBTAG00000003987	7:53427445-53436775	0	0	0	OK
ENSBTAG00000003994	ENSBTAG00000003994	4:76705104-76712709	0	0	0	OK
ENSBTAG00000004046	ENSBTAG00000004046	15:83630776-83663175	0	0	0	OK
ENSBTAG00000004050	ENSBTAG00000004050	28:32381530-32382170	0	0	0	OK
ENSBTAG00000004054	ENSBTAG00000004054	21:62980456-63032069	0	0	0	OK
ENSBTAG00000004061	ENSBTAG00000004061	1:44755393-44770677	0	0	0	OK
ENSBTAG00000004073	ENSBTAG00000004073	11:25016565-25173982	0	0	0	OK
ENSBTAG00000004075	ENSBTAG00000004075	13:46682709-46685834	0	0	0	OK
ENSBTAG00000004082	ENSBTAG00000004082	6:71053201-71053458	0	0	0	OK
ENSBTAG00000004093	ENSBTAG00000004093	23:50354842-50357631	0	0	0	OK
ENSBTAG00000004095	ENSBTAG00000004095	11:105856511-105860113	0	0	0	OK
ENSBTAG00000004106	ENSBTAG00000004106	25:35147771-35173861	0	0	0	OK
ENSBTAG00000004112	ENSBTAG00000004112	7:45433858-45436865	0	0	0	OK
ENSBTAG00000004118	ENSBTAG00000004118	22:49242705-49256766	0	0	0	OK
ENSBTAG00000004120	ENSBTAG00000004120	22:50348516-50359203	0	0	0	OK
ENSBTAG00000004134	ENSBTAG00000004134	8:8437129-8468012	0	0	0	OK
ENSBTAG00000004148	ENSBTAG00000004148	13:74337276-74339570	0	0	0	OK
ENSBTAG00000004151	ENSBTAG00000004151	X:102292853-102293918	0	0	0	OK
ENSBTAG00000004153	ENSBTAG00000004153	7:61104005-61108298	0	0	0	OK
ENSBTAG00000004159	ENSBTAG00000004159	11:27260474-27263699	0	0	0	OK
ENSBTAG00000004173	ENSBTAG00000004173	27:26008822-26026228	0	0	0	OK
ENSBTAG00000004191	ENSBTAG00000004191	7:3381931-3387142	0	0	0	OK
ENSBTAG00000004197	ENSBTAG00000004197	17:55418581-55419667	0	0	0	OK
ENSBTAG00000004199	ENSBTAG00000004199	17:55400570-55418222	0	0	0	OK
ENSBTAG00000004203	ENSBTAG00000004203	17:55365285-55394855	0	0	0	OK
ENSBTAG00000004206	ENSBTAG00000004206	15:81672292-81677418	0	0	0	OK
ENSBTAG00000004212	ENSBTAG00000004212	29:48876964-48914063	0	0	0	OK
ENSBTAG00000004215	ENSBTAG00000004215	4:113564406-113567496	0	0	0	OK

ENSBTAG00000004217	ENSBTAG00000004217	28:2840519-2841803	0	0	0	OK
ENSBTAG00000004230	ENSBTAG00000004230	6:20111149-20138597	0	0	0	OK
ENSBTAG00000004261	ENSBTAG00000004261	6:109224626-109228237	0	0	0	OK
ENSBTAG00000004267	ENSBTAG00000004267	2:38004586-38112887	0	0	0	OK
ENSBTAG00000004268	ENSBTAG00000004268	7:17750386-17752622	0	0	0	OK
ENSBTAG00000004272	ENSBTAG00000004272	21:59310737-59322006	0	0	0	OK
ENSBTAG00000004273	ENSBTAG00000004273	13:42951020-42960765	0	0	0	OK
ENSBTAG00000004287	ENSBTAG00000004287	6:105171715-105275204	0	0	0	OK
ENSBTAG00000004313	ENSBTAG00000004313	2:20854098-20856159	0	0	0	OK
ENSBTAG00000004318	ENSBTAG00000004318	26:23563110-23761992	0	0	0	OK
ENSBTAG00000004321	ENSBTAG00000004321	26:23593340-23611728	0	0	0	OK
ENSBTAG00000004329	ENSBTAG00000004329	6:58599881-58660258	0	0	0	OK
ENSBTAG00000004348	ENSBTAG00000004348	29:46913942-46923884	0	0	0	OK
ENSBTAG00000004364	ENSBTAG00000004364	11:47736287-47754240	0	0	0	OK
ENSBTAG00000004369	ENSBTAG00000004369	7:15043600-15045442	0	0	0	OK
ENSBTAG00000004378	ENSBTAG00000004378	5:57333495-57335062	0	0	0	OK
ENSBTAG00000004381	ENSBTAG00000004381	3:88032647-88033607	0	0	0	OK
ENSBTAG00000004394	ENSBTAG00000004394	9:28880109-29001337	0	0	0	OK
ENSBTAG00000004398	ENSBTAG00000004398	4:57127736-58083528	0	0	0	OK
ENSBTAG00000004402	ENSBTAG00000004402	13:57855966-57864630	0	0	0	OK
ENSBTAG00000004405	ENSBTAG00000004405	4:88570538-88642925	0	0	0	OK
ENSBTAG00000004406	ENSBTAG00000004406	19:8651762-8720657	0	0	0	OK
ENSBTAG00000004412	ENSBTAG00000004412	6:85473853-85512994	0	0	0	OK
ENSBTAG00000004414	ENSBTAG00000004414	16:24125198-24137830	0	0	0	OK
ENSBTAG00000004415	ENSBTAG00000004415	7:51683086-51684304	0	0	0	OK
ENSBTAG00000004418	ENSBTAG00000004418	2:133373262-133376607	0	0	0	OK
ENSBTAG00000004422	ENSBTAG00000004422	3:48600669-48699217	0	0	0	OK
ENSBTAG00000004429	ENSBTAG00000004429	13:65340131-65343889	0	0	0	OK
ENSBTAG00000004437	ENSBTAG00000004437	28:42618237-42618617	0	0	0	OK
ENSBTAG00000004443	ENSBTAG00000004443	15:46852253-46853210	0	0	0	OK

ENSBTAG00000004459	ENSBTAG00000004459	1:45281218-45394335	0	0	0	OK
ENSBTAG00000004468	ENSBTAG00000004468	8:86686387-86689386	0	0	0	OK
ENSBTAG00000004472	ENSBTAG00000004472	9:96486513-96497234	0	0	0	OK
ENSBTAG00000004475	ENSBTAG00000004475	19:18027468-18028392	0	0	0	OK
ENSBTAG00000004512	ENSBTAG00000004512	22:57210749-57216906	0	0	0	OK
ENSBTAG00000004515	ENSBTAG00000004515	28:6492388-6559855	0	0	0	OK
ENSBTAG00000004521	ENSBTAG00000004521	7:22533095-22537819	0	0	0	OK
ENSBTAG00000004526	ENSBTAG00000004526	18:2084511-2126855	0	0	0	OK
ENSBTAG00000004530	ENSBTAG00000004530	1:3805673-3806442	0	0	0	OK
ENSBTAG00000004532	ENSBTAG00000004532	5:83260873-83278970	0	0	0	OK
ENSBTAG00000004541	ENSBTAG00000004541	5:104163581-104173568	0	0	0	OK
ENSBTAG00000004544	ENSBTAG00000004544	11:4414188-4424370	0	0	0	OK
ENSBTAG00000004565	ENSBTAG00000004565	11:77493036-77493712	0	0	0	OK
ENSBTAG00000004567	ENSBTAG00000004567	11:66471948-66490577	0	0	0	OK
ENSBTAG00000004569	ENSBTAG00000004569	5:4824768-4866969	0	0	0	OK
ENSBTAG00000004574	ENSBTAG00000004574	6:12092954-12201473	0	0	0	OK
ENSBTAG00000004578	ENSBTAG00000004578	8:69964099-69970068	0	0	0	OK
ENSBTAG00000004583	ENSBTAG00000004583	15:84584460-84611997	0	0	0	OK
ENSBTAG00000004591	ENSBTAG00000004591	3:44372995-44473523	0	0	0	OK
ENSBTAG00000004594	ENSBTAG00000004594	15:6390159-6400322	0	0	0	OK
ENSBTAG00000004599	ENSBTAG00000004599	1:57178553-57192433	0	0	0	OK
ENSBTAG00000004601	ENSBTAG00000004601	1:57156325-57169463	0	0	0	OK
ENSBTAG00000004604	ENSBTAG00000004604	7:5157900-5168539	0	0	0	OK
ENSBTAG00000004632	ENSBTAG00000004632	19:51499065-51528822	0	0	0.235828	OK
ENSBTAG00000004651	ENSBTAG00000004651	19:36223569-36225626	0	0	0	OK
ENSBTAG00000004657	ENSBTAG00000004657	7:82834995-82835982	0	0	0	OK
ENSBTAG00000004664	ENSBTAG00000004664	11:98931356-98945011	0	0	0	OK
ENSBTAG00000004667	ENSBTAG00000004667	11:93900518-93901442	0	0	0	OK
ENSBTAG00000004692	ENSBTAG00000004692	10:38912007-38912352	0	0	0	OK
ENSBTAG00000004702	ENSBTAG00000004702	28:11270629-11272802	0	0	0	OK

ENSBTAG00000004714	ENSBTAG00000004714	7:17746039-17748352	0	0	0	OK
ENSBTAG00000004716	ENSBTAG00000004716	7:17737515-17738903	0	0	0	OK
ENSBTAG00000004743	ENSBTAG00000004743	17:29475157-29475634	0	0	0	OK
ENSBTAG00000004750	ENSBTAG00000004750	13:67460323-67498847	0	0	0	OK
ENSBTAG00000004765	ENSBTAG00000004765	15:50306182-50307139	0	0	0	OK
ENSBTAG00000004771	ENSBTAG00000004771	15:44237475-44237790	0	0	0	OK
ENSBTAG00000004776	ENSBTAG00000004776	3:10029473-10030882	0	0	0	OK
ENSBTAG00000004782	ENSBTAG00000004782	29:45972076-45974235	0	0	0	OK
ENSBTAG00000004785	ENSBTAG00000004785	29:45988624-45992117	0	0	0	OK
ENSBTAG00000004787	ENSBTAG00000004787	19:50243828-50258586	0	0	0	OK
ENSBTAG00000004793	ENSBTAG00000004793	6:87694411-87705727	0	0	0	OK
ENSBTAG00000004797	ENSBTAG00000004797	6:106552948-106577901	0	0	0	OK
ENSBTAG00000004805	ENSBTAG00000004805	18:15625554-15928962	0	0	0	OK
ENSBTAG00000004821	ENSBTAG00000004821	10:27490261-27491179	0	0	0	OK
ENSBTAG00000004823	ENSBTAG00000004823	11:48529513-48535995	0	0	0	OK
ENSBTAG00000004844	ENSBTAG00000004844	18:25334587-25339259	0	0	0	OK
ENSBTAG00000004848	ENSBTAG00000004848	11:95780658-95805530	0	0	0	OK
ENSBTAG00000004854	ENSBTAG00000004854	10:62467610-62489654	0	0	0	OK
ENSBTAG00000004890	ENSBTAG00000004890	9:34421935-34422367	0	0	0	OK
ENSBTAG00000004905	ENSBTAG00000004905	19:42366659-42370493	0	0	0	OK
ENSBTAG00000004906	ENSBTAG00000004906	14:71808696-71818065	0	0	0	OK
ENSBTAG00000004920	ENSBTAG00000004920	20:24442439-24442887	0	0	0	OK
ENSBTAG00000004925	ENSBTAG00000004925	18:60249392-60304930	0	0	0	OK
ENSBTAG00000004926	ENSBTAG00000004926	10:42685929-42696776	0	0	0	OK
ENSBTAG00000004929	ENSBTAG00000004929	20:32633115-32650605	0	0	0	OK
ENSBTAG00000004937	ENSBTAG00000004937	22:60217807-60229341	0	0	0	OK
ENSBTAG00000004943	ENSBTAG00000004943	6:3413085-3419194	0	0	0	OK
ENSBTAG00000004953	ENSBTAG00000004953	16:3208945-3219936	0	0	0	OK
ENSBTAG00000004958	ENSBTAG00000004958	4:119522031-119565916	0	0	0	OK
ENSBTAG00000004993	ENSBTAG00000004993	13:42398734-42399402	0	0	0	OK

ENSBTAG00000004996	ENSBTAG00000004996	8:100213152-100219346	0	0	0	OK
ENSBTAG00000005007	ENSBTAG00000005007	26:10251023-10251836	0	0	0	OK
ENSBTAG00000005031	ENSBTAG00000005031	25:26513087-26518561	0	0	0.197951	OK
ENSBTAG00000005041	ENSBTAG00000005041	11:72920570-72971981	0	0	0	OK
ENSBTAG00000005042	ENSBTAG00000005042	X:91223899-91228056	0	0	0	OK
ENSBTAG00000005045	ENSBTAG00000005045	19:38202538-38207784	0	0	0	OK
ENSBTAG00000005048	ENSBTAG00000005048	19:29581009-29594334	0	0	0	OK
ENSBTAG00000005051	ENSBTAG00000005051	19:29597618-29605540	0	0	0	OK
ENSBTAG00000005052	ENSBTAG00000005052	19:29608714-29648238	0	0	0	OK
ENSBTAG00000005057	ENSBTAG00000005057	26:38923062-38945878	0	0	0	OK
ENSBTAG00000005064	ENSBTAG00000005064	3:24105658-24200029	0	0	0	OK
ENSBTAG00000005073	ENSBTAG00000005073	29:43986565-43987804	0	0	0	OK
ENSBTAG00000005101	ENSBTAG00000005101	1:28411203-28411543	0	0	0	OK
ENSBTAG00000005128	ENSBTAG00000005128	17:73201459-73202019	0	0	0	OK
ENSBTAG00000005140	ENSBTAG00000005140	2:133632741-133634622	0	0	0	OK
ENSBTAG00000005141	ENSBTAG00000005141	10:68778346-68974093	0	0	0	OK
ENSBTAG00000005142	ENSBTAG00000005142	20:33667204-33669805	0	0	0	OK
ENSBTAG00000005148	ENSBTAG00000005148	25:1159756-1163098	0	0	0	OK
ENSBTAG00000005151	ENSBTAG00000005151	11:61759949-61969748	0	0	0	OK
ENSBTAG00000005152	ENSBTAG00000005152	18:34368484-34393020	0	0	0	OK
ENSBTAG00000005156	ENSBTAG00000005156	15:22701524-22707582	0	0	0	OK
ENSBTAG00000005165	ENSBTAG00000005165	28:13651047-13678154	0	0	0	OK
ENSBTAG00000005168	ENSBTAG00000005168	3:120676093-120714918	0	0	0	OK
ENSBTAG00000005176	ENSBTAG00000005176	20:36635642-36656528	0	0	0	OK
ENSBTAG00000005182	ENSBTAG00000005182	23:27647068-27652297	0	0	0	OK
ENSBTAG00000005183	ENSBTAG00000005183	17:65863711-65882658	0	0	0	OK
ENSBTAG00000005189	ENSBTAG00000005189	11:102950014-102961170	0	0	0	OK
ENSBTAG00000005202	ENSBTAG00000005202	13:39015340-39016050	0	0	0	OK
ENSBTAG00000005203	ENSBTAG00000005203	1:112777478-112802942	0	0	0	OK
ENSBTAG00000005217	ENSBTAG00000005217	10:61556467-61557103	0	0	0	OK

ENSBTAG00000005218	ENSBTAG00000005218	15:38466099-38626862	0	0	0	OK
ENSBTAG00000005222	ENSBTAG00000005222	8:60400366-60409620	0	0	0	OK
ENSBTAG00000005230	ENSBTAG00000005230	1:110277781-110286193	0	0	0	OK
ENSBTAG00000005239	ENSBTAG00000005239	2:100306470-100307061	0	0	0	OK
ENSBTAG00000005244	ENSBTAG00000005244	12:32842603-32844825	0	0	0	OK
ENSBTAG00000005262	ENSBTAG00000005262	17:277494-277802	0	0	0	OK
ENSBTAG00000005263	ENSBTAG00000005263	17:325196-483093	0	0	0	OK
ENSBTAG00000005265	ENSBTAG00000005265	7:83599488-83607997	0	0	0	OK
ENSBTAG00000005299	ENSBTAG00000005299	12:53310603-53407024	0	0	0	OK
ENSBTAG00000005306	ENSBTAG00000005306	22:56530331-56533155	0	0	0	OK
ENSBTAG00000005324	ENSBTAG00000005324	24:62485473-62489683	0	0	0	OK
ENSBTAG00000005330	ENSBTAG00000005330	18:46342487-46345919	0	0	0	OK
ENSBTAG00000005333	ENSBTAG00000005333	5:74170470-74181260	0	0	0	OK
ENSBTAG00000005340	ENSBTAG00000005340	15:35019669-35267343	0	0	0	OK
ENSBTAG00000005344	ENSBTAG00000005344	10:11332414-11344167	0	0	0	OK
ENSBTAG00000005352	ENSBTAG00000005352	19:21145240-21152189	0	0	0	OK
ENSBTAG00000005390	ENSBTAG00000005390	18:49309014-49313926	0	0	0	OK
ENSBTAG00000005395	ENSBTAG00000005395	10:83185490-83187781	0	0	0	OK
ENSBTAG00000005396	ENSBTAG00000005396	8:7983900-8000085	0	0	0	OK
ENSBTAG00000005400	ENSBTAG00000005400	9:44166444-44204874	0	0	0	OK
ENSBTAG00000005408	ENSBTAG00000005408	2:89920585-89927819	0	0	0	OK
ENSBTAG00000005414	ENSBTAG00000005414	1:51022815-51023423	0	0	0	OK
ENSBTAG00000005416	ENSBTAG00000005416	2:89936348-89943891	0	0	0	OK
ENSBTAG00000005431	ENSBTAG00000005431	22:17961210-18018234	0	0	0	OK
ENSBTAG00000005437	ENSBTAG00000005437	3:17907654-17912860	0	0	0	OK
ENSBTAG00000005445	ENSBTAG00000005445	3:78601769-78644543	0	0	0	OK
ENSBTAG00000005446	ENSBTAG00000005446	25:35091299-35095676	0	0	0	OK
ENSBTAG00000005449	ENSBTAG00000005449	9:50599571-50623262	0	0	0	OK
ENSBTAG00000005450	ENSBTAG00000005450	25:37189785-37242576	0	0	0	OK
ENSBTAG00000005470	ENSBTAG00000005470	8:70429461-70480013	0	0	0	OK

ENSBTAG00000005482	ENSBTAG00000005482	29:8298777-8370800	0	0	0	OK
ENSBTAG00000005490	ENSBTAG00000005490	22:51556259-51569916	0	0	0	OK
ENSBTAG00000005495	ENSBTAG00000005495	8:77028521-77200434	0	0	0	OK
ENSBTAG00000005499	ENSBTAG00000005499	4:5727482-5820313	0	0	0	OK
ENSBTAG00000005504	ENSBTAG00000005504	13:64624465-64626041	0	0	0	OK
ENSBTAG00000005522	ENSBTAG00000005522	1:122821127-122842538	0	0	0	OK
ENSBTAG00000005524	ENSBTAG00000005524	4:48545065-48575834	0	0	0	OK
ENSBTAG00000005525	ENSBTAG00000005525	11:93069013-93090242	0	0	0	OK
ENSBTAG00000005527	ENSBTAG00000005527	18:53668985-53677248	0	0	0	OK
ENSBTAG00000005540	ENSBTAG00000005540	1:264297-264843	0	0	0	OK
ENSBTAG00000005546	ENSBTAG00000005546	19:38518636-38521061	0	0	0	OK
ENSBTAG00000005559	ENSBTAG00000005559	14:39523633-39524177	0	0	0	OK
ENSBTAG00000005564	ENSBTAG00000005564	10:14878654-15004134	0	0	0	OK
ENSBTAG00000005573	ENSBTAG00000005573	13:65744268-65745100	0	0	0	OK
ENSBTAG00000005576	ENSBTAG00000005576	18:57259805-57262108	0	0	0	OK
ENSBTAG00000005577	ENSBTAG00000005577	29:44552308-44553484	0	0	0	OK
ENSBTAG00000005584	ENSBTAG00000005584	2:119050743-119051409	0	0	0	OK
ENSBTAG00000005588	ENSBTAG00000005588	23:27206811-27209706	0	0	0.411099	OK
ENSBTAG00000005596	ENSBTAG00000005596	2:105338357-105367369	0	0	0	OK
ENSBTAG00000005600	ENSBTAG00000005600	25:39119524-39120499	0	0	0	OK
ENSBTAG00000005603	ENSBTAG00000005603	6:92645056-92646326	0	0	0	OK
ENSBTAG00000005606	ENSBTAG00000005606	5:26188848-26191440	0	0	0	OK
ENSBTAG00000005617	ENSBTAG00000005617	22:61189668-61201147	0	0	0	OK
ENSBTAG00000005622	ENSBTAG00000005622	25:10288890-10325021	0	0	0	OK
ENSBTAG00000005624	ENSBTAG00000005624	15:79740976-79741885	0	0	0	OK
ENSBTAG00000005634	ENSBTAG00000005634	23:27339520-27344476	0	0	0	OK
ENSBTAG00000005648	ENSBTAG00000005648	17:11830251-11831926	0	0	0	OK
ENSBTAG00000005652	ENSBTAG00000005652	1:83540705-83546032	0	0	0	OK
ENSBTAG00000005654	ENSBTAG00000005654	11:49933203-49934214	0	0	0	OK
ENSBTAG00000005657	ENSBTAG00000005657	1:83531112-83533268	0	0	0	OK

ENSBTAG00000005668	ENSBTAG00000005668	6:23816192-23898886	0	0	0	OK
ENSBTAG00000005688	ENSBTAG00000005688	11:106447319-106450536	0	0	0	OK
ENSBTAG00000005695	ENSBTAG00000005695	17:35146243-35205444	0	0	0.250371	OK
ENSBTAG00000005711	ENSBTAG00000005711	6:106483715-107356158	0	0	0	OK
ENSBTAG00000005715	ENSBTAG00000005715	26:25872736-25875735	0	0	0	OK
ENSBTAG00000005717	ENSBTAG00000005717	3:111524191-111525013	0	0	0	OK
ENSBTAG00000005722	ENSBTAG00000005722	16:52997829-52998556	0	0	0	OK
ENSBTAG00000005725	ENSBTAG00000005725	27:34707900-34774452	0	0	0	OK
ENSBTAG00000005742	ENSBTAG00000005742	1:9242709-9250037	0	0	0	OK
ENSBTAG00000005743	ENSBTAG00000005743	20:10034941-10062660	0	0	0	OK
ENSBTAG00000005756	ENSBTAG00000005756	29:45223677-45229753	0	0	0	OK
ENSBTAG00000005759	ENSBTAG00000005759	2:133289294-133295334	0	0	0	OK
ENSBTAG00000005787	ENSBTAG00000005787	1:53295476-53368940	0	0	0	OK
ENSBTAG00000005791	ENSBTAG00000005791	26:45635843-45660024	0	0	0	OK
ENSBTAG00000005798	ENSBTAG00000005798	25:34320558-34327971	0	0	0	OK
ENSBTAG00000005826	ENSBTAG00000005826	8:46138548-46180420	0	0	0	OK
ENSBTAG00000005827	ENSBTAG00000005827	9:40873046-41046003	0	0	0	OK
ENSBTAG00000005833	ENSBTAG00000005833	5:87967759-88017062	0	0	0	OK
ENSBTAG00000005843	ENSBTAG00000005843	15:25293298-25304051	0	0	0	OK
ENSBTAG00000005846	ENSBTAG00000005846	21:34181384-34185382	0	0	0	OK
ENSBTAG00000005864	ENSBTAG00000005864	3:69195015-69212777	0	0	0	OK
ENSBTAG00000005874	ENSBTAG00000005874	15:79958957-79959914	0	0	0	OK
ENSBTAG00000005885	ENSBTAG00000005885	23:30585769-30586705	0	0	0	OK
ENSBTAG00000005895	ENSBTAG00000005895	11:47210062-47210732	0	0	0	OK
ENSBTAG00000005907	ENSBTAG00000005907	8:11372937-11389199	0	0	0	OK
ENSBTAG00000005917	ENSBTAG00000005917	24:582651-659851	0	0	0	OK
ENSBTAG00000005941	ENSBTAG00000005941	10:24062421-24062915	0	0	0	OK
ENSBTAG00000005954	ENSBTAG00000005954	10:104224180-104269841	0	0	0	OK
ENSBTAG00000005955	ENSBTAG00000005955	9:96765676-96782003	0	0	0	OK
ENSBTAG00000005965	ENSBTAG00000005965	26:50854862-50882327	0	0	0	OK

ENSBTAG00000005975	ENSBTAG00000005975	23:15000259-15005748	0	0	0	OK
ENSBTAG00000005985	ENSBTAG00000005985	24:5614220-5621035	0	0	0	OK
ENSBTAG00000005986	ENSBTAG00000005986	2:4722618-4723281	0	0	0	OK
ENSBTAG00000005989	ENSBTAG00000005989	6:38574589-38600027	0	0	0	OK
ENSBTAG00000005998	ENSBTAG00000005998	17:18892762-18910926	0	0	0	OK
ENSBTAG00000006022	ENSBTAG00000006022	4:113528364-113533520	0	0	0	OK
ENSBTAG00000006024	ENSBTAG00000006024	21:34903757-34906887	0	0	0	OK
ENSBTAG00000006025	ENSBTAG00000006025	11:59767827-59790133	0	0	0	OK
ENSBTAG00000006033	ENSBTAG00000006033	X:4068879-4072037	0	0	0	OK
ENSBTAG00000006042	ENSBTAG00000006042	28:43803995-43807271	0	0	0	OK
ENSBTAG00000006046	ENSBTAG00000006046	27:15623108-15623317	0	0	0	OK
ENSBTAG00000006048	ENSBTAG00000006048	19:45348334-45367549	0	0	0	OK
ENSBTAG00000006056	ENSBTAG00000006056	19:45444315-45446228	0	0	0	OK
ENSBTAG00000006063	ENSBTAG00000006063	13:47779509-47787910	0	0	0	OK
ENSBTAG00000006069	ENSBTAG00000006069	7:19691005-19692540	0	0	0	OK
ENSBTAG00000006077	ENSBTAG00000006077	7:13826162-13828718	0	0	0	OK
ENSBTAG00000006081	ENSBTAG00000006081	3:30761232-30766807	0	0	0	OK
ENSBTAG00000006083	ENSBTAG00000006083	7:13785086-13789391	0	0	0	OK
ENSBTAG00000006088	ENSBTAG00000006088	19:58659932-58678995	0	0	0	OK
ENSBTAG00000006093	ENSBTAG00000006093	15:3699420-3700944	0	0	0	OK
ENSBTAG00000006104	ENSBTAG00000006104	5:113255671-113264464	0	0	0	OK
ENSBTAG00000006110	ENSBTAG00000006110	7:96682483-96694175	0	0	0	OK
ENSBTAG00000006128	ENSBTAG00000006128	3:101791332-101791992	0	0	0	OK
ENSBTAG00000006133	ENSBTAG00000006133	7:104576794-104577753	0	0	0	OK
ENSBTAG00000006150	ENSBTAG00000006150	11:9121669-9159584	0	0	0	OK
ENSBTAG00000006156	ENSBTAG00000006156	6:115687876-115716694	0	0	0	OK
ENSBTAG00000006157	ENSBTAG00000006157	2:4111870-4149372	0	0	0	OK
ENSBTAG00000006159	ENSBTAG00000006159	2:41361136-41491255	0	0	0	OK
ENSBTAG00000006179	ENSBTAG00000006179	22:60039909-60040605	0	0	0	OK
ENSBTAG00000006187	ENSBTAG00000006187	19:34685356-34687892	0	0	0	OK

ENSBTAG00000006194	ENSBTAG00000006194	29:44673878-44679325	0	0	0	OK
ENSBTAG00000006218	ENSBTAG00000006218	26:47537543-47539155	0	0	0	OK
ENSBTAG00000006232	ENSBTAG00000006232	4:114730381-114755250	0	0	0	OK
ENSBTAG00000006251	ENSBTAG00000006251	7:13927959-13928266	0	0	0	OK
ENSBTAG00000006262	ENSBTAG00000006262	2:4780175-4819192	0	0	0	OK
ENSBTAG00000006264	ENSBTAG00000006264	25:18124562-18143260	0	0	0	OK
ENSBTAG00000006267	ENSBTAG00000006267	10:63632193-63633045	0	0	0	OK
ENSBTAG00000006268	ENSBTAG00000006268	X:40501901-40519262	0	0	0	OK
ENSBTAG00000006293	ENSBTAG00000006293	X:18201669-18238357	0	0	0	OK
ENSBTAG00000006294	ENSBTAG00000006294	2:35003727-35003992	0	0	0	OK
ENSBTAG00000006305	ENSBTAG00000006305	11:98552023-98561576	0	0	0	OK
ENSBTAG00000006307	ENSBTAG00000006307	6:104006455-104056137	0	0	0	OK
ENSBTAG00000006338	ENSBTAG00000006338	3:106787760-106791865	0	0	0	OK
ENSBTAG00000006339	ENSBTAG00000006339	23:29403152-29403978	0	0	0	OK
ENSBTAG00000006341	ENSBTAG00000006341	10:70145553-70186590	0	0	0	OK
ENSBTAG00000006342	ENSBTAG00000006342	2:125727884-125730128	0	0	0	OK
ENSBTAG00000006343	ENSBTAG00000006343	11:6721484-6757777	0	0	0	OK
ENSBTAG00000006353	ENSBTAG00000006353	18:39163672-39171071	0	0	0	OK
ENSBTAG00000006363	ENSBTAG00000006363	21:52763448-52764730	0	0	0	OK
ENSBTAG00000006371	ENSBTAG00000006371	7:40583482-40600676	0	0	0	OK
ENSBTAG00000006388	ENSBTAG00000006388	1:71710367-71721053	0	0	0	OK
ENSBTAG00000006391	ENSBTAG00000006391	25:21498278-21509168	0	0	0	OK
ENSBTAG00000006397	ENSBTAG00000006397	19:29352213-29352959	0	0	0	OK
ENSBTAG00000006398	ENSBTAG00000006398	9:89692606-89692771	0	0	0	OK
ENSBTAG00000006403	ENSBTAG00000006403	16:24876775-24886962	0	0	0	OK
ENSBTAG00000006409	ENSBTAG00000006409	1:98357199-98369907	0	0	0	OK
ENSBTAG00000006414	ENSBTAG00000006414	18:35476832-35491550	0	0	0	OK
ENSBTAG00000006421	ENSBTAG00000006421	15:81133287-81134238	0	0	0	OK
ENSBTAG00000006424	ENSBTAG00000006424	18:62717574-62721350	0	0	0	OK
ENSBTAG00000006428	ENSBTAG00000006428	7:41154754-41179540	0	0	0	OK

ENSBTAG00000006431	ENSBTAG00000006431	28:26063127-26063775	0	0	0	OK
ENSBTAG00000006438	ENSBTAG00000006438	2:135418585-135422028	0	0	0	OK
ENSBTAG00000006441	ENSBTAG00000006441	3:32003755-32020661	0	0	0	OK
ENSBTAG00000006443	ENSBTAG00000006443	15:76098493-76144104	0	0	0	OK
ENSBTAG00000006445	ENSBTAG00000006445	26:10474367-10478110	0	0	0	OK
ENSBTAG00000006457	ENSBTAG00000006457	25:27801695-27805181	0	0	0	OK
ENSBTAG00000006474	ENSBTAG00000006474	11:29626086-29636759	0	0	0	OK
ENSBTAG00000006487	ENSBTAG00000006487	18:63381415-63388728	0	0	0	OK
ENSBTAG00000006488	ENSBTAG00000006488	7:8255704-8256652	0	0	0	OK
ENSBTAG00000006497	ENSBTAG00000006497	11:68276293-68277306	0	0	0	OK
ENSBTAG00000006514	ENSBTAG00000006514	10:25547526-25548213	0	0	0	OK
ENSBTAG00000006523	ENSBTAG00000006523	9:97399158-97404522	0	0	0	OK
ENSBTAG00000006529	ENSBTAG00000006529	13:18182706-18224123	0	0	0	OK
ENSBTAG00000006530	ENSBTAG00000006530	5:82260538-82260790	0	0	0	OK
ENSBTAG00000006539	ENSBTAG00000006539	13:60226027-60232034	0	0	0	OK
ENSBTAG00000006557	ENSBTAG00000006557	5:30918837-30922282	0	0	0	OK
ENSBTAG00000006558	ENSBTAG00000006558	11:103346205-103350967	0	0	0	OK
ENSBTAG00000006568	ENSBTAG00000006568	X:60197182-60213594	0	0	0	OK
ENSBTAG00000006570	ENSBTAG00000006570	22:15327003-15345250	0	0	0	OK
ENSBTAG00000006572	ENSBTAG00000006572	11:103876014-103883072	0	0	0	OK
ENSBTAG00000006574	ENSBTAG00000006574	16:70894433-70900751	0	0	0	OK
ENSBTAG00000006578	ENSBTAG00000006578	13:51880157-51883728	0	0	0	OK
ENSBTAG00000006600	ENSBTAG00000006600	3:72477170-72478766	0	0	0	OK
ENSBTAG00000006606	ENSBTAG00000006606	19:22835875-22841764	0	0	0	OK
ENSBTAG00000006629	ENSBTAG00000006629	29:28095910-28096888	0	0	0	OK
ENSBTAG00000006644	ENSBTAG00000006644	15:54998957-55022311	0	0	0	OK
ENSBTAG00000006646	ENSBTAG00000006646	18:56534832-56545053	0	0	0	OK
ENSBTAG00000006647	ENSBTAG00000006647	12:18319334-18349305	0	0	0	OK
ENSBTAG00000006648	ENSBTAG00000006648	21:1746232-1751043	0	0	0	OK
ENSBTAG00000006651	ENSBTAG00000006651	8:39603072-39614295	0	0	0	OK

ENSBTAG00000006654	ENSBTAG00000006654	2:35247177-35342975	0	0	0	OK
ENSBTAG00000006655	ENSBTAG00000006655	4:107308520-107309079	0	0	0	OK
ENSBTAG00000006656	ENSBTAG00000006656	17:75036893-75038127	0	0	0	OK
ENSBTAG00000006715	ENSBTAG00000006715	8:6410066-6449539	0	0	0	OK
ENSBTAG00000006721	ENSBTAG00000006721	4:28446102-28456952	0	0	0	OK
ENSBTAG00000006724	ENSBTAG00000006724	1:72708756-72726620	0	0	0	OK
ENSBTAG00000006727	ENSBTAG00000006727	22:25460260-25461074	0	0	0	OK
ENSBTAG00000006742	ENSBTAG00000006742	13:41248124-41257312	0	0	0	OK
ENSBTAG00000006754	ENSBTAG00000006754	18:55722335-55729578	0	0	0	OK
ENSBTAG00000006760	ENSBTAG00000006760	11:61479109-61482379	0	0	0	OK
ENSBTAG00000006761	ENSBTAG00000006761	23:35059435-35072284	0	0	0	OK
ENSBTAG00000006767	ENSBTAG00000006767	14:84125769-84167593	0	0	0	OK
ENSBTAG00000006777	ENSBTAG00000006777	19:20707030-20709465	0	0	0	OK
ENSBTAG00000006807	ENSBTAG00000006807	19:42313514-42317810	0	0	0	OK
ENSBTAG00000006813	ENSBTAG00000006813	1:77092287-77092759	0	0	0	OK
ENSBTAG00000006839	ENSBTAG00000006839	18:57092301-57096458	0	0	0	OK
ENSBTAG00000006841	ENSBTAG00000006841	18:57103009-57127435	0	0	0	OK
ENSBTAG00000006843	ENSBTAG00000006843	20:7973679-7992443	0	0	0	OK
ENSBTAG00000006852	ENSBTAG00000006852	11:36683362-36862401	0	0	0	OK
ENSBTAG00000006853	ENSBTAG00000006853	8:16750579-16751227	0	0	0	OK
ENSBTAG00000006861	ENSBTAG00000006861	11:36887204-36913561	0	0	0	OK
ENSBTAG00000006924	ENSBTAG00000006924	4:17882059-17883051	0	0	0	OK
ENSBTAG00000006954	ENSBTAG00000006954	18:58151317-58157607	0	0	0	OK
ENSBTAG00000006963	ENSBTAG00000006963	11:98191879-98195943	0	0	0	OK
ENSBTAG00000006965	ENSBTAG00000006965	18:63645888-63660919	0	0	0	OK
ENSBTAG00000006972	ENSBTAG00000006972	20:71496993-71511149	0	0	0	OK
ENSBTAG00000006973	ENSBTAG00000006973	5:116124286-116144383	0	0	0	OK
ENSBTAG00000006978	ENSBTAG00000006978	7:35662598-35763653	0	0	0	OK
ENSBTAG00000006984	ENSBTAG00000006984	16:5105576-5124229	0	0	0	OK
ENSBTAG00000006986	ENSBTAG00000006986	6:26695824-26714361	0	0	0	OK

ENSBTAG00000007003	ENSBTAG00000007003	24:35935175-35945792	0	0	0	OK
ENSBTAG00000007009	ENSBTAG00000007009	21:68751571-68752167	0	0	0	OK
ENSBTAG00000007024	ENSBTAG00000007024	5:104077162-104077952	0	0	0	OK
ENSBTAG00000007044	ENSBTAG00000007044	26:24284482-24287960	0	0	0	OK
ENSBTAG00000007051	ENSBTAG00000007051	27:23856776-23857658	0	0	0	OK
ENSBTAG00000007063	ENSBTAG00000007063	29:43774444-43775105	0	0	0	OK
ENSBTAG00000007077	ENSBTAG00000007077	11:72497318-72504279	0	0	0.31806	OK
ENSBTAG00000007083	ENSBTAG00000007083	19:45525636-45538595	0	0	0	OK
ENSBTAG00000007100	ENSBTAG00000007100	1:146785259-146840277	0	0	0	OK
ENSBTAG00000007114	ENSBTAG00000007114	10:7013634-7044633	0	0	0	OK
ENSBTAG00000007135	ENSBTAG00000007135	11:56528810-56531769	0	0	0	OK
ENSBTAG00000007140	ENSBTAG00000007140	23:13102246-13103226	0	0	0	OK
ENSBTAG00000007146	ENSBTAG00000007146	4:67482870-67604560	0	0	0	OK
ENSBTAG00000007175	ENSBTAG00000007175	5:50592289-50595766	0	0	0	OK
ENSBTAG00000007177	ENSBTAG00000007177	25:9330474-9359612	0	0	0	OK
ENSBTAG00000007189	ENSBTAG00000007189	6:92061507-92076962	0	0	0.241325	OK
ENSBTAG00000007191	ENSBTAG00000007191	19:14823469-14831157	0	0	0	OK
ENSBTAG00000007195	ENSBTAG00000007195	3:104603592-104605588	0	0	0	OK
ENSBTAG00000007200	ENSBTAG00000007200	18:62573343-62574837	0	0	0	OK
ENSBTAG00000007231	ENSBTAG00000007231	7:97475074-97501919	0	0	0	OK
ENSBTAG00000007233	ENSBTAG00000007233	21:66917779-67066000	0	0	0	OK
ENSBTAG00000007238	ENSBTAG00000007238	19:19950583-19952293	0	0	0	OK
ENSBTAG00000007239	ENSBTAG00000007239	2:44850891-44867293	0	0	0	OK
ENSBTAG00000007248	ENSBTAG00000007248	26:50680586-50681525	0	0	0	OK
ENSBTAG00000007275	ENSBTAG00000007275	23:15090106-15094736	0	0	0	OK
ENSBTAG00000007280	ENSBTAG00000007280	2:119029184-119030313	0	0	0	OK
ENSBTAG00000007286	ENSBTAG00000007286	23:31645470-31645860	0	0	0	OK
ENSBTAG00000007295	ENSBTAG00000007295	25:10702095-10703471	0	0	0	OK
ENSBTAG00000007296	ENSBTAG00000007296	1:82549641-82550013	0	0	0	OK
ENSBTAG00000007297	ENSBTAG00000007297	3:11692421-11693378	0	0	0	OK

ENSBTAG00000007312	ENSBTAG00000007312	7:17811221-17814125	0	0	0	OK
ENSBTAG00000007321	ENSBTAG00000007321	20:13393478-13436297	0	0	0	OK
ENSBTAG00000007324	ENSBTAG00000007324	15:71243844-71245767	0	0	0	OK
ENSBTAG00000007325	ENSBTAG00000007325	25:994571-996276	0	0	0	OK
ENSBTAG00000007329	ENSBTAG00000007329	12:19111393-19147324	0	0	0	OK
ENSBTAG00000007332	ENSBTAG00000007332	15:29521979-29530109	0	0	0	OK
ENSBTAG00000007336	ENSBTAG00000007336	18:46778206-46779500	0	0	0	OK
ENSBTAG00000007338	ENSBTAG00000007338	18:46779847-46782546	0	0	0	OK
ENSBTAG00000007340	ENSBTAG00000007340	27:5024116-5031818	0	0	0	OK
ENSBTAG00000007346	ENSBTAG00000007346	21:33872673-33878166	0	0	0	OK
ENSBTAG00000007347	ENSBTAG00000007347	24:57125860-57134072	0	0	0	OK
ENSBTAG00000007350	ENSBTAG00000007350	7:54001449-54004030	0	0	0	OK
ENSBTAG00000007351	ENSBTAG00000007351	21:55198613-55199386	0	0	0	OK
ENSBTAG00000007354	ENSBTAG00000007354	19:50009721-50010311	0	0	0	OK
ENSBTAG00000007361	ENSBTAG00000007361	29:43704112-43708218	0	0	0	OK
ENSBTAG00000007363	ENSBTAG00000007363	22:58693673-58701742	0	0	0	OK
ENSBTAG00000007371	ENSBTAG00000007371	10:9369309-9520700	0	0	0	OK
ENSBTAG00000007378	ENSBTAG00000007378	11:70656464-70733268	0	0	0	OK
ENSBTAG00000007385	ENSBTAG00000007385	10:28548977-28564464	0	0	0	OK
ENSBTAG00000007387	ENSBTAG00000007387	14:57239786-57249241	0	0	0	OK
ENSBTAG00000007389	ENSBTAG00000007389	19:43673502-43687259	0	0	0	OK
ENSBTAG00000007440	ENSBTAG00000007440	8:39050747-39061810	0	0	0	OK
ENSBTAG00000007461	ENSBTAG00000007461	20:31324012-31350848	0	0	0	OK
ENSBTAG00000007473	ENSBTAG00000007473	27:4766513-4783646	0	0	0	OK
ENSBTAG00000007482	ENSBTAG00000007482	7:3274169-3274557	0	0	0	OK
ENSBTAG00000007488	ENSBTAG00000007488	18:36008029-36029234	0	0	0	OK
ENSBTAG00000007516	ENSBTAG00000007516	10:104175767-104176772	0	0	0	OK
ENSBTAG00000007518	ENSBTAG00000007518	10:104182288-104218056	0	0	0	OK
ENSBTAG00000007532	ENSBTAG00000007532	23:28600595-28610740	0	0	0	OK
ENSBTAG00000007546	ENSBTAG00000007546	5:110565205-110567190	0	0	0	OK

ENSBTAG00000007547	ENSBTAG00000007547	19:63738187-63745798	0	0	0	OK
ENSBTAG00000007568	ENSBTAG00000007568	6:38279965-38291339	0	0	0	OK
ENSBTAG00000007572	ENSBTAG00000007572	4:93640141-93642824	0	0	0	OK
ENSBTAG00000007586	ENSBTAG00000007586	10:82347062-82386494	0	0	0	OK
ENSBTAG00000007595	ENSBTAG00000007595	16:26908536-26927548	0	0	0	OK
ENSBTAG00000007608	ENSBTAG00000007608	4:56366856-56442208	0	0	0	OK
ENSBTAG00000007611	ENSBTAG00000007611	5:107418041-107424743	0	0	0	OK
ENSBTAG00000007616	ENSBTAG00000007616	18:36611037-36616277	0	0	0	OK
ENSBTAG00000007622	ENSBTAG00000007622	29:50352063-50361497	0	0	0	OK
ENSBTAG00000007629	ENSBTAG00000007629	27:37234388-37240538	0	0	0	OK
ENSBTAG00000007646	ENSBTAG00000007646	X:135421260-135430737	0	0	0	OK
ENSBTAG00000007654	ENSBTAG00000007654	X:90149332-90230185	0	0	0	OK
ENSBTAG00000007660	ENSBTAG00000007660	7:18343921-18347588	0	0	0	OK
ENSBTAG00000007678	ENSBTAG00000007678	13:37127925-37198158	0	0	0	OK
ENSBTAG00000007692	ENSBTAG00000007692	6:92487185-92487991	0	0	0	OK
ENSBTAG00000007693	ENSBTAG00000007693	16:3244644-3250256	0	0	0	OK
ENSBTAG00000007694	ENSBTAG00000007694	9:103936142-103982549	0	0	0	OK
ENSBTAG00000007695	ENSBTAG00000007695	6:87141555-87159096	0	0	0	OK
ENSBTAG00000007702	ENSBTAG00000007702	13:29959799-29965680	0	0	0	OK
ENSBTAG00000007721	ENSBTAG00000007721	13:79580468-79597861	0	0	0	OK
ENSBTAG00000007728	ENSBTAG00000007728	17:71681087-71700449	0	0	0	OK
ENSBTAG00000007729	ENSBTAG00000007729	20:6205299-6206217	0	0	0	OK
ENSBTAG00000007737	ENSBTAG00000007737	7:4535466-4537851	0	0	0	OK
ENSBTAG00000007739	ENSBTAG00000007739	7:4531209-4533855	0	0	0	OK
ENSBTAG00000007740	ENSBTAG00000007740	15:77249973-77251941	0	0	0.523532	OK
ENSBTAG00000007761	ENSBTAG00000007761	8:61954946-61981732	0	0	0	OK
ENSBTAG00000007785	ENSBTAG00000007785	7:50959964-51005245	0	0	0	OK
ENSBTAG00000007787	ENSBTAG00000007787	2:5934155-6032782	0	0	0	OK
ENSBTAG00000007810	ENSBTAG00000007810	3:88263776-88264749	0	0	0	OK
ENSBTAG00000007816	ENSBTAG00000007816	6:87555287-87556157	0	0	0	OK

ENSBTAG00000007838	ENSBTAG00000007838	14:1620569-1622643	0	0	0	OK
ENSBTAG00000007839	ENSBTAG00000007839	X:85677238-85678114	0	0	0	OK
ENSBTAG00000007847	ENSBTAG00000007847	29:9325135-9355669	0	0	0	OK
ENSBTAG00000007849	ENSBTAG00000007849	29:38567090-38571882	0	0	0	OK
ENSBTAG00000007851	ENSBTAG00000007851	16:4608638-4612613	0	0	0	OK
ENSBTAG00000007866	ENSBTAG00000007866	19:32829906-32869783	0	0	0	OK
ENSBTAG00000007878	ENSBTAG00000007878	28:43204019-43239165	0	0	0	OK
ENSBTAG00000007883	ENSBTAG00000007883	26:11110264-11111041	0	0	0	OK
ENSBTAG00000007897	ENSBTAG00000007897	11:74109409-74116893	0	0	0	OK
ENSBTAG00000007898	ENSBTAG00000007898	2:24956533-24987973	0	0	0	OK
ENSBTAG00000007914	ENSBTAG00000007914	4:92315806-92316363	0	0	0	OK
ENSBTAG00000007916	ENSBTAG00000007916	19:56171931-56175103	0	0	0	OK
ENSBTAG00000007920	ENSBTAG00000007920	13:51736323-51765187	0	0	0	OK
ENSBTAG00000007932	ENSBTAG00000007932	13:62106256-62151619	0	0	0	OK
ENSBTAG00000007934	ENSBTAG00000007934	25:27911079-27915275	0	0	0	OK
ENSBTAG00000007939	ENSBTAG00000007939	1:34970459-35004356	0	0	0	OK
ENSBTAG00000007956	ENSBTAG00000007956	26:18430346-18431174	0	0	0	OK
ENSBTAG00000007983	ENSBTAG00000007983	4:113629208-113630153	0	0	0	OK
ENSBTAG00000007996	ENSBTAG00000007996	2:133610638-133623061	0	0	0	OK
ENSBTAG00000007997	ENSBTAG00000007997	16:19881715-19882860	0	0	0	OK
ENSBTAG00000007998	ENSBTAG00000007998	2:133502453-133607696	0	0	0	OK
ENSBTAG00000008009	ENSBTAG00000008009	19:35543310-35546100	0	0	0	OK
ENSBTAG00000008013	ENSBTAG00000008013	22:53448775-53486047	0	0	0	OK
ENSBTAG00000008026	ENSBTAG00000008026	13:52575277-52576188	0	0	0	OK
ENSBTAG00000008027	ENSBTAG00000008027	13:52563656-52565676	0	0	0	OK
ENSBTAG00000008084	ENSBTAG00000008084	18:47150304-47180829	0	0	0	OK
ENSBTAG00000008085	ENSBTAG00000008085	23:22245232-22261583	0	0	0	OK
ENSBTAG00000008096	ENSBTAG00000008096	23:44010443-44017425	0	0	0	OK
ENSBTAG00000008127	ENSBTAG00000008127	5:103612391-103617657	0	0	0	OK
ENSBTAG00000008135	ENSBTAG00000008135	10:89914333-89923167	0	0	0	OK

ENSBTAG00000008143	ENSBTAG00000008143	4:78160490-78174798	0	0	0	OK
ENSBTAG00000008144	ENSBTAG00000008144	9:41200984-41236600	0	0	0	OK
ENSBTAG00000008154	ENSBTAG00000008154	19:48624049-48625915	0	0	0	OK
ENSBTAG00000008155	ENSBTAG00000008155	4:7612874-7652099	0	0	0	OK
ENSBTAG00000008158	ENSBTAG00000008158	24:54728526-54786445	0	0	0	OK
ENSBTAG00000008169	ENSBTAG00000008169	24:54209586-54228633	0	0	0	OK
ENSBTAG00000008188	ENSBTAG00000008188	25:19975918-19981315	0	0	0	OK
ENSBTAG00000008195	ENSBTAG00000008195	25:28005385-28014212	0	0	0.209466	OK
ENSBTAG00000008197	ENSBTAG00000008197	7:16998452-17003707	0	0	0	OK
ENSBTAG00000008204	ENSBTAG00000008204	8:51344226-51383813	0	0	0	OK
ENSBTAG00000008219	ENSBTAG00000008219	15:25256165-25263448	0	0	0.221333	OK
ENSBTAG00000008222	ENSBTAG00000008222	25:4119350-4130232	0	0	0	OK
ENSBTAG00000008238	ENSBTAG00000008238	3:16967601-16971446	0	0	0	OK
ENSBTAG00000008244	ENSBTAG00000008244	22:15829644-15831175	0	0	0	OK
ENSBTAG00000008278	ENSBTAG00000008278	X:40097614-40102129	0	0	0	OK
ENSBTAG00000008291	ENSBTAG00000008291	13:65052809-65106553	0	0	0	OK
ENSBTAG00000008312	ENSBTAG00000008312	15:50008043-50009176	0	0	0	OK
ENSBTAG00000008318	ENSBTAG00000008318	19:26031609-26042006	0	0	0	OK
ENSBTAG00000008325	ENSBTAG00000008325	27:28188742-28189079	0	0	0	OK
ENSBTAG00000008328	ENSBTAG00000008328	19:57552925-57556471	0	0	0	OK
ENSBTAG00000008329	ENSBTAG00000008329	2:38682029-38711144	0	0	0	OK
ENSBTAG00000008331	ENSBTAG00000008331	2:121604098-121611301	0	0	0.292041	OK
ENSBTAG00000008340	ENSBTAG00000008340	7:64945748-64961110	0	0	0	OK
ENSBTAG00000008347	ENSBTAG00000008347	15:54065465-54120351	0	0	0	OK
ENSBTAG00000008361	ENSBTAG00000008361	4:12493205-12520270	0	0	0	OK
ENSBTAG00000008368	ENSBTAG00000008368	19:40363702-40372714	0	0	0	OK
ENSBTAG00000008369	ENSBTAG00000008369	3:21553391-21555392	0	0	0	OK
ENSBTAG00000008384	ENSBTAG00000008384	21:66877660-66882899	0	0	0	OK
ENSBTAG00000008389	ENSBTAG00000008389	26:42659875-42716633	0	0	0	OK
ENSBTAG00000008397	ENSBTAG00000008397	7:22088468-22099654	0	0	0	OK

ENSBTAG00000008406	ENSBTAG00000008406	22:51969880-51971755	0	0	0	OK
ENSBTAG00000008426	ENSBTAG00000008426	8:79242447-79242725	0	0	0	OK
ENSBTAG00000008434	ENSBTAG00000008434	5:110125414-110132060	0	0	0	OK
ENSBTAG00000008438	ENSBTAG00000008438	17:4732241-4868678	0	0	0	OK
ENSBTAG00000008481	ENSBTAG00000008481	19:24470225-24471218	0	0	0	OK
ENSBTAG00000008487	ENSBTAG00000008487	25:34091770-34096054	0	0	0	OK
ENSBTAG00000008502	ENSBTAG00000008502	18:49711778-49714686	0	0	0	OK
ENSBTAG00000008512	ENSBTAG00000008512	14:56652420-56658519	0	0	0	OK
ENSBTAG00000008517	ENSBTAG00000008517	23:47623802-47624354	0	0	0	OK
ENSBTAG00000008519	ENSBTAG00000008519	27:4798610-4801398	0	0	0	OK
ENSBTAG00000008530	ENSBTAG00000008530	29:1681371-1719249	0	0	0	OK
ENSBTAG00000008537	ENSBTAG00000008537	8:77106497-77165165	0	0	0	OK
ENSBTAG00000008539	ENSBTAG00000008539	8:77234223-77236116	0	0	0	OK
ENSBTAG00000008541	ENSBTAG00000008541	5:93926790-93950162	0	0	0	OK
ENSBTAG00000008555	ENSBTAG00000008555	X:78379314-78398023	0	0	0	OK
ENSBTAG00000008578	ENSBTAG00000008578	7:4818616-4831161	0	0	0	OK
ENSBTAG00000008583	ENSBTAG00000008583	28:26876455-26894874	0	0	0	OK
ENSBTAG00000008587	ENSBTAG00000008587	17:73309906-73315353	0	0	0	OK
ENSBTAG00000008595	ENSBTAG00000008595	5:38397359-38571901	0	0	0	OK
ENSBTAG00000008632	ENSBTAG00000008632	25:26389489-26390167	0	0	0	OK
ENSBTAG00000008634	ENSBTAG00000008634	19:56394187-56401350	0	0	0	OK
ENSBTAG00000008637	ENSBTAG00000008637	27:16702702-16711994	0	0	0	OK
ENSBTAG00000008642	ENSBTAG00000008642	12:32523507-32556412	0	0	0	OK
ENSBTAG00000008661	ENSBTAG00000008661	15:22575918-22582373	0	0	0	OK
ENSBTAG00000008692	ENSBTAG00000008692	7:45592772-45595693	0	0	0	OK
ENSBTAG00000008711	ENSBTAG00000008711	3:121098865-121111324	0	0	0	OK
ENSBTAG00000008716	ENSBTAG00000008716	2:26591741-26599728	0	0	0.170277	OK
ENSBTAG00000008723	ENSBTAG00000008723	24:58886143-58924772	0	0	0	OK
ENSBTAG00000008726	ENSBTAG00000008726	11:100548299-100564121	0	0	0	OK
ENSBTAG00000008735	ENSBTAG00000008735	10:89026340-89044338	0	0	0	OK

ENSBTAG00000008749	ENSBTAG00000008749	9:76409931-76410750	0	0	0	OK
ENSBTAG00000008752	ENSBTAG00000008752	7:51153751-51182289	0	0	0	OK
ENSBTAG00000008763	ENSBTAG00000008763	X:10981500-10983415	0	0	0	OK
ENSBTAG00000008765	ENSBTAG00000008765	11:11202918-11207042	0	0	0	OK
ENSBTAG00000008778	ENSBTAG00000008778	22:61123419-61130341	0	0	0	OK
ENSBTAG00000008783	ENSBTAG00000008783	26:12376624-12513781	0	0	0	OK
ENSBTAG00000008789	ENSBTAG00000008789	7:21575112-21577011	0	0	0	OK
ENSBTAG00000008807	ENSBTAG00000008807	10:46461586-46466076	0	0	0	OK
ENSBTAG00000008833	ENSBTAG00000008833	23:27459276-27461382	0	0	0	OK
ENSBTAG00000008857	ENSBTAG00000008857	5:110136655-110138547	0	0	0	OK
ENSBTAG00000008858	ENSBTAG00000008858	5:116071376-116109883	0	0	0	OK
ENSBTAG00000008864	ENSBTAG00000008864	14:39369424-39402263	0	0	0	OK
ENSBTAG00000008870	ENSBTAG00000008870	13:74191214-74196365	0	0	0	OK
ENSBTAG00000008892	ENSBTAG00000008892	15:47464648-47465611	0	0	0	OK
ENSBTAG00000008894	ENSBTAG00000008894	3:41127031-41127631	0	0	0	OK
ENSBTAG00000008897	ENSBTAG00000008897	5:28640674-28677837	0	0	0	OK
ENSBTAG00000008898	ENSBTAG00000008898	3:116266171-116268268	0	0	0	OK
ENSBTAG00000008910	ENSBTAG00000008910	5:76012700-76013957	0	0	0	OK
ENSBTAG00000008913	ENSBTAG00000008913	19:17622148-17634850	0	0	0	OK
ENSBTAG00000008945	ENSBTAG00000008945	17:63278103-63289816	0	0	0	OK
ENSBTAG00000008962	ENSBTAG00000008962	8:92744049-92751166	0	0	0	OK
ENSBTAG00000009002	ENSBTAG00000009002	4:116237817-116238487	0	0	0	OK
ENSBTAG00000009013	ENSBTAG00000009013	2:115488209-115488855	0	0	0	OK
ENSBTAG00000009023	ENSBTAG00000009023	5:18010338-18047085	0	0	0	OK
ENSBTAG00000009039	ENSBTAG00000009039	10:27343497-27344469	0	0	0	OK
ENSBTAG00000009071	ENSBTAG00000009071	7:15302197-15320851	0	0	0	OK
ENSBTAG00000009073	ENSBTAG00000009073	8:86579143-86579452	0	0	0	OK
ENSBTAG00000009084	ENSBTAG00000009084	1:57752208-57786798	0	0	0	OK
ENSBTAG00000009087	ENSBTAG00000009087	8:102784500-102790524	0	0	0	OK
ENSBTAG00000009104	ENSBTAG00000009104	1:118537062-118539344	0	0	0	OK

ENSBTAG00000009110	ENSBTAG00000009110	17:18734078-18735046	0	0	0	OK
ENSBTAG00000009112	ENSBTAG00000009112	23:24637968-24702830	0	0	0	OK
ENSBTAG00000009124	ENSBTAG00000009124	29:29274998-29317604	0	0	0	OK
ENSBTAG00000009139	ENSBTAG00000009139	24:43861726-43919241	0	0	0	OK
ENSBTAG00000009142	ENSBTAG00000009142	24:43942057-43967106	0	0	0	OK
ENSBTAG00000009156	ENSBTAG00000009156	2:132751702-132754808	0	0	0	OK
ENSBTAG00000009167	ENSBTAG00000009167	17:49336514-49365143	0	0	0	OK
ENSBTAG00000009174	ENSBTAG00000009174	18:55878570-55899034	0	0	0	OK
ENSBTAG00000009181	ENSBTAG00000009181	26:51126573-51200732	0	0	0	OK
ENSBTAG00000009200	ENSBTAG00000009200	25:3499874-3505894	0	0	0	OK
ENSBTAG00000009206	ENSBTAG00000009206	13:61924321-61925629	0	0	0	OK
ENSBTAG00000009211	ENSBTAG00000009211	18:62537350-62541236	0	0	0	OK
ENSBTAG00000009212	ENSBTAG00000009212	3:8301475-8302729	0	0	0	OK
ENSBTAG00000009220	ENSBTAG00000009220	22:31620949-31621322	0	0	0	OK
ENSBTAG00000009230	ENSBTAG00000009230	11:197982-230642	0	0	0	OK
ENSBTAG00000009233	ENSBTAG00000009233	25:38832484-38841319	0	0	0	OK
ENSBTAG00000009246	ENSBTAG00000009246	11:14683784-14696398	0	0	0	OK
ENSBTAG00000009251	ENSBTAG00000009251	2:136317924-136337845	0	0	0	OK
ENSBTAG00000009252	ENSBTAG00000009252	5:99463833-99478716	0	0	0	OK
ENSBTAG00000009268	ENSBTAG00000009268	29:18522320-18522888	0	0	0	OK
ENSBTAG00000009273	ENSBTAG00000009273	7:44174212-44175537	0	0	0	OK
ENSBTAG00000009275	ENSBTAG00000009275	9:91082263-91096760	0	0	0	OK
ENSBTAG00000009280	ENSBTAG00000009280	6:10923399-10924214	0	0	0	OK
ENSBTAG00000009294	ENSBTAG00000009294	21:66740465-66760017	0	0	0	OK
ENSBTAG00000009304	ENSBTAG00000009304	18:43254167-43260903	0	0	0	OK
ENSBTAG00000009307	ENSBTAG00000009307	16:51688691-51698728	0	0	0	OK
ENSBTAG00000009308	ENSBTAG00000009308	12:1527006-1752049	0	0	0	OK
ENSBTAG00000009345	ENSBTAG00000009345	19:62506949-62514776	0	0	0	OK
ENSBTAG00000009364	ENSBTAG00000009364	18:61714019-61719354	0	0	0	OK
ENSBTAG00000009368	ENSBTAG00000009368	3:107709949-107724368	0	0	0	OK

ENSBTAG00000009372	ENSBTAG00000009372	10:26841793-26858144	0	0	0	OK
ENSBTAG00000009374	ENSBTAG00000009374	10:26799839-26814032	0	0	0	OK
ENSBTAG00000009378	ENSBTAG00000009378	14:79759567-79767626	0	0	0	OK
ENSBTAG00000009407	ENSBTAG00000009407	19:36170452-36207184	0	0	0	OK
ENSBTAG00000009416	ENSBTAG00000009416	12:15203255-15220754	0	0	0	OK
ENSBTAG00000009417	ENSBTAG00000009417	8:48892498-48902995	0	0	0	OK
ENSBTAG00000009432	ENSBTAG00000009432	3:14605970-14627746	0	0	0	OK
ENSBTAG00000009447	ENSBTAG00000009447	5:106812248-106852877	0	0	0	OK
ENSBTAG00000009457	ENSBTAG00000009457	X:52793106-52794418	0	0	0	OK
ENSBTAG00000009468	ENSBTAG00000009468	20:14723559-14746316	0	0	0	OK
ENSBTAG00000009477	ENSBTAG00000009477	X:135257226-135366022	0	0	0	OK
ENSBTAG00000009479	ENSBTAG00000009479	7:46069105-46070318	0	0	0	OK
ENSBTAG00000009487	ENSBTAG00000009487	29:41795547-41796692	0	0	0	OK
ENSBTAG00000009498	ENSBTAG00000009498	26:9498225-9595849	0	0	0	OK
ENSBTAG00000009509	ENSBTAG00000009509	5:113575148-113586140	0	0	0	OK
ENSBTAG00000009511	ENSBTAG00000009511	15:13987257-13987770	0	0	0	OK
ENSBTAG00000009530	ENSBTAG00000009530	7:53726355-53727631	0	0	0	OK
ENSBTAG00000009532	ENSBTAG00000009532	5:116959715-116966318	0	0	0	OK
ENSBTAG00000009537	ENSBTAG00000009537	5:100328705-100340514	0	0	0	OK
ENSBTAG00000009539	ENSBTAG00000009539	14:43781698-43783047	0	0	0	OK
ENSBTAG00000009542	ENSBTAG00000009542	5:57522061-57527396	0	0	0	OK
ENSBTAG00000009558	ENSBTAG00000009558	4:106936772-106937743	0	0	0	OK
ENSBTAG00000009570	ENSBTAG00000009570	7:16878926-16880796	0	0	0	OK
ENSBTAG00000009580	ENSBTAG00000009580	2:127429584-127431204	0	0	0	OK
ENSBTAG00000009599	ENSBTAG00000009599	11:106427894-106430697	0	0	0	OK
ENSBTAG00000009620	ENSBTAG00000009620	9:104927414-105066196	0	0	0	OK
ENSBTAG00000009626	ENSBTAG00000009626	13:75227427-75231110	0	0	0	OK
ENSBTAG00000009642	ENSBTAG00000009642	10:76927472-76928759	0	0	0	OK
ENSBTAG00000009664	ENSBTAG00000009664	2:118927758-118962018	0	0	0	OK
ENSBTAG00000009674	ENSBTAG00000009674	3:95726473-95760764	0	0	0	OK

ENSBTAG00000009692	ENSBTAG00000009692	8:9214581-9215012	0	0	0	OK
ENSBTAG00000009693	ENSBTAG00000009693	26:15754510-15850136	0	0	0	OK
ENSBTAG00000009701	ENSBTAG00000009701	10:21383450-21386379	0	0	0	OK
ENSBTAG00000009733	ENSBTAG00000009733	8:82460863-82491694	0	0	0	OK
ENSBTAG00000009734	ENSBTAG00000009734	8:63338260-63365679	0	0	0	OK
ENSBTAG00000009737	ENSBTAG00000009737	7:1418470-1421386	0	0	0	OK
ENSBTAG00000009750	ENSBTAG00000009750	7:21432607-21435431	0	0	0	OK
ENSBTAG00000009763	ENSBTAG00000009763	28:44833887-44836186	0	0	0	OK
ENSBTAG00000009775	ENSBTAG00000009775	X:34402711-34407507	0	0	0	OK
ENSBTAG00000009812	ENSBTAG00000009812	6:90645963-90648076	0	0	0	OK
ENSBTAG00000009816	ENSBTAG00000009816	14:1838134-1840081	0	0	0	OK
ENSBTAG00000009823	ENSBTAG00000009823	4:87410390-87412230	0	0	0	OK
ENSBTAG00000009837	ENSBTAG00000009837	25:19268280-19279662	0	0	0	OK
ENSBTAG00000009848	ENSBTAG00000009848	1:117829438-117835976	0	0	0	OK
ENSBTAG00000009862	ENSBTAG00000009862	10:70069548-70098697	0	0	0	OK
ENSBTAG00000009886	ENSBTAG00000009886	5:110660390-110673555	0	0	0	OK
ENSBTAG00000009900	ENSBTAG00000009900	12:1244344-1245089	0	0	0	OK
ENSBTAG00000009904	ENSBTAG00000009904	5:65663319-65676286	0	0	0	OK
ENSBTAG00000009914	ENSBTAG00000009914	20:70986109-70991390	0	0	0	OK
ENSBTAG00000009916	ENSBTAG00000009916	14:18015439-18033296	0	0	0	OK
ENSBTAG00000009951	ENSBTAG00000009951	25:1746199-1747344	0	0	0	OK
ENSBTAG00000009952	ENSBTAG00000009952	2:88897205-88908836	0	0	0	OK
ENSBTAG00000009956	ENSBTAG00000009956	5:113444185-113456257	0	0	0	OK
ENSBTAG00000009959	ENSBTAG00000009959	X:39828309-39835611	0	0	0	OK
ENSBTAG00000009965	ENSBTAG00000009965	25:1767069-1779723	0	0	0	OK
ENSBTAG00000009966	ENSBTAG00000009966	21:69960276-69967811	0	0	0	OK
ENSBTAG00000009975	ENSBTAG00000009975	7:3631054-3685738	0	0	0	OK
ENSBTAG00000009976	ENSBTAG00000009976	2:84413661-84414878	0	0	0	OK
ENSBTAG00000009991	ENSBTAG00000009991	19:54020559-54024284	0	0	0	OK
ENSBTAG00000010005	ENSBTAG00000010005	15:79629872-79630918	0	0	0	OK

ENSBTAG00000010010	ENSBTAG00000010010	6:8031524-8032117	0	0	0	OK
ENSBTAG00000010016	ENSBTAG00000010016	16:72622939-72635365	0	0	0	OK
ENSBTAG00000010020	ENSBTAG00000010020	7:39875476-39876428	0	0	0	OK
ENSBTAG00000010035	ENSBTAG00000010035	2:117432359-117433630	0	0	0	OK
ENSBTAG00000010057	ENSBTAG00000010057	21:35264589-35267979	0	0	0	OK
ENSBTAG00000010068	ENSBTAG00000010068	7:51421164-51427158	0	0	0	OK
ENSBTAG00000010073	ENSBTAG00000010073	19:20810486-20815216	0	0	0	OK
ENSBTAG00000010083	ENSBTAG00000010083	11:73215423-73246167	0	0	0	OK
ENSBTAG00000010105	ENSBTAG00000010105	26:15233564-15256879	0	0	0	OK
ENSBTAG00000010123	ENSBTAG00000010123	18:53040104-53042792	0	0	0	OK
ENSBTAG00000010144	ENSBTAG00000010144	5:70596428-70602003	0	0	0	OK
ENSBTAG00000010153	ENSBTAG00000010153	6:95065255-95136945	0	0	0	OK
ENSBTAG00000010156	ENSBTAG00000010156	12:15518991-15522535	0	0	0	OK
ENSBTAG00000010168	ENSBTAG00000010168	4:24220692-24222563	0	0	0	OK
ENSBTAG00000010175	ENSBTAG00000010175	3:110299483-110302198	0	0	0	OK
ENSBTAG00000010177	ENSBTAG00000010177	16:1781212-1794807	0	0	0	OK
ENSBTAG00000010181	ENSBTAG00000010181	6:118335667-118349721	0	0	0	OK
ENSBTAG00000010225	ENSBTAG00000010225	2:4554333-4565801	0	0	0	OK
ENSBTAG00000010228	ENSBTAG00000010228	11:97475305-97481276	0	0	0	OK
ENSBTAG00000010232	ENSBTAG00000010232	3:107558701-107564198	0	0	0	OK
ENSBTAG00000010238	ENSBTAG00000010238	10:20687229-20689253	0	0	0	OK
ENSBTAG00000010244	ENSBTAG00000010244	23:19167357-19333272	0	0	0	OK
ENSBTAG00000010247	ENSBTAG00000010247	8:97035506-97073759	0	0	0	OK
ENSBTAG00000010248	ENSBTAG00000010248	27:5715152-5716104	0	0	0	OK
ENSBTAG00000010270	ENSBTAG00000010270	10:85431809-85434976	0	0	0	OK
ENSBTAG00000010273	ENSBTAG00000010273	6:91065005-91071187	0	0	0	OK
ENSBTAG00000010277	ENSBTAG00000010277	13:41104462-41107079	0	0	0	OK
ENSBTAG00000010293	ENSBTAG00000010293	2:93858352-93910303	0	0	0	OK
ENSBTAG00000010298	ENSBTAG00000010298	10:50655753-50687544	0	0	0	OK
ENSBTAG00000010336	ENSBTAG00000010336	29:50856121-50863486	0	0	0	OK

ENSBTAG00000010355	ENSBTAG00000010355	27:19018676-19052353	0	0	0	OK
ENSBTAG00000010378	ENSBTAG00000010378	2:122620932-122638734	0	0	0	OK
ENSBTAG00000010379	ENSBTAG00000010379	7:4363529-4447524	0	0	0	OK
ENSBTAG00000010393	ENSBTAG00000010393	2:125439384-125462744	0	0	0	OK
ENSBTAG00000010399	ENSBTAG00000010399	10:11563977-11652151	0	0	0	OK
ENSBTAG00000010407	ENSBTAG00000010407	21:61181837-61183400	0	0	0	OK
ENSBTAG00000010442	ENSBTAG00000010442	26:11280206-11335233	0	0	0	OK
ENSBTAG00000010445	ENSBTAG00000010445	3:59874335-59892332	0	0	0	OK
ENSBTAG00000010447	ENSBTAG00000010447	29:50238210-50276209	0	0	0	OK
ENSBTAG00000010448	ENSBTAG00000010448	1:139678040-139842029	0	0	0	OK
ENSBTAG00000010461	ENSBTAG00000010461	26:50661557-50662728	0	0	0	OK
ENSBTAG00000010470	ENSBTAG00000010470	29:41698904-41699788	0	0	0	OK
ENSBTAG00000010472	ENSBTAG00000010472	10:59543077-59543674	0	0	0	OK
ENSBTAG00000010486	ENSBTAG00000010486	15:577326-578564	0	0	0	OK
ENSBTAG00000010500	ENSBTAG00000010500	28:3323729-3333934	0	0	0	OK
ENSBTAG00000010503	ENSBTAG00000010503	10:70929453-70932764	0	0	0	OK
ENSBTAG00000010509	ENSBTAG00000010509	19:56275537-56279838	0	0	0	OK
ENSBTAG00000010530	ENSBTAG00000010530	5:89217218-89222769	0	0	0	OK
ENSBTAG00000010531	ENSBTAG00000010531	11:20493492-20499001	0	0	0	OK
ENSBTAG00000010534	ENSBTAG00000010534	19:35557244-35646258	0	0	0	OK
ENSBTAG00000010535	ENSBTAG00000010535	8:101520565-101552954	0	0	0	OK
ENSBTAG00000010536	ENSBTAG00000010536	21:42404326-42405349	0	0	0	OK
ENSBTAG00000010544	ENSBTAG00000010544	11:55038344-55039157	0	0	0	OK
ENSBTAG00000010550	ENSBTAG00000010550	1:97308270-97324962	0	0	0	OK
ENSBTAG00000010560	ENSBTAG00000010560	3:33081436-33086434	0	0	0	OK
ENSBTAG00000010578	ENSBTAG00000010578	7:62769960-62773956	0	0	0	OK
ENSBTAG00000010579	ENSBTAG00000010579	2:98416823-98456944	0	0	0	OK
ENSBTAG00000010641	ENSBTAG00000010641	10:46788193-46828932	0	0	0	OK
ENSBTAG00000010670	ENSBTAG00000010670	3:9942946-9950485	0	0	0	OK
ENSBTAG00000010673	ENSBTAG00000010673	3:15587569-15591685	0	0	0	OK

ENSBTAG00000010676	ENSBTAG00000010676	3:117880749-117900879	0	0	0	OK
ENSBTAG00000010679	ENSBTAG00000010679	1:98303614-98330683	0	0	0	OK
ENSBTAG00000010680	ENSBTAG00000010680	12:51005935-51007152	0	0	0	OK
ENSBTAG00000010682	ENSBTAG00000010682	23:27975618-27989666	0	0	0	OK
ENSBTAG00000010690	ENSBTAG00000010690	12:32352253-32357227	0	0	0	OK
ENSBTAG00000010694	ENSBTAG00000010694	28:14359125-14416928	0	0	0	OK
ENSBTAG00000010704	ENSBTAG00000010704	4:92953571-93043887	0	0	0	OK
ENSBTAG00000010722	ENSBTAG00000010722	2:98497588-98498081	0	0	0	OK
ENSBTAG00000010723	ENSBTAG00000010723	23:7405844-7407102	0	0	0	OK
ENSBTAG00000010724	ENSBTAG00000010724	3:80704133-80704719	0	0	0	OK
ENSBTAG00000010730	ENSBTAG00000010730	23:10627243-10628260	0	0	0	OK
ENSBTAG00000010735	ENSBTAG00000010735	7:39207844-39208828	0	0	0	OK
ENSBTAG00000010740	ENSBTAG00000010740	7:39210709-39230761	0	0	0	OK
ENSBTAG00000010741	ENSBTAG00000010741	2:26780741-26798046	0	0	0	OK
ENSBTAG00000010766	ENSBTAG00000010766	10:36896852-36905744	0	0	0	OK
ENSBTAG00000010772	ENSBTAG00000010772	7:44780280-44783986	0	0	0	OK
ENSBTAG00000010784	ENSBTAG00000010784	23:31278370-31281323	0	0	0	OK
ENSBTAG00000010787	ENSBTAG00000010787	12:27400068-27417180	0	0	0	OK
ENSBTAG00000010788	ENSBTAG00000010788	19:10143538-10164011	0	0	0	OK
ENSBTAG00000010796	ENSBTAG00000010796	26:42871214-42877424	0	0	0	OK
ENSBTAG00000010797	ENSBTAG00000010797	26:42882755-42885754	0	0	0	OK
ENSBTAG00000010798	ENSBTAG00000010798	7:52272185-52284986	0	0	0	OK
ENSBTAG00000010813	ENSBTAG00000010813	14:17228478-17229057	0	0	0	OK
ENSBTAG00000010814	ENSBTAG00000010814	3:92898616-92898991	0	0	0	OK
ENSBTAG00000010821	ENSBTAG00000010821	8:24830046-24856903	0	0	0	OK
ENSBTAG00000010829	ENSBTAG00000010829	8:73871607-73877643	0	0	0	OK
ENSBTAG00000010847	ENSBTAG00000010847	17:66074399-66097412	0	0	0	OK
ENSBTAG00000010849	ENSBTAG00000010849	11:2768908-2773197	0	0	0	OK
ENSBTAG00000010873	ENSBTAG00000010873	13:54893953-54894622	0	0	0	OK
ENSBTAG00000010874	ENSBTAG00000010874	1:95795891-95799185	0	0	0	OK

ENSBTAG00000010877	ENSBTAG00000010877	23:9683946-9694015	0	0	0	OK
ENSBTAG00000010880	ENSBTAG00000010880	29:50285048-50287648	0	0	0	OK
ENSBTAG00000010881	ENSBTAG00000010881	X:4074730-4095081	0	0	0	OK
ENSBTAG00000010882	ENSBTAG00000010882	21:68908373-68911835	0	0	0	OK
ENSBTAG00000010889	ENSBTAG00000010889	15:61763305-61767248	0	0	0	OK
ENSBTAG00000010896	ENSBTAG00000010896	6:113624672-113627173	0	0	0	OK
ENSBTAG00000010898	ENSBTAG00000010898	11:14781532-14827970	0	0	0	OK
ENSBTAG00000010909	ENSBTAG00000010909	16:73764975-73768065	0	0	0	OK
ENSBTAG00000010923	ENSBTAG00000010923	X:137004144-137023608	0	0	0	OK
ENSBTAG00000010928	ENSBTAG00000010928	5:28857807-28860739	0	0	0	OK
ENSBTAG00000010930	ENSBTAG00000010930	28:26626941-26633237	0	0	0	OK
ENSBTAG00000010938	ENSBTAG00000010938	2:136647773-136650918	0	0	0	OK
ENSBTAG00000010939	ENSBTAG00000010939	X:126349634-126368264	0	0	0	OK
ENSBTAG00000010940	ENSBTAG00000010940	2:136635159-136637353	0	0	0	OK
ENSBTAG00000010948	ENSBTAG00000010948	X:38498586-38523525	0	0	0	OK
ENSBTAG00000010955	ENSBTAG00000010955	6:92758456-92792459	0	0	0	OK
ENSBTAG00000010959	ENSBTAG00000010959	7:24515177-24521784	0	0	0	OK
ENSBTAG00000010964	ENSBTAG00000010964	21:27608608-27617253	0	0	0	OK
ENSBTAG00000010967	ENSBTAG00000010967	28:17675517-17764274	0	0	0	OK
ENSBTAG00000010970	ENSBTAG00000010970	29:13455662-13456145	0	0	0	OK
ENSBTAG00000010981	ENSBTAG00000010981	21:31017715-31023825	0	0	0	OK
ENSBTAG00000010984	ENSBTAG00000010984	7:47554104-47556032	0	0	0	OK
ENSBTAG00000010991	ENSBTAG00000010991	24:25854674-25863535	0	0	0	OK
ENSBTAG00000010995	ENSBTAG00000010995	21:68789513-68803852	0	0	0	OK
ENSBTAG00000011014	ENSBTAG00000011014	28:28254031-28256945	0	0	0	OK
ENSBTAG00000011045	ENSBTAG00000011045	20:10390981-10399140	0	0	0	OK
ENSBTAG00000011057	ENSBTAG00000011057	23:7662063-7667198	0	0	0	OK
ENSBTAG00000011059	ENSBTAG00000011059	3:95924171-95928574	0	0	0	OK
ENSBTAG00000011080	ENSBTAG00000011080	26:21823911-21829144	0	0	0	OK
ENSBTAG00000011096	ENSBTAG00000011096	5:80616118-80659407	0	0	0	OK

ENSBTAG00000011097	ENSBTAG00000011097	3:78872533-78900130	0	0	0	OK
ENSBTAG00000011113	ENSBTAG00000011113	21:39655562-39657038	0	0	0	OK
ENSBTAG00000011116	ENSBTAG00000011116	1:127041076-127043157	0	0	0	OK
ENSBTAG00000011119	ENSBTAG00000011119	18:55409550-55410492	0	0	0	OK
ENSBTAG00000011120	ENSBTAG00000011120	4:114520833-114541871	0	0	0	OK
ENSBTAG00000011132	ENSBTAG00000011132	29:9706417-9758502	0	0	0	OK
ENSBTAG00000011133	ENSBTAG00000011133	2:112680365-112747206	0	0	0	OK
ENSBTAG00000011134	ENSBTAG00000011134	7:5800190-5803107	0	0	0	OK
ENSBTAG00000011136	ENSBTAG00000011136	7:5781792-5792042	0	0	0	OK
ENSBTAG00000011137	ENSBTAG00000011137	26:18660894-18665346	0	0	0	OK
ENSBTAG00000011138	ENSBTAG00000011138	1:67398384-67457625	0	0	0	OK
ENSBTAG00000011139	ENSBTAG00000011139	1:102337856-102409795	0	0	0	OK
ENSBTAG00000011144	ENSBTAG00000011144	X:38667357-38672737	0	0	0	OK
ENSBTAG00000011145	ENSBTAG00000011145	4:18973420-18980828	0	0	0	OK
ENSBTAG00000011161	ENSBTAG00000011161	8:38683732-38684116	0	0	0	OK
ENSBTAG00000011163	ENSBTAG00000011163	6:105926863-105931259	0	0	0	OK
ENSBTAG00000011191	ENSBTAG00000011191	4:107987827-107988772	0	0	0	OK
ENSBTAG00000011197	ENSBTAG00000011197	26:762047-814255	0	0	0	OK
ENSBTAG00000011202	ENSBTAG00000011202	8:85913463-85928906	0	0	0	OK
ENSBTAG00000011212	ENSBTAG00000011212	18:48648906-48661802	0	0	0	OK
ENSBTAG00000011214	ENSBTAG00000011214	16:47623670-47629295	0	0	0	OK
ENSBTAG00000011236	ENSBTAG00000011236	9:82632472-82633336	0	0	0	OK
ENSBTAG00000011254	ENSBTAG00000011254	7:18496339-18497281	0	0	0	OK
ENSBTAG00000011263	ENSBTAG00000011263	13:65206237-65212123	0	0	0	OK
ENSBTAG00000011266	ENSBTAG00000011266	15:24917132-25116350	0	0	0	OK
ENSBTAG00000011267	ENSBTAG00000011267	1:118097063-118140747	0	0	0	OK
ENSBTAG00000011271	ENSBTAG00000011271	14:65898280-65981442	0	0	0	OK
ENSBTAG00000011277	ENSBTAG00000011277	X:44838039-44838528	0	0	0	OK
ENSBTAG00000011299	ENSBTAG00000011299	6:12611739-12612251	0	0	0	OK
ENSBTAG00000011312	ENSBTAG00000011312	13:41031398-41032534	0	0	0	OK

ENSBTAG00000011350	ENSBTAG00000011350	X:39727311-39736823	0	0	0	OK
ENSBTAG00000011363	ENSBTAG00000011363	3:50905458-51074025	0	0	0	OK
ENSBTAG00000011367	ENSBTAG00000011367	5:10409388-10566010	0	0	0	OK
ENSBTAG00000011370	ENSBTAG00000011370	29:44840075-44840543	0	0	0	OK
ENSBTAG00000011398	ENSBTAG00000011398	7:40310285-40311313	0	0	0	OK
ENSBTAG00000011411	ENSBTAG00000011411	8:60238493-60240044	0	0	0	OK
ENSBTAG00000011413	ENSBTAG00000011413	8:60244673-60247201	0	0	0	OK
ENSBTAG00000011423	ENSBTAG00000011423	1:100791771-100827942	0	0	0	OK
ENSBTAG00000011444	ENSBTAG00000011444	8:60390944-60393499	0	0	0	OK
ENSBTAG00000011460	ENSBTAG00000011460	19:42684411-42689873	0	0	0	OK
ENSBTAG00000011467	ENSBTAG00000011467	29:43825076-43830937	0	0	0	OK
ENSBTAG00000011489	ENSBTAG00000011489	26:21760436-21762788	0	0	0	OK
ENSBTAG00000011521	ENSBTAG00000011521	15:79670544-79671495	0	0	0	OK
ENSBTAG00000011527	ENSBTAG00000011527	19:56955612-56957740	0	0	0	OK
ENSBTAG00000011533	ENSBTAG00000011533	18:14321862-14326425	0	0	0	OK
ENSBTAG00000011537	ENSBTAG00000011537	3:65200087-65200398	0	0	0	OK
ENSBTAG00000011554	ENSBTAG00000011554	11:104296134-104302894	0	0	0	OK
ENSBTAG00000011562	ENSBTAG00000011562	26:51537508-51609090	0	0	0	OK
ENSBTAG00000011573	ENSBTAG00000011573	27:13749507-13826909	0	0	0	OK
ENSBTAG00000011575	ENSBTAG00000011575	19:51429343-51431873	0	0	0	OK
ENSBTAG00000011582	ENSBTAG00000011582	2:122814785-122817735	0	0	0	OK
ENSBTAG00000011602	ENSBTAG00000011602	7:8710305-8722572	0	0	0	OK
ENSBTAG00000011611	ENSBTAG00000011611	6:17789597-17801277	0	0	0	OK
ENSBTAG00000011624	ENSBTAG00000011624	18:55834674-55836133	0	0	0	OK
ENSBTAG00000011631	ENSBTAG00000011631	1:114718439-114719062	0	0	0	OK
ENSBTAG00000011633	ENSBTAG00000011633	22:48147954-48148287	0	0	0	OK
ENSBTAG00000011638	ENSBTAG00000011638	13:54009225-54009998	0	0	0	OK
ENSBTAG00000011662	ENSBTAG00000011662	13:54295398-54296819	0	0	0	OK
ENSBTAG00000011666	ENSBTAG00000011666	29:18084680-18090595	0	0	0	OK
ENSBTAG00000011671	ENSBTAG00000011671	23:31604876-31605513	0	0	0	OK

ENSBTAG00000011693	ENSBTAG00000011693	18:63117357-63118908	0	0	0	OK
ENSBTAG00000011702	ENSBTAG00000011702	22:13271525-13302316	0	0	0	OK
ENSBTAG00000011704	ENSBTAG00000011704	13:63150592-63151022	0	0	0	OK
ENSBTAG00000011720	ENSBTAG00000011720	5:57004645-57026537	0	0	0	OK
ENSBTAG00000011723	ENSBTAG00000011723	18:51520759-51578983	0	0	0	OK
ENSBTAG00000011727	ENSBTAG00000011727	11:65813299-65826759	0	0	0	OK
ENSBTAG00000011731	ENSBTAG00000011731	19:40694078-40695765	0	0	0	OK
ENSBTAG00000011735	ENSBTAG00000011735	9:29748715-29749078	0	0	0	OK
ENSBTAG00000011759	ENSBTAG00000011759	19:45288724-45295349	0	0	0	OK
ENSBTAG00000011764	ENSBTAG00000011764	10:51842777-51843501	0	0	0	OK
ENSBTAG00000011771	ENSBTAG00000011771	17:66628655-66632360	0	0	0	OK
ENSBTAG00000011783	ENSBTAG00000011783	X:108331470-108332164	0	0	0	OK
ENSBTAG00000011812	ENSBTAG00000011812	20:16996170-17006619	0	0	0	OK
ENSBTAG00000011817	ENSBTAG00000011817	6:66519801-66605788	0	0	0	OK
ENSBTAG00000011830	ENSBTAG00000011830	29:46456304-46456753	0	0	0	OK
ENSBTAG00000011836	ENSBTAG00000011836	8:85477016-85479293	0	0	0	OK
ENSBTAG00000011855	ENSBTAG00000011855	12:24708379-24717631	0	0	0	OK
ENSBTAG00000011866	ENSBTAG00000011866	28:27226794-27231724	0	0	0	OK
ENSBTAG00000011871	ENSBTAG00000011871	1:57672615-57701243	0	0	0	OK
ENSBTAG00000011873	ENSBTAG00000011873	15:54587989-54588289	0	0	0	OK
ENSBTAG00000011874	ENSBTAG00000011874	8:44898337-44901902	0	0	0	OK
ENSBTAG00000011895	ENSBTAG00000011895	20:182975-204785	0	0	0	OK
ENSBTAG00000011905	ENSBTAG00000011905	15:38900250-38977399	0	0	0	OK
ENSBTAG00000011913	ENSBTAG00000011913	5:69972286-69979795	0	0	0	OK
ENSBTAG00000011914	ENSBTAG00000011914	15:40235177-40236713	0	0	0	OK
ENSBTAG00000011941	ENSBTAG00000011941	5:44351614-44357569	0	0	0	OK
ENSBTAG00000011954	ENSBTAG00000011954	24:58659024-58670236	0	0	0	OK
ENSBTAG00000011961	ENSBTAG00000011961	6:90673204-90674173	0	0	0	OK
ENSBTAG00000011969	ENSBTAG00000011969	25:34858434-34861074	0	0	0	OK
ENSBTAG00000011987	ENSBTAG00000011987	10:87913059-87921648	0	0	0	OK

ENSBTAG00000011998	ENSBTAG00000011998	10:62284070-62295576	0	0	0	OK
ENSBTAG00000012023	ENSBTAG00000012023	1:95159025-95166199	0	0	0	OK
ENSBTAG00000012025	ENSBTAG00000012025	3:3692342-3865786	0	0	0	OK
ENSBTAG00000012029	ENSBTAG00000012029	5:100949063-100961494	0	0	0	OK
ENSBTAG00000012030	ENSBTAG00000012030	5:114494263-114530242	0	0	0	OK
ENSBTAG00000012040	ENSBTAG00000012040	19:21391292-21400011	0	0	0	OK
ENSBTAG00000012046	ENSBTAG00000012046	7:13851130-13852933	0	0	0	OK
ENSBTAG00000012048	ENSBTAG00000012048	7:16571429-16587354	0	0	0	OK
ENSBTAG00000012059	ENSBTAG00000012059	18:13938826-13945489	0	0	0	OK
ENSBTAG00000012061	ENSBTAG00000012061	3:108508804-108510061	0	0	0	OK
ENSBTAG00000012073	ENSBTAG00000012073	22:495505-677104	0	0	0	OK
ENSBTAG00000012082	ENSBTAG00000012082	25:2439750-2441210	0	0	0	OK
ENSBTAG00000012084	ENSBTAG00000012084	X:40924912-40945291	0	0	0	OK
ENSBTAG00000012087	ENSBTAG00000012087	23:39239185-39240382	0	0	0	OK
ENSBTAG00000012089	ENSBTAG00000012089	X:137548585-137556460	0	0	0	OK
ENSBTAG00000012106	ENSBTAG00000012106	10:90002635-90134649	0	0	0	OK
ENSBTAG00000012132	ENSBTAG00000012132	28:3187635-3188295	0	0	0	OK
ENSBTAG00000012144	ENSBTAG00000012144	11:105562886-105571338	0	0	0	OK
ENSBTAG00000012146	ENSBTAG00000012146	29:45938166-45941106	0	0	0.314199	OK
ENSBTAG00000012161	ENSBTAG00000012161	7:17795678-17796192	0	0	0	OK
ENSBTAG00000012169	ENSBTAG00000012169	29:29974802-29979585	0	0	0	OK
ENSBTAG00000012172	ENSBTAG00000012172	7:45330547-45336636	0	0	0	OK
ENSBTAG00000012177	ENSBTAG00000012177	18:53681069-53683928	0	0	0	OK
ENSBTAG00000012180	ENSBTAG00000012180	10:35856696-35871287	0	0	0	OK
ENSBTAG00000012185	ENSBTAG00000012185	5:102158067-102168602	0	0	0	OK
ENSBTAG00000012189	ENSBTAG00000012189	18:62677952-62679862	0	0	0	OK
ENSBTAG00000012199	ENSBTAG00000012199	21:33646396-33647525	0	0	0	OK
ENSBTAG00000012222	ENSBTAG00000012222	18:2183490-2237316	0	0	0	OK
ENSBTAG00000012279	ENSBTAG00000012279	15:1778698-1802177	0	0	0	OK
ENSBTAG00000012285	ENSBTAG00000012285	10:88763704-88818475	0	0	0	OK

ENSBTAG00000012301	ENSBTAG00000012301	4:89778487-89779050	0	0	0	OK
ENSBTAG00000012322	ENSBTAG00000012322	22:51001139-51002896	0	0	0	OK
ENSBTAG00000012325	ENSBTAG00000012325	15:51490774-51491839	0	0	0	OK
ENSBTAG00000012326	ENSBTAG00000012326	21:20345646-20346465	0	0	0	OK
ENSBTAG00000012341	ENSBTAG00000012341	13:61082323-61106498	0	0	0	OK
ENSBTAG00000012344	ENSBTAG00000012344	19:28590313-28595213	0	0	0	OK
ENSBTAG00000012368	ENSBTAG00000012368	17:35462668-35469800	0	0	0	OK
ENSBTAG00000012372	ENSBTAG00000012372	3:101939346-101955118	0	0	0	OK
ENSBTAG00000012376	ENSBTAG00000012376	2:49736039-49736399	0	0	0	OK
ENSBTAG00000012377	ENSBTAG00000012377	13:12576390-12590757	0	0	0	OK
ENSBTAG00000012393	ENSBTAG00000012393	28:46147259-46156093	0	0	0	OK
ENSBTAG00000012401	ENSBTAG00000012401	1:94276996-94277402	0	0	0	OK
ENSBTAG00000012408	ENSBTAG00000012408	10:55120521-55158773	0	0	0	OK
ENSBTAG00000012413	ENSBTAG00000012413	1:75616650-75895219	0	0	0	OK
ENSBTAG00000012416	ENSBTAG00000012416	26:25938180-25941636	0	0	0	OK
ENSBTAG00000012419	ENSBTAG00000012419	3:29643175-29648016	0	0	0	OK
ENSBTAG00000012425	ENSBTAG00000012425	5:115850419-115851139	0	0	0	OK
ENSBTAG00000012436	ENSBTAG00000012436	19:28375016-28377490	0	0	0	OK
ENSBTAG00000012441	ENSBTAG00000012441	24:49071139-49098714	0	0	0	OK
ENSBTAG00000012462	ENSBTAG00000012462	16:35727293-35733187	0	0	0	OK
ENSBTAG00000012463	ENSBTAG00000012463	1:88632286-88663997	0	0	0	OK
ENSBTAG00000012470	ENSBTAG00000012470	3:16783419-16785104	0	0	0	OK
ENSBTAG00000012471	ENSBTAG00000012471	3:16772701-16783233	0	0	0	OK
ENSBTAG00000012484	ENSBTAG00000012484	11:99314599-99318324	0	0	0	OK
ENSBTAG00000012489	ENSBTAG00000012489	21:31860206-31983703	0	0	0	OK
ENSBTAG00000012502	ENSBTAG00000012502	3:57337985-57338852	0	0	0	OK
ENSBTAG00000012511	ENSBTAG00000012511	29:43190200-43201983	0	0	0	OK
ENSBTAG00000012522	ENSBTAG00000012522	18:52380157-52391523	0	0	0	OK
ENSBTAG00000012529	ENSBTAG00000012529	5:45830157-45834981	0	0	0	OK
ENSBTAG00000012533	ENSBTAG00000012533	X:114089197-114089749	0	0	0	OK

ENSBTAG00000012535	ENSBTAG00000012535	8:5632224-5644628	0	0	0	OK
ENSBTAG00000012546	ENSBTAG00000012546	15:77811543-77812485	0	0	0	OK
ENSBTAG00000012549	ENSBTAG00000012549	15:79276427-79277363	0	0	0	OK
ENSBTAG00000012561	ENSBTAG00000012561	2:6511309-6523389	0	0	0	OK
ENSBTAG00000012563	ENSBTAG00000012563	29:47656593-47657614	0	0	0	OK
ENSBTAG00000012589	ENSBTAG00000012589	2:86449524-86451564	0	0	0	OK
ENSBTAG00000012590	ENSBTAG00000012590	18:49094424-49108117	0	0	0	OK
ENSBTAG00000012605	ENSBTAG00000012605	16:45219135-45241137	0	0	0	OK
ENSBTAG00000012609	ENSBTAG00000012609	5:120032225-120034043	0	0	0	OK
ENSBTAG00000012621	ENSBTAG00000012621	15:82033374-82042917	0	0	0	OK
ENSBTAG00000012622	ENSBTAG00000012622	4:32250995-32269539	0	0	0	OK
ENSBTAG00000012623	ENSBTAG00000012623	X:105124225-105150720	0	0	0	OK
ENSBTAG00000012625	ENSBTAG00000012625	2:96254082-96254476	0	0	0	OK
ENSBTAG00000012637	ENSBTAG00000012637	11:45690019-45699417	0	0	0	OK
ENSBTAG00000012638	ENSBTAG00000012638	3:17163819-17165262	0	0	0	OK
ENSBTAG00000012640	ENSBTAG00000012640	3:17146917-17147898	0	0	0	OK
ENSBTAG00000012642	ENSBTAG00000012642	18:34912744-34918691	0	0	0	OK
ENSBTAG00000012649	ENSBTAG00000012649	4:83517253-83524725	0	0	0	OK
ENSBTAG00000012654	ENSBTAG00000012654	28:29850370-29851646	0	0	0	OK
ENSBTAG00000012658	ENSBTAG00000012658	6:2089640-2119479	0	0	0	OK
ENSBTAG00000012668	ENSBTAG00000012668	25:36844018-36855872	0	0	0	OK
ENSBTAG00000012694	ENSBTAG00000012694	3:6975866-6995683	0	0	0	OK
ENSBTAG00000012699	ENSBTAG00000012699	26:18522298-18528929	0	0	0	OK
ENSBTAG00000012700	ENSBTAG00000012700	3:1230987-1314962	0	0	0	OK
ENSBTAG00000012705	ENSBTAG00000012705	16:28088796-28092872	0	0	0	OK
ENSBTAG00000012706	ENSBTAG00000012706	1:80673150-80675983	0	0	0	OK
ENSBTAG00000012710	ENSBTAG00000012710	13:66863224-66872531	0	0	0	OK
ENSBTAG00000012719	ENSBTAG00000012719	26:18575001-18625622	0	0	0	OK
ENSBTAG00000012722	ENSBTAG00000012722	3:98267304-98314888	0	0	0	OK
ENSBTAG00000012726	ENSBTAG00000012726	10:21658160-21663680	0	0	0	OK

ENSBTAG00000012735	ENSBTAG00000012735	11:98400783-98406834	0	0	0	OK
ENSBTAG00000012736	ENSBTAG00000012736	11:98406892-98408784	0	0	0	OK
ENSBTAG00000012752	ENSBTAG00000012752	23:16656284-16660495	0	0	0	OK
ENSBTAG00000012763	ENSBTAG00000012763	2:90089712-90090737	0	0	0	OK
ENSBTAG00000012765	ENSBTAG00000012765	16:12544469-12545294	0	0	0	OK
ENSBTAG00000012782	ENSBTAG00000012782	5:30070403-30072958	0	0	0	OK
ENSBTAG00000012785	ENSBTAG00000012785	18:25970367-25977551	0	0	0	OK
ENSBTAG00000012788	ENSBTAG00000012788	17:64995247-64997121	0	0	0	OK
ENSBTAG00000012801	ENSBTAG00000012801	2:38326896-38363855	0	0	0	OK
ENSBTAG00000012805	ENSBTAG00000012805	4:25213087-25251241	0	0	0	OK
ENSBTAG00000012815	ENSBTAG00000012815	5:100388588-100401104	0	0	0	OK
ENSBTAG00000012822	ENSBTAG00000012822	23:15892877-15893213	0	0	0	OK
ENSBTAG00000012826	ENSBTAG00000012826	17:30291317-30319495	0	0	0	OK
ENSBTAG00000012828	ENSBTAG00000012828	5:1454192-1506151	0	0	0	OK
ENSBTAG00000012834	ENSBTAG00000012834	7:63648252-63653794	0	0	0	OK
ENSBTAG00000012845	ENSBTAG00000012845	19:20648943-20656236	0	0	0	OK
ENSBTAG00000012876	ENSBTAG00000012876	20:17609459-17610173	0	0	0	OK
ENSBTAG00000012880	ENSBTAG00000012880	11:103874071-103875832	0	0	0	OK
ENSBTAG00000012884	ENSBTAG00000012884	3:10579368-10582728	0	0	0	OK
ENSBTAG00000012886	ENSBTAG00000012886	3:10531765-10532707	0	0	0	OK
ENSBTAG00000012889	ENSBTAG00000012889	24:5357672-5459491	0	0	0	OK
ENSBTAG00000012892	ENSBTAG00000012892	17:70539384-70542488	0	0	0	OK
ENSBTAG00000012896	ENSBTAG00000012896	5:57899028-57901211	0	0	0	OK
ENSBTAG00000012898	ENSBTAG00000012898	10:46946053-46949421	0	0	0	OK
ENSBTAG00000012902	ENSBTAG00000012902	22:21327750-21374042	0	0	0	OK
ENSBTAG00000012909	ENSBTAG00000012909	21:31258090-31265286	0	0	0	OK
ENSBTAG00000012914	ENSBTAG00000012914	26:21240569-21244552	0	0	0	OK
ENSBTAG00000012915	ENSBTAG00000012915	18:52395544-52400150	0	0	0	OK
ENSBTAG00000012916	ENSBTAG00000012916	8:31871389-31872132	0	0	0	OK
ENSBTAG00000012928	ENSBTAG00000012928	25:26460141-26468245	0	0	0	OK

ENSBTAG00000012936	ENSBTAG00000012936	15:84856202-84860720	0	0	0	OK
ENSBTAG00000012949	ENSBTAG00000012949	11:67283596-67289445	0	0	0	OK
ENSBTAG00000012954	ENSBTAG00000012954	X:92016869-92017574	0	0	0	OK
ENSBTAG00000012955	ENSBTAG00000012955	26:35686370-35717037	0	0	0	OK
ENSBTAG00000012962	ENSBTAG00000012962	3:34283444-34303242	0	0	0	OK
ENSBTAG00000012970	ENSBTAG00000012970	26:44619641-44693891	0	0	0	OK
ENSBTAG00000012971	ENSBTAG00000012971	20:55661195-55662245	0	0	0	OK
ENSBTAG00000013003	ENSBTAG00000013003	29:50036100-50037262	0	0	0	OK
ENSBTAG00000013017	ENSBTAG00000013017	3:33952093-33961478	0	0	0	OK
ENSBTAG00000013020	ENSBTAG00000013020	18:65527579-65527912	0	0	0	OK
ENSBTAG00000013026	ENSBTAG00000013026	22:37867679-37884047	0	0	0	OK
ENSBTAG00000013027	ENSBTAG00000013027	22:15190717-15196325	0	0	0	OK
ENSBTAG00000013031	ENSBTAG00000013031	10:38473474-38507255	0	0	0	OK
ENSBTAG00000013036	ENSBTAG00000013036	17:74115868-74117434	0	0	0	OK
ENSBTAG00000013038	ENSBTAG00000013038	17:74120668-74134769	0	0	0	OK
ENSBTAG00000013051	ENSBTAG00000013051	25:9994131-9994413	0	0	0	OK
ENSBTAG00000013059	ENSBTAG00000013059	22:32285751-32286398	0	0	0	OK
ENSBTAG00000013063	ENSBTAG00000013063	11:87994385-88010369	0	0	0	OK
ENSBTAG00000013064	ENSBTAG00000013064	16:13744180-13774629	0	0	0	OK
ENSBTAG00000013068	ENSBTAG00000013068	28:8111376-8123251	0	0	0	OK
ENSBTAG00000013077	ENSBTAG00000013077	X:75149332-75151831	0	0	0	OK
ENSBTAG00000013090	ENSBTAG00000013090	28:26794898-26801991	0	0	0	OK
ENSBTAG00000013106	ENSBTAG00000013106	18:57194636-57205675	0	0	0	OK
ENSBTAG00000013123	ENSBTAG00000013123	5:103853321-103857548	0	0	0	OK
ENSBTAG00000013126	ENSBTAG00000013126	5:103847446-103853139	0	0	0	OK
ENSBTAG00000013132	ENSBTAG00000013132	10:10890934-10899893	0	0	0	OK
ENSBTAG00000013134	ENSBTAG00000013134	19:44268493-44271758	0	0	0	OK
ENSBTAG00000013136	ENSBTAG00000013136	10:102666362-102692145	0	0	0	OK
ENSBTAG00000013148	ENSBTAG00000013148	1:77596385-77611958	0	0	0	OK
ENSBTAG00000013168	ENSBTAG00000013168	4:15600772-15603972	0	0	0	OK

ENSBTAG00000013176	ENSBTAG00000013176	9:104268692-104380527	0	0	0	OK
ENSBTAG00000013196	ENSBTAG00000013196	2:85077800-85097107	0	0	0	OK
ENSBTAG00000013202	ENSBTAG00000013202	5:91771435-91820146	0	0	0	OK
ENSBTAG00000013215	ENSBTAG00000013215	2:104492354-104560643	0	0	0	OK
ENSBTAG00000013218	ENSBTAG00000013218	2:25566350-25599241	0	0	0	OK
ENSBTAG00000013231	ENSBTAG00000013231	8:51330086-51336595	0	0	0	OK
ENSBTAG00000013241	ENSBTAG00000013241	3:91901852-91911965	0	0	0	OK
ENSBTAG00000013248	ENSBTAG00000013248	4:107741182-107742118	0	0	0	OK
ENSBTAG00000013264	ENSBTAG00000013264	28:33926496-33931777	0	0	0	OK
ENSBTAG00000013273	ENSBTAG00000013273	19:33888403-33930428	0	0	0	OK
ENSBTAG00000013274	ENSBTAG00000013274	19:33878365-33886577	0	0	0	OK
ENSBTAG00000013275	ENSBTAG00000013275	16:42876000-42881565	0	0	0	OK
ENSBTAG00000013315	ENSBTAG00000013315	5:57119916-57125290	0	0	0	OK
ENSBTAG00000013321	ENSBTAG00000013321	1:44393264-44490892	0	0	0	OK
ENSBTAG00000013326	ENSBTAG00000013326	17:13364488-13446148	0	0	0	OK
ENSBTAG00000013345	ENSBTAG00000013345	18:61832252-61835027	0	0	0	OK
ENSBTAG00000013349	ENSBTAG00000013349	X:69100654-69101990	0	0	0	OK
ENSBTAG00000013352	ENSBTAG00000013352	21:47196836-47198449	0	0	0	OK
ENSBTAG00000013358	ENSBTAG00000013358	7:84523779-84525639	0	0	0	OK
ENSBTAG00000013363	ENSBTAG00000013363	3:106638794-106667878	0	0	0	OK
ENSBTAG00000013368	ENSBTAG00000013368	26:10542378-10570091	0	0	0	OK
ENSBTAG00000013398	ENSBTAG00000013398	29:1934149-1936897	0	0	0	OK
ENSBTAG00000013406	ENSBTAG00000013406	5:6266017-6274473	0	0	0	OK
ENSBTAG00000013419	ENSBTAG00000013419	29:24810564-24824722	0	0	0	OK
ENSBTAG00000013420	ENSBTAG00000013420	8:96964124-97006477	0	0	0	OK
ENSBTAG00000013423	ENSBTAG00000013423	21:61986089-61996011	0	0	0	OK
ENSBTAG00000013440	ENSBTAG00000013440	18:46544685-46552113	0	0	0	OK
ENSBTAG00000013456	ENSBTAG00000013456	1:77415803-77436583	0	0	0	OK
ENSBTAG00000013480	ENSBTAG00000013480	10:36429385-36460034	0	0	0	OK
ENSBTAG00000013510	ENSBTAG00000013510	11:98425081-98429110	0	0	0.27626	OK

ENSBTAG00000013529	ENSBTAG00000013529	19:45233933-45237988	0	0	0	OK
ENSBTAG00000013530	ENSBTAG00000013530	23:27399305-27402825	0	0	0	OK
ENSBTAG00000013531	ENSBTAG00000013531	16:44017900-44032547	0	0	0	OK
ENSBTAG00000013560	ENSBTAG00000013560	16:55487725-55491592	0	0	0	OK
ENSBTAG00000013581	ENSBTAG00000013581	27:25422501-25432327	0	0	0	OK
ENSBTAG00000013592	ENSBTAG00000013592	28:13223727-13246045	0	0	0	OK
ENSBTAG00000013596	ENSBTAG00000013596	5:64763618-64815380	0	0	0	OK
ENSBTAG00000013598	ENSBTAG00000013598	14:58537070-58653100	0	0	0	OK
ENSBTAG00000013613	ENSBTAG00000013613	7:6313717-6319745	0	0	0	OK
ENSBTAG00000013629	ENSBTAG00000013629	1:140944008-140958949	0	0	0	OK
ENSBTAG00000013635	ENSBTAG00000013635	5:95353126-95363059	0	0	0	OK
ENSBTAG00000013640	ENSBTAG00000013640	5:101402261-101419313	0	0	0	OK
ENSBTAG00000013660	ENSBTAG00000013660	20:52768972-52769341	0	0	0	OK
ENSBTAG00000013665	ENSBTAG00000013665	24:850315-872350	0	0	0	OK
ENSBTAG00000013676	ENSBTAG00000013676	11:26077550-26115657	0	0	0	OK
ENSBTAG00000013685	ENSBTAG00000013685	19:42248646-42252437	0	0	0	OK
ENSBTAG00000013691	ENSBTAG00000013691	7:43156369-43157305	0	0	0	OK
ENSBTAG00000013693	ENSBTAG00000013693	3:86526728-86547281	0	0	0	OK
ENSBTAG00000013717	ENSBTAG00000013717	26:25881751-25885322	0	0	0	OK
ENSBTAG00000013723	ENSBTAG00000013723	22:57865202-57909687	0	0	0	OK
ENSBTAG00000013734	ENSBTAG00000013734	11:28808720-28816690	0	0	0	OK
ENSBTAG00000013735	ENSBTAG00000013735	28:8143950-8232969	0	0	0.219108	OK
ENSBTAG00000013749	ENSBTAG00000013749	11:28834379-28871742	0	0	0	OK
ENSBTAG00000013755	ENSBTAG00000013755	1:69801843-69899676	0	0	0	OK
ENSBTAG00000013759	ENSBTAG00000013759	5:107047162-107052441	0	0	0	OK
ENSBTAG00000013784	ENSBTAG00000013784	18:26098514-26132551	0	0	0	OK
ENSBTAG00000013789	ENSBTAG00000013789	3:11039858-11040806	0	0	0	OK
ENSBTAG00000013792	ENSBTAG00000013792	19:56072683-56078553	0	0	0	OK
ENSBTAG00000013799	ENSBTAG00000013799	2:107981291-107982291	0	0	0	OK
ENSBTAG00000013849	ENSBTAG00000013849	16:13213114-13240290	0	0	0	OK

ENSBTAG00000013855	ENSBTAG00000013855	19:40907226-40913526	0	0	0	OK
ENSBTAG00000013858	ENSBTAG00000013858	25:7931599-7955194	0	0	0	OK
ENSBTAG00000013863	ENSBTAG00000013863	20:4449108-4452189	0	0	0	OK
ENSBTAG00000013866	ENSBTAG00000013866	3:16505731-16507247	0	0	0	OK
ENSBTAG00000013875	ENSBTAG00000013875	26:37796164-37801021	0	0	0	OK
ENSBTAG00000013895	ENSBTAG00000013895	29:44496358-44505000	0	0	0	OK
ENSBTAG00000013898	ENSBTAG00000013898	19:51601520-51601969	0	0	0	OK
ENSBTAG00000013923	ENSBTAG00000013923	11:19815844-19846493	0	0	0	OK
ENSBTAG00000013924	ENSBTAG00000013924	18:56404236-56406668	0	0	0	OK
ENSBTAG00000013928	ENSBTAG00000013928	13:75077193-75084338	0	0	0	OK
ENSBTAG00000013957	ENSBTAG00000013957	2:30642214-30666124	0	0	0	OK
ENSBTAG00000013981	ENSBTAG00000013981	21:45302739-45321878	0	0	0	OK
ENSBTAG00000013990	ENSBTAG00000013990	2:107544682-107556681	0	0	0	OK
ENSBTAG00000013992	ENSBTAG00000013992	10:86702096-86708378	0	0	0	OK
ENSBTAG00000013994	ENSBTAG00000013994	15:34136077-34139761	0	0	0	OK
ENSBTAG00000014008	ENSBTAG00000014008	8:71698453-71702901	0	0	0	OK
ENSBTAG00000014016	ENSBTAG00000014016	4:5424473-5494384	0	0	0	OK
ENSBTAG00000014017	ENSBTAG00000014017	6:108902715-108930463	0	0	0	OK
ENSBTAG00000014020	ENSBTAG00000014020	28:18363060-18471853	0	0	0	OK
ENSBTAG00000014027	ENSBTAG00000014027	10:3921476-3962283	0	0	0	OK
ENSBTAG00000014028	ENSBTAG00000014028	26:43096099-43109229	0	0	0	OK
ENSBTAG00000014034	ENSBTAG00000014034	1:128687855-128704514	0	0	0	OK
ENSBTAG00000014048	ENSBTAG00000014048	13:48282727-48394530	0	0	0	OK
ENSBTAG00000014054	ENSBTAG00000014054	9:63346331-63382321	0	0	0	OK
ENSBTAG00000014056	ENSBTAG00000014056	27:37195912-37209034	0	0	0	OK
ENSBTAG00000014058	ENSBTAG00000014058	6:116534189-116998914	0	0	0	OK
ENSBTAG00000014072	ENSBTAG00000014072	11:33348436-33349207	0	0	0	OK
ENSBTAG00000014089	ENSBTAG00000014089	21:34063457-34085961	0	0	0	OK
ENSBTAG00000014090	ENSBTAG00000014090	11:2526185-2537850	0	0	0	OK
ENSBTAG00000014092	ENSBTAG00000014092	10:19039467-19068075	0	0	0	OK

ENSBTAG00000014094	ENSBTAG00000014094	2:96813210-96816216	0	0	0	OK
ENSBTAG00000014114	ENSBTAG00000014114	7:4461163-4465421	0	0	0	OK
ENSBTAG00000014118	ENSBTAG00000014118	25:1070286-1081303	0	0	0	OK
ENSBTAG00000014122	ENSBTAG00000014122	29:29991453-29999118	0	0	0	OK
ENSBTAG00000014129	ENSBTAG00000014129	5:57648525-57651738	0	0	0	OK
ENSBTAG00000014130	ENSBTAG00000014130	14:66637800-66647721	0	0	0	OK
ENSBTAG00000014146	ENSBTAG00000014146	27:6752942-6759799	0	0	0	OK
ENSBTAG00000014150	ENSBTAG00000014150	11:98801790-98805189	0	0	0	OK
ENSBTAG00000014154	ENSBTAG00000014154	5:99775090-99779613	0	0	0	OK
ENSBTAG00000014186	ENSBTAG00000014186	13:75518487-75534491	0	0	0	OK
ENSBTAG00000014204	ENSBTAG00000014204	3:16830510-16837422	0	0	0	OK
ENSBTAG00000014218	ENSBTAG00000014218	26:6852969-6855647	0	0	0	OK
ENSBTAG00000014221	ENSBTAG00000014221	2:136457564-136461977	0	0	0	OK
ENSBTAG00000014223	ENSBTAG00000014223	X:57523484-57525442	0	0	0	OK
ENSBTAG00000014228	ENSBTAG00000014228	4:52589573-52812807	0	0	0	OK
ENSBTAG00000014234	ENSBTAG00000014234	10:78878184-78893823	0	0	0	OK
ENSBTAG00000014235	ENSBTAG00000014235	3:111426911-111431172	0	0	0	OK
ENSBTAG00000014238	ENSBTAG00000014238	5:109564502-109578159	0	0	0	OK
ENSBTAG00000014252	ENSBTAG00000014252	13:6743961-6879038	0	0	0	OK
ENSBTAG00000014259	ENSBTAG00000014259	24:30685542-30788156	0	0	0	OK
ENSBTAG00000014262	ENSBTAG00000014262	4:25097883-25161432	0	0	0	OK
ENSBTAG00000014274	ENSBTAG00000014274	12:37793171-37794787	0	0	0	OK
ENSBTAG00000014304	ENSBTAG00000014304	X:113924566-113949565	0	0	0	OK
ENSBTAG00000014320	ENSBTAG00000014320	7:33128259-33152038	0	0	0	OK
ENSBTAG00000014329	ENSBTAG00000014329	13:74314686-74317437	0	0	0	OK
ENSBTAG00000014336	ENSBTAG00000014336	26:23674347-23684808	0	0	0	OK
ENSBTAG00000014343	ENSBTAG00000014343	25:35987214-35992174	0	0	0	OK
ENSBTAG00000014358	ENSBTAG00000014358	3:110118544-110120286	0	0	0	OK
ENSBTAG00000014365	ENSBTAG00000014365	7:11459645-11468452	0	0	0	OK
ENSBTAG00000014368	ENSBTAG00000014368	3:9272216-9293445	0	0	0	OK

ENSBTAG00000014370	ENSBTAG00000014370	18:15541253-15594951	0	0	0	OK
ENSBTAG00000014384	ENSBTAG00000014384	10:89399254-89405252	0	0	0	OK
ENSBTAG00000014397	ENSBTAG00000014397	X:23751992-23753633	0	0	0	OK
ENSBTAG00000014417	ENSBTAG00000014417	25:33157513-33168430	0	0	0	OK
ENSBTAG00000014418	ENSBTAG00000014418	22:41319550-42108622	0	0	0	OK
ENSBTAG00000014423	ENSBTAG00000014423	16:47992812-48000514	0	0	0	OK
ENSBTAG00000014435	ENSBTAG00000014435	23:27775677-27779418	0	0	0	OK
ENSBTAG00000014439	ENSBTAG00000014439	16:35975650-36045361	0	0	0	OK
ENSBTAG00000014448	ENSBTAG00000014448	25:28101589-28157501	0	0	0	OK
ENSBTAG00000014459	ENSBTAG00000014459	1:83918060-83925285	0	0	0	OK
ENSBTAG00000014476	ENSBTAG00000014476	21:55610773-55626668	0	0	0	OK
ENSBTAG00000014496	ENSBTAG00000014496	9:103460840-103461953	0	0	0	OK
ENSBTAG00000014501	ENSBTAG00000014501	10:65766163-65843942	0	0	0	OK
ENSBTAG00000014511	ENSBTAG00000014511	16:52642149-52651239	0	0	0	OK
ENSBTAG00000014532	ENSBTAG00000014532	4:107067494-107068361	0	0	0	OK
ENSBTAG00000014541	ENSBTAG00000014541	25:42573238-42589974	0	0	0	OK
ENSBTAG00000014546	ENSBTAG00000014546	5:100280954-100295523	0	0	0	OK
ENSBTAG00000014553	ENSBTAG00000014553	18:35165957-35205066	0	0	0	OK
ENSBTAG00000014556	ENSBTAG00000014556	18:35205884-35206635	0	0	0	OK
ENSBTAG00000014580	ENSBTAG00000014580	3:7881816-7886772	0	0	0	OK
ENSBTAG00000014585	ENSBTAG00000014585	18:54185870-54188079	0	0	0	OK
ENSBTAG00000014594	ENSBTAG00000014594	15:79873734-79874785	0	0	0	OK
ENSBTAG00000014606	ENSBTAG00000014606	15:30085142-30094889	0	0	0	OK
ENSBTAG00000014614	ENSBTAG00000014614	26:10662361-10714982	0	0	0.622347	OK
ENSBTAG00000014616	ENSBTAG00000014616	6:46251863-46286668	0	0	0	OK
ENSBTAG00000014619	ENSBTAG00000014619	12:33156436-33186087	0	0	0	OK
ENSBTAG00000014646	ENSBTAG00000014646	17:74392830-74397072	0	0	0	OK
ENSBTAG00000014657	ENSBTAG00000014657	10:72878883-72926592	0	0	0	OK
ENSBTAG00000014660	ENSBTAG00000014660	1:71697276-71709973	0	0	0	OK
ENSBTAG00000014666	ENSBTAG00000014666	22:59687611-59688166	0	0	0	OK

ENSBTAG00000014667	ENSBTAG00000014667	11:13393545-13397285	0	0	0	OK
ENSBTAG00000014668	ENSBTAG00000014668	11:13405926-13415101	0	0	0	OK
ENSBTAG00000014678	ENSBTAG00000014678	11:103301663-103306381	0	0	0	OK
ENSBTAG00000014685	ENSBTAG00000014685	X:18120060-18151262	0	0	0	OK
ENSBTAG00000014693	ENSBTAG00000014693	19:28156791-28157769	0	0	0	OK
ENSBTAG00000014695	ENSBTAG00000014695	19:28158381-28159607	0	0	0	OK
ENSBTAG00000014704	ENSBTAG00000014704	27:14445324-14447115	0	0	0	OK
ENSBTAG00000014707	ENSBTAG00000014707	16:52714626-52715665	0	0	0	OK
ENSBTAG00000014710	ENSBTAG00000014710	3:31746447-31764423	0	0	0	OK
ENSBTAG00000014712	ENSBTAG00000014712	7:19302912-19308138	0	0	0	OK
ENSBTAG00000014713	ENSBTAG00000014713	1:109661961-109704520	0	0	0	OK
ENSBTAG00000014718	ENSBTAG00000014718	29:44766935-44768173	0	0	0	OK
ENSBTAG00000014727	ENSBTAG00000014727	1:81043609-81057778	0	0	0	OK
ENSBTAG00000014744	ENSBTAG00000014744	7:47943545-47959145	0	0	0	OK
ENSBTAG00000014760	ENSBTAG00000014760	8:63509719-63510841	0	0	0	OK
ENSBTAG00000014762	ENSBTAG00000014762	21:20013718-20031094	0	0	0	OK
ENSBTAG00000014765	ENSBTAG00000014765	5:13756350-13756910	0	0	0	OK
ENSBTAG00000014772	ENSBTAG00000014772	7:77264727-77270660	0	0	0	OK
ENSBTAG00000014781	ENSBTAG00000014781	8:101049649-101050143	0	0	0	OK
ENSBTAG00000014783	ENSBTAG00000014783	2:96836603-96838606	0	0	0	OK
ENSBTAG00000014805	ENSBTAG00000014805	6:98990765-99029322	0	0	0	OK
ENSBTAG00000014831	ENSBTAG00000014831	26:13270025-13274922	0	0	0	OK
ENSBTAG00000014833	ENSBTAG00000014833	3:121271950-121274330	0	0	0	OK
ENSBTAG00000014853	ENSBTAG00000014853	15:49681261-49682212	0	0	0	OK
ENSBTAG00000014863	ENSBTAG00000014863	2:79255848-79304396	0	0	0	OK
ENSBTAG00000014869	ENSBTAG00000014869	13:63550490-63551256	0	0	0	OK
ENSBTAG00000014871	ENSBTAG00000014871	4:115629802-115631329	0	0	0	OK
ENSBTAG00000014878	ENSBTAG00000014878	18:46994990-46996544	0	0	0	OK
ENSBTAG00000014886	ENSBTAG00000014886	24:51117495-51135900	0	0	0	OK
ENSBTAG00000014916	ENSBTAG00000014916	7:2885095-2894691	0	0	0	OK

ENSBTAG00000014932	ENSBTAG00000014932	X:139018054-139033616	0	0	0	OK
ENSBTAG00000014934	ENSBTAG00000014934	8:62368708-62419901	0	0	0	OK
ENSBTAG00000014964	ENSBTAG00000014964	19:56465682-56472219	0	0	0	OK
ENSBTAG00000014970	ENSBTAG00000014970	3:33134981-33139308	0	0	0	OK
ENSBTAG00000014971	ENSBTAG00000014971	22:1445066-1451951	0	0	0	OK
ENSBTAG00000014977	ENSBTAG00000014977	5:14991696-15013533	0	0	0	OK
ENSBTAG00000014979	ENSBTAG00000014979	7:21594501-21604031	0	0	0	OK
ENSBTAG00000014982	ENSBTAG00000014982	7:21606601-21610676	0	0	0	OK
ENSBTAG00000014987	ENSBTAG00000014987	5:120779314-120780157	0	0	0	OK
ENSBTAG00000014988	ENSBTAG00000014988	2:120278332-120279307	0	0	0	OK
ENSBTAG00000014990	ENSBTAG00000014990	28:44123873-44125448	0	0	0	OK
ENSBTAG00000015001	ENSBTAG00000015001	18:46960040-46961903	0	0	0	OK
ENSBTAG00000015002	ENSBTAG00000015002	18:46961969-46963214	0	0	0	OK
ENSBTAG00000015009	ENSBTAG00000015009	25:36788649-36791965	0	0	0	OK
ENSBTAG00000015012	ENSBTAG00000015012	11:74480104-74483864	0	0	0	OK
ENSBTAG00000015033	ENSBTAG00000015033	9:298691-299576	0	0	0	OK
ENSBTAG00000015054	ENSBTAG00000015054	2:96826376-96828228	0	0	0	OK
ENSBTAG00000015056	ENSBTAG00000015056	1:130685148-130710118	0	0	0	OK
ENSBTAG00000015069	ENSBTAG00000015069	1:46355782-46370616	0	0	0	OK
ENSBTAG00000015074	ENSBTAG00000015074	11:106247576-106250629	0	0	0	OK
ENSBTAG00000015075	ENSBTAG00000015075	17:70305453-70316306	0	0	0	OK
ENSBTAG00000015081	ENSBTAG00000015081	11:106231443-106233013	0	0	0.536417	OK
ENSBTAG00000015084	ENSBTAG00000015084	18:24163232-24165538	0	0	0	OK
ENSBTAG00000015087	ENSBTAG00000015087	15:55380884-55387365	0	0	0	OK
ENSBTAG00000015092	ENSBTAG00000015092	5:110021364-110026384	0	0	0	OK
ENSBTAG00000015094	ENSBTAG00000015094	9:71833314-71851906	0	0	0	OK
ENSBTAG00000015098	ENSBTAG00000015098	11:96092485-96113995	0	0	0	OK
ENSBTAG00000015099	ENSBTAG00000015099	20:4626431-4632649	0	0	0	OK
ENSBTAG00000015100	ENSBTAG00000015100	20:4641617-4671634	0	0	0	OK
ENSBTAG00000015117	ENSBTAG00000015117	7:13721213-13723683	0	0	0	OK

ENSBTAG00000015128	ENSBTAG00000015128	18:57431503-57436686	0	0	0	OK
ENSBTAG00000015143	ENSBTAG00000015143	21:45486925-45487620	0	0	0	OK
ENSBTAG00000015145	ENSBTAG00000015145	3:18768795-18770416	0	0	0	OK
ENSBTAG00000015147	ENSBTAG00000015147	3:18799611-18810545	0	0	0	OK
ENSBTAG00000015156	ENSBTAG00000015156	21:57388582-57395511	0	0	0	OK
ENSBTAG00000015160	ENSBTAG00000015160	21:70999272-71004734	0	0	0	OK
ENSBTAG00000015169	ENSBTAG00000015169	2:61919642-61949692	0	0	0	OK
ENSBTAG00000015193	ENSBTAG00000015193	11:10489731-10497683	0	0	0	OK
ENSBTAG00000015204	ENSBTAG00000015204	X:128771953-128825159	0	0	0	OK
ENSBTAG00000015209	ENSBTAG00000015209	6:108490180-108501580	0	0	0	OK
ENSBTAG00000015229	ENSBTAG00000015229	14:32050487-32143515	0	0	0	OK
ENSBTAG00000015260	ENSBTAG00000015260	18:49578802-49580639	0	0	0	OK
ENSBTAG00000015265	ENSBTAG00000015265	6:37117549-37119127	0	0	0	OK
ENSBTAG00000015274	ENSBTAG00000015274	23:35105134-35113759	0	0	0	OK
ENSBTAG00000015283	ENSBTAG00000015283	22:56985248-56989012	0	0	0	OK
ENSBTAG00000015285	ENSBTAG00000015285	3:101816843-101818956	0	0	0	OK
ENSBTAG00000015299	ENSBTAG00000015299	19:50827279-50828335	0	0	0	OK
ENSBTAG00000015301	ENSBTAG00000015301	23:15930028-15941569	0	0	0	OK
ENSBTAG00000015347	ENSBTAG00000015347	5:31013103-31014446	0	0	0	OK
ENSBTAG00000015351	ENSBTAG00000015351	5:99407005-99439453	0	0	0	OK
ENSBTAG00000015364	ENSBTAG00000015364	5:31000182-31003266	0	0	0	OK
ENSBTAG00000015369	ENSBTAG00000015369	3:19762895-19767967	0	0	0	OK
ENSBTAG00000015380	ENSBTAG00000015380	3:33152577-33183100	0	0	0	OK
ENSBTAG00000015387	ENSBTAG00000015387	19:50579226-50604298	0	0	0	OK
ENSBTAG00000015388	ENSBTAG00000015388	18:55710192-55713956	0	0	0	OK
ENSBTAG00000015394	ENSBTAG00000015394	19:50537803-50546687	0	0	0	OK
ENSBTAG00000015395	ENSBTAG00000015395	5:45728875-45729747	0	0	0	OK
ENSBTAG00000015396	ENSBTAG00000015396	23:39835981-39846198	0	0	0	OK
ENSBTAG00000015407	ENSBTAG00000015407	5:45721766-45727180	0	0	0	OK
ENSBTAG00000015409	ENSBTAG00000015409	6:105515159-105531715	0	0	0	OK

ENSBTAG00000015419	ENSBTAG00000015419	7:62923975-62978142	0	0	0	OK
ENSBTAG00000015427	ENSBTAG00000015427	18:2662462-2766583	0	0	0	OK
ENSBTAG00000015428	ENSBTAG00000015428	18:2845659-2858813	0	0	0	OK
ENSBTAG00000015439	ENSBTAG00000015439	7:53613733-53616916	0	0	0	OK
ENSBTAG00000015449	ENSBTAG00000015449	6:92450702-92479053	0	0	0	OK
ENSBTAG00000015465	ENSBTAG00000015465	7:61133265-61145126	0	0	0	OK
ENSBTAG00000015473	ENSBTAG00000015473	11:37823445-37825428	0	0	0	OK
ENSBTAG00000015483	ENSBTAG00000015483	22:12678579-12679638	0	0	0	OK
ENSBTAG00000015493	ENSBTAG00000015493	22:12681778-12688177	0	0	0	OK
ENSBTAG00000015504	ENSBTAG00000015504	20:31571709-31578258	0	0	0	OK
ENSBTAG00000015510	ENSBTAG00000015510	4:106989815-106993917	0	0	0	OK
ENSBTAG00000015529	ENSBTAG00000015529	22:58747918-58754568	0	0	0	OK
ENSBTAG00000015538	ENSBTAG00000015538	23:7841999-7858371	0	0	0	OK
ENSBTAG00000015550	ENSBTAG00000015550	23:7865123-7872669	0	0	0	OK
ENSBTAG00000015551	ENSBTAG00000015551	4:100280751-100281437	0	0	0	OK
ENSBTAG00000015556	ENSBTAG00000015556	22:32583220-32607984	0	0	0	OK
ENSBTAG00000015574	ENSBTAG00000015574	16:44427128-44447695	0	0	0	OK
ENSBTAG00000015576	ENSBTAG00000015576	18:54516898-54532816	0	0	0	OK
ENSBTAG00000015587	ENSBTAG00000015587	11:78968979-78975466	0	0	0	OK
ENSBTAG00000015595	ENSBTAG00000015595	5:73990686-74009154	0	0	0	OK
ENSBTAG00000015602	ENSBTAG00000015602	7:1317087-1334619	0	0	0.295796	OK
ENSBTAG00000015606	ENSBTAG00000015606	16:53641325-53773013	0	0	0	OK
ENSBTAG00000015608	ENSBTAG00000015608	8:94205190-94210057	0	0	0	OK
ENSBTAG00000015614	ENSBTAG00000015614	12:32604930-32646201	0	0	0	OK
ENSBTAG00000015618	ENSBTAG00000015618	7:4711419-4713394	0	0	0	OK
ENSBTAG00000015628	ENSBTAG00000015628	15:47267702-47272165	0	0	0	OK
ENSBTAG00000015649	ENSBTAG00000015649	6:59823315-59866460	0	0	0	OK
ENSBTAG00000015678	ENSBTAG00000015678	2:5719427-5744015	0	0	0	OK
ENSBTAG00000015685	ENSBTAG00000015685	8:105033047-105040479	0	0	0	OK
ENSBTAG00000015686	ENSBTAG00000015686	18:2976671-2983194	0	0	0	OK

ENSBTAG00000015719	ENSBTAG00000015719	11:28871451-28902847	0	0	0	OK
ENSBTAG00000015727	ENSBTAG00000015727	7:41823771-41840054	0	0	0	OK
ENSBTAG00000015728	ENSBTAG00000015728	1:37960748-38021040	0	0	0	OK
ENSBTAG00000015731	ENSBTAG00000015731	24:56404066-56446678	0	0	0	OK
ENSBTAG00000015734	ENSBTAG00000015734	14:78559843-78593622	0	0	0	OK
ENSBTAG00000015741	ENSBTAG00000015741	3:9371594-9372444	0	0	0	OK
ENSBTAG00000015745	ENSBTAG00000015745	29:41112114-41130629	0	0	0	OK
ENSBTAG00000015749	ENSBTAG00000015749	4:74836425-74849155	0	0	0	OK
ENSBTAG00000015763	ENSBTAG00000015763	19:24809408-24824295	0	0	0	OK
ENSBTAG00000015780	ENSBTAG00000015780	21:32577839-32595997	0	0	0	OK
ENSBTAG00000015784	ENSBTAG00000015784	3:14254485-14259736	0	0	0	OK
ENSBTAG00000015793	ENSBTAG00000015793	29:7560394-7625766	0	0	0	OK
ENSBTAG00000015796	ENSBTAG00000015796	13:54382946-54408528	0	0	0	OK
ENSBTAG00000015797	ENSBTAG00000015797	10:87591789-87592726	0	0	0	OK
ENSBTAG00000015812	ENSBTAG00000015812	1:4059170-4059362	0	0	0	OK
ENSBTAG00000015824	ENSBTAG00000015824	15:42719432-42753584	0	0	0	OK
ENSBTAG00000015829	ENSBTAG00000015829	23:19388893-19395134	0	0	0	OK
ENSBTAG00000015836	ENSBTAG00000015836	18:53302938-53313115	0	0	0	OK
ENSBTAG00000015840	ENSBTAG00000015840	2:20759889-20761196	0	0	0	OK
ENSBTAG00000015844	ENSBTAG00000015844	4:11035478-11039782	0	0	0	OK
ENSBTAG00000015880	ENSBTAG00000015880	21:58949072-59007741	0	0	0	OK
ENSBTAG00000015892	ENSBTAG00000015892	1:65922481-65928617	0	0	0	OK
ENSBTAG00000015897	ENSBTAG00000015897	22:1310942-1311505	0	0	0	OK
ENSBTAG00000015907	ENSBTAG00000015907	20:70929225-70933190	0	0	0	OK
ENSBTAG00000015925	ENSBTAG00000015925	4:14364654-14452240	0	0	0	OK
ENSBTAG00000015929	ENSBTAG00000015929	11:105147823-105148760	0	0	0	OK
ENSBTAG00000015939	ENSBTAG00000015939	15:30743666-30758246	0	0	0	OK
ENSBTAG00000015944	ENSBTAG00000015944	21:31196239-31215312	0	0	0	OK
ENSBTAG00000015952	ENSBTAG00000015952	25:42330025-42362918	0	0	0	OK
ENSBTAG00000015953	ENSBTAG00000015953	7:23018545-23020546	0	0	0	OK

ENSBTAG00000015957	ENSBTAG00000015957	7:22993178-23001067	0	0	0	OK
ENSBTAG00000015965	ENSBTAG00000015965	11:154023-181573	0	0	0	OK
ENSBTAG00000015972	ENSBTAG00000015972	16:12762909-12783624	0	0	0	OK
ENSBTAG00000015974	ENSBTAG00000015974	16:72570591-72596973	0	0	0	OK
ENSBTAG00000016019	ENSBTAG00000016019	X:48844284-48844653	0	0	0	OK
ENSBTAG00000016033	ENSBTAG00000016033	2:20824234-20825910	0	0	0	OK
ENSBTAG00000016043	ENSBTAG00000016043	5:103964865-103971313	0	0	0	OK
ENSBTAG00000016057	ENSBTAG00000016057	16:49332769-49353517	0	0	0	OK
ENSBTAG00000016059	ENSBTAG00000016059	17:38326305-38327234	0	0	0	OK
ENSBTAG00000016069	ENSBTAG00000016069	22:44150603-44169288	0	0	0	OK
ENSBTAG00000016073	ENSBTAG00000016073	8:102987420-103024296	0	0	0	OK
ENSBTAG00000016093	ENSBTAG00000016093	X:92294751-92297791	0	0	0	OK
ENSBTAG00000016098	ENSBTAG00000016098	22:16769772-16777323	0	0	0	OK
ENSBTAG00000016101	ENSBTAG00000016101	22:16760054-16764930	0	0	0	OK
ENSBTAG00000016114	ENSBTAG00000016114	18:55294575-55303680	0	0	0	OK
ENSBTAG00000016116	ENSBTAG00000016116	12:33647814-33648405	0	0	0	OK
ENSBTAG00000016119	ENSBTAG00000016119	15:48240560-48241508	0	0	0	OK
ENSBTAG00000016146	ENSBTAG00000016146	15:81091986-81092922	0	0	0	OK
ENSBTAG00000016147	ENSBTAG00000016147	17:35347483-35351627	0	0	0	OK
ENSBTAG00000016148	ENSBTAG00000016148	11:106405781-106411025	0	0	0	OK
ENSBTAG00000016153	ENSBTAG00000016153	22:52155117-52157001	0	0	0	OK
ENSBTAG00000016159	ENSBTAG00000016159	14:23263814-23264810	0	0	0	OK
ENSBTAG00000016163	ENSBTAG00000016163	17:71434467-71437372	0	0	0	OK
ENSBTAG00000016171	ENSBTAG00000016171	13:61745577-61750870	0	0	0	OK
ENSBTAG00000016177	ENSBTAG00000016177	27:18454361-18472436	0	0	0	OK
ENSBTAG00000016183	ENSBTAG00000016183	2:104782833-104808656	0	0	0	OK
ENSBTAG00000016192	ENSBTAG00000016192	2:95475473-95499166	0	0	0	OK
ENSBTAG00000016209	ENSBTAG00000016209	14:2834780-2835985	0	0	0	OK
ENSBTAG00000016210	ENSBTAG00000016210	14:2829284-2831334	0	0	0	OK
ENSBTAG00000016216	ENSBTAG00000016216	20:5409906-5410676	0	0	0	OK

ENSBTAG00000016224	ENSBTAG00000016224	8:112896492-112901718	0	0	0	OK
ENSBTAG00000016227	ENSBTAG00000016227	5:67070163-67070823	0	0	0	OK
ENSBTAG00000016259	ENSBTAG00000016259	8:103577523-103643619	0	0	0	OK
ENSBTAG00000016269	ENSBTAG00000016269	24:50870261-50928290	0	0	0	OK
ENSBTAG00000016270	ENSBTAG00000016270	11:78507371-78514602	0	0	0	OK
ENSBTAG00000016271	ENSBTAG00000016271	5:60556050-60564297	0	0	0	OK
ENSBTAG00000016278	ENSBTAG00000016278	14:68467562-68470952	0	0	0	OK
ENSBTAG00000016284	ENSBTAG00000016284	14:44097763-44187670	0	0	0	OK
ENSBTAG00000016285	ENSBTAG00000016285	10:89533856-89537697	0	0	0	OK
ENSBTAG00000016290	ENSBTAG00000016290	6:87976519-88030195	0	0	0	OK
ENSBTAG00000016291	ENSBTAG00000016291	10:36523868-36525354	0	0	0	OK
ENSBTAG00000016306	ENSBTAG00000016306	1:133654481-133655032	0	0	0	OK
ENSBTAG00000016311	ENSBTAG00000016311	10:79600114-79620580	0	0	0	OK
ENSBTAG00000016320	ENSBTAG00000016320	15:44880075-44917824	0	0	0	OK
ENSBTAG00000016330	ENSBTAG00000016330	25:3856224-3886874	0	0	0	OK
ENSBTAG00000016335	ENSBTAG00000016335	26:22385931-22388123	0	0	0	OK
ENSBTAG00000016337	ENSBTAG00000016337	23:32835595-32849025	0	0	0	OK
ENSBTAG00000016344	ENSBTAG00000016344	19:28913866-28961787	0	0	0	OK
ENSBTAG00000016347	ENSBTAG00000016347	16:41137642-41138515	0	0	0	OK
ENSBTAG00000016357	ENSBTAG00000016357	9:71884620-71913826	0	0	0	OK
ENSBTAG00000016360	ENSBTAG00000016360	25:9519175-9524548	0	0	0	OK
ENSBTAG00000016362	ENSBTAG00000016362	5:28743169-28758751	0	0	0	OK
ENSBTAG00000016363	ENSBTAG00000016363	1:45489051-45521372	0	0	0	OK
ENSBTAG00000016367	ENSBTAG00000016367	11:93101472-93121906	0	0	0	OK
ENSBTAG00000016381	ENSBTAG00000016381	10:21779557-21783181	0	0	0	OK
ENSBTAG00000016384	ENSBTAG00000016384	19:42301510-42309720	0	0	0	OK
ENSBTAG00000016391	ENSBTAG00000016391	9:16325797-16326091	0	0	0	OK
ENSBTAG00000016432	ENSBTAG00000016432	19:35389674-35420462	0	0	0	OK
ENSBTAG00000016465	ENSBTAG00000016465	29:48930323-48950644	0	0	0	OK
ENSBTAG00000016477	ENSBTAG00000016477	7:22569682-22574912	0	0	0	OK

ENSBTAG00000016483	ENSBTAG00000016483	X:8047269-8048341	0	0	0	OK
ENSBTAG00000016494	ENSBTAG00000016494	15:53663175-53669426	0	0	0	OK
ENSBTAG00000016498	ENSBTAG00000016498	9:82244597-82330643	0	0	0	OK
ENSBTAG00000016501	ENSBTAG00000016501	15:77496429-77513093	0	0	0	OK
ENSBTAG00000016504	ENSBTAG00000016504	3:115843769-116226449	0	0	0	OK
ENSBTAG00000016510	ENSBTAG00000016510	5:113971401-113979372	0	0	0	OK
ENSBTAG00000016520	ENSBTAG00000016520	25:987389-989247	0	0	0	OK
ENSBTAG00000016548	ENSBTAG00000016548	15:22829845-22834357	0	0	0	OK
ENSBTAG00000016552	ENSBTAG00000016552	25:1348414-1349600	0	0	0	OK
ENSBTAG00000016555	ENSBTAG00000016555	29:43149609-43152143	0	0	0.774527	OK
ENSBTAG00000016574	ENSBTAG00000016574	25:292323-299280	0	0	0	OK
ENSBTAG00000016595	ENSBTAG00000016595	7:53047809-53118491	0	0	0	OK
ENSBTAG00000016602	ENSBTAG00000016602	11:10120951-10122607	0	0	0	OK
ENSBTAG00000016603	ENSBTAG00000016603	21:68569748-68585001	0	0	0	OK
ENSBTAG00000016607	ENSBTAG00000016607	11:10105100-10106503	0	0	0	OK
ENSBTAG00000016620	ENSBTAG00000016620	24:46018901-46034227	0	0	0	OK
ENSBTAG00000016648	ENSBTAG00000016648	7:44816939-44823914	0	0	0	OK
ENSBTAG00000016664	ENSBTAG00000016664	13:54502133-54505099	0	0	0	OK
ENSBTAG00000016677	ENSBTAG00000016677	13:31280683-31287466	0	0	0	OK
ENSBTAG00000016679	ENSBTAG00000016679	17:41262753-41307126	0	0	0	OK
ENSBTAG00000016688	ENSBTAG00000016688	X:66722398-66723397	0	0	0	OK
ENSBTAG00000016695	ENSBTAG00000016695	18:10500703-10513342	0	0	0	OK
ENSBTAG00000016705	ENSBTAG00000016705	3:28865271-28879550	0	0	0	OK
ENSBTAG00000016713	ENSBTAG00000016713	10:73648526-73649642	0	0	0	OK
ENSBTAG00000016724	ENSBTAG00000016724	13:58207244-58222951	0	0	0	OK
ENSBTAG00000016733	ENSBTAG00000016733	16:47678490-47693841	0	0	0	OK
ENSBTAG00000016750	ENSBTAG00000016750	8:103339337-103383299	0	0	0	OK
ENSBTAG00000016774	ENSBTAG00000016774	29:46994503-46995463	0	0	0	OK
ENSBTAG00000016789	ENSBTAG00000016789	7:55024472-55025390	0	0	0	OK
ENSBTAG00000016794	ENSBTAG00000016794	11:5786451-5802753	0	0	0	OK

ENSBTAG00000016819	ENSBTAG00000016819	2:122723224-122783830	0	0	0	OK
ENSBTAG00000016822	ENSBTAG00000016822	10:45874977-45880918	0	0	0	OK
ENSBTAG00000016833	ENSBTAG00000016833	23:9723790-9726250	0	0	0	OK
ENSBTAG00000016876	ENSBTAG00000016876	5:89341931-89342981	0	0	0	OK
ENSBTAG00000016877	ENSBTAG00000016877	2:133088082-133100875	0	0	0	OK
ENSBTAG00000016899	ENSBTAG00000016899	3:103212682-103217887	0	0	0	OK
ENSBTAG00000016902	ENSBTAG00000016902	20:13805927-13851473	0	0	0	OK
ENSBTAG00000016926	ENSBTAG00000016926	13:58709491-58725285	0	0	0	OK
ENSBTAG00000016939	ENSBTAG00000016939	11:68446368-68485438	0	0	0	OK
ENSBTAG00000016977	ENSBTAG00000016977	X:38815312-38838616	0	0	0.411644	OK
ENSBTAG00000016988	ENSBTAG00000016988	7:15532997-15545250	0	0	0	OK
ENSBTAG00000016989	ENSBTAG00000016989	X:148576704-148582356	0	0	0	OK
ENSBTAG00000016995	ENSBTAG00000016995	5:109404796-109430265	0	0	0	OK
ENSBTAG00000016996	ENSBTAG00000016996	5:31713905-31738308	0	0	0	OK
ENSBTAG00000016997	ENSBTAG00000016997	18:56740608-56746100	0	0	0	OK
ENSBTAG00000016998	ENSBTAG00000016998	12:19275073-19276925	0	0	0	OK
ENSBTAG00000017000	ENSBTAG00000017000	9:76064701-76079984	0	0	0	OK
ENSBTAG00000017005	ENSBTAG00000017005	11:106391055-106393576	0	0	0	OK
ENSBTAG00000017009	ENSBTAG00000017009	18:7719671-7733832	0	0	0	OK
ENSBTAG00000017017	ENSBTAG00000017017	X:134046107-134155813	0	0	0	OK
ENSBTAG00000017020	ENSBTAG00000017020	X:134101038-134104560	0	0	0	OK
ENSBTAG00000017026	ENSBTAG00000017026	20:18563738-18654726	0	0	0	OK
ENSBTAG00000017035	ENSBTAG00000017035	18:46164775-46174400	0	0	0	OK
ENSBTAG00000017040	ENSBTAG00000017040	14:2624757-2628462	0	0	0	OK
ENSBTAG00000017053	ENSBTAG00000017053	28:492422-525181	0	0	0	OK
ENSBTAG00000017062	ENSBTAG00000017062	5:119998126-119999276	0	0	0	OK
ENSBTAG00000017073	ENSBTAG00000017073	6:31710449-31727197	0	0	0	OK
ENSBTAG00000017081	ENSBTAG00000017081	19:34984927-34986841	0	0	0	OK
ENSBTAG00000017096	ENSBTAG00000017096	21:60464153-60489125	0	0	0	OK
ENSBTAG00000017102	ENSBTAG00000017102	22:14944372-14944971	0	0	0	OK

ENSBTAG00000017113	ENSBTAG00000017113	5:28129659-28132288	0	0	0	OK
ENSBTAG00000017118	ENSBTAG00000017118	23:39611983-39619623	0	0	0	OK
ENSBTAG00000017131	ENSBTAG00000017131	6:90258975-90280522	0	0	0	OK
ENSBTAG00000017137	ENSBTAG00000017137	8:40073250-40092028	0	0	0	OK
ENSBTAG00000017150	ENSBTAG00000017150	15:65991131-66030964	0	0	0	OK
ENSBTAG00000017167	ENSBTAG00000017167	17:2652179-2657479	0	0	0	OK
ENSBTAG00000017200	ENSBTAG00000017200	10:75783881-75877395	0	0	0	OK
ENSBTAG00000017201	ENSBTAG00000017201	21:42070331-42117187	0	0	0	OK
ENSBTAG00000017205	ENSBTAG00000017205	9:38847223-38856926	0	0	0	OK
ENSBTAG00000017212	ENSBTAG00000017212	21:55974567-55975823	0	0	0	OK
ENSBTAG00000017214	ENSBTAG00000017214	2:5512931-5514784	0	0	0	OK
ENSBTAG00000017227	ENSBTAG00000017227	23:28921928-28922867	0	0	0	OK
ENSBTAG00000017241	ENSBTAG00000017241	X:81297833-81298336	0	0	0	OK
ENSBTAG00000017246	ENSBTAG00000017246	19:33853787-33855685	0	0	0	OK
ENSBTAG00000017249	ENSBTAG00000017249	14:38554722-38585200	0	0	0	OK
ENSBTAG00000017252	ENSBTAG00000017252	19:57122384-57126123	0	0	0	OK
ENSBTAG00000017253	ENSBTAG00000017253	2:124913877-124949961	0	0	0	OK
ENSBTAG00000017256	ENSBTAG00000017256	3:26584422-26598906	0	0	0	OK
ENSBTAG00000017267	ENSBTAG00000017267	21:34156311-34167331	0	0	0	OK
ENSBTAG00000017273	ENSBTAG00000017273	7:4704032-4708458	0	0	0	OK
ENSBTAG00000017279	ENSBTAG00000017279	24:42332693-42368659	0	0	0	OK
ENSBTAG00000017294	ENSBTAG00000017294	8:105216968-105220438	0	0	0	OK
ENSBTAG00000017298	ENSBTAG00000017298	29:18250501-18284062	0	0	0	OK
ENSBTAG00000017301	ENSBTAG00000017301	16:3726353-3731874	0	0	0	OK
ENSBTAG00000017305	ENSBTAG00000017305	17:73920710-73935352	0	0	0	OK
ENSBTAG00000017310	ENSBTAG00000017310	1:2259114-2360138	0	0	0	OK
ENSBTAG00000017312	ENSBTAG00000017312	1:80250204-80251648	0	0	0	OK
ENSBTAG00000017318	ENSBTAG00000017318	11:21988494-22046009	0	0	0	OK
ENSBTAG00000017330	ENSBTAG00000017330	11:73186221-73186856	0	0	0	OK
ENSBTAG00000017343	ENSBTAG00000017343	14:47263661-47281305	0	0	0	OK

ENSBTAG00000017345	ENSBTAG00000017345	19:3876961-3879198	0	0	0	OK
ENSBTAG00000017348	ENSBTAG00000017348	6:96723381-96746176	0	0	0	OK
ENSBTAG00000017375	ENSBTAG00000017375	6:14515393-14565920	0	0	0	OK
ENSBTAG00000017385	ENSBTAG00000017385	5:63208484-63209321	0	0	0	OK
ENSBTAG00000017391	ENSBTAG00000017391	25:20685204-20800341	0	0	0	OK
ENSBTAG00000017430	ENSBTAG00000017430	X:39805524-39808817	0	0	0	OK
ENSBTAG00000017431	ENSBTAG00000017431	2:119524746-119525703	0	0	0	OK
ENSBTAG00000017436	ENSBTAG00000017436	2:90461665-90585163	0	0	0	OK
ENSBTAG00000017442	ENSBTAG00000017442	10:4598735-4606922	0	0	0	OK
ENSBTAG00000017452	ENSBTAG00000017452	2:128984353-129018364	0	0	0	OK
ENSBTAG00000017460	ENSBTAG00000017460	11:37859123-37861215	0	0	0	OK
ENSBTAG00000017463	ENSBTAG00000017463	5:111467543-111471171	0	0	0	OK
ENSBTAG00000017466	ENSBTAG00000017466	10:19980211-20021927	0	0	0	OK
ENSBTAG00000017481	ENSBTAG00000017481	7:35557617-35557964	0	0	0	OK
ENSBTAG00000017482	ENSBTAG00000017482	7:4669452-4672648	0	0	0	OK
ENSBTAG00000017496	ENSBTAG00000017496	6:108851270-108852813	0	0	0	OK
ENSBTAG00000017500	ENSBTAG00000017500	11:29759819-29808312	0	0	0	OK
ENSBTAG00000017502	ENSBTAG00000017502	3:104370158-104404495	0	0	0	OK
ENSBTAG00000017521	ENSBTAG00000017521	28:24845409-24845868	0	0	0	OK
ENSBTAG00000017524	ENSBTAG00000017524	13:84024136-84031205	0	0	0	OK
ENSBTAG00000017528	ENSBTAG00000017528	18:13958994-13964622	0	0	0	OK
ENSBTAG00000017530	ENSBTAG00000017530	8:39532017-39568029	0	0	0	OK
ENSBTAG00000017533	ENSBTAG00000017533	23:6530408-6580289	0	0	0	OK
ENSBTAG00000017542	ENSBTAG00000017542	23:9340954-9353750	0	0	0	OK
ENSBTAG00000017543	ENSBTAG00000017543	5:55842228-55864827	0	0	0	OK
ENSBTAG00000017547	ENSBTAG00000017547	22:357511-370121	0	0	0	OK
ENSBTAG00000017557	ENSBTAG00000017557	9:43793888-43832666	0	0	0	OK
ENSBTAG00000017568	ENSBTAG00000017568	13:75195343-75199068	0	0	0	OK
ENSBTAG00000017587	ENSBTAG00000017587	13:75168820-75170108	0	0	0	OK
ENSBTAG00000017595	ENSBTAG00000017595	3:57105059-57144607	0	0	0	OK

ENSBTAG00000017615	ENSBTAG00000017615	3:117645911-117646680	0	0	0	OK
ENSBTAG00000017629	ENSBTAG00000017629	2:58552536-58555808	0	0	0	OK
ENSBTAG00000017633	ENSBTAG00000017633	X:130307268-130317159	0	0	0	OK
ENSBTAG00000017648	ENSBTAG00000017648	7:48524555-48525038	0	0	0	OK
ENSBTAG00000017660	ENSBTAG00000017660	X:91260327-91261416	0	0	0	OK
ENSBTAG00000017664	ENSBTAG00000017664	4:39116166-39197870	0	0	0	OK
ENSBTAG00000017665	ENSBTAG00000017665	10:20922259-20934454	0	0	0	OK
ENSBTAG00000017677	ENSBTAG00000017677	10:58748956-58787385	0	0	0	OK
ENSBTAG00000017691	ENSBTAG00000017691	16:57861755-57862145	0	0	0	OK
ENSBTAG00000017697	ENSBTAG00000017697	25:27222529-27226056	0	0	0	OK
ENSBTAG00000017698	ENSBTAG00000017698	24:30986485-31007475	0	0	0	OK
ENSBTAG00000017733	ENSBTAG00000017733	14:79372711-79388600	0	0	0	OK
ENSBTAG00000017744	ENSBTAG00000017744	7:39268353-39337797	0	0	0	OK
ENSBTAG00000017752	ENSBTAG00000017752	18:7356787-7366900	0	0	0	OK
ENSBTAG00000017762	ENSBTAG00000017762	18:54195284-54195883	0	0	0	OK
ENSBTAG00000017763	ENSBTAG00000017763	8:87891325-87893182	0	0	0	OK
ENSBTAG00000017780	ENSBTAG00000017780	26:51520774-51535696	0	0	0	OK
ENSBTAG00000017786	ENSBTAG00000017786	3:9991098-10000540	0	0	0	OK
ENSBTAG00000017794	ENSBTAG00000017794	15:30302981-30307214	0	0	0	OK
ENSBTAG00000017798	ENSBTAG00000017798	10:81521059-81533819	0	0	0	OK
ENSBTAG00000017802	ENSBTAG00000017802	27:41695664-41771738	0	0	0	OK
ENSBTAG00000017803	ENSBTAG00000017803	8:112502333-112512347	0	0	0	OK
ENSBTAG00000017805	ENSBTAG00000017805	29:50710075-50739409	0	0	0	OK
ENSBTAG00000017815	ENSBTAG00000017815	19:29278756-29489242	0	0	0	OK
ENSBTAG00000017821	ENSBTAG00000017821	7:28185888-28186394	0	0	0	OK
ENSBTAG00000017856	ENSBTAG00000017856	3:8461936-8463395	0	0	0	OK
ENSBTAG00000017871	ENSBTAG00000017871	25:41099258-41171209	0	0	0	OK
ENSBTAG00000017882	ENSBTAG00000017882	1:145885349-145887681	0	0	0	OK
ENSBTAG00000017883	ENSBTAG00000017883	16:55611220-55645394	0	0	0	OK
ENSBTAG00000017885	ENSBTAG00000017885	20:63111707-63127170	0	0	0	OK

ENSBTAG00000017892	ENSBTAG00000017892	3:106900110-106914177	0	0	0	OK
ENSBTAG00000017901	ENSBTAG00000017901	15:22174762-22198030	0	0	0	OK
ENSBTAG00000017912	ENSBTAG00000017912	7:14915033-14915996	0	0	0	OK
ENSBTAG00000017913	ENSBTAG00000017913	26:43276922-43288958	0	0	0	OK
ENSBTAG00000017916	ENSBTAG00000017916	8:23065725-23066197	0	0	0	OK
ENSBTAG00000017924	ENSBTAG00000017924	21:50322854-50324578	0	0	0	OK
ENSBTAG00000017925	ENSBTAG00000017925	5:120016888-120020570	0	0	0	OK
ENSBTAG00000017931	ENSBTAG00000017931	X:127720573-127720884	0	0	0	OK
ENSBTAG00000017945	ENSBTAG00000017945	X:137046592-137057690	0	0	0	OK
ENSBTAG00000017946	ENSBTAG00000017946	28:41921826-41924801	0	0	0	OK
ENSBTAG00000017948	ENSBTAG00000017948	X:102213441-102214191	0	0	0	OK
ENSBTAG00000017949	ENSBTAG00000017949	23:50550186-50568522	0	0	0	OK
ENSBTAG00000017953	ENSBTAG00000017953	11:47717009-47717450	0	0	0	OK
ENSBTAG00000017957	ENSBTAG00000017957	7:52295317-52300482	0	0	0	OK
ENSBTAG00000017967	ENSBTAG00000017967	5:57091366-57112468	0	0	0	OK
ENSBTAG00000017978	ENSBTAG00000017978	X:63347689-63348061	0	0	0	OK
ENSBTAG00000017980	ENSBTAG00000017980	11:101158277-101158682	0	0	0	OK
ENSBTAG00000017982	ENSBTAG00000017982	11:101169689-101200648	0	0	0	OK
ENSBTAG00000018024	ENSBTAG00000018024	27:41866519-41883521	0	0	0	OK
ENSBTAG00000018039	ENSBTAG00000018039	10:95430415-95431403	0	0	0	OK
ENSBTAG00000018048	ENSBTAG00000018048	17:74425041-74490537	0	0	0	OK
ENSBTAG00000018054	ENSBTAG00000018054	11:47787638-47795270	0	0	0	OK
ENSBTAG00000018058	ENSBTAG00000018058	3:113208932-113216709	0	0	0	OK
ENSBTAG00000018073	ENSBTAG00000018073	5:114596005-114608640	0	0	0	OK
ENSBTAG00000018082	ENSBTAG00000018082	25:26747354-26762056	0	0	0	OK
ENSBTAG00000018127	ENSBTAG00000018127	10:72757581-72768778	0	0	0	OK
ENSBTAG00000018134	ENSBTAG00000018134	6:91126255-91138391	0	0	0	OK
ENSBTAG00000018155	ENSBTAG00000018155	6:100006744-100011712	0	0	0	OK
ENSBTAG00000018182	ENSBTAG00000018182	15:48794813-48795755	0	0	0	OK
ENSBTAG00000018188	ENSBTAG00000018188	29:18074820-18083006	0	0	0	OK

ENSBTAG00000018195	ENSBTAG00000018195	25:20622351-20623126	0	0	0	OK
ENSBTAG00000018200	ENSBTAG00000018200	17:16347924-16363886	0	0	0	OK
ENSBTAG00000018216	ENSBTAG00000018216	24:50444720-50460419	0	0	0	OK
ENSBTAG00000018232	ENSBTAG00000018232	12:23294962-23308189	0	0	0	OK
ENSBTAG00000018244	ENSBTAG00000018244	18:1339477-1342619	0	0	0	OK
ENSBTAG00000018246	ENSBTAG00000018246	15:17760028-17798653	0	0	0	OK
ENSBTAG00000018250	ENSBTAG00000018250	25:16793081-16813965	0	0	0	OK
ENSBTAG00000018257	ENSBTAG00000018257	15:66207586-66231698	0	0	0	OK
ENSBTAG00000018259	ENSBTAG00000018259	15:84061204-84062140	0	0	0	OK
ENSBTAG00000018274	ENSBTAG00000018274	29:48027925-48030331	0	0	0	OK
ENSBTAG00000018275	ENSBTAG00000018275	2:5015010-5016211	0	0	0	OK
ENSBTAG00000018278	ENSBTAG00000018278	1:922634-929992	0	0	0	OK
ENSBTAG00000018281	ENSBTAG00000018281	3:94323975-94331673	0	0	0	OK
ENSBTAG00000018282	ENSBTAG00000018282	2:89948474-89963535	0	0	0	OK
ENSBTAG00000018283	ENSBTAG00000018283	22:11619694-11643912	0	0	0	OK
ENSBTAG00000018290	ENSBTAG00000018290	7:48919354-48923271	0	0	0	OK
ENSBTAG00000018312	ENSBTAG00000018312	1:104146630-104147374	0	0	0	OK
ENSBTAG00000018323	ENSBTAG00000018323	X:21831080-21969960	0	0	0	OK
ENSBTAG00000018326	ENSBTAG00000018326	18:46057591-46066618	0	0	0	OK
ENSBTAG00000018332	ENSBTAG00000018332	17:56659324-56700484	0	0	0	OK
ENSBTAG00000018356	ENSBTAG00000018356	28:42111713-42119577	0	0	0	OK
ENSBTAG00000018385	ENSBTAG00000018385	2:125733244-125757519	0	0	0	OK
ENSBTAG00000018413	ENSBTAG00000018413	2:126799590-126807056	0	0	0	OK
ENSBTAG00000018416	ENSBTAG00000018416	19:28873594-28886401	0	0	0	OK
ENSBTAG00000018420	ENSBTAG00000018420	19:49312497-49328600	0	0	0	OK
ENSBTAG00000018442	ENSBTAG00000018442	4:105909791-105915720	0	0	0	OK
ENSBTAG00000018446	ENSBTAG00000018446	2:34173877-34193022	0	0	0	OK
ENSBTAG00000018448	ENSBTAG00000018448	3:69221073-69302542	0	0	0	OK
ENSBTAG00000018452	ENSBTAG00000018452	20:7992718-8003011	0	0	0	OK
ENSBTAG00000018453	ENSBTAG00000018453	27:14706020-14708632	0	0	0	OK

ENSBTAG00000018473	ENSBTAG00000018473	2:71099130-71134074	0	0	0	OK
ENSBTAG00000018479	ENSBTAG00000018479	15:78571081-78576838	0	0	0	OK
ENSBTAG00000018490	ENSBTAG00000018490	15:46438268-46439222	0	0	0	OK
ENSBTAG00000018495	ENSBTAG00000018495	27:36248820-36253367	0	0	0	OK
ENSBTAG00000018503	ENSBTAG00000018503	7:54857212-54878162	0	0	0	OK
ENSBTAG00000018527	ENSBTAG00000018527	21:25317911-25392905	0	0	0	OK
ENSBTAG00000018528	ENSBTAG00000018528	9:50963544-51013480	0	0	0	OK
ENSBTAG00000018531	ENSBTAG00000018531	6:87759437-87768834	0	0	0	OK
ENSBTAG00000018534	ENSBTAG00000018534	17:74613479-74620081	0	0	0	OK
ENSBTAG00000018539	ENSBTAG00000018539	18:57338708-57343795	0	0	0	OK
ENSBTAG00000018540	ENSBTAG00000018540	29:6147989-6326526	0	0	0	OK
ENSBTAG00000018549	ENSBTAG00000018549	2:101882144-101924558	0	0	0	OK
ENSBTAG00000018552	ENSBTAG00000018552	2:102927401-103134122	0	0	0	OK
ENSBTAG00000018554	ENSBTAG00000018554	11:43948905-43949800	0	0	0	OK
ENSBTAG00000018575	ENSBTAG00000018575	5:1907924-2352693	0	0	0	OK
ENSBTAG00000018594	ENSBTAG00000018594	18:46649251-46654186	0	0	0	OK
ENSBTAG00000018607	ENSBTAG00000018607	1:145940971-145973811	0	0	0	OK
ENSBTAG00000018628	ENSBTAG00000018628	22:49499797-49501972	0	0	0	OK
ENSBTAG00000018634	ENSBTAG00000018634	9:19526851-19547939	0	0	0	OK
ENSBTAG00000018652	ENSBTAG00000018652	18:51830894-51837501	0	0	0	OK
ENSBTAG00000018655	ENSBTAG00000018655	7:20683773-20695698	0	0	0	OK
ENSBTAG00000018661	ENSBTAG00000018661	19:54647277-54666987	0	0	0	OK
ENSBTAG00000018663	ENSBTAG00000018663	19:57642754-57655943	0	0	0	OK
ENSBTAG00000018673	ENSBTAG00000018673	7:2540994-2546082	0	0	0	OK
ENSBTAG00000018688	ENSBTAG00000018688	1:150144148-150153934	0	0	0	OK
ENSBTAG00000018715	ENSBTAG00000018715	4:81642480-81644519	0	0	0	OK
ENSBTAG00000018724	ENSBTAG00000018724	16:53064137-53068821	0	0	0	OK
ENSBTAG00000018726	ENSBTAG00000018726	3:106154950-106169324	0	0	0	OK
ENSBTAG00000018731	ENSBTAG00000018731	1:46322717-46323776	0	0	0	OK
ENSBTAG00000018737	ENSBTAG00000018737	3:51282135-51325805	0	0	0	OK

ENSBTAG00000018742	ENSBTAG00000018742	15:78613665-78633634	0	0	0	OK
ENSBTAG00000018743	ENSBTAG00000018743	5:17892373-17902271	0	0	0	OK
ENSBTAG00000018753	ENSBTAG00000018753	2:62843282-63111812	0	0	0	OK
ENSBTAG00000018759	ENSBTAG00000018759	19:41259126-41259981	0	0	0	OK
ENSBTAG00000018778	ENSBTAG00000018778	8:49200918-49307438	0	0	0	OK
ENSBTAG00000018784	ENSBTAG00000018784	13:57889706-57899205	0	0	0	OK
ENSBTAG00000018787	ENSBTAG00000018787	23:45185213-45191309	0	0	0	OK
ENSBTAG00000018792	ENSBTAG00000018792	29:43416169-43433570	0	0	0	OK
ENSBTAG00000018807	ENSBTAG00000018807	3:34221091-34235694	0	0	0	OK
ENSBTAG00000018823	ENSBTAG00000018823	19:44775156-44782140	0	0	0	OK
ENSBTAG00000018829	ENSBTAG00000018829	12:19493437-19511216	0	0	0	OK
ENSBTAG00000018840	ENSBTAG00000018840	19:36032857-36035427	0	0	0	OK
ENSBTAG00000018841	ENSBTAG00000018841	8:100604291-100681999	0	0	0	OK
ENSBTAG00000018857	ENSBTAG00000018857	28:200774-243023	0	0	0	OK
ENSBTAG00000018861	ENSBTAG00000018861	5:49606969-49682912	0	0	0	OK
ENSBTAG00000018864	ENSBTAG00000018864	27:14164458-14209872	0	0	0	OK
ENSBTAG00000018885	ENSBTAG00000018885	6:48314929-48315299	0	0	0	OK
ENSBTAG00000018890	ENSBTAG00000018890	15:79791487-79792452	0	0	0	OK
ENSBTAG00000018894	ENSBTAG00000018894	7:17569053-17579485	0	0	0	OK
ENSBTAG00000018906	ENSBTAG00000018906	18:35339675-35341472	0	0	0	OK
ENSBTAG00000018918	ENSBTAG00000018918	22:51540980-51542102	0	0	0	OK
ENSBTAG00000018926	ENSBTAG00000018926	7:46075147-46076063	0	0	0	OK
ENSBTAG00000018960	ENSBTAG00000018960	28:12923498-12925584	0	0	0	OK
ENSBTAG00000018986	ENSBTAG00000018986	X:62476951-62508324	0	0	0	OK
ENSBTAG00000018987	ENSBTAG00000018987	3:16507483-16507861	0	0	0	OK
ENSBTAG00000018994	ENSBTAG00000018994	1:95752048-95767928	0	0	0	OK
ENSBTAG00000019003	ENSBTAG00000019003	2:105576567-105577158	0	0	0	OK
ENSBTAG00000019007	ENSBTAG00000019007	1:128036021-128036420	0	0	0	OK
ENSBTAG00000019025	ENSBTAG00000019025	7:19698986-19704035	0	0	0	OK
ENSBTAG00000019038	ENSBTAG00000019038	3:10295276-10296206	0	0	0	OK

ENSBTAG00000019041	ENSBTAG00000019041	22:33069683-33589723	0	0	0	OK
ENSBTAG00000019044	ENSBTAG00000019044	19:52198616-52263294	0	0	0	OK
ENSBTAG00000019048	ENSBTAG00000019048	22:36737620-36738157	0	0	0	OK
ENSBTAG00000019075	ENSBTAG00000019075	2:24512077-24590687	0	0	0	OK
ENSBTAG00000019078	ENSBTAG00000019078	22:51890962-51891301	0	0	0	OK
ENSBTAG00000019080	ENSBTAG00000019080	15:39728331-39730868	0	0	0	OK
ENSBTAG00000019082	ENSBTAG00000019082	19:15297465-15309154	0	0	0	OK
ENSBTAG00000019084	ENSBTAG00000019084	5:56340055-56350443	0	0	0	OK
ENSBTAG00000019094	ENSBTAG00000019094	19:15273797-15282574	0	0	0	OK
ENSBTAG00000019095	ENSBTAG00000019095	19:43377278-43379246	0	0	0	OK
ENSBTAG00000019103	ENSBTAG00000019103	25:2206390-2209499	0	0	0	OK
ENSBTAG00000019106	ENSBTAG00000019106	22:13214223-13216690	0	0	0	OK
ENSBTAG00000019155	ENSBTAG00000019155	5:44127710-44133546	0	0	0	OK
ENSBTAG00000019159	ENSBTAG00000019159	5:75550903-75568048	0	0	0	OK
ENSBTAG00000019168	ENSBTAG00000019168	15:52623452-52627368	0	0	0	OK
ENSBTAG00000019181	ENSBTAG00000019181	25:38227395-38280018	0	0	0	OK
ENSBTAG00000019199	ENSBTAG00000019199	24:35360072-35374961	0	0	0	OK
ENSBTAG00000019203	ENSBTAG00000019203	3:16887101-16888570	0	0	0	OK
ENSBTAG00000019237	ENSBTAG00000019237	23:47333402-47348212	0	0	0	OK
ENSBTAG00000019246	ENSBTAG00000019246	15:32412563-32424510	0	0	0	OK
ENSBTAG00000019247	ENSBTAG00000019247	X:147944472-147944824	0	0	0	OK
ENSBTAG00000019249	ENSBTAG00000019249	25:1486478-1492437	0	0	0	OK
ENSBTAG00000019268	ENSBTAG00000019268	12:26114596-26115193	0	0	0	OK
ENSBTAG00000019276	ENSBTAG00000019276	4:33507616-33508901	0	0	0	OK
ENSBTAG00000019277	ENSBTAG00000019277	5:30444364-30463019	0	0	0	OK
ENSBTAG00000019282	ENSBTAG00000019282	17:68524675-68530920	0	0	0	OK
ENSBTAG00000019284	ENSBTAG00000019284	11:84076853-84078206	0	0	0	OK
ENSBTAG00000019300	ENSBTAG00000019300	23:39209389-39232415	0	0	0	OK
ENSBTAG00000019310	ENSBTAG00000019310	25:39761773-39816244	0	0	0	OK
ENSBTAG00000019317	ENSBTAG00000019317	19:51422925-51427197	0	0	0	OK

ENSBTAG00000019330	ENSBTAG00000019330	22:30035341-30044548	0	0	0	OK
ENSBTAG00000019339	ENSBTAG00000019339	16:30390091-30490348	0	0	0	OK
ENSBTAG00000019343	ENSBTAG00000019343	23:15467449-15482249	0	0	0	OK
ENSBTAG00000019350	ENSBTAG00000019350	21:47310561-47831598	0	0	0	OK
ENSBTAG00000019357	ENSBTAG00000019357	25:26993386-26996169	0	0	0	OK
ENSBTAG00000019369	ENSBTAG00000019369	29:50560712-50570675	0	0	0	OK
ENSBTAG00000019390	ENSBTAG00000019390	10:46749739-46768704	0	0	0	OK
ENSBTAG00000019401	ENSBTAG00000019401	3:70887241-70997136	0	0	0	OK
ENSBTAG00000019403	ENSBTAG00000019403	4:32057861-32077033	0	0	0	OK
ENSBTAG00000019410	ENSBTAG00000019410	9:41469870-41554001	0	0	0	OK
ENSBTAG00000019412	ENSBTAG00000019412	25:17269947-17407207	0	0	0	OK
ENSBTAG00000019419	ENSBTAG00000019419	7:45427273-45433438	0	0	0	OK
ENSBTAG00000019435	ENSBTAG00000019435	25:37329919-37331436	0	0	0	OK
ENSBTAG00000019440	ENSBTAG00000019440	12:90532575-90541989	0	0	0	OK
ENSBTAG00000019447	ENSBTAG00000019447	4:71118137-71121493	0	0	0	OK
ENSBTAG00000019451	ENSBTAG00000019451	6:116194012-116240769	0	0	0	OK
ENSBTAG00000019457	ENSBTAG00000019457	7:68106043-68141498	0	0	0	OK
ENSBTAG00000019459	ENSBTAG00000019459	11:36341722-36351231	0	0	0	OK
ENSBTAG00000019471	ENSBTAG00000019471	16:28231306-28245538	0	0	0	OK
ENSBTAG00000019490	ENSBTAG00000019490	6:33228240-33228987	0	0	0	OK
ENSBTAG00000019496	ENSBTAG00000019496	10:67155090-67171864	0	0	0	OK
ENSBTAG00000019501	ENSBTAG00000019501	12:17960582-17967118	0	0	0	OK
ENSBTAG00000019516	ENSBTAG00000019516	17:74927011-74927547	0	0	0	OK
ENSBTAG00000019518	ENSBTAG00000019518	11:72273636-72277103	0	0	0	OK
ENSBTAG00000019521	ENSBTAG00000019521	25:27737581-27738205	0	0	0	OK
ENSBTAG00000019525	ENSBTAG00000019525	28:29613179-29630076	0	0	0	OK
ENSBTAG00000019536	ENSBTAG00000019536	22:23229271-23251050	0	0	0	OK
ENSBTAG00000019543	ENSBTAG00000019543	25:2281944-2285931	0	0	0	OK
ENSBTAG00000019544	ENSBTAG00000019544	15:48884647-48885589	0	0	0	OK
ENSBTAG00000019552	ENSBTAG00000019552	X:3468826-3477212	0	0	0	OK

ENSBTAG00000019569	ENSBTAG00000019569	29:50695517-50700467	0	0	0	OK
ENSBTAG00000019579	ENSBTAG00000019579	29:50654118-50655720	0	0	0	OK
ENSBTAG00000019587	ENSBTAG00000019587	14:40275715-40307947	0	0	0	OK
ENSBTAG00000019601	ENSBTAG00000019601	10:58569061-58646799	0	0	0	OK
ENSBTAG00000019622	ENSBTAG00000019622	15:55237978-55238659	0	0	0	OK
ENSBTAG00000019628	ENSBTAG00000019628	11:44451546-44467795	0	0	0	OK
ENSBTAG00000019644	ENSBTAG00000019644	15:75074939-75265705	0	0	0	OK
ENSBTAG00000019670	ENSBTAG00000019670	1:116881871-116882819	0	0	0	OK
ENSBTAG00000019678	ENSBTAG00000019678	18:46870115-46875864	0	0	0	OK
ENSBTAG00000019696	ENSBTAG00000019696	29:45503290-45506372	0	0	0	OK
ENSBTAG00000019701	ENSBTAG00000019701	11:6003174-6008528	0	0	0	OK
ENSBTAG00000019716	ENSBTAG00000019716	6:90559881-90563647	0	0	0	OK
ENSBTAG00000019718	ENSBTAG00000019718	7:45465833-45467519	0	0	0	OK
ENSBTAG00000019733	ENSBTAG00000019733	3:65921136-66062039	0	0	0	OK
ENSBTAG00000019734	ENSBTAG00000019734	10:20341749-20354153	0	0	0	OK
ENSBTAG00000019746	ENSBTAG00000019746	22:44952364-44995732	0	0	0	OK
ENSBTAG00000019768	ENSBTAG00000019768	15:45331658-45332588	0	0	0	OK
ENSBTAG00000019771	ENSBTAG00000019771	7:44926930-44939347	0	0	0	OK
ENSBTAG00000019772	ENSBTAG00000019772	22:17817625-17827292	0	0	0	OK
ENSBTAG00000019777	ENSBTAG00000019777	5:103961031-103963273	0	0	0	OK
ENSBTAG00000019783	ENSBTAG00000019783	5:103939885-103942299	0	0	0	OK
ENSBTAG00000019788	ENSBTAG00000019788	5:107264249-107325273	0	0	0	OK
ENSBTAG00000019803	ENSBTAG00000019803	25:110167-112894	0	0	0	OK
ENSBTAG00000019811	ENSBTAG00000019811	13:51110901-51173199	0	0	0	OK
ENSBTAG00000019824	ENSBTAG00000019824	4:47508241-47508671	0	0	0	OK
ENSBTAG00000019830	ENSBTAG00000019830	21:19858316-19867590	0	0	0	OK
ENSBTAG00000019831	ENSBTAG00000019831	21:19867699-19898710	0	0	0	OK
ENSBTAG00000019840	ENSBTAG00000019840	17:35104574-35104944	0	0	0	OK
ENSBTAG00000019879	ENSBTAG00000019879	10:63804150-63804644	0	0	0	OK
ENSBTAG00000019890	ENSBTAG00000019890	16:79333028-79353972	0	0	0	OK

ENSBTAG00000019894	ENSBTAG00000019894	17:74711563-74715846	0	0	0	OK
ENSBTAG00000019911	ENSBTAG00000019911	19:43468380-43469490	0	0	0	OK
ENSBTAG00000019924	ENSBTAG00000019924	10:65907457-65971345	0	0	0	OK
ENSBTAG00000019942	ENSBTAG00000019942	X:42008983-42011006	0	0	0	OK
ENSBTAG00000019946	ENSBTAG00000019946	5:25289515-25300934	0	0	0	OK
ENSBTAG00000019956	ENSBTAG00000019956	3:99484968-99521190	0	0	0	OK
ENSBTAG00000019961	ENSBTAG00000019961	12:90758545-90763027	0	0	0	OK
ENSBTAG00000019971	ENSBTAG00000019971	12:91039121-91065655	0	0	0	OK
ENSBTAG00000019972	ENSBTAG00000019972	19:52090694-52101811	0	0	0	OK
ENSBTAG00000019993	ENSBTAG00000019993	X:13559122-13564931	0	0	0	OK
ENSBTAG00000019995	ENSBTAG00000019995	29:9263217-9300511	0	0	0	OK
ENSBTAG00000020018	ENSBTAG00000020018	9:35040650-35138636	0	0	0	OK
ENSBTAG00000020027	ENSBTAG00000020027	3:9442356-9442758	0	0	0	OK
ENSBTAG00000020033	ENSBTAG00000020033	15:50488661-50489603	0	0	0	OK
ENSBTAG00000020055	ENSBTAG00000020055	8:6449814-6473747	0	0	0	OK
ENSBTAG00000020074	ENSBTAG00000020074	18:53211189-53218864	0	0	0	OK
ENSBTAG00000020095	ENSBTAG00000020095	15:84485247-84492848	0	0	0	OK
ENSBTAG00000020097	ENSBTAG00000020097	9:76937938-76952270	0	0	0	OK
ENSBTAG00000020116	ENSBTAG00000020116	23:28469734-28473401	0	0	0	OK
ENSBTAG00000020140	ENSBTAG00000020140	8:29522479-29525223	0	0	0	OK
ENSBTAG00000020152	ENSBTAG00000020152	3:8395604-8407551	0	0	0	OK
ENSBTAG00000020180	ENSBTAG00000020180	3:50448642-50467156	0	0	0	OK
ENSBTAG00000020183	ENSBTAG00000020183	4:95209523-95233877	0	0	0	OK
ENSBTAG00000020191	ENSBTAG00000020191	2:107646741-107650448	0	0	0	OK
ENSBTAG00000020201	ENSBTAG00000020201	10:7992985-7995677	0	0	0	OK
ENSBTAG00000020209	ENSBTAG00000020209	25:2744937-2746130	0	0	0	OK
ENSBTAG00000020226	ENSBTAG00000020226	14:4182521-4185763	0	0	0	OK
ENSBTAG00000020233	ENSBTAG00000020233	26:34884593-34917883	0	0	0	OK
ENSBTAG00000020251	ENSBTAG00000020251	18:51707794-51717365	0	0	0	OK
ENSBTAG00000020253	ENSBTAG00000020253	19:27733907-27735191	0	0	0	OK

ENSBTAG00000020270	ENSBTAG00000020270	6:23557310-23679508	0	0	0	OK
ENSBTAG00000020273	ENSBTAG00000020273	16:4721588-4723835	0	0	0	OK
ENSBTAG00000020321	ENSBTAG00000020321	19:39013106-39021625	0	0	0	OK
ENSBTAG00000020330	ENSBTAG00000020330	5:22086070-22088781	0	0	0	OK
ENSBTAG00000020346	ENSBTAG00000020346	17:54003919-54039850	0	0	0	OK
ENSBTAG00000020348	ENSBTAG00000020348	11:2231383-2245550	0	0	0	OK
ENSBTAG00000020350	ENSBTAG00000020350	11:2250454-2252492	0	0	0	OK
ENSBTAG00000020352	ENSBTAG00000020352	10:16192252-16247610	0	0	0	OK
ENSBTAG00000020353	ENSBTAG00000020353	7:40254114-40254661	0	0	0	OK
ENSBTAG00000020368	ENSBTAG00000020368	9:44674888-44677439	0	0	0	OK
ENSBTAG00000020369	ENSBTAG00000020369	25:1536096-1538058	0	0	0	OK
ENSBTAG00000020397	ENSBTAG00000020397	13:58439174-58446547	0	0	0	OK
ENSBTAG00000020405	ENSBTAG00000020405	14:17132668-17140075	0	0	0.470584	OK
ENSBTAG00000020415	ENSBTAG00000020415	18:57360331-57364101	0	0	0	OK
ENSBTAG00000020420	ENSBTAG00000020420	X:134965646-134991291	0	0	0	OK
ENSBTAG00000020431	ENSBTAG00000020431	19:26683194-26691719	0	0	0	OK
ENSBTAG00000020432	ENSBTAG00000020432	19:26678019-26682795	0	0	0	OK
ENSBTAG00000020439	ENSBTAG00000020439	25:37524460-37532400	0	0	0	OK
ENSBTAG00000020440	ENSBTAG00000020440	25:37513720-37524164	0	0	0	OK
ENSBTAG00000020449	ENSBTAG00000020449	18:57795756-57808326	0	0	0	OK
ENSBTAG00000020450	ENSBTAG00000020450	18:57809112-57810395	0	0	0	OK
ENSBTAG00000020457	ENSBTAG00000020457	18:50771354-50785670	0	0	0	OK
ENSBTAG00000020466	ENSBTAG00000020466	15:9659319-9691005	0	0	0	OK
ENSBTAG00000020469	ENSBTAG00000020469	10:89619637-89648224	0	0	0	OK
ENSBTAG00000020490	ENSBTAG00000020490	11:103572294-103607342	0	0	0	OK
ENSBTAG00000020495	ENSBTAG00000020495	GJ058424.1:10381-37633	0	0	0	OK
ENSBTAG00000020499	ENSBTAG00000020499	2:133176677-133189041	0	0	0	OK
ENSBTAG00000020513	ENSBTAG00000020513	X:84738869-84745441	0	0	0	OK
ENSBTAG00000020514	ENSBTAG00000020514	21:8377895-8452307	0	0	0	OK
ENSBTAG00000020518	ENSBTAG00000020518	16:46249162-46265920	0	0	0	OK

ENSBTAG00000020530	ENSBTAG00000020530	12:32720218-32723627	0	0	0	OK
ENSBTAG00000020532	ENSBTAG00000020532	17:51707858-51708401	0	0	0	OK
ENSBTAG00000020541	ENSBTAG00000020541	6:37677098-37679897	0	0	0	OK
ENSBTAG00000020542	ENSBTAG00000020542	3:16143798-16162170	0	0	0	OK
ENSBTAG00000020550	ENSBTAG00000020550	4:14575665-14576961	0	0	0	OK
ENSBTAG00000020555	ENSBTAG00000020555	13:61584390-61595672	0	0	0	OK
ENSBTAG00000020558	ENSBTAG00000020558	18:53057716-53059957	0	0	0	OK
ENSBTAG00000020561	ENSBTAG00000020561	X:126287039-126303727	0	0	0	OK
ENSBTAG00000020566	ENSBTAG00000020566	17:73694206-73778104	0	0	0	OK
ENSBTAG00000020575	ENSBTAG00000020575	11:107202289-107203655	0	0	0	OK
ENSBTAG00000020591	ENSBTAG00000020591	16:40005643-40029767	0	0	0	OK
ENSBTAG00000020592	ENSBTAG00000020592	3:120133412-120134255	0	0	0	OK
ENSBTAG00000020598	ENSBTAG00000020598	6:104598244-104657218	0	0	0	OK
ENSBTAG00000020606	ENSBTAG00000020606	2:107881215-107884050	0	0	0	OK
ENSBTAG00000020615	ENSBTAG00000020615	7:9168857-9178475	0	0	0	OK
ENSBTAG00000020620	ENSBTAG00000020620	4:58724264-58724900	0	0	0	OK
ENSBTAG00000020632	ENSBTAG00000020632	18:36911357-36924689	0	0	0	OK
ENSBTAG00000020635	ENSBTAG00000020635	3:20534020-20600696	0	0	0	OK
ENSBTAG00000020644	ENSBTAG00000020644	X:17087446-17122426	0	0	0	OK
ENSBTAG00000020645	ENSBTAG00000020645	22:50670851-50691007	0	0	0	OK
ENSBTAG00000020662	ENSBTAG00000020662	5:57774485-57823736	0	0	0	OK
ENSBTAG00000020673	ENSBTAG00000020673	15:49098191-49099151	0	0	0	OK
ENSBTAG00000020685	ENSBTAG00000020685	4:14000445-14001033	0	0	0	OK
ENSBTAG00000020714	ENSBTAG00000020714	18:1799803-1821405	0	0	0	OK
ENSBTAG00000020721	ENSBTAG00000020721	7:2905628-2909524	0	0	0	OK
ENSBTAG00000020723	ENSBTAG00000020723	9:92570120-92570394	0	0	0	OK
ENSBTAG00000020729	ENSBTAG00000020729	10:72617791-72641194	0	0	0	OK
ENSBTAG00000020734	ENSBTAG00000020734	25:16543862-16550325	0	0	0	OK
ENSBTAG00000020737	ENSBTAG00000020737	25:787492-790981	0	0	0	OK
ENSBTAG00000020760	ENSBTAG00000020760	17:74261018-74271390	0	0	0	OK

ENSBTAG00000020767	ENSBTAG00000020767	3:7070263-7074707	0	0	0	OK
ENSBTAG00000020773	ENSBTAG00000020773	29:44661697-44665098	0	0	0	OK
ENSBTAG00000020774	ENSBTAG00000020774	19:10634792-10635464	0	0	0	OK
ENSBTAG00000020794	ENSBTAG00000020794	2:71309223-71309861	0	0	0	OK
ENSBTAG00000020795	ENSBTAG00000020795	13:55358719-55359979	0	0	0	OK
ENSBTAG00000020797	ENSBTAG00000020797	13:55346047-55356672	0	0	0	OK
ENSBTAG00000020801	ENSBTAG00000020801	14:11839258-11871079	0	0	0	OK
ENSBTAG00000020803	ENSBTAG00000020803	X:141098690-141104299	0	0	0	OK
ENSBTAG00000020807	ENSBTAG00000020807	29:43990459-43991956	0	0	0	OK
ENSBTAG00000020813	ENSBTAG00000020813	13:74217758-74219386	0	0	0	OK
ENSBTAG00000020814	ENSBTAG00000020814	10:47955994-47957472	0	0	0	OK
ENSBTAG00000020817	ENSBTAG00000020817	9:10202747-10301176	0	0	0	OK
ENSBTAG00000020826	ENSBTAG00000020826	X:82414978-82455573	0	0	0	OK
ENSBTAG00000020836	ENSBTAG00000020836	18:11196384-11208223	0	0	0	OK
ENSBTAG00000020838	ENSBTAG00000020838	3:10977983-10978928	0	0	0	OK
ENSBTAG00000020872	ENSBTAG00000020872	18:54753333-54767002	0	0	0	OK
ENSBTAG00000020883	ENSBTAG00000020883	17:35618099-35622852	0	0	0	OK
ENSBTAG00000020889	ENSBTAG00000020889	18:34668690-34677726	0	0	0	OK
ENSBTAG00000020893	ENSBTAG00000020893	11:78889150-78907349	0	0	0	OK
ENSBTAG00000020894	ENSBTAG00000020894	11:78862494-78880461	0	0	0	OK
ENSBTAG00000020896	ENSBTAG00000020896	25:41349161-41350363	0	0	0	OK
ENSBTAG00000020898	ENSBTAG00000020898	25:41372193-41390545	0	0	0	OK
ENSBTAG00000020918	ENSBTAG00000020918	26:44416759-44420132	0	0	0	OK
ENSBTAG00000020919	ENSBTAG00000020919	4:69252419-69255793	0	0	0	OK
ENSBTAG00000020930	ENSBTAG00000020930	21:49571620-49593136	0	0	0	OK
ENSBTAG00000020931	ENSBTAG00000020931	4:67065204-67401294	0	0	0	OK
ENSBTAG00000020934	ENSBTAG00000020934	10:65471176-65490132	0	0	0	OK
ENSBTAG00000020962	ENSBTAG00000020962	22:50587356-50589110	0	0	0	OK
ENSBTAG00000020995	ENSBTAG00000020995	6:102287967-102288349	0	0	0	OK
ENSBTAG00000020998	ENSBTAG00000020998	22:60179707-60211026	0	0	0	OK

ENSBTAG00000021003	ENSBTAG00000021003	24:2406660-2418040	0	0	0	OK
ENSBTAG00000021019	ENSBTAG00000021019	10:65176254-65202411	0	0	0	OK
ENSBTAG00000021059	ENSBTAG00000021059	X:50951670-50973156	0	0	0	OK
ENSBTAG00000021062	ENSBTAG00000021062	19:48733737-48742209	0	0	0	OK
ENSBTAG00000021079	ENSBTAG00000021079	3:103324512-103327560	0	0	0	OK
ENSBTAG00000021098	ENSBTAG00000021098	7:43566614-43567568	0	0	0	OK
ENSBTAG00000021101	ENSBTAG00000021101	3:42891830-42892982	0	0	0	OK
ENSBTAG00000021109	ENSBTAG00000021109	16:31435844-31436558	0	0	0	OK
ENSBTAG00000021119	ENSBTAG00000021119	22:53432382-53437016	0	0	0	OK
ENSBTAG00000021150	ENSBTAG00000021150	15:45311135-45312104	0	0	0	OK
ENSBTAG00000021161	ENSBTAG00000021161	11:6279239-6297039	0	0	0	OK
ENSBTAG00000021175	ENSBTAG00000021175	9:15862736-15894731	0	0	0	OK
ENSBTAG00000021185	ENSBTAG00000021185	18:55145743-55157457	0	0	0	OK
ENSBTAG00000021188	ENSBTAG00000021188	15:84347032-84351272	0	0	0	OK
ENSBTAG00000021196	ENSBTAG00000021196	5:68866723-68876104	0	0	0	OK
ENSBTAG00000021199	ENSBTAG00000021199	21:69847358-69873999	0	0	0	OK
ENSBTAG00000021200	ENSBTAG00000021200	16:1774373-1777002	0	0	0	OK
ENSBTAG00000021208	ENSBTAG00000021208	14:64315459-64352488	0	0	0	OK
ENSBTAG00000021210	ENSBTAG00000021210	16:47798008-47800497	0	0	0	OK
ENSBTAG00000021233	ENSBTAG00000021233	15:82924406-82925351	0	0	0	OK
ENSBTAG00000021246	ENSBTAG00000021246	26:23728580-23741299	0	0	0	OK
ENSBTAG00000021248	ENSBTAG00000021248	25:38963388-38974572	0	0	0	OK
ENSBTAG00000021260	ENSBTAG00000021260	4:115335970-115360689	0	0	0	OK
ENSBTAG00000021261	ENSBTAG00000021261	7:46213581-46213977	0	0	0	OK
ENSBTAG00000021262	ENSBTAG00000021262	17:73479565-73484880	0	0	0	OK
ENSBTAG00000021275	ENSBTAG00000021275	21:22043353-22046525	0	0	0	OK
ENSBTAG00000021283	ENSBTAG00000021283	11:86759337-86761647	0	0	0	OK
ENSBTAG00000021322	ENSBTAG00000021322	2:116332505-116343431	0	0	0	OK
ENSBTAG00000021326	ENSBTAG00000021326	2:116759407-116762490	0	0	0	OK
ENSBTAG00000021361	ENSBTAG00000021361	9:69266999-69311228	0	0	0	OK

ENSBTAG00000021373	ENSBTAG00000021373	11:103080226-103098809	0	0	0	OK
ENSBTAG00000021375	ENSBTAG00000021375	15:82754158-82755118	0	0	0	OK
ENSBTAG00000021378	ENSBTAG00000021378	3:16818413-16824125	0	0	0	OK
ENSBTAG00000021390	ENSBTAG00000021390	22:51834382-51835268	0	0	0	OK
ENSBTAG00000021407	ENSBTAG00000021407	25:2578660-2583865	0	0	0	OK
ENSBTAG00000021421	ENSBTAG00000021421	X:39925627-39929382	0	0	0	OK
ENSBTAG00000021423	ENSBTAG00000021423	13:54264622-54266440	0	0	0	OK
ENSBTAG00000021439	ENSBTAG00000021439	X:39304238-39312739	0	0	0	OK
ENSBTAG00000021444	ENSBTAG00000021444	3:118630616-118690526	0	0	0	OK
ENSBTAG00000021446	ENSBTAG00000021446	9:69015441-69015687	0	0	0	OK
ENSBTAG00000021460	ENSBTAG00000021460	5:58463854-58464790	0	0	0	OK
ENSBTAG00000021467	ENSBTAG00000021467	5:27044008-27047853	0	0	0	OK
ENSBTAG00000021485	ENSBTAG00000021485	3:20406683-20413546	0	0	0	OK
ENSBTAG00000021492	ENSBTAG00000021492	16:62462601-62470464	0	0	0	OK
ENSBTAG00000021493	ENSBTAG00000021493	25:9998857-9999359	0	0	0	OK
ENSBTAG00000021503	ENSBTAG00000021503	12:58979621-58980609	0	0	0	OK
ENSBTAG00000021506	ENSBTAG00000021506	22:32552970-32574431	0	0	0	OK
ENSBTAG00000021515	ENSBTAG00000021515	1:71396753-71409483	0	0	0	OK
ENSBTAG00000021522	ENSBTAG00000021522	25:13674071-13693829	0	0	0	OK
ENSBTAG00000021525	ENSBTAG00000021525	2:125426085-125427402	0	0	0	OK
ENSBTAG00000021537	ENSBTAG00000021537	3:48398160-48410727	0	0	0	OK
ENSBTAG00000021544	ENSBTAG00000021544	3:103422604-103429520	0	0	0	OK
ENSBTAG00000021549	ENSBTAG00000021549	11:106334244-106335688	0	0	0	OK
ENSBTAG00000021591	ENSBTAG00000021591	3:19054052-19057123	0	0	0	OK
ENSBTAG00000021594	ENSBTAG00000021594	3:19030950-19051640	0	0	0	OK
ENSBTAG00000021607	ENSBTAG00000021607	20:18200879-18371920	0	0	0	OK
ENSBTAG00000021620	ENSBTAG00000021620	2:129716208-129723902	0	0	0	OK
ENSBTAG00000021621	ENSBTAG00000021621	5:57967851-57968814	0	0	0	OK
ENSBTAG00000021624	ENSBTAG00000021624	X:66893738-66896567	0	0	0	OK
ENSBTAG00000021639	ENSBTAG00000021639	14:2892787-2893978	0	0	0	OK

ENSBTAG00000021649	ENSBTAG00000021649	3:14353657-14354168	0	0	0	OK
ENSBTAG00000021663	ENSBTAG00000021663	9:63462323-63474252	0	0	0	OK
ENSBTAG00000021667	ENSBTAG00000021667	9:26693804-26856917	0	0	0	OK
ENSBTAG00000021672	ENSBTAG00000021672	16:13314191-13318292	0	0	0	OK
ENSBTAG00000021684	ENSBTAG00000021684	X:140434144-140435278	0	0	0	OK
ENSBTAG00000021693	ENSBTAG00000021693	21:34144322-34144922	0	0	0	OK
ENSBTAG00000021717	ENSBTAG00000021717	21:62701849-62743837	0	0	0	OK
ENSBTAG00000021727	ENSBTAG00000021727	13:55085431-55135137	0	0	0	OK
ENSBTAG00000021767	ENSBTAG00000021767	5:23370704-23411877	0	0	0	OK
ENSBTAG00000021771	ENSBTAG00000021771	1:145057827-145075459	0	0	0	OK
ENSBTAG00000021785	ENSBTAG00000021785	25:26549203-26565653	0	0	0	OK
ENSBTAG00000021789	ENSBTAG00000021789	18:51504689-51507155	0	0	0	OK
ENSBTAG00000021790	ENSBTAG00000021790	X:75160535-75265102	0	0	0	OK
ENSBTAG00000021807	ENSBTAG00000021807	5:56744604-56748242	0	0	0	OK
ENSBTAG00000021809	ENSBTAG00000021809	15:64453866-64477077	0	0	0	OK
ENSBTAG00000021811	ENSBTAG00000021811	13:60729755-60773778	0	0	0	OK
ENSBTAG00000021816	ENSBTAG00000021816	13:52826625-52827312	0	0	0	OK
ENSBTAG00000021821	ENSBTAG00000021821	14:31840097-31840918	0	0	0	OK
ENSBTAG00000021837	ENSBTAG00000021837	13:54270411-54275998	0	0	0	OK
ENSBTAG00000021851	ENSBTAG00000021851	4:48870589-48922462	0	0	0	OK
ENSBTAG00000021856	ENSBTAG00000021856	26:19164409-19197680	0	0	0	OK
ENSBTAG00000021864	ENSBTAG00000021864	19:25847058-25849237	0	0	0	OK
ENSBTAG00000021865	ENSBTAG00000021865	19:25840191-25845818	0	0	0	OK
ENSBTAG00000021876	ENSBTAG00000021876	10:56678900-56805084	0	0	0	OK
ENSBTAG00000021885	ENSBTAG00000021885	13:60684666-60691970	0	0	0	OK
ENSBTAG00000021900	ENSBTAG00000021900	3:55231809-55365736	0	0	0	OK
ENSBTAG00000021910	ENSBTAG00000021910	1:90517078-90605473	0	0	0	OK
ENSBTAG00000021932	ENSBTAG00000021932	19:27436502-27438226	0	0	0	OK
ENSBTAG00000021940	ENSBTAG00000021940	6:45940146-46057441	0	0	0	OK
ENSBTAG00000021941	ENSBTAG00000021941	4:117555540-117803944	0	0	0	OK

ENSBTAG00000021954	ENSBTAG00000021954	2:28094269-28095174	0	0	0	OK
ENSBTAG00000021960	ENSBTAG00000021960	20:23975639-23980445	0	0	0	OK
ENSBTAG00000021980	ENSBTAG00000021980	5:84286535-84308915	0	0	0	OK
ENSBTAG00000021986	ENSBTAG00000021986	10:67756278-67765176	0	0	0	OK
ENSBTAG00000021991	ENSBTAG00000021991	8:91818659-91914721	0	0	0	OK
ENSBTAG00000022006	ENSBTAG00000022006	19:56359578-56363139	0	0	0	OK
ENSBTAG00000022007	ENSBTAG00000022007	13:66491916-66534355	0	0	0	OK
ENSBTAG00000022022	ENSBTAG00000022022	17:73486796-73498181	0	0	0	OK
ENSBTAG00000022027	ENSBTAG00000022027	17:73316335-73319008	0	0	0	OK
ENSBTAG00000022028	ENSBTAG00000022028	17:73241609-73243689	0	0	0	OK
ENSBTAG00000022030	ENSBTAG00000022030	17:73204693-73205970	0	0	0	OK
ENSBTAG00000022067	ENSBTAG00000022067	17:56607850-56616410	0	0	0	OK
ENSBTAG00000022083	ENSBTAG00000022083	5:82539064-82539763	0	0	0	OK
ENSBTAG00000022114	ENSBTAG00000022114	14:17254749-17298970	0	0	0	OK
ENSBTAG00000022128	ENSBTAG00000022128	X:38597969-38599160	0	0	0	OK
ENSBTAG00000022142	ENSBTAG00000022142	11:107148107-107149594	0	0	0	OK
ENSBTAG00000022183	ENSBTAG00000022183	18:84258-85115	0	0	0	OK
ENSBTAG00000022185	ENSBTAG00000022185	29:46923945-46947691	0	0	0	OK
ENSBTAG00000022209	ENSBTAG00000022209	29:46042200-46046958	0	0	0	OK
ENSBTAG00000022227	ENSBTAG00000022227	1:122911095-122937932	0	0	0	OK
ENSBTAG00000022246	ENSBTAG00000022246	29:45624665-45626149	0	0	0	OK
ENSBTAG00000022292	ENSBTAG00000022292	X:55167866-55170570	0	0	0	OK
ENSBTAG00000022293	ENSBTAG00000022293	29:41693758-41695210	0	0	0	OK
ENSBTAG00000022372	ENSBTAG00000022372	29:28137004-28137881	0	0	0	OK
ENSBTAG00000022376	ENSBTAG00000022376	29:28275088-28276018	0	0	0	OK
ENSBTAG00000022394	ENSBTAG00000022394	29:26711466-26714908	0	0	0	OK
ENSBTAG00000022396	ENSBTAG00000022396	29:26668046-26671801	0	0	0	OK
ENSBTAG00000022399	ENSBTAG00000022399	X:10114746-10116139	0	0	0	OK
ENSBTAG00000022427	ENSBTAG00000022427	29:13581142-13581886	0	0	0	OK
ENSBTAG00000022471	ENSBTAG00000022471	14:29765170-29793700	0	0	0	OK

ENSBTAG00000022484	ENSBTAG00000022484	12:69742531-69743248	0	0	0	OK
ENSBTAG00000022498	ENSBTAG00000022498	4:43383974-43384500	0	0	0	OK
ENSBTAG00000022529	ENSBTAG00000022529	X:101196295-101197412	0	0	0	OK
ENSBTAG00000022534	ENSBTAG00000022534	13:53319469-53320263	0	0	0	OK
ENSBTAG00000022539	ENSBTAG00000022539	14:23735754-23737474	0	0	0	OK
ENSBTAG00000022562	ENSBTAG00000022562	13:66892460-66893403	0	0	0	OK
ENSBTAG00000022571	ENSBTAG00000022571	13:43909080-43917999	0	0	0	OK
ENSBTAG00000022583	ENSBTAG00000022583	10:61600902-61640848	0	0	0	OK
ENSBTAG00000022603	ENSBTAG00000022603	5:6130466-6132109	0	0	0	OK
ENSBTAG00000022671	ENSBTAG00000022671	2:131304771-131315806	0	0	0	OK
ENSBTAG00000022690	ENSBTAG00000022690	7:43139372-43140311	0	0	0	OK
ENSBTAG00000022699	ENSBTAG00000022699	22:17832936-17846997	0	0	0	OK
ENSBTAG00000022714	ENSBTAG00000022714	20:24659524-24660204	0	0	0	OK
ENSBTAG00000022733	ENSBTAG00000022733	22:10586395-10588222	0	0	0	OK
ENSBTAG00000022736	ENSBTAG00000022736	23:19562566-19564449	0	0	0	OK
ENSBTAG00000022751	ENSBTAG00000022751	22:2807582-2978687	0	0	0	OK
ENSBTAG00000022767	ENSBTAG00000022767	27:20233577-20234153	0	0	0	OK
ENSBTAG00000022801	ENSBTAG00000022801	10:60078423-60128552	0	0	0	OK
ENSBTAG00000022825	ENSBTAG00000022825	25:38281391-38287463	0	0	0	OK
ENSBTAG00000022826	ENSBTAG00000022826	24:58747712-58751841	0	0	0	OK
ENSBTAG00000022861	ENSBTAG00000022861	5:99860608-99870803	0	0	0	OK
ENSBTAG00000022880	ENSBTAG00000022880	4:89213078-89213890	0	0	0	OK
ENSBTAG00000022895	ENSBTAG00000022895	24:807376-845750	0	0	0	OK
ENSBTAG00000022908	ENSBTAG00000022908	1:42095388-42096366	0	0	0	OK
ENSBTAG00000022942	ENSBTAG00000022942	16:28045122-28045563	0	0	0	OK
ENSBTAG00000022947	ENSBTAG00000022947	21:21965065-21965413	0	0	0	OK
ENSBTAG00000022971	ENSBTAG00000022971	5:44421168-44426117	0	0	0	OK
ENSBTAG00000022986	ENSBTAG00000022986	5:119408850-119590555	0	0	0	OK
ENSBTAG00000022989	ENSBTAG00000022989	21:14690051-14729722	0	0	0	OK
ENSBTAG00000023000	ENSBTAG00000023000	6:28075089-28075890	0	0	0	OK

ENSBTAG00000023054	ENSBTAG00000023054	28:25013023-25040775	0	0	0	OK
ENSBTAG00000023064	ENSBTAG00000023064	11:103802442-103822759	0	0	0	OK
ENSBTAG00000023074	ENSBTAG00000023074	28:3833610-3865953	0	0	0	OK
ENSBTAG00000023079	ENSBTAG00000023079	21:1632140-1649614	0	0	0	OK
ENSBTAG00000023125	ENSBTAG00000023125	15:47734612-47735572	0	0	0	OK
ENSBTAG00000023143	ENSBTAG00000023143	8:26010375-26010691	0	0	0	OK
ENSBTAG00000023157	ENSBTAG00000023157	27:5367341-5369810	0	0	0	OK
ENSBTAG00000023163	ENSBTAG00000023163	11:592072-595510	0	0	0	OK
ENSBTAG00000023187	ENSBTAG00000023187	12:91154818-91156716	0	0	0	OK
ENSBTAG00000023192	ENSBTAG00000023192	5:101751208-101757820	0	0	0	OK
ENSBTAG00000023206	ENSBTAG00000023206	24:22586220-22586611	0	0	0	OK
ENSBTAG00000023249	ENSBTAG00000023249	25:17400-17763	0	0	0	OK
ENSBTAG00000023270	ENSBTAG00000023270	24:10434673-10515343	0	0	0	OK
ENSBTAG00000023277	ENSBTAG00000023277	X:84901861-84902344	0	0	0	OK
ENSBTAG00000023300	ENSBTAG00000023300	29:29490137-29492835	0	0	0	OK
ENSBTAG00000023301	ENSBTAG00000023301	29:29484202-29486362	0	0	0	OK
ENSBTAG00000023333	ENSBTAG00000023333	18:63421901-63425192	0	0	0	OK
ENSBTAG00000023343	ENSBTAG00000023343	18:62547219-62549950	0	0	0	OK
ENSBTAG00000023349	ENSBTAG00000023349	18:62194104-62195025	0	0	0	OK
ENSBTAG00000023365	ENSBTAG00000023365	18:57927151-57929923	0	0	0	OK
ENSBTAG00000023367	ENSBTAG00000023367	18:53954605-53955352	0	0	0	OK
ENSBTAG00000023372	ENSBTAG00000023372	11:82694506-82697306	0	0	0	OK
ENSBTAG00000023407	ENSBTAG00000023407	1:141269172-141271197	0	0	0	OK
ENSBTAG00000023412	ENSBTAG00000023412	8:19629741-19632939	0	0	0	OK
ENSBTAG00000023431	ENSBTAG00000023431	17:66475469-66476704	0	0	0	OK
ENSBTAG00000023434	ENSBTAG00000023434	18:51949245-51953706	0	0	0	OK
ENSBTAG00000023439	ENSBTAG00000023439	18:52247310-52248128	0	0	0	OK
ENSBTAG00000023445	ENSBTAG00000023445	1:118300797-118318258	0	0	0	OK
ENSBTAG00000023447	ENSBTAG00000023447	1:117439604-117440224	0	0	0	OK
ENSBTAG00000023471	ENSBTAG00000023471	5:28525102-28525496	0	0	0	OK

ENSBTAG00000023507	ENSBTAG00000023507	7:44009278-44010145	0	0	0	OK
ENSBTAG00000023511	ENSBTAG00000023511	15:79921912-79922869	0	0	0	OK
ENSBTAG00000023513	ENSBTAG00000023513	1:86666812-86672081	0	0	0	OK
ENSBTAG00000023523	ENSBTAG00000023523	8:78327712-78418760	0	0	0	OK
ENSBTAG00000023530	ENSBTAG00000023530	14:78605823-78605938	0	0	0	OK
ENSBTAG00000023542	ENSBTAG00000023542	15:81105853-81107065	0	0	0	OK
ENSBTAG00000023562	ENSBTAG00000023562	18:48398053-48406309	0	0	0	OK
ENSBTAG00000023629	ENSBTAG00000023629	26:20527170-20527751	0	0	0	OK
ENSBTAG00000023632	ENSBTAG00000023632	18:34918529-34922481	0	0	0	OK
ENSBTAG00000023668	ENSBTAG00000023668	1:41955546-41957126	0	0	0	OK
ENSBTAG00000023731	ENSBTAG00000023731	18:14757331-14759082	0	0	0	OK
ENSBTAG00000023752	ENSBTAG00000023752	18:14086351-14089008	0	0	0	OK
ENSBTAG00000023787	ENSBTAG00000023787	11:105837852-105839073	0	0	0	OK
ENSBTAG00000023801	ENSBTAG00000023801	11:102686676-102693228	0	0	0	OK
ENSBTAG00000023823	ENSBTAG00000023823	11:98339923-98340241	0	0	0	OK
ENSBTAG00000023845	ENSBTAG00000023845	26:43034074-43052353	0	0	0	OK
ENSBTAG00000023860	ENSBTAG00000023860	11:94931693-94932632	0	0	0	OK
ENSBTAG00000023895	ENSBTAG00000023895	17:21513448-21513959	0	0	0	OK
ENSBTAG00000023918	ENSBTAG00000023918	22:58768218-58769892	0	0	0	OK
ENSBTAG00000023964	ENSBTAG00000023964	2:115803381-115803879	0	0	0	OK
ENSBTAG00000023971	ENSBTAG00000023971	26:2899612-2900885	0	0	0	OK
ENSBTAG00000023998	ENSBTAG00000023998	18:45687883-45689249	0	0	0	OK
ENSBTAG00000024003	ENSBTAG00000024003	2:89166305-89166719	0	0	0	OK
ENSBTAG00000024019	ENSBTAG00000024019	11:36563189-36567516	0	0	0	OK
ENSBTAG00000024041	ENSBTAG00000024041	7:22368334-22368762	0	0	0	OK
ENSBTAG00000024042	ENSBTAG00000024042	11:21226394-21234596	0	0	0	OK
ENSBTAG00000024043	ENSBTAG00000024043	5:83355781-83356247	0	0	0	OK
ENSBTAG00000024061	ENSBTAG00000024061	11:11221414-11227722	0	0	0	OK
ENSBTAG00000024080	ENSBTAG00000024080	6:90654020-90654357	0	0	0	OK
ENSBTAG00000024085	ENSBTAG00000024085	3:113204846-113205856	0	0	0	OK

ENSBTAG00000024088	ENSBTAG00000024088	3:110799991-110800501	0	0	0	OK
ENSBTAG00000024125	ENSBTAG00000024125	6:27014752-27015007	0	0	0	OK
ENSBTAG00000024141	ENSBTAG00000024141	13:167858-168738	0	0	0	OK
ENSBTAG00000024144	ENSBTAG00000024144	3:100472498-100483275	0	0	0	OK
ENSBTAG00000024177	ENSBTAG00000024177	23:30760351-30760837	0	0	0	OK
ENSBTAG00000024178	ENSBTAG00000024178	23:30757224-30757615	0	0	0	OK
ENSBTAG00000024179	ENSBTAG00000024179	23:30747807-30748119	0	0	0	OK
ENSBTAG00000024180	ENSBTAG00000024180	23:30741967-30742360	0	0	0	OK
ENSBTAG00000024185	ENSBTAG00000024185	23:30724825-30725137	0	0	0	OK
ENSBTAG00000024188	ENSBTAG00000024188	23:30713381-30713762	0	0	0	OK
ENSBTAG00000024195	ENSBTAG00000024195	3:92779833-92789221	0	0	0	OK
ENSBTAG00000024201	ENSBTAG00000024201	3:89319671-89320157	0	0	0	OK
ENSBTAG00000024206	ENSBTAG00000024206	10:68011046-68011987	0	0	0	OK
ENSBTAG00000024233	ENSBTAG00000024233	8:54950669-54951447	0	0	0	OK
ENSBTAG00000024242	ENSBTAG00000024242	4:106625821-106626356	0	0	0	OK
ENSBTAG00000024275	ENSBTAG00000024275	7:85267806-85556934	0	0	0	OK
ENSBTAG00000024311	ENSBTAG00000024311	21:60886044-60886971	0	0	0	OK
ENSBTAG00000024319	ENSBTAG00000024319	3:29495990-29496518	0	0	0	OK
ENSBTAG00000024334	ENSBTAG00000024334	4:72574388-72574868	0	0	0	OK
ENSBTAG00000024341	ENSBTAG00000024341	4:69349547-69354521	0	0	0	OK
ENSBTAG00000024394	ENSBTAG00000024394	4:35563537-35789896	0	0	0	OK
ENSBTAG00000024397	ENSBTAG00000024397	7:10150807-10151876	0	0	0	OK
ENSBTAG00000024423	ENSBTAG00000024423	15:80602079-80603051	0	0	0	OK
ENSBTAG00000024445	ENSBTAG00000024445	6:81299793-81300203	0	0	0	OK
ENSBTAG00000024453	ENSBTAG00000024453	16:73866648-73868568	0	0	0	OK
ENSBTAG00000024476	ENSBTAG00000024476	3:15667484-15670986	0	0	0	OK
ENSBTAG00000024496	ENSBTAG00000024496	16:53272045-53288451	0	0	0.457584	OK
ENSBTAG00000024503	ENSBTAG00000024503	3:8305543-8308776	0	0	0	OK
ENSBTAG00000024530	ENSBTAG00000024530	27:23016750-23017142	0	0	0	OK
ENSBTAG00000024555	ENSBTAG00000024555	16:32961374-33072297	0	0	0	OK

ENSBTAG00000024560	ENSBTAG00000024560	16:30304539-30359147	0	0	0	OK
ENSBTAG00000024582	ENSBTAG00000024582	10:39735370-39736135	0	0	0	OK
ENSBTAG00000024585	ENSBTAG00000024585	28:12902004-12921748	0	0	0	OK
ENSBTAG00000024604	ENSBTAG00000024604	11:94221938-94319233	0	0	0	OK
ENSBTAG00000024605	ENSBTAG00000024605	17:65042589-65045024	0	0	0	OK
ENSBTAG00000024608	ENSBTAG00000024608	13:59957516-59958130	0	0	0	OK
ENSBTAG00000024611	ENSBTAG00000024611	29:38276110-38285529	0	0	0	OK
ENSBTAG00000024623	ENSBTAG00000024623	1:113594592-113595103	0	0	0	OK
ENSBTAG00000024659	ENSBTAG00000024659	10:24959559-24960058	0	0	0	OK
ENSBTAG00000024675	ENSBTAG00000024675	X:79070577-79071600	0	0	0	OK
ENSBTAG00000024731	ENSBTAG00000024731	29:32860722-32861454	0	0	0	OK
ENSBTAG00000024751	ENSBTAG00000024751	9:88249141-88250182	0	0	0	OK
ENSBTAG00000024765	ENSBTAG00000024765	5:29228770-29229333	0	0	0	OK
ENSBTAG00000024771	ENSBTAG00000024771	5:103458791-103460314	0	0	0	OK
ENSBTAG00000024787	ENSBTAG00000024787	6:108508716-108523815	0	0	0	OK
ENSBTAG00000024788	ENSBTAG00000024788	15:79260538-79261447	0	0	0	OK
ENSBTAG00000024809	ENSBTAG00000024809	7:43823208-43837867	0	0	0	OK
ENSBTAG00000024811	ENSBTAG00000024811	28:44980460-44981830	0	0	0	OK
ENSBTAG00000024901	ENSBTAG00000024901	19:57500201-57504157	0	0	0	OK
ENSBTAG00000024911	ENSBTAG00000024911	26:10464921-10496553	0	0	0	OK
ENSBTAG00000024914	ENSBTAG00000024914	19:54584762-54585281	0	0	0	OK
ENSBTAG00000024975	ENSBTAG00000024975	19:45520280-45526187	0	0	0	OK
ENSBTAG00000024979	ENSBTAG00000024979	19:42896708-42897222	0	0	0	OK
ENSBTAG00000024984	ENSBTAG00000024984	14:72224800-72229478	0	0	0	OK
ENSBTAG00000024991	ENSBTAG00000024991	22:57250456-57257322	0	0	0	OK
ENSBTAG00000024999	ENSBTAG00000024999	26:26000424-26001359	0	0	0	OK
ENSBTAG00000025023	ENSBTAG00000025023	18:65787331-65794000	0	0	0	OK
ENSBTAG00000025042	ENSBTAG00000025042	7:103822590-103823967	0	0	0	OK
ENSBTAG00000025046	ENSBTAG00000025046	19:35025248-35041293	0	0	0	OK
ENSBTAG00000025078	ENSBTAG00000025078	19:33778486-33811728	0	0	0	OK

ENSBTAG00000025108	ENSBTAG00000025108	9:44313103-44313896	0	0	0	OK
ENSBTAG00000025110	ENSBTAG00000025110	4:63933401-63937149	0	0	0	OK
ENSBTAG00000025113	ENSBTAG00000025113	16:8241904-8243480	0	0	0	OK
ENSBTAG00000025121	ENSBTAG00000025121	19:24977283-24978133	0	0	0	OK
ENSBTAG00000025124	ENSBTAG00000025124	7:64233852-64237033	0	0	0	OK
ENSBTAG00000025146	ENSBTAG00000025146	1:121430238-121435262	0	0	0	OK
ENSBTAG00000025167	ENSBTAG00000025167	19:21378076-21382996	0	0	0	OK
ENSBTAG00000025183	ENSBTAG00000025183	19:36610212-36616333	0	0	0	OK
ENSBTAG00000025192	ENSBTAG00000025192	12:90906359-90915170	0	0	0	OK
ENSBTAG00000025202	ENSBTAG00000025202	19:18462776-18463392	0	0	0	OK
ENSBTAG00000025211	ENSBTAG00000025211	12:89339067-89339790	0	0	0	OK
ENSBTAG00000025221	ENSBTAG00000025221	12:82994773-83001410	0	0	0	OK
ENSBTAG00000025226	ENSBTAG00000025226	7:52997526-52998020	0	0	0	OK
ENSBTAG00000025257	ENSBTAG00000025257	19:14653466-14655028	0	0	0	OK
ENSBTAG00000025260	ENSBTAG00000025260	19:14614458-14615311	0	0	0	OK
ENSBTAG00000025274	ENSBTAG00000025274	11:105860109-105862549	0	0	0	OK
ENSBTAG00000025280	ENSBTAG00000025280	23:50603364-50617511	0	0	0	OK
ENSBTAG00000025288	ENSBTAG00000025288	18:64117290-64118229	0	0	0	OK
ENSBTAG00000025317	ENSBTAG00000025317	23:34806598-34818877	0	0	0	OK
ENSBTAG00000025383	ENSBTAG00000025383	12:36491413-36491611	0	0	0	OK
ENSBTAG00000025385	ENSBTAG00000025385	10:83480587-83481217	0	0	0	OK
ENSBTAG00000025402	ENSBTAG00000025402	12:36076411-36105865	0	0	0	OK
ENSBTAG00000025405	ENSBTAG00000025405	10:87110562-87132202	0	0	0	OK
ENSBTAG00000025425	ENSBTAG00000025425	7:40127135-40131892	0	0	0	OK
ENSBTAG00000025430	ENSBTAG00000025430	8:100056482-100056883	0	0	0	OK
ENSBTAG00000025448	ENSBTAG00000025448	7:22745269-22748344	0	0	0	OK
ENSBTAG00000025485	ENSBTAG00000025485	23:24851501-24872822	0	0	0	OK
ENSBTAG00000025502	ENSBTAG00000025502	12:11080475-11116234	0	0	0	OK
ENSBTAG00000025595	ENSBTAG00000025595	22:7111727-7112287	0	0	0	OK
ENSBTAG00000025604	ENSBTAG00000025604	10:22015187-22015810	0	0	0	OK

ENSBTAG00000025617	ENSBTAG00000025617	23:10528443-10544184	0	0	0	OK
ENSBTAG00000025621	ENSBTAG00000025621	3:54445871-54446858	0	0	0	OK
ENSBTAG00000025651	ENSBTAG00000025651	X:6197161-6197546	0	0	0	OK
ENSBTAG00000025755	ENSBTAG00000025755	10:21466336-21494696	0	0	0	OK
ENSBTAG00000025756	ENSBTAG00000025756	8:91639208-91673045	0	0	0	OK
ENSBTAG00000025814	ENSBTAG00000025814	10:19143083-19158799	0	0	0	OK
ENSBTAG00000025822	ENSBTAG00000025822	10:15009939-15019251	0	0	0	OK
ENSBTAG00000025847	ENSBTAG00000025847	1:105226964-105228019	0	0	0	OK
ENSBTAG00000025848	ENSBTAG00000025848	X:91768032-91770790	0	0	0	OK
ENSBTAG00000025882	ENSBTAG00000025882	23:31914983-31915367	0	0	0	OK
ENSBTAG00000025908	ENSBTAG00000025908	7:12007281-12008496	0	0	0	OK
ENSBTAG00000026011	ENSBTAG00000026011	X:13673662-13674034	0	0	0	OK
ENSBTAG00000026034	ENSBTAG00000026034	11:23659701-23660017	0	0	0	OK
ENSBTAG00000026065	ENSBTAG00000026065	28:2854634-2855792	0	0	0	OK
ENSBTAG00000026068	ENSBTAG00000026068	16:2117056-2117707	0	0	0	OK
ENSBTAG00000026078	ENSBTAG00000026078	5:59562260-59563319	0	0	0	OK
ENSBTAG00000026088	ENSBTAG00000026088	5:44572854-44578603	0	0	0	OK
ENSBTAG00000026119	ENSBTAG00000026119	13:74996641-75000946	0	0	0	OK
ENSBTAG00000026157	ENSBTAG00000026157	14:50606042-50606353	0	0	0	OK
ENSBTAG00000026163	ENSBTAG00000026163	23:26354053-26354780	0	0	0	OK
ENSBTAG00000026185	ENSBTAG00000026185	26:50636585-50638962	0	0	0	OK
ENSBTAG00000026231	ENSBTAG00000026231	14:72800008-72800402	0	0	0	OK
ENSBTAG00000026243	ENSBTAG00000026243	25:36643094-36644330	0	0	0	OK
ENSBTAG00000026254	ENSBTAG00000026254	14:48940215-48959150	0	0	0	OK
ENSBTAG00000026259	ENSBTAG00000026259	1:421944-422280	0	0	0	OK
ENSBTAG00000026260	ENSBTAG00000026260	1:484626-492432	0	0	0	OK
ENSBTAG00000026273	ENSBTAG00000026273	25:35734570-35743074	0	0	0	OK
ENSBTAG00000026306	ENSBTAG00000026306	14:23067158-23068103	0	0	0	OK
ENSBTAG00000026319	ENSBTAG00000026319	25:26614814-26618147	0	0	0	OK
ENSBTAG00000026322	ENSBTAG00000026322	5:44652534-44656613	0	0	0	OK

ENSBTAG00000026327	ENSBTAG00000026327	14:1505029-1507633	0	0	0	OK
ENSBTAG00000026340	ENSBTAG00000026340	14:2915387-2937057	0	0	0	OK
ENSBTAG00000026344	ENSBTAG00000026344	14:2426856-2427912	0	0	0	OK
ENSBTAG00000026356	ENSBTAG00000026356	14:1795350-1804562	0	0	0	OK
ENSBTAG00000026371	ENSBTAG00000026371	25:10743975-11273969	0	0	0	OK
ENSBTAG00000026376	ENSBTAG00000026376	25:10417664-10425321	0	0	0	OK
ENSBTAG00000026394	ENSBTAG00000026394	25:3744166-3749205	0	0	0	OK
ENSBTAG00000026415	ENSBTAG00000026415	25:1349892-1351145	0	0	0	OK
ENSBTAG00000026417	ENSBTAG00000026417	25:214005-214773	0	0	0	OK
ENSBTAG00000026418	ENSBTAG00000026418	25:210951-211719	0	0	0	OK
ENSBTAG00000026437	ENSBTAG00000026437	17:39881229-39884057	0	0	0	OK
ENSBTAG00000026489	ENSBTAG00000026489	5:113437813-113438567	0	0	0	OK
ENSBTAG00000026495	ENSBTAG00000026495	5:110923000-110924709	0	0	0	OK
ENSBTAG00000026497	ENSBTAG00000026497	5:114071761-114072868	0	0	0	OK
ENSBTAG00000026505	ENSBTAG00000026505	20:21948511-21948937	0	0	0	OK
ENSBTAG00000026515	ENSBTAG00000026515	27:31188488-31188877	0	0	0	OK
ENSBTAG00000026522	ENSBTAG00000026522	5:105562078-105568210	0	0	0	OK
ENSBTAG00000026604	ENSBTAG00000026604	9:61434664-61436740	0	0	0	OK
ENSBTAG00000026645	ENSBTAG00000026645	19:56993407-56999724	0	0	0	OK
ENSBTAG00000026650	ENSBTAG00000026650	22:54722904-54723165	0	0	0	OK
ENSBTAG00000026710	ENSBTAG00000026710	2:119657308-119664584	0	0	0	OK
ENSBTAG00000026732	ENSBTAG00000026732	17:64899651-64902627	0	0	0	OK
ENSBTAG00000026768	ENSBTAG00000026768	29:50027264-50034123	0	0	0	OK
ENSBTAG00000026769	ENSBTAG00000026769	29:49955759-49957635	0	0	0	OK
ENSBTAG00000026809	ENSBTAG00000026809	11:21030607-21031350	0	0	0	OK
ENSBTAG00000026822	ENSBTAG00000026822	2:74044251-74045343	0	0	0	OK
ENSBTAG00000026825	ENSBTAG00000026825	2:71607733-71613808	0	0	0	OK
ENSBTAG00000026836	ENSBTAG00000026836	1:45667460-45822623	0	0	0	OK
ENSBTAG00000026848	ENSBTAG00000026848	3:11468020-11468512	0	0	0	OK
ENSBTAG00000026861	ENSBTAG00000026861	12:2067557-2068642	0	0	0	OK

ENSBTAG00000026880	ENSBTAG00000026880	5:27711063-27729751	0	0	0	OK
ENSBTAG00000026882	ENSBTAG00000026882	2:30091988-30092690	0	0	0	OK
ENSBTAG00000026886	ENSBTAG00000026886	21:70113044-70118400	0	0	0	OK
ENSBTAG00000026936	ENSBTAG00000026936	6:14171356-14198589	0	0	0	OK
ENSBTAG00000026992	ENSBTAG00000026992	8:86988543-86988998	0	0	0	OK
ENSBTAG00000026993	ENSBTAG00000026993	5:47877714-47894116	0	0	0	OK
ENSBTAG00000027049	ENSBTAG00000027049	9:9903137-9933669	0	0	0	OK
ENSBTAG00000027052	ENSBTAG00000027052	X:51424184-51425182	0	0	0	OK
ENSBTAG00000027075	ENSBTAG00000027075	21:14304504-14304970	0	0	0	OK
ENSBTAG00000027113	ENSBTAG00000027113	1:42119013-42119928	0	0	0	OK
ENSBTAG00000027170	ENSBTAG00000027170	10:24734911-24735442	0	0	0	OK
ENSBTAG00000027221	ENSBTAG00000027221	13:54047558-54048023	0	0	0	OK
ENSBTAG00000027225	ENSBTAG00000027225	27:5124122-5125987	0	0	0	OK
ENSBTAG00000027246	ENSBTAG00000027246	23:28918427-28920742	0	0	0	OK
ENSBTAG00000027251	ENSBTAG00000027251	10:65355115-65357062	0	0	0	OK
ENSBTAG00000027279	ENSBTAG00000027279	23:29411405-29412041	0	0	0	OK
ENSBTAG00000027319	ENSBTAG00000027319	4:8566241-8568524	0	0	0	OK
ENSBTAG00000027328	ENSBTAG00000027328	13:75885058-75888911	0	0	0	OK
ENSBTAG00000027348	ENSBTAG00000027348	15:84427063-84434956	0	0	0	OK
ENSBTAG00000027361	ENSBTAG00000027361	13:65563082-65564614	0	0	0	OK
ENSBTAG00000027383	ENSBTAG00000027383	13:61572837-61577455	0	0	0	OK
ENSBTAG00000027384	ENSBTAG00000027384	13:61562052-61566096	0	0	0	OK
ENSBTAG00000027386	ENSBTAG00000027386	13:61234713-61235657	0	0	0	OK
ENSBTAG00000027396	ENSBTAG00000027396	7:66385715-66386142	0	0	0	OK
ENSBTAG00000027397	ENSBTAG00000027397	13:57120665-57121565	0	0	0	OK
ENSBTAG00000027412	ENSBTAG00000027412	13:51930066-51930888	0	0	0	OK
ENSBTAG00000027416	ENSBTAG00000027416	13:43493943-43494348	0	0	0	OK
ENSBTAG00000027420	ENSBTAG00000027420	13:42488774-42493105	0	0	0	OK
ENSBTAG00000027426	ENSBTAG00000027426	16:64843772-64844924	0	0	0	OK
ENSBTAG00000027489	ENSBTAG00000027489	15:83994306-83995251	0	0	0	OK

ENSBTAG00000027525	ENSBTAG00000027525	4:79255148-79256324	0	0	0	OK
ENSBTAG00000027562	ENSBTAG00000027562	15:76813866-76814512	0	0	0	OK
ENSBTAG00000027563	ENSBTAG00000027563	15:75154392-75187019	0	0	0	OK
ENSBTAG00000027577	ENSBTAG00000027577	15:65131003-65132948	0	0	0	OK
ENSBTAG00000027578	ENSBTAG00000027578	15:64807909-64808301	0	0	0	OK
ENSBTAG00000027610	ENSBTAG00000027610	15:54468006-54468423	0	0	0	OK
ENSBTAG00000027626	ENSBTAG00000027626	27:36108532-36125175	0	0	0	OK
ENSBTAG00000027629	ENSBTAG00000027629	27:36255858-36265466	0	0	0	OK
ENSBTAG00000027630	ENSBTAG00000027630	6:23289383-23302661	0	0	0	OK
ENSBTAG00000027651	ENSBTAG00000027651	15:48812518-48813463	0	0	0	OK
ENSBTAG00000027653	ENSBTAG00000027653	15:48822546-48823484	0	0	0	OK
ENSBTAG00000027654	ENSBTAG00000027654	27:32951593-32973435	0	0	0	OK
ENSBTAG00000027655	ENSBTAG00000027655	6:14411561-14419292	0	0	0	OK
ENSBTAG00000027699	ENSBTAG00000027699	7:9260885-9261821	0	0	0	OK
ENSBTAG00000027716	ENSBTAG00000027716	15:42332730-42333027	0	0	0	OK
ENSBTAG00000027723	ENSBTAG00000027723	15:43886242-43887140	0	0	0	OK
ENSBTAG00000027759	ENSBTAG00000027759	15:30714144-30716388	0	0	0	OK
ENSBTAG00000027766	ENSBTAG00000027766	15:30448398-30450337	0	0	0	OK
ENSBTAG00000027768	ENSBTAG00000027768	7:43507540-43508496	0	0	0	OK
ENSBTAG00000027786	ENSBTAG00000027786	11:35028763-35029620	0	0	0	OK
ENSBTAG00000027787	ENSBTAG00000027787	18:65621141-65621509	0	0	0	OK
ENSBTAG00000027803	ENSBTAG00000027803	X:79506819-79508013	0	0	0	OK
ENSBTAG00000027809	ENSBTAG00000027809	16:56941659-57039130	0	0	0	OK
ENSBTAG00000027863	ENSBTAG00000027863	21:40890192-40890804	0	0	0	OK
ENSBTAG00000027865	ENSBTAG00000027865	20:24048536-24059238	0	0	0	OK
ENSBTAG00000027878	ENSBTAG00000027878	7:6445373-6445871	0	0	0	OK
ENSBTAG00000027879	ENSBTAG00000027879	9:102880276-102893601	0	0	0	OK
ENSBTAG00000027911	ENSBTAG00000027911	7:42312179-42313388	0	0	0	OK
ENSBTAG00000027924	ENSBTAG00000027924	20:24570226-24596518	0	0	0	OK
ENSBTAG00000027942	ENSBTAG00000027942	15:83027016-83028426	0	0	0	OK

ENSBTAG00000027955	ENSBTAG00000027955	23:29451996-29452938	0	0	0	OK
ENSBTAG00000027962	ENSBTAG00000027962	2:112675321-112675995	0	0	0	OK
ENSBTAG00000028022	ENSBTAG00000028022	16:16739077-16739198	0	0	0	OK
ENSBTAG00000028036	ENSBTAG00000028036	3:23105493-23105657	0	0	0	OK
ENSBTAG00000028043	ENSBTAG00000028043	21:63716089-63716242	0	0	0	OK
ENSBTAG00000028061	ENSBTAG00000028061	10:9218606-9218717	0	0	0	OK
ENSBTAG00000028065	ENSBTAG00000028065	6:62581479-62581594	0	0	0	OK
ENSBTAG00000028094	ENSBTAG00000028094	24:11157560-11157751	0	0	0	OK
ENSBTAG00000028103	ENSBTAG00000028103	5:85914708-85914817	0	0	0	OK
ENSBTAG00000028105	ENSBTAG00000028105	25:7317053-7317191	0	0	0	OK
ENSBTAG00000028119	ENSBTAG00000028119	2:40593051-40593207	0	0	0	OK
ENSBTAG00000028153	ENSBTAG00000028153	24:46051053-46051160	0	0	0	OK
ENSBTAG00000028229	ENSBTAG00000028229	3:9952211-9952373	0	0	0	OK
ENSBTAG00000028255	ENSBTAG00000028255	3:99385487-99385595	0	0	0	OK
ENSBTAG00000028273	ENSBTAG00000028273	16:38305775-38305893	0	0	0	OK
ENSBTAG00000028324	ENSBTAG00000028324	21:42557932-42558055	0	0	0	OK
ENSBTAG00000028330	ENSBTAG00000028330	3:46837608-46837822	0	0	0	OK
ENSBTAG00000028336	ENSBTAG00000028336	18:8443110-8443227	0	0	0	OK
ENSBTAG00000028355	ENSBTAG00000028355	21:55796999-55797118	0	0	0	OK
ENSBTAG00000028358	ENSBTAG00000028358	7:53516526-53516623	0	0	0	OK
ENSBTAG00000028359	ENSBTAG00000028359	19:9798917-9799131	0	0	0	OK
ENSBTAG00000028362	ENSBTAG00000028362	10:91894376-91894482	0	0	0	OK
ENSBTAG00000028373	ENSBTAG00000028373	7:111118342-111118494	0	0	0	OK
ENSBTAG00000028375	ENSBTAG00000028375	9:92428961-92429070	0	0	0	OK
ENSBTAG00000028419	ENSBTAG00000028419	21:45400517-45400676	0	0	0	OK
ENSBTAG00000028421	ENSBTAG00000028421	19:15193840-15194001	0	0	0	OK
ENSBTAG00000028430	ENSBTAG00000028430	18:43698038-43698153	0	0	0	OK
ENSBTAG00000028457	ENSBTAG00000028457	4:89006560-89006664	0	0	0	OK
ENSBTAG00000028463	ENSBTAG00000028463	6:4903634-4903741	0	0	0	OK
ENSBTAG00000028470	ENSBTAG00000028470	18:19597431-19597548	0	0	0	OK

ENSBTAG00000028471	ENSBTAG00000028471	22:8077308-8077405	0	0	0	OK
ENSBTAG00000028498	ENSBTAG00000028498	7:41231561-41231683	0	0	0	OK
ENSBTAG00000028556	ENSBTAG00000028556	3:22834235-22834402	0	0	0	OK
ENSBTAG00000028572	ENSBTAG00000028572	2:34923409-34923520	0	0	0	OK
ENSBTAG00000028575	ENSBTAG00000028575	24:28822882-28823043	0	0	0	OK
ENSBTAG00000028582	ENSBTAG00000028582	1:42481846-42482009	0	0	0	OK
ENSBTAG00000028588	ENSBTAG00000028588	10:6669362-6669499	0	0	0	OK
ENSBTAG00000028616	ENSBTAG00000028616	9:86836801-86836911	0	0	0	OK
ENSBTAG00000028620	ENSBTAG00000028620	22:2565862-2565970	0	0	0	OK
ENSBTAG00000028628	ENSBTAG00000028628	11:57242753-57242916	0	0	0	OK
ENSBTAG00000028631	ENSBTAG00000028631	24:43002878-43002997	0	0	0	OK
ENSBTAG00000028641	ENSBTAG00000028641	20:27866522-27866632	0	0	0	OK
ENSBTAG00000028668	ENSBTAG00000028668	7:89140310-89140422	0	0	0	OK
ENSBTAG00000028669	ENSBTAG00000028669	13:65689871-65689978	0	0	0	OK
ENSBTAG00000028675	ENSBTAG00000028675	6:46501971-46502082	0	0	0	OK
ENSBTAG00000028682	ENSBTAG00000028682	29:47825555-47825673	0	0	0	OK
ENSBTAG00000028683	ENSBTAG00000028683	5:90424776-90424893	0	0	0	OK
ENSBTAG00000028692	ENSBTAG00000028692	19:50079701-50079819	0	0	0	OK
ENSBTAG00000028696	ENSBTAG00000028696	19:9827637-9827847	0	0	0	OK
ENSBTAG00000028700	ENSBTAG00000028700	14:33207210-33207319	0	0	0	OK
ENSBTAG00000028704	ENSBTAG00000028704	7:27257902-27258023	0	0	0	OK
ENSBTAG00000028707	ENSBTAG00000028707	2:134259753-134259966	0	0	0	OK
ENSBTAG00000028794	ENSBTAG00000028794	23:37388761-37388880	0	0	0	OK
ENSBTAG00000028809	ENSBTAG00000028809	2:94467706-94467870	0	0	0	OK
ENSBTAG00000028811	ENSBTAG00000028811	4:104194264-104194479	0	0	0	OK
ENSBTAG00000028813	ENSBTAG00000028813	1:24669305-24669424	0	0	0	OK
ENSBTAG00000028814	ENSBTAG00000028814	10:77300496-77300602	0	0	0	OK
ENSBTAG00000028847	ENSBTAG00000028847	24:30805145-30805263	0	0	0	OK
ENSBTAG00000028857	ENSBTAG00000028857	3:99742714-99742852	0	0	0	OK
ENSBTAG00000028860	ENSBTAG00000028860	4:114202277-114202408	0	0	0	OK

ENSBTAG00000028880	ENSBTAG00000028880	16:58216049-58216160	0	0	0	OK
ENSBTAG00000028889	ENSBTAG00000028889	14:24970515-24970679	0	0	0	OK
ENSBTAG00000028898	ENSBTAG00000028898	8:92439946-92440065	0	0	0	OK
ENSBTAG00000028919	ENSBTAG00000028919	9:74586366-74586481	0	0	0	OK
ENSBTAG00000028923	ENSBTAG00000028923	8:33318601-33318744	0	0	0	OK
ENSBTAG00000028938	ENSBTAG00000028938	X:81639522-81639633	0	0	0	OK
ENSBTAG00000028976	ENSBTAG00000028976	25:42114157-42114265	0	0	0	OK
ENSBTAG00000028980	ENSBTAG00000028980	6:7250044-7250158	0	0	0	OK
ENSBTAG00000028988	ENSBTAG00000028988	16:65966750-65966879	0	0	0	OK
ENSBTAG00000028990	ENSBTAG00000028990	5:47602842-47603007	0	0	0	OK
ENSBTAG00000029007	ENSBTAG00000029007	16:74707386-74707565	0	0	0	OK
ENSBTAG00000029040	ENSBTAG00000029040	18:40195466-40195556	0	0	0	OK
ENSBTAG00000029055	ENSBTAG00000029055	7:63563543-63563704	0	0	0	OK
ENSBTAG00000029069	ENSBTAG00000029069	17:55562619-55562800	0	0	0	OK
ENSBTAG00000029075	ENSBTAG00000029075	8:69506898-69507006	0	0	0	OK
ENSBTAG00000029080	ENSBTAG00000029080	8:75108589-75108708	0	0	0	OK
ENSBTAG00000029086	ENSBTAG00000029086	21:1302797-1302923	0	0	0	OK
ENSBTAG00000029106	ENSBTAG00000029106	1:82250679-82250794	0	0	0	OK
ENSBTAG00000029114	ENSBTAG00000029114	24:11711408-11711534	0	0	0	OK
ENSBTAG00000029115	ENSBTAG00000029115	8:64096203-64096322	0	0	0	OK
ENSBTAG00000029119	ENSBTAG00000029119	16:66901986-66902096	0	0	0	OK
ENSBTAG00000029126	ENSBTAG00000029126	5:89609740-89609850	0	0	0	OK
ENSBTAG00000029127	ENSBTAG00000029127	17:40930349-40930461	0	0	0	OK
ENSBTAG00000029159	ENSBTAG00000029159	14:58668405-58668522	0	0	0	OK
ENSBTAG00000029168	ENSBTAG00000029168	6:3875372-3875586	0	0	0	OK
ENSBTAG00000029173	ENSBTAG00000029173	23:16386088-16386203	0	0	0	OK
ENSBTAG00000029179	ENSBTAG00000029179	12:19083249-19083355	0	0	0	OK
ENSBTAG00000029206	ENSBTAG00000029206	7:16731170-16731290	0	0	0	OK
ENSBTAG00000029209	ENSBTAG00000029209	5:108007776-108007881	0	0	0	OK
ENSBTAG00000029237	ENSBTAG00000029237	4:113107109-113107230	0	0	0	OK

ENSBTAG00000029250	ENSBTAG00000029250	7:47929941-47930060	0	0	0	OK
ENSBTAG00000029283	ENSBTAG00000029283	7:51745433-51745547	0	0	0	OK
ENSBTAG00000029291	ENSBTAG00000029291	25:7564681-7564794	0	0	0	OK
ENSBTAG00000029292	ENSBTAG00000029292	4:40887315-40887433	0	0	0	OK
ENSBTAG00000029295	ENSBTAG00000029295	19:22856213-22856325	0	0	0	OK
ENSBTAG00000029303	ENSBTAG00000029303	5:19683028-19683146	0	0	0	OK
ENSBTAG00000029316	ENSBTAG00000029316	7:68693466-68693599	0	0	0	OK
ENSBTAG00000029317	ENSBTAG00000029317	18:58436584-58436710	0	0	0	OK
ENSBTAG00000029343	ENSBTAG00000029343	5:111866511-111866634	0	0	0	OK
ENSBTAG00000029403	ENSBTAG00000029403	22:1243087-1243229	0	0	0	OK
ENSBTAG00000029452	ENSBTAG00000029452	1:93356879-93356990	0	0	0	OK
ENSBTAG00000029461	ENSBTAG00000029461	15:71047555-71047665	0	0	0	OK
ENSBTAG00000029474	ENSBTAG00000029474	23:8607361-8607480	0	0	0	OK
ENSBTAG00000029491	ENSBTAG00000029491	17:54130953-54131087	0	0	3.0904	OK
ENSBTAG00000029512	ENSBTAG00000029512	10:43211765-43211926	0	0	0	OK
ENSBTAG00000029540	ENSBTAG00000029540	3:51058571-51058690	0	0	0	OK
ENSBTAG00000029544	ENSBTAG00000029544	10:81528970-81529108	0	0	0	OK
ENSBTAG00000029569	ENSBTAG00000029569	14:16832033-16832155	0	0	0	OK
ENSBTAG00000029576	ENSBTAG00000029576	4:115995077-115995189	0	0	0	OK
ENSBTAG00000029583	ENSBTAG00000029583	20:4806230-4806349	0	0	0	OK
ENSBTAG00000029592	ENSBTAG00000029592	X:148538916-148539037	0	0	0	OK
ENSBTAG00000029625	ENSBTAG00000029625	6:50213887-50214005	0	0	0	OK
ENSBTAG00000029640	ENSBTAG00000029640	18:14877691-14877845	0	0	0	OK
ENSBTAG00000029651	ENSBTAG00000029651	20:19840989-19841103	0	0	0	OK
ENSBTAG00000029663	ENSBTAG00000029663	11:56977657-56977781	0	0	0	OK
ENSBTAG00000029683	ENSBTAG00000029683	12:4035319-4035486	0	0	0	OK
ENSBTAG00000029713	ENSBTAG00000029713	5:115017093-115017212	0	0	0	OK
ENSBTAG00000029760	ENSBTAG00000029760	20:15213923-15214005	0	0	0	OK
ENSBTAG00000029761	ENSBTAG00000029761	8:70060383-70060465	0	0	0	OK
ENSBTAG00000029762	ENSBTAG00000029762	4:94411430-94411500	0	0	0	OK

ENSBTAG00000029763	ENSBTAG00000029763	2:107667515-107667585	0	0	0	OK
ENSBTAG00000029764	ENSBTAG00000029764	12:66226835-66226917	0	0	0	OK
ENSBTAG00000029765	ENSBTAG00000029765	16:77478032-77478113	0	0	0	OK
ENSBTAG00000029768	ENSBTAG00000029768	1:19931184-19931265	0	0	0	OK
ENSBTAG00000029769	ENSBTAG00000029769	17:74967065-74967144	0	0	0	OK
ENSBTAG00000029770	ENSBTAG00000029770	21:67564783-67564886	0	0	0	OK
ENSBTAG00000029771	ENSBTAG00000029771	18:37088136-37088230	0	0	0	OK
ENSBTAG00000029772	ENSBTAG00000029772	5:117119384-117119458	0	0	0	OK
ENSBTAG00000029774	ENSBTAG00000029774	X:96383531-96383614	0	0	0	OK
ENSBTAG00000029775	ENSBTAG00000029775	19:52189628-52189720	0	0	0	OK
ENSBTAG00000029776	ENSBTAG00000029776	8:9153379-9153464	0	0	0	OK
ENSBTAG00000029777	ENSBTAG00000029777	7:1146987-1147082	0	0	0	OK
ENSBTAG00000029778	ENSBTAG00000029778	8:100305159-100305229	0	0	0	OK
ENSBTAG00000029781	ENSBTAG00000029781	X:17918526-17918607	0	0	0	OK
ENSBTAG00000029782	ENSBTAG00000029782	21:67457236-67457311	0	0	0	OK
ENSBTAG00000029783	ENSBTAG00000029783	6:41545028-41545138	0	0	0	OK
ENSBTAG00000029785	ENSBTAG00000029785	X:17918007-17918090	0	0	0	OK
ENSBTAG00000029787	ENSBTAG00000029787	19:10349505-10349590	0	0	0	OK
ENSBTAG00000029788	ENSBTAG00000029788	5:26199800-26199885	0	0	0	OK
ENSBTAG00000029789	ENSBTAG00000029789	21:67593240-67593348	0	0	0	OK
ENSBTAG00000029790	ENSBTAG00000029790	13:55237543-55237619	0	0	0	OK
ENSBTAG00000029791	ENSBTAG00000029791	21:67592845-67592971	0	0	0	OK
ENSBTAG00000029792	ENSBTAG00000029792	21:67416594-67416709	0	0	0	OK
ENSBTAG00000029793	ENSBTAG00000029793	13:51742416-51742496	0	0	0	OK
ENSBTAG00000029794	ENSBTAG00000029794	21:67603522-67603629	0	0	0	OK
ENSBTAG00000029796	ENSBTAG00000029796	24:21530899-21531008	0	0	0	OK
ENSBTAG00000029797	ENSBTAG00000029797	3:74462294-74462380	0	0	0	OK
ENSBTAG00000029798	ENSBTAG00000029798	X:92886500-92886586	0	0	0	OK
ENSBTAG00000029800	ENSBTAG00000029800	4:95070992-95071084	0	0	0	OK
ENSBTAG00000029802	ENSBTAG00000029802	12:66227254-66227334	0	0	0	OK

ENSBTAG00000029803	ENSBTAG00000029803	X:103538834-103538944	0	0	0	OK
ENSBTAG00000029804	ENSBTAG00000029804	9:10768280-10768347	0	0	0	OK
ENSBTAG00000029805	ENSBTAG00000029805	3:106059357-106059462	0	0	0	OK
ENSBTAG00000029807	ENSBTAG00000029807	X:18179069-18179151	0	0	0	OK
ENSBTAG00000029808	ENSBTAG00000029808	21:67603387-67603477	0	0	0	OK
ENSBTAG00000029809	ENSBTAG00000029809	3:45448507-45448609	0	0	0	OK
ENSBTAG00000029810	ENSBTAG00000029810	X:74328421-74328492	0	0	0	OK
ENSBTAG00000029811	ENSBTAG00000029811	7:63067300-63067366	0	0	0	OK
ENSBTAG00000029812	ENSBTAG00000029812	19:27571340-27571429	0	0	0	OK
ENSBTAG00000029814	ENSBTAG00000029814	22:14870572-14870669	0	0	0	OK
ENSBTAG00000029815	ENSBTAG00000029815	16:3031950-3032045	0	0	0	OK
ENSBTAG00000029817	ENSBTAG00000029817	25:36892248-36892325	0	0	0	OK
ENSBTAG00000029818	ENSBTAG00000029818	X:34664589-34664670	0	0	0	OK
ENSBTAG00000029820	ENSBTAG00000029820	21:67565467-67565553	0	0	0	OK
ENSBTAG00000029822	ENSBTAG00000029822	19:21799471-21799580	0	0	0	OK
ENSBTAG00000029825	ENSBTAG00000029825	22:49142027-49142117	0	0	0	OK
ENSBTAG00000029826	ENSBTAG00000029826	23:24312329-24312413	0	0	0	OK
ENSBTAG00000029827	ENSBTAG00000029827	1:19881346-19881431	0	0	0	OK
ENSBTAG00000029828	ENSBTAG00000029828	X:82024078-82024163	0	0	0	OK
ENSBTAG00000029829	ENSBTAG00000029829	21:67431417-67431509	0	0	4.50124	OK
ENSBTAG00000029830	ENSBTAG00000029830	7:74185034-74185133	0	0	0	OK
ENSBTAG00000029833	ENSBTAG00000029833	15:55314008-55314103	0	0	0	OK
ENSBTAG00000029836	ENSBTAG00000029836	9:10730928-10731012	0	0	0	OK
ENSBTAG00000029839	ENSBTAG00000029839	7:12915071-12915168	0	0	0	OK
ENSBTAG00000029841	ENSBTAG00000029841	11:95709410-95709520	0	0	0	OK
ENSBTAG00000029842	ENSBTAG00000029842	28:41329054-41329149	0	0	0	OK
ENSBTAG00000029843	ENSBTAG00000029843	26:11287989-11288076	0	0	0	OK
ENSBTAG00000029844	ENSBTAG00000029844	18:58015534-58015620	0	0	0	OK
ENSBTAG00000029845	ENSBTAG00000029845	21:67563097-67563179	0	0	0	OK
ENSBTAG00000029846	ENSBTAG00000029846	8:83009822-83009919	0	0	0	OK

ENSBTAG00000029847	ENSBTAG00000029847	12:66226695-66226767	0	0	0	OK
ENSBTAG00000029848	ENSBTAG00000029848	25:13344233-13344320	0	0	0	OK
ENSBTAG00000029849	ENSBTAG00000029849	16:79685931-79686041	0	0	0	OK
ENSBTAG00000029850	ENSBTAG00000029850	2:107133393-107133478	0	0	0	OK
ENSBTAG00000029851	ENSBTAG00000029851	24:58095641-58095726	0	0	0	OK
ENSBTAG00000029852	ENSBTAG00000029852	5:25849763-25849853	0	0	0	OK
ENSBTAG00000029853	ENSBTAG00000029853	8:86887430-86887517	0	0	0	OK
ENSBTAG00000029854	ENSBTAG00000029854	16:75799965-75800034	0	0	0	OK
ENSBTAG00000029855	ENSBTAG00000029855	3:80666416-80666499	0	0	0	OK
ENSBTAG00000029856	ENSBTAG00000029856	15:22134724-22134808	0	0	0	OK
ENSBTAG00000029857	ENSBTAG00000029857	7:62810732-62810820	0	0	0	OK
ENSBTAG00000029859	ENSBTAG00000029859	15:52956181-52956249	0	0	0	OK
ENSBTAG00000029860	ENSBTAG00000029860	12:66227004-66227075	0	0	0	OK
ENSBTAG00000029861	ENSBTAG00000029861	22:49189339-49189422	0	0	0	OK
ENSBTAG00000029862	ENSBTAG00000029862	8:47259538-47259634	0	0	0	OK
ENSBTAG00000029863	ENSBTAG00000029863	10:21369590-21369673	0	0	0	OK
ENSBTAG00000029864	ENSBTAG00000029864	X:22083688-22083771	0	0	0	OK
ENSBTAG00000029865	ENSBTAG00000029865	14:4030314-4030394	0	0	0	OK
ENSBTAG00000029867	ENSBTAG00000029867	12:19596345-19596428	0	0	0	OK
ENSBTAG00000029868	ENSBTAG00000029868	19:27441651-27441763	0	0	0	OK
ENSBTAG00000029869	ENSBTAG00000029869	5:55977922-55978006	0	0	0	OK
ENSBTAG00000029870	ENSBTAG00000029870	X:96382627-96382746	0	0	0	OK
ENSBTAG00000029871	ENSBTAG00000029871	3:106062491-106062583	0	0	0	OK
ENSBTAG00000029875	ENSBTAG00000029875	X:35018700-35018781	0	0	0	OK
ENSBTAG00000029877	ENSBTAG00000029877	4:119541696-119541783	0	0	0	OK
ENSBTAG00000029878	ENSBTAG00000029878	X:92886826-92886912	0	0	0	OK
ENSBTAG00000029880	ENSBTAG00000029880	18:58015035-58015114	0	0	0	OK
ENSBTAG00000029881	ENSBTAG00000029881	15:33347567-33347640	0	0	0	OK
ENSBTAG00000029884	ENSBTAG00000029884	4:95402307-95402393	0	0	0	OK
ENSBTAG00000029885	ENSBTAG00000029885	X:18179220-18179312	0	0	0	OK

ENSBTAG00000029886	ENSBTAG00000029886	16:40485806-40485916	0	0	0	OK
ENSBTAG00000029887	ENSBTAG00000029887	21:66867085-66867176	0	0	0	OK
ENSBTAG00000029889	ENSBTAG00000029889	X:34665623-34665708	0	0	0	OK
ENSBTAG00000029890	ENSBTAG00000029890	7:16508920-16509000	0	0	0	OK
ENSBTAG00000029891	ENSBTAG00000029891	17:74100538-74100620	0	0	0	OK
ENSBTAG00000029892	ENSBTAG00000029892	19:38549253-38549362	0	0	0	OK
ENSBTAG00000029893	ENSBTAG00000029893	7:90554676-90554763	0	0	0	OK
ENSBTAG00000029895	ENSBTAG00000029895	20:444315-444379	0	0	0	OK
ENSBTAG00000029896	ENSBTAG00000029896	11:95710625-95710714	0	0	0	OK
ENSBTAG00000029897	ENSBTAG00000029897	19:11033061-11033153	0	0	0	OK
ENSBTAG00000029898	ENSBTAG00000029898	16:59666088-59666184	0	0	0	OK
ENSBTAG00000029901	ENSBTAG00000029901	7:12981969-12982042	0	0	0	OK
ENSBTAG00000029902	ENSBTAG00000029902	8:105147046-105147142	0	0	0	OK
ENSBTAG00000029903	ENSBTAG00000029903	11:38494725-38494834	0	0	0	OK
ENSBTAG00000029904	ENSBTAG00000029904	X:103538140-103538250	0	0	0	OK
ENSBTAG00000029905	ENSBTAG00000029905	19:23651139-23651240	0	0	0	OK
ENSBTAG00000029906	ENSBTAG00000029906	24:34844414-34844501	0	0	0	OK
ENSBTAG00000029907	ENSBTAG00000029907	21:67600489-67600558	0	0	0	OK
ENSBTAG00000029909	ENSBTAG00000029909	8:78575530-78575639	0	0	0	OK
ENSBTAG00000029910	ENSBTAG00000029910	X:17918132-17918201	0	0	0	OK
ENSBTAG00000029911	ENSBTAG00000029911	21:67431233-67431323	0	0	4.60127	OK
ENSBTAG00000029912	ENSBTAG00000029912	1:19930453-19930547	0	0	0	OK
ENSBTAG00000029913	ENSBTAG00000029913	22:51543951-51544038	0	0	0	OK
ENSBTAG00000029914	ENSBTAG00000029914	19:18811261-18811372	0	0	0	OK
ENSBTAG00000029915	ENSBTAG00000029915	4:93220882-93220966	0	0	0	OK
ENSBTAG00000029916	ENSBTAG00000029916	1:107923235-107923328	0	0	0	OK
ENSBTAG00000029917	ENSBTAG00000029917	5:62117708-62117808	0	0	0	OK
ENSBTAG00000029918	ENSBTAG00000029918	18:58014867-58014937	0	0	0	OK
ENSBTAG00000029920	ENSBTAG00000029920	25:36892448-36892532	0	0	0	OK
ENSBTAG00000029921	ENSBTAG00000029921	3:33964963-33965044	0	0	0	OK

ENSBTAG00000029922	ENSBTAG00000029922	21:21250100-21250190	0	0	0	OK
ENSBTAG00000029924	ENSBTAG00000029924	16:45197389-45197496	0	0	0	OK
ENSBTAG00000029925	ENSBTAG00000029925	15:33298814-33298902	0	0	0	OK
ENSBTAG00000029926	ENSBTAG00000029926	24:34841095-34841180	0	0	0	OK
ENSBTAG00000029927	ENSBTAG00000029927	2:20797605-20797704	0	0	0	OK
ENSBTAG00000029929	ENSBTAG00000029929	1:107923378-107923476	0	0	0	OK
ENSBTAG00000029930	ENSBTAG00000029930	19:23382428-23382523	0	0	0	OK
ENSBTAG00000029931	ENSBTAG00000029931	16:40491601-40491705	0	0	0	OK
ENSBTAG00000029932	ENSBTAG00000029932	8:39940825-39940916	0	0	0	OK
ENSBTAG00000029933	ENSBTAG00000029933	18:53654145-53654239	0	0	0	OK
ENSBTAG00000029935	ENSBTAG00000029935	8:86885224-86885311	0	0	0	OK
ENSBTAG00000029937	ENSBTAG00000029937	22:11457899-11457989	0	0	0	OK
ENSBTAG00000029938	ENSBTAG00000029938	18:24992695-24992779	0	0	0	OK
ENSBTAG00000029939	ENSBTAG00000029939	15:74545184-74545278	0	0	0	OK
ENSBTAG00000029940	ENSBTAG00000029940	29:43731659-43731765	0	0	0	OK
ENSBTAG00000029942	ENSBTAG00000029942	16:77478592-77478680	0	0	0	OK
ENSBTAG00000029945	ENSBTAG00000029945	4:70414250-70414318	0	0	0	OK
ENSBTAG00000029948	ENSBTAG00000029948	12:66227132-66227219	0	0	0	OK
ENSBTAG00000029949	ENSBTAG00000029949	13:64900479-64900582	0	0	0	OK
ENSBTAG00000029950	ENSBTAG00000029950	X:23387290-23387380	0	0	0	OK
ENSBTAG00000029951	ENSBTAG00000029951	12:66226553-66226637	0	0	0	OK
ENSBTAG00000029953	ENSBTAG00000029953	19:20797021-20797105	0	0	0	OK
ENSBTAG00000029954	ENSBTAG00000029954	8:83010369-83010450	0	0	0	OK
ENSBTAG00000029955	ENSBTAG00000029955	19:18824220-18824301	0	0	0	OK
ENSBTAG00000029956	ENSBTAG00000029956	8:23661685-23661769	0	0	0	OK
ENSBTAG00000029957	ENSBTAG00000029957	22:51543469-51543560	0	0	0	OK
ENSBTAG00000029959	ENSBTAG00000029959	5:103859435-103859500	0	0	0	OK
ENSBTAG00000029960	ENSBTAG00000029960	27:21315202-21315275	0	0	0	OK
ENSBTAG00000029961	ENSBTAG00000029961	21:67603237-67603338	0	0	0	OK
ENSBTAG00000029962	ENSBTAG00000029962	13:54827543-54827630	0	0	0	OK

ENSBTAG00000029965	ENSBTAG00000029965	4:101436257-101436384	0	0	0	OK
ENSBTAG00000029967	ENSBTAG00000029967	20:189856-189928	0	0	0	OK
ENSBTAG00000029968	ENSBTAG00000029968	7:20584156-20584238	0	0	0	OK
ENSBTAG00000029969	ENSBTAG00000029969	7:12915237-12915350	0	0	0	OK
ENSBTAG00000029970	ENSBTAG00000029970	4:69327759-69327844	0	0	0	OK
ENSBTAG00000029972	ENSBTAG00000029972	14:8084720-8084808	0	0	0	OK
ENSBTAG00000029973	ENSBTAG00000029973	X:17918367-17918442	0	0	0	OK
ENSBTAG00000029974	ENSBTAG00000029974	11:98867656-98867756	0	0	0	OK
ENSBTAG00000029975	ENSBTAG00000029975	5:113410749-113410818	0	0	0	OK
ENSBTAG00000029976	ENSBTAG00000029976	25:14176047-14176110	0	0	0	OK
ENSBTAG00000029977	ENSBTAG00000029977	4:94411192-94411295	0	0	0	OK
ENSBTAG00000029978	ENSBTAG00000029978	X:17917701-17917775	0	0	0	OK
ENSBTAG00000029979	ENSBTAG00000029979	11:104131408-104131481	0	0	0	OK
ENSBTAG00000029980	ENSBTAG00000029980	4:95402693-95402774	0	0	0	OK
ENSBTAG00000029981	ENSBTAG00000029981	8:86884864-86884958	0	0	0	OK
ENSBTAG00000029982	ENSBTAG00000029982	19:9527304-9527391	0	0	0	OK
ENSBTAG00000029983	ENSBTAG00000029983	6:14163274-14163342	0	0	0	OK
ENSBTAG00000029987	ENSBTAG00000029987	14:8080291-8080361	0	0	0	OK
ENSBTAG00000029988	ENSBTAG00000029988	5:25109056-25109150	0	0	0	OK
ENSBTAG00000029989	ENSBTAG00000029989	8:83009606-83009680	0	0	0	OK
ENSBTAG00000029990	ENSBTAG00000029990	2:62007751-62007833	0	0	0	OK
ENSBTAG00000029991	ENSBTAG00000029991	15:22135425-22135502	0	0	0	OK
ENSBTAG00000029993	ENSBTAG00000029993	16:79685759-79685869	0	0	0	OK
ENSBTAG00000029994	ENSBTAG00000029994	5:51209079-51209164	0	0	0	OK
ENSBTAG00000029995	ENSBTAG00000029995	21:67589863-67589943	0	0	0	OK
ENSBTAG00000029996	ENSBTAG00000029996	7:12981790-12981868	0	0	0	OK
ENSBTAG00000029998	ENSBTAG00000029998	16:52521323-52521409	0	0	0	OK
ENSBTAG00000029999	ENSBTAG00000029999	18:34957609-34957701	0	0	4.50124	OK
ENSBTAG00000030002	ENSBTAG00000030002	21:67427769-67427883	0	0	3.63258	OK
ENSBTAG00000030003	ENSBTAG00000030003	21:67429739-67429853	0	0	3.63258	OK

ENSBTAG00000030004	ENSBTAG00000030004	21:67428645-67428769	0	0	3.33963	OK
ENSBTAG00000030010	ENSBTAG00000030010	3:15480724-15480820	0	0	0	OK
ENSBTAG00000030013	ENSBTAG00000030013	X:92901849-92901929	0	0	0	OK
ENSBTAG00000030014	ENSBTAG00000030014	10:55017683-55017778	0	0	0	OK
ENSBTAG00000030016	ENSBTAG00000030016	21:67604688-67604767	0	0	0	OK
ENSBTAG00000030017	ENSBTAG00000030017	21:67578838-67578919	0	0	0	OK
ENSBTAG00000030019	ENSBTAG00000030019	X:81951372-81951478	0	0	0	OK
ENSBTAG00000030020	ENSBTAG00000030020	14:66995148-66995243	0	0	0	OK
ENSBTAG00000030024	ENSBTAG00000030024	X:18180132-18180229	0	0	0	OK
ENSBTAG00000030026	ENSBTAG00000030026	21:67583557-67583641	0	0	0	OK
ENSBTAG00000030027	ENSBTAG00000030027	20:23967268-23967366	0	0	0	OK
ENSBTAG00000030029	ENSBTAG00000030029	1:99671695-99671790	0	0	0	OK
ENSBTAG00000030030	ENSBTAG00000030030	4:92126803-92126899	0	0	0	OK
ENSBTAG00000030035	ENSBTAG00000030035	21:67586357-67586448	0	0	0	OK
ENSBTAG00000030038	ENSBTAG00000030038	8:52243196-52243317	0	0	0	OK
ENSBTAG00000030039	ENSBTAG00000030039	X:110273859-110273942	0	0	0	OK
ENSBTAG00000030040	ENSBTAG00000030040	X:2445300-2445380	0	0	0	OK
ENSBTAG00000030046	ENSBTAG00000030046	11:38511116-38511202	0	0	0	OK
ENSBTAG00000030047	ENSBTAG00000030047	19:38293011-38293077	0	0	0	OK
ENSBTAG00000030049	ENSBTAG00000030049	10:47321060-47321145	0	0	0	OK
ENSBTAG00000030051	ENSBTAG00000030051	20:23962985-23963091	0	0	0	OK
ENSBTAG00000030052	ENSBTAG00000030052	21:33857311-33857386	0	0	0	OK
ENSBTAG00000030053	ENSBTAG00000030053	16:24306023-24306120	0	0	0	OK
ENSBTAG00000030055	ENSBTAG00000030055	26:25988239-25988338	0	0	0	OK
ENSBTAG00000030056	ENSBTAG00000030056	16:24305736-24305815	0	0	0	OK
ENSBTAG00000030059	ENSBTAG00000030059	X:17917856-17917924	0	0	0	OK
ENSBTAG00000030060	ENSBTAG00000030060	19:36871897-36871986	0	0	0	OK
ENSBTAG00000030062	ENSBTAG00000030062	16:52521991-52522050	0	0	0	OK
ENSBTAG00000030064	ENSBTAG00000030064	20:6239917-6239993	0	0	0	OK
ENSBTAG00000030066	ENSBTAG00000030066	19:10339844-10339958	0	0	0	OK

ENSBTAG00000030067	ENSBTAG00000030067	14:69317026-69317102	0	0	0	OK
ENSBTAG00000030068	ENSBTAG00000030068	18:56407842-56407942	0	0	0	OK
ENSBTAG00000030071	ENSBTAG00000030071	7:12981641-12981713	0	0	0	OK
ENSBTAG00000030073	ENSBTAG00000030073	21:66706847-66706941	0	0	0	OK
ENSBTAG00000030082	ENSBTAG00000030082	22:35681614-35681690	0	0	0	OK
ENSBTAG00000030083	ENSBTAG00000030083	15:74519281-74519380	0	0	0	OK
ENSBTAG00000030084	ENSBTAG00000030084	X:18185079-18185185	0	0	0	OK
ENSBTAG00000030085	ENSBTAG00000030085	28:30855850-30855935	0	0	0	OK
ENSBTAG00000030086	ENSBTAG00000030086	24:35602254-35602346	0	0	0	OK
ENSBTAG00000030089	ENSBTAG00000030089	3:120467947-120468035	0	0	0	OK
ENSBTAG00000030090	ENSBTAG00000030090	13:70737769-70737854	0	0	0	OK
ENSBTAG00000030095	ENSBTAG00000030095	4:9585458-9585553	0	0	0	OK
ENSBTAG00000030097	ENSBTAG00000030097	29:37318999-37319066	0	0	0	OK
ENSBTAG00000030099	ENSBTAG00000030099	16:50741092-50741187	0	0	0	OK
ENSBTAG00000030100	ENSBTAG00000030100	25:13790124-13790206	0	0	0	OK
ENSBTAG00000030102	ENSBTAG00000030102	3:49797089-49797206	0	0	0	OK
ENSBTAG00000030104	ENSBTAG00000030104	X:35020217-35020322	0	0	0	OK
ENSBTAG00000030105	ENSBTAG00000030105	1:149720301-149720392	0	0	0	OK
ENSBTAG00000030106	ENSBTAG00000030106	X:35021154-35021234	0	0	0	OK
ENSBTAG00000030107	ENSBTAG00000030107	X:3841099-3841212	0	0	0	OK
ENSBTAG00000030108	ENSBTAG00000030108	18:53945875-53945994	0	0	0	OK
ENSBTAG00000030109	ENSBTAG00000030109	19:31333511-31333611	0	0	0	OK
ENSBTAG00000030110	ENSBTAG00000030110	12:10980686-10980782	0	0	0	OK
ENSBTAG00000030111	ENSBTAG00000030111	5:48164006-48164126	0	0	0	OK
ENSBTAG00000030113	ENSBTAG00000030113	19:27441342-27441429	0	0	0	OK
ENSBTAG00000030114	ENSBTAG00000030114	7:62809303-62809404	0	0	0	OK
ENSBTAG00000030117	ENSBTAG00000030117	11:98997149-98997245	0	0	0	OK
ENSBTAG00000030118	ENSBTAG00000030118	4:94406699-94406810	0	0	0	OK
ENSBTAG00000030119	ENSBTAG00000030119	3:32399960-32400092	0	0	0	OK
ENSBTAG00000030121	ENSBTAG00000030121	20:23967392-23967480	0	0	0	OK

ENSBTAG00000030122	ENSBTAG00000030122	15:82197710-82197799	0	0	0	OK
ENSBTAG00000030123	ENSBTAG00000030123	29:43731459-43731542	0	0	0	OK
ENSBTAG00000030124	ENSBTAG00000030124	1:5378509-5378601	0	0	0	OK
ENSBTAG00000030164	ENSBTAG00000030164	19:57835001-57839034	0	0	0	OK
ENSBTAG00000030169	ENSBTAG00000030169	19:56923423-56931160	0	0	0	OK
ENSBTAG00000030182	ENSBTAG00000030182	5:119832292-119837101	0	0	0	OK
ENSBTAG00000030185	ENSBTAG00000030185	5:119672822-119673035	0	0	0	OK
ENSBTAG00000030186	ENSBTAG00000030186	19:53108150-53108444	0	0	0	OK
ENSBTAG00000030193	ENSBTAG00000030193	3:120439885-120441226	0	0	0	OK
ENSBTAG00000030204	ENSBTAG00000030204	7:105359722-105360594	0	0	0	OK
ENSBTAG00000030221	ENSBTAG00000030221	3:119842349-119843529	0	0	0	OK
ENSBTAG00000030239	ENSBTAG00000030239	3:113532379-113533309	0	0	0	OK
ENSBTAG00000030246	ENSBTAG00000030246	11:105698113-105702610	0	0	0.193421	OK
ENSBTAG00000030274	ENSBTAG00000030274	7:81396100-81396804	0	0	0	OK
ENSBTAG00000030281	ENSBTAG00000030281	11:104186562-104187141	0	0	0	OK
ENSBTAG00000030282	ENSBTAG00000030282	7:74091291-74096990	0	0	0	OK
ENSBTAG00000030296	ENSBTAG00000030296	11:106244290-106246501	0	0	0	OK
ENSBTAG00000030306	ENSBTAG00000030306	19:43889805-43889996	0	0	0	OK
ENSBTAG00000030308	ENSBTAG00000030308	19:43871987-43872177	0	0	0	OK
ENSBTAG00000030314	ENSBTAG00000030314	19:43855646-43855835	0	0	0	OK
ENSBTAG00000030319	ENSBTAG00000030319	11:103181660-103192049	0	0	0	OK
ENSBTAG00000030337	ENSBTAG00000030337	3:107700014-107705848	0	0	0	OK
ENSBTAG00000030338	ENSBTAG00000030338	3:107697177-107698719	0	0	0	OK
ENSBTAG00000030340	ENSBTAG00000030340	2:134294881-134340689	0	0	0	OK
ENSBTAG00000030344	ENSBTAG00000030344	18:63812329-63812668	0	0	0	OK
ENSBTAG00000030404	ENSBTAG00000030404	5:101442522-101442823	0	0	0	OK
ENSBTAG00000030413	ENSBTAG00000030413	2:130466428-130478320	0	0	0	OK
ENSBTAG00000030426	ENSBTAG00000030426	5:100183939-100193142	0	0	0	OK
ENSBTAG00000030431	ENSBTAG00000030431	5:98028809-98029368	0	0	0	OK
ENSBTAG00000030439	ENSBTAG00000030439	10:17200007-17200480	0	0	0	OK

ENSBTAG00000030454	ENSBTAG00000030454	18:59385749-59387124	0	0	0	OK
ENSBTAG00000030456	ENSBTAG00000030456	10:101186185-101224038	0	0	0	OK
ENSBTAG00000030463	ENSBTAG00000030463	5:99230572-99231472	0	0	0	OK
ENSBTAG00000030471	ENSBTAG00000030471	5:99065709-99066627	0	0	0	OK
ENSBTAG00000030472	ENSBTAG00000030472	5:99035751-99036690	0	0	0	OK
ENSBTAG00000030484	ENSBTAG00000030484	18:57461616-57465567	0	0	0	OK
ENSBTAG00000030494	ENSBTAG00000030494	22:1944675-1946366	0	0	0	OK
ENSBTAG00000030521	ENSBTAG00000030521	11:99645085-99646294	0	0	0	OK
ENSBTAG00000030532	ENSBTAG00000030532	3:100179027-100183069	0	0	0	OK
ENSBTAG00000030533	ENSBTAG00000030533	22:16360722-16361139	0	0	0	OK
ENSBTAG00000030544	ENSBTAG00000030544	18:56261436-56262086	0	0	0	OK
ENSBTAG00000030553	ENSBTAG00000030553	3:98704902-98724886	0	0	0	OK
ENSBTAG00000030563	ENSBTAG00000030563	18:56456529-56462123	0	0	0	OK
ENSBTAG00000030575	ENSBTAG00000030575	5:84233718-84236923	0	0	0	OK
ENSBTAG00000030589	ENSBTAG00000030589	7:53455879-53461671	0	0	0	OK
ENSBTAG00000030592	ENSBTAG00000030592	1:154150027-154150249	0	0	0	OK
ENSBTAG00000030593	ENSBTAG00000030593	1:155176263-155184715	0	0	0	OK
ENSBTAG00000030616	ENSBTAG00000030616	7:46188838-46202621	0	0	0	OK
ENSBTAG00000030624	ENSBTAG00000030624	13:74202931-74205250	0	0	0	OK
ENSBTAG00000030632	ENSBTAG00000030632	5:76497228-76503464	0	0	0	OK
ENSBTAG00000030663	ENSBTAG00000030663	3:85983260-85983424	0	0	0	OK
ENSBTAG00000030671	ENSBTAG00000030671	3:86650797-86718972	0	0	0	OK
ENSBTAG00000030672	ENSBTAG00000030672	3:105846148-105847499	0	0	0	OK
ENSBTAG00000030678	ENSBTAG00000030678	11:93927185-93927978	0	0	0	OK
ENSBTAG00000030718	ENSBTAG00000030718	2:119614003-119634945	0	0	0	OK
ENSBTAG00000030735	ENSBTAG00000030735	7:42303402-42303827	0	0	0	OK
ENSBTAG00000030764	ENSBTAG00000030764	1:147136867-147136983	0	0	0	OK
ENSBTAG00000030798	ENSBTAG00000030798	13:75179363-75189939	0	0	0	OK
ENSBTAG00000030800	ENSBTAG00000030800	13:75192738-75193896	0	0	0	OK
ENSBTAG00000030802	ENSBTAG00000030802	13:75164670-75166835	0	0	0	OK

ENSBTAG00000030814	ENSBTAG00000030814	1:144176744-144180011	0	0	0	OK
ENSBTAG00000030820	ENSBTAG00000030820	19:35473701-35486296	0	0	0	OK
ENSBTAG00000030836	ENSBTAG00000030836	1:143737581-143755849	0	0	0	OK
ENSBTAG00000030841	ENSBTAG00000030841	10:21008953-21009475	0	0	0	OK
ENSBTAG00000030852	ENSBTAG00000030852	3:79604695-79823433	0	0	0	OK
ENSBTAG00000030855	ENSBTAG00000030855	22:60960189-61060022	0	0	0	OK
ENSBTAG00000030857	ENSBTAG00000030857	4:120138831-120138950	0	0	0	OK
ENSBTAG00000030864	ENSBTAG00000030864	11:74997149-75023692	0	0	0	OK
ENSBTAG00000030876	ENSBTAG00000030876	4:119160834-119164828	0	0	0	OK
ENSBTAG00000030898	ENSBTAG00000030898	15:84666773-84691574	0	0	0	OK
ENSBTAG00000030909	ENSBTAG00000030909	15:79346443-79347350	0	0	0	OK
ENSBTAG00000030915	ENSBTAG00000030915	22:59715781-59732104	0	0	0	OK
ENSBTAG00000030922	ENSBTAG00000030922	18:52265370-52271123	0	0	0	OK
ENSBTAG00000030927	ENSBTAG00000030927	17:74195601-74196988	0	0	0	OK
ENSBTAG00000030932	ENSBTAG00000030932	3:66307327-66318248	0	0	0	OK
ENSBTAG00000030953	ENSBTAG00000030953	8:93237901-93239274	0	0	0	OK
ENSBTAG00000030999	ENSBTAG00000030999	29:50887354-50951170	0	0	0	OK
ENSBTAG00000031004	ENSBTAG00000031004	18:51240177-51242430	0	0	0	OK
ENSBTAG00000031018	ENSBTAG00000031018	17:74419286-74424549	0	0	0	OK
ENSBTAG00000031029	ENSBTAG00000031029	15:79216405-79217317	0	0	0	OK
ENSBTAG00000031032	ENSBTAG00000031032	15:79315292-79316222	0	0	0	OK
ENSBTAG00000031052	ENSBTAG00000031052	2:107620704-107621826	0	0	0	OK
ENSBTAG00000031059	ENSBTAG00000031059	4:113562347-113562992	0	0	0	OK
ENSBTAG00000031061	ENSBTAG00000031061	29:50689637-50694424	0	0	0	OK
ENSBTAG00000031069	ENSBTAG00000031069	7:32140925-32313012	0	0	0	OK
ENSBTAG00000031076	ENSBTAG00000031076	5:56683155-56683671	0	0	0	OK
ENSBTAG00000031082	ENSBTAG00000031082	3:66755613-66755946	0	0	0	OK
ENSBTAG00000031096	ENSBTAG00000031096	5:59633533-59634472	0	0	0	OK
ENSBTAG00000031097	ENSBTAG00000031097	5:59618500-59619439	0	0	0	OK
ENSBTAG00000031100	ENSBTAG00000031100	5:59494170-59495131	0	0	0	OK

ENSBTAG00000031106	ENSBTAG00000031106	4:113292526-113313063	0	0	0	OK
ENSBTAG00000031107	ENSBTAG00000031107	19:32341961-32426347	0	0	0	OK
ENSBTAG00000031111	ENSBTAG00000031111	5:58796712-58797757	0	0	0	OK
ENSBTAG00000031119	ENSBTAG00000031119	15:79208320-79209247	0	0	0	OK
ENSBTAG00000031134	ENSBTAG00000031134	17:72629358-72630106	0	0	0	OK
ENSBTAG00000031162	ENSBTAG00000031162	4:107700101-107701018	0	0	0	OK
ENSBTAG00000031185	ENSBTAG00000031185	11:72405109-72409422	0	0	0	OK
ENSBTAG00000031186	ENSBTAG00000031186	3:54759287-54769809	0	0	0	OK
ENSBTAG00000031194	ENSBTAG00000031194	29:49329503-49330559	0	0	0	OK
ENSBTAG00000031197	ENSBTAG00000031197	6:106868231-106872198	0	0	0	OK
ENSBTAG00000031205	ENSBTAG00000031205	7:23580041-23580833	0	0	0	OK
ENSBTAG00000031210	ENSBTAG00000031210	15:76128117-76135346	0	0	0	OK
ENSBTAG00000031212	ENSBTAG00000031212	4:107344453-107347462	0	0	0	OK
ENSBTAG00000031232	ENSBTAG00000031232	4:106495473-106495981	0	0	0	OK
ENSBTAG00000031235	ENSBTAG00000031235	7:23184381-23186782	0	0	0	OK
ENSBTAG00000031242	ENSBTAG00000031242	21:71052183-71052852	0	0	0	OK
ENSBTAG00000031254	ENSBTAG00000031254	13:61612682-61615456	0	0	0	OK
ENSBTAG00000031256	ENSBTAG00000031256	X:133528331-133528652	0	0	0	OK
ENSBTAG00000031266	ENSBTAG00000031266	18:49142855-49142964	0	0	0	OK
ENSBTAG00000031271	ENSBTAG00000031271	29:49026019-49026889	0	0	0	OK
ENSBTAG00000031277	ENSBTAG00000031277	1:131242443-131243577	0	0	0	OK
ENSBTAG00000031295	ENSBTAG00000031295	8:103452038-103512716	0	0	0	OK
ENSBTAG00000031306	ENSBTAG00000031306	1:123009128-123060090	0	0	0	OK
ENSBTAG00000031347	ENSBTAG00000031347	11:70914103-70953329	0	0	0	OK
ENSBTAG00000031364	ENSBTAG00000031364	6:108717033-108719413	0	0	0	OK
ENSBTAG00000031373	ENSBTAG00000031373	13:63196225-63207043	0	0	0	OK
ENSBTAG00000031375	ENSBTAG00000031375	13:63136696-63143555	0	0	0	OK
ENSBTAG00000031376	ENSBTAG00000031376	13:63086221-63095440	0	0	0	OK
ENSBTAG00000031388	ENSBTAG00000031388	10:54903474-54970680	0	0	0	OK
ENSBTAG00000031395	ENSBTAG00000031395	6:109868826-109870154	0	0	0	OK

ENSBTAG00000031420	ENSBTAG00000031420	18:46193809-46194096	0	0	0	OK
ENSBTAG00000031441	ENSBTAG00000031441	18:46067592-46078935	0	0	0	OK
ENSBTAG00000031454	ENSBTAG00000031454	22:53236357-53240657	0	0	0	OK
ENSBTAG00000031462	ENSBTAG00000031462	13:59979126-59980694	0	0	0	OK
ENSBTAG00000031464	ENSBTAG00000031464	4:84662807-84663581	0	0	0	OK
ENSBTAG00000031473	ENSBTAG00000031473	2:89967560-90005390	0	0	0	OK
ENSBTAG00000031483	ENSBTAG00000031483	29:40214777-40215227	0	0	0	OK
ENSBTAG00000031507	ENSBTAG00000031507	16:55896305-55899061	0	0	0	OK
ENSBTAG00000031517	ENSBTAG00000031517	8:83243781-83253558	0	0	0	OK
ENSBTAG00000031523	ENSBTAG00000031523	X:102588096-102590541	0	0	0	OK
ENSBTAG00000031553	ENSBTAG00000031553	6:108855976-108863733	0	0	0	OK
ENSBTAG00000031558	ENSBTAG00000031558	21:38276191-38276344	0	0	0	OK
ENSBTAG00000031573	ENSBTAG00000031573	7:21290812-21294189	0	0	0	OK
ENSBTAG00000031582	ENSBTAG00000031582	25:28034287-28034644	0	0	0	OK
ENSBTAG00000031583	ENSBTAG00000031583	25:28027756-28028658	0	0	0	OK
ENSBTAG00000031594	ENSBTAG00000031594	22:52202472-52203563	0	0	0	OK
ENSBTAG00000031599	ENSBTAG00000031599	22:52167380-52169563	0	0	0	OK
ENSBTAG00000031600	ENSBTAG00000031600	3:106880038-106880542	0	0	0	OK
ENSBTAG00000031612	ENSBTAG00000031612	22:52109193-52118716	0	0	0	OK
ENSBTAG00000031625	ENSBTAG00000031625	13:55519322-55521407	0	0	0	OK
ENSBTAG00000031655	ENSBTAG00000031655	3:34438480-34438841	0	0	0	OK
ENSBTAG00000031656	ENSBTAG00000031656	3:34418711-34426687	0	0	0	OK
ENSBTAG00000031677	ENSBTAG00000031677	18:47608985-47609106	0	0	0	OK
ENSBTAG00000031704	ENSBTAG00000031704	1:100345022-100375298	0	0	0	OK
ENSBTAG00000031711	ENSBTAG00000031711	2:21060666-21061224	0	0	0	OK
ENSBTAG00000031716	ENSBTAG00000031716	13:55053689-55056880	0	0	0	OK
ENSBTAG00000031717	ENSBTAG00000031717	23:31644097-31644508	0	0	0	OK
ENSBTAG00000031719	ENSBTAG00000031719	23:31669662-31669974	0	0	0	OK
ENSBTAG00000031721	ENSBTAG00000031721	23:31670730-31671141	0	0	0	OK
ENSBTAG00000031724	ENSBTAG00000031724	9:97560723-97561701	0	0	0	OK

ENSBTAG00000031727	ENSBTAG00000031727	21:64608763-64608882	0	0	0	OK
ENSBTAG00000031731	ENSBTAG00000031731	11:49760291-49761138	0	0	0	OK
ENSBTAG00000031738	ENSBTAG00000031738	23:31652710-31653146	0	0	0	OK
ENSBTAG00000031750	ENSBTAG00000031750	6:99655643-99690417	0	0	0	OK
ENSBTAG00000031756	ENSBTAG00000031756	23:31456885-31457197	0	0	0	OK
ENSBTAG00000031758	ENSBTAG00000031758	23:31461682-31462143	0	0	0	OK
ENSBTAG00000031759	ENSBTAG00000031759	23:31463931-31464342	0	0	0	OK
ENSBTAG00000031760	ENSBTAG00000031760	23:31472338-31472719	0	0	0	OK
ENSBTAG00000031761	ENSBTAG00000031761	23:31474029-31474440	0	0	0	OK
ENSBTAG00000031762	ENSBTAG00000031762	23:31479374-31479686	0	0	0	OK
ENSBTAG00000031763	ENSBTAG00000031763	23:31485394-31485706	0	0	0	OK
ENSBTAG00000031764	ENSBTAG00000031764	13:54707288-54707613	0	0	0	OK
ENSBTAG00000031766	ENSBTAG00000031766	23:31497479-31497890	0	0	0	OK
ENSBTAG00000031767	ENSBTAG00000031767	23:31517215-31517625	0	0	0	OK
ENSBTAG00000031770	ENSBTAG00000031770	23:31525878-31526259	0	0	0	OK
ENSBTAG00000031773	ENSBTAG00000031773	23:31654043-31654454	0	0	0	OK
ENSBTAG00000031774	ENSBTAG00000031774	23:31530242-31531841	0	0	0	OK
ENSBTAG00000031776	ENSBTAG00000031776	23:31539668-31540049	0	0	0	OK
ENSBTAG00000031778	ENSBTAG00000031778	23:31554349-31555332	0	0	0	OK
ENSBTAG00000031785	ENSBTAG00000031785	23:31589470-31602081	0	0	0	OK
ENSBTAG00000031792	ENSBTAG00000031792	23:28714469-28717896	0	0	0	OK
ENSBTAG00000031793	ENSBTAG00000031793	8:77063060-77073920	0	0	0	OK
ENSBTAG00000031794	ENSBTAG00000031794	7:19296436-19296982	0	0	0	OK
ENSBTAG00000031800	ENSBTAG00000031800	13:54608179-54609543	0	0	0	OK
ENSBTAG00000031825	ENSBTAG00000031825	23:28813848-28814950	0	0	0	OK
ENSBTAG00000031828	ENSBTAG00000031828	11:49135530-49140113	0	0	0	OK
ENSBTAG00000031832	ENSBTAG00000031832	23:28992087-28993026	0	0	0	OK
ENSBTAG00000031851	ENSBTAG00000031851	17:66557142-66573393	0	0	0	OK
ENSBTAG00000031861	ENSBTAG00000031861	4:114803156-114855282	0	0	0	OK
ENSBTAG00000031863	ENSBTAG00000031863	4:93460873-93462935	0	0	0	OK

ENSBTAG00000031869	ENSBTAG00000031869	23:30161122-30171348	0	0	0	OK
ENSBTAG00000031873	ENSBTAG00000031873	23:30305068-30312399	0	0	0	OK
ENSBTAG00000031874	ENSBTAG00000031874	23:30332528-30343084	0	0	0	OK
ENSBTAG00000031881	ENSBTAG00000031881	23:30464776-30465957	0	0	0	OK
ENSBTAG00000031888	ENSBTAG00000031888	23:30766890-30767343	0	0	0	OK
ENSBTAG00000031903	ENSBTAG00000031903	15:51733362-51734307	0	0	0	OK
ENSBTAG00000031906	ENSBTAG00000031906	13:54428989-54430673	0	0	0	OK
ENSBTAG00000031924	ENSBTAG00000031924	15:51392457-51393411	0	0	0	OK
ENSBTAG00000031937	ENSBTAG00000031937	15:50907141-50908279	0	0	0	OK
ENSBTAG00000031943	ENSBTAG00000031943	15:50884387-50885332	0	0	0	OK
ENSBTAG00000031958	ENSBTAG00000031958	4:89154605-89161368	0	0	0	OK
ENSBTAG00000031965	ENSBTAG00000031965	4:83280520-83280990	0	0	0	OK
ENSBTAG00000031981	ENSBTAG00000031981	10:37989935-37995280	0	0	0	OK
ENSBTAG00000032022	ENSBTAG00000032022	6:94108966-94109493	0	0	0	OK
ENSBTAG00000032034	ENSBTAG00000032034	6:95908623-95908893	0	0	0	OK
ENSBTAG00000032047	ENSBTAG00000032047	17:65508335-65514043	0	0	0	OK
ENSBTAG00000032049	ENSBTAG00000032049	4:88193846-88194752	0	0	0	OK
ENSBTAG00000032056	ENSBTAG00000032056	15:46440582-46468602	0	0	0	OK
ENSBTAG00000032057	ENSBTAG00000032057	3:28623390-28623702	0	0	0	OK
ENSBTAG00000032074	ENSBTAG00000032074	6:92416962-92417126	0	0	0	OK
ENSBTAG00000032093	ENSBTAG00000032093	8:76329356-76348121	0	0	0	OK
ENSBTAG00000032114	ENSBTAG00000032114	1:88482653-88483052	0	0	0	OK
ENSBTAG00000032166	ENSBTAG00000032166	4:75013860-75132831	0	0	0	OK
ENSBTAG00000032193	ENSBTAG00000032193	3:23593212-23614190	0	0	0	OK
ENSBTAG00000032198	ENSBTAG00000032198	25:219742-220147	0	0	0	OK
ENSBTAG00000032206	ENSBTAG00000032206	8:63560432-63578800	0	0	0	OK
ENSBTAG00000032255	ENSBTAG00000032255	15:48060529-48061492	0	0	0	OK
ENSBTAG00000032259	ENSBTAG00000032259	15:48045977-48046289	0	0	0	OK
ENSBTAG00000032262	ENSBTAG00000032262	15:48016202-48017237	0	0	0	OK
ENSBTAG00000032264	ENSBTAG00000032264	2:55560966-55561101	0	0	0	OK

ENSBTAG00000032274	ENSBTAG00000032274	15:47772018-47772948	0	0	0	OK
ENSBTAG00000032279	ENSBTAG00000032279	22:49579354-49580862	0	0	0	OK
ENSBTAG00000032281	ENSBTAG00000032281	14:75849443-75850834	0	0	0	OK
ENSBTAG00000032299	ENSBTAG00000032299	26:37073737-37131447	0	0	0	OK
ENSBTAG00000032307	ENSBTAG00000032307	X:125258254-125259234	0	0	0	OK
ENSBTAG00000032308	ENSBTAG00000032308	3:21433393-21433557	0	0	0	OK
ENSBTAG00000032339	ENSBTAG00000032339	15:44246870-44247423	0	0	0	OK
ENSBTAG00000032347	ENSBTAG00000032347	22:48146335-48146712	0	0	0	OK
ENSBTAG00000032350	ENSBTAG00000032350	6:86034661-86055504	0	0	0	OK
ENSBTAG00000032369	ENSBTAG00000032369	2:44930494-44950481	0	0	0	OK
ENSBTAG00000032373	ENSBTAG00000032373	25:20247254-20288197	0	0	0.242597	OK
ENSBTAG00000032374	ENSBTAG00000032374	22:47582814-47590574	0	0	0	OK
ENSBTAG00000032382	ENSBTAG00000032382	9:15435069-15435755	0	0	0	OK
ENSBTAG00000032402	ENSBTAG00000032402	3:22861820-22861984	0	0	0	OK
ENSBTAG00000032405	ENSBTAG00000032405	2:39571128-39572189	0	0	0	OK
ENSBTAG00000032406	ENSBTAG00000032406	3:22829734-22829898	0	0	0	OK
ENSBTAG00000032407	ENSBTAG00000032407	3:22756450-22756614	0	0	0	OK
ENSBTAG00000032446	ENSBTAG00000032446	3:18288307-18288811	0	0	0	OK
ENSBTAG00000032450	ENSBTAG00000032450	X:57583909-57584338	0	0	0	OK
ENSBTAG00000032451	ENSBTAG00000032451	3:20825189-20825680	0	0	0	OK
ENSBTAG00000032452	ENSBTAG00000032452	3:20823725-20824139	0	0	0	OK
ENSBTAG00000032455	ENSBTAG00000032455	3:20793581-20793962	0	0	0	OK
ENSBTAG00000032458	ENSBTAG00000032458	3:20791343-20791757	0	0	0	OK
ENSBTAG00000032485	ENSBTAG00000032485	X:68428001-68491176	0	0	0	OK
ENSBTAG00000032531	ENSBTAG00000032531	22:48611897-48613043	0	0	0	OK
ENSBTAG00000032557	ENSBTAG00000032557	29:40927661-40931388	0	0	0	OK
ENSBTAG00000032560	ENSBTAG00000032560	6:73762655-73770020	0	0	0	OK
ENSBTAG00000032567	ENSBTAG00000032567	5:30654294-30657317	0	0	0	OK
ENSBTAG00000032591	ENSBTAG00000032591	14:63311965-63328221	0	0	0	OK
ENSBTAG00000032598	ENSBTAG00000032598	4:64131286-64143143	0	0	0	OK

ENSBTAG00000032613	ENSBTAG00000032613	10:30077157-30135194	0	0	0	OK
ENSBTAG00000032615	ENSBTAG00000032615	18:22622802-22622916	0	0	0	OK
ENSBTAG00000032624	ENSBTAG00000032624	23:25080358-25080477	0	0	0	OK
ENSBTAG00000032642	ENSBTAG00000032642	23:24935522-24957718	0	0	0	OK
ENSBTAG00000032643	ENSBTAG00000032643	25:12904754-12905757	0	0	0	OK
ENSBTAG00000032655	ENSBTAG00000032655	28:46035469-46035863	0	0	0	OK
ENSBTAG00000032660	ENSBTAG00000032660	6:71186362-71245780	0	0	0	OK
ENSBTAG00000032669	ENSBTAG00000032669	8:60546081-60547020	0	0	0	OK
ENSBTAG00000032671	ENSBTAG00000032671	8:60668702-60669659	0	0	0	OK
ENSBTAG00000032684	ENSBTAG00000032684	1:71489284-71516789	0	0	0	OK
ENSBTAG00000032686	ENSBTAG00000032686	10:21462582-21464193	0	0	0	OK
ENSBTAG00000032694	ENSBTAG00000032694	13:42500946-42508871	0	0	0	OK
ENSBTAG00000032702	ENSBTAG00000032702	18:24012775-24013408	0	0	0	OK
ENSBTAG00000032727	ENSBTAG00000032727	12:55049152-55049533	0	0	0	OK
ENSBTAG00000032728	ENSBTAG00000032728	6:68966711-68966834	0	0	0	OK
ENSBTAG00000032730	ENSBTAG00000032730	29:39933365-39937116	0	0	0	OK
ENSBTAG00000032733	ENSBTAG00000032733	3:19090894-19098231	0	0	0	OK
ENSBTAG00000032761	ENSBTAG00000032761	26:24303868-24309613	0	0	0	OK
ENSBTAG00000032774	ENSBTAG00000032774	28:41912321-41914565	0	0	0	OK
ENSBTAG00000032783	ENSBTAG00000032783	10:27303084-27304025	0	0	0	OK
ENSBTAG00000032789	ENSBTAG00000032789	10:27267831-27268755	0	0	0	OK
ENSBTAG00000032801	ENSBTAG00000032801	10:26916705-26917662	0	0	0	OK
ENSBTAG00000032806	ENSBTAG00000032806	X:58242197-58242746	0	0	0	OK
ENSBTAG00000032827	ENSBTAG00000032827	13:38990409-38990528	0	0	0	OK
ENSBTAG00000032831	ENSBTAG00000032831	14:42647472-42647670	0	0	0	OK
ENSBTAG00000032833	ENSBTAG00000032833	X:137215427-137216823	0	0	0	OK
ENSBTAG00000032869	ENSBTAG00000032869	10:26631718-26632354	0	0	0	OK
ENSBTAG00000032872	ENSBTAG00000032872	22:37731239-37737102	0	0	0	OK
ENSBTAG00000032873	ENSBTAG00000032873	10:26320716-26321148	0	0	0	OK
ENSBTAG00000032875	ENSBTAG00000032875	X:50786253-50787236	0	0	0	OK

ENSBTAG00000032878	ENSBTAG00000032878	12:51361364-51361727	0	0	0	OK
ENSBTAG00000032899	ENSBTAG00000032899	5:29080925-29082000	0	0	0	OK
ENSBTAG00000032954	ENSBTAG00000032954	22:35335189-35335581	0	0	0	OK
ENSBTAG00000032964	ENSBTAG00000032964	24:46409593-46453472	0	0	0	OK
ENSBTAG00000032979	ENSBTAG00000032979	12:41907040-41907707	0	0	0	OK
ENSBTAG00000032983	ENSBTAG00000032983	8:53347352-53348693	0	0	0	OK
ENSBTAG00000032984	ENSBTAG00000032984	12:39941062-39942391	0	0	0	OK
ENSBTAG00000033015	ENSBTAG00000033015	25:1391461-1393086	0	0	0	OK
ENSBTAG00000033036	ENSBTAG00000033036	10:25348377-25348883	0	0	0	OK
ENSBTAG00000033045	ENSBTAG00000033045	10:25139501-25140062	0	0	0	OK
ENSBTAG00000033055	ENSBTAG00000033055	7:11771566-11772284	0	0	0	OK
ENSBTAG00000033061	ENSBTAG00000033061	7:11600989-11601710	0	0	0	OK
ENSBTAG00000033078	ENSBTAG00000033078	18:18539430-18554094	0	0	0	OK
ENSBTAG00000033083	ENSBTAG00000033083	9:56339758-56340420	0	0	0	OK
ENSBTAG00000033109	ENSBTAG00000033109	9:52048372-52048800	0	0	0	OK
ENSBTAG00000033117	ENSBTAG00000033117	23:31530229-31531841	0	0	0	OK
ENSBTAG00000033128	ENSBTAG00000033128	14:32213576-32215193	0	0	0	OK
ENSBTAG00000033136	ENSBTAG00000033136	16:33181538-33188176	0	0	0	OK
ENSBTAG00000033146	ENSBTAG00000033146	7:8104566-8105517	0	0	0	OK
ENSBTAG00000033167	ENSBTAG00000033167	X:38965051-38967060	0	0	0	OK
ENSBTAG00000033180	ENSBTAG00000033180	16:31589395-32333109	0	0	0	OK
ENSBTAG00000033187	ENSBTAG00000033187	20:31128230-31128991	0	0	0	OK
ENSBTAG00000033195	ENSBTAG00000033195	9:27271800-27348055	0	0	0	OK
ENSBTAG00000033222	ENSBTAG00000033222	7:6450961-6476687	0	0	0	OK
ENSBTAG00000033224	ENSBTAG00000033224	20:31696672-31697454	0	0	0	OK
ENSBTAG00000033237	ENSBTAG00000033237	29:36926677-36927214	0	0	0	OK
ENSBTAG00000033252	ENSBTAG00000033252	16:30379089-30379491	0	0	0	OK
ENSBTAG00000033254	ENSBTAG00000033254	2:18424772-18431102	0	0	0	OK
ENSBTAG00000033269	ENSBTAG00000033269	4:43987726-43987890	0	0	0	OK
ENSBTAG00000033270	ENSBTAG00000033270	19:20411438-20412406	0	0	0	OK

ENSBTAG00000033275	ENSBTAG00000033275	22:20841407-20841516	0	0	0	OK
ENSBTAG00000033284	ENSBTAG00000033284	14:25052884-25058779	0	0	0	OK
ENSBTAG00000033290	ENSBTAG00000033290	4:43684550-43688401	0	0	0	OK
ENSBTAG00000033308	ENSBTAG00000033308	27:34978848-34979357	0	0	0	OK
ENSBTAG00000033309	ENSBTAG00000033309	27:35392952-35393071	0	0	0	OK
ENSBTAG00000033312	ENSBTAG00000033312	27:34367777-34483222	0	0	0	OK
ENSBTAG00000033322	ENSBTAG00000033322	16:29552151-29561665	0	0	0	OK
ENSBTAG00000033326	ENSBTAG00000033326	18:14578305-14584239	0	0	0	OK
ENSBTAG00000033330	ENSBTAG00000033330	2:20839235-20841537	0	0	0	OK
ENSBTAG00000033344	ENSBTAG00000033344	10:18889117-18907145	0	0	0	OK
ENSBTAG00000033346	ENSBTAG00000033346	6:37159363-37187478	0	0	0	OK
ENSBTAG00000033351	ENSBTAG00000033351	6:41685690-41707024	0	0	0	OK
ENSBTAG00000033352	ENSBTAG00000033352	8:43916604-43972570	0	0	0	OK
ENSBTAG00000033359	ENSBTAG00000033359	4:39525512-39526944	0	0	0	OK
ENSBTAG00000033365	ENSBTAG00000033365	9:40335193-40461911	0	0	0	OK
ENSBTAG00000033375	ENSBTAG00000033375	29:29564774-29566069	0	0	0	OK
ENSBTAG00000033377	ENSBTAG00000033377	29:29595188-29596363	0	0	0	OK
ENSBTAG00000033386	ENSBTAG00000033386	12:29991970-30015315	0	0	0	OK
ENSBTAG00000033396	ENSBTAG00000033396	8:46235395-46339984	0	0	0	OK
ENSBTAG00000033437	ENSBTAG00000033437	1:42203168-42204113	0	0	0	OK
ENSBTAG00000033446	ENSBTAG00000033446	14:17140120-17171601	0	0	0	OK
ENSBTAG00000033464	ENSBTAG00000033464	29:28523796-28537744	0	0	0	OK
ENSBTAG00000033470	ENSBTAG00000033470	7:61446178-61487954	0	0	0	OK
ENSBTAG00000033481	ENSBTAG00000033481	25:865751-867111	0	0	0	OK
ENSBTAG00000033502	ENSBTAG00000033502	29:27570025-27573346	0	0	0	OK
ENSBTAG00000033510	ENSBTAG00000033510	15:29378621-29389165	0	0	0	OK
ENSBTAG00000033511	ENSBTAG00000033511	29:26864728-26865660	0	0	0	OK
ENSBTAG00000033513	ENSBTAG00000033513	23:34719326-34731151	0	0	0	OK
ENSBTAG00000033515	ENSBTAG00000033515	23:3946456-4067555	0	0	0	OK
ENSBTAG00000033523	ENSBTAG00000033523	2:14443705-14445836	0	0	0	OK

ENSBTAG00000033526	ENSBTAG00000033526	25:676664-677047	0	0	0	OK
ENSBTAG00000033554	ENSBTAG00000033554	9:34623077-34661582	0	0	0	OK
ENSBTAG00000033555	ENSBTAG00000033555	28:2606214-2607156	0	0	0	OK
ENSBTAG00000033558	ENSBTAG00000033558	17:51260766-51262423	0	0	0	OK
ENSBTAG00000033565	ENSBTAG00000033565	20:55967803-55968473	0	0	0	OK
ENSBTAG00000033568	ENSBTAG00000033568	7:55006521-55007458	0	0	0	OK
ENSBTAG00000033604	ENSBTAG00000033604	18:59050674-59050995	0	0	0	OK
ENSBTAG00000033620	ENSBTAG00000033620	17:51872340-51874762	0	0	0	OK
ENSBTAG00000033622	ENSBTAG00000033622	14:26380393-26380557	0	0	0	OK
ENSBTAG00000033627	ENSBTAG00000033627	14:23724625-23725771	0	0	0	OK
ENSBTAG00000033672	ENSBTAG00000033672	25:560781-562400	0	0	0	OK
ENSBTAG00000033685	ENSBTAG00000033685	26:17279382-17293746	0	0	0	OK
ENSBTAG00000033695	ENSBTAG00000033695	8:23287348-23287909	0	0	0	OK
ENSBTAG00000033708	ENSBTAG00000033708	8:23196388-23197073	0	0	0	OK
ENSBTAG00000033739	ENSBTAG00000033739	25:493354-495342	0	0	0	OK
ENSBTAG00000033740	ENSBTAG00000033740	1:116197010-116197376	0	0	0	OK
ENSBTAG00000033748	ENSBTAG00000033748	11:7196919-7221257	0	0	0	OK
ENSBTAG00000033749	ENSBTAG00000033749	X:42130044-42131016	0	0	0	OK
ENSBTAG00000033759	ENSBTAG00000033759	7:61639307-61734503	0	0	0	OK
ENSBTAG00000033772	ENSBTAG00000033772	11:6985151-6985270	0	0	0	OK
ENSBTAG00000033802	ENSBTAG00000033802	3:120199292-120199804	0	0	0	OK
ENSBTAG00000033839	ENSBTAG00000033839	8:29148161-29148280	0	0	0	OK
ENSBTAG00000033841	ENSBTAG00000033841	19:12536190-12538358	0	0	0	OK
ENSBTAG00000033891	ENSBTAG00000033891	10:42701306-42702665	0	0	0	OK
ENSBTAG00000033897	ENSBTAG00000033897	20:14459357-14459991	0	0	0	OK
ENSBTAG00000033967	ENSBTAG00000033967	17:23530452-23531007	0	0	0	OK
ENSBTAG00000033993	ENSBTAG00000033993	23:34693484-34706026	0	0	0	OK
ENSBTAG00000033994	ENSBTAG00000033994	16:71077716-71077907	0	0	0	OK
ENSBTAG00000034024	ENSBTAG00000034024	25:192904-194061	0	0	0	OK
ENSBTAG00000034045	ENSBTAG00000034045	X:30989739-31029258	0	0	0	OK

ENSBTAG00000034089	ENSBTAG00000034089	17:19435948-19438716	0	0	0	OK
ENSBTAG00000034113	ENSBTAG00000034113	7:67469563-67692108	0	0	0	OK
ENSBTAG00000034145	ENSBTAG00000034145	13:17873658-17883719	0	0	0	OK
ENSBTAG00000034170	ENSBTAG00000034170	20:11746000-11746270	0	0	0	OK
ENSBTAG00000034174	ENSBTAG00000034174	X:139064202-139064448	0	0	0	OK
ENSBTAG00000034187	ENSBTAG00000034187	8:24128821-24128928	0	0	0	OK
ENSBTAG00000034220	ENSBTAG00000034220	8:22147413-22151549	0	0	0	OK
ENSBTAG00000034238	ENSBTAG00000034238	25:2536213-2540270	0	0	0	OK
ENSBTAG00000034244	ENSBTAG00000034244	29:39345958-39355136	0	0	0	OK
ENSBTAG00000034261	ENSBTAG00000034261	14:338621-339191	0	0	0	OK
ENSBTAG00000034266	ENSBTAG00000034266	X:129382028-129382406	0	0	0	OK
ENSBTAG00000034284	ENSBTAG00000034284	1:33853692-33854670	0	0	0	OK
ENSBTAG00000034308	ENSBTAG00000034308	X:12700266-12701262	0	0	0	OK
ENSBTAG00000034315	ENSBTAG00000034315	9:24048704-24049412	0	0	0	OK
ENSBTAG00000034320	ENSBTAG00000034320	8:23035514-23036091	0	0	0	OK
ENSBTAG00000034326	ENSBTAG00000034326	8:22920116-22920704	0	0	0	OK
ENSBTAG00000034328	ENSBTAG00000034328	7:388808-388927	0	0	0	OK
ENSBTAG00000034337	ENSBTAG00000034337	27:16404736-16404951	0	0	0	OK
ENSBTAG00000034346	ENSBTAG00000034346	15:22105613-22120867	0	0	0	OK
ENSBTAG00000034376	ENSBTAG00000034376	4:20621605-20622383	0	0	0	OK
ENSBTAG00000034385	ENSBTAG00000034385	5:103987751-103993598	0	0	0	OK
ENSBTAG00000034396	ENSBTAG00000034396	19:13192528-13194040	0	0	0	OK
ENSBTAG00000034398	ENSBTAG00000034398	21:5373899-5374265	0	0	0	OK
ENSBTAG00000034411	ENSBTAG00000034411	6:62877286-62880849	0	0	0	OK
ENSBTAG00000034431	ENSBTAG00000034431	20:71475002-71476030	0	0	0	OK
ENSBTAG00000034433	ENSBTAG00000034433	19:12844737-12845367	0	0	0	OK
ENSBTAG00000034437	ENSBTAG00000034437	X:119164232-119165237	0	0	0	OK
ENSBTAG00000034444	ENSBTAG00000034444	X:40831322-40832788	0	0	0	OK
ENSBTAG00000034463	ENSBTAG00000034463	10:11033433-11033780	0	0	0	OK
ENSBTAG00000034495	ENSBTAG00000034495	12:15329214-15465651	0	0	0	OK

ENSBTAG00000034496	ENSBTAG00000034496	22:22098223-22098436	0	0	0	OK
ENSBTAG00000034503	ENSBTAG00000034503	2:43732219-43732799	0	0	0	OK
ENSBTAG00000034514	ENSBTAG00000034514	6:26691064-26692155	0	0	0	OK
ENSBTAG00000034519	ENSBTAG00000034519	23:8269051-8271772	0	0	0	OK
ENSBTAG00000034522	ENSBTAG00000034522	17:11923470-12064069	0	0	0	OK
ENSBTAG00000034551	ENSBTAG00000034551	24:51031798-51031962	0	0	0	OK
ENSBTAG00000034555	ENSBTAG00000034555	24:51182583-51183438	0	0	0	OK
ENSBTAG00000034580	ENSBTAG00000034580	GJ058433.1:35910-36045	0	0	0	OK
ENSBTAG00000034582	ENSBTAG00000034582	23:7150820-7151201	0	0	0	OK
ENSBTAG00000034611	ENSBTAG00000034611	16:2120376-2121226	0	0	0	OK
ENSBTAG00000034626	ENSBTAG00000034626	8:10095869-10133299	0	0	0	OK
ENSBTAG00000034632	ENSBTAG00000034632	16:2975237-3005255	0	0	0	OK
ENSBTAG00000034633	ENSBTAG00000034633	28:8951739-9026350	0	0	0	OK
ENSBTAG00000034662	ENSBTAG00000034662	9:18473882-18475244	0	0	0	OK
ENSBTAG00000034689	ENSBTAG00000034689	18:2396334-2482363	0	0	0	OK
ENSBTAG00000034700	ENSBTAG00000034700	6:2602608-2653192	0	0	0	OK
ENSBTAG00000034724	ENSBTAG00000034724	15:13611758-13612139	0	0	0	OK
ENSBTAG00000034753	ENSBTAG00000034753	12:14101257-14126892	0	0	0	OK
ENSBTAG00000034761	ENSBTAG00000034761	17:51645902-51648371	0	0	0	OK
ENSBTAG00000034766	ENSBTAG00000034766	19:9877637-9877801	0	0	0	OK
ENSBTAG00000034770	ENSBTAG00000034770	19:9836918-9837133	0	0	0	OK
ENSBTAG00000034785	ENSBTAG00000034785	12:13183733-13266310	0	0	0	OK
ENSBTAG00000034844	ENSBTAG00000034844	6:8432001-8432157	0	0	0	OK
ENSBTAG00000034845	ENSBTAG00000034845	29:7416801-7417182	0	0	0	OK
ENSBTAG00000034849	ENSBTAG00000034849	6:9049870-9050251	0	0	0	OK
ENSBTAG00000034879	ENSBTAG00000034879	23:10282782-10283741	0	0	0	OK
ENSBTAG00000034891	ENSBTAG00000034891	29:5668910-5671609	0	0	0	OK
ENSBTAG00000034901	ENSBTAG00000034901	7:2484674-2485418	0	0	0	OK
ENSBTAG00000034905	ENSBTAG00000034905	4:9583691-9607123	0	0	0	OK
ENSBTAG00000034917	ENSBTAG00000034917	7:10995633-10996758	0	0	0	OK

ENSBTAG00000034921	ENSBTAG00000034921	29:5720358-5721510	0	0	0	OK
ENSBTAG00000034925	ENSBTAG00000034925	9:12969174-12970434	0	0	0	OK
ENSBTAG00000034940	ENSBTAG00000034940	29:5232245-5240315	0	0	0	OK
ENSBTAG00000034948	ENSBTAG00000034948	8:6624457-6624615	0	0	0	OK
ENSBTAG00000034954	ENSBTAG00000034954	27:5560975-5564586	0	0	0	OK
ENSBTAG00000034973	ENSBTAG00000034973	7:2302463-2303051	0	0	0	OK
ENSBTAG00000034985	ENSBTAG00000034985	5:5557938-5559917	0	0	0	OK
ENSBTAG00000035006	ENSBTAG00000035006	15:49922807-49923752	0	0	0	OK
ENSBTAG00000035008	ENSBTAG00000035008	15:49939350-49940289	0	0	0	OK
ENSBTAG00000035012	ENSBTAG00000035012	17:25033670-25037093	0	0	0	OK
ENSBTAG00000035013	ENSBTAG00000035013	27:3150949-3151920	0	0	0	OK
ENSBTAG00000035021	ENSBTAG00000035021	27:3522782-3523156	0	0	0	OK
ENSBTAG00000035030	ENSBTAG00000035030	10:4409117-4418520	0	0	0	OK
ENSBTAG00000035031	ENSBTAG00000035031	15:80842357-80843299	0	0	0	OK
ENSBTAG00000035041	ENSBTAG00000035041	26:5312987-5313106	0	0	0	OK
ENSBTAG00000035076	ENSBTAG00000035076	9:9904567-9904899	0	0	0	OK
ENSBTAG00000035150	ENSBTAG00000035150	X:142878330-142878450	0	0	0	OK
ENSBTAG00000035158	ENSBTAG00000035158	14:1770659-1772329	0	0	0	OK
ENSBTAG00000035172	ENSBTAG00000035172	19:6547150-6549406	0	0	0	OK
ENSBTAG00000035183	ENSBTAG00000035183	27:6194469-6196146	0	0	0	OK
ENSBTAG00000035184	ENSBTAG00000035184	21:4989829-4990204	0	0	0	OK
ENSBTAG00000035199	ENSBTAG00000035199	13:2891528-2891726	0	0	0	OK
ENSBTAG00000035206	ENSBTAG00000035206	28:16655902-16656452	0	0	0	OK
ENSBTAG00000035268	ENSBTAG00000035268	21:1666052-1666171	0	0	0	OK
ENSBTAG00000035293	ENSBTAG00000035293	8:1558326-1559366	0	0	0	OK
ENSBTAG00000035370	ENSBTAG00000035370	9:73110607-73149994	0	0	0	OK
ENSBTAG00000035373	ENSBTAG00000035373	25:27227589-27230804	0	0	0	OK
ENSBTAG00000035379	ENSBTAG00000035379	9:30223947-30224832	0	0	0	OK
ENSBTAG00000035392	ENSBTAG00000035392	21:45339881-45340345	0	0	0	OK
ENSBTAG00000035480	ENSBTAG00000035480	6:69985403-69985513	0	0	0	OK

ENSBTAG00000035487	ENSBTAG00000035487	15:79563650-79564604	0	0	0	OK
ENSBTAG00000035489	ENSBTAG00000035489	15:79507388-79508721	0	0	0	OK
ENSBTAG00000035493	ENSBTAG00000035493	12:25281100-25281531	0	0	0	OK
ENSBTAG00000035530	ENSBTAG00000035530	10:25361714-25362423	0	0	0	OK
ENSBTAG00000035606	ENSBTAG00000035606	17:36034531-36037275	0	0	0	OK
ENSBTAG00000035697	ENSBTAG00000035697	3:77870348-77950527	0	0	0	OK
ENSBTAG00000035735	ENSBTAG00000035735	15:6585515-6607013	0	0	0	OK
ENSBTAG00000035745	ENSBTAG00000035745	X:118671360-118675384	0	0	0	OK
ENSBTAG00000035768	ENSBTAG00000035768	8:7099560-7102535	0	0	0	OK
ENSBTAG00000035776	ENSBTAG00000035776	6:96794893-97503220	0	0	0	OK
ENSBTAG00000035777	ENSBTAG00000035777	7:15482924-15485011	0	0	0	OK
ENSBTAG00000035803	ENSBTAG00000035803	15:232040-232974	0	0	0	OK
ENSBTAG00000035883	ENSBTAG00000035883	13:84235899-84236838	0	0	0	OK
ENSBTAG00000035926	ENSBTAG00000035926	12:707017-708407	0	0	0	OK
ENSBTAG00000035945	ENSBTAG00000035945	4:50336300-50347162	0	0	0	OK
ENSBTAG00000035952	ENSBTAG00000035952	8:95784642-95785681	0	0	0	OK
ENSBTAG00000035966	ENSBTAG00000035966	7:59053806-59075334	0	0	0	OK
ENSBTAG00000035986	ENSBTAG00000035986	15:80477851-80478754	0	0	0	OK
ENSBTAG00000035988	ENSBTAG00000035988	15:80420320-80421262	0	0	0	OK
ENSBTAG00000035989	ENSBTAG00000035989	15:80737479-80738442	0	0	0	OK
ENSBTAG00000035995	ENSBTAG00000035995	21:69455151-69466051	0	0	0	OK
ENSBTAG00000036028	ENSBTAG00000036028	X:57184739-57186320	0	0	0	OK
ENSBTAG00000036045	ENSBTAG00000036045	14:249365-249486	0	0	0	OK
ENSBTAG00000036061	ENSBTAG00000036061	9:88262867-88266693	0	0	0	OK
ENSBTAG00000036078	ENSBTAG00000036078	5:97081920-97101600	0	0	0	OK
ENSBTAG00000036099	ENSBTAG00000036099	11:99347611-99352446	0	0	0	OK
ENSBTAG00000036127	ENSBTAG00000036127	26:23747357-23764458	0	0	0	OK
ENSBTAG00000036131	ENSBTAG00000036131	15:81172710-81173673	0	0	0	OK
ENSBTAG00000036144	ENSBTAG00000036144	X:23534849-23539249	0	0	0	OK
ENSBTAG00000036175	ENSBTAG00000036175	7:9452978-9453902	0	0	0	OK

ENSBTAG00000036202	ENSBTAG00000036202	10:23221369-23221949	0	0	0	OK
ENSBTAG00000036258	ENSBTAG00000036258	2:25570736-25571355	0	0	0	OK
ENSBTAG00000036287	ENSBTAG00000036287	9:85917824-86020619	0	0	0	OK
ENSBTAG00000036321	ENSBTAG00000036321	17:25066970-25068298	0	0	0	OK
ENSBTAG00000036356	ENSBTAG00000036356	21:67566816-67566899	0	0	0	OK
ENSBTAG00000036357	ENSBTAG00000036357	22:9738597-9738681	0	0	0	OK
ENSBTAG00000036358	ENSBTAG00000036358	27:4787123-4787336	0	0	0	OK
ENSBTAG00000036359	ENSBTAG00000036359	X:67978439-67978550	0	0	0	OK
ENSBTAG00000036360	ENSBTAG00000036360	21:67573234-67573315	0	0	0	OK
ENSBTAG00000036361	ENSBTAG00000036361	19:39081164-39081250	0	0	0	OK
ENSBTAG00000036362	ENSBTAG00000036362	25:36892045-36892125	0	0	0	OK
ENSBTAG00000036363	ENSBTAG00000036363	13:55227291-55227386	0	0	0	OK
ENSBTAG00000036364	ENSBTAG00000036364	21:67603864-67603945	0	0	0	OK
ENSBTAG00000036365	ENSBTAG00000036365	16:77561998-77562079	0	0	0	OK
ENSBTAG00000036366	ENSBTAG00000036366	4:10653526-10653616	0	0	0	OK
ENSBTAG00000036367	ENSBTAG00000036367	X:82023910-82024005	0	0	0	OK
ENSBTAG00000036368	ENSBTAG00000036368	21:67583072-67583147	0	0	0	OK
ENSBTAG00000036369	ENSBTAG00000036369	15:33353394-33353470	0	0	0	OK
ENSBTAG00000036371	ENSBTAG00000036371	25:13339482-13339565	0	0	0	OK
ENSBTAG00000036372	ENSBTAG00000036372	1:79250530-79250616	0	0	0	OK
ENSBTAG00000036373	ENSBTAG00000036373	1:59398272-59398366	0	0	0	OK
ENSBTAG00000036374	ENSBTAG00000036374	X:92898441-92898506	0	0	0	OK
ENSBTAG00000036375	ENSBTAG00000036375	20:19588110-19588208	0	0	0	OK
ENSBTAG00000036376	ENSBTAG00000036376	X:92903795-92903880	0	0	0	OK
ENSBTAG00000036379	ENSBTAG00000036379	21:67561868-67561954	0	0	0	OK
ENSBTAG00000036380	ENSBTAG00000036380	21:25913770-25913853	0	0	0	OK
ENSBTAG00000036381	ENSBTAG00000036381	5:26157878-26157971	0	0	0	OK
ENSBTAG00000036383	ENSBTAG00000036383	21:67566506-67566589	0	0	0	OK
ENSBTAG00000036386	ENSBTAG00000036386	19:38496983-38497085	0	0	0	OK
ENSBTAG00000036388	ENSBTAG00000036388	29:50061106-50061174	0	0	0	OK

ENSBTAG00000036389	ENSBTAG00000036389	12:19596199-19596288	0	0	0	OK
ENSBTAG00000036391	ENSBTAG00000036391	19:23651569-23651680	0	0	0	OK
ENSBTAG00000036392	ENSBTAG00000036392	18:61145843-61145922	0	0	0	OK
ENSBTAG00000036395	ENSBTAG00000036395	16:52520088-52520172	0	0	0	OK
ENSBTAG00000036396	ENSBTAG00000036396	21:67569675-67569760	0	0	0	OK
ENSBTAG00000036397	ENSBTAG00000036397	6:14162435-14162510	0	0	0	OK
ENSBTAG00000036398	ENSBTAG00000036398	21:67587314-67587411	0	0	0	OK
ENSBTAG00000036399	ENSBTAG00000036399	21:67563550-67563654	0	0	0	OK
ENSBTAG00000036400	ENSBTAG00000036400	23:24308041-24308127	0	0	0	OK
ENSBTAG00000036402	ENSBTAG00000036402	21:67593936-67594047	0	0	0	OK
ENSBTAG00000036403	ENSBTAG00000036403	X:92893817-92893907	0	0	0	OK
ENSBTAG00000036404	ENSBTAG00000036404	1:10227275-10227340	0	0	0	OK
ENSBTAG00000036406	ENSBTAG00000036406	9:63751181-63751261	0	0	0	OK
ENSBTAG00000036407	ENSBTAG00000036407	21:28046408-28046518	0	0	0	OK
ENSBTAG00000036408	ENSBTAG00000036408	6:14163148-14163218	0	0	0	OK
ENSBTAG00000036410	ENSBTAG00000036410	5:103859004-103859094	0	0	0	OK
ENSBTAG00000036411	ENSBTAG00000036411	X:81951222-81951294	0	0	0	OK
ENSBTAG00000036414	ENSBTAG00000036414	19:20796859-20796930	0	0	0	OK
ENSBTAG00000036415	ENSBTAG00000036415	21:67598848-67598967	0	0	0	OK
ENSBTAG00000036417	ENSBTAG00000036417	5:117120187-117120270	0	0	0	OK
ENSBTAG00000036418	ENSBTAG00000036418	X:99936304-99936412	0	0	0	OK
ENSBTAG00000036419	ENSBTAG00000036419	X:18185438-18185534	0	0	0	OK
ENSBTAG00000036420	ENSBTAG00000036420	X:92899697-92899781	0	0	0	OK
ENSBTAG00000036421	ENSBTAG00000036421	14:30815410-30815521	0	0	0	OK
ENSBTAG00000036422	ENSBTAG00000036422	6:119145903-119145984	0	0	0	OK
ENSBTAG00000036423	ENSBTAG00000036423	26:22930889-22930995	0	0	0	OK
ENSBTAG00000036425	ENSBTAG00000036425	21:19989063-19989161	0	0	0	OK
ENSBTAG00000036426	ENSBTAG00000036426	21:67598038-67598122	0	0	0	OK
ENSBTAG00000036428	ENSBTAG00000036428	6:14162971-14163041	0	0	0	OK
ENSBTAG00000036429	ENSBTAG00000036429	6:14162578-14162646	0	0	0	OK

ENSBTAG00000036430	ENSBTAG00000036430	16:77562537-77562625	0	0	0	OK
ENSBTAG00000036431	ENSBTAG00000036431	21:67571824-67571904	0	0	0	OK
ENSBTAG00000036433	ENSBTAG00000036433	21:67584371-67584449	0	0	0	OK
ENSBTAG00000036435	ENSBTAG00000036435	11:38482487-38482597	0	0	0	OK
ENSBTAG00000036436	ENSBTAG00000036436	2:107963021-107963111	0	0	0	OK
ENSBTAG00000036437	ENSBTAG00000036437	19:35249051-35249145	0	0	0	OK
ENSBTAG00000036438	ENSBTAG00000036438	29:16686223-16686332	0	0	0	OK
ENSBTAG00000036440	ENSBTAG00000036440	17:46348795-46348858	0	0	0	OK
ENSBTAG00000036442	ENSBTAG00000036442	22:42636103-42636210	0	0	0	OK
ENSBTAG00000036443	ENSBTAG00000036443	17:74100849-74100944	0	0	0	OK
ENSBTAG00000036444	ENSBTAG00000036444	21:67565765-67565853	0	0	0	OK
ENSBTAG00000036445	ENSBTAG00000036445	21:67594685-67594765	0	0	0	OK
ENSBTAG00000036451	ENSBTAG00000036451	15:2385320-2385438	0	0	0	OK
ENSBTAG00000036475	ENSBTAG00000036475	4:99730753-99730872	0	0	0	OK
ENSBTAG00000036476	ENSBTAG00000036476	4:84700294-84700412	0	0	0	OK
ENSBTAG00000036480	ENSBTAG00000036480	5:92496479-92496597	0	0	0	OK
ENSBTAG00000036488	ENSBTAG00000036488	27:18627504-18627623	0	0	0	OK
ENSBTAG00000036516	ENSBTAG00000036516	16:44727383-44727503	0	0	0	OK
ENSBTAG00000036542	ENSBTAG00000036542	8:103059950-103060057	0	0	0	OK
ENSBTAG00000036553	ENSBTAG00000036553	12:9986933-9987011	0	0	0	OK
ENSBTAG00000036562	ENSBTAG00000036562	5:67134733-67134897	0	0	0	OK
ENSBTAG00000036578	ENSBTAG00000036578	9:67558795-67558914	0	0	0	OK
ENSBTAG00000036587	ENSBTAG00000036587	7:100463077-100463285	0	0	0	OK
ENSBTAG00000036603	ENSBTAG00000036603	6:107122793-107122915	0	0	0	OK
ENSBTAG00000036616	ENSBTAG00000036616	22:30953128-30953243	0	0	0	OK
ENSBTAG00000036618	ENSBTAG00000036618	7:87601104-87601319	0	0	0	OK
ENSBTAG00000036621	ENSBTAG00000036621	5:95409620-95409742	0	0	0	OK
ENSBTAG00000036625	ENSBTAG00000036625	12:69697977-69698166	0	0	0	OK
ENSBTAG00000036628	ENSBTAG00000036628	16:31142357-31142475	0	0	0	OK
ENSBTAG00000036635	ENSBTAG00000036635	10:70444592-70444715	0	0	0	OK

ENSBTAG00000036637	ENSBTAG00000036637	25:35347881-35348001	0	0	0	OK
ENSBTAG00000036644	ENSBTAG00000036644	16:34666249-34666362	0	0	0	OK
ENSBTAG00000036646	ENSBTAG00000036646	4:89159276-89159400	0	0	0	OK
ENSBTAG00000036649	ENSBTAG00000036649	6:105911051-105911170	0	0	0	OK
ENSBTAG00000036666	ENSBTAG00000036666	24:8343334-8343443	0	0	0	OK
ENSBTAG00000036678	ENSBTAG00000036678	27:22455704-22455824	0	0	0	OK
ENSBTAG00000036732	ENSBTAG00000036732	24:21076437-21076550	0	0	0	OK
ENSBTAG00000036743	ENSBTAG00000036743	25:18809552-18809671	0	0	0	OK
ENSBTAG00000036744	ENSBTAG00000036744	15:18251927-18252044	0	0	0	OK
ENSBTAG00000036754	ENSBTAG00000036754	2:38143424-38143572	0	0	0	OK
ENSBTAG00000036766	ENSBTAG00000036766	20:22349406-22349558	0	0	0	OK
ENSBTAG00000036777	ENSBTAG00000036777	27:38100690-38100807	0	0	0	OK
ENSBTAG00000036794	ENSBTAG00000036794	17:54615160-54615277	0	0	0	OK
ENSBTAG00000036807	ENSBTAG00000036807	25:23596788-23596907	0	0	0	OK
ENSBTAG00000036823	ENSBTAG00000036823	28:4487758-4487879	0	0	0	OK
ENSBTAG00000036841	ENSBTAG00000036841	1:15438159-15438279	0	0	0	OK
ENSBTAG00000036851	ENSBTAG00000036851	19:52366967-52367112	0	0	0	OK
ENSBTAG00000036856	ENSBTAG00000036856	7:23852875-23852996	0	0	0	OK
ENSBTAG00000036858	ENSBTAG00000036858	11:74853109-74853239	0	0	0	OK
ENSBTAG00000036889	ENSBTAG00000036889	11:51840157-51840279	0	0	0	OK
ENSBTAG00000036902	ENSBTAG00000036902	14:68580403-68580521	0	0	0	OK
ENSBTAG00000036908	ENSBTAG00000036908	X:17252323-17252442	0	0	0	OK
ENSBTAG00000036921	ENSBTAG00000036921	8:60639419-60639535	0	0	0	OK
ENSBTAG00000036927	ENSBTAG00000036927	19:9806853-9807012	0	0	0	OK
ENSBTAG00000036929	ENSBTAG00000036929	X:61789726-61789936	0	0	0	OK
ENSBTAG00000036933	ENSBTAG00000036933	1:90393020-90393138	0	0	0	OK
ENSBTAG00000036942	ENSBTAG00000036942	14:11759868-11759979	0	0	0	OK
ENSBTAG00000036945	ENSBTAG00000036945	16:14276653-14276772	0	0	0	OK
ENSBTAG00000036946	ENSBTAG00000036946	1:72885068-72885214	0	0	0	OK
ENSBTAG00000036950	ENSBTAG00000036950	5:42917113-42917233	0	0	0	OK

ENSBTAG00000036958	ENSBTAG00000036958	29:29826313-29826473	0	0	0	OK
ENSBTAG00000036962	ENSBTAG00000036962	24:62710864-62710984	0	0	0	OK
ENSBTAG00000036968	ENSBTAG00000036968	10:74049335-74049548	0	0	0	OK
ENSBTAG00000036970	ENSBTAG00000036970	27:36072655-36072769	0	0	0	OK
ENSBTAG00000036978	ENSBTAG00000036978	9:55267189-55267308	0	0	0	OK
ENSBTAG00000036982	ENSBTAG00000036982	10:64188219-64188336	0	0	0	OK
ENSBTAG00000036993	ENSBTAG00000036993	2:48352290-48352468	0	0	0	OK
ENSBTAG00000036995	ENSBTAG00000036995	9:70106219-70106320	0	0	0	OK
ENSBTAG00000036997	ENSBTAG00000036997	19:9863517-9863697	0	0	0	OK
ENSBTAG00000037006	ENSBTAG00000037006	26:25366086-25366206	0	0	0	OK
ENSBTAG00000037009	ENSBTAG00000037009	11:84872439-84872570	0	0	0	OK
ENSBTAG00000037010	ENSBTAG00000037010	25:4802877-4802997	0	0	0	OK
ENSBTAG00000037013	ENSBTAG00000037013	21:45387108-45387272	0	0	0	OK
ENSBTAG00000037026	ENSBTAG00000037026	11:90874838-90874945	0	0	0	OK
ENSBTAG00000037030	ENSBTAG00000037030	27:37373800-37374001	0	0	0	OK
ENSBTAG00000037072	ENSBTAG00000037072	16:45211153-45211316	0	0	0	OK
ENSBTAG00000037087	ENSBTAG00000037087	8:903155-903275	0	0	0	OK
ENSBTAG00000037096	ENSBTAG00000037096	1:150617256-150617374	0	0	0	OK
ENSBTAG00000037108	ENSBTAG00000037108	4:104930512-104930631	0	0	0	OK
ENSBTAG00000037110	ENSBTAG00000037110	5:114006210-114006360	0	0	0	OK
ENSBTAG00000037111	ENSBTAG00000037111	8:66080571-66080675	0	0	0	OK
ENSBTAG00000037147	ENSBTAG00000037147	20:64476312-64476463	0	0	0	OK
ENSBTAG00000037153	ENSBTAG00000037153	11:23132696-23132810	0	0	0	OK
ENSBTAG00000037167	ENSBTAG00000037167	11:50559387-50559506	0	0	0	OK
ENSBTAG00000037174	ENSBTAG00000037174	28:1329274-1329394	0	0	0	OK
ENSBTAG00000037179	ENSBTAG00000037179	21:45436285-45436507	0	0	0	OK
ENSBTAG00000037182	ENSBTAG00000037182	7:41267943-41268065	0	0	0	OK
ENSBTAG00000037187	ENSBTAG00000037187	3:22850388-22850546	0	0	0	OK
ENSBTAG00000037199	ENSBTAG00000037199	10:65798495-65798619	0	0	0	OK
ENSBTAG00000037205	ENSBTAG00000037205	13:60055443-60055560	0	0	0	OK

ENSBTAG00000037206	ENSBTAG00000037206	20:39331272-39331423	0	0	0	OK
ENSBTAG00000037226	ENSBTAG00000037226	10:51823325-51823444	0	0	0	OK
ENSBTAG00000037229	ENSBTAG00000037229	7:38692504-38692623	0	0	0	OK
ENSBTAG00000037239	ENSBTAG00000037239	18:10408494-10408613	0	0	0	OK
ENSBTAG00000037243	ENSBTAG00000037243	17:55250870-55250978	0	0	0	OK
ENSBTAG00000037250	ENSBTAG00000037250	17:61342996-61343081	0	0	0	OK
ENSBTAG00000037257	ENSBTAG00000037257	23:51943047-51943121	0	0	0	OK
ENSBTAG00000037261	ENSBTAG00000037261	19:56065972-56066043	0	0	0	OK
ENSBTAG00000037263	ENSBTAG00000037263	21:67602343-67602427	0	0	0	OK
ENSBTAG00000037264	ENSBTAG00000037264	27:36261817-36261941	0	0	0	OK
ENSBTAG00000037265	ENSBTAG00000037265	21:67423436-67423532	0	0	0	OK
ENSBTAG00000037268	ENSBTAG00000037268	23:16064875-16064961	0	0	0	OK
ENSBTAG00000037274	ENSBTAG00000037274	1:112211118-112211190	0	0	0	OK
ENSBTAG00000037275	ENSBTAG00000037275	3:16374603-16374686	0	0	0	OK
ENSBTAG00000037277	ENSBTAG00000037277	3:113079271-113079366	0	0	0	OK
ENSBTAG00000037288	ENSBTAG00000037288	2:108527224-108527289	0	0	0	OK
ENSBTAG00000037291	ENSBTAG00000037291	14:66995016-66995093	0	0	0	OK
ENSBTAG00000037292	ENSBTAG00000037292	8:15159559-15159636	0	0	0	OK
ENSBTAG00000037293	ENSBTAG00000037293	23:52424202-52424293	0	0	0	OK
ENSBTAG00000037296	ENSBTAG00000037296	16:50640459-50640548	0	0	0	OK
ENSBTAG00000037297	ENSBTAG00000037297	27:33441611-33441671	0	0	0	OK
ENSBTAG00000037300	ENSBTAG00000037300	11:74482708-74482801	0	0	0	OK
ENSBTAG00000037302	ENSBTAG00000037302	21:44439575-44439657	0	0	0	OK
ENSBTAG00000037304	ENSBTAG00000037304	6:93691970-93692054	0	0	0	OK
ENSBTAG00000037306	ENSBTAG00000037306	5:70793314-70793420	0	0	0	OK
ENSBTAG00000037307	ENSBTAG00000037307	7:50687423-50687499	0	0	0	OK
ENSBTAG00000037311	ENSBTAG00000037311	6:6499943-6500030	0	0	0	OK
ENSBTAG00000037316	ENSBTAG00000037316	29:27239795-27239877	0	0	0	OK
ENSBTAG00000037319	ENSBTAG00000037319	13:58091935-58092013	0	0	0	OK
ENSBTAG00000037322	ENSBTAG00000037322	18:33396193-33396277	0	0	0	OK

ENSBTAG00000037325	ENSBTAG00000037325	2:46645169-46645242	0	0	0	OK
ENSBTAG00000037328	ENSBTAG00000037328	26:10652803-10652870	0	0	0	OK
ENSBTAG00000037330	ENSBTAG00000037330	7:93431856-93431943	0	0	0	OK
ENSBTAG00000037332	ENSBTAG00000037332	28:14487362-14487431	0	0	0	OK
ENSBTAG00000037336	ENSBTAG00000037336	6:21286137-21286215	0	0	0	OK
ENSBTAG00000037339	ENSBTAG00000037339	10:21340622-21340702	0	0	0	OK
ENSBTAG00000037348	ENSBTAG00000037348	25:1792864-1792957	0	0	0	OK
ENSBTAG00000037350	ENSBTAG00000037350	23:27501550-27501629	0	0	0	OK
ENSBTAG00000037353	ENSBTAG00000037353	X:35631518-35631585	0	0	0	OK
ENSBTAG00000037354	ENSBTAG00000037354	1:25664274-25664345	0	0	0	OK
ENSBTAG00000037357	ENSBTAG00000037357	11:15471296-15471417	0	0	0	OK
ENSBTAG00000037358	ENSBTAG00000037358	22:55072938-55073012	0	0	0	OK
ENSBTAG00000037360	ENSBTAG00000037360	11:72985385-72985468	0	0	0	OK
ENSBTAG00000037363	ENSBTAG00000037363	4:117156949-117157039	0	0	0	OK
ENSBTAG00000037365	ENSBTAG00000037365	2:15282525-15282618	0	0	0	OK
ENSBTAG00000037369	ENSBTAG00000037369	8:15183223-15183304	0	0	0	OK
ENSBTAG00000037377	ENSBTAG00000037377	22:49516823-49520467	0	0	0	OK
ENSBTAG00000037381	ENSBTAG00000037381	23:28626338-28638869	0	0	0	OK
ENSBTAG00000037389	ENSBTAG00000037389	15:66987986-67099792	0	0	0	OK
ENSBTAG00000037399	ENSBTAG00000037399	14:35568650-35569172	0	0	0	OK
ENSBTAG00000037404	ENSBTAG00000037404	29:45517058-45519576	0	0	0	OK
ENSBTAG00000037410	ENSBTAG00000037410	22:49603517-49607502	0	0	0	OK
ENSBTAG00000037411	ENSBTAG00000037411	10:27216969-27217899	0	0	0	OK
ENSBTAG00000037413	ENSBTAG00000037413	X:62882938-63056847	0	0	0	OK
ENSBTAG00000037415	ENSBTAG00000037415	17:60146303-60146762	0	0	0	OK
ENSBTAG00000037416	ENSBTAG00000037416	X:111752239-111752593	0	0	0	OK
ENSBTAG00000037418	ENSBTAG00000037418	7:45067546-45070582	0	0	0	OK
ENSBTAG00000037432	ENSBTAG00000037432	3:103480290-103481238	0	0	0	OK
ENSBTAG00000037454	ENSBTAG00000037454	25:34863904-34864017	0	0	0	OK
ENSBTAG00000037461	ENSBTAG00000037461	18:46857106-46858153	0	0	0	OK

ENSBTAG00000037492	ENSBTAG00000037492	13:59724646-59724754	0	0	0	OK
ENSBTAG00000037494	ENSBTAG00000037494	16:13512072-13536147	0	0	0	OK
ENSBTAG00000037496	ENSBTAG00000037496	X:119317658-119319029	0	0	0	OK
ENSBTAG00000037500	ENSBTAG00000037500	25:1744660-1744720	0	0	0	OK
ENSBTAG00000037505	ENSBTAG00000037505	15:47784032-47784971	0	0	0	OK
ENSBTAG00000037510	ENSBTAG00000037510	4:114111964-114116379	0	0	0	OK
ENSBTAG00000037516	ENSBTAG00000037516	13:16262483-16263567	0	0	0	OK
ENSBTAG00000037538	ENSBTAG00000037538	15:83931871-83932810	0	0	0	OK
ENSBTAG00000037542	ENSBTAG00000037542	11:93584333-93585269	0	0	0	OK
ENSBTAG00000037547	ENSBTAG00000037547	1:1776498-1777332	0	0	0	OK
ENSBTAG00000037552	ENSBTAG00000037552	5:244153-268891	0	0	0	OK
ENSBTAG00000037555	ENSBTAG00000037555	3:103434900-103443375	0	0	0	OK
ENSBTAG00000037576	ENSBTAG00000037576	20:57027188-57145663	0	0	0	OK
ENSBTAG00000037582	ENSBTAG00000037582	18:62378121-62380014	0	0	0	OK
ENSBTAG00000037586	ENSBTAG00000037586	11:21434979-21435448	0	0	0	OK
ENSBTAG00000037595	ENSBTAG00000037595	18:65993794-65995066	0	0	0	OK
ENSBTAG00000037596	ENSBTAG00000037596	5:17740810-17740929	0	0	0	OK
ENSBTAG00000037598	ENSBTAG00000037598	9:100279907-100281548	0	0	0	OK
ENSBTAG00000037600	ENSBTAG00000037600	8:84843153-84843546	0	0	0	OK
ENSBTAG00000037604	ENSBTAG00000037604	19:28012877-28016763	0	0	0	OK
ENSBTAG00000037605	ENSBTAG00000037605	23:25426329-25430097	0	0	0	OK
ENSBTAG00000037624	ENSBTAG00000037624	23:51081802-51081911	0	0	0	OK
ENSBTAG00000037626	ENSBTAG00000037626	22:57714721-57714862	0	0	0	OK
ENSBTAG00000037629	ENSBTAG00000037629	5:59716892-59717840	0	0	0	OK
ENSBTAG00000037632	ENSBTAG00000037632	5:113614529-113620220	0	0	0	OK
ENSBTAG00000037637	ENSBTAG00000037637	X:55905196-55905343	0	0	0	OK
ENSBTAG00000037647	ENSBTAG00000037647	10:28167664-28168600	0	0	0	OK
ENSBTAG00000037660	ENSBTAG00000037660	19:36433971-36434837	0	0	0	OK
ENSBTAG00000037666	ENSBTAG00000037666	15:79585673-79586573	0	0	0	OK
ENSBTAG00000037679	ENSBTAG00000037679	10:21447235-21448497	0	0	0	OK

ENSBTAG00000037709	ENSBTAG00000037709	13:75339810-75340081	0	0	0	OK
ENSBTAG00000037718	ENSBTAG00000037718	9:71803196-71804207	0	0	0	OK
ENSBTAG00000037743	ENSBTAG00000037743	5:103664842-103687826	0	0	0	OK
ENSBTAG00000037757	ENSBTAG00000037757	13:52885988-52942347	0	0	0	OK
ENSBTAG00000037767	ENSBTAG00000037767	11:93638722-93639658	0	0	0	OK
ENSBTAG00000037778	ENSBTAG00000037778	6:90811061-90813079	0	0	0	OK
ENSBTAG00000037779	ENSBTAG00000037779	X:47257803-47257925	0	0	0	OK
ENSBTAG00000037782	ENSBTAG00000037782	25:1628446-1628539	0	0	0	OK
ENSBTAG00000037788	ENSBTAG00000037788	X:125748739-125748852	0	0	0	OK
ENSBTAG00000037793	ENSBTAG00000037793	11:89100477-89101344	0	0	0	OK
ENSBTAG00000037800	ENSBTAG00000037800	5:111045316-111048603	0	0	0	OK
ENSBTAG00000037811	ENSBTAG00000037811	19:16232967-16234839	0	0	0	OK
ENSBTAG00000037815	ENSBTAG00000037815	15:48997630-48999478	0	0	0	OK
ENSBTAG00000037816	ENSBTAG00000037816	2:82928222-82928444	0	0	0	OK
ENSBTAG00000037818	ENSBTAG00000037818	18:56567742-56568270	0	0	0	OK
ENSBTAG00000037830	ENSBTAG00000037830	18:63213693-63215905	0	0	0	OK
ENSBTAG00000037831	ENSBTAG00000037831	7:111119132-111119239	0	0	0	OK
ENSBTAG00000037833	ENSBTAG00000037833	13:46545941-46546045	0	0	0	OK
ENSBTAG00000037842	ENSBTAG00000037842	5:58260956-58261892	0	0	0	OK
ENSBTAG00000037846	ENSBTAG00000037846	4:5204023-5204133	0	0	0	OK
ENSBTAG00000037853	ENSBTAG00000037853	15:82890480-82891458	0	0	0	OK
ENSBTAG00000037868	ENSBTAG00000037868	X:96939476-96939600	0	0	0	OK
ENSBTAG00000037878	ENSBTAG00000037878	15:80338055-80338526	0	0	0	OK
ENSBTAG00000037881	ENSBTAG00000037881	18:63915226-63915565	0	0	0	OK
ENSBTAG00000037886	ENSBTAG00000037886	15:50348406-50349375	0	0	0	OK
ENSBTAG00000037897	ENSBTAG00000037897	14:13596702-13596765	0	0	0	OK
ENSBTAG00000037905	ENSBTAG00000037905	7:39194947-39195033	0	0	0	OK
ENSBTAG00000037913	ENSBTAG00000037913	28:44925708-44927696	0	0	0	OK
ENSBTAG00000037924	ENSBTAG00000037924	10:23978359-23978755	0	0	0	OK
ENSBTAG00000037925	ENSBTAG00000037925	13:74347295-74347738	0	0	0	OK

ENSBTAG00000037931	ENSBTAG00000037931	X:97604640-97605063	0	0	0	OK
ENSBTAG00000037938	ENSBTAG00000037938	21:45695685-45724815	0	0	0	OK
ENSBTAG00000037943	ENSBTAG00000037943	19:41974545-41975145	0	0	0	OK
ENSBTAG00000037948	ENSBTAG00000037948	3:95691966-95692099	0	0	0	OK
ENSBTAG00000037953	ENSBTAG00000037953	21:55967973-55968074	0	0	0	OK
ENSBTAG00000037959	ENSBTAG00000037959	10:26998518-26999445	0	0	0	OK
ENSBTAG00000037966	ENSBTAG00000037966	18:50368467-50369398	0	0	0	OK
ENSBTAG00000037971	ENSBTAG00000037971	8:66102894-66114341	0	0	0	OK
ENSBTAG00000037977	ENSBTAG00000037977	21:68151113-68151509	0	0	0	OK
ENSBTAG00000037988	ENSBTAG00000037988	23:30115122-30124154	0	0	0	OK
ENSBTAG00000038011	ENSBTAG00000038011	11:44206548-44365502	0	0	0	OK
ENSBTAG00000038026	ENSBTAG00000038026	1:32160179-32160333	0	0	0	OK
ENSBTAG00000038027	ENSBTAG00000038027	5:33113670-33113964	0	0	0	OK
ENSBTAG00000038030	ENSBTAG00000038030	4:12616772-12618524	0	0	0	OK
ENSBTAG00000038031	ENSBTAG00000038031	15:46624352-46625288	0	0	0	OK
ENSBTAG00000038035	ENSBTAG00000038035	20:60254277-60254347	0	0	0	OK
ENSBTAG00000038043	ENSBTAG00000038043	5:41696371-41801868	0	0	0	OK
ENSBTAG00000038047	ENSBTAG00000038047	15:30202577-30203886	0	0	0	OK
ENSBTAG00000038050	ENSBTAG00000038050	18:47700142-47727252	0	0	0	OK
ENSBTAG00000038055	ENSBTAG00000038055	7:45789551-45801206	0	0	0	OK
ENSBTAG00000038057	ENSBTAG00000038057	13:84229446-84230490	0	0	0	OK
ENSBTAG00000038062	ENSBTAG00000038062	15:16242001-16243411	0	0	0	OK
ENSBTAG00000038063	ENSBTAG00000038063	26:10211197-10247852	0	0	0	OK
ENSBTAG00000038067	ENSBTAG00000038067	18:24106721-24108521	0	0	0	OK
ENSBTAG00000038073	ENSBTAG00000038073	21:68149405-68149541	0	0	0	OK
ENSBTAG00000038074	ENSBTAG00000038074	25:36995860-37002546	0	0	0	OK
ENSBTAG00000038080	ENSBTAG00000038080	21:35229656-35231716	0	0	0	OK
ENSBTAG00000038082	ENSBTAG00000038082	3:120079761-120081083	0	0	0	OK
ENSBTAG00000038086	ENSBTAG00000038086	8:60681840-60682800	0	0	0	OK
ENSBTAG00000038094	ENSBTAG00000038094	23:9451989-9452059	0	0	0	OK

ENSBTAG00000038106	ENSBTAG00000038106	9:31434816-31434919	0	0	0	OK
ENSBTAG00000038116	ENSBTAG00000038116	11:49493203-49497907	0	0	0	OK
ENSBTAG00000038125	ENSBTAG00000038125	18:58791961-58792300	0	0	0	OK
ENSBTAG00000038146	ENSBTAG00000038146	5:113596983-113607641	0	0	0	OK
ENSBTAG00000038151	ENSBTAG00000038151	7:17118953-17125096	0	0	0	OK
ENSBTAG00000038155	ENSBTAG00000038155	10:39410673-39410837	0	0	0	OK
ENSBTAG00000038166	ENSBTAG00000038166	28:2751059-2751974	0	0	0	OK
ENSBTAG00000038170	ENSBTAG00000038170	15:50767633-50768575	0	0	0	OK
ENSBTAG00000038176	ENSBTAG00000038176	10:13515814-13515957	0	0	0	OK
ENSBTAG00000038178	ENSBTAG00000038178	28:13138754-13142845	0	0	0	OK
ENSBTAG00000038182	ENSBTAG00000038182	29:28302881-28303964	0	0	0	OK
ENSBTAG00000038183	ENSBTAG00000038183	11:100050815-100051802	0	0	0	OK
ENSBTAG00000038205	ENSBTAG00000038205	X:67896114-67896222	0	0	0	OK
ENSBTAG00000038206	ENSBTAG00000038206	21:67581369-67581455	0	0	0	OK
ENSBTAG00000038207	ENSBTAG00000038207	5:71177183-71177276	0	0	0	OK
ENSBTAG00000038214	ENSBTAG00000038214	6:86982495-87008610	0	0	0	OK
ENSBTAG00000038224	ENSBTAG00000038224	11:78270222-78274248	0	0	0	OK
ENSBTAG00000038238	ENSBTAG00000038238	16:60651005-60651572	0	0	0	OK
ENSBTAG00000038241	ENSBTAG00000038241	4:113602366-113604046	0	0	0	OK
ENSBTAG00000038242	ENSBTAG00000038242	28:19569093-19569184	0	0	0	OK
ENSBTAG00000038245	ENSBTAG00000038245	15:45542445-45543414	0	0	0	OK
ENSBTAG00000038252	ENSBTAG00000038252	1:47898424-47898483	0	0	0	OK
ENSBTAG00000038257	ENSBTAG00000038257	11:25403-26705	0	0	0	OK
ENSBTAG00000038259	ENSBTAG00000038259	10:55107741-55107854	0	0	0	OK
ENSBTAG00000038260	ENSBTAG00000038260	13:44203635-44214277	0	0	0	OK
ENSBTAG00000038272	ENSBTAG00000038272	12:90032242-90033352	0	0	0	OK
ENSBTAG00000038276	ENSBTAG00000038276	14:61523729-61523875	0	0	0	OK
ENSBTAG00000038307	ENSBTAG00000038307	4:106541483-106541954	0	0	0	OK
ENSBTAG00000038317	ENSBTAG00000038317	15:45528724-45529666	0	0	0	OK
ENSBTAG00000038322	ENSBTAG00000038322	18:55369205-55371100	0	0	0	OK

ENSBTAG00000038330	ENSBTAG00000038330	25:556859-558249	0	0	0	OK
ENSBTAG00000038337	ENSBTAG00000038337	7:52285862-52287943	0	0	0	OK
ENSBTAG00000038351	ENSBTAG00000038351	5:441592-442266	0	0	0	OK
ENSBTAG00000038366	ENSBTAG00000038366	3:11883111-11883708	0	0	0	OK
ENSBTAG00000038368	ENSBTAG00000038368	6:16367634-16367865	0	0	0	OK
ENSBTAG00000038370	ENSBTAG00000038370	28:16247242-16247390	0	0	0	OK
ENSBTAG00000038379	ENSBTAG00000038379	X:39983831-39984337	0	0	0	OK
ENSBTAG00000038386	ENSBTAG00000038386	19:41985181-41986261	0	0	0	OK
ENSBTAG00000038401	ENSBTAG00000038401	15:34432513-34432631	0	0	0	OK
ENSBTAG00000038409	ENSBTAG00000038409	7:45577376-45579729	0	0	0	OK
ENSBTAG00000038411	ENSBTAG00000038411	8:62722029-62722587	0	0	0	OK
ENSBTAG00000038416	ENSBTAG00000038416	18:63588417-63589305	0	0	0	OK
ENSBTAG00000038421	ENSBTAG00000038421	6:2385667-2396509	0	0	0	OK
ENSBTAG00000038423	ENSBTAG00000038423	21:67579422-67579525	0	0	0	OK
ENSBTAG00000038426	ENSBTAG00000038426	3:11881615-11882486	0	0	0	OK
ENSBTAG00000038430	ENSBTAG00000038430	23:34219297-34219825	0	0	0	OK
ENSBTAG00000038433	ENSBTAG00000038433	1:6609180-6609531	0	0	0	OK
ENSBTAG00000038437	ENSBTAG00000038437	7:12768810-12770589	0	0	0	OK
ENSBTAG00000038438	ENSBTAG00000038438	10:36757275-36758438	0	0	0	OK
ENSBTAG00000038439	ENSBTAG00000038439	5:110122672-110124878	0	0	0	OK
ENSBTAG00000038440	ENSBTAG00000038440	11:106437551-106440173	0	0	0	OK
ENSBTAG00000038463	ENSBTAG00000038463	5:62043142-62043212	0	0	0	OK
ENSBTAG00000038472	ENSBTAG00000038472	23:29186865-29187861	0	0	0	OK
ENSBTAG00000038476	ENSBTAG00000038476	3:20770058-20770469	0	0	1.00758	OK
ENSBTAG00000038483	ENSBTAG00000038483	9:71706284-71707316	0	0	0	OK
ENSBTAG00000038486	ENSBTAG00000038486	8:112092504-112094645	0	0	0	OK
ENSBTAG00000038494	ENSBTAG00000038494	21:20357102-20358125	0	0	0	OK
ENSBTAG00000038504	ENSBTAG00000038504	27:6002870-6003728	0	0	0	OK
ENSBTAG00000038512	ENSBTAG00000038512	1:22474170-22497927	0	0	0.402443	OK
ENSBTAG00000038527	ENSBTAG00000038527	4:18950186-18950489	0	0	0	OK

ENSBTAG00000038532	ENSBTAG00000038532	15:52563233-52567329	0	0	0	OK
ENSBTAG00000038539	ENSBTAG00000038539	15:81299557-81300502	0	0	0	OK
ENSBTAG00000038540	ENSBTAG00000038540	26:25060218-25073503	0	0	0	OK
ENSBTAG00000038544	ENSBTAG00000038544	10:25193629-25194177	0	0	0	OK
ENSBTAG00000038546	ENSBTAG00000038546	22:30220159-30220277	0	0	0	OK
ENSBTAG00000038565	ENSBTAG00000038565	10:23330787-23331395	0	0	0	OK
ENSBTAG00000038593	ENSBTAG00000038593	8:47569779-47569889	0	0	0	OK
ENSBTAG00000038598	ENSBTAG00000038598	15:45573877-45575215	0	0	0	OK
ENSBTAG00000038627	ENSBTAG00000038627	25:39128426-39130640	0	0	0	OK
ENSBTAG00000038636	ENSBTAG00000038636	15:50339075-50340023	0	0	0	OK
ENSBTAG00000038637	ENSBTAG00000038637	15:48763976-48764918	0	0	0	OK
ENSBTAG00000038639	ENSBTAG00000038639	6:92600176-92604818	0	0	0	OK
ENSBTAG00000038641	ENSBTAG00000038641	15:45214159-45215098	0	0	0	OK
ENSBTAG00000038650	ENSBTAG00000038650	15:84526490-84543454	0	0	0	OK
ENSBTAG00000038659	ENSBTAG00000038659	6:64749942-64750866	0	0	0	OK
ENSBTAG00000038667	ENSBTAG00000038667	10:77878594-77879947	0	0	0	OK
ENSBTAG00000038668	ENSBTAG00000038668	4:89042206-89042406	0	0	0	OK
ENSBTAG00000038677	ENSBTAG00000038677	23:52397482-52398414	0	0	0	OK
ENSBTAG00000038679	ENSBTAG00000038679	15:49262900-49263848	0	0	0	OK
ENSBTAG00000038697	ENSBTAG00000038697	1:154682044-154682120	0	0	0	OK
ENSBTAG00000038706	ENSBTAG00000038706	18:24112350-24114381	0	0	0	OK
ENSBTAG00000038711	ENSBTAG00000038711	18:50804597-50807705	0	0	0	OK
ENSBTAG00000038714	ENSBTAG00000038714	7:14644744-14646270	0	0	0	OK
ENSBTAG00000038716	ENSBTAG00000038716	16:51766101-51819356	0	0	0	OK
ENSBTAG00000038718	ENSBTAG00000038718	23:34576554-34581696	0	0	0	OK
ENSBTAG00000038722	ENSBTAG00000038722	7:43482714-43483650	0	0	0	OK
ENSBTAG00000038735	ENSBTAG00000038735	18:56026309-56027284	0	0	0	OK
ENSBTAG00000038739	ENSBTAG00000038739	24:58770278-58790832	0	0	0	OK
ENSBTAG00000038748	ENSBTAG00000038748	15:49022977-49024619	0	0	0	OK
ENSBTAG00000038751	ENSBTAG00000038751	X:124336589-124337612	0	0	0	OK

ENSBTAG00000038757	ENSBTAG00000038757	25:208738-209518	0	0	0	OK
ENSBTAG00000038765	ENSBTAG00000038765	19:38396504-38396586	0	0	0	OK
ENSBTAG00000038777	ENSBTAG00000038777	8:60332038-60332994	0	0	0.985986	OK
ENSBTAG00000038782	ENSBTAG00000038782	23:30061278-30061716	0	0	0	OK
ENSBTAG00000038783	ENSBTAG00000038783	23:32293814-32295598	0	0	0	OK
ENSBTAG00000038813	ENSBTAG00000038813	X:47666073-47666188	0	0	0	OK
ENSBTAG00000038835	ENSBTAG00000038835	11:67246029-67253662	0	0	0	OK
ENSBTAG00000038838	ENSBTAG00000038838	13:58308599-58308740	0	0	0	OK
ENSBTAG00000038842	ENSBTAG00000038842	10:20777430-20779257	0	0	0	OK
ENSBTAG00000038843	ENSBTAG00000038843	5:99988120-99992305	0	0	0	OK
ENSBTAG00000038848	ENSBTAG00000038848	11:73976999-73977079	0	0	0	OK
ENSBTAG00000038858	ENSBTAG00000038858	18:34394578-34414048	0	0	0	OK
ENSBTAG00000038868	ENSBTAG00000038868	10:26984484-26985506	0	0	0	OK
ENSBTAG00000038873	ENSBTAG00000038873	15:44199003-44199141	0	0	0	OK
ENSBTAG00000038876	ENSBTAG00000038876	X:124199123-124201485	0	0	0	OK
ENSBTAG00000038879	ENSBTAG00000038879	26:24230070-24230219	0	0	0	OK
ENSBTAG00000038882	ENSBTAG00000038882	5:116348275-116348340	0	0	0	OK
ENSBTAG00000038891	ENSBTAG00000038891	9:88244952-88245726	0	0	0	OK
ENSBTAG00000038899	ENSBTAG00000038899	15:49527629-49528595	0	0	0	OK
ENSBTAG00000038908	ENSBTAG00000038908	23:30396497-30397436	0	0	0	OK
ENSBTAG00000038913	ENSBTAG00000038913	1:22552001-22552769	0	0	0	OK
ENSBTAG00000038918	ENSBTAG00000038918	29:39594292-39605710	0	0	0	OK
ENSBTAG00000038919	ENSBTAG00000038919	X:132694941-132695098	0	0	0	OK
ENSBTAG00000038933	ENSBTAG00000038933	X:39276179-39286093	0	0	0	OK
ENSBTAG00000038934	ENSBTAG00000038934	4:106521517-106521937	0	0	0	OK
ENSBTAG00000038941	ENSBTAG00000038941	11:93853094-93854133	0	0	0	OK
ENSBTAG00000038943	ENSBTAG00000038943	4:106506511-106507022	0	0	0	OK
ENSBTAG00000038955	ENSBTAG00000038955	23:27411031-27412890	0	0	0	OK
ENSBTAG00000038960	ENSBTAG00000038960	4:106536502-106537046	0	0	0	OK
ENSBTAG00000038963	ENSBTAG00000038963	10:28211978-28212917	0	0	0	OK

ENSBTAG00000038969	ENSBTAG00000038969	15:83347222-83347515	0	0	0	OK
ENSBTAG00000038972	ENSBTAG00000038972	11:103327150-103328557	0	0	0	OK
ENSBTAG00000038979	ENSBTAG00000038979	3:34401024-34405907	0	0	0	OK
ENSBTAG00000038985	ENSBTAG00000038985	20:41814150-41816893	0	0	0	OK
ENSBTAG00000038993	ENSBTAG00000038993	26:24215410-24215535	0	0	0	OK
ENSBTAG00000039011	ENSBTAG00000039011	1:83718072-83721672	0	0	0	OK
ENSBTAG00000039014	ENSBTAG00000039014	18:14775249-14787642	0	0	0	OK
ENSBTAG00000039018	ENSBTAG00000039018	19:24506057-24507032	0	0	0	OK
ENSBTAG00000039023	ENSBTAG00000039023	17:53969694-53970480	0	0	0	OK
ENSBTAG00000039029	ENSBTAG00000039029	15:48181825-48182813	0	0	0	OK
ENSBTAG00000039031	ENSBTAG00000039031	14:25067485-25067823	0	0	0	OK
ENSBTAG00000039038	ENSBTAG00000039038	10:24007142-24008531	0	0	0	OK
ENSBTAG00000039054	ENSBTAG00000039054	15:49486828-49487785	0	0	0	OK
ENSBTAG00000039056	ENSBTAG00000039056	15:49039182-49040991	0	0	0	OK
ENSBTAG00000039059	ENSBTAG00000039059	17:74811512-74812320	0	0	0	OK
ENSBTAG00000039065	ENSBTAG00000039065	12:77945881-77946970	0	0	0	OK
ENSBTAG00000039066	ENSBTAG00000039066	X:73236406-73236682	0	0	0	OK
ENSBTAG00000039067	ENSBTAG00000039067	7:54027465-54029850	0	0	0	OK
ENSBTAG00000039068	ENSBTAG00000039068	14:52949755-52950966	0	0	0	OK
ENSBTAG00000039074	ENSBTAG00000039074	5:115871445-115871556	0	0	0	OK
ENSBTAG00000039090	ENSBTAG00000039090	X:98101930-98103366	0	0	0	OK
ENSBTAG00000039104	ENSBTAG00000039104	18:20502889-20502995	0	0	0	OK
ENSBTAG00000039105	ENSBTAG00000039105	3:16880768-16884904	0	0	0	OK
ENSBTAG00000039111	ENSBTAG00000039111	18:58115781-58117110	0	0	0	OK
ENSBTAG00000039113	ENSBTAG00000039113	11:80933934-80934516	0	0	0	OK
ENSBTAG00000039118	ENSBTAG00000039118	21:67578685-67578767	0	0	0	OK
ENSBTAG00000039119	ENSBTAG00000039119	19:55904212-55905449	0	0	0	OK
ENSBTAG00000039127	ENSBTAG00000039127	8:7028940-7029047	0	0	0	OK
ENSBTAG00000039130	ENSBTAG00000039130	7:14670707-14672994	0	0	0	OK
ENSBTAG00000039133	ENSBTAG00000039133	22:37738075-37748331	0	0	0	OK

ENSBTAG00000039134	ENSBTAG00000039134	15:50702646-50703600	0	0	0	OK
ENSBTAG00000039138	ENSBTAG00000039138	11:105594962-105595605	0	0	0	OK
ENSBTAG00000039145	ENSBTAG00000039145	23:30646720-30647644	0	0	0	OK
ENSBTAG00000039154	ENSBTAG00000039154	28:42584643-42585670	0	0	0	OK
ENSBTAG00000039169	ENSBTAG00000039169	9:31895266-31896325	0	0	0	OK
ENSBTAG00000039170	ENSBTAG00000039170	5:44502010-44507108	0	0	0	OK
ENSBTAG00000039177	ENSBTAG00000039177	4:116214848-116214949	0	0	0	OK
ENSBTAG00000039182	ENSBTAG00000039182	15:81042866-81043799	0	0	0	OK
ENSBTAG00000039185	ENSBTAG00000039185	15:51093564-51094498	0	0	0	OK
ENSBTAG00000039194	ENSBTAG00000039194	22:61118757-61122753	0	0	0	OK
ENSBTAG00000039196	ENSBTAG00000039196	16:4939774-4950977	0	0	0	OK
ENSBTAG00000039199	ENSBTAG00000039199	13:70021530-70021623	0	0	0	OK
ENSBTAG00000039201	ENSBTAG00000039201	11:92215373-92221901	0	0	0	OK
ENSBTAG00000039208	ENSBTAG00000039208	13:57866137-57869829	0	0	0	OK
ENSBTAG00000039212	ENSBTAG00000039212	18:57517496-57524958	0	0	0	OK
ENSBTAG00000039213	ENSBTAG00000039213	6:67068196-67069074	0	0	0	OK
ENSBTAG00000039215	ENSBTAG00000039215	18:62827494-62833717	0	0	0	OK
ENSBTAG00000039219	ENSBTAG00000039219	21:67565298-67565395	0	0	0	OK
ENSBTAG00000039222	ENSBTAG00000039222	6:58117210-58118061	0	0	0	OK
ENSBTAG00000039235	ENSBTAG00000039235	10:53493436-53493587	0	0	0	OK
ENSBTAG00000039237	ENSBTAG00000039237	17:72870338-72870659	0	0	0	OK
ENSBTAG00000039242	ENSBTAG00000039242	17:45155078-45163447	0	0	0	OK
ENSBTAG00000039248	ENSBTAG00000039248	24:49849032-49849533	0	0	0	OK
ENSBTAG00000039255	ENSBTAG00000039255	1:5095765-5096443	0	0	0	OK
ENSBTAG00000039272	ENSBTAG00000039272	15:79715022-79715952	0	0	0	OK
ENSBTAG00000039283	ENSBTAG00000039283	19:54102470-54103533	0	0	0	OK
ENSBTAG00000039285	ENSBTAG00000039285	X:91537428-91538439	0	0	0	OK
ENSBTAG00000039296	ENSBTAG00000039296	24:25568961-25591458	0	0	0	OK
ENSBTAG00000039298	ENSBTAG00000039298	15:50132965-50133925	0	0	0	OK
ENSBTAG00000039299	ENSBTAG00000039299	19:28602301-28602961	0	0	0	OK

ENSBTAG00000039300	ENSBTAG00000039300	15:82985494-82986444	0	0	0	OK
ENSBTAG00000039304	ENSBTAG00000039304	4:88929051-88929255	0	0	0	OK
ENSBTAG00000039306	ENSBTAG00000039306	15:82827817-82828753	0	0	0	OK
ENSBTAG00000039315	ENSBTAG00000039315	10:27062436-27063384	0	0	0	OK
ENSBTAG00000039324	ENSBTAG00000039324	26:13474080-13474854	0	0	0	OK
ENSBTAG00000039326	ENSBTAG00000039326	23:45312386-45348035	0	0	0	OK
ENSBTAG00000039330	ENSBTAG00000039330	15:50252450-50253413	0	0	0	OK
ENSBTAG00000039334	ENSBTAG00000039334	1:121288784-121289057	0	0	0	OK
ENSBTAG00000039346	ENSBTAG00000039346	2:120869239-120871886	0	0	0	OK
ENSBTAG00000039351	ENSBTAG00000039351	X:52187478-52187581	0	0	0	OK
ENSBTAG00000039390	ENSBTAG00000039390	23:29092561-29093488	0	0	0	OK
ENSBTAG00000039401	ENSBTAG00000039401	4:106621080-106621541	0	0	0	OK
ENSBTAG00000039402	ENSBTAG00000039402	10:22318714-22462591	0	0	0	OK
ENSBTAG00000039406	ENSBTAG00000039406	26:5717206-5718151	0	0	0	OK
ENSBTAG00000039412	ENSBTAG00000039412	17:55575431-55575548	0	0	0	OK
ENSBTAG00000039413	ENSBTAG00000039413	18:63016438-63019902	0	0	0	OK
ENSBTAG00000039434	ENSBTAG00000039434	X:18698171-18698995	0	0	0	OK
ENSBTAG00000039437	ENSBTAG00000039437	2:59654283-59655602	0	0	0	OK
ENSBTAG00000039440	ENSBTAG00000039440	19:21117461-21117982	0	0	0	OK
ENSBTAG00000039441	ENSBTAG00000039441	18:43324645-43325359	0	0	0	OK
ENSBTAG00000039449	ENSBTAG00000039449	19:57464898-57465001	0	0	0	OK
ENSBTAG00000039456	ENSBTAG00000039456	9:39398264-39398790	0	0	0	OK
ENSBTAG00000039461	ENSBTAG00000039461	29:37695482-37703529	0	0	0	OK
ENSBTAG00000039463	ENSBTAG00000039463	29:42360324-42372760	0	0	0	OK
ENSBTAG00000039467	ENSBTAG00000039467	15:51347102-51348056	0	0	0	OK
ENSBTAG00000039487	ENSBTAG00000039487	3:17320182-17340116	0	0	0	OK
ENSBTAG00000039492	ENSBTAG00000039492	23:31120659-31121052	0	0	0	OK
ENSBTAG00000039496	ENSBTAG00000039496	5:97921578-97921773	0	0	0	OK
ENSBTAG00000039497	ENSBTAG00000039497	12:57534532-57534614	0	0	0	OK
ENSBTAG00000039499	ENSBTAG00000039499	14:42985264-42985951	0	0	0	OK

ENSBTAG00000039510	ENSBTAG00000039510	15:28563803-28563875	0	0	0	OK
ENSBTAG00000039517	ENSBTAG00000039517	29:27253933-27255336	0	0	0	OK
ENSBTAG00000039525	ENSBTAG00000039525	8:22932013-22932600	0	0	0	OK
ENSBTAG00000039546	ENSBTAG00000039546	10:25388758-25389222	0	0	0	OK
ENSBTAG00000039550	ENSBTAG00000039550	19:43923765-43923956	0	0	0	OK
ENSBTAG00000039555	ENSBTAG00000039555	7:88648456-88650528	0	0	0	OK
ENSBTAG00000039559	ENSBTAG00000039559	4:106576881-106577463	0	0	0	OK
ENSBTAG00000039562	ENSBTAG00000039562	23:29149937-29150906	0	0	0	OK
ENSBTAG00000039563	ENSBTAG00000039563	19:6355435-6378384	0	0	0	OK
ENSBTAG00000039564	ENSBTAG00000039564	24:520729-521668	0	0	0	OK
ENSBTAG00000039565	ENSBTAG00000039565	24:12878018-12878100	0	0	0	OK
ENSBTAG00000039575	ENSBTAG00000039575	1:52383026-52383141	0	0	0	OK
ENSBTAG00000039582	ENSBTAG00000039582	17:18698707-18702613	0	0	0	OK
ENSBTAG00000039588	ENSBTAG00000039588	4:113763390-113768447	0	0	0	OK
ENSBTAG00000039592	ENSBTAG00000039592	15:82970319-82971267	0	0	0	OK
ENSBTAG00000039597	ENSBTAG00000039597	1:4893289-4894998	0	0	0	OK
ENSBTAG00000039607	ENSBTAG00000039607	19:55841227-55844057	0	0	0	OK
ENSBTAG00000039614	ENSBTAG00000039614	4:107720863-107721805	0	0	0	OK
ENSBTAG00000039631	ENSBTAG00000039631	14:50324596-50324748	0	0	0	OK
ENSBTAG00000039645	ENSBTAG00000039645	6:117269864-117271675	0	0	0	OK
ENSBTAG00000039657	ENSBTAG00000039657	23:31579046-31589230	0	0	0	OK
ENSBTAG00000039659	ENSBTAG00000039659	1:72540581-72540720	0	0	0	OK
ENSBTAG00000039674	ENSBTAG00000039674	4:106616279-106616726	0	0	0	OK
ENSBTAG00000039675	ENSBTAG00000039675	8:23007454-23008041	0	0	0	OK
ENSBTAG00000039676	ENSBTAG00000039676	29:28297562-28298747	0	0	0	OK
ENSBTAG00000039677	ENSBTAG00000039677	23:30620726-30621668	0	0	0	OK
ENSBTAG00000039698	ENSBTAG00000039698	21:1050188-1050299	0	0	0	OK
ENSBTAG00000039700	ENSBTAG00000039700	9:21755702-21755853	0	0	0	OK
ENSBTAG00000039704	ENSBTAG00000039704	1:15808718-15808830	0	0	0	OK
ENSBTAG00000039718	ENSBTAG00000039718	14:46817758-46821444	0	0	0	OK

ENSBTAG00000039727	ENSBTAG00000039727	22:10774893-10790070	0	0	0	OK
ENSBTAG00000039745	ENSBTAG00000039745	20:59220508-59220575	0	0	0	OK
ENSBTAG00000039753	ENSBTAG00000039753	X:115078601-115079967	0	0	0	OK
ENSBTAG00000039756	ENSBTAG00000039756	5:59656415-59657366	0	0	0	OK
ENSBTAG00000039764	ENSBTAG00000039764	16:63689679-63690654	0	0	0	OK
ENSBTAG00000039768	ENSBTAG00000039768	29:26717797-26720685	0	0	0	OK
ENSBTAG00000039769	ENSBTAG00000039769	17:51532091-51533990	0	0	0	OK
ENSBTAG00000039787	ENSBTAG00000039787	6:87378397-87392750	0	0	0	OK
ENSBTAG00000039805	ENSBTAG00000039805	7:42688917-42691147	0	0	0	OK
ENSBTAG00000039808	ENSBTAG00000039808	21:59517403-59530028	0	0	0	OK
ENSBTAG00000039810	ENSBTAG00000039810	23:33569430-33570050	0	0	0	OK
ENSBTAG00000039821	ENSBTAG00000039821	15:79359176-79360603	0	0	0	OK
ENSBTAG00000039842	ENSBTAG00000039842	11:99007029-99007234	0	0	0	OK
ENSBTAG00000039849	ENSBTAG00000039849	18:60579686-60586354	0	0	0	OK
ENSBTAG00000039850	ENSBTAG00000039850	24:33315173-33315817	0	0	0	OK
ENSBTAG00000039856	ENSBTAG00000039856	27:5221876-5227956	0	0	0	OK
ENSBTAG00000039858	ENSBTAG00000039858	26:29887785-29887867	0	0	0	OK
ENSBTAG00000039875	ENSBTAG00000039875	9:88307675-88310265	0	0	0	OK
ENSBTAG00000039877	ENSBTAG00000039877	7:10173390-10174827	0	0	0	OK
ENSBTAG00000039890	ENSBTAG00000039890	X:18728864-18729206	0	0	0	OK
ENSBTAG00000039892	ENSBTAG00000039892	18:63179642-63192125	0	0	0	OK
ENSBTAG00000039906	ENSBTAG00000039906	5:84672041-84672190	0	0	0	OK
ENSBTAG00000039912	ENSBTAG00000039912	11:54416614-54416778	0	0	0	OK
ENSBTAG00000039914	ENSBTAG00000039914	18:2607954-2611931	0	0	0	OK
ENSBTAG00000039917	ENSBTAG00000039917	15:82874704-82875640	0	0	0	OK
ENSBTAG00000039928	ENSBTAG00000039928	4:114032642-114038805	0	0	0	OK
ENSBTAG00000039929	ENSBTAG00000039929	20:39875725-39877132	0	0	0	OK
ENSBTAG00000039930	ENSBTAG00000039930	7:42296506-42297460	0	0	0	OK
ENSBTAG00000039932	ENSBTAG00000039932	18:63186252-63188384	0	0	0	OK
ENSBTAG00000039946	ENSBTAG00000039946	18:65553156-65584171	0	0	0	OK

ENSBTAG00000039950	ENSBTAG00000039950	10:82574441-82575288	0	0	0	OK
ENSBTAG00000039956	ENSBTAG00000039956	4:106474215-106474729	0	0	0	OK
ENSBTAG00000039962	ENSBTAG00000039962	25:26851887-26855546	0	0	0	OK
ENSBTAG00000039963	ENSBTAG00000039963	8:23053976-23054543	0	0	0	OK
ENSBTAG00000039969	ENSBTAG00000039969	18:60352472-60353630	0	0	0	OK
ENSBTAG00000039974	ENSBTAG00000039974	25:857166-858273	0	0	0	OK
ENSBTAG00000039989	ENSBTAG00000039989	25:20382746-20382890	0	0	0	OK
ENSBTAG00000040006	ENSBTAG00000040006	23:10481197-10487651	0	0	0	OK
ENSBTAG00000040013	ENSBTAG00000040013	21:67579061-67579154	0	0	0	OK
ENSBTAG00000040014	ENSBTAG00000040014	25:39070439-39075699	0	0	0	OK
ENSBTAG00000040018	ENSBTAG00000040018	28:18349472-18349742	0	0	0	OK
ENSBTAG00000040026	ENSBTAG00000040026	23:29660888-29662505	0	0	0	OK
ENSBTAG00000040033	ENSBTAG00000040033	7:43044538-43045551	0	0	0	OK
ENSBTAG00000040054	ENSBTAG00000040054	18:55235066-55235856	0	0	0	OK
ENSBTAG00000040060	ENSBTAG00000040060	3:102712153-102713309	0	0	0	OK
ENSBTAG00000040069	ENSBTAG00000040069	21:19984961-19985051	0	0	0	OK
ENSBTAG00000040070	ENSBTAG00000040070	29:41378326-41381132	0	0	0	OK
ENSBTAG00000040105	ENSBTAG00000040105	2:24001746-24001848	0	0	0	OK
ENSBTAG00000040109	ENSBTAG00000040109	7:42579837-42580806	0	0	0	OK
ENSBTAG00000040116	ENSBTAG00000040116	22:59841217-59841862	0	0	0	OK
ENSBTAG00000040134	ENSBTAG00000040134	21:35184045-35187216	0	0	0	OK
ENSBTAG00000040139	ENSBTAG00000040139	15:49890844-49891791	0	0	0	OK
ENSBTAG00000040140	ENSBTAG00000040140	29:38838946-38844641	0	0	0	OK
ENSBTAG00000040144	ENSBTAG00000040144	1:58015563-58028842	0	0	0	OK
ENSBTAG00000040151	ENSBTAG00000040151	10:67576389-67631089	0	0	0	OK
ENSBTAG00000040153	ENSBTAG00000040153	7:17164340-17169606	0	0	0	OK
ENSBTAG00000040155	ENSBTAG00000040155	1:8328038-8328350	0	0	0	OK
ENSBTAG00000040156	ENSBTAG00000040156	2:112985258-112985402	0	0	0	OK
ENSBTAG00000040161	ENSBTAG00000040161	1:71521907-71529868	0	0	0	OK
ENSBTAG00000040167	ENSBTAG00000040167	10:92889960-92890752	0	0	0	OK

ENSBTAG00000040177	ENSBTAG00000040177	18:57439358-57445450	0	0	0	OK
ENSBTAG00000040185	ENSBTAG00000040185	15:49084882-49085845	0	0	0	OK
ENSBTAG00000040197	ENSBTAG00000040197	15:82801351-82802648	0	0	0	OK
ENSBTAG00000040202	ENSBTAG00000040202	29:17496571-17512594	0	0	0	OK
ENSBTAG00000040210	ENSBTAG00000040210	2:125629833-125630345	0	0	0	OK
ENSBTAG00000040222	ENSBTAG00000040222	26:97401-106867	0	0	0	OK
ENSBTAG00000040229	ENSBTAG00000040229	19:42101946-42102951	0	0	0	OK
ENSBTAG00000040232	ENSBTAG00000040232	13:25333572-25333710	0	0	0	OK
ENSBTAG00000040239	ENSBTAG00000040239	29:27523130-27524063	0	0	0	OK
ENSBTAG00000040245	ENSBTAG00000040245	1:83546861-83546947	0	0	0	OK
ENSBTAG00000040253	ENSBTAG00000040253	7:73611116-73672127	0	0	0	OK
ENSBTAG00000040254	ENSBTAG00000040254	21:67570007-67570112	0	0	0	OK
ENSBTAG00000040261	ENSBTAG00000040261	4:106549713-106550191	0	0	0	OK
ENSBTAG00000040263	ENSBTAG00000040263	26:25038734-25038826	0	0	0	OK
ENSBTAG00000040277	ENSBTAG00000040277	23:31534730-31535522	0	0	0	OK
ENSBTAG00000040281	ENSBTAG00000040281	4:106449618-106450270	0	0	0	OK
ENSBTAG00000040282	ENSBTAG00000040282	19:7613375-7614074	0	0	0	OK
ENSBTAG00000040302	ENSBTAG00000040302	5:112802156-112802210	0	0	0	OK
ENSBTAG00000040319	ENSBTAG00000040319	25:27811920-27812877	0	0	0	OK
ENSBTAG00000040320	ENSBTAG00000040320	15:81949076-81956356	0	0	0	OK
ENSBTAG00000040321	ENSBTAG00000040321	14:25153582-25179651	0	0	0	OK
ENSBTAG00000040324	ENSBTAG00000040324	6:32045-33242	0	0	0	OK
ENSBTAG00000040336	ENSBTAG00000040336	17:45191288-45218103	0	0	0	OK
ENSBTAG00000040344	ENSBTAG00000040344	5:52821140-52821521	0	0	0	OK
ENSBTAG00000040347	ENSBTAG00000040347	12:68232525-68740385	0	0	0	OK
ENSBTAG00000040348	ENSBTAG00000040348	X:22975002-22975128	0	0	0	OK
ENSBTAG00000040354	ENSBTAG00000040354	9:71504220-71512705	0	0	0	OK
ENSBTAG00000040363	ENSBTAG00000040363	X:58503871-58505529	0	0	0	OK
ENSBTAG00000040367	ENSBTAG00000040367	2:92993921-92995706	0	0	0	OK
ENSBTAG00000040370	ENSBTAG00000040370	17:25054141-25057633	0	0	0	OK

ENSBTAG00000040374	ENSBTAG00000040374	4:105899672-105900550	0	0	0	OK
ENSBTAG00000040382	ENSBTAG00000040382	3:120067370-120068342	0	0	0	OK
ENSBTAG00000040387	ENSBTAG00000040387	4:106545617-106546074	0	0	0	OK
ENSBTAG00000040388	ENSBTAG00000040388	4:106527297-106530985	0	0	0	OK
ENSBTAG00000040393	ENSBTAG00000040393	1:62162948-62171172	0	0	0	OK
ENSBTAG00000040406	ENSBTAG00000040406	X:113801667-113803821	0	0	0	OK
ENSBTAG00000040409	ENSBTAG00000040409	16:5847771-5876373	0	0	0	OK
ENSBTAG00000040413	ENSBTAG00000040413	18:34775140-34787908	0	0	0	OK
ENSBTAG00000040414	ENSBTAG00000040414	3:111030117-111030977	0	0	0	OK
ENSBTAG00000040416	ENSBTAG00000040416	22:57711376-57711516	0	0	0	OK
ENSBTAG00000040418	ENSBTAG00000040418	1:19864722-19865715	0	0	0	OK
ENSBTAG00000040421	ENSBTAG00000040421	4:49410315-49410416	0	0	0	OK
ENSBTAG00000040425	ENSBTAG00000040425	15:79697097-79698027	0	0	0	OK
ENSBTAG00000040449	ENSBTAG00000040449	15:16261702-16296156	0	0	0	OK
ENSBTAG00000040459	ENSBTAG00000040459	10:28122622-28123573	0	0	0	OK
ENSBTAG00000040461	ENSBTAG00000040461	14:79862684-79877931	0	0	0	OK
ENSBTAG00000040462	ENSBTAG00000040462	11:90263731-90263859	0	0	0	OK
ENSBTAG00000040480	ENSBTAG00000040480	15:47891404-47892364	0	0	0	OK
ENSBTAG00000040494	ENSBTAG00000040494	2:23299146-23299368	0	0	0	OK
ENSBTAG00000040497	ENSBTAG00000040497	2:127299733-127310992	0	0	0	OK
ENSBTAG00000040504	ENSBTAG00000040504	10:25034410-25034965	0	0	0	OK
ENSBTAG00000040518	ENSBTAG00000040518	2:122343233-122344583	0	0	0	OK
ENSBTAG00000040526	ENSBTAG00000040526	15:49752407-49753371	0	0	0	OK
ENSBTAG00000040533	ENSBTAG00000040533	9:71769999-71771031	0	0	0	OK
ENSBTAG00000040550	ENSBTAG00000040550	18:20318438-20318891	0	0	0	OK
ENSBTAG00000040564	ENSBTAG00000040564	10:72298984-72299176	0	0	0	OK
ENSBTAG00000040572	ENSBTAG00000040572	20:44339042-44339422	0	0	0	OK
ENSBTAG00000040590	ENSBTAG00000040590	14:65660647-65660904	0	0	0	OK
ENSBTAG00000040595	ENSBTAG00000040595	8:103183422-103183550	0	0	0	OK
ENSBTAG00000040597	ENSBTAG00000040597	1:65265656-65292980	0	0	0	OK

ENSBTAG00000040598	ENSBTAG00000040598	3:121305729-121309419	0	0	0	OK
ENSBTAG00000040601	ENSBTAG00000040601	28:29983512-29983606	0	0	0	OK
ENSBTAG00000040604	ENSBTAG00000040604	26:46201327-46201444	0	0	0	OK
ENSBTAG00000040609	ENSBTAG00000040609	10:28255474-28256440	0	0	0	OK
ENSBTAG00000042079	ENSBTAG00000042079	22:5332040-5332147	0	0	0	OK
ENSBTAG00000042080	ENSBTAG00000042080	8:6126619-6126724	0	0	0	OK
ENSBTAG00000042081	ENSBTAG00000042081	10:69424952-69425065	0	0	0	OK
ENSBTAG00000042082	ENSBTAG00000042082	14:15986907-15987007	0	0	0	OK
ENSBTAG00000042083	ENSBTAG00000042083	6:100341140-100341274	0	0	0	OK
ENSBTAG00000042084	ENSBTAG00000042084	20:36615738-36615845	0	0	0	OK
ENSBTAG00000042085	ENSBTAG00000042085	11:94384740-94384847	0	0	0	OK
ENSBTAG00000042086	ENSBTAG00000042086	13:62848411-62848518	0	0	0	OK
ENSBTAG00000042087	ENSBTAG00000042087	3:57008649-57008799	0	0	0	OK
ENSBTAG00000042089	ENSBTAG00000042089	12:51428083-51428190	0	0	0	OK
ENSBTAG00000042090	ENSBTAG00000042090	20:62335947-62336051	0	0	0	OK
ENSBTAG00000042091	ENSBTAG00000042091	3:32589300-32589404	0	0	0	OK
ENSBTAG00000042092	ENSBTAG00000042092	24:46370313-46370417	0	0	0	OK
ENSBTAG00000042094	ENSBTAG00000042094	1:83475434-83475510	0	0	0	OK
ENSBTAG00000042095	ENSBTAG00000042095	11:27470074-27470181	0	0	0	OK
ENSBTAG00000042096	ENSBTAG00000042096	18:22553057-22553182	0	0	0	OK
ENSBTAG00000042097	ENSBTAG00000042097	21:43033763-43033870	0	0	0	OK
ENSBTAG00000042098	ENSBTAG00000042098	19:33814288-33814358	0	0	0	OK
ENSBTAG00000042099	ENSBTAG00000042099	13:78103077-78103169	0	0	0	OK
ENSBTAG00000042100	ENSBTAG00000042100	12:32854928-32854999	0	0	0	OK
ENSBTAG00000042101	ENSBTAG00000042101	13:76217355-76217459	0	0	0	OK
ENSBTAG00000042102	ENSBTAG00000042102	18:15834274-15834381	0	0	0	OK
ENSBTAG00000042103	ENSBTAG00000042103	20:56188529-56188636	0	0	0	OK
ENSBTAG00000042105	ENSBTAG00000042105	19:18763077-18763212	0	0	0	OK
ENSBTAG00000042106	ENSBTAG00000042106	11:45964038-45964164	0	0	0	OK
ENSBTAG00000042107	ENSBTAG00000042107	5:99553714-99554036	0	0	0	OK

ENSBTAG00000042110	ENSBTAG00000042110	27:9388657-9388780	0	0	0	OK
ENSBTAG00000042111	ENSBTAG00000042111	15:64650587-64650690	0	0	0	OK
ENSBTAG00000042113	ENSBTAG00000042113	27:30358354-30358491	0	0	0	OK
ENSBTAG00000042114	ENSBTAG00000042114	X:21977895-21978030	0	0	0	OK
ENSBTAG00000042115	ENSBTAG00000042115	21:2138851-2138942	0	0	0	OK
ENSBTAG00000042117	ENSBTAG00000042117	22:48728806-48728883	0	0	0	OK
ENSBTAG00000042118	ENSBTAG00000042118	11:79514890-79515190	0	0	0	OK
ENSBTAG00000042119	ENSBTAG00000042119	27:37086800-37086904	0	0	0	OK
ENSBTAG00000042125	ENSBTAG00000042125	21:2228538-2228588	0	0	0	OK
ENSBTAG00000042126	ENSBTAG00000042126	16:37582335-37582467	0	0	0	OK
ENSBTAG00000042127	ENSBTAG00000042127	28:24281577-24281720	0	0	0	OK
ENSBTAG00000042129	ENSBTAG00000042129	2:128245273-128245380	0	0	0	OK
ENSBTAG00000042130	ENSBTAG00000042130	28:25130714-25130821	0	0	0	OK
ENSBTAG00000042131	ENSBTAG00000042131	13:73738217-73738289	0	0	0	OK
ENSBTAG00000042132	ENSBTAG00000042132	6:33818551-33818658	0	0	0	OK
ENSBTAG00000042133	ENSBTAG00000042133	11:2573069-2573176	0	0	0	OK
ENSBTAG00000042134	ENSBTAG00000042134	15:34217727-34217813	0	0	0	OK
ENSBTAG00000042135	ENSBTAG00000042135	11:29645317-29645452	0	0	0	OK
ENSBTAG00000042136	ENSBTAG00000042136	X:87243996-87244103	0	0	0	OK
ENSBTAG00000042137	ENSBTAG00000042137	6:17689713-17689819	0	0	0	OK
ENSBTAG00000042139	ENSBTAG00000042139	28:44101814-44101921	0	0	0	OK
ENSBTAG00000042140	ENSBTAG00000042140	21:1410449-1410555	0	0	0	OK
ENSBTAG00000042141	ENSBTAG00000042141	14:44677204-44677336	0	0	0	OK
ENSBTAG00000042142	ENSBTAG00000042142	2:123179898-123179981	0	0	0	OK
ENSBTAG00000042143	ENSBTAG00000042143	7:15891231-15891318	0	0	0	OK
ENSBTAG00000042148	ENSBTAG00000042148	9:13640815-13640920	0	0	0	OK
ENSBTAG00000042149	ENSBTAG00000042149	27:24398381-24398488	0	0	0	OK
ENSBTAG00000042150	ENSBTAG00000042150	23:34394246-34394355	0	0	0	OK
ENSBTAG00000042152	ENSBTAG00000042152	17:19053384-19053494	0	0	0	OK
ENSBTAG00000042153	ENSBTAG00000042153	14:31505037-31505144	0	0	0	OK

ENSBTAG00000042154	ENSBTAG00000042154	17:75037474-75037596	0	0	0	OK
ENSBTAG00000042156	ENSBTAG00000042156	6:27336718-27336829	0	0	0	OK
ENSBTAG00000042157	ENSBTAG00000042157	X:137187089-137187197	0	0	0	OK
ENSBTAG00000042158	ENSBTAG00000042158	14:716783-716890	0	0	0	OK
ENSBTAG00000042159	ENSBTAG00000042159	19:36353253-36353340	0	0	0	OK
ENSBTAG00000042160	ENSBTAG00000042160	22:50815489-50815595	0	0	0	OK
ENSBTAG00000042163	ENSBTAG00000042163	X:97223226-97223292	0	0	0	OK
ENSBTAG00000042164	ENSBTAG00000042164	10:12768966-12769073	0	0	0	OK
ENSBTAG00000042166	ENSBTAG00000042166	5:31282387-31282519	0	0	0	OK
ENSBTAG00000042168	ENSBTAG00000042168	24:48965076-48965183	0	0	0	OK
ENSBTAG00000042169	ENSBTAG00000042169	5:56977942-56978022	0	0	0	OK
ENSBTAG00000042171	ENSBTAG00000042171	14:66777377-66777512	0	0	0	OK
ENSBTAG00000042173	ENSBTAG00000042173	15:54044310-54044418	0	0	0	OK
ENSBTAG00000042175	ENSBTAG00000042175	21:46078057-46078164	0	0	0	OK
ENSBTAG00000042176	ENSBTAG00000042176	13:66811068-66811199	0	0	0	OK
ENSBTAG00000042177	ENSBTAG00000042177	2:63676664-63676771	0	0	0	OK
ENSBTAG00000042178	ENSBTAG00000042178	5:108045290-108045397	0	0	0	OK
ENSBTAG00000042179	ENSBTAG00000042179	11:5770162-5770266	0	0	0	OK
ENSBTAG00000042180	ENSBTAG00000042180	16:56691482-56691559	0	0	0	OK
ENSBTAG00000042181	ENSBTAG00000042181	2:91210140-91210227	0	0	0	OK
ENSBTAG00000042182	ENSBTAG00000042182	18:47321735-47321842	0	0	0	OK
ENSBTAG00000042183	ENSBTAG00000042183	4:59551832-59551962	0	0	0	OK
ENSBTAG00000042184	ENSBTAG00000042184	15:43939303-43939407	0	0	0	OK
ENSBTAG00000042186	ENSBTAG00000042186	26:22676068-22676182	0	0	0	OK
ENSBTAG00000042188	ENSBTAG00000042188	10:37139530-37139637	0	0	0	OK
ENSBTAG00000042189	ENSBTAG00000042189	21:29064984-29065066	0	0	0	OK
ENSBTAG00000042190	ENSBTAG00000042190	10:12189892-12190008	0	0	0	OK
ENSBTAG00000042191	ENSBTAG00000042191	13:81838598-81838678	0	0	0	OK
ENSBTAG00000042192	ENSBTAG00000042192	10:77679288-77679395	0	0	0	OK
ENSBTAG00000042194	ENSBTAG00000042194	24:21154679-21154786	0	0	0	OK

ENSBTAG00000042195	ENSBTAG00000042195	5:77638002-77638109	0	0	0	OK
ENSBTAG00000042196	ENSBTAG00000042196	2:12832528-12832662	0	0	0	OK
ENSBTAG00000042198	ENSBTAG00000042198	13:52985031-52985103	0	0	0	OK
ENSBTAG00000042199	ENSBTAG00000042199	21:67503571-67503646	0	0	0	OK
ENSBTAG00000042200	ENSBTAG00000042200	21:46402384-46402491	0	0	0	OK
ENSBTAG00000042201	ENSBTAG00000042201	19:21104193-21104325	0	0	0	OK
ENSBTAG00000042203	ENSBTAG00000042203	28:9410032-9410139	0	0	0	OK
ENSBTAG00000042204	ENSBTAG00000042204	X:111895692-111895799	0	0	0	OK
ENSBTAG00000042206	ENSBTAG00000042206	24:49621161-49621227	0	0	0	OK
ENSBTAG00000042209	ENSBTAG00000042209	9:46359121-46359228	0	0	0	OK
ENSBTAG00000042211	ENSBTAG00000042211	8:71337382-71337518	0	0	0	OK
ENSBTAG00000042213	ENSBTAG00000042213	6:17683279-17683414	0	0	0	OK
ENSBTAG00000042215	ENSBTAG00000042215	17:64790996-64791128	0	0	0	OK
ENSBTAG00000042216	ENSBTAG00000042216	23:39322725-39322832	0	0	0	OK
ENSBTAG00000042217	ENSBTAG00000042217	16:56693444-56693513	0	0	0	OK
ENSBTAG00000042218	ENSBTAG00000042218	3:19286826-19286957	0	0	0	OK
ENSBTAG00000042219	ENSBTAG00000042219	11:75551695-75551802	0	0	0	OK
ENSBTAG00000042220	ENSBTAG00000042220	24:54290444-54290551	0	0	0	OK
ENSBTAG00000042224	ENSBTAG00000042224	26:39599914-39600044	0	0	0	OK
ENSBTAG00000042225	ENSBTAG00000042225	29:9105519-9105589	0	0	0	OK
ENSBTAG00000042226	ENSBTAG00000042226	22:12730754-12730905	0	0	0	OK
ENSBTAG00000042227	ENSBTAG00000042227	19:6298937-6299022	0	0	0	OK
ENSBTAG00000042228	ENSBTAG00000042228	24:37069078-37069148	0	0	0	OK
ENSBTAG00000042230	ENSBTAG00000042230	12:33978759-33978865	0	0	0	OK
ENSBTAG00000042231	ENSBTAG00000042231	18:38814161-38814269	0	0	0	OK
ENSBTAG00000042232	ENSBTAG00000042232	13:26087681-26087788	0	0	0	OK
ENSBTAG00000042233	ENSBTAG00000042233	10:13321819-13321891	0	0	0	OK
ENSBTAG00000042234	ENSBTAG00000042234	13:53463240-53463375	0	0	0	OK
ENSBTAG00000042235	ENSBTAG00000042235	29:41853448-41853518	0	0	0	OK
ENSBTAG00000042236	ENSBTAG00000042236	15:29140969-29141096	0	0	0	OK

ENSBTAG00000042237	ENSBTAG00000042237	5:44133032-44133160	0	0	0 OK
ENSBTAG00000042238	ENSBTAG00000042238	28:7576626-7576749	0	0	0 OK
ENSBTAG00000042240	ENSBTAG00000042240	17:73984080-73984184	0	0	0 OK
ENSBTAG00000042241	ENSBTAG00000042241	8:85079591-85079684	0	0	0 OK
ENSBTAG00000042242	ENSBTAG00000042242	X:136960916-136961023	0	0	0 OK
ENSBTAG00000042244	ENSBTAG00000042244	28:11791961-11792064	0	0	0 OK
ENSBTAG00000042245	ENSBTAG00000042245	16:45429774-45429878	0	0	0 OK
ENSBTAG00000042246	ENSBTAG00000042246	20:30654589-30654696	0	0	0 OK
ENSBTAG00000042250	ENSBTAG00000042250	14:31836773-31836905	0	0	0 OK
ENSBTAG00000042254	ENSBTAG00000042254	18:33999186-33999293	0	0	0 OK
ENSBTAG00000042255	ENSBTAG00000042255	5:109463612-109463719	0	0	0 OK
ENSBTAG00000042256	ENSBTAG00000042256	7:52209219-52209419	0	0	0 OK
ENSBTAG00000042257	ENSBTAG00000042257	23:16876167-16876274	0	0	0 OK
ENSBTAG00000042258	ENSBTAG00000042258	28:25643316-25643386	0	0	0 OK
ENSBTAG00000042259	ENSBTAG00000042259	8:51238434-51238514	0	0	0 OK
ENSBTAG00000042260	ENSBTAG00000042260	9:61263525-61263590	0	0	0 OK
ENSBTAG00000042261	ENSBTAG00000042261	1:10273736-10273840	0	0	0 OK
ENSBTAG00000042263	ENSBTAG00000042263	7:42021610-42021717	0	0	0 OK
ENSBTAG00000042264	ENSBTAG00000042264	28:25643112-25643192	0	0	0 OK
ENSBTAG00000042265	ENSBTAG00000042265	2:69856394-69856501	0	0	0 OK
ENSBTAG00000042266	ENSBTAG00000042266	28:11597514-11597617	0	0	0 OK
ENSBTAG00000042268	ENSBTAG00000042268	19:21491301-21491372	0	0	0 OK
ENSBTAG00000042269	ENSBTAG00000042269	4:119404148-119404274	0	0	0 OK
ENSBTAG00000042271	ENSBTAG00000042271	5:76325754-76325863	0	0	0 OK
ENSBTAG00000042272	ENSBTAG00000042272	10:93638608-93638752	0	0	0 OK
ENSBTAG00000042273	ENSBTAG00000042273	29:41693827-41693976	0	0	0 OK
ENSBTAG00000042274	ENSBTAG00000042274	20:27987576-27987683	0	0	0 OK
ENSBTAG00000042275	ENSBTAG00000042275	9:71975432-71975505	0	0	0 OK
ENSBTAG00000042276	ENSBTAG00000042276	25:27095801-27095932	0	0	0 OK
ENSBTAG00000042278	ENSBTAG00000042278	2:43578068-43578172	0	0	0 OK

ENSBTAG00000042279	ENSBTAG00000042279	2:18021756-18021860	0	0	0	OK
ENSBTAG00000042280	ENSBTAG00000042280	29:1062857-1062984	0	0	0	OK
ENSBTAG00000042281	ENSBTAG00000042281	28:25099334-25099468	0	0	0	OK
ENSBTAG00000042282	ENSBTAG00000042282	8:66517406-66517687	0	0	0	OK
ENSBTAG00000042283	ENSBTAG00000042283	7:19774955-19775035	0	0	0	OK
ENSBTAG00000042284	ENSBTAG00000042284	29:49184918-49185025	0	0	0	OK
ENSBTAG00000042285	ENSBTAG00000042285	11:7804806-7804909	0	0	0	OK
ENSBTAG00000042287	ENSBTAG00000042287	29:41499349-41499475	0	0	0	OK
ENSBTAG00000042289	ENSBTAG00000042289	5:45288077-45288209	0	0	0	OK
ENSBTAG00000042290	ENSBTAG00000042290	11:46263275-46263379	0	0	0	OK
ENSBTAG00000042291	ENSBTAG00000042291	6:103939421-103939525	0	0	0	OK
ENSBTAG00000042292	ENSBTAG00000042292	3:101817653-101817726	0	0	0	OK
ENSBTAG00000042293	ENSBTAG00000042293	14:68470545-68470677	0	0	0	OK
ENSBTAG00000042294	ENSBTAG00000042294	3:66860320-66860424	0	0	0	OK
ENSBTAG00000042295	ENSBTAG00000042295	11:506783-506888	0	0	0	OK
ENSBTAG00000042297	ENSBTAG00000042297	20:33669524-33669606	0	0	0	OK
ENSBTAG00000042298	ENSBTAG00000042298	5:26719614-26719721	0	0	0	OK
ENSBTAG00000042299	ENSBTAG00000042299	22:4436746-4436852	0	0	0	OK
ENSBTAG00000042300	ENSBTAG00000042300	10:6391324-6391427	0	0	0	OK
ENSBTAG00000042301	ENSBTAG00000042301	15:17349532-17349658	0	0	0	OK
ENSBTAG00000042303	ENSBTAG00000042303	X:118280889-118281023	0	0	0	OK
ENSBTAG00000042304	ENSBTAG00000042304	25:26778319-26778426	0	0	0	OK
ENSBTAG00000042305	ENSBTAG00000042305	19:15087727-15087824	0	0	0	OK
ENSBTAG00000042307	ENSBTAG00000042307	8:10139600-10139707	0	0	0	OK
ENSBTAG00000042308	ENSBTAG00000042308	28:32325621-32325728	0	0	0	OK
ENSBTAG00000042309	ENSBTAG00000042309	15:49723261-49723368	0	0	0	OK
ENSBTAG00000042310	ENSBTAG00000042310	5:26551239-26551383	0	0	0	OK
ENSBTAG00000042311	ENSBTAG00000042311	10:66636439-66636546	0	0	0	OK
ENSBTAG00000042312	ENSBTAG00000042312	15:54511860-54511967	0	0	0	OK
ENSBTAG00000042313	ENSBTAG00000042313	18:35719531-35719639	0	0	0	OK

ENSBTAG00000042314	ENSBTAG00000042314	22:12113129-12113236	0	0	0	OK
ENSBTAG00000042315	ENSBTAG00000042315	2:30748243-30748350	0	0	0	OK
ENSBTAG00000042316	ENSBTAG00000042316	21:1600478-1600585	0	0	0	OK
ENSBTAG00000042317	ENSBTAG00000042317	23:34538738-34538846	0	0	0	OK
ENSBTAG00000042319	ENSBTAG00000042319	15:54889031-54889168	0	0	0	OK
ENSBTAG00000042320	ENSBTAG00000042320	13:81838842-81838914	0	0	0	OK
ENSBTAG00000042321	ENSBTAG00000042321	7:68270170-68270304	0	0	0	OK
ENSBTAG00000042322	ENSBTAG00000042322	13:68006941-68007075	0	0	0	OK
ENSBTAG00000042323	ENSBTAG00000042323	5:14842739-14842846	0	0	0	OK
ENSBTAG00000042325	ENSBTAG00000042325	8:40259495-40259603	0	0	0	OK
ENSBTAG00000042326	ENSBTAG00000042326	5:58353381-58353485	0	0	0	OK
ENSBTAG00000042327	ENSBTAG00000042327	4:26098354-26098459	0	0	0	OK
ENSBTAG00000042329	ENSBTAG00000042329	11:94037105-94037216	0	0	0	OK
ENSBTAG00000042330	ENSBTAG00000042330	20:21886625-21886712	0	0	0	OK
ENSBTAG00000042331	ENSBTAG00000042331	1:8652129-8652252	0	0	0	OK
ENSBTAG00000042332	ENSBTAG00000042332	14:5309455-5309562	0	0	0	OK
ENSBTAG00000042333	ENSBTAG00000042333	5:82807897-82808001	0	0	0	OK
ENSBTAG00000042334	ENSBTAG00000042334	13:69811969-69812076	0	0	0	OK
ENSBTAG00000042335	ENSBTAG00000042335	15:44469599-44469728	0	0	0	OK
ENSBTAG00000042339	ENSBTAG00000042339	16:56692229-56692310	0	0	0	OK
ENSBTAG00000042343	ENSBTAG00000042343	6:105387091-105387193	0	0	0	OK
ENSBTAG00000042344	ENSBTAG00000042344	23:34613902-34614010	0	0	0	OK
ENSBTAG00000042345	ENSBTAG00000042345	17:64051484-64051591	0	0	0	OK
ENSBTAG00000042346	ENSBTAG00000042346	21:67499217-67499291	0	0	0	OK
ENSBTAG00000042347	ENSBTAG00000042347	14:62123070-62123196	0	0	0	OK
ENSBTAG00000042349	ENSBTAG00000042349	7:9160849-9160956	0	0	0	OK
ENSBTAG00000042350	ENSBTAG00000042350	11:101639012-101639098	0	0	0	OK
ENSBTAG00000042351	ENSBTAG00000042351	19:17768043-17768150	0	0	0	OK
ENSBTAG00000042353	ENSBTAG00000042353	3:69321762-69321834	0	0	0	OK
ENSBTAG00000042354	ENSBTAG00000042354	15:44470840-44470970	0	0	0	OK

ENSBTAG00000042355	ENSBTAG00000042355	14:52373125-52373232	0	0	0	OK
ENSBTAG00000042357	ENSBTAG00000042357	3:50790726-50790822	0	0	0	OK
ENSBTAG00000042358	ENSBTAG00000042358	3:100085088-100085168	0	0	0	OK
ENSBTAG00000042359	ENSBTAG00000042359	26:45708789-45708896	0	0	0	OK
ENSBTAG00000042360	ENSBTAG00000042360	1:155691882-155691989	0	0	0	OK
ENSBTAG00000042361	ENSBTAG00000042361	10:34152724-34152831	0	0	0	OK
ENSBTAG00000042362	ENSBTAG00000042362	1:121125762-121125895	0	0	0	OK
ENSBTAG00000042363	ENSBTAG00000042363	16:31624093-31624220	0	0	0	OK
ENSBTAG00000042364	ENSBTAG00000042364	X:80218516-80218623	0	0	0	OK
ENSBTAG00000042365	ENSBTAG00000042365	21:67532231-67532303	0	0	0	OK
ENSBTAG00000042366	ENSBTAG00000042366	25:27988512-27988646	0	0	0	OK
ENSBTAG00000042368	ENSBTAG00000042368	24:49618581-49618645	0	0	0	OK
ENSBTAG00000042369	ENSBTAG00000042369	3:91994927-91995058	0	0	0	OK
ENSBTAG00000042370	ENSBTAG00000042370	X:39039616-39039747	0	0	0	OK
ENSBTAG00000042371	ENSBTAG00000042371	26:43214773-43214880	0	0	0	OK
ENSBTAG00000042373	ENSBTAG00000042373	X:123251703-123251810	0	0	0	OK
ENSBTAG00000042375	ENSBTAG00000042375	14:55732562-55732665	0	0	0	OK
ENSBTAG00000042376	ENSBTAG00000042376	20:31587916-31588045	0	0	0	OK
ENSBTAG00000042377	ENSBTAG00000042377	18:56396889-56396974	0	0	0	OK
ENSBTAG00000042378	ENSBTAG00000042378	15:29686489-29686623	0	0	0	OK
ENSBTAG00000042380	ENSBTAG00000042380	3:92725889-92726019	0	0	0	OK
ENSBTAG00000042381	ENSBTAG00000042381	11:104314158-104314231	0	0	0	OK
ENSBTAG00000042382	ENSBTAG00000042382	28:41535976-41536083	0	0	0	OK
ENSBTAG00000042383	ENSBTAG00000042383	7:21255394-21255459	0	0	0	OK
ENSBTAG00000042384	ENSBTAG00000042384	17:6425801-6425905	0	0	0	OK
ENSBTAG00000042385	ENSBTAG00000042385	17:44371844-44371948	0	0	0	OK
ENSBTAG00000042387	ENSBTAG00000042387	19:23881169-23881263	0	0	0	OK
ENSBTAG00000042388	ENSBTAG00000042388	26:9384929-9385047	0	0	0	OK
ENSBTAG00000042389	ENSBTAG00000042389	10:71922534-71922639	0	0	0	OK
ENSBTAG00000042390	ENSBTAG00000042390	1:55636974-55637080	0	0	0	OK

ENSBTAG00000042391	ENSBTAG00000042391	6:70053897-70054019	0	0	0	OK
ENSBTAG00000042393	ENSBTAG00000042393	16:66374575-66374678	0	0	0	OK
ENSBTAG00000042394	ENSBTAG00000042394	25:1520843-1520974	0	0	0	OK
ENSBTAG00000042395	ENSBTAG00000042395	7:73654938-73655045	0	0	0	OK
ENSBTAG00000042396	ENSBTAG00000042396	16:56907662-56907764	0	0	0	OK
ENSBTAG00000042397	ENSBTAG00000042397	21:2232313-2232394	0	0	0	OK
ENSBTAG00000042398	ENSBTAG00000042398	19:49114968-49115103	0	0	0	OK
ENSBTAG00000042400	ENSBTAG00000042400	9:73656229-73656336	0	0	0	OK
ENSBTAG00000042401	ENSBTAG00000042401	21:67489519-67489594	0	0	0	OK
ENSBTAG00000042402	ENSBTAG00000042402	2:13767665-13767772	0	0	0	OK
ENSBTAG00000042403	ENSBTAG00000042403	22:21976770-21976877	0	0	0	OK
ENSBTAG00000042404	ENSBTAG00000042404	3:19579309-19579416	0	0	0	OK
ENSBTAG00000042405	ENSBTAG00000042405	7:40722341-40722448	0	0	0	OK
ENSBTAG00000042406	ENSBTAG00000042406	10:13320917-13320988	0	0	0	OK
ENSBTAG00000042407	ENSBTAG00000042407	15:69877186-69877293	0	0	0	OK
ENSBTAG00000042408	ENSBTAG00000042408	12:32854555-32854682	0	0	0	OK
ENSBTAG00000042409	ENSBTAG00000042409	4:10266500-10266612	0	0	0	OK
ENSBTAG00000042411	ENSBTAG00000042411	3:42476550-42476656	0	0	0	OK
ENSBTAG00000042412	ENSBTAG00000042412	21:67510857-67510931	0	0	0	OK
ENSBTAG00000042414	ENSBTAG00000042414	5:94455658-94455841	0	0	0	OK
ENSBTAG00000042415	ENSBTAG00000042415	19:10116414-10116521	0	0	0	OK
ENSBTAG00000042416	ENSBTAG00000042416	19:33577135-33577456	0	0	0	OK
ENSBTAG00000042417	ENSBTAG00000042417	5:63091054-63091302	0	0	0	OK
ENSBTAG00000042418	ENSBTAG00000042418	7:4787912-4788015	0	0	0	OK
ENSBTAG00000042420	ENSBTAG00000042420	5:66658141-66658248	0	0	0	OK
ENSBTAG00000042421	ENSBTAG00000042421	21:56158814-56158888	0	0	0	OK
ENSBTAG00000042423	ENSBTAG00000042423	17:30118537-30118671	0	0	0	OK
ENSBTAG00000042425	ENSBTAG00000042425	7:3644148-3644255	0	0	0	OK
ENSBTAG00000042426	ENSBTAG00000042426	X:61727516-61727621	0	0	0	OK
ENSBTAG00000042427	ENSBTAG00000042427	6:42431794-42431898	0	0	0	OK

ENSBTAG00000042428	ENSBTAG00000042428	6:117286694-117286801	0	0	0	OK
ENSBTAG00000042429	ENSBTAG00000042429	20:3272365-3272470	0	0	0	OK
ENSBTAG00000042430	ENSBTAG00000042430	18:15272619-15272749	0	0	0	OK
ENSBTAG00000042432	ENSBTAG00000042432	4:61666832-61666939	0	0	0	OK
ENSBTAG00000042433	ENSBTAG00000042433	18:18713274-18713356	0	0	0	OK
ENSBTAG00000042434	ENSBTAG00000042434	3:50787795-50787928	0	0	0	OK
ENSBTAG00000042438	ENSBTAG00000042438	21:67535302-67535376	0	0	0	OK
ENSBTAG00000042439	ENSBTAG00000042439	18:18713496-18713566	0	0	0	OK
ENSBTAG00000042440	ENSBTAG00000042440	4:77312168-77312300	0	0	0	OK
ENSBTAG00000042442	ENSBTAG00000042442	21:67526469-67526539	0	0	0	OK
ENSBTAG00000042443	ENSBTAG00000042443	3:108721103-108721211	0	0	0	OK
ENSBTAG00000042444	ENSBTAG00000042444	11:50365378-50365485	0	0	0	OK
ENSBTAG00000042445	ENSBTAG00000042445	18:53346141-53346248	0	0	0	OK
ENSBTAG00000042446	ENSBTAG00000042446	7:39898217-39898324	0	0	0	OK
ENSBTAG00000042447	ENSBTAG00000042447	19:33814770-33814837	0	0	0	OK
ENSBTAG00000042448	ENSBTAG00000042448	16:26360397-26360505	0	0	0	OK
ENSBTAG00000042449	ENSBTAG00000042449	15:39699723-39699853	0	0	0	OK
ENSBTAG00000042450	ENSBTAG00000042450	X:3895946-3896073	0	0	0	OK
ENSBTAG00000042452	ENSBTAG00000042452	25:2663189-2663305	0	0	0	OK
ENSBTAG00000042453	ENSBTAG00000042453	6:44553809-44553916	0	0	0	OK
ENSBTAG00000042454	ENSBTAG00000042454	12:23546236-23546343	0	0	0	OK
ENSBTAG00000042455	ENSBTAG00000042455	25:20029942-20030050	0	0	0	OK
ENSBTAG00000042456	ENSBTAG00000042456	5:111267150-111267214	0	0	0	OK
ENSBTAG00000042458	ENSBTAG00000042458	8:60244065-60244343	0	0	0	OK
ENSBTAG00000042459	ENSBTAG00000042459	20:49070194-49070327	0	0	0	OK
ENSBTAG00000042460	ENSBTAG00000042460	1:134948226-134948330	0	0	0	OK
ENSBTAG00000042461	ENSBTAG00000042461	19:55824567-55824652	0	0	0	OK
ENSBTAG00000042462	ENSBTAG00000042462	8:32189113-32189220	0	0	0	OK
ENSBTAG00000042463	ENSBTAG00000042463	1:140993152-140993232	0	0	0	OK
ENSBTAG00000042464	ENSBTAG00000042464	29:41855394-41855457	0	0	0	OK

ENSBTAG00000042466	ENSBTAG00000042466	X:125687187-125687335	0	0	0	OK
ENSBTAG00000042467	ENSBTAG00000042467	1:142200180-142200287	0	0	0	OK
ENSBTAG00000042468	ENSBTAG00000042468	5:81764762-81764864	0	0	0	OK
ENSBTAG00000042470	ENSBTAG00000042470	24:35127005-35127111	0	0	0	OK
ENSBTAG00000042471	ENSBTAG00000042471	29:1067323-1067450	0	0	0	OK
ENSBTAG00000042472	ENSBTAG00000042472	7:39973218-39973325	0	0	0	OK
ENSBTAG00000042473	ENSBTAG00000042473	12:22972443-22972577	0	0	0	OK
ENSBTAG00000042474	ENSBTAG00000042474	1:118527998-118528130	0	0	0	OK
ENSBTAG00000042475	ENSBTAG00000042475	1:81060419-81060551	0	0	0	OK
ENSBTAG00000042476	ENSBTAG00000042476	5:6423133-6423240	0	0	0	OK
ENSBTAG00000042478	ENSBTAG00000042478	10:8390696-8390830	0	0	0	OK
ENSBTAG00000042479	ENSBTAG00000042479	29:41855107-41855183	0	0	0	OK
ENSBTAG00000042480	ENSBTAG00000042480	17:12421049-12421153	0	0	0	OK
ENSBTAG00000042481	ENSBTAG00000042481	11:80251928-80252035	0	0	0	OK
ENSBTAG00000042482	ENSBTAG00000042482	11:104184904-104185036	0	0	0	OK
ENSBTAG00000042483	ENSBTAG00000042483	5:116232074-116232189	0	0	0	OK
ENSBTAG00000042484	ENSBTAG00000042484	29:41853939-41854066	0	0	0	OK
ENSBTAG00000042485	ENSBTAG00000042485	14:39493837-39493944	0	0	0	OK
ENSBTAG00000042488	ENSBTAG00000042488	13:47553869-47553976	0	0	0	OK
ENSBTAG00000042489	ENSBTAG00000042489	10:89677566-89677692	0	0	0	OK
ENSBTAG00000042490	ENSBTAG00000042490	20:24356249-24356373	0	0	0	OK
ENSBTAG00000042491	ENSBTAG00000042491	21:28785429-28785527	0	0	0	OK
ENSBTAG00000042492	ENSBTAG00000042492	14:71615394-71615529	0	0	0	OK
ENSBTAG00000042493	ENSBTAG00000042493	X:40366529-40366664	0	0	0	OK
ENSBTAG00000042494	ENSBTAG00000042494	25:26725470-26725577	0	0	0	OK
ENSBTAG00000042498	ENSBTAG00000042498	8:59427077-59427213	0	0	0	OK
ENSBTAG00000042499	ENSBTAG00000042499	14:63439285-63439411	0	0	0	OK
ENSBTAG00000042500	ENSBTAG00000042500	7:3875087-3875194	0	0	0	OK
ENSBTAG00000042501	ENSBTAG00000042501	13:64227614-64227799	0	0	0	OK
ENSBTAG00000042502	ENSBTAG00000042502	27:24093624-24093759	0	0	0	OK

ENSBTAG00000042503	ENSBTAG00000042503	6:99074103-99074243	0	0	0	OK
ENSBTAG00000042504	ENSBTAG00000042504	16:56692802-56692867	0	0	0	OK
ENSBTAG00000042506	ENSBTAG00000042506	3:101870235-101870351	0	0	0	OK
ENSBTAG00000042507	ENSBTAG00000042507	2:94925437-94925571	0	0	0	OK
ENSBTAG00000042508	ENSBTAG00000042508	21:67504076-67504151	0	0	0	OK
ENSBTAG00000042509	ENSBTAG00000042509	3:92240451-92240558	0	0	0	OK
ENSBTAG00000042511	ENSBTAG00000042511	X:15367741-15367848	0	0	0	OK
ENSBTAG00000042513	ENSBTAG00000042513	1:111089820-111089927	0	0	0	OK
ENSBTAG00000042514	ENSBTAG00000042514	4:21852512-21852634	0	0	0	OK
ENSBTAG00000042515	ENSBTAG00000042515	7:97567333-97567444	0	0	0	OK
ENSBTAG00000042517	ENSBTAG00000042517	7:23651623-23651730	0	0	0	OK
ENSBTAG00000042518	ENSBTAG00000042518	7:73786653-73786783	0	0	0	OK
ENSBTAG00000042519	ENSBTAG00000042519	21:67530801-67530873	0	0	0	OK
ENSBTAG00000042520	ENSBTAG00000042520	7:53322493-53322600	0	0	0	OK
ENSBTAG00000042521	ENSBTAG00000042521	13:70472053-70472160	0	0	0	OK
ENSBTAG00000042522	ENSBTAG00000042522	17:35096105-35096234	0	0	0	OK
ENSBTAG00000042523	ENSBTAG00000042523	28:41548682-41548785	0	0	0	OK
ENSBTAG00000042524	ENSBTAG00000042524	29:41852745-41852871	0	0	0	OK
ENSBTAG00000042525	ENSBTAG00000042525	25:2678320-2678423	0	0	0	OK
ENSBTAG00000042526	ENSBTAG00000042526	29:41854353-41854429	0	0	0	OK
ENSBTAG00000042527	ENSBTAG00000042527	13:67711974-67712081	0	0	0	OK
ENSBTAG00000042528	ENSBTAG00000042528	10:58356882-58356989	0	0	0	OK
ENSBTAG00000042529	ENSBTAG00000042529	14:36241731-36241837	0	0	0	OK
ENSBTAG00000042530	ENSBTAG00000042530	9:64122541-64122668	0	0	0	OK
ENSBTAG00000042531	ENSBTAG00000042531	5:108787983-108788073	0	0	0	OK
ENSBTAG00000042532	ENSBTAG00000042532	16:44768106-44768213	0	0	0	OK
ENSBTAG00000042533	ENSBTAG00000042533	13:76654191-76654298	0	0	0	OK
ENSBTAG00000042535	ENSBTAG00000042535	1:98082323-98082450	0	0	0	OK
ENSBTAG00000042536	ENSBTAG00000042536	5:31283917-31284056	0	0	0	OK
ENSBTAG00000042537	ENSBTAG00000042537	20:36523494-36523601	0	0	0	OK

ENSBTAG00000042538	ENSBTAG00000042538	16:74574336-74574443	0	0	0	OK
ENSBTAG00000042539	ENSBTAG00000042539	4:47483217-47483324	0	0	0	OK
ENSBTAG00000042540	ENSBTAG00000042540	2:87253458-87253565	0	0	0	OK
ENSBTAG00000042541	ENSBTAG00000042541	10:51948279-51948379	0	0	0	OK
ENSBTAG00000042543	ENSBTAG00000042543	21:67524075-67524143	0	0	0	OK
ENSBTAG00000042545	ENSBTAG00000042545	3:101817128-101817197	0	0	0	OK
ENSBTAG00000042546	ENSBTAG00000042546	6:60473610-60473733	0	0	0	OK
ENSBTAG00000042548	ENSBTAG00000042548	21:58367858-58367964	0	0	0	OK
ENSBTAG00000042549	ENSBTAG00000042549	9:93107026-93107170	0	0	0	OK
ENSBTAG00000042550	ENSBTAG00000042550	1:113137831-113137945	0	0	0	OK
ENSBTAG00000042551	ENSBTAG00000042551	1:119920563-119920669	0	0	0	OK
ENSBTAG00000042552	ENSBTAG00000042552	9:64623546-64623653	0	0	0	OK
ENSBTAG00000042553	ENSBTAG00000042553	2:125437887-125437961	0	0	0	OK
ENSBTAG00000042555	ENSBTAG00000042555	19:39500906-39501012	0	0	0	OK
ENSBTAG00000042556	ENSBTAG00000042556	X:34302843-34302950	0	0	0	OK
ENSBTAG00000042557	ENSBTAG00000042557	1:9865764-9865871	0	0	0	OK
ENSBTAG00000042558	ENSBTAG00000042558	6:62225326-62225649	0	0	0	OK
ENSBTAG00000042559	ENSBTAG00000042559	3:14901012-14901146	0	0	0	OK
ENSBTAG00000042561	ENSBTAG00000042561	12:54672433-54672540	0	0	0	OK
ENSBTAG00000042562	ENSBTAG00000042562	27:45359133-45359243	0	0	0	OK
ENSBTAG00000042563	ENSBTAG00000042563	17:9732392-9732499	0	0	0	OK
ENSBTAG00000042564	ENSBTAG00000042564	1:108528698-108528805	0	0	0	OK
ENSBTAG00000042565	ENSBTAG00000042565	11:104235860-104235967	0	0	0	OK
ENSBTAG00000042566	ENSBTAG00000042566	6:97960918-97961053	0	0	0	OK
ENSBTAG00000042567	ENSBTAG00000042567	10:73514416-73514519	0	0	0	OK
ENSBTAG00000042569	ENSBTAG00000042569	12:8621006-8621132	0	0	0	OK
ENSBTAG00000042573	ENSBTAG00000042573	5:89375485-89375588	0	0	0	OK
ENSBTAG00000042574	ENSBTAG00000042574	6:50595204-50595338	0	0	0	OK
ENSBTAG00000042575	ENSBTAG00000042575	8:92031272-92031373	0	0	0	OK
ENSBTAG00000042576	ENSBTAG00000042576	16:81132983-81133090	0	0	0	OK

ENSBTAG00000042577	ENSBTAG00000042577	14:23254312-23254419	0	0	0	OK
ENSBTAG00000042578	ENSBTAG00000042578	28:4845472-4845594	0	0	0	OK
ENSBTAG00000042579	ENSBTAG00000042579	10:25815733-25815843	0	0	0	OK
ENSBTAG00000042580	ENSBTAG00000042580	2:112052887-112053013	0	0	0	OK
ENSBTAG00000042581	ENSBTAG00000042581	1:152076535-152076667	0	0	0	OK
ENSBTAG00000042582	ENSBTAG00000042582	7:68428431-68428535	0	0	0	OK
ENSBTAG00000042584	ENSBTAG00000042584	6:27715119-27715222	0	0	0	OK
ENSBTAG00000042586	ENSBTAG00000042586	10:14658956-14659063	0	0	0	OK
ENSBTAG00000042587	ENSBTAG00000042587	23:33047246-33047357	0	0	0	OK
ENSBTAG00000042588	ENSBTAG00000042588	21:67501905-67501978	0	0	0	OK
ENSBTAG00000042589	ENSBTAG00000042589	18:46721751-46721854	0	0	0	OK
ENSBTAG00000042590	ENSBTAG00000042590	19:24566528-24566635	0	0	0	OK
ENSBTAG00000042592	ENSBTAG00000042592	18:24264483-24264590	0	0	0	OK
ENSBTAG00000042593	ENSBTAG00000042593	1:58736243-58736350	0	0	0	OK
ENSBTAG00000042594	ENSBTAG00000042594	10:12184642-12184760	0	0	0	OK
ENSBTAG00000042595	ENSBTAG00000042595	1:6120225-6120348	0	0	0	OK
ENSBTAG00000042597	ENSBTAG00000042597	2:30272850-30272957	0	0	0	OK
ENSBTAG00000042598	ENSBTAG00000042598	18:29603093-29603222	0	0	0	OK
ENSBTAG00000042599	ENSBTAG00000042599	13:73593939-73594045	0	0	0	OK
ENSBTAG00000042600	ENSBTAG00000042600	11:48600029-48600165	0	0	0	OK
ENSBTAG00000042601	ENSBTAG00000042601	9:39583058-39583372	0	0	0	OK
ENSBTAG00000042602	ENSBTAG00000042602	16:24379872-24380004	0	0	0	OK
ENSBTAG00000042603	ENSBTAG00000042603	21:67519503-67519575	0	0	0	OK
ENSBTAG00000042604	ENSBTAG00000042604	14:83907052-83907159	0	0	0	OK
ENSBTAG00000042605	ENSBTAG00000042605	8:38392483-38392623	0	0	0	OK
ENSBTAG00000042607	ENSBTAG00000042607	13:52985361-52985432	0	0	0	OK
ENSBTAG00000042608	ENSBTAG00000042608	4:118126731-118126838	0	0	0	OK
ENSBTAG00000042610	ENSBTAG00000042610	1:70869063-70869135	0	0	0	OK
ENSBTAG00000042611	ENSBTAG00000042611	28:29038414-29038521	0	0	0	OK
ENSBTAG00000042612	ENSBTAG00000042612	5:37800783-37800930	0	0	0	OK

ENSBTAG00000042613	ENSBTAG00000042613	18:57320899-57320993	0	0	0	OK
ENSBTAG00000042614	ENSBTAG00000042614	X:97732695-97732824	0	0	0	OK
ENSBTAG00000042615	ENSBTAG00000042615	10:32229110-32229310	0	0	0	OK
ENSBTAG00000042616	ENSBTAG00000042616	5:100916812-100916919	0	0	0	OK
ENSBTAG00000042618	ENSBTAG00000042618	12:50588112-50588457	0	0	0	OK
ENSBTAG00000042619	ENSBTAG00000042619	3:24490721-24490828	0	0	0	OK
ENSBTAG00000042620	ENSBTAG00000042620	14:47440617-47440752	0	0	0	OK
ENSBTAG00000042621	ENSBTAG00000042621	10:21552824-21552932	0	0	0	OK
ENSBTAG00000042622	ENSBTAG00000042622	2:47644964-47645071	0	0	0	OK
ENSBTAG00000042623	ENSBTAG00000042623	1:111299802-111299909	0	0	0	OK
ENSBTAG00000042624	ENSBTAG00000042624	7:55546681-55546805	0	0	0	OK
ENSBTAG00000042625	ENSBTAG00000042625	27:18252516-18252589	0	0	0	OK
ENSBTAG00000042626	ENSBTAG00000042626	7:75244500-75244626	0	0	0	OK
ENSBTAG00000042629	ENSBTAG00000042629	13:71975037-71975144	0	0	0	OK
ENSBTAG00000042630	ENSBTAG00000042630	19:27539052-27539197	0	0	0	OK
ENSBTAG00000042631	ENSBTAG00000042631	13:52985857-52985943	0	0	0	OK
ENSBTAG00000042633	ENSBTAG00000042633	5:112947482-112947589	0	0	0	OK
ENSBTAG00000042634	ENSBTAG00000042634	18:2577621-2577728	0	0	0	OK
ENSBTAG00000042636	ENSBTAG00000042636	23:50554658-50554764	0	0	0	OK
ENSBTAG00000042637	ENSBTAG00000042637	24:22759776-22759879	0	0	0	OK
ENSBTAG00000042638	ENSBTAG00000042638	5:113110354-113110471	0	0	0	OK
ENSBTAG00000042639	ENSBTAG00000042639	1:82813170-82813277	0	0	0	OK
ENSBTAG00000042640	ENSBTAG00000042640	21:61222678-61222785	0	0	0	OK
ENSBTAG00000042642	ENSBTAG00000042642	X:81513552-81513658	0	0	0	OK
ENSBTAG00000042643	ENSBTAG00000042643	7:61294715-61294819	0	0	0	OK
ENSBTAG00000042644	ENSBTAG00000042644	13:47845900-47846006	0	0	0	OK
ENSBTAG00000042645	ENSBTAG00000042645	3:101818497-101818601	0	0	0	OK
ENSBTAG00000042646	ENSBTAG00000042646	28:39047645-39047748	0	0	0	OK
ENSBTAG00000042647	ENSBTAG00000042647	17:13367244-13367351	0	0	0	OK
ENSBTAG00000042652	ENSBTAG00000042652	13:67988117-67988250	0	0	0	OK

ENSBTAG00000042653	ENSBTAG00000042653	24:1241932-1242039	0	0	0	OK
ENSBTAG00000042654	ENSBTAG00000042654	X:97810381-97810509	0	0	0	OK
ENSBTAG00000042655	ENSBTAG00000042655	16:38505000-38505108	0	0	0	OK
ENSBTAG00000042657	ENSBTAG00000042657	4:63704611-63704718	0	0	0	OK
ENSBTAG00000042659	ENSBTAG00000042659	16:76839471-76839606	0	0	0	OK
ENSBTAG00000042660	ENSBTAG00000042660	1:67510385-67510492	0	0	0	OK
ENSBTAG00000042661	ENSBTAG00000042661	3:107091585-107091720	0	0	0	OK
ENSBTAG00000042662	ENSBTAG00000042662	13:38537848-38538084	0	0	0	OK
ENSBTAG00000042663	ENSBTAG00000042663	19:50529210-50529414	0	0	0	OK
ENSBTAG00000042664	ENSBTAG00000042664	2:112850119-112850220	0	0	0	OK
ENSBTAG00000042665	ENSBTAG00000042665	6:46721278-46721385	0	0	0	OK
ENSBTAG00000042667	ENSBTAG00000042667	7:15592641-15592776	0	0	0	OK
ENSBTAG00000042668	ENSBTAG00000042668	2:118846642-118846749	0	0	0	OK
ENSBTAG00000042669	ENSBTAG00000042669	25:3753389-3753523	0	0	0	OK
ENSBTAG00000042673	ENSBTAG00000042673	21:23389603-23389732	0	0	0	OK
ENSBTAG00000042674	ENSBTAG00000042674	8:74377683-74377790	0	0	0	OK
ENSBTAG00000042676	ENSBTAG00000042676	4:112950966-112951062	0	0	0	OK
ENSBTAG00000042677	ENSBTAG00000042677	5:57121851-57121922	0	0	0	OK
ENSBTAG00000042679	ENSBTAG00000042679	21:67514398-67514470	0	0	0	OK
ENSBTAG00000042680	ENSBTAG00000042680	19:18581904-18582027	0	0	0	OK
ENSBTAG00000042681	ENSBTAG00000042681	7:40670260-40670368	0	0	0	OK
ENSBTAG00000042683	ENSBTAG00000042683	5:112591237-112591341	0	0	0	OK
ENSBTAG00000042684	ENSBTAG00000042684	15:35945602-35945689	0	0	0	OK
ENSBTAG00000042685	ENSBTAG00000042685	3:66338791-66338905	0	0	0	OK
ENSBTAG00000042687	ENSBTAG00000042687	5:29788722-29788829	0	0	0	OK
ENSBTAG00000042688	ENSBTAG00000042688	25:1521489-1521623	0	0	0	OK
ENSBTAG00000042689	ENSBTAG00000042689	5:37360294-37360401	0	0	0	OK
ENSBTAG00000042690	ENSBTAG00000042690	21:67520756-67520828	0	0	0	OK
ENSBTAG00000042691	ENSBTAG00000042691	26:18544895-18545017	0	0	0	OK
ENSBTAG00000042692	ENSBTAG00000042692	X:38876243-38876369	0	0	0	OK

ENSBTAG00000042693	ENSBTAG00000042693	7:40263147-40263254	0	0	0	OK
ENSBTAG00000042696	ENSBTAG00000042696	17:6703754-6703823	0	0	0	OK
ENSBTAG00000042697	ENSBTAG00000042697	5:42000471-42000578	0	0	0	OK
ENSBTAG00000042699	ENSBTAG00000042699	1:84254611-84254743	0	0	0	OK
ENSBTAG00000042700	ENSBTAG00000042700	19:16292949-16293056	0	0	0	OK
ENSBTAG00000042702	ENSBTAG00000042702	1:81060095-81060236	0	0	0	OK
ENSBTAG00000042704	ENSBTAG00000042704	13:27186288-27186412	0	0	0	OK
ENSBTAG00000042705	ENSBTAG00000042705	22:7776522-7776626	0	0	0	OK
ENSBTAG00000042706	ENSBTAG00000042706	19:55826141-55826219	0	0	0	OK
ENSBTAG00000042707	ENSBTAG00000042707	27:33061912-33062017	0	0	0	OK
ENSBTAG00000042708	ENSBTAG00000042708	25:36666932-36667039	0	0	0	OK
ENSBTAG00000042709	ENSBTAG00000042709	16:33607026-33607133	0	0	0	OK
ENSBTAG00000042710	ENSBTAG00000042710	17:59589336-59589443	0	0	0	OK
ENSBTAG00000042711	ENSBTAG00000042711	29:1079121-1079201	0	0	0	OK
ENSBTAG00000042712	ENSBTAG00000042712	24:2729062-2729197	0	0	0	OK
ENSBTAG00000042713	ENSBTAG00000042713	21:69641535-69641661	0	0	0	OK
ENSBTAG00000042714	ENSBTAG00000042714	X:18444017-18444127	0	0	0	OK
ENSBTAG00000042715	ENSBTAG00000042715	29:15500709-15500818	0	0	0	OK
ENSBTAG00000042718	ENSBTAG00000042718	3:16321432-16321567	0	0	0	OK
ENSBTAG00000042719	ENSBTAG00000042719	21:2239028-2239108	0	0	0	OK
ENSBTAG00000042720	ENSBTAG00000042720	X:139603335-139603443	0	0	0	OK
ENSBTAG00000042721	ENSBTAG00000042721	4:95366407-95366541	0	0	0	OK
ENSBTAG00000042722	ENSBTAG00000042722	22:9370819-9370922	0	0	0	OK
ENSBTAG00000042723	ENSBTAG00000042723	2:125390298-125390431	0	0	0	OK
ENSBTAG00000042724	ENSBTAG00000042724	13:16659677-16659780	0	0	0	OK
ENSBTAG00000042726	ENSBTAG00000042726	3:83329908-83330015	0	0	0	OK
ENSBTAG00000042727	ENSBTAG00000042727	21:67466610-67466680	0	0	0	OK
ENSBTAG00000042728	ENSBTAG00000042728	6:103943310-103943417	0	0	0	OK
ENSBTAG00000042729	ENSBTAG00000042729	9:101870815-101870922	0	0	0	OK
ENSBTAG00000042730	ENSBTAG00000042730	9:89150808-89150943	0	0	0	OK

ENSBTAG00000042731	ENSBTAG00000042731	10:48024971-48025076	0	0	0	OK
ENSBTAG00000042733	ENSBTAG00000042733	29:47287567-47287660	0	0	0	OK
ENSBTAG00000042734	ENSBTAG00000042734	2:26593528-26593635	0	0	0	OK
ENSBTAG00000042735	ENSBTAG00000042735	18:47397032-47397159	0	0	0	OK
ENSBTAG00000042736	ENSBTAG00000042736	3:67131321-67131429	0	0	0	OK
ENSBTAG00000042737	ENSBTAG00000042737	X:147792501-147792603	0	0	0	OK
ENSBTAG00000042738	ENSBTAG00000042738	3:106752649-106752756	0	0	0	OK
ENSBTAG00000042739	ENSBTAG00000042739	21:67484307-67484382	0	0	0	OK
ENSBTAG00000042741	ENSBTAG00000042741	19:49114707-49114777	0	0	0	OK
ENSBTAG00000042742	ENSBTAG00000042742	18:57319858-57319952	0	0	0	OK
ENSBTAG00000042743	ENSBTAG00000042743	X:47806716-47806849	0	0	0	OK
ENSBTAG00000042744	ENSBTAG00000042744	13:67986417-67986549	0	0	0	OK
ENSBTAG00000042746	ENSBTAG00000042746	2:110679232-110679339	0	0	0	OK
ENSBTAG00000042749	ENSBTAG00000042749	5:95825894-95826000	0	0	0	OK
ENSBTAG00000042750	ENSBTAG00000042750	11:36826307-36826435	0	0	0	OK
ENSBTAG00000042751	ENSBTAG00000042751	25:1693736-1693819	0	0	0	OK
ENSBTAG00000042753	ENSBTAG00000042753	21:42296835-42296984	0	0	0	OK
ENSBTAG00000042755	ENSBTAG00000042755	23:35722113-35722222	0	0	0	OK
ENSBTAG00000042757	ENSBTAG00000042757	9:97483109-97483239	0	0	0	OK
ENSBTAG00000042758	ENSBTAG00000042758	29:41853676-41853743	0	0	0	OK
ENSBTAG00000042760	ENSBTAG00000042760	17:23991157-23991264	0	0	0	OK
ENSBTAG00000042761	ENSBTAG00000042761	10:12405972-12406080	0	0	0	OK
ENSBTAG00000042762	ENSBTAG00000042762	5:112499441-112499572	0	0	0	OK
ENSBTAG00000042763	ENSBTAG00000042763	22:34693336-34693462	0	0	0	OK
ENSBTAG00000042764	ENSBTAG00000042764	19:46363349-46363456	0	0	0	OK
ENSBTAG00000042765	ENSBTAG00000042765	8:105380727-105380861	0	0	0	OK
ENSBTAG00000042767	ENSBTAG00000042767	9:50907634-50907738	0	0	0	OK
ENSBTAG00000042769	ENSBTAG00000042769	21:2236970-2237049	0	0	0	OK
ENSBTAG00000042770	ENSBTAG00000042770	8:4114520-4114662	0	0	0	OK
ENSBTAG00000042771	ENSBTAG00000042771	10:62298835-62298967	0	0	0	OK

ENSBTAG00000042772	ENSBTAG00000042772	1:91892151-91892224	0	0	0	OK
ENSBTAG00000042773	ENSBTAG00000042773	2:68791416-68791546	0	0	0	OK
ENSBTAG00000042774	ENSBTAG00000042774	16:70267199-70267306	0	0	0	OK
ENSBTAG00000042775	ENSBTAG00000042775	2:123192311-123192396	0	0	0	OK
ENSBTAG00000042776	ENSBTAG00000042776	3:77934833-77934940	0	0	0	OK
ENSBTAG00000042777	ENSBTAG00000042777	21:23274653-23274801	0	0	0	OK
ENSBTAG00000042780	ENSBTAG00000042780	19:23879872-23879967	0	0	0	OK
ENSBTAG00000042781	ENSBTAG00000042781	7:4387474-4387578	0	0	0	OK
ENSBTAG00000042782	ENSBTAG00000042782	15:53718870-53718974	0	0	0	OK
ENSBTAG00000042783	ENSBTAG00000042783	24:42924276-42924376	0	0	0	OK
ENSBTAG00000042784	ENSBTAG00000042784	18:57319374-57319466	0	0	0	OK
ENSBTAG00000042785	ENSBTAG00000042785	16:1439944-1440051	0	0	0	OK
ENSBTAG00000042786	ENSBTAG00000042786	18:14533789-14533871	0	0	0	OK
ENSBTAG00000042787	ENSBTAG00000042787	19:18482606-18482737	0	0	0	OK
ENSBTAG00000042788	ENSBTAG00000042788	2:118230052-118230161	0	0	0	OK
ENSBTAG00000042789	ENSBTAG00000042789	3:105578503-105578607	0	0	0	OK
ENSBTAG00000042790	ENSBTAG00000042790	7:52936423-52936529	0	0	0	OK
ENSBTAG00000042792	ENSBTAG00000042792	18:33990686-33991116	0	0	0	OK
ENSBTAG00000042795	ENSBTAG00000042795	4:82597221-82597349	0	0	0	OK
ENSBTAG00000042797	ENSBTAG00000042797	8:26648554-26648661	0	0	0	OK
ENSBTAG00000042799	ENSBTAG00000042799	X:16165875-16165982	0	0	0	OK
ENSBTAG00000042801	ENSBTAG00000042801	9:71976826-71976903	0	0	0	OK
ENSBTAG00000042803	ENSBTAG00000042803	7:1660818-1660948	0	0	0	OK
ENSBTAG00000042804	ENSBTAG00000042804	1:41209067-41209173	0	0	0	OK
ENSBTAG00000042805	ENSBTAG00000042805	21:67539802-67539874	0	0	0	OK
ENSBTAG00000042806	ENSBTAG00000042806	18:1650490-1650570	0	0	0	OK
ENSBTAG00000042807	ENSBTAG00000042807	28:8664806-8664913	0	0	0	OK
ENSBTAG00000042808	ENSBTAG00000042808	13:24168114-24168220	0	0	0	OK
ENSBTAG00000042810	ENSBTAG00000042810	2:88003257-88003360	0	0	0	OK
ENSBTAG00000042811	ENSBTAG00000042811	22:20840723-20840830	0	0	0	OK

ENSBTAG00000042812	ENSBTAG00000042812	21:2133093-2133185	0	0	0	OK
ENSBTAG00000042813	ENSBTAG00000042813	10:48008440-48008572	0	0	0	OK
ENSBTAG00000042814	ENSBTAG00000042814	1:117220928-117221040	0	0	0	OK
ENSBTAG00000042815	ENSBTAG00000042815	7:59609542-59609649	0	0	0	OK
ENSBTAG00000042816	ENSBTAG00000042816	17:5757359-5757465	0	0	0	OK
ENSBTAG00000042817	ENSBTAG00000042817	4:103195859-103195956	0	0	0	OK
ENSBTAG00000042818	ENSBTAG00000042818	2:31220644-31220751	0	0	0	OK
ENSBTAG00000042819	ENSBTAG00000042819	7:88650305-88650368	0	0	0	OK
ENSBTAG00000042820	ENSBTAG00000042820	11:20976370-20976474	0	0	0	OK
ENSBTAG00000042821	ENSBTAG00000042821	29:5703058-5703167	0	0	0	OK
ENSBTAG00000042823	ENSBTAG00000042823	21:43216351-43216458	0	0	0	OK
ENSBTAG00000042825	ENSBTAG00000042825	2:131814027-131814135	0	0	0	OK
ENSBTAG00000042826	ENSBTAG00000042826	2:24119470-24119577	0	0	0	OK
ENSBTAG00000042827	ENSBTAG00000042827	19:42497765-42497869	0	0	0	OK
ENSBTAG00000042829	ENSBTAG00000042829	11:63943851-63943974	0	0	0	OK
ENSBTAG00000042830	ENSBTAG00000042830	6:37920070-37920177	0	0	0	OK
ENSBTAG00000042832	ENSBTAG00000042832	4:77949092-77949199	0	0	0	OK
ENSBTAG00000042833	ENSBTAG00000042833	9:71977855-71977985	0	0	0	OK
ENSBTAG00000042834	ENSBTAG00000042834	14:32834386-32834493	0	0	0	OK
ENSBTAG00000042835	ENSBTAG00000042835	24:57177472-57177579	0	0	0	OK
ENSBTAG00000042836	ENSBTAG00000042836	19:27917463-27917598	0	0	0	OK
ENSBTAG00000042837	ENSBTAG00000042837	27:18252332-18252409	0	0	0	OK
ENSBTAG00000042838	ENSBTAG00000042838	16:62596618-62596725	0	0	0	OK
ENSBTAG00000042839	ENSBTAG00000042839	1:59234430-59234537	0	0	0	OK
ENSBTAG00000042840	ENSBTAG00000042840	19:616012-616119	0	0	0	OK
ENSBTAG00000042841	ENSBTAG00000042841	4:99693540-99693648	0	0	0	OK
ENSBTAG00000042842	ENSBTAG00000042842	5:111258703-111258838	0	0	0	OK
ENSBTAG00000042843	ENSBTAG00000042843	8:94230961-94231065	0	0	0	OK
ENSBTAG00000042844	ENSBTAG00000042844	15:17612524-17612631	0	0	0	OK
ENSBTAG00000042845	ENSBTAG00000042845	21:67515973-67516045	0	0	0	OK

ENSBTAG00000042846	ENSBTAG00000042846	29:1066599-1066673	0	0	0	OK
ENSBTAG00000042847	ENSBTAG00000042847	22:33129160-33129267	0	0	0	OK
ENSBTAG00000042848	ENSBTAG00000042848	5:78714558-78714659	0	0	0	OK
ENSBTAG00000042849	ENSBTAG00000042849	12:44865038-44865164	0	0	0	OK
ENSBTAG00000042851	ENSBTAG00000042851	16:58520020-58520149	0	0	0	OK
ENSBTAG00000042852	ENSBTAG00000042852	19:36589049-36589183	0	0	0	OK
ENSBTAG00000042853	ENSBTAG00000042853	8:11009160-11009235	0	0	0	OK
ENSBTAG00000042854	ENSBTAG00000042854	10:81098621-81098728	0	0	0	OK
ENSBTAG00000042855	ENSBTAG00000042855	11:51605825-51605956	0	0	0	OK
ENSBTAG00000042857	ENSBTAG00000042857	3:100529056-100529163	0	0	0	OK
ENSBTAG00000042858	ENSBTAG00000042858	2:18513102-18513245	0	0	0	OK
ENSBTAG00000042862	ENSBTAG00000042862	9:32980713-32980839	0	0	0	OK
ENSBTAG00000042863	ENSBTAG00000042863	22:56985901-56986041	0	0	0	OK
ENSBTAG00000042864	ENSBTAG00000042864	4:89714319-89714426	0	0	0	OK
ENSBTAG00000042865	ENSBTAG00000042865	24:61149634-61149703	0	0	0	OK
ENSBTAG00000042866	ENSBTAG00000042866	2:2860086-2860216	0	0	0	OK
ENSBTAG00000042867	ENSBTAG00000042867	19:37023663-37023770	0	0	0	OK
ENSBTAG00000042868	ENSBTAG00000042868	19:27920358-27920502	0	0	0	OK
ENSBTAG00000042869	ENSBTAG00000042869	21:65162860-65162967	0	0	0	OK
ENSBTAG00000042870	ENSBTAG00000042870	24:37069281-37069361	0	0	0	OK
ENSBTAG00000042871	ENSBTAG00000042871	2:125436794-125436925	0	0	0	OK
ENSBTAG00000042873	ENSBTAG00000042873	19:21483244-21483352	0	0	0	OK
ENSBTAG00000042874	ENSBTAG00000042874	3:84008572-84008674	0	0	0	OK
ENSBTAG00000042875	ENSBTAG00000042875	22:12740260-12740409	0	0	0	OK
ENSBTAG00000042876	ENSBTAG00000042876	19:37368256-37368364	0	0	0	OK
ENSBTAG00000042877	ENSBTAG00000042877	17:45513543-45513650	0	0	0	OK
ENSBTAG00000042878	ENSBTAG00000042878	13:29800604-29800728	0	0	0	OK
ENSBTAG00000042879	ENSBTAG00000042879	2:27306677-27306780	0	0	0	OK
ENSBTAG00000042880	ENSBTAG00000042880	2:106981630-106981767	0	0	0	OK
ENSBTAG00000042881	ENSBTAG00000042881	6:16307380-16307515	0	0	0	OK

ENSBTAG00000042882	ENSBTAG00000042882	21:67492479-67492553	0	0	0	OK
ENSBTAG00000042883	ENSBTAG00000042883	18:56397450-56397539	0	0	0	OK
ENSBTAG00000042884	ENSBTAG00000042884	X:85197993-85198063	0	0	0	OK
ENSBTAG00000042885	ENSBTAG00000042885	X:39864691-39864798	0	0	0	OK
ENSBTAG00000042886	ENSBTAG00000042886	23:34674410-34674519	0	0	0	OK
ENSBTAG00000042887	ENSBTAG00000042887	2:88498950-88499057	0	0	0	OK
ENSBTAG00000042888	ENSBTAG00000042888	16:44685965-44686072	0	0	0	OK
ENSBTAG00000042889	ENSBTAG00000042889	X:141847352-141847459	0	0	0	OK
ENSBTAG00000042890	ENSBTAG00000042890	13:32429505-32429591	0	0	0	OK
ENSBTAG00000042891	ENSBTAG00000042891	2:131334481-131334588	0	0	0	OK
ENSBTAG00000042893	ENSBTAG00000042893	18:1594821-1594925	0	0	0	OK
ENSBTAG00000042896	ENSBTAG00000042896	10:81712431-81712534	0	0	0	OK
ENSBTAG00000042897	ENSBTAG00000042897	19:42516885-42517005	0	0	0	OK
ENSBTAG00000042898	ENSBTAG00000042898	29:1078893-1078965	0	0	0	OK
ENSBTAG00000042899	ENSBTAG00000042899	6:73746598-73746725	0	0	0	OK
ENSBTAG00000042902	ENSBTAG00000042902	11:19895666-19895773	0	0	0	OK
ENSBTAG00000042905	ENSBTAG00000042905	28:25549742-25549846	0	0	0	OK
ENSBTAG00000042907	ENSBTAG00000042907	6:50977331-50977443	0	0	0	OK
ENSBTAG00000042910	ENSBTAG00000042910	7:19775199-19775269	0	0	0	OK
ENSBTAG00000042911	ENSBTAG00000042911	8:50983591-50983661	0	0	0	OK
ENSBTAG00000042914	ENSBTAG00000042914	4:73579672-73579781	0	0	0	OK
ENSBTAG00000042915	ENSBTAG00000042915	X:85198205-85198285	0	0	0	OK
ENSBTAG00000042916	ENSBTAG00000042916	15:55374441-55374585	0	0	0	OK
ENSBTAG00000042917	ENSBTAG00000042917	8:59682716-59682823	0	0	0	OK
ENSBTAG00000042918	ENSBTAG00000042918	2:129469309-129469419	0	0	0	OK
ENSBTAG00000042919	ENSBTAG00000042919	5:101201528-101201635	0	0	0	OK
ENSBTAG00000042920	ENSBTAG00000042920	29:2528372-2528481	0	0	0	OK
ENSBTAG00000042921	ENSBTAG00000042921	22:52757611-52757714	0	0	0	OK
ENSBTAG00000042922	ENSBTAG00000042922	26:20894774-20894881	0	0	0	OK
ENSBTAG00000042924	ENSBTAG00000042924	5:107865784-107865917	0	0	0	OK

ENSBTAG00000042925	ENSBTAG00000042925	8:16438377-16438466	0	0	0	OK
ENSBTAG00000042926	ENSBTAG00000042926	5:1158053-1158160	0	0	0	OK
ENSBTAG00000042927	ENSBTAG00000042927	6:31415762-31415874	0	0	0	OK
ENSBTAG00000042930	ENSBTAG00000042930	11:104183741-104183865	0	0	0	OK
ENSBTAG00000042931	ENSBTAG00000042931	16:56694019-56694082	0	0	0	OK
ENSBTAG00000042933	ENSBTAG00000042933	2:36501065-36501201	0	0	0	OK
ENSBTAG00000042934	ENSBTAG00000042934	X:103147031-103147137	0	0	0	OK
ENSBTAG00000042935	ENSBTAG00000042935	5:112759732-112759839	0	0	0	OK
ENSBTAG00000042936	ENSBTAG00000042936	7:41764651-41764730	0	0	0	OK
ENSBTAG00000042937	ENSBTAG00000042937	27:36622574-36622681	0	0	0	OK
ENSBTAG00000042938	ENSBTAG00000042938	5:44390511-44390615	0	0	0	OK
ENSBTAG00000042939	ENSBTAG00000042939	2:8001399-8001504	0	0	0	OK
ENSBTAG00000042940	ENSBTAG00000042940	3:45755685-45755792	0	0	0	OK
ENSBTAG00000042941	ENSBTAG00000042941	21:27129689-27129824	0	0	0	OK
ENSBTAG00000042942	ENSBTAG00000042942	22:10122124-10122230	0	0	0	OK
ENSBTAG00000042943	ENSBTAG00000042943	12:48186736-48186843	0	0	0	OK
ENSBTAG00000042944	ENSBTAG00000042944	20:20900430-20900534	0	0	0	OK
ENSBTAG00000042946	ENSBTAG00000042946	5:111262310-111262404	0	0	0	OK
ENSBTAG00000042947	ENSBTAG00000042947	4:34832629-34832736	0	0	0	OK
ENSBTAG00000042948	ENSBTAG00000042948	6:71604757-71604864	0	0	0	OK
ENSBTAG00000042949	ENSBTAG00000042949	4:37896811-37896918	0	0	0	OK
ENSBTAG00000042950	ENSBTAG00000042950	3:69323074-69323153	0	0	0	OK
ENSBTAG00000042951	ENSBTAG00000042951	6:24060224-24060327	0	0	0	OK
ENSBTAG00000042952	ENSBTAG00000042952	5:10275512-10275638	0	0	0	OK
ENSBTAG00000042953	ENSBTAG00000042953	21:67531086-67531158	0	0	0	OK
ENSBTAG00000042954	ENSBTAG00000042954	X:134636092-134636196	0	0	0	OK
ENSBTAG00000042955	ENSBTAG00000042955	9:30187174-30187281	0	0	0	OK
ENSBTAG00000042956	ENSBTAG00000042956	3:101781320-101781437	0	0	0	OK
ENSBTAG00000042957	ENSBTAG00000042957	5:71251610-71251717	0	0	0	OK
ENSBTAG00000042960	ENSBTAG00000042960	4:62492410-62492741	0	0	0	OK

ENSBTAG00000042961	ENSBTAG00000042961	19:57276184-57276291	0	0	0	OK
ENSBTAG00000042962	ENSBTAG00000042962	19:6256947-6257032	0	0	0	OK
ENSBTAG00000042963	ENSBTAG00000042963	29:1065600-1065737	0	0	0	OK
ENSBTAG00000042965	ENSBTAG00000042965	20:60123870-60123977	0	0	0	OK
ENSBTAG00000042966	ENSBTAG00000042966	21:30124642-30124749	0	0	0	OK
ENSBTAG00000042968	ENSBTAG00000042968	15:17996368-17996475	0	0	0	OK
ENSBTAG00000042969	ENSBTAG00000042969	19:18708751-18708858	0	0	0	OK
ENSBTAG00000042970	ENSBTAG00000042970	18:39373802-39373909	0	0	0	OK
ENSBTAG00000042971	ENSBTAG00000042971	26:6765487-6765594	0	0	0	OK
ENSBTAG00000042972	ENSBTAG00000042972	20:8133958-8134060	0	0	0	OK
ENSBTAG00000042973	ENSBTAG00000042973	19:53571989-53572065	0	0	0	OK
ENSBTAG00000042974	ENSBTAG00000042974	16:56693778-56693860	0	0	0	OK
ENSBTAG00000042976	ENSBTAG00000042976	13:40793315-40793450	0	0	0	OK
ENSBTAG00000042977	ENSBTAG00000042977	10:81143496-81143603	0	0	0	OK
ENSBTAG00000042980	ENSBTAG00000042980	X:146523692-146523799	0	0	0	OK
ENSBTAG00000042981	ENSBTAG00000042981	24:37530613-37530716	0	0	0	OK
ENSBTAG00000042982	ENSBTAG00000042982	26:42369694-42369801	0	0	0	OK
ENSBTAG00000042984	ENSBTAG00000042984	23:27488288-27488419	0	0	0	OK
ENSBTAG00000042986	ENSBTAG00000042986	2:125436172-125436307	0	0	0	OK
ENSBTAG00000042987	ENSBTAG00000042987	12:21694195-21694327	0	0	0	OK
ENSBTAG00000042988	ENSBTAG00000042988	15:46677929-46678035	0	0	0	OK
ENSBTAG00000042989	ENSBTAG00000042989	23:27325235-27325302	0	0	0	OK
ENSBTAG00000042990	ENSBTAG00000042990	18:48237625-48237730	0	0	0	OK
ENSBTAG00000042991	ENSBTAG00000042991	X:98705902-98706008	0	0	0	OK
ENSBTAG00000042992	ENSBTAG00000042992	12:60428296-60428403	0	0	0	OK
ENSBTAG00000042993	ENSBTAG00000042993	22:18940824-18940931	0	0	0	OK
ENSBTAG00000042995	ENSBTAG00000042995	14:41513835-41513938	0	0	0	OK
ENSBTAG00000042996	ENSBTAG00000042996	22:31094554-31094661	0	0	0	OK
ENSBTAG00000042997	ENSBTAG00000042997	11:7255468-7255575	0	0	0	OK
ENSBTAG00000042998	ENSBTAG00000042998	9:71844588-71844686	0	0	0	OK

ENSBTAG00000042999	ENSBTAG00000042999	22:17102897-17103004	0	0	0	OK
ENSBTAG00000043000	ENSBTAG00000043000	29:1066221-1066355	0	0	0	OK
ENSBTAG00000043001	ENSBTAG00000043001	2:22303364-22303470	0	0	0	OK
ENSBTAG00000043002	ENSBTAG00000043002	X:39823099-39823227	0	0	0	OK
ENSBTAG00000043003	ENSBTAG00000043003	26:15519512-15519806	0	0	0	OK
ENSBTAG00000043004	ENSBTAG00000043004	23:27837207-27837314	0	0	0	OK
ENSBTAG00000043005	ENSBTAG00000043005	18:51812573-51812698	0	0	0	OK
ENSBTAG00000043006	ENSBTAG00000043006	4:25825582-25825648	0	0	0	OK
ENSBTAG00000043009	ENSBTAG00000043009	15:47923064-47923170	0	0	0	OK
ENSBTAG00000043011	ENSBTAG00000043011	3:69180242-69180349	0	0	0	OK
ENSBTAG00000043012	ENSBTAG00000043012	23:32700053-32700160	0	0	0	OK
ENSBTAG00000043013	ENSBTAG00000043013	10:45930447-45930554	0	0	0	OK
ENSBTAG00000043014	ENSBTAG00000043014	23:50519766-50519872	0	0	0	OK
ENSBTAG00000043015	ENSBTAG00000043015	3:87445318-87445418	0	0	0	OK
ENSBTAG00000043016	ENSBTAG00000043016	11:104313767-104313828	0	0	0	OK
ENSBTAG00000043018	ENSBTAG00000043018	21:52067123-52067263	0	0	0	OK
ENSBTAG00000043019	ENSBTAG00000043019	X:134018853-134018961	0	0	0	OK
ENSBTAG00000043020	ENSBTAG00000043020	23:39248789-39248896	0	0	0	OK
ENSBTAG00000043021	ENSBTAG00000043021	20:53673005-53673136	0	0	0	OK
ENSBTAG00000043023	ENSBTAG00000043023	7:67319859-67319962	0	0	0	OK
ENSBTAG00000043024	ENSBTAG00000043024	2:118777019-118777123	0	0	0	OK
ENSBTAG00000043025	ENSBTAG00000043025	19:28383353-28383463	0	0	0	OK
ENSBTAG00000043026	ENSBTAG00000043026	8:77394103-77394210	0	0	0	OK
ENSBTAG00000043028	ENSBTAG00000043028	16:73144948-73145049	0	0	0	OK
ENSBTAG00000043029	ENSBTAG00000043029	5:76346984-76347091	0	0	0	OK
ENSBTAG00000043030	ENSBTAG00000043030	1:142530265-142530573	0	0	0	OK
ENSBTAG00000043032	ENSBTAG00000043032	28:39702816-39702923	0	0	0	OK
ENSBTAG00000043033	ENSBTAG00000043033	7:57304369-57304476	0	0	0	OK
ENSBTAG00000043034	ENSBTAG00000043034	21:67522315-67522390	0	0	0	OK
ENSBTAG00000043035	ENSBTAG00000043035	10:43192079-43192186	0	0	0	OK

ENSBTAG00000043036	ENSBTAG00000043036	13:78103994-78104085	0	0	0	OK
ENSBTAG00000043037	ENSBTAG00000043037	22:43581388-43581496	0	0	0	OK
ENSBTAG00000043038	ENSBTAG00000043038	4:35686155-35686260	0	0	0	OK
ENSBTAG00000043039	ENSBTAG00000043039	19:9486355-9486459	0	0	0	OK
ENSBTAG00000043040	ENSBTAG00000043040	21:67500941-67501018	0	0	0	OK
ENSBTAG00000043042	ENSBTAG00000043042	15:79513803-79513908	0	0	0	OK
ENSBTAG00000043043	ENSBTAG00000043043	4:18916668-18916818	0	0	0	OK
ENSBTAG00000043044	ENSBTAG00000043044	3:83725814-83725919	0	0	0	OK
ENSBTAG00000043045	ENSBTAG00000043045	9:39797080-39797187	0	0	0	OK
ENSBTAG00000043046	ENSBTAG00000043046	1:140992953-140993023	0	0	0	OK
ENSBTAG00000043047	ENSBTAG00000043047	11:98192709-98192838	0	0	0	OK
ENSBTAG00000043048	ENSBTAG00000043048	5:57120776-57120851	0	0	0	OK
ENSBTAG00000043049	ENSBTAG00000043049	18:1645719-1645810	0	0	0	OK
ENSBTAG00000043050	ENSBTAG00000043050	11:56863701-56863810	0	0	0	OK
ENSBTAG00000043051	ENSBTAG00000043051	5:66861000-66861106	0	0	0	OK
ENSBTAG00000043053	ENSBTAG00000043053	X:58011410-58011517	0	0	0	OK
ENSBTAG00000043054	ENSBTAG00000043054	11:26220038-26220125	0	0	0	OK
ENSBTAG00000043055	ENSBTAG00000043055	23:34438722-34438831	0	0	0	OK
ENSBTAG00000043056	ENSBTAG00000043056	23:6053957-6054061	0	0	0	OK
ENSBTAG00000043057	ENSBTAG00000043057	23:10581540-10581646	0	0	0	OK
ENSBTAG00000043059	ENSBTAG00000043059	8:99352723-99352824	0	0	0	OK
ENSBTAG00000043064	ENSBTAG00000043064	1:10612425-10612532	0	0	0	OK
ENSBTAG00000043066	ENSBTAG00000043066	17:10817955-10818059	0	0	0	OK
ENSBTAG00000043067	ENSBTAG00000043067	9:21837090-21837216	0	0	0	OK
ENSBTAG00000043068	ENSBTAG00000043068	9:47370073-47370205	0	0	0	OK
ENSBTAG00000043070	ENSBTAG00000043070	10:96942680-96942798	0	0	0	OK
ENSBTAG00000043071	ENSBTAG00000043071	17:6163767-6163904	0	0	0	OK
ENSBTAG00000043072	ENSBTAG00000043072	8:51238641-51238699	0	0	0	OK
ENSBTAG00000043073	ENSBTAG00000043073	22:56660674-56660781	0	0	0	OK
ENSBTAG00000043077	ENSBTAG00000043077	13:52986832-52986968	0	0	0	OK

ENSBTAG00000043078	ENSBTAG00000043078	8:73367167-73367271	0	0	0	OK
ENSBTAG00000043079	ENSBTAG00000043079	3:50229829-50229964	0	0	0	OK
ENSBTAG00000043080	ENSBTAG00000043080	21:2241134-2241215	0	0	0	OK
ENSBTAG00000043081	ENSBTAG00000043081	28:25207974-25208039	0	0	0	OK
ENSBTAG00000043083	ENSBTAG00000043083	20:22800132-22800236	0	0	0	OK
ENSBTAG00000043084	ENSBTAG00000043084	23:9872549-9872656	0	0	0	OK
ENSBTAG00000043085	ENSBTAG00000043085	10:6959429-6959545	0	0	0	OK
ENSBTAG00000043086	ENSBTAG00000043086	4:112975141-112975253	0	0	0	OK
ENSBTAG00000043088	ENSBTAG00000043088	15:74800363-74800465	0	0	0	OK
ENSBTAG00000043089	ENSBTAG00000043089	19:5426460-5426560	0	0	0	OK
ENSBTAG00000043090	ENSBTAG00000043090	26:42383779-42383880	0	0	0	OK
ENSBTAG00000043091	ENSBTAG00000043091	20:17695158-17695251	0	0	0	OK
ENSBTAG00000043092	ENSBTAG00000043092	11:49866378-49866485	0	0	0	OK
ENSBTAG00000043095	ENSBTAG00000043095	9:26690577-26690685	0	0	0	OK
ENSBTAG00000043096	ENSBTAG00000043096	15:21250164-21250271	0	0	0	OK
ENSBTAG00000043097	ENSBTAG00000043097	1:41529796-41529903	0	0	0	OK
ENSBTAG00000043098	ENSBTAG00000043098	15:77567438-77567549	0	0	0	OK
ENSBTAG00000043099	ENSBTAG00000043099	5:110777688-110777795	0	0	0	OK
ENSBTAG00000043100	ENSBTAG00000043100	21:68797763-68797880	0	0	0	OK
ENSBTAG00000043101	ENSBTAG00000043101	11:104312992-104313063	0	0	0	OK
ENSBTAG00000043103	ENSBTAG00000043103	8:74374319-74374456	0	0	0	OK
ENSBTAG00000043104	ENSBTAG00000043104	5:56978186-56978258	0	0	0	OK
ENSBTAG00000043105	ENSBTAG00000043105	8:48526947-48527063	0	0	0	OK
ENSBTAG00000043106	ENSBTAG00000043106	10:35726853-35726958	0	0	0	OK
ENSBTAG00000043107	ENSBTAG00000043107	2:119912873-119912980	0	0	0	OK
ENSBTAG00000043109	ENSBTAG00000043109	4:71415779-71415905	0	0	0	OK
ENSBTAG00000043111	ENSBTAG00000043111	2:63812154-63812280	0	0	0	OK
ENSBTAG00000043112	ENSBTAG00000043112	X:91819201-91819308	0	0	0	OK
ENSBTAG00000043114	ENSBTAG00000043114	X:67841391-67841498	0	0	0	OK
ENSBTAG00000043116	ENSBTAG00000043116	13:70424001-70424108	0	0	0	OK

ENSBTAG00000043117	ENSBTAG00000043117	19:41767731-41767857	0	0	0	OK
ENSBTAG00000043118	ENSBTAG00000043118	26:43412790-43412897	0	0	0	OK
ENSBTAG00000043119	ENSBTAG00000043119	2:104584182-104584303	0	0	0	OK
ENSBTAG00000043120	ENSBTAG00000043120	14:84619376-84619483	0	0	0	OK
ENSBTAG00000043121	ENSBTAG00000043121	5:9174030-9174166	0	0	0	OK
ENSBTAG00000043122	ENSBTAG00000043122	15:75509786-75509909	0	0	0	OK
ENSBTAG00000043123	ENSBTAG00000043123	19:17494126-17494233	0	0	0	OK
ENSBTAG00000043126	ENSBTAG00000043126	1:93217160-93217289	0	0	0	OK
ENSBTAG00000043127	ENSBTAG00000043127	X:73081671-73081774	0	0	0	OK
ENSBTAG00000043128	ENSBTAG00000043128	X:3998578-3998712	0	0	0	OK
ENSBTAG00000043130	ENSBTAG00000043130	X:40711310-40711396	0	0	0	OK
ENSBTAG00000043131	ENSBTAG00000043131	8:84413001-84413107	0	0	0	OK
ENSBTAG00000043132	ENSBTAG00000043132	20:1923842-1923948	0	0	0	OK
ENSBTAG00000043133	ENSBTAG00000043133	23:41248766-41248871	0	0	0	OK
ENSBTAG00000043135	ENSBTAG00000043135	2:73664454-73664580	0	0	0	OK
ENSBTAG00000043137	ENSBTAG00000043137	21:8873091-8873190	0	0	0	OK
ENSBTAG00000043138	ENSBTAG00000043138	15:46509411-46509515	0	0	0	OK
ENSBTAG00000043139	ENSBTAG00000043139	5:115027050-115027180	0	0	0	OK
ENSBTAG00000043140	ENSBTAG00000043140	22:48726936-48727013	0	0	0	OK
ENSBTAG00000043141	ENSBTAG00000043141	X:2261403-2261510	0	0	0	OK
ENSBTAG00000043142	ENSBTAG00000043142	24:48564977-48565084	0	0	0	OK
ENSBTAG00000043143	ENSBTAG00000043143	23:33127080-33127187	0	0	0	OK
ENSBTAG00000043144	ENSBTAG00000043144	4:94749887-94749994	0	0	0	OK
ENSBTAG00000043145	ENSBTAG00000043145	10:51505238-51505345	0	0	0	OK
ENSBTAG00000043146	ENSBTAG00000043146	29:45049044-45049151	0	0	0	OK
ENSBTAG00000043147	ENSBTAG00000043147	16:17066086-17066194	0	0	0	OK
ENSBTAG00000043148	ENSBTAG00000043148	10:11589432-11589539	0	0	0	OK
ENSBTAG00000043150	ENSBTAG00000043150	21:55803571-55803643	0	0	0	OK
ENSBTAG00000043151	ENSBTAG00000043151	23:27325743-27325806	0	0	0	OK
ENSBTAG00000043152	ENSBTAG00000043152	2:129988122-129988229	0	0	0	OK

ENSBTAG00000043153	ENSBTAG00000043153	8:60699246-60699372	0	0	0	OK
ENSBTAG00000043154	ENSBTAG00000043154	2:136603348-136603455	0	0	0	OK
ENSBTAG00000043156	ENSBTAG00000043156	8:61969008-61969115	0	0	0	OK
ENSBTAG00000043157	ENSBTAG00000043157	1:98401439-98401869	0	0	0	OK
ENSBTAG00000043158	ENSBTAG00000043158	11:6211556-6211670	0	0	0	OK
ENSBTAG00000043159	ENSBTAG00000043159	9:56657731-56657838	0	0	0	OK
ENSBTAG00000043160	ENSBTAG00000043160	25:9134443-9134550	0	0	0	OK
ENSBTAG00000043161	ENSBTAG00000043161	5:75252431-75252535	0	0	0	OK
ENSBTAG00000043162	ENSBTAG00000043162	28:44410272-44410472	0	0	0	OK
ENSBTAG00000043163	ENSBTAG00000043163	13:3593381-3593489	0	0	0	OK
ENSBTAG00000043164	ENSBTAG00000043164	11:10856503-10856607	0	0	0	OK
ENSBTAG00000043165	ENSBTAG00000043165	19:37550143-37550278	0	0	0	OK
ENSBTAG00000043166	ENSBTAG00000043166	2:94925090-94925164	0	0	0	OK
ENSBTAG00000043167	ENSBTAG00000043167	29:50769383-50769518	0	0	0	OK
ENSBTAG00000043168	ENSBTAG00000043168	18:56405736-56405823	0	0	0	OK
ENSBTAG00000043169	ENSBTAG00000043169	28:17291998-17292105	0	0	0	OK
ENSBTAG00000043170	ENSBTAG00000043170	3:99562452-99562559	0	0	0	OK
ENSBTAG00000043173	ENSBTAG00000043173	25:13695951-13696058	0	0	0	OK
ENSBTAG00000043175	ENSBTAG00000043175	2:23620289-23620396	0	0	0	OK
ENSBTAG00000043177	ENSBTAG00000043177	X:92194786-92194921	0	0	0	OK
ENSBTAG00000043178	ENSBTAG00000043178	12:1225020-1225124	0	0	0	OK
ENSBTAG00000043179	ENSBTAG00000043179	21:67512482-67512555	0	0	0	OK
ENSBTAG00000043180	ENSBTAG00000043180	24:47177401-47177505	0	0	0	OK
ENSBTAG00000043181	ENSBTAG00000043181	16:33060367-33060474	0	0	0	OK
ENSBTAG00000043182	ENSBTAG00000043182	1:141077216-141077323	0	0	0	OK
ENSBTAG00000043183	ENSBTAG00000043183	6:115682605-115682712	0	0	0	OK
ENSBTAG00000043184	ENSBTAG00000043184	20:45414398-45414504	0	0	0	OK
ENSBTAG00000043185	ENSBTAG00000043185	22:8270850-8270955	0	0	0	OK
ENSBTAG00000043186	ENSBTAG00000043186	3:103254569-103254669	0	0	0	OK
ENSBTAG00000043187	ENSBTAG00000043187	21:31740509-31740635	0	0	0	OK

ENSBTAG00000043188	ENSBTAG00000043188	X:25930245-25930352	0	0	0	OK
ENSBTAG00000043189	ENSBTAG00000043189	X:91266946-91267049	0	0	0	OK
ENSBTAG00000043190	ENSBTAG00000043190	22:14406300-14406369	0	0	0	OK
ENSBTAG00000043192	ENSBTAG00000043192	18:26421774-26421910	0	0	0	OK
ENSBTAG00000043194	ENSBTAG00000043194	2:91158139-91158224	0	0	0	OK
ENSBTAG00000043195	ENSBTAG00000043195	24:36318833-36318940	0	0	0	OK
ENSBTAG00000043196	ENSBTAG00000043196	10:25821327-25821436	0	0	0	OK
ENSBTAG00000043197	ENSBTAG00000043197	18:56396136-56396220	0	0	0	OK
ENSBTAG00000043198	ENSBTAG00000043198	3:44300128-44300234	0	0	0	OK
ENSBTAG00000043199	ENSBTAG00000043199	25:27124890-27124995	0	0	0	OK
ENSBTAG00000043200	ENSBTAG00000043200	17:56879745-56879852	0	0	0	OK
ENSBTAG00000043201	ENSBTAG00000043201	23:34365079-34365184	0	0	0	OK
ENSBTAG00000043202	ENSBTAG00000043202	9:64688688-64688758	0	0	0	OK
ENSBTAG00000043203	ENSBTAG00000043203	X:85171801-85171931	0	0	0	OK
ENSBTAG00000043205	ENSBTAG00000043205	2:126440072-126440179	0	0	0	OK
ENSBTAG00000043206	ENSBTAG00000043206	22:47261241-47261348	0	0	0	OK
ENSBTAG00000043207	ENSBTAG00000043207	16:56694436-56694516	0	0	0	OK
ENSBTAG00000043209	ENSBTAG00000043209	6:90451089-90451422	0	0	0	OK
ENSBTAG00000043210	ENSBTAG00000043210	6:72222719-72222826	0	0	0	OK
ENSBTAG00000043211	ENSBTAG00000043211	13:47114651-47114758	0	0	0	OK
ENSBTAG00000043212	ENSBTAG00000043212	14:36819378-36819467	0	0	0	OK
ENSBTAG00000043213	ENSBTAG00000043213	2:112265025-112265132	0	0	0	OK
ENSBTAG00000043214	ENSBTAG00000043214	5:56652650-56652800	0	0	0	OK
ENSBTAG00000043216	ENSBTAG00000043216	21:45902128-45902235	0	0	0	OK
ENSBTAG00000043217	ENSBTAG00000043217	9:97489074-97489211	0	0	0	OK
ENSBTAG00000043218	ENSBTAG00000043218	29:26861452-26861584	0	0	0	OK
ENSBTAG00000043219	ENSBTAG00000043219	6:71526867-71526974	0	0	0	OK
ENSBTAG00000043220	ENSBTAG00000043220	2:119511851-119511958	0	0	0	OK
ENSBTAG00000043221	ENSBTAG00000043221	1:152859143-152859270	0	0	0	OK
ENSBTAG00000043222	ENSBTAG00000043222	26:21014544-21014692	0	0	0	OK

ENSBTAG00000043223	ENSBTAG00000043223	21:55931007-55931114	0	0	0	OK
ENSBTAG00000043224	ENSBTAG00000043224	7:41766065-41766132	0	0	0	OK
ENSBTAG00000043225	ENSBTAG00000043225	8:85392055-85392162	0	0	0	OK
ENSBTAG00000043226	ENSBTAG00000043226	11:26575237-26575367	0	0	0	OK
ENSBTAG00000043227	ENSBTAG00000043227	11:99292405-99292509	0	0	0	OK
ENSBTAG00000043228	ENSBTAG00000043228	23:40173579-40173686	0	0	0	OK
ENSBTAG00000043229	ENSBTAG00000043229	6:107980498-107980618	0	0	0	OK
ENSBTAG00000043230	ENSBTAG00000043230	18:56336227-56336334	0	0	0	OK
ENSBTAG00000043231	ENSBTAG00000043231	6:93015295-93015402	0	0	0	OK
ENSBTAG00000043232	ENSBTAG00000043232	3:8401076-8401183	0	0	0	OK
ENSBTAG00000043233	ENSBTAG00000043233	8:85922131-85922258	0	0	0	OK
ENSBTAG00000043234	ENSBTAG00000043234	14:33116510-33116597	0	0	0	OK
ENSBTAG00000043235	ENSBTAG00000043235	13:25002182-25002313	0	0	0	OK
ENSBTAG00000043236	ENSBTAG00000043236	5:5805439-5805536	0	0	0	OK
ENSBTAG00000043237	ENSBTAG00000043237	X:95552021-95552148	0	0	0	OK
ENSBTAG00000043239	ENSBTAG00000043239	17:58614234-58614333	0	0	0	OK
ENSBTAG00000043240	ENSBTAG00000043240	18:55096589-55096699	0	0	0	OK
ENSBTAG00000043241	ENSBTAG00000043241	17:5594142-5594248	0	0	0	OK
ENSBTAG00000043242	ENSBTAG00000043242	6:3989484-3989591	0	0	0	OK
ENSBTAG00000043243	ENSBTAG00000043243	12:15519279-15519411	0	0	0	OK
ENSBTAG00000043244	ENSBTAG00000043244	19:9450872-9450979	0	0	0	OK
ENSBTAG00000043245	ENSBTAG00000043245	6:67638312-67638414	0	0	0	OK
ENSBTAG00000043246	ENSBTAG00000043246	11:96242760-96242864	0	0	0	OK
ENSBTAG00000043247	ENSBTAG00000043247	8:1346951-1347058	0	0	0	OK
ENSBTAG00000043249	ENSBTAG00000043249	5:31274160-31274296	0	0	0	OK
ENSBTAG00000043250	ENSBTAG00000043250	23:24977641-24977972	0	0	0	OK
ENSBTAG00000043251	ENSBTAG00000043251	24:48503024-48503130	0	0	0	OK
ENSBTAG00000043252	ENSBTAG00000043252	25:7902272-7902375	0	0	0	OK
ENSBTAG00000043253	ENSBTAG00000043253	11:9691437-9691543	0	0	0	OK
ENSBTAG00000043254	ENSBTAG00000043254	26:20371948-20372055	0	0	0	OK

ENSBTAG00000043255	ENSBTAG00000043255	23:34799388-34799677	0	0	0	OK
ENSBTAG00000043256	ENSBTAG00000043256	X:17320539-17320646	0	0	0	OK
ENSBTAG00000043257	ENSBTAG00000043257	9:18833842-18833949	0	0	0	OK
ENSBTAG00000043258	ENSBTAG00000043258	29:1064654-1064785	0	0	0	OK
ENSBTAG00000043259	ENSBTAG00000043259	7:74046181-74046288	0	0	0	OK
ENSBTAG00000043260	ENSBTAG00000043260	18:57322361-57322452	0	0	0	OK
ENSBTAG00000043261	ENSBTAG00000043261	2:91209280-91209369	0	0	0	OK
ENSBTAG00000043262	ENSBTAG00000043262	24:34278047-34278154	0	0	0	OK
ENSBTAG00000043263	ENSBTAG00000043263	24:49620124-49620187	0	0	0	OK
ENSBTAG00000043264	ENSBTAG00000043264	13:73738445-73738525	0	0	0	OK
ENSBTAG00000043266	ENSBTAG00000043266	2:632719-632828	0	0	0	OK
ENSBTAG00000043267	ENSBTAG00000043267	23:27947119-27947226	0	0	0	OK
ENSBTAG00000043268	ENSBTAG00000043268	16:56692480-56692561	0	0	0	OK
ENSBTAG00000043272	ENSBTAG00000043272	13:75302319-75302425	0	0	0	OK
ENSBTAG00000043273	ENSBTAG00000043273	7:38319494-38319618	0	0	0	OK
ENSBTAG00000043275	ENSBTAG00000043275	20:66662745-66662852	0	0	0	OK
ENSBTAG00000043276	ENSBTAG00000043276	15:35992881-35992988	0	0	0	OK
ENSBTAG00000043277	ENSBTAG00000043277	29:25586443-25586578	0	0	0	OK
ENSBTAG00000043278	ENSBTAG00000043278	1:72350841-72350948	0	0	0	OK
ENSBTAG00000043279	ENSBTAG00000043279	7:16763244-16763351	0	0	0	OK
ENSBTAG00000043281	ENSBTAG00000043281	20:66668617-66668724	0	0	0	OK
ENSBTAG00000043282	ENSBTAG00000043282	20:66736972-66737105	0	0	0	OK
ENSBTAG00000043284	ENSBTAG00000043284	4:44695823-44695930	0	0	0	OK
ENSBTAG00000043286	ENSBTAG00000043286	18:39601214-39601318	0	0	0	OK
ENSBTAG00000043287	ENSBTAG00000043287	4:68943156-68943227	0	0	0	OK
ENSBTAG00000043288	ENSBTAG00000043288	16:32198964-32199071	0	0	0	OK
ENSBTAG00000043290	ENSBTAG00000043290	19:28862247-28862351	0	0	0	OK
ENSBTAG00000043291	ENSBTAG00000043291	22:48688955-48689081	0	0	0	OK
ENSBTAG00000043293	ENSBTAG00000043293	1:101556755-101556863	0	0	0	OK
ENSBTAG00000043294	ENSBTAG00000043294	2:2128013-2128126	0	0	0	OK

ENSBTAG00000043295	ENSBTAG00000043295	10:1763368-1763505	0	0	0	OK
ENSBTAG00000043296	ENSBTAG00000043296	2:86422360-86422462	0	0	0	OK
ENSBTAG00000043297	ENSBTAG00000043297	2:120041107-120041187	0	0	0	OK
ENSBTAG00000043298	ENSBTAG00000043298	1:150977221-150977328	0	0	0	OK
ENSBTAG00000043299	ENSBTAG00000043299	5:25177107-25177410	0	0	0	OK
ENSBTAG00000043300	ENSBTAG00000043300	9:63733385-63733492	0	0	0	OK
ENSBTAG00000043301	ENSBTAG00000043301	24:49620763-49620828	0	0	0	OK
ENSBTAG00000043302	ENSBTAG00000043302	29:41853134-41853202	0	0	0	OK
ENSBTAG00000043303	ENSBTAG00000043303	12:54444669-54444774	0	0	0	OK
ENSBTAG00000043304	ENSBTAG00000043304	19:60532855-60532954	0	0	0	OK
ENSBTAG00000043305	ENSBTAG00000043305	15:43791936-43792121	0	0	0	OK
ENSBTAG00000043306	ENSBTAG00000043306	13:52987712-52987784	0	0	0	OK
ENSBTAG00000043307	ENSBTAG00000043307	23:42612289-42612396	0	0	0	OK
ENSBTAG00000043308	ENSBTAG00000043308	2:38485127-38485234	0	0	0	OK
ENSBTAG00000043311	ENSBTAG00000043311	7:43673388-43673492	0	0	0	OK
ENSBTAG00000043312	ENSBTAG00000043312	3:114583847-114583927	0	0	0	OK
ENSBTAG00000043313	ENSBTAG00000043313	14:65091808-65091920	0	0	0	OK
ENSBTAG00000043314	ENSBTAG00000043314	21:67537172-67537248	0	0	0	OK
ENSBTAG00000043315	ENSBTAG00000043315	2:125437144-125437270	0	0	0	OK
ENSBTAG00000043316	ENSBTAG00000043316	19:21192862-21192969	0	0	0	OK
ENSBTAG00000043317	ENSBTAG00000043317	11:6796439-6796525	0	0	0	OK
ENSBTAG00000043318	ENSBTAG00000043318	2:20305576-20305683	0	0	0	OK
ENSBTAG00000043319	ENSBTAG00000043319	16:74864353-74864471	0	0	0	OK
ENSBTAG00000043320	ENSBTAG00000043320	X:30406598-30406706	0	0	0	OK
ENSBTAG00000043322	ENSBTAG00000043322	20:39784710-39784817	0	0	0	OK
ENSBTAG00000043323	ENSBTAG00000043323	10:30914417-30914523	0	0	0	OK
ENSBTAG00000043324	ENSBTAG00000043324	28:7940875-7941010	0	0	0	OK
ENSBTAG00000043325	ENSBTAG00000043325	15:35944468-35944558	0	0	0	OK
ENSBTAG00000043327	ENSBTAG00000043327	10:69940618-69940750	0	0	0	OK
ENSBTAG00000043328	ENSBTAG00000043328	1:136990202-136990338	0	0	0	OK

ENSBTAG00000043329	ENSBTAG00000043329	17:46072722-46072853	0	0	0	OK
ENSBTAG00000043330	ENSBTAG00000043330	23:34951339-34951448	0	0	0	OK
ENSBTAG00000043331	ENSBTAG00000043331	9:67831508-67831614	0	0	0	OK
ENSBTAG00000043332	ENSBTAG00000043332	14:81603142-81603244	0	0	0	OK
ENSBTAG00000043333	ENSBTAG00000043333	6:47351712-47351816	0	0	0	OK
ENSBTAG00000043334	ENSBTAG00000043334	2:62150385-62150492	0	0	0	OK
ENSBTAG00000043335	ENSBTAG00000043335	14:64324525-64324632	0	0	0	OK
ENSBTAG00000043338	ENSBTAG00000043338	27:8947209-8947314	0	0	0	OK
ENSBTAG00000043339	ENSBTAG00000043339	19:56736294-56736397	0	0	0	OK
ENSBTAG00000043340	ENSBTAG00000043340	5:60411588-60411695	0	0	0	OK
ENSBTAG00000043341	ENSBTAG00000043341	11:104314557-104314622	0	0	0	OK
ENSBTAG00000043342	ENSBTAG00000043342	21:67487061-67487136	0	0	0	OK
ENSBTAG00000043343	ENSBTAG00000043343	12:80935414-80935519	0	0	0	OK
ENSBTAG00000043344	ENSBTAG00000043344	8:60925684-60925816	0	0	0	OK
ENSBTAG00000043347	ENSBTAG00000043347	X:39043978-39044110	0	0	0	OK
ENSBTAG00000043348	ENSBTAG00000043348	8:101307562-101307665	0	0	0	OK
ENSBTAG00000043349	ENSBTAG00000043349	3:100085316-100085388	0	0	0	OK
ENSBTAG00000043350	ENSBTAG00000043350	13:78104296-78104388	0	0	0	OK
ENSBTAG00000043353	ENSBTAG00000043353	3:28579774-28580078	0	0	0	OK
ENSBTAG00000043354	ENSBTAG00000043354	21:67494575-67494648	0	0	0	OK
ENSBTAG00000043356	ENSBTAG00000043356	10:6986543-6986659	0	0	0	OK
ENSBTAG00000043360	ENSBTAG00000043360	2:72279634-72279772	0	0	0	OK
ENSBTAG00000043361	ENSBTAG00000043361	18:62451475-62451579	0	0	0	OK
ENSBTAG00000043362	ENSBTAG00000043362	4:77353528-77353635	0	0	0	OK
ENSBTAG00000043364	ENSBTAG00000043364	X:145883944-145884049	0	0	0	OK
ENSBTAG00000043365	ENSBTAG00000043365	20:59258098-59258209	0	0	0	OK
ENSBTAG00000043366	ENSBTAG00000043366	6:45527602-45527709	0	0	0	OK
ENSBTAG00000043367	ENSBTAG00000043367	14:56072496-56072620	0	0	0	OK
ENSBTAG00000043368	ENSBTAG00000043368	7:1748889-1748996	0	0	0	OK
ENSBTAG00000043369	ENSBTAG00000043369	5:44710295-44710398	0	0	0	OK

ENSBTAG00000043370	ENSBTAG00000043370	16:19247161-19247268	0	0	0	OK
ENSBTAG00000043371	ENSBTAG00000043371	12:53164190-53164508	0	0	0	OK
ENSBTAG00000043372	ENSBTAG00000043372	10:53416858-53416962	0	0	0	OK
ENSBTAG00000043373	ENSBTAG00000043373	11:101635010-101635096	0	0	0	OK
ENSBTAG00000043374	ENSBTAG00000043374	11:12694360-12694467	0	0	0	OK
ENSBTAG00000043375	ENSBTAG00000043375	17:30376333-30376436	0	0	0	OK
ENSBTAG00000043376	ENSBTAG00000043376	1:70869303-70869383	0	0	0	OK
ENSBTAG00000043377	ENSBTAG00000043377	10:13321372-13321470	0	0	0	OK
ENSBTAG00000043378	ENSBTAG00000043378	16:56691272-56691349	0	0	0	OK
ENSBTAG00000043379	ENSBTAG00000043379	26:30591893-30592000	0	0	0	OK
ENSBTAG00000043380	ENSBTAG00000043380	2:125334329-125334436	0	0	0	OK
ENSBTAG00000043381	ENSBTAG00000043381	1:130704028-130704157	0	0	0	OK
ENSBTAG00000043382	ENSBTAG00000043382	26:13739035-13739113	0	0	0	OK
ENSBTAG00000043383	ENSBTAG00000043383	11:104312259-104312334	0	0	0	OK
ENSBTAG00000043385	ENSBTAG00000043385	4:72942644-72942751	0	0	0	OK
ENSBTAG00000043387	ENSBTAG00000043387	8:106605280-106605387	0	0	0	OK
ENSBTAG00000043388	ENSBTAG00000043388	19:13934855-13934972	0	0	0	OK
ENSBTAG00000043389	ENSBTAG00000043389	24:33361046-33361153	0	0	0	OK
ENSBTAG00000043390	ENSBTAG00000043390	17:61582100-61582207	0	0	0	OK
ENSBTAG00000043391	ENSBTAG00000043391	11:21594041-21594151	0	0	0	OK
ENSBTAG00000043392	ENSBTAG00000043392	23:27566646-27566725	0	0	0	OK
ENSBTAG00000043394	ENSBTAG00000043394	29:1066899-1067022	0	0	0	OK
ENSBTAG00000043395	ENSBTAG00000043395	8:23582947-23583055	0	0	0	OK
ENSBTAG00000043396	ENSBTAG00000043396	15:45632508-45632615	0	0	0	OK
ENSBTAG00000043398	ENSBTAG00000043398	4:77227280-77227416	0	0	0	OK
ENSBTAG00000043399	ENSBTAG00000043399	1:1367492-1367618	0	0	0	OK
ENSBTAG00000043400	ENSBTAG00000043400	24:62166070-62166177	0	0	0	OK
ENSBTAG00000043402	ENSBTAG00000043402	X:7847684-7847816	0	0	0	OK
ENSBTAG00000043403	ENSBTAG00000043403	8:17751660-17751767	0	0	0	OK
ENSBTAG00000043404	ENSBTAG00000043404	23:16194683-16194790	0	0	0	OK

ENSBTAG00000043405	ENSBTAG00000043405	X:128524419-128524523	0	0	0	OK
ENSBTAG00000043406	ENSBTAG00000043406	8:104406896-104407028	0	0	0	OK
ENSBTAG00000043407	ENSBTAG00000043407	9:53110946-53111053	0	0	0	OK
ENSBTAG00000043408	ENSBTAG00000043408	26:15444524-15444842	0	0	0	OK
ENSBTAG00000043409	ENSBTAG00000043409	3:22481974-22482081	0	0	0	OK
ENSBTAG00000043410	ENSBTAG00000043410	14:68662953-68663057	0	0	0	OK
ENSBTAG00000043411	ENSBTAG00000043411	4:23248710-23248813	0	0	0	OK
ENSBTAG00000043412	ENSBTAG00000043412	13:67989255-67989389	0	0	0	OK
ENSBTAG00000043413	ENSBTAG00000043413	21:67529222-67529297	0	0	0	OK
ENSBTAG00000043414	ENSBTAG00000043414	19:55824021-55824094	0	0	0	OK
ENSBTAG00000043415	ENSBTAG00000043415	26:15746439-15746542	0	0	0	OK
ENSBTAG00000043416	ENSBTAG00000043416	28:38764870-38764974	0	0	0	OK
ENSBTAG00000043417	ENSBTAG00000043417	21:67505259-67505337	0	0	0	OK
ENSBTAG00000043418	ENSBTAG00000043418	19:38715464-38715571	0	0	0	OK
ENSBTAG00000043419	ENSBTAG00000043419	7:15990337-15990441	0	0	0	OK
ENSBTAG00000043422	ENSBTAG00000043422	19:12980537-12980639	0	0	0	OK
ENSBTAG00000043423	ENSBTAG00000043423	23:19823238-19823345	0	0	0	OK
ENSBTAG00000043424	ENSBTAG00000043424	3:64144055-64144161	0	0	0	OK
ENSBTAG00000043425	ENSBTAG00000043425	16:33055762-33055890	0	0	0	OK
ENSBTAG00000043426	ENSBTAG00000043426	15:28379974-28380112	0	0	0	OK
ENSBTAG00000043427	ENSBTAG00000043427	22:13791670-13791777	0	0	0	OK
ENSBTAG00000043428	ENSBTAG00000043428	3:98857774-98857881	0	0	0	OK
ENSBTAG00000043430	ENSBTAG00000043430	8:2445171-2445296	0	0	0	OK
ENSBTAG00000043431	ENSBTAG00000043431	9:46775155-46775262	0	0	0	OK
ENSBTAG00000043432	ENSBTAG00000043432	16:56693108-56693169	0	0	0	OK
ENSBTAG00000043433	ENSBTAG00000043433	18:65657926-65658031	0	0	0	OK
ENSBTAG00000043434	ENSBTAG00000043434	7:40159616-40159719	0	0	0	OK
ENSBTAG00000043437	ENSBTAG00000043437	19:12591389-12591494	0	0	0	OK
ENSBTAG00000043438	ENSBTAG00000043438	17:5220659-5220766	0	0	0	OK
ENSBTAG00000043439	ENSBTAG00000043439	26:15337972-15338079	0	0	0	OK

ENSBTAG00000043440	ENSBTAG00000043440	21:43032509-43032616	0	0	0	OK
ENSBTAG00000043442	ENSBTAG00000043442	19:33813458-33813532	0	0	0	OK
ENSBTAG00000043443	ENSBTAG00000043443	4:534789-534896	0	0	0	OK
ENSBTAG00000043444	ENSBTAG00000043444	25:31327870-31327960	0	0	0	OK
ENSBTAG00000043445	ENSBTAG00000043445	26:8714321-8714425	0	0	0	OK
ENSBTAG00000043446	ENSBTAG00000043446	3:15691546-15691677	0	0	0	OK
ENSBTAG00000043447	ENSBTAG00000043447	3:69322441-69322524	0	0	0	OK
ENSBTAG00000043449	ENSBTAG00000043449	2:129669301-129669408	0	0	0	OK
ENSBTAG00000043450	ENSBTAG00000043450	21:2968964-2969067	0	0	0	OK
ENSBTAG00000043451	ENSBTAG00000043451	X:119854608-119854708	0	0	0	OK
ENSBTAG00000043452	ENSBTAG00000043452	1:98415990-98416097	0	0	0	OK
ENSBTAG00000043453	ENSBTAG00000043453	18:56397173-56397244	0	0	0	OK
ENSBTAG00000043454	ENSBTAG00000043454	28:22361589-22361695	0	0	0	OK
ENSBTAG00000043455	ENSBTAG00000043455	18:23903710-23903817	0	0	0	OK
ENSBTAG00000043458	ENSBTAG00000043458	27:29229224-29229550	0	0	0	OK
ENSBTAG00000043459	ENSBTAG00000043459	13:75595370-75595457	0	0	0	OK
ENSBTAG00000043460	ENSBTAG00000043460	7:47729752-47729858	0	0	0	OK
ENSBTAG00000043461	ENSBTAG00000043461	4:112970774-112970876	0	0	0	OK
ENSBTAG00000043462	ENSBTAG00000043462	15:34218135-34218223	0	0	0	OK
ENSBTAG00000043464	ENSBTAG00000043464	2:124915368-124915472	0	0	0	OK
ENSBTAG00000043465	ENSBTAG00000043465	21:22114782-22114889	0	0	0	OK
ENSBTAG00000043467	ENSBTAG00000043467	17:37256865-37256980	0	0	0	OK
ENSBTAG00000043468	ENSBTAG00000043468	9:64688983-64689053	0	0	0	OK
ENSBTAG00000043469	ENSBTAG00000043469	24:45528127-45528459	0	0	0	OK
ENSBTAG00000043471	ENSBTAG00000043471	5:87004658-87004987	0	0	0	OK
ENSBTAG00000043472	ENSBTAG00000043472	18:39455001-39455087	0	0	0	OK
ENSBTAG00000043473	ENSBTAG00000043473	8:19959635-19959745	0	0	0	OK
ENSBTAG00000043474	ENSBTAG00000043474	10:74432660-74432800	0	0	0	OK
ENSBTAG00000043475	ENSBTAG00000043475	10:19540357-19540464	0	0	0	OK
ENSBTAG00000043476	ENSBTAG00000043476	7:15560204-15560339	0	0	0	OK

ENSBTAG00000043477	ENSBTAG00000043477	18:45181562-45181668	0	0	0	OK
ENSBTAG00000043478	ENSBTAG00000043478	8:102117077-102117210	0	0	0	OK
ENSBTAG00000043480	ENSBTAG00000043480	15:34216817-34216901	0	0	0	OK
ENSBTAG00000043481	ENSBTAG00000043481	10:33970285-33970419	0	0	0	OK
ENSBTAG00000043482	ENSBTAG00000043482	22:45657967-45658271	0	0	0	OK
ENSBTAG00000043483	ENSBTAG00000043483	23:27571311-27571388	0	0	0	OK
ENSBTAG00000043484	ENSBTAG00000043484	5:28986875-28987006	0	0	0	OK
ENSBTAG00000043485	ENSBTAG00000043485	12:54200070-54200177	0	0	0	OK
ENSBTAG00000043486	ENSBTAG00000043486	16:53138749-53138853	0	0	0	OK
ENSBTAG00000043487	ENSBTAG00000043487	10:66349617-66349724	0	0	0	OK
ENSBTAG00000043488	ENSBTAG00000043488	1:9555604-9555711	0	0	0	OK
ENSBTAG00000043489	ENSBTAG00000043489	2:24026140-24026247	0	0	0	OK
ENSBTAG00000043490	ENSBTAG00000043490	21:67524992-67525064	0	0	0	OK
ENSBTAG00000043491	ENSBTAG00000043491	10:52386197-52386304	0	0	0	OK
ENSBTAG00000043492	ENSBTAG00000043492	6:76856520-76856616	0	0	0	OK
ENSBTAG00000043493	ENSBTAG00000043493	11:44828269-44828376	0	0	0	OK
ENSBTAG00000043496	ENSBTAG00000043496	27:4677301-4677407	0	0	0	OK
ENSBTAG00000043497	ENSBTAG00000043497	25:9254768-9254874	0	0	0	OK
ENSBTAG00000043498	ENSBTAG00000043498	14:49972277-49972385	0	0	0	OK
ENSBTAG00000043499	ENSBTAG00000043499	2:22530275-22530407	0	0	0	OK
ENSBTAG00000043502	ENSBTAG00000043502	5:111263670-111263764	0	0	0	OK
ENSBTAG00000043503	ENSBTAG00000043503	28:28378867-28378974	0	0	0	OK
ENSBTAG00000043505	ENSBTAG00000043505	2:40571289-40571396	0	0	0	OK
ENSBTAG00000043507	ENSBTAG00000043507	24:45690536-45690663	0	0	0	OK
ENSBTAG00000043508	ENSBTAG00000043508	19:28432320-28432456	0	0	0	OK
ENSBTAG00000043509	ENSBTAG00000043509	29:41854756-41854828	0	0	0	OK
ENSBTAG00000043511	ENSBTAG00000043511	14:78968453-78968579	0	0	0	OK
ENSBTAG00000043512	ENSBTAG00000043512	7:5206707-5206841	0	0	0	OK
ENSBTAG00000043514	ENSBTAG00000043514	2:120454392-120454526	0	0	0	OK
ENSBTAG00000043516	ENSBTAG00000043516	9:71329419-71329523	0	0	0	OK

ENSBTAG00000043517	ENSBTAG00000043517	4:70560497-70560604	0	0	0	OK
ENSBTAG00000043518	ENSBTAG00000043518	23:13900772-13900880	0	0	0	OK
ENSBTAG00000043519	ENSBTAG00000043519	4:31677006-31677079	0	0	0	OK
ENSBTAG00000043520	ENSBTAG00000043520	11:98050848-98050955	0	0	0	OK
ENSBTAG00000043524	ENSBTAG00000043524	7:46184614-46184721	0	0	0	OK
ENSBTAG00000043525	ENSBTAG00000043525	17:71099539-71099640	0	0	0	OK
ENSBTAG00000043526	ENSBTAG00000043526	23:34508695-34508804	0	0	0	OK
ENSBTAG00000043527	ENSBTAG00000043527	13:24181077-24181174	0	0	0	OK
ENSBTAG00000043528	ENSBTAG00000043528	18:26412043-26412182	0	0	0	OK
ENSBTAG00000043529	ENSBTAG00000043529	19:54811232-54811364	0	0	0	OK
ENSBTAG00000043530	ENSBTAG00000043530	6:8023995-8024126	0	0	0	OK
ENSBTAG00000043531	ENSBTAG00000043531	10:13320180-13320250	0	0	0	OK
ENSBTAG00000043532	ENSBTAG00000043532	2:60261792-60261899	0	0	0	OK
ENSBTAG00000043534	ENSBTAG00000043534	19:45632902-45633006	0	0	0	OK
ENSBTAG00000043540	ENSBTAG00000043540	15:43696404-43696511	0	0	0	OK
ENSBTAG00000043541	ENSBTAG00000043541	4:77227803-77227938	0	0	0	OK
ENSBTAG00000043542	ENSBTAG00000043542	9:13349086-13349189	0	0	0	OK
ENSBTAG00000043544	ENSBTAG00000043544	MT:11906-11976	0	0	0	OK
ENSBTAG00000043545	ENSBTAG00000043545	MT:7228-7299	0	0	5.83259	OK
ENSBTAG00000043547	ENSBTAG00000043547	MT:5375-5444	0	0	0	OK
ENSBTAG00000043548	ENSBTAG00000043548	MT:363-430	0	0	0	OK
ENSBTAG00000043549	ENSBTAG00000043549	MT:4196-4265	0	0	0	OK
ENSBTAG00000043551	ENSBTAG00000043551	MT:11976-12036	0	0	0	OK
ENSBTAG00000043552	ENSBTAG00000043552	MT:10169-10238	0	0	0	OK
ENSBTAG00000043554	ENSBTAG00000043554	MT:9753-9822	0	0	0	OK
ENSBTAG00000043555	ENSBTAG00000043555	MT:3023-3098	0	0	0	OK
ENSBTAG00000043556	ENSBTAG00000043556	MT:7373-8057	0	0	0	OK
ENSBTAG00000043557	ENSBTAG00000043557	MT:5550-5617	0	0	0	OK
ENSBTAG00000043562	ENSBTAG00000043562	MT:1385-1452	0	0	0	OK
ENSBTAG00000043565	ENSBTAG00000043565	MT:4122-4194	0	0	0	OK

ENSBTAG00000043566	ENSBTAG00000043566	MT:12037-12108	0	0	0	OK
ENSBTAG00000043567	ENSBTAG00000043567	MT:430-1385	0	0	0	OK
ENSBTAG00000043570	ENSBTAG00000043570	MT:1452-3023	0	0	0	OK
ENSBTAG00000043571	ENSBTAG00000043571	MT:4265-5307	0	0	0	OK
ENSBTAG00000043572	ENSBTAG00000043572	MT:15657-15726	0	0	0	OK
ENSBTAG00000043573	ENSBTAG00000043573	MT:5445-5518	0	0	0	OK
ENSBTAG00000043574	ENSBTAG00000043574	MT:7303-7372	0	0	0	OK
ENSBTAG00000043575	ENSBTAG00000043575	MT:4056-4125	0	0	0	OK
ENSBTAG00000043576	ENSBTAG00000043576	MT:8060-8127	0	0	0	OK
ENSBTAG00000043578	ENSBTAG00000043578	21:68150603-68151104	0	0	0	OK
ENSBTAG00000043579	ENSBTAG00000043579	MT:5307-5374	0	0	0	OK
ENSBTAG00000043580	ENSBTAG00000043580	MT:5617-5685	0	0	0	OK
ENSBTAG00000043582	ENSBTAG00000043582	MT:15725-15791	0	0	0	OK
ENSBTAG00000043583	ENSBTAG00000043583	MT:14440-14509	0	0	0	OK
ENSBTAG00000043587	ENSBTAG00000043587	1:104836506-104836612	0	0	0	OK
ENSBTAG00000043589	ENSBTAG00000043589	10:26825619-26825767	0	0	0	OK
ENSBTAG00000043590	ENSBTAG00000043590	3:16327517-16327654	0	0	0	OK
ENSBTAG00000043591	ENSBTAG00000043591	1:133222533-133222640	0	0	0	OK
ENSBTAG00000043592	ENSBTAG00000043592	20:34868076-34868255	0	0	0	OK
ENSBTAG00000043593	ENSBTAG00000043593	2:38731245-38731353	0	0	0	OK
ENSBTAG00000043594	ENSBTAG00000043594	17:41819281-41819393	0	0	0	OK
ENSBTAG00000043595	ENSBTAG00000043595	1:106376297-106376395	0	0	0	OK
ENSBTAG00000043599	ENSBTAG00000043599	21:2234418-2234498	0	0	0	OK
ENSBTAG00000043602	ENSBTAG00000043602	4:43796711-43797026	0	0	0	OK
ENSBTAG00000043604	ENSBTAG00000043604	7:68212766-68212867	0	0	0	OK
ENSBTAG00000043605	ENSBTAG00000043605	3:9089633-9089726	0	0	0	OK
ENSBTAG00000043606	ENSBTAG00000043606	7:54463501-54463639	0	0	0	OK
ENSBTAG00000043607	ENSBTAG00000043607	6:100450277-100450384	0	0	0	OK
ENSBTAG00000043609	ENSBTAG00000043609	11:64583016-64583121	0	0	0	OK
ENSBTAG00000043611	ENSBTAG00000043611	10:71111345-71111443	0	0	0	OK

ENSBTAG00000043612	ENSBTAG00000043612	24:23301995-23302360	0	0	0	OK
ENSBTAG00000043614	ENSBTAG00000043614	14:38493062-38493170	0	0	0	OK
ENSBTAG00000043615	ENSBTAG00000043615	8:39377714-39377817	0	0	0	OK
ENSBTAG00000043619	ENSBTAG00000043619	17:64436917-64437022	0	0	0	OK
ENSBTAG00000043620	ENSBTAG00000043620	6:20263199-20263284	0	0	0	OK
ENSBTAG00000043622	ENSBTAG00000043622	8:55270787-55270891	0	0	0	OK
ENSBTAG00000043625	ENSBTAG00000043625	10:32720822-32720898	0	0	0	OK
ENSBTAG00000043626	ENSBTAG00000043626	3:35149071-35149178	0	0	0	OK
ENSBTAG00000043627	ENSBTAG00000043627	6:81308204-81308310	0	0	0	OK
ENSBTAG00000043628	ENSBTAG00000043628	23:44035253-44035371	0	0	0	OK
ENSBTAG00000043632	ENSBTAG00000043632	X:126205166-126205270	0	0	0	OK
ENSBTAG00000043634	ENSBTAG00000043634	X:39927079-39927195	0	0	0	OK
ENSBTAG00000043636	ENSBTAG00000043636	10:31820028-31820161	0	0	0	OK
ENSBTAG00000043637	ENSBTAG00000043637	2:136045212-136045348	0	0	0	OK
ENSBTAG00000043639	ENSBTAG00000043639	5:38303246-38303571	0	0	0	OK
ENSBTAG00000043641	ENSBTAG00000043641	26:39601060-39601190	0	0	0	OK
ENSBTAG00000043642	ENSBTAG00000043642	7:8724960-8725015	0	0	0	OK
ENSBTAG00000043643	ENSBTAG00000043643	3:107486375-107486515	0	0	0	OK
ENSBTAG00000043644	ENSBTAG00000043644	28:9665773-9665886	0	0	0	OK
ENSBTAG00000043645	ENSBTAG00000043645	29:50534653-50534970	0	0	0	OK
ENSBTAG00000043647	ENSBTAG00000043647	X:91141061-91141158	0	0	0	OK
ENSBTAG00000043649	ENSBTAG00000043649	19:28790341-28790482	0	0	0	OK
ENSBTAG00000043651	ENSBTAG00000043651	8:43826047-43826141	0	0	0	OK
ENSBTAG00000043654	ENSBTAG00000043654	7:24030680-24030782	0	0	0	OK
ENSBTAG00000043655	ENSBTAG00000043655	23:39196639-39196756	0	0	0	OK
ENSBTAG00000043656	ENSBTAG00000043656	10:45469220-45469348	0	0	0	OK
ENSBTAG00000043657	ENSBTAG00000043657	6:31544854-31545141	0	0	0	OK
ENSBTAG00000043658	ENSBTAG00000043658	18:65601077-65601197	0	0	0	OK
ENSBTAG00000043660	ENSBTAG00000043660	15:29994077-29994167	0	0	0	OK
ENSBTAG00000043661	ENSBTAG00000043661	11:67419493-67419823	0	0	0	OK

ENSBTAG00000043662	ENSBTAG00000043662	7:68320752-68320892	0	0	0	OK
ENSBTAG00000043663	ENSBTAG00000043663	21:12866646-12866774	0	0	0	OK
ENSBTAG00000043664	ENSBTAG00000043664	19:19577331-19577455	0	0	0	OK
ENSBTAG00000043666	ENSBTAG00000043666	2:54543158-54543270	0	0	0	OK
ENSBTAG00000043668	ENSBTAG00000043668	9:103492667-103492748	0	0	0	OK
ENSBTAG00000043669	ENSBTAG00000043669	1:30990462-30990565	0	0	0	OK
ENSBTAG00000043670	ENSBTAG00000043670	21:67540632-67540707	0	0	0	OK
ENSBTAG00000043671	ENSBTAG00000043671	4:38487939-38488262	0	0	0	OK
ENSBTAG00000043673	ENSBTAG00000043673	5:99542007-99542250	0	0	0	OK
ENSBTAG00000043674	ENSBTAG00000043674	4:21534216-21534334	0	0	0	OK
ENSBTAG00000043675	ENSBTAG00000043675	2:26334406-26334514	0	0	0	OK
ENSBTAG00000043676	ENSBTAG00000043676	3:111019522-111019616	0	0	0	OK
ENSBTAG00000043677	ENSBTAG00000043677	27:44582786-44582891	0	0	0	OK
ENSBTAG00000043680	ENSBTAG00000043680	8:37092102-37092418	0	0	0	OK
ENSBTAG00000043683	ENSBTAG00000043683	18:45937115-45937204	0	0	0	OK
ENSBTAG00000043685	ENSBTAG00000043685	11:51321048-51321334	0	0	0	OK
ENSBTAG00000043686	ENSBTAG00000043686	14:22654043-22654320	0	0	0	OK
ENSBTAG00000043688	ENSBTAG00000043688	17:1172864-1173022	0	0	0	OK
ENSBTAG00000043690	ENSBTAG00000043690	17:72542794-72542877	0	0	0	OK
ENSBTAG00000043691	ENSBTAG00000043691	16:19094223-19094347	0	0	0	OK
ENSBTAG00000043692	ENSBTAG00000043692	X:61189175-61189282	0	0	0	OK
ENSBTAG00000043693	ENSBTAG00000043693	21:22611794-22611892	0	0	0	OK
ENSBTAG00000043694	ENSBTAG00000043694	21:67543570-67543644	0	0	0	OK
ENSBTAG00000043695	ENSBTAG00000043695	X:18178908-18178985	0	0	0	OK
ENSBTAG00000043696	ENSBTAG00000043696	X:3095788-3095916	0	0	0	OK
ENSBTAG00000043697	ENSBTAG00000043697	13:54375927-54376019	0	0	0	OK
ENSBTAG00000043698	ENSBTAG00000043698	22:60790444-60790742	0	0	0	OK
ENSBTAG00000043699	ENSBTAG00000043699	20:11849043-11849138	0	0	0	OK
ENSBTAG00000043700	ENSBTAG00000043700	11:78383547-78383654	0	0	0	OK
ENSBTAG00000043701	ENSBTAG00000043701	11:51276457-51276566	0	0	0	OK

ENSBTAG00000043702	ENSBTAG00000043702	8:67635227-67635310	0	0	0	OK
ENSBTAG00000043704	ENSBTAG00000043704	10:21543307-21543413	0	0	0	OK
ENSBTAG00000043706	ENSBTAG00000043706	15:64758866-64758947	0	0	0	OK
ENSBTAG00000043707	ENSBTAG00000043707	9:9719398-9719475	0	0	0	OK
ENSBTAG00000043708	ENSBTAG00000043708	13:70484898-70485002	0	0	0	OK
ENSBTAG00000043710	ENSBTAG00000043710	28:13949616-13949748	0	0	0	OK
ENSBTAG00000043712	ENSBTAG00000043712	11:24355691-24355809	0	0	0	OK
ENSBTAG00000043713	ENSBTAG00000043713	2:34184467-34184570	0	0	0	OK
ENSBTAG00000043716	ENSBTAG00000043716	10:89955225-89955308	0	0	0	OK
ENSBTAG00000043717	ENSBTAG00000043717	25:20969531-20969661	0	0	0	OK
ENSBTAG00000043719	ENSBTAG00000043719	18:35812391-35812519	0	0	0	OK
ENSBTAG00000043720	ENSBTAG00000043720	17:28632428-28632735	0	0	0	OK
ENSBTAG00000043721	ENSBTAG00000043721	23:38778768-38778906	0	0	0	OK
ENSBTAG00000043722	ENSBTAG00000043722	11:100958374-100958475	0	0	0	OK
ENSBTAG00000043723	ENSBTAG00000043723	27:39792750-39793060	0	0	0	OK
ENSBTAG00000043724	ENSBTAG00000043724	13:77049014-77049296	0	0	0	OK
ENSBTAG00000043725	ENSBTAG00000043725	23:51686147-51686257	0	0	0	OK
ENSBTAG00000043726	ENSBTAG00000043726	1:18963333-18963620	0	0	0	OK
ENSBTAG00000043729	ENSBTAG00000043729	23:28178035-28178111	0	0	0	OK
ENSBTAG00000043730	ENSBTAG00000043730	8:101643907-101644031	0	0	0	OK
ENSBTAG00000043732	ENSBTAG00000043732	10:86172178-86172364	0	0	0	OK
ENSBTAG00000043733	ENSBTAG00000043733	10:9094226-9094355	0	0	0	OK
ENSBTAG00000043734	ENSBTAG00000043734	7:85090719-85090826	0	0	0	OK
ENSBTAG00000043738	ENSBTAG00000043738	15:43689412-43689680	0	0	0	OK
ENSBTAG00000043739	ENSBTAG00000043739	2:102308566-102308692	0	0	0	OK
ENSBTAG00000043742	ENSBTAG00000043742	1:130552669-130552817	0	0	0	OK
ENSBTAG00000043744	ENSBTAG00000043744	6:45845699-45845802	0	0	0	OK
ENSBTAG00000043745	ENSBTAG00000043745	19:51988253-51988578	0	0	0	OK
ENSBTAG00000043746	ENSBTAG00000043746	9:59485092-59485415	0	0	0	OK
ENSBTAG00000043747	ENSBTAG00000043747	7:64078260-64078448	0	0	0	OK

ENSBTAG00000043750	ENSBTAG00000043750	19:16496345-16496452	0	0	0	OK
ENSBTAG00000043751	ENSBTAG00000043751	1:24817330-24817475	0	0	0	OK
ENSBTAG00000043753	ENSBTAG00000043753	4:117273498-117273594	0	0	0	OK
ENSBTAG00000043754	ENSBTAG00000043754	16:40692471-40692591	0	0	0	OK
ENSBTAG00000043755	ENSBTAG00000043755	12:88799763-88799872	0	0	0	OK
ENSBTAG00000043756	ENSBTAG00000043756	7:36393619-36393706	0	0	0	OK
ENSBTAG00000043758	ENSBTAG00000043758	20:34910495-34910591	0	0	0	OK
ENSBTAG00000043759	ENSBTAG00000043759	23:28893270-28893376	0	0	0	OK
ENSBTAG00000043760	ENSBTAG00000043760	3:82403864-82403999	0	0	0	OK
ENSBTAG00000043761	ENSBTAG00000043761	15:50835618-50835723	0	0	0	OK
ENSBTAG00000043764	ENSBTAG00000043764	28:9466709-9466823	0	0	0	OK
ENSBTAG00000043765	ENSBTAG00000043765	7:29199130-29199281	0	0	0	OK
ENSBTAG00000043766	ENSBTAG00000043766	8:66760045-66760149	0	0	0	OK
ENSBTAG00000043768	ENSBTAG00000043768	7:65665987-65666305	0	0	0	OK
ENSBTAG00000043769	ENSBTAG00000043769	17:18491499-18491606	0	0	0	OK
ENSBTAG00000043770	ENSBTAG00000043770	18:65015748-65015855	0	0	0	OK
ENSBTAG00000043771	ENSBTAG00000043771	24:58771044-58771144	0	0	0	OK
ENSBTAG00000043774	ENSBTAG00000043774	10:103345893-103346020	0	0	0	OK
ENSBTAG00000043775	ENSBTAG00000043775	20:41332246-41332328	0	0	0	OK
ENSBTAG00000043776	ENSBTAG00000043776	7:51510848-51510920	0	0	0	OK
ENSBTAG00000043778	ENSBTAG00000043778	7:13916771-13916843	0	0	0	OK
ENSBTAG00000043779	ENSBTAG00000043779	23:30161351-30161432	0	0	0	OK
ENSBTAG00000043781	ENSBTAG00000043781	19:10017664-10017778	0	0	0	OK
ENSBTAG00000043783	ENSBTAG00000043783	16:52099562-52099668	0	0	0	OK
ENSBTAG00000043784	ENSBTAG00000043784	29:28734153-28734468	0	0	0	OK
ENSBTAG00000043785	ENSBTAG00000043785	6:116016507-116016614	0	0	0	OK
ENSBTAG00000043786	ENSBTAG00000043786	19:23709234-23709342	0	0	0	OK
ENSBTAG00000043787	ENSBTAG00000043787	21:55903042-55903125	0	0	0	OK
ENSBTAG00000043789	ENSBTAG00000043789	7:45557260-45557342	0	0	5.05017	OK
ENSBTAG00000043791	ENSBTAG00000043791	7:35455092-35455198	0	0	0	OK

ENSBTAG00000043792	ENSBTAG00000043792	5:36510984-36511116	0	0	0	OK
ENSBTAG00000043793	ENSBTAG00000043793	3:99558233-99558349	0	0	0	OK
ENSBTAG00000043794	ENSBTAG00000043794	3:64875241-64875348	0	0	0	OK
ENSBTAG00000043795	ENSBTAG00000043795	13:47172816-47172885	0	0	0	OK
ENSBTAG00000043797	ENSBTAG00000043797	24:11009206-11009326	0	0	0	OK
ENSBTAG00000043798	ENSBTAG00000043798	19:21997718-21997876	0	0	0	OK
ENSBTAG00000043799	ENSBTAG00000043799	20:20653611-20653737	0	0	0	OK
ENSBTAG00000043800	ENSBTAG00000043800	17:6371340-6371447	0	0	0	OK
ENSBTAG00000043801	ENSBTAG00000043801	X:67814530-67814610	0	0	0	OK
ENSBTAG00000043802	ENSBTAG00000043802	1:124790930-124791039	0	0	0	OK
ENSBTAG00000043803	ENSBTAG00000043803	1:85947383-85947462	0	0	0	OK
ENSBTAG00000043804	ENSBTAG00000043804	4:69842057-69842339	0	0	0	OK
ENSBTAG00000043805	ENSBTAG00000043805	4:16928408-16928510	0	0	0	OK
ENSBTAG00000043806	ENSBTAG00000043806	17:30096343-30096524	0	0	0	OK
ENSBTAG00000043807	ENSBTAG00000043807	23:3166199-3166327	0	0	0	OK
ENSBTAG00000043808	ENSBTAG00000043808	9:17767954-17768268	0	0	0	OK
ENSBTAG00000043813	ENSBTAG00000043813	20:45050486-45050605	0	0	0	OK
ENSBTAG00000043814	ENSBTAG00000043814	15:58572779-58572893	0	0	0	OK
ENSBTAG00000043815	ENSBTAG00000043815	28:10204747-10204830	0	0	0	OK
ENSBTAG00000043816	ENSBTAG00000043816	10:50657424-50657551	0	0	0	OK
ENSBTAG00000043821	ENSBTAG00000043821	24:41213948-41214064	0	0	0	OK
ENSBTAG00000043822	ENSBTAG00000043822	9:70075396-70075542	0	0	0	OK
ENSBTAG00000043823	ENSBTAG00000043823	16:1060806-1060907	0	0	0	OK
ENSBTAG00000043825	ENSBTAG00000043825	X:67911306-67911386	0	0	0	OK
ENSBTAG00000043826	ENSBTAG00000043826	13:54338296-54338371	0	0	0	OK
ENSBTAG00000043827	ENSBTAG00000043827	5:15694026-15694135	0	0	0	OK
ENSBTAG00000043828	ENSBTAG00000043828	3:14749430-14749512	0	0	5.05017	OK
ENSBTAG00000043829	ENSBTAG00000043829	25:7961233-7961359	0	0	0	OK
ENSBTAG00000043830	ENSBTAG00000043830	4:92491706-92491998	0	0	0	OK
ENSBTAG00000043831	ENSBTAG00000043831	1:81856869-81856984	0	0	0	OK

ENSBTAG00000043832	ENSBTAG00000043832	7:73155273-73155413	0	0	0	OK
ENSBTAG00000043833	ENSBTAG00000043833	11:11660490-11660611	0	0	0	OK
ENSBTAG00000043834	ENSBTAG00000043834	X:79525739-79525906	0	0	0	OK
ENSBTAG00000043835	ENSBTAG00000043835	8:90998331-90998429	0	0	0	OK
ENSBTAG00000043836	ENSBTAG00000043836	5:97762964-97763067	0	0	0	OK
ENSBTAG00000043838	ENSBTAG00000043838	4:7324920-7325266	0	0	0	OK
ENSBTAG00000043840	ENSBTAG00000043840	2:21695247-21695354	0	0	0	OK
ENSBTAG00000043841	ENSBTAG00000043841	17:35170556-35170715	0	0	0	OK
ENSBTAG00000043843	ENSBTAG00000043843	9:41625276-41625382	0	0	0	OK
ENSBTAG00000043845	ENSBTAG00000043845	9:67307014-67307122	0	0	0	OK
ENSBTAG00000043846	ENSBTAG00000043846	9:41593111-41593241	0	0	0	OK
ENSBTAG00000043847	ENSBTAG00000043847	6:21985075-21985153	0	0	5.30915	OK
ENSBTAG00000043848	ENSBTAG00000043848	7:64051499-64051617	0	0	0	OK
ENSBTAG00000043850	ENSBTAG00000043850	17:52813384-52813637	0	0	0	OK
ENSBTAG00000043853	ENSBTAG00000043853	19:29708265-29708364	0	0	0	OK
ENSBTAG00000043854	ENSBTAG00000043854	8:60687377-60687531	0	0	0	OK
ENSBTAG00000043857	ENSBTAG00000043857	17:67452349-67452648	0	0	0	OK
ENSBTAG00000043859	ENSBTAG00000043859	21:67472566-67472637	0	0	0	OK
ENSBTAG00000043860	ENSBTAG00000043860	8:30858199-30858546	0	0	0	OK
ENSBTAG00000043862	ENSBTAG00000043862	2:27437823-27437934	0	0	0	OK
ENSBTAG00000043863	ENSBTAG00000043863	6:106113747-106113867	0	0	0	OK
ENSBTAG00000043864	ENSBTAG00000043864	2:123153219-123153301	0	0	0	OK
ENSBTAG00000043866	ENSBTAG00000043866	16:51556863-51556999	0	0	0	OK
ENSBTAG00000043867	ENSBTAG00000043867	10:42802647-42802775	0	0	0	OK
ENSBTAG00000043869	ENSBTAG00000043869	X:101844089-101844186	0	0	0	OK
ENSBTAG00000043870	ENSBTAG00000043870	X:84623893-84624016	0	0	0	OK
ENSBTAG00000043871	ENSBTAG00000043871	11:52476269-52476375	0	0	0	OK
ENSBTAG00000043872	ENSBTAG00000043872	1:97923986-97924166	0	0	0	OK
ENSBTAG00000043873	ENSBTAG00000043873	4:111494220-111494324	0	0	0	OK
ENSBTAG00000043874	ENSBTAG00000043874	16:11333162-11333315	0	0	0	OK

ENSBTAG00000043875	ENSBTAG00000043875	20:63120845-63120975	0	0	0	OK
ENSBTAG00000043876	ENSBTAG00000043876	3:54610766-54610931	0	0	0	OK
ENSBTAG00000043877	ENSBTAG00000043877	18:40291165-40291290	0	0	0	OK
ENSBTAG00000043879	ENSBTAG00000043879	15:7017348-7017465	0	0	0	OK
ENSBTAG00000043880	ENSBTAG00000043880	9:71432740-71432854	0	0	0	OK
ENSBTAG00000043883	ENSBTAG00000043883	X:45518604-45518685	0	0	0	OK
ENSBTAG00000043887	ENSBTAG00000043887	19:53572228-53572308	0	0	0	OK
ENSBTAG00000043888	ENSBTAG00000043888	X:72546576-72546705	0	0	0	OK
ENSBTAG00000043892	ENSBTAG00000043892	5:100491778-100492098	0	0	0	OK
ENSBTAG00000043893	ENSBTAG00000043893	24:22336668-22336783	0	0	0	OK
ENSBTAG00000043896	ENSBTAG00000043896	9:73931378-73931491	0	0	0	OK
ENSBTAG00000043897	ENSBTAG00000043897	18:26271610-26271711	0	0	0	OK
ENSBTAG00000043898	ENSBTAG00000043898	17:11854230-11854544	0	0	0	OK
ENSBTAG00000043899	ENSBTAG00000043899	1:116821878-116822010	0	0	0	OK
ENSBTAG00000043900	ENSBTAG00000043900	26:31096430-31096739	0	0	0	OK
ENSBTAG00000043903	ENSBTAG00000043903	5:12226289-12226409	0	0	0	OK
ENSBTAG00000043904	ENSBTAG00000043904	4:43820202-43820347	0	0	0	OK
ENSBTAG00000043905	ENSBTAG00000043905	4:53904094-53904231	0	0	0	OK
ENSBTAG00000043907	ENSBTAG00000043907	7:74190854-74190951	0	0	0	OK
ENSBTAG00000043909	ENSBTAG00000043909	20:70834694-70834808	0	0	0	OK
ENSBTAG00000043911	ENSBTAG00000043911	21:10798657-10798704	0	0	0	OK
ENSBTAG00000043914	ENSBTAG00000043914	22:48727548-48727624	0	0	0	OK
ENSBTAG00000043915	ENSBTAG00000043915	19:21491511-21491591	0	0	0	OK
ENSBTAG00000043917	ENSBTAG00000043917	23:47679961-47680063	0	0	0	OK
ENSBTAG00000043918	ENSBTAG00000043918	23:47337649-47337787	0	0	0	OK
ENSBTAG00000043919	ENSBTAG00000043919	8:33178578-33178676	0	0	0	OK
ENSBTAG00000043920	ENSBTAG00000043920	27:21750202-21750303	0	0	0	OK
ENSBTAG00000043923	ENSBTAG00000043923	14:25492089-25492184	0	0	0	OK
ENSBTAG00000043926	ENSBTAG00000043926	10:51885644-51885757	0	0	0	OK
ENSBTAG00000043927	ENSBTAG00000043927	7:63703332-63703441	0	0	0	OK

ENSBTAG00000043928	ENSBTAG00000043928	17:64226561-64226709	0	0	0	OK
ENSBTAG00000043929	ENSBTAG00000043929	1:17827696-17827803	0	0	0	OK
ENSBTAG00000043931	ENSBTAG00000043931	12:67470068-67470140	0	0	0	OK
ENSBTAG00000043932	ENSBTAG00000043932	22:56750173-56750299	0	0	0	OK
ENSBTAG00000043933	ENSBTAG00000043933	21:67534045-67534118	0	0	0	OK
ENSBTAG00000043935	ENSBTAG00000043935	17:53702534-53702849	0	0	0	OK
ENSBTAG00000043936	ENSBTAG00000043936	4:112931231-112931323	0	0	0	OK
ENSBTAG00000043938	ENSBTAG00000043938	14:19388493-19388652	0	0	0	OK
ENSBTAG00000043939	ENSBTAG00000043939	2:100738722-100739050	0	0	0	OK
ENSBTAG00000043940	ENSBTAG00000043940	14:83209931-83210074	0	0	0	OK
ENSBTAG00000043941	ENSBTAG00000043941	9:55916368-55916682	0	0	0	OK
ENSBTAG00000043942	ENSBTAG00000043942	6:96128404-96128509	0	0	0	OK
ENSBTAG00000043943	ENSBTAG00000043943	3:113842545-113842633	0	0	0	OK
ENSBTAG00000043944	ENSBTAG00000043944	21:19802391-19802679	0	0	0	OK
ENSBTAG00000043945	ENSBTAG00000043945	4:11941697-11941801	0	0	0	OK
ENSBTAG00000043946	ENSBTAG00000043946	11:11303148-11303443	0	0	0	OK
ENSBTAG00000043955	ENSBTAG00000043955	4:5660974-5724601	0	0	0	OK
ENSBTAG00000043957	ENSBTAG00000043957	1:148301329-148303092	0	0	0	OK
ENSBTAG00000043960	ENSBTAG00000043960	6:90842933-90985937	0	0	0	OK
ENSBTAG00000043964	ENSBTAG00000043964	13:20592074-20613350	0	0	0	OK
ENSBTAG00000043968	ENSBTAG00000043968	1:131326517-131326954	0	0	0	OK
ENSBTAG00000043975	ENSBTAG00000043975	9:90836601-90852725	0	0	0	OK
ENSBTAG00000043978	ENSBTAG00000043978	14:28538432-28539187	0	0	0	OK
ENSBTAG00000043983	ENSBTAG00000043983	8:17241723-17245917	0	0	0	OK
ENSBTAG00000043992	ENSBTAG00000043992	20:15075232-15320360	0	0	0	OK
ENSBTAG00000044000	ENSBTAG00000044000	5:57042182-57059973	0	0	0	OK
ENSBTAG00000044009	ENSBTAG00000044009	2:14502889-14623643	0	0	0	OK
ENSBTAG00000044010	ENSBTAG00000044010	20:28721654-28743621	0	0	0	OK
ENSBTAG00000044011	ENSBTAG00000044011	13:26026826-26035948	0	0	0	OK
ENSBTAG00000044012	ENSBTAG00000044012	4:28474601-28480333	0	0	0	OK

ENSBTAG00000044017	ENSBTAG00000044017	5:48563805-48743354	0	0	0	OK
ENSBTAG00000044018	ENSBTAG00000044018	7:17868240-17869206	0	0	0	OK
ENSBTAG00000044025	ENSBTAG00000044025	X:53319094-53332474	0	0	0	OK
ENSBTAG00000044029	ENSBTAG00000044029	10:28594115-28788420	0	0	0	OK
ENSBTAG00000044037	ENSBTAG00000044037	X:72745651-72755858	0	0	0	OK
ENSBTAG00000044040	ENSBTAG00000044040	25:37532739-37544696	0	0	0	OK
ENSBTAG00000044046	ENSBTAG00000044046	7:69585065-70027839	0	0	0	OK
ENSBTAG00000044065	ENSBTAG00000044065	19:49840493-49880949	0	0	0	OK
ENSBTAG00000044067	ENSBTAG00000044067	7:59288867-59309661	0	0	0	OK
ENSBTAG00000044068	ENSBTAG00000044068	4:5908622-6036680	0	0	0	OK
ENSBTAG00000044079	ENSBTAG00000044079	22:48842267-48845476	0	0	0	OK
ENSBTAG00000044080	ENSBTAG00000044080	14:33559383-33644209	0	0	0	OK
ENSBTAG00000044081	ENSBTAG00000044081	16:27058046-27058913	0	0	0	OK
ENSBTAG00000044093	ENSBTAG00000044093	2:121232761-121236649	0	0	0	OK
ENSBTAG00000044112	ENSBTAG00000044112	13:24392818-24417185	0	0	0	OK
ENSBTAG00000044138	ENSBTAG00000044138	19:55669705-55674680	0	0	0.657324	OK
ENSBTAG00000044139	ENSBTAG00000044139	27:16630108-16630996	0	0	0	OK
ENSBTAG00000044148	ENSBTAG00000044148	1:76666303-76675855	0	0	0	OK
ENSBTAG00000044150	ENSBTAG00000044150	3:3045780-3119590	0	0	0	OK
ENSBTAG00000044154	ENSBTAG00000044154	1:4257433-4257698	0	0	0	OK
ENSBTAG00000044175	ENSBTAG00000044175	20:13952310-13989063	0	0	0	OK
ENSBTAG00000044176	ENSBTAG00000044176	5:41609579-41673874	0	0	0	OK
ENSBTAG00000044183	ENSBTAG00000044183	7:61723860-61727921	0	0	0	OK
ENSBTAG00000044194	ENSBTAG00000044194	27:19073727-19108500	0	0	0	OK
ENSBTAG00000044208	ENSBTAG00000044208	27:24815380-24828237	0	0	0	OK
ENSBTAG00000044210	ENSBTAG00000044210	13:59349055-59363436	0	0	0	OK
ENSBTAG00000044212	ENSBTAG00000044212	24:29485847-29491222	0	0	0	OK
ENSBTAG00000044214	ENSBTAG00000044214	27:25707597-25707675	0	0	0	OK
ENSBTAG00000044215	ENSBTAG00000044215	8:39407225-39407302	0	0	0	OK
ENSBTAG00000044217	ENSBTAG00000044217	14:56717005-56717141	0	0	0	OK

ENSBTAG00000044218	ENSBTAG00000044218	11:43765946-43766054	0	0	0	OK
ENSBTAG00000044220	ENSBTAG00000044220	1:20511000-20511106	0	0	0	OK
ENSBTAG00000044221	ENSBTAG00000044221	20:56153007-56153138	0	0	0	OK
ENSBTAG00000044222	ENSBTAG00000044222	6:115567391-115567464	0	0	0	OK
ENSBTAG00000044223	ENSBTAG00000044223	9:63078829-63078899	0	0	0	OK
ENSBTAG00000044224	ENSBTAG00000044224	8:1172020-1172099	0	0	0	OK
ENSBTAG00000044225	ENSBTAG00000044225	18:48594019-48594085	0	0	0	OK
ENSBTAG00000044226	ENSBTAG00000044226	3:21142275-21142429	0	0	0	OK
ENSBTAG00000044227	ENSBTAG00000044227	5:21914177-21914251	0	0	0	OK
ENSBTAG00000044228	ENSBTAG00000044228	24:27484194-27484281	0	0	0	OK
ENSBTAG00000044229	ENSBTAG00000044229	20:7153585-7153720	0	0	0	OK
ENSBTAG00000044230	ENSBTAG00000044230	19:38259068-38259284	0	0	0	OK
ENSBTAG00000044231	ENSBTAG00000044231	22:1593416-1593522	0	0	0	OK
ENSBTAG00000044232	ENSBTAG00000044232	14:56969253-56969325	0	0	0	OK
ENSBTAG00000044233	ENSBTAG00000044233	22:47402829-47402923	0	0	0	OK
ENSBTAG00000044235	ENSBTAG00000044235	16:9815283-9815377	0	0	0	OK
ENSBTAG00000044236	ENSBTAG00000044236	12:29660159-29660238	0	0	0	OK
ENSBTAG00000044237	ENSBTAG00000044237	18:8655921-8656025	0	0	0	OK
ENSBTAG00000044240	ENSBTAG00000044240	13:53142237-53142313	0	0	0	OK
ENSBTAG00000044244	ENSBTAG00000044244	20:36470634-36470758	0	0	0	OK
ENSBTAG00000044246	ENSBTAG00000044246	7:59327870-59327939	0	0	0	OK
ENSBTAG00000044247	ENSBTAG00000044247	21:1594957-1595061	0	0	0	OK
ENSBTAG00000044248	ENSBTAG00000044248	2:26760049-26760156	0	0	0	OK
ENSBTAG00000044251	ENSBTAG00000044251	22:35483804-35483888	0	0	0	OK
ENSBTAG00000044253	ENSBTAG00000044253	6:20549887-20549970	0	0	0	OK
ENSBTAG00000044255	ENSBTAG00000044255	4:49798033-49798109	0	0	0	OK
ENSBTAG00000044256	ENSBTAG00000044256	11:20899157-20899228	0	0	0	OK
ENSBTAG00000044257	ENSBTAG00000044257	13:33672328-33672439	0	0	0	OK
ENSBTAG00000044258	ENSBTAG00000044258	10:12690726-12690806	0	0	0	OK
ENSBTAG00000044259	ENSBTAG00000044259	26:42262244-42262349	0	0	0	OK

ENSBTAG00000044260	ENSBTAG00000044260	8:25184207-25184341	0	0	0	OK
ENSBTAG00000044261	ENSBTAG00000044261	23:8321331-8321457	0	0	0	OK
ENSBTAG00000044262	ENSBTAG00000044262	29:44088086-44088159	0	0	0	OK
ENSBTAG00000044263	ENSBTAG00000044263	14:2071850-2071925	0	0	0	OK
ENSBTAG00000044264	ENSBTAG00000044264	4:44250983-44251099	0	0	0	OK
ENSBTAG00000044265	ENSBTAG00000044265	19:31973540-31973612	0	0	0	OK
ENSBTAG00000044266	ENSBTAG00000044266	X:77525238-77525329	0	0	0	OK
ENSBTAG00000044267	ENSBTAG00000044267	18:38894144-38894238	0	0	0	OK
ENSBTAG00000044269	ENSBTAG00000044269	19:49902107-49902218	0	0	0	OK
ENSBTAG00000044270	ENSBTAG00000044270	1:148908098-148908170	0	0	0	OK
ENSBTAG00000044271	ENSBTAG00000044271	7:136888-136976	0	0	0	OK
ENSBTAG00000044272	ENSBTAG00000044272	29:17373081-17373155	0	0	0	OK
ENSBTAG00000044273	ENSBTAG00000044273	10:82029038-82029143	0	0	0	OK
ENSBTAG00000044274	ENSBTAG00000044274	19:38413083-38413155	0	0	0	OK
ENSBTAG00000044275	ENSBTAG00000044275	12:15518104-15518233	0	0	0	OK
ENSBTAG00000044277	ENSBTAG00000044277	7:67372916-67372992	0	0	0	OK
ENSBTAG00000044278	ENSBTAG00000044278	1:112427937-112428061	0	0	0	OK
ENSBTAG00000044279	ENSBTAG00000044279	25:35187446-35187544	0	0	0	OK
ENSBTAG00000044280	ENSBTAG00000044280	18:1620737-1620808	0	0	0	OK
ENSBTAG00000044282	ENSBTAG00000044282	11:289867-289939	0	0	0	OK
ENSBTAG00000044283	ENSBTAG00000044283	23:11130055-11130306	0	0	0	OK
ENSBTAG00000044284	ENSBTAG00000044284	2:121518938-121519017	0	0	0	OK
ENSBTAG00000044285	ENSBTAG00000044285	16:36033832-36033896	0	0	0	OK
ENSBTAG00000044286	ENSBTAG00000044286	7:13109204-13109323	0	0	0	OK
ENSBTAG00000044287	ENSBTAG00000044287	19:44398561-44398629	0	0	0	OK
ENSBTAG00000044288	ENSBTAG00000044288	19:50931427-50931490	0	0	0	OK
ENSBTAG00000044289	ENSBTAG00000044289	10:10652218-10652346	0	0	0	OK
ENSBTAG00000044291	ENSBTAG00000044291	1:93280498-93280759	0	0	0	OK
ENSBTAG00000044293	ENSBTAG00000044293	9:22326482-22326550	0	0	0	OK
ENSBTAG00000044294	ENSBTAG00000044294	4:49304451-49304527	0	0	0	OK

ENSBTAG00000044295	ENSBTAG00000044295	7:77618756-77618864	0	0	0	OK
ENSBTAG00000044298	ENSBTAG00000044298	3:50819060-50819166	0	0	0	OK
ENSBTAG00000044299	ENSBTAG00000044299	1:3580209-3580286	0	0	0	OK
ENSBTAG00000044300	ENSBTAG00000044300	28:10826206-10826301	0	0	0	OK
ENSBTAG00000044301	ENSBTAG00000044301	20:3976542-3976615	0	0	0	OK
ENSBTAG00000044303	ENSBTAG00000044303	24:37318413-37318537	0	0	0	OK
ENSBTAG00000044304	ENSBTAG00000044304	12:91046172-91046251	0	0	0	OK
ENSBTAG00000044306	ENSBTAG00000044306	26:29357368-29357450	0	0	0	OK
ENSBTAG00000044307	ENSBTAG00000044307	18:42468714-42468817	0	0	0	OK
ENSBTAG00000044308	ENSBTAG00000044308	16:58628127-58628268	0	0	0	OK
ENSBTAG00000044309	ENSBTAG00000044309	4:75346003-75346141	0	0	0	OK
ENSBTAG00000044311	ENSBTAG00000044311	1:81062766-81062835	0	0	0	OK
ENSBTAG00000044315	ENSBTAG00000044315	11:97204070-97204440	0	0	0	OK
ENSBTAG00000044316	ENSBTAG00000044316	22:11405904-11405983	0	0	0	OK
ENSBTAG00000044317	ENSBTAG00000044317	6:2713196-2713253	0	0	0	OK
ENSBTAG00000044318	ENSBTAG00000044318	1:68426381-68426496	0	0	0	OK
ENSBTAG00000044319	ENSBTAG00000044319	20:21887967-21888085	0	0	0	OK
ENSBTAG00000044320	ENSBTAG00000044320	20:28572648-28572720	0	0	0	OK
ENSBTAG00000044322	ENSBTAG00000044322	15:66264847-66264925	0	0	0	OK
ENSBTAG00000044324	ENSBTAG00000044324	16:14336691-14336777	0	0	0	OK
ENSBTAG00000044325	ENSBTAG00000044325	7:106584577-106584684	0	0	0	OK
ENSBTAG00000044326	ENSBTAG00000044326	21:67459123-67459195	0	0	0	OK
ENSBTAG00000044327	ENSBTAG00000044327	7:103430194-103430296	0	0	0	OK
ENSBTAG00000044329	ENSBTAG00000044329	17:60646467-60646572	0	0	0	OK
ENSBTAG00000044330	ENSBTAG00000044330	21:67461532-67461604	0	0	0	OK
ENSBTAG00000044331	ENSBTAG00000044331	23:11060138-11060210	0	0	0	OK
ENSBTAG00000044332	ENSBTAG00000044332	22:56643352-56643456	0	0	0	OK
ENSBTAG00000044335	ENSBTAG00000044335	7:62949521-62949626	0	0	0	OK
ENSBTAG00000044336	ENSBTAG00000044336	8:62550678-62550749	0	0	0	OK
ENSBTAG00000044337	ENSBTAG00000044337	1:102829715-102830131	0	0	0	OK

ENSBTAG00000044339	ENSBTAG00000044339	5:57400204-57400281	0	0	0	OK
ENSBTAG00000044341	ENSBTAG00000044341	8:42817026-42817113	0	0	0	OK
ENSBTAG00000044343	ENSBTAG00000044343	1:61585761-61585985	0	0	0	OK
ENSBTAG00000044345	ENSBTAG00000044345	21:67444057-67444134	0	0	0	OK
ENSBTAG00000044346	ENSBTAG00000044346	1:110115004-110115068	0	0	0	OK
ENSBTAG00000044347	ENSBTAG00000044347	29:36494177-36494277	0	0	0	OK
ENSBTAG00000044348	ENSBTAG00000044348	8:907841-907899	0	0	0	OK
ENSBTAG00000044350	ENSBTAG00000044350	7:58004420-58004544	0	0	0	OK
ENSBTAG00000044351	ENSBTAG00000044351	10:13373030-13373162	0	0	0	OK
ENSBTAG00000044352	ENSBTAG00000044352	8:30260471-30260561	0	0	0	OK
ENSBTAG00000044354	ENSBTAG00000044354	6:16192076-16192184	0	0	0	OK
ENSBTAG00000044355	ENSBTAG00000044355	19:40516422-40516496	0	0	0	OK
ENSBTAG00000044361	ENSBTAG00000044361	2:86339907-86340032	0	0	0	OK
ENSBTAG00000044362	ENSBTAG00000044362	13:24932489-24932759	0	0	0	OK
ENSBTAG00000044363	ENSBTAG00000044363	5:56293170-56293243	0	0	0	OK
ENSBTAG00000044364	ENSBTAG00000044364	7:33278650-33278776	0	0	0	OK
ENSBTAG00000044365	ENSBTAG00000044365	6:51192006-51192105	0	0	0	OK
ENSBTAG00000044366	ENSBTAG00000044366	6:111785060-111785127	0	0	0	OK
ENSBTAG00000044367	ENSBTAG00000044367	2:114388637-114388715	0	0	0	OK
ENSBTAG00000044369	ENSBTAG00000044369	21:57742112-57742174	0	0	0	OK
ENSBTAG00000044370	ENSBTAG00000044370	11:85408710-85408763	0	0	0	OK
ENSBTAG00000044371	ENSBTAG00000044371	7:89154111-89154189	0	0	0	OK
ENSBTAG00000044373	ENSBTAG00000044373	21:26083904-26084001	0	0	0	OK
ENSBTAG00000044374	ENSBTAG00000044374	6:118328386-118328465	0	0	0	OK
ENSBTAG00000044375	ENSBTAG00000044375	2:134015864-134015952	0	0	0	OK
ENSBTAG00000044376	ENSBTAG00000044376	1:65144680-65144802	0	0	0	OK
ENSBTAG00000044377	ENSBTAG00000044377	5:44366490-44366579	0	0	0	OK
ENSBTAG00000044378	ENSBTAG00000044378	3:49095344-49095450	0	0	0	OK
ENSBTAG00000044379	ENSBTAG00000044379	10:15115567-15115681	0	0	0	OK
ENSBTAG00000044380	ENSBTAG00000044380	8:81563880-81564027	0	0	0	OK

ENSBTAG00000044381	ENSBTAG00000044381	10:103798514-103798616	0	0	0	OK
ENSBTAG00000044384	ENSBTAG00000044384	X:93637742-93637845	0	0	0	OK
ENSBTAG00000044386	ENSBTAG00000044386	22:43741295-43741374	0	0	0	OK
ENSBTAG00000044387	ENSBTAG00000044387	X:96874801-96874907	0	0	0	OK
ENSBTAG00000044389	ENSBTAG00000044389	3:77587679-77587761	0	0	0	OK
ENSBTAG00000044390	ENSBTAG00000044390	X:22738349-22738418	0	0	0	OK
ENSBTAG00000044391	ENSBTAG00000044391	X:140273347-140273476	0	0	0	OK
ENSBTAG00000044393	ENSBTAG00000044393	22:1992327-1992422	0	0	0	OK
ENSBTAG00000044394	ENSBTAG00000044394	19:11089937-11090017	0	0	0	OK
ENSBTAG00000044395	ENSBTAG00000044395	19:22824634-22824710	0	0	0	OK
ENSBTAG00000044396	ENSBTAG00000044396	6:85794658-85794760	0	0	0	OK
ENSBTAG00000044397	ENSBTAG00000044397	3:74551046-74551122	0	0	0	OK
ENSBTAG00000044398	ENSBTAG00000044398	10:65462366-65462440	0	0	0	OK
ENSBTAG00000044400	ENSBTAG00000044400	13:75111838-75111921	0	0	0	OK
ENSBTAG00000044401	ENSBTAG00000044401	19:18707013-18707208	0	0	0	OK
ENSBTAG00000044402	ENSBTAG00000044402	16:659396-659500	0	0	0	OK
ENSBTAG00000044405	ENSBTAG00000044405	4:15761228-15761287	0	0	0	OK
ENSBTAG00000044406	ENSBTAG00000044406	14:1883848-1883971	0	0	0	OK
ENSBTAG00000044407	ENSBTAG00000044407	3:21301095-21301258	0	0	0	OK
ENSBTAG00000044409	ENSBTAG00000044409	3:78825778-78825898	0	0	3.45095	OK
ENSBTAG00000044410	ENSBTAG00000044410	18:7789880-7789984	0	0	0	OK
ENSBTAG00000044412	ENSBTAG00000044412	24:49737879-49737949	0	0	0	OK
ENSBTAG00000044413	ENSBTAG00000044413	26:32098725-32098797	0	0	0	OK
ENSBTAG00000044414	ENSBTAG00000044414	13:30248051-30248120	0	0	0	OK
ENSBTAG00000044417	ENSBTAG00000044417	26:22391793-22391902	0	0	0	OK
ENSBTAG00000044418	ENSBTAG00000044418	21:24336670-24336752	0	0	5.05017	OK
ENSBTAG00000044419	ENSBTAG00000044419	2:118975647-118975746	0	0	0	OK
ENSBTAG00000044420	ENSBTAG00000044420	10:80097212-80097322	0	0	0	OK
ENSBTAG00000044422	ENSBTAG00000044422	7:51349700-51349770	0	0	0	OK
ENSBTAG00000044425	ENSBTAG00000044425	10:13199985-13200119	0	0	0	OK

ENSBTAG00000044426	ENSBTAG00000044426	18:48043146-48043216	0	0	0	OK
ENSBTAG00000044427	ENSBTAG00000044427	10:26814255-26814588	0	0	0	OK
ENSBTAG00000044428	ENSBTAG00000044428	17:53123180-53123278	0	0	0	OK
ENSBTAG00000044429	ENSBTAG00000044429	15:32050807-32050882	0	0	0	OK
ENSBTAG00000044430	ENSBTAG00000044430	3:34571921-34572054	0	0	0	OK
ENSBTAG00000044432	ENSBTAG00000044432	22:41695475-41695554	0	0	0	OK
ENSBTAG00000044433	ENSBTAG00000044433	17:1257098-1257213	0	0	0	OK
ENSBTAG00000044436	ENSBTAG00000044436	X:117482294-117482386	0	0	0	OK
ENSBTAG00000044438	ENSBTAG00000044438	14:15494282-15494310	0	0	0	OK
ENSBTAG00000044440	ENSBTAG00000044440	6:14822353-14822485	0	0	0	OK
ENSBTAG00000044441	ENSBTAG00000044441	2:126126019-126126185	0	0	0	OK
ENSBTAG00000044442	ENSBTAG00000044442	6:15710014-15710085	0	0	0	OK
ENSBTAG00000044443	ENSBTAG00000044443	19:55419631-55419819	0	0	0	OK
ENSBTAG00000044444	ENSBTAG00000044444	28:16048757-16048829	0	0	0	OK
ENSBTAG00000044446	ENSBTAG00000044446	16:12858248-12858340	0	0	0	OK
ENSBTAG00000044447	ENSBTAG00000044447	13:5748521-5748666	0	0	0	OK
ENSBTAG00000044449	ENSBTAG00000044449	5:118347308-118347375	0	0	0	OK
ENSBTAG00000044450	ENSBTAG00000044450	19:26741712-26741788	0	0	0	OK
ENSBTAG00000044451	ENSBTAG00000044451	5:112896829-112896899	0	0	0	OK
ENSBTAG00000044452	ENSBTAG00000044452	20:14354228-14354294	0	0	0	OK
ENSBTAG00000044453	ENSBTAG00000044453	4:113212666-113212975	0	0	0	OK
ENSBTAG00000044454	ENSBTAG00000044454	18:39351756-39351834	0	0	0	OK
ENSBTAG00000044456	ENSBTAG00000044456	13:59489949-59490018	0	0	0	OK
ENSBTAG00000044457	ENSBTAG00000044457	18:64484603-64484701	0	0	0	OK
ENSBTAG00000044458	ENSBTAG00000044458	14:48094676-48094754	0	0	0	OK
ENSBTAG00000044459	ENSBTAG00000044459	14:15792578-15792680	0	0	0	OK
ENSBTAG00000044461	ENSBTAG00000044461	X:89548252-89548339	0	0	0	OK
ENSBTAG00000044462	ENSBTAG00000044462	2:27629812-27629887	0	0	0	OK
ENSBTAG00000044464	ENSBTAG00000044464	15:24814538-24814642	0	0	0	OK
ENSBTAG00000044465	ENSBTAG00000044465	8:59737059-59737139	0	0	0	OK

ENSBTAG00000044466	ENSBTAG00000044466	25:34073887-34074016	0	0	0	OK
ENSBTAG00000044467	ENSBTAG00000044467	5:90112819-90112889	0	0	0	OK
ENSBTAG00000044469	ENSBTAG00000044469	11:37182358-37182428	0	0	0	OK
ENSBTAG00000044470	ENSBTAG00000044470	19:50423905-50423966	0	0	0	OK
ENSBTAG00000044473	ENSBTAG00000044473	8:5672115-5672188	0	0	0	OK
ENSBTAG00000044474	ENSBTAG00000044474	3:12933979-12934049	0	0	0	OK
ENSBTAG00000044475	ENSBTAG00000044475	18:3975992-3976055	0	0	0	OK
ENSBTAG00000044476	ENSBTAG00000044476	9:54215268-54215394	0	0	0	OK
ENSBTAG00000044477	ENSBTAG00000044477	6:112441111-112441219	0	0	0	OK
ENSBTAG00000044478	ENSBTAG00000044478	5:113634244-113634320	0	0	0	OK
ENSBTAG00000044480	ENSBTAG00000044480	4:27944181-27944266	0	0	0	OK
ENSBTAG00000044481	ENSBTAG00000044481	25:17005110-17005184	0	0	0	OK
ENSBTAG00000044482	ENSBTAG00000044482	23:19281044-19281151	0	0	0	OK
ENSBTAG00000044483	ENSBTAG00000044483	11:48422106-48422245	0	0	0	OK
ENSBTAG00000044485	ENSBTAG00000044485	8:74339194-74339301	0	0	0	OK
ENSBTAG00000044487	ENSBTAG00000044487	7:80345573-80345719	0	0	0	OK
ENSBTAG00000044489	ENSBTAG00000044489	8:102208754-102208824	0	0	0	OK
ENSBTAG00000044490	ENSBTAG00000044490	5:57937674-57937828	0	0	0	OK
ENSBTAG00000044491	ENSBTAG00000044491	2:49852589-49852696	0	0	0	OK
ENSBTAG00000044492	ENSBTAG00000044492	18:62670080-62670269	0	0	2.19108	OK
ENSBTAG00000044493	ENSBTAG00000044493	11:26395433-26395548	0	0	0	OK
ENSBTAG00000044495	ENSBTAG00000044495	23:5598009-5598050	0	0	0	OK
ENSBTAG00000044496	ENSBTAG00000044496	20:29416726-29416828	0	0	0	OK
ENSBTAG00000044497	ENSBTAG00000044497	16:75828986-75829096	0	0	0	OK
ENSBTAG00000044498	ENSBTAG00000044498	27:1684520-1684624	0	0	0	OK
ENSBTAG00000044499	ENSBTAG00000044499	4:98069973-98070046	0	0	0	OK
ENSBTAG00000044500	ENSBTAG00000044500	20:63769783-63769853	0	0	0	OK
ENSBTAG00000044501	ENSBTAG00000044501	22:40172532-40172602	0	0	0	OK
ENSBTAG00000044502	ENSBTAG00000044502	23:41012112-41012183	0	0	0	OK
ENSBTAG00000044503	ENSBTAG00000044503	X:107160808-107160904	0	0	0	OK

ENSBTAG00000044504	ENSBTAG00000044504	17:29254582-29254676	0	0	0	OK
ENSBTAG00000044506	ENSBTAG00000044506	X:60366503-60366591	0	0	0	OK
ENSBTAG00000044507	ENSBTAG00000044507	16:62860054-62860148	0	0	0	OK
ENSBTAG00000044508	ENSBTAG00000044508	18:65740116-65740218	0	0	0	OK
ENSBTAG00000044509	ENSBTAG00000044509	15:52321936-52322008	0	0	0	OK
ENSBTAG00000044510	ENSBTAG00000044510	1:47097434-47097548	0	0	0	OK
ENSBTAG00000044512	ENSBTAG00000044512	13:76536638-76536785	0	0	0	OK
ENSBTAG00000044513	ENSBTAG00000044513	19:44003220-44003300	0	0	0	OK
ENSBTAG00000044514	ENSBTAG00000044514	12:11277715-11277838	0	0	0	OK
ENSBTAG00000044515	ENSBTAG00000044515	15:53094798-53094872	0	0	0	OK
ENSBTAG00000044517	ENSBTAG00000044517	18:52699616-52699737	0	0	0	OK
ENSBTAG00000044518	ENSBTAG00000044518	10:51826399-51826528	0	0	0	OK
ENSBTAG00000044519	ENSBTAG00000044519	1:153891168-153891255	0	0	0	OK
ENSBTAG00000044520	ENSBTAG00000044520	1:54406682-54406783	0	0	0	OK
ENSBTAG00000044521	ENSBTAG00000044521	16:42084904-42085048	0	0	0	OK
ENSBTAG00000044522	ENSBTAG00000044522	7:18379979-18380037	0	0	0	OK
ENSBTAG00000044523	ENSBTAG00000044523	8:107024953-107025034	0	0	0	OK
ENSBTAG00000044524	ENSBTAG00000044524	29:33306277-33306349	0	0	0	OK
ENSBTAG00000044525	ENSBTAG00000044525	19:20463273-20463373	0	0	0	OK
ENSBTAG00000044526	ENSBTAG00000044526	3:9881757-9881830	0	0	0	OK
ENSBTAG00000044528	ENSBTAG00000044528	25:16618106-16618216	0	0	0	OK
ENSBTAG00000044530	ENSBTAG00000044530	19:15745578-15745700	0	0	0	OK
ENSBTAG00000044532	ENSBTAG00000044532	11:45640369-45640481	0	0	0	OK
ENSBTAG00000044535	ENSBTAG00000044535	1:73230875-73230948	0	0	0	OK
ENSBTAG00000044536	ENSBTAG00000044536	18:65899432-65899580	0	0	0	OK
ENSBTAG00000044537	ENSBTAG00000044537	13:34110816-34110942	0	0	0	OK
ENSBTAG00000044538	ENSBTAG00000044538	19:43627212-43627331	0	0	0	OK
ENSBTAG00000044539	ENSBTAG00000044539	6:73511078-73511139	0	0	0	OK
ENSBTAG00000044540	ENSBTAG00000044540	3:42786017-42786224	0	0	0	OK
ENSBTAG00000044541	ENSBTAG00000044541	22:34887598-34887671	0	0	0	OK

ENSBTAG00000044543	ENSBTAG00000044543	22:15881962-15882043	0	0	0	OK
ENSBTAG00000044544	ENSBTAG00000044544	4:13552299-13552431	0	0	0	OK
ENSBTAG00000044545	ENSBTAG00000044545	11:20980213-20980310	0	0	0	OK
ENSBTAG00000044546	ENSBTAG00000044546	14:396451-396519	0	0	0	OK
ENSBTAG00000044547	ENSBTAG00000044547	21:62871947-62872045	0	0	0	OK
ENSBTAG00000044548	ENSBTAG00000044548	4:47367035-47367106	0	0	0	OK
ENSBTAG00000044550	ENSBTAG00000044550	23:28601332-28601408	0	0	0	OK
ENSBTAG00000044551	ENSBTAG00000044551	21:61304010-61304084	0	0	0	OK
ENSBTAG00000044552	ENSBTAG00000044552	25:34226933-34227082	0	0	0	OK
ENSBTAG00000044553	ENSBTAG00000044553	11:73158303-73158383	0	0	0	OK
ENSBTAG00000044554	ENSBTAG00000044554	12:4035643-4035735	0	0	0	OK
ENSBTAG00000044555	ENSBTAG00000044555	6:118433706-118433861	0	0	0	OK
ENSBTAG00000044557	ENSBTAG00000044557	10:66059707-66059800	0	0	0	OK
ENSBTAG00000044558	ENSBTAG00000044558	9:76799261-76799536	0	0	0	OK
ENSBTAG00000044560	ENSBTAG00000044560	8:18910427-18910519	0	0	0	OK
ENSBTAG00000044561	ENSBTAG00000044561	23:35647119-35647199	0	0	0	OK
ENSBTAG00000044563	ENSBTAG00000044563	10:104029106-104029173	0	0	0	OK
ENSBTAG00000044564	ENSBTAG00000044564	27:34944420-34944485	0	0	0	OK
ENSBTAG00000044565	ENSBTAG00000044565	12:53250954-53251022	0	0	0	OK
ENSBTAG00000044566	ENSBTAG00000044566	13:23417725-23417802	0	0	0	OK
ENSBTAG00000044567	ENSBTAG00000044567	20:23537896-23537983	0	0	0	OK
ENSBTAG00000044568	ENSBTAG00000044568	19:23234715-23234816	0	0	0	OK
ENSBTAG00000044570	ENSBTAG00000044570	25:5437422-5437524	0	0	0	OK
ENSBTAG00000044571	ENSBTAG00000044571	9:22987355-22987510	0	0	0	OK
ENSBTAG00000044572	ENSBTAG00000044572	3:18845836-18845914	0	0	0	OK
ENSBTAG00000044573	ENSBTAG00000044573	11:32132044-32132141	0	0	0	OK
ENSBTAG00000044574	ENSBTAG00000044574	X:88113516-88113622	0	0	0	OK
ENSBTAG00000044575	ENSBTAG00000044575	6:20550045-20550160	0	0	0	OK
ENSBTAG00000044576	ENSBTAG00000044576	5:40662179-40662257	0	0	0	OK
ENSBTAG00000044578	ENSBTAG00000044578	2:67201178-67201255	0	0	0	OK

ENSBTAG00000044579	ENSBTAG00000044579	9:25474383-25474444	0	0	0	OK
ENSBTAG00000044580	ENSBTAG00000044580	25:35300127-35300206	0	0	0	OK
ENSBTAG00000044581	ENSBTAG00000044581	9:23397953-23398013	0	0	0	OK
ENSBTAG00000044582	ENSBTAG00000044582	23:14325178-14325285	0	0	0	OK
ENSBTAG00000044583	ENSBTAG00000044583	10:46360718-46360787	0	0	0	OK
ENSBTAG00000044584	ENSBTAG00000044584	X:63983918-63983995	0	0	0	OK
ENSBTAG00000044585	ENSBTAG00000044585	4:63430382-63430457	0	0	0	OK
ENSBTAG00000044586	ENSBTAG00000044586	22:13593172-13593436	0	0	0	OK
ENSBTAG00000044587	ENSBTAG00000044587	21:23130018-23130111	0	0	0	OK
ENSBTAG00000044588	ENSBTAG00000044588	2:101691541-101691619	0	0	0	OK
ENSBTAG00000044589	ENSBTAG00000044589	15:34628900-34628974	0	0	0	OK
ENSBTAG00000044590	ENSBTAG00000044590	11:11316130-11316193	0	0	0	OK
ENSBTAG00000044591	ENSBTAG00000044591	13:78741826-78741957	0	0	0	OK
ENSBTAG00000044592	ENSBTAG00000044592	5:27772778-27772847	0	0	0	OK
ENSBTAG00000044593	ENSBTAG00000044593	15:78167891-78167962	0	0	0	OK
ENSBTAG00000044594	ENSBTAG00000044594	X:92897951-92898034	0	0	0	OK
ENSBTAG00000044595	ENSBTAG00000044595	X:125623601-125623731	0	0	0	OK
ENSBTAG00000044597	ENSBTAG00000044597	18:21207310-21207418	0	0	0	OK
ENSBTAG00000044598	ENSBTAG00000044598	1:17600001-17600086	0	0	0	OK
ENSBTAG00000044599	ENSBTAG00000044599	2:96389753-96389847	0	0	0	OK
ENSBTAG00000044600	ENSBTAG00000044600	3:34569127-34569463	0	0	0	OK
ENSBTAG00000044601	ENSBTAG00000044601	19:7336968-7337037	0	0	0	OK
ENSBTAG00000044602	ENSBTAG00000044602	12:14450799-14450840	0	0	0	OK
ENSBTAG00000044603	ENSBTAG00000044603	1:42315900-42316003	0	0	0	OK
ENSBTAG00000044604	ENSBTAG00000044604	10:36862417-36862608	0	0	0	OK
ENSBTAG00000044606	ENSBTAG00000044606	25:1744907-1744984	0	0	0	OK
ENSBTAG00000044607	ENSBTAG00000044607	22:52249301-52249410	0	0	0	OK
ENSBTAG00000044608	ENSBTAG00000044608	26:31325372-31325440	0	0	0	OK
ENSBTAG00000044611	ENSBTAG00000044611	3:55877345-55877451	0	0	0	OK
ENSBTAG00000044614	ENSBTAG00000044614	6:109824824-109824948	0	0	0	OK

ENSBTAG00000044615	ENSBTAG00000044615	9:83978004-83978137	0	0	0	OK
ENSBTAG00000044616	ENSBTAG00000044616	14:22646986-22647093	0	0	0	OK
ENSBTAG00000044617	ENSBTAG00000044617	4:67916862-67916968	0	0	0	OK
ENSBTAG00000044618	ENSBTAG00000044618	19:3899299-3899416	0	0	0	OK
ENSBTAG00000044619	ENSBTAG00000044619	16:79548701-79548808	0	0	0	OK
ENSBTAG00000044620	ENSBTAG00000044620	26:49743765-49743839	0	0	0	OK
ENSBTAG00000044621	ENSBTAG00000044621	4:62553332-62553439	0	0	0	OK
ENSBTAG00000044622	ENSBTAG00000044622	11:10862185-10862252	0	0	0	OK
ENSBTAG00000044623	ENSBTAG00000044623	5:101542230-101542293	0	0	0	OK
ENSBTAG00000044625	ENSBTAG00000044625	27:39897912-39897987	0	0	0	OK
ENSBTAG00000044626	ENSBTAG00000044626	29:22408993-22409065	0	0	0	OK
ENSBTAG00000044628	ENSBTAG00000044628	19:63467705-63467782	0	0	0	OK
ENSBTAG00000044630	ENSBTAG00000044630	2:106733738-106733811	0	0	0	OK
ENSBTAG00000044631	ENSBTAG00000044631	3:113615087-113615349	0	0	0	OK
ENSBTAG00000044632	ENSBTAG00000044632	10:66315115-66315185	0	0	0	OK
ENSBTAG00000044633	ENSBTAG00000044633	8:11390417-11390484	0	0	0	OK
ENSBTAG00000044635	ENSBTAG00000044635	X:85693324-85693403	0	0	0	OK
ENSBTAG00000044636	ENSBTAG00000044636	5:44913052-44913159	0	0	0	OK
ENSBTAG00000044637	ENSBTAG00000044637	28:24991759-24991835	0	0	0	OK
ENSBTAG00000044640	ENSBTAG00000044640	6:62343690-62343767	0	0	0	OK
ENSBTAG00000044642	ENSBTAG00000044642	19:22228106-22228213	0	0	0	OK
ENSBTAG00000044643	ENSBTAG00000044643	19:51516010-51516089	0	0	0	OK
ENSBTAG00000044645	ENSBTAG00000044645	21:67464184-67464256	0	0	0	OK
ENSBTAG00000044646	ENSBTAG00000044646	6:4615138-4615243	0	0	0	OK
ENSBTAG00000044649	ENSBTAG00000044649	6:107675829-107675896	0	0	0	OK
ENSBTAG00000044651	ENSBTAG00000044651	9:61686480-61686571	0	0	0	OK
ENSBTAG00000044653	ENSBTAG00000044653	18:5080336-5080492	0	0	0	OK
ENSBTAG00000044654	ENSBTAG00000044654	7:67867801-67867895	0	0	0	OK
ENSBTAG00000044657	ENSBTAG00000044657	3:101831088-101831187	0	0	0	OK
ENSBTAG00000044658	ENSBTAG00000044658	2:81124643-81124730	0	0	0	OK

ENSBTAG00000044659	ENSBTAG00000044659	21:61984641-61984915	0	0	0	OK
ENSBTAG00000044660	ENSBTAG00000044660	17:68738323-68738418	0	0	0	OK
ENSBTAG00000044661	ENSBTAG00000044661	15:40356860-40356921	0	0	0	OK
ENSBTAG00000044663	ENSBTAG00000044663	5:69712508-69712643	0	0	0	OK
ENSBTAG00000044664	ENSBTAG00000044664	4:88623862-88623925	0	0	0	OK
ENSBTAG00000044665	ENSBTAG00000044665	18:1561037-1561150	0	0	0	OK
ENSBTAG00000044666	ENSBTAG00000044666	X:132302881-132302952	0	0	0	OK
ENSBTAG00000044667	ENSBTAG00000044667	1:113337318-113337455	0	0	0	OK
ENSBTAG00000044668	ENSBTAG00000044668	1:59042657-59042756	0	0	0	OK
ENSBTAG00000044669	ENSBTAG00000044669	7:34859537-34859644	0	0	0	OK
ENSBTAG00000044670	ENSBTAG00000044670	10:59950255-59950342	0	0	0	OK
ENSBTAG00000044671	ENSBTAG00000044671	20:68416884-68416988	0	0	0	OK
ENSBTAG00000044673	ENSBTAG00000044673	4:15091619-15091724	0	0	0	OK
ENSBTAG00000044674	ENSBTAG00000044674	19:47914712-47914789	0	0	0	OK
ENSBTAG00000044675	ENSBTAG00000044675	8:62600140-62600199	0	0	0	OK
ENSBTAG00000044676	ENSBTAG00000044676	5:111047103-111047210	0	0	0	OK
ENSBTAG00000044677	ENSBTAG00000044677	15:60877670-60877942	0	0	0	OK
ENSBTAG00000044678	ENSBTAG00000044678	24:33897668-33897742	0	0	0	OK
ENSBTAG00000044679	ENSBTAG00000044679	X:125429932-125430026	0	0	0	OK
ENSBTAG00000044680	ENSBTAG00000044680	5:41964061-41964126	0	0	0	OK
ENSBTAG00000044684	ENSBTAG00000044684	2:18651775-18651847	0	0	0	OK
ENSBTAG00000044687	ENSBTAG00000044687	24:35132241-35132413	0	0	0	OK
ENSBTAG00000044688	ENSBTAG00000044688	13:73795112-73795235	0	0	0	OK
ENSBTAG00000044689	ENSBTAG00000044689	7:106419499-106419595	0	0	0	OK
ENSBTAG00000044690	ENSBTAG00000044690	13:68394367-68394502	0	0	0	OK
ENSBTAG00000044691	ENSBTAG00000044691	10:15665208-15665484	0	0	0	OK
ENSBTAG00000044694	ENSBTAG00000044694	28:39005578-39005681	0	0	0	OK
ENSBTAG00000044695	ENSBTAG00000044695	6:107198999-107199075	0	0	0	OK
ENSBTAG00000044696	ENSBTAG00000044696	5:6580064-6580174	0	0	0	OK
ENSBTAG00000044697	ENSBTAG00000044697	4:106200335-106200408	0	0	0	OK

ENSBTAG00000044698	ENSBTAG00000044698	13:24073636-24073742	0	0	0	OK
ENSBTAG00000044699	ENSBTAG00000044699	16:44211333-44211439	0	0	0	OK
ENSBTAG00000044700	ENSBTAG00000044700	1:68965794-68965945	0	0	0	OK
ENSBTAG00000044702	ENSBTAG00000044702	26:42482648-42482717	0	0	0	OK
ENSBTAG00000044703	ENSBTAG00000044703	17:22084815-22084943	0	0	0	OK
ENSBTAG00000044704	ENSBTAG00000044704	10:14673372-14673507	0	0	0	OK
ENSBTAG00000044706	ENSBTAG00000044706	10:70122669-70122796	0	0	0	OK
ENSBTAG00000044708	ENSBTAG00000044708	23:13082432-13082501	0	0	0	OK
ENSBTAG00000044709	ENSBTAG00000044709	21:21157503-21157577	0	0	0	OK
ENSBTAG00000044711	ENSBTAG00000044711	14:31487738-31487823	0	0	0	OK
ENSBTAG00000044712	ENSBTAG00000044712	25:2988662-2988738	0	0	0	OK
ENSBTAG00000044713	ENSBTAG00000044713	3:88672258-88672352	0	0	0	OK
ENSBTAG00000044714	ENSBTAG00000044714	28:35311134-35311208	0	0	0	OK
ENSBTAG00000044715	ENSBTAG00000044715	3:16078447-16078515	0	0	0	OK
ENSBTAG00000044716	ENSBTAG00000044716	3:112907946-112908051	0	0	0	OK
ENSBTAG00000044717	ENSBTAG00000044717	17:53770212-53770282	0	0	0	OK
ENSBTAG00000044719	ENSBTAG00000044719	3:106636945-106637092	0	0	0	OK
ENSBTAG00000044720	ENSBTAG00000044720	6:103487841-103487912	0	0	0	OK
ENSBTAG00000044721	ENSBTAG00000044721	17:54442059-54442171	0	0	0	OK
ENSBTAG00000044722	ENSBTAG00000044722	25:15950863-15950951	0	0	0	OK
ENSBTAG00000044723	ENSBTAG00000044723	11:55128046-55128147	0	0	0	OK
ENSBTAG00000044724	ENSBTAG00000044724	18:42439917-42440035	0	0	0	OK
ENSBTAG00000044726	ENSBTAG00000044726	26:15882416-15882654	0	0	0	OK
ENSBTAG00000044727	ENSBTAG00000044727	4:57048250-57048355	0	0	0	OK
ENSBTAG00000044730	ENSBTAG00000044730	14:19598493-19598607	0	0	0	OK
ENSBTAG00000044732	ENSBTAG00000044732	17:6408080-6408200	0	0	0	OK
ENSBTAG00000044733	ENSBTAG00000044733	14:52592734-52592833	0	0	0	OK
ENSBTAG00000044734	ENSBTAG00000044734	22:27052396-27052495	0	0	0	OK
ENSBTAG00000044735	ENSBTAG00000044735	23:34814415-34814524	0	0	0	OK
ENSBTAG00000044736	ENSBTAG00000044736	5:30952152-30952226	0	0	0	OK

ENSBTAG00000044737	ENSBTAG00000044737	1:103256444-103256543	0	0	0	OK
ENSBTAG00000044738	ENSBTAG00000044738	4:62009853-62009958	0	0	0	OK
ENSBTAG00000044740	ENSBTAG00000044740	16:25011032-25011150	0	0	0	OK
ENSBTAG00000044741	ENSBTAG00000044741	15:42441793-42441945	0	0	0	OK
ENSBTAG00000044742	ENSBTAG00000044742	15:48977300-48977457	0	0	0	OK
ENSBTAG00000044743	ENSBTAG00000044743	28:5592552-5592659	0	0	0	OK
ENSBTAG00000044744	ENSBTAG00000044744	16:34790014-34790162	0	0	0	OK
ENSBTAG00000044745	ENSBTAG00000044745	X:60339848-60339927	0	0	0	OK
ENSBTAG00000044746	ENSBTAG00000044746	X:13429074-13429145	0	0	0	OK
ENSBTAG00000044747	ENSBTAG00000044747	23:38834972-38835081	0	0	0	OK
ENSBTAG00000044748	ENSBTAG00000044748	10:58120393-58120570	0	0	0	OK
ENSBTAG00000044751	ENSBTAG00000044751	13:21387646-21387716	0	0	0	OK
ENSBTAG00000044752	ENSBTAG00000044752	3:43428496-43428611	0	0	0	OK
ENSBTAG00000044753	ENSBTAG00000044753	9:97240339-97240423	0	0	0	OK
ENSBTAG00000044754	ENSBTAG00000044754	3:82678453-82678692	0	0	0	OK
ENSBTAG00000044756	ENSBTAG00000044756	3:4507414-4507511	0	0	0	OK
ENSBTAG00000044757	ENSBTAG00000044757	21:62872117-62872222	0	0	0	OK
ENSBTAG00000044758	ENSBTAG00000044758	21:5654804-5654871	0	0	0	OK
ENSBTAG00000044759	ENSBTAG00000044759	X:17826577-17826670	0	0	0	OK
ENSBTAG00000044760	ENSBTAG00000044760	21:67473627-67473700	0	0	0	OK
ENSBTAG00000044764	ENSBTAG00000044764	9:30296055-30296182	0	0	0	OK
ENSBTAG00000044766	ENSBTAG00000044766	14:64910448-64910552	0	0	0	OK
ENSBTAG00000044767	ENSBTAG00000044767	X:15419510-15419605	0	0	0	OK
ENSBTAG00000044769	ENSBTAG00000044769	21:2151595-2151677	0	0	0	OK
ENSBTAG00000044770	ENSBTAG00000044770	24:20801902-20801974	0	0	0	OK
ENSBTAG00000044771	ENSBTAG00000044771	1:41819289-41819388	0	0	0	OK
ENSBTAG00000044773	ENSBTAG00000044773	1:128208752-128208849	0	0	0	OK
ENSBTAG00000044774	ENSBTAG00000044774	15:64494841-64494934	0	0	0	OK
ENSBTAG00000044775	ENSBTAG00000044775	25:33439512-33439590	0	0	0	OK
ENSBTAG00000044776	ENSBTAG00000044776	4:48894614-48894744	0	0	0	OK

ENSBTAG00000044777	ENSBTAG00000044777	27:30853749-30853849	0	0	0	OK
ENSBTAG00000044778	ENSBTAG00000044778	5:109867101-109867200	0	0	0	OK
ENSBTAG00000044779	ENSBTAG00000044779	9:68677459-68677555	0	0	0	OK
ENSBTAG00000044780	ENSBTAG00000044780	4:26718559-26718680	0	0	0	OK
ENSBTAG00000044781	ENSBTAG00000044781	2:87546863-87546968	0	0	0	OK
ENSBTAG00000044782	ENSBTAG00000044782	5:65529889-65529999	0	0	0	OK
ENSBTAG00000044784	ENSBTAG00000044784	X:76958437-76958718	0	0	0	OK
ENSBTAG00000044785	ENSBTAG00000044785	20:64514526-64514631	0	0	0	OK
ENSBTAG00000044786	ENSBTAG00000044786	3:66943925-66944024	0	0	0	OK
ENSBTAG00000044787	ENSBTAG00000044787	15:65135403-65135481	0	0	0	OK
ENSBTAG00000044788	ENSBTAG00000044788	1:81060818-81060995	0	0	0	OK
ENSBTAG00000044789	ENSBTAG00000044789	7:104154442-104154514	0	0	0	OK
ENSBTAG00000044790	ENSBTAG00000044790	8:97227794-97227899	0	0	0	OK
ENSBTAG00000044791	ENSBTAG00000044791	26:40335420-40335516	0	0	0	OK
ENSBTAG00000044792	ENSBTAG00000044792	7:51115528-51115664	0	0	0	OK
ENSBTAG00000044793	ENSBTAG00000044793	21:49192780-49192874	0	0	0	OK
ENSBTAG00000044794	ENSBTAG00000044794	27:18642139-18642266	0	0	0	OK
ENSBTAG00000044795	ENSBTAG00000044795	9:22318431-22318514	0	0	0	OK
ENSBTAG00000044796	ENSBTAG00000044796	11:99019124-99019219	0	0	0	OK
ENSBTAG00000044797	ENSBTAG00000044797	21:68761088-68761184	0	0	0	OK
ENSBTAG00000044799	ENSBTAG00000044799	15:47272309-47272384	0	0	0	OK
ENSBTAG00000044800	ENSBTAG00000044800	6:107741097-107741169	0	0	0	OK
ENSBTAG00000044801	ENSBTAG00000044801	X:10126463-10126534	0	0	0	OK
ENSBTAG00000044802	ENSBTAG00000044802	7:68160605-68160744	0	0	0	OK
ENSBTAG00000044803	ENSBTAG00000044803	X:9189805-9189885	0	0	0	OK
ENSBTAG00000044805	ENSBTAG00000044805	3:18745669-18745954	0	0	0	OK
ENSBTAG00000044807	ENSBTAG00000044807	2:131659722-131659819	0	0	0	OK
ENSBTAG00000044808	ENSBTAG00000044808	18:14003562-14003632	0	0	0	OK
ENSBTAG00000044809	ENSBTAG00000044809	10:6578920-6579013	0	0	0	OK
ENSBTAG00000044810	ENSBTAG00000044810	4:3520859-3520992	0	0	0	OK

ENSBTAG00000044811	ENSBTAG00000044811	11:95816938-95817043	0	0	0	OK
ENSBTAG00000044812	ENSBTAG00000044812	5:111265046-111265102	0	0	0	OK
ENSBTAG00000044815	ENSBTAG00000044815	19:16404167-16404249	0	0	0	OK
ENSBTAG00000044816	ENSBTAG00000044816	16:31286341-31286412	0	0	0	OK
ENSBTAG00000044817	ENSBTAG00000044817	3:102923771-102923838	0	0	0	OK
ENSBTAG00000044819	ENSBTAG00000044819	16:72727893-72727965	0	0	0	OK
ENSBTAG00000044821	ENSBTAG00000044821	11:55919306-55919413	0	0	0	OK
ENSBTAG00000044823	ENSBTAG00000044823	20:17726985-17727075	0	0	0	OK
ENSBTAG00000044824	ENSBTAG00000044824	5:112517316-112517484	0	0	0	OK
ENSBTAG00000044826	ENSBTAG00000044826	9:24637585-24637690	0	0	0	OK
ENSBTAG00000044830	ENSBTAG00000044830	25:42293997-42294075	0	0	0	OK
ENSBTAG00000044831	ENSBTAG00000044831	4:42270314-42270440	0	0	0	OK
ENSBTAG00000044832	ENSBTAG00000044832	29:13237946-13238024	0	0	0	OK
ENSBTAG00000044833	ENSBTAG00000044833	26:23608960-23609031	0	0	0	OK
ENSBTAG00000044835	ENSBTAG00000044835	2:79246931-79246987	0	0	0	OK
ENSBTAG00000044837	ENSBTAG00000044837	19:28200450-28200590	0	0	0	OK
ENSBTAG00000044838	ENSBTAG00000044838	17:48160068-48160174	0	0	0	OK
ENSBTAG00000044840	ENSBTAG00000044840	19:38374503-38374566	0	0	0	OK
ENSBTAG00000044842	ENSBTAG00000044842	5:72549079-72549211	0	0	0	OK
ENSBTAG00000044843	ENSBTAG00000044843	1:80674815-80674891	0	0	0	OK
ENSBTAG00000044844	ENSBTAG00000044844	18:44843075-44843171	0	0	0	OK
ENSBTAG00000044845	ENSBTAG00000044845	13:1221601-1221691	0	0	0	OK
ENSBTAG00000044846	ENSBTAG00000044846	14:71208916-71209040	0	0	0	OK
ENSBTAG00000044847	ENSBTAG00000044847	8:112989900-112990005	0	0	0	OK
ENSBTAG00000044848	ENSBTAG00000044848	5:40138048-40138157	0	0	0	OK
ENSBTAG00000044849	ENSBTAG00000044849	15:67874694-67874766	0	0	0	OK
ENSBTAG00000044850	ENSBTAG00000044850	6:72912793-72912895	0	0	0	OK
ENSBTAG00000044851	ENSBTAG00000044851	13:12415353-12415446	0	0	0	OK
ENSBTAG00000044854	ENSBTAG00000044854	9:61590491-61590596	0	0	0	OK
ENSBTAG00000044855	ENSBTAG00000044855	17:55198804-55198938	0	0	0	OK

ENSBTAG00000044856	ENSBTAG00000044856	19:27919233-27919374	0	0	0	OK
ENSBTAG00000044859	ENSBTAG00000044859	29:45520785-45520846	0	0	0	OK
ENSBTAG00000044861	ENSBTAG00000044861	3:113598482-113598756	0	0	0	OK
ENSBTAG00000044862	ENSBTAG00000044862	19:27298366-27298439	0	0	0	OK
ENSBTAG00000044864	ENSBTAG00000044864	7:51240604-51240731	0	0	0	OK
ENSBTAG00000044865	ENSBTAG00000044865	21:67578311-67578397	0	0	0	OK
ENSBTAG00000044867	ENSBTAG00000044867	4:76606177-76606255	0	0	0	OK
ENSBTAG00000044868	ENSBTAG00000044868	4:93800640-93800709	0	0	0	OK
ENSBTAG00000044869	ENSBTAG00000044869	7:17935949-17936025	0	0	0	OK
ENSBTAG00000044870	ENSBTAG00000044870	14:35694822-35694922	0	0	0	OK
ENSBTAG00000044871	ENSBTAG00000044871	7:50951304-50951373	0	0	0	OK
ENSBTAG00000044872	ENSBTAG00000044872	3:26327369-26327655	0	0	0	OK
ENSBTAG00000044873	ENSBTAG00000044873	22:51328303-51328406	0	0	0	OK
ENSBTAG00000044874	ENSBTAG00000044874	5:12612284-12612385	0	0	0	OK
ENSBTAG00000044875	ENSBTAG00000044875	25:34438287-34438389	0	0	0	OK
ENSBTAG00000044876	ENSBTAG00000044876	13:27105358-27105465	0	0	0	OK
ENSBTAG00000044877	ENSBTAG00000044877	9:102717724-102717826	0	0	0	OK
ENSBTAG00000044879	ENSBTAG00000044879	5:57337562-57337633	0	0	0	OK
ENSBTAG00000044880	ENSBTAG00000044880	25:26692646-26692721	0	0	0	OK
ENSBTAG00000044881	ENSBTAG00000044881	9:95776709-95776947	0	0	0	OK
ENSBTAG00000044882	ENSBTAG00000044882	26:11238878-11238968	0	0	0	OK
ENSBTAG00000044883	ENSBTAG00000044883	12:31820734-31820812	0	0	0	OK
ENSBTAG00000044884	ENSBTAG00000044884	19:45204923-45204997	0	0	0	OK
ENSBTAG00000044885	ENSBTAG00000044885	11:94574529-94574657	0	0	0	OK
ENSBTAG00000044886	ENSBTAG00000044886	16:2517337-2517409	0	0	0	OK
ENSBTAG00000044887	ENSBTAG00000044887	26:20756298-20756381	0	0	0	OK
ENSBTAG00000044888	ENSBTAG00000044888	28:15505052-15505127	0	0	0	OK
ENSBTAG00000044889	ENSBTAG00000044889	4:104225255-104225349	0	0	0	OK
ENSBTAG00000044890	ENSBTAG00000044890	6:85709659-85709770	0	0	0	OK
ENSBTAG00000044891	ENSBTAG00000044891	X:18412661-18412801	0	0	0	OK

ENSBTAG00000044892	ENSBTAG00000044892	23:8738051-8738114	0	0	0	OK
ENSBTAG00000044893	ENSBTAG00000044893	8:45349534-45349639	0	0	0	OK
ENSBTAG00000044894	ENSBTAG00000044894	21:43043226-43043320	0	0	0	OK
ENSBTAG00000044896	ENSBTAG00000044896	27:18005181-18005309	0	0	0	OK
ENSBTAG00000044897	ENSBTAG00000044897	5:110023642-110023709	0	0	0	OK
ENSBTAG00000044898	ENSBTAG00000044898	7:52207255-52207396	0	0	0	OK
ENSBTAG00000044899	ENSBTAG00000044899	7:55523565-55523633	0	0	0	OK
ENSBTAG00000044900	ENSBTAG00000044900	21:22859904-22859978	0	0	0	OK
ENSBTAG00000044901	ENSBTAG00000044901	8:110852441-110852514	0	0	0	OK
ENSBTAG00000044903	ENSBTAG00000044903	16:39694468-39694529	0	0	0	OK
ENSBTAG00000044904	ENSBTAG00000044904	2:133678349-133678448	0	0	0	OK
ENSBTAG00000044905	ENSBTAG00000044905	29:25208518-25208593	0	0	0	OK
ENSBTAG00000044906	ENSBTAG00000044906	8:85557521-85557597	0	0	0	OK
ENSBTAG00000044907	ENSBTAG00000044907	27:34608026-34608131	0	0	0	OK
ENSBTAG00000044911	ENSBTAG00000044911	3:9625567-9625664	0	0	0	OK
ENSBTAG00000044912	ENSBTAG00000044912	5:31298269-31298407	0	0	0	OK
ENSBTAG00000044913	ENSBTAG00000044913	1:92811411-92811508	0	0	0	OK
ENSBTAG00000044914	ENSBTAG00000044914	9:65003565-65003656	0	0	0	OK
ENSBTAG00000044915	ENSBTAG00000044915	10:75891835-75891917	0	0	0	OK
ENSBTAG00000044916	ENSBTAG00000044916	2:44049601-44049693	0	0	0	OK
ENSBTAG00000044918	ENSBTAG00000044918	19:56342634-56342737	0	0	0	OK
ENSBTAG00000044920	ENSBTAG00000044920	26:31904023-31904090	0	0	0	OK
ENSBTAG00000044921	ENSBTAG00000044921	10:67304416-67304486	0	0	0	OK
ENSBTAG00000044922	ENSBTAG00000044922	2:119789736-119789811	0	0	0	OK
ENSBTAG00000044923	ENSBTAG00000044923	5:51686533-51686601	0	0	0	OK
ENSBTAG00000044925	ENSBTAG00000044925	4:86942132-86942473	0	0	0	OK
ENSBTAG00000044926	ENSBTAG00000044926	16:78343037-78343107	0	0	0	OK
ENSBTAG00000044928	ENSBTAG00000044928	22:57489760-57489838	0	0	0	OK
ENSBTAG00000044929	ENSBTAG00000044929	4:10654195-10654277	0	0	0	OK
ENSBTAG00000044930	ENSBTAG00000044930	1:148056940-148057014	0	0	0	OK

ENSBTAG00000044931	ENSBTAG00000044931	4:7635339-7635443	0	0	0	OK
ENSBTAG00000044932	ENSBTAG00000044932	27:35773997-35774132	0	0	0	OK
ENSBTAG00000044933	ENSBTAG00000044933	16:8241152-8241213	0	0	0	OK
ENSBTAG00000044934	ENSBTAG00000044934	1:42547014-42547151	0	0	0	OK
ENSBTAG00000044935	ENSBTAG00000044935	X:64541921-64541995	0	0	0	OK
ENSBTAG00000044938	ENSBTAG00000044938	15:25536647-25536760	0	0	0	OK
ENSBTAG00000044940	ENSBTAG00000044940	19:45893858-45893938	0	0	0	OK
ENSBTAG00000044942	ENSBTAG00000044942	7:15917818-15917890	0	0	0	OK
ENSBTAG00000044943	ENSBTAG00000044943	5:43428242-43428355	0	0	0	OK
ENSBTAG00000044944	ENSBTAG00000044944	4:50927597-50927705	0	0	0	OK
ENSBTAG00000044946	ENSBTAG00000044946	23:30835082-30835202	0	0	0	OK
ENSBTAG00000044948	ENSBTAG00000044948	2:115529719-115529778	0	0	0	OK
ENSBTAG00000044950	ENSBTAG00000044950	2:88058595-88058694	0	0	0	OK
ENSBTAG00000044951	ENSBTAG00000044951	9:26303341-26303413	0	0	0	OK
ENSBTAG00000044952	ENSBTAG00000044952	8:10782808-10782902	0	0	0	OK
ENSBTAG00000044953	ENSBTAG00000044953	3:33702815-33702894	0	0	0	OK
ENSBTAG00000044954	ENSBTAG00000044954	20:10171655-10171733	0	0	0	OK
ENSBTAG00000044955	ENSBTAG00000044955	19:54657732-54657811	0	0	0	OK
ENSBTAG00000044956	ENSBTAG00000044956	19:21433031-21433103	0	0	0	OK
ENSBTAG00000044957	ENSBTAG00000044957	12:52368150-52368261	0	0	0	OK
ENSBTAG00000044959	ENSBTAG00000044959	25:42282746-42282828	0	0	0	OK
ENSBTAG00000044962	ENSBTAG00000044962	10:88843396-88843492	0	0	0	OK
ENSBTAG00000044963	ENSBTAG00000044963	10:76759086-76759190	0	0	0	OK
ENSBTAG00000044965	ENSBTAG00000044965	12:33587192-33587281	0	0	0	OK
ENSBTAG00000044966	ENSBTAG00000044966	22:5838521-5838627	0	0	0	OK
ENSBTAG00000044967	ENSBTAG00000044967	4:39753148-39753244	0	0	0	OK
ENSBTAG00000044968	ENSBTAG00000044968	19:15222331-15222393	0	0	0	OK
ENSBTAG00000044970	ENSBTAG00000044970	9:102306272-102306376	0	0	0	OK
ENSBTAG00000044971	ENSBTAG00000044971	4:64018809-64018894	0	0	0	OK
ENSBTAG00000044972	ENSBTAG00000044972	22:48727998-48728080	0	0	0	OK

ENSBTAG00000044973	ENSBTAG00000044973	2:106321080-106321176	0	0	0	OK
ENSBTAG00000044975	ENSBTAG00000044975	2:100443937-100444029	0	0	0	OK
ENSBTAG00000044976	ENSBTAG00000044976	9:40834404-40834544	0	0	0	OK
ENSBTAG00000044977	ENSBTAG00000044977	21:67538584-67538656	0	0	0	OK
ENSBTAG00000044978	ENSBTAG00000044978	1:130032880-130032950	0	0	0	OK
ENSBTAG00000044980	ENSBTAG00000044980	23:34751705-34751814	0	0	0	OK
ENSBTAG00000044981	ENSBTAG00000044981	9:28046279-28046356	0	0	0	OK
ENSBTAG00000044982	ENSBTAG00000044982	13:45812444-45812549	0	0	0	OK
ENSBTAG00000044983	ENSBTAG00000044983	11:9805443-9805518	0	0	0	OK
ENSBTAG00000044984	ENSBTAG00000044984	9:82537356-82537484	0	0	0	OK
ENSBTAG00000044985	ENSBTAG00000044985	15:5999802-5999883	0	0	0	OK
ENSBTAG00000044986	ENSBTAG00000044986	22:59818211-59818288	0	0	0	OK
ENSBTAG00000044987	ENSBTAG00000044987	11:1600489-1600596	0	0	0	OK
ENSBTAG00000044988	ENSBTAG00000044988	13:16759238-16759338	0	0	0	OK
ENSBTAG00000044989	ENSBTAG00000044989	2:128381290-128381399	0	0	0	OK
ENSBTAG00000044990	ENSBTAG00000044990	13:54863729-54863861	0	0	0	OK
ENSBTAG00000044993	ENSBTAG00000044993	11:17415883-17415944	0	0	0	OK
ENSBTAG00000044998	ENSBTAG00000044998	29:30096634-30096715	0	0	0	OK
ENSBTAG00000044999	ENSBTAG00000044999	3:107466834-107466971	0	0	0	OK
ENSBTAG00000045001	ENSBTAG00000045001	19:20036039-20036128	0	0	0	OK
ENSBTAG00000045002	ENSBTAG00000045002	26:12787574-12787657	0	0	0	OK
ENSBTAG00000045003	ENSBTAG00000045003	1:112973111-112973184	0	0	0	OK
ENSBTAG00000045004	ENSBTAG00000045004	7:90962402-90962504	0	0	0	OK
ENSBTAG00000045005	ENSBTAG00000045005	14:28338275-28338335	0	0	0	OK
ENSBTAG00000045008	ENSBTAG00000045008	1:18801458-18801565	0	0	0	OK
ENSBTAG00000045009	ENSBTAG00000045009	16:73657748-73657821	0	0	0	OK
ENSBTAG00000045010	ENSBTAG00000045010	2:5398826-5398902	0	0	0	OK
ENSBTAG00000045012	ENSBTAG00000045012	3:15757389-15757492	0	0	0	OK
ENSBTAG00000045014	ENSBTAG00000045014	20:7288530-7288624	0	0	0	OK
ENSBTAG00000045015	ENSBTAG00000045015	9:76831896-76831990	0	0	0	OK

ENSBTAG00000045016	ENSBTAG00000045016	10:103044183-103044250	0	0	0	OK
ENSBTAG00000045017	ENSBTAG00000045017	4:52989409-52989499	0	0	0	OK
ENSBTAG00000045018	ENSBTAG00000045018	16:40328192-40328385	0	0	0	OK
ENSBTAG00000045020	ENSBTAG00000045020	24:38169934-38170063	0	0	0	OK
ENSBTAG00000045021	ENSBTAG00000045021	5:49530782-49530852	0	0	0	OK
ENSBTAG00000045022	ENSBTAG00000045022	19:40077049-40077182	0	0	0	OK
ENSBTAG00000045023	ENSBTAG00000045023	5:30251636-30251717	0	0	0	OK
ENSBTAG00000045024	ENSBTAG00000045024	1:9062784-9062846	0	0	0	OK
ENSBTAG00000045025	ENSBTAG00000045025	6:25655007-25655183	0	0	0	OK
ENSBTAG00000045026	ENSBTAG00000045026	17:54997497-54997628	0	0	0	OK
ENSBTAG00000045027	ENSBTAG00000045027	22:56699825-56699902	0	0	0	OK
ENSBTAG00000045029	ENSBTAG00000045029	10:103831663-103831718	0	0	0	OK
ENSBTAG00000045030	ENSBTAG00000045030	6:22490528-22490628	0	0	0	OK
ENSBTAG00000045031	ENSBTAG00000045031	14:29716315-29716441	0	0	0	OK
ENSBTAG00000045032	ENSBTAG00000045032	2:125646956-125647070	0	0	0	OK
ENSBTAG00000045033	ENSBTAG00000045033	X:40089604-40089666	0	0	0	OK
ENSBTAG00000045036	ENSBTAG00000045036	13:14951269-14951369	0	0	0	OK
ENSBTAG00000045038	ENSBTAG00000045038	11:18407794-18407886	0	0	0	OK
ENSBTAG00000045039	ENSBTAG00000045039	24:49943457-49943600	0	0	0	OK
ENSBTAG00000045040	ENSBTAG00000045040	29:51051770-51051846	0	0	0	OK
ENSBTAG00000045042	ENSBTAG00000045042	21:4941796-4941886	0	0	0	OK
ENSBTAG00000045043	ENSBTAG00000045043	1:30811261-30811352	0	0	0	OK
ENSBTAG00000045044	ENSBTAG00000045044	24:31054808-31054880	0	0	0	OK
ENSBTAG00000045045	ENSBTAG00000045045	12:39640395-39640466	0	0	0	OK
ENSBTAG00000045046	ENSBTAG00000045046	28:36484870-36485006	0	0	0	OK
ENSBTAG00000045047	ENSBTAG00000045047	9:90775657-90775965	0	0	0	OK
ENSBTAG00000045048	ENSBTAG00000045048	16:23447452-23447535	0	0	0	OK
ENSBTAG00000045049	ENSBTAG00000045049	X:124330457-124330580	0	0	0	OK
ENSBTAG00000045051	ENSBTAG00000045051	29:20332363-20332462	0	0	0	OK
ENSBTAG00000045052	ENSBTAG00000045052	X:67800434-67800529	0	0	0	OK

ENSBTAG00000045054	ENSBTAG00000045054	8:88893435-88893566	0	0	0	OK
ENSBTAG00000045055	ENSBTAG00000045055	24:35900578-35900669	0	0	0	OK
ENSBTAG00000045056	ENSBTAG00000045056	20:71861823-71861932	0	0	0	OK
ENSBTAG00000045057	ENSBTAG00000045057	11:16092976-16093092	0	0	0	OK
ENSBTAG00000045058	ENSBTAG00000045058	15:38352795-38352933	0	0	0	OK
ENSBTAG00000045059	ENSBTAG00000045059	22:25266502-25266607	0	0	0	OK
ENSBTAG00000045060	ENSBTAG00000045060	7:18373014-18373100	0	0	0	OK
ENSBTAG00000045061	ENSBTAG00000045061	14:53947635-53947726	0	0	0	OK
ENSBTAG00000045062	ENSBTAG00000045062	2:91219565-91219651	0	0	0	OK
ENSBTAG00000045063	ENSBTAG00000045063	X:11423937-11424010	0	0	0	OK
ENSBTAG00000045064	ENSBTAG00000045064	8:40878225-40878299	0	0	0	OK
ENSBTAG00000045065	ENSBTAG00000045065	8:68967802-68967911	0	0	0	OK
ENSBTAG00000045067	ENSBTAG00000045067	19:40992602-40992706	0	0	0	OK
ENSBTAG00000045068	ENSBTAG00000045068	3:28485588-28485684	0	0	0	OK
ENSBTAG00000045071	ENSBTAG00000045071	11:95982800-95982910	0	0	0	OK
ENSBTAG00000045072	ENSBTAG00000045072	17:10384045-10384123	0	0	0	OK
ENSBTAG00000045073	ENSBTAG00000045073	27:18068924-18069022	0	0	0	OK
ENSBTAG00000045074	ENSBTAG00000045074	2:95834549-95834636	0	0	0	OK
ENSBTAG00000045075	ENSBTAG00000045075	26:11949148-11949209	0	0	0	OK
ENSBTAG00000045076	ENSBTAG00000045076	11:83421372-83421448	0	0	0	OK
ENSBTAG00000045077	ENSBTAG00000045077	24:32848227-32848332	0	0	0	OK
ENSBTAG00000045078	ENSBTAG00000045078	4:17462502-17462655	0	0	0	OK
ENSBTAG00000045079	ENSBTAG00000045079	17:27154128-27154224	0	0	0	OK
ENSBTAG00000045080	ENSBTAG00000045080	29:1064934-1065005	0	0	0	OK
ENSBTAG00000045081	ENSBTAG00000045081	7:56029105-56029203	0	0	0	OK
ENSBTAG00000045082	ENSBTAG00000045082	26:20756088-20756231	0	0	0	OK
ENSBTAG00000045083	ENSBTAG00000045083	2:98059794-98059863	0	0	0	OK
ENSBTAG00000045084	ENSBTAG00000045084	19:50968695-50968758	0	0	6.57324	OK
ENSBTAG00000045085	ENSBTAG00000045085	24:51313724-51313832	0	0	0	OK
ENSBTAG00000045087	ENSBTAG00000045087	21:5806137-5806261	0	0	0	OK

ENSBTAG00000045089	ENSBTAG00000045089	10:52672051-52672118	0	0	0	OK
ENSBTAG00000045091	ENSBTAG00000045091	10:70639174-70639243	0	0	0	OK
ENSBTAG00000045092	ENSBTAG00000045092	2:93071374-93071480	0	0	0	OK
ENSBTAG00000045094	ENSBTAG00000045094	17:73635820-73635896	0	0	0	OK
ENSBTAG00000045095	ENSBTAG00000045095	4:49761306-49761382	0	0	0	OK
ENSBTAG00000045096	ENSBTAG00000045096	21:55818274-55818343	0	0	0	OK
ENSBTAG00000045097	ENSBTAG00000045097	14:24955768-24955835	0	0	0	OK
ENSBTAG00000045098	ENSBTAG00000045098	15:43545902-43545980	0	0	0	OK
ENSBTAG00000045099	ENSBTAG00000045099	12:36660547-36660653	0	0	0	OK
ENSBTAG00000045100	ENSBTAG00000045100	14:7325359-7325483	0	0	0	OK
ENSBTAG00000045101	ENSBTAG00000045101	1:67363712-67363923	0	0	0	OK
ENSBTAG00000045102	ENSBTAG00000045102	5:103856021-103856266	0	0	0	OK
ENSBTAG00000045103	ENSBTAG00000045103	2:7214195-7214275	0	0	0	OK
ENSBTAG00000045104	ENSBTAG00000045104	18:18611421-18611526	0	0	0	OK
ENSBTAG00000045105	ENSBTAG00000045105	16:44004092-44004153	0	0	6.78875	OK
ENSBTAG00000045106	ENSBTAG00000045106	26:41328009-41328095	0	0	0	OK
ENSBTAG00000045107	ENSBTAG00000045107	X:106947238-106947341	0	0	0	OK
ENSBTAG00000045108	ENSBTAG00000045108	14:50162666-50162746	0	0	0	OK
ENSBTAG00000045111	ENSBTAG00000045111	24:14627781-14627848	0	0	0	OK
ENSBTAG00000045112	ENSBTAG00000045112	27:18417502-18417581	0	0	0	OK
ENSBTAG00000045113	ENSBTAG00000045113	14:39156395-39156483	0	0	0	OK
ENSBTAG00000045114	ENSBTAG00000045114	29:29690396-29690513	0	0	0	OK
ENSBTAG00000045115	ENSBTAG00000045115	18:36974590-36974677	0	0	0	OK
ENSBTAG00000045116	ENSBTAG00000045116	24:55115178-55115351	0	0	0	OK
ENSBTAG00000045117	ENSBTAG00000045117	1:73566952-73567026	0	0	0	OK
ENSBTAG00000045118	ENSBTAG00000045118	10:4663359-4663443	0	0	0	OK
ENSBTAG00000045119	ENSBTAG00000045119	2:43678326-43678408	0	0	0	OK
ENSBTAG00000045120	ENSBTAG00000045120	7:80361821-80361924	0	0	0	OK
ENSBTAG00000045121	ENSBTAG00000045121	3:22685790-22685940	0	0	0	OK
ENSBTAG00000045123	ENSBTAG00000045123	4:78666107-78666191	0	0	0	OK

ENSBTAG00000045124	ENSBTAG00000045124	2:106797178-106797260	0	0	0	OK
ENSBTAG00000045125	ENSBTAG00000045125	11:326096-326180	0	0	0	OK
ENSBTAG00000045129	ENSBTAG00000045129	13:75111988-75112131	0	0	0	OK
ENSBTAG00000045130	ENSBTAG00000045130	26:21566315-21566435	0	0	0	OK
ENSBTAG00000045131	ENSBTAG00000045131	14:43762553-43762656	0	0	0	OK
ENSBTAG00000045132	ENSBTAG00000045132	6:99976543-99976614	0	0	0	OK
ENSBTAG00000045133	ENSBTAG00000045133	X:145222492-145222580	0	0	0	OK
ENSBTAG00000045134	ENSBTAG00000045134	10:8182946-8183084	0	0	0	OK
ENSBTAG00000045135	ENSBTAG00000045135	8:76698454-76698535	0	0	0	OK
ENSBTAG00000045137	ENSBTAG00000045137	5:84244499-84244574	0	0	0	OK
ENSBTAG00000045138	ENSBTAG00000045138	1:85713138-85713217	0	0	0	OK
ENSBTAG00000045139	ENSBTAG00000045139	2:35078307-35078424	0	0	0	OK
ENSBTAG00000045140	ENSBTAG00000045140	5:64962167-64962241	0	0	0	OK
ENSBTAG00000045141	ENSBTAG00000045141	20:62074380-62074484	0	0	0	OK
ENSBTAG00000045142	ENSBTAG00000045142	7:58135752-58135805	0	0	0	OK
ENSBTAG00000045144	ENSBTAG00000045144	3:102310323-102310397	0	0	0	OK
ENSBTAG00000045146	ENSBTAG00000045146	2:58619450-58619545	0	0	0	OK
ENSBTAG00000045147	ENSBTAG00000045147	25:26681102-26681168	0	0	0	OK
ENSBTAG00000045148	ENSBTAG00000045148	26:25360583-25360637	0	0	0	OK
ENSBTAG00000045149	ENSBTAG00000045149	19:18813555-18813618	0	0	0	OK
ENSBTAG00000045150	ENSBTAG00000045150	X:96903229-96903333	0	0	0	OK
ENSBTAG00000045152	ENSBTAG00000045152	8:90947609-90947705	0	0	0	OK
ENSBTAG00000045153	ENSBTAG00000045153	11:457738-457872	0	0	0	OK
ENSBTAG00000045154	ENSBTAG00000045154	5:19375907-19376010	0	0	0	OK
ENSBTAG00000045156	ENSBTAG00000045156	12:16382771-16382916	0	0	0	OK
ENSBTAG00000045157	ENSBTAG00000045157	4:38133404-38133495	0	0	0	OK
ENSBTAG00000045158	ENSBTAG00000045158	10:33675853-33675952	0	0	0	OK
ENSBTAG00000045159	ENSBTAG00000045159	21:67486580-67486652	0	0	0	OK
ENSBTAG00000045161	ENSBTAG00000045161	16:32781474-32781544	0	0	0	OK
ENSBTAG00000045162	ENSBTAG00000045162	1:46562373-46562479	0	0	0	OK

ENSBTAG00000045163	ENSBTAG00000045163	8:39952532-39952662	0	0	0	OK
ENSBTAG00000045164	ENSBTAG00000045164	18:57894883-57894983	0	0	0	OK
ENSBTAG00000045166	ENSBTAG00000045166	8:76679782-76679833	0	0	0	OK
ENSBTAG00000045167	ENSBTAG00000045167	3:3156421-3156493	0	0	0	OK
ENSBTAG00000045168	ENSBTAG00000045168	3:81707612-81707748	0	0	0	OK
ENSBTAG00000045169	ENSBTAG00000045169	10:73632147-73632220	0	0	0	OK
ENSBTAG00000045170	ENSBTAG00000045170	12:88860305-88860331	0	0	0	OK
ENSBTAG00000045171	ENSBTAG00000045171	8:6899877-6899947	0	0	0	OK
ENSBTAG00000045172	ENSBTAG00000045172	2:135810540-135810604	0	0	0	OK
ENSBTAG00000045173	ENSBTAG00000045173	3:7477864-7478000	0	0	0	OK
ENSBTAG00000045174	ENSBTAG00000045174	19:15308147-15308218	0	0	0	OK
ENSBTAG00000045176	ENSBTAG00000045176	17:22719707-22719815	0	0	0	OK
ENSBTAG00000045178	ENSBTAG00000045178	14:17809980-17810053	0	0	0	OK
ENSBTAG00000045179	ENSBTAG00000045179	5:22762055-22762156	0	0	0	OK
ENSBTAG00000045180	ENSBTAG00000045180	25:37107906-37108002	0	0	0	OK
ENSBTAG00000045181	ENSBTAG00000045181	23:30788152-30788230	0	0	0	OK
ENSBTAG00000045183	ENSBTAG00000045183	18:23993518-23993597	0	0	0	OK
ENSBTAG00000045184	ENSBTAG00000045184	21:55317320-55317406	0	0	0	OK
ENSBTAG00000045186	ENSBTAG00000045186	24:57362769-57362874	0	0	0	OK
ENSBTAG00000045187	ENSBTAG00000045187	3:110389860-110389958	0	0	0	OK
ENSBTAG00000045188	ENSBTAG00000045188	12:33142211-33142332	0	0	0	OK
ENSBTAG00000045189	ENSBTAG00000045189	13:21557630-21557737	0	0	0	OK
ENSBTAG00000045191	ENSBTAG00000045191	7:36582449-36582515	0	0	0	OK
ENSBTAG00000045193	ENSBTAG00000045193	3:67155695-67155799	0	0	3.98186	OK
ENSBTAG00000045194	ENSBTAG00000045194	26:45398045-45398146	0	0	0	OK
ENSBTAG00000045195	ENSBTAG00000045195	12:69031282-69031351	0	0	0	OK
ENSBTAG00000045196	ENSBTAG00000045196	10:55497249-55497362	0	0	0	OK
ENSBTAG00000045197	ENSBTAG00000045197	17:70739900-70739997	0	0	0	OK
ENSBTAG00000045199	ENSBTAG00000045199	10:42231364-42231440	0	0	0	OK
ENSBTAG00000045200	ENSBTAG00000045200	14:12821788-12821863	0	0	0	OK

ENSBTAG00000045201	ENSBTAG00000045201	X:116573546-116573621	0	0	0	OK
ENSBTAG00000045202	ENSBTAG00000045202	21:20767268-20767360	0	0	0	OK
ENSBTAG00000045203	ENSBTAG00000045203	8:85346635-85346768	0	0	0	OK
ENSBTAG00000045206	ENSBTAG00000045206	7:4484107-4484207	0	0	0	OK
ENSBTAG00000045207	ENSBTAG00000045207	14:49138350-49138484	0	0	0	OK
ENSBTAG00000045209	ENSBTAG00000045209	3:14896532-14896666	0	0	0	OK
ENSBTAG00000045210	ENSBTAG00000045210	9:94314513-94314599	0	0	0	OK
ENSBTAG00000045214	ENSBTAG00000045214	4:12798099-12798204	0	0	0	OK
ENSBTAG00000045215	ENSBTAG00000045215	20:58336710-58336816	0	0	0	OK
ENSBTAG00000045216	ENSBTAG00000045216	25:14171740-14171863	0	0	0	OK
ENSBTAG00000045217	ENSBTAG00000045217	24:22781555-22781625	0	0	0	OK
ENSBTAG00000045218	ENSBTAG00000045218	5:56655699-56655767	0	0	0	OK
ENSBTAG00000045219	ENSBTAG00000045219	9:73213350-73213452	0	0	0	OK
ENSBTAG00000045220	ENSBTAG00000045220	13:39836872-39836938	0	0	0	OK
ENSBTAG00000045221	ENSBTAG00000045221	2:13121228-13121356	0	0	0	OK
ENSBTAG00000045222	ENSBTAG00000045222	X:31246182-31246252	0	0	0	OK
ENSBTAG00000045223	ENSBTAG00000045223	4:66346470-66346546	0	0	0	OK
ENSBTAG00000045224	ENSBTAG00000045224	14:65081264-65081409	0	0	0	OK
ENSBTAG00000045225	ENSBTAG00000045225	13:55374499-55374578	0	0	0	OK
ENSBTAG00000045226	ENSBTAG00000045226	11:15501201-15501304	0	0	0	OK
ENSBTAG00000045228	ENSBTAG00000045228	6:72991324-72991419	0	0	0	OK
ENSBTAG00000045230	ENSBTAG00000045230	26:12253101-12253155	0	0	0	OK
ENSBTAG00000045231	ENSBTAG00000045231	13:42120607-42120711	0	0	0	OK
ENSBTAG00000045233	ENSBTAG00000045233	8:45491852-45491945	0	0	0	OK
ENSBTAG00000045234	ENSBTAG00000045234	10:104007812-104007915	0	0	0	OK
ENSBTAG00000045235	ENSBTAG00000045235	24:49943667-49943750	0	0	0	OK
ENSBTAG00000045236	ENSBTAG00000045236	13:83962475-83962542	0	0	0	OK
ENSBTAG00000045238	ENSBTAG00000045238	7:82645309-82645378	0	0	0	OK
ENSBTAG00000045239	ENSBTAG00000045239	12:30587083-30587189	0	0	0	OK
ENSBTAG00000045240	ENSBTAG00000045240	18:40207305-40207400	0	0	0	OK

ENSBTAG00000045242	ENSBTAG00000045242	8:80155443-80155546	0	0	0	OK
ENSBTAG00000045243	ENSBTAG00000045243	7:85233542-85233680	0	0	0	OK
ENSBTAG00000045244	ENSBTAG00000045244	29:50439135-50439199	0	0	0	OK
ENSBTAG00000045246	ENSBTAG00000045246	X:123004241-123004409	0	0	0	OK
ENSBTAG00000045247	ENSBTAG00000045247	26:37419075-37419149	0	0	0	OK
ENSBTAG00000045248	ENSBTAG00000045248	5:48952297-48952372	0	0	0	OK
ENSBTAG00000045249	ENSBTAG00000045249	11:101415995-101416089	0	0	0	OK
ENSBTAG00000045250	ENSBTAG00000045250	12:90660499-90660575	0	0	0	OK
ENSBTAG00000045251	ENSBTAG00000045251	7:87445334-87445422	0	0	0	OK
ENSBTAG00000045252	ENSBTAG00000045252	17:14543025-14543100	0	0	0	OK
ENSBTAG00000045253	ENSBTAG00000045253	12:51289835-51289927	0	0	0	OK
ENSBTAG00000045255	ENSBTAG00000045255	18:29266029-29266133	0	0	0	OK
ENSBTAG00000045256	ENSBTAG00000045256	11:48636810-48636937	0	0	0	OK
ENSBTAG00000045258	ENSBTAG00000045258	1:89603581-89603684	0	0	0	OK
ENSBTAG00000045260	ENSBTAG00000045260	1:137789064-137789318	0	0	0	OK
ENSBTAG00000045261	ENSBTAG00000045261	19:34255697-34255794	0	0	0	OK
ENSBTAG00000045262	ENSBTAG00000045262	12:79320444-79320545	0	0	0	OK
ENSBTAG00000045263	ENSBTAG00000045263	15:39162244-39162317	0	0	0	OK
ENSBTAG00000045264	ENSBTAG00000045264	7:110106819-110106899	0	0	0	OK
ENSBTAG00000045265	ENSBTAG00000045265	17:12916977-12917048	0	0	0	OK
ENSBTAG00000045268	ENSBTAG00000045268	6:47184384-47184455	0	0	0	OK
ENSBTAG00000045269	ENSBTAG00000045269	14:39024819-39024892	0	0	0	OK
ENSBTAG00000045270	ENSBTAG00000045270	7:21271930-21272002	0	0	0	OK
ENSBTAG00000045271	ENSBTAG00000045271	3:10688899-10688967	0	0	0	OK
ENSBTAG00000045272	ENSBTAG00000045272	1:152111843-152111950	0	0	0	OK
ENSBTAG00000045276	ENSBTAG00000045276	10:15241724-15241798	0	0	0	OK
ENSBTAG00000045277	ENSBTAG00000045277	19:50889624-50889687	0	0	0	OK
ENSBTAG00000045279	ENSBTAG00000045279	23:33385229-33385322	0	0	0	OK
ENSBTAG00000045280	ENSBTAG00000045280	8:69015773-69015866	0	0	0	OK
ENSBTAG00000045281	ENSBTAG00000045281	27:33164738-33164812	0	0	0	OK

ENSBTAG00000045282	ENSBTAG00000045282	X:126787999-126788104	0	0	0	OK
ENSBTAG00000045283	ENSBTAG00000045283	15:27334362-27334453	0	0	0	OK
ENSBTAG00000045284	ENSBTAG00000045284	3:670581-670693	0	0	3.69745	OK
ENSBTAG00000045285	ENSBTAG00000045285	28:7879942-7880010	0	0	0	OK
ENSBTAG00000045286	ENSBTAG00000045286	20:37066114-37066184	0	0	0	OK
ENSBTAG00000045287	ENSBTAG00000045287	7:26020851-26020921	0	0	0	OK
ENSBTAG00000045289	ENSBTAG00000045289	12:56028530-56028590	0	0	0	OK
ENSBTAG00000045290	ENSBTAG00000045290	6:99588367-99588444	0	0	0	OK
ENSBTAG00000045291	ENSBTAG00000045291	9:89564116-89564189	0	0	0	OK
ENSBTAG00000045292	ENSBTAG00000045292	19:62526145-62526277	0	0	0	OK
ENSBTAG00000045293	ENSBTAG00000045293	19:27918315-27918460	0	0	0	OK
ENSBTAG00000045294	ENSBTAG00000045294	1:140081312-140081419	0	0	0	OK
ENSBTAG00000045297	ENSBTAG00000045297	7:14661866-14661957	0	0	0	OK
ENSBTAG00000045299	ENSBTAG00000045299	15:39596106-39596178	0	0	0	OK
ENSBTAG00000045300	ENSBTAG00000045300	13:72652207-72652350	0	0	0	OK
ENSBTAG00000045301	ENSBTAG00000045301	7:92746708-92746778	0	0	0	OK
ENSBTAG00000045303	ENSBTAG00000045303	4:50323244-50323319	0	0	0	OK
ENSBTAG00000045304	ENSBTAG00000045304	20:55327201-55327502	0	0	0	OK
ENSBTAG00000045305	ENSBTAG00000045305	6:63346180-63346282	0	0	0	OK
ENSBTAG00000045306	ENSBTAG00000045306	7:51509434-51509499	0	0	0	OK
ENSBTAG00000045307	ENSBTAG00000045307	18:56285097-56285175	0	0	0	OK
ENSBTAG00000045308	ENSBTAG00000045308	21:2141301-2141395	0	0	0	OK
ENSBTAG00000045309	ENSBTAG00000045309	5:117119639-117119712	0	0	0	OK
ENSBTAG00000045310	ENSBTAG00000045310	13:43388069-43388198	0	0	0	OK
ENSBTAG00000045312	ENSBTAG00000045312	7:105296443-105296545	0	0	0	OK
ENSBTAG00000045313	ENSBTAG00000045313	22:42549222-42549322	0	0	0	OK
ENSBTAG00000045314	ENSBTAG00000045314	9:52421919-52422010	0	0	0	OK
ENSBTAG00000045316	ENSBTAG00000045316	2:91220353-91220437	0	0	0	OK
ENSBTAG00000045317	ENSBTAG00000045317	12:69282643-69282725	0	0	0	OK
ENSBTAG00000045318	ENSBTAG00000045318	27:17287891-17288010	0	0	0	OK

ENSBTAG00000045319	ENSBTAG00000045319	9:53400511-53400621	0	0	0	OK
ENSBTAG00000045320	ENSBTAG00000045320	24:18118074-18118168	0	0	0	OK
ENSBTAG00000045321	ENSBTAG00000045321	8:48360399-48360475	0	0	0	OK
ENSBTAG00000045322	ENSBTAG00000045322	21:40604218-40604336	0	0	0	OK
ENSBTAG00000045323	ENSBTAG00000045323	3:19159707-19159778	0	0	0	OK
ENSBTAG00000045324	ENSBTAG00000045324	23:34487278-34487370	0	0	0	OK
ENSBTAG00000045325	ENSBTAG00000045325	19:47886184-47886258	0	0	0	OK
ENSBTAG00000045326	ENSBTAG00000045326	21:67468482-67468554	0	0	0	OK
ENSBTAG00000045327	ENSBTAG00000045327	25:1522970-1523097	0	0	0	OK
ENSBTAG00000045328	ENSBTAG00000045328	9:91240216-91240304	0	0	0	OK
ENSBTAG00000045329	ENSBTAG00000045329	10:71809954-71810032	0	0	0	OK
ENSBTAG00000045330	ENSBTAG00000045330	2:130903851-130903927	0	0	0	OK
ENSBTAG00000045331	ENSBTAG00000045331	6:24463188-24463278	0	0	0	OK
ENSBTAG00000045332	ENSBTAG00000045332	6:109095916-109095988	0	0	0	OK
ENSBTAG00000045333	ENSBTAG00000045333	1:44371073-44371185	0	0	3.69745	OK
ENSBTAG00000045335	ENSBTAG00000045335	1:52194905-52194979	0	0	0	OK
ENSBTAG00000045337	ENSBTAG00000045337	23:7424172-7424240	0	0	0	OK
ENSBTAG00000045338	ENSBTAG00000045338	X:60752892-60752971	0	0	0	OK
ENSBTAG00000045339	ENSBTAG00000045339	10:68505504-68505566	0	0	0	OK
ENSBTAG00000045340	ENSBTAG00000045340	X:66217552-66217651	0	0	0	OK
ENSBTAG00000045341	ENSBTAG00000045341	3:46861490-46861593	0	0	0	OK
ENSBTAG00000045343	ENSBTAG00000045343	10:94153748-94153853	0	0	0	OK
ENSBTAG00000045344	ENSBTAG00000045344	X:92180968-92181029	0	0	0	OK
ENSBTAG00000045345	ENSBTAG00000045345	3:80952848-80952940	0	0	0	OK
ENSBTAG00000045347	ENSBTAG00000045347	2:122624675-122624797	0	0	0	OK
ENSBTAG00000045349	ENSBTAG00000045349	6:86241886-86241979	0	0	0	OK
ENSBTAG00000045350	ENSBTAG00000045350	11:82971326-82971429	0	0	0	OK
ENSBTAG00000045351	ENSBTAG00000045351	19:8866052-8866167	0	0	0	OK
ENSBTAG00000045354	ENSBTAG00000045354	1:72134325-72134452	0	0	0	OK
ENSBTAG00000045355	ENSBTAG00000045355	23:18333849-18333981	0	0	0	OK

ENSBTAG00000045358	ENSBTAG00000045358	7:63524521-63524601	0	0	0	OK
ENSBTAG00000045359	ENSBTAG00000045359	27:38109425-38109462	0	0	0	OK
ENSBTAG00000045360	ENSBTAG00000045360	10:36288596-36288712	0	0	0	OK
ENSBTAG00000045361	ENSBTAG00000045361	16:51909328-51909436	0	0	0	OK
ENSBTAG00000045363	ENSBTAG00000045363	23:22084899-22084994	0	0	0	OK
ENSBTAG00000045364	ENSBTAG00000045364	23:50677369-50677478	0	0	0	OK
ENSBTAG00000045366	ENSBTAG00000045366	2:33376330-33376527	0	0	0	OK
ENSBTAG00000045367	ENSBTAG00000045367	18:8523218-8523349	0	0	0	OK
ENSBTAG00000045368	ENSBTAG00000045368	2:103724974-103725108	0	0	0	OK
ENSBTAG00000045369	ENSBTAG00000045369	15:77643814-77643889	0	0	0	OK
ENSBTAG00000045370	ENSBTAG00000045370	18:44862089-44862235	0	0	0	OK
ENSBTAG00000045371	ENSBTAG00000045371	14:4295225-4295353	0	0	0	OK
ENSBTAG00000045372	ENSBTAG00000045372	17:72584387-72584491	0	0	0	OK
ENSBTAG00000045373	ENSBTAG00000045373	16:50502446-50502521	0	0	0	OK
ENSBTAG00000045375	ENSBTAG00000045375	2:24194739-24194815	0	0	0	OK
ENSBTAG00000045376	ENSBTAG00000045376	21:2135602-2135691	0	0	0	OK
ENSBTAG00000045377	ENSBTAG00000045377	8:97676391-97676517	0	0	0	OK
ENSBTAG00000045379	ENSBTAG00000045379	24:25397996-25398143	0	0	0	OK
ENSBTAG00000045380	ENSBTAG00000045380	8:62566670-62566742	0	0	0	OK
ENSBTAG00000045382	ENSBTAG00000045382	5:28431514-28431616	0	0	0	OK
ENSBTAG00000045383	ENSBTAG00000045383	21:34419748-34419890	0	0	0	OK
ENSBTAG00000045384	ENSBTAG00000045384	5:14477780-14477881	0	0	0	OK
ENSBTAG00000045385	ENSBTAG00000045385	26:18422851-18422984	0	0	0	OK
ENSBTAG00000045386	ENSBTAG00000045386	4:96417189-96417261	0	0	0	OK
ENSBTAG00000045387	ENSBTAG00000045387	X:129285061-129285138	0	0	0	OK
ENSBTAG00000045389	ENSBTAG00000045389	4:7772739-7772838	0	0	0	OK
ENSBTAG00000045390	ENSBTAG00000045390	6:27041346-27041409	0	0	0	OK
ENSBTAG00000045391	ENSBTAG00000045391	24:24491629-24491928	0	0	0	OK
ENSBTAG00000045392	ENSBTAG00000045392	12:11448062-11448153	0	0	0	OK
ENSBTAG00000045393	ENSBTAG00000045393	X:128508931-128509030	0	0	0	OK

ENSBTAG00000045394	ENSBTAG00000045394	X:28754754-28755087	0	0	0	OK
ENSBTAG00000045395	ENSBTAG00000045395	3:23879752-23880001	0	0	0	OK
ENSBTAG00000045396	ENSBTAG00000045396	15:41549189-41549298	0	0	0	OK
ENSBTAG00000045397	ENSBTAG00000045397	8:60746949-60747068	0	0	0	OK
ENSBTAG00000045398	ENSBTAG00000045398	14:14953689-14953753	0	0	0	OK
ENSBTAG00000045399	ENSBTAG00000045399	21:56595689-56595759	0	0	0	OK
ENSBTAG00000045400	ENSBTAG00000045400	15:65665620-65665692	0	0	0	OK
ENSBTAG00000045401	ENSBTAG00000045401	29:24371815-24371978	0	0	0	OK
ENSBTAG00000045402	ENSBTAG00000045402	3:96420814-96420943	0	0	0	OK
ENSBTAG00000045403	ENSBTAG00000045403	23:30145497-30145574	0	0	0	OK
ENSBTAG00000045404	ENSBTAG00000045404	26:13442692-13442835	0	0	0	OK
ENSBTAG00000045405	ENSBTAG00000045405	X:134464694-134464799	0	0	0	OK
ENSBTAG00000045406	ENSBTAG00000045406	9:31523124-31523251	0	0	0	OK
ENSBTAG00000045407	ENSBTAG00000045407	2:129294990-129295076	0	0	0	OK
ENSBTAG00000045408	ENSBTAG00000045408	12:84414064-84414170	0	0	0	OK
ENSBTAG00000045409	ENSBTAG00000045409	X:101508727-101508801	0	0	0	OK
ENSBTAG00000045410	ENSBTAG00000045410	7:26957581-26957671	0	0	0	OK
ENSBTAG00000045411	ENSBTAG00000045411	1:98054759-98054857	0	0	0	OK
ENSBTAG00000045413	ENSBTAG00000045413	7:104956930-104957242	0	0	0	OK
ENSBTAG00000045414	ENSBTAG00000045414	9:78077570-78077657	0	0	0	OK
ENSBTAG00000045415	ENSBTAG00000045415	21:31240949-31241020	0	0	0	OK
ENSBTAG00000045416	ENSBTAG00000045416	16:16028586-16028680	0	0	0	OK
ENSBTAG00000045417	ENSBTAG00000045417	16:22529497-22529597	0	0	0	OK
ENSBTAG00000045418	ENSBTAG00000045418	7:63886911-63886993	0	0	0	OK
ENSBTAG00000045419	ENSBTAG00000045419	6:27777684-27778005	0	0	0	OK
ENSBTAG00000045421	ENSBTAG00000045421	5:76049714-76049777	0	0	0	OK
ENSBTAG00000045422	ENSBTAG00000045422	21:67542561-67542707	0	0	0	OK
ENSBTAG00000045423	ENSBTAG00000045423	17:29521905-29522038	0	0	0	OK
ENSBTAG00000045424	ENSBTAG00000045424	1:4411216-4411313	0	0	0	OK
ENSBTAG00000045425	ENSBTAG00000045425	X:18416453-18416552	0	0	0	OK

ENSBTAG00000045426	ENSBTAG00000045426	21:2156238-2156319	0	0	0	OK
ENSBTAG00000045428	ENSBTAG00000045428	21:38128595-38128697	0	0	0	OK
ENSBTAG00000045429	ENSBTAG00000045429	9:95289914-95289978	0	0	0	OK
ENSBTAG00000045430	ENSBTAG00000045430	10:605976-606061	0	0	0	OK
ENSBTAG00000045431	ENSBTAG00000045431	2:90042281-90042389	0	0	0	OK
ENSBTAG00000045434	ENSBTAG00000045434	11:78678890-78678978	0	0	0	OK
ENSBTAG00000045435	ENSBTAG00000045435	3:95367238-95367341	0	0	0	OK
ENSBTAG00000045436	ENSBTAG00000045436	1:66048195-66048318	0	0	0	OK
ENSBTAG00000045437	ENSBTAG00000045437	10:34736652-34736722	0	0	0	OK
ENSBTAG00000045438	ENSBTAG00000045438	3:91507444-91507549	0	0	0	OK
ENSBTAG00000045439	ENSBTAG00000045439	6:72184949-72185041	0	0	0	OK
ENSBTAG00000045440	ENSBTAG00000045440	X:2991586-2991656	0	0	0	OK
ENSBTAG00000045442	ENSBTAG00000045442	9:63312778-63312879	0	0	0	OK
ENSBTAG00000045443	ENSBTAG00000045443	12:7911190-7911297	0	0	0	OK
ENSBTAG00000045444	ENSBTAG00000045444	25:31535486-31535581	0	0	0	OK
ENSBTAG00000045445	ENSBTAG00000045445	29:36102282-36102397	0	0	0	OK
ENSBTAG00000045448	ENSBTAG00000045448	5:44255548-44255662	0	0	0	OK
ENSBTAG00000045449	ENSBTAG00000045449	19:54221184-54221249	0	0	0	OK
ENSBTAG00000045450	ENSBTAG00000045450	13:6773006-6773068	0	0	0	OK
ENSBTAG00000045451	ENSBTAG00000045451	11:3870243-3870339	0	0	0	OK
ENSBTAG00000045453	ENSBTAG00000045453	13:46736891-46736981	0	0	0	OK
ENSBTAG00000045454	ENSBTAG00000045454	5:47950270-47950388	0	0	0	OK
ENSBTAG00000045455	ENSBTAG00000045455	1:70786713-70786824	0	0	0	OK
ENSBTAG00000045456	ENSBTAG00000045456	23:34868561-34868670	0	0	0	OK
ENSBTAG00000045458	ENSBTAG00000045458	7:28718742-28718809	0	0	0	OK
ENSBTAG00000045459	ENSBTAG00000045459	21:2262455-2262522	0	0	0	OK
ENSBTAG00000045460	ENSBTAG00000045460	5:104207319-104207457	0	0	0	OK
ENSBTAG00000045461	ENSBTAG00000045461	1:134556608-134556678	0	0	0	OK
ENSBTAG00000045462	ENSBTAG00000045462	20:68311019-68311146	0	0	0	OK
ENSBTAG00000045463	ENSBTAG00000045463	22:55540215-55540346	0	0	0	OK

ENSBTAG00000045464	ENSBTAG00000045464	X:101291087-101291191	0	0	0	OK
ENSBTAG00000045465	ENSBTAG00000045465	9:64172665-64172746	0	0	0	OK
ENSBTAG00000045466	ENSBTAG00000045466	8:40562530-40562600	0	0	0	OK
ENSBTAG00000045468	ENSBTAG00000045468	7:5766036-5766170	0	0	0	OK
ENSBTAG00000045469	ENSBTAG00000045469	5:112366949-112367027	0	0	0	OK
ENSBTAG00000045470	ENSBTAG00000045470	2:118290678-118290745	0	0	0	OK
ENSBTAG00000045471	ENSBTAG00000045471	X:26593694-26593787	0	0	0	OK
ENSBTAG00000045472	ENSBTAG00000045472	22:47338046-47338115	0	0	0	OK
ENSBTAG00000045473	ENSBTAG00000045473	7:49108506-49108599	0	0	0	OK
ENSBTAG00000045474	ENSBTAG00000045474	11:37219620-37219693	0	0	0	OK
ENSBTAG00000045475	ENSBTAG00000045475	19:56849680-56849752	0	0	0	OK
ENSBTAG00000045477	ENSBTAG00000045477	23:29928603-29928700	0	0	0	OK
ENSBTAG00000045478	ENSBTAG00000045478	14:1566932-1567001	0	0	0	OK
ENSBTAG00000045479	ENSBTAG00000045479	1:6827405-6827512	0	0	0	OK
ENSBTAG00000045480	ENSBTAG00000045480	7:5884982-5885086	0	0	0	OK
ENSBTAG00000045481	ENSBTAG00000045481	X:55906854-55907006	0	0	0	OK
ENSBTAG00000045482	ENSBTAG00000045482	28:41498832-41498922	0	0	0	OK
ENSBTAG00000045484	ENSBTAG00000045484	19:20743700-20743797	0	0	0	OK
ENSBTAG00000045485	ENSBTAG00000045485	2:16973493-16973557	0	0	0	OK
ENSBTAG00000045486	ENSBTAG00000045486	8:100667108-100667226	0	0	0	OK
ENSBTAG00000045487	ENSBTAG00000045487	5:38297151-38298101	0	0	0	OK
ENSBTAG00000045489	ENSBTAG00000045489	21:3672033-3672126	0	0	0	OK
ENSBTAG00000045491	ENSBTAG00000045491	27:37951736-37951901	0	0	0	OK
ENSBTAG00000045494	ENSBTAG00000045494	18:50714405-50714475	0	0	0	OK
ENSBTAG00000045495	ENSBTAG00000045495	7:40465633-40466179	0	0	0	OK
ENSBTAG00000045496	ENSBTAG00000045496	6:70048449-70048518	0	0	0	OK
ENSBTAG00000045497	ENSBTAG00000045497	21:66764060-66764393	0	0	0	OK
ENSBTAG00000045498	ENSBTAG00000045498	18:55660401-55697135	0	0	0	OK
ENSBTAG00000045501	ENSBTAG00000045501	X:140472912-140473020	0	0	0	OK
ENSBTAG00000045502	ENSBTAG00000045502	X:17862230-17862527	0	0	0	OK

ENSBTAG00000045503	ENSBTAG00000045503	28:46267506-46267626	0	0	0	OK
ENSBTAG00000045504	ENSBTAG00000045504	9:42351249-42352038	0	0	0	OK
ENSBTAG00000045506	ENSBTAG00000045506	8:104395017-104395167	0	0	0	OK
ENSBTAG00000045508	ENSBTAG00000045508	21:45415303-45415467	0	0	0	OK
ENSBTAG00000045512	ENSBTAG00000045512	12:78671173-78671280	0	0	0	OK
ENSBTAG00000045515	ENSBTAG00000045515	3:119207506-119208625	0	0	0	OK
ENSBTAG00000045516	ENSBTAG00000045516	16:34565693-34565773	0	0	0	OK
ENSBTAG00000045517	ENSBTAG00000045517	26:29697180-29697697	0	0	0	OK
ENSBTAG00000045519	ENSBTAG00000045519	6:107739968-107740041	0	0	0	OK
ENSBTAG00000045521	ENSBTAG00000045521	10:27084384-27084553	0	0	0	OK
ENSBTAG00000045522	ENSBTAG00000045522	19:56993589-56993689	0	0	0	OK
ENSBTAG00000045525	ENSBTAG00000045525	X:1632346-1632415	0	0	0	OK
ENSBTAG00000045526	ENSBTAG00000045526	13:40230296-40231781	0	0	0	OK
ENSBTAG00000045530	ENSBTAG00000045530	3:34395250-34395683	0	0	0	OK
ENSBTAG00000045531	ENSBTAG00000045531	27:16848689-16850282	0	0	0	OK
ENSBTAG00000045532	ENSBTAG00000045532	4:61197128-61197198	0	0	0	OK
ENSBTAG00000045533	ENSBTAG00000045533	9:56846524-56846603	0	0	0	OK
ENSBTAG00000045534	ENSBTAG00000045534	15:6164732-6164798	0	0	0	OK
ENSBTAG00000045536	ENSBTAG00000045536	2:19705981-19706055	0	0	0	OK
ENSBTAG00000045540	ENSBTAG00000045540	X:32347674-32348901	0	0	0	OK
ENSBTAG00000045541	ENSBTAG00000045541	13:2875480-2875589	0	0	0	OK
ENSBTAG00000045542	ENSBTAG00000045542	5:488995-515104	0	0	0	OK
ENSBTAG00000045544	ENSBTAG00000045544	X:144159055-144234222	0	0	0	OK
ENSBTAG00000045545	ENSBTAG00000045545	11:93691881-93692865	0	0	0	OK
ENSBTAG00000045546	ENSBTAG00000045546	22:38576804-38576895	0	0	0	OK
ENSBTAG00000045548	ENSBTAG00000045548	17:74286481-74297800	0	0	0	OK
ENSBTAG00000045549	ENSBTAG00000045549	17:59651988-59652055	0	0	0	OK
ENSBTAG00000045551	ENSBTAG00000045551	23:10574087-10576698	0	0	0	OK
ENSBTAG00000045553	ENSBTAG00000045553	X:18063532-18063639	0	0	0	OK
ENSBTAG00000045557	ENSBTAG00000045557	14:954554-954673	0	0	0	OK

ENSBTAG00000045559	ENSBTAG00000045559	21:14799048-14799274	0	0	0	OK
ENSBTAG00000045560	ENSBTAG00000045560	19:149229-150708	0	0	0	OK
ENSBTAG00000045562	ENSBTAG00000045562	13:69035557-69035627	0	0	0	OK
ENSBTAG00000045563	ENSBTAG00000045563	3:18268046-18269612	0	0	0	OK
ENSBTAG00000045566	ENSBTAG00000045566	12:77988224-77988904	0	0	0	OK
ENSBTAG00000045568	ENSBTAG00000045568	24:13077338-13078261	0	0	0	OK
ENSBTAG00000045569	ENSBTAG00000045569	X:19267991-19269260	0	0	0	OK
ENSBTAG00000045570	ENSBTAG00000045570	6:48464279-48464799	0	0	0	OK
ENSBTAG00000045571	ENSBTAG00000045571	18:59167479-59168680	0	0	0	OK
ENSBTAG00000045573	ENSBTAG00000045573	7:45028193-45028263	0	0	0	OK
ENSBTAG00000045575	ENSBTAG00000045575	5:114343282-114343741	0	0	0	OK
ENSBTAG00000045579	ENSBTAG00000045579	14:1204507-1204909	0	0	0	OK
ENSBTAG00000045583	ENSBTAG00000045583	9:54750524-54751998	0	0	0	OK
ENSBTAG00000045585	ENSBTAG00000045585	8:58589958-58590784	0	0	0	OK
ENSBTAG00000045587	ENSBTAG00000045587	29:50787208-50787314	0	0	0	OK
ENSBTAG00000045592	ENSBTAG00000045592	4:57168910-57169757	0	0	0	OK
ENSBTAG00000045595	ENSBTAG00000045595	10:22585602-22586185	0	0	0	OK
ENSBTAG00000045597	ENSBTAG00000045597	1:128247012-128247114	0	0	0	OK
ENSBTAG00000045600	ENSBTAG00000045600	12:8525706-8525825	0	0	0	OK
ENSBTAG00000045602	ENSBTAG00000045602	6:72480985-72487734	0	0	0	OK
ENSBTAG00000045604	ENSBTAG00000045604	10:82634714-82666926	0	0	0	OK
ENSBTAG00000045605	ENSBTAG00000045605	6:28869173-28869276	0	0	0	OK
ENSBTAG00000045606	ENSBTAG00000045606	19:9265566-9266502	0	0	0	OK
ENSBTAG00000045607	ENSBTAG00000045607	21:25902416-25902501	0	0	0	OK
ENSBTAG00000045609	ENSBTAG00000045609	13:29782457-29784020	0	0	0	OK
ENSBTAG00000045611	ENSBTAG00000045611	21:67564943-67565045	0	0	0	OK
ENSBTAG00000045613	ENSBTAG00000045613	8:59158913-59158967	0	0	0	OK
ENSBTAG00000045614	ENSBTAG00000045614	X:71950728-71951187	0	0	0	OK
ENSBTAG00000045615	ENSBTAG00000045615	7:64487940-64491261	0	0	0	OK
ENSBTAG00000045616	ENSBTAG00000045616	X:99529830-99530700	0	0	0	OK

ENSBTAG00000045619	ENSBTAG00000045619	10:79824701-79824857	0	0	0	OK
ENSBTAG00000045620	ENSBTAG00000045620	19:43863079-43863268	0	0	0	OK
ENSBTAG00000045623	ENSBTAG00000045623	7:44490471-44491549	0	0	0	OK
ENSBTAG00000045626	ENSBTAG00000045626	27:4898753-4899642	0	0	0	OK
ENSBTAG00000045627	ENSBTAG00000045627	X:28466653-28466760	0	0	0	OK
ENSBTAG00000045628	ENSBTAG00000045628	10:14329837-14334934	0	0	0	OK
ENSBTAG00000045629	ENSBTAG00000045629	3:72854737-72854844	0	0	0	OK
ENSBTAG00000045630	ENSBTAG00000045630	13:74983236-75016391	0	0	0	OK
ENSBTAG00000045631	ENSBTAG00000045631	13:5748804-5749032	0	0	0	OK
ENSBTAG00000045632	ENSBTAG00000045632	18:50715410-50715480	0	0	0	OK
ENSBTAG00000045634	ENSBTAG00000045634	10:60206010-60207710	0	0	0	OK
ENSBTAG00000045636	ENSBTAG00000045636	X:91466817-91466900	0	0	0	OK
ENSBTAG00000045638	ENSBTAG00000045638	7:9427579-9428747	0	0	0	OK
ENSBTAG00000045639	ENSBTAG00000045639	15:45416109-45418116	0	0	0	OK
ENSBTAG00000045640	ENSBTAG00000045640	21:3692815-3692908	0	0	0	OK
ENSBTAG00000045641	ENSBTAG00000045641	19:20704464-20704531	0	0	0	OK
ENSBTAG00000045643	ENSBTAG00000045643	3:99427365-99429471	0	0	0	OK
ENSBTAG00000045644	ENSBTAG00000045644	7:43382433-43383384	0	0	0	OK
ENSBTAG00000045646	ENSBTAG00000045646	1:95963740-95963893	0	0	0	OK
ENSBTAG00000045647	ENSBTAG00000045647	3:114715939-114716518	0	0	0	OK
ENSBTAG00000045648	ENSBTAG00000045648	4:25032854-25050074	0	0	0	OK
ENSBTAG00000045649	ENSBTAG00000045649	27:5457174-5465029	0	0	0	OK
ENSBTAG00000045650	ENSBTAG00000045650	29:33618539-33618730	0	0	0	OK
ENSBTAG00000045657	ENSBTAG00000045657	6:92538757-92538827	0	0	0	OK
ENSBTAG00000045658	ENSBTAG00000045658	15:43705885-43706823	0	0	0	OK
ENSBTAG00000045659	ENSBTAG00000045659	11:47151862-47152392	0	0	0	OK
ENSBTAG00000045660	ENSBTAG00000045660	X:111387159-111387711	0	0	0	OK
ENSBTAG00000045663	ENSBTAG00000045663	3:117980134-117980199	0	0	0	OK
ENSBTAG00000045665	ENSBTAG00000045665	17:15274688-15274943	0	0	0	OK
ENSBTAG00000045666	ENSBTAG00000045666	22:33123-34397	0	0	0	OK

ENSBTAG00000045668	ENSBTAG00000045668	2:125501621-125501825	0	0	0	OK
ENSBTAG00000045670	ENSBTAG00000045670	29:38594535-38600471	0	0	0	OK
ENSBTAG00000045671	ENSBTAG00000045671	13:70650513-70651308	0	0	0	OK
ENSBTAG00000045672	ENSBTAG00000045672	3:16143202-16143325	0	0	0	OK
ENSBTAG00000045673	ENSBTAG00000045673	4:30124742-30125198	0	0	0	OK
ENSBTAG00000045674	ENSBTAG00000045674	8:23231883-23232444	0	0	0	OK
ENSBTAG00000045675	ENSBTAG00000045675	X:39355161-39363370	0	0	0	OK
ENSBTAG00000045676	ENSBTAG00000045676	4:1268552-1268662	0	0	0	OK
ENSBTAG00000045677	ENSBTAG00000045677	4:107295009-107297429	0	0	0	OK
ENSBTAG00000045679	ENSBTAG00000045679	10:82557429-82587611	0	0	0	OK
ENSBTAG00000045680	ENSBTAG00000045680	25:8995452-8995710	0	0	0	OK
ENSBTAG00000045681	ENSBTAG00000045681	23:3198182-3198256	0	0	0	OK
ENSBTAG00000045682	ENSBTAG00000045682	10:59981335-59981439	0	0	0	OK
ENSBTAG00000045686	ENSBTAG00000045686	6:107740454-107740527	0	0	0	OK
ENSBTAG00000045687	ENSBTAG00000045687	12:40839512-40839612	0	0	0	OK
ENSBTAG00000045688	ENSBTAG00000045688	9:403684-403791	0	0	0	OK
ENSBTAG00000045691	ENSBTAG00000045691	7:42505372-42506299	0	0	0	OK
ENSBTAG00000045692	ENSBTAG00000045692	18:34196398-34215940	0	0	0	OK
ENSBTAG00000045693	ENSBTAG00000045693	25:139603-139695	0	0	0	OK
ENSBTAG00000045694	ENSBTAG00000045694	X:82779028-82779631	0	0	0	OK
ENSBTAG00000045695	ENSBTAG00000045695	20:71302632-71318130	0	0	0	OK
ENSBTAG00000045698	ENSBTAG00000045698	23:3198341-3198415	0	0	0	OK
ENSBTAG00000045700	ENSBTAG00000045700	9:97638102-97638181	0	0	5.24195	OK
ENSBTAG00000045705	ENSBTAG00000045705	19:51087031-51087291	0	0	0	OK
ENSBTAG00000045707	ENSBTAG00000045707	14:541817-541936	0	0	0	OK
ENSBTAG00000045709	ENSBTAG00000045709	15:80389093-80390035	0	0	0	OK
ENSBTAG00000045710	ENSBTAG00000045710	2:66269409-66269501	0	0	0	OK
ENSBTAG00000045712	ENSBTAG00000045712	3:21284206-21284362	0	0	0	OK
ENSBTAG00000045713	ENSBTAG00000045713	16:18341162-18341296	0	0	0	OK
ENSBTAG00000045716	ENSBTAG00000045716	16:42633975-42634091	0	0	0	OK

ENSBTAG00000045718	ENSBTAG00000045718	3:14462415-14462523	0	0	0	OK
ENSBTAG00000045719	ENSBTAG00000045719	7:48644080-48644153	0	0	0	OK
ENSBTAG00000045721	ENSBTAG00000045721	12:81323147-81323251	0	0	0	OK
ENSBTAG00000045722	ENSBTAG00000045722	5:59876136-59881842	0	0	0	OK
ENSBTAG00000045723	ENSBTAG00000045723	15:80807805-80808747	0	0	0	OK
ENSBTAG00000045724	ENSBTAG00000045724	17:12651154-12651232	0	0	0	OK
ENSBTAG00000045725	ENSBTAG00000045725	9:19811008-19811114	0	0	0	OK
ENSBTAG00000045726	ENSBTAG00000045726	9:15798578-15802324	0	0	0	OK
ENSBTAG00000045729	ENSBTAG00000045729	16:68078642-68078846	0	0	0	OK
ENSBTAG00000045730	ENSBTAG00000045730	21:46413844-46413909	0	0	0	OK
ENSBTAG00000045731	ENSBTAG00000045731	18:64184832-64187720	0	0	0	OK
ENSBTAG00000045732	ENSBTAG00000045732	19:44296164-44312850	0	0	0	OK
ENSBTAG00000045735	ENSBTAG00000045735	4:1017735-1017845	0	0	0	OK
ENSBTAG00000045736	ENSBTAG00000045736	X:101169451-101169859	0	0	0	OK
ENSBTAG00000045738	ENSBTAG00000045738	17:35870787-35873721	0	0	0	OK
ENSBTAG00000045739	ENSBTAG00000045739	15:80990804-80991764	0	0	0	OK
ENSBTAG00000045740	ENSBTAG00000045740	26:25785191-25786143	0	0	0	OK
ENSBTAG00000045749	ENSBTAG00000045749	22:34753798-34753905	0	0	0	OK
ENSBTAG00000045750	ENSBTAG00000045750	X:143364825-143375658	0	0	0	OK
ENSBTAG00000045752	ENSBTAG00000045752	5:59372194-59373133	0	0	0	OK
ENSBTAG00000045753	ENSBTAG00000045753	13:50358161-50359402	0	0	0	OK
ENSBTAG00000045755	ENSBTAG00000045755	7:22384883-22385179	0	0	0	OK
ENSBTAG00000045756	ENSBTAG00000045756	X:53200773-53201043	0	0	0	OK
ENSBTAG00000045757	ENSBTAG00000045757	22:48988983-48991875	0	0	0	OK
ENSBTAG00000045758	ENSBTAG00000045758	10:22674451-22675008	0	0	0	OK
ENSBTAG00000045761	ENSBTAG00000045761	11:104943120-104943245	0	0	0	OK
ENSBTAG00000045766	ENSBTAG00000045766	11:25042327-25042434	0	0	0	OK
ENSBTAG00000045771	ENSBTAG00000045771	1:143368302-143368674	0	0	0	OK
ENSBTAG00000045774	ENSBTAG00000045774	10:82172714-82172815	0	0	0	OK
ENSBTAG00000045778	ENSBTAG00000045778	18:45406678-45406784	0	0	0	OK

ENSBTAG00000045779	ENSBTAG00000045779	X:3777246-3794469	0	0	0.29081	OK
ENSBTAG00000045780	ENSBTAG00000045780	14:394935-395055	0	0	0	OK
ENSBTAG00000045781	ENSBTAG00000045781	1:40496876-40497173	0	0	0	OK
ENSBTAG00000045783	ENSBTAG00000045783	16:5201142-5201319	0	0	0	OK
ENSBTAG00000045784	ENSBTAG00000045784	13:51873664-51876027	0	0	0	OK
ENSBTAG00000045786	ENSBTAG00000045786	5:99823288-99830338	0	0	0	OK
ENSBTAG00000045788	ENSBTAG00000045788	28:35433479-35433834	0	0	1.16652	OK
ENSBTAG00000045789	ENSBTAG00000045789	16:49259286-49262028	0	0	0	OK
ENSBTAG00000045792	ENSBTAG00000045792	3:101819038-101819118	0	0	0	OK
ENSBTAG00000045793	ENSBTAG00000045793	19:23875289-23875382	0	0	0	OK
ENSBTAG00000045796	ENSBTAG00000045796	1:75986736-75986831	0	0	0	OK
ENSBTAG00000045797	ENSBTAG00000045797	9:42120207-42120285	0	0	0	OK
ENSBTAG00000045798	ENSBTAG00000045798	X:34377970-34378351	0	0	0	OK
ENSBTAG00000045804	ENSBTAG00000045804	6:38541578-38541674	0	0	0	OK
ENSBTAG00000045806	ENSBTAG00000045806	18:46243827-46244730	0	0	0	OK
ENSBTAG00000045807	ENSBTAG00000045807	5:60077563-60078505	0	0	0	OK
ENSBTAG00000045809	ENSBTAG00000045809	X:134370953-134372211	0	0	0	OK
ENSBTAG00000045810	ENSBTAG00000045810	17:72880943-72881236	0	0	0	OK
ENSBTAG00000045811	ENSBTAG00000045811	X:64805798-64805989	0	0	0	OK
ENSBTAG00000045813	ENSBTAG00000045813	20:47991927-47992701	0	0	0	OK
ENSBTAG00000045820	ENSBTAG00000045820	1:56028466-56029112	0	0	0	OK
ENSBTAG00000045822	ENSBTAG00000045822	15:74915625-74916613	0	0	0	OK
ENSBTAG00000045823	ENSBTAG00000045823	10:20196626-20196696	0	0	0	OK
ENSBTAG00000045826	ENSBTAG00000045826	28:28021566-28021938	0	0	0	OK
ENSBTAG00000045830	ENSBTAG00000045830	27:20592772-20593785	0	0	0	OK
ENSBTAG00000045831	ENSBTAG00000045831	16:38639783-38639873	0	0	0	OK
ENSBTAG00000045836	ENSBTAG00000045836	21:71493482-71493560	0	0	0	OK
ENSBTAG00000045840	ENSBTAG00000045840	19:38542845-38542920	0	0	0	OK
ENSBTAG00000045842	ENSBTAG00000045842	5:52148240-52148717	0	0	0	OK
ENSBTAG00000045843	ENSBTAG00000045843	2:21766611-21766718	0	0	0	OK

ENSBTAG00000045844	ENSBTAG00000045844	21:60869326-60869445	0	0	0	OK
ENSBTAG00000045846	ENSBTAG00000045846	9:82145987-82146093	0	0	0	OK
ENSBTAG00000045847	ENSBTAG00000045847	10:62412033-62412276	0	0	0	OK
ENSBTAG00000045848	ENSBTAG00000045848	5:88326752-88326827	0	0	0	OK
ENSBTAG00000045849	ENSBTAG00000045849	10:102885319-102887015	0	0	0	OK
ENSBTAG00000045851	ENSBTAG00000045851	17:60293166-60293999	0	0	0	OK
ENSBTAG00000045852	ENSBTAG00000045852	8:97112931-97113246	0	0	0	OK
ENSBTAG00000045853	ENSBTAG00000045853	X:41130958-41132723	0	0	0	OK
ENSBTAG00000045854	ENSBTAG00000045854	18:62895515-62898446	0	0	0	OK
ENSBTAG00000045855	ENSBTAG00000045855	1:55802105-55802433	0	0	0	OK
ENSBTAG00000045856	ENSBTAG00000045856	7:19075176-19075273	0	0	0	OK
ENSBTAG00000045858	ENSBTAG00000045858	7:53859675-53860389	0	0	0	OK
ENSBTAG00000045859	ENSBTAG00000045859	7:53979050-53981402	0	0	0	OK
ENSBTAG00000045861	ENSBTAG00000045861	18:63479270-63486993	0	0	0	OK
ENSBTAG00000045863	ENSBTAG00000045863	10:25059684-25060164	0	0	0	OK
ENSBTAG00000045864	ENSBTAG00000045864	18:62347608-62348205	0	0	0	OK
ENSBTAG00000045865	ENSBTAG00000045865	14:72697087-72697387	0	0	0	OK
ENSBTAG00000045866	ENSBTAG00000045866	10:15570427-15571044	0	0	0	OK
ENSBTAG00000045869	ENSBTAG00000045869	20:58492915-58493009	0	0	0	OK
ENSBTAG00000045870	ENSBTAG00000045870	22:58391420-58391488	0	0	0	OK
ENSBTAG00000045871	ENSBTAG00000045871	4:112126679-112126805	0	0	0	OK
ENSBTAG00000045872	ENSBTAG00000045872	15:49911997-49912940	0	0	0	OK
ENSBTAG00000045873	ENSBTAG00000045873	1:5891440-5891949	0	0	1.34017	OK
ENSBTAG00000045874	ENSBTAG00000045874	13:15197608-15197702	0	0	0	OK
ENSBTAG00000045875	ENSBTAG00000045875	5:75117001-75117069	0	0	0	OK
ENSBTAG00000045876	ENSBTAG00000045876	X:60057721-60075100	0	0	0	OK
ENSBTAG00000045878	ENSBTAG00000045878	15:62799409-62799790	0	0	0	OK
ENSBTAG00000045880	ENSBTAG00000045880	18:57938694-57953490	0	0	0	OK
ENSBTAG00000045881	ENSBTAG00000045881	15:14156931-14157020	0	0	0	OK
ENSBTAG00000045883	ENSBTAG00000045883	10:25090079-25090882	0	0	0	OK

ENSBTAG00000045886	ENSBTAG00000045886	10:87269506-87280022	0	0	0	OK
ENSBTAG00000045889	ENSBTAG00000045889	3:28737676-28745359	0	0	0	OK
ENSBTAG00000045890	ENSBTAG00000045890	X:119366304-119367844	0	0	0	OK
ENSBTAG00000045891	ENSBTAG00000045891	X:55970536-55970635	0	0	0	OK
ENSBTAG00000045894	ENSBTAG00000045894	21:60864852-60864971	0	0	0	OK
ENSBTAG00000045896	ENSBTAG00000045896	25:38041959-38053172	0	0	0	OK
ENSBTAG00000045897	ENSBTAG00000045897	1:51289616-51289708	0	0	0	OK
ENSBTAG00000045898	ENSBTAG00000045898	9:96440861-96441347	0	0	0	OK
ENSBTAG00000045900	ENSBTAG00000045900	8:60510529-60511468	0	0	0	OK
ENSBTAG00000045901	ENSBTAG00000045901	25:2435643-2438483	0	0	0	OK
ENSBTAG00000045906	ENSBTAG00000045906	9:84191530-84191621	0	0	0	OK
ENSBTAG00000045907	ENSBTAG00000045907	12:87654332-87671973	0	0	0	OK
ENSBTAG00000045908	ENSBTAG00000045908	X:7651150-7651257	0	0	0	OK
ENSBTAG00000045909	ENSBTAG00000045909	7:102227148-102236125	0	0	0	OK
ENSBTAG00000045912	ENSBTAG00000045912	3:83945361-83946502	0	0	0	OK
ENSBTAG00000045913	ENSBTAG00000045913	10:59982198-59982302	0	0	0	OK
ENSBTAG00000045914	ENSBTAG00000045914	7:21247238-21247649	0	0	0	OK
ENSBTAG00000045915	ENSBTAG00000045915	29:50791816-50794320	0	0	0	OK
ENSBTAG00000045916	ENSBTAG00000045916	19:43883311-43883502	0	0	0	OK
ENSBTAG00000045917	ENSBTAG00000045917	6:116957906-116959264	0	0	0	OK
ENSBTAG00000045918	ENSBTAG00000045918	16:51729725-51729814	0	0	0	OK
ENSBTAG00000045919	ENSBTAG00000045919	5:62391853-62392231	0	0	0	OK
ENSBTAG00000045920	ENSBTAG00000045920	12:82085940-82086059	0	0	0	OK
ENSBTAG00000045926	ENSBTAG00000045926	21:71492951-71493032	0	0	0	OK
ENSBTAG00000045927	ENSBTAG00000045927	7:11301948-11302988	0	0	0	OK
ENSBTAG00000045928	ENSBTAG00000045928	13:67102206-67118598	0	0	5.52152	OK
ENSBTAG00000045930	ENSBTAG00000045930	18:49191749-49192151	0	0	0	OK
ENSBTAG00000045931	ENSBTAG00000045931	X:79554828-79602478	0	0	0	OK
ENSBTAG00000045933	ENSBTAG00000045933	X:93386610-93386676	0	0	0	OK
ENSBTAG00000045935	ENSBTAG00000045935	11:28600746-28600832	0	0	0	OK

ENSBTAG00000045938	ENSBTAG00000045938	17:35912720-35914373	0	0	0	OK
ENSBTAG00000045941	ENSBTAG00000045941	7:104690241-104705356	0	0	0	OK
ENSBTAG00000045942	ENSBTAG00000045942	22:49629873-49630194	0	0	0	OK
ENSBTAG00000045944	ENSBTAG00000045944	15:61420029-61420149	0	0	0	OK
ENSBTAG00000045947	ENSBTAG00000045947	X:40060593-40061079	0	0	0	OK
ENSBTAG00000045949	ENSBTAG00000045949	28:44195942-44205538	0	0	0	OK
ENSBTAG00000045950	ENSBTAG00000045950	12:57714877-57718931	0	0	0	OK
ENSBTAG00000045953	ENSBTAG00000045953	10:23636370-23636869	0	0	0	OK
ENSBTAG00000045955	ENSBTAG00000045955	17:74607592-74609032	0	0	0	OK
ENSBTAG00000045956	ENSBTAG00000045956	11:70865037-70865115	0	0	0	OK
ENSBTAG00000045960	ENSBTAG00000045960	21:17516745-17516849	0	0	0	OK
ENSBTAG00000045961	ENSBTAG00000045961	1:69280211-69280296	0	0	0	OK
ENSBTAG00000045962	ENSBTAG00000045962	2:25920716-25920820	0	0	0	OK
ENSBTAG00000045963	ENSBTAG00000045963	21:57844280-57844399	0	0	0	OK
ENSBTAG00000045964	ENSBTAG00000045964	19:12570838-12573533	0	0	0	OK
ENSBTAG00000045965	ENSBTAG00000045965	6:92540267-92540337	0	0	0	OK
ENSBTAG00000045967	ENSBTAG00000045967	3:15657631-15658331	0	0	0	OK
ENSBTAG00000045968	ENSBTAG00000045968	18:55776308-55776371	0	0	0	OK
ENSBTAG00000045970	ENSBTAG00000045970	3:117916706-117917945	0	0	0	OK
ENSBTAG00000045972	ENSBTAG00000045972	7:8154656-8155607	0	0	0	OK
ENSBTAG00000045973	ENSBTAG00000045973	19:43829755-43829946	0	0	0	OK
ENSBTAG00000045974	ENSBTAG00000045974	1:32836013-32836606	0	0	0	OK
ENSBTAG00000045975	ENSBTAG00000045975	21:3675814-3675906	0	0	0	OK
ENSBTAG00000045980	ENSBTAG00000045980	10:42902259-42902938	0	0	0	OK
ENSBTAG00000045981	ENSBTAG00000045981	7:22195392-22195989	0	0	0	OK
ENSBTAG00000045983	ENSBTAG00000045983	5:112600380-112600457	0	0	0	OK
ENSBTAG00000045984	ENSBTAG00000045984	1:559138-559877	0	0	0	OK
ENSBTAG00000045985	ENSBTAG00000045985	18:60233004-60243938	0	0	0	OK
ENSBTAG00000045987	ENSBTAG00000045987	3:5352170-5352859	0	0	0	OK
ENSBTAG00000045988	ENSBTAG00000045988	14:321351-321482	0	0	0	OK

ENSBTAG00000045989	ENSBTAG00000045989	18:63119872-63124888	0	0	0	OK
ENSBTAG00000045991	ENSBTAG00000045991	1:98127788-98128106	0	0	0	OK
ENSBTAG00000045992	ENSBTAG00000045992	12:58187518-58187639	0	0	0	OK
ENSBTAG00000045993	ENSBTAG00000045993	10:20960284-20960867	0	0	0	OK
ENSBTAG00000045997	ENSBTAG00000045997	18:46468397-46469045	0	0	0	OK
ENSBTAG00000045999	ENSBTAG00000045999	9:9699646-9700027	0	0	0	OK
ENSBTAG00000046000	ENSBTAG00000046000	2:8844052-8844553	0	0	0	OK
ENSBTAG00000046001	ENSBTAG00000046001	19:43534126-43534233	0	0	0	OK
ENSBTAG00000046008	ENSBTAG00000046008	1:120714266-120714944	0	0	0	OK
ENSBTAG00000046010	ENSBTAG00000046010	25:38342492-38343056	0	0	0	OK
ENSBTAG00000046013	ENSBTAG00000046013	29:29453499-29459846	0	0	0	OK
ENSBTAG00000046014	ENSBTAG00000046014	25:42272934-42282988	0	0	0	OK
ENSBTAG00000046015	ENSBTAG00000046015	1:143370685-143371060	0	0	0	OK
ENSBTAG00000046021	ENSBTAG00000046021	19:32537448-32537540	0	0	0	OK
ENSBTAG00000046025	ENSBTAG00000046025	29:49990735-49990808	0	0	0	OK
ENSBTAG00000046029	ENSBTAG00000046029	2:94382386-94382773	0	0	0	OK
ENSBTAG00000046030	ENSBTAG00000046030	X:59637826-59638519	0	0	0	OK
ENSBTAG00000046032	ENSBTAG00000046032	2:49400487-49401260	0	0	0	OK
ENSBTAG00000046033	ENSBTAG00000046033	25:21476162-21488221	0	0	0	OK
ENSBTAG00000046036	ENSBTAG00000046036	22:16535372-16536133	0	0	0	OK
ENSBTAG00000046039	ENSBTAG00000046039	8:51614614-51615168	0	0	0	OK
ENSBTAG00000046040	ENSBTAG00000046040	1:131089307-131090084	0	0	0	OK
ENSBTAG00000046043	ENSBTAG00000046043	8:13051734-13052613	0	0	0	OK
ENSBTAG00000046048	ENSBTAG00000046048	10:79815456-79816448	0	0	0	OK
ENSBTAG00000046049	ENSBTAG00000046049	11:72059856-72060691	0	0	0	OK
ENSBTAG00000046050	ENSBTAG00000046050	2:125499759-125499963	0	0	0	OK
ENSBTAG00000046052	ENSBTAG00000046052	10:36017218-36018364	0	0	0	OK
ENSBTAG00000046053	ENSBTAG00000046053	21:3699791-3699884	0	0	0	OK
ENSBTAG00000046055	ENSBTAG00000046055	21:1291699-1291806	0	0	0	OK
ENSBTAG00000046057	ENSBTAG00000046057	23:49386707-49387742	0	0	0	OK

ENSBTAG00000046058	ENSBTAG00000046058	10:26543971-26544568	0	0	0	OK
ENSBTAG00000046059	ENSBTAG00000046059	X:45145243-45145350	0	0	0	OK
ENSBTAG00000046060	ENSBTAG00000046060	16:12661604-12662451	0	0	0	OK
ENSBTAG00000046062	ENSBTAG00000046062	16:49429154-49429874	0	0	0	OK
ENSBTAG00000046063	ENSBTAG00000046063	19:47441443-47441573	0	0	0	OK
ENSBTAG00000046064	ENSBTAG00000046064	18:48485493-48485586	0	0	0	OK
ENSBTAG00000046065	ENSBTAG00000046065	18:41163929-41164024	0	0	0	OK
ENSBTAG00000046067	ENSBTAG00000046067	17:73346079-73346140	0	0	0	OK
ENSBTAG00000046070	ENSBTAG00000046070	16:72422138-72422861	0	0	0	OK
ENSBTAG00000046071	ENSBTAG00000046071	27:6476078-6478832	0	0	0	OK
ENSBTAG00000046072	ENSBTAG00000046072	1:3607746-3607842	0	0	0	OK
ENSBTAG00000046074	ENSBTAG00000046074	13:2800006-2802674	0	0	0	OK
ENSBTAG00000046078	ENSBTAG00000046078	19:43226783-43226918	0	0	0	OK
ENSBTAG00000046079	ENSBTAG00000046079	8:97126435-97127407	0	0	0	OK
ENSBTAG00000046081	ENSBTAG00000046081	3:21199662-21199826	0	0	0	OK
ENSBTAG00000046082	ENSBTAG00000046082	17:16725950-16726102	0	0	0	OK
ENSBTAG00000046090	ENSBTAG00000046090	29:46969454-46970420	0	0	0	OK
ENSBTAG00000046091	ENSBTAG00000046091	16:66897683-66898319	0	0	0	OK
ENSBTAG00000046093	ENSBTAG00000046093	21:2083791-2083885	0	0	0	OK
ENSBTAG00000046096	ENSBTAG00000046096	23:31113275-31113572	0	0	1.39432	OK
ENSBTAG00000046099	ENSBTAG00000046099	15:45959239-45959320	0	0	0	OK
ENSBTAG00000046100	ENSBTAG00000046100	26:14093094-14093609	0	0	0	OK
ENSBTAG00000046106	ENSBTAG00000046106	7:46406445-46406564	0	0	0	OK
ENSBTAG00000046107	ENSBTAG00000046107	23:16837458-16843931	0	0	0	OK
ENSBTAG00000046108	ENSBTAG00000046108	5:84751934-84752420	0	0	0	OK
ENSBTAG00000046111	ENSBTAG00000046111	18:63725627-63729366	0	0	0	OK
ENSBTAG00000046112	ENSBTAG00000046112	3:45449361-45449470	0	0	0	OK
ENSBTAG00000046113	ENSBTAG00000046113	21:45378249-45378413	0	0	0	OK
ENSBTAG00000046116	ENSBTAG00000046116	23:26688280-26690021	0	0	0	OK
ENSBTAG00000046117	ENSBTAG00000046117	11:63290421-63395507	0	0	0	OK

ENSBTAG00000046119	ENSBTAG00000046119	8:22739270-22739969	0	0	0	OK
ENSBTAG00000046120	ENSBTAG00000046120	9:96791120-96791565	0	0	0	OK
ENSBTAG00000046122	ENSBTAG00000046122	11:105790353-105790698	0	0	0	OK
ENSBTAG00000046123	ENSBTAG00000046123	X:143103986-143315918	0	0	0	OK
ENSBTAG00000046125	ENSBTAG00000046125	X:114450904-114451546	0	0	0	OK
ENSBTAG00000046128	ENSBTAG00000046128	13:45054152-45054234	0	0	0	OK
ENSBTAG00000046131	ENSBTAG00000046131	X:143535532-143537923	0	0	0	OK
ENSBTAG00000046133	ENSBTAG00000046133	8:59158036-59158093	0	0	0	OK
ENSBTAG00000046134	ENSBTAG00000046134	9:77236220-77236303	0	0	0	OK
ENSBTAG00000046136	ENSBTAG00000046136	19:50680460-50680523	0	0	0	OK
ENSBTAG00000046141	ENSBTAG00000046141	21:67602717-67602821	0	0	0	OK
ENSBTAG00000046145	ENSBTAG00000046145	15:19828449-19828534	0	0	0	OK
ENSBTAG00000046146	ENSBTAG00000046146	X:28372396-28372486	0	0	0	OK
ENSBTAG00000046147	ENSBTAG00000046147	X:90724328-90724421	0	0	0	OK
ENSBTAG00000046151	ENSBTAG00000046151	X:123659619-123660449	0	0	0	OK
ENSBTAG00000046153	ENSBTAG00000046153	24:44291871-44292990	0	0	0	OK
ENSBTAG00000046154	ENSBTAG00000046154	19:19989507-19989591	0	0	0	OK
ENSBTAG00000046156	ENSBTAG00000046156	18:1748744-1760788	0	0	0	OK
ENSBTAG00000046157	ENSBTAG00000046157	10:24149310-24149779	0	0	0	OK
ENSBTAG00000046162	ENSBTAG00000046162	19:42818404-42850694	0	0	0	OK
ENSBTAG00000046164	ENSBTAG00000046164	4:106472835-106473327	0	0	0	OK
ENSBTAG00000046165	ENSBTAG00000046165	17:74471760-74472114	0	0	0	OK
ENSBTAG00000046166	ENSBTAG00000046166	14:3554061-3564952	0	0	0	OK
ENSBTAG00000046168	ENSBTAG00000046168	9:75308952-75309469	0	0	0	OK
ENSBTAG00000046170	ENSBTAG00000046170	24:477734-478896	0	0	0	OK
ENSBTAG00000046173	ENSBTAG00000046173	5:120924136-120932984	0	0	0	OK
ENSBTAG00000046174	ENSBTAG00000046174	19:62550514-62575992	0	0	0	OK
ENSBTAG00000046175	ENSBTAG00000046175	3:7540340-7542460	0	0	0	OK
ENSBTAG00000046179	ENSBTAG00000046179	25:31466261-31466383	0	0	0	OK
ENSBTAG00000046182	ENSBTAG00000046182	3:105998434-105998515	0	0	0	OK

ENSBTAG00000046183	ENSBTAG00000046183	19:43912414-43912605	0	0	0	OK
ENSBTAG00000046184	ENSBTAG00000046184	10:43935667-43936221	0	0	0	OK
ENSBTAG00000046186	ENSBTAG00000046186	21:70114199-70114755	0	0	0	OK
ENSBTAG00000046193	ENSBTAG00000046193	23:4158400-4180362	0	0	0	OK
ENSBTAG00000046194	ENSBTAG00000046194	7:41164727-41164796	0	0	0	OK
ENSBTAG00000046197	ENSBTAG00000046197	5:16534697-16534778	0	0	0	OK
ENSBTAG00000046198	ENSBTAG00000046198	21:67598342-67598451	0	0	0	OK
ENSBTAG00000046200	ENSBTAG00000046200	7:44096133-44097048	0	0	0	OK
ENSBTAG00000046201	ENSBTAG00000046201	X:143993399-144006853	0	0	0	OK
ENSBTAG00000046204	ENSBTAG00000046204	27:4996158-4999264	0	0	0	OK
ENSBTAG00000046205	ENSBTAG00000046205	3:21434198-21434335	0	0	0	OK
ENSBTAG00000046206	ENSBTAG00000046206	20:71972393-71972507	0	0	0	OK
ENSBTAG00000046207	ENSBTAG00000046207	10:27882290-27883226	0	0	0	OK
ENSBTAG00000046208	ENSBTAG00000046208	14:1782900-1788087	0	0	0	OK
ENSBTAG00000046209	ENSBTAG00000046209	17:20132500-20132665	0	0	0	OK
ENSBTAG00000046212	ENSBTAG00000046212	3:18332098-18332193	0	0	0	OK
ENSBTAG00000046214	ENSBTAG00000046214	12:19542745-19542831	0	0	0	OK
ENSBTAG00000046215	ENSBTAG00000046215	11:105892685-105893315	0	0	0	OK
ENSBTAG00000046219	ENSBTAG00000046219	9:91215611-91216688	0	0	0	OK
ENSBTAG00000046222	ENSBTAG00000046222	1:84255079-84255254	0	0	0	OK
ENSBTAG00000046224	ENSBTAG00000046224	10:60735197-60735289	0	0	0	OK
ENSBTAG00000046225	ENSBTAG00000046225	19:41853945-41854242	0	0	0	OK
ENSBTAG00000046226	ENSBTAG00000046226	19:51110370-51110433	0	0	0	OK
ENSBTAG00000046227	ENSBTAG00000046227	7:92872310-92873366	0	0	0	OK
ENSBTAG00000046230	ENSBTAG00000046230	9:67929103-67929504	0	0	0	OK
ENSBTAG00000046232	ENSBTAG00000046232	9:28544215-28544698	0	0	0	OK
ENSBTAG00000046234	ENSBTAG00000046234	7:45995153-45995312	0	0	0	OK
ENSBTAG00000046235	ENSBTAG00000046235	9:61890329-61907177	0	0	0	OK
ENSBTAG00000046236	ENSBTAG00000046236	3:8693216-8693801	0	0	0	OK
ENSBTAG00000046237	ENSBTAG00000046237	23:21839484-21840055	0	0	0	OK

ENSBTAG00000046238	ENSBTAG00000046238	4:98081232-98081287	0	0	0	OK
ENSBTAG00000046240	ENSBTAG00000046240	25:17730045-17730115	0	0	0	OK
ENSBTAG00000046241	ENSBTAG00000046241	14:499610-499730	0	0	0	OK
ENSBTAG00000046244	ENSBTAG00000046244	2:108038073-108038142	0	0	0	OK
ENSBTAG00000046245	ENSBTAG00000046245	7:45034372-45034833	0	0	0	OK
ENSBTAG00000046246	ENSBTAG00000046246	19:9817744-9817959	0	0	0	OK
ENSBTAG00000046247	ENSBTAG00000046247	29:27338163-27339246	0	0	0	OK
ENSBTAG00000046250	ENSBTAG00000046250	5:120010009-120010543	0	0	0	OK
ENSBTAG00000046253	ENSBTAG00000046253	13:74537698-74537790	0	0	0	OK
ENSBTAG00000046254	ENSBTAG00000046254	15:24946854-24948115	0	0	0	OK
ENSBTAG00000046258	ENSBTAG00000046258	8:8401225-8405958	0	0	0	OK
ENSBTAG00000046259	ENSBTAG00000046259	11:38297997-38298497	0	0	0	OK
ENSBTAG00000046260	ENSBTAG00000046260	19:46754051-46754429	0	0	0	OK
ENSBTAG00000046261	ENSBTAG00000046261	3:106294418-106294690	0	0	0	OK
ENSBTAG00000046262	ENSBTAG00000046262	7:53973805-53976193	0	0	0	OK
ENSBTAG00000046263	ENSBTAG00000046263	7:45563502-45566823	0	0	0	OK
ENSBTAG00000046266	ENSBTAG00000046266	7:19187446-19190363	0	0	0	OK
ENSBTAG00000046267	ENSBTAG00000046267	28:17759830-17759924	0	0	0	OK
ENSBTAG00000046268	ENSBTAG00000046268	5:99599781-99604410	0	0	0	OK
ENSBTAG00000046269	ENSBTAG00000046269	24:39080443-39081202	0	0	0	OK
ENSBTAG00000046270	ENSBTAG00000046270	6:20442287-20442550	0	0	0	OK
ENSBTAG00000046272	ENSBTAG00000046272	7:66858269-66858331	0	0	0	OK
ENSBTAG00000046275	ENSBTAG00000046275	4:18061967-18062249	0	0	0	OK
ENSBTAG00000046278	ENSBTAG00000046278	15:80371553-80372495	0	0	0	OK
ENSBTAG00000046284	ENSBTAG00000046284	5:21181754-21181833	0	0	0	OK
ENSBTAG00000046285	ENSBTAG00000046285	15:80254690-80256208	0	0	0	OK
ENSBTAG00000046287	ENSBTAG00000046287	X:124658070-124658186	0	0	0	OK
ENSBTAG00000046288	ENSBTAG00000046288	X:3313815-3316393	0	0	0	OK
ENSBTAG00000046289	ENSBTAG00000046289	27:6982215-6982322	0	0	0	OK
ENSBTAG00000046290	ENSBTAG00000046290	19:30400966-30401380	0	0	0	OK

ENSBTAG00000046291	ENSBTAG00000046291	18:55697573-55707567	0	0	0	OK
ENSBTAG00000046293	ENSBTAG00000046293	3:23556080-23558293	0	0	0	OK
ENSBTAG00000046296	ENSBTAG00000046296	28:10258875-10258984	0	0	0	OK
ENSBTAG00000046298	ENSBTAG00000046298	5:120064153-120094175	0	0	0	OK
ENSBTAG00000046300	ENSBTAG00000046300	17:5226753-5226847	0	0	0	OK
ENSBTAG00000046302	ENSBTAG00000046302	17:35768715-35771692	0	0	0	OK
ENSBTAG00000046306	ENSBTAG00000046306	17:51355675-51357428	0	0	0	OK
ENSBTAG00000046307	ENSBTAG00000046307	14:20738813-20740407	0	0	0	OK
ENSBTAG00000046309	ENSBTAG00000046309	10:73129828-73133979	0	0	0	OK
ENSBTAG00000046310	ENSBTAG00000046310	11:65502805-65503144	0	0	0	OK
ENSBTAG00000046311	ENSBTAG00000046311	10:56820936-56821305	0	0	0	OK
ENSBTAG00000046312	ENSBTAG00000046312	X:83235870-83235978	0	0	0	OK
ENSBTAG00000046313	ENSBTAG00000046313	1:52363680-52364911	0	0	0	OK
ENSBTAG00000046314	ENSBTAG00000046314	X:56796340-56826755	0	0	0	OK
ENSBTAG00000046324	ENSBTAG00000046324	5:100926602-100938555	0	0	0	OK
ENSBTAG00000046327	ENSBTAG00000046327	X:72161025-72161711	0	0	0	OK
ENSBTAG00000046328	ENSBTAG00000046328	X:140154705-140182539	0	0	0	OK
ENSBTAG00000046329	ENSBTAG00000046329	1:137147858-137148728	0	0	0	OK
ENSBTAG00000046330	ENSBTAG00000046330	22:35086554-35086887	0	0	0	OK
ENSBTAG00000046331	ENSBTAG00000046331	10:93652213-93652294	0	0	0	OK
ENSBTAG00000046337	ENSBTAG00000046337	24:43249499-43250564	0	0	0	OK
ENSBTAG00000046338	ENSBTAG00000046338	GJ060120.1:1008-8398	0	0	0	OK
ENSBTAG00000046341	ENSBTAG00000046341	4:105529330-105564689	0	0	0	OK
ENSBTAG00000046349	ENSBTAG00000046349	7:75043588-75043969	0	0	0	OK
ENSBTAG00000046351	ENSBTAG00000046351	2:66268593-66268685	0	0	0	OK
ENSBTAG00000046352	ENSBTAG00000046352	13:35065346-35065642	0	0	0	OK
ENSBTAG00000046353	ENSBTAG00000046353	3:113947489-113948484	0	0	0	OK
ENSBTAG00000046354	ENSBTAG00000046354	15:77964412-77966408	0	0	0	OK
ENSBTAG00000046355	ENSBTAG00000046355	28:30009323-30009656	0	0	0	OK
ENSBTAG00000046356	ENSBTAG00000046356	3:54946555-54946626	0	0	0	OK

ENSBTAG00000046357	ENSBTAG00000046357	28:27680679-27685834	0	0	0	OK
ENSBTAG00000046360	ENSBTAG00000046360	29:7530171-7530778	0	0	0	OK
ENSBTAG00000046361	ENSBTAG00000046361	10:25221273-25221738	0	0	0	OK
ENSBTAG00000046362	ENSBTAG00000046362	26:31745741-31750006	0	0	0	OK
ENSBTAG00000046363	ENSBTAG00000046363	10:38814395-38814674	0	0	0	OK
ENSBTAG00000046364	ENSBTAG00000046364	21:71189270-71383753	0	0	0	OK
ENSBTAG00000046366	ENSBTAG00000046366	X:143692534-143693164	0	0	0	OK
ENSBTAG00000046367	ENSBTAG00000046367	18:47862387-47883111	0	0	0	OK
ENSBTAG00000046368	ENSBTAG00000046368	3:20805718-20806132	0	0	0	OK
ENSBTAG00000046369	ENSBTAG00000046369	1:23101042-23101153	0	0	0	OK
ENSBTAG00000046371	ENSBTAG00000046371	27:1330180-1330256	0	0	0	OK
ENSBTAG00000046373	ENSBTAG00000046373	23:31914308-31914703	0	0	0	OK
ENSBTAG00000046375	ENSBTAG00000046375	13:74236865-74238520	0	0	0	OK
ENSBTAG00000046378	ENSBTAG00000046378	15:49154821-49155792	0	0	0	OK
ENSBTAG00000046381	ENSBTAG00000046381	23:29993262-29993369	0	0	0	OK
ENSBTAG00000046382	ENSBTAG00000046382	24:278433-278552	0	0	0	OK
ENSBTAG00000046386	ENSBTAG00000046386	12:24926156-24926477	0	0	0	OK
ENSBTAG00000046388	ENSBTAG00000046388	18:54224746-54224815	0	0	6.00165	OK
ENSBTAG00000046389	ENSBTAG00000046389	5:100095827-100101039	0	0	0	OK
ENSBTAG00000046390	ENSBTAG00000046390	10:27243252-27244179	0	0	0	OK
ENSBTAG00000046392	ENSBTAG00000046392	5:74631081-74631702	0	0	0	OK
ENSBTAG00000046393	ENSBTAG00000046393	1:35649283-35649390	0	0	0	OK
ENSBTAG00000046397	ENSBTAG00000046397	7:11217023-11218082	0	0	0	OK
ENSBTAG00000046399	ENSBTAG00000046399	25:11704357-11718601	0	0	0	OK
ENSBTAG00000046400	ENSBTAG00000046400	2:80999167-80999262	0	0	0	OK
ENSBTAG00000046402	ENSBTAG00000046402	5:71355229-71355736	0	0	0	OK
ENSBTAG00000046403	ENSBTAG00000046403	18:61013840-61013933	0	0	0	OK
ENSBTAG00000046404	ENSBTAG00000046404	27:6904604-6904711	0	0	0	OK
ENSBTAG00000046405	ENSBTAG00000046405	27:5793033-5797904	0	0	3.73076	OK
ENSBTAG00000046407	ENSBTAG00000046407	X:107569249-107570686	0	0	0	OK

ENSBTAG00000046410	ENSBTAG00000046410	29:28231631-28232567	0	0	0	OK
ENSBTAG00000046411	ENSBTAG00000046411	24:57803094-57803213	0	0	0	OK
ENSBTAG00000046413	ENSBTAG00000046413	6:32202194-32205548	0	0	0	OK
ENSBTAG00000046414	ENSBTAG00000046414	7:8900739-8900801	0	0	6.67926	OK
ENSBTAG00000046415	ENSBTAG00000046415	11:105904769-105905783	0	0	0	OK
ENSBTAG00000046416	ENSBTAG00000046416	11:105915063-105916356	0	0	0	OK
ENSBTAG00000046417	ENSBTAG00000046417	7:42833644-42834607	0	0	0	OK
ENSBTAG00000046418	ENSBTAG00000046418	11:105872248-105872719	0	0	0	OK
ENSBTAG00000046420	ENSBTAG00000046420	21:67514963-67515038	0	0	0	OK
ENSBTAG00000046422	ENSBTAG00000046422	14:62374865-62374961	0	0	0	OK
ENSBTAG00000046425	ENSBTAG00000046425	18:49605113-49606340	0	0	0	OK
ENSBTAG00000046426	ENSBTAG00000046426	10:25167873-25168957	0	0	0	OK
ENSBTAG00000046428	ENSBTAG00000046428	21:67577930-67578036	0	0	0	OK
ENSBTAG00000046429	ENSBTAG00000046429	9:71425709-71426680	0	0	0	OK
ENSBTAG00000046431	ENSBTAG00000046431	8:71181972-71184938	0	0	0	OK
ENSBTAG00000046432	ENSBTAG00000046432	10:555201-555884	0	0	0	OK
ENSBTAG00000046434	ENSBTAG00000046434	22:54085984-54086089	0	0	0	OK
ENSBTAG00000046435	ENSBTAG00000046435	14:1321086-1322214	0	0	0	OK
ENSBTAG00000046437	ENSBTAG00000046437	29:51025432-51025530	0	0	0	OK
ENSBTAG00000046438	ENSBTAG00000046438	3:28875279-28875606	0	0	0	OK
ENSBTAG00000046439	ENSBTAG00000046439	17:50734878-50736971	0	0	0	OK
ENSBTAG00000046443	ENSBTAG00000046443	8:22954215-22954854	0	0	0	OK
ENSBTAG00000046445	ENSBTAG00000046445	16:424691-425632	0	0	0	OK
ENSBTAG00000046446	ENSBTAG00000046446	5:59531213-59532137	0	0	0	OK
ENSBTAG00000046447	ENSBTAG00000046447	1:148569291-148581291	0	0	0	OK
ENSBTAG00000046449	ENSBTAG00000046449	20:1935205-1940216	0	0	0	OK
ENSBTAG00000046450	ENSBTAG00000046450	3:60346811-60347472	0	0	0	OK
ENSBTAG00000046451	ENSBTAG00000046451	18:54101132-54104913	0	0	0	OK
ENSBTAG00000046453	ENSBTAG00000046453	28:10933403-10934282	0	0	0	OK
ENSBTAG00000046455	ENSBTAG00000046455	21:71432820-71432927	0	0	0	OK

ENSBTAG00000046456	ENSBTAG00000046456	8:23310185-23310956	0	0	0	OK
ENSBTAG00000046457	ENSBTAG00000046457	4:72533215-72533908	0	0	0	OK
ENSBTAG00000046464	ENSBTAG00000046464	10:22740901-22742574	0	0	0	OK
ENSBTAG00000046465	ENSBTAG00000046465	28:3368076-3368182	0	0	0	OK
ENSBTAG00000046469	ENSBTAG00000046469	X:77981839-77981946	0	0	0	OK
ENSBTAG00000046473	ENSBTAG00000046473	6:32225725-32227610	0	0	0	OK
ENSBTAG00000046475	ENSBTAG00000046475	X:73683357-73683464	0	0	0	OK
ENSBTAG00000046477	ENSBTAG00000046477	10:23448187-23448748	0	0	0	OK
ENSBTAG00000046478	ENSBTAG00000046478	10:25385631-25387286	0	0	0	OK
ENSBTAG00000046479	ENSBTAG00000046479	12:78672932-78673039	0	0	0	OK
ENSBTAG00000046480	ENSBTAG00000046480	21:67589464-67589564	0	0	0	OK
ENSBTAG00000046483	ENSBTAG00000046483	X:143750455-143750560	0	0	0	OK
ENSBTAG00000046487	ENSBTAG00000046487	11:53847827-53848119	0	0	0	OK
ENSBTAG00000046490	ENSBTAG00000046490	3:75739297-75739400	0	0	0	OK
ENSBTAG00000046491	ENSBTAG00000046491	4:60018208-60019082	0	0	0	OK
ENSBTAG00000046492	ENSBTAG00000046492	1:38712790-38713251	0	0	0	OK
ENSBTAG00000046494	ENSBTAG00000046494	16:44011278-44012877	0	0	0	OK
ENSBTAG00000046495	ENSBTAG00000046495	9:74453534-74454206	0	0	0	OK
ENSBTAG00000046496	ENSBTAG00000046496	4:106504177-106504600	0	0	0	OK
ENSBTAG00000046499	ENSBTAG00000046499	26:25928927-25930145	0	0	0	OK
ENSBTAG00000046500	ENSBTAG00000046500	10:23592375-23593078	0	0	0	OK
ENSBTAG00000046501	ENSBTAG00000046501	5:33932226-33932301	0	0	0	OK
ENSBTAG00000046502	ENSBTAG00000046502	16:63030134-63128053	0	0	0	OK
ENSBTAG00000046504	ENSBTAG00000046504	9:55820113-55820217	0	0	0	OK
ENSBTAG00000046506	ENSBTAG00000046506	X:57676226-57676827	0	0	0	OK
ENSBTAG00000046507	ENSBTAG00000046507	25:34409280-34409377	0	0	0	OK
ENSBTAG00000046511	ENSBTAG00000046511	5:44599375-44607131	0	0	0	OK
ENSBTAG00000046513	ENSBTAG00000046513	7:10556259-10557186	0	0	0	OK
ENSBTAG00000046514	ENSBTAG00000046514	26:24664880-24679483	0	0	0	OK
ENSBTAG00000046515	ENSBTAG00000046515	17:36079228-36079342	0	0	0	OK

ENSBTAG00000046518	ENSBTAG00000046518	X:143125003-143125384	0	0	0	OK
ENSBTAG00000046521	ENSBTAG00000046521	2:93684244-93684415	0	0	0	OK
ENSBTAG00000046522	ENSBTAG00000046522	X:67815910-67815996	0	0	0	OK
ENSBTAG00000046523	ENSBTAG00000046523	25:303-423	0	0	0	OK
ENSBTAG00000046524	ENSBTAG00000046524	19:20705825-20705895	0	0	0	OK
ENSBTAG00000046525	ENSBTAG00000046525	18:8934123-8934199	0	0	0	OK
ENSBTAG00000046526	ENSBTAG00000046526	8:80824528-80836135	0	0	0	OK
ENSBTAG00000046528	ENSBTAG00000046528	11:70864291-70864367	0	0	0	OK
ENSBTAG00000046529	ENSBTAG00000046529	4:77369434-77369487	0	0	0	OK
ENSBTAG00000046530	ENSBTAG00000046530	10:22844924-22845432	0	0	0	OK
ENSBTAG00000046531	ENSBTAG00000046531	X:95330252-95330600	0	0	0	OK
ENSBTAG00000046532	ENSBTAG00000046532	19:9830971-9831184	0	0	0	OK
ENSBTAG00000046534	ENSBTAG00000046534	22:61401188-61402113	0	0	0	OK
ENSBTAG00000046535	ENSBTAG00000046535	8:29660829-29660932	0	0	0	OK
ENSBTAG00000046536	ENSBTAG00000046536	11:93670179-93671118	0	0	0	OK
ENSBTAG00000046537	ENSBTAG00000046537	6:92539368-92539438	0	0	0	OK
ENSBTAG00000046538	ENSBTAG00000046538	17:9640669-9640768	0	0	0	OK
ENSBTAG00000046539	ENSBTAG00000046539	X:47650080-47650494	0	0	0	OK
ENSBTAG00000046540	ENSBTAG00000046540	21:59822519-59830426	0	0	0	OK
ENSBTAG00000046541	ENSBTAG00000046541	15:48593525-48594479	0	0	0	OK
ENSBTAG00000046543	ENSBTAG00000046543	21:3664036-3664103	0	0	0	OK
ENSBTAG00000046546	ENSBTAG00000046546	10:24242923-24243386	0	0	0	OK
ENSBTAG00000046547	ENSBTAG00000046547	24:43217454-43224463	0	0	0	OK
ENSBTAG00000046550	ENSBTAG00000046550	9:20861146-20861267	0	0	0	OK
ENSBTAG00000046551	ENSBTAG00000046551	25:33716082-33716205	0	0	0	OK
ENSBTAG00000046552	ENSBTAG00000046552	12:81157176-81158069	0	0	0	OK
ENSBTAG00000046556	ENSBTAG00000046556	23:36292361-36293944	0	0	0	OK
ENSBTAG00000046557	ENSBTAG00000046557	25:30052450-30052521	0	0	0	OK
ENSBTAG00000046558	ENSBTAG00000046558	20:70441411-70441518	0	0	0	OK
ENSBTAG00000046559	ENSBTAG00000046559	19:8089642-8090023	0	0	0	OK

ENSBTAG00000046560	ENSBTAG00000046560	X:18835411-18835689	0	0	0	OK
ENSBTAG00000046562	ENSBTAG00000046562	13:49326847-49326948	0	0	0	OK
ENSBTAG00000046567	ENSBTAG00000046567	15:6455959-6456032	0	0	0	OK
ENSBTAG00000046568	ENSBTAG00000046568	21:2081014-2081105	0	0	0	OK
ENSBTAG00000046571	ENSBTAG00000046571	4:120316399-120316492	0	0	0	OK
ENSBTAG00000046572	ENSBTAG00000046572	2:34156110-34156185	0	0	0	OK
ENSBTAG00000046575	ENSBTAG00000046575	20:26174066-26174830	0	0	0	OK
ENSBTAG00000046576	ENSBTAG00000046576	23:13043441-13043516	0	0	0	OK
ENSBTAG00000046577	ENSBTAG00000046577	1:115663235-115663342	0	0	0	OK
ENSBTAG00000046581	ENSBTAG00000046581	23:32218408-32218500	0	0	0	OK
ENSBTAG00000046582	ENSBTAG00000046582	X:111529261-111529813	0	0	0	OK
ENSBTAG00000046586	ENSBTAG00000046586	4:71515017-71515715	0	0	0	OK
ENSBTAG00000046587	ENSBTAG00000046587	29:44770864-44771529	0	0	0	OK
ENSBTAG00000046589	ENSBTAG00000046589	X:111447209-111448433	0	0	0	OK
ENSBTAG00000046591	ENSBTAG00000046591	21:20144493-20144827	0	0	0	OK
ENSBTAG00000046592	ENSBTAG00000046592	14:412105-412212	0	0	0	OK
ENSBTAG00000046596	ENSBTAG00000046596	7:87898171-87898305	0	0	0	OK
ENSBTAG00000046597	ENSBTAG00000046597	28:42138337-42139699	0	0	0	OK
ENSBTAG00000046598	ENSBTAG00000046598	8:22941497-22942085	0	0	0	OK
ENSBTAG00000046601	ENSBTAG00000046601	5:37603460-37604197	0	0	0	OK
ENSBTAG00000046603	ENSBTAG00000046603	X:75524157-75524241	0	0	0	OK
ENSBTAG00000046604	ENSBTAG00000046604	10:30039412-30039967	0	0	0	OK
ENSBTAG00000046605	ENSBTAG00000046605	2:28141167-28141273	0	0	0	OK
ENSBTAG00000046606	ENSBTAG00000046606	23:52488069-52488188	0	0	0	OK
ENSBTAG00000046613	ENSBTAG00000046613	X:136261572-136261679	0	0	0	OK
ENSBTAG00000046615	ENSBTAG00000046615	19:39746946-39747171	0	0	0	OK
ENSBTAG00000046617	ENSBTAG00000046617	14:36234751-36234858	0	0	0	OK
ENSBTAG00000046619	ENSBTAG00000046619	1:19773-19899	0	0	0	OK
ENSBTAG00000046622	ENSBTAG00000046622	25:28507374-28507684	0	0	0	OK
ENSBTAG00000046624	ENSBTAG00000046624	15:50094317-50095966	0	0	0	OK

ENSBTAG00000046625	ENSBTAG00000046625	4:77365883-77366798	0	0	0	OK
ENSBTAG00000046627	ENSBTAG00000046627	15:50687362-50688310	0	0	0	OK
ENSBTAG00000046628	ENSBTAG00000046628	5:44631280-44638274	0	0	0	OK
ENSBTAG00000046629	ENSBTAG00000046629	8:23016039-23016627	0	0	0	OK
ENSBTAG00000046633	ENSBTAG00000046633	21:69185854-69186382	0	0	0	OK
ENSBTAG00000046636	ENSBTAG00000046636	15:42331672-42351530	0	0	0	OK
ENSBTAG00000046640	ENSBTAG00000046640	12:52352245-52352324	0	0	0	OK
ENSBTAG00000046641	ENSBTAG00000046641	24:10072078-10072187	0	0	0	OK
ENSBTAG00000046643	ENSBTAG00000046643	10:25303472-25304172	0	0	0	OK
ENSBTAG00000046646	ENSBTAG00000046646	13:69685431-69685503	0	0	0	OK
ENSBTAG00000046647	ENSBTAG00000046647	29:37430399-37430498	0	0	0	OK
ENSBTAG00000046651	ENSBTAG00000046651	10:27230230-27231172	0	0	0	OK
ENSBTAG00000046653	ENSBTAG00000046653	X:32717107-32719660	0	0	0	OK
ENSBTAG00000046656	ENSBTAG00000046656	3:10236167-10237078	0	0	0	OK
ENSBTAG00000046657	ENSBTAG00000046657	17:50927146-50927590	0	0	0	OK
ENSBTAG00000046658	ENSBTAG00000046658	20:34708762-34708835	0	0	0	OK
ENSBTAG00000046659	ENSBTAG00000046659	X:98345057-98346008	0	0	0	OK
ENSBTAG00000046660	ENSBTAG00000046660	4:106441002-106441506	0	0	0	OK
ENSBTAG00000046661	ENSBTAG00000046661	7:13754306-13754377	0	0	0	OK
ENSBTAG00000046666	ENSBTAG00000046666	18:49888148-49890488	0	0	0	OK
ENSBTAG00000046667	ENSBTAG00000046667	12:8501887-8502006	0	0	0	OK
ENSBTAG00000046669	ENSBTAG00000046669	17:25116914-25121853	0	0	0	OK
ENSBTAG00000046673	ENSBTAG00000046673	13:71326364-71326745	0	0	0	OK
ENSBTAG00000046674	ENSBTAG00000046674	GJ059463.1:7702-7821	0	0	0	OK
ENSBTAG00000046675	ENSBTAG00000046675	26:47180462-47180538	0	0	0	OK
ENSBTAG00000046676	ENSBTAG00000046676	17:49265075-49265631	0	0	0	OK
ENSBTAG00000046677	ENSBTAG00000046677	17:75103301-75104723	0	0	0	OK
ENSBTAG00000046678	ENSBTAG00000046678	X:82642267-82642870	0	0	0	OK
ENSBTAG00000046681	ENSBTAG00000046681	2:125500692-125500896	0	0	0	OK
ENSBTAG00000046687	ENSBTAG00000046687	8:40213499-40213679	0	0	0	OK

ENSBTAG00000046688	ENSBTAG00000046688	7:34615434-34616823	0	0	0	OK
ENSBTAG00000046689	ENSBTAG00000046689	17:5105466-5105545	0	0	0	OK
ENSBTAG00000046691	ENSBTAG00000046691	23:52494698-52495637	0	0	0	OK
ENSBTAG00000046692	ENSBTAG00000046692	7:91916211-91916311	0	0	0	OK
ENSBTAG00000046693	ENSBTAG00000046693	19:57472338-57477414	0	0	0	OK
ENSBTAG00000046695	ENSBTAG00000046695	8:59157113-59157170	0	0	0	OK
ENSBTAG00000046696	ENSBTAG00000046696	7:29648291-29648900	0	0	0	OK
ENSBTAG00000046697	ENSBTAG00000046697	11:54629428-54629512	0	0	0	OK
ENSBTAG00000046698	ENSBTAG00000046698	17:110944-111886	0	0	0	OK
ENSBTAG00000046699	ENSBTAG00000046699	2:79975338-80043984	0	0	0	OK
ENSBTAG00000046700	ENSBTAG00000046700	6:99833915-99835304	0	0	0	OK
ENSBTAG00000046701	ENSBTAG00000046701	26:18442779-18443469	0	0	0	OK
ENSBTAG00000046702	ENSBTAG00000046702	8:10000249-10000393	0	0	0	OK
ENSBTAG00000046704	ENSBTAG00000046704	13:74757039-74757219	0	0	0	OK
ENSBTAG00000046705	ENSBTAG00000046705	7:44969943-44970050	0	0	0	OK
ENSBTAG00000046706	ENSBTAG00000046706	7:9762645-9762752	0	0	0	OK
ENSBTAG00000046709	ENSBTAG00000046709	X:39209796-39211770	0	0	0	OK
ENSBTAG00000046710	ENSBTAG00000046710	15:80502820-80503741	0	0	0	OK
ENSBTAG00000046713	ENSBTAG00000046713	9:74338935-74339254	0	0	0	OK
ENSBTAG00000046715	ENSBTAG00000046715	12:44321994-44322044	0	0	0	OK
ENSBTAG00000046718	ENSBTAG00000046718	7:28548893-28554475	0	0	0	OK
ENSBTAG00000046719	ENSBTAG00000046719	14:478517-478614	0	0	0	OK
ENSBTAG00000046721	ENSBTAG00000046721	5:113841714-113842282	0	0	0	OK
ENSBTAG00000046725	ENSBTAG00000046725	13:75316422-75318650	0	0	0	OK
ENSBTAG00000046726	ENSBTAG00000046726	9:93261249-93261906	0	0	0	OK
ENSBTAG00000046731	ENSBTAG00000046731	22:58798303-58798431	0	0	0	OK
ENSBTAG00000046732	ENSBTAG00000046732	15:6178031-6188969	0	0	0	OK
ENSBTAG00000046733	ENSBTAG00000046733	9:102132403-102133397	0	0	0	OK
ENSBTAG00000046734	ENSBTAG00000046734	4:120765616-120765736	0	0	0	OK
ENSBTAG00000046735	ENSBTAG00000046735	5:51133609-51133684	0	0	0	OK

ENSBTAG00000046742	ENSBTAG00000046742	5:100479658-100484421	0	0	0	OK
ENSBTAG00000046743	ENSBTAG00000046743	19:22151864-22151934	0	0	0	OK
ENSBTAG00000046747	ENSBTAG00000046747	17:60146173-60146573	0	0	0	OK
ENSBTAG00000046749	ENSBTAG00000046749	18:29444421-29509220	0	0	0	OK
ENSBTAG00000046751	ENSBTAG00000046751	11:105116895-105117015	0	0	0	OK
ENSBTAG00000046753	ENSBTAG00000046753	25:36521454-36523178	0	0	0	OK
ENSBTAG00000046754	ENSBTAG00000046754	GJ060027.1:2379-2486	0	0	0	OK
ENSBTAG00000046755	ENSBTAG00000046755	1:23993691-23995967	0	0	0	OK
ENSBTAG00000046756	ENSBTAG00000046756	24:47788916-47788982	0	0	0	OK
ENSBTAG00000046758	ENSBTAG00000046758	16:52656950-52657700	0	0	0	OK
ENSBTAG00000046759	ENSBTAG00000046759	27:28427577-28427682	0	0	0	OK
ENSBTAG00000046761	ENSBTAG00000046761	5:60098739-60099837	0	0	0	OK
ENSBTAG00000046762	ENSBTAG00000046762	15:48687391-48687961	0	0	0	OK
ENSBTAG00000046763	ENSBTAG00000046763	22:43336992-43357321	0	0	0	OK
ENSBTAG00000046766	ENSBTAG00000046766	3:22441637-22443112	0	0	0	OK
ENSBTAG00000046767	ENSBTAG00000046767	GJ059486.1:45913-45985	0	0	0	OK
ENSBTAG00000046769	ENSBTAG00000046769	X:99507838-99509617	0	0	0	OK
ENSBTAG00000046770	ENSBTAG00000046770	X:103249226-103249315	0	0	0	OK
ENSBTAG00000046772	ENSBTAG00000046772	18:5244683-5253673	0	0	0	OK
ENSBTAG00000046774	ENSBTAG00000046774	X:92807956-92830879	0	0	0	OK
ENSBTAG00000046779	ENSBTAG00000046779	29:46591195-46591315	0	0	0	OK
ENSBTAG00000046780	ENSBTAG00000046780	21:1309834-1309954	0	0	0	OK
ENSBTAG00000046782	ENSBTAG00000046782	8:58256523-58257396	0	0	0	OK
ENSBTAG00000046783	ENSBTAG00000046783	2:94202054-94202354	0	0	0	OK
ENSBTAG00000046785	ENSBTAG00000046785	13:70491282-70491347	0	0	0	OK
ENSBTAG00000046787	ENSBTAG00000046787	7:43308300-43310539	0	0	0	OK
ENSBTAG00000046788	ENSBTAG00000046788	6:92136090-92136498	0	0	0	OK
ENSBTAG00000046790	ENSBTAG00000046790	1:83341311-83342875	0	0	0	OK
ENSBTAG00000046791	ENSBTAG00000046791	19:50853964-50854027	0	0	0	OK
ENSBTAG00000046793	ENSBTAG00000046793	4:106426050-106426877	0	0	0	OK

ENSBTAG00000046795	ENSBTAG00000046795	8:1172980-1173065	0	0	0	OK
ENSBTAG00000046798	ENSBTAG00000046798	7:11262123-11263101	0	0	0	OK
ENSBTAG00000046799	ENSBTAG00000046799	16:56530556-56532447	0	0	0	OK
ENSBTAG00000046800	ENSBTAG00000046800	5:120061385-120063155	0	0	0	OK
ENSBTAG00000046804	ENSBTAG00000046804	1:4036041-4036305	0	0	0	OK
ENSBTAG00000046806	ENSBTAG00000046806	X:79982281-79984425	0	0	0	OK
ENSBTAG00000046809	ENSBTAG00000046809	9:105686118-105690814	0	0	0	OK
ENSBTAG00000046810	ENSBTAG00000046810	20:13970055-13970540	0	0	0	OK
ENSBTAG00000046811	ENSBTAG00000046811	15:83864402-83865341	0	0	0	OK
ENSBTAG00000046812	ENSBTAG00000046812	3:100059949-100061720	0	0	0	OK
ENSBTAG00000046815	ENSBTAG00000046815	8:23045590-23046178	0	0	0	OK
ENSBTAG00000046816	ENSBTAG00000046816	15:50728983-50729949	0	0	0	OK
ENSBTAG00000046817	ENSBTAG00000046817	4:45475064-45475528	0	0	0	OK
ENSBTAG00000046818	ENSBTAG00000046818	X:9100432-9101239	0	0	0	OK
ENSBTAG00000046820	ENSBTAG00000046820	X:62152312-62152687	0	0	0	OK
ENSBTAG00000046821	ENSBTAG00000046821	14:81018672-81019595	0	0	0	OK
ENSBTAG00000046822	ENSBTAG00000046822	14:288464-288571	0	0	0	OK
ENSBTAG00000046824	ENSBTAG00000046824	8:22707572-22708142	0	0	0	OK
ENSBTAG00000046826	ENSBTAG00000046826	2:137027870-137027990	0	0	0	OK
ENSBTAG00000046828	ENSBTAG00000046828	21:71011579-71016820	0	0	0	OK
ENSBTAG00000046829	ENSBTAG00000046829	15:49355000-49355936	0	0	0	OK
ENSBTAG00000046831	ENSBTAG00000046831	2:90615055-90615159	0	0	0	OK
ENSBTAG00000046832	ENSBTAG00000046832	2:96865143-96865506	0	0	0	OK
ENSBTAG00000046833	ENSBTAG00000046833	17:37404575-37404821	0	0	0	OK
ENSBTAG00000046834	ENSBTAG00000046834	6:107740184-107740257	0	0	0	OK
ENSBTAG00000046842	ENSBTAG00000046842	17:64875705-64875846	0	0	0	OK
ENSBTAG00000046843	ENSBTAG00000046843	25:24227086-24227177	0	0	0	OK
ENSBTAG00000046847	ENSBTAG00000046847	14:38434807-38434891	0	0	0	OK
ENSBTAG00000046852	ENSBTAG00000046852	17:37253414-37253726	0	0	0	OK
ENSBTAG00000046854	ENSBTAG00000046854	1:62392320-62392881	0	0	0	OK

ENSBTAG00000046856	ENSBTAG00000046856	20:18039372-18039433	0	0	0	OK
ENSBTAG00000046857	ENSBTAG00000046857	21:71394346-71399231	0	0	0	OK
ENSBTAG00000046858	ENSBTAG00000046858	21:4987949-4988052	0	0	0	OK
ENSBTAG00000046859	ENSBTAG00000046859	1:144607394-144607589	0	0	0	OK
ENSBTAG00000046860	ENSBTAG00000046860	29:40742015-40742073	0	0	0	OK
ENSBTAG00000046863	ENSBTAG00000046863	26:12325285-12325402	0	0	0	OK
ENSBTAG00000046865	ENSBTAG00000046865	7:19209050-19209431	0	0	0	OK
ENSBTAG00000046867	ENSBTAG00000046867	7:105424553-105425499	0	0	0	OK
ENSBTAG00000046869	ENSBTAG00000046869	GJ060032.1:1986-8794	0	0	0	OK
ENSBTAG00000046873	ENSBTAG00000046873	7:64220246-64220506	0	0	0	OK
ENSBTAG00000046874	ENSBTAG00000046874	6:92540380-92540437	0	0	0	OK
ENSBTAG00000046876	ENSBTAG00000046876	25:1048610-1048737	0	0	0	OK
ENSBTAG00000046877	ENSBTAG00000046877	8:89663096-89663215	0	0	0	OK
ENSBTAG00000046878	ENSBTAG00000046878	2:26749066-26749423	0	0	0	OK
ENSBTAG00000046880	ENSBTAG00000046880	18:65053395-65053502	0	0	0	OK
ENSBTAG00000046881	ENSBTAG00000046881	23:7335322-7335432	0	0	0	OK
ENSBTAG00000046883	ENSBTAG00000046883	25:305836-305956	0	0	0	OK
ENSBTAG00000046884	ENSBTAG00000046884	5:60032487-60033426	0	0	0	OK
ENSBTAG00000046886	ENSBTAG00000046886	23:50390116-50394279	0	0	0	OK
ENSBTAG00000046887	ENSBTAG00000046887	17:65456340-65456721	0	0	0	OK
ENSBTAG00000046889	ENSBTAG00000046889	12:85610278-85610762	0	0	0	OK
ENSBTAG00000046890	ENSBTAG00000046890	23:34642065-34642174	0	0	0	OK
ENSBTAG00000046891	ENSBTAG00000046891	19:43848108-43848299	0	0	0	OK
ENSBTAG00000046893	ENSBTAG00000046893	3:93560775-93560854	0	0	0	OK
ENSBTAG00000046894	ENSBTAG00000046894	15:45240536-45241148	0	0	0	OK
ENSBTAG00000046895	ENSBTAG00000046895	X:44322143-44322244	0	0	0	OK
ENSBTAG00000046896	ENSBTAG00000046896	1:114670535-114670831	0	0	0	OK
ENSBTAG00000046897	ENSBTAG00000046897	10:23498101-23498662	0	0	0	OK
ENSBTAG00000046898	ENSBTAG00000046898	24:49139652-49139731	0	0	0	OK
ENSBTAG00000046899	ENSBTAG00000046899	X:3808288-3808376	0	0	0	OK

ENSBTAG00000046901	ENSBTAG00000046901	24:54256858-54258626	0	0	0	OK
ENSBTAG00000046903	ENSBTAG00000046903	28:25142734-25143338	0	0	0	OK
ENSBTAG00000046905	ENSBTAG00000046905	4:40541700-40542459	0	0	0	OK
ENSBTAG00000046906	ENSBTAG00000046906	10:21945868-21946789	0	0	0	OK
ENSBTAG00000046907	ENSBTAG00000046907	19:41967572-41968202	0	0	0	OK
ENSBTAG00000046913	ENSBTAG00000046913	15:49804639-49805584	0	0	0	OK
ENSBTAG00000046916	ENSBTAG00000046916	12:13335048-13335130	0	0	0	OK
ENSBTAG00000046920	ENSBTAG00000046920	23:26878521-26878592	0	0	0	OK
ENSBTAG00000046923	ENSBTAG00000046923	15:79602956-79605038	0	0	0	OK
ENSBTAG00000046926	ENSBTAG00000046926	7:51944663-51945231	0	0	0	OK
ENSBTAG00000046927	ENSBTAG00000046927	5:113271806-113272487	0	0	0	OK
ENSBTAG00000046929	ENSBTAG00000046929	26:50598823-50598943	0	0	0	OK
ENSBTAG00000046930	ENSBTAG00000046930	16:63239567-63239631	0	0	0	OK
ENSBTAG00000046931	ENSBTAG00000046931	1:61772398-61772809	0	0	0	OK
ENSBTAG00000046933	ENSBTAG00000046933	6:109121768-109121950	0	0	0	OK
ENSBTAG00000046935	ENSBTAG00000046935	1:69393953-69395622	0	0	0	OK
ENSBTAG00000046938	ENSBTAG00000046938	27:15439638-15462319	0	0	0	OK
ENSBTAG00000046943	ENSBTAG00000046943	25:38492937-38495056	0	0	0	OK
ENSBTAG00000046944	ENSBTAG00000046944	19:14602390-14605464	0	0	0	OK
ENSBTAG00000046946	ENSBTAG00000046946	16:4635437-4635507	0	0	0	OK
ENSBTAG00000046947	ENSBTAG00000046947	18:63922823-63923147	0	0	0	OK
ENSBTAG00000046955	ENSBTAG00000046955	6:107740075-107740148	0	0	0	OK
ENSBTAG00000046958	ENSBTAG00000046958	7:10906923-10908273	0	0	0	OK
ENSBTAG00000046959	ENSBTAG00000046959	1:35655411-35660398	0	0	0	OK
ENSBTAG00000046962	ENSBTAG00000046962	10:22552578-22553139	0	0	0	OK
ENSBTAG00000046964	ENSBTAG00000046964	14:30487542-30487663	0	0	0	OK
ENSBTAG00000046965	ENSBTAG00000046965	25:1017615-1022334	0	0	0	OK
ENSBTAG00000046968	ENSBTAG00000046968	15:74440699-74440796	0	0	0	OK
ENSBTAG00000046969	ENSBTAG00000046969	8:88647287-88648324	0	0	0	OK
ENSBTAG00000046970	ENSBTAG00000046970	10:27123659-27124592	0	0	0	OK

ENSBTAG00000046972	ENSBTAG00000046972	X:57493562-57493793	0	0	0	OK
ENSBTAG00000046973	ENSBTAG00000046973	15:83960647-83961584	0	0	0	OK
ENSBTAG00000046974	ENSBTAG00000046974	19:12622716-12623538	0	0	0	OK
ENSBTAG00000046975	ENSBTAG00000046975	19:14568541-14573104	0	0	0	OK
ENSBTAG00000046976	ENSBTAG00000046976	24:29988649-29988756	0	0	0	OK
ENSBTAG00000046977	ENSBTAG00000046977	2:133149364-133157295	0	0	0	OK
ENSBTAG00000046978	ENSBTAG00000046978	X:2195795-2198128	0	0	0	OK
ENSBTAG00000046984	ENSBTAG00000046984	7:43967503-43968901	0	0	0	OK
ENSBTAG00000046985	ENSBTAG00000046985	X:62939282-62939380	0	0	0	OK
ENSBTAG00000046986	ENSBTAG00000046986	17:51813719-51818775	0	0	0	OK
ENSBTAG00000046987	ENSBTAG00000046987	18:65988434-65989328	0	0	0	OK
ENSBTAG00000046988	ENSBTAG00000046988	2:132691771-132692743	0	0	0	OK
ENSBTAG00000046991	ENSBTAG00000046991	X:133787633-133787740	0	0	0	OK
ENSBTAG00000046992	ENSBTAG00000046992	23:31918886-31919276	0	0	0	OK
ENSBTAG00000046994	ENSBTAG00000046994	5:59061441-59062380	0	0	0	OK
ENSBTAG00000046995	ENSBTAG00000046995	10:25161286-25162115	0	0	0	OK
ENSBTAG00000046997	ENSBTAG00000046997	20:25631427-25632999	0	0	0	OK
ENSBTAG00000046998	ENSBTAG00000046998	X:110261027-110261872	0	0	0	OK
ENSBTAG00000046999	ENSBTAG00000046999	7:70814931-70815017	0	0	0	OK
ENSBTAG00000047001	ENSBTAG00000047001	10:23549338-23549910	0	0	0	OK
ENSBTAG00000047002	ENSBTAG00000047002	21:55336800-55352114	0	0	0	OK
ENSBTAG00000047004	ENSBTAG00000047004	9:20058719-20059216	0	0	0	OK
ENSBTAG00000047005	ENSBTAG00000047005	12:69593014-69593122	0	0	0	OK
ENSBTAG00000047006	ENSBTAG00000047006	11:89658574-89658861	0	0	0	OK
ENSBTAG00000047008	ENSBTAG00000047008	12:69574458-69575727	0	0	0	OK
ENSBTAG00000047009	ENSBTAG00000047009	23:19953076-19966930	0	0	0	OK
ENSBTAG00000047010	ENSBTAG00000047010	14:5596395-5596459	0	0	0	OK
ENSBTAG00000047012	ENSBTAG00000047012	1:53913504-53913597	0	0	0	OK
ENSBTAG00000047015	ENSBTAG00000047015	10:25111715-25112298	0	0	0	OK
ENSBTAG00000047017	ENSBTAG00000047017	7:8668210-8668318	0	0	0	OK

ENSBTAG00000047018	ENSBTAG00000047018	GJ058729.1:1655-1761	0	0	0	OK
ENSBTAG00000047019	ENSBTAG00000047019	X:139567422-139568354	0	0	0	OK
ENSBTAG00000047022	ENSBTAG00000047022	14:2854603-2856167	0	0	0	OK
ENSBTAG00000047023	ENSBTAG00000047023	19:43833326-43833517	0	0	0	OK
ENSBTAG00000047028	ENSBTAG00000047028	GJ059463.1:5714-5841	0	0	0	OK
ENSBTAG00000047032	ENSBTAG00000047032	9:409004-409418	0	0	0	OK
ENSBTAG00000047033	ENSBTAG00000047033	6:25804078-25804169	0	0	0	OK
ENSBTAG00000047034	ENSBTAG00000047034	2:2893419-2893497	0	0	0	OK
ENSBTAG00000047036	ENSBTAG00000047036	2:65526344-65527741	0	0	0	OK
ENSBTAG00000047037	ENSBTAG00000047037	9:61758629-61758717	0	0	0	OK
ENSBTAG00000047038	ENSBTAG00000047038	3:107513771-107515427	0	0	0	OK
ENSBTAG00000047039	ENSBTAG00000047039	23:27083668-27085420	0	0	0.376467	OK
ENSBTAG00000047041	ENSBTAG00000047041	10:22821327-22821865	0	0	0	OK
ENSBTAG00000047042	ENSBTAG00000047042	2:66273057-66273127	0	0	0	OK
ENSBTAG00000047044	ENSBTAG00000047044	21:2075525-2075618	0	0	0	OK
ENSBTAG00000047045	ENSBTAG00000047045	9:54749708-54750219	0	0	0	OK
ENSBTAG00000047046	ENSBTAG00000047046	3:35810480-35810572	0	0	0	OK
ENSBTAG00000047047	ENSBTAG00000047047	3:20750-21682	0	0	0	OK
ENSBTAG00000047050	ENSBTAG00000047050	25:39840012-39840088	0	0	0	OK
ENSBTAG00000047052	ENSBTAG00000047052	3:58436759-58436876	0	0	0	OK
ENSBTAG00000047053	ENSBTAG00000047053	18:60117463-60131456	0	0	0	OK
ENSBTAG00000047056	ENSBTAG00000047056	5:59296560-59297496	0	0	0	OK
ENSBTAG00000047057	ENSBTAG00000047057	23:34726691-34726796	0	0	0	OK
ENSBTAG00000047058	ENSBTAG00000047058	10:24680224-24680776	0	0	0	OK
ENSBTAG00000047060	ENSBTAG00000047060	19:54343818-54343903	0	0	0	OK
ENSBTAG00000047062	ENSBTAG00000047062	18:43834324-43846316	0	0	0	OK
ENSBTAG00000047063	ENSBTAG00000047063	3:10388029-10388959	0	0	0	OK
ENSBTAG00000047066	ENSBTAG00000047066	12:64197135-64197240	0	0	0	OK
ENSBTAG00000047067	ENSBTAG00000047067	10:68185329-68185436	0	0	0	OK
ENSBTAG00000047068	ENSBTAG00000047068	X:143378429-143385607	0	0	0	OK

ENSBTAG00000047070	ENSBTAG00000047070	15:29483437-29483544	0	0	0	OK
ENSBTAG00000047071	ENSBTAG00000047071	3:45832958-45833078	0	0	0	OK
ENSBTAG00000047073	ENSBTAG00000047073	16:70925148-70928253	0	0	0	OK
ENSBTAG00000047074	ENSBTAG00000047074	3:121410994-121411101	0	0	0	OK
ENSBTAG00000047075	ENSBTAG00000047075	19:58435867-58436204	0	0	0	OK
ENSBTAG00000047079	ENSBTAG00000047079	14:43280119-43280224	0	0	0	OK
ENSBTAG00000047084	ENSBTAG00000047084	17:64910530-64913110	0	0	0	OK
ENSBTAG00000047085	ENSBTAG00000047085	17:51374859-51380364	0	0	0	OK
ENSBTAG00000047086	ENSBTAG00000047086	23:29290571-29291495	0	0	0	OK
ENSBTAG00000047087	ENSBTAG00000047087	20:23931159-23937410	0	0	0	OK
ENSBTAG00000047090	ENSBTAG00000047090	29:26876565-26877501	0	0	0	OK
ENSBTAG00000047091	ENSBTAG00000047091	10:23407962-23408414	0	0	0	OK
ENSBTAG00000047092	ENSBTAG00000047092	X:143557039-143559418	0	0	0	OK
ENSBTAG00000047093	ENSBTAG00000047093	1:47062524-47063840	0	0	0	OK
ENSBTAG00000047094	ENSBTAG00000047094	29:23030371-23030521	0	0	0	OK
ENSBTAG00000047095	ENSBTAG00000047095	1:41997950-41998880	0	0	0	OK
ENSBTAG00000047096	ENSBTAG00000047096	10:24142587-24143051	0	0	0	OK
ENSBTAG00000047097	ENSBTAG00000047097	10:23631952-23632510	0	0	0	OK
ENSBTAG00000047099	ENSBTAG00000047099	8:60644674-60645634	0	0	0	OK
ENSBTAG00000047100	ENSBTAG00000047100	29:27896691-27897633	0	0	0	OK
ENSBTAG00000047103	ENSBTAG00000047103	8:78188625-78204330	0	0	0	OK
ENSBTAG00000047106	ENSBTAG00000047106	23:34103969-34104076	0	0	0	OK
ENSBTAG00000047108	ENSBTAG00000047108	X:37993669-37994022	0	0	0	OK
ENSBTAG00000047113	ENSBTAG00000047113	24:34579396-34581445	0	0	0	OK
ENSBTAG00000047115	ENSBTAG00000047115	27:40026280-40026379	0	0	0	OK
ENSBTAG00000047116	ENSBTAG00000047116	20:71565055-71570576	0	0	0	OK
ENSBTAG00000047117	ENSBTAG00000047117	23:34700911-34701021	0	0	0	OK
ENSBTAG00000047118	ENSBTAG00000047118	9:23781160-23791282	0	0	0.474357	OK
ENSBTAG00000047120	ENSBTAG00000047120	X:41124894-41126541	0	0	0	OK
ENSBTAG00000047121	ENSBTAG00000047121	17:73113556-73146553	0	0	0	OK

ENSBTAG00000047122	ENSBTAG00000047122	21:60891280-60891399	0	0	0	OK
ENSBTAG00000047123	ENSBTAG00000047123	6:8901323-8901445	0	0	0	OK
ENSBTAG00000047124	ENSBTAG00000047124	GJ059670.1:10970-18530	0	0	0	OK
ENSBTAG00000047126	ENSBTAG00000047126	23:34583867-34583976	0	0	0	OK
ENSBTAG00000047127	ENSBTAG00000047127	7:45498565-45502456	0	0	0	OK
ENSBTAG00000047128	ENSBTAG00000047128	18:13927667-13928674	0	0	0	OK
ENSBTAG00000047129	ENSBTAG00000047129	15:57234743-57235883	0	0	0	OK
ENSBTAG00000047131	ENSBTAG00000047131	7:79488955-79489035	0	0	0	OK
ENSBTAG00000047132	ENSBTAG00000047132	7:12764567-12765176	0	0	0	OK
ENSBTAG00000047136	ENSBTAG00000047136	14:26180763-26180919	0	0	0	OK
ENSBTAG00000047140	ENSBTAG00000047140	27:26217343-26217634	0	0	0	OK
ENSBTAG00000047142	ENSBTAG00000047142	8:23207798-23208359	0	0	0	OK
ENSBTAG00000047143	ENSBTAG00000047143	21:66651993-66652155	0	0	0	OK
ENSBTAG00000047144	ENSBTAG00000047144	1:98818164-98818518	0	0	0	OK
ENSBTAG00000047146	ENSBTAG00000047146	9:18971762-18972255	0	0	0	OK
ENSBTAG00000047149	ENSBTAG00000047149	11:59750644-59752481	0	0	0	OK
ENSBTAG00000047152	ENSBTAG00000047152	29:26838442-26838549	0	0	0	OK
ENSBTAG00000047153	ENSBTAG00000047153	X:84067696-84068405	0	0	0	OK
ENSBTAG00000047154	ENSBTAG00000047154	8:25409463-25409555	0	0	0	OK
ENSBTAG00000047157	ENSBTAG00000047157	4:61197028-61197085	0	0	0	OK
ENSBTAG00000047158	ENSBTAG00000047158	18:49288006-49294023	0	0	0	OK
ENSBTAG00000047160	ENSBTAG00000047160	11:29180293-29180371	0	0	0	OK
ENSBTAG00000047162	ENSBTAG00000047162	3:538284-538916	0	0	0	OK
ENSBTAG00000047163	ENSBTAG00000047163	25:39052115-39052198	0	0	4.98932	OK
ENSBTAG00000047166	ENSBTAG00000047166	21:30998213-31006059	0	0	0	OK
ENSBTAG00000047168	ENSBTAG00000047168	X:58269721-58271618	0	0	0	OK
ENSBTAG00000047170	ENSBTAG00000047170	10:65747189-65747567	0	0	0	OK
ENSBTAG00000047171	ENSBTAG00000047171	7:1529073-1529161	0	0	0	OK
ENSBTAG00000047173	ENSBTAG00000047173	8:19979270-19979389	0	0	0	OK
ENSBTAG00000047178	ENSBTAG00000047178	15:49571361-49572392	0	0	0	OK

ENSBTAG00000047179	ENSBTAG00000047179	5:9976744-9977709	0	0	0	OK
ENSBTAG00000047180	ENSBTAG00000047180	7:43119731-43120670	0	0	0	OK
ENSBTAG00000047183	ENSBTAG00000047183	1:20472811-20473172	0	0	0	OK
ENSBTAG00000047184	ENSBTAG00000047184	9:65241867-65241939	0	0	0	OK
ENSBTAG00000047185	ENSBTAG00000047185	11:47008474-47008921	0	0	0	OK
ENSBTAG00000047186	ENSBTAG00000047186	19:36214292-36217603	0	0	0	OK
ENSBTAG00000047189	ENSBTAG00000047189	3:134489-134615	0	0	0	OK
ENSBTAG00000047192	ENSBTAG00000047192	21:3690045-3690138	0	0	0	OK
ENSBTAG00000047193	ENSBTAG00000047193	4:106547469-106547892	0	0	0	OK
ENSBTAG00000047194	ENSBTAG00000047194	X:91391321-91391794	0	0	0	OK
ENSBTAG00000047196	ENSBTAG00000047196	18:50611275-50611827	0	0	0	OK
ENSBTAG00000047197	ENSBTAG00000047197	2:81373656-81373769	0	0	0	OK
ENSBTAG00000047199	ENSBTAG00000047199	5:29600736-29601755	0	0	0	OK
ENSBTAG00000047200	ENSBTAG00000047200	22:50608456-50609166	0	0	0	OK
ENSBTAG00000047201	ENSBTAG00000047201	12:7666812-7666965	0	0	0	OK
ENSBTAG00000047203	ENSBTAG00000047203	5:14721025-14748058	0	0	0	OK
ENSBTAG00000047204	ENSBTAG00000047204	29:27855406-27856318	0	0	0	OK
ENSBTAG00000047207	ENSBTAG00000047207	8:72265978-72266893	0	0	0	OK
ENSBTAG00000047208	ENSBTAG00000047208	20:37459223-37460183	0	0	0	OK
ENSBTAG00000047210	ENSBTAG00000047210	29:31113680-31114595	0	0	0	OK
ENSBTAG00000047211	ENSBTAG00000047211	X:57984882-57986035	0	0	0	OK
ENSBTAG00000047212	ENSBTAG00000047212	14:17734174-17748402	0	0	0	OK
ENSBTAG00000047219	ENSBTAG00000047219	18:47431024-47437065	0	0	0	OK
ENSBTAG00000047220	ENSBTAG00000047220	3:107619978-107620039	0	0	0	OK
ENSBTAG00000047221	ENSBTAG00000047221	8:7872801-7872895	0	0	0	OK
ENSBTAG00000047226	ENSBTAG00000047226	11:93121980-93138808	0	0	0	OK
ENSBTAG00000047227	ENSBTAG00000047227	28:26916376-26918507	0	0	0	OK
ENSBTAG00000047229	ENSBTAG00000047229	21:71390599-71392110	0	0	0	OK
ENSBTAG00000047231	ENSBTAG00000047231	16:49293851-49303508	0	0	0	OK
ENSBTAG00000047232	ENSBTAG00000047232	15:48752738-48753695	0	0	0	OK

ENSBTAG00000047233	ENSBTAG00000047233	16:30214934-30215409	0	0	0	OK
ENSBTAG00000047234	ENSBTAG00000047234	1:51727650-51727765	0	0	0	OK
ENSBTAG00000047236	ENSBTAG00000047236	18:63789244-63795222	0	0	0	OK
ENSBTAG00000047237	ENSBTAG00000047237	8:60492809-60493748	0	0	0	OK
ENSBTAG00000047240	ENSBTAG00000047240	21:54197038-54197308	0	0	0	OK
ENSBTAG00000047241	ENSBTAG00000047241	7:28498779-28498886	0	0	0	OK
ENSBTAG00000047242	ENSBTAG00000047242	15:41408727-41409024	0	0	0	OK
ENSBTAG00000047243	ENSBTAG00000047243	15:77016621-77016719	0	0	0	OK
ENSBTAG00000047245	ENSBTAG00000047245	19:51001352-51001415	0	0	0	OK
ENSBTAG00000047247	ENSBTAG00000047247	1:108086074-108086680	0	0	0	OK
ENSBTAG00000047248	ENSBTAG00000047248	3:63013692-63014043	0	0	0	OK
ENSBTAG00000047250	ENSBTAG00000047250	1:106878904-106879135	0	0	0	OK
ENSBTAG00000047252	ENSBTAG00000047252	21:2078253-2078346	0	0	0	OK
ENSBTAG00000047253	ENSBTAG00000047253	10:25641872-25642829	0	0	0	OK
ENSBTAG00000047257	ENSBTAG00000047257	8:68409845-68410140	0	0	0	OK
ENSBTAG00000047258	ENSBTAG00000047258	10:22631612-22632173	0	0	0	OK
ENSBTAG00000047259	ENSBTAG00000047259	5:59683718-59685168	0	0	0	OK
ENSBTAG00000047261	ENSBTAG00000047261	X:82120701-82120791	0	0	0	OK
ENSBTAG00000047263	ENSBTAG00000047263	3:56097407-56097492	0	0	0	OK
ENSBTAG00000047265	ENSBTAG00000047265	29:43163336-43163913	0	0	0	OK
ENSBTAG00000047266	ENSBTAG00000047266	25:35186321-35186681	0	0	0	OK
ENSBTAG00000047269	ENSBTAG00000047269	18:42198045-42198113	0	0	0	OK
ENSBTAG00000047273	ENSBTAG00000047273	5:49450263-49450390	0	0	0	OK
ENSBTAG00000047276	ENSBTAG00000047276	10:23157032-23157612	0	0	0	OK
ENSBTAG00000047277	ENSBTAG00000047277	18:46516556-46516985	0	0	0	OK
ENSBTAG00000047278	ENSBTAG00000047278	X:90791401-90793658	0	0	0	OK
ENSBTAG00000047279	ENSBTAG00000047279	X:99588578-99589450	0	0	0	OK
ENSBTAG00000047280	ENSBTAG00000047280	9:32815160-32815684	0	0	0	OK
ENSBTAG00000047286	ENSBTAG00000047286	4:46611813-46612625	0	0	0	OK
ENSBTAG00000047288	ENSBTAG00000047288	1:1675215-1675527	0	0	0	OK

ENSBTAG00000047289	ENSBTAG00000047289	GJ058256.1:80-200	0	0	0	OK
ENSBTAG00000047290	ENSBTAG00000047290	X:19517746-19519510	0	0	0	OK
ENSBTAG00000047291	ENSBTAG00000047291	20:12787056-12787175	0	0	0	OK
ENSBTAG00000047292	ENSBTAG00000047292	5:58597619-58598558	0	0	0	OK
ENSBTAG00000047296	ENSBTAG00000047296	19:8714870-8714961	0	0	0	OK
ENSBTAG00000047297	ENSBTAG00000047297	11:106387776-106389024	0	0	0	OK
ENSBTAG00000047299	ENSBTAG00000047299	17:74049771-74053881	0	0	0	OK
ENSBTAG00000047301	ENSBTAG00000047301	18:57618758-57622795	0	0	0	OK
ENSBTAG00000047303	ENSBTAG00000047303	14:24019277-24100855	0	0	0	OK
ENSBTAG00000047305	ENSBTAG00000047305	10:21920976-21921897	0	0	0	OK
ENSBTAG00000047306	ENSBTAG00000047306	10:22007816-22009402	0	0	0	OK
ENSBTAG00000047308	ENSBTAG00000047308	X:2791360-2791426	0	0	0	OK
ENSBTAG00000047309	ENSBTAG00000047309	9:27620128-27621291	0	0	0	OK
ENSBTAG00000047310	ENSBTAG00000047310	29:27957583-27958516	0	0	0	OK
ENSBTAG00000047311	ENSBTAG00000047311	10:27257685-27258612	0	0	0	OK
ENSBTAG00000047313	ENSBTAG00000047313	19:11490262-11492611	0	0	0	OK
ENSBTAG00000047315	ENSBTAG00000047315	13:24480937-24482339	0	0	0	OK
ENSBTAG00000047319	ENSBTAG00000047319	21:69533537-69534140	0	0	0	OK
ENSBTAG00000047320	ENSBTAG00000047320	13:50370105-50392917	0	0	0	OK
ENSBTAG00000047325	ENSBTAG00000047325	19:37995627-37998546	0	0	0	OK
ENSBTAG00000047328	ENSBTAG00000047328	21:1306298-1306417	0	0	0	OK
ENSBTAG00000047329	ENSBTAG00000047329	X:18750838-18750898	0	0	0	OK
ENSBTAG00000047330	ENSBTAG00000047330	14:46644608-46649827	0	0	0	OK
ENSBTAG00000047334	ENSBTAG00000047334	X:110906090-110906197	0	0	0	OK
ENSBTAG00000047335	ENSBTAG00000047335	6:89681429-89681497	0	0	0	OK
ENSBTAG00000047337	ENSBTAG00000047337	1:17397104-17397210	0	0	0	OK
ENSBTAG00000047343	ENSBTAG00000047343	12:19400263-19401145	0	0	0	OK
ENSBTAG00000047349	ENSBTAG00000047349	29:49781524-49781800	0	0	0	OK
ENSBTAG00000047352	ENSBTAG00000047352	16:57678381-57678450	0	0	0	OK
ENSBTAG00000047353	ENSBTAG00000047353	28:13245688-13245771	0	0	0	OK

ENSBTAG00000047354	ENSBTAG00000047354	5:32529438-32529956	0	0	0	OK
ENSBTAG00000047356	ENSBTAG00000047356	15:49339174-49340178	0	0	0	OK
ENSBTAG00000047358	ENSBTAG00000047358	5:29135857-29148172	0	0	0	OK
ENSBTAG00000047359	ENSBTAG00000047359	8:7995320-7995614	0	0	0	OK
ENSBTAG00000047360	ENSBTAG00000047360	12:74198711-74199129	0	0	0	OK
ENSBTAG00000047363	ENSBTAG00000047363	27:5327038-5328959	0	0	0	OK
ENSBTAG00000047365	ENSBTAG00000047365	2:43575859-43577277	0	0	0	OK
ENSBTAG00000047366	ENSBTAG00000047366	21:23871436-23872914	0	0	0	OK
ENSBTAG00000047367	ENSBTAG00000047367	18:39902860-39905155	0	0	0	OK
ENSBTAG00000047370	ENSBTAG00000047370	18:50716330-50716387	0	0	0	OK
ENSBTAG00000047372	ENSBTAG00000047372	4:72184681-72184793	0	0	0	OK
ENSBTAG00000047373	ENSBTAG00000047373	7:40166127-40166247	0	0	0	OK
ENSBTAG00000047376	ENSBTAG00000047376	X:83607425-83616158	0	0	0	OK
ENSBTAG00000047377	ENSBTAG00000047377	7:15655243-15659146	0	0	0	OK
ENSBTAG00000047378	ENSBTAG00000047378	8:29850752-29850826	0	0	0	OK
ENSBTAG00000047381	ENSBTAG00000047381	3:53471265-53471335	0	0	0	OK
ENSBTAG00000047382	ENSBTAG00000047382	2:127067056-127067144	0	0	0	OK
ENSBTAG00000047384	ENSBTAG00000047384	15:20577412-20578372	0	0	0	OK
ENSBTAG00000047386	ENSBTAG00000047386	2:97474799-97474905	0	0	0	OK
ENSBTAG00000047388	ENSBTAG00000047388	6:103614681-103614788	0	0	0	OK
ENSBTAG00000047390	ENSBTAG00000047390	12:46512878-46512947	0	0	0	OK
ENSBTAG00000047392	ENSBTAG00000047392	X:4347073-4347711	0	0	0	OK
ENSBTAG00000047393	ENSBTAG00000047393	15:54844146-54844253	0	0	0	OK
ENSBTAG00000047394	ENSBTAG00000047394	20:62003763-62003836	0	0	0	OK
ENSBTAG00000047395	ENSBTAG00000047395	16:66172055-66173159	0	0	0	OK
ENSBTAG00000047398	ENSBTAG00000047398	10:23778291-23779414	0	0	0	OK
ENSBTAG00000047403	ENSBTAG00000047403	2:2026956-2027063	0	0	0	OK
ENSBTAG00000047405	ENSBTAG00000047405	18:65798024-65798861	0	0	0	OK
ENSBTAG00000047407	ENSBTAG00000047407	28:13051113-13051204	0	0	0	OK
ENSBTAG00000047408	ENSBTAG00000047408	19:50684206-50684466	0	0	0	OK

ENSBTAG00000047409	ENSBTAG00000047409	21:14630145-14630559	0	0	0	OK
ENSBTAG00000047421	ENSBTAG00000047421	27:5160481-5162196	0	0	0	OK
ENSBTAG00000047422	ENSBTAG00000047422	1:57170509-57170617	0	0	0	OK
ENSBTAG00000047423	ENSBTAG00000047423	18:50856437-50858587	0	0	0	OK
ENSBTAG00000047425	ENSBTAG00000047425	15:8921977-8922841	0	0	0	OK
ENSBTAG00000047426	ENSBTAG00000047426	2:46723404-46759004	0	0	0	OK
ENSBTAG00000047427	ENSBTAG00000047427	27:11905037-11905288	0	0	0	OK
ENSBTAG00000047429	ENSBTAG00000047429	7:9520133-9521102	0	0	0	OK
ENSBTAG00000047430	ENSBTAG00000047430	13:20789980-20790062	0	0	0	OK
ENSBTAG00000047431	ENSBTAG00000047431	11:87176850-87176982	0	0	0	OK
ENSBTAG00000047432	ENSBTAG00000047432	6:103930899-103931310	0	0	0	OK
ENSBTAG00000047436	ENSBTAG00000047436	29:10733839-10734839	0	0	0	OK
ENSBTAG00000047437	ENSBTAG00000047437	16:33040838-33041352	0	0	0	OK
ENSBTAG00000047438	ENSBTAG00000047438	21:71472723-71472797	0	0	0	OK
ENSBTAG00000047441	ENSBTAG00000047441	22:13679509-13681231	0	0	0	OK
ENSBTAG00000047442	ENSBTAG00000047442	8:49499777-49516927	0	0	0	OK
ENSBTAG00000047444	ENSBTAG00000047444	10:22846083-22846644	0	0	0	OK
ENSBTAG00000047445	ENSBTAG00000047445	2:66272622-66272679	0	0	0	OK
ENSBTAG00000047446	ENSBTAG00000047446	17:66843951-66844021	0	0	0	OK
ENSBTAG00000047450	ENSBTAG00000047450	26:24945603-24949627	0	0	0	OK
ENSBTAG00000047451	ENSBTAG00000047451	21:67593791-67593898	0	0	0	OK
ENSBTAG00000047452	ENSBTAG00000047452	2:85799096-85800290	0	0	0	OK
ENSBTAG00000047454	ENSBTAG00000047454	13:29915525-29938309	0	0	0	OK
ENSBTAG00000047455	ENSBTAG00000047455	2:54612880-54613000	0	0	0	OK
ENSBTAG00000047456	ENSBTAG00000047456	12:78670670-78670777	0	0	0	OK
ENSBTAG00000047459	ENSBTAG00000047459	4:111541949-111542117	0	0	0	OK
ENSBTAG00000047460	ENSBTAG00000047460	18:29341667-29341822	0	0	0	OK
ENSBTAG00000047461	ENSBTAG00000047461	15:5313516-5463876	0	0	0	OK
ENSBTAG00000047467	ENSBTAG00000047467	2:129726529-129728612	0	0	0	OK
ENSBTAG00000047468	ENSBTAG00000047468	3:2867605-2868345	0	0	0	OK

ENSBTAG00000047469	ENSBTAG00000047469	X:143779554-143779660	0	0	0	OK
ENSBTAG00000047470	ENSBTAG00000047470	19:9810550-9810763	0	0	0	OK
ENSBTAG00000047472	ENSBTAG00000047472	15:84237710-84238682	0	0	0	OK
ENSBTAG00000047474	ENSBTAG00000047474	26:25812625-25813057	0	0	0	OK
ENSBTAG00000047475	ENSBTAG00000047475	11:49714024-49715693	0	0	0	OK
ENSBTAG00000047476	ENSBTAG00000047476	10:6286508-6286614	0	0	0	OK
ENSBTAG00000047477	ENSBTAG00000047477	15:49110531-49111470	0	0	0	OK
ENSBTAG00000047480	ENSBTAG00000047480	7:58629302-58629421	0	0	0	OK
ENSBTAG00000047481	ENSBTAG00000047481	8:59157000-59157070	0	0	0	OK
ENSBTAG00000047483	ENSBTAG00000047483	10:27081654-27082170	0	0	0	OK
ENSBTAG00000047484	ENSBTAG00000047484	17:60417161-60437117	0	0	0	OK
ENSBTAG00000047487	ENSBTAG00000047487	1:750706-750813	0	0	0	OK
ENSBTAG00000047490	ENSBTAG00000047490	2:99704434-99714269	0	0	0	OK
ENSBTAG00000047492	ENSBTAG00000047492	X:129071910-129072017	0	0	0	OK
ENSBTAG00000047494	ENSBTAG00000047494	X:126238632-126238711	0	0	0	OK
ENSBTAG00000047495	ENSBTAG00000047495	29:49842968-49848639	0	0	0	OK
ENSBTAG00000047496	ENSBTAG00000047496	3:119879389-119880190	0	0	0	OK
ENSBTAG00000047497	ENSBTAG00000047497	4:34171129-34172107	0	0	0	OK
ENSBTAG00000047500	ENSBTAG00000047500	15:50755825-50756771	0	0	0	OK
ENSBTAG00000047501	ENSBTAG00000047501	6:92538870-92538927	0	0	0	OK
ENSBTAG00000047503	ENSBTAG00000047503	11:1961072-1961481	0	0	0	OK
ENSBTAG00000047504	ENSBTAG00000047504	21:45406960-45407124	0	0	0	OK
ENSBTAG00000047505	ENSBTAG00000047505	18:53852956-53853373	0	0	0	OK
ENSBTAG00000047506	ENSBTAG00000047506	4:87632811-87632873	0	0	0	OK
ENSBTAG00000047508	ENSBTAG00000047508	18:62351041-62351560	0	0	0	OK
ENSBTAG00000047509	ENSBTAG00000047509	22:59627520-59642750	0	0	0	OK
ENSBTAG00000047510	ENSBTAG00000047510	19:56560581-56562005	0	0	0	OK
ENSBTAG00000047511	ENSBTAG00000047511	8:107410462-107471104	0	0	0	OK
ENSBTAG00000047515	ENSBTAG00000047515	19:41846175-41846472	0	0	0	OK
ENSBTAG00000047516	ENSBTAG00000047516	23:52431381-52432726	0	0	0	OK

ENSBTAG00000047517	ENSBTAG00000047517	22:50258588-50258665	0	0	0	OK
ENSBTAG00000047518	ENSBTAG00000047518	X:35613767-35614942	0	0	0	OK
ENSBTAG00000047520	ENSBTAG00000047520	25:13909187-13914702	0	0	0	OK
ENSBTAG00000047521	ENSBTAG00000047521	6:65458692-65458754	0	0	0	OK
ENSBTAG00000047522	ENSBTAG00000047522	10:23748172-23748752	0	0	0	OK
ENSBTAG00000047523	ENSBTAG00000047523	2:33990542-33990621	0	0	0	OK
ENSBTAG00000047526	ENSBTAG00000047526	17:74622500-74623699	0	0	0	OK
ENSBTAG00000047527	ENSBTAG00000047527	29:198198-198318	0	0	0	OK
ENSBTAG00000047529	ENSBTAG00000047529	17:72849359-72849927	0	0	0	OK
ENSBTAG00000047530	ENSBTAG00000047530	29:27330763-27331735	0	0	0	OK
ENSBTAG00000047532	ENSBTAG00000047532	14:73628948-73742529	0	0	0	OK
ENSBTAG00000047533	ENSBTAG00000047533	7:18573973-18574825	0	0	0	OK
ENSBTAG00000047535	ENSBTAG00000047535	28:32908770-32909599	0	0	0	OK
ENSBTAG00000047536	ENSBTAG00000047536	X:111483753-111486589	0	0	0	OK
ENSBTAG00000047540	ENSBTAG00000047540	19:21262114-21262183	0	0	0	OK
ENSBTAG00000047542	ENSBTAG00000047542	X:90561955-90562052	0	0	0	OK
ENSBTAG00000047544	ENSBTAG00000047544	8:22534796-22534890	0	0	0	OK
ENSBTAG00000047546	ENSBTAG00000047546	7:31173256-31173637	0	0	0	OK
ENSBTAG00000047549	ENSBTAG00000047549	X:69381842-69381949	0	0	0	OK
ENSBTAG00000047551	ENSBTAG00000047551	8:22607642-22634001	0	0	0	OK
ENSBTAG00000047552	ENSBTAG00000047552	15:817907-818000	0	0	0	OK
ENSBTAG00000047553	ENSBTAG00000047553	14:1116843-1116962	0	0	0	OK
ENSBTAG00000047554	ENSBTAG00000047554	7:59386957-59387051	0	0	0	OK
ENSBTAG00000047555	ENSBTAG00000047555	4:12724890-12725271	0	0	0	OK
ENSBTAG00000047560	ENSBTAG00000047560	7:44716585-44717848	0	0	0	OK
ENSBTAG00000047562	ENSBTAG00000047562	4:106485260-106485682	0	0	0	OK
ENSBTAG00000047563	ENSBTAG00000047563	25:2433849-2434503	0	0	0	OK
ENSBTAG00000047564	ENSBTAG00000047564	10:89305805-89305912	0	0	0	OK
ENSBTAG00000047565	ENSBTAG00000047565	25:3846-3966	0	0	0	OK
ENSBTAG00000047568	ENSBTAG00000047568	18:65076968-65078903	0	0	0	OK

ENSBTAG00000047570	ENSBTAG00000047570	1:110023856-110026785	0	0	0	OK
ENSBTAG00000047571	ENSBTAG00000047571	18:45438595-45440643	0	0	0	OK
ENSBTAG00000047573	ENSBTAG00000047573	15:15602647-15602734	0	0	0	OK
ENSBTAG00000047574	ENSBTAG00000047574	14:40529993-40530440	0	0	0	OK
ENSBTAG00000047576	ENSBTAG00000047576	7:11324605-11325992	0	0	0	OK
ENSBTAG00000047577	ENSBTAG00000047577	X:39325156-39331488	0	0	0	OK
ENSBTAG00000047583	ENSBTAG00000047583	X:1969594-1969679	0	0	0	OK
ENSBTAG00000047584	ENSBTAG00000047584	3:21188028-21188192	0	0	0	OK
ENSBTAG00000047587	ENSBTAG00000047587	21:26811824-26837752	0	0	0	OK
ENSBTAG00000047588	ENSBTAG00000047588	20:60955355-60955448	0	0	0	OK
ENSBTAG00000047589	ENSBTAG00000047589	7:6703831-6705051	0	0	0	OK
ENSBTAG00000047590	ENSBTAG00000047590	1:84255522-84255653	0	0	0	OK
ENSBTAG00000047592	ENSBTAG00000047592	4:114825311-114825795	0	0	0	OK
ENSBTAG00000047593	ENSBTAG00000047593	1:142497253-142497366	0	0	0	OK
ENSBTAG00000047597	ENSBTAG00000047597	17:51217468-51219592	0	0	0	OK
ENSBTAG00000047602	ENSBTAG00000047602	18:61635153-61636026	0	0	0	OK
ENSBTAG00000047603	ENSBTAG00000047603	16:415780-416614	0	0	0	OK
ENSBTAG00000047607	ENSBTAG00000047607	14:38761880-38762441	0	0	0	OK
ENSBTAG00000047614	ENSBTAG00000047614	29:1881123-1882754	0	0	0	OK
ENSBTAG00000047618	ENSBTAG00000047618	4:54132249-54132360	0	0	0	OK
ENSBTAG00000047620	ENSBTAG00000047620	X:4092795-4092893	0	0	0	OK
ENSBTAG00000047621	ENSBTAG00000047621	16:4998444-5005553	0	0	0	OK
ENSBTAG00000047622	ENSBTAG00000047622	10:2099896-2099968	0	0	0	OK
ENSBTAG00000047623	ENSBTAG00000047623	19:39880554-39880651	0	0	0	OK
ENSBTAG00000047624	ENSBTAG00000047624	10:24105130-24105633	0	0	0	OK
ENSBTAG00000047626	ENSBTAG00000047626	17:75054451-75056174	0	0	0	OK
ENSBTAG00000047627	ENSBTAG00000047627	7:15981625-15981691	0	0	0	OK
ENSBTAG00000047629	ENSBTAG00000047629	5:113266462-113269283	0	0	0	OK
ENSBTAG00000047632	ENSBTAG00000047632	21:71468375-71469950	0	0	0	OK
ENSBTAG00000047636	ENSBTAG00000047636	7:90531549-90532067	0	0	0	OK

ENSBTAG00000047637	ENSBTAG00000047637	11:46404610-46404925	0	0	0	OK
ENSBTAG00000047638	ENSBTAG00000047638	13:50369149-50386926	0	0	0	OK
ENSBTAG00000047639	ENSBTAG00000047639	10:24115720-24116278	0	0	0	OK
ENSBTAG00000047640	ENSBTAG00000047640	21:67582089-67582219	0	0	0	OK
ENSBTAG00000047641	ENSBTAG00000047641	28:2975210-2975284	0	0	0	OK
ENSBTAG00000047642	ENSBTAG00000047642	26:42208421-42209072	0	0	0	OK
ENSBTAG00000047645	ENSBTAG00000047645	10:29927783-29933155	0	0	0	OK
ENSBTAG00000047648	ENSBTAG00000047648	16:71149230-71153633	0	0	0	OK
ENSBTAG00000047651	ENSBTAG00000047651	1:94556234-94556385	0	0	0	OK
ENSBTAG00000047653	ENSBTAG00000047653	13:50375753-50380459	0	0	0	OK
ENSBTAG00000047656	ENSBTAG00000047656	14:12227908-12227978	0	0	0	OK
ENSBTAG00000047657	ENSBTAG00000047657	4:106499947-106500417	0	0	0	OK
ENSBTAG00000047659	ENSBTAG00000047659	11:2390343-2390799	0	0	0	OK
ENSBTAG00000047660	ENSBTAG00000047660	7:61882215-61882563	0	0	0	OK
ENSBTAG00000047661	ENSBTAG00000047661	14:46883461-46891306	0	0	0	OK
ENSBTAG00000047664	ENSBTAG00000047664	17:73812706-73828749	0	0	0.472733	OK
ENSBTAG00000047667	ENSBTAG00000047667	19:50848868-50849924	0	0	0	OK
ENSBTAG00000047668	ENSBTAG00000047668	7:21583268-21585597	0	0	0	OK
ENSBTAG00000047669	ENSBTAG00000047669	1:125812466-125812573	0	0	0	OK
ENSBTAG00000047670	ENSBTAG00000047670	18:44305868-44378890	0	0	0	OK
ENSBTAG00000047673	ENSBTAG00000047673	25:18566259-18570516	0	0	0	OK
ENSBTAG00000047674	ENSBTAG00000047674	1:23767707-23767826	0	0	0	OK
ENSBTAG00000047676	ENSBTAG00000047676	17:74439063-74456082	0	0	0	OK
ENSBTAG00000047678	ENSBTAG00000047678	13:72854906-72854978	0	0	0	OK
ENSBTAG00000047682	ENSBTAG00000047682	28:13282644-13282727	0	0	0	OK
ENSBTAG00000047683	ENSBTAG00000047683	10:23470078-23470639	0	0	0	OK
ENSBTAG00000047684	ENSBTAG00000047684	4:83419663-83420214	0	0	0	OK
ENSBTAG00000047686	ENSBTAG00000047686	9:74021312-74021407	0	0	0	OK
ENSBTAG00000047688	ENSBTAG00000047688	X:140516897-140517280	0	0	0	OK
ENSBTAG00000047690	ENSBTAG00000047690	10:24282905-24283878	0	0	0	OK

ENSBTAG00000047692	ENSBTAG00000047692	26:4113516-4113586	0	0	0	OK
ENSBTAG00000047693	ENSBTAG00000047693	11:93290981-93291917	0	0	0	OK
ENSBTAG00000047694	ENSBTAG00000047694	21:55966631-55968362	0	0	0	OK
ENSBTAG00000047695	ENSBTAG00000047695	7:971808-972995	0	0	0	OK
ENSBTAG00000047700	ENSBTAG00000047700	21:71453626-71454755	0	0	0	OK
ENSBTAG00000047702	ENSBTAG00000047702	X:40058808-40063513	0	0	0	OK
ENSBTAG00000047703	ENSBTAG00000047703	16:12657121-12657935	0	0	0	OK
ENSBTAG00000047704	ENSBTAG00000047704	13:54247556-54249072	0	0	0	OK
ENSBTAG00000047706	ENSBTAG00000047706	27:13339705-13346831	0	0	0	OK
ENSBTAG00000047707	ENSBTAG00000047707	23:7054418-7063641	0	0	0	OK
ENSBTAG00000047708	ENSBTAG00000047708	X:33639091-33646559	0	0	0	OK
ENSBTAG00000047710	ENSBTAG00000047710	21:53268932-53269714	0	0	0	OK
ENSBTAG00000047711	ENSBTAG00000047711	X:4064718-4065528	0	0	0	OK
ENSBTAG00000047714	ENSBTAG00000047714	15:50747287-50748235	0	0	0	OK
ENSBTAG00000047720	ENSBTAG00000047720	23:2508843-2508950	0	0	0	OK
ENSBTAG00000047723	ENSBTAG00000047723	19:6736680-6736750	0	0	0	OK
ENSBTAG00000047724	ENSBTAG00000047724	18:45562956-45564333	0	0	0	OK
ENSBTAG00000047725	ENSBTAG00000047725	4:106491636-106492130	0	0	0	OK
ENSBTAG00000047727	ENSBTAG00000047727	16:2897227-2899884	0	0	0	OK
ENSBTAG00000047729	ENSBTAG00000047729	21:20082489-20103922	0	0	0	OK
ENSBTAG00000047730	ENSBTAG00000047730	29:27963586-27964956	0	0	0	OK
ENSBTAG00000047732	ENSBTAG00000047732	21:3697063-3697156	0	0	0	OK
ENSBTAG00000047733	ENSBTAG00000047733	5:58050315-58051245	0	0	0	OK
ENSBTAG00000047735	ENSBTAG00000047735	6:48036645-48036732	0	0	0	OK
ENSBTAG00000047736	ENSBTAG00000047736	23:29572203-29573142	0	0	0	OK
ENSBTAG00000047737	ENSBTAG00000047737	X:40752943-40753240	0	0	0	OK
ENSBTAG00000047738	ENSBTAG00000047738	15:18705270-18705396	0	0	0	OK
ENSBTAG00000047741	ENSBTAG00000047741	5:65367267-65368393	0	0	0	OK
ENSBTAG00000047742	ENSBTAG00000047742	5:34837192-34837274	0	0	0	OK
ENSBTAG00000047746	ENSBTAG00000047746	10:25636814-25637771	0	0	0	OK

ENSBTAG00000047747	ENSBTAG00000047747	7:55061327-55085013	0	0	0	OK
ENSBTAG00000047750	ENSBTAG00000047750	3:53471378-53471425	0	0	0	OK
ENSBTAG00000047751	ENSBTAG00000047751	X:143667399-143667506	0	0	0	OK
ENSBTAG00000047754	ENSBTAG00000047754	8:59157923-59157993	0	0	0	OK
ENSBTAG00000047757	ENSBTAG00000047757	X:3536249-3537654	0	0	0	OK
ENSBTAG00000047759	ENSBTAG00000047759	8:111441734-111441802	0	0	0	OK
ENSBTAG00000047762	ENSBTAG00000047762	26:50444281-50444422	0	0	0	OK
ENSBTAG00000047763	ENSBTAG00000047763	23:29032405-29033332	0	0	0	OK
ENSBTAG00000047765	ENSBTAG00000047765	17:74676729-74677594	0	0	0	OK
ENSBTAG00000047766	ENSBTAG00000047766	16:75539495-75540404	0	0	0	OK
ENSBTAG00000047768	ENSBTAG00000047768	X:79540624-79544590	0	0	0	OK
ENSBTAG00000047769	ENSBTAG00000047769	21:71457044-71457122	0	0	0	OK
ENSBTAG00000047772	ENSBTAG00000047772	11:66837192-66838229	0	0	0	OK
ENSBTAG00000047773	ENSBTAG00000047773	13:59417326-59417442	0	0	0	OK
ENSBTAG00000047774	ENSBTAG00000047774	X:143948521-143953690	0	0	0	OK
ENSBTAG00000047775	ENSBTAG00000047775	23:29994765-29994872	0	0	0	OK
ENSBTAG00000047777	ENSBTAG00000047777	19:11622862-11623428	0	0	0	OK
ENSBTAG00000047778	ENSBTAG00000047778	2:54116504-54116624	0	0	0	OK
ENSBTAG00000047779	ENSBTAG00000047779	13:5424636-5425326	0	0	0	OK
ENSBTAG00000047781	ENSBTAG00000047781	25:39308650-39309366	0	0	0	OK
ENSBTAG00000047783	ENSBTAG00000047783	2:81639370-81640236	0	0	0	OK
ENSBTAG00000047785	ENSBTAG00000047785	11:98474174-98474240	0	0	0	OK
ENSBTAG00000047787	ENSBTAG00000047787	18:39393579-39393972	0	0	0	OK
ENSBTAG00000047789	ENSBTAG00000047789	23:29878489-29878784	0	0	0	OK
ENSBTAG00000047790	ENSBTAG00000047790	2:66273170-66273227	0	0	0	OK
ENSBTAG00000047791	ENSBTAG00000047791	18:58617314-58618271	0	0	0	OK
ENSBTAG00000047792	ENSBTAG00000047792	14:356331-356451	0	0	0	OK
ENSBTAG00000047793	ENSBTAG00000047793	29:49836431-49840072	0	0	0	OK
ENSBTAG00000047795	ENSBTAG00000047795	17:74987640-74987728	0	0	0	OK
ENSBTAG00000047796	ENSBTAG00000047796	7:8009653-8010628	0	0	0	OK

ENSBTAG00000047797	ENSBTAG00000047797	19:18811424-18811513	0	0	0	OK
ENSBTAG00000047798	ENSBTAG00000047798	6:64750380-64750486	0	0	0	OK
ENSBTAG00000047800	ENSBTAG00000047800	14:493875-493995	0	0	0	OK
ENSBTAG00000047803	ENSBTAG00000047803	15:51071882-51073006	0	0	0	OK
ENSBTAG00000047805	ENSBTAG00000047805	17:74022979-74024014	0	0	0	OK
ENSBTAG00000047807	ENSBTAG00000047807	11:106168108-106172938	0	0	0	OK
ENSBTAG00000047808	ENSBTAG00000047808	25:35930031-35940138	0	0	0	OK
ENSBTAG00000047809	ENSBTAG00000047809	24:47271782-47297664	0	0	0	OK
ENSBTAG00000047811	ENSBTAG00000047811	29:44583535-44584162	0	0	2.22642	OK
ENSBTAG00000047813	ENSBTAG00000047813	3:119926497-119927582	0	0	0	OK
ENSBTAG00000047814	ENSBTAG00000047814	6:56819195-56819900	0	0	0	OK
ENSBTAG00000047818	ENSBTAG00000047818	5:51991216-52115320	0	0	0	OK
ENSBTAG00000047820	ENSBTAG00000047820	19:13707020-13707088	0	0	0	OK
ENSBTAG00000047821	ENSBTAG00000047821	6:33473221-33473374	0	0	0	OK
ENSBTAG00000047824	ENSBTAG00000047824	16:31136170-31137374	0	0	0	OK
ENSBTAG00000047826	ENSBTAG00000047826	18:51458326-51458394	0	0	0	OK
ENSBTAG00000047830	ENSBTAG00000047830	3:84031307-84033450	0	0	0	OK
ENSBTAG00000047832	ENSBTAG00000047832	17:52348778-52348836	0	0	0	OK
ENSBTAG00000047834	ENSBTAG00000047834	14:10171319-10250757	0	0	0	OK
ENSBTAG00000047835	ENSBTAG00000047835	7:61034481-61036502	0	0	0	OK
ENSBTAG00000047836	ENSBTAG00000047836	8:64586966-64587266	0	0	0	OK
ENSBTAG00000047838	ENSBTAG00000047838	3:53472422-53472479	0	0	0	OK
ENSBTAG00000047840	ENSBTAG00000047840	29:27845518-27846536	0	0	0	OK
ENSBTAG00000047841	ENSBTAG00000047841	1:81854672-81855114	0	0	0	OK
ENSBTAG00000047844	ENSBTAG00000047844	4:106539760-106540179	0	0	0	OK
ENSBTAG00000047848	ENSBTAG00000047848	5:102201490-102202286	0	0	0	OK
ENSBTAG00000047849	ENSBTAG00000047849	2:89699628-89699997	0	0	0	OK
ENSBTAG00000047851	ENSBTAG00000047851	22:35066535-35066688	0	0	0	OK
ENSBTAG00000047852	ENSBTAG00000047852	3:53472309-53472379	0	0	0	OK
ENSBTAG00000047853	ENSBTAG00000047853	26:331216-332026	0	0	0	OK

ENSBTAG00000047854	ENSBTAG00000047854	21:16563597-16563698	0	0	0	OK
ENSBTAG00000047858	ENSBTAG00000047858	19:25901824-25902636	0	0	0	OK
ENSBTAG00000047859	ENSBTAG00000047859	19:6397623-6397708	0	0	0	OK
ENSBTAG00000047861	ENSBTAG00000047861	X:135102418-135107595	0	0	0	OK
ENSBTAG00000047862	ENSBTAG00000047862	2:85132986-85133945	0	0	0	OK
ENSBTAG00000047863	ENSBTAG00000047863	4:69458129-69458416	0	0	0	OK
ENSBTAG00000047864	ENSBTAG00000047864	21:52176460-52176532	0	0	0	OK
ENSBTAG00000047865	ENSBTAG00000047865	21:3611983-3612057	0	0	0	OK
ENSBTAG00000047866	ENSBTAG00000047866	19:48447144-48447444	0	0	0	OK
ENSBTAG00000047867	ENSBTAG00000047867	X:56483671-56484314	0	0	0	OK
ENSBTAG00000047870	ENSBTAG00000047870	19:9252859-9253795	0	0	0	OK
ENSBTAG00000047875	ENSBTAG00000047875	19:49332529-49332630	0	0	0	OK
ENSBTAG00000047878	ENSBTAG00000047878	X:29112773-29112890	0	0	0	OK
ENSBTAG00000047880	ENSBTAG00000047880	2:31688428-31699171	0	0	0	OK
ENSBTAG00000047882	ENSBTAG00000047882	6:85-192	0	0	0	OK
ENSBTAG00000047884	ENSBTAG00000047884	X:58997459-58998076	0	0	0	OK
ENSBTAG00000047887	ENSBTAG00000047887	12:89870159-89870234	0	0	0	OK
ENSBTAG00000047889	ENSBTAG00000047889	21:67604364-67604474	0	0	0	OK
ENSBTAG00000047894	ENSBTAG00000047894	13:68195056-68199035	0	0	0	OK
ENSBTAG00000047895	ENSBTAG00000047895	7:16149562-16149665	0	0	0	OK
ENSBTAG00000047896	ENSBTAG00000047896	17:74924019-74924274	0	0	0	OK
ENSBTAG00000047898	ENSBTAG00000047898	X:67203798-67203868	0	0	0	OK
ENSBTAG00000047899	ENSBTAG00000047899	5:112286610-112287081	0	0	0	OK
ENSBTAG00000047901	ENSBTAG00000047901	21:67449938-67450059	0	0	0	OK
ENSBTAG00000047903	ENSBTAG00000047903	15:57729265-57729401	0	0	0	OK
ENSBTAG00000047905	ENSBTAG00000047905	21:45295074-45341768	0	0	0	OK
ENSBTAG00000047906	ENSBTAG00000047906	20:70995209-70996377	0	0	0	OK
ENSBTAG00000047909	ENSBTAG00000047909	10:23727118-23727821	0	0	0	OK
ENSBTAG00000047911	ENSBTAG00000047911	23:2596382-2596946	0	0	0	OK
ENSBTAG00000047913	ENSBTAG00000047913	2:66272513-66272580	0	0	0	OK

ENSBTAG00000047915	ENSBTAG00000047915	9:82961183-82961276	0	0	0	OK
ENSBTAG00000047918	ENSBTAG00000047918	18:50715310-50715367	0	0	0	OK
ENSBTAG00000047920	ENSBTAG00000047920	5:57999925-58000852	0	0	0	OK
ENSBTAG00000047922	ENSBTAG00000047922	4:106501805-106502222	0	0	0	OK
ENSBTAG00000047923	ENSBTAG00000047923	21:3678568-3678661	0	0	0	OK
ENSBTAG00000047928	ENSBTAG00000047928	28:24126762-24126888	0	0	0	OK
ENSBTAG00000047929	ENSBTAG00000047929	29:5293485-5293594	0	0	0	OK
ENSBTAG00000047930	ENSBTAG00000047930	27:42203625-42288998	0	0	0	OK
ENSBTAG00000047931	ENSBTAG00000047931	5:58161351-58162290	0	0	0	OK
ENSBTAG00000047937	ENSBTAG00000047937	18:1878528-1881664	0	0	0	OK
ENSBTAG00000047938	ENSBTAG00000047938	18:26859232-26859315	0	0	0	OK
ENSBTAG00000047940	ENSBTAG00000047940	X:10599368-10599821	0	0	0	OK
ENSBTAG00000047941	ENSBTAG00000047941	8:73225644-73225751	0	0	0	OK
ENSBTAG00000047943	ENSBTAG00000047943	1:18902909-18904544	0	0	0	OK
ENSBTAG00000047947	ENSBTAG00000047947	8:100352337-100352634	0	0	0	OK
ENSBTAG00000047950	ENSBTAG00000047950	11:105132278-105132398	0	0	0	OK
ENSBTAG00000047951	ENSBTAG00000047951	20:18197999-18198163	0	0	0	OK
ENSBTAG00000047952	ENSBTAG00000047952	10:24111826-24112287	0	0	0	OK
ENSBTAG00000047959	ENSBTAG00000047959	21:71456964-71457042	0	0	0	OK
ENSBTAG00000047960	ENSBTAG00000047960	15:50040919-50041904	0	0	0	OK
ENSBTAG00000047964	ENSBTAG00000047964	11:105918625-105920031	0	0	0	OK
ENSBTAG00000047965	ENSBTAG00000047965	22:11122581-11124014	0	0	0	OK
ENSBTAG00000047967	ENSBTAG00000047967	5:59678029-59678974	0	0	0	OK
ENSBTAG00000047968	ENSBTAG00000047968	15:82375830-82377891	0	0	0	OK
ENSBTAG00000047970	ENSBTAG00000047970	21:71403464-71404530	0	0	0	OK
ENSBTAG00000047971	ENSBTAG00000047971	X:36572544-36577772	0	0	0	OK
ENSBTAG00000047972	ENSBTAG00000047972	16:81368725-81379604	0	0	0	OK
ENSBTAG00000047975	ENSBTAG00000047975	13:82696137-82803966	0	0	0	OK
ENSBTAG00000047976	ENSBTAG00000047976	11:88922157-88922235	0	0	0	OK
ENSBTAG00000047978	ENSBTAG00000047978	1:158298804-158299185	0	0	0	OK

ENSBTAG00000047979	ENSBTAG00000047979	4:117153119-117411239	0	0	0	OK
ENSBTAG00000047980	ENSBTAG00000047980	5:58207647-58208574	0	0	0	OK
ENSBTAG00000047981	ENSBTAG00000047981	10:23577089-23577668	0	0	0	OK
ENSBTAG00000047983	ENSBTAG00000047983	5:69189810-69192111	0	0	0	OK
ENSBTAG00000047987	ENSBTAG00000047987	13:80553197-80553283	0	0	0	OK
ENSBTAG00000047989	ENSBTAG00000047989	29:38920417-38929107	0	0	0	OK
ENSBTAG00000047992	ENSBTAG00000047992	19:27121210-27121973	0	0	0	OK
ENSBTAG00000047993	ENSBTAG00000047993	6:69904264-69904343	0	0	0	OK
ENSBTAG00000047995	ENSBTAG00000047995	23:17525619-17525724	0	0	0	OK
ENSBTAG00000047997	ENSBTAG00000047997	X:141075571-141085602	0	0	0	OK
ENSBTAG00000048000	ENSBTAG00000048000	18:50716430-50716500	0	0	0	OK
ENSBTAG00000048002	ENSBTAG00000048002	21:14484198-14484581	0	0	0	OK
ENSBTAG00000048005	ENSBTAG00000048005	18:64216860-64229275	0	0	0	OK
ENSBTAG00000048008	ENSBTAG00000048008	4:89234911-89235796	0	0	0	OK
ENSBTAG00000048009	ENSBTAG00000048009	13:61501525-61501651	0	0	0	OK
ENSBTAG00000048010	ENSBTAG00000048010	21:71160885-71167980	0	0	0	OK
ENSBTAG00000048011	ENSBTAG00000048011	1:98071468-98072479	0	0	0	OK
ENSBTAG00000048012	ENSBTAG00000048012	4:106327729-106328348	0	0	0	OK
ENSBTAG00000048018	ENSBTAG00000048018	29:28255645-28256799	0	0	0	OK
ENSBTAG00000048023	ENSBTAG00000048023	15:43731787-43735570	0	0	0.821655	OK
ENSBTAG00000048026	ENSBTAG00000048026	14:53855845-53855965	0	0	0	OK
ENSBTAG00000048027	ENSBTAG00000048027	X:15164513-15164620	0	0	0	OK
ENSBTAG00000048028	ENSBTAG00000048028	X:48483484-48483610	0	0	0	OK
ENSBTAG00000048031	ENSBTAG00000048031	15:79293429-79294440	0	0	0	OK
ENSBTAG00000048035	ENSBTAG00000048035	2:73424378-73424446	0	0	0	OK
ENSBTAG00000048036	ENSBTAG00000048036	28:30008048-30008387	0	0	0	OK
ENSBTAG00000048038	ENSBTAG00000048038	X:2676172-2676268	0	0	0	OK
ENSBTAG00000048039	ENSBTAG00000048039	2:54615878-54615998	0	0	0	OK
ENSBTAG00000048040	ENSBTAG00000048040	14:37989628-37990502	0	0	0	OK
ENSBTAG00000048041	ENSBTAG00000048041	11:2929638-2943356	0	0	0	OK

ENSBTAG00000048042	ENSBTAG00000048042	21:49526701-49532179	0	0	0	OK
ENSBTAG00000048045	ENSBTAG00000048045	15:45189117-45189730	0	0	0	OK
ENSBTAG00000048046	ENSBTAG00000048046	9:10869561-10869854	0	0	0	OK
ENSBTAG00000048047	ENSBTAG00000048047	13:15207088-15208538	0	0	0	OK
ENSBTAG00000048048	ENSBTAG00000048048	21:6101144-6102636	0	0	0	OK
ENSBTAG00000048051	ENSBTAG00000048051	X:58514389-58516607	0	0	0	OK
ENSBTAG00000048055	ENSBTAG00000048055	13:71232642-71233124	0	0	0	OK
ENSBTAG00000048058	ENSBTAG00000048058	10:4608188-4608558	0	0	0	OK
ENSBTAG00000048064	ENSBTAG00000048064	10:23202965-23208280	0	0	0	OK
ENSBTAG00000048065	ENSBTAG00000048065	11:86510269-86510581	0	0	0	OK
ENSBTAG00000048066	ENSBTAG00000048066	7:10430012-10431363	0	0	0	OK
ENSBTAG00000048067	ENSBTAG00000048067	19:39912108-39913248	0	0	0	OK
ENSBTAG00000048069	ENSBTAG00000048069	7:42190888-42191833	0	0	0	OK
ENSBTAG00000048070	ENSBTAG00000048070	11:70872263-70872348	0	0	0	OK
ENSBTAG00000048075	ENSBTAG00000048075	18:52732799-52747249	0	0	0	OK
ENSBTAG00000048076	ENSBTAG00000048076	21:55746780-55746877	0	0	0	OK
ENSBTAG00000048080	ENSBTAG00000048080	X:64079843-64080463	0	0	0	OK
ENSBTAG00000048085	ENSBTAG00000048085	19:8678913-8680081	0	0	0	OK
ENSBTAG00000048091	ENSBTAG00000048091	11:103283460-103287512	0	0	0	OK
ENSBTAG00000048093	ENSBTAG00000048093	18:8166055-8166120	0	0	0	OK
ENSBTAG00000048097	ENSBTAG00000048097	4:110971235-110971427	0	0	0	OK
ENSBTAG00000048100	ENSBTAG00000048100	18:51638902-51638992	0	0	0	OK
ENSBTAG00000048101	ENSBTAG00000048101	21:67516937-67517012	0	0	0	OK
ENSBTAG00000048102	ENSBTAG00000048102	X:143736737-143800954	0	0	0	OK
ENSBTAG00000048105	ENSBTAG00000048105	20:47189231-47190249	0	0	0	OK
ENSBTAG00000048108	ENSBTAG00000048108	X:47585522-47585896	0	0	0	OK
ENSBTAG00000048110	ENSBTAG00000048110	1:138151817-138151889	0	0	0	OK
ENSBTAG00000048111	ENSBTAG00000048111	3:61303496-61303615	0	0	0	OK
ENSBTAG00000048113	ENSBTAG00000048113	21:36134506-36134571	0	0	0	OK
ENSBTAG00000048116	ENSBTAG00000048116	23:14510928-14511022	0	0	0	OK

ENSBTAG00000048117	ENSBTAG00000048117	6:107740563-107740636	0	0	0	OK
ENSBTAG00000048119	ENSBTAG00000048119	6:108909399-108910432	0	0	0	OK
ENSBTAG00000048120	ENSBTAG00000048120	3:34060768-34060962	0	0	0	OK
ENSBTAG00000048121	ENSBTAG00000048121	21:2130285-2130377	0	0	0	OK
ENSBTAG00000048124	ENSBTAG00000048124	X:59797014-59797081	0	0	0	OK
ENSBTAG00000048126	ENSBTAG00000048126	X:15232586-15233199	0	0	0	OK
ENSBTAG00000048128	ENSBTAG00000048128	23:3198447-3198521	0	0	0	OK
ENSBTAG00000048130	ENSBTAG00000048130	2:10883786-10883950	0	0	0	OK
ENSBTAG00000048131	ENSBTAG00000048131	15:37591188-37591304	0	0	0	OK
ENSBTAG00000048132	ENSBTAG00000048132	7:45051048-45051155	0	0	0	OK
ENSBTAG00000048134	ENSBTAG00000048134	1:146903811-146903896	0	0	0	OK
ENSBTAG00000048135	ENSBTAG00000048135	20:71902470-71904001	0	0	0	OK
ENSBTAG00000048139	ENSBTAG00000048139	10:83872633-83872928	0	0	0	OK
ENSBTAG00000048140	ENSBTAG00000048140	25:42443399-42466498	0	0	0	OK
ENSBTAG00000048143	ENSBTAG00000048143	X:57246029-57246538	0	0	0	OK
ENSBTAG00000048144	ENSBTAG00000048144	1:23770025-23770144	0	0	0	OK
ENSBTAG00000048145	ENSBTAG00000048145	18:63239509-63242898	0	0	0	OK
ENSBTAG00000048146	ENSBTAG00000048146	21:67575238-67575342	0	0	0	OK
ENSBTAG00000048149	ENSBTAG00000048149	15:31142707-31142790	0	0	0	OK
ENSBTAG00000048150	ENSBTAG00000048150	1:119962056-119962374	0	0	0	OK
ENSBTAG00000048154	ENSBTAG00000048154	25:42259656-42260709	0	0	0	OK
ENSBTAG00000048155	ENSBTAG00000048155	11:106822996-106831824	0	0	0	OK
ENSBTAG00000048161	ENSBTAG00000048161	12:10499014-10500693	0	0	0	OK
ENSBTAG00000048163	ENSBTAG00000048163	X:93468214-93468595	0	0	0	OK
ENSBTAG00000048164	ENSBTAG00000048164	8:40335867-40336983	0	0	0	OK
ENSBTAG00000048165	ENSBTAG00000048165	1:12234086-12234183	0	0	0	OK
ENSBTAG00000048166	ENSBTAG00000048166	15:48672665-48673628	0	0	0	OK
ENSBTAG00000048167	ENSBTAG00000048167	17:64874907-64875048	0	0	0	OK
ENSBTAG00000048168	ENSBTAG00000048168	5:59292314-59293254	0	0	0	OK
ENSBTAG00000048171	ENSBTAG00000048171	27:4888372-4890323	0	0	0	OK

ENSBTAG00000048172	ENSBTAG00000048172	X:142664999-142690152	0	0	0	OK
ENSBTAG00000048174	ENSBTAG00000048174	18:50714305-50714362	0	0	0	OK
ENSBTAG00000048177	ENSBTAG00000048177	18:8454174-8456489	0	0	0	OK
ENSBTAG00000048178	ENSBTAG00000048178	27:36313186-36313272	0	0	0	OK
ENSBTAG00000048179	ENSBTAG00000048179	3:83474567-83474966	0	0	0	OK
ENSBTAG00000048180	ENSBTAG00000048180	28:16436541-16436646	0	0	0	OK
ENSBTAG00000048181	ENSBTAG00000048181	X:71160480-71160587	0	0	0	OK
ENSBTAG00000048182	ENSBTAG00000048182	2:62637763-62637841	0	0	0	OK
ENSBTAG00000048183	ENSBTAG00000048183	19:43901107-43901298	0	0	0	OK
ENSBTAG00000048185	ENSBTAG00000048185	9:2105886-2106183	0	0	0	OK
ENSBTAG00000048189	ENSBTAG00000048189	4:106543414-106543839	0	0	0	OK
ENSBTAG00000048191	ENSBTAG00000048191	18:59526946-59527003	0	0	0	OK
ENSBTAG00000048194	ENSBTAG00000048194	23:29004624-29005551	0	0	0	OK
ENSBTAG00000048195	ENSBTAG00000048195	1:71840197-71862076	0	0	0	OK
ENSBTAG00000048196	ENSBTAG00000048196	6:93865920-93867076	0	0	0	OK
ENSBTAG00000048197	ENSBTAG00000048197	8:103310497-103310604	0	0	0	OK
ENSBTAG00000048198	ENSBTAG00000048198	17:39082852-39082971	0	0	0	OK
ENSBTAG00000048200	ENSBTAG00000048200	1:130413416-130413546	0	0	0	OK
ENSBTAG00000048201	ENSBTAG00000048201	8:99681618-99681726	0	0	0	OK
ENSBTAG00000048205	ENSBTAG00000048205	6:92539481-92539538	0	0	0	OK
ENSBTAG00000048206	ENSBTAG00000048206	X:70356124-70357012	0	0	0	OK
ENSBTAG00000048207	ENSBTAG00000048207	11:4274107-4274592	0	0	0	OK
ENSBTAG00000048210	ENSBTAG00000048210	2:105374918-105375498	0	0	0	OK
ENSBTAG00000048211	ENSBTAG00000048211	17:46403661-46403749	0	0	0	OK
ENSBTAG00000048212	ENSBTAG00000048212	3:114001422-114002288	0	0	0	OK
ENSBTAG00000048215	ENSBTAG00000048215	X:32685377-32687929	0	0	0	OK
ENSBTAG00000048216	ENSBTAG00000048216	19:22194037-22194712	0	0	0	OK
ENSBTAG00000048217	ENSBTAG00000048217	19:20706672-20706743	0	0	0	OK
ENSBTAG00000048219	ENSBTAG00000048219	2:102923132-102923306	0	0	0	OK
ENSBTAG00000048223	ENSBTAG00000048223	15:47668550-47669498	0	0	0	OK

ENSBTAG00000048227	ENSBTAG00000048227	4:78619811-78619886	0	0	0	OK
ENSBTAG00000048228	ENSBTAG00000048228	2:125434926-125438150	0	0	0	OK
ENSBTAG00000048229	ENSBTAG00000048229	25:1394200-1417716	0	0	0	OK
ENSBTAG00000048231	ENSBTAG00000048231	25:39153843-39154122	0	0	0	OK
ENSBTAG00000048232	ENSBTAG00000048232	2:136913911-136914031	0	0	0	OK
ENSBTAG00000048234	ENSBTAG00000048234	16:81545842-81552388	0	0	0	OK
ENSBTAG00000048235	ENSBTAG00000048235	23:31918124-31918508	0	0	0	OK
ENSBTAG00000048236	ENSBTAG00000048236	10:23923497-23924002	0	0	0	OK
ENSBTAG00000048237	ENSBTAG00000048237	12:35630410-35647612	0	0	0	OK
ENSBTAG00000048239	ENSBTAG00000048239	9:65966260-65966391	0	0	0	OK
ENSBTAG00000048240	ENSBTAG00000048240	29:26908726-26909662	0	0	0	OK
ENSBTAG00000048241	ENSBTAG00000048241	X:34662659-34662756	0	0	0	OK
ENSBTAG00000048242	ENSBTAG00000048242	14:490117-490223	0	0	0	OK
ENSBTAG00000048243	ENSBTAG00000048243	22:29659445-29659547	0	0	0	OK
ENSBTAG00000048244	ENSBTAG00000048244	X:3562343-3562664	0	0	0	OK
ENSBTAG00000048247	ENSBTAG00000048247	2:130553869-130553935	0	0	6.27445	OK
ENSBTAG00000048248	ENSBTAG00000048248	29:26839219-26839326	0	0	0	OK
ENSBTAG00000048249	ENSBTAG00000048249	27:5788619-5790534	0	0	0	OK
ENSBTAG00000048250	ENSBTAG00000048250	6:87189735-87204439	0	0	0	OK
ENSBTAG00000048251	ENSBTAG00000048251	25:497907-498318	0	0	0	OK
ENSBTAG00000048252	ENSBTAG00000048252	8:87909357-87909451	0	0	0	OK
ENSBTAG00000048254	ENSBTAG00000048254	9:106653-106770	0	0	0	OK
ENSBTAG00000048258	ENSBTAG00000048258	7:47450516-47450612	0	0	4.31369	OK
ENSBTAG00000048259	ENSBTAG00000048259	27:39093990-39096822	0	0	0	OK
ENSBTAG00000048260	ENSBTAG00000048260	19:118122-119195	0	0	0	OK
ENSBTAG00000048261	ENSBTAG00000048261	18:64989576-64990500	0	0	0	OK
ENSBTAG00000048266	ENSBTAG00000048266	14:278095-278215	0	0	0	OK
ENSBTAG00000048267	ENSBTAG00000048267	16:49263301-49266770	0	0	0	OK
ENSBTAG00000048269	ENSBTAG00000048269	7:17905773-17906022	0	0	0	OK
ENSBTAG00000048270	ENSBTAG00000048270	7:1497657-1497800	0	0	0	OK

ENSBTAG00000048276	ENSBTAG00000048276	1:144192082-144196576	0	0	0	OK
ENSBTAG00000048281	ENSBTAG00000048281	X:24998137-24998914	0	0	0	OK
ENSBTAG00000048283	ENSBTAG00000048283	18:56994817-56996954	0	0	0	OK
ENSBTAG00000048284	ENSBTAG00000048284	21:1883666-1883785	0	0	0	OK
ENSBTAG00000048285	ENSBTAG00000048285	5:121158934-121159016	0	0	0	OK
ENSBTAG00000048287	ENSBTAG00000048287	X:140308767-140315911	0	0	0	OK
ENSBTAG00000048290	ENSBTAG00000048290	15:62465963-62484613	0	0	0	OK
ENSBTAG00000048293	ENSBTAG00000048293	19:43876414-43876605	0	0	0	OK
ENSBTAG00000048294	ENSBTAG00000048294	5:97529337-97529411	0	0	0	OK
ENSBTAG00000048296	ENSBTAG00000048296	X:135092214-135124289	0	0	0	OK
ENSBTAG00000048297	ENSBTAG00000048297	11:96264483-96264596	0	0	0	OK
ENSBTAG00000048299	ENSBTAG00000048299	26:3999123-3999191	0	0	0	OK
ENSBTAG00000048300	ENSBTAG00000048300	15:49242516-49243452	0	0	0	OK
ENSBTAG00000048301	ENSBTAG00000048301	15:49176141-49177077	0	0	0	OK
ENSBTAG00000048302	ENSBTAG00000048302	10:22718370-22719629	0	0	0	OK
ENSBTAG00000048305	ENSBTAG00000048305	1:133427062-133427245	0	0	0	OK
ENSBTAG00000048306	ENSBTAG00000048306	2:44142618-44145088	0	0	0	OK
ENSBTAG00000048308	ENSBTAG00000048308	16:47960245-47967797	0	0	0	OK
ENSBTAG00000048311	ENSBTAG00000048311	28:44858571-44859537	0	0	0	OK
ENSBTAG00000048312	ENSBTAG00000048312	3:58512741-58516154	0	0	0	OK
ENSBTAG00000048314	ENSBTAG00000048314	21:26597056-26600056	0	0	0	OK
ENSBTAG00000048315	ENSBTAG00000048315	7:42177316-42178276	0	0	0	OK
ENSBTAG00000048316	ENSBTAG00000048316	15:85246727-85265482	0	0	0	OK
ENSBTAG00000000027	ENSBTAG00000000027	18:53961669-53964590	0	0	0	OK

KM_status
ENSBTAT00000035907
ENSBTAT00000054432
ENSBTAT00000061773
ENSBTAT00000052701
ENSBTAT00000063721
ENSBTAT00000062429
ENSBTAT00000060811
ENSBTAT00000059920
ENSBTAT00000004256
ENSBTAT00000007946
ENSBTAT00000059709
ENSBTAT00000034970
ENSBTAT00000040601
ENSBTAT00000063500
ENSBTAT00000057100
ENSBTAT00000061848
ENSBTAT00000060703
ENSBTAT00000031172
ENSBTAT00000059116
ENSBTAT00000018472
ENSBTAT00000060085
ENSBTAT00000059469
ENSBTAT00000062440
ENSBTAT00000059744
ENSBTAT00000064588
ENSBTAT00000014400
ENSBTAT00000062503
ENSBTAT00000043704
ENSBTAT00000059640

ENSBTAT00000063431
ENSBTAT00000060117
ENSBTAT00000049965
ENSBTAT00000065101
ENSBTAT00000055966
ENSBTAT00000007219
ENSBTAT00000059670
ENSBTAT00000047372
ENSBTAT00000003178
ENSBTAT00000060957
ENSBTAT00000004322
ENSBTAT00000046285
ENSBTAT00000041456
ENSBTAT00000060185
ENSBTAT00000041666
ENSBTAT00000009293
ENSBTAT00000059708
ENSBTAT00000064826
ENSBTAT00000060263
ENSBTAT00000065489
ENSBTAT00000064095
ENSBTAT00000007553
ENSBTAT00000061905
ENSBTAT00000060513
ENSBTAT00000065446
ENSBTAT00000062374
ENSBTAT00000043415
ENSBTAT00000061756
ENSBTAT00000038266
ENSBTAT00000054510

ENSBTAT00000042549
ENSBTAT00000063176
ENSBTAT00000042753
ENSBTAT00000064912
ENSBTAT00000065953
ENSBTAT00000026906
ENSBTAT00000026120
ENSBTAT00000022049
ENSBTAT00000057493
ENSBTAT00000053216
ENSBTAT00000043204
ENSBTAT00000052158
ENSBTAT00000059915
ENSBTAT00000000135
ENSBTAT00000004166
ENSBTAT00000064549
ENSBTAT00000053779
ENSBTAT00000025702
ENSBTAT00000043146
ENSBTAT00000064568
ENSBTAT00000063946
ENSBTAT00000002313
ENSBTAT00000001124
ENSBTAT00000062925
ENSBTAT00000011001
ENSBTAT00000001091
ENSBTAT00000059641
ENSBTAT00000059748
ENSBTAT00000009471
ENSBTAT00000046439

ENSBTAT00000019237
ENSBTAT00000023226
ENSBTAT00000027402
ENSBTAT00000000600
ENSBTAT00000014734
ENSBTAT00000065988
ENSBTAT00000057114
ENSBTAT00000008738
ENSBTAT00000020914
ENSBTAT00000009509
ENSBTAT00000061837
ENSBTAT00000062960
ENSBTAT00000031637
ENSBTAT00000005921
ENSBTAT00000007241
ENSBTAT00000059427
ENSBTAT00000045410
ENSBTAT00000063470
ENSBTAT00000018979
ENSBTAT00000011694
ENSBTAT00000030222
ENSBTAT00000009713
ENSBTAT00000006935
ENSBTAT00000065839
ENSBTAT00000063877
ENSBTAT00000065003
ENSBTAT00000017500
ENSBTAT00000066050
ENSBTAT00000027871
ENSBTAT00000063932

ENSBTAT00000053433
ENSBTAT00000044388
ENSBTAT00000025932
ENSBTAT00000000528
ENSBTAT00000063194
ENSBTAT00000043149
ENSBTAT00000009254
ENSBTAT00000061646
ENSBTAT00000057012
ENSBTAT00000024334
ENSBTAT00000064369
ENSBTAT00000065876
ENSBTAT00000045049
ENSBTAT00000022514
ENSBTAT00000053602
ENSBTAT00000063683
ENSBTAT00000064673
ENSBTAT00000039001
ENSBTAT00000051459
ENSBTAT00000012860
ENSBTAT00000021652
ENSBTAT00000065146
ENSBTAT00000027911
ENSBTAT00000065576
ENSBTAT00000064571
ENSBTAT00000002110
ENSBTAT00000003076
ENSBTAT00000053588
ENSBTAT00000021871
ENSBTAT00000055683

ENSBTAT00000030008
ENSBTAT00000033010
ENSBTAT00000065055
ENSBTAT00000056660
ENSBTAT00000062467
ENSBTAT00000063490
ENSBTAT00000026112
ENSBTAT00000005753
ENSBTAT00000023806
ENSBTAT00000034754
ENSBTAT00000012218
ENSBTAT00000064301
ENSBTAT00000054025
ENSBTAT00000066072
ENSBTAT00000010778
ENSBTAT00000063115
ENSBTAT00000065916
ENSBTAT00000063645
ENSBTAT00000064922
ENSBTAT00000063624
ENSBTAT00000033888
ENSBTAT00000064438
ENSBTAT00000037762
ENSBTAT00000003449
ENSBTAT00000009732
ENSBTAT00000026044
ENSBTAT00000064058
ENSBTAT00000022306
ENSBTAT00000043315
ENSBTAT00000018428

ENSBTAT00000066202
ENSBTAT00000063198
ENSBTAT00000003445
ENSBTAT00000022882
ENSBTAT00000053888
ENSBTAT00000032277
ENSBTAT00000008038
ENSBTAT00000028648
ENSBTAT00000064904
ENSBTAT00000053747
ENSBTAT00000009750
ENSBTAT00000035679
ENSBTAT00000014627
ENSBTAT00000049425
ENSBTAT00000003368
ENSBTAT00000053713
ENSBTAT00000027007
ENSBTAT00000002868
ENSBTAT00000002627
ENSBTAT00000065328
ENSBTAT00000064137
ENSBTAT00000063767
ENSBTAT00000009699
ENSBTAT00000011106
ENSBTAT00000053943
ENSBTAT00000019735
ENSBTAT00000060720
ENSBTAT00000044308
ENSBTAT00000054445
ENSBTAT00000016363

ENSBTAT00000046641
ENSBTAT00000066177
ENSBTAT00000000605
ENSBTAT00000014540
ENSBTAT00000005407
ENSBTAT00000053458
ENSBTAT00000015922
ENSBTAT00000010110
ENSBTAT00000004867
ENSBTAT00000026265
ENSBTAT00000013438
ENSBTAT00000044309
ENSBTAT00000014602
ENSBTAT00000016463
ENSBTAT00000065205
ENSBTAT00000059588
ENSBTAT00000026336
ENSBTAT00000009774
ENSBTAT00000043650
ENSBTAT00000065705
ENSBTAT00000027391
ENSBTAT00000056383
ENSBTAT00000019336
ENSBTAT00000023981
ENSBTAT00000065997
ENSBTAT00000063406
ENSBTAT00000035967
ENSBTAT00000026237
ENSBTAT00000062708
ENSBTAT00000053197

ENSBTAT00000012283
ENSBTAT00000052414
ENSBTAT00000011273
ENSBTAT00000011811
ENSBTAT00000021162
ENSBTAT00000025591
ENSBTAT00000057026
ENSBTAT00000021774
ENSBTAT00000043317
ENSBTAT00000003220
ENSBTAT00000022119
ENSBTAT00000039235
ENSBTAT00000063065
ENSBTAT00000027336
ENSBTAT00000063806
ENSBTAT00000056997
ENSBTAT00000011172
ENSBTAT00000008736
ENSBTAT00000038676
ENSBTAT00000065287
ENSBTAT00000022040
ENSBTAT00000016497
ENSBTAT00000065629
ENSBTAT00000008355
ENSBTAT00000023987
ENSBTAT00000021755
ENSBTAT00000052546
ENSBTAT00000007323
ENSBTAT00000011446
ENSBTAT00000061269

ENSBTAT00000011204
ENSBTAT00000029418
ENSBTAT00000064142
ENSBTAT00000048277
ENSBTAT00000062286
ENSBTAT00000024318
ENSBTAT00000023471
ENSBTAT00000021548
ENSBTAT00000051964
ENSBTAT00000003550
ENSBTAT00000055312
ENSBTAT00000005381
ENSBTAT00000015337
ENSBTAT00000032405
ENSBTAT00000026378
ENSBTAT00000047858
ENSBTAT00000018048
ENSBTAT00000014103
ENSBTAT00000043145
ENSBTAT00000033404
ENSBTAT00000063056
ENSBTAT00000065281
ENSBTAT00000044457
ENSBTAT00000005367
ENSBTAT00000043153
ENSBTAT00000006328
ENSBTAT00000009534
ENSBTAT00000018478
ENSBTAT00000065745
ENSBTAT00000001222

ENSBTAT00000029140
ENSBTAT00000024314
ENSBTAT00000003943
ENSBTAT00000052466
ENSBTAT00000005885
ENSBTAT00000065380
ENSBTAT00000049255
ENSBTAT00000015969
ENSBTAT00000003140
ENSBTAT00000045374
ENSBTAT00000024678
ENSBTAT00000031714
ENSBTAT00000064766
ENSBTAT00000018295
ENSBTAT00000002512
ENSBTAT00000030641
ENSBTAT00000021344
ENSBTAT00000061530
ENSBTAT00000063239
ENSBTAT00000002256
ENSBTAT00000003547
ENSBTAT00000011206
ENSBTAT00000012004
ENSBTAT00000017616
ENSBTAT00000013194
ENSBTAT00000039893
ENSBTAT00000057243
ENSBTAT00000018750
ENSBTAT00000009843
ENSBTAT00000065694

ENSBTAT00000064859
ENSBTAT00000063021
ENSBTAT00000050479
ENSBTAT00000035866
ENSBTAT00000022251
ENSBTAT00000015385
ENSBTAT00000045168
ENSBTAT00000064706
ENSBTAT00000007927
ENSBTAT00000001766
ENSBTAT00000060810
ENSBTAT00000029468
ENSBTAT00000046765
ENSBTAT00000037464
ENSBTAT00000005544
ENSBTAT00000022546
ENSBTAT00000024387
ENSBTAT00000063139
ENSBTAT00000000926
ENSBTAT00000065937
ENSBTAT00000023195
ENSBTAT00000056163
ENSBTAT00000020797
ENSBTAT00000065982
ENSBTAT00000004835
ENSBTAT00000006955
ENSBTAT00000046579
ENSBTAT00000029408
ENSBTAT00000007080
ENSBTAT00000065664

ENSBTAT00000064816
ENSBTAT00000015619
ENSBTAT00000007776
ENSBTAT00000009267
ENSBTAT00000043470
ENSBTAT00000007033
ENSBTAT00000066068
ENSBTAT00000007071
ENSBTAT00000022638
ENSBTAT00000022906
ENSBTAT00000057534
ENSBTAT00000034860
ENSBTAT00000015938
ENSBTAT00000002349
ENSBTAT00000065865
ENSBTAT00000004206
ENSBTAT00000066224
ENSBTAT00000017001
ENSBTAT00000027987
ENSBTAT00000002241
ENSBTAT00000065125
ENSBTAT00000034158
ENSBTAT00000044696
ENSBTAT00000029245
ENSBTAT00000029529
ENSBTAT00000048313
ENSBTAT00000030590
ENSBTAT00000013858
ENSBTAT00000016117
ENSBTAT00000053059

ENSBTAT00000034668
ENSBTAT00000063912
ENSBTAT00000006197
ENSBTAT00000052029
ENSBTAT00000000510
ENSBTAT00000016357
ENSBTAT00000063611
ENSBTAT00000042815
ENSBTAT00000063019
ENSBTAT00000052411
ENSBTAT00000013392
ENSBTAT00000002903
ENSBTAT00000012479
ENSBTAT00000027639
ENSBTAT00000064304
ENSBTAT00000065986
ENSBTAT00000026114
ENSBTAT00000035350
ENSBTAT00000061511
ENSBTAT00000045185
ENSBTAT00000030411
ENSBTAT00000019924
ENSBTAT00000027627
ENSBTAT00000066146
ENSBTAT00000062452
ENSBTAT00000045319
ENSBTAT00000005371
ENSBTAT00000034315
ENSBTAT00000013689
ENSBTAT00000021414

ENSBTAT00000028109
ENSBTAT00000005636
ENSBTAT00000018362
ENSBTAT00000026000
ENSBTAT00000038959
ENSBTAT00000015499
ENSBTAT00000028122
ENSBTAT00000063716
ENSBTAT00000064747
ENSBTAT00000002291
ENSBTAT00000045242
ENSBTAT00000064920
ENSBTAT00000034726
ENSBTAT00000008839
ENSBTAT00000013154
ENSBTAT00000027106
ENSBTAT00000023143
ENSBTAT00000018501
ENSBTAT00000026527
ENSBTAT00000020787
ENSBTAT00000014674
ENSBTAT00000034824
ENSBTAT00000063485
ENSBTAT00000054257
ENSBTAT00000048872
ENSBTAT00000025008
ENSBTAT00000034384
ENSBTAT00000026102
ENSBTAT00000006673
ENSBTAT00000060706

ENSBTAT00000066329
ENSBTAT00000023144
ENSBTAT00000014178
ENSBTAT00000012920
ENSBTAT00000065385
ENSBTAT00000055752
ENSBTAT00000040162
ENSBTAT00000036824
ENSBTAT00000036468
ENSBTAT00000017322
ENSBTAT00000053227
ENSBTAT00000006596
ENSBTAT00000065853
ENSBTAT00000029233
ENSBTAT00000056395
ENSBTAT00000012696
ENSBTAT00000037406
ENSBTAT00000029331
ENSBTAT00000027554
ENSBTAT00000022847
ENSBTAT00000045429
ENSBTAT00000025132
ENSBTAT00000004005
ENSBTAT00000055740
ENSBTAT00000002015
ENSBTAT00000053963
ENSBTAT00000003640
ENSBTAT00000052636
ENSBTAT00000024455
ENSBTAT00000013326

ENSBTAT00000063926
ENSBTAT00000004519
ENSBTAT00000013952
ENSBTAT00000064830
ENSBTAT00000042598
ENSBTAT00000033672
ENSBTAT00000011392
ENSBTAT00000065214
ENSBTAT00000001573
ENSBTAT00000055351
ENSBTAT00000004036
ENSBTAT00000014209
ENSBTAT00000017459
ENSBTAT00000023089
ENSBTAT00000054198
ENSBTAT00000015832
ENSBTAT00000052370
ENSBTAT00000043046
ENSBTAT00000065532
ENSBTAT00000057417
ENSBTAT00000065553
ENSBTAT00000005327
ENSBTAT00000065381
ENSBTAT00000042617
ENSBTAT00000002612
ENSBTAT00000065049
ENSBTAT00000003099
ENSBTAT00000036462
ENSBTAT00000063674
ENSBTAT00000003253

ENSBTAT00000039401
ENSBTAT00000064644
ENSBTAT00000002666
ENSBTAT00000024621
ENSBTAT00000047798
ENSBTAT00000002369
ENSBTAT00000002656
ENSBTAT00000009507
ENSBTAT00000026547
ENSBTAT00000054428
ENSBTAT00000008515
ENSBTAT00000019979
ENSBTAT00000057215
ENSBTAT00000002904
ENSBTAT00000007554
ENSBTAT00000000541
ENSBTAT00000028392
ENSBTAT00000024635
ENSBTAT00000002262
ENSBTAT00000066284
ENSBTAT00000001530
ENSBTAT00000012563
ENSBTAT00000052340
ENSBTAT00000064292
ENSBTAT00000010195
ENSBTAT00000004476
ENSBTAT00000019692
ENSBTAT00000066267
ENSBTAT00000063699
ENSBTAT00000004639

ENSBTAT00000049496
ENSBTAT00000018429
ENSBTAT00000061525
ENSBTAT00000024680
ENSBTAT00000055040
ENSBTAT00000000568
ENSBTAT00000001810
ENSBTAT00000015681
ENSBTAT00000037103
ENSBTAT00000022236
ENSBTAT00000007844
ENSBTAT00000018109
ENSBTAT00000018200
ENSBTAT00000023774
ENSBTAT00000064055
ENSBTAT00000056888
ENSBTAT00000016949
ENSBTAT00000042675
ENSBTAT00000019897
ENSBTAT00000007588
ENSBTAT00000063051
ENSBTAT00000066112
ENSBTAT00000065135
ENSBTAT00000002337
ENSBTAT00000065106
ENSBTAT00000052642
ENSBTAT00000021593
ENSBTAT00000002087
ENSBTAT00000029807
ENSBTAT00000022056

ENSBTAT00000003131
ENSBTAT00000015203
ENSBTAT00000034488
ENSBTAT00000009465
ENSBTAT00000063009
ENSBTAT00000019915
ENSBTAT00000037991
ENSBTAT00000000876
ENSBTAT00000005072
ENSBTAT00000007978
ENSBTAT00000061201
ENSBTAT00000001539
ENSBTAT00000004973
ENSBTAT00000064010
ENSBTAT00000043836
ENSBTAT00000001070
ENSBTAT00000063823
ENSBTAT00000000065
ENSBTAT00000014108
ENSBTAT00000024728
ENSBTAT00000001428
ENSBTAT00000043099
ENSBTAT00000027188
ENSBTAT00000001745
ENSBTAT00000032323
ENSBTAT00000030211
ENSBTAT00000022318
ENSBTAT00000018998
ENSBTAT00000029052
ENSBTAT00000007796

ENSBTAT00000027434
ENSBTAT00000065971
ENSBTAT00000025483
ENSBTAT00000013434
ENSBTAT00000022057
ENSBTAT00000011467
ENSBTAT00000011196
ENSBTAT00000049484
ENSBTAT00000001510
ENSBTAT00000007518
ENSBTAT00000001898
ENSBTAT00000008933
ENSBTAT00000063088
ENSBTAT00000065232
ENSBTAT00000063089
ENSBTAT00000027992
ENSBTAT00000017527
ENSBTAT00000054173
ENSBTAT00000000606
ENSBTAT00000019835
ENSBTAT00000026501
ENSBTAT00000064518
ENSBTAT00000056436
ENSBTAT00000004402
ENSBTAT00000002024
ENSBTAT00000022920
ENSBTAT00000045034
ENSBTAT00000027321
ENSBTAT00000053610
ENSBTAT00000015478

ENSBTAT00000048617
ENSBTAT00000011810
ENSBTAT00000027564
ENSBTAT00000002474
ENSBTAT00000023923
ENSBTAT00000011019
ENSBTAT00000000437
ENSBTAT00000004828
ENSBTAT00000001431
ENSBTAT00000000560
ENSBTAT00000034126
ENSBTAT00000056028
ENSBTAT00000019771
ENSBTAT00000030916
ENSBTAT00000064493
ENSBTAT00000064317
ENSBTAT00000013333
ENSBTAT00000003304
ENSBTAT00000010618
ENSBTAT00000019274
ENSBTAT00000023345
ENSBTAT00000062860
ENSBTAT00000064761
ENSBTAT00000023715
ENSBTAT00000047326
ENSBTAT00000051901
ENSBTAT00000032947
ENSBTAT00000042722
ENSBTAT00000006571
ENSBTAT00000002792

ENSBTAT00000024194
ENSBTAT00000014304
ENSBTAT00000009472
ENSBTAT00000061325
ENSBTAT00000066282
ENSBTAT00000019239
ENSBTAT00000012885
ENSBTAT00000056212
ENSBTAT00000064945
ENSBTAT00000005794
ENSBTAT00000013014
ENSBTAT00000063331
ENSBTAT00000019772
ENSBTAT00000064294
ENSBTAT00000065539
ENSBTAT00000008137
ENSBTAT00000043686
ENSBTAT00000021264
ENSBTAT00000010214
ENSBTAT00000056782
ENSBTAT00000001716
ENSBTAT00000029003
ENSBTAT00000003422
ENSBTAT00000001919
ENSBTAT00000018512
ENSBTAT00000002408
ENSBTAT00000018163
ENSBTAT00000034484
ENSBTAT00000014593
ENSBTAT00000028982

ENSBTAT00000012148
ENSBTAT00000014049
ENSBTAT00000008174
ENSBTAT00000014023
ENSBTAT00000027669
ENSBTAT00000011465
ENSBTAT00000022616
ENSBTAT00000024376
ENSBTAT00000017456
ENSBTAT00000013971
ENSBTAT00000014309
ENSBTAT00000001425
ENSBTAT00000016937
ENSBTAT00000065893
ENSBTAT00000006835
ENSBTAT00000063233
ENSBTAT00000040145
ENSBTAT00000009504
ENSBTAT00000046547
ENSBTAT00000016705
ENSBTAT00000006599
ENSBTAT00000044341
ENSBTAT00000005326
ENSBTAT00000012592
ENSBTAT00000049643
ENSBTAT00000066066
ENSBTAT00000066054
ENSBTAT00000037556
ENSBTAT00000065154
ENSBTAT00000018181

ENSBTAT00000044328
ENSBTAT00000031232
ENSBTAT00000005279
ENSBTAT00000047689
ENSBTAT00000017992
ENSBTAT00000021750
ENSBTAT00000003907
ENSBTAT00000012578
ENSBTAT00000022145
ENSBTAT00000018785
ENSBTAT00000001623
ENSBTAT00000015137
ENSBTAT00000023107
ENSBTAT00000021365
ENSBTAT00000060515
ENSBTAT00000007809
ENSBTAT00000001031
ENSBTAT00000012903
ENSBTAT00000008682
ENSBTAT00000017410
ENSBTAT00000055865
ENSBTAT00000020126
ENSBTAT00000042908
ENSBTAT00000003590
ENSBTAT00000029098
ENSBTAT00000020341
ENSBTAT00000043701
ENSBTAT00000007150
ENSBTAT00000055739
ENSBTAT00000023620

ENSBTAT00000002234
ENSBTAT00000038548
ENSBTAT00000062474
ENSBTAT00000027366
ENSBTAT00000003414
ENSBTAT00000004790
ENSBTAT00000018267
ENSBTAT00000057103
ENSBTAT00000010620
ENSBTAT00000010907
ENSBTAT00000028966
ENSBTAT00000017092
ENSBTAT00000002130
ENSBTAT00000004764
ENSBTAT00000064481
ENSBTAT00000001512
ENSBTAT00000065216
ENSBTAT00000064864
ENSBTAT00000064194
ENSBTAT00000050510
ENSBTAT00000032902
ENSBTAT00000018417
ENSBTAT00000024900
ENSBTAT00000023446
ENSBTAT00000003129
ENSBTAT00000028059
ENSBTAT00000018970
ENSBTAT00000034505
ENSBTAT00000001596
ENSBTAT00000063434

ENSBTAT00000017882
ENSBTAT00000055247
ENSBTAT00000055065
ENSBTAT00000018403
ENSBTAT00000007273
ENSBTAT00000020194
ENSBTAT00000015231
ENSBTAT00000019675
ENSBTAT00000026407
ENSBTAT00000019055
ENSBTAT00000004608
ENSBTAT00000000049
ENSBTAT00000016997
ENSBTAT00000065883
ENSBTAT00000065752
ENSBTAT00000005167
ENSBTAT00000004382
ENSBTAT00000017822
ENSBTAT00000064557
ENSBTAT00000052536
ENSBTAT00000024507
ENSBTAT00000009724
ENSBTAT00000016769
ENSBTAT00000016144
ENSBTAT00000044692
ENSBTAT00000006427
ENSBTAT00000044296
ENSBTAT00000043067
ENSBTAT00000033400
ENSBTAT00000064131

ENSBTAT00000056457
ENSBTAT00000063979
ENSBTAT00000064218
ENSBTAT00000021521
ENSBTAT00000011007
ENSBTAT00000021451
ENSBTAT00000053147
ENSBTAT00000035103
ENSBTAT00000007808
ENSBTAT00000006005
ENSBTAT00000020193
ENSBTAT00000047914
ENSBTAT00000020207
ENSBTAT00000062944
ENSBTAT00000007622
ENSBTAT00000016931
ENSBTAT00000032575
ENSBTAT00000029397
ENSBTAT00000006962
ENSBTAT00000021448
ENSBTAT00000001164
ENSBTAT00000006882
ENSBTAT00000063193
ENSBTAT00000046098
ENSBTAT00000039272
ENSBTAT00000012144
ENSBTAT00000022146
ENSBTAT00000030288
ENSBTAT00000011068
ENSBTAT00000056957

ENSBTAT00000055380
ENSBTAT00000017571
ENSBTAT00000029297
ENSBTAT00000017687
ENSBTAT00000001415
ENSBTAT00000057260
ENSBTAT00000009270
ENSBTAT00000064108
ENSBTAT00000029332
ENSBTAT00000034655
ENSBTAT00000002397
ENSBTAT00000024179
ENSBTAT00000009431
ENSBTAT00000030613
ENSBTAT00000018418
ENSBTAT00000054001
ENSBTAT00000000651
ENSBTAT00000020110
ENSBTAT00000019293
ENSBTAT00000065114
ENSBTAT00000008995
ENSBTAT00000003266
ENSBTAT00000004193
ENSBTAT00000007041
ENSBTAT00000062519
ENSBTAT00000014355
ENSBTAT00000008645
ENSBTAT00000015095
ENSBTAT00000027652
ENSBTAT00000020990

ENSBTAT00000025446
ENSBTAT00000006224
ENSBTAT00000044551
ENSBTAT00000062879
ENSBTAT00000025279
ENSBTAT00000038538
ENSBTAT00000013961
ENSBTAT00000066025
ENSBTAT00000061286
ENSBTAT00000014947
ENSBTAT00000003766
ENSBTAT00000057596
ENSBTAT00000026640
ENSBTAT00000012306
ENSBTAT00000064311
ENSBTAT00000046739
ENSBTAT00000015491
ENSBTAT00000065426
ENSBTAT00000064523
ENSBTAT00000026830
ENSBTAT00000044934
ENSBTAT00000065895
ENSBTAT00000064145
ENSBTAT00000056396
ENSBTAT00000061052
ENSBTAT00000023844
ENSBTAT00000048363
ENSBTAT00000021458
ENSBTAT00000042886
ENSBTAT00000060562

ENSBTAT00000002850
ENSBTAT00000027343
ENSBTAT00000008803
ENSBTAT00000037203
ENSBTAT00000026593
ENSBTAT00000033712
ENSBTAT00000006153
ENSBTAT00000063943
ENSBTAT00000006663
ENSBTAT00000015059
ENSBTAT00000007734
ENSBTAT00000043080
ENSBTAT00000053928
ENSBTAT00000012952
ENSBTAT00000026054
ENSBTAT00000065196
ENSBTAT00000043695
ENSBTAT00000056532
ENSBTAT00000024480
ENSBTAT00000019284
ENSBTAT00000063742
ENSBTAT00000053525
ENSBTAT00000025357
ENSBTAT00000015092
ENSBTAT00000015907
ENSBTAT00000035910
ENSBTAT00000065474
ENSBTAT00000063043
ENSBTAT00000025126
ENSBTAT00000009059

ENSBTAT00000045434
ENSBTAT00000065632
ENSBTAT00000063507
ENSBTAT00000008028
ENSBTAT00000006031
ENSBTAT00000020109
ENSBTAT00000004915
ENSBTAT00000024491
ENSBTAT00000011742
ENSBTAT00000053856
ENSBTAT00000052684
ENSBTAT00000061975
ENSBTAT00000022029
ENSBTAT00000043348
ENSBTAT00000043569
ENSBTAT00000024921
ENSBTAT00000019496
ENSBTAT00000034609
ENSBTAT00000049472
ENSBTAT00000019690
ENSBTAT00000052718
ENSBTAT00000004536
ENSBTAT00000063276
ENSBTAT00000038591
ENSBTAT00000023779
ENSBTAT00000010506
ENSBTAT00000064726
ENSBTAT00000037612
ENSBTAT00000006774
ENSBTAT00000057440

ENSBTAT00000024101
ENSBTAT00000015230
ENSBTAT00000001319
ENSBTAT00000014927
ENSBTAT00000051947
ENSBTAT00000065550
ENSBTAT00000064404
ENSBTAT00000052188
ENSBTAT00000047273
ENSBTAT00000063380
ENSBTAT00000018888
ENSBTAT00000012119
ENSBTAT00000002365
ENSBTAT00000004844
ENSBTAT00000032525
ENSBTAT00000030210
ENSBTAT00000023866
ENSBTAT00000023084
ENSBTAT00000014999
ENSBTAT00000004177
ENSBTAT00000063550
ENSBTAT00000042877
ENSBTAT00000007959
ENSBTAT00000015483
ENSBTAT00000020887
ENSBTAT00000013803
ENSBTAT00000019345
ENSBTAT00000048021
ENSBTAT00000018207
ENSBTAT00000025933

ENSBTAT00000016300
ENSBTAT00000027744
ENSBTAT00000010058
ENSBTAT00000054778
ENSBTAT00000062561
ENSBTAT00000062119
ENSBTAT00000055322
ENSBTAT00000027654
ENSBTAT00000016196
ENSBTAT00000017892
ENSBTAT00000064714
ENSBTAT00000006356
ENSBTAT00000025032
ENSBTAT00000001429
ENSBTAT00000047740
ENSBTAT00000062985
ENSBTAT00000002707
ENSBTAT00000055458
ENSBTAT00000005840
ENSBTAT00000044940
ENSBTAT00000065702
ENSBTAT00000057223
ENSBTAT00000006831
ENSBTAT00000012504
ENSBTAT00000052931
ENSBTAT00000001762
ENSBTAT00000028915
ENSBTAT00000021078
ENSBTAT00000026023
ENSBTAT00000020267

ENSBTAT00000031822
ENSBTAT00000019085
ENSBTAT00000018000
ENSBTAT00000010314
ENSBTAT00000065004
ENSBTAT00000014490
ENSBTAT00000020600
ENSBTAT00000030158
ENSBTAT00000016540
ENSBTAT00000047412
ENSBTAT00000020679
ENSBTAT00000027131
ENSBTAT00000056210
ENSBTAT00000047753
ENSBTAT00000004620
ENSBTAT00000005016
ENSBTAT00000009888
ENSBTAT00000023975
ENSBTAT00000004277
ENSBTAT00000057552
ENSBTAT00000006203
ENSBTAT00000022848
ENSBTAT00000065418
ENSBTAT00000065267
ENSBTAT00000066030
ENSBTAT00000062397
ENSBTAT00000021708
ENSBTAT00000006725
ENSBTAT00000064837
ENSBTAT00000000177

ENSBTAT00000015274
ENSBTAT00000026323
ENSBTAT00000032282
ENSBTAT00000024411
ENSBTAT00000028780
ENSBTAT00000063294
ENSBTAT00000001047
ENSBTAT00000066296
ENSBTAT00000028950
ENSBTAT00000050547
ENSBTAT00000037060
ENSBTAT00000029705
ENSBTAT00000036229
ENSBTAT00000026223
ENSBTAT00000000459
ENSBTAT00000043198
ENSBTAT00000011384
ENSBTAT00000008092
ENSBTAT00000006510
ENSBTAT00000000277
ENSBTAT00000023232
ENSBTAT00000066297
ENSBTAT00000063682
ENSBTAT00000002709
ENSBTAT00000036417
ENSBTAT00000033398
ENSBTAT00000027262
ENSBTAT00000061618
ENSBTAT00000009415
ENSBTAT00000008072

ENSBTAT00000053881
ENSBTAT00000024561
ENSBTAT00000025445
ENSBTAT00000008363
ENSBTAT00000010974
ENSBTAT00000011996
ENSBTAT00000025122
ENSBTAT00000004104
ENSBTAT00000066019
ENSBTAT00000065190
ENSBTAT00000024729
ENSBTAT00000023562
ENSBTAT00000000073
ENSBTAT00000023164
ENSBTAT00000047510
ENSBTAT00000021025
ENSBTAT00000008025
ENSBTAT00000050568
ENSBTAT00000053242
ENSBTAT00000037231
ENSBTAT00000009346
ENSBTAT00000050213
ENSBTAT00000017401
ENSBTAT00000013267
ENSBTAT00000053239
ENSBTAT00000000438
ENSBTAT00000008214
ENSBTAT00000000687
ENSBTAT00000012794
ENSBTAT00000052239

ENSBTAT00000046161
ENSBTAT00000014245
ENSBTAT00000028107
ENSBTAT00000024410
ENSBTAT00000006460
ENSBTAT00000028569
ENSBTAT00000017036
ENSBTAT00000022392
ENSBTAT00000003955
ENSBTAT00000054542
ENSBTAT00000008128
ENSBTAT00000018749
ENSBTAT00000025185
ENSBTAT00000023511
ENSBTAT00000061583
ENSBTAT00000015156
ENSBTAT00000021685
ENSBTAT00000064324
ENSBTAT00000057049
ENSBTAT00000044202
ENSBTAT00000039630
ENSBTAT00000012861
ENSBTAT00000052761
ENSBTAT00000031940
ENSBTAT00000029212
ENSBTAT00000064715
ENSBTAT00000006887
ENSBTAT00000043181
ENSBTAT00000005938
ENSBTAT00000045384

ENSBTAT00000028563
ENSBTAT00000004785
ENSBTAT00000010867
ENSBTAT00000025388
ENSBTAT00000025049
ENSBTAT00000004157
ENSBTAT00000036888
ENSBTAT00000017706
ENSBTAT00000001270
ENSBTAT00000000043
ENSBTAT00000003469
ENSBTAT00000009997
ENSBTAT00000056959
ENSBTAT00000005251
ENSBTAT00000018063
ENSBTAT00000049153
ENSBTAT00000028788
ENSBTAT00000063203
ENSBTAT00000012049
ENSBTAT00000063574
ENSBTAT00000063945
ENSBTAT00000009796
ENSBTAT00000022125
ENSBTAT00000064623
ENSBTAT00000064440
ENSBTAT00000022257
ENSBTAT00000046015
ENSBTAT00000061126
ENSBTAT00000007769
ENSBTAT00000034671

ENSBTAT00000027117
ENSBTAT00000021903
ENSBTAT00000063306
ENSBTAT00000054953
ENSBTAT00000024088
ENSBTAT00000047653
ENSBTAT00000065677
ENSBTAT00000063843
ENSBTAT00000027848
ENSBTAT00000024214
ENSBTAT00000012154
ENSBTAT00000034713
ENSBTAT00000008181
ENSBTAT00000023762
ENSBTAT00000048967
ENSBTAT00000006812
ENSBTAT00000027593
ENSBTAT00000016922
ENSBTAT00000056018
ENSBTAT00000002289
ENSBTAT00000024031
ENSBTAT00000012416
ENSBTAT00000000695
ENSBTAT00000063003
ENSBTAT00000006914
ENSBTAT00000054396
ENSBTAT00000011820
ENSBTAT00000019214
ENSBTAT00000027974
ENSBTAT00000015294

ENSBTAT00000017614
ENSBTAT00000010283
ENSBTAT00000007578
ENSBTAT00000010306
ENSBTAT00000013208
ENSBTAT00000053598
ENSBTAT00000020781
ENSBTAT00000029040
ENSBTAT00000021523
ENSBTAT00000065903
ENSBTAT00000017430
ENSBTAT00000043777
ENSBTAT00000029105
ENSBTAT00000047557
ENSBTAT00000018843
ENSBTAT00000052388
ENSBTAT00000009029
ENSBTAT00000015581
ENSBTAT00000025807
ENSBTAT00000026853
ENSBTAT00000026197
ENSBTAT00000038177
ENSBTAT00000009073
ENSBTAT00000026889
ENSBTAT00000021830
ENSBTAT00000044250
ENSBTAT00000009887
ENSBTAT00000053689
ENSBTAT00000006622
ENSBTAT00000012013

ENSBTAT00000046706
ENSBTAT00000017819
ENSBTAT00000000463
ENSBTAT00000003773
ENSBTAT00000006219
ENSBTAT00000043431
ENSBTAT00000014267
ENSBTAT00000025176
ENSBTAT00000028273
ENSBTAT00000049901
ENSBTAT00000065501
ENSBTAT00000032692
ENSBTAT00000016038
ENSBTAT00000028801
ENSBTAT00000029102
ENSBTAT00000003575
ENSBTAT00000009307
ENSBTAT00000008066
ENSBTAT00000049438
ENSBTAT00000047408
ENSBTAT00000008663
ENSBTAT00000006297
ENSBTAT00000047794
ENSBTAT00000023709
ENSBTAT00000027552
ENSBTAT00000029488
ENSBTAT00000055916
ENSBTAT00000010973
ENSBTAT00000039858
ENSBTAT00000054878

ENSBTAT00000002418
ENSBTAT00000011325
ENSBTAT00000026898
ENSBTAT00000011048
ENSBTAT00000001335
ENSBTAT00000064535
ENSBTAT00000017240
ENSBTAT00000055401
ENSBTAT00000016591
ENSBTAT00000035541
ENSBTAT00000053925
ENSBTAT00000034262
ENSBTAT00000018399
ENSBTAT00000022314
ENSBTAT00000053640
ENSBTAT00000022416
ENSBTAT00000003175
ENSBTAT00000020855
ENSBTAT00000009464
ENSBTAT00000009683
ENSBTAT00000009756
ENSBTAT00000020514
ENSBTAT00000026592
ENSBTAT00000023834
ENSBTAT00000010057
ENSBTAT00000013600
ENSBTAT00000002384
ENSBTAT00000024942
ENSBTAT00000050282
ENSBTAT00000020974

ENSBTAT00000053267
ENSBTAT00000056613
ENSBTAT00000043371
ENSBTAT00000063495
ENSBTAT00000065248
ENSBTAT00000003079
ENSBTAT00000065621
ENSBTAT00000052577
ENSBTAT00000006653
ENSBTAT00000037234
ENSBTAT00000044818
ENSBTAT00000020473
ENSBTAT00000018525
ENSBTAT00000027940
ENSBTAT00000007837
ENSBTAT00000049151
ENSBTAT00000003467
ENSBTAT00000037840
ENSBTAT00000020624
ENSBTAT00000015117
ENSBTAT00000011308
ENSBTAT00000012662
ENSBTAT00000055152
ENSBTAT00000063670
ENSBTAT00000021590
ENSBTAT00000055137
ENSBTAT00000052302
ENSBTAT00000028517
ENSBTAT00000056087
ENSBTAT00000054541

ENSBTAT00000000683
ENSBTAT00000012241
ENSBTAT00000020230
ENSBTAT00000000714
ENSBTAT00000046350
ENSBTAT00000016702
ENSBTAT00000055723
ENSBTAT00000065377
ENSBTAT00000017381
ENSBTAT00000013450
ENSBTAT00000065651
ENSBTAT00000044329
ENSBTAT00000038679
ENSBTAT00000002194
ENSBTAT00000013923
ENSBTAT00000024305
ENSBTAT00000009210
ENSBTAT00000027782
ENSBTAT00000047143
ENSBTAT00000028518
ENSBTAT00000000231
ENSBTAT00000027540
ENSBTAT00000043559
ENSBTAT00000020131
ENSBTAT00000001787
ENSBTAT00000036248
ENSBTAT00000065182
ENSBTAT00000006567
ENSBTAT00000055138
ENSBTAT00000014572

ENSBTAT00000001430
ENSBTAT00000010543
ENSBTAT00000045174
ENSBTAT00000044681
ENSBTAT00000036293
ENSBTAT00000026663
ENSBTAT00000003831
ENSBTAT00000015898
ENSBTAT00000004042
ENSBTAT00000006924
ENSBTAT00000004918
ENSBTAT00000021995
ENSBTAT00000066184
ENSBTAT00000004931
ENSBTAT00000011658
ENSBTAT00000061005
ENSBTAT00000013399
ENSBTAT00000039021
ENSBTAT00000036734
ENSBTAT00000065107
ENSBTAT00000066252
ENSBTAT00000005226
ENSBTAT00000005562
ENSBTAT00000061391
ENSBTAT00000008009
ENSBTAT00000066102
ENSBTAT00000045129
ENSBTAT00000018426
ENSBTAT00000013902
ENSBTAT00000047887

ENSBTAT00000048644
ENSBTAT00000014871
ENSBTAT00000066318
ENSBTAT00000001503
ENSBTAT00000014457
ENSBTAT00000066026
ENSBTAT00000039541
ENSBTAT00000002177
ENSBTAT00000028257
ENSBTAT00000011066
ENSBTAT00000018681
ENSBTAT00000056966
ENSBTAT00000017386
ENSBTAT00000034015
ENSBTAT00000001104
ENSBTAT00000035283
ENSBTAT00000006986
ENSBTAT00000043791
ENSBTAT00000002858
ENSBTAT00000028665
ENSBTAT00000015648
ENSBTAT00000063424
ENSBTAT00000007608
ENSBTAT00000028720
ENSBTAT00000007836
ENSBTAT00000003460
ENSBTAT00000036029
ENSBTAT00000004170
ENSBTAT00000016978
ENSBTAT00000005577

ENSBTAT00000031313
ENSBTAT00000024638
ENSBTAT00000009979
ENSBTAT00000043831
ENSBTAT00000022259
ENSBTAT00000064876
ENSBTAT00000000543
ENSBTAT00000063686
ENSBTAT00000014032
ENSBTAT00000064886
ENSBTAT00000027152
ENSBTAT00000010602
ENSBTAT00000028587
ENSBTAT00000000582
ENSBTAT00000038996
ENSBTAT00000001821
ENSBTAT00000000465
ENSBTAT00000054183
ENSBTAT00000028625
ENSBTAT00000013964
ENSBTAT00000025983
ENSBTAT00000027181
ENSBTAT00000019861
ENSBTAT00000009158
ENSBTAT00000013662
ENSBTAT00000026211
ENSBTAT00000028208
ENSBTAT00000020075
ENSBTAT00000052838
ENSBTAT00000011932

ENSBTAT00000060944
ENSBTAT00000033464
ENSBTAT00000064620
ENSBTAT00000014865
ENSBTAT00000005174
ENSBTAT00000066334
ENSBTAT00000028606
ENSBTAT00000023836
ENSBTAT00000003882
ENSBTAT00000038247
ENSBTAT00000029027
ENSBTAT00000034326
ENSBTAT00000034686
ENSBTAT00000063509
ENSBTAT00000034453
ENSBTAT00000053258
ENSBTAT00000004084
ENSBTAT00000023313
ENSBTAT00000017477
ENSBTAT00000039777
ENSBTAT00000063079
ENSBTAT00000017485
ENSBTAT00000011374
ENSBTAT00000034291
ENSBTAT00000025631
ENSBTAT00000064938
ENSBTAT00000066330
ENSBTAT00000055871
ENSBTAT00000022675
ENSBTAT00000063895

ENSBTAT00000011088
ENSBTAT00000046646
ENSBTAT00000034072
ENSBTAT00000007743
ENSBTAT00000010609
ENSBTAT00000052929
ENSBTAT00000031490
ENSBTAT00000002122
ENSBTAT00000018609
ENSBTAT00000038352
ENSBTAT00000022885
ENSBTAT00000015368
ENSBTAT00000020546
ENSBTAT00000039859
ENSBTAT00000022373
ENSBTAT00000044381
ENSBTAT00000056992
ENSBTAT00000020952
ENSBTAT00000002603
ENSBTAT00000038488
ENSBTAT00000008574
ENSBTAT00000025482
ENSBTAT00000006632
ENSBTAT00000023448
ENSBTAT00000000872
ENSBTAT00000005286
ENSBTAT00000057153
ENSBTAT00000007420
ENSBTAT00000065770
ENSBTAT00000042563

ENSBTAT00000010971
ENSBTAT00000010762
ENSBTAT00000045713
ENSBTAT00000033658
ENSBTAT00000020729
ENSBTAT00000012676
ENSBTAT00000017177
ENSBTAT00000010675
ENSBTAT00000064309
ENSBTAT00000016974
ENSBTAT00000021263
ENSBTAT00000065475
ENSBTAT00000006156
ENSBTAT00000020675
ENSBTAT00000013992
ENSBTAT00000053695
ENSBTAT00000031205
ENSBTAT00000001588
ENSBTAT00000004224
ENSBTAT00000056434
ENSBTAT00000015679
ENSBTAT00000063059
ENSBTAT00000017446
ENSBTAT00000001749
ENSBTAT00000010969
ENSBTAT00000020765
ENSBTAT00000020581
ENSBTAT00000022142
ENSBTAT00000001024
ENSBTAT00000060964

ENSBTAT00000014394
ENSBTAT00000019309
ENSBTAT00000063462
ENSBTAT00000024068
ENSBTAT00000064229
ENSBTAT00000053098
ENSBTAT00000063854
ENSBTAT00000013751
ENSBTAT00000062341
ENSBTAT00000008327
ENSBTAT00000003479
ENSBTAT00000000009
ENSBTAT00000015606
ENSBTAT00000052057
ENSBTAT00000048677
ENSBTAT00000017251
ENSBTAT00000052339
ENSBTAT00000038295
ENSBTAT00000036581
ENSBTAT00000001835
ENSBTAT00000021161
ENSBTAT00000039003
ENSBTAT00000024395
ENSBTAT00000024606
ENSBTAT00000052802
ENSBTAT00000017350
ENSBTAT00000063301
ENSBTAT00000063935
ENSBTAT00000021019
ENSBTAT00000036738

ENSBTAT00000006987
ENSBTAT00000064931
ENSBTAT00000061041
ENSBTAT00000016019
ENSBTAT00000019282
ENSBTAT00000008434
ENSBTAT00000052413
ENSBTAT00000024626
ENSBTAT00000054284
ENSBTAT00000027342
ENSBTAT00000010219
ENSBTAT00000064693
ENSBTAT00000019811
ENSBTAT00000020941
ENSBTAT00000022163
ENSBTAT00000026255
ENSBTAT00000027238
ENSBTAT00000015149
ENSBTAT00000020305
ENSBTAT00000020903
ENSBTAT00000028149
ENSBTAT00000010481
ENSBTAT00000038682
ENSBTAT00000028434
ENSBTAT00000020394
ENSBTAT00000014069
ENSBTAT00000005817
ENSBTAT00000014423
ENSBTAT00000028239
ENSBTAT00000000583

ENSBTAT00000054917
ENSBTAT00000037046
ENSBTAT00000021584
ENSBTAT00000054742
ENSBTAT00000063995
ENSBTAT00000023031
ENSBTAT00000063492
ENSBTAT00000042999
ENSBTAT00000006715
ENSBTAT00000018248
ENSBTAT00000060547
ENSBTAT00000014656
ENSBTAT00000050795
ENSBTAT00000056703
ENSBTAT00000054611
ENSBTAT00000009931
ENSBTAT00000028175
ENSBTAT00000005231
ENSBTAT00000015993
ENSBTAT00000024447
ENSBTAT00000055915
ENSBTAT00000027219
ENSBTAT00000001255
ENSBTAT00000008607
ENSBTAT00000027446
ENSBTAT00000052181
ENSBTAT00000013165
ENSBTAT00000009492
ENSBTAT00000045141
ENSBTAT00000016104

ENSBTAT00000003881
ENSBTAT00000020105
ENSBTAT00000061352
ENSBTAT00000053873
ENSBTAT00000016356
ENSBTAT00000004143
ENSBTAT00000023729
ENSBTAT00000049176
ENSBTAT00000027197
ENSBTAT00000019646
ENSBTAT00000037513
ENSBTAT00000026100
ENSBTAT00000009938
ENSBTAT00000063796
ENSBTAT00000063283
ENSBTAT00000035974
ENSBTAT00000065682
ENSBTAT00000009755
ENSBTAT00000045258
ENSBTAT00000019713
ENSBTAT00000020046
ENSBTAT00000061184
ENSBTAT00000024512
ENSBTAT00000042779
ENSBTAT00000035023
ENSBTAT00000053320
ENSBTAT00000025346
ENSBTAT00000029618
ENSBTAT00000007812
ENSBTAT00000025426

ENSBTAT00000065899
ENSBTAT00000004243
ENSBTAT00000063147
ENSBTAT00000020521
ENSBTAT00000047964
ENSBTAT00000021935
ENSBTAT00000004035
ENSBTAT00000043006
ENSBTAT00000000482
ENSBTAT00000030064
ENSBTAT00000020080
ENSBTAT00000040008
ENSBTAT00000052111
ENSBTAT00000017212
ENSBTAT00000021912
ENSBTAT00000004617
ENSBTAT00000056161
ENSBTAT00000052300
ENSBTAT00000046515
ENSBTAT00000035615
ENSBTAT00000057500
ENSBTAT00000010469
ENSBTAT00000057031
ENSBTAT00000039086
ENSBTAT00000038564
ENSBTAT00000028893
ENSBTAT00000062956
ENSBTAT00000003005
ENSBTAT00000026034
ENSBTAT00000025502

ENSBTAT00000019148
ENSBTAT00000053430
ENSBTAT00000014499
ENSBTAT00000025880
ENSBTAT00000019716
ENSBTAT00000054328
ENSBTAT00000029250
ENSBTAT00000012820
ENSBTAT00000015841
ENSBTAT00000046779
ENSBTAT00000054188
ENSBTAT00000026028
ENSBTAT00000064254
ENSBTAT00000023700
ENSBTAT00000050396
ENSBTAT00000004006
ENSBTAT00000054347
ENSBTAT00000029023
ENSBTAT00000007698
ENSBTAT00000001416
ENSBTAT00000052730
ENSBTAT00000018365
ENSBTAT00000064433
ENSBTAT00000008264
ENSBTAT00000043056
ENSBTAT00000014364
ENSBTAT00000017976
ENSBTAT00000011744
ENSBTAT00000013795
ENSBTAT00000018536

ENSBTAT00000027387
ENSBTAT00000032980
ENSBTAT00000030324
ENSBTAT00000007484
ENSBTAT00000017703
ENSBTAT00000010748
ENSBTAT00000029100
ENSBTAT00000008157
ENSBTAT00000055903
ENSBTAT00000007146
ENSBTAT00000045774
ENSBTAT00000054272
ENSBTAT00000021789
ENSBTAT00000036420
ENSBTAT00000001802
ENSBTAT00000012174
ENSBTAT00000027504
ENSBTAT00000053488
ENSBTAT00000017950
ENSBTAT00000007538
ENSBTAT00000042823
ENSBTAT00000000539
ENSBTAT00000014520
ENSBTAT00000008381
ENSBTAT00000027357
ENSBTAT00000018789
ENSBTAT00000043127
ENSBTAT00000025650
ENSBTAT00000065406
ENSBTAT00000010237

ENSBTAT00000006068
ENSBTAT00000065800
ENSBTAT00000019973
ENSBTAT00000022212
ENSBTAT00000015389
ENSBTAT00000027338
ENSBTAT00000031311
ENSBTAT00000009824
ENSBTAT00000024758
ENSBTAT00000002028
ENSBTAT00000057506
ENSBTAT00000056411
ENSBTAT00000023213
ENSBTAT00000008844
ENSBTAT00000036866
ENSBTAT00000006050
ENSBTAT00000011013
ENSBTAT00000052213
ENSBTAT00000011408
ENSBTAT00000063795
ENSBTAT00000028125
ENSBTAT00000017194
ENSBTAT00000011024
ENSBTAT00000063256
ENSBTAT00000024028
ENSBTAT00000001288
ENSBTAT00000020547
ENSBTAT00000022753
ENSBTAT00000011264
ENSBTAT00000010617

ENSBTAT00000036299
ENSBTAT00000030135
ENSBTAT00000014131
ENSBTAT00000002720
ENSBTAT00000043784
ENSBTAT00000024223
ENSBTAT00000015778
ENSBTAT00000024078
ENSBTAT00000012921
ENSBTAT00000054712
ENSBTAT00000013838
ENSBTAT00000063359
ENSBTAT00000056935
ENSBTAT00000008234
ENSBTAT00000061487
ENSBTAT00000046889
ENSBTAT00000043790
ENSBTAT00000062262
ENSBTAT00000012650
ENSBTAT00000013887
ENSBTAT00000000283
ENSBTAT00000065541
ENSBTAT00000000937
ENSBTAT00000048078
ENSBTAT00000019529
ENSBTAT00000057249
ENSBTAT00000064295
ENSBTAT00000002045
ENSBTAT00000035430
ENSBTAT00000018587

ENSBTAT00000065570
ENSBTAT00000005060
ENSBTAT00000023734
ENSBTAT00000016565
ENSBTAT00000016358
ENSBTAT00000009403
ENSBTAT00000005062
ENSBTAT00000004655
ENSBTAT00000066089
ENSBTAT00000052820
ENSBTAT00000027059
ENSBTAT00000019161
ENSBTAT00000001144
ENSBTAT00000007133
ENSBTAT00000053586
ENSBTAT00000055905
ENSBTAT00000015716
ENSBTAT00000064601
ENSBTAT00000043252
ENSBTAT00000044927
ENSBTAT00000026146
ENSBTAT00000028669
ENSBTAT00000023182
ENSBTAT00000063099
ENSBTAT00000053657
ENSBTAT00000016946
ENSBTAT00000029532
ENSBTAT00000008936
ENSBTAT00000036072
ENSBTAT00000026230

ENSBTAT00000015746
ENSBTAT00000054707
ENSBTAT00000009864
ENSBTAT00000019129
ENSBTAT00000006684
ENSBTAT00000022217
ENSBTAT00000014112
ENSBTAT00000017267
ENSBTAT00000045518
ENSBTAT00000063581
ENSBTAT00000053398
ENSBTAT00000013116
ENSBTAT00000064403
ENSBTAT00000061578
ENSBTAT00000003768
ENSBTAT00000043154
ENSBTAT00000026267
ENSBTAT00000008330
ENSBTAT00000064434
ENSBTAT00000000477
ENSBTAT00000043552
ENSBTAT00000017628
ENSBTAT00000013006
ENSBTAT00000063185
ENSBTAT00000043987
ENSBTAT00000024049
ENSBTAT00000027222
ENSBTAT00000042953
ENSBTAT00000043409
ENSBTAT00000057200

ENSBTAT00000005042
ENSBTAT00000004932
ENSBTAT00000025934
ENSBTAT00000019611
ENSBTAT00000013231
ENSBTAT00000012731
ENSBTAT00000043597
ENSBTAT00000040129
ENSBTAT00000001461
ENSBTAT00000011578
ENSBTAT00000032977
ENSBTAT00000065950
ENSBTAT00000022017
ENSBTAT00000034734
ENSBTAT00000004019
ENSBTAT00000004167
ENSBTAT00000066326
ENSBTAT00000048167
ENSBTAT00000028349
ENSBTAT00000055611
ENSBTAT00000019759
ENSBTAT00000013117
ENSBTAT00000020090
ENSBTAT00000018555
ENSBTAT00000045760
ENSBTAT00000015617
ENSBTAT00000025464
ENSBTAT00000018131
ENSBTAT00000008501
ENSBTAT00000024634

ENSBTAT00000004287
ENSBTAT00000003732
ENSBTAT00000003591
ENSBTAT00000064071
ENSBTAT00000034683
ENSBTAT00000032973
ENSBTAT00000053684
ENSBTAT00000029264
ENSBTAT00000005182
ENSBTAT00000022547
ENSBTAT00000015056
ENSBTAT00000005506
ENSBTAT00000026396
ENSBTAT00000018492
ENSBTAT00000001988
ENSBTAT00000005795
ENSBTAT00000019530
ENSBTAT00000043437
ENSBTAT00000010444
ENSBTAT00000062996
ENSBTAT00000028590
ENSBTAT00000063300
ENSBTAT00000029261
ENSBTAT00000022750
ENSBTAT00000052566
ENSBTAT00000015383
ENSBTAT00000035104
ENSBTAT00000007916
ENSBTAT00000066281
ENSBTAT00000005552

ENSBTAT00000005370
ENSBTAT00000039410
ENSBTAT00000004527
ENSBTAT00000056426
ENSBTAT00000025159
ENSBTAT00000010999
ENSBTAT00000052066
ENSBTAT00000022387
ENSBTAT00000011995
ENSBTAT00000028610
ENSBTAT00000032217
ENSBTAT00000028571
ENSBTAT00000019137
ENSBTAT00000027608
ENSBTAT00000004556
ENSBTAT00000061198
ENSBTAT00000012946
ENSBTAT00000024074
ENSBTAT00000018806
ENSBTAT00000025570
ENSBTAT00000012229
ENSBTAT00000004127
ENSBTAT00000054262
ENSBTAT00000024485
ENSBTAT00000025466
ENSBTAT00000056441
ENSBTAT00000002439
ENSBTAT00000025389
ENSBTAT00000012378
ENSBTAT00000021704

ENSBTAT00000044642
ENSBTAT00000009220
ENSBTAT00000043454
ENSBTAT00000006465
ENSBTAT00000065092
ENSBTAT00000045228
ENSBTAT00000027172
ENSBTAT00000036905
ENSBTAT00000027147
ENSBTAT00000039179
ENSBTAT00000000851
ENSBTAT00000007450
ENSBTAT00000000595
ENSBTAT00000000044
ENSBTAT00000009028
ENSBTAT00000053498
ENSBTAT00000054949
ENSBTAT00000047612
ENSBTAT00000036999
ENSBTAT00000053025
ENSBTAT00000045591
ENSBTAT00000016198
ENSBTAT00000024399
ENSBTAT00000018722
ENSBTAT00000056960
ENSBTAT00000044138
ENSBTAT00000010779
ENSBTAT00000054047
ENSBTAT00000000704
ENSBTAT00000064156

ENSBTAT00000043518
ENSBTAT00000009151
ENSBTAT00000055788
ENSBTAT00000001735
ENSBTAT00000003618
ENSBTAT00000002270
ENSBTAT00000064829
ENSBTAT00000044075
ENSBTAT00000008715
ENSBTAT00000034642
ENSBTAT00000066312
ENSBTAT00000010991
ENSBTAT00000025454
ENSBTAT00000064929
ENSBTAT00000063217
ENSBTAT00000013968
ENSBTAT00000007682
ENSBTAT00000029216
ENSBTAT00000006532
ENSBTAT00000002578
ENSBTAT00000065477
ENSBTAT00000049751
ENSBTAT00000004268
ENSBTAT00000050739
ENSBTAT00000019411
ENSBTAT00000008357
ENSBTAT00000027462
ENSBTAT00000020289
ENSBTAT00000027256
ENSBTAT00000013918

ENSBTAT00000022696
ENSBTAT00000025363
ENSBTAT00000034065
ENSBTAT00000044315
ENSBTAT00000065846
ENSBTAT00000052678
ENSBTAT00000000737
ENSBTAT00000024198
ENSBTAT00000065602
ENSBTAT00000007397
ENSBTAT00000023970
ENSBTAT00000065450
ENSBTAT00000060569
ENSBTAT00000030664
ENSBTAT00000006231
ENSBTAT00000064659
ENSBTAT00000054307
ENSBTAT00000021674
ENSBTAT00000010998
ENSBTAT00000063238
ENSBTAT00000027140
ENSBTAT00000002790
ENSBTAT00000002664
ENSBTAT00000028937
ENSBTAT00000038532
ENSBTAT00000064597
ENSBTAT00000049195
ENSBTAT00000024663
ENSBTAT00000054323
ENSBTAT00000023476

ENSBTAT00000050147
ENSBTAT00000056753
ENSBTAT00000022302
ENSBTAT00000044000
ENSBTAT00000000422
ENSBTAT00000026716
ENSBTAT00000065312
ENSBTAT00000043822
ENSBTAT00000044829
ENSBTAT00000053130
ENSBTAT00000019669
ENSBTAT00000063703
ENSBTAT00000035548
ENSBTAT00000053379
ENSBTAT00000004406
ENSBTAT00000055896
ENSBTAT00000054550
ENSBTAT00000056859
ENSBTAT00000038974
ENSBTAT00000014920
ENSBTAT00000037984
ENSBTAT00000008639
ENSBTAT00000004684
ENSBTAT00000036501
ENSBTAT00000023098
ENSBTAT00000035844
ENSBTAT00000029669
ENSBTAT00000015932
ENSBTAT00000005747
ENSBTAT00000002710

ENSBTAT00000044729
ENSBTAT00000011978
ENSBTAT00000006097
ENSBTAT00000012309
ENSBTAT00000022472
ENSBTAT00000042995
ENSBTAT00000044822
ENSBTAT00000038015
ENSBTAT00000053240
ENSBTAT00000061032
ENSBTAT00000035516
ENSBTAT00000017333
ENSBTAT00000056701
ENSBTAT00000024363
ENSBTAT00000052295
ENSBTAT00000024366
ENSBTAT00000062952
ENSBTAT00000063639
ENSBTAT00000011265
ENSBTAT00000064053
ENSBTAT00000009126
ENSBTAT00000004752
ENSBTAT00000052030
ENSBTAT00000002387
ENSBTAT00000030496
ENSBTAT00000045048
ENSBTAT00000026634
ENSBTAT00000050183
ENSBTAT00000005216
ENSBTAT00000045594

ENSBTAT00000021243
ENSBTAT00000013548
ENSBTAT00000011449
ENSBTAT00000034240
ENSBTAT00000054300
ENSBTAT00000049093
ENSBTAT00000000458
ENSBTAT00000000233
ENSBTAT00000044898
ENSBTAT00000010653
ENSBTAT00000009687
ENSBTAT00000047409
ENSBTAT00000018317
ENSBTAT00000065359
ENSBTAT00000020009
ENSBTAT00000033253
ENSBTAT00000029032
ENSBTAT00000030625
ENSBTAT00000013932
ENSBTAT00000054202
ENSBTAT00000002376
ENSBTAT00000003015
ENSBTAT00000016160
ENSBTAT00000048201
ENSBTAT00000016317
ENSBTAT00000065256
ENSBTAT00000014524
ENSBTAT00000005528
ENSBTAT00000035803
ENSBTAT00000002176

ENSBTAT00000026119
ENSBTAT00000015827
ENSBTAT00000035936
ENSBTAT00000006587
ENSBTAT00000063372
ENSBTAT00000001490
ENSBTAT00000010480
ENSBTAT00000015924
ENSBTAT00000055936
ENSBTAT00000020549
ENSBTAT00000064291
ENSBTAT00000028562
ENSBTAT00000031291
ENSBTAT00000017951
ENSBTAT00000023578
ENSBTAT00000056380
ENSBTAT00000024149
ENSBTAT00000012884
ENSBTAT00000011920
ENSBTAT00000018153
ENSBTAT00000044780
ENSBTAT00000052837
ENSBTAT00000063269
ENSBTAT00000040222
ENSBTAT00000004187
ENSBTAT00000032362
ENSBTAT00000007083
ENSBTAT00000004198
ENSBTAT00000035769
ENSBTAT00000006977

ENSBTAT00000010966
ENSBTAT00000050472
ENSBTAT00000023992
ENSBTAT00000000786
ENSBTAT00000065979
ENSBTAT00000044438
ENSBTAT00000002532
ENSBTAT00000045223
ENSBTAT00000001380
ENSBTAT00000054304
ENSBTAT00000020869
ENSBTAT00000063036
ENSBTAT00000014661
ENSBTAT00000045740
ENSBTAT00000005359
ENSBTAT00000011341
ENSBTAT00000005698
ENSBTAT00000065591
ENSBTAT00000056199
ENSBTAT00000011030
ENSBTAT00000013451
ENSBTAT00000064173
ENSBTAT00000064485
ENSBTAT00000012471
ENSBTAT00000002916
ENSBTAT00000006980
ENSBTAT00000042610
ENSBTAT00000034807
ENSBTAT00000055419
ENSBTAT00000021229

ENSBTAT00000063848
ENSBTAT00000021105
ENSBTAT00000001962
ENSBTAT00000065585
ENSBTAT00000064195
ENSBTAT00000005511
ENSBTAT00000028000
ENSBTAT00000008811
ENSBTAT00000020361
ENSBTAT00000037557
ENSBTAT00000064146
ENSBTAT00000066165
ENSBTAT00000024107
ENSBTAT00000037047
ENSBTAT00000009616
ENSBTAT00000064799
ENSBTAT00000045435
ENSBTAT00000012872
ENSBTAT00000006178
ENSBTAT00000034135
ENSBTAT00000024766
ENSBTAT00000014821
ENSBTAT00000015411
ENSBTAT00000024007
ENSBTAT00000002451
ENSBTAT00000001819
ENSBTAT00000035381
ENSBTAT00000055858
ENSBTAT00000003504
ENSBTAT00000011457

ENSBTAT00000012469
ENSBTAT00000020881
ENSBTAT00000039270
ENSBTAT00000007399
ENSBTAT00000020332
ENSBTAT00000014907
ENSBTAT00000063164
ENSBTAT00000052346
ENSBTAT00000049133
ENSBTAT00000005406
ENSBTAT00000017923
ENSBTAT00000054362
ENSBTAT00000023204
ENSBTAT00000048878
ENSBTAT00000006960
ENSBTAT00000056233
ENSBTAT00000008351
ENSBTAT00000049737
ENSBTAT00000004161
ENSBTAT00000002027
ENSBTAT00000006118
ENSBTAT00000061318
ENSBTAT00000027056
ENSBTAT00000006275
ENSBTAT00000007469
ENSBTAT00000018241
ENSBTAT00000001011
ENSBTAT00000050141
ENSBTAT00000038601
ENSBTAT00000012845

ENSBTAT00000054850
ENSBTAT00000045323
ENSBTAT00000038531
ENSBTAT00000023357
ENSBTAT00000002846
ENSBTAT00000019065
ENSBTAT00000063575
ENSBTAT00000052501
ENSBTAT00000038770
ENSBTAT00000063647
ENSBTAT00000000991
ENSBTAT00000025792
ENSBTAT00000065927
ENSBTAT00000034413
ENSBTAT00000003635
ENSBTAT00000026280
ENSBTAT00000016760
ENSBTAT00000066269
ENSBTAT00000061283
ENSBTAT00000036408
ENSBTAT00000013876
ENSBTAT00000009977
ENSBTAT00000018041
ENSBTAT00000063834
ENSBTAT00000006866
ENSBTAT00000052575
ENSBTAT00000013687
ENSBTAT00000045942
ENSBTAT00000027111
ENSBTAT00000008895

ENSBTAT00000001066
ENSBTAT00000003815
ENSBTAT00000064394
ENSBTAT00000015915
ENSBTAT00000018213
ENSBTAT00000005678
ENSBTAT00000002832
ENSBTAT00000009799
ENSBTAT00000014868
ENSBTAT00000064908
ENSBTAT00000056786
ENSBTAT00000066126
ENSBTAT00000011401
ENSBTAT00000027255
ENSBTAT00000066143
ENSBTAT00000053092
ENSBTAT00000020710
ENSBTAT00000023087
ENSBTAT00000011311
ENSBTAT00000020668
ENSBTAT00000055384
ENSBTAT00000003320
ENSBTAT00000021782
ENSBTAT00000036672
ENSBTAT00000065455
ENSBTAT00000001482
ENSBTAT00000036489
ENSBTAT00000011730
ENSBTAT00000026059
ENSBTAT00000054080

ENSBTAT00000013323
ENSBTAT00000024024
ENSBTAT00000028802
ENSBTAT00000002978
ENSBTAT00000011099
ENSBTAT00000063824
ENSBTAT00000004122
ENSBTAT00000046636
ENSBTAT00000022423
ENSBTAT00000063195
ENSBTAT00000065209
ENSBTAT00000013401
ENSBTAT00000026550
ENSBTAT00000027371
ENSBTAT00000023236
ENSBTAT00000024996
ENSBTAT00000021606
ENSBTAT00000027160
ENSBTAT00000061217
ENSBTAT00000004683
ENSBTAT00000008833
ENSBTAT00000011412
ENSBTAT00000006327
ENSBTAT00000055047
ENSBTAT00000021827
ENSBTAT00000065579
ENSBTAT00000008725
ENSBTAT00000006374
ENSBTAT00000020940
ENSBTAT00000019623

ENSBTAT00000057350
ENSBTAT00000020388
ENSBTAT00000048657
ENSBTAT00000052971
ENSBTAT00000034773
ENSBTAT00000000014
ENSBTAT00000027019
ENSBTAT00000019892
ENSBTAT00000018253
ENSBTAT00000008123
ENSBTAT00000020266
ENSBTAT00000012395
ENSBTAT00000015886
ENSBTAT00000012036
ENSBTAT00000012054
ENSBTAT00000063219
ENSBTAT00000064882
ENSBTAT00000028746
ENSBTAT00000025657
ENSBTAT00000046451
ENSBTAT00000016953
ENSBTAT00000043435
ENSBTAT00000009469
ENSBTAT00000011487
ENSBTAT00000025164
ENSBTAT00000043851
ENSBTAT00000037298
ENSBTAT00000063915
ENSBTAT00000065653
ENSBTAT00000006726

ENSBTAT00000025038
ENSBTAT00000009909
ENSBTAT00000063277
ENSBTAT00000052461
ENSBTAT00000001844
ENSBTAT00000020860
ENSBTAT00000038298
ENSBTAT00000048797
ENSBTAT00000064215
ENSBTAT00000015299
ENSBTAT00000025259
ENSBTAT00000031053
ENSBTAT00000061544
ENSBTAT00000021100
ENSBTAT00000000089
ENSBTAT00000045778
ENSBTAT00000064529
ENSBTAT00000008359
ENSBTAT00000008361
ENSBTAT00000063551
ENSBTAT00000018914
ENSBTAT00000033918
ENSBTAT00000001952
ENSBTAT00000063249
ENSBTAT00000013695
ENSBTAT00000024529
ENSBTAT00000048598
ENSBTAT00000055549
ENSBTAT00000023625
ENSBTAT00000050805

ENSBTAT00000017844
ENSBTAT00000004163
ENSBTAT00000056961
ENSBTAT00000020438
ENSBTAT00000003042
ENSBTAT00000037465
ENSBTAT00000024240
ENSBTAT00000064748
ENSBTAT00000008954
ENSBTAT00000013417
ENSBTAT00000040049
ENSBTAT00000003098
ENSBTAT00000012317
ENSBTAT00000064948
ENSBTAT00000024749
ENSBTAT00000024292
ENSBTAT00000013270
ENSBTAT00000019185
ENSBTAT00000003072
ENSBTAT00000001888
ENSBTAT00000029144
ENSBTAT00000019169
ENSBTAT00000025615
ENSBTAT00000056003
ENSBTAT00000008819
ENSBTAT00000023419
ENSBTAT00000028151
ENSBTAT00000028552
ENSBTAT00000065441
ENSBTAT00000022304

ENSBTAT00000053875
ENSBTAT00000010694
ENSBTAT00000000588
ENSBTAT00000007132
ENSBTAT00000037682
ENSBTAT00000012042
ENSBTAT00000063797
ENSBTAT00000020153
ENSBTAT00000019242
ENSBTAT00000039564
ENSBTAT00000006714
ENSBTAT00000044676
ENSBTAT00000063114
ENSBTAT00000008500
ENSBTAT00000020595
ENSBTAT00000049517
ENSBTAT00000031335
ENSBTAT00000027963
ENSBTAT00000030297
ENSBTAT00000006022
ENSBTAT00000027334
ENSBTAT00000043427
ENSBTAT00000065052
ENSBTAT00000012106
ENSBTAT00000011395
ENSBTAT00000018111
ENSBTAT00000053944
ENSBTAT00000009271
ENSBTAT00000047907
ENSBTAT00000014422

ENSBTAT00000021201
ENSBTAT00000030194
ENSBTAT00000033237
ENSBTAT00000003937
ENSBTAT00000035878
ENSBTAT00000023029
ENSBTAT00000009295
ENSBTAT00000022357
ENSBTAT00000042833
ENSBTAT00000028146
ENSBTAT00000064083
ENSBTAT00000002602
ENSBTAT00000025981
ENSBTAT00000050603
ENSBTAT00000010604
ENSBTAT00000054735
ENSBTAT00000001531
ENSBTAT00000044120
ENSBTAT00000000604
ENSBTAT00000056156
ENSBTAT00000036701
ENSBTAT00000023945
ENSBTAT00000019763
ENSBTAT00000061342
ENSBTAT00000046575
ENSBTAT00000027125
ENSBTAT00000038099
ENSBTAT00000023934
ENSBTAT00000011510
ENSBTAT00000064473

ENSBTAT00000016385
ENSBTAT00000029564
ENSBTAT00000002779
ENSBTAT00000018782
ENSBTAT00000022625
ENSBTAT00000028798
ENSBTAT00000005681
ENSBTAT00000010906
ENSBTAT00000030757
ENSBTAT00000022113
ENSBTAT00000007549
ENSBTAT00000065149
ENSBTAT00000026209
ENSBTAT00000044965
ENSBTAT00000065497
ENSBTAT00000026779
ENSBTAT00000000061
ENSBTAT00000009718
ENSBTAT00000000497
ENSBTAT00000002263
ENSBTAT00000063124
ENSBTAT00000010606
ENSBTAT00000052926
ENSBTAT00000051941
ENSBTAT00000023167
ENSBTAT00000027055
ENSBTAT00000016916
ENSBTAT00000023021
ENSBTAT00000000484
ENSBTAT00000040022

ENSBTAT00000011639
ENSBTAT00000008619
ENSBTAT00000066300
ENSBTAT00000012410
ENSBTAT00000055185
ENSBTAT00000043142
ENSBTAT00000013362
ENSBTAT00000049498
ENSBTAT00000026048
ENSBTAT00000004442
ENSBTAT00000027547
ENSBTAT00000006954
ENSBTAT00000014709
ENSBTAT00000024011
ENSBTAT00000052576
ENSBTAT00000035149
ENSBTAT00000064554
ENSBTAT00000064753
ENSBTAT00000063302
ENSBTAT00000004724
ENSBTAT00000001640
ENSBTAT00000008696
ENSBTAT00000018420
ENSBTAT00000024940
ENSBTAT00000013364
ENSBTAT00000064573
ENSBTAT00000005810
ENSBTAT00000007331
ENSBTAT00000002826
ENSBTAT00000019943

ENSBTAT00000006585
ENSBTAT00000021104
ENSBTAT00000007200
ENSBTAT00000001923
ENSBTAT00000040261
ENSBTAT00000019737
ENSBTAT00000009438
ENSBTAT00000062932
ENSBTAT00000018787
ENSBTAT00000044590
ENSBTAT00000056150
ENSBTAT00000012263
ENSBTAT00000054308
ENSBTAT00000032542
ENSBTAT00000017660
ENSBTAT00000022393
ENSBTAT00000036961
ENSBTAT00000019114
ENSBTAT00000055254
ENSBTAT00000037014
ENSBTAT00000063047
ENSBTAT00000015978
ENSBTAT00000009694
ENSBTAT00000000564
ENSBTAT00000063684
ENSBTAT00000061595
ENSBTAT00000063033
ENSBTAT00000057258
ENSBTAT00000064240
ENSBTAT00000044924

ENSBTAT00000002607
ENSBTAT00000013688
ENSBTAT00000056820
ENSBTAT00000037589
ENSBTAT00000054448
ENSBTAT00000044432
ENSBTAT00000010255
ENSBTAT00000001392
ENSBTAT00000034259
ENSBTAT00000055603
ENSBTAT00000064654
ENSBTAT00000047205
ENSBTAT00000009328
ENSBTAT00000009844
ENSBTAT00000055476
ENSBTAT00000018184
ENSBTAT00000065040
ENSBTAT00000052924
ENSBTAT00000015726
ENSBTAT00000006877
ENSBTAT00000065908
ENSBTAT00000022484
ENSBTAT00000011645
ENSBTAT00000057469
ENSBTAT00000017600
ENSBTAT00000025873
ENSBTAT00000011688
ENSBTAT00000045191
ENSBTAT00000045699
ENSBTAT00000037205

ENSBTAT00000004465
ENSBTAT00000018343
ENSBTAT00000009741
ENSBTAT00000014402
ENSBTAT00000061105
ENSBTAT00000010294
ENSBTAT00000056578
ENSBTAT00000008942
ENSBTAT00000010454
ENSBTAT00000035766
ENSBTAT00000028786
ENSBTAT00000026937
ENSBTAT00000008975
ENSBTAT00000056475
ENSBTAT00000006353
ENSBTAT00000000410
ENSBTAT00000001959
ENSBTAT00000030851
ENSBTAT00000002247
ENSBTAT00000056138
ENSBTAT00000032529
ENSBTAT00000012348
ENSBTAT00000066131
ENSBTAT00000013515
ENSBTAT00000043890
ENSBTAT00000003585
ENSBTAT00000052988
ENSBTAT00000045068
ENSBTAT00000020152
ENSBTAT00000063810

ENSBTAT00000014089
ENSBTAT00000016159
ENSBTAT00000052907
ENSBTAT00000011713
ENSBTAT00000049339
ENSBTAT00000025391
ENSBTAT00000046566
ENSBTAT00000000800
ENSBTAT00000013398
ENSBTAT00000002109
ENSBTAT00000054093
ENSBTAT00000021401
ENSBTAT00000036043
ENSBTAT00000013519
ENSBTAT00000037734
ENSBTAT00000014058
ENSBTAT00000051882
ENSBTAT00000011191
ENSBTAT00000023770
ENSBTAT00000053029
ENSBTAT00000013338
ENSBTAT00000053219
ENSBTAT00000055483
ENSBTAT00000021003
ENSBTAT00000037275
ENSBTAT00000038118
ENSBTAT00000066036
ENSBTAT00000020822
ENSBTAT00000008918
ENSBTAT00000035020

ENSBTAT00000009560
ENSBTAT00000008802
ENSBTAT00000015413
ENSBTAT00000049635
ENSBTAT00000043370
ENSBTAT00000014919
ENSBTAT00000027243
ENSBTAT00000002984
ENSBTAT00000028955
ENSBTAT00000056257
ENSBTAT00000011140
ENSBTAT00000046785
ENSBTAT00000003341
ENSBTAT00000011955
ENSBTAT00000049389
ENSBTAT00000016520
ENSBTAT00000025071
ENSBTAT00000049094
ENSBTAT00000065607
ENSBTAT00000032165
ENSBTAT00000055528
ENSBTAT00000015039
ENSBTAT00000056100
ENSBTAT00000003470
ENSBTAT00000006606
ENSBTAT00000007711
ENSBTAT00000008268
ENSBTAT00000007883
ENSBTAT00000002282
ENSBTAT00000065516

ENSBTAT00000031716
ENSBTAT00000014039
ENSBTAT00000054642
ENSBTAT00000000461
ENSBTAT00000026212
ENSBTAT00000023179
ENSBTAT00000007987
ENSBTAT00000003997
ENSBTAT00000002552
ENSBTAT00000052580
ENSBTAT00000055237
ENSBTAT00000036270
ENSBTAT00000062963
ENSBTAT00000011778
ENSBTAT00000052480
ENSBTAT00000010960
ENSBTAT00000053421
ENSBTAT00000004472
ENSBTAT00000003619
ENSBTAT00000055300
ENSBTAT00000018746
ENSBTAT00000012476
ENSBTAT00000018626
ENSBTAT00000052977
ENSBTAT00000016852
ENSBTAT00000044672
ENSBTAT00000019433
ENSBTAT00000001897
ENSBTAT00000063261
ENSBTAT00000038334

ENSBTAT00000021357
ENSBTAT00000017422
ENSBTAT00000064021
ENSBTAT00000016176
ENSBTAT00000054423
ENSBTAT00000039087
ENSBTAT00000036789
ENSBTAT00000055361
ENSBTAT00000007595
ENSBTAT00000057033
ENSBTAT00000000396
ENSBTAT00000007556
ENSBTAT00000019791
ENSBTAT00000037368
ENSBTAT00000027509
ENSBTAT00000017091
ENSBTAT00000021677
ENSBTAT00000054833
ENSBTAT00000018285
ENSBTAT00000044682
ENSBTAT00000028155
ENSBTAT00000020200
ENSBTAT00000011211
ENSBTAT00000030528
ENSBTAT00000030589
ENSBTAT00000032016
ENSBTAT00000001878
ENSBTAT00000046748
ENSBTAT00000046105
ENSBTAT00000016154

ENSBTAT00000011804
ENSBTAT00000017392
ENSBTAT00000005622
ENSBTAT00000013033
ENSBTAT00000028307
ENSBTAT00000027699
ENSBTAT00000028899
ENSBTAT00000027787
ENSBTAT00000047352
ENSBTAT00000029167
ENSBTAT00000044712
ENSBTAT00000018256
ENSBTAT00000017593
ENSBTAT00000015161
ENSBTAT00000061070
ENSBTAT00000063030
ENSBTAT00000027187
ENSBTAT00000024228
ENSBTAT00000008633
ENSBTAT00000020928
ENSBTAT00000030260
ENSBTAT00000063982
ENSBTAT00000066035
ENSBTAT00000027890
ENSBTAT00000064809
ENSBTAT00000056993
ENSBTAT00000025736
ENSBTAT00000057240
ENSBTAT00000065260
ENSBTAT00000019321

ENSBTAT00000001437
ENSBTAT00000020942
ENSBTAT00000014663
ENSBTAT00000066161
ENSBTAT00000054106
ENSBTAT00000018242
ENSBTAT00000025231
ENSBTAT00000008030
ENSBTAT00000014758
ENSBTAT00000016674
ENSBTAT00000005592
ENSBTAT00000054266
ENSBTAT00000037230
ENSBTAT00000031089
ENSBTAT00000066189
ENSBTAT00000011453
ENSBTAT00000022752
ENSBTAT00000005277
ENSBTAT00000017995
ENSBTAT00000056032
ENSBTAT00000064464
ENSBTAT00000022843
ENSBTAT00000011858
ENSBTAT00000012940
ENSBTAT00000046067
ENSBTAT00000026551
ENSBTAT00000002434
ENSBTAT00000027625
ENSBTAT00000064383
ENSBTAT00000055953

ENSBTAT00000052136
ENSBTAT00000031107
ENSBTAT00000046902
ENSBTAT00000065348
ENSBTAT00000056828
ENSBTAT00000056556
ENSBTAT00000016010
ENSBTAT00000005163
ENSBTAT00000028551
ENSBTAT00000001114
ENSBTAT00000008284
ENSBTAT00000036100
ENSBTAT00000021120
ENSBTAT00000008837
ENSBTAT00000020159
ENSBTAT00000005043
ENSBTAT00000010773
ENSBTAT00000007447
ENSBTAT00000023480
ENSBTAT00000020709
ENSBTAT00000017630
ENSBTAT00000005527
ENSBTAT00000013696
ENSBTAT00000008651
ENSBTAT00000052149
ENSBTAT00000065856
ENSBTAT00000019225
ENSBTAT00000065194
ENSBTAT00000023907
ENSBTAT00000065819

ENSBTAT00000011586
ENSBTAT00000017425
ENSBTAT00000057184
ENSBTAT00000022253
ENSBTAT00000028662
ENSBTAT00000064645
ENSBTAT00000002733
ENSBTAT00000055437
ENSBTAT00000008485
ENSBTAT00000008649
ENSBTAT00000055838
ENSBTAT00000013625
ENSBTAT00000054434
ENSBTAT00000012015
ENSBTAT00000014447
ENSBTAT00000022801
ENSBTAT00000055090
ENSBTAT00000009330
ENSBTAT00000013559
ENSBTAT00000015639
ENSBTAT00000029403
ENSBTAT00000063058
ENSBTAT00000033676
ENSBTAT00000005377
ENSBTAT00000016618
ENSBTAT00000025773
ENSBTAT00000003192
ENSBTAT00000010869
ENSBTAT00000063394
ENSBTAT00000012514

ENSBTAT00000064629
ENSBTAT00000006105
ENSBTAT00000034449
ENSBTAT00000011897
ENSBTAT00000007176
ENSBTAT00000055491
ENSBTAT00000011447
ENSBTAT00000016056
ENSBTAT00000021168
ENSBTAT00000025822
ENSBTAT00000024233
ENSBTAT00000053644
ENSBTAT00000035966
ENSBTAT00000045509
ENSBTAT00000017584
ENSBTAT00000052513
ENSBTAT00000005734
ENSBTAT00000005239
ENSBTAT00000010949
ENSBTAT00000035354
ENSBTAT00000014000
ENSBTAT00000051911
ENSBTAT00000063572
ENSBTAT00000011832
ENSBTAT00000053278
ENSBTAT00000000837
ENSBTAT00000061353
ENSBTAT00000054535
ENSBTAT00000013443
ENSBTAT00000015301

ENSBTAT00000049720
ENSBTAT00000019601
ENSBTAT00000004201
ENSBTAT00000056189
ENSBTAT00000031191
ENSBTAT00000036932
ENSBTAT00000064911
ENSBTAT00000025383
ENSBTAT00000002896
ENSBTAT00000017242
ENSBTAT00000052791
ENSBTAT00000013534
ENSBTAT00000056729
ENSBTAT00000062966
ENSBTAT00000002689
ENSBTAT00000005409
ENSBTAT00000043743
ENSBTAT00000023280
ENSBTAT00000022120
ENSBTAT00000052569
ENSBTAT00000010619
ENSBTAT00000054658
ENSBTAT00000052762
ENSBTAT00000016528
ENSBTAT00000066185
ENSBTAT00000026308
ENSBTAT00000066246
ENSBTAT00000016388
ENSBTAT00000026911
ENSBTAT00000020676

ENSBTAT00000047081
ENSBTAT00000008662
ENSBTAT00000054216
ENSBTAT00000064569
ENSBTAT00000057245
ENSBTAT00000001367
ENSBTAT00000018737
ENSBTAT00000001925
ENSBTAT00000003681
ENSBTAT00000056572
ENSBTAT00000064018
ENSBTAT00000044112
ENSBTAT00000054289
ENSBTAT00000026958
ENSBTAT00000000780
ENSBTAT00000014996
ENSBTAT00000025431
ENSBTAT00000027845
ENSBTAT00000024709
ENSBTAT00000061508
ENSBTAT00000032906
ENSBTAT00000057261
ENSBTAT00000007865
ENSBTAT00000004870
ENSBTAT00000039493
ENSBTAT00000006784
ENSBTAT00000063559
ENSBTAT00000019487
ENSBTAT00000028605
ENSBTAT00000009250

ENSBTAT00000007616
ENSBTAT00000050331
ENSBTAT00000063361
ENSBTAT00000030542
ENSBTAT00000020651
ENSBTAT00000024299
ENSBTAT00000064409
ENSBTAT00000039020
ENSBTAT00000007470
ENSBTAT00000065111
ENSBTAT00000066340
ENSBTAT00000012094
ENSBTAT00000011879
ENSBTAT00000023472
ENSBTAT00000065357
ENSBTAT00000006392
ENSBTAT00000007284
ENSBTAT00000065600
ENSBTAT00000053470
ENSBTAT00000057313
ENSBTAT00000024401
ENSBTAT00000057306
ENSBTAT00000019647
ENSBTAT00000009113
ENSBTAT00000020511
ENSBTAT00000053781
ENSBTAT00000000502
ENSBTAT00000009858
ENSBTAT00000037443
ENSBTAT00000065012

ENSBTAT00000014853
ENSBTAT00000053218
ENSBTAT00000032754
ENSBTAT00000056136
ENSBTAT00000000008
ENSBTAT00000063718
ENSBTAT00000047341
ENSBTAT00000002585
ENSBTAT00000006445
ENSBTAT00000063700
ENSBTAT00000065521
ENSBTAT00000064964
ENSBTAT00000020965
ENSBTAT00000044908
ENSBTAT00000052230
ENSBTAT00000004739
ENSBTAT00000000299
ENSBTAT00000006026
ENSBTAT00000023104
ENSBTAT00000016758
ENSBTAT00000038757
ENSBTAT00000065732
ENSBTAT00000060577
ENSBTAT00000010700
ENSBTAT00000053537
ENSBTAT00000064190
ENSBTAT00000047906
ENSBTAT00000056901
ENSBTAT00000063978
ENSBTAT00000024429

ENSBTAT00000006478
ENSBTAT00000031530
ENSBTAT00000010658
ENSBTAT00000016505
ENSBTAT00000017083
ENSBTAT00000061022
ENSBTAT00000012234
ENSBTAT00000054909
ENSBTAT00000056307
ENSBTAT00000004418
ENSBTAT00000001847
ENSBTAT00000057220
ENSBTAT00000063714
ENSBTAT00000063063
ENSBTAT00000024941
ENSBTAT00000043378
ENSBTAT00000002260
ENSBTAT00000035505
ENSBTAT00000016202
ENSBTAT00000063646
ENSBTAT00000056172
ENSBTAT00000023034
ENSBTAT00000008847
ENSBTAT00000009953
ENSBTAT00000022496
ENSBTAT00000064988
ENSBTAT00000053553
ENSBTAT00000011922
ENSBTAT00000046307
ENSBTAT00000037254

ENSBTAT00000049199
ENSBTAT00000014880
ENSBTAT00000063242
ENSBTAT00000066287
ENSBTAT00000011768
ENSBTAT00000049560
ENSBTAT00000017277
ENSBTAT00000024171
ENSBTAT00000066038
ENSBTAT00000033229
ENSBTAT00000063032
ENSBTAT00000039375
ENSBTAT00000007481
ENSBTAT00000017248
ENSBTAT00000048631
ENSBTAT00000040335
ENSBTAT00000037519
ENSBTAT00000003497
ENSBTAT00000017664
ENSBTAT00000014244
ENSBTAT00000013201
ENSBTAT00000003296
ENSBTAT00000054484
ENSBTAT00000031535
ENSBTAT00000001384
ENSBTAT00000035207
ENSBTAT00000061359
ENSBTAT00000056344
ENSBTAT00000009739
ENSBTAT00000008423

ENSBTAT00000029707
ENSBTAT00000028051
ENSBTAT00000056746
ENSBTAT00000044904
ENSBTAT00000002652
ENSBTAT00000056675
ENSBTAT00000044505
ENSBTAT00000039896
ENSBTAT00000038449
ENSBTAT00000004233
ENSBTAT00000026306
ENSBTAT00000008808
ENSBTAT00000032325
ENSBTAT00000032609
ENSBTAT00000038197
ENSBTAT00000046011
ENSBTAT00000020561
ENSBTAT00000004048
ENSBTAT00000065343
ENSBTAT00000066042
ENSBTAT00000003055
ENSBTAT00000064406
ENSBTAT00000006381
ENSBTAT00000039838
ENSBTAT00000028764
ENSBTAT00000006410
ENSBTAT00000026185
ENSBTAT00000035069
ENSBTAT00000050193
ENSBTAT00000020400

ENSBTAT00000017828
ENSBTAT00000014877
ENSBTAT00000065771
ENSBTAT00000023205
ENSBTAT00000047487
ENSBTAT00000014016
ENSBTAT00000055059
ENSBTAT00000025435
ENSBTAT00000065220
ENSBTAT00000066335
ENSBTAT00000001214
ENSBTAT00000017744
ENSBTAT00000010310
ENSBTAT00000025838
ENSBTAT00000022561
ENSBTAT00000033730
ENSBTAT00000019519
ENSBTAT00000057102
ENSBTAT00000030305
ENSBTAT00000004671
ENSBTAT00000056197
ENSBTAT00000054270
ENSBTAT00000026239
ENSBTAT00000052324
ENSBTAT00000007238
ENSBTAT00000006604
ENSBTAT00000011800
ENSBTAT00000020248
ENSBTAT00000005451
ENSBTAT00000063521

ENSBTAT00000009856
ENSBTAT00000064143
ENSBTAT00000006679
ENSBTAT00000039536
ENSBTAT00000002617
ENSBTAT00000003923
ENSBTAT00000063675
ENSBTAT00000055100
ENSBTAT00000010865
ENSBTAT00000010751
ENSBTAT00000014410
ENSBTAT00000033135
ENSBTAT00000001984
ENSBTAT00000002809
ENSBTAT00000002011
ENSBTAT00000055484
ENSBTAT00000024080
ENSBTAT00000030695
ENSBTAT00000065852
ENSBTAT00000009761
ENSBTAT00000009552
ENSBTAT00000043320
ENSBTAT00000012905
ENSBTAT00000005068
ENSBTAT00000053429
ENSBTAT00000065958
ENSBTAT00000033775
ENSBTAT00000013804
ENSBTAT00000021497
ENSBTAT00000025481

ENSBTAT00000065078
ENSBTAT00000063764
ENSBTAT00000037026
ENSBTAT00000048651
ENSBTAT00000017099
ENSBTAT00000052953
ENSBTAT00000049235
ENSBTAT00000053317
ENSBTAT00000008406
ENSBTAT00000017810
ENSBTAT00000008517
ENSBTAT00000063770
ENSBTAT00000054398
ENSBTAT00000019297
ENSBTAT00000021482
ENSBTAT00000044060
ENSBTAT00000026679
ENSBTAT00000007900
ENSBTAT00000012866
ENSBTAT00000037271
ENSBTAT00000053032
ENSBTAT00000009548
ENSBTAT00000002315
ENSBTAT00000066336
ENSBTAT00000053096
ENSBTAT00000015331
ENSBTAT00000052263
ENSBTAT00000023209
ENSBTAT00000043166
ENSBTAT00000005121

ENSBTAT00000026776
ENSBTAT00000026124
ENSBTAT00000037724
ENSBTAT00000033358
ENSBTAT00000028840
ENSBTAT00000009996
ENSBTAT00000027341
ENSBTAT00000052191
ENSBTAT00000030963
ENSBTAT00000028574
ENSBTAT00000014296
ENSBTAT00000012633
ENSBTAT00000049318
ENSBTAT00000055062
ENSBTAT00000010601
ENSBTAT00000025989
ENSBTAT00000016573
ENSBTAT00000065555
ENSBTAT00000054597
ENSBTAT00000064465
ENSBTAT00000025112
ENSBTAT00000011080
ENSBTAT00000055016
ENSBTAT00000064483
ENSBTAT00000012535
ENSBTAT00000020929
ENSBTAT00000002684
ENSBTAT00000025866
ENSBTAT00000026574
ENSBTAT00000017162

ENSBTAT00000039933
ENSBTAT00000038451
ENSBTAT00000006500
ENSBTAT00000050604
ENSBTAT00000000925
ENSBTAT00000035004
ENSBTAT00000008138
ENSBTAT00000004148
ENSBTAT00000005802
ENSBTAT00000025468
ENSBTAT00000014138
ENSBTAT00000027915
ENSBTAT00000007701
ENSBTAT00000028887
ENSBTAT00000022409
ENSBTAT00000002929
ENSBTAT00000018598
ENSBTAT00000007050
ENSBTAT00000042918
ENSBTAT00000002301
ENSBTAT00000022723
ENSBTAT00000004238
ENSBTAT00000022956
ENSBTAT00000019761
ENSBTAT00000030920
ENSBTAT00000019167
ENSBTAT00000028550
ENSBTAT00000012212
ENSBTAT00000061143
ENSBTAT00000065362

ENSBTAT00000034744
ENSBTAT00000007803
ENSBTAT00000004502
ENSBTAT00000006865
ENSBTAT00000030309
ENSBTAT00000063307
ENSBTAT00000019596
ENSBTAT00000044879
ENSBTAT00000007087
ENSBTAT00000014027
ENSBTAT00000033844
ENSBTAT00000039305
ENSBTAT00000012877
ENSBTAT00000064651
ENSBTAT00000036721
ENSBTAT00000031885
ENSBTAT00000009672
ENSBTAT00000052335
ENSBTAT00000044735
ENSBTAT00000012046
ENSBTAT00000005418
ENSBTAT00000065496
ENSBTAT00000064558
ENSBTAT00000010129
ENSBTAT00000006198
ENSBTAT00000007636
ENSBTAT00000009379
ENSBTAT00000000733
ENSBTAT00000065162
ENSBTAT00000016718

ENSBTAT00000001549
ENSBTAT00000065091
ENSBTAT00000014212
ENSBTAT00000053934
ENSBTAT00000002582
ENSBTAT00000005307
ENSBTAT00000012706
ENSBTAT00000018911
ENSBTAT00000064354
ENSBTAT00000004604
ENSBTAT00000010270
ENSBTAT00000018721
ENSBTAT00000039850
ENSBTAT00000007649
ENSBTAT00000044029
ENSBTAT00000007596
ENSBTAT00000024227
ENSBTAT00000065207
ENSBTAT00000057521
ENSBTAT00000054494
ENSBTAT00000009988
ENSBTAT00000037233
ENSBTAT00000017493
ENSBTAT00000010936
ENSBTAT00000053250
ENSBTAT00000050368
ENSBTAT00000002226
ENSBTAT00000017451
ENSBTAT00000004327
ENSBTAT00000007461

ENSBTAT00000056599
ENSBTAT00000023242
ENSBTAT00000017837
ENSBTAT00000053065
ENSBTAT00000029280
ENSBTAT00000002691
ENSBTAT00000005031
ENSBTAT00000055588
ENSBTAT00000062955
ENSBTAT00000005171
ENSBTAT00000048179
ENSBTAT00000008008
ENSBTAT00000013083
ENSBTAT00000006094
ENSBTAT00000005720
ENSBTAT00000038656
ENSBTAT00000037425
ENSBTAT00000030398
ENSBTAT00000006785
ENSBTAT00000003875
ENSBTAT00000033652
ENSBTAT00000015449
ENSBTAT00000017294
ENSBTAT00000049418
ENSBTAT00000034627
ENSBTAT00000046532
ENSBTAT00000052094
ENSBTAT00000010364
ENSBTAT00000011406
ENSBTAT00000050188

ENSBTAT00000013193
ENSBTAT00000036068
ENSBTAT00000002952
ENSBTAT00000064186
ENSBTAT00000019398
ENSBTAT00000066144
ENSBTAT00000030147
ENSBTAT00000054989
ENSBTAT00000017003
ENSBTAT00000015689
ENSBTAT00000001397
ENSBTAT00000022266
ENSBTAT00000013436
ENSBTAT00000066141
ENSBTAT00000055920
ENSBTAT00000050476
ENSBTAT00000024152
ENSBTAT00000028825
ENSBTAT00000064709
ENSBTAT00000050119
ENSBTAT00000063652
ENSBTAT00000063206
ENSBTAT00000025309
ENSBTAT00000006913
ENSBTAT00000006728
ENSBTAT00000063259
ENSBTAT00000026804
ENSBTAT00000052327
ENSBTAT00000016186
ENSBTAT00000004132

ENSBTAT00000021334
ENSBTAT00000016998
ENSBTAT00000056243
ENSBTAT00000020113
ENSBTAT00000008370
ENSBTAT00000030493
ENSBTAT00000008235
ENSBTAT00000010143
ENSBTAT00000064277
ENSBTAT00000064660
ENSBTAT00000012769
ENSBTAT00000043163
ENSBTAT00000065608
ENSBTAT00000009025
ENSBTAT00000019005
ENSBTAT00000001353
ENSBTAT00000023979
ENSBTAT00000054096
ENSBTAT00000027919
ENSBTAT00000021159
ENSBTAT00000016243
ENSBTAT00000064965
ENSBTAT00000021795
ENSBTAT00000031376
ENSBTAT00000000021
ENSBTAT00000011745
ENSBTAT00000063769
ENSBTAT00000065806
ENSBTAT00000001205
ENSBTAT00000013967

ENSBTAT00000047926
ENSBTAT00000001700
ENSBTAT00000002625
ENSBTAT00000009921
ENSBTAT00000057288
ENSBTAT00000009340
ENSBTAT00000066279
ENSBTAT00000063844
ENSBTAT00000022448
ENSBTAT00000035608
ENSBTAT00000006008
ENSBTAT00000053175
ENSBTAT00000062993
ENSBTAT00000011643
ENSBTAT00000036655
ENSBTAT00000065928
ENSBTAT00000008754
ENSBTAT00000008924
ENSBTAT00000006185
ENSBTAT00000025417
ENSBTAT00000018161
ENSBTAT00000052685
ENSBTAT00000066225
ENSBTAT00000004302
ENSBTAT00000008774
ENSBTAT00000032269
ENSBTAT00000040292
ENSBTAT00000022827
ENSBTAT00000034516
ENSBTAT00000031279

ENSBTAT00000011112
ENSBTAT00000021373
ENSBTAT00000013248
ENSBTAT00000015122
ENSBTAT00000024994
ENSBTAT00000023247
ENSBTAT00000037458
ENSBTAT00000007147
ENSBTAT00000053056
ENSBTAT00000013713
ENSBTAT00000049875
ENSBTAT00000004437
ENSBTAT00000010501
ENSBTAT00000025758
ENSBTAT00000022554
ENSBTAT00000020543
ENSBTAT00000026622
ENSBTAT00000063408
ENSBTAT00000061601
ENSBTAT00000043402
ENSBTAT00000066040
ENSBTAT00000006236
ENSBTAT00000017152
ENSBTAT00000010043
ENSBTAT00000052679
ENSBTAT00000017558
ENSBTAT00000066306
ENSBTAT00000036817
ENSBTAT00000049975
ENSBTAT00000063356

ENSBTAT00000037882
ENSBTAT00000000314
ENSBTAT00000000407
ENSBTAT00000003964
ENSBTAT00000066183
ENSBTAT00000001347
ENSBTAT00000061277
ENSBTAT00000020096
ENSBTAT00000001747
ENSBTAT00000029373
ENSBTAT00000063873
ENSBTAT00000056450
ENSBTAT00000014293
ENSBTAT00000053027
ENSBTAT00000020625
ENSBTAT00000011091
ENSBTAT00000007835
ENSBTAT00000022804
ENSBTAT00000055861
ENSBTAT00000012262
ENSBTAT00000003401
ENSBTAT00000025129
ENSBTAT00000020149
ENSBTAT00000020261
ENSBTAT00000006967
ENSBTAT00000021793
ENSBTAT00000043199
ENSBTAT00000063696
ENSBTAT00000020300
ENSBTAT00000052007

ENSBTAT00000000230
ENSBTAT00000052754
ENSBTAT00000012028
ENSBTAT00000045235
ENSBTAT00000008960
ENSBTAT00000056261
ENSBTAT00000045031
ENSBTAT00000012385
ENSBTAT00000064102
ENSBTAT00000023657
ENSBTAT00000021620
ENSBTAT00000006572
ENSBTAT00000008373
ENSBTAT00000054481
ENSBTAT00000053008
ENSBTAT00000043770
ENSBTAT00000016420
ENSBTAT00000015396
ENSBTAT00000023090
ENSBTAT00000029679
ENSBTAT00000031396
ENSBTAT00000013415
ENSBTAT00000028703
ENSBTAT00000002126
ENSBTAT00000033179
ENSBTAT00000055467
ENSBTAT00000044998
ENSBTAT00000057448
ENSBTAT00000004666
ENSBTAT00000019414

ENSBTAT00000038473
ENSBTAT00000002553
ENSBTAT00000061494
ENSBTAT00000013142
ENSBTAT00000026919
ENSBTAT00000027335
ENSBTAT00000023720
ENSBTAT00000014019
ENSBTAT00000007563
ENSBTAT00000011468
ENSBTAT00000003484
ENSBTAT00000006716
ENSBTAT00000039673
ENSBTAT00000053858
ENSBTAT00000020078
ENSBTAT00000026686
ENSBTAT00000008771
ENSBTAT00000029049
ENSBTAT00000056882
ENSBTAT00000049054
ENSBTAT00000006896
ENSBTAT00000052235
ENSBTAT00000019855
ENSBTAT00000024157
ENSBTAT00000008143
ENSBTAT00000004658
ENSBTAT00000036968
ENSBTAT00000000596
ENSBTAT00000018897
ENSBTAT00000028534

ENSBTAT00000015125
ENSBTAT00000021532
ENSBTAT00000028542
ENSBTAT00000026133
ENSBTAT00000020079
ENSBTAT00000025877
ENSBTAT00000008335
ENSBTAT00000020097
ENSBTAT00000043205
ENSBTAT00000027138
ENSBTAT00000042784
ENSBTAT00000014847
ENSBTAT00000019807
ENSBTAT00000001455
ENSBTAT00000052497
ENSBTAT00000021199
ENSBTAT00000025496
ENSBTAT00000001689
ENSBTAT00000013963
ENSBTAT00000046279
ENSBTAT00000034650
ENSBTAT00000006434
ENSBTAT00000064814
ENSBTAT00000064746
ENSBTAT00000063171
ENSBTAT00000002789
ENSBTAT00000002605
ENSBTAT00000011104
ENSBTAT00000013493
ENSBTAT00000063948

ENSBTAT00000032377
ENSBTAT00000002166
ENSBTAT00000053869
ENSBTAT00000053270
ENSBTAT00000063204
ENSBTAT00000052106
ENSBTAT00000006782
ENSBTAT00000055990
ENSBTAT00000030356
ENSBTAT00000019285
ENSBTAT00000006659
ENSBTAT00000025252
ENSBTAT00000007528
ENSBTAT00000045686
ENSBTAT00000003683
ENSBTAT00000045270
ENSBTAT00000013685
ENSBTAT00000063384
ENSBTAT00000063928
ENSBTAT00000054281
ENSBTAT00000063232
ENSBTAT00000027549
ENSBTAT00000015004
ENSBTAT00000012382
ENSBTAT00000018774
ENSBTAT00000015583
ENSBTAT00000063898
ENSBTAT00000021381
ENSBTAT00000004882
ENSBTAT00000053634

ENSBTAT00000002084
ENSBTAT00000023301
ENSBTAT00000019142
ENSBTAT00000016139
ENSBTAT00000023393
ENSBTAT00000034186
ENSBTAT00000034509
ENSBTAT00000019429
ENSBTAT00000019304
ENSBTAT00000007918
ENSBTAT00000013956
ENSBTAT00000031819
ENSBTAT00000008246
ENSBTAT00000065750
ENSBTAT00000020255
ENSBTAT00000018469
ENSBTAT00000010159
ENSBTAT00000017478
ENSBTAT00000064499
ENSBTAT00000004051
ENSBTAT00000011347
ENSBTAT00000023399
ENSBTAT00000027912
ENSBTAT00000003187
ENSBTAT00000020102
ENSBTAT00000009728
ENSBTAT00000011684
ENSBTAT00000037598
ENSBTAT00000000690
ENSBTAT00000024658

ENSBTAT00000009974
ENSBTAT00000056291
ENSBTAT00000039656
ENSBTAT00000046117
ENSBTAT00000011393
ENSBTAT00000018071
ENSBTAT00000047650
ENSBTAT00000038453
ENSBTAT00000057061
ENSBTAT00000008492
ENSBTAT00000018096
ENSBTAT00000002066
ENSBTAT00000064958
ENSBTAT00000008384
ENSBTAT00000063965
ENSBTAT00000044793
ENSBTAT00000038044
ENSBTAT00000015470
ENSBTAT00000003144
ENSBTAT00000024134
ENSBTAT00000015131
ENSBTAT00000054443
ENSBTAT00000053200
ENSBTAT00000032752
ENSBTAT00000023517
ENSBTAT00000013646
ENSBTAT00000022819
ENSBTAT00000014060
ENSBTAT00000047554
ENSBTAT00000054687

ENSBTAT00000054608
ENSBTAT00000025636
ENSBTAT00000024481
ENSBTAT00000012175
ENSBTAT00000053849
ENSBTAT00000009011
ENSBTAT00000039717
ENSBTAT00000001259
ENSBTAT00000057146
ENSBTAT00000002182
ENSBTAT00000057128
ENSBTAT00000010530
ENSBTAT00000007709
ENSBTAT00000018066
ENSBTAT00000044849
ENSBTAT00000021826
ENSBTAT00000027705
ENSBTAT00000005978
ENSBTAT00000024053
ENSBTAT00000020655
ENSBTAT00000035893
ENSBTAT00000005667
ENSBTAT00000061127
ENSBTAT00000004899
ENSBTAT00000024779
ENSBTAT00000000160
ENSBTAT00000065859
ENSBTAT00000049824
ENSBTAT00000064656
ENSBTAT00000007681

ENSBTAT00000045958
ENSBTAT00000056618
ENSBTAT00000033644
ENSBTAT00000025497
ENSBTAT00000012670
ENSBTAT00000053751
ENSBTAT00000003653
ENSBTAT00000029207
ENSBTAT00000004896
ENSBTAT00000049486
ENSBTAT00000017897
ENSBTAT00000048017
ENSBTAT00000022785
ENSBTAT00000055067
ENSBTAT00000001040
ENSBTAT00000065897
ENSBTAT00000027331
ENSBTAT00000052796
ENSBTAT00000013287
ENSBTAT00000065327
ENSBTAT00000045121
ENSBTAT00000021092
ENSBTAT00000052198
ENSBTAT00000028770
ENSBTAT00000055085
ENSBTAT00000014724
ENSBTAT00000061538
ENSBTAT00000002938
ENSBTAT00000014925
ENSBTAT00000002419

ENSBTAT00000000321
ENSBTAT00000028827
ENSBTAT00000010457
ENSBTAT00000056524
ENSBTAT00000009462
ENSBTAT00000054562
ENSBTAT00000055310
ENSBTAT00000002703
ENSBTAT00000065073
ENSBTAT00000038847
ENSBTAT00000022890
ENSBTAT00000018618
ENSBTAT00000044005
ENSBTAT00000021330
ENSBTAT00000026335
ENSBTAT00000034714
ENSBTAT00000046618
ENSBTAT00000064900
ENSBTAT00000064414
ENSBTAT00000049113
ENSBTAT00000055370
ENSBTAT00000014015
ENSBTAT00000027750
ENSBTAT00000049122
ENSBTAT00000002235
ENSBTAT00000018757
ENSBTAT00000023240
ENSBTAT00000008192
ENSBTAT00000016263
ENSBTAT00000019910

ENSBTAT00000000829
ENSBTAT00000022779
ENSBTAT00000011824
ENSBTAT00000013472
ENSBTAT00000048867
ENSBTAT00000037914
ENSBTAT00000066053
ENSBTAT00000025606
ENSBTAT00000063570
ENSBTAT00000003660
ENSBTAT00000012669
ENSBTAT00000032931
ENSBTAT00000024611
ENSBTAT00000064123
ENSBTAT00000007220
ENSBTAT00000065031
ENSBTAT00000065008
ENSBTAT00000007466
ENSBTAT00000064586
ENSBTAT00000048966
ENSBTAT00000012474
ENSBTAT00000031067
ENSBTAT00000057465
ENSBTAT00000013161
ENSBTAT00000004922
ENSBTAT00000064139
ENSBTAT00000035454
ENSBTAT00000027668
ENSBTAT00000057496
ENSBTAT00000052204

ENSBTAT00000005468
ENSBTAT00000052563
ENSBTAT00000065520
ENSBTAT00000003107
ENSBTAT00000013953
ENSBTAT00000035442
ENSBTAT00000026055
ENSBTAT00000049465
ENSBTAT00000057443
ENSBTAT00000035017
ENSBTAT00000013651
ENSBTAT00000056710
ENSBTAT00000015799
ENSBTAT00000006956
ENSBTAT00000013705
ENSBTAT00000052567
ENSBTAT00000064918
ENSBTAT00000002548
ENSBTAT00000066077
ENSBTAT00000065611
ENSBTAT00000020506
ENSBTAT00000002806
ENSBTAT00000006247
ENSBTAT00000011980
ENSBTAT00000001303
ENSBTAT00000039155
ENSBTAT00000008858
ENSBTAT00000027233
ENSBTAT00000022660
ENSBTAT00000015116

ENSBTAT00000054836
ENSBTAT00000056561
ENSBTAT00000052103
ENSBTAT00000047639
ENSBTAT00000028707
ENSBTAT00000064976
ENSBTAT00000019467
ENSBTAT00000038666
ENSBTAT00000001160
ENSBTAT00000005354
ENSBTAT00000064781
ENSBTAT00000050504
ENSBTAT00000028775
ENSBTAT00000013588
ENSBTAT00000002223
ENSBTAT00000000316
ENSBTAT00000066032
ENSBTAT00000013021
ENSBTAT00000007767
ENSBTAT00000020621
ENSBTAT00000034888
ENSBTAT00000063516
ENSBTAT00000064897
ENSBTAT00000027797
ENSBTAT00000009545
ENSBTAT00000052404
ENSBTAT00000011923
ENSBTAT00000018076
ENSBTAT00000003617
ENSBTAT00000065360

ENSBTAT00000056770
ENSBTAT00000025421
ENSBTAT00000026051
ENSBTAT00000056350
ENSBTAT00000066268
ENSBTAT00000022798
ENSBTAT00000064857
ENSBTAT00000030301
ENSBTAT00000061410
ENSBTAT00000008622
ENSBTAT00000043613
ENSBTAT00000004557
ENSBTAT00000004727
ENSBTAT00000002747
ENSBTAT00000018984
ENSBTAT00000011915
ENSBTAT00000016162
ENSBTAT00000009371
ENSBTAT00000023798
ENSBTAT00000064730
ENSBTAT00000002523
ENSBTAT00000048060
ENSBTAT00000043922
ENSBTAT00000016138
ENSBTAT00000007874
ENSBTAT00000055822
ENSBTAT00000039197
ENSBTAT00000018159
ENSBTAT00000023094
ENSBTAT00000002338

ENSBTAT00000052364
ENSBTAT00000064757
ENSBTAT00000021840
ENSBTAT00000001667
ENSBTAT00000025303
ENSBTAT00000013826
ENSBTAT00000014390
ENSBTAT00000065689
ENSBTAT00000052179
ENSBTAT00000045260
ENSBTAT00000044246
ENSBTAT00000002533
ENSBTAT00000006740
ENSBTAT00000033438
ENSBTAT00000054971
ENSBTAT00000065879
ENSBTAT00000052515
ENSBTAT00000004892
ENSBTAT00000063098
ENSBTAT00000053288
ENSBTAT00000002647
ENSBTAT00000009998
ENSBTAT00000001478
ENSBTAT00000005670
ENSBTAT00000064619
ENSBTAT00000052815
ENSBTAT00000023466
ENSBTAT00000007503
ENSBTAT00000011585
ENSBTAT00000039787

ENSBTAT00000064289
ENSBTAT00000016387
ENSBTAT00000000375
ENSBTAT00000016839
ENSBTAT00000022534
ENSBTAT00000034514
ENSBTAT00000042892
ENSBTAT00000005782
ENSBTAT00000064263
ENSBTAT00000007075
ENSBTAT00000013570
ENSBTAT00000009355
ENSBTAT00000017847
ENSBTAT00000013340
ENSBTAT00000056247
ENSBTAT00000018146
ENSBTAT00000063061
ENSBTAT00000019262
ENSBTAT00000019593
ENSBTAT00000010857
ENSBTAT00000014010
ENSBTAT00000064779
ENSBTAT00000047622
ENSBTAT00000014647
ENSBTAT00000047359
ENSBTAT00000037774
ENSBTAT00000019403
ENSBTAT00000065695
ENSBTAT00000006974
ENSBTAT00000049158

ENSBTAT00000029156
ENSBTAT00000035638
ENSBTAT00000064898
ENSBTAT00000056373
ENSBTAT00000027004
ENSBTAT00000064359
ENSBTAT00000054942
ENSBTAT00000008882
ENSBTAT00000026503
ENSBTAT00000025123
ENSBTAT00000064390
ENSBTAT00000062946
ENSBTAT00000054517
ENSBTAT00000050636
ENSBTAT00000055024
ENSBTAT00000060571
ENSBTAT00000055997
ENSBTAT00000026618
ENSBTAT00000002359
ENSBTAT00000027863
ENSBTAT00000053276
ENSBTAT00000028680
ENSBTAT00000054084
ENSBTAT00000029246
ENSBTAT00000017292
ENSBTAT00000038516
ENSBTAT00000046336
ENSBTAT00000053356
ENSBTAT00000033962
ENSBTAT00000055642

ENSBTAT00000008672
ENSBTAT00000027794
ENSBTAT00000020878
ENSBTAT00000028967
ENSBTAT00000049554
ENSBTAT00000053949
ENSBTAT00000054879
ENSBTAT00000022687
ENSBTAT00000017336
ENSBTAT00000056094
ENSBTAT00000032523
ENSBTAT00000064500
ENSBTAT00000009611
ENSBTAT00000054068
ENSBTAT00000031158
ENSBTAT00000038612
ENSBTAT00000055663
ENSBTAT00000064789
ENSBTAT00000064763
ENSBTAT00000064828
ENSBTAT00000063583
ENSBTAT00000024649
ENSBTAT00000019026
ENSBTAT00000049479
ENSBTAT00000027948
ENSBTAT00000019795
ENSBTAT00000002161
ENSBTAT00000054996
ENSBTAT00000020362
ENSBTAT00000023645

ENSBTAT00000021383
ENSBTAT00000001225
ENSBTAT00000052609
ENSBTAT00000028523
ENSBTAT00000025760
ENSBTAT00000023694
ENSBTAT00000063717
ENSBTAT00000064471
ENSBTAT00000004553
ENSBTAT00000037995
ENSBTAT00000021864
ENSBTAT00000053736
ENSBTAT00000026765
ENSBTAT00000004673
ENSBTAT00000028766
ENSBTAT00000028164
ENSBTAT00000025762
ENSBTAT00000063904
ENSBTAT00000056927
ENSBTAT00000005693
ENSBTAT00000065158
ENSBTAT00000057015
ENSBTAT00000035247
ENSBTAT00000008851
ENSBTAT00000047594
ENSBTAT00000034803
ENSBTAT00000055132
ENSBTAT00000016277
ENSBTAT00000012721
ENSBTAT00000003781

ENSBTAT00000064782
ENSBTAT00000038641
ENSBTAT00000054789
ENSBTAT00000063027
ENSBTAT00000000186
ENSBTAT00000019438
ENSBTAT00000052252
ENSBTAT00000055191
ENSBTAT00000056791
ENSBTAT00000056611
ENSBTAT00000022082
ENSBTAT00000053718
ENSBTAT00000018387
ENSBTAT00000001386
ENSBTAT00000056744
ENSBTAT00000064135
ENSBTAT00000000457
ENSBTAT00000003013
ENSBTAT00000010834
ENSBTAT00000020424
ENSBTAT00000028787
ENSBTAT00000026261
ENSBTAT00000057473
ENSBTAT00000037884
ENSBTAT00000000722
ENSBTAT00000016073
ENSBTAT00000019002
ENSBTAT00000065171
ENSBTAT00000050775
ENSBTAT00000004559

ENSBTAT00000021303
ENSBTAT00000012084
ENSBTAT00000014624
ENSBTAT00000052814
ENSBTAT00000038616
ENSBTAT00000044427
ENSBTAT00000005101
ENSBTAT00000009385
ENSBTAT00000039389
ENSBTAT00000003359
ENSBTAT00000065047
ENSBTAT00000045682
ENSBTAT00000014782
ENSBTAT00000008763
ENSBTAT00000037267
ENSBTAT00000029311
ENSBTAT00000009970
ENSBTAT00000043921
ENSBTAT00000021649
ENSBTAT00000023886
ENSBTAT00000022971
ENSBTAT00000029059
ENSBTAT00000001184
ENSBTAT00000015674
ENSBTAT00000020056
ENSBTAT00000063522
ENSBTAT00000012496
ENSBTAT00000018866
ENSBTAT00000025927
ENSBTAT00000035274

ENSBTAT00000051963
ENSBTAT00000001565
ENSBTAT00000051996
ENSBTAT00000039259
ENSBTAT00000037320
ENSBTAT00000020263
ENSBTAT00000054029
ENSBTAT00000036333
ENSBTAT00000000389
ENSBTAT00000015469
ENSBTAT00000052148
ENSBTAT00000057086
ENSBTAT00000052670
ENSBTAT00000055325
ENSBTAT00000054275
ENSBTAT00000063319
ENSBTAT00000021095
ENSBTAT00000011263
ENSBTAT00000064905
ENSBTAT00000020412
ENSBTAT00000065098
ENSBTAT00000016791
ENSBTAT00000055146
ENSBTAT00000007076
ENSBTAT00000064389
ENSBTAT00000018305
ENSBTAT00000001886
ENSBTAT00000004734
ENSBTAT00000019687
ENSBTAT00000055087

ENSBTAT00000052741
ENSBTAT00000055245
ENSBTAT00000016751
ENSBTAT00000002854
ENSBTAT00000013855
ENSBTAT00000026288
ENSBTAT00000017854
ENSBTAT00000029153
ENSBTAT00000054036
ENSBTAT00000010061
ENSBTAT00000011282
ENSBTAT00000000246
ENSBTAT00000004887
ENSBTAT00000029103
ENSBTAT00000052768
ENSBTAT00000054523
ENSBTAT00000047455
ENSBTAT00000006188
ENSBTAT00000061099
ENSBTAT00000005398
ENSBTAT00000063132
ENSBTAT00000026902
ENSBTAT00000053675
ENSBTAT00000053753
ENSBTAT00000029514
ENSBTAT00000027942
ENSBTAT00000004656
ENSBTAT00000021097
ENSBTAT00000012108
ENSBTAT00000063012

ENSBTAT00000056783
ENSBTAT00000024202
ENSBTAT00000045301
ENSBTAT00000014699
ENSBTAT00000025456
ENSBTAT00000001351
ENSBTAT00000030292
ENSBTAT00000032178
ENSBTAT00000022454
ENSBTAT00000056352
ENSBTAT00000004165
ENSBTAT00000064270
ENSBTAT00000054433
ENSBTAT00000013158
ENSBTAT00000064303
ENSBTAT00000066101
ENSBTAT00000005113
ENSBTAT00000032158
ENSBTAT00000032332
ENSBTAT00000000718
ENSBTAT00000063496
ENSBTAT00000029432
ENSBTAT00000065383
ENSBTAT00000019876
ENSBTAT00000064530
ENSBTAT00000003785
ENSBTAT00000045306
ENSBTAT00000014448
ENSBTAT00000053995
ENSBTAT00000036014

ENSBTAT00000028055
ENSBTAT00000007781
ENSBTAT00000022058
ENSBTAT00000003695
ENSBTAT00000054790
ENSBTAT00000049506
ENSBTAT00000063892
ENSBTAT00000047548
ENSBTAT00000003331
ENSBTAT00000028235
ENSBTAT00000036194
ENSBTAT00000052901
ENSBTAT00000015869
ENSBTAT00000032910
ENSBTAT00000055790
ENSBTAT00000052482
ENSBTAT00000052511
ENSBTAT00000003668
ENSBTAT00000001660
ENSBTAT00000051891
ENSBTAT00000057203
ENSBTAT00000053244
ENSBTAT00000036791
ENSBTAT00000023641
ENSBTAT00000054547
ENSBTAT00000013580
ENSBTAT00000003214
ENSBTAT00000048616
ENSBTAT00000022530
ENSBTAT00000049535

ENSBTAT00000031787
ENSBTAT00000023594
ENSBTAT00000054501
ENSBTAT00000001832
ENSBTAT00000014576
ENSBTAT00000004404
ENSBTAT00000001146
ENSBTAT00000009171
ENSBTAT00000050065
ENSBTAT00000049004
ENSBTAT00000008228
ENSBTAT00000065279
ENSBTAT00000053229
ENSBTAT00000000738
ENSBTAT00000030816
ENSBTAT00000064752
ENSBTAT00000052087
ENSBTAT00000031287
ENSBTAT00000003318
ENSBTAT00000056090
ENSBTAT00000053437
ENSBTAT00000001245
ENSBTAT00000020640
ENSBTAT00000007294
ENSBTAT00000046446
ENSBTAT00000022398
ENSBTAT00000052821
ENSBTAT00000003234
ENSBTAT00000049419
ENSBTAT00000002324

ENSBTAT00000037657
ENSBTAT00000038978
ENSBTAT00000053303
ENSBTAT00000020023
ENSBTAT00000011005
ENSBTAT00000030667
ENSBTAT00000020250
ENSBTAT00000004052
ENSBTAT00000025950
ENSBTAT00000043746
ENSBTAT00000011203
ENSBTAT00000000918
ENSBTAT00000011803
ENSBTAT00000021223
ENSBTAT00000006195
ENSBTAT00000064370
ENSBTAT00000031581
ENSBTAT00000055433
ENSBTAT00000000740
ENSBTAT00000016449
ENSBTAT00000029007
ENSBTAT00000020054
ENSBTAT00000009957
ENSBTAT00000001266
ENSBTAT00000009101
ENSBTAT00000064189
ENSBTAT00000028994
ENSBTAT00000052366
ENSBTAT00000053583
ENSBTAT00000022664

ENSBTAT00000028584
ENSBTAT00000027966
ENSBTAT00000004546
ENSBTAT00000065545
ENSBTAT00000056973
ENSBTAT00000029128
ENSBTAT00000064077
ENSBTAT00000020842
ENSBTAT00000014525
ENSBTAT00000003991
ENSBTAT00000064144
ENSBTAT00000025979
ENSBTAT00000024247
ENSBTAT00000018833
ENSBTAT00000035588
ENSBTAT00000015892
ENSBTAT00000008602
ENSBTAT00000056037
ENSBTAT00000023304
ENSBTAT00000064531
ENSBTAT00000044810
ENSBTAT00000033403
ENSBTAT00000065088
ENSBTAT00000037216
ENSBTAT00000054186
ENSBTAT00000053720
ENSBTAT00000021099
ENSBTAT00000043417
ENSBTAT00000063250
ENSBTAT00000017111

ENSBTAT00000026427
ENSBTAT00000001268
ENSBTAT00000027978
ENSBTAT00000066249
ENSBTAT00000055011
ENSBTAT00000017878
ENSBTAT00000046152
ENSBTAT00000013268
ENSBTAT00000064261
ENSBTAT00000064850
ENSBTAT00000054253
ENSBTAT00000021975
ENSBTAT00000065451
ENSBTAT00000064874
ENSBTAT00000002986
ENSBTAT00000024876
ENSBTAT00000007611
ENSBTAT00000047909
ENSBTAT00000054828
ENSBTAT00000007362
ENSBTAT00000054172
ENSBTAT00000053955
ENSBTAT00000032937
ENSBTAT00000048242
ENSBTAT00000015414
ENSBTAT00000014308
ENSBTAT00000010992
ENSBTAT00000008146
ENSBTAT00000060567
ENSBTAT00000064582

ENSBTAT00000003783
ENSBTAT00000044016
ENSBTAT00000020786
ENSBTAT00000055366
ENSBTAT00000021217
ENSBTAT00000025376
ENSBTAT00000006850
ENSBTAT00000003759
ENSBTAT00000053788
ENSBTAT00000008489
ENSBTAT00000011999
ENSBTAT00000008822
ENSBTAT00000016588
ENSBTAT00000049507
ENSBTAT00000064375
ENSBTAT00000006933
ENSBTAT00000045547
ENSBTAT00000002225
ENSBTAT00000020947
ENSBTAT00000007451
ENSBTAT00000018052
ENSBTAT00000023849
ENSBTAT00000025608
ENSBTAT00000008961
ENSBTAT00000065766
ENSBTAT00000066071
ENSBTAT00000065258
ENSBTAT00000030296
ENSBTAT00000020582
ENSBTAT00000012510

ENSBTAT00000035344
ENSBTAT00000014458
ENSBTAT00000064033
ENSBTAT00000053704
ENSBTAT00000053280
ENSBTAT00000030507
ENSBTAT00000030970
ENSBTAT00000027889
ENSBTAT00000011702
ENSBTAT00000065323
ENSBTAT00000056321
ENSBTAT00000009698
ENSBTAT00000000486
ENSBTAT00000030224
ENSBTAT00000043771
ENSBTAT00000001447
ENSBTAT00000052464
ENSBTAT00000017467
ENSBTAT00000006347
ENSBTAT00000017578
ENSBTAT00000003183
ENSBTAT00000018899
ENSBTAT00000044848
ENSBTAT00000013139
ENSBTAT00000019292
ENSBTAT00000024220
ENSBTAT00000037031
ENSBTAT00000053776
ENSBTAT00000003825
ENSBTAT00000001562

ENSBTAT00000036189
ENSBTAT00000028579
ENSBTAT00000031383
ENSBTAT00000003598
ENSBTAT00000005478
ENSBTAT00000028897
ENSBTAT00000004616
ENSBTAT00000025458
ENSBTAT00000025533
ENSBTAT00000066257
ENSBTAT00000020320
ENSBTAT00000035742
ENSBTAT00000028854
ENSBTAT00000008139
ENSBTAT00000016376
ENSBTAT00000025374
ENSBTAT00000005457
ENSBTAT00000056802
ENSBTAT00000064843
ENSBTAT00000002662
ENSBTAT00000024954
ENSBTAT00000043719
ENSBTAT00000000069
ENSBTAT00000064327
ENSBTAT00000027515
ENSBTAT00000044283
ENSBTAT00000013221
ENSBTAT00000012404
ENSBTAT00000001063
ENSBTAT00000065054

ENSBTAT00000006855
ENSBTAT00000048309
ENSBTAT00000002731
ENSBTAT00000005756
ENSBTAT00000022591
ENSBTAT00000035282
ENSBTAT00000055870
ENSBTAT00000065398
ENSBTAT00000056844
ENSBTAT00000003604
ENSBTAT00000009943
ENSBTAT00000046001
ENSBTAT00000007664
ENSBTAT00000018054
ENSBTAT00000056674
ENSBTAT00000022976
ENSBTAT00000015822
ENSBTAT00000064548
ENSBTAT00000006938
ENSBTAT00000033022
ENSBTAT00000009669
ENSBTAT00000012225
ENSBTAT00000056376
ENSBTAT00000037130
ENSBTAT00000014074
ENSBTAT00000034535
ENSBTAT00000011345
ENSBTAT00000032490
ENSBTAT00000030532
ENSBTAT00000063326

ENSBTAT00000064488
ENSBTAT00000001720
ENSBTAT00000007443
ENSBTAT00000003182
ENSBTAT00000063692
ENSBTAT00000031476
ENSBTAT00000064161
ENSBTAT00000034519
ENSBTAT00000057355
ENSBTAT00000016245
ENSBTAT00000008067
ENSBTAT00000032734
ENSBTAT00000010403
ENSBTAT00000010709
ENSBTAT00000015304
ENSBTAT00000054123
ENSBTAT00000063545
ENSBTAT00000055982
ENSBTAT00000014180
ENSBTAT00000024417
ENSBTAT00000043774
ENSBTAT00000033701
ENSBTAT00000026798
ENSBTAT00000011240
ENSBTAT00000003716
ENSBTAT00000040273
ENSBTAT00000054222
ENSBTAT00000031059
ENSBTAT00000039582
ENSBTAT00000061311

ENSBTAT00000010650
ENSBTAT00000008842
ENSBTAT00000033184
ENSBTAT00000003795
ENSBTAT00000033700
ENSBTAT00000023584
ENSBTAT00000052698
ENSBTAT00000027885
ENSBTAT00000043532
ENSBTAT00000018957
ENSBTAT00000017463
ENSBTAT00000024070
ENSBTAT00000014879
ENSBTAT00000018484
ENSBTAT00000052586
ENSBTAT00000056477
ENSBTAT00000064567
ENSBTAT00000005694
ENSBTAT00000027906
ENSBTAT00000025501
ENSBTAT00000053205
ENSBTAT00000055268
ENSBTAT00000054017
ENSBTAT00000015745
ENSBTAT00000036709
ENSBTAT00000010744
ENSBTAT00000015658
ENSBTAT00000010442
ENSBTAT00000024699
ENSBTAT00000055097

ENSBTAT00000003306
ENSBTAT00000052156
ENSBTAT00000025517
ENSBTAT00000003842
ENSBTAT00000046340
ENSBTAT00000064204
ENSBTAT00000001446
ENSBTAT00000052769
ENSBTAT00000008409
ENSBTAT00000006354
ENSBTAT00000006870
ENSBTAT00000003191
ENSBTAT00000004776
ENSBTAT00000057431
ENSBTAT00000013304
ENSBTAT00000004903
ENSBTAT00000023088
ENSBTAT00000017898
ENSBTAT00000027494
ENSBTAT00000065671
ENSBTAT00000040383
ENSBTAT00000001982
ENSBTAT00000008348
ENSBTAT00000007021
ENSBTAT00000009810
ENSBTAT00000064742
ENSBTAT00000031458
ENSBTAT00000000579
ENSBTAT00000012815
ENSBTAT00000017636

ENSBTAT00000001343
ENSBTAT00000001761
ENSBTAT00000010366
ENSBTAT00000042971
ENSBTAT00000063202
ENSBTAT00000021699
ENSBTAT00000056075
ENSBTAT00000012291
ENSBTAT00000007694
ENSBTAT00000001247
ENSBTAT00000030546
ENSBTAT00000001458
ENSBTAT00000054397
ENSBTAT00000010691
ENSBTAT00000023600
ENSBTAT00000013112
ENSBTAT00000004180
ENSBTAT00000053279
ENSBTAT00000056324
ENSBTAT00000031362
ENSBTAT00000021186
ENSBTAT00000027186
ENSBTAT00000017339
ENSBTAT00000064214
ENSBTAT00000034217
ENSBTAT00000055778
ENSBTAT00000024135
ENSBTAT00000045791
ENSBTAT00000024537
ENSBTAT00000050503

ENSBTAT00000017938
ENSBTAT00000025200
ENSBTAT00000014926
ENSBTAT00000022814
ENSBTAT00000003515
ENSBTAT00000004440
ENSBTAT00000017122
ENSBTAT00000057538
ENSBTAT00000026122
ENSBTAT00000063597
ENSBTAT00000023743
ENSBTAT00000011863
ENSBTAT00000024840
ENSBTAT00000008035
ENSBTAT00000053935
ENSBTAT00000017275
ENSBTAT00000039415
ENSBTAT00000025441
ENSBTAT00000038879
ENSBTAT00000017907
ENSBTAT00000045761
ENSBTAT00000007295
ENSBTAT00000063842
ENSBTAT00000053650
ENSBTAT00000011379
ENSBTAT00000001406
ENSBTAT00000044684
ENSBTAT00000027134
ENSBTAT00000061054
ENSBTAT00000039095

ENSBTAT00000008621
ENSBTAT00000027003
ENSBTAT00000034317
ENSBTAT00000047119
ENSBTAT00000015401
ENSBTAT00000065633
ENSBTAT00000019622
ENSBTAT00000032460
ENSBTAT00000065828
ENSBTAT00000062934
ENSBTAT00000012520
ENSBTAT00000020766
ENSBTAT00000005998
ENSBTAT00000044657
ENSBTAT00000011218
ENSBTAT00000026905
ENSBTAT00000046794
ENSBTAT00000011436
ENSBTAT00000052262
ENSBTAT00000011250
ENSBTAT00000017259
ENSBTAT00000005662
ENSBTAT00000025568
ENSBTAT00000027708
ENSBTAT00000000598
ENSBTAT00000039533
ENSBTAT00000014645
ENSBTAT00000009235
ENSBTAT00000007039
ENSBTAT00000048771

ENSBTAT00000055091
ENSBTAT00000052281
ENSBTAT00000014564
ENSBTAT00000003517
ENSBTAT00000004221
ENSBTAT00000025572
ENSBTAT00000004091
ENSBTAT00000064852
ENSBTAT00000024863
ENSBTAT00000030925
ENSBTAT00000021387
ENSBTAT00000034504
ENSBTAT00000043363
ENSBTAT00000036864
ENSBTAT00000004648
ENSBTAT00000052687
ENSBTAT00000027749
ENSBTAT00000000331
ENSBTAT00000028091
ENSBTAT00000027774
ENSBTAT00000065494
ENSBTAT00000024467
ENSBTAT00000025637
ENSBTAT00000014924
ENSBTAT00000021138
ENSBTAT00000001630
ENSBTAT00000042938
ENSBTAT00000043251
ENSBTAT00000002341
ENSBTAT00000014941

ENSBTAT00000004757
ENSBTAT00000025324
ENSBTAT00000055986
ENSBTAT00000031704
ENSBTAT00000015327
ENSBTAT00000005771
ENSBTAT00000030838
ENSBTAT00000040169
ENSBTAT00000063048
ENSBTAT00000046166
ENSBTAT00000029327
ENSBTAT00000032726
ENSBTAT00000001451
ENSBTAT00000038491
ENSBTAT00000011697
ENSBTAT00000026175
ENSBTAT00000006718
ENSBTAT00000046494
ENSBTAT00000019146
ENSBTAT00000055632
ENSBTAT00000033544
ENSBTAT00000004547
ENSBTAT00000045204
ENSBTAT00000029117
ENSBTAT00000011826
ENSBTAT00000026218
ENSBTAT00000014216
ENSBTAT00000055637
ENSBTAT00000045869
ENSBTAT00000002645

ENSBTAT00000030870
ENSBTAT00000017776
ENSBTAT00000005881
ENSBTAT00000015638
ENSBTAT00000014270
ENSBTAT00000054451
ENSBTAT00000003688
ENSBTAT00000047833
ENSBTAT00000024243
ENSBTAT00000024307
ENSBTAT00000031564
ENSBTAT00000014521
ENSBTAT00000004696
ENSBTAT00000053597
ENSBTAT00000065964
ENSBTAT00000048180
ENSBTAT00000022287
ENSBTAT00000017382
ENSBTAT00000063303
ENSBTAT00000063853
ENSBTAT00000000686
ENSBTAT00000001544
ENSBTAT00000013707
ENSBTAT00000020608
ENSBTAT00000018822
ENSBTAT00000033862
ENSBTAT00000017380
ENSBTAT00000022206
ENSBTAT00000033734
ENSBTAT00000037329

ENSBTAT00000024898
ENSBTAT00000051927
ENSBTAT00000019135
ENSBTAT00000003777
ENSBTAT00000064609
ENSBTAT00000026087
ENSBTAT00000024009
ENSBTAT00000006940
ENSBTAT00000010687
ENSBTAT00000063986
ENSBTAT00000037370
ENSBTAT00000055625
ENSBTAT00000054154
ENSBTAT00000021969
ENSBTAT00000027405
ENSBTAT00000011959
ENSBTAT00000001176
ENSBTAT00000027028
ENSBTAT00000020755
ENSBTAT00000049732
ENSBTAT00000006175
ENSBTAT00000047515
ENSBTAT00000020198
ENSBTAT00000025211
ENSBTAT00000003409
ENSBTAT00000065674
ENSBTAT00000003706
ENSBTAT00000024525
ENSBTAT00000024880
ENSBTAT00000006502

ENSBTAT00000018586
ENSBTAT00000020610
ENSBTAT00000024963
ENSBTAT00000056005
ENSBTAT00000011489
ENSBTAT00000034536
ENSBTAT00000045015
ENSBTAT00000017680
ENSBTAT00000026477
ENSBTAT00000064577
ENSBTAT00000014533
ENSBTAT00000013114
ENSBTAT00000019616
ENSBTAT00000016958
ENSBTAT00000019422
ENSBTAT00000063918
ENSBTAT00000000294
ENSBTAT00000038100
ENSBTAT00000033028
ENSBTAT00000019112
ENSBTAT00000033473
ENSBTAT00000001346
ENSBTAT00000060978
ENSBTAT00000056224
ENSBTAT00000012071
ENSBTAT00000029080
ENSBTAT00000042945
ENSBTAT00000025681
ENSBTAT00000005626
ENSBTAT00000053822

ENSBTAT00000026729
ENSBTAT00000030714
ENSBTAT00000022701
ENSBTAT00000027183
ENSBTAT00000026585
ENSBTAT00000020510
ENSBTAT00000017800
ENSBTAT00000001651
ENSBTAT00000020757
ENSBTAT00000009720
ENSBTAT00000028361
ENSBTAT00000065813
ENSBTAT00000001710
ENSBTAT00000025691
ENSBTAT00000000080
ENSBTAT00000052667
ENSBTAT00000065725
ENSBTAT00000043366
ENSBTAT00000046389
ENSBTAT00000029896
ENSBTAT00000026189
ENSBTAT00000020002
ENSBTAT00000002954
ENSBTAT00000019999
ENSBTAT00000022431
ENSBTAT00000010126
ENSBTAT00000045224
ENSBTAT00000004068
ENSBTAT00000013678
ENSBTAT00000035771

ENSBTAT00000056549
ENSBTAT00000046627
ENSBTAT00000027192
ENSBTAT00000024524
ENSBTAT00000001914
ENSBTAT00000039537
ENSBTAT00000009561
ENSBTAT00000036177
ENSBTAT00000024910
ENSBTAT00000008088
ENSBTAT00000015713
ENSBTAT00000028683
ENSBTAT00000025341
ENSBTAT00000061226
ENSBTAT00000046644
ENSBTAT00000000709
ENSBTAT00000045150
ENSBTAT00000038788
ENSBTAT00000057456
ENSBTAT00000007260
ENSBTAT00000064044
ENSBTAT00000022774
ENSBTAT00000004371
ENSBTAT00000031134
ENSBTAT00000026739
ENSBTAT00000021647
ENSBTAT00000001191
ENSBTAT00000043789
ENSBTAT00000047799
ENSBTAT00000029965

ENSBTAT00000004289
ENSBTAT00000013807
ENSBTAT00000022914
ENSBTAT00000016994
ENSBTAT00000062997
ENSBTAT00000006649
ENSBTAT00000023485
ENSBTAT00000009384
ENSBTAT00000053102
ENSBTAT00000056512
ENSBTAT00000021725
ENSBTAT00000066076
ENSBTAT00000010165
ENSBTAT00000043711
ENSBTAT00000021074
ENSBTAT00000046112
ENSBTAT00000024821
ENSBTAT00000011514
ENSBTAT00000010652
ENSBTAT00000065996
ENSBTAT00000022833
ENSBTAT00000010679
ENSBTAT00000022778
ENSBTAT00000037243
ENSBTAT00000004298
ENSBTAT00000017637
ENSBTAT00000006508
ENSBTAT00000012322
ENSBTAT00000045390
ENSBTAT00000018384

ENSBTAT00000026444
ENSBTAT00000064436
ENSBTAT00000024595
ENSBTAT00000016767
ENSBTAT00000012701
ENSBTAT00000055472
ENSBTAT00000057486
ENSBTAT00000013370
ENSBTAT00000005757
ENSBTAT00000004988
ENSBTAT00000055123
ENSBTAT00000019534
ENSBTAT00000008944
ENSBTAT00000020682
ENSBTAT00000026745
ENSBTAT00000004955
ENSBTAT00000046701
ENSBTAT00000012057
ENSBTAT00000040300
ENSBTAT00000025321
ENSBTAT00000021310
ENSBTAT00000054320
ENSBTAT00000017893
ENSBTAT00000035414
ENSBTAT00000025619
ENSBTAT00000061545
ENSBTAT00000009678
ENSBTAT00000055657
ENSBTAT00000018830
ENSBTAT00000045767

ENSBTAT00000009115
ENSBTAT00000004901
ENSBTAT00000052038
ENSBTAT00000040101
ENSBTAT00000017943
ENSBTAT00000052928
ENSBTAT00000009711
ENSBTAT00000014275
ENSBTAT00000003086
ENSBTAT00000064437
ENSBTAT00000006815
ENSBTAT00000014809
ENSBTAT00000027516
ENSBTAT00000056398
ENSBTAT00000063126
ENSBTAT00000002787
ENSBTAT00000025095
ENSBTAT00000004838
ENSBTAT00000021301
ENSBTAT00000020381
ENSBTAT00000057347
ENSBTAT00000034103
ENSBTAT00000014953
ENSBTAT00000017537
ENSBTAT00000030163
ENSBTAT00000004230
ENSBTAT00000017524
ENSBTAT00000057533
ENSBTAT00000019001
ENSBTAT00000004373

ENSBTAT00000065336
ENSBTAT00000020163
ENSBTAT00000038884
ENSBTAT00000027834
ENSBTAT00000017085
ENSBTAT00000006843
ENSBTAT00000029284
ENSBTAT00000044574
ENSBTAT00000005408
ENSBTAT00000038128
ENSBTAT00000004602
ENSBTAT00000018858
ENSBTAT00000044801
ENSBTAT00000010484
ENSBTAT00000034995
ENSBTAT00000061587
ENSBTAT00000014802
ENSBTAT00000002805
ENSBTAT00000052449
ENSBTAT00000005165
ENSBTAT00000028319
ENSBTAT00000004200
ENSBTAT00000017140
ENSBTAT00000000631
ENSBTAT00000053693
ENSBTAT00000015392
ENSBTAT00000015905
ENSBTAT00000027443
ENSBTAT00000039029
ENSBTAT00000009060

ENSBTAT00000037080
ENSBTAT00000046521
ENSBTAT00000008144
ENSBTAT00000048374
ENSBTAT00000020742
ENSBTAT00000057384
ENSBTAT00000030463
ENSBTAT00000010711
ENSBTAT00000038690
ENSBTAT00000032389
ENSBTAT00000020351
ENSBTAT00000025680
ENSBTAT00000013990
ENSBTAT00000002065
ENSBTAT00000061481
ENSBTAT00000027899
ENSBTAT00000025275
ENSBTAT00000006917
ENSBTAT00000000431
ENSBTAT00000034200
ENSBTAT00000004210
ENSBTAT00000021288
ENSBTAT00000064288
ENSBTAT00000012770
ENSBTAT00000026575
ENSBTAT00000003960
ENSBTAT00000012808
ENSBTAT00000060975
ENSBTAT00000064946
ENSBTAT00000042733

ENSBTAT00000053687
ENSBTAT00000001580
ENSBTAT00000026699
ENSBTAT00000044994
ENSBTAT00000022817
ENSBTAT00000017865
ENSBTAT00000063105
ENSBTAT00000034879
ENSBTAT00000006400
ENSBTAT00000014805
ENSBTAT00000009668
ENSBTAT00000043078
ENSBTAT00000021140
ENSBTAT00000029142
ENSBTAT00000009487
ENSBTAT00000001417
ENSBTAT00000007471
ENSBTAT00000044548
ENSBTAT00000014702
ENSBTAT00000002823
ENSBTAT00000028697
ENSBTAT00000025696
ENSBTAT00000002352
ENSBTAT00000004114
ENSBTAT00000046335
ENSBTAT00000005324
ENSBTAT00000017596
ENSBTAT00000023275
ENSBTAT00000039972
ENSBTAT00000055149

ENSBTAT00000022459
ENSBTAT00000002767
ENSBTAT00000008129
ENSBTAT00000007226
ENSBTAT00000066065
ENSBTAT00000015548
ENSBTAT00000043709
ENSBTAT00000010818
ENSBTAT00000000357
ENSBTAT00000014310
ENSBTAT00000031559
ENSBTAT00000006983
ENSBTAT00000056728
ENSBTAT00000017427
ENSBTAT00000011757
ENSBTAT00000015277
ENSBTAT00000029977
ENSBTAT00000063768
ENSBTAT00000021758
ENSBTAT00000016890
ENSBTAT00000002800
ENSBTAT00000061381
ENSBTAT00000009364
ENSBTAT00000066105
ENSBTAT00000014989
ENSBTAT00000001883
ENSBTAT00000047170
ENSBTAT00000034572
ENSBTAT00000065397
ENSBTAT00000056635

ENSBTAT00000052674
ENSBTAT00000018398
ENSBTAT00000017182
ENSBTAT00000034119
ENSBTAT00000017827
ENSBTAT00000064559
ENSBTAT00000033093
ENSBTAT00000007523
ENSBTAT00000012311
ENSBTAT00000016664
ENSBTAT00000028751
ENSBTAT00000013714
ENSBTAT00000010103
ENSBTAT00000025580
ENSBTAT00000026719
ENSBTAT00000009483
ENSBTAT00000028765
ENSBTAT00000018581
ENSBTAT00000047351
ENSBTAT00000002566
ENSBTAT00000002483
ENSBTAT00000003605
ENSBTAT00000029508
ENSBTAT00000019629
ENSBTAT00000019353
ENSBTAT00000018591
ENSBTAT00000010872
ENSBTAT00000002922
ENSBTAT00000021396
ENSBTAT00000022809

ENSBTAT00000019995
ENSBTAT00000010965
ENSBTAT00000006577
ENSBTAT00000000919
ENSBTAT00000012785
ENSBTAT00000012345
ENSBTAT00000024373
ENSBTAT00000004800
ENSBTAT00000012239
ENSBTAT00000000923
ENSBTAT00000031780
ENSBTAT00000007967
ENSBTAT00000046328
ENSBTAT00000019521
ENSBTAT00000049292
ENSBTAT00000063463
ENSBTAT00000007661
ENSBTAT00000015862
ENSBTAT00000017090
ENSBTAT00000048333
ENSBTAT00000066229
ENSBTAT00000005061
ENSBTAT00000005784
ENSBTAT00000028079
ENSBTAT00000026619
ENSBTAT00000063430
ENSBTAT00000007228
ENSBTAT00000014549
ENSBTAT00000021306
ENSBTAT00000029320

ENSBTAT00000028266
ENSBTAT00000065061
ENSBTAT00000023165
ENSBTAT00000022192
ENSBTAT00000015897
ENSBTAT00000064237
ENSBTAT00000017043
ENSBTAT00000043362
ENSBTAT00000025809
ENSBTAT00000048660
ENSBTAT00000008669
ENSBTAT00000002962
ENSBTAT00000003426
ENSBTAT00000053131
ENSBTAT00000043951
ENSBTAT00000005975
ENSBTAT00000010627
ENSBTAT00000002887
ENSBTAT00000025316
ENSBTAT00000003380
ENSBTAT00000020385
ENSBTAT00000032386
ENSBTAT00000011445
ENSBTAT00000053516
ENSBTAT00000026372
ENSBTAT00000019853
ENSBTAT00000001658
ENSBTAT00000009870
ENSBTAT00000057299
ENSBTAT00000010220

ENSBTAT00000019342
ENSBTAT00000012908
ENSBTAT00000017264
ENSBTAT00000052638
ENSBTAT00000020986
ENSBTAT00000018947
ENSBTAT00000008830
ENSBTAT00000018816
ENSBTAT00000018042
ENSBTAT00000026596
ENSBTAT00000022912
ENSBTAT00000014357
ENSBTAT00000016938
ENSBTAT00000029895
ENSBTAT00000023587
ENSBTAT00000000017
ENSBTAT00000006533
ENSBTAT00000019196
ENSBTAT00000061609
ENSBTAT00000026641
ENSBTAT00000010415
ENSBTAT00000012002
ENSBTAT00000017658
ENSBTAT00000028427
ENSBTAT00000016667
ENSBTAT00000022002
ENSBTAT00000014650
ENSBTAT00000002618
ENSBTAT00000010846
ENSBTAT00000008078

ENSBTAT00000004087
ENSBTAT00000045265
ENSBTAT00000008253
ENSBTAT00000033876
ENSBTAT00000054776
ENSBTAT00000000201
ENSBTAT00000006968
ENSBTAT00000036314
ENSBTAT00000001193
ENSBTAT00000056449
ENSBTAT00000037202
ENSBTAT00000039224
ENSBTAT00000052708
ENSBTAT00000008509
ENSBTAT00000026322
ENSBTAT00000011020
ENSBTAT00000028623
ENSBTAT00000049352
ENSBTAT00000053914
ENSBTAT00000010066
ENSBTAT00000008884
ENSBTAT00000049639
ENSBTAT00000055387
ENSBTAT00000020359
ENSBTAT00000061142
ENSBTAT00000016017
ENSBTAT00000016421
ENSBTAT00000022111
ENSBTAT00000008957
ENSBTAT00000064594

ENSBTAT00000057256
ENSBTAT00000056453
ENSBTAT00000043110
ENSBTAT00000031148
ENSBTAT00000023676
ENSBTAT00000025921
ENSBTAT00000016519
ENSBTAT00000054050
ENSBTAT00000027076
ENSBTAT00000022455
ENSBTAT00000040031
ENSBTAT00000035753
ENSBTAT00000045287
ENSBTAT00000000639
ENSBTAT00000022950
ENSBTAT00000020403
ENSBTAT00000037574
ENSBTAT00000033684
ENSBTAT00000021579
ENSBTAT00000048665
ENSBTAT00000016680
ENSBTAT00000009791
ENSBTAT00000031518
ENSBTAT00000003873
ENSBTAT00000053463
ENSBTAT00000021852
ENSBTAT00000020226
ENSBTAT00000028452
ENSBTAT00000064561
ENSBTAT00000032175

ENSBTAT00000002973
ENSBTAT00000061230
ENSBTAT00000002111
ENSBTAT00000011977
ENSBTAT00000048506
ENSBTAT00000028759
ENSBTAT00000016276
ENSBTAT00000020951
ENSBTAT00000014193
ENSBTAT00000008722
ENSBTAT00000014350
ENSBTAT00000004806
ENSBTAT00000012168
ENSBTAT00000045398
ENSBTAT00000036722
ENSBTAT00000002361
ENSBTAT00000002010
ENSBTAT00000012048
ENSBTAT00000010703
ENSBTAT00000009521
ENSBTAT00000043726
ENSBTAT00000015633
ENSBTAT00000016857
ENSBTAT00000011464
ENSBTAT00000017167
ENSBTAT00000022719
ENSBTAT00000007935
ENSBTAT00000024287
ENSBTAT00000039965
ENSBTAT00000061524

ENSBTAT00000039686
ENSBTAT00000000628
ENSBTAT00000029286
ENSBTAT00000003850
ENSBTAT00000053054
ENSBTAT00000013500
ENSBTAT00000004182
ENSBTAT00000061426
ENSBTAT00000016310
ENSBTAT00000043708
ENSBTAT00000001995
ENSBTAT00000034302
ENSBTAT00000010138
ENSBTAT00000061241
ENSBTAT00000043111
ENSBTAT00000031806
ENSBTAT00000000033
ENSBTAT00000000074
ENSBTAT00000000086
ENSBTAT00000000087
ENSBTAT00000046747
ENSBTAT00000000112
ENSBTAT00000000115
ENSBTAT00000014522
ENSBTAT00000044762
ENSBTAT00000000123
ENSBTAT00000000127
ENSBTAT00000000129
ENSBTAT00000000139
ENSBTAT00000000163

ENSBTAT00000000176
ENSBTAT00000000179
ENSBTAT00000000188
ENSBTAT00000000191
ENSBTAT00000000196
ENSBTAT00000000214
ENSBTAT00000000240
ENSBTAT00000000284
ENSBTAT00000000289
ENSBTAT00000000304
ENSBTAT00000000305
ENSBTAT00000043661
ENSBTAT00000000337
ENSBTAT00000000339
ENSBTAT00000000349
ENSBTAT00000000351
ENSBTAT00000000356
ENSBTAT00000000370
ENSBTAT00000000378
ENSBTAT00000000398
ENSBTAT00000035645
ENSBTAT00000000449
ENSBTAT00000000453
ENSBTAT00000000467
ENSBTAT00000000490
ENSBTAT00000000492
ENSBTAT00000000500
ENSBTAT00000000499
ENSBTAT00000000506
ENSBTAT00000000507

ENSBTAT00000000513
ENSBTAT00000007153
ENSBTAT00000005580
ENSBTAT00000000546
ENSBTAT00000000558
ENSBTAT00000000561
ENSBTAT00000000611
ENSBTAT00000000615
ENSBTAT00000000662
ENSBTAT00000000669
ENSBTAT00000000700
ENSBTAT00000000705
ENSBTAT00000000721
ENSBTAT00000000732
ENSBTAT00000000730
ENSBTAT00000000735
ENSBTAT00000000739
ENSBTAT00000000779
ENSBTAT00000000790
ENSBTAT00000000804
ENSBTAT00000000816
ENSBTAT00000054406
ENSBTAT00000000841
ENSBTAT00000000847
ENSBTAT00000000857
ENSBTAT00000000902
ENSBTAT00000000946
ENSBTAT00000000962
ENSBTAT00000000968
ENSBTAT00000000969

ENSBTAT00000000977
ENSBTAT00000000986
ENSBTAT00000000989
ENSBTAT00000001021
ENSBTAT00000001037
ENSBTAT00000001061
ENSBTAT00000001065
ENSBTAT00000001068
ENSBTAT00000001069
ENSBTAT00000001086
ENSBTAT00000001087
ENSBTAT00000001094
ENSBTAT00000001098
ENSBTAT00000001108
ENSBTAT00000001135
ENSBTAT00000001141
ENSBTAT00000001166
ENSBTAT00000001168
ENSBTAT00000001170
ENSBTAT00000032610
ENSBTAT00000044807
ENSBTAT00000001202
ENSBTAT00000001208
ENSBTAT00000001223
ENSBTAT00000001253
ENSBTAT00000001254
ENSBTAT00000038931
ENSBTAT00000001262
ENSBTAT00000001269
ENSBTAT00000001280

ENSBTAT00000043653
ENSBTAT00000001300
ENSBTAT00000001313
ENSBTAT00000001312
ENSBTAT00000001320
ENSBTAT00000001332
ENSBTAT00000001337
ENSBTAT00000001379
ENSBTAT00000001385
ENSBTAT00000001408
ENSBTAT00000046952
ENSBTAT00000001452
ENSBTAT00000001453
ENSBTAT00000001464
ENSBTAT00000001465
ENSBTAT00000001470
ENSBTAT00000001494
ENSBTAT00000004493
ENSBTAT00000001522
ENSBTAT00000001533
ENSBTAT00000001538
ENSBTAT00000001560
ENSBTAT00000028229
ENSBTAT00000053281
ENSBTAT00000001615
ENSBTAT00000001639
ENSBTAT00000001648
ENSBTAT00000001699
ENSBTAT00000001718
ENSBTAT00000001730

ENSBTAT00000001770
ENSBTAT00000039626
ENSBTAT00000001777
ENSBTAT00000051973
ENSBTAT00000001791
ENSBTAT00000056287
ENSBTAT00000001799
ENSBTAT00000001801
ENSBTAT00000001812
ENSBTAT00000001824
ENSBTAT00000001854
ENSBTAT00000001857
ENSBTAT00000001863
ENSBTAT00000048930
ENSBTAT00000001866
ENSBTAT00000001872
ENSBTAT00000052705
ENSBTAT00000001895
ENSBTAT00000001896
ENSBTAT00000001899
ENSBTAT00000001908
ENSBTAT00000055411
ENSBTAT00000026794
ENSBTAT00000001933
ENSBTAT00000052383
ENSBTAT00000001965
ENSBTAT00000001978
ENSBTAT00000038842
ENSBTAT00000001987
ENSBTAT00000001990

ENSBTAT00000001998
ENSBTAT00000002020
ENSBTAT00000002051
ENSBTAT00000002107
ENSBTAT00000002115
ENSBTAT00000002142
ENSBTAT00000002188
ENSBTAT00000002213
ENSBTAT00000002199
ENSBTAT00000002224
ENSBTAT00000002237
ENSBTAT00000002271
ENSBTAT00000002279
ENSBTAT00000002294
ENSBTAT00000002297
ENSBTAT00000052793
ENSBTAT00000002335
ENSBTAT00000002345
ENSBTAT00000002357
ENSBTAT00000029527
ENSBTAT00000002364
ENSBTAT00000002374
ENSBTAT00000002386
ENSBTAT00000002400
ENSBTAT00000057319
ENSBTAT00000002422
ENSBTAT00000002427
ENSBTAT00000002447
ENSBTAT00000002453
ENSBTAT00000031803

ENSBTAT00000002471
ENSBTAT00000002492
ENSBTAT00000002495
ENSBTAT00000002500
ENSBTAT00000002505
ENSBTAT00000002506
ENSBTAT00000002513
ENSBTAT00000002522
ENSBTAT00000002544
ENSBTAT00000002563
ENSBTAT00000002574
ENSBTAT00000002594
ENSBTAT00000002595
ENSBTAT00000043636
ENSBTAT00000002651
ENSBTAT00000002671
ENSBTAT00000031167
ENSBTAT00000002696
ENSBTAT00000002701
ENSBTAT00000043764
ENSBTAT00000002725
ENSBTAT00000002738
ENSBTAT00000002742
ENSBTAT00000002771
ENSBTAT00000002774
ENSBTAT00000002778
ENSBTAT00000002786
ENSBTAT00000002827
ENSBTAT00000002828
ENSBTAT00000002834

ENSBTAT00000002839
ENSBTAT00000002843
ENSBTAT00000002913
ENSBTAT00000002933
ENSBTAT00000002946
ENSBTAT00000014774
ENSBTAT00000034881
ENSBTAT00000002985
ENSBTAT00000043753
ENSBTAT00000003010
ENSBTAT00000003026
ENSBTAT00000003039
ENSBTAT00000019132
ENSBTAT00000003073
ENSBTAT00000003082
ENSBTAT00000003109
ENSBTAT00000003130
ENSBTAT00000003145
ENSBTAT00000003149
ENSBTAT00000003168
ENSBTAT00000003177
ENSBTAT00000003176
ENSBTAT00000003195
ENSBTAT00000003197
ENSBTAT00000003200
ENSBTAT00000003201
ENSBTAT00000003203
ENSBTAT00000003211
ENSBTAT00000003210
ENSBTAT00000003218

ENSBTAT00000003221
ENSBTAT00000003225
ENSBTAT00000027778
ENSBTAT00000003245
ENSBTAT00000003250
ENSBTAT00000003257
ENSBTAT00000003269
ENSBTAT00000003273
ENSBTAT00000003276
ENSBTAT00000003285
ENSBTAT00000003309
ENSBTAT00000003312
ENSBTAT00000003315
ENSBTAT00000003321
ENSBTAT00000003330
ENSBTAT00000048362
ENSBTAT00000003375
ENSBTAT00000003379
ENSBTAT00000054280
ENSBTAT00000003385
ENSBTAT00000003394
ENSBTAT00000003402
ENSBTAT00000003415
ENSBTAT00000003411
ENSBTAT00000003419
ENSBTAT00000003420
ENSBTAT00000003429
ENSBTAT00000003463
ENSBTAT00000003465
ENSBTAT00000003464

ENSBTAT00000003506
ENSBTAT00000055751
ENSBTAT00000003529
ENSBTAT00000003544
ENSBTAT00000003553
ENSBTAT00000003555
ENSBTAT00000003560
ENSBTAT00000003564
ENSBTAT00000003593
ENSBTAT00000003613
ENSBTAT00000003636
ENSBTAT00000003637
ENSBTAT00000003703
ENSBTAT00000052423
ENSBTAT00000003763
ENSBTAT00000003762
ENSBTAT00000003771
ENSBTAT00000003789
ENSBTAT00000003791
ENSBTAT00000003804
ENSBTAT00000003810
ENSBTAT00000003817
ENSBTAT00000003829
ENSBTAT00000003843
ENSBTAT00000016862
ENSBTAT00000003884
ENSBTAT00000003905
ENSBTAT00000003912
ENSBTAT00000003927
ENSBTAT00000003930

ENSBTAT00000020273
ENSBTAT00000003959
ENSBTAT00000003996
ENSBTAT00000004009
ENSBTAT00000004018
ENSBTAT00000004029
ENSBTAT00000004037
ENSBTAT00000004044
ENSBTAT00000004047
ENSBTAT00000004049
ENSBTAT00000004055
ENSBTAT00000004073
ENSBTAT00000004075
ENSBTAT00000004098
ENSBTAT00000004106
ENSBTAT00000004128
ENSBTAT00000004152
ENSBTAT00000004154
ENSBTAT00000004179
ENSBTAT00000004188
ENSBTAT00000004191
ENSBTAT00000034938
ENSBTAT00000004199
ENSBTAT00000037742
ENSBTAT00000004222
ENSBTAT00000004227
ENSBTAT00000004229
ENSBTAT00000004257
ENSBTAT00000055228
ENSBTAT00000034421

ENSBTAT00000004279
ENSBTAT00000004291
ENSBTAT00000004350
ENSBTAT00000004362
ENSBTAT00000004370
ENSBTAT00000004385
ENSBTAT00000043239
ENSBTAT00000004393
ENSBTAT00000004395
ENSBTAT00000004526
ENSBTAT00000004541
ENSBTAT00000004552
ENSBTAT00000004550
ENSBTAT00000004555
ENSBTAT00000004562
ENSBTAT00000031486
ENSBTAT00000004597
ENSBTAT00000004611
ENSBTAT00000004615
ENSBTAT00000004621
ENSBTAT00000004630
ENSBTAT00000004632
ENSBTAT00000064701
ENSBTAT00000004651
ENSBTAT00000004660
ENSBTAT00000004668
ENSBTAT00000004729
ENSBTAT00000004737
ENSBTAT00000004751
ENSBTAT00000004760

ENSBTAT00000004769
ENSBTAT00000004771
ENSBTAT00000004808
ENSBTAT00000061152
ENSBTAT00000043796
ENSBTAT00000004834
ENSBTAT00000004858
ENSBTAT00000004861
ENSBTAT00000004894
ENSBTAT00000004897
ENSBTAT00000050672
ENSBTAT00000004942
ENSBTAT00000004994
ENSBTAT00000005022
ENSBTAT00000005032
ENSBTAT00000005055
ENSBTAT00000005064
ENSBTAT00000005090
ENSBTAT00000005080
ENSBTAT00000005108
ENSBTAT00000005111
ENSBTAT00000005119
ENSBTAT00000005120
ENSBTAT00000005135
ENSBTAT00000005143
ENSBTAT00000005146
ENSBTAT00000005160
ENSBTAT00000005180
ENSBTAT00000005175
ENSBTAT00000005162

ENSBTAT00000005168
ENSBTAT00000005204
ENSBTAT00000005207
ENSBTAT00000053426
ENSBTAT00000005289
ENSBTAT00000005294
ENSBTAT00000021001
ENSBTAT00000005303
ENSBTAT00000033090
ENSBTAT00000005323
ENSBTAT00000005333
ENSBTAT00000005346
ENSBTAT00000005347
ENSBTAT00000005368
ENSBTAT00000005375
ENSBTAT00000005380
ENSBTAT00000005382
ENSBTAT00000005411
ENSBTAT00000005424
ENSBTAT00000005429
ENSBTAT00000005437
ENSBTAT00000005445
ENSBTAT00000005462
ENSBTAT00000005487
ENSBTAT00000005494
ENSBTAT00000005496
ENSBTAT00000005508
ENSBTAT00000005510
ENSBTAT00000005521
ENSBTAT00000005523

ENSBTAT00000005524
ENSBTAT00000005542
ENSBTAT00000005582
ENSBTAT00000005591
ENSBTAT00000005590
ENSBTAT00000005596
ENSBTAT00000066106
ENSBTAT00000005621
ENSBTAT00000005651
ENSBTAT00000005655
ENSBTAT00000005659
ENSBTAT00000005668
ENSBTAT00000005688
ENSBTAT00000005725
ENSBTAT00000005708
ENSBTAT00000005745
ENSBTAT00000005750
ENSBTAT00000005765
ENSBTAT00000005770
ENSBTAT00000005773
ENSBTAT00000005778
ENSBTAT00000005780
ENSBTAT00000005785
ENSBTAT00000005787
ENSBTAT00000005788
ENSBTAT00000005791
ENSBTAT00000005796
ENSBTAT00000005807
ENSBTAT00000005814
ENSBTAT00000005825

ENSBTAT00000005845
ENSBTAT00000005865
ENSBTAT00000005869
ENSBTAT00000056064
ENSBTAT00000005927
ENSBTAT00000005929
ENSBTAT00000005939
ENSBTAT00000005943
ENSBTAT00000005950
ENSBTAT00000005959
ENSBTAT00000005968
ENSBTAT00000009353
ENSBTAT00000005996
ENSBTAT00000006000
ENSBTAT00000006002
ENSBTAT00000006007
ENSBTAT00000006011
ENSBTAT00000006015
ENSBTAT00000006027
ENSBTAT00000006029
ENSBTAT00000006033
ENSBTAT00000006038
ENSBTAT00000006040
ENSBTAT00000006075
ENSBTAT00000006106
ENSBTAT00000006113
ENSBTAT00000006120
ENSBTAT00000006124
ENSBTAT00000006158
ENSBTAT00000004319

ENSBTAT00000006187
ENSBTAT00000006189
ENSBTAT00000006228
ENSBTAT00000006235
ENSBTAT00000006255
ENSBTAT00000006266
ENSBTAT00000006274
ENSBTAT00000006279
ENSBTAT00000006283
ENSBTAT00000006285
ENSBTAT00000053975
ENSBTAT00000006298
ENSBTAT00000006309
ENSBTAT00000006329
ENSBTAT00000006331
ENSBTAT00000006363
ENSBTAT00000006372
ENSBTAT00000006382
ENSBTAT00000006433
ENSBTAT00000006450
ENSBTAT00000006455
ENSBTAT00000006474
ENSBTAT00000054007
ENSBTAT00000006480
ENSBTAT00000006486
ENSBTAT00000006494
ENSBTAT00000006503
ENSBTAT00000063964
ENSBTAT00000055262
ENSBTAT00000006568

ENSBTAT00000006575
ENSBTAT00000006592
ENSBTAT00000006628
ENSBTAT00000006645
ENSBTAT00000027503
ENSBTAT00000006656
ENSBTAT00000006658
ENSBTAT00000035070
ENSBTAT00000006662
ENSBTAT00000006666
ENSBTAT00000006675
ENSBTAT00000006686
ENSBTAT00000006724
ENSBTAT00000052902
ENSBTAT00000006779
ENSBTAT00000006780
ENSBTAT00000006781
ENSBTAT00000006786
ENSBTAT00000006794
ENSBTAT00000006795
ENSBTAT00000006791
ENSBTAT00000006810
ENSBTAT00000006833
ENSBTAT00000006816
ENSBTAT00000031166
ENSBTAT00000006828
ENSBTAT00000006841
ENSBTAT00000006853
ENSBTAT00000006854
ENSBTAT00000006872

ENSBTAT00000006874
ENSBTAT00000055552
ENSBTAT00000044731
ENSBTAT00000006897
ENSBTAT00000006899
ENSBTAT00000006922
ENSBTAT00000055600
ENSBTAT00000006931
ENSBTAT00000006979
ENSBTAT00000006982
ENSBTAT00000007006
ENSBTAT00000062974
ENSBTAT00000007014
ENSBTAT00000007022
ENSBTAT00000007027
ENSBTAT00000007037
ENSBTAT00000007090
ENSBTAT00000012088
ENSBTAT00000007096
ENSBTAT00000007100
ENSBTAT00000007115
ENSBTAT00000007124
ENSBTAT00000007126
ENSBTAT00000007149
ENSBTAT00000007155
ENSBTAT00000007164
ENSBTAT00000007165
ENSBTAT00000007168
ENSBTAT00000007170
ENSBTAT00000007195

ENSBTAT00000007209
ENSBTAT00000007217
ENSBTAT00000015661
ENSBTAT00000007229
ENSBTAT00000007235
ENSBTAT00000031950
ENSBTAT00000007265
ENSBTAT00000007266
ENSBTAT00000007271
ENSBTAT00000007283
ENSBTAT00000007292
ENSBTAT00000007307
ENSBTAT00000047844
ENSBTAT00000007322
ENSBTAT00000007326
ENSBTAT00000007327
ENSBTAT00000007335
ENSBTAT00000007340
ENSBTAT00000007349
ENSBTAT00000007354
ENSBTAT00000034249
ENSBTAT00000007361
ENSBTAT00000043678
ENSBTAT00000007391
ENSBTAT00000023775
ENSBTAT00000007405
ENSBTAT00000007421
ENSBTAT00000032212
ENSBTAT00000007429
ENSBTAT00000007431

ENSBTAT00000007448
ENSBTAT00000007473
ENSBTAT00000007480
ENSBTAT00000007502
ENSBTAT00000007505
ENSBTAT00000007517
ENSBTAT00000007522
ENSBTAT00000007525
ENSBTAT00000049039
ENSBTAT00000007547
ENSBTAT00000007564
ENSBTAT00000007570
ENSBTAT00000007606
ENSBTAT00000007615
ENSBTAT00000007623
ENSBTAT00000007663
ENSBTAT00000007665
ENSBTAT00000007669
ENSBTAT00000007680
ENSBTAT00000007687
ENSBTAT00000007708
ENSBTAT00000007721
ENSBTAT00000007736
ENSBTAT00000007745
ENSBTAT00000007761
ENSBTAT00000007772
ENSBTAT00000007799
ENSBTAT00000007813
ENSBTAT00000007814
ENSBTAT00000007831

ENSBTAT00000007840
ENSBTAT00000007856
ENSBTAT00000007857
ENSBTAT00000007860
ENSBTAT00000007876
ENSBTAT00000007912
ENSBTAT00000007913
ENSBTAT00000055769
ENSBTAT00000007925
ENSBTAT00000007934
ENSBTAT00000007942
ENSBTAT00000007945
ENSBTAT00000007955
ENSBTAT00000007965
ENSBTAT00000007972
ENSBTAT00000007986
ENSBTAT00000007990
ENSBTAT00000046003
ENSBTAT00000008000
ENSBTAT00000030588
ENSBTAT00000008022
ENSBTAT00000034999
ENSBTAT00000008059
ENSBTAT00000008070
ENSBTAT00000008093
ENSBTAT00000008097
ENSBTAT00000008098
ENSBTAT00000008100
ENSBTAT00000008122
ENSBTAT00000008130

ENSBTAT00000008136
ENSBTAT00000008160
ENSBTAT00000008180
ENSBTAT00000008204
ENSBTAT00000008216
ENSBTAT00000046009
ENSBTAT00000008222
ENSBTAT00000008224
ENSBTAT00000008260
ENSBTAT00000008261
ENSBTAT00000054038
ENSBTAT00000008272
ENSBTAT00000055812
ENSBTAT00000008315
ENSBTAT00000008321
ENSBTAT00000008319
ENSBTAT00000008322
ENSBTAT00000008329
ENSBTAT00000008347
ENSBTAT00000008362
ENSBTAT00000050590
ENSBTAT00000008382
ENSBTAT00000008389
ENSBTAT00000008388
ENSBTAT00000008390
ENSBTAT00000044885
ENSBTAT00000008410
ENSBTAT00000008421
ENSBTAT00000008425
ENSBTAT00000008427

ENSBTAT00000008432
ENSBTAT00000008439
ENSBTAT00000008447
ENSBTAT00000008450
ENSBTAT00000008453
ENSBTAT00000008467
ENSBTAT00000008487
ENSBTAT00000008502
ENSBTAT00000008503
ENSBTAT00000008514
ENSBTAT00000008541
ENSBTAT00000008569
ENSBTAT00000008576
ENSBTAT00000008575
ENSBTAT00000008588
ENSBTAT00000008614
ENSBTAT00000008615
ENSBTAT00000053224
ENSBTAT00000008631
ENSBTAT00000008634
ENSBTAT00000008635
ENSBTAT00000008640
ENSBTAT00000008670
ENSBTAT00000008666
ENSBTAT00000023880
ENSBTAT00000008726
ENSBTAT00000008734
ENSBTAT00000008733
ENSBTAT00000008737
ENSBTAT00000008739

ENSBTAT00000046172
ENSBTAT00000008743
ENSBTAT00000008744
ENSBTAT00000008831
ENSBTAT00000047621
ENSBTAT00000001128
ENSBTAT00000008843
ENSBTAT00000008861
ENSBTAT00000008883
ENSBTAT00000008893
ENSBTAT00000035519
ENSBTAT00000008903
ENSBTAT00000008914
ENSBTAT00000043064
ENSBTAT00000008958
ENSBTAT00000008984
ENSBTAT00000043330
ENSBTAT00000036838
ENSBTAT00000008994
ENSBTAT00000008998
ENSBTAT00000009010
ENSBTAT00000009085
ENSBTAT00000031814
ENSBTAT00000009152
ENSBTAT00000054525
ENSBTAT00000048744
ENSBTAT00000042589
ENSBTAT00000009173
ENSBTAT00000009178
ENSBTAT00000048431

ENSBTAT00000009214
ENSBTAT00000009223
ENSBTAT00000009243
ENSBTAT00000009263
ENSBTAT00000009269
ENSBTAT00000009286
ENSBTAT00000009303
ENSBTAT00000009312
ENSBTAT00000009338
ENSBTAT00000009362
ENSBTAT00000009389
ENSBTAT00000009396
ENSBTAT00000009404
ENSBTAT00000009443
ENSBTAT00000009444
ENSBTAT00000009461
ENSBTAT00000009463
ENSBTAT00000009466
ENSBTAT00000009474
ENSBTAT00000009510
ENSBTAT00000009513
ENSBTAT00000009522
ENSBTAT00000009523
ENSBTAT00000009537
ENSBTAT00000009568
ENSBTAT00000009573
ENSBTAT00000009581
ENSBTAT00000009599
ENSBTAT00000009600
ENSBTAT00000009601

ENSBTAT00000009621
ENSBTAT00000009630
ENSBTAT00000009633
ENSBTAT00000037579
ENSBTAT00000009641
ENSBTAT00000009643
ENSBTAT00000009651
ENSBTAT00000009652
ENSBTAT00000009654
ENSBTAT00000009665
ENSBTAT00000009666
ENSBTAT00000022499
ENSBTAT00000009670
ENSBTAT00000009673
ENSBTAT00000002174
ENSBTAT00000009684
ENSBTAT00000009693
ENSBTAT00000009702
ENSBTAT00000009709
ENSBTAT00000009712
ENSBTAT00000009714
ENSBTAT00000009784
ENSBTAT00000009815
ENSBTAT00000009827
ENSBTAT00000009842
ENSBTAT00000032517
ENSBTAT00000009891
ENSBTAT00000009893
ENSBTAT00000012653
ENSBTAT00000026623

ENSBTAT00000009930
ENSBTAT00000009958
ENSBTAT00000009961
ENSBTAT00000020908
ENSBTAT00000009990
ENSBTAT00000010002
ENSBTAT00000010006
ENSBTAT00000010015
ENSBTAT00000010022
ENSBTAT00000010035
ENSBTAT00000010060
ENSBTAT00000010069
ENSBTAT00000010078
ENSBTAT00000010101
ENSBTAT00000010114
ENSBTAT00000010116
ENSBTAT00000010118
ENSBTAT00000010119
ENSBTAT00000010127
ENSBTAT00000010153
ENSBTAT00000010163
ENSBTAT00000010164
ENSBTAT00000010176
ENSBTAT00000036534
ENSBTAT00000010178
ENSBTAT00000010202
ENSBTAT00000010236
ENSBTAT00000010242
ENSBTAT00000010273
ENSBTAT00000010279

ENSBTAT00000055203
ENSBTAT00000010313
ENSBTAT00000010324
ENSBTAT00000032121
ENSBTAT00000010325
ENSBTAT00000010350
ENSBTAT00000010363
ENSBTAT00000010367
ENSBTAT00000010386
ENSBTAT00000010387
ENSBTAT00000010410
ENSBTAT00000010408
ENSBTAT00000045589
ENSBTAT00000056914
ENSBTAT00000010431
ENSBTAT00000010436
ENSBTAT00000010458
ENSBTAT00000033361
ENSBTAT00000010513
ENSBTAT00000010514
ENSBTAT00000010516
ENSBTAT00000010531
ENSBTAT00000044459
ENSBTAT00000010554
ENSBTAT00000010555
ENSBTAT00000010632
ENSBTAT00000010633
ENSBTAT00000010649
ENSBTAT00000010685
ENSBTAT00000043035

ENSBTAT00000010704
ENSBTAT00000010705
ENSBTAT00000010719
ENSBTAT00000010721
ENSBTAT00000010727
ENSBTAT00000010737
ENSBTAT00000052375
ENSBTAT00000010780
ENSBTAT00000010781
ENSBTAT00000010790
ENSBTAT00000010804
ENSBTAT00000037485
ENSBTAT00000010838
ENSBTAT00000010845
ENSBTAT00000010891
ENSBTAT00000010911
ENSBTAT00000053127
ENSBTAT00000010954
ENSBTAT00000010964
ENSBTAT00000040027
ENSBTAT00000010968
ENSBTAT00000038048
ENSBTAT00000010982
ENSBTAT00000045090
ENSBTAT00000011009
ENSBTAT00000011018
ENSBTAT00000011016
ENSBTAT00000011035
ENSBTAT00000011042
ENSBTAT00000011050

ENSBTAT00000011063
ENSBTAT00000011086
ENSBTAT00000011095
ENSBTAT00000029827
ENSBTAT00000011165
ENSBTAT00000011185
ENSBTAT00000030060
ENSBTAT00000011219
ENSBTAT00000011230
ENSBTAT00000024416
ENSBTAT00000011246
ENSBTAT00000011253
ENSBTAT00000011255
ENSBTAT00000011257
ENSBTAT00000011278
ENSBTAT00000011312
ENSBTAT00000011320
ENSBTAT00000044066
ENSBTAT00000011339
ENSBTAT00000011383
ENSBTAT00000011385
ENSBTAT00000011391
ENSBTAT00000011396
ENSBTAT00000011420
ENSBTAT00000011458
ENSBTAT00000011478
ENSBTAT00000011484
ENSBTAT00000011491
ENSBTAT00000011496
ENSBTAT00000043072

ENSBTAT00000011527
ENSBTAT00000011530
ENSBTAT00000011543
ENSBTAT00000011546
ENSBTAT00000011567
ENSBTAT00000011575
ENSBTAT00000011581
ENSBTAT00000011600
ENSBTAT00000011633
ENSBTAT00000011663
ENSBTAT00000011665
ENSBTAT00000011670
ENSBTAT00000011681
ENSBTAT00000011709
ENSBTAT00000011711
ENSBTAT00000047025
ENSBTAT00000011716
ENSBTAT00000011728
ENSBTAT00000011732
ENSBTAT00000011776
ENSBTAT00000011798
ENSBTAT00000011851
ENSBTAT00000011864
ENSBTAT00000011881
ENSBTAT00000000590
ENSBTAT00000053009
ENSBTAT00000011960
ENSBTAT00000011976
ENSBTAT00000011979
ENSBTAT00000012000

ENSBTAT00000012008
ENSBTAT00000012010
ENSBTAT00000012027
ENSBTAT00000012044
ENSBTAT00000012047
ENSBTAT00000012064
ENSBTAT00000012083
ENSBTAT00000012090
ENSBTAT00000012098
ENSBTAT00000012120
ENSBTAT00000012128
ENSBTAT00000012136
ENSBTAT00000012138
ENSBTAT00000012151
ENSBTAT00000012159
ENSBTAT00000012170
ENSBTAT00000012183
ENSBTAT00000037763
ENSBTAT00000012190
ENSBTAT00000012211
ENSBTAT00000055168
ENSBTAT00000012219
ENSBTAT00000012224
ENSBTAT00000012243
ENSBTAT00000012256
ENSBTAT00000012259
ENSBTAT00000012261
ENSBTAT00000012301
ENSBTAT00000012324
ENSBTAT00000012331

ENSBTAT00000036235
ENSBTAT00000063765
ENSBTAT00000012342
ENSBTAT00000034943
ENSBTAT00000049172
ENSBTAT00000012394
ENSBTAT00000012411
ENSBTAT00000012430
ENSBTAT00000065896
ENSBTAT00000012465
ENSBTAT00000012475
ENSBTAT00000043278
ENSBTAT00000012485
ENSBTAT00000012499
ENSBTAT00000012512
ENSBTAT00000012515
ENSBTAT00000012538
ENSBTAT00000012540
ENSBTAT00000012546
ENSBTAT00000012548
ENSBTAT00000012550
ENSBTAT00000012574
ENSBTAT00000045880
ENSBTAT00000012599
ENSBTAT00000012632
ENSBTAT00000061523
ENSBTAT00000039210
ENSBTAT00000012705
ENSBTAT00000012738
ENSBTAT00000012762

ENSBTAT00000012782
ENSBTAT00000012783
ENSBTAT00000008806
ENSBTAT00000012835
ENSBTAT00000012834
ENSBTAT00000012836
ENSBTAT00000012858
ENSBTAT00000052182
ENSBTAT00000012893
ENSBTAT00000012939
ENSBTAT00000012942
ENSBTAT00000012950
ENSBTAT00000012963
ENSBTAT00000012983
ENSBTAT00000043680
ENSBTAT00000013035
ENSBTAT00000050340
ENSBTAT00000013069
ENSBTAT00000056162
ENSBTAT00000013086
ENSBTAT00000013127
ENSBTAT00000053405
ENSBTAT00000013133
ENSBTAT00000013141
ENSBTAT00000013144
ENSBTAT00000013145
ENSBTAT00000013152
ENSBTAT00000013153
ENSBTAT00000013171
ENSBTAT00000057082

ENSBTAT00000013202
ENSBTAT00000013209
ENSBTAT00000013213
ENSBTAT00000013235
ENSBTAT00000013262
ENSBTAT00000013281
ENSBTAT00000035265
ENSBTAT00000013310
ENSBTAT00000048596
ENSBTAT00000013354
ENSBTAT00000013386
ENSBTAT00000045455
ENSBTAT00000013402
ENSBTAT00000020978
ENSBTAT00000013427
ENSBTAT00000013429
ENSBTAT00000013431
ENSBTAT00000013496
ENSBTAT00000038767
ENSBTAT00000013507
ENSBTAT00000036478
ENSBTAT00000013537
ENSBTAT00000013538
ENSBTAT00000013539
ENSBTAT00000013568
ENSBTAT00000013571
ENSBTAT00000013574
ENSBTAT00000013618
ENSBTAT00000013602
ENSBTAT00000055244

ENSBTAT00000013675
ENSBTAT00000013699
ENSBTAT00000013712
ENSBTAT00000013718
ENSBTAT00000013728
ENSBTAT00000013736
ENSBTAT00000050296
ENSBTAT00000013784
ENSBTAT00000013787
ENSBTAT00000013791
ENSBTAT00000057235
ENSBTAT00000013832
ENSBTAT00000056282
ENSBTAT00000056863
ENSBTAT00000013871
ENSBTAT00000013873
ENSBTAT00000013885
ENSBTAT00000013921
ENSBTAT00000013922
ENSBTAT00000013929
ENSBTAT00000013931
ENSBTAT00000013937
ENSBTAT00000013944
ENSBTAT00000013947
ENSBTAT00000013959
ENSBTAT00000013986
ENSBTAT00000013988
ENSBTAT00000014064
ENSBTAT00000014115
ENSBTAT00000033972

ENSBTAT00000014122
ENSBTAT00000014128
ENSBTAT00000014127
ENSBTAT00000014143
ENSBTAT00000014141
ENSBTAT00000031361
ENSBTAT00000063175
ENSBTAT00000014198
ENSBTAT00000022382
ENSBTAT00000014202
ENSBTAT00000014208
ENSBTAT00000014213
ENSBTAT00000064630
ENSBTAT00000014219
ENSBTAT00000014256
ENSBTAT00000014258
ENSBTAT00000014277
ENSBTAT00000014278
ENSBTAT00000014279
ENSBTAT00000014297
ENSBTAT00000014299
ENSBTAT00000010823
ENSBTAT00000014324
ENSBTAT00000061123
ENSBTAT00000014354
ENSBTAT00000014368
ENSBTAT00000014397
ENSBTAT00000014398
ENSBTAT00000014445
ENSBTAT00000014446

ENSBTAT00000014449
ENSBTAT00000014452
ENSBTAT00000014453
ENSBTAT00000044628
ENSBTAT00000014462
ENSBTAT00000014473
ENSBTAT00000014475
ENSBTAT00000014489
ENSBTAT00000014511
ENSBTAT00000014517
ENSBTAT00000014519
ENSBTAT00000014527
ENSBTAT00000014528
ENSBTAT00000014529
ENSBTAT00000014537
ENSBTAT00000014548
ENSBTAT00000014552
ENSBTAT00000014559
ENSBTAT00000014562
ENSBTAT00000014565
ENSBTAT00000014575
ENSBTAT00000014579
ENSBTAT00000014585
ENSBTAT00000014592
ENSBTAT00000014629
ENSBTAT00000014665
ENSBTAT00000052700
ENSBTAT00000014685
ENSBTAT00000014710
ENSBTAT00000014730

ENSBTAT00000049276
ENSBTAT00000014755
ENSBTAT00000014759
ENSBTAT00000014771
ENSBTAT00000014768
ENSBTAT00000014789
ENSBTAT00000014784
ENSBTAT00000014785
ENSBTAT00000014791
ENSBTAT00000014795
ENSBTAT00000014792
ENSBTAT00000014794
ENSBTAT00000014800
ENSBTAT00000014801
ENSBTAT00000014822
ENSBTAT00000014829
ENSBTAT00000014860
ENSBTAT00000014872
ENSBTAT00000014878
ENSBTAT00000014888
ENSBTAT00000014893
ENSBTAT00000014922
ENSBTAT00000014946
ENSBTAT00000014966
ENSBTAT00000014968
ENSBTAT00000014972
ENSBTAT00000014975
ENSBTAT00000014983
ENSBTAT00000015008
ENSBTAT00000015024

ENSBTAT00000015084
ENSBTAT00000040289
ENSBTAT00000015103
ENSBTAT00000015107
ENSBTAT00000015147
ENSBTAT00000046141
ENSBTAT00000015165
ENSBTAT00000015176
ENSBTAT00000015208
ENSBTAT00000015228
ENSBTAT00000015237
ENSBTAT00000015268
ENSBTAT00000055192
ENSBTAT00000015317
ENSBTAT00000015324
ENSBTAT00000015330
ENSBTAT00000015353
ENSBTAT00000015362
ENSBTAT00000015375
ENSBTAT00000015378
ENSBTAT00000038024
ENSBTAT00000015416
ENSBTAT00000035535
ENSBTAT00000015438
ENSBTAT00000015448
ENSBTAT00000015450
ENSBTAT00000015456
ENSBTAT00000015485
ENSBTAT00000015490
ENSBTAT00000015495

ENSBTAT00000015528
ENSBTAT00000030824
ENSBTAT00000015544
ENSBTAT00000015564
ENSBTAT00000015567
ENSBTAT00000015577
ENSBTAT00000065469
ENSBTAT00000026433
ENSBTAT00000015618
ENSBTAT00000015622
ENSBTAT00000015620
ENSBTAT00000015642
ENSBTAT00000015680
ENSBTAT00000015688
ENSBTAT00000015700
ENSBTAT00000015704
ENSBTAT00000015733
ENSBTAT00000015750
ENSBTAT00000015755
ENSBTAT00000015760
ENSBTAT00000015757
ENSBTAT00000015780
ENSBTAT00000015794
ENSBTAT00000015804
ENSBTAT00000015807
ENSBTAT00000015846
ENSBTAT00000015866
ENSBTAT00000015874
ENSBTAT00000044397
ENSBTAT00000015904

ENSBTAT00000031441
ENSBTAT00000015939
ENSBTAT00000015947
ENSBTAT00000015955
ENSBTAT00000015956
ENSBTAT00000015973
ENSBTAT00000015987
ENSBTAT00000015985
ENSBTAT00000015994
ENSBTAT00000015990
ENSBTAT00000016016
ENSBTAT00000016020
ENSBTAT00000013106
ENSBTAT00000016029
ENSBTAT00000016034
ENSBTAT00000016066
ENSBTAT00000016095
ENSBTAT00000016109
ENSBTAT00000016111
ENSBTAT00000016133
ENSBTAT00000016141
ENSBTAT00000016142
ENSBTAT00000016153
ENSBTAT00000016157
ENSBTAT00000016163
ENSBTAT00000016167
ENSBTAT00000016179
ENSBTAT00000016212
ENSBTAT00000040234
ENSBTAT00000016290

ENSBTAT00000016320
ENSBTAT00000016348
ENSBTAT00000053323
ENSBTAT00000016351
ENSBTAT00000057062
ENSBTAT00000016378
ENSBTAT00000016413
ENSBTAT00000016415
ENSBTAT00000016418
ENSBTAT00000016423
ENSBTAT00000046344
ENSBTAT00000016454
ENSBTAT00000016464
ENSBTAT00000016471
ENSBTAT00000016475
ENSBTAT00000016482
ENSBTAT00000016488
ENSBTAT00000016506
ENSBTAT00000046721
ENSBTAT00000016532
ENSBTAT00000016535
ENSBTAT00000016548
ENSBTAT00000016549
ENSBTAT00000016564
ENSBTAT00000016569
ENSBTAT00000016590
ENSBTAT00000016610
ENSBTAT00000012076
ENSBTAT00000016634
ENSBTAT00000016638

ENSBTAT00000016640
ENSBTAT00000016651
ENSBTAT00000034415
ENSBTAT00000016673
ENSBTAT00000029946
ENSBTAT00000016712
ENSBTAT00000016714
ENSBTAT00000016736
ENSBTAT00000016742
ENSBTAT00000016755
ENSBTAT00000016756
ENSBTAT00000016757
ENSBTAT00000016759
ENSBTAT00000016771
ENSBTAT00000063314
ENSBTAT00000016774
ENSBTAT00000016776
ENSBTAT00000016780
ENSBTAT00000016785
ENSBTAT00000016796
ENSBTAT00000001414
ENSBTAT00000016871
ENSBTAT00000016879
ENSBTAT00000016878
ENSBTAT00000016887
ENSBTAT00000016889
ENSBTAT00000016891
ENSBTAT00000016906
ENSBTAT00000043151
ENSBTAT00000016918

ENSBTAT00000016929
ENSBTAT00000016930
ENSBTAT00000016951
ENSBTAT00000016960
ENSBTAT00000016962
ENSBTAT00000016984
ENSBTAT00000016991
ENSBTAT00000016993
ENSBTAT00000017006
ENSBTAT00000017010
ENSBTAT00000017022
ENSBTAT00000017035
ENSBTAT00000017042
ENSBTAT00000030419
ENSBTAT00000017050
ENSBTAT00000017066
ENSBTAT00000017109
ENSBTAT00000017114
ENSBTAT00000017120
ENSBTAT00000017126
ENSBTAT00000017129
ENSBTAT00000017134
ENSBTAT00000017141
ENSBTAT00000017146
ENSBTAT00000017150
ENSBTAT00000017158
ENSBTAT00000017163
ENSBTAT00000017164
ENSBTAT00000017165
ENSBTAT00000017178

ENSBTAT00000017187
ENSBTAT00000017203
ENSBTAT00000017619
ENSBTAT00000017209
ENSBTAT00000017232
ENSBTAT00000017243
ENSBTAT00000017244
ENSBTAT00000017289
ENSBTAT00000017305
ENSBTAT00000017310
ENSBTAT00000017319
ENSBTAT00000017320
ENSBTAT00000017324
ENSBTAT00000017330
ENSBTAT00000017332
ENSBTAT00000017347
ENSBTAT00000017360
ENSBTAT00000017362
ENSBTAT00000048654
ENSBTAT00000017376
ENSBTAT00000017387
ENSBTAT00000017405
ENSBTAT00000017423
ENSBTAT00000017442
ENSBTAT00000017445
ENSBTAT00000017457
ENSBTAT00000017458
ENSBTAT00000017461
ENSBTAT00000017476
ENSBTAT00000017525

ENSBTAT00000044700
ENSBTAT00000017564
ENSBTAT00000017574
ENSBTAT00000017586
ENSBTAT00000017590
ENSBTAT00000017603
ENSBTAT00000017615
ENSBTAT00000017620
ENSBTAT00000046662
ENSBTAT00000017653
ENSBTAT00000065404
ENSBTAT00000017655
ENSBTAT00000017710
ENSBTAT00000017716
ENSBTAT00000017721
ENSBTAT00000020050
ENSBTAT00000017755
ENSBTAT00000017757
ENSBTAT00000017770
ENSBTAT00000017775
ENSBTAT00000017781
ENSBTAT00000017825
ENSBTAT00000017835
ENSBTAT00000017856
ENSBTAT00000017857
ENSBTAT00000017860
ENSBTAT00000017884
ENSBTAT00000017899
ENSBTAT00000017926
ENSBTAT00000017963

ENSBTAT00000065358
ENSBTAT00000017991
ENSBTAT00000017996
ENSBTAT00000018029
ENSBTAT00000047911
ENSBTAT00000025240
ENSBTAT00000018079
ENSBTAT00000018083
ENSBTAT00000054391
ENSBTAT00000018116
ENSBTAT00000018123
ENSBTAT00000018130
ENSBTAT00000046406
ENSBTAT00000018162
ENSBTAT00000046128
ENSBTAT00000018185
ENSBTAT00000030772
ENSBTAT00000018202
ENSBTAT00000018224
ENSBTAT00000018234
ENSBTAT00000018250
ENSBTAT00000018249
ENSBTAT00000018269
ENSBTAT00000018278
ENSBTAT00000018281
ENSBTAT00000018322
ENSBTAT00000055273
ENSBTAT00000018321
ENSBTAT00000018339
ENSBTAT00000018395

ENSBTAT00000018402
ENSBTAT00000018406
ENSBTAT00000018411
ENSBTAT00000018414
ENSBTAT00000032764
ENSBTAT00000018453
ENSBTAT00000018455
ENSBTAT00000018493
ENSBTAT00000018491
ENSBTAT00000066322
ENSBTAT00000018541
ENSBTAT00000018578
ENSBTAT00000018592
ENSBTAT00000018595
ENSBTAT00000018597
ENSBTAT00000018610
ENSBTAT00000018632
ENSBTAT00000018621
ENSBTAT00000018637
ENSBTAT00000018634
ENSBTAT00000018631
ENSBTAT00000018647
ENSBTAT00000039347
ENSBTAT00000046847
ENSBTAT00000018679
ENSBTAT00000018687
ENSBTAT00000018697
ENSBTAT00000018730
ENSBTAT00000026200
ENSBTAT00000018735

ENSBTAT00000018740
ENSBTAT00000018754
ENSBTAT00000018766
ENSBTAT00000030276
ENSBTAT00000018777
ENSBTAT00000018778
ENSBTAT00000018803
ENSBTAT00000018807
ENSBTAT00000018811
ENSBTAT00000018862
ENSBTAT00000018877
ENSBTAT00000018901
ENSBTAT00000018903
ENSBTAT00000018906
ENSBTAT00000018918
ENSBTAT00000043345
ENSBTAT00000018920
ENSBTAT00000037702
ENSBTAT00000018946
ENSBTAT00000018944
ENSBTAT00000048357
ENSBTAT00000018966
ENSBTAT00000019004
ENSBTAT00000053358
ENSBTAT00000019052
ENSBTAT00000019063
ENSBTAT00000019073
ENSBTAT00000019091
ENSBTAT00000057152
ENSBTAT00000034482

ENSBTAT00000019107
ENSBTAT00000019123
ENSBTAT00000019145
ENSBTAT00000019176
ENSBTAT00000019177
ENSBTAT00000019184
ENSBTAT00000019199
ENSBTAT00000034160
ENSBTAT00000064807
ENSBTAT00000045750
ENSBTAT00000019248
ENSBTAT00000037034
ENSBTAT00000019278
ENSBTAT00000057381
ENSBTAT00000019316
ENSBTAT00000019323
ENSBTAT00000019331
ENSBTAT00000019346
ENSBTAT00000019347
ENSBTAT00000019405
ENSBTAT00000019413
ENSBTAT00000057312
ENSBTAT00000019450
ENSBTAT00000037115
ENSBTAT00000019464
ENSBTAT00000019468
ENSBTAT00000019502
ENSBTAT00000019517
ENSBTAT00000019520
ENSBTAT00000019525

ENSBTAT00000019527
ENSBTAT00000019528
ENSBTAT00000019538
ENSBTAT00000019547
ENSBTAT00000019555
ENSBTAT00000019556
ENSBTAT00000019570
ENSBTAT00000019573
ENSBTAT00000019575
ENSBTAT00000019577
ENSBTAT00000040084
ENSBTAT00000019583
ENSBTAT00000045810
ENSBTAT00000019620
ENSBTAT00000019638
ENSBTAT00000065058
ENSBTAT00000019642
ENSBTAT00000019650
ENSBTAT00000015430
ENSBTAT00000019664
ENSBTAT00000019702
ENSBTAT00000019753
ENSBTAT00000019755
ENSBTAT00000019780
ENSBTAT00000019792
ENSBTAT00000019798
ENSBTAT00000054539
ENSBTAT00000019808
ENSBTAT00000019818
ENSBTAT00000019857

ENSBTAT00000050377
ENSBTAT00000019879
ENSBTAT00000019921
ENSBTAT00000019928
ENSBTAT00000019929
ENSBTAT00000019938
ENSBTAT00000019942
ENSBTAT00000019944
ENSBTAT00000019952
ENSBTAT00000019953
ENSBTAT00000019955
ENSBTAT00000032087
ENSBTAT00000019969
ENSBTAT00000019980
ENSBTAT00000019984
ENSBTAT00000020010
ENSBTAT00000029019
ENSBTAT00000020040
ENSBTAT00000017901
ENSBTAT00000020065
ENSBTAT00000044111
ENSBTAT00000054490
ENSBTAT00000020072
ENSBTAT00000039737
ENSBTAT00000020084
ENSBTAT00000020086
ENSBTAT00000020093
ENSBTAT00000020092
ENSBTAT00000020094
ENSBTAT00000020114

ENSBTAT00000020127
ENSBTAT00000020145
ENSBTAT00000020148
ENSBTAT00000020150
ENSBTAT00000020160
ENSBTAT00000020166
ENSBTAT00000020184
ENSBTAT00000020215
ENSBTAT00000020229
ENSBTAT00000020233
ENSBTAT00000020262
ENSBTAT00000020299
ENSBTAT00000020304
ENSBTAT00000020313
ENSBTAT00000020323
ENSBTAT00000020326
ENSBTAT00000020343
ENSBTAT00000020347
ENSBTAT00000020402
ENSBTAT00000020407
ENSBTAT00000020414
ENSBTAT00000020427
ENSBTAT00000020444
ENSBTAT00000020449
ENSBTAT00000020452
ENSBTAT00000020461
ENSBTAT00000020462
ENSBTAT00000038477
ENSBTAT00000020476
ENSBTAT00000020480

ENSBTAT00000020493
ENSBTAT00000020504
ENSBTAT00000020505
ENSBTAT00000063152
ENSBTAT00000020539
ENSBTAT00000020555
ENSBTAT00000033091
ENSBTAT00000020577
ENSBTAT00000020594
ENSBTAT00000038149
ENSBTAT00000033428
ENSBTAT00000020635
ENSBTAT00000020642
ENSBTAT00000020661
ENSBTAT00000020662
ENSBTAT00000020672
ENSBTAT00000020700
ENSBTAT00000020691
ENSBTAT00000020704
ENSBTAT00000020715
ENSBTAT00000020727
ENSBTAT00000020730
ENSBTAT00000020732
ENSBTAT00000020741
ENSBTAT00000020744
ENSBTAT00000020758
ENSBTAT00000020782
ENSBTAT00000020811
ENSBTAT00000020826
ENSBTAT00000020830

ENSBTAT00000020863
ENSBTAT00000020877
ENSBTAT00000020876
ENSBTAT00000020880
ENSBTAT00000020889
ENSBTAT00000020900
ENSBTAT00000020909
ENSBTAT00000020911
ENSBTAT00000020932
ENSBTAT00000020950
ENSBTAT00000020956
ENSBTAT00000020971
ENSBTAT00000039334
ENSBTAT00000020979
ENSBTAT00000021000
ENSBTAT00000021020
ENSBTAT00000057413
ENSBTAT00000021035
ENSBTAT00000021043
ENSBTAT00000021062
ENSBTAT00000045119
ENSBTAT00000021123
ENSBTAT00000021128
ENSBTAT00000021145
ENSBTAT00000021172
ENSBTAT00000056246
ENSBTAT00000061522
ENSBTAT00000021203
ENSBTAT00000053527
ENSBTAT00000021212

ENSBTAT00000021215
ENSBTAT00000021230
ENSBTAT00000021249
ENSBTAT00000021251
ENSBTAT00000021322
ENSBTAT00000021336
ENSBTAT00000021348
ENSBTAT00000021369
ENSBTAT00000021371
ENSBTAT00000021386
ENSBTAT00000043826
ENSBTAT00000021425
ENSBTAT00000021429
ENSBTAT00000030804
ENSBTAT00000021449
ENSBTAT00000021450
ENSBTAT00000021454
ENSBTAT00000021500
ENSBTAT00000021491
ENSBTAT00000021490
ENSBTAT00000021503
ENSBTAT00000021510
ENSBTAT00000021515
ENSBTAT00000021525
ENSBTAT00000021534
ENSBTAT00000038261
ENSBTAT00000038271
ENSBTAT00000048865
ENSBTAT00000021572
ENSBTAT00000021578

ENSBTAT00000021587
ENSBTAT00000021592
ENSBTAT00000021628
ENSBTAT00000021641
ENSBTAT00000021642
ENSBTAT00000021643
ENSBTAT00000021651
ENSBTAT00000021659
ENSBTAT00000021661
ENSBTAT00000021671
ENSBTAT00000021672
ENSBTAT00000021695
ENSBTAT00000043322
ENSBTAT00000045558
ENSBTAT00000021722
ENSBTAT00000021730
ENSBTAT00000021731
ENSBTAT00000021739
ENSBTAT00000021749
ENSBTAT00000021759
ENSBTAT00000021762
ENSBTAT00000021764
ENSBTAT00000004503
ENSBTAT00000021769
ENSBTAT00000021784
ENSBTAT00000013107
ENSBTAT00000021796
ENSBTAT00000043580
ENSBTAT00000021892
ENSBTAT00000021901

ENSBTAT00000021911
ENSBTAT00000021927
ENSBTAT00000037722
ENSBTAT00000043933
ENSBTAT00000061206
ENSBTAT00000021948
ENSBTAT00000009636
ENSBTAT00000022009
ENSBTAT00000022012
ENSBTAT00000022022
ENSBTAT00000022048
ENSBTAT00000022077
ENSBTAT00000022088
ENSBTAT00000022087
ENSBTAT00000022092
ENSBTAT00000016659
ENSBTAT00000040082
ENSBTAT00000022161
ENSBTAT00000022175
ENSBTAT00000022178
ENSBTAT00000022189
ENSBTAT00000022193
ENSBTAT00000022204
ENSBTAT00000022215
ENSBTAT00000022232
ENSBTAT00000022240
ENSBTAT00000022272
ENSBTAT00000053940
ENSBTAT00000022334
ENSBTAT00000022342

ENSBTAT00000022375
ENSBTAT00000022378
ENSBTAT00000022391
ENSBTAT00000022449
ENSBTAT00000022450
ENSBTAT00000022477
ENSBTAT00000022481
ENSBTAT00000056553
ENSBTAT00000022529
ENSBTAT00000022579
ENSBTAT00000022592
ENSBTAT00000016571
ENSBTAT00000022601
ENSBTAT00000053272
ENSBTAT00000022603
ENSBTAT00000055638
ENSBTAT00000022606
ENSBTAT00000022613
ENSBTAT00000022619
ENSBTAT00000022629
ENSBTAT00000022630
ENSBTAT00000022639
ENSBTAT00000022650
ENSBTAT00000022654
ENSBTAT00000022677
ENSBTAT00000022685
ENSBTAT00000022700
ENSBTAT00000022714
ENSBTAT00000022730
ENSBTAT00000022735

ENSBTAT00000022764
ENSBTAT00000022755
ENSBTAT00000022772
ENSBTAT00000022777
ENSBTAT00000022792
ENSBTAT00000049569
ENSBTAT00000022858
ENSBTAT00000046059
ENSBTAT00000038007
ENSBTAT00000022876
ENSBTAT00000022878
ENSBTAT00000022884
ENSBTAT00000022904
ENSBTAT00000022919
ENSBTAT00000022924
ENSBTAT00000022930
ENSBTAT00000022932
ENSBTAT00000022936
ENSBTAT00000022949
ENSBTAT00000022955
ENSBTAT00000022970
ENSBTAT00000022991
ENSBTAT00000022993
ENSBTAT00000022994
ENSBTAT00000023005
ENSBTAT00000023019
ENSBTAT00000023020
ENSBTAT00000023028
ENSBTAT00000023039
ENSBTAT00000023054

ENSBTAT00000023059
ENSBTAT00000023064
ENSBTAT00000023103
ENSBTAT00000023116
ENSBTAT00000023123
ENSBTAT00000023169
ENSBTAT00000023175
ENSBTAT00000055126
ENSBTAT00000023186
ENSBTAT00000023194
ENSBTAT00000023208
ENSBTAT00000023214
ENSBTAT00000023219
ENSBTAT00000023230
ENSBTAT00000023241
ENSBTAT00000023255
ENSBTAT00000023260
ENSBTAT00000023262
ENSBTAT00000023292
ENSBTAT00000043123
ENSBTAT00000023305
ENSBTAT00000023306
ENSBTAT00000023310
ENSBTAT00000023319
ENSBTAT00000044911
ENSBTAT00000023325
ENSBTAT00000023342
ENSBTAT00000023354
ENSBTAT00000023389
ENSBTAT00000023395

ENSBTAT00000023421
ENSBTAT00000023445
ENSBTAT00000023455
ENSBTAT00000023475
ENSBTAT00000023483
ENSBTAT00000023490
ENSBTAT00000023493
ENSBTAT00000023512
ENSBTAT00000023528
ENSBTAT00000023537
ENSBTAT00000023541
ENSBTAT00000023581
ENSBTAT00000057329
ENSBTAT00000023603
ENSBTAT00000023617
ENSBTAT00000023618
ENSBTAT00000023643
ENSBTAT00000023651
ENSBTAT00000023660
ENSBTAT00000023664
ENSBTAT00000023670
ENSBTAT00000023671
ENSBTAT00000043956
ENSBTAT00000044575
ENSBTAT00000023693
ENSBTAT00000023735
ENSBTAT00000023757
ENSBTAT00000023769
ENSBTAT00000023771
ENSBTAT00000023772

ENSBTAT00000023781
ENSBTAT00000023791
ENSBTAT00000023807
ENSBTAT00000057027
ENSBTAT00000023819
ENSBTAT00000023840
ENSBTAT00000052338
ENSBTAT00000023846
ENSBTAT00000023859
ENSBTAT00000023860
ENSBTAT00000023863
ENSBTAT00000029986
ENSBTAT00000023871
ENSBTAT00000023876
ENSBTAT00000023908
ENSBTAT00000023918
ENSBTAT00000023922
ENSBTAT00000023924
ENSBTAT00000023994
ENSBTAT00000024014
ENSBTAT00000029374
ENSBTAT00000064784
ENSBTAT00000024036
ENSBTAT00000024060
ENSBTAT00000024072
ENSBTAT00000024128
ENSBTAT00000024140
ENSBTAT00000024161
ENSBTAT00000024196
ENSBTAT00000024209

ENSBTAT00000024218
ENSBTAT00000024226
ENSBTAT00000024245
ENSBTAT00000024268
ENSBTAT00000063809
ENSBTAT00000024288
ENSBTAT00000024290
ENSBTAT00000024300
ENSBTAT00000024302
ENSBTAT00000024322
ENSBTAT00000024323
ENSBTAT00000024326
ENSBTAT00000024329
ENSBTAT00000024331
ENSBTAT00000024335
ENSBTAT00000024340
ENSBTAT00000024367
ENSBTAT00000024378
ENSBTAT00000024386
ENSBTAT00000024393
ENSBTAT00000024426
ENSBTAT00000024465
ENSBTAT00000024500
ENSBTAT00000024503
ENSBTAT00000024509
ENSBTAT00000024541
ENSBTAT00000024550
ENSBTAT00000024552
ENSBTAT00000024557
ENSBTAT00000024559

ENSBTAT00000024586
ENSBTAT00000039622
ENSBTAT00000024605
ENSBTAT00000047361
ENSBTAT00000024632
ENSBTAT00000024660
ENSBTAT00000037865
ENSBTAT00000024666
ENSBTAT00000024669
ENSBTAT00000024677
ENSBTAT00000024676
ENSBTAT00000024688
ENSBTAT00000024690
ENSBTAT00000024692
ENSBTAT00000049552
ENSBTAT00000045069
ENSBTAT00000024762
ENSBTAT00000024788
ENSBTAT00000024794
ENSBTAT00000024820
ENSBTAT00000024823
ENSBTAT00000042576
ENSBTAT00000024834
ENSBTAT00000024853
ENSBTAT00000024873
ENSBTAT00000024911
ENSBTAT00000064858
ENSBTAT00000024925
ENSBTAT00000054699
ENSBTAT00000024943

ENSBTAT00000024956
ENSBTAT00000024959
ENSBTAT00000024972
ENSBTAT00000024979
ENSBTAT00000025003
ENSBTAT00000025007
ENSBTAT00000025010
ENSBTAT00000025016
ENSBTAT00000025035
ENSBTAT00000054338
ENSBTAT00000025061
ENSBTAT00000025086
ENSBTAT00000025085
ENSBTAT00000049768
ENSBTAT00000053671
ENSBTAT00000025113
ENSBTAT00000025139
ENSBTAT00000020283
ENSBTAT00000025148
ENSBTAT00000064305
ENSBTAT00000025174
ENSBTAT00000025186
ENSBTAT00000025237
ENSBTAT00000054636
ENSBTAT00000025270
ENSBTAT00000025281
ENSBTAT00000025292
ENSBTAT00000025298
ENSBTAT00000025322
ENSBTAT00000025339

ENSBTAT00000025342
ENSBTAT00000025350
ENSBTAT00000025347
ENSBTAT00000038492
ENSBTAT00000025398
ENSBTAT00000025399
ENSBTAT00000025404
ENSBTAT00000025401
ENSBTAT00000025423
ENSBTAT00000025420
ENSBTAT00000025433
ENSBTAT00000025437
ENSBTAT00000025495
ENSBTAT00000025499
ENSBTAT00000025512
ENSBTAT00000030984
ENSBTAT00000025556
ENSBTAT00000025561
ENSBTAT00000025617
ENSBTAT00000047079
ENSBTAT00000025629
ENSBTAT00000025630
ENSBTAT00000025656
ENSBTAT00000025667
ENSBTAT00000025668
ENSBTAT00000025674
ENSBTAT00000025677
ENSBTAT00000025698
ENSBTAT00000025710
ENSBTAT00000025728

ENSBTAT00000025742
ENSBTAT00000025755
ENSBTAT00000025763
ENSBTAT00000045803
ENSBTAT00000024083
ENSBTAT00000025806
ENSBTAT00000025837
ENSBTAT00000025849
ENSBTAT00000025851
ENSBTAT00000025858
ENSBTAT00000025864
ENSBTAT00000053067
ENSBTAT00000054736
ENSBTAT00000025894
ENSBTAT00000025902
ENSBTAT00000025911
ENSBTAT00000042844
ENSBTAT00000025922
ENSBTAT00000025936
ENSBTAT00000025959
ENSBTAT00000043712
ENSBTAT00000025978
ENSBTAT00000025997
ENSBTAT00000025999
ENSBTAT00000026002
ENSBTAT00000026009
ENSBTAT00000026031
ENSBTAT00000026036
ENSBTAT00000026039
ENSBTAT00000026053

ENSBTAT00000026074
ENSBTAT00000060988
ENSBTAT00000026091
ENSBTAT00000026116
ENSBTAT00000026139
ENSBTAT00000026148
ENSBTAT00000026177
ENSBTAT00000026216
ENSBTAT00000026225
ENSBTAT00000044861
ENSBTAT00000026259
ENSBTAT00000026275
ENSBTAT00000026278
ENSBTAT00000026299
ENSBTAT00000026300
ENSBTAT00000061607
ENSBTAT00000026343
ENSBTAT00000026348
ENSBTAT00000026346
ENSBTAT00000026353
ENSBTAT00000048850
ENSBTAT00000036296
ENSBTAT00000026386
ENSBTAT00000061291
ENSBTAT00000026412
ENSBTAT00000026420
ENSBTAT00000026421
ENSBTAT00000037698
ENSBTAT00000026485
ENSBTAT00000026498

ENSBTAT00000043931
ENSBTAT00000026525
ENSBTAT00000026549
ENSBTAT00000026566
ENSBTAT00000026571
ENSBTAT00000026582
ENSBTAT00000026587
ENSBTAT00000026604
ENSBTAT00000026602
ENSBTAT00000026630
ENSBTAT00000026632
ENSBTAT00000026661
ENSBTAT00000026673
ENSBTAT00000026695
ENSBTAT00000026720
ENSBTAT00000053125
ENSBTAT00000026770
ENSBTAT00000026774
ENSBTAT00000011795
ENSBTAT00000026824
ENSBTAT00000026841
ENSBTAT00000032159
ENSBTAT00000026883
ENSBTAT00000026892
ENSBTAT00000026904
ENSBTAT00000026914
ENSBTAT00000026936
ENSBTAT00000031607
ENSBTAT00000026978
ENSBTAT00000045004

ENSBTAT00000027016
ENSBTAT00000027017
ENSBTAT00000027082
ENSBTAT00000027091
ENSBTAT00000056042
ENSBTAT00000027119
ENSBTAT00000027120
ENSBTAT00000027122
ENSBTAT00000027123
ENSBTAT00000055764
ENSBTAT00000047285
ENSBTAT00000027185
ENSBTAT00000027191
ENSBTAT00000027207
ENSBTAT00000027212
ENSBTAT00000045508
ENSBTAT00000027230
ENSBTAT00000037140
ENSBTAT00000027241
ENSBTAT00000027250
ENSBTAT00000027251
ENSBTAT00000027261
ENSBTAT00000027270
ENSBTAT00000027275
ENSBTAT00000027302
ENSBTAT00000030504
ENSBTAT00000032588
ENSBTAT00000027332
ENSBTAT00000027333
ENSBTAT00000027339

ENSBTAT00000027352
ENSBTAT00000027356
ENSBTAT00000027369
ENSBTAT00000027370
ENSBTAT00000027375
ENSBTAT00000027390
ENSBTAT00000027393
ENSBTAT00000027396
ENSBTAT00000029392
ENSBTAT00000044045
ENSBTAT00000027435
ENSBTAT00000027436
ENSBTAT00000044884
ENSBTAT00000027458
ENSBTAT00000027472
ENSBTAT00000027478
ENSBTAT00000032316
ENSBTAT00000027499
ENSBTAT00000027510
ENSBTAT00000027511
ENSBTAT00000027539
ENSBTAT00000027553
ENSBTAT00000027565
ENSBTAT00000004644
ENSBTAT00000048852
ENSBTAT00000027616
ENSBTAT00000027623
ENSBTAT00000027628
ENSBTAT00000027633
ENSBTAT00000027666

ENSBTAT00000027674
ENSBTAT00000027681
ENSBTAT00000027683
ENSBTAT00000034457
ENSBTAT00000027713
ENSBTAT00000027714
ENSBTAT00000040368
ENSBTAT00000027722
ENSBTAT00000027728
ENSBTAT00000027735
ENSBTAT00000027736
ENSBTAT00000027741
ENSBTAT00000027751
ENSBTAT00000027768
ENSBTAT00000027770
ENSBTAT00000027807
ENSBTAT00000027819
ENSBTAT00000032353
ENSBTAT00000027836
ENSBTAT00000027837
ENSBTAT00000027839
ENSBTAT00000027841
ENSBTAT00000063746
ENSBTAT00000027866
ENSBTAT00000027878
ENSBTAT00000027880
ENSBTAT00000027883
ENSBTAT00000027917
ENSBTAT00000027962
ENSBTAT00000027964

ENSBTAT00000027969
ENSBTAT00000061150
ENSBTAT00000028045
ENSBTAT00000028049
ENSBTAT00000028074
ENSBTAT00000028103
ENSBTAT00000028110
ENSBTAT00000028120
ENSBTAT00000028139
ENSBTAT00000032152
ENSBTAT00000028202
ENSBTAT00000028218
ENSBTAT00000028232
ENSBTAT00000028236
ENSBTAT00000028244
ENSBTAT00000028246
ENSBTAT00000028248
ENSBTAT00000028260
ENSBTAT00000028261
ENSBTAT00000019371
ENSBTAT00000028310
ENSBTAT00000028313
ENSBTAT00000028329
ENSBTAT00000028339
ENSBTAT00000028330
ENSBTAT00000028350
ENSBTAT00000028357
ENSBTAT00000028426
ENSBTAT00000028430
ENSBTAT00000028479

ENSBTAT00000028493
ENSBTAT00000028496
ENSBTAT00000028499
ENSBTAT00000028513
ENSBTAT00000048500
ENSBTAT00000028556
ENSBTAT00000045348
ENSBTAT00000055374
ENSBTAT00000028589
ENSBTAT00000028591
ENSBTAT00000028607
ENSBTAT00000028615
ENSBTAT00000028638
ENSBTAT00000028646
ENSBTAT00000046692
ENSBTAT00000028659
ENSBTAT00000028661
ENSBTAT00000028676
ENSBTAT00000028684
ENSBTAT00000028686
ENSBTAT00000028701
ENSBTAT00000028708
ENSBTAT00000028712
ENSBTAT00000046490
ENSBTAT00000028777
ENSBTAT00000028793
ENSBTAT00000028809
ENSBTAT00000038127
ENSBTAT00000028814
ENSBTAT00000028836

ENSBTAT00000028851
ENSBTAT00000028866
ENSBTAT00000051965
ENSBTAT00000028880
ENSBTAT00000044068
ENSBTAT00000028907
ENSBTAT00000028949
ENSBTAT00000028963
ENSBTAT00000052393
ENSBTAT00000029020
ENSBTAT00000029038
ENSBTAT00000031986
ENSBTAT00000029044
ENSBTAT00000029067
ENSBTAT00000029070
ENSBTAT00000029073
ENSBTAT00000029078
ENSBTAT00000029089
ENSBTAT00000054910
ENSBTAT00000029129
ENSBTAT00000029136
ENSBTAT00000029147
ENSBTAT00000029148
ENSBTAT00000061231
ENSBTAT00000029170
ENSBTAT00000029201
ENSBTAT00000029211
ENSBTAT00000029239
ENSBTAT00000065786
ENSBTAT00000029251

ENSBTAT00000038468
ENSBTAT00000029279
ENSBTAT00000029308
ENSBTAT00000043779
ENSBTAT00000029325
ENSBTAT00000029353
ENSBTAT00000043682
ENSBTAT00000029424
ENSBTAT00000029458
ENSBTAT00000029459
ENSBTAT00000029461
ENSBTAT00000029610
ENSBTAT00000029688
ENSBTAT00000029813
ENSBTAT00000009159
ENSBTAT00000029860
ENSBTAT00000039475
ENSBTAT00000044489
ENSBTAT00000029979
ENSBTAT00000030005
ENSBTAT00000030041
ENSBTAT00000030129
ENSBTAT00000030134
ENSBTAT00000030281
ENSBTAT00000030284
ENSBTAT00000030326
ENSBTAT00000030328
ENSBTAT00000030331
ENSBTAT00000030370
ENSBTAT00000030426

ENSBTAT00000030439
ENSBTAT00000028781
ENSBTAT00000030499
ENSBTAT00000030506
ENSBTAT00000030510
ENSBTAT00000030562
ENSBTAT00000028488
ENSBTAT00000015489
ENSBTAT00000030621
ENSBTAT00000030742
ENSBTAT00000064079
ENSBTAT00000030791
ENSBTAT00000030821
ENSBTAT00000030853
ENSBTAT00000031643
ENSBTAT00000049776
ENSBTAT00000005884
ENSBTAT00000030952
ENSBTAT00000055646
ENSBTAT00000030986
ENSBTAT00000031060
ENSBTAT00000031093
ENSBTAT00000031116
ENSBTAT00000008047
ENSBTAT00000031177
ENSBTAT00000031184
ENSBTAT00000031211
ENSBTAT00000031229
ENSBTAT00000031231
ENSBTAT00000031240

ENSBTAT00000031344
ENSBTAT00000031357
ENSBTAT00000031377
ENSBTAT00000031388
ENSBTAT00000031447
ENSBTAT00000031484
ENSBTAT00000031499
ENSBTAT00000048985
ENSBTAT00000052308
ENSBTAT00000031546
ENSBTAT00000026486
ENSBTAT00000007966
ENSBTAT00000005546
ENSBTAT00000026908
ENSBTAT00000047485
ENSBTAT00000031699
ENSBTAT00000031738
ENSBTAT00000031754
ENSBTAT00000004038
ENSBTAT00000031796
ENSBTAT00000031807
ENSBTAT00000031817
ENSBTAT00000031889
ENSBTAT00000031901
ENSBTAT00000031936
ENSBTAT00000055955
ENSBTAT00000031948
ENSBTAT00000044404
ENSBTAT00000018187
ENSBTAT00000032006

ENSBTAT00000057116
ENSBTAT00000032086
ENSBTAT00000032097
ENSBTAT00000012912
ENSBTAT00000032123
ENSBTAT00000004430
ENSBTAT00000032182
ENSBTAT00000032324
ENSBTAT00000032340
ENSBTAT00000032401
ENSBTAT00000032494
ENSBTAT00000032540
ENSBTAT00000032623
ENSBTAT00000032657
ENSBTAT00000004594
ENSBTAT00000032748
ENSBTAT00000027932
ENSBTAT00000032833
ENSBTAT00000055181
ENSBTAT00000028027
ENSBTAT00000018033
ENSBTAT00000033044
ENSBTAT00000033058
ENSBTAT00000033078
ENSBTAT00000011226
ENSBTAT00000033131
ENSBTAT00000023283
ENSBTAT00000063161
ENSBTAT00000033208
ENSBTAT00000033218

ENSBTAT00000033224
ENSBTAT00000000975
ENSBTAT00000033317
ENSBTAT00000033319
ENSBTAT00000033366
ENSBTAT00000033367
ENSBTAT00000033368
ENSBTAT00000033370
ENSBTAT00000033375
ENSBTAT00000033378
ENSBTAT00000043254
ENSBTAT00000024234
ENSBTAT00000033409
ENSBTAT00000033457
ENSBTAT00000033472
ENSBTAT00000042719
ENSBTAT00000033610
ENSBTAT00000006677
ENSBTAT00000033656
ENSBTAT00000033679
ENSBTAT00000061438
ENSBTAT00000023985
ENSBTAT00000033864
ENSBTAT00000033903
ENSBTAT00000033923
ENSBTAT00000033975
ENSBTAT00000034039
ENSBTAT00000034054
ENSBTAT00000012998
ENSBTAT00000034177

ENSBTAT00000034185
ENSBTAT00000007086
ENSBTAT00000024349
ENSBTAT00000001088
ENSBTAT00000034274
ENSBTAT00000057439
ENSBTAT00000030188
ENSBTAT00000015298
ENSBTAT00000007729
ENSBTAT00000034352
ENSBTAT00000034446
ENSBTAT00000016352
ENSBTAT00000034480
ENSBTAT00000034486
ENSBTAT00000034513
ENSBTAT00000016656
ENSBTAT00000034546
ENSBTAT00000055493
ENSBTAT00000010641
ENSBTAT00000034704
ENSBTAT00000011233
ENSBTAT00000034826
ENSBTAT00000034833
ENSBTAT00000034843
ENSBTAT00000034856
ENSBTAT00000034863
ENSBTAT00000004508
ENSBTAT00000034966
ENSBTAT00000034976
ENSBTAT00000035050

ENSBTAT00000038553
ENSBTAT00000035106
ENSBTAT00000055820
ENSBTAT00000035139
ENSBTAT00000035145
ENSBTAT00000054299
ENSBTAT00000035254
ENSBTAT00000035290
ENSBTAT00000035312
ENSBTAT00000035330
ENSBTAT00000035341
ENSBTAT00000035359
ENSBTAT00000035366
ENSBTAT00000009446
ENSBTAT00000035434
ENSBTAT00000008372
ENSBTAT00000026816
ENSBTAT00000012751
ENSBTAT00000035521
ENSBTAT00000035646
ENSBTAT00000009155
ENSBTAT00000035680
ENSBTAT00000035684
ENSBTAT00000035714
ENSBTAT00000043991
ENSBTAT00000035773
ENSBTAT00000035828
ENSBTAT00000035859
ENSBTAT00000036093
ENSBTAT00000036108

ENSBTAT00000036142
ENSBTAT00000036150
ENSBTAT00000036205
ENSBTAT00000036401
ENSBTAT00000057428
ENSBTAT00000036507
ENSBTAT00000036523
ENSBTAT00000036586
ENSBTAT00000024034
ENSBTAT00000036665
ENSBTAT00000036708
ENSBTAT00000036890
ENSBTAT00000036918
ENSBTAT00000036964
ENSBTAT00000036972
ENSBTAT00000027976
ENSBTAT00000007924
ENSBTAT00000018539
ENSBTAT00000037061
ENSBTAT00000037068
ENSBTAT00000037095
ENSBTAT00000037185
ENSBTAT00000012252
ENSBTAT00000009150
ENSBTAT00000037244
ENSBTAT00000037245
ENSBTAT00000037268
ENSBTAT00000037324
ENSBTAT00000037353
ENSBTAT00000017351

ENSBTAT00000037367
ENSBTAT00000037390
ENSBTAT00000037397
ENSBTAT00000037423
ENSBTAT00000037460
ENSBTAT00000037468
ENSBTAT00000056899
ENSBTAT00000037541
ENSBTAT00000022034
ENSBTAT00000037545
ENSBTAT00000037567
ENSBTAT00000037660
ENSBTAT00000037671
ENSBTAT00000037673
ENSBTAT00000037688
ENSBTAT00000030994
ENSBTAT00000037720
ENSBTAT00000037876
ENSBTAT00000037969
ENSBTAT00000037975
ENSBTAT00000038103
ENSBTAT00000038144
ENSBTAT00000038235
ENSBTAT00000038236
ENSBTAT00000007099
ENSBTAT00000038344
ENSBTAT00000038349
ENSBTAT00000057490
ENSBTAT00000038384
ENSBTAT00000038407

ENSBTAT00000038471
ENSBTAT00000053960
ENSBTAT00000038460
ENSBTAT00000049376
ENSBTAT00000038638
ENSBTAT00000008243
ENSBTAT00000038755
ENSBTAT00000038758
ENSBTAT00000013301
ENSBTAT00000018558
ENSBTAT00000037058
ENSBTAT00000008981
ENSBTAT00000019635
ENSBTAT00000012533
ENSBTAT00000039065
ENSBTAT00000039090
ENSBTAT00000039166
ENSBTAT00000055970
ENSBTAT00000039223
ENSBTAT00000039245
ENSBTAT00000039296
ENSBTAT00000039297
ENSBTAT00000039303
ENSBTAT00000019241
ENSBTAT00000039322
ENSBTAT00000039344
ENSBTAT00000039360
ENSBTAT00000039366
ENSBTAT00000039377
ENSBTAT00000039492

ENSBTAT00000039566
ENSBTAT00000039634
ENSBTAT00000047360
ENSBTAT00000039684
ENSBTAT00000039687
ENSBTAT00000010166
ENSBTAT00000039788
ENSBTAT00000004244
ENSBTAT00000039792
ENSBTAT00000039825
ENSBTAT00000039827
ENSBTAT00000039828
ENSBTAT00000039829
ENSBTAT00000053859
ENSBTAT00000039940
ENSBTAT00000021494
ENSBTAT00000021196
ENSBTAT00000040033
ENSBTAT00000040038
ENSBTAT00000040067
ENSBTAT00000013247
ENSBTAT00000040092
ENSBTAT00000019777
ENSBTAT00000039779
ENSBTAT00000040181
ENSBTAT00000024473
ENSBTAT00000040201
ENSBTAT00000040257
ENSBTAT00000040276
ENSBTAT00000051994

ENSBTAT00000040314
ENSBTAT00000040333
ENSBTAT00000040404
ENSBTAT00000040418
ENSBTAT00000040425
ENSBTAT00000040443
ENSBTAT00000040447
ENSBTAT00000040476
ENSBTAT00000040485
ENSBTAT00000040487
ENSBTAT00000040501
ENSBTAT00000040535
ENSBTAT00000040611
ENSBTAT00000040637
ENSBTAT00000040655
ENSBTAT00000040706
ENSBTAT00000040712
ENSBTAT00000040718
ENSBTAT00000040737
ENSBTAT00000040740
ENSBTAT00000040741
ENSBTAT00000040744
ENSBTAT00000040755
ENSBTAT00000040757
ENSBTAT00000040800
ENSBTAT00000040802
ENSBTAT00000040811
ENSBTAT00000040838
ENSBTAT00000040844
ENSBTAT00000040851

ENSBTAT00000040852
ENSBTAT00000040879
ENSBTAT00000040937
ENSBTAT00000040953
ENSBTAT00000040956
ENSBTAT00000040963
ENSBTAT00000040969
ENSBTAT00000040997
ENSBTAT00000041001
ENSBTAT00000041009
ENSBTAT00000041012
ENSBTAT00000041022
ENSBTAT00000041049
ENSBTAT00000041050
ENSBTAT00000041056
ENSBTAT00000041063
ENSBTAT00000041064
ENSBTAT00000041073
ENSBTAT00000041077
ENSBTAT00000041081
ENSBTAT00000041085
ENSBTAT00000041088
ENSBTAT00000041174
ENSBTAT00000041189
ENSBTAT00000041191
ENSBTAT00000041193
ENSBTAT00000041194
ENSBTAT00000041227
ENSBTAT00000041237
ENSBTAT00000041240

ENSBTAT00000041260
ENSBTAT00000041269
ENSBTAT00000041278
ENSBTAT00000041299
ENSBTAT00000041303
ENSBTAT00000041318
ENSBTAT00000041356
ENSBTAT00000041360
ENSBTAT00000041368
ENSBTAT00000041370
ENSBTAT00000041387
ENSBTAT00000041420
ENSBTAT00000041435
ENSBTAT00000041449
ENSBTAT00000041455
ENSBTAT00000041460
ENSBTAT00000041466
ENSBTAT00000041486
ENSBTAT00000041494
ENSBTAT00000041495
ENSBTAT00000041499
ENSBTAT00000041506
ENSBTAT00000041507
ENSBTAT00000041539
ENSBTAT00000041547
ENSBTAT00000041552
ENSBTAT00000041558
ENSBTAT00000041585
ENSBTAT00000041588
ENSBTAT00000041616

ENSBTAT00000041629
ENSBTAT00000041662
ENSBTAT00000041670
ENSBTAT00000041671
ENSBTAT00000041674
ENSBTAT00000041682
ENSBTAT00000041695
ENSBTAT00000041696
ENSBTAT00000041722
ENSBTAT00000041782
ENSBTAT00000041831
ENSBTAT00000041840
ENSBTAT00000041853
ENSBTAT00000041870
ENSBTAT00000041891
ENSBTAT00000041919
ENSBTAT00000041923
ENSBTAT00000041948
ENSBTAT00000041955
ENSBTAT00000041962
ENSBTAT00000041971
ENSBTAT00000042004
ENSBTAT00000042019
ENSBTAT00000042030
ENSBTAT00000042042
ENSBTAT00000042062
ENSBTAT00000042092
ENSBTAT00000042139
ENSBTAT00000042140
ENSBTAT00000042141

ENSBTAT00000042142
ENSBTAT00000042143
ENSBTAT00000042144
ENSBTAT00000042147
ENSBTAT00000042148
ENSBTAT00000042149
ENSBTAT00000042150
ENSBTAT00000042151
ENSBTAT00000042153
ENSBTAT00000042154
ENSBTAT00000042155
ENSBTAT00000042156
ENSBTAT00000042157
ENSBTAT00000042160
ENSBTAT00000042161
ENSBTAT00000042162
ENSBTAT00000042164
ENSBTAT00000042166
ENSBTAT00000042167
ENSBTAT00000042168
ENSBTAT00000042169
ENSBTAT00000042170
ENSBTAT00000042171
ENSBTAT00000042172
ENSBTAT00000042173
ENSBTAT00000042175
ENSBTAT00000042176
ENSBTAT00000042177
ENSBTAT00000042179
ENSBTAT00000042181

ENSBTAT00000042182
ENSBTAT00000042183
ENSBTAT00000042184
ENSBTAT00000042186
ENSBTAT00000042187
ENSBTAT00000042188
ENSBTAT00000042189
ENSBTAT00000042190
ENSBTAT00000042191
ENSBTAT00000042193
ENSBTAT00000042194
ENSBTAT00000042196
ENSBTAT00000042197
ENSBTAT00000042199
ENSBTAT00000042201
ENSBTAT00000042204
ENSBTAT00000042205
ENSBTAT00000042206
ENSBTAT00000042207
ENSBTAT00000042208
ENSBTAT00000042209
ENSBTAT00000042212
ENSBTAT00000042215
ENSBTAT00000042218
ENSBTAT00000042220
ENSBTAT00000042221
ENSBTAT00000042222
ENSBTAT00000042223
ENSBTAT00000042224
ENSBTAT00000042225

ENSBTAT00000042226
ENSBTAT00000042227
ENSBTAT00000042228
ENSBTAT00000042229
ENSBTAT00000042230
ENSBTAT00000042231
ENSBTAT00000042232
ENSBTAT00000042233
ENSBTAT00000042234
ENSBTAT00000042235
ENSBTAT00000042236
ENSBTAT00000042238
ENSBTAT00000042239
ENSBTAT00000042240
ENSBTAT00000042241
ENSBTAT00000042242
ENSBTAT00000042243
ENSBTAT00000042244
ENSBTAT00000042246
ENSBTAT00000042247
ENSBTAT00000042248
ENSBTAT00000042249
ENSBTAT00000042250
ENSBTAT00000042254
ENSBTAT00000042256
ENSBTAT00000042257
ENSBTAT00000042259
ENSBTAT00000042260
ENSBTAT00000042263
ENSBTAT00000042264

ENSBTAT00000042265
ENSBTAT00000042266
ENSBTAT00000042268
ENSBTAT00000042269
ENSBTAT00000042270
ENSBTAT00000042271
ENSBTAT00000042272
ENSBTAT00000042274
ENSBTAT00000042275
ENSBTAT00000042276
ENSBTAT00000042277
ENSBTAT00000042280
ENSBTAT00000042281
ENSBTAT00000042282
ENSBTAT00000042283
ENSBTAT00000042284
ENSBTAT00000042285
ENSBTAT00000042286
ENSBTAT00000042288
ENSBTAT00000042289
ENSBTAT00000042290
ENSBTAT00000042291
ENSBTAT00000042292
ENSBTAT00000042293
ENSBTAT00000042294
ENSBTAT00000042295
ENSBTAT00000042296
ENSBTAT00000042297
ENSBTAT00000042299
ENSBTAT00000042300

ENSBTAT00000042301
ENSBTAT00000042303
ENSBTAT00000042304
ENSBTAT00000042305
ENSBTAT00000042306
ENSBTAT00000042308
ENSBTAT00000042309
ENSBTAT00000042310
ENSBTAT00000042311
ENSBTAT00000042312
ENSBTAT00000042314
ENSBTAT00000042316
ENSBTAT00000042317
ENSBTAT00000042318
ENSBTAT00000042319
ENSBTAT00000042321
ENSBTAT00000042324
ENSBTAT00000042327
ENSBTAT00000042328
ENSBTAT00000042329
ENSBTAT00000042330
ENSBTAT00000042332
ENSBTAT00000042333
ENSBTAT00000042334
ENSBTAT00000042335
ENSBTAT00000042336
ENSBTAT00000042338
ENSBTAT00000042339
ENSBTAT00000042340
ENSBTAT00000042341

ENSBTAT00000042344
ENSBTAT00000042346
ENSBTAT00000042347
ENSBTAT00000042348
ENSBTAT00000042349
ENSBTAT00000042351
ENSBTAT00000042352
ENSBTAT00000042353
ENSBTAT00000042354
ENSBTAT00000042355
ENSBTAT00000042356
ENSBTAT00000042357
ENSBTAT00000042358
ENSBTAT00000042359
ENSBTAT00000042360
ENSBTAT00000042361
ENSBTAT00000042362
ENSBTAT00000042366
ENSBTAT00000042367
ENSBTAT00000042368
ENSBTAT00000042369
ENSBTAT00000042370
ENSBTAT00000042372
ENSBTAT00000042373
ENSBTAT00000042374
ENSBTAT00000042375
ENSBTAT00000042377
ENSBTAT00000042378
ENSBTAT00000042381
ENSBTAT00000042382

ENSBTAT00000042383
ENSBTAT00000042389
ENSBTAT00000042392
ENSBTAT00000042393
ENSBTAT00000042395
ENSBTAT00000042396
ENSBTAT00000042398
ENSBTAT00000042399
ENSBTAT00000042403
ENSBTAT00000042405
ENSBTAT00000042406
ENSBTAT00000042408
ENSBTAT00000042409
ENSBTAT00000042414
ENSBTAT00000042417
ENSBTAT00000042418
ENSBTAT00000042419
ENSBTAT00000042425
ENSBTAT00000042426
ENSBTAT00000042428
ENSBTAT00000042430
ENSBTAT00000042431
ENSBTAT00000042432
ENSBTAT00000042434
ENSBTAT00000042435
ENSBTAT00000042438
ENSBTAT00000042439
ENSBTAT00000042441
ENSBTAT00000042443
ENSBTAT00000042445

ENSBTAT00000042446
ENSBTAT00000042447
ENSBTAT00000042450
ENSBTAT00000042452
ENSBTAT00000042461
ENSBTAT00000042462
ENSBTAT00000042463
ENSBTAT00000042464
ENSBTAT00000042465
ENSBTAT00000042468
ENSBTAT00000042469
ENSBTAT00000042474
ENSBTAT00000042476
ENSBTAT00000042478
ENSBTAT00000042479
ENSBTAT00000042481
ENSBTAT00000042483
ENSBTAT00000042484
ENSBTAT00000042485
ENSBTAT00000042486
ENSBTAT00000042487
ENSBTAT00000042488
ENSBTAT00000042489
ENSBTAT00000042490
ENSBTAT00000042492
ENSBTAT00000042493
ENSBTAT00000042496
ENSBTAT00000042497
ENSBTAT00000030494
ENSBTAT00000042499

ENSBTAT00000042500
ENSBTAT00000042501
ENSBTAT00000042502
ENSBTAT00000042547
ENSBTAT00000065462
ENSBTAT00000022358
ENSBTAT00000037488
ENSBTAT00000042591
ENSBTAT00000021281
ENSBTAT00000019097
ENSBTAT00000052827
ENSBTAT00000042693
ENSBTAT00000016080
ENSBTAT00000032574
ENSBTAT00000042757
ENSBTAT00000042759
ENSBTAT00000042776
ENSBTAT00000042796
ENSBTAT00000042798
ENSBTAT00000040435
ENSBTAT00000042813
ENSBTAT00000042835
ENSBTAT00000042836
ENSBTAT00000006694
ENSBTAT00000065276
ENSBTAT00000042930
ENSBTAT00000042939
ENSBTAT00000042959
ENSBTAT00000042967
ENSBTAT00000042977

ENSBTAT00000015500
ENSBTAT00000042997
ENSBTAT00000043000
ENSBTAT00000043009
ENSBTAT00000043010
ENSBTAT00000002923
ENSBTAT00000014630
ENSBTAT00000043070
ENSBTAT00000043104
ENSBTAT00000043105
ENSBTAT00000043124
ENSBTAT00000064734
ENSBTAT00000019218
ENSBTAT00000043182
ENSBTAT00000043202
ENSBTAT00000031821
ENSBTAT00000043219
ENSBTAT00000035343
ENSBTAT00000043285
ENSBTAT00000043297
ENSBTAT00000043341
ENSBTAT00000033420
ENSBTAT00000043364
ENSBTAT00000039063
ENSBTAT00000043432
ENSBTAT00000043446
ENSBTAT00000043477
ENSBTAT00000043527
ENSBTAT00000043530
ENSBTAT00000043536

ENSBTAT00000043564
ENSBTAT00000043576
ENSBTAT00000028333
ENSBTAT00000043607
ENSBTAT00000043626
ENSBTAT00000043631
ENSBTAT00000043641
ENSBTAT00000043656
ENSBTAT00000006046
ENSBTAT00000043721
ENSBTAT00000043744
ENSBTAT00000043738
ENSBTAT00000043768
ENSBTAT00000031774
ENSBTAT00000031066
ENSBTAT00000052387
ENSBTAT00000065619
ENSBTAT00000043887
ENSBTAT00000043907
ENSBTAT00000043923
ENSBTAT00000024492
ENSBTAT00000053045
ENSBTAT00000043967
ENSBTAT00000043971
ENSBTAT00000043983
ENSBTAT00000012155
ENSBTAT00000043998
ENSBTAT00000044012
ENSBTAT00000008283
ENSBTAT00000044017

ENSBTAT00000018668
ENSBTAT00000044024
ENSBTAT00000044037
ENSBTAT00000044039
ENSBTAT00000044059
ENSBTAT00000044109
ENSBTAT00000044161
ENSBTAT00000024148
ENSBTAT00000044171
ENSBTAT00000044175
ENSBTAT00000044192
ENSBTAT00000044198
ENSBTAT00000044201
ENSBTAT00000065452
ENSBTAT00000044228
ENSBTAT00000044237
ENSBTAT00000044263
ENSBTAT00000044267
ENSBTAT00000041741
ENSBTAT00000044295
ENSBTAT00000044307
ENSBTAT00000044340
ENSBTAT00000044362
ENSBTAT00000044419
ENSBTAT00000044441
ENSBTAT00000044451
ENSBTAT00000044454
ENSBTAT00000044455
ENSBTAT00000061338
ENSBTAT00000044483

ENSBTAT00000044527
ENSBTAT00000044557
ENSBTAT00000010816
ENSBTAT00000044584
ENSBTAT00000044586
ENSBTAT00000044591
ENSBTAT00000044622
ENSBTAT00000020712
ENSBTAT00000055547
ENSBTAT00000044683
ENSBTAT00000044721
ENSBTAT00000044743
ENSBTAT00000044763
ENSBTAT00000044774
ENSBTAT00000044775
ENSBTAT00000044791
ENSBTAT00000034610
ENSBTAT00000044792
ENSBTAT00000044816
ENSBTAT00000020802
ENSBTAT00000017049
ENSBTAT00000044882
ENSBTAT00000044912
ENSBTAT00000044952
ENSBTAT00000044962
ENSBTAT00000044971
ENSBTAT00000044972
ENSBTAT00000044974
ENSBTAT00000044975
ENSBTAT00000044979

ENSBTAT00000044982
ENSBTAT00000044991
ENSBTAT00000044999
ENSBTAT00000045018
ENSBTAT00000045033
ENSBTAT00000045035
ENSBTAT00000045036
ENSBTAT00000045039
ENSBTAT00000045041
ENSBTAT00000045043
ENSBTAT00000045044
ENSBTAT00000045040
ENSBTAT00000045045
ENSBTAT00000045046
ENSBTAT00000045047
ENSBTAT00000045050
ENSBTAT00000002124
ENSBTAT00000045053
ENSBTAT00000027308
ENSBTAT00000039714
ENSBTAT00000045075
ENSBTAT00000045076
ENSBTAT00000027984
ENSBTAT00000055432
ENSBTAT00000045135
ENSBTAT00000045140
ENSBTAT00000032794
ENSBTAT00000029578
ENSBTAT00000006102
ENSBTAT00000045181

ENSBTAT00000035587
ENSBTAT00000009301
ENSBTAT00000003993
ENSBTAT00000045214
ENSBTAT00000045226
ENSBTAT00000020409
ENSBTAT00000045249
ENSBTAT00000015205
ENSBTAT00000045292
ENSBTAT00000039809
ENSBTAT00000045309
ENSBTAT00000038934
ENSBTAT00000045347
ENSBTAT00000045417
ENSBTAT00000045443
ENSBTAT00000045464
ENSBTAT00000045470
ENSBTAT00000045474
ENSBTAT00000045476
ENSBTAT00000045499
ENSBTAT00000045522
ENSBTAT00000032050
ENSBTAT00000045634
ENSBTAT00000045669
ENSBTAT00000011044
ENSBTAT00000045683
ENSBTAT00000028824
ENSBTAT00000025810
ENSBTAT00000045758
ENSBTAT00000052348

ENSBTAT00000045768
ENSBTAT00000045775
ENSBTAT00000045777
ENSBTAT00000045806
ENSBTAT00000024774
ENSBTAT00000045823
ENSBTAT00000045877
ENSBTAT00000045888
ENSBTAT00000025005
ENSBTAT00000021580
ENSBTAT00000045934
ENSBTAT00000045935
ENSBTAT00000010665
ENSBTAT00000045971
ENSBTAT00000030526
ENSBTAT00000045973
ENSBTAT00000045974
ENSBTAT00000046016
ENSBTAT00000037576
ENSBTAT00000046019
ENSBTAT00000046022
ENSBTAT00000046026
ENSBTAT00000046029
ENSBTAT00000046071
ENSBTAT00000046158
ENSBTAT00000046195
ENSBTAT00000046199
ENSBTAT00000046215
ENSBTAT00000013555
ENSBTAT00000046264

ENSBTAT00000046291
ENSBTAT00000046300
ENSBTAT00000040658
ENSBTAT00000002908
ENSBTAT00000005410
ENSBTAT00000016860
ENSBTAT00000031096
ENSBTAT00000064400
ENSBTAT00000031236
ENSBTAT00000046408
ENSBTAT00000046413
ENSBTAT00000064917
ENSBTAT00000026123
ENSBTAT00000046475
ENSBTAT00000040565
ENSBTAT00000046479
ENSBTAT00000046481
ENSBTAT00000046123
ENSBTAT00000046544
ENSBTAT00000015296
ENSBTAT00000064910
ENSBTAT00000002208
ENSBTAT00000031749
ENSBTAT00000046602
ENSBTAT00000032422
ENSBTAT00000053697
ENSBTAT00000046664
ENSBTAT00000046670
ENSBTAT00000046671
ENSBTAT00000046677

ENSBTAT00000046681
ENSBTAT00000003434
ENSBTAT00000035337
ENSBTAT00000046801
ENSBTAT00000046822
ENSBTAT00000046825
ENSBTAT00000046828
ENSBTAT00000046873
ENSBTAT00000046900
ENSBTAT00000046912
ENSBTAT00000046929
ENSBTAT00000013229
ENSBTAT00000046962
ENSBTAT00000046971
ENSBTAT00000001659
ENSBTAT00000047042
ENSBTAT00000047063
ENSBTAT00000047077
ENSBTAT00000030422
ENSBTAT00000047113
ENSBTAT00000047138
ENSBTAT00000047145
ENSBTAT00000047154
ENSBTAT00000013175
ENSBTAT00000047207
ENSBTAT00000047229
ENSBTAT00000066201
ENSBTAT00000056768
ENSBTAT00000047281
ENSBTAT00000047282

ENSBTAT00000040496
ENSBTAT00000047298
ENSBTAT00000047310
ENSBTAT00000047345
ENSBTAT00000047346
ENSBTAT00000056907
ENSBTAT00000047381
ENSBTAT00000016201
ENSBTAT00000054133
ENSBTAT00000007964
ENSBTAT00000047427
ENSBTAT00000047431
ENSBTAT00000023667
ENSBTAT00000054129
ENSBTAT00000047451
ENSBTAT00000047477
ENSBTAT00000047481
ENSBTAT00000047495
ENSBTAT00000047514
ENSBTAT00000008053
ENSBTAT00000047576
ENSBTAT00000047600
ENSBTAT00000047606
ENSBTAT00000016731
ENSBTAT00000047640
ENSBTAT00000001633
ENSBTAT00000056752
ENSBTAT00000011706
ENSBTAT00000001189
ENSBTAT00000016870

ENSBTAT00000026313
ENSBTAT00000047671
ENSBTAT00000047690
ENSBTAT00000049902
ENSBTAT00000047705
ENSBTAT00000013293
ENSBTAT00000047746
ENSBTAT00000047761
ENSBTAT00000047772
ENSBTAT00000047775
ENSBTAT00000047834
ENSBTAT00000004744
ENSBTAT00000047871
ENSBTAT00000006179
ENSBTAT00000056123
ENSBTAT00000033854
ENSBTAT00000047919
ENSBTAT00000036399
ENSBTAT00000047942
ENSBTAT00000047957
ENSBTAT00000016633
ENSBTAT00000048035
ENSBTAT00000048029
ENSBTAT00000048092
ENSBTAT00000048096
ENSBTAT00000048193
ENSBTAT00000048220
ENSBTAT00000048223
ENSBTAT00000048243
ENSBTAT00000048290

ENSBTAT00000048335
ENSBTAT00000048360
ENSBTAT00000048418
ENSBTAT00000064169
ENSBTAT00000016624
ENSBTAT00000048462
ENSBTAT00000055845
ENSBTAT00000037528
ENSBTAT00000012682
ENSBTAT00000048558
ENSBTAT00000048556
ENSBTAT00000048577
ENSBTAT00000030657
ENSBTAT00000048604
ENSBTAT00000048614
ENSBTAT00000048619
ENSBTAT00000048626
ENSBTAT00000048643
ENSBTAT00000048647
ENSBTAT00000048691
ENSBTAT00000004678
ENSBTAT00000048729
ENSBTAT00000048734
ENSBTAT00000048749
ENSBTAT00000048772
ENSBTAT00000023331
ENSBTAT00000048782
ENSBTAT00000048791
ENSBTAT00000048819
ENSBTAT00000048870

ENSBTAT00000048868
ENSBTAT00000048881
ENSBTAT00000024022
ENSBTAT00000048901
ENSBTAT00000048906
ENSBTAT00000048944
ENSBTAT00000048949
ENSBTAT00000048981
ENSBTAT00000048984
ENSBTAT00000049024
ENSBTAT00000049038
ENSBTAT00000054874
ENSBTAT00000001099
ENSBTAT00000049099
ENSBTAT00000049126
ENSBTAT00000052013
ENSBTAT00000049181
ENSBTAT00000049203
ENSBTAT00000049218
ENSBTAT00000049221
ENSBTAT00000049224
ENSBTAT00000049233
ENSBTAT00000049315
ENSBTAT00000049313
ENSBTAT00000049320
ENSBTAT00000049345
ENSBTAT00000049353
ENSBTAT00000036488
ENSBTAT00000049374
ENSBTAT00000049392

ENSBTAT00000049391
ENSBTAT00000049400
ENSBTAT00000023889
ENSBTAT00000040854
ENSBTAT00000018930
ENSBTAT00000019827
ENSBTAT00000011655
ENSBTAT00000026692
ENSBTAT00000049490
ENSBTAT00000049474
ENSBTAT00000049493
ENSBTAT00000049500
ENSBTAT00000049503
ENSBTAT00000028631
ENSBTAT00000049518
ENSBTAT00000049553
ENSBTAT00000049641
ENSBTAT00000049648
ENSBTAT00000048945
ENSBTAT00000049679
ENSBTAT00000049678
ENSBTAT00000049696
ENSBTAT00000034356
ENSBTAT00000049766
ENSBTAT00000049801
ENSBTAT00000049877
ENSBTAT00000023534
ENSBTAT00000054559
ENSBTAT00000035268
ENSBTAT00000040332

ENSBTAT00000056297
ENSBTAT00000049985
ENSBTAT00000049990
ENSBTAT00000050022
ENSBTAT00000050094
ENSBTAT00000009857
ENSBTAT00000050220
ENSBTAT00000050230
ENSBTAT00000050255
ENSBTAT00000050264
ENSBTAT00000050265
ENSBTAT00000050286
ENSBTAT00000030308
ENSBTAT00000040346
ENSBTAT00000050438
ENSBTAT00000028668
ENSBTAT00000050462
ENSBTAT00000050482
ENSBTAT00000004342
ENSBTAT00000008346
ENSBTAT00000050485
ENSBTAT00000018283
ENSBTAT00000050534
ENSBTAT00000025021
ENSBTAT00000050559
ENSBTAT00000054100
ENSBTAT00000050619
ENSBTAT00000053409
ENSBTAT00000050635
ENSBTAT00000023382

ENSBTAT00000022824
ENSBTAT00000050760
ENSBTAT00000050788
ENSBTAT00000055669
ENSBTAT00000050856
ENSBTAT00000050857
ENSBTAT00000050858
ENSBTAT00000050859
ENSBTAT00000050860
ENSBTAT00000050861
ENSBTAT00000050862
ENSBTAT00000050863
ENSBTAT00000050864
ENSBTAT00000050865
ENSBTAT00000050866
ENSBTAT00000050867
ENSBTAT00000050868
ENSBTAT00000050869
ENSBTAT00000050871
ENSBTAT00000050872
ENSBTAT00000050873
ENSBTAT00000050874
ENSBTAT00000050875
ENSBTAT00000050876
ENSBTAT00000050879
ENSBTAT00000050880
ENSBTAT00000050881
ENSBTAT00000050883
ENSBTAT00000050886
ENSBTAT00000050888

ENSBTAT00000050889
ENSBTAT00000050891
ENSBTAT00000050892
ENSBTAT00000050895
ENSBTAT00000050896
ENSBTAT00000050897
ENSBTAT00000050898
ENSBTAT00000050899
ENSBTAT00000050900
ENSBTAT00000050902
ENSBTAT00000050903
ENSBTAT00000050904
ENSBTAT00000050906
ENSBTAT00000050907
ENSBTAT00000050908
ENSBTAT00000050910
ENSBTAT00000050911
ENSBTAT00000050914
ENSBTAT00000050915
ENSBTAT00000050917
ENSBTAT00000050918
ENSBTAT00000050919
ENSBTAT00000050920
ENSBTAT00000050921
ENSBTAT00000050922
ENSBTAT00000050923
ENSBTAT00000050925
ENSBTAT00000050926
ENSBTAT00000050928
ENSBTAT00000050929

ENSBTAT00000050930
ENSBTAT00000050931
ENSBTAT00000050933
ENSBTAT00000050935
ENSBTAT00000050936
ENSBTAT00000050937
ENSBTAT00000050938
ENSBTAT00000050940
ENSBTAT00000050942
ENSBTAT00000050943
ENSBTAT00000050944
ENSBTAT00000050945
ENSBTAT00000050951
ENSBTAT00000050975
ENSBTAT00000050976
ENSBTAT00000050980
ENSBTAT00000050988
ENSBTAT00000051016
ENSBTAT00000051042
ENSBTAT00000051053
ENSBTAT00000051062
ENSBTAT00000051078
ENSBTAT00000051087
ENSBTAT00000051103
ENSBTAT00000051116
ENSBTAT00000051118
ENSBTAT00000051121
ENSBTAT00000051125
ENSBTAT00000051128
ENSBTAT00000051135

ENSBTAT00000051137
ENSBTAT00000051144
ENSBTAT00000051146
ENSBTAT00000051149
ENSBTAT00000051166
ENSBTAT00000051178
ENSBTAT00000051232
ENSBTAT00000051243
ENSBTAT00000051244
ENSBTAT00000051254
ENSBTAT00000051266
ENSBTAT00000051277
ENSBTAT00000051294
ENSBTAT00000051307
ENSBTAT00000051323
ENSBTAT00000051341
ENSBTAT00000051351
ENSBTAT00000051356
ENSBTAT00000051358
ENSBTAT00000051389
ENSBTAT00000051402
ENSBTAT00000051408
ENSBTAT00000051421
ENSBTAT00000051427
ENSBTAT00000051429
ENSBTAT00000051433
ENSBTAT00000051442
ENSBTAT00000051445
ENSBTAT00000051446
ENSBTAT00000051450

ENSBTAT00000051458
ENSBTAT00000051462
ENSBTAT00000051468
ENSBTAT00000051470
ENSBTAT00000051478
ENSBTAT00000051482
ENSBTAT00000051493
ENSBTAT00000051495
ENSBTAT00000051497
ENSBTAT00000051506
ENSBTAT00000051509
ENSBTAT00000051510
ENSBTAT00000051513
ENSBTAT00000051526
ENSBTAT00000051530
ENSBTAT00000051572
ENSBTAT00000051587
ENSBTAT00000051596
ENSBTAT00000051608
ENSBTAT00000051610
ENSBTAT00000051611
ENSBTAT00000051647
ENSBTAT00000051653
ENSBTAT00000051667
ENSBTAT00000051674
ENSBTAT00000051679
ENSBTAT00000051682
ENSBTAT00000051687
ENSBTAT00000051699
ENSBTAT00000051705

ENSBTAT00000051706
ENSBTAT00000051726
ENSBTAT00000051729
ENSBTAT00000051739
ENSBTAT00000051743
ENSBTAT00000051750
ENSBTAT00000051757
ENSBTAT00000051761
ENSBTAT00000051763
ENSBTAT00000051764
ENSBTAT00000051765
ENSBTAT00000051768
ENSBTAT00000051774
ENSBTAT00000051775
ENSBTAT00000051777
ENSBTAT00000051788
ENSBTAT00000051791
ENSBTAT00000051792
ENSBTAT00000051793
ENSBTAT00000051796
ENSBTAT00000051797
ENSBTAT00000051800
ENSBTAT00000051802
ENSBTAT00000051804
ENSBTAT00000051806
ENSBTAT00000051807
ENSBTAT00000051811
ENSBTAT00000051816
ENSBTAT00000051819
ENSBTAT00000051822

ENSBTAT00000051825
ENSBTAT00000051828
ENSBTAT00000051830
ENSBTAT00000051832
ENSBTAT00000051836
ENSBTAT00000051839
ENSBTAT00000051848
ENSBTAT00000051850
ENSBTAT00000051853
ENSBTAT00000051854
ENSBTAT00000051857
ENSBTAT00000051858
ENSBTAT00000051860
ENSBTAT00000051863
ENSBTAT00000051865
ENSBTAT00000051869
ENSBTAT00000024782
ENSBTAT00000009916
ENSBTAT00000052439
ENSBTAT00000056897
ENSBTAT00000053062
ENSBTAT00000054070
ENSBTAT00000054043
ENSBTAT00000053988
ENSBTAT00000053293
ENSBTAT00000052785
ENSBTAT00000054521
ENSBTAT00000056174
ENSBTAT00000053616
ENSBTAT00000055698

ENSBTAT00000057098
ENSBTAT00000054620
ENSBTAT00000053241
ENSBTAT00000054214
ENSBTAT00000055994
ENSBTAT00000055206
ENSBTAT00000052506
ENSBTAT00000054274
ENSBTAT00000052043
ENSBTAT00000054480
ENSBTAT00000046813
ENSBTAT00000033276
ENSBTAT00000018034
ENSBTAT00000057144
ENSBTAT00000020563
ENSBTAT00000054004
ENSBTAT00000056910
ENSBTAT00000055048
ENSBTAT00000051986
ENSBTAT00000001422
ENSBTAT00000019939
ENSBTAT00000051843
ENSBTAT00000055760
ENSBTAT00000053189
ENSBTAT00000052775
ENSBTAT00000051948
ENSBTAT00000049813
ENSBTAT00000054483
ENSBTAT00000056528
ENSBTAT00000052538

ENSBTAT00000043331
ENSBTAT00000054907
ENSBTAT00000055395
ENSBTAT00000045103
ENSBTAT00000053513
ENSBTAT00000045994
ENSBTAT00000051855
ENSBTAT00000054195
ENSBTAT00000056370
ENSBTAT00000052834
ENSBTAT00000010196
ENSBTAT00000013146
ENSBTAT00000045696
ENSBTAT00000056431
ENSBTAT00000053562
ENSBTAT00000056991
ENSBTAT00000053390
ENSBTAT00000037755
ENSBTAT00000054242
ENSBTAT00000052598
ENSBTAT00000052401
ENSBTAT00000051799
ENSBTAT00000053149
ENSBTAT00000052460
ENSBTAT00000055027
ENSBTAT00000055839
ENSBTAT00000056023
ENSBTAT00000052457
ENSBTAT00000049348
ENSBTAT00000057022

ENSBTAT00000057287
ENSBTAT00000057550
ENSBTAT00000055532
ENSBTAT00000052234
ENSBTAT00000053345
ENSBTAT00000055502
ENSBTAT00000053332
ENSBTAT00000050194
ENSBTAT00000054753
ENSBTAT00000057234
ENSBTAT00000055080
ENSBTAT00000057070
ENSBTAT00000053872
ENSBTAT00000031174
ENSBTAT00000053108
ENSBTAT00000051769
ENSBTAT00000056538
ENSBTAT00000055530
ENSBTAT00000054598
ENSBTAT00000052722
ENSBTAT00000055828
ENSBTAT00000056091
ENSBTAT00000049487
ENSBTAT00000002092
ENSBTAT00000057495
ENSBTAT00000054755
ENSBTAT00000017363
ENSBTAT00000054210
ENSBTAT00000055323
ENSBTAT00000052085

ENSBTAT00000057444
ENSBTAT00000027753
ENSBTAT00000052622
ENSBTAT00000055282
ENSBTAT00000052334
ENSBTAT00000054492
ENSBTAT00000054184
ENSBTAT00000053334
ENSBTAT00000053035
ENSBTAT00000052912
ENSBTAT00000052524
ENSBTAT00000054390
ENSBTAT00000054922
ENSBTAT00000056022
ENSBTAT00000055327
ENSBTAT00000055587
ENSBTAT00000056229
ENSBTAT00000001575
ENSBTAT00000056211
ENSBTAT00000057250
ENSBTAT00000055036
ENSBTAT00000051753
ENSBTAT00000055463
ENSBTAT00000053648
ENSBTAT00000022850
ENSBTAT00000052847
ENSBTAT00000056757
ENSBTAT00000052905
ENSBTAT00000054421
ENSBTAT00000054463

ENSBTAT00000057315
ENSBTAT00000057188
ENSBTAT00000053143
ENSBTAT00000056139
ENSBTAT00000056122
ENSBTAT00000054719
ENSBTAT00000052276
ENSBTAT00000043148
ENSBTAT00000054134
ENSBTAT00000003926
ENSBTAT00000056472
ENSBTAT00000055969
ENSBTAT00000057526
ENSBTAT00000053741
ENSBTAT00000031114
ENSBTAT00000065672
ENSBTAT00000056346
ENSBTAT00000036249
ENSBTAT00000057464
ENSBTAT00000051866
ENSBTAT00000029133
ENSBTAT00000054631
ENSBTAT00000056971
ENSBTAT00000045062
ENSBTAT00000046084
ENSBTAT00000057233
ENSBTAT00000052975
ENSBTAT00000054759
ENSBTAT00000053236
ENSBTAT00000052944

ENSBTAT00000027602
ENSBTAT00000057372
ENSBTAT00000046357
ENSBTAT00000054081
ENSBTAT00000054805
ENSBTAT00000050271
ENSBTAT00000056904
ENSBTAT00000052633
ENSBTAT00000051893
ENSBTAT00000056748
ENSBTAT00000053406
ENSBTAT00000002257
ENSBTAT00000055304
ENSBTAT00000006013
ENSBTAT00000051902
ENSBTAT00000055454
ENSBTAT00000055525
ENSBTAT00000054567
ENSBTAT00000054593
ENSBTAT00000053512
ENSBTAT00000046456
ENSBTAT00000021335
ENSBTAT00000055107
ENSBTAT00000060953
ENSBTAT00000004340
ENSBTAT00000055072
ENSBTAT00000057054
ENSBTAT00000054988
ENSBTAT00000045694
ENSBTAT00000054725

ENSBTAT00000054450
ENSBTAT00000054356
ENSBTAT00000056113
ENSBTAT00000055316
ENSBTAT00000057576
ENSBTAT00000056656
ENSBTAT00000055297
ENSBTAT00000054256
ENSBTAT00000003514
ENSBTAT00000055860
ENSBTAT00000052552
ENSBTAT00000054860
ENSBTAT00000052963
ENSBTAT00000053461
ENSBTAT00000056942
ENSBTAT00000057446
ENSBTAT00000056142
ENSBTAT00000053728
ENSBTAT00000052925
ENSBTAT00000056535
ENSBTAT00000017749
ENSBTAT00000055619
ENSBTAT00000053956
ENSBTAT00000063830
ENSBTAT00000053230
ENSBTAT00000054125
ENSBTAT00000053520
ENSBTAT00000053636
ENSBTAT00000055640
ENSBTAT00000055408

ENSBTAT00000053567
ENSBTAT00000056852
ENSBTAT00000056397
ENSBTAT00000030022
ENSBTAT00000053577
ENSBTAT00000056946
ENSBTAT00000004669
ENSBTAT00000052917
ENSBTAT00000057380
ENSBTAT00000053302
ENSBTAT00000053981
ENSBTAT00000051943
ENSBTAT00000057501
ENSBTAT00000053945
ENSBTAT00000055945
ENSBTAT00000056403
ENSBTAT00000057036
ENSBTAT00000054682
ENSBTAT00000057011
ENSBTAT00000043834
ENSBTAT00000054298
ENSBTAT00000055189
ENSBTAT00000046979
ENSBTAT00000054489
ENSBTAT00000052784
ENSBTAT00000054409
ENSBTAT00000034708
ENSBTAT00000051846
ENSBTAT00000054111
ENSBTAT00000053491

ENSBTAT00000055872
ENSBTAT00000056731
ENSBTAT00000054426
ENSBTAT00000054548
ENSBTAT00000057343
ENSBTAT00000056893
ENSBTAT00000040584
ENSBTAT00000053877
ENSBTAT00000053028
ENSBTAT00000043637
ENSBTAT00000033461
ENSBTAT00000037383
ENSBTAT00000053274
ENSBTAT00000056576
ENSBTAT00000055031
ENSBTAT00000053055
ENSBTAT00000028845
ENSBTAT00000056558
ENSBTAT00000056176
ENSBTAT00000055522
ENSBTAT00000049105
ENSBTAT00000056931
ENSBTAT00000001911
ENSBTAT00000057398
ENSBTAT00000056972
ENSBTAT00000056596
ENSBTAT00000052805
ENSBTAT00000055977
ENSBTAT00000053048
ENSBTAT00000056963

ENSBTAT00000056317
ENSBTAT00000052071
ENSBTAT00000057187
ENSBTAT00000056921
ENSBTAT00000052806
ENSBTAT00000055064
ENSBTAT00000056231
ENSBTAT00000053587
ENSBTAT00000022269
ENSBTAT00000052830
ENSBTAT00000053526
ENSBTAT00000056720
ENSBTAT00000057165
ENSBTAT00000054475
ENSBTAT00000007516
ENSBTAT00000028085
ENSBTAT00000057189
ENSBTAT00000054746
ENSBTAT00000056065
ENSBTAT00000051951
ENSBTAT00000052723
ENSBTAT00000054099
ENSBTAT00000028992
ENSBTAT00000025892
ENSBTAT00000057034
ENSBTAT00000056151
ENSBTAT00000014123
ENSBTAT00000052535
ENSBTAT00000053976
ENSBTAT00000056128

ENSBTAT00000055338
ENSBTAT00000056358
ENSBTAT00000048608
ENSBTAT00000053997
ENSBTAT00000056314
ENSBTAT00000052050
ENSBTAT00000055909
ENSBTAT00000054883
ENSBTAT00000056360
ENSBTAT00000051955
ENSBTAT00000054995
ENSBTAT00000054045
ENSBTAT00000055713
ENSBTAT00000057366
ENSBTAT00000056278
ENSBTAT00000006318
ENSBTAT00000053051
ENSBTAT00000055798
ENSBTAT00000055662
ENSBTAT00000056804
ENSBTAT00000056983
ENSBTAT00000052185
ENSBTAT00000055702
ENSBTAT00000057588
ENSBTAT00000056015
ENSBTAT00000054054
ENSBTAT00000052997
ENSBTAT00000053184
ENSBTAT00000051748
ENSBTAT00000000867

ENSBTAT00000057378
ENSBTAT00000054716
ENSBTAT00000053373
ENSBTAT00000052282
ENSBTAT00000053957
ENSBTAT00000056355
ENSBTAT00000047913
ENSBTAT00000028685
ENSBTAT00000053843
ENSBTAT00000023402
ENSBTAT00000031925
ENSBTAT00000057487
ENSBTAT00000056967
ENSBTAT00000056283
ENSBTAT00000052736
ENSBTAT00000050266
ENSBTAT00000054277
ENSBTAT00000049047
ENSBTAT00000053797
ENSBTAT00000054065
ENSBTAT00000026050
ENSBTAT00000056082
ENSBTAT00000045825
ENSBTAT00000053990
ENSBTAT00000057578
ENSBTAT00000002894
ENSBTAT00000055041
ENSBTAT00000053857
ENSBTAT00000054507
ENSBTAT00000053504

ENSBTAT00000057554
ENSBTAT00000052434
ENSBTAT00000014406
ENSBTAT00000053863
ENSBTAT00000054166
ENSBTAT00000052225
ENSBTAT00000056259
ENSBTAT00000051953
ENSBTAT00000057148
ENSBTAT00000054401
ENSBTAT00000054925
ENSBTAT00000055482
ENSBTAT00000056595
ENSBTAT00000052801
ENSBTAT00000023553
ENSBTAT00000052545
ENSBTAT00000046740
ENSBTAT00000055448
ENSBTAT00000055754
ENSBTAT00000052022
ENSBTAT00000054741
ENSBTAT00000004865
ENSBTAT00000002273
ENSBTAT00000056936
ENSBTAT00000009020
ENSBTAT00000053199
ENSBTAT00000056505
ENSBTAT00000052868
ENSBTAT00000057570
ENSBTAT00000054915

ENSBTAT00000053012
ENSBTAT00000052344
ENSBTAT00000064059
ENSBTAT00000055882
ENSBTAT00000055005
ENSBTAT00000025538
ENSBTAT00000002201
ENSBTAT00000055578
ENSBTAT00000052109
ENSBTAT00000057467
ENSBTAT00000055443
ENSBTAT00000053580
ENSBTAT00000050415
ENSBTAT00000057065
ENSBTAT00000052402
ENSBTAT00000056258
ENSBTAT00000056435
ENSBTAT00000053253
ENSBTAT00000056869
ENSBTAT00000027035
ENSBTAT00000052250
ENSBTAT00000055066
ENSBTAT00000056095
ENSBTAT00000038258
ENSBTAT00000054389
ENSBTAT00000056781
ENSBTAT00000057403
ENSBTAT00000055136
ENSBTAT00000044482
ENSBTAT00000065402

ENSBTAT00000053329
ENSBTAT00000057394
ENSBTAT00000054643
ENSBTAT00000053546
ENSBTAT00000038997
ENSBTAT00000016802
ENSBTAT00000055302
ENSBTAT00000056670
ENSBTAT00000056069
ENSBTAT00000057212
ENSBTAT00000045897
ENSBTAT00000056772
ENSBTAT00000007393
ENSBTAT00000055337
ENSBTAT00000052426
ENSBTAT00000056811
ENSBTAT00000055981
ENSBTAT00000053053
ENSBTAT00000022469
ENSBTAT00000004196
ENSBTAT00000053793
ENSBTAT00000045444
ENSBTAT00000057480
ENSBTAT00000054768
ENSBTAT00000045009
ENSBTAT00000053207
ENSBTAT00000052972
ENSBTAT00000055852
ENSBTAT00000051814
ENSBTAT00000056446

ENSBTAT00000057411
ENSBTAT00000053962
ENSBTAT00000055142
ENSBTAT00000056054
ENSBTAT00000059071
ENSBTAT00000059072
ENSBTAT00000059073
ENSBTAT00000059074
ENSBTAT00000059075
ENSBTAT00000059076
ENSBTAT00000059077
ENSBTAT00000059078
ENSBTAT00000059079
ENSBTAT00000059081
ENSBTAT00000059082
ENSBTAT00000059083
ENSBTAT00000059084
ENSBTAT00000059086
ENSBTAT00000059087
ENSBTAT00000059088
ENSBTAT00000059089
ENSBTAT00000059090
ENSBTAT00000059091
ENSBTAT00000059092
ENSBTAT00000059093
ENSBTAT00000059094
ENSBTAT00000059095
ENSBTAT00000059097
ENSBTAT00000059098
ENSBTAT00000059099

ENSBTAT00000059102
ENSBTAT00000059103
ENSBTAT00000059105
ENSBTAT00000059106
ENSBTAT00000059107
ENSBTAT00000059109
ENSBTAT00000059110
ENSBTAT00000059111
ENSBTAT00000059117
ENSBTAT00000059118
ENSBTAT00000059119
ENSBTAT00000059121
ENSBTAT00000059122
ENSBTAT00000059123
ENSBTAT00000059124
ENSBTAT00000059125
ENSBTAT00000059126
ENSBTAT00000059127
ENSBTAT00000059128
ENSBTAT00000059129
ENSBTAT00000059131
ENSBTAT00000059132
ENSBTAT00000059133
ENSBTAT00000059134
ENSBTAT00000059135
ENSBTAT00000059140
ENSBTAT00000059141
ENSBTAT00000059142
ENSBTAT00000059144
ENSBTAT00000059145

ENSBTAT00000059146
ENSBTAT00000059148
ENSBTAT00000059149
ENSBTAT00000059150
ENSBTAT00000059151
ENSBTAT00000059152
ENSBTAT00000059155
ENSBTAT00000059156
ENSBTAT00000059158
ENSBTAT00000059160
ENSBTAT00000059161
ENSBTAT00000059163
ENSBTAT00000059165
ENSBTAT00000059167
ENSBTAT00000059168
ENSBTAT00000059169
ENSBTAT00000059170
ENSBTAT00000059171
ENSBTAT00000059172
ENSBTAT00000059173
ENSBTAT00000059174
ENSBTAT00000059175
ENSBTAT00000059176
ENSBTAT00000059178
ENSBTAT00000059180
ENSBTAT00000059181
ENSBTAT00000059182
ENSBTAT00000059183
ENSBTAT00000059184
ENSBTAT00000059186

ENSBTAT00000059187
ENSBTAT00000059188
ENSBTAT00000059190
ENSBTAT00000059191
ENSBTAT00000059192
ENSBTAT00000059193
ENSBTAT00000059195
ENSBTAT00000059196
ENSBTAT00000059198
ENSBTAT00000059201
ENSBTAT00000059203
ENSBTAT00000059205
ENSBTAT00000059207
ENSBTAT00000059208
ENSBTAT00000059209
ENSBTAT00000059210
ENSBTAT00000059211
ENSBTAT00000059212
ENSBTAT00000059216
ENSBTAT00000059217
ENSBTAT00000059218
ENSBTAT00000059219
ENSBTAT00000059220
ENSBTAT00000059222
ENSBTAT00000059223
ENSBTAT00000059224
ENSBTAT00000059225
ENSBTAT00000059226
ENSBTAT00000059227
ENSBTAT00000059228

ENSBTAT00000059229
ENSBTAT00000059230
ENSBTAT00000059232
ENSBTAT00000059233
ENSBTAT00000059234
ENSBTAT00000059236
ENSBTAT00000059237
ENSBTAT00000059238
ENSBTAT00000059242
ENSBTAT00000059246
ENSBTAT00000059247
ENSBTAT00000059248
ENSBTAT00000059249
ENSBTAT00000059250
ENSBTAT00000059251
ENSBTAT00000059252
ENSBTAT00000059253
ENSBTAT00000059255
ENSBTAT00000059256
ENSBTAT00000059257
ENSBTAT00000059258
ENSBTAT00000059260
ENSBTAT00000059261
ENSBTAT00000059263
ENSBTAT00000059264
ENSBTAT00000059265
ENSBTAT00000059266
ENSBTAT00000059267
ENSBTAT00000059268
ENSBTAT00000059270

ENSBTAT00000059271
ENSBTAT00000059272
ENSBTAT00000059273
ENSBTAT00000059274
ENSBTAT00000059275
ENSBTAT00000059276
ENSBTAT00000059277
ENSBTAT00000059279
ENSBTAT00000059281
ENSBTAT00000059282
ENSBTAT00000059283
ENSBTAT00000059284
ENSBTAT00000059285
ENSBTAT00000059286
ENSBTAT00000059287
ENSBTAT00000059289
ENSBTAT00000059290
ENSBTAT00000059291
ENSBTAT00000059292
ENSBTAT00000059293
ENSBTAT00000059295
ENSBTAT00000059296
ENSBTAT00000059297
ENSBTAT00000059299
ENSBTAT00000059300
ENSBTAT00000059301
ENSBTAT00000059302
ENSBTAT00000059303
ENSBTAT00000059304
ENSBTAT00000059305

ENSBTAT00000059306
ENSBTAT00000059307
ENSBTAT00000059308
ENSBTAT00000059309
ENSBTAT00000059311
ENSBTAT00000059312
ENSBTAT00000059313
ENSBTAT00000059314
ENSBTAT00000059315
ENSBTAT00000059317
ENSBTAT00000059318
ENSBTAT00000059319
ENSBTAT00000059321
ENSBTAT00000059322
ENSBTAT00000059323
ENSBTAT00000059324
ENSBTAT00000059325
ENSBTAT00000059326
ENSBTAT00000059327
ENSBTAT00000059331
ENSBTAT00000059335
ENSBTAT00000059336
ENSBTAT00000059337
ENSBTAT00000059338
ENSBTAT00000059339
ENSBTAT00000059341
ENSBTAT00000059342
ENSBTAT00000059343
ENSBTAT00000059345
ENSBTAT00000059346

ENSBTAT00000059347
ENSBTAT00000059349
ENSBTAT00000059350
ENSBTAT00000059351
ENSBTAT00000059352
ENSBTAT00000059353
ENSBTAT00000059354
ENSBTAT00000059355
ENSBTAT00000059356
ENSBTAT00000059357
ENSBTAT00000059358
ENSBTAT00000059360
ENSBTAT00000059361
ENSBTAT00000059362
ENSBTAT00000059363
ENSBTAT00000059365
ENSBTAT00000059367
ENSBTAT00000059368
ENSBTAT00000059369
ENSBTAT00000059370
ENSBTAT00000059372
ENSBTAT00000059373
ENSBTAT00000059374
ENSBTAT00000059375
ENSBTAT00000059376
ENSBTAT00000059377
ENSBTAT00000059379
ENSBTAT00000059380
ENSBTAT00000059381
ENSBTAT00000059382

ENSBTAT00000059383
ENSBTAT00000059385
ENSBTAT00000059386
ENSBTAT00000059387
ENSBTAT00000059388
ENSBTAT00000059389
ENSBTAT00000059390
ENSBTAT00000059392
ENSBTAT00000059393
ENSBTAT00000059394
ENSBTAT00000059395
ENSBTAT00000059396
ENSBTAT00000059397
ENSBTAT00000059398
ENSBTAT00000059399
ENSBTAT00000059400
ENSBTAT00000059401
ENSBTAT00000059403
ENSBTAT00000059404
ENSBTAT00000059406
ENSBTAT00000059407
ENSBTAT00000059408
ENSBTAT00000059409
ENSBTAT00000059410
ENSBTAT00000059412
ENSBTAT00000059413
ENSBTAT00000059415
ENSBTAT00000059417
ENSBTAT00000059418
ENSBTAT00000059419

ENSBTAT00000059420
ENSBTAT00000059421
ENSBTAT00000059422
ENSBTAT00000059424
ENSBTAT00000059425
ENSBTAT00000059426
ENSBTAT00000059430
ENSBTAT00000059431
ENSBTAT00000059432
ENSBTAT00000059434
ENSBTAT00000059435
ENSBTAT00000059436
ENSBTAT00000059437
ENSBTAT00000059438
ENSBTAT00000059439
ENSBTAT00000059440
ENSBTAT00000059441
ENSBTAT00000059442
ENSBTAT00000059444
ENSBTAT00000059445
ENSBTAT00000059446
ENSBTAT00000059447
ENSBTAT00000059448
ENSBTAT00000059450
ENSBTAT00000059451
ENSBTAT00000059452
ENSBTAT00000059453
ENSBTAT00000059454
ENSBTAT00000059455
ENSBTAT00000059456

ENSBTAT00000059458
ENSBTAT00000059459
ENSBTAT00000059460
ENSBTAT00000059462
ENSBTAT00000059463
ENSBTAT00000059464
ENSBTAT00000059465
ENSBTAT00000059466
ENSBTAT00000059467
ENSBTAT00000059468
ENSBTAT00000059470
ENSBTAT00000059471
ENSBTAT00000059472
ENSBTAT00000059473
ENSBTAT00000059474
ENSBTAT00000059475
ENSBTAT00000059476
ENSBTAT00000059477
ENSBTAT00000059480
ENSBTAT00000059481
ENSBTAT00000059482
ENSBTAT00000059483
ENSBTAT00000059484
ENSBTAT00000059485
ENSBTAT00000059486
ENSBTAT00000059490
ENSBTAT00000059491
ENSBTAT00000059492
ENSBTAT00000059493
ENSBTAT00000059494

ENSBTAT00000059495
ENSBTAT00000059496
ENSBTAT00000059498
ENSBTAT00000059499
ENSBTAT00000059500
ENSBTAT00000059501
ENSBTAT00000059503
ENSBTAT00000059505
ENSBTAT00000059506
ENSBTAT00000059507
ENSBTAT00000059509
ENSBTAT00000059510
ENSBTAT00000059511
ENSBTAT00000059512
ENSBTAT00000059513
ENSBTAT00000059514
ENSBTAT00000059515
ENSBTAT00000059516
ENSBTAT00000059517
ENSBTAT00000059518
ENSBTAT00000059519
ENSBTAT00000059520
ENSBTAT00000059521
ENSBTAT00000059522
ENSBTAT00000059523
ENSBTAT00000059524
ENSBTAT00000059525
ENSBTAT00000059527
ENSBTAT00000059528
ENSBTAT00000059529

ENSBTAT00000059530
ENSBTAT00000059531
ENSBTAT00000059532
ENSBTAT00000059533
ENSBTAT00000059535
ENSBTAT00000059537
ENSBTAT00000059538
ENSBTAT00000059540
ENSBTAT00000059541
ENSBTAT00000059542
ENSBTAT00000059543
ENSBTAT00000059544
ENSBTAT00000059545
ENSBTAT00000059547
ENSBTAT00000059548
ENSBTAT00000059549
ENSBTAT00000059550
ENSBTAT00000059551
ENSBTAT00000059553
ENSBTAT00000059554
ENSBTAT00000059555
ENSBTAT00000059556
ENSBTAT00000059557
ENSBTAT00000059558
ENSBTAT00000059559
ENSBTAT00000059561
ENSBTAT00000059565
ENSBTAT00000059566
ENSBTAT00000059567
ENSBTAT00000059568

ENSBTAT00000059569
ENSBTAT00000059570
ENSBTAT00000059571
ENSBTAT00000059572
ENSBTAT00000059573
ENSBTAT00000059574
ENSBTAT00000059576
ENSBTAT00000059578
ENSBTAT00000059579
ENSBTAT00000059580
ENSBTAT00000059581
ENSBTAT00000059582
ENSBTAT00000059584
ENSBTAT00000059585
ENSBTAT00000059586
ENSBTAT00000059587
ENSBTAT00000059589
ENSBTAT00000059590
ENSBTAT00000059591
ENSBTAT00000059592
ENSBTAT00000059593
ENSBTAT00000059594
ENSBTAT00000059595
ENSBTAT00000059596
ENSBTAT00000059597
ENSBTAT00000059599
ENSBTAT00000059600
ENSBTAT00000059602
ENSBTAT00000059603
ENSBTAT00000059604

ENSBTAT00000059605
ENSBTAT00000059606
ENSBTAT00000059607
ENSBTAT00000059608
ENSBTAT00000059610
ENSBTAT00000059611
ENSBTAT00000059612
ENSBTAT00000059613
ENSBTAT00000059614
ENSBTAT00000059615
ENSBTAT00000059616
ENSBTAT00000059617
ENSBTAT00000059618
ENSBTAT00000059621
ENSBTAT00000059622
ENSBTAT00000059623
ENSBTAT00000059625
ENSBTAT00000059626
ENSBTAT00000059628
ENSBTAT00000059629
ENSBTAT00000059630
ENSBTAT00000059631
ENSBTAT00000059632
ENSBTAT00000059634
ENSBTAT00000059635
ENSBTAT00000059636
ENSBTAT00000059637
ENSBTAT00000059638
ENSBTAT00000059639
ENSBTAT00000059644

ENSBTAT00000059645
ENSBTAT00000059646
ENSBTAT00000059647
ENSBTAT00000059649
ENSBTAT00000059651
ENSBTAT00000059652
ENSBTAT00000059653
ENSBTAT00000059654
ENSBTAT00000059655
ENSBTAT00000059656
ENSBTAT00000059657
ENSBTAT00000059659
ENSBTAT00000059660
ENSBTAT00000059661
ENSBTAT00000059665
ENSBTAT00000059666
ENSBTAT00000059668
ENSBTAT00000059669
ENSBTAT00000059671
ENSBTAT00000059672
ENSBTAT00000059673
ENSBTAT00000059675
ENSBTAT00000059676
ENSBTAT00000059677
ENSBTAT00000059679
ENSBTAT00000059680
ENSBTAT00000059681
ENSBTAT00000059682
ENSBTAT00000059683
ENSBTAT00000059684

ENSBTAT00000059685
ENSBTAT00000059688
ENSBTAT00000059689
ENSBTAT00000059691
ENSBTAT00000059692
ENSBTAT00000059694
ENSBTAT00000059696
ENSBTAT00000059697
ENSBTAT00000059698
ENSBTAT00000059699
ENSBTAT00000059700
ENSBTAT00000059701
ENSBTAT00000059702
ENSBTAT00000059703
ENSBTAT00000059704
ENSBTAT00000059705
ENSBTAT00000059706
ENSBTAT00000059707
ENSBTAT00000059710
ENSBTAT00000059711
ENSBTAT00000059712
ENSBTAT00000059713
ENSBTAT00000059714
ENSBTAT00000059715
ENSBTAT00000059716
ENSBTAT00000059718
ENSBTAT00000059719
ENSBTAT00000059720
ENSBTAT00000059721
ENSBTAT00000059722

ENSBTAT00000059723
ENSBTAT00000059725
ENSBTAT00000059726
ENSBTAT00000059727
ENSBTAT00000059728
ENSBTAT00000059729
ENSBTAT00000059730
ENSBTAT00000059731
ENSBTAT00000059733
ENSBTAT00000059734
ENSBTAT00000059735
ENSBTAT00000059736
ENSBTAT00000059738
ENSBTAT00000059741
ENSBTAT00000059742
ENSBTAT00000059743
ENSBTAT00000059745
ENSBTAT00000059747
ENSBTAT00000059749
ENSBTAT00000059750
ENSBTAT00000059752
ENSBTAT00000059753
ENSBTAT00000059754
ENSBTAT00000059755
ENSBTAT00000059756
ENSBTAT00000059757
ENSBTAT00000059759
ENSBTAT00000059761
ENSBTAT00000059762
ENSBTAT00000059763

ENSBTAT00000059764
ENSBTAT00000059765
ENSBTAT00000059766
ENSBTAT00000059767
ENSBTAT00000059768
ENSBTAT00000059769
ENSBTAT00000059772
ENSBTAT00000059773
ENSBTAT00000059774
ENSBTAT00000059775
ENSBTAT00000059776
ENSBTAT00000059777
ENSBTAT00000059778
ENSBTAT00000059779
ENSBTAT00000059780
ENSBTAT00000059781
ENSBTAT00000059782
ENSBTAT00000059784
ENSBTAT00000059787
ENSBTAT00000059789
ENSBTAT00000059791
ENSBTAT00000059793
ENSBTAT00000059795
ENSBTAT00000059796
ENSBTAT00000059797
ENSBTAT00000059798
ENSBTAT00000059799
ENSBTAT00000059800
ENSBTAT00000059802
ENSBTAT00000059803

ENSBTAT00000059804
ENSBTAT00000059805
ENSBTAT00000059806
ENSBTAT00000059807
ENSBTAT00000059808
ENSBTAT00000059809
ENSBTAT00000059810
ENSBTAT00000059811
ENSBTAT00000059812
ENSBTAT00000059813
ENSBTAT00000059815
ENSBTAT00000059817
ENSBTAT00000059818
ENSBTAT00000059819
ENSBTAT00000059821
ENSBTAT00000059822
ENSBTAT00000059824
ENSBTAT00000059825
ENSBTAT00000059826
ENSBTAT00000059827
ENSBTAT00000059828
ENSBTAT00000059829
ENSBTAT00000059830
ENSBTAT00000059831
ENSBTAT00000059832
ENSBTAT00000059833
ENSBTAT00000059834
ENSBTAT00000059835
ENSBTAT00000059836
ENSBTAT00000059837

ENSBTAT00000059838
ENSBTAT00000059839
ENSBTAT00000059840
ENSBTAT00000059841
ENSBTAT00000059843
ENSBTAT00000059844
ENSBTAT00000059845
ENSBTAT00000059846
ENSBTAT00000059847
ENSBTAT00000059849
ENSBTAT00000059850
ENSBTAT00000059854
ENSBTAT00000059855
ENSBTAT00000059856
ENSBTAT00000059857
ENSBTAT00000059858
ENSBTAT00000059859
ENSBTAT00000059860
ENSBTAT00000059861
ENSBTAT00000059862
ENSBTAT00000059863
ENSBTAT00000059865
ENSBTAT00000059866
ENSBTAT00000059867
ENSBTAT00000059868
ENSBTAT00000059869
ENSBTAT00000059870
ENSBTAT00000059871
ENSBTAT00000059872
ENSBTAT00000059873

ENSBTAT00000059874
ENSBTAT00000059875
ENSBTAT00000059876
ENSBTAT00000059877
ENSBTAT00000059878
ENSBTAT00000059879
ENSBTAT00000059880
ENSBTAT00000059881
ENSBTAT00000059882
ENSBTAT00000059883
ENSBTAT00000059885
ENSBTAT00000059888
ENSBTAT00000059889
ENSBTAT00000059890
ENSBTAT00000059891
ENSBTAT00000059894
ENSBTAT00000059897
ENSBTAT00000059899
ENSBTAT00000059902
ENSBTAT00000059903
ENSBTAT00000059906
ENSBTAT00000059907
ENSBTAT00000059908
ENSBTAT00000059909
ENSBTAT00000059910
ENSBTAT00000059911
ENSBTAT00000059912
ENSBTAT00000059913
ENSBTAT00000059914
ENSBTAT00000059916

ENSBTAT00000059917
ENSBTAT00000059918
ENSBTAT00000059919
ENSBTAT00000059922
ENSBTAT00000059923
ENSBTAT00000059925
ENSBTAT00000059926
ENSBTAT00000059927
ENSBTAT00000059928
ENSBTAT00000059929
ENSBTAT00000059930
ENSBTAT00000059931
ENSBTAT00000059932
ENSBTAT00000059933
ENSBTAT00000059934
ENSBTAT00000059935
ENSBTAT00000059936
ENSBTAT00000059938
ENSBTAT00000059939
ENSBTAT00000059940
ENSBTAT00000059941
ENSBTAT00000059942
ENSBTAT00000059943
ENSBTAT00000059944
ENSBTAT00000059945
ENSBTAT00000059946
ENSBTAT00000059947
ENSBTAT00000059948
ENSBTAT00000059949
ENSBTAT00000059952

ENSBTAT00000059953
ENSBTAT00000059954
ENSBTAT00000059955
ENSBTAT00000059957
ENSBTAT00000059958
ENSBTAT00000059960
ENSBTAT00000059961
ENSBTAT00000059962
ENSBTAT00000059963
ENSBTAT00000059964
ENSBTAT00000059965
ENSBTAT00000059966
ENSBTAT00000059968
ENSBTAT00000059969
ENSBTAT00000059972
ENSBTAT00000059973
ENSBTAT00000059974
ENSBTAT00000059976
ENSBTAT00000059978
ENSBTAT00000059979
ENSBTAT00000059980
ENSBTAT00000059981
ENSBTAT00000059982
ENSBTAT00000059983
ENSBTAT00000059984
ENSBTAT00000059985
ENSBTAT00000059987
ENSBTAT00000059988
ENSBTAT00000059989
ENSBTAT00000059990

ENSBTAT00000059991
ENSBTAT00000059992
ENSBTAT00000059993
ENSBTAT00000059994
ENSBTAT00000059995
ENSBTAT00000059996
ENSBTAT00000059997
ENSBTAT00000059998
ENSBTAT00000060001
ENSBTAT00000060003
ENSBTAT00000060004
ENSBTAT00000060005
ENSBTAT00000060006
ENSBTAT00000060007
ENSBTAT00000060008
ENSBTAT00000060010
ENSBTAT00000060011
ENSBTAT00000060012
ENSBTAT00000060013
ENSBTAT00000060015
ENSBTAT00000060016
ENSBTAT00000060017
ENSBTAT00000060018
ENSBTAT00000060020
ENSBTAT00000060021
ENSBTAT00000060022
ENSBTAT00000060024
ENSBTAT00000060025
ENSBTAT00000060026
ENSBTAT00000060027

ENSBTAT00000060028
ENSBTAT00000060029
ENSBTAT00000060030
ENSBTAT00000060031
ENSBTAT00000060032
ENSBTAT00000060034
ENSBTAT00000060035
ENSBTAT00000060036
ENSBTAT00000060037
ENSBTAT00000060038
ENSBTAT00000060039
ENSBTAT00000060040
ENSBTAT00000060041
ENSBTAT00000060042
ENSBTAT00000060043
ENSBTAT00000060045
ENSBTAT00000060046
ENSBTAT00000060047
ENSBTAT00000060048
ENSBTAT00000060049
ENSBTAT00000060051
ENSBTAT00000060056
ENSBTAT00000060058
ENSBTAT00000060059
ENSBTAT00000060060
ENSBTAT00000060062
ENSBTAT00000060063
ENSBTAT00000060064
ENSBTAT00000060065
ENSBTAT00000060069

ENSBTAT00000060070
ENSBTAT00000060071
ENSBTAT00000060072
ENSBTAT00000060073
ENSBTAT00000060075
ENSBTAT00000060076
ENSBTAT00000060077
ENSBTAT00000060078
ENSBTAT00000060080
ENSBTAT00000060081
ENSBTAT00000060082
ENSBTAT00000060083
ENSBTAT00000060084
ENSBTAT00000060087
ENSBTAT00000060088
ENSBTAT00000060089
ENSBTAT00000060090
ENSBTAT00000060091
ENSBTAT00000060092
ENSBTAT00000060093
ENSBTAT00000060095
ENSBTAT00000060096
ENSBTAT00000060097
ENSBTAT00000060098
ENSBTAT00000060099
ENSBTAT00000060101
ENSBTAT00000060103
ENSBTAT00000060104
ENSBTAT00000060106
ENSBTAT00000060108

ENSBTAT00000060109
ENSBTAT00000060110
ENSBTAT00000060111
ENSBTAT00000060112
ENSBTAT00000060113
ENSBTAT00000060114
ENSBTAT00000060115
ENSBTAT00000060118
ENSBTAT00000060119
ENSBTAT00000060120
ENSBTAT00000060122
ENSBTAT00000060123
ENSBTAT00000060124
ENSBTAT00000060125
ENSBTAT00000060127
ENSBTAT00000060129
ENSBTAT00000060130
ENSBTAT00000060131
ENSBTAT00000060132
ENSBTAT00000060133
ENSBTAT00000060134
ENSBTAT00000060135
ENSBTAT00000060136
ENSBTAT00000060137
ENSBTAT00000060138
ENSBTAT00000060139
ENSBTAT00000060140
ENSBTAT00000060142
ENSBTAT00000060143
ENSBTAT00000060144

ENSBTAT00000060145
ENSBTAT00000060146
ENSBTAT00000060148
ENSBTAT00000060149
ENSBTAT00000060150
ENSBTAT00000060151
ENSBTAT00000060152
ENSBTAT00000060153
ENSBTAT00000060154
ENSBTAT00000060155
ENSBTAT00000060156
ENSBTAT00000060157
ENSBTAT00000060158
ENSBTAT00000060159
ENSBTAT00000060160
ENSBTAT00000060161
ENSBTAT00000060162
ENSBTAT00000060165
ENSBTAT00000060167
ENSBTAT00000060169
ENSBTAT00000060170
ENSBTAT00000060171
ENSBTAT00000060172
ENSBTAT00000060173
ENSBTAT00000060174
ENSBTAT00000060175
ENSBTAT00000060176
ENSBTAT00000060177
ENSBTAT00000060178
ENSBTAT00000060179

ENSBTAT00000060180
ENSBTAT00000060181
ENSBTAT00000060182
ENSBTAT00000060184
ENSBTAT00000060186
ENSBTAT00000060187
ENSBTAT00000060188
ENSBTAT00000060189
ENSBTAT00000060190
ENSBTAT00000060191
ENSBTAT00000060192
ENSBTAT00000060193
ENSBTAT00000060194
ENSBTAT00000060195
ENSBTAT00000060197
ENSBTAT00000060198
ENSBTAT00000060199
ENSBTAT00000060201
ENSBTAT00000060202
ENSBTAT00000060203
ENSBTAT00000060204
ENSBTAT00000060205
ENSBTAT00000060206
ENSBTAT00000060208
ENSBTAT00000060209
ENSBTAT00000060210
ENSBTAT00000060211
ENSBTAT00000060212
ENSBTAT00000060213
ENSBTAT00000060214

ENSBTAT00000060215
ENSBTAT00000060216
ENSBTAT00000060217
ENSBTAT00000060218
ENSBTAT00000060219
ENSBTAT00000060220
ENSBTAT00000060221
ENSBTAT00000060222
ENSBTAT00000060223
ENSBTAT00000060224
ENSBTAT00000060225
ENSBTAT00000060226
ENSBTAT00000060227
ENSBTAT00000060228
ENSBTAT00000060229
ENSBTAT00000060231
ENSBTAT00000060232
ENSBTAT00000060233
ENSBTAT00000060234
ENSBTAT00000060235
ENSBTAT00000060236
ENSBTAT00000060237
ENSBTAT00000060238
ENSBTAT00000060239
ENSBTAT00000060241
ENSBTAT00000060242
ENSBTAT00000060243
ENSBTAT00000060244
ENSBTAT00000060245
ENSBTAT00000060246

ENSBTAT00000060247
ENSBTAT00000060248
ENSBTAT00000060249
ENSBTAT00000060250
ENSBTAT00000060251
ENSBTAT00000060252
ENSBTAT00000060253
ENSBTAT00000060254
ENSBTAT00000060255
ENSBTAT00000060256
ENSBTAT00000060258
ENSBTAT00000060259
ENSBTAT00000060260
ENSBTAT00000060264
ENSBTAT00000060265
ENSBTAT00000060267
ENSBTAT00000060268
ENSBTAT00000060269
ENSBTAT00000060270
ENSBTAT00000060271
ENSBTAT00000060273
ENSBTAT00000060274
ENSBTAT00000060276
ENSBTAT00000060278
ENSBTAT00000060279
ENSBTAT00000060280
ENSBTAT00000060282
ENSBTAT00000060283
ENSBTAT00000060285
ENSBTAT00000060286

ENSBTAT00000060287
ENSBTAT00000060288
ENSBTAT00000060289
ENSBTAT00000060290
ENSBTAT00000060291
ENSBTAT00000060292
ENSBTAT00000060293
ENSBTAT00000060294
ENSBTAT00000060295
ENSBTAT00000060296
ENSBTAT00000060297
ENSBTAT00000060298
ENSBTAT00000060299
ENSBTAT00000060300
ENSBTAT00000060303
ENSBTAT00000060304
ENSBTAT00000060305
ENSBTAT00000060306
ENSBTAT00000060307
ENSBTAT00000060308
ENSBTAT00000060309
ENSBTAT00000060310
ENSBTAT00000060311
ENSBTAT00000060312
ENSBTAT00000060314
ENSBTAT00000060315
ENSBTAT00000060316
ENSBTAT00000060317
ENSBTAT00000060319
ENSBTAT00000060320

ENSBTAT00000060321
ENSBTAT00000060322
ENSBTAT00000060323
ENSBTAT00000060324
ENSBTAT00000060325
ENSBTAT00000060326
ENSBTAT00000060327
ENSBTAT00000060330
ENSBTAT00000060331
ENSBTAT00000060332
ENSBTAT00000060333
ENSBTAT00000060334
ENSBTAT00000060335
ENSBTAT00000060336
ENSBTAT00000060339
ENSBTAT00000060340
ENSBTAT00000060341
ENSBTAT00000060342
ENSBTAT00000060345
ENSBTAT00000060346
ENSBTAT00000060348
ENSBTAT00000060352
ENSBTAT00000060353
ENSBTAT00000060354
ENSBTAT00000060356
ENSBTAT00000060357
ENSBTAT00000060358
ENSBTAT00000060359
ENSBTAT00000060360
ENSBTAT00000060361

ENSBTAT00000060362
ENSBTAT00000060363
ENSBTAT00000060364
ENSBTAT00000060365
ENSBTAT00000060366
ENSBTAT00000060367
ENSBTAT00000060368
ENSBTAT00000060369
ENSBTAT00000060370
ENSBTAT00000060371
ENSBTAT00000060372
ENSBTAT00000060373
ENSBTAT00000060374
ENSBTAT00000060375
ENSBTAT00000060377
ENSBTAT00000060379
ENSBTAT00000060380
ENSBTAT00000060381
ENSBTAT00000060382
ENSBTAT00000060383
ENSBTAT00000060384
ENSBTAT00000060386
ENSBTAT00000060387
ENSBTAT00000060388
ENSBTAT00000060390
ENSBTAT00000060391
ENSBTAT00000060392
ENSBTAT00000060394
ENSBTAT00000060395
ENSBTAT00000060396

ENSBTAT00000060397
ENSBTAT00000060398
ENSBTAT00000060399
ENSBTAT00000060400
ENSBTAT00000060401
ENSBTAT00000060402
ENSBTAT00000060403
ENSBTAT00000060404
ENSBTAT00000060405
ENSBTAT00000060406
ENSBTAT00000060407
ENSBTAT00000060408
ENSBTAT00000060409
ENSBTAT00000060410
ENSBTAT00000060411
ENSBTAT00000060414
ENSBTAT00000060415
ENSBTAT00000060416
ENSBTAT00000060417
ENSBTAT00000060418
ENSBTAT00000060419
ENSBTAT00000060420
ENSBTAT00000060422
ENSBTAT00000060423
ENSBTAT00000060424
ENSBTAT00000060425
ENSBTAT00000060426
ENSBTAT00000060429
ENSBTAT00000060430
ENSBTAT00000060431

ENSBTAT00000060432
ENSBTAT00000060434
ENSBTAT00000060435
ENSBTAT00000060436
ENSBTAT00000060437
ENSBTAT00000060438
ENSBTAT00000060439
ENSBTAT00000060441
ENSBTAT00000060442
ENSBTAT00000060443
ENSBTAT00000060444
ENSBTAT00000060445
ENSBTAT00000060446
ENSBTAT00000060447
ENSBTAT00000060450
ENSBTAT00000060451
ENSBTAT00000060452
ENSBTAT00000060453
ENSBTAT00000060454
ENSBTAT00000060456
ENSBTAT00000060457
ENSBTAT00000060459
ENSBTAT00000060460
ENSBTAT00000060461
ENSBTAT00000060463
ENSBTAT00000060464
ENSBTAT00000060465
ENSBTAT00000060466
ENSBTAT00000060467
ENSBTAT00000060468

ENSBTAT00000060469
ENSBTAT00000060470
ENSBTAT00000060472
ENSBTAT00000060473
ENSBTAT00000060474
ENSBTAT00000060475
ENSBTAT00000060476
ENSBTAT00000060477
ENSBTAT00000060478
ENSBTAT00000060479
ENSBTAT00000060480
ENSBTAT00000060481
ENSBTAT00000060482
ENSBTAT00000060483
ENSBTAT00000060484
ENSBTAT00000060485
ENSBTAT00000060488
ENSBTAT00000060489
ENSBTAT00000060490
ENSBTAT00000060491
ENSBTAT00000060494
ENSBTAT00000060495
ENSBTAT00000060497
ENSBTAT00000060499
ENSBTAT00000060500
ENSBTAT00000060501
ENSBTAT00000060503
ENSBTAT00000060504
ENSBTAT00000060506
ENSBTAT00000060508

ENSBTAT00000060509
ENSBTAT00000060510
ENSBTAT00000060511
ENSBTAT00000060512
ENSBTAT00000060516
ENSBTAT00000060517
ENSBTAT00000060518
ENSBTAT00000060519
ENSBTAT00000060520
ENSBTAT00000060521
ENSBTAT00000060522
ENSBTAT00000060523
ENSBTAT00000060524
ENSBTAT00000060526
ENSBTAT00000060532
ENSBTAT00000060533
ENSBTAT00000060534
ENSBTAT00000060555
ENSBTAT00000060545
ENSBTAT00000060559
ENSBTAT00000060538
ENSBTAT00000060557
ENSBTAT00000060568
ENSBTAT00000060565
ENSBTAT00000060574
ENSBTAT00000060551
ENSBTAT00000060549
ENSBTAT00000060541
ENSBTAT00000060560
ENSBTAT00000060573

ENSBTAT00000060542
ENSBTAT00000060546
ENSBTAT00000060540
ENSBTAT00000060548
ENSBTAT00000060572
ENSBTAT00000060563
ENSBTAT00000060570
ENSBTAT00000060558
ENSBTAT00000060537
ENSBTAT00000060550
ENSBTAT00000060564
ENSBTAT00000060536
ENSBTAT00000060544
ENSBTAT00000060576
ENSBTAT00000060579
ENSBTAT00000060581
ENSBTAT00000060582
ENSBTAT00000060583
ENSBTAT00000060584
ENSBTAT00000060585
ENSBTAT00000060586
ENSBTAT00000060587
ENSBTAT00000060591
ENSBTAT00000060594
ENSBTAT00000060596
ENSBTAT00000060597
ENSBTAT00000060598
ENSBTAT00000060599
ENSBTAT00000060601
ENSBTAT00000060603

ENSBTAT00000060604
ENSBTAT00000060606
ENSBTAT00000060607
ENSBTAT00000060611
ENSBTAT00000060612
ENSBTAT00000060614
ENSBTAT00000060617
ENSBTAT00000060618
ENSBTAT00000060619
ENSBTAT00000060620
ENSBTAT00000060624
ENSBTAT00000060626
ENSBTAT00000060628
ENSBTAT00000060629
ENSBTAT00000060631
ENSBTAT00000060633
ENSBTAT00000060634
ENSBTAT00000060635
ENSBTAT00000060636
ENSBTAT00000060637
ENSBTAT00000060639
ENSBTAT00000060641
ENSBTAT00000060643
ENSBTAT00000060646
ENSBTAT00000060647
ENSBTAT00000060648
ENSBTAT00000060649
ENSBTAT00000060650
ENSBTAT00000060652
ENSBTAT00000060653

ENSBTAT00000060654
ENSBTAT00000060655
ENSBTAT00000060656
ENSBTAT00000060658
ENSBTAT00000060660
ENSBTAT00000060661
ENSBTAT00000060662
ENSBTAT00000060663
ENSBTAT00000060665
ENSBTAT00000060666
ENSBTAT00000060667
ENSBTAT00000060668
ENSBTAT00000060669
ENSBTAT00000060672
ENSBTAT00000060675
ENSBTAT00000060677
ENSBTAT00000060678
ENSBTAT00000060680
ENSBTAT00000060682
ENSBTAT00000060683
ENSBTAT00000060684
ENSBTAT00000060685
ENSBTAT00000060686
ENSBTAT00000060687
ENSBTAT00000060688
ENSBTAT00000060689
ENSBTAT00000060690
ENSBTAT00000060691
ENSBTAT00000060692
ENSBTAT00000060693

ENSBTAT00000060694
ENSBTAT00000060696
ENSBTAT00000060698
ENSBTAT00000060699
ENSBTAT00000060700
ENSBTAT00000060702
ENSBTAT00000060704
ENSBTAT00000060705
ENSBTAT00000060708
ENSBTAT00000060709
ENSBTAT00000060711
ENSBTAT00000060712
ENSBTAT00000060713
ENSBTAT00000060714
ENSBTAT00000060715
ENSBTAT00000060716
ENSBTAT00000060717
ENSBTAT00000060718
ENSBTAT00000060721
ENSBTAT00000060722
ENSBTAT00000060724
ENSBTAT00000060725
ENSBTAT00000060726
ENSBTAT00000060730
ENSBTAT00000060731
ENSBTAT00000060734
ENSBTAT00000060736
ENSBTAT00000060737
ENSBTAT00000060738
ENSBTAT00000060739

ENSBTAT00000060742
ENSBTAT00000060743
ENSBTAT00000060745
ENSBTAT00000060746
ENSBTAT00000060747
ENSBTAT00000060748
ENSBTAT00000060750
ENSBTAT00000060751
ENSBTAT00000060752
ENSBTAT00000060753
ENSBTAT00000060756
ENSBTAT00000060757
ENSBTAT00000060758
ENSBTAT00000060760
ENSBTAT00000060761
ENSBTAT00000060762
ENSBTAT00000060763
ENSBTAT00000060766
ENSBTAT00000060767
ENSBTAT00000060768
ENSBTAT00000060770
ENSBTAT00000060771
ENSBTAT00000060773
ENSBTAT00000060775
ENSBTAT00000060776
ENSBTAT00000060777
ENSBTAT00000060778
ENSBTAT00000060779
ENSBTAT00000060781
ENSBTAT00000060783

ENSBTAT00000060784
ENSBTAT00000060785
ENSBTAT00000060786
ENSBTAT00000060787
ENSBTAT00000060789
ENSBTAT00000060790
ENSBTAT00000060791
ENSBTAT00000060792
ENSBTAT00000060793
ENSBTAT00000060794
ENSBTAT00000060795
ENSBTAT00000060796
ENSBTAT00000060797
ENSBTAT00000060798
ENSBTAT00000060799
ENSBTAT00000060800
ENSBTAT00000060805
ENSBTAT00000060806
ENSBTAT00000060807
ENSBTAT00000060808
ENSBTAT00000060813
ENSBTAT00000060814
ENSBTAT00000060815
ENSBTAT00000060817
ENSBTAT00000060818
ENSBTAT00000060819
ENSBTAT00000060820
ENSBTAT00000060821
ENSBTAT00000060822
ENSBTAT00000060823

ENSBTAT00000060824
ENSBTAT00000060825
ENSBTAT00000060826
ENSBTAT00000060827
ENSBTAT00000060828
ENSBTAT00000060830
ENSBTAT00000060832
ENSBTAT00000060833
ENSBTAT00000060835
ENSBTAT00000060837
ENSBTAT00000060838
ENSBTAT00000060839
ENSBTAT00000060840
ENSBTAT00000060842
ENSBTAT00000060845
ENSBTAT00000060846
ENSBTAT00000060849
ENSBTAT00000060851
ENSBTAT00000060852
ENSBTAT00000060854
ENSBTAT00000060855
ENSBTAT00000060856
ENSBTAT00000060858
ENSBTAT00000060859
ENSBTAT00000060861
ENSBTAT00000060862
ENSBTAT00000060863
ENSBTAT00000060864
ENSBTAT00000060865
ENSBTAT00000060866

ENSBTAT00000060867
ENSBTAT00000060868
ENSBTAT00000060869
ENSBTAT00000060871
ENSBTAT00000060872
ENSBTAT00000060875
ENSBTAT00000060879
ENSBTAT00000060880
ENSBTAT00000060884
ENSBTAT00000060885
ENSBTAT00000060888
ENSBTAT00000060889
ENSBTAT00000060890
ENSBTAT00000060891
ENSBTAT00000060892
ENSBTAT00000060895
ENSBTAT00000060896
ENSBTAT00000060897
ENSBTAT00000060899
ENSBTAT00000060901
ENSBTAT00000060903
ENSBTAT00000060906
ENSBTAT00000060907
ENSBTAT00000060909
ENSBTAT00000060910
ENSBTAT00000060911
ENSBTAT00000060912
ENSBTAT00000060915
ENSBTAT00000060918
ENSBTAT00000060919

ENSBTAT00000060920
ENSBTAT00000060921
ENSBTAT00000060923
ENSBTAT00000060924
ENSBTAT00000060925
ENSBTAT00000060927
ENSBTAT00000060928
ENSBTAT00000060930
ENSBTAT00000060931
ENSBTAT00000060932
ENSBTAT00000060933
ENSBTAT00000060934
ENSBTAT00000060935
ENSBTAT00000060936
ENSBTAT00000060937
ENSBTAT00000060938
ENSBTAT00000061416
ENSBTAT00000061350
ENSBTAT00000061329
ENSBTAT00000061579
ENSBTAT00000061604
ENSBTAT00000061219
ENSBTAT00000061465
ENSBTAT00000061341
ENSBTAT00000061221
ENSBTAT00000027860
ENSBTAT00000060951
ENSBTAT00000061571
ENSBTAT00000061362
ENSBTAT00000061548

ENSBTAT00000060985
ENSBTAT00000061253
ENSBTAT00000061466
ENSBTAT00000060967
ENSBTAT00000061498
ENSBTAT00000061310
ENSBTAT00000061429
ENSBTAT00000061409
ENSBTAT00000060982
ENSBTAT00000061274
ENSBTAT00000061397
ENSBTAT00000061366
ENSBTAT00000061614
ENSBTAT00000061294
ENSBTAT00000061486
ENSBTAT00000061186
ENSBTAT00000061004
ENSBTAT00000061012
ENSBTAT00000061309
ENSBTAT00000061561
ENSBTAT00000061053
ENSBTAT00000060946
ENSBTAT00000061218
ENSBTAT00000060981
ENSBTAT00000061049
ENSBTAT00000061470
ENSBTAT00000061462
ENSBTAT00000061647
ENSBTAT00000061648
ENSBTAT00000061650

ENSBTAT00000061651
ENSBTAT00000061653
ENSBTAT00000061654
ENSBTAT00000061655
ENSBTAT00000061656
ENSBTAT00000061657
ENSBTAT00000061658
ENSBTAT00000061659
ENSBTAT00000061660
ENSBTAT00000061661
ENSBTAT00000061662
ENSBTAT00000061663
ENSBTAT00000061664
ENSBTAT00000061665
ENSBTAT00000061666
ENSBTAT00000061668
ENSBTAT00000061669
ENSBTAT00000061670
ENSBTAT00000061673
ENSBTAT00000061677
ENSBTAT00000061679
ENSBTAT00000061680
ENSBTAT00000061681
ENSBTAT00000061684
ENSBTAT00000061686
ENSBTAT00000061688
ENSBTAT00000061689
ENSBTAT00000061690
ENSBTAT00000061691
ENSBTAT00000061692

ENSBTAT00000061693
ENSBTAT00000061694
ENSBTAT00000061695
ENSBTAT00000061696
ENSBTAT00000061697
ENSBTAT00000061698
ENSBTAT00000061699
ENSBTAT00000061700
ENSBTAT00000061702
ENSBTAT00000061703
ENSBTAT00000061704
ENSBTAT00000061705
ENSBTAT00000061706
ENSBTAT00000061707
ENSBTAT00000061708
ENSBTAT00000061710
ENSBTAT00000061711
ENSBTAT00000061712
ENSBTAT00000061713
ENSBTAT00000061715
ENSBTAT00000061716
ENSBTAT00000061717
ENSBTAT00000061718
ENSBTAT00000061719
ENSBTAT00000061720
ENSBTAT00000061721
ENSBTAT00000061722
ENSBTAT00000061724
ENSBTAT00000061726
ENSBTAT00000061727

ENSBTAT00000061728
ENSBTAT00000061731
ENSBTAT00000061732
ENSBTAT00000061733
ENSBTAT00000061734
ENSBTAT00000061736
ENSBTAT00000061737
ENSBTAT00000061739
ENSBTAT00000061740
ENSBTAT00000061741
ENSBTAT00000061742
ENSBTAT00000061744
ENSBTAT00000061748
ENSBTAT00000061749
ENSBTAT00000061750
ENSBTAT00000061751
ENSBTAT00000061752
ENSBTAT00000061753
ENSBTAT00000061755
ENSBTAT00000061757
ENSBTAT00000061758
ENSBTAT00000061759
ENSBTAT00000061760
ENSBTAT00000061762
ENSBTAT00000061763
ENSBTAT00000061764
ENSBTAT00000061765
ENSBTAT00000061768
ENSBTAT00000061769
ENSBTAT00000061770

ENSBTAT00000061772
ENSBTAT00000061774
ENSBTAT00000061776
ENSBTAT00000061778
ENSBTAT00000061779
ENSBTAT00000061780
ENSBTAT00000061781
ENSBTAT00000061783
ENSBTAT00000061784
ENSBTAT00000061785
ENSBTAT00000061787
ENSBTAT00000061788
ENSBTAT00000061794
ENSBTAT00000061795
ENSBTAT00000061796
ENSBTAT00000061797
ENSBTAT00000061798
ENSBTAT00000061799
ENSBTAT00000061800
ENSBTAT00000061802
ENSBTAT00000061803
ENSBTAT00000061804
ENSBTAT00000061806
ENSBTAT00000061807
ENSBTAT00000061808
ENSBTAT00000061809
ENSBTAT00000061810
ENSBTAT00000061811
ENSBTAT00000061812
ENSBTAT00000061813

ENSBTAT00000061814
ENSBTAT00000061817
ENSBTAT00000061819
ENSBTAT00000061820
ENSBTAT00000061822
ENSBTAT00000061823
ENSBTAT00000061824
ENSBTAT00000061826
ENSBTAT00000061827
ENSBTAT00000061828
ENSBTAT00000061829
ENSBTAT00000061830
ENSBTAT00000061831
ENSBTAT00000061833
ENSBTAT00000061834
ENSBTAT00000061835
ENSBTAT00000061838
ENSBTAT00000061839
ENSBTAT00000061840
ENSBTAT00000061842
ENSBTAT00000061843
ENSBTAT00000061845
ENSBTAT00000061846
ENSBTAT00000061847
ENSBTAT00000061850
ENSBTAT00000061851
ENSBTAT00000061852
ENSBTAT00000061853
ENSBTAT00000061855
ENSBTAT00000061858

ENSBTAT00000061859
ENSBTAT00000061860
ENSBTAT00000061861
ENSBTAT00000061862
ENSBTAT00000061863
ENSBTAT00000061865
ENSBTAT00000061866
ENSBTAT00000061869
ENSBTAT00000061871
ENSBTAT00000061873
ENSBTAT00000061874
ENSBTAT00000061875
ENSBTAT00000061876
ENSBTAT00000061877
ENSBTAT00000061879
ENSBTAT00000061880
ENSBTAT00000061882
ENSBTAT00000061883
ENSBTAT00000061884
ENSBTAT00000061885
ENSBTAT00000061886
ENSBTAT00000061887
ENSBTAT00000061889
ENSBTAT00000061890
ENSBTAT00000061891
ENSBTAT00000061892
ENSBTAT00000061894
ENSBTAT00000061895
ENSBTAT00000061897
ENSBTAT00000061898

ENSBTAT00000061899
ENSBTAT00000061900
ENSBTAT00000061902
ENSBTAT00000061903
ENSBTAT00000061906
ENSBTAT00000061907
ENSBTAT00000061908
ENSBTAT00000061909
ENSBTAT00000061910
ENSBTAT00000061911
ENSBTAT00000061913
ENSBTAT00000061914
ENSBTAT00000061915
ENSBTAT00000061916
ENSBTAT00000061918
ENSBTAT00000061920
ENSBTAT00000061922
ENSBTAT00000061923
ENSBTAT00000061924
ENSBTAT00000061925
ENSBTAT00000061926
ENSBTAT00000061928
ENSBTAT00000061929
ENSBTAT00000061930
ENSBTAT00000061931
ENSBTAT00000061932
ENSBTAT00000061933
ENSBTAT00000061934
ENSBTAT00000061935
ENSBTAT00000061936

ENSBTAT00000061937
ENSBTAT00000061939
ENSBTAT00000061940
ENSBTAT00000061941
ENSBTAT00000061942
ENSBTAT00000061943
ENSBTAT00000061945
ENSBTAT00000061946
ENSBTAT00000061947
ENSBTAT00000061948
ENSBTAT00000061950
ENSBTAT00000061951
ENSBTAT00000061952
ENSBTAT00000061953
ENSBTAT00000061954
ENSBTAT00000061955
ENSBTAT00000061956
ENSBTAT00000061957
ENSBTAT00000061958
ENSBTAT00000061959
ENSBTAT00000061961
ENSBTAT00000061963
ENSBTAT00000061965
ENSBTAT00000061968
ENSBTAT00000061969
ENSBTAT00000061970
ENSBTAT00000061971
ENSBTAT00000061972
ENSBTAT00000061973
ENSBTAT00000061974

ENSBTAT00000061976
ENSBTAT00000061977
ENSBTAT00000061978
ENSBTAT00000061979
ENSBTAT00000061980
ENSBTAT00000061981
ENSBTAT00000061983
ENSBTAT00000061984
ENSBTAT00000061985
ENSBTAT00000061986
ENSBTAT00000061987
ENSBTAT00000061988
ENSBTAT00000061990
ENSBTAT00000061991
ENSBTAT00000061993
ENSBTAT00000061994
ENSBTAT00000061996
ENSBTAT00000061997
ENSBTAT00000061998
ENSBTAT00000061999
ENSBTAT00000062000
ENSBTAT00000062001
ENSBTAT00000062003
ENSBTAT00000062004
ENSBTAT00000062005
ENSBTAT00000062006
ENSBTAT00000062007
ENSBTAT00000062008
ENSBTAT00000062009
ENSBTAT00000062011

ENSBTAT00000062012
ENSBTAT00000062013
ENSBTAT00000062014
ENSBTAT00000062015
ENSBTAT00000062016
ENSBTAT00000062017
ENSBTAT00000062018
ENSBTAT00000062019
ENSBTAT00000062020
ENSBTAT00000062021
ENSBTAT00000062022
ENSBTAT00000062023
ENSBTAT00000062024
ENSBTAT00000062025
ENSBTAT00000062026
ENSBTAT00000062027
ENSBTAT00000062028
ENSBTAT00000062030
ENSBTAT00000062031
ENSBTAT00000062032
ENSBTAT00000062033
ENSBTAT00000062034
ENSBTAT00000062035
ENSBTAT00000062036
ENSBTAT00000062037
ENSBTAT00000062039
ENSBTAT00000062040
ENSBTAT00000062041
ENSBTAT00000062044
ENSBTAT00000062047

ENSBTAT00000062048
ENSBTAT00000062049
ENSBTAT00000062050
ENSBTAT00000062051
ENSBTAT00000062052
ENSBTAT00000062053
ENSBTAT00000062054
ENSBTAT00000062055
ENSBTAT00000062056
ENSBTAT00000062058
ENSBTAT00000062059
ENSBTAT00000062061
ENSBTAT00000062063
ENSBTAT00000062064
ENSBTAT00000062065
ENSBTAT00000062066
ENSBTAT00000062068
ENSBTAT00000062069
ENSBTAT00000062070
ENSBTAT00000062073
ENSBTAT00000062075
ENSBTAT00000062076
ENSBTAT00000062078
ENSBTAT00000062079
ENSBTAT00000062082
ENSBTAT00000062084
ENSBTAT00000062086
ENSBTAT00000062087
ENSBTAT00000062090
ENSBTAT00000062091

ENSBTAT00000062092
ENSBTAT00000062093
ENSBTAT00000062094
ENSBTAT00000062096
ENSBTAT00000062097
ENSBTAT00000062098
ENSBTAT00000062099
ENSBTAT00000062100
ENSBTAT00000062101
ENSBTAT00000062102
ENSBTAT00000062103
ENSBTAT00000062104
ENSBTAT00000062106
ENSBTAT00000062107
ENSBTAT00000062108
ENSBTAT00000062109
ENSBTAT00000062110
ENSBTAT00000062111
ENSBTAT00000062112
ENSBTAT00000062113
ENSBTAT00000062117
ENSBTAT00000062120
ENSBTAT00000062121
ENSBTAT00000062122
ENSBTAT00000062123
ENSBTAT00000062124
ENSBTAT00000062127
ENSBTAT00000062128
ENSBTAT00000062129
ENSBTAT00000064391

ENSBTAT00000062131
ENSBTAT00000062132
ENSBTAT00000062133
ENSBTAT00000062135
ENSBTAT00000062136
ENSBTAT00000062137
ENSBTAT00000062139
ENSBTAT00000062141
ENSBTAT00000062142
ENSBTAT00000062144
ENSBTAT00000062145
ENSBTAT00000062146
ENSBTAT00000062147
ENSBTAT00000062148
ENSBTAT00000062149
ENSBTAT00000062150
ENSBTAT00000062152
ENSBTAT00000062153
ENSBTAT00000062154
ENSBTAT00000062155
ENSBTAT00000062156
ENSBTAT00000062157
ENSBTAT00000062159
ENSBTAT00000062160
ENSBTAT00000062163
ENSBTAT00000062165
ENSBTAT00000062166
ENSBTAT00000062167
ENSBTAT00000062168
ENSBTAT00000062169

ENSBTAT00000062170
ENSBTAT00000062171
ENSBTAT00000062173
ENSBTAT00000062174
ENSBTAT00000062175
ENSBTAT00000062176
ENSBTAT00000062177
ENSBTAT00000062178
ENSBTAT00000062179
ENSBTAT00000062180
ENSBTAT00000062181
ENSBTAT00000062184
ENSBTAT00000062185
ENSBTAT00000062186
ENSBTAT00000062187
ENSBTAT00000062189
ENSBTAT00000062190
ENSBTAT00000062191
ENSBTAT00000062192
ENSBTAT00000062193
ENSBTAT00000062197
ENSBTAT00000062199
ENSBTAT00000062200
ENSBTAT00000062202
ENSBTAT00000062203
ENSBTAT00000062204
ENSBTAT00000062206
ENSBTAT00000062207
ENSBTAT00000062208
ENSBTAT00000062209

ENSBTAT00000062210
ENSBTAT00000062211
ENSBTAT00000062212
ENSBTAT00000062213
ENSBTAT00000062214
ENSBTAT00000062215
ENSBTAT00000062217
ENSBTAT00000062218
ENSBTAT00000062219
ENSBTAT00000062220
ENSBTAT00000062221
ENSBTAT00000062222
ENSBTAT00000062223
ENSBTAT00000062224
ENSBTAT00000062225
ENSBTAT00000062226
ENSBTAT00000062227
ENSBTAT00000062228
ENSBTAT00000062229
ENSBTAT00000062230
ENSBTAT00000062232
ENSBTAT00000062233
ENSBTAT00000062234
ENSBTAT00000062235
ENSBTAT00000062236
ENSBTAT00000062238
ENSBTAT00000062240
ENSBTAT00000062241
ENSBTAT00000062242
ENSBTAT00000062243

ENSBTAT00000062244
ENSBTAT00000062245
ENSBTAT00000062248
ENSBTAT00000062249
ENSBTAT00000062250
ENSBTAT00000062252
ENSBTAT00000062254
ENSBTAT00000062256
ENSBTAT00000062257
ENSBTAT00000062259
ENSBTAT00000062263
ENSBTAT00000062264
ENSBTAT00000062265
ENSBTAT00000062266
ENSBTAT00000062268
ENSBTAT00000062270
ENSBTAT00000062271
ENSBTAT00000062273
ENSBTAT00000062275
ENSBTAT00000062276
ENSBTAT00000062277
ENSBTAT00000062278
ENSBTAT00000062279
ENSBTAT00000062280
ENSBTAT00000062281
ENSBTAT00000062282
ENSBTAT00000062283
ENSBTAT00000062284
ENSBTAT00000062287
ENSBTAT00000062288

ENSBTAT00000062289
ENSBTAT00000062292
ENSBTAT00000062294
ENSBTAT00000062295
ENSBTAT00000062297
ENSBTAT00000062298
ENSBTAT00000062300
ENSBTAT00000062301
ENSBTAT00000062302
ENSBTAT00000062303
ENSBTAT00000062304
ENSBTAT00000062305
ENSBTAT00000062306
ENSBTAT00000062307
ENSBTAT00000062308
ENSBTAT00000062309
ENSBTAT00000062310
ENSBTAT00000062312
ENSBTAT00000062313
ENSBTAT00000062314
ENSBTAT00000062315
ENSBTAT00000062316
ENSBTAT00000062317
ENSBTAT00000062318
ENSBTAT00000062319
ENSBTAT00000062320
ENSBTAT00000062321
ENSBTAT00000062322
ENSBTAT00000062323
ENSBTAT00000062324

ENSBTAT00000062325
ENSBTAT00000062326
ENSBTAT00000062327
ENSBTAT00000062329
ENSBTAT00000062330
ENSBTAT00000062331
ENSBTAT00000062332
ENSBTAT00000062333
ENSBTAT00000062334
ENSBTAT00000062336
ENSBTAT00000062337
ENSBTAT00000062338
ENSBTAT00000062339
ENSBTAT00000062340
ENSBTAT00000062344
ENSBTAT00000062345
ENSBTAT00000062346
ENSBTAT00000062347
ENSBTAT00000062348
ENSBTAT00000062349
ENSBTAT00000062351
ENSBTAT00000062353
ENSBTAT00000062354
ENSBTAT00000062355
ENSBTAT00000062356
ENSBTAT00000062358
ENSBTAT00000062359
ENSBTAT00000062361
ENSBTAT00000062362
ENSBTAT00000062363

ENSBTAT00000062364
ENSBTAT00000062365
ENSBTAT00000062366
ENSBTAT00000062367
ENSBTAT00000062368
ENSBTAT00000062371
ENSBTAT00000062373
ENSBTAT00000062375
ENSBTAT00000062376
ENSBTAT00000062377
ENSBTAT00000062379
ENSBTAT00000062381
ENSBTAT00000062383
ENSBTAT00000062384
ENSBTAT00000062385
ENSBTAT00000062386
ENSBTAT00000062387
ENSBTAT00000062388
ENSBTAT00000062389
ENSBTAT00000062390
ENSBTAT00000062392
ENSBTAT00000062395
ENSBTAT00000062396
ENSBTAT00000062398
ENSBTAT00000062399
ENSBTAT00000062400
ENSBTAT00000062401
ENSBTAT00000062403
ENSBTAT00000062404
ENSBTAT00000062405

ENSBTAT00000062406
ENSBTAT00000062408
ENSBTAT00000062409
ENSBTAT00000062410
ENSBTAT00000062411
ENSBTAT00000062413
ENSBTAT00000062414
ENSBTAT00000062415
ENSBTAT00000062416
ENSBTAT00000062417
ENSBTAT00000062418
ENSBTAT00000062419
ENSBTAT00000062420
ENSBTAT00000062421
ENSBTAT00000062422
ENSBTAT00000062423
ENSBTAT00000062426
ENSBTAT00000062431
ENSBTAT00000062432
ENSBTAT00000062434
ENSBTAT00000062435
ENSBTAT00000062436
ENSBTAT00000062437
ENSBTAT00000062438
ENSBTAT00000062441
ENSBTAT00000062442
ENSBTAT00000062443
ENSBTAT00000062445
ENSBTAT00000062447
ENSBTAT00000062448

ENSBTAT00000062449
ENSBTAT00000062450
ENSBTAT00000062451
ENSBTAT00000062453
ENSBTAT00000062454
ENSBTAT00000062455
ENSBTAT00000062456
ENSBTAT00000062457
ENSBTAT00000062458
ENSBTAT00000062459
ENSBTAT00000062460
ENSBTAT00000062462
ENSBTAT00000062463
ENSBTAT00000062464
ENSBTAT00000062465
ENSBTAT00000062466
ENSBTAT00000062469
ENSBTAT00000062471
ENSBTAT00000062472
ENSBTAT00000062473
ENSBTAT00000062475
ENSBTAT00000062476
ENSBTAT00000062477
ENSBTAT00000062478
ENSBTAT00000062479
ENSBTAT00000062480
ENSBTAT00000062481
ENSBTAT00000062482
ENSBTAT00000062484
ENSBTAT00000062485

ENSBTAT00000062487
ENSBTAT00000062488
ENSBTAT00000062489
ENSBTAT00000062490
ENSBTAT00000062491
ENSBTAT00000062492
ENSBTAT00000062493
ENSBTAT00000062494
ENSBTAT00000062495
ENSBTAT00000062496
ENSBTAT00000062497
ENSBTAT00000062498
ENSBTAT00000062500
ENSBTAT00000062501
ENSBTAT00000062504
ENSBTAT00000062505
ENSBTAT00000062506
ENSBTAT00000062507
ENSBTAT00000062508
ENSBTAT00000062509
ENSBTAT00000062510
ENSBTAT00000062511
ENSBTAT00000062512
ENSBTAT00000062513
ENSBTAT00000062514
ENSBTAT00000062515
ENSBTAT00000062516
ENSBTAT00000062517
ENSBTAT00000062518
ENSBTAT00000062520

ENSBTAT00000062522
ENSBTAT00000062524
ENSBTAT00000062525
ENSBTAT00000062527
ENSBTAT00000062528
ENSBTAT00000062529
ENSBTAT00000062530
ENSBTAT00000062531
ENSBTAT00000062532
ENSBTAT00000062533
ENSBTAT00000062534
ENSBTAT00000062535
ENSBTAT00000062536
ENSBTAT00000062537
ENSBTAT00000062538
ENSBTAT00000062539
ENSBTAT00000062540
ENSBTAT00000062541
ENSBTAT00000062544
ENSBTAT00000062545
ENSBTAT00000062546
ENSBTAT00000062547
ENSBTAT00000062548
ENSBTAT00000062549
ENSBTAT00000062550
ENSBTAT00000062551
ENSBTAT00000062552
ENSBTAT00000062553
ENSBTAT00000062554
ENSBTAT00000062556

ENSBTAT00000062557
ENSBTAT00000062558
ENSBTAT00000062562
ENSBTAT00000062563
ENSBTAT00000062564
ENSBTAT00000062565
ENSBTAT00000062566
ENSBTAT00000062567
ENSBTAT00000062568
ENSBTAT00000062570
ENSBTAT00000062571
ENSBTAT00000062572
ENSBTAT00000062573
ENSBTAT00000062574
ENSBTAT00000062575
ENSBTAT00000062577
ENSBTAT00000062579
ENSBTAT00000062580
ENSBTAT00000062581
ENSBTAT00000062582
ENSBTAT00000062583
ENSBTAT00000062585
ENSBTAT00000062586
ENSBTAT00000062587
ENSBTAT00000062589
ENSBTAT00000062590
ENSBTAT00000062591
ENSBTAT00000062592
ENSBTAT00000062594
ENSBTAT00000062595

ENSBTAT00000062596
ENSBTAT00000062597
ENSBTAT00000062599
ENSBTAT00000062600
ENSBTAT00000062601
ENSBTAT00000062602
ENSBTAT00000062603
ENSBTAT00000062604
ENSBTAT00000062605
ENSBTAT00000062606
ENSBTAT00000062607
ENSBTAT00000062609
ENSBTAT00000062611
ENSBTAT00000062612
ENSBTAT00000062613
ENSBTAT00000062614
ENSBTAT00000062616
ENSBTAT00000062617
ENSBTAT00000062619
ENSBTAT00000062620
ENSBTAT00000062621
ENSBTAT00000062622
ENSBTAT00000062624
ENSBTAT00000062626
ENSBTAT00000062627
ENSBTAT00000062628
ENSBTAT00000062629
ENSBTAT00000062630
ENSBTAT00000062632
ENSBTAT00000062633

ENSBTAT00000062634
ENSBTAT00000062635
ENSBTAT00000062636
ENSBTAT00000062639
ENSBTAT00000062640
ENSBTAT00000062642
ENSBTAT00000062643
ENSBTAT00000062647
ENSBTAT00000062648
ENSBTAT00000062649
ENSBTAT00000062650
ENSBTAT00000062651
ENSBTAT00000062652
ENSBTAT00000062653
ENSBTAT00000062654
ENSBTAT00000062655
ENSBTAT00000062656
ENSBTAT00000062657
ENSBTAT00000062658
ENSBTAT00000062659
ENSBTAT00000062661
ENSBTAT00000062663
ENSBTAT00000062664
ENSBTAT00000062666
ENSBTAT00000062667
ENSBTAT00000062668
ENSBTAT00000062669
ENSBTAT00000062671
ENSBTAT00000062672
ENSBTAT00000062673

ENSBTAT00000062675
ENSBTAT00000062676
ENSBTAT00000062677
ENSBTAT00000062679
ENSBTAT00000062680
ENSBTAT00000062681
ENSBTAT00000062682
ENSBTAT00000062683
ENSBTAT00000062684
ENSBTAT00000062685
ENSBTAT00000062686
ENSBTAT00000062688
ENSBTAT00000062689
ENSBTAT00000062691
ENSBTAT00000062693
ENSBTAT00000062694
ENSBTAT00000062695
ENSBTAT00000062696
ENSBTAT00000062697
ENSBTAT00000062698
ENSBTAT00000062701
ENSBTAT00000062702
ENSBTAT00000062703
ENSBTAT00000062704
ENSBTAT00000062705
ENSBTAT00000062709
ENSBTAT00000062710
ENSBTAT00000062712
ENSBTAT00000062713
ENSBTAT00000062714

ENSBTAT00000062715
ENSBTAT00000062716
ENSBTAT00000062717
ENSBTAT00000062718
ENSBTAT00000062719
ENSBTAT00000062720
ENSBTAT00000062722
ENSBTAT00000062723
ENSBTAT00000062724
ENSBTAT00000062725
ENSBTAT00000062726
ENSBTAT00000062727
ENSBTAT00000062730
ENSBTAT00000062732
ENSBTAT00000062733
ENSBTAT00000062734
ENSBTAT00000062736
ENSBTAT00000062737
ENSBTAT00000062738
ENSBTAT00000062739
ENSBTAT00000062740
ENSBTAT00000062741
ENSBTAT00000062742
ENSBTAT00000062743
ENSBTAT00000062745
ENSBTAT00000062746
ENSBTAT00000062747
ENSBTAT00000062749
ENSBTAT00000062750
ENSBTAT00000062751

ENSBTAT00000062752
ENSBTAT00000062753
ENSBTAT00000062754
ENSBTAT00000062755
ENSBTAT00000062756
ENSBTAT00000062757
ENSBTAT00000062758
ENSBTAT00000062759
ENSBTAT00000062760
ENSBTAT00000062761
ENSBTAT00000062762
ENSBTAT00000062763
ENSBTAT00000062764
ENSBTAT00000062765
ENSBTAT00000062766
ENSBTAT00000062768
ENSBTAT00000062770
ENSBTAT00000062771
ENSBTAT00000062772
ENSBTAT00000062773
ENSBTAT00000062774
ENSBTAT00000062776
ENSBTAT00000062777
ENSBTAT00000062778
ENSBTAT00000062780
ENSBTAT00000062782
ENSBTAT00000062783
ENSBTAT00000062784
ENSBTAT00000062787
ENSBTAT00000062788

ENSBTAT00000062791
ENSBTAT00000062792
ENSBTAT00000062793
ENSBTAT00000062794
ENSBTAT00000062796
ENSBTAT00000062797
ENSBTAT00000062799
ENSBTAT00000062800
ENSBTAT00000062801
ENSBTAT00000062802
ENSBTAT00000062803
ENSBTAT00000062804
ENSBTAT00000062805
ENSBTAT00000062806
ENSBTAT00000062808
ENSBTAT00000062809
ENSBTAT00000062810
ENSBTAT00000062812
ENSBTAT00000062813
ENSBTAT00000062815
ENSBTAT00000062816
ENSBTAT00000062817
ENSBTAT00000062818
ENSBTAT00000062819
ENSBTAT00000062820
ENSBTAT00000062822
ENSBTAT00000062823
ENSBTAT00000062824
ENSBTAT00000062825
ENSBTAT00000062826

ENSBTAT00000062827
ENSBTAT00000062828
ENSBTAT00000062829
ENSBTAT00000062830
ENSBTAT00000062831
ENSBTAT00000062832
ENSBTAT00000062833
ENSBTAT00000062834
ENSBTAT00000062835
ENSBTAT00000062836
ENSBTAT00000062837
ENSBTAT00000062838
ENSBTAT00000062839
ENSBTAT00000062840
ENSBTAT00000062841
ENSBTAT00000062842
ENSBTAT00000062843
ENSBTAT00000062844
ENSBTAT00000062846
ENSBTAT00000062847
ENSBTAT00000062848
ENSBTAT00000062849
ENSBTAT00000062850
ENSBTAT00000062851
ENSBTAT00000062852
ENSBTAT00000062854
ENSBTAT00000062855
ENSBTAT00000062856
ENSBTAT00000062857
ENSBTAT00000062858

ENSBTAT00000062859
ENSBTAT00000062861
ENSBTAT00000062862
ENSBTAT00000062863
ENSBTAT00000062864
ENSBTAT00000062867
ENSBTAT00000062868
ENSBTAT00000062869
ENSBTAT00000062870
ENSBTAT00000062871
ENSBTAT00000062872
ENSBTAT00000062873
ENSBTAT00000062875
ENSBTAT00000062876
ENSBTAT00000062877
ENSBTAT00000062878
ENSBTAT00000062881
ENSBTAT00000062882
ENSBTAT00000062883
ENSBTAT00000062884
ENSBTAT00000062886
ENSBTAT00000062887
ENSBTAT00000062888
ENSBTAT00000062889
ENSBTAT00000062891
ENSBTAT00000062892
ENSBTAT00000062893
ENSBTAT00000062894
ENSBTAT00000062895
ENSBTAT00000062896

ENSBTAT00000062897
ENSBTAT00000062898
ENSBTAT00000062899
ENSBTAT00000062901
ENSBTAT00000062902
ENSBTAT00000062903
ENSBTAT00000062904
ENSBTAT00000062905
ENSBTAT00000062906
ENSBTAT00000062907
ENSBTAT00000062908
ENSBTAT00000062910
ENSBTAT00000062911
ENSBTAT00000062912
ENSBTAT00000062913
ENSBTAT00000062914
ENSBTAT00000062915
ENSBTAT00000062917
ENSBTAT00000062918
ENSBTAT00000062919
ENSBTAT00000062931
ENSBTAT00000063024
ENSBTAT00000065955
ENSBTAT00000064425
ENSBTAT00000063034
ENSBTAT00000064646
ENSBTAT00000065097
ENSBTAT00000063187
ENSBTAT00000065180
ENSBTAT00000063257

ENSBTAT00000062943
ENSBTAT00000063866
ENSBTAT00000063920
ENSBTAT00000063947
ENSBTAT00000062983
ENSBTAT00000063251
ENSBTAT00000063689
ENSBTAT00000064447
ENSBTAT00000062916
ENSBTAT00000066217
ENSBTAT00000063212
ENSBTAT00000065902
ENSBTAT00000065528
ENSBTAT00000065535
ENSBTAT00000064846
ENSBTAT00000065726
ENSBTAT00000066228
ENSBTAT00000062662
ENSBTAT00000063173
ENSBTAT00000005676
ENSBTAT00000064273
ENSBTAT00000063410
ENSBTAT00000063725
ENSBTAT00000064216
ENSBTAT00000064775
ENSBTAT00000066024
ENSBTAT00000063637
ENSBTAT00000064395
ENSBTAT00000064721
ENSBTAT00000065947

ENSBTAT00000063706
ENSBTAT00000052333
ENSBTAT00000065064
ENSBTAT00000065127
ENSBTAT00000064379
ENSBTAT00000065572
ENSBTAT00000064401
ENSBTAT00000064256
ENSBTAT00000064308
ENSBTAT00000065815
ENSBTAT00000063281
ENSBTAT00000063925
ENSBTAT00000065639
ENSBTAT00000066014
ENSBTAT00000040570
ENSBTAT00000064599
ENSBTAT00000063476
ENSBTAT00000065785
ENSBTAT00000064300
ENSBTAT00000064408
ENSBTAT00000064762
ENSBTAT00000063799
ENSBTAT00000066010
ENSBTAT00000065307
ENSBTAT00000064694
ENSBTAT00000063816
ENSBTAT00000065718
ENSBTAT00000066004
ENSBTAT00000063158
ENSBTAT00000064453

ENSBTAT00000062986
ENSBTAT00000051401
ENSBTAT00000063663
ENSBTAT00000065724
ENSBTAT00000064191
ENSBTAT00000066309
ENSBTAT00000060014
ENSBTAT00000065188
ENSBTAT00000063980
ENSBTAT00000065289
ENSBTAT00000064338
ENSBTAT00000065414
ENSBTAT00000053918
ENSBTAT00000045557
ENSBTAT00000063940
ENSBTAT00000059213
ENSBTAT00000063136
ENSBTAT00000064439
ENSBTAT00000063101
ENSBTAT00000064285
ENSBTAT00000065023
ENSBTAT00000049993
ENSBTAT00000064086
ENSBTAT00000065339
ENSBTAT00000016972
ENSBTAT00000065589
ENSBTAT00000064739
ENSBTAT00000065778
ENSBTAT00000063026
ENSBTAT00000056267

ENSBTAT00000063360
ENSBTAT00000012687
ENSBTAT00000063890
ENSBTAT00000066232
ENSBTAT00000063347
ENSBTAT00000064512
ENSBTAT00000057342
ENSBTAT00000049212
ENSBTAT00000063208
ENSBTAT00000064616
ENSBTAT00000066216
ENSBTAT00000064477
ENSBTAT00000062948
ENSBTAT00000065356
ENSBTAT00000065168
ENSBTAT00000066064
ENSBTAT00000066001
ENSBTAT00000064232
ENSBTAT00000065764
ENSBTAT00000064260
ENSBTAT00000064818
ENSBTAT00000062700
ENSBTAT00000066276
ENSBTAT00000064607
ENSBTAT00000064790
ENSBTAT00000063956
ENSBTAT00000064805
ENSBTAT00000065765
ENSBTAT00000065440
ENSBTAT00000063807

ENSBTAT00000065325
ENSBTAT00000062588
ENSBTAT00000063066
ENSBTAT00000053805
ENSBTAT00000064770
ENSBTAT00000065095
ENSBTAT00000062785
ENSBTAT00000064564
ENSBTAT00000063958
ENSBTAT00000065340
ENSBTAT00000063972
ENSBTAT00000065517
ENSBTAT00000065046
ENSBTAT00000064061
ENSBTAT00000066049
ENSBTAT00000065250
ENSBTAT00000065137
ENSBTAT00000066120
ENSBTAT00000065838
ENSBTAT00000064157
ENSBTAT00000018767
ENSBTAT00000064312
ENSBTAT00000064661
ENSBTAT00000062950
ENSBTAT00000050719
ENSBTAT00000059562
ENSBTAT00000066247
ENSBTAT00000064310
ENSBTAT00000065649
ENSBTAT00000064022

ENSBTAT00000065949
ENSBTAT00000062987
ENSBTAT00000066181
ENSBTAT00000064624
ENSBTAT00000063148
ENSBTAT00000031648
ENSBTAT00000063616
ENSBTAT00000064811
ENSBTAT00000063648
ENSBTAT00000065382
ENSBTAT00000063267
ENSBTAT00000051747
ENSBTAT00000064470
ENSBTAT00000063053
ENSBTAT00000063172
ENSBTAT00000063213
ENSBTAT00000065442
ENSBTAT00000063327
ENSBTAT00000064993
ENSBTAT00000064870
ENSBTAT00000065920
ENSBTAT00000064839
ENSBTAT00000065217
ENSBTAT00000063722
ENSBTAT00000065592
ENSBTAT00000063752
ENSBTAT00000063992
ENSBTAT00000065781
ENSBTAT00000063038
ENSBTAT00000063379

ENSBTAT00000065763
ENSBTAT00000063295
ENSBTAT00000064474
ENSBTAT00000063109
ENSBTAT00000065420
ENSBTAT00000063288
ENSBTAT00000065685
ENSBTAT00000064768
ENSBTAT00000064016
ENSBTAT00000066238
ENSBTAT00000066092
ENSBTAT00000065905
ENSBTAT00000064522
ENSBTAT00000063662
ENSBTAT00000064546
ENSBTAT00000065669
ENSBTAT00000063355
ENSBTAT00000063064
ENSBTAT00000063532
ENSBTAT00000066069
ENSBTAT00000064121
ENSBTAT00000063117
ENSBTAT00000064697
ENSBTAT00000062984
ENSBTAT00000065968
ENSBTAT00000065000
ENSBTAT00000065837
ENSBTAT00000066015
ENSBTAT00000065265
ENSBTAT00000064415

ENSBTAT00000064821
ENSBTAT00000062920
ENSBTAT00000054544
ENSBTAT00000063819
ENSBTAT00000064754
ENSBTAT00000066007
ENSBTAT00000065544
ENSBTAT00000065321
ENSBTAT00000063153
ENSBTAT00000065558
ENSBTAT00000064881
ENSBTAT00000065347
ENSBTAT00000064074
ENSBTAT00000063075
ENSBTAT00000063864
ENSBTAT00000065630
ENSBTAT00000064533
ENSBTAT00000065845
ENSBTAT00000066208
ENSBTAT00000065962
ENSBTAT00000065934
ENSBTAT00000066264
ENSBTAT00000063229
ENSBTAT00000063524
ENSBTAT00000063735
ENSBTAT00000066223
ENSBTAT00000065573
ENSBTAT00000065645
ENSBTAT00000064103
ENSBTAT00000066180

ENSBTAT00000053282
ENSBTAT00000065467
ENSBTAT00000063341
ENSBTAT00000065015
ENSBTAT00000064323
ENSBTAT00000063554
ENSBTAT00000066119
ENSBTAT00000052245
ENSBTAT00000065335
ENSBTAT00000064618
ENSBTAT00000065628
ENSBTAT00000064494
ENSBTAT00000062924
ENSBTAT00000065230
ENSBTAT00000034857
ENSBTAT00000064977
ENSBTAT00000064328
ENSBTAT00000063181
ENSBTAT00000063335
ENSBTAT00000065873
ENSBTAT00000066262
ENSBTAT00000065519
ENSBTAT00000065661
ENSBTAT00000063023
ENSBTAT00000065504
ENSBTAT00000064939
ENSBTAT00000064418
ENSBTAT00000054265
ENSBTAT00000005026
ENSBTAT00000063191

ENSBTAT00000064815
ENSBTAT00000066039
ENSBTAT00000064267
ENSBTAT00000064454
ENSBTAT00000064090
ENSBTAT00000064783
ENSBTAT00000066114
ENSBTAT00000063726
ENSBTAT00000064713
ENSBTAT00000062953
ENSBTAT00000063471
ENSBTAT00000064866
ENSBTAT00000063461
ENSBTAT00000066210
ENSBTAT00000066260
ENSBTAT00000063542
ENSBTAT00000063393
ENSBTAT00000055186
ENSBTAT00000064986
ENSBTAT00000065234
ENSBTAT00000066083
ENSBTAT00000064435
ENSBTAT00000066123
ENSBTAT00000065093
ENSBTAT00000064166
ENSBTAT00000059667
ENSBTAT00000064185
ENSBTAT00000064276
ENSBTAT00000060681
ENSBTAT00000065079

ENSBTAT00000063357
ENSBTAT00000063651
ENSBTAT00000065310
ENSBTAT00000062930
ENSBTAT00000063937
ENSBTAT00000065001
ENSBTAT00000064457
ENSBTAT00000065191
ENSBTAT00000064663
ENSBTAT00000063527
ENSBTAT00000062433
ENSBTAT00000064906
ENSBTAT00000064210
ENSBTAT00000064284
ENSBTAT00000044064
ENSBTAT00000063861
ENSBTAT00000063655
ENSBTAT00000063310
ENSBTAT00000066097
ENSBTAT00000049925
ENSBTAT00000051767
ENSBTAT00000064497
ENSBTAT00000065810
ENSBTAT00000063442
ENSBTAT00000001559
ENSBTAT00000065840
ENSBTAT00000064405
ENSBTAT00000064642
ENSBTAT00000063227
ENSBTAT00000063200

ENSBTAT00000065202
ENSBTAT00000064934
ENSBTAT00000064804
ENSBTAT00000063826
ENSBTAT00000063580
ENSBTAT00000063633
ENSBTAT00000063168
ENSBTAT00000066283
ENSBTAT00000064743
ENSBTAT00000066057
ENSBTAT00000064979
ENSBTAT00000063851
ENSBTAT00000063086
ENSBTAT00000065563
ENSBTAT00000015486
ENSBTAT00000065739
ENSBTAT00000063263
ENSBTAT00000063236
ENSBTAT00000053898
ENSBTAT00000065588
ENSBTAT00000065554
ENSBTAT00000063313
ENSBTAT00000065374
ENSBTAT00000063323
ENSBTAT00000063042
ENSBTAT00000064352
ENSBTAT00000062941
ENSBTAT00000065802
ENSBTAT00000064482
ENSBTAT00000063129

ENSBTAT00000064337
ENSBTAT00000064257
ENSBTAT00000064527
ENSBTAT00000036339
ENSBTAT00000065951
ENSBTAT00000063977
ENSBTAT00000065663
ENSBTAT00000063973
ENSBTAT00000062938
ENSBTAT00000065604
ENSBTAT00000064797
ENSBTAT00000065751
ENSBTAT00000064353
ENSBTAT00000064865
ENSBTAT00000064980
ENSBTAT00000064648
ENSBTAT00000065710
ENSBTAT00000065721
ENSBTAT00000051944
ENSBTAT00000065811
ENSBTAT00000066265
ENSBTAT00000065975
ENSBTAT00000065306
ENSBTAT00000011370
ENSBTAT00000066203
ENSBTAT00000063619
ENSBTAT00000065748
ENSBTAT00000064733
ENSBTAT00000064019
ENSBTAT00000066272

ENSBTAT00000063632
ENSBTAT00000063345
ENSBTAT00000065466
ENSBTAT00000065197
ENSBTAT00000063289
ENSBTAT00000063170
ENSBTAT00000065515
ENSBTAT00000064388
ENSBTAT00000064248
ENSBTAT00000066061
ENSBTAT00000065564
ENSBTAT00000063237
ENSBTAT00000064125
ENSBTAT00000065577
ENSBTAT00000065512
ENSBTAT00000065686
ENSBTAT00000064514
ENSBTAT00000063220
ENSBTAT00000006472
ENSBTAT00000066277
ENSBTAT00000065892
ENSBTAT00000064209
ENSBTAT00000018344
ENSBTAT00000065320
ENSBTAT00000062949
ENSBTAT00000052309
ENSBTAT00000064962
ENSBTAT00000065818
ENSBTAT00000063142
ENSBTAT00000063096

ENSBTAT00000063630
ENSBTAT00000063960
ENSBTAT00000065170
ENSBTAT00000064160
ENSBTAT00000065910
ENSBTAT00000064593
ENSBTAT00000051978
ENSBTAT00000064621
ENSBTAT00000065236
ENSBTAT00000065719
ENSBTAT00000063800
ENSBTAT00000063285
ENSBTAT00000063186
ENSBTAT00000046511
ENSBTAT00000063840
ENSBTAT00000063549
ENSBTAT00000065605
ENSBTAT00000063050
ENSBTAT00000063832
ENSBTAT00000064989
ENSBTAT00000044261
ENSBTAT00000064719
ENSBTAT00000062995
ENSBTAT00000065110
ENSBTAT00000063860
ENSBTAT00000064041
ENSBTAT00000063959
ENSBTAT00000064100
ENSBTAT00000065219
ENSBTAT00000064238

ENSBTAT00000065071
ENSBTAT00000008318
ENSBTAT00000065872
ENSBTAT00000064380
ENSBTAT00000065502
ENSBTAT00000066289
ENSBTAT00000063653
ENSBTAT00000066337
ENSBTAT00000047733
ENSBTAT00000066341
ENSBTAT00000064738
ENSBTAT00000064247
ENSBTAT00000064589
ENSBTAT00000065959
ENSBTAT00000065757
ENSBTAT00000065063
ENSBTAT00000064972
ENSBTAT00000063111
ENSBTAT00000037859
ENSBTAT00000063880
ENSBTAT00000065790
ENSBTAT00000063573
ENSBTAT00000052770
ENSBTAT00000065298
ENSBTAT00000066009
ENSBTAT00000065448
ENSBTAT00000064193
ENSBTAT00000063297
ENSBTAT00000064333
ENSBTAT00000066321

ENSBTAT00000066136
ENSBTAT00000050486
ENSBTAT00000064348
ENSBTAT00000063859
ENSBTAT00000066088
ENSBTAT00000063443
ENSBTAT00000052141
ENSBTAT00000065437
ENSBTAT00000059951
ENSBTAT00000065709
ENSBTAT00000065536
ENSBTAT00000049536
ENSBTAT00000065744
ENSBTAT00000063517
ENSBTAT00000045246
ENSBTAT00000064562
ENSBTAT00000065985
ENSBTAT00000065874
ENSBTAT00000065714
ENSBTAT00000063784
ENSBTAT00000063097
ENSBTAT00000054467
ENSBTAT00000063950
ENSBTAT00000044015
ENSBTAT00000063511
ENSBTAT00000065889
ENSBTAT00000063744
ENSBTAT00000063292
ENSBTAT00000064199
ENSBTAT00000065679

ENSBTAT00000065295
ENSBTAT00000063942
ENSBTAT00000054704
ENSBTAT00000066142
ENSBTAT00000065697
ENSBTAT00000063405
ENSBTAT00000066194
ENSBTAT00000063801
ENSBTAT00000065954
ENSBTAT00000065172
ENSBTAT00000065169
ENSBTAT00000064707
ENSBTAT00000065789
ENSBTAT00000063754
ENSBTAT00000064361
ENSBTAT00000063787
ENSBTAT00000063299
ENSBTAT00000056628
ENSBTAT00000065850
ENSBTAT00000065291
ENSBTAT00000017034
ENSBTAT00000064476
ENSBTAT00000063346
ENSBTAT00000063389
ENSBTAT00000063421
ENSBTAT00000064778
ENSBTAT00000004235
ENSBTAT00000049823
ENSBTAT00000063610
ENSBTAT00000065998

ENSBTAT00000065399
ENSBTAT00000063822
ENSBTAT00000063857
ENSBTAT00000063454
ENSBTAT00000064649
ENSBTAT00000064771
ENSBTAT00000063377
ENSBTAT00000066085
ENSBTAT00000064871
ENSBTAT00000065140
ENSBTAT00000064544
ENSBTAT00000066190
ENSBTAT00000064365
ENSBTAT00000064854
ENSBTAT00000063155
ENSBTAT00000064613
ENSBTAT00000063900
ENSBTAT00000064676
ENSBTAT00000045063
ENSBTAT00000064538
ENSBTAT00000063882
ENSBTAT00000065658
ENSBTAT00000063184
ENSBTAT00000063413
ENSBTAT00000065060
ENSBTAT00000064035
ENSBTAT00000064038
ENSBTAT00000062526
ENSBTAT00000059724
ENSBTAT00000064794

ENSBTAT00000063518
ENSBTAT00000063446
ENSBTAT00000065878
ENSBTAT00000063080
ENSBTAT00000063530
ENSBTAT00000064978
ENSBTAT00000063845
ENSBTAT00000066125
ENSBTAT00000059967
ENSBTAT00000064265
ENSBTAT00000065016
ENSBTAT00000065471
ENSBTAT00000064987
ENSBTAT00000064330
ENSBTAT00000063594
ENSBTAT00000063450
ENSBTAT00000064236
ENSBTAT00000063592
ENSBTAT00000063073
ENSBTAT00000063366
ENSBTAT00000065992
ENSBTAT00000065609
ENSBTAT00000065484
ENSBTAT00000063556
ENSBTAT00000064625
ENSBTAT00000063774
ENSBTAT00000063779
ENSBTAT00000064346
ENSBTAT00000065974
ENSBTAT00000064487

ENSBTAT00000063608
ENSBTAT00000064960
ENSBTAT00000027399
ENSBTAT00000064068
ENSBTAT00000052553
ENSBTAT00000063636
ENSBTAT00000063241
ENSBTAT00000064150
ENSBTAT00000050159
ENSBTAT00000065834
ENSBTAT00000065017
ENSBTAT00000063234
ENSBTAT00000010593
ENSBTAT00000065981
ENSBTAT00000064117
ENSBTAT00000061967
ENSBTAT00000065024
ENSBTAT00000064970
ENSBTAT00000065652
ENSBTAT00000063037
ENSBTAT00000049548
ENSBTAT00000050480
ENSBTAT00000065885
ENSBTAT00000063563
ENSBTAT00000065644
ENSBTAT00000065833
ENSBTAT00000065527
ENSBTAT00000066058
ENSBTAT00000064650
ENSBTAT00000064446

ENSBTAT00000016457
ENSBTAT00000065792
ENSBTAT00000064664
ENSBTAT00000063358
ENSBTAT00000010642
ENSBTAT00000064933
ENSBTAT00000065293
ENSBTAT00000063160
ENSBTAT00000053848
ENSBTAT00000056709
ENSBTAT00000066034
ENSBTAT00000066153
ENSBTAT00000063849
ENSBTAT00000062926
ENSBTAT00000065481
ENSBTAT00000063223
ENSBTAT00000066154
ENSBTAT00000064896
ENSBTAT00000065423
ENSBTAT00000065495
ENSBTAT00000064793
ENSBTAT00000066008
ENSBTAT00000065960
ENSBTAT00000065038
ENSBTAT00000063680
ENSBTAT00000065389
ENSBTAT00000063400
ENSBTAT00000064534
ENSBTAT00000063996
ENSBTAT00000064266

ENSBTAT00000064255
ENSBTAT00000064069
ENSBTAT00000065797
ENSBTAT00000065042
ENSBTAT00000063818
ENSBTAT00000065659
ENSBTAT00000063167
ENSBTAT00000064983
ENSBTAT00000063713
ENSBTAT00000062989
ENSBTAT00000065200
ENSBTAT00000054757
ENSBTAT00000065804
ENSBTAT00000063548
ENSBTAT00000065762
ENSBTAT00000063178
ENSBTAT00000065449
ENSBTAT00000066338
ENSBTAT00000064491
ENSBTAT00000063869
ENSBTAT00000064413
ENSBTAT00000064115
ENSBTAT00000065183
ENSBTAT00000064382
ENSBTAT00000064940
ENSBTAT00000063865
ENSBTAT00000064424
ENSBTAT00000063264
ENSBTAT00000066278
ENSBTAT00000066302

ENSBTAT00000063466
ENSBTAT00000066176
ENSBTAT00000064198
ENSBTAT00000063783
ENSBTAT00000066250
ENSBTAT00000063896
ENSBTAT00000010109
ENSBTAT00000020279
ENSBTAT00000054368
ENSBTAT00000063049
ENSBTAT00000056009
ENSBTAT00000063526
ENSBTAT00000062959
ENSBTAT00000065225
ENSBTAT00000064020
ENSBTAT00000065851
ENSBTAT00000063650
ENSBTAT00000030915
ENSBTAT00000065775
ENSBTAT00000065122
ENSBTAT00000064832
ENSBTAT00000062342
ENSBTAT00000036975
ENSBTAT00000065525
ENSBTAT00000064128
ENSBTAT00000059254
ENSBTAT00000066333
ENSBTAT00000064133
ENSBTAT00000063368
ENSBTAT00000065808

ENSBTAT00000065468
ENSBTAT00000063966
ENSBTAT00000064579
ENSBTAT00000065454
ENSBTAT00000062962
ENSBTAT00000065761
ENSBTAT00000065068
ENSBTAT00000063427
ENSBTAT00000065344
ENSBTAT00000063673
ENSBTAT00000063499
ENSBTAT00000063629
ENSBTAT00000064683
ENSBTAT00000065215
ENSBTAT00000063364
ENSBTAT00000064765
ENSBTAT00000063135
ENSBTAT00000065415
ENSBTAT00000044257
ENSBTAT00000064598
ENSBTAT00000054415
ENSBTAT00000063093
ENSBTAT00000065089
ENSBTAT00000063734
ENSBTAT00000065231
ENSBTAT00000063669
ENSBTAT00000064769
ENSBTAT00000064178
ENSBTAT00000051760
ENSBTAT00000066074

ENSBTAT00000066237
ENSBTAT00000066148
ENSBTAT00000063990
ENSBTAT00000064220
ENSBTAT00000064280
ENSBTAT00000063814
ENSBTAT00000066342
ENSBTAT00000064196
ENSBTAT00000063970
ENSBTAT00000063044
ENSBTAT00000063055
ENSBTAT00000064717
ENSBTAT00000063260
ENSBTAT00000065316
ENSBTAT00000065777
ENSBTAT00000038922
ENSBTAT00000065508
ENSBTAT00000063886
ENSBTAT00000064342
ENSBTAT00000063910
ENSBTAT00000064729
ENSBTAT00000065915
ENSBTAT00000009854
ENSBTAT00000066239
ENSBTAT00000064622
ENSBTAT00000064015
ENSBTAT00000063348
ENSBTAT00000065087
ENSBTAT00000065666
ENSBTAT00000064009

ENSBTAT00000066091
ENSBTAT00000063782
ENSBTAT00000065436
ENSBTAT00000065654
ENSBTAT00000063273
ENSBTAT00000027317
ENSBTAT00000057090
ENSBTAT00000063733
ENSBTAT00000064177
ENSBTAT00000064350
ENSBTAT00000065227
ENSBTAT00000063072
ENSBTAT00000059717
ENSBTAT00000065832
ENSBTAT00000065594
ENSBTAT00000038957
ENSBTAT00000065102
ENSBTAT00000064281
ENSBTAT00000063719
ENSBTAT00000066245
ENSBTAT00000043352
ENSBTAT00000063897
ENSBTAT00000063005
ENSBTAT00000066320
ENSBTAT00000063691
ENSBTAT00000064235
ENSBTAT00000051821
ENSBTAT00000064925
ENSBTAT00000065050
ENSBTAT00000063385

ENSBTAT00000065081
ENSBTAT00000053887
ENSBTAT00000065704
ENSBTAT00000063731
ENSBTAT00000063709
ENSBTAT00000065022
ENSBTAT00000064710
ENSBTAT00000064113
ENSBTAT00000065884
ENSBTAT00000065410
ENSBTAT00000065133
ENSBTAT00000063123
ENSBTAT00000064286
ENSBTAT00000066303
ENSBTAT00000065737
ENSBTAT00000064985
ENSBTAT00000065540
ENSBTAT00000052269
ENSBTAT00000065128
ENSBTAT00000064943
ENSBTAT00000063211
ENSBTAT00000066288
ENSBTAT00000063729
ENSBTAT00000049516
ENSBTAT00000051803
ENSBTAT00000065603
ENSBTAT00000064894
ENSBTAT00000064658
ENSBTAT00000049451
ENSBTAT00000065700

ENSBTAT00000066215
ENSBTAT00000064459
ENSBTAT00000066298
ENSBTAT00000063791
ENSBTAT00000063381
ENSBTAT00000062964
ENSBTAT00000065733
ENSBTAT00000063108
ENSBTAT00000064685
ENSBTAT00000064796
ENSBTAT00000062958
ENSBTAT00000054037
ENSBTAT00000034045
ENSBTAT00000065922
ENSBTAT00000065924
ENSBTAT00000064175
ENSBTAT00000031459
ENSBTAT00000066116
ENSBTAT00000063332
ENSBTAT00000015816
ENSBTAT00000064590
ENSBTAT00000064750
ENSBTAT00000066168
ENSBTAT00000063601
ENSBTAT00000064831
ENSBTAT00000064550
ENSBTAT00000063436
ENSBTAT00000048459
ENSBTAT00000063528
ENSBTAT00000044102

ENSBTAT00000064298
ENSBTAT00000064689
ENSBTAT00000064212
ENSBTAT00000063658
ENSBTAT00000064584
ENSBTAT00000065977
ENSBTAT00000063600
ENSBTAT00000063987
ENSBTAT00000064024
ENSBTAT00000064702
ENSBTAT00000064192
ENSBTAT00000065048
ENSBTAT00000040482
ENSBTAT00000063589
ENSBTAT00000065871
ENSBTAT00000066027
ENSBTAT00000064094
ENSBTAT00000048804
ENSBTAT00000065482
ENSBTAT00000066311
ENSBTAT00000063750
ENSBTAT00000063137
ENSBTAT00000065791
ENSBTAT00000066060
ENSBTAT00000063014
ENSBTAT00000063369
ENSBTAT00000064449
ENSBTAT00000064274
ENSBTAT00000064973
ENSBTAT00000063502

ENSBTAT00000066182
ENSBTAT00000002318
ENSBTAT00000063498
ENSBTAT00000063000
ENSBTAT00000065094
ENSBTAT00000043502
ENSBTAT00000063745
ENSBTAT00000064028
ENSBTAT00000065831
ENSBTAT00000063248
ENSBTAT00000063221
ENSBTAT00000064448
ENSBTAT00000063041
ENSBTAT00000064853
ENSBTAT00000064200
ENSBTAT00000063016
ENSBTAT00000055184
ENSBTAT00000057360
ENSBTAT00000065318
ENSBTAT00000063679
ENSBTAT00000065638
ENSBTAT00000065193
ENSBTAT00000064067
ENSBTAT00000065914
ENSBTAT00000063134
ENSBTAT00000064264
ENSBTAT00000065746
ENSBTAT00000063903
ENSBTAT00000063025
ENSBTAT00000006176

ENSBTAT00000046574
ENSBTAT00000065826
ENSBTAT00000015461
ENSBTAT00000046378
ENSBTAT00000038960
ENSBTAT00000064495
ENSBTAT00000063438
ENSBTAT00000066324
ENSBTAT00000063338
ENSBTAT00000064087
ENSBTAT00000038517
ENSBTAT00000063595
ENSBTAT00000066240
ENSBTAT00000065222
ENSBTAT00000064118
ENSBTAT00000065701
ENSBTAT00000053953
ENSBTAT00000063615
ENSBTAT00000064666
ENSBTAT00000065478
ENSBTAT00000064226
ENSBTAT00000064468
ENSBTAT00000063083
ENSBTAT00000064603
ENSBTAT00000064744
ENSBTAT00000063308
ENSBTAT00000065366
ENSBTAT00000065026
ENSBTAT00000063480
ENSBTAT00000066310

ENSBTAT00000063598
ENSBTAT00000065617
ENSBTAT00000064576
ENSBTAT00000063761
ENSBTAT00000064046
ENSBTAT00000063336
ENSBTAT00000065888
ENSBTAT00000053046
ENSBTAT00000064580
ENSBTAT00000064367
ENSBTAT00000066332
ENSBTAT00000064592
ENSBTAT00000036187
ENSBTAT00000065743
ENSBTAT00000063763
ENSBTAT00000063240
ENSBTAT00000062968
ENSBTAT00000063737
ENSBTAT00000063766
ENSBTAT00000066198
ENSBTAT00000065386
ENSBTAT00000066086
ENSBTAT00000065769
ENSBTAT00000064472
ENSBTAT00000065308
ENSBTAT00000062981
ENSBTAT00000063710
ENSBTAT00000053876
ENSBTAT00000064411
ENSBTAT00000063197

ENSBTAT00000063416
ENSBTAT00000064222
ENSBTAT00000064684
ENSBTAT00000065479
ENSBTAT00000052551
ENSBTAT00000066254
ENSBTAT00000064239
ENSBTAT00000063906
ENSBTAT00000063376
ENSBTAT00000066193
ENSBTAT00000064225
ENSBTAT00000064759
ENSBTAT00000063342
ENSBTAT00000064833
ENSBTAT00000064581
ENSBTAT00000063224
ENSBTAT00000064731
ENSBTAT00000065668
ENSBTAT00000059788
ENSBTAT00000063828
ENSBTAT00000063941
ENSBTAT00000065807
ENSBTAT00000065557
ENSBTAT00000065741
ENSBTAT00000064884
ENSBTAT00000065615
ENSBTAT00000065460
ENSBTAT00000063641
ENSBTAT00000064732
ENSBTAT00000065530

ENSBTAT00000054984
ENSBTAT00000063120
ENSBTAT00000066253
ENSBTAT00000065598
ENSBTAT00000049290
ENSBTAT00000064259
ENSBTAT00000065261
ENSBTAT00000064444
ENSBTAT00000062543
ENSBTAT00000065919
ENSBTAT00000010518
ENSBTAT00000065490
ENSBTAT00000064386
ENSBTAT00000065228
ENSBTAT00000066226
ENSBTAT00000065032
ENSBTAT00000065812
ENSBTAT00000064795
ENSBTAT00000064916
ENSBTAT00000064326
ENSBTAT00000066159
ENSBTAT00000064595
ENSBTAT00000063497
ENSBTAT00000064877
ENSBTAT00000064526
ENSBTAT00000064889
ENSBTAT00000064508
ENSBTAT00000064907
ENSBTAT00000015440
ENSBTAT00000066304

ENSBTAT00000063254
ENSBTAT00000065342
ENSBTAT00000065002
ENSBTAT00000063230
ENSBTAT00000053411
ENSBTAT00000064791
ENSBTAT00000064520
ENSBTAT00000065453
ENSBTAT00000064691
ENSBTAT00000066271
ENSBTAT00000064996
ENSBTAT00000064231
ENSBTAT00000064941
ENSBTAT00000059104
ENSBTAT00000061714
ENSBTAT00000065583
ENSBTAT00000063411
ENSBTAT00000054788
ENSBTAT00000064180
ENSBTAT00000064355
ENSBTAT00000065816
ENSBTAT00000065933
ENSBTAT00000064431
ENSBTAT00000061735
ENSBTAT00000063274
ENSBTAT00000065742
ENSBTAT00000065703
ENSBTAT00000056451
ENSBTAT00000064111
ENSBTAT00000063634

ENSBTAT00000065253
ENSBTAT00000064283
ENSBTAT00000063390
ENSBTAT00000064167
ENSBTAT00000064250
ENSBTAT00000065470
ENSBTAT00000065062
ENSBTAT00000064555
ENSBTAT00000032988
ENSBTAT00000064360
ENSBTAT00000066047
ENSBTAT00000065030
ENSBTAT00000063460
ENSBTAT00000063553
ENSBTAT00000064808
ENSBTAT00000065631
ENSBTAT00000063739
ENSBTAT00000063665
ENSBTAT00000065134
ENSBTAT00000054734
ENSBTAT00000051794
ENSBTAT00000051476
ENSBTAT00000066093
ENSBTAT00000065396
ENSBTAT00000064792
ENSBTAT00000064776
ENSBTAT00000065513
ENSBTAT00000064947
ENSBTAT00000065458
ENSBTAT00000063068

ENSBTAT00000063226
ENSBTAT00000065221
ENSBTAT00000064374
ENSBTAT00000064955
ENSBTAT00000053003
ENSBTAT00000011326
ENSBTAT00000063727
ENSBTAT00000063179
ENSBTAT00000064165
ENSBTAT00000051826
ENSBTAT00000063371
ENSBTAT00000063045
ENSBTAT00000066243
ENSBTAT00000064926
ENSBTAT00000030104
ENSBTAT00000065350
ENSBTAT00000063841
ENSBTAT00000064930
ENSBTAT00000063493
ENSBTAT00000065961
ENSBTAT00000064227
ENSBTAT00000064703
ENSBTAT00000065116
ENSBTAT00000065301
ENSBTAT00000065870
ENSBTAT00000064278
ENSBTAT00000064154
ENSBTAT00000064211
ENSBTAT00000065204
ENSBTAT00000065984

ENSBTAT00000063756
ENSBTAT00000065655
ENSBTAT00000062942
ENSBTAT00000065642
ENSBTAT00000065218
ENSBTAT00000064566
ENSBTAT00000064129
ENSBTAT00000065643
ENSBTAT00000065991
ENSBTAT00000065090
ENSBTAT00000063494
ENSBTAT00000054585
ENSBTAT00000065351
ENSBTAT00000065053
ENSBTAT00000066006
ENSBTAT00000063773
ENSBTAT00000065798
ENSBTAT00000066227
ENSBTAT00000062731
ENSBTAT00000064164
ENSBTAT00000064153
ENSBTAT00000065529
ENSBTAT00000062994
ENSBTAT00000063165
ENSBTAT00000063760
ENSBTAT00000066293
ENSBTAT00000063078
ENSBTAT00000063205
ENSBTAT00000062939
ENSBTAT00000065378

ENSBTAT00000065238
ENSBTAT00000064727
ENSBTAT00000063708
ENSBTAT00000064097
ENSBTAT00000063930
ENSBTAT00000064306
ENSBTAT00000065304
ENSBTAT00000065755
ENSBTAT00000066213
ENSBTAT00000065976
ENSBTAT00000063154
ENSBTAT00000065722
ENSBTAT00000063576
ENSBTAT00000064611
ENSBTAT00000063279
ENSBTAT00000062845
ENSBTAT00000020629
ENSBTAT00000055722
ENSBTAT00000024997
ENSBTAT00000064001
ENSBTAT00000064012
ENSBTAT00000063459
ENSBTAT00000055881
ENSBTAT00000065754
ENSBTAT00000064377
ENSBTAT00000063839
ENSBTAT00000064271
ENSBTAT00000063196
ENSBTAT00000063352
ENSBTAT00000065405

ENSBTAT00000064420
ENSBTAT00000064758
ENSBTAT00000063715
ENSBTAT00000065756
ENSBTAT00000066204
ENSBTAT00000062970
ENSBTAT00000066295
ENSBTAT00000063649
ENSBTAT00000064245
ENSBTAT00000065273
ENSBTAT00000065590
ENSBTAT00000062933
ENSBTAT00000063569
ENSBTAT00000065538
ENSBTAT00000029898
ENSBTAT00000063955
ENSBTAT00000063902
ENSBTAT00000066290
ENSBTAT00000062957
ENSBTAT00000065886
ENSBTAT00000062980
ENSBTAT00000064138
ENSBTAT00000049216
ENSBTAT00000063329
ENSBTAT00000065571
ENSBTAT00000065622
ENSBTAT00000064017
ENSBTAT00000065912
ENSBTAT00000064197
ENSBTAT00000053325

ENSBTAT00000066164
ENSBTAT00000063802
ENSBTAT00000065747
ENSBTAT00000055288
ENSBTAT00000065616
ENSBTAT00000063271
ENSBTAT00000064201
ENSBTAT00000065349
ENSBTAT00000064872
ENSBTAT00000064072
ENSBTAT00000066059
ENSBTAT00000064116
ENSBTAT00000065841
ENSBTAT00000064772
ENSBTAT00000063362
ENSBTAT00000064332
ENSBTAT00000066096
ENSBTAT00000063803
ENSBTAT00000010169
ENSBTAT00000064174
ENSBTAT00000064188
ENSBTAT00000065584
ENSBTAT00000057095
ENSBTAT00000065408
ENSBTAT00000063106
ENSBTAT00000063934
ENSBTAT00000065009
ENSBTAT00000063407
ENSBTAT00000063157
ENSBTAT00000057092

ENSBTAT00000064320
ENSBTAT00000064610
ENSBTAT00000065801
ENSBTAT00000063008
ENSBTAT00000063587
ENSBTAT00000064313
ENSBTAT00000063672
ENSBTAT00000065163
ENSBTAT00000057531
ENSBTAT00000065734
ENSBTAT00000063392
ENSBTAT00000065119
ENSBTAT00000064070
ENSBTAT00000063852
ENSBTAT00000064466
ENSBTAT00000065730
ENSBTAT00000065586
ENSBTAT00000063182
ENSBTAT00000063578
ENSBTAT00000064528
ENSBTAT00000063711
ENSBTAT00000065178
ENSBTAT00000065010
ENSBTAT00000064982
ENSBTAT00000064967
ENSBTAT00000063697
ENSBTAT00000064878
ENSBTAT00000063881
ENSBTAT00000065369
ENSBTAT00000064105

ENSBTAT00000066023
ENSBTAT00000066022
ENSBTAT00000065324
ENSBTAT00000064293
ENSBTAT00000064888
ENSBTAT00000064171
ENSBTAT00000063210
ENSBTAT00000063214
ENSBTAT00000065368
ENSBTAT00000064883
ENSBTAT00000064682
ENSBTAT00000049664
ENSBTAT00000065574
ENSBTAT00000064919
ENSBTAT00000064351
ENSBTAT00000063325
ENSBTAT00000063612
ENSBTAT00000003578
ENSBTAT00000066129
ENSBTAT00000049497
ENSBTAT00000065412
ENSBTAT00000019662
ENSBTAT00000066347
ENSBTAT00000065999
ENSBTAT00000064336
ENSBTAT00000066118
ENSBTAT00000065142
ENSBTAT00000064819
ENSBTAT00000066048
ENSBTAT00000063293

ENSBTAT00000043677
ENSBTAT00000063382
ENSBTAT00000065233
ENSBTAT00000064643
ENSBTAT00000065715
ENSBTAT00000064112
ENSBTAT00000064961
ENSBTAT00000065562
ENSBTAT00000063081
ENSBTAT00000063506
ENSBTAT00000064132
ENSBTAT00000065599
ENSBTAT00000055627
ENSBTAT00000064421
ENSBTAT00000063334
ENSBTAT00000063951
ENSBTAT00000066339
ENSBTAT00000065131
ENSBTAT00000063312
ENSBTAT00000064671
ENSBTAT00000063606
ENSBTAT00000063091
ENSBTAT00000063102
ENSBTAT00000064136
ENSBTAT00000065965
ENSBTAT00000038853
ENSBTAT00000064319
ENSBTAT00000064856
ENSBTAT00000064570
ENSBTAT00000065065

ENSBTAT00000066242
ENSBTAT00000063013
ENSBTAT00000065297
ENSBTAT00000048783
ENSBTAT00000065970
ENSBTAT00000048960
ENSBTAT00000064290
ENSBTAT00000063730
ENSBTAT00000064780
ENSBTAT00000064851
ENSBTAT00000053756
ENSBTAT00000064066
ENSBTAT00000064507
ENSBTAT00000065363
ENSBTAT00000064678
ENSBTAT00000063837
ENSBTAT00000063291
ENSBTAT00000063403
ENSBTAT00000065847
ENSBTAT00000066151
ENSBTAT00000065860
ENSBTAT00000063913
ENSBTAT00000065284
ENSBTAT00000065066
ENSBTAT00000062927
ENSBTAT00000065434
ENSBTAT00000066285
ENSBTAT00000063515
ENSBTAT00000063455
ENSBTAT00000056389

ENSBTAT00000066079
ENSBTAT00000063333
ENSBTAT00000063622
ENSBTAT00000065354
ENSBTAT00000065018
ENSBTAT00000064540
ENSBTAT00000062976
ENSBTAT00000064556
ENSBTAT00000064902
ENSBTAT00000063445
ENSBTAT00000064486
ENSBTAT00000066155
ENSBTAT00000065007
ENSBTAT00000063582
ENSBTAT00000063590
ENSBTAT00000065353
ENSBTAT00000054752
ENSBTAT00000065637
ENSBTAT00000062083
ENSBTAT00000025515
ENSBTAT00000063015
ENSBTAT00000065623
ENSBTAT00000063113
ENSBTAT00000064315
ENSBTAT00000063638
ENSBTAT00000063659
ENSBTAT00000063591
ENSBTAT00000066175
ENSBTAT00000063891
ENSBTAT00000063757

ENSBTAT00000066104
ENSBTAT00000063001
ENSBTAT00000065939
ENSBTAT00000063621
ENSBTAT00000064605
ENSBTAT00000062940
ENSBTAT00000063850
ENSBTAT00000065077
ENSBTAT00000066043
ENSBTAT00000065676
ENSBTAT00000065195
ENSBTAT00000063983
ENSBTAT00000063107
ENSBTAT00000063541
ENSBTAT00000065329
ENSBTAT00000066149
ENSBTAT00000062961
ENSBTAT00000064836
ENSBTAT00000065177
ENSBTAT00000064504
ENSBTAT00000062937
ENSBTAT00000063993
ENSBTAT00000063962
ENSBTAT00000065904
ENSBTAT00000063077
ENSBTAT00000064847
ENSBTAT00000065112
ENSBTAT00000064634
ENSBTAT00000064820
ENSBTAT00000066291

ENSBTAT00000063183
ENSBTAT00000066135
ENSBTAT00000065799
ENSBTAT00000064282
ENSBTAT00000063535
ENSBTAT00000064099
ENSBTAT00000063441
ENSBTAT00000066241
ENSBTAT00000064356
ENSBTAT00000057018
ENSBTAT00000066062
ENSBTAT00000066255
ENSBTAT00000065229
ENSBTAT00000066235
ENSBTAT00000066346
ENSBTAT00000064887
ENSBTAT00000065822
ENSBTAT00000054965
ENSBTAT00000063007
ENSBTAT00000064995
ENSBTAT00000063127
ENSBTAT00000065388
ENSBTAT00000064029
ENSBTAT00000007095
ENSBTAT00000064672
ENSBTAT00000063593
ENSBTAT00000064669
ENSBTAT00000063131
ENSBTAT00000063190
ENSBTAT00000064524

ENSBTAT00000065109
ENSBTAT00000017288
ENSBTAT00000063423
ENSBTAT00000064441
ENSBTAT00000063642
ENSBTAT00000064578
ENSBTAT00000007960
ENSBTAT00000065942
ENSBTAT00000063899
ENSBTAT00000044234
ENSBTAT00000064536
ENSBTAT00000061709
ENSBTAT00000063738
ENSBTAT00000065925
ENSBTAT00000066084
ENSBTAT00000065640
ENSBTAT00000064047
ENSBTAT00000064297
ENSBTAT00000054267
ENSBTAT00000065825
ENSBTAT00000066020
ENSBTAT00000065875
ENSBTAT00000065059

Bos taurus keratin-like (LOC615589), mRNA. [Source:RefSeq mRNA;Acc:NM_001098467]
Bos taurus zinc finger protein 266 (ZNF266), mRNA. [Source:RefSeq mRNA;Acc:NM_001104968]
U6 spliceosomal RNA [Source:RFAM;Acc:RF00026]
Uncharacterized protein [Source:UniProtKB/TrEMBL;Acc:G5E6A4]
U6 spliceosomal RNA [Source:RFAM;Acc:RF00026]
U6 spliceosomal RNA [Source:RFAM;Acc:RF00026]
U6 spliceosomal RNA [Source:RFAM;Acc:RF00026]
U6 spliceosomal RNA [Source:RFAM;Acc:RF00026]
Bos taurus solute carrier organic anion transporter family, member 1A2 (SLCO1A2), mRNA. [Source:RefSeq mRNA;Acc:NM_174654]
Bos taurus bolA homolog 1 (E. coli) (BOLA1), mRNA. [Source:RefSeq mRNA;Acc:NM_001034352]
U6 spliceosomal RNA [Source:RFAM;Acc:RF00026]
Bos taurus Lix1 homolog (chicken) (LIX1), mRNA. [Source:RefSeq mRNA;Acc:NM_001078003]
5S ribosomal RNA [Source:RFAM;Acc:RF00001]
5S ribosomal RNA [Source:RFAM;Acc:RF00001]
5S ribosomal RNA [Source:RFAM;Acc:RF00001]
5S ribosomal RNA [Source:RFAM;Acc:RF00001]
5S ribosomal RNA [Source:RFAM;Acc:RF00001]

U6atac minor spliceosomal RNA [Source:RFAM;Acc:RF00619]
Bos taurus polymerase (RNA) III (DNA directed) polypeptide G (32kD)-like (POLR3GL), mRNA. [Source:RefSeq mRNA;Acc:NM_001075213]
Small nucleolar RNA SNORA11 [Source:RFAM;Acc:RF00614]
U6atac minor spliceosomal RNA [Source:RFAM;Acc:RF00619]
Small nucleolar RNA SNORA70 [Source:RFAM;Acc:RF00156]
Small nucleolar RNA SNORA3/SNORA45 family [Source:RFAM;Acc:RF00334]
Small nucleolar RNA SNORA18 [Source:RFAM;Acc:RF00425]
Bos taurus ankyrin repeat domain 39 (ANKRD39), mRNA. [Source:RefSeq mRNA;Acc:NM_001075471]
U6atac minor spliceosomal RNA [Source:RFAM;Acc:RF00619]

Small nucleolar RNA SNORA35 [Source:RFAM;Acc:RF00566]

5S ribosomal RNA [Source:RFAM;Acc:RF00001]
U11 spliceosomal RNA [Source:RFAM;Acc:RF00548]
5S ribosomal RNA [Source:RFAM;Acc:RF00001]
chromosome 1 open reading frame 233 [Source:HGNC Symbol;Acc:HGNC:42951]
5S ribosomal RNA [Source:RFAM;Acc:RF00001]
Uncharacterized protein [Source:UniProtKB/TrEMBL;Acc:F1N372]
Small nucleolar RNA SNORA71 [Source:RFAM;Acc:RF00056]
Bos taurus GS homeobox 1 (GSX1), mRNA. [Source:RefSeq mRNA;Acc:NM_001105448]
Bos taurus F-box and leucine-rich repeat protein 21 (FBXL21), mRNA. [Source:RefSeq mRNA;Acc:NM_001034252]
THAP domain-containing protein 7 [Source:RefSeq peptide;Acc:NP_001091935]
neurogenin 1 [Source:HGNC Symbol;Acc:HGNC:7764]
Bos taurus major histocompatibility complex, class II, DQ alpha 5 (BOLA-DQA5), mRNA. [Source:RefSeq mRNA;Acc:NM_001012675]
5S ribosomal RNA [Source:RFAM;Acc:RF00001]
Small nucleolar RNA SNORA70 [Source:RFAM;Acc:RF00156]
U1 spliceosomal RNA [Source:RFAM;Acc:RF00003]
Bos taurus biogenesis of lysosomal organelles complex-1, subunit 3 (BLOC1S3), mRNA. [Source:RefSeq mRNA;Acc:NM_001099086]
7SK RNA [Source:RFAM;Acc:RF00100]
5.8S ribosomal RNA [Source:RFAM;Acc:RF00002]
Small nucleolar RNA SNORA20 [Source:RFAM;Acc:RF00401]
Uncharacterized protein [Source:UniProtKB/TrEMBL;Acc:G3N0D3]
Bos taurus chromosome 11 open reading frame, human C9orf169 (C11H9orf169), mRNA. [Source:RefSeq mRNA;Acc:NM_001078088]
Bos taurus SRY (sex determining region Y)-box 17 (SOX17), mRNA. [Source:RefSeq mRNA;Acc:NM_001206251]
5S ribosomal RNA [Source:RFAM;Acc:RF00001]
Small nucleolar RNA SNORA70 [Source:RFAM;Acc:RF00156]
Bos taurus leucine rich repeat containing 30 (LRRC30), mRNA. [Source:RefSeq mRNA;Acc:NM_001102510]
Small nucleolar RNA SNORA43 [Source:RFAM;Acc:RF00416]
dishevelled-binding antagonist of beta-catenin 3 [Source:HGNC Symbol;Acc:HGNC:30745]
Small nucleolar RNA SNORA70 [Source:RFAM;Acc:RF00156]
Bos taurus lysozyme (LYZ), mRNA. [Source:RefSeq mRNA;Acc:NM_001078159]
Histone H4 [Source:UniProtKB/Swiss-Prot;Acc:P62803]

Bos taurus BTB (POZ) domain containing 17 (BTBD17), mRNA. [Source:RefSeq mRNA;Acc:NM_001205464]
XK-related protein [Source:UniProtKB/TrEMBL;Acc:G5E6L6]
Bos taurus EGF-like-domain, multiple 7 (EGFL7), mRNA. [Source:RefSeq mRNA;Acc:NM_001078038]
5S ribosomal RNA [Source:RFAM;Acc:RF00001]
leucine rich repeat containing 26 [Source:HGNC Symbol;Acc:HGNC:31409]
Bos taurus transmembrane protein 151A (TMEM151A), mRNA. [Source:RefSeq mRNA;Acc:NM_001083782]
Bos taurus optic atrophy 3 (autosomal recessive, with chorea and spastic paraplegia) (OPA3), transcript variant 2, mRNA. [Source:RefSeq mRNA;Acc:NM_00124
Bos taurus luteinizing hormone/choriogonadotropin receptor (LHCGR), mRNA. [Source:RefSeq mRNA;Acc:NM_174381]
UDP-Gal:betaGal beta 1,3-galactosyltransferase polypeptide 6 [Source:HGNC Symbol;Acc:HGNC:17978]
forkhead box L1 [Source:HGNC Symbol;Acc:HGNC:3817]
claudin 14 [Source:HGNC Symbol;Acc:HGNC:2035]
family with sequence similarity 229, member A [Source:HGNC Symbol;Acc:HGNC:44652]
Small nucleolar RNA SNORA62/SNORA6 family [Source:RFAM;Acc:RF00091]
Hedgehog protein [Source:UniProtKB/TrEMBL;Acc:F1MFP2]
Sp5 transcription factor [Source:HGNC Symbol;Acc:HGNC:14529]
Bos taurus heparan sulfate 6-O-sulfotransferase 3 (HS6ST3), mRNA. [Source:RefSeq mRNA;Acc:NM_001205484]

v-maf avian musculoaponeurotic fibrosarcoma oncogene homolog K [Source:HGNC Symbol;Acc:HGNC:6782]
Uncharacterized protein [Source:UniProtKB/TrEMBL;Acc:E1BMY6]
neuropeptide W [Source:HGNC Symbol;Acc:HGNC:30509]
interleukin 11 [Source:HGNC Symbol;Acc:HGNC:5966]
hyaluronan and proteoglycan link protein 4 [Source:HGNC Symbol;Acc:HGNC:31357]
Bos taurus zinc finger protein 9 (ZCCHC13), mRNA. [Source:RefSeq mRNA;Acc:NM_001206479]
Bos taurus defensin beta 7 (DEFB7), mRNA. [Source:RefSeq mRNA;Acc:NM_001102362]
Bos taurus cyclin-dependent kinase inhibitor 1A (p21, Cip1) (CDKN1A), mRNA. [Source:RefSeq mRNA;Acc:NM_001098958]
Bos taurus mitochondrial ribosomal protein S26 (MRPS26), mRNA. [Source:RefSeq mRNA;Acc:NM_001034514]
Small nucleolar RNA SNORD15 [Source:RFAM;Acc:RF00067]
U4 spliceosomal RNA [Source:RFAM;Acc:RF00015]

transmembrane protein 151B [Source:HGNC Symbol;Acc:HGNC:21315]

Bos taurus adaptor-related protein complex 1, sigma 1 subunit (AP1S1), mRNA. [Source:RefSeq mRNA;Acc:NM_001077110]
kelch repeat and BTB (POZ) domain containing 13 [Source:HGNC Symbol;Acc:HGNC:37227]
Bos taurus NK2 homeobox 5 (NKX2-5), mRNA. [Source:RefSeq mRNA;Acc:NM_001046443]
Bos taurus DnaJ (Hsp40) homolog, subfamily C, member 24 (DNAJC24), mRNA. [Source:RefSeq mRNA;Acc:NM_001078102]
Bos taurus ribosomal protein S29 (RPS29), mRNA. [Source:RefSeq mRNA;Acc:NM_174804]
Uncharacterized protein [Source:UniProtKB/TrEMBL;Acc:G3MXE1]
zinc finger protein 628 [Source:HGNC Symbol;Acc:HGNC:28054]
testis-specific serine kinase 6 [Source:HGNC Symbol;Acc:HGNC:30410]
round spermatid basic protein 1-like [Source:HGNC Symbol;Acc:HGNC:24765]
Bos taurus Fanconi anemia, complementation group F (FANCF), mRNA. [Source:RefSeq mRNA;Acc:NM_001105425]
U2 spliceosomal RNA [Source:RFAM;Acc:RF00004]
Metazoan signal recognition particle RNA [Source:RFAM;Acc:RF00017]
Bos taurus bitter taste receptor Bota-T2R65A (T2R65A), mRNA. [Source:RefSeq mRNA;Acc:NM_001046634]
family with sequence similarity 163, member B [Source:HGNC Symbol;Acc:HGNC:33277]
Bos taurus small nuclear ribonucleoprotein polypeptide G (SNRPG), mRNA. [Source:RefSeq mRNA;Acc:NM_001040543]
Small nucleolar RNA SNORA75 [Source:RFAM;Acc:RF00072]
Bos taurus inhibin, beta B (INHBB), mRNA. [Source:RefSeq mRNA;Acc:NM_176852]
Bos taurus RNA-binding protein S1, serine-rich domain-like (LOC508834), mRNA. [Source:RefSeq mRNA;Acc:NM_001206658]
Uncharacterized protein [Source:UniProtKB/TrEMBL;Acc:G3MZF9]
purine-rich element binding protein A [Source:HGNC Symbol;Acc:HGNC:9701]
Bos taurus coiled-coil domain containing 72 (TMA7), mRNA. [Source:RefSeq mRNA;Acc:NM_001163803]
zinc finger CCCH-type containing 12D [Source:HGNC Symbol;Acc:HGNC:21175]
Bos taurus deoxyhypusine hydroxylase/monooxygenase (DOHH), mRNA. [Source:RefSeq mRNA;Acc:NM_001075886]
growth arrest-specific 1 [Source:HGNC Symbol;Acc:HGNC:4165]
Bos taurus chromosome 19 open reading frame, human C17orf103 (C19H17orf103), mRNA. [Source:RefSeq mRNA;Acc:NM_001102331]
coiled-coil domain containing 177 [Source:HGNC Symbol;Acc:HGNC:23243]
Bos taurus heat shock 70kDa protein 1A (HSPA1A), mRNA. [Source:RefSeq mRNA;Acc:NM_203322]
Metazoan signal recognition particle RNA [Source:RFAM;Acc:RF00017]
Uncharacterized protein [Source:UniProtKB/TrEMBL;Acc:E1BEK6]
Histone H4 [Source:UniProtKB/Swiss-Prot;Acc:P62803]

tRNA methyltransferase 61 homolog A (S. cerevisiae) [Source:HGNC Symbol;Acc:HGNC:23790]
Bos taurus actin-like 7B (ACTL7B), mRNA. [Source:RefSeq mRNA;Acc:NM_001075697]
Bos taurus neurturin (NRTN), mRNA. [Source:RefSeq mRNA;Acc:NM_001205919]
small integral membrane protein 15 [Source:HGNC Symbol;Acc:HGNC:33861]
Uncharacterized protein [Source:UniProtKB/TrEMBL;Acc:E1BMY5]
Bos taurus coiled-coil glutamate-rich protein 1 (CCER1), mRNA. [Source:RefSeq mRNA;Acc:NM_001038578]
7SK RNA [Source:RFAM;Acc:RF00100]
Bos taurus IQ motif containing F5 (IQCF5), mRNA. [Source:RefSeq mRNA;Acc:NM_001077060]
kelch repeat and BTB (POZ) domain containing 11 [Source:HGNC Symbol;Acc:HGNC:29104]
brain expressed, associated with NEDD4, 1 [Source:HGNC Symbol;Acc:HGNC:24160]
Metazoan signal recognition particle RNA [Source:RFAM;Acc:RF00017]
Histone H2A type 1 [Source:UniProtKB/Swiss-Prot;Acc:P0C0S9]
Uncharacterized protein [Source:UniProtKB/TrEMBL;Acc:F6RSR1]
Bos taurus myeloid-associated differentiation marker-like (LOC618633), mRNA. [Source:RefSeq mRNA;Acc:NM_001103302]
histone cluster 1, H1e [Source:HGNC Symbol;Acc:HGNC:4718]
Uncharacterized protein [Source:UniProtKB/TrEMBL;Acc:G3N1W0]

U1 spliceosomal RNA [Source:RFAM;Acc:RF00003]
CD70 molecule [Source:HGNC Symbol;Acc:HGNC:11937]
elastin microfibril interfacer 3 [Source:HGNC Symbol;Acc:HGNC:16123]
homeobox B5 [Source:HGNC Symbol;Acc:HGNC:5116]
frizzled class receptor 5 [Source:HGNC Symbol;Acc:HGNC:4043]
Metazoan signal recognition particle RNA [Source:RFAM;Acc:RF00017]
forkhead box N3 [Source:HGNC Symbol;Acc:HGNC:1928]

armadillo repeat containing 5 [Source:HGNC Symbol;Acc:HGNC:25781]
Histone H2A [Source:UniProtKB/TrEMBL;Acc:F1MRN2]
Bos taurus cerebral dopamine neurotrophic factor (CDNF), mRNA. [Source:RefSeq mRNA;Acc:NM_001205769]
U2 spliceosomal RNA [Source:RFAM;Acc:RF00004]

T cell receptor alpha variable 17 [Source:HGNC Symbol;Acc:HGNC:12113]
SERTA domain containing 2 [Source:HGNC Symbol;Acc:HGNC:30784]
Uncharacterized protein [Source:UniProtKB/TrEMBL;Acc:G3MYB8]
synaptotagmin VIII [Source:HGNC Symbol;Acc:HGNC:19264]
Small Cajal body specific RNA 6 [Source:RFAM;Acc:RF00478]
long intergenic non-protein coding RNA 116 [Source:HGNC Symbol;Acc:HGNC:27339]

Suppressor of cytokine signaling 1; Suppressor of cytokine signaling 1-like; Uncharacterized protein [Source:UniProtKB/TrEMBL;Acc:F1MSN0]
H6 family homeobox 2 [Source:HGNC Symbol;Acc:HGNC:5018]
reprimo-like [Source:HGNC Symbol;Acc:HGNC:32422]
Uncharacterized protein [Source:UniProtKB/TrEMBL;Acc:F1MB80]
Bos taurus uncharacterized LOC509087 (DKFZP761E198), mRNA. [Source:RefSeq mRNA;Acc:NM_001014877]
Bos taurus transition protein 2 (during histone to protamine replacement) (TNP2), mRNA. [Source:RefSeq mRNA;Acc:NM_174200]
fucosyltransferase 7 (alpha (1,3) fucosyltransferase) [Source:HGNC Symbol;Acc:HGNC:4018]
SWIM-type zinc finger 7 associated protein 1 [Source:HGNC Symbol;Acc:HGNC:26638]
chromosome 17 open reading frame 50 [Source:HGNC Symbol;Acc:HGNC:29581]
Bos taurus chromosome 7 open reading frame, human C19orf52 (C7H19orf52), mRNA. [Source:RefSeq mRNA;Acc:NM_001099699]
Metazoan signal recognition particle RNA [Source:RFAM;Acc:RF00017]
one cut homeobox 3 [Source:HGNC Symbol;Acc:HGNC:13399]
Metazoan signal recognition particle RNA [Source:RFAM;Acc:RF00017]
Uncharacterized protein [Source:UniProtKB/TrEMBL;Acc:E1BPR8]
2-aminoethanethiol (cysteamine) dioxygenase [Source:HGNC Symbol;Acc:HGNC:23506]
Uncharacterized protein [Source:UniProtKB/TrEMBL;Acc:F1MIS2]
wingless-type MMTV integration site family, member 9B [Source:HGNC Symbol;Acc:HGNC:12779]
Bos taurus zinc finger, AN1-type domain 2A (ZFAND2A), mRNA. [Source:RefSeq mRNA;Acc:NM_001083762]
Uncharacterized protein [Source:UniProtKB/TrEMBL;Acc:F1N656]
coiled-coil domain containing 182 [Source:HGNC Symbol;Acc:HGNC:49392]
nucleoredoxin-like 1 [Source:HGNC Symbol;Acc:HGNC:25179]
Bos taurus mercaptopyruvate sulfurtransferase (MPST), transcript variant 1, mRNA. [Source:RefSeq mRNA;Acc:NM_001034291]
Bos taurus gap junction protein, alpha 4, 37kDa (GJA4), mRNA. [Source:RefSeq mRNA;Acc:NM_001083738]

family with sequence similarity 181, member A [Source:HGNC Symbol;Acc:HGNC:20491]
Uncharacterized protein [Source:UniProtKB/TrEMBL;Acc:G3N129]
immunoglobulin superfamily containing leucine-rich repeat 2 [Source:HGNC Symbol;Acc:HGNC:29286]
Uncharacterized protein [Source:UniProtKB/TrEMBL;Acc:G3MWQ7]
chromosome 9 open reading frame 172 [Source:HGNC Symbol;Acc:HGNC:37284]

SH3 domain binding kinase family, member 2 [Source:HGNC Symbol;Acc:HGNC:34416]
engrailed homeobox 2 [Source:HGNC Symbol;Acc:HGNC:3343]
Uncharacterized protein [Source:UniProtKB/TrEMBL;Acc:G3MWN3]
Bos taurus cerebellar degeneration-related protein 2-like (CDR2L), mRNA. [Source:RefSeq mRNA;Acc:NM_001192968]
Bos taurus protein arginine methyltransferase 6 (PRMT6), mRNA. [Source:RefSeq mRNA;Acc:NM_001014962]
docking protein 3 [Source:HGNC Symbol;Acc:HGNC:24583]
Bos taurus keratin associated protein LOC617218 (LOC617218), mRNA. [Source:RefSeq mRNA;Acc:NM_001079791]
Histone H2A [Source:UniProtKB/TrEMBL;Acc:F1MLQ1]
Sp8 transcription factor [Source:HGNC Symbol;Acc:HGNC:19196]
Bos taurus sarcoglycan, beta (43kDa dystrophin-associated glycoprotein) (SGCB), mRNA. [Source:RefSeq mRNA;Acc:NM_001102188]
wingless-type MMTV integration site family, member 9A [Source:HGNC Symbol;Acc:HGNC:12778]
Bos taurus myogenic differentiation 1 (MYOD1), mRNA. [Source:RefSeq mRNA;Acc:NM_001040478]
Bos taurus family with sequence similarity 167, member B (FAM167B), mRNA. [Source:RefSeq mRNA;Acc:NM_001101268]
actin-like 10 [Source:HGNC Symbol;Acc:HGNC:16127]
mitochondrial coiled-coil domain 1 [Source:HGNC Symbol;Acc:HGNC:20668]
Uncharacterized protein [Source:UniProtKB/TrEMBL;Acc:G3N3D8]
Bos taurus macrophage migration inhibitory factor (glycosylation-inhibiting factor) (MIF), mRNA. [Source:RefSeq mRNA;Acc:NM_001033608]
Bos taurus suppressor of cytokine signaling 3 (SOCS3), mRNA. [Source:RefSeq mRNA;Acc:NM_174466]
Bos taurus galactose-3-O-sulfotransferase 1 (GAL3ST1), mRNA. [Source:RefSeq mRNA;Acc:NM_001101972]
Bos taurus crystallin, beta A2 (CRYBA2), mRNA. [Source:RefSeq mRNA;Acc:NM_174524]
7SK RNA [Source:RFAM;Acc:RF00100]
syntaphilin [Source:HGNC Symbol;Acc:HGNC:15931]
Uncharacterized protein [Source:UniProtKB/TrEMBL;Acc:E1BG10]

Uncharacterized protein [Source:UniProtKB/TrEMBL;Acc:G3MZW0]
leucine-rich single-pass membrane protein 2 [Source:HGNC Symbol;Acc:HGNC:26781]
C2 calcium-dependent domain-containing protein 4A [Source:UniProtKB/TrEMBL;Acc:G3N1Z1]
chromosome 11 open reading frame 87 [Source:HGNC Symbol;Acc:HGNC:33788]
Bos taurus bone gamma-carboxyglutamate (gla) protein (BGLAP), mRNA. [Source:RefSeq mRNA;Acc:NM_174249]
breast cancer anti-estrogen resistance 1 [Source:HGNC Symbol;Acc:HGNC:971]
Bos taurus growth differentiation factor 6 (GDF6), mRNA. [Source:RefSeq mRNA;Acc:NM_001001140]

GATA binding protein 2 [Source:HGNC Symbol;Acc:HGNC:4171]
Uncharacterized protein [Source:UniProtKB/TrEMBL;Acc:G5E6P9]
Bos taurus selenoprotein M (SELM), mRNA. [Source:RefSeq mRNA;Acc:NM_001163171]
Bos taurus histone deacetylase 10 (HDAC10), mRNA. [Source:RefSeq mRNA;Acc:NM_001075460]
forkhead box A2 [Source:HGNC Symbol;Acc:HGNC:5022]
Uncharacterized protein [Source:UniProtKB/TrEMBL;Acc:G3MZY0]
7SK RNA [Source:RFAM;Acc:RF00100]
Bos taurus apolipoprotein A-V (APOA5), mRNA. [Source:RefSeq mRNA;Acc:NM_001083492]
CMRF35-like molecule 9 [Source:UniProtKB/Swiss-Prot;Acc:A5D7B2]
Uncharacterized protein [Source:UniProtKB/TrEMBL;Acc:E1BMJ4]

Bos taurus apolipoprotein C-IV (APOC4), mRNA. [Source:RefSeq mRNA;Acc:NM_001034807]

Bos taurus phosphoglycerate mutase 2 (muscle) (PGAM2), mRNA. [Source:RefSeq mRNA;Acc:NM_001038111]
Bos taurus nuclear protein, transcriptional regulator, 1 (NUPR1), mRNA. [Source:RefSeq mRNA;Acc:NM_001114515]
sosondowah ankyrin repeat domain family member D [Source:HGNC Symbol;Acc:HGNC:32960]

proline rich 22 [Source:HGNC Symbol;Acc:HGNC:28354]
LY6/PLAUR domain containing 6B [Source:HGNC Symbol;Acc:HGNC:27018]
Small Cajal body-specific RNA 7 [Source:RFAM;Acc:RF01295]
Bos taurus nudix (nucleoside diphosphate linked moiety X)-type motif 16 (NUDT16), mRNA. [Source:RefSeq mRNA;Acc:NM_001075560]

Bos taurus coagulation factor II (thrombin) receptor-like 3 (F2RL3), mRNA. [Source:RefSeq mRNA;Acc:NM_001076138]

tumor suppressor candidate 1 [Source:HGNC Symbol;Acc:HGNC:31010]

malignant fibrous histiocytoma amplified sequence 1 [Source:HGNC Symbol;Acc:HGNC:16982]
Uncharacterized protein [Source:UniProtKB/TrEMBL;Acc:E1BBD9]
Histone H2A type 1 [Source:UniProtKB/Swiss-Prot;Acc:P0C0S9]

Bos taurus thiosulfate sulfurtransferase (rhodanese) (TST), mRNA. [Source:RefSeq mRNA;Acc:NM_177489]
hydroxyacylglutathione hydrolase-like [Source:HGNC Symbol;Acc:HGNC:14177]
mex-3 RNA binding family member B [Source:HGNC Symbol;Acc:HGNC:25297]
Bos taurus fibroblast growth factor 22 (FGF22), mRNA. [Source:RefSeq mRNA;Acc:NM_001205861]

Bos taurus mediator complex subunit 9 (MED9), mRNA. [Source:RefSeq mRNA;Acc:NM_001080916]
SH3 domain binding kinase 1 [Source:HGNC Symbol;Acc:HGNC:17699]
F-box and leucine-rich repeat protein 19 [Source:HGNC Symbol;Acc:HGNC:25300]
Uncharacterized protein [Source:UniProtKB/TrEMBL;Acc:F1MYN9]
Bos taurus zinc finger protein 668 (ZNF668), mRNA. [Source:RefSeq mRNA;Acc:NM_001076953]

Bos taurus hemoglobin, theta 1 (HBQ1), mRNA. [Source:RefSeq mRNA;Acc:NM_001080333]
Transport and Golgi organization protein 2 homolog [Source:UniProtKB/TrEMBL;Acc:F6Q0Y7]
Bos taurus twist homolog 1 (Drosophila) (TWIST1), mRNA. [Source:RefSeq mRNA;Acc:NM_001191145]
NEDD4 binding protein 3 [Source:HGNC Symbol;Acc:HGNC:29852]
B-cell CLL/lymphoma 11B (zinc finger protein) [Source:HGNC Symbol;Acc:HGNC:13222]
Bos taurus immediate early response 2 (IER2), mRNA. [Source:RefSeq mRNA;Acc:NM_001075871]
prolyl-tRNA synthetase 2, mitochondrial (putative) [Source:HGNC Symbol;Acc:HGNC:30563]
Bos taurus zinc finger protein 205 (ZNF205), mRNA. [Source:RefSeq mRNA;Acc:NM_001034473]
Uncharacterized protein [Source:UniProtKB/TrEMBL;Acc:E1BF77]
hexose-6-phosphate dehydrogenase (glucose 1-dehydrogenase) [Source:HGNC Symbol;Acc:HGNC:4795]

Bos taurus chymotrypsinogen B1 (CTRB1), mRNA. [Source:RefSeq mRNA;Acc:NM_001105330]
Bos taurus claudin 5 (CLDN5), mRNA. [Source:RefSeq mRNA;Acc:NM_001076460]
Bos taurus keratin associated protein 3-1 (KRTAP3-1), mRNA. [Source:RefSeq mRNA;Acc:NM_001077104]
Bos taurus lipoma HMGIC fusion partner (LHFP), mRNA. [Source:RefSeq mRNA;Acc:NM_001077990]
Small nucleolar RNA U85 [Source:RFAM;Acc:RF01296]
nuclear factor of activated T-cells, cytoplasmic, calcineurin-dependent 2 [Source:HGNC Symbol;Acc:HGNC:7776]

Uncharacterized protein [Source:UniProtKB/TrEMBL;Acc:E1BBY0]
Bos taurus sphingosine-1-phosphate receptor 2 (S1PR2), mRNA. [Source:RefSeq mRNA;Acc:NM_001081541]
C-type lectin domain family 2, member L [Source:HGNC Symbol;Acc:HGNC:21969]
neuronal differentiation 2 [Source:HGNC Symbol;Acc:HGNC:7763]
Bos taurus programmed cell death 1 (PDCD1), mRNA. [Source:RefSeq mRNA;Acc:NM_001083506]
Uncharacterized protein [Source:UniProtKB/TrEMBL;Acc:G3X7J3]
Bos taurus runt-related transcription factor 3 (RUNX3), mRNA. [Source:RefSeq mRNA;Acc:NM_001193158]
BRCA1 associated RING domain 1 [Source:HGNC Symbol;Acc:HGNC:952]
ankyrin repeat domain 60 [Source:HGNC Symbol;Acc:HGNC:16217]
Bos taurus cerebellin 3 precursor (CBLN3), mRNA. [Source:RefSeq mRNA;Acc:NM_001079603]
Uncharacterized protein [Source:UniProtKB/TrEMBL;Acc:E1BMY7]
Histone H2B [Source:UniProtKB/TrEMBL;Acc:F1N5F3]
Uncharacterized protein [Source:UniProtKB/TrEMBL;Acc:E1BHJ2]
Bos taurus trafficking protein particle complex 1 (TRAPPC1), mRNA. [Source:RefSeq mRNA;Acc:NM_001076413]
Bos taurus zinc finger, FYVE domain containing 21 (ZFYVE21), mRNA. [Source:RefSeq mRNA;Acc:NM_001079587]
Bos taurus transmembrane protein 247 (TMEM247), mRNA. [Source:RefSeq mRNA;Acc:NM_001077069]
Uncharacterized protein [Source:UniProtKB/TrEMBL;Acc:F1N3V7]
Bos taurus polymerase (RNA) II (DNA directed) polypeptide F (POLR2F), mRNA. [Source:RefSeq mRNA;Acc:NM_001037475]
Bos taurus transmembrane protein 204 (TMEM204), mRNA. [Source:RefSeq mRNA;Acc:NM_001076377]
Bos taurus anaphase promoting complex subunit 11 (ANAPC11), mRNA. [Source:RefSeq mRNA;Acc:NM_001113724]
Uncharacterized protein [Source:UniProtKB/TrEMBL;Acc:G3N1L3]
Bos taurus homeobox C13 (HOXC13), mRNA. [Source:RefSeq mRNA;Acc:NM_001083490]

Bos taurus solute carrier family 5 (sodium/glucose cotransporter), member 12 (SLC5A12), mRNA. [Source:RefSeq mRNA;Acc:NM_001101059]
Bos taurus urocortin (UCN), mRNA. [Source:RefSeq mRNA;Acc:NM_001032301]
Bos taurus growth arrest and DNA-damage-inducible, gamma (GADD45G), mRNA. [Source:RefSeq mRNA;Acc:NM_001045901]
U2 spliceosomal RNA [Source:RFAM;Acc:RF00004]

zinc finger protein 70 [Source:HGNC Symbol;Acc:HGNC:13140]
Bos taurus testis expressed 264 (TEX264), mRNA. [Source:RefSeq mRNA;Acc:NM_001038169]
tripartite motif containing 56 [Source:HGNC Symbol;Acc:HGNC:19028]
Bos taurus phosphatidylinositol glycan anchor biosynthesis, class M (PIGM), mRNA. [Source:RefSeq mRNA;Acc:NM_001015563]
family with sequence similarity 222, member A [Source:HGNC Symbol;Acc:HGNC:25915]
Bos taurus cytochrome c oxidase subunit Vb (COX5B), mRNA. [Source:RefSeq mRNA;Acc:NM_001034046]

late endosomal/lysosomal adaptor, MAPK and MTOR activator 5 [Source:HGNC Symbol;Acc:HGNC:17955]
Bos taurus immunity-related GTPase family, cinema (IRGC), mRNA. [Source:RefSeq mRNA;Acc:NM_001076146]
TRABD protein; Uncharacterized protein [Source:UniProtKB/TrEMBL;Acc:A6QPJ3]
Uncharacterized protein [Source:UniProtKB/TrEMBL;Acc:G3N028]
Bos taurus UDP-GlcNAc:betaGal beta-1,3-N-acetylglucosaminyltransferase 8 (B3GNT8), mRNA. [Source:RefSeq mRNA;Acc:NM_001076040]
Bos taurus olfactomedin 1 (OLFM1), mRNA. [Source:RefSeq mRNA;Acc:NM_001101879]
Uncharacterized protein [Source:UniProtKB/TrEMBL;Acc:G3MY40]
Bos taurus zinc binding alcohol dehydrogenase domain containing 2 (ZADH2), mRNA. [Source:RefSeq mRNA;Acc:NM_001075964]
Bos taurus WAP, follistatin/kazal, immunoglobulin, kunitz and netrin domain containing 1 (WFIKKN1), mRNA. [Source:RefSeq mRNA;Acc:NM_001205796]
forkhead box D3 [Source:HGNC Symbol;Acc:HGNC:3804]
Bos taurus chromobox homolog 8 (CBX8), mRNA. [Source:RefSeq mRNA;Acc:NM_001077862]
transmembrane protein 8C [Source:HGNC Symbol;Acc:HGNC:33778]
Bos taurus T-cell leukemia homeobox 3 (TLX3), mRNA. [Source:RefSeq mRNA;Acc:NM_001193191]

Uncharacterized protein [Source:UniProtKB/TrEMBL;Acc:F1MI86]
Uncharacterized protein [Source:UniProtKB/TrEMBL;Acc:G3MY12]
Bos taurus unc-119 homolog B (C. elegans) (UNC119B), mRNA. [Source:RefSeq mRNA;Acc:NM_001206287]
Uncharacterized protein [Source:UniProtKB/TrEMBL;Acc:G3MXP0]

neurotrophin 4 [Source:HGNC Symbol;Acc:HGNC:8024]
neurexophilin 4 [Source:HGNC Symbol;Acc:HGNC:8078]
Uncharacterized protein [Source:UniProtKB/TrEMBL;Acc:F1MZ28]
zinc finger and BTB domain-containing protein 7A [Source:RefSeq peptide;Acc:NP_001178176]
Bos taurus HIG1 hypoxia inducible domain family, member 1B (HIGD1B), mRNA. [Source:RefSeq mRNA;Acc:NM_001077896]
dystroglycan 1 (dystrophin-associated glycoprotein 1) [Source:HGNC Symbol;Acc:HGNC:2666]
transmembrane protein 119 [Source:HGNC Symbol;Acc:HGNC:27884]
Metazoan signal recognition particle RNA [Source:RFAM;Acc:RF00017]
Bos taurus ring finger protein 113A (RNF113A), mRNA. [Source:RefSeq mRNA;Acc:NM_001007807]
Bos taurus wingless-type MMTV integration site family, member 5B (WNT5B), mRNA. [Source:RefSeq mRNA;Acc:NM_001205628]
7SK RNA [Source:RFAM;Acc:RF00100]
Bos taurus coiled-coil-helix-coiled-coil-helix domain containing 10 (CHCHD10), mRNA. [Source:RefSeq mRNA;Acc:NM_001083774]
Bos taurus distal-less homeobox 2 (DLX2), mRNA. [Source:RefSeq mRNA;Acc:NM_001192662]
Bos taurus protamine 2 (PRM2), mRNA. [Source:RefSeq mRNA;Acc:NM_174157]
transmembrane inner ear [Source:HGNC Symbol;Acc:HGNC:30800]
Bos taurus beta-1,3-glucuronyltransferase 3 (glucuronosyltransferase I) (B3GAT3), mRNA. [Source:RefSeq mRNA;Acc:NM_205805]
Bos taurus thymosin beta 4, X-linked (TMSB4), mRNA. [Source:RefSeq mRNA;Acc:NM_001113231]
transmembrane protein 121 [Source:HGNC Symbol;Acc:HGNC:20511]
Bos taurus TAF10 RNA polymerase II, TATA box binding protein (TBP)-associated factor, 30kDa (TAF10), mRNA. [Source:RefSeq mRNA;Acc:NM_001098968]
zinc finger protein 648 [Source:HGNC Symbol;Acc:HGNC:18190]
neuronal tyrosine-phosphorylated phosphoinositide-3-kinase adaptor 1 [Source:HGNC Symbol;Acc:HGNC:22009]
trophoblast glycoprotein [Source:HGNC Symbol;Acc:HGNC:12004]
Bos taurus cytosolic iron-sulfur protein assembly 1 (CIAO1), mRNA. [Source:RefSeq mRNA;Acc:NM_001037482]
C2 calcium-dependent domain containing 4D [Source:HGNC Symbol;Acc:HGNC:37210]
Bos taurus Sjogren syndrome/scleroderma autoantigen 1 (SSSCA1), mRNA. [Source:RefSeq mRNA;Acc:NM_001038528]
TGFB-induced factor homeobox 2 [Source:HGNC Symbol;Acc:HGNC:15764]
Uncharacterized protein [Source:UniProtKB/TrEMBL;Acc:E1BAB3]
zinc finger protein 74 [Source:HGNC Symbol;Acc:HGNC:13144]
Bos taurus coiled-coil domain containing 85B (CCDC85B), mRNA. [Source:RefSeq mRNA;Acc:NM_001144087]
Uncharacterized protein [Source:UniProtKB/TrEMBL;Acc:G3N2R3]

family with sequence similarity 170, member B [Source:HGNC Symbol;Acc:HGNC:19736]
Bos taurus mitochondrial ribosomal protein L14 (MRPL14), mRNA. [Source:RefSeq mRNA;Acc:NM_001076337]
EF-hand and coiled-coil domain containing 1 [Source:HGNC Symbol;Acc:HGNC:25692]
Bos taurus frizzled family receptor 10 (FZD10), mRNA. [Source:RefSeq mRNA;Acc:NM_001098033]
extracellular leucine-rich repeat and fibronectin type III domain containing 1 [Source:HGNC Symbol;Acc:HGNC:33154]
regulatory factor X-associated protein [Source:HGNC Symbol;Acc:HGNC:9988]
Uncharacterized protein [Source:UniProtKB/TrEMBL;Acc:G3MZI3]
Bos taurus calcineurin-like phosphoesterase domain containing 1 (CPPED1), mRNA. [Source:RefSeq mRNA;Acc:NM_001031771]
Bos taurus methylmalonic aciduria (cobalamin deficiency) cblC type, with homocystinuria (MMACHC), mRNA. [Source:RefSeq mRNA;Acc:NM_001015588]
Bos taurus fatty acid desaturase domain family, member 6 (FADS6), mRNA. [Source:RefSeq mRNA;Acc:NM_001081722]
Fer3-like bHLH transcription factor [Source:HGNC Symbol;Acc:HGNC:16660]
Uncharacterized protein [Source:UniProtKB/TrEMBL;Acc:E1BIW5]
insulin receptor substrate 2 [Source:HGNC Symbol;Acc:HGNC:6126]
60S ribosomal protein L36 [Source:UniProtKB/Swiss-Prot;Acc:Q3T171]
Bos taurus melanoma-associated antigen 10-like (LOC618806), mRNA. [Source:RefSeq mRNA;Acc:NM_001206356]
Bos taurus lipase maturation factor 1 (LMF1), mRNA. [Source:RefSeq mRNA;Acc:NM_001075190]
Tigger transposable element-derived protein 5 [Source:UniProtKB/Swiss-Prot;Acc:G3MY25]
zinc finger protein 428 [Source:HGNC Symbol;Acc:HGNC:20804]
family with sequence similarity 196, member A [Source:HGNC Symbol;Acc:HGNC:33859]
Bos taurus chromosome 19 open reading frame, human C17orf59 (C19H17orf59), mRNA. [Source:RefSeq mRNA;Acc:NM_001040565]
Uncharacterized protein [Source:UniProtKB/TrEMBL;Acc:G3MZK2]
sonic hedgehog [Source:HGNC Symbol;Acc:HGNC:10848]
dishevelled-binding antagonist of beta-catenin 2 [Source:HGNC Symbol;Acc:HGNC:21231]
Bos taurus germinal histone H4-like (LOC530773), mRNA. [Source:RefSeq mRNA;Acc:NM_001145871]
coiled-coil domain containing 42B [Source:HGNC Symbol;Acc:HGNC:37100]
Bos taurus melanoma antigen family B-like (MGC133764), mRNA. [Source:RefSeq mRNA;Acc:NM_001080372]
Bos taurus chromosome 3 open reading frame, human C1orf183 (FAM212B), mRNA. [Source:RefSeq mRNA;Acc:NM_001101261]
Bos taurus SERTA domain containing 1 (SERTAD1), mRNA. [Source:RefSeq mRNA;Acc:NM_001040564]
homeobox C8 [Source:HGNC Symbol;Acc:HGNC:5129]
immunity-related GTPase family, Q [Source:HGNC Symbol;Acc:HGNC:24868]

Uncharacterized protein [Source:UniProtKB/TrEMBL;Acc:G3MVZ8]

zinc finger, DHHC-type containing 8 [Source:HGNC Symbol;Acc:HGNC:18474]
Bos taurus coiled-coil domain containing 116 (CCDC116), mRNA. [Source:RefSeq mRNA;Acc:NM_001038222]
canopy FGF signaling regulator 1 [Source:HGNC Symbol;Acc:HGNC:27786]
Bos taurus KCNE1-like (KCNE1L), mRNA. [Source:RefSeq mRNA;Acc:NM_001077875]
chibby homolog 3 (Drosophila) [Source:HGNC Symbol;Acc:HGNC:33278]
kelch domain containing 7A [Source:HGNC Symbol;Acc:HGNC:26791]
Uncharacterized protein [Source:UniProtKB/TrEMBL;Acc:G3MWT1]
Bos taurus chemokine binding protein 2 (CCBP2), mRNA. [Source:RefSeq mRNA;Acc:NM_001015581]

Bos taurus MAS-related GPR, member X2 (MRGPRX2), mRNA. [Source:RefSeq mRNA;Acc:NM_001192450]
chromosome 1 open reading frame 87 [Source:HGNC Symbol;Acc:HGNC:28547]
frizzled class receptor 9 [Source:HGNC Symbol;Acc:HGNC:4047]
Uncharacterized protein [Source:UniProtKB/TrEMBL;Acc:G3N2D9]

Bos taurus oocyte expressed protein homolog (dog) (OOEP), mRNA. [Source:RefSeq mRNA;Acc:NM_001077869]
ephrin-B2 [Source:HGNC Symbol;Acc:HGNC:3227]
v-maf avian musculoaponeurotic fibrosarcoma oncogene homolog [Source:HGNC Symbol;Acc:HGNC:6776]
Uncharacterized protein [Source:UniProtKB/TrEMBL;Acc:F1N066]

Uncharacterized protein [Source:UniProtKB/TrEMBL;Acc:E1BKL5]
Bos taurus ribosomal protein S15A (RPS15A), mRNA. [Source:RefSeq mRNA;Acc:NM_001037443]

7SK RNA [Source:RFAM;Acc:RF00100]
RAB20, member RAS oncogene family [Source:HGNC Symbol;Acc:HGNC:18260]

zinc finger, BED-type containing 1 [Source:HGNC Symbol;Acc:HGNC:447]
Bos taurus ChaC, cation transport regulator homolog 1 (E. coli) (CHAC1), mRNA. [Source:RefSeq mRNA;Acc:NM_001098882]
kelch-like family member 25 [Source:HGNC Symbol;Acc:HGNC:25732]

Bos taurus tumor necrosis factor receptor superfamily, member 6b, decoy (TNFRSF6B), mRNA. [Source:RefSeq mRNA;Acc:NM_001101306]
Bos taurus leucine rich repeat containing 8 family, member E (LRRC8E), mRNA. [Source:RefSeq mRNA;Acc:NM_001205622]
Bos taurus parahox cluster neighbor (PRHOXNB), mRNA. [Source:RefSeq mRNA;Acc:NM_001099703]
Bos taurus PWWP domain containing 2B (PWWP2B), mRNA. [Source:RefSeq mRNA;Acc:NM_001075856]
Uncharacterized protein [Source:UniProtKB/TrEMBL;Acc:F1N3R2]
Bos taurus histone cluster 1, H1c (HIST1H1C), mRNA. [Source:RefSeq mRNA;Acc:NM_001083425]
Bos taurus POU class 5 homeobox 1 (POU5F1), mRNA. [Source:RefSeq mRNA;Acc:NM_174580]

CXXC finger protein 4 [Source:HGNC Symbol;Acc:HGNC:24593]
Uncharacterized protein [Source:UniProtKB/TrEMBL;Acc:E1B7S4]
Uncharacterized protein [Source:UniProtKB/TrEMBL;Acc:G3MZV1]
transmembrane protein 235 [Source:HGNC Symbol;Acc:HGNC:27563]
Bos taurus fibroblast growth factor 16 (FGF16), mRNA. [Source:RefSeq mRNA;Acc:NM_001192777]
Bos taurus inhibin, alpha (INHA), mRNA. [Source:RefSeq mRNA;Acc:NM_174094]
Bos taurus zinc finger protein 830 (ZNF830), mRNA. [Source:RefSeq mRNA;Acc:NM_001082612]
Uncharacterized protein [Source:UniProtKB/TrEMBL;Acc:E1BAB2]
Bos taurus Ras-related associated with diabetes (RRAD), mRNA. [Source:RefSeq mRNA;Acc:NM_001045913]
protein phosphatase 1, regulatory subunit 26 [Source:HGNC Symbol;Acc:HGNC:29089]
Bos taurus angiopoietin-like 6 (ANGPTL6), mRNA. [Source:RefSeq mRNA;Acc:NM_001128070]
Bos taurus phosphatidic acid phosphatase type 2 domain containing 2 (PPAPDC2), mRNA. [Source:RefSeq mRNA;Acc:NM_001024573]
Bos taurus hexamethylene bis-acetamide inducible 2 (HEXIM2), mRNA. [Source:RefSeq mRNA;Acc:NM_001193043]
Histone H1.1 [Source:UniProtKB/Swiss-Prot;Acc:G3N131]

Bos taurus lymphocyte antigen 6 complex, locus D (LY6D), mRNA. [Source:RefSeq mRNA;Acc:NM_001076517]
Bos taurus tubulin, beta 1 class VI (TUBB1), mRNA. [Source:RefSeq mRNA;Acc:NM_001144100]
Bos taurus translocase of inner mitochondrial membrane 10 homolog (yeast) (TIMM10), mRNA. [Source:RefSeq mRNA;Acc:NM_001046132]
Bos taurus chromosome 1 open reading frame, human C21orf2 (C1H21orf2), mRNA. [Source:RefSeq mRNA;Acc:NM_001075781]
Bos taurus thioesterase superfamily member 6 (THEM6), mRNA. [Source:RefSeq mRNA;Acc:NM_001099375]
7SK RNA [Source:RFAM;Acc:RF00100]

ribosomal protein L38 [Source:HGNC Symbol;Acc:HGNC:10349]
suppressor of cytokine signaling 6 [Source:HGNC Symbol;Acc:HGNC:16833]
Bos taurus male-enhanced antigen 1 (MEA1), mRNA. [Source:RefSeq mRNA;Acc:NM_174648]
Bos taurus splicing factor 3b, subunit 5, 10kDa (SF3B5), mRNA. [Source:RefSeq mRNA;Acc:NM_001025350]
Bos taurus uncharacterized LOC100196902 (LOC100196902), mRNA. [Source:RefSeq mRNA;Acc:NM_001143749]

Bos taurus Rho GDP dissociation inhibitor (GDI) gamma (ARHGDIG), mRNA. [Source:RefSeq mRNA;Acc:NM_001076286]
Bos taurus transmembrane protein 171 (TMEM171), mRNA. [Source:RefSeq mRNA;Acc:NM_001014951]
AT rich interactive domain 3C (BRIGHT-like) [Source:HGNC Symbol;Acc:HGNC:21209]
Bos taurus leucine rich repeat containing 8 family, member A (LRRC8A), mRNA. [Source:RefSeq mRNA;Acc:NM_001076807]
G protein-coupled receptor 101 [Source:HGNC Symbol;Acc:HGNC:14963]
MAX dimerization protein 3 [Source:HGNC Symbol;Acc:HGNC:14008]
proline-rich transmembrane protein 4 [Source:HGNC Symbol;Acc:HGNC:37280]
family with sequence similarity 69, member B [Source:HGNC Symbol;Acc:HGNC:28290]
kelch-like family member 34 [Source:HGNC Symbol;Acc:HGNC:26634]
Bos taurus homeobox C5 (HOXC5), mRNA. [Source:RefSeq mRNA;Acc:NM_001191351]
tripartite motif containing 72, E3 ubiquitin protein ligase [Source:HGNC Symbol;Acc:HGNC:32671]
leucine-rich repeats and transmembrane domains 2 [Source:HGNC Symbol;Acc:HGNC:32443]
lymphotoxin beta (TNF superfamily, member 3) [Source:HGNC Symbol;Acc:HGNC:6711]
Bos taurus uncharacterized LOC515358 (LOC515358), mRNA. [Source:RefSeq mRNA;Acc:NM_001100368]
H6 family homeobox 3 [Source:HGNC Symbol;Acc:HGNC:5019]
synaptotagmin VI [Source:HGNC Symbol;Acc:HGNC:18638]
tigger transposable element derived 3 [Source:HGNC Symbol;Acc:HGNC:18334]
retinoic acid induced 2 [Source:HGNC Symbol;Acc:HGNC:9835]
ribosomal protein S16 [Source:HGNC Symbol;Acc:HGNC:10396]
Uncharacterized protein [Source:UniProtKB/TrEMBL;Acc:E1BAF8]
Bos taurus family with sequence similarity 195, member A (FAM195A), mRNA. [Source:RefSeq mRNA;Acc:NM_001079511]
Uncharacterized protein [Source:UniProtKB/TrEMBL;Acc:E1B9G9]
transmembrane protein 175 [Source:RefSeq peptide;Acc:NP_001069081]
Bos taurus bystin-like (BYSL), mRNA. [Source:RefSeq mRNA;Acc:NM_001014924]

Bos taurus ribosomal protein L36a-like (RPL36A), mRNA. [Source:RefSeq mRNA;Acc:NM_001034316]
Bos taurus fem-1 homolog a (C. elegans) (FEM1A), mRNA. [Source:RefSeq mRNA;Acc:NM_001046226]

transmembrane protein 200C [Source:HGNC Symbol;Acc:HGNC:37208]
Bos taurus serine/threonine kinase 25 (STK25), mRNA. [Source:RefSeq mRNA;Acc:NM_183407]
TLR4 interactor with leucine-rich repeats [Source:HGNC Symbol;Acc:HGNC:22200]
Bos taurus family with sequence similarity 110, member A (FAM110A), mRNA. [Source:RefSeq mRNA;Acc:NM_001014961]
Uncharacterized protein [Source:UniProtKB/TrEMBL;Acc:G3N3U4]
Bos taurus RAS-like, family 10, member A (RASL10A), mRNA. [Source:RefSeq mRNA;Acc:NM_001083645]
Bos taurus uncharacterized LOC618701 (LOC618701), mRNA. [Source:RefSeq mRNA;Acc:NM_001099723]
Bos taurus GRIP and coiled-coil domain containing 1 (GCC1), mRNA. [Source:RefSeq mRNA;Acc:NM_001205744]
Bos taurus cyclin-dependent kinase inhibitor 2D (p19, inhibits CDK4) (CDKN2D), mRNA. [Source:RefSeq mRNA;Acc:NM_001046050]
Bos taurus TBC1 domain family, member 10C (TBC1D10C), mRNA. [Source:RefSeq mRNA;Acc:NM_001102060]
zinc finger and BTB domain containing 47 [Source:HGNC Symbol;Acc:HGNC:26955]
Bos taurus programmed cell death 7 (PDCD7), mRNA. [Source:RefSeq mRNA;Acc:NM_001205449]
family with sequence similarity 110, member B [Source:HGNC Symbol;Acc:HGNC:28587]
Uncharacterized protein [Source:UniProtKB/TrEMBL;Acc:G3MYW8]
Bos taurus H2A histone family, member J (H2AFJ), mRNA. [Source:RefSeq mRNA;Acc:NM_001078089]
Bos taurus ribosomal protein L23a (RPL23A), mRNA. [Source:RefSeq mRNA;Acc:NM_001045958]
family with sequence similarity 43, member A [Source:HGNC Symbol;Acc:HGNC:26888]
Uncharacterized protein [Source:UniProtKB/TrEMBL;Acc:G3MXM9]
Bos taurus tyrosine 3-monooxygenase/tryptophan 5-monooxygenase activation protein, gamma polypeptide (YWHAG), mRNA. [Source:RefSeq mRNA;Acc:NM_
Uncharacterized protein [Source:UniProtKB/TrEMBL;Acc:G3N1F3]
Uncharacterized protein [Source:UniProtKB/TrEMBL;Acc:E1B871]

purinergic receptor P2Y, G-protein coupled, 8 [Source:HGNC Symbol;Acc:HGNC:15524]
Bos taurus mediator complex subunit 19 (MED19), mRNA. [Source:RefSeq mRNA;Acc:NM_001105348]
Bos taurus MPV17 mitochondrial membrane protein-like 2 (MPV17L2), mRNA. [Source:RefSeq mRNA;Acc:NM_001098145]
retrotransposon gag domain containing 4 [Source:HGNC Symbol;Acc:HGNC:29430]
G protein-coupled receptor 3 [Source:HGNC Symbol;Acc:HGNC:4484]

Bos taurus zinc finger protein 133 (ZNF133), mRNA. [Source:RefSeq mRNA;Acc:NM_001110090]

Bos taurus ribosomal protein L19 (RPL19), mRNA. [Source:RefSeq mRNA;Acc:NM_001040516]
Bos taurus polyglutamine binding protein 1 (PQBP1), mRNA. [Source:RefSeq mRNA;Acc:NM_001046389]
Bos taurus polymerase (DNA-directed), epsilon 4 (p12 subunit) (POLE4), mRNA. [Source:RefSeq mRNA;Acc:NM_001101225]
H1 histone family, member O, oocyte-specific [Source:HGNC Symbol;Acc:HGNC:18463]
cytochrome c oxidase assembly factor 7 (putative) [Source:HGNC Symbol;Acc:HGNC:25716]
Bos taurus chromobox homolog 7 (CBX7), mRNA. [Source:RefSeq mRNA;Acc:NM_001192618]
Bos taurus aquaporin 11 (AQP11), mRNA. [Source:RefSeq mRNA;Acc:NM_001110069]
Bos taurus chromosome 14 open reading frame, human C8orf59 (C14H8orf59), mRNA. [Source:RefSeq mRNA;Acc:NM_001110068]
Bos taurus RNA binding motif protein 15B (RBM15B), mRNA. [Source:RefSeq mRNA;Acc:NM_001206581]
Bos taurus mitochondrial ribosomal protein L11 (MRPL11), mRNA. [Source:RefSeq mRNA;Acc:NM_001014932]
T cell receptor alpha variable 14/delta variable 4 [Source:HGNC Symbol;Acc:HGNC:12110]
SAC3 domain containing 1 [Source:HGNC Symbol;Acc:HGNC:30179]
Bos taurus transcription factor 15 (basic helix-loop-helix) (TCF15), mRNA. [Source:RefSeq mRNA;Acc:NM_001081721]
Bos taurus RNA binding motif protein 4 (RBM4), mRNA. [Source:RefSeq mRNA;Acc:NM_001077010]
insulin receptor substrate 1 [Source:HGNC Symbol;Acc:HGNC:6125]
Uncharacterized protein [Source:UniProtKB/TrEMBL;Acc:E1BHT3]
Bos taurus chromosome 16 open reading frame, human C1orf93 (FAM213B), mRNA. [Source:RefSeq mRNA;Acc:NM_001040598]
Solute carrier family 10 member 6 [Source:UniProtKB/Swiss-Prot;Acc:A6QP84]
Bos taurus potassium voltage-gated channel, Isk-related family, member 1 (KCNE1), mRNA. [Source:RefSeq mRNA;Acc:NM_001077977]
Bos taurus radial spoke head 9 homolog (Chlamydomonas) (RSPH9), mRNA. [Source:RefSeq mRNA;Acc:NM_001046267]
meningioma (disrupted in balanced translocation) 1 [Source:HGNC Symbol;Acc:HGNC:7180]
Bos taurus caveolin 2 (CAV2), mRNA. [Source:RefSeq mRNA;Acc:NM_001007808]
Bos taurus TCF3 (E2A) fusion partner (in childhood Leukemia) (TFPT), mRNA. [Source:RefSeq mRNA;Acc:NM_001075274]
Bos taurus outer dense fiber of sperm tails 1 (ODF1), mRNA. [Source:RefSeq mRNA;Acc:NM_174131]
Bos taurus armadillo repeat containing 7 (ARMC7), mRNA. [Source:RefSeq mRNA;Acc:NM_001083504]

Bos taurus vasorin (VASN), mRNA. [Source:RefSeq mRNA;Acc:NM_001083796]
Bos taurus fracture callus 1 homolog (rat) (FXC1), mRNA. [Source:RefSeq mRNA;Acc:NM_001040530]

Uncharacterized protein [Source:UniProtKB/TrEMBL;Acc:G5E613]
Bos taurus charged multivesicular body protein 2A (CHMP2A), mRNA. [Source:RefSeq mRNA;Acc:NM_001034550]
chromosome 11 open reading frame 53 [Source:HGNC Symbol;Acc:HGNC:30527]
kallikrein-related peptidase 15 [Source:HGNC Symbol;Acc:HGNC:20453]
olfactory receptor, family 5, subfamily C, member 1 [Source:HGNC Symbol;Acc:HGNC:8331]
Bos taurus tubulin, beta 3 class III (TUBB3), mRNA. [Source:RefSeq mRNA;Acc:NM_001077127]
Bos taurus claudin 3 (CLDN3), mRNA. [Source:RefSeq mRNA;Acc:NM_205801]
centromere protein P [Source:RefSeq peptide;Acc:NP_001099085]
archaelysin family metallopeptidase 1 [Source:HGNC Symbol;Acc:HGNC:22231]
Bos taurus transmembrane protein 42 (TMEM42), mRNA. [Source:RefSeq mRNA;Acc:NM_001076298]
Olfactory receptor [Source:UniProtKB/TrEMBL;Acc:G5E579]
translocase of inner mitochondrial membrane 13 homolog (yeast) [Source:HGNC Symbol;Acc:HGNC:11816]
Uncharacterized protein [Source:UniProtKB/TrEMBL;Acc:G3N106]
calcium binding protein 7 [Source:HGNC Symbol;Acc:HGNC:20834]
Bos taurus transmembrane protein 86A (TMEM86A), mRNA. [Source:RefSeq mRNA;Acc:NM_001035388]
wingless-type MMTV integration site family, member 3A [Source:HGNC Symbol;Acc:HGNC:15983]
Bos taurus BTB (POZ) domain containing 6 (BTBD6), mRNA. [Source:RefSeq mRNA;Acc:NM_001105050]

Bos taurus Sp9 transcription factor homolog (mouse) (SP9), mRNA. [Source:RefSeq mRNA;Acc:NM_001205839]
Bos taurus vertebrae development homolog (pig) (VRTN), mRNA. [Source:RefSeq mRNA;Acc:NM_001206630]
Bos taurus netrin 3 (NTN3), mRNA. [Source:RefSeq mRNA;Acc:NM_001206905]
Bos taurus RNA binding motif protein, X-linked-like 2 (RBMXL2), mRNA. [Source:RefSeq mRNA;Acc:NM_001046610]
Histone H2B [Source:UniProtKB/TrEMBL;Acc:G5E6I9]
low density lipoprotein receptor class A domain containing 2 [Source:HGNC Symbol;Acc:HGNC:32071]
OTU deubiquitinase 1 [Source:HGNC Symbol;Acc:HGNC:27346]
transmembrane protein 74 [Source:HGNC Symbol;Acc:HGNC:26409]
Kruppel-like factor 9 [Source:HGNC Symbol;Acc:HGNC:1123]
ADP-ribosylation factor-like 6 interacting protein 4 [Source:HGNC Symbol;Acc:HGNC:18076]
Uncharacterized protein [Source:UniProtKB/TrEMBL;Acc:F1MB13]
thyrotrophic embryonic factor [Source:HGNC Symbol;Acc:HGNC:11722]

Bos taurus tetraspanin 3 (TSPAN3), mRNA. [Source:RefSeq mRNA;Acc:NM_001035487]

Bos taurus POU class 3 homeobox 2 (POU3F2), mRNA. [Source:RefSeq mRNA;Acc:NM_001080348]
Bos taurus guanylate cyclase activator 2B (uroguanylin) (GUCA2B), mRNA. [Source:RefSeq mRNA;Acc:NM_001205816]

carcinoembryonic antigen-related cell adhesion molecule 16 [Source:HGNC Symbol;Acc:HGNC:31948]
Intraflagellar transport protein 27 homolog [Source:UniProtKB/Swiss-Prot;Acc:Q0VCN3]
Bos taurus GH3 domain containing (GHDC), mRNA. [Source:RefSeq mRNA;Acc:NM_001075703]
enoyl CoA hydratase domain containing 1 [Source:HGNC Symbol;Acc:HGNC:21489]
Bos taurus leucine rich repeat containing 59 (LRRC59), mRNA. [Source:RefSeq mRNA;Acc:NM_001034578]
Bos taurus Kruppel-like factor 7 (ubiquitous) (KLF7), mRNA. [Source:RefSeq mRNA;Acc:NM_001192919]
Bos taurus melanoma-associated antigen 10-like (LOC787822), mRNA. [Source:RefSeq mRNA;Acc:NM_001101301]
Bos taurus progastricsin (pepsinogen C) (PGC), mRNA. [Source:RefSeq mRNA;Acc:NM_001205791]
Bos taurus cell death-inducing DFFA-like effector a (CIDEA), mRNA. [Source:RefSeq mRNA;Acc:NM_001083449]
smoothelin-like 1 [Source:HGNC Symbol;Acc:HGNC:32394]
iroquois homeobox 1 [Source:HGNC Symbol;Acc:HGNC:14358]
transmembrane protein 200B [Source:HGNC Symbol;Acc:HGNC:33785]
ribosomal protein L22 [Source:HGNC Symbol;Acc:HGNC:10315]
KH and NYN domain containing [Source:HGNC Symbol;Acc:HGNC:20166]
Bos taurus docking protein 1, 62kDa (downstream of tyrosine kinase 1) (DOK1), mRNA. [Source:RefSeq mRNA;Acc:NM_001024543]
NLR family, pyrin domain containing 6 [Source:HGNC Symbol;Acc:HGNC:22944]
Bos taurus leucine rich repeat containing 41 (LRRC41), mRNA. [Source:RefSeq mRNA;Acc:NM_001045873]
Bos taurus gap junction protein, alpha 3, 46kDa (GJA3), mRNA. [Source:RefSeq mRNA;Acc:NM_001166296]
Bos taurus interleukin 1, beta (IL1B), mRNA. [Source:RefSeq mRNA;Acc:NM_174093]
Bos taurus chromosome 23 open reading frame, human C6orf47 (C23H6orf47), mRNA. [Source:RefSeq mRNA;Acc:NM_001101267]
Uncharacterized protein [Source:UniProtKB/TrEMBL;Acc:F1N130]
Uncharacterized protein [Source:UniProtKB/TrEMBL;Acc:E1BP06]
Bos taurus ras homolog gene family, member C (RHOC), mRNA. [Source:RefSeq mRNA;Acc:NM_001046138]
chromosome 12 open reading frame 43 [Source:HGNC Symbol;Acc:HGNC:25719]
Bos taurus BarH-like homeobox 2 (BARHL2), mRNA. [Source:RefSeq mRNA;Acc:NM_001192297]

frizzled class receptor 2 [Source:HGNC Symbol;Acc:HGNC:4040]
bicaudal D homolog 2 (Drosophila) [Source:HGNC Symbol;Acc:HGNC:17208]
Uncharacterized protein [Source:UniProtKB/TrEMBL;Acc:F6RCH9]
acrosin [Source:HGNC Symbol;Acc:HGNC:126]
Bos taurus mitochondrial ribosomal protein L28 (MRPL28), mRNA. [Source:RefSeq mRNA;Acc:NM_001045996]

Bos taurus aquaporin 3 (Gill blood group) (AQP3), mRNA. [Source:RefSeq mRNA;Acc:NM_001079794]

UFM1-specific peptidase 1 (non-functional) [Source:HGNC Symbol;Acc:HGNC:33821]
Bos taurus gap junction protein, beta 4, 30.3kDa (GJB4), mRNA. [Source:RefSeq mRNA;Acc:NM_001191371]
Bos taurus indolethylamine N-methyltransferase (INMT), mRNA. [Source:RefSeq mRNA;Acc:NM_001192648]
Bos taurus EH-domain containing 4 (EHD4), mRNA. [Source:RefSeq mRNA;Acc:NM_001192053]
Uncharacterized protein [Source:UniProtKB/TrEMBL;Acc:G3MWP5]
defensin, beta 112 [Source:HGNC Symbol;Acc:HGNC:18093]
Uncharacterized protein [Source:UniProtKB/TrEMBL;Acc:G3MYV8]
chromosome 15 open reading frame 39 [Source:HGNC Symbol;Acc:HGNC:24497]
Bos taurus cytotoxic T-lymphocyte-associated protein 4 (CTLA4), mRNA. [Source:RefSeq mRNA;Acc:NM_174297]

Bos taurus polymerase (RNA) II (DNA directed) polypeptide H (POLR2H), mRNA. [Source:RefSeq mRNA;Acc:NM_001192065]
Bos taurus tetratricopeptide repeat domain 36 (TTC36), mRNA. [Source:RefSeq mRNA;Acc:NM_001040515]
mitochondrial ribosomal protein L40 [Source:HGNC Symbol;Acc:HGNC:14491]
Uncharacterized protein [Source:UniProtKB/TrEMBL;Acc:G3MYA4]

v-maf avian musculoaponeurotic fibrosarcoma oncogene homolog B [Source:HGNC Symbol;Acc:HGNC:6408]
Bos taurus mannosyl (alpha-1,3-)-glycoprotein beta-1,2-N-acetylglucosaminyltransferase (MGAT1), mRNA. [Source:RefSeq mRNA;Acc:NM_001015653]

Histone H2B [Source:UniProtKB/TrEMBL;Acc:F1MUD2]
chromosome 12 open reading frame 65 [Source:HGNC Symbol;Acc:HGNC:26784]
Uncharacterized protein [Source:UniProtKB/TrEMBL;Acc:E1BCY5]
sterile alpha motif domain containing 10 [Source:HGNC Symbol;Acc:HGNC:16129]

Uncharacterized protein [Source:UniProtKB/TrEMBL;Acc:E1B8L4]
Bos taurus ankyrin repeat and SOCS box containing 6 (ASB6), mRNA. [Source:RefSeq mRNA;Acc:NM_001205661]

Bos taurus chromosome 11 open reading frame, human C2orf50 (C11H2orf50), transcript variant 1, mRNA. [Source:RefSeq mRNA;Acc:NM_001075480]
Bos taurus adrenergic, beta-3-, receptor (ADRB3), mRNA. [Source:RefSeq mRNA;Acc:NM_174232]
Uncharacterized protein [Source:UniProtKB/TrEMBL;Acc:F1MWU5]
Bos taurus Ets2 repressor factor (ERF), mRNA. [Source:RefSeq mRNA;Acc:NM_001205465]
family with sequence similarity 83, member G [Source:HGNC Symbol;Acc:HGNC:32554]
SH2 domain containing 5 [Source:HGNC Symbol;Acc:HGNC:28819]
free fatty acid receptor 4 [Source:HGNC Symbol;Acc:HGNC:19061]
splA/ryanodine receptor domain and SOCS box containing 1 [Source:HGNC Symbol;Acc:HGNC:30628]
Uncharacterized protein [Source:UniProtKB/TrEMBL;Acc:F1MJB5]
butyrobetaine (gamma), 2-oxoglutarate dioxygenase (gamma-butyrobetaine hydroxylase) 1 [Source:HGNC Symbol;Acc:HGNC:964]
chromosome 14 open reading frame 180 [Source:HGNC Symbol;Acc:HGNC:33795]
transmembrane protein 61 [Source:HGNC Symbol;Acc:HGNC:27296]
cardiotrophin-like cytokine factor 1 [Source:HGNC Symbol;Acc:HGNC:17412]
deafness, autosomal recessive 31 [Source:HGNC Symbol;Acc:HGNC:16361]

G protein-coupled receptor 31 [Source:HGNC Symbol;Acc:HGNC:4486]
Bos taurus CCAAT/enhancer binding protein (C/EBP), gamma (CEBPG), mRNA. [Source:RefSeq mRNA;Acc:NM_001034801]
7SK RNA [Source:RFAM;Acc:RF00100]
Bos taurus chromosome 7 open reading frame, human C19orf24 (C7H19orf24), mRNA. [Source:RefSeq mRNA;Acc:NM_001206241]
Olfactory receptor [Source:UniProtKB/TrEMBL;Acc:G5E5K4]
Bos taurus insulin-like growth factor binding protein, acid labile subunit (IGFALS), mRNA. [Source:RefSeq mRNA;Acc:NM_001075963]

Bos taurus arginine vasopressin-induced 1 (AVPI1), mRNA. [Source:RefSeq mRNA;Acc:NM_001034760]
forkhead box F2 [Source:HGNC Symbol;Acc:HGNC:3810]

zinc finger protein 771 [Source:HGNC Symbol;Acc:HGNC:29653]

coiled-coil domain-containing protein 8 [Source:RefSeq peptide;Acc:NP_001030562]
Bos taurus myosin, light chain 6, alkali, smooth muscle and non-muscle (MYL6), mRNA. [Source:RefSeq mRNA;Acc:NM_175780]

Bos taurus AT hook, DNA binding motif, containing 1 (AHDC1), mRNA. [Source:RefSeq mRNA;Acc:NM_001206409]
ring finger protein 208 [Source:HGNC Symbol;Acc:HGNC:25420]
leucine carboxyl methyltransferase 2 [Source:HGNC Symbol;Acc:HGNC:17558]

ribosomal protein S26 [Source:HGNC Symbol;Acc:HGNC:10414]
bromo adjacent homology domain containing 1 [Source:HGNC Symbol;Acc:HGNC:29153]
VGF nerve growth factor inducible [Source:HGNC Symbol;Acc:HGNC:12684]
Bos taurus ras homolog gene family, member B (RHOB), mRNA. [Source:RefSeq mRNA;Acc:NM_001077922]
nuclear receptor subfamily 0, group B, member 2 [Source:HGNC Symbol;Acc:HGNC:7961]
Uncharacterized protein [Source:UniProtKB/TrEMBL;Acc:G3MY10]
transcription elongation factor A (SII)-like 7 [Source:HGNC Symbol;Acc:HGNC:28336]
Bos taurus chromosome 29 open reading frame, human C11orf68 (C29H11orf68), mRNA. [Source:RefSeq mRNA;Acc:NM_001105480]
Bos taurus crystallin, zeta (quinone reductase) (CRYZ), mRNA. [Source:RefSeq mRNA;Acc:NM_174025]
G protein-coupled receptor 20 [Source:HGNC Symbol;Acc:HGNC:4475]
potassium voltage-gated channel, delayed-rectifier, subfamily S, member 2 [Source:HGNC Symbol;Acc:HGNC:6301]
Uncharacterized protein [Source:UniProtKB/TrEMBL;Acc:F1N7Z3]
leucine rich repeat containing 32 [Source:HGNC Symbol;Acc:HGNC:4161]
Bos taurus NADH dehydrogenase (ubiquinone) 1 beta subcomplex, 2, 8kDa (NDUFB2), mRNA. [Source:RefSeq mRNA;Acc:NM_175828]
Bos taurus SH3-binding domain protein 5-like (SH3BP5L), mRNA. [Source:RefSeq mRNA;Acc:NM_001075802]
Bos taurus purinergic receptor P2Y, G-protein coupled, 1 (P2RY1), mRNA. [Source:RefSeq mRNA;Acc:NM_174410]
Bos taurus coiled-coil domain containing 71 (CCDC71), mRNA. [Source:RefSeq mRNA;Acc:NM_001046000]
mannosyl-oligosaccharide glucosidase [Source:HGNC Symbol;Acc:HGNC:24862]
Bos taurus solute carrier family 25, member 29 (SLC25A29), mRNA. [Source:RefSeq mRNA;Acc:NM_001077871]
Olfactory receptor [Source:UniProtKB/TrEMBL;Acc:F1MLN0]
Bos taurus cathepsin H (CTSH), mRNA. [Source:RefSeq mRNA;Acc:NM_001034385]
Bos taurus zinc finger protein 704 (ZNF704), mRNA. [Source:RefSeq mRNA;Acc:NM_001205753]

wingless-type MMTV integration site family, member 10A [Source:HGNC Symbol;Acc:HGNC:13829]
Bos taurus SH3-domain GRB2-like endophilin B2 (SH3GLB2), mRNA. [Source:RefSeq mRNA;Acc:NM_001076802]
Bos taurus replication protein A2, 32kDa (RPA2), mRNA. [Source:RefSeq mRNA;Acc:NM_001045984]
Bos taurus ventral anterior homeobox 2 (VAX2), mRNA. [Source:RefSeq mRNA;Acc:NM_001192313]
Uncharacterized protein [Source:UniProtKB/TrEMBL;Acc:F1MHW5]
Bos taurus cysteine-rich C-terminal 1 (CRCT1), mRNA. [Source:RefSeq mRNA;Acc:NM_001077520]
Bos taurus tribbles homolog 3 (Drosophila) (TRIB3), mRNA. [Source:RefSeq mRNA;Acc:NM_001076103]
Bos taurus ribosomal protein, large, P1 (RPLP1), mRNA. [Source:RefSeq mRNA;Acc:NM_001025340]
Bos taurus glycine/arginine rich protein 1 (FAM110D), mRNA. [Source:RefSeq mRNA;Acc:NM_001099203]
Bos taurus transmembrane protein 11 (TMEM11), mRNA. [Source:RefSeq mRNA;Acc:NM_001097998]
Bos taurus potassium channel, subfamily K, member 7 (KCNK7), mRNA. [Source:RefSeq mRNA;Acc:NM_001192725]
Bos taurus SAYSVFN motif domain containing 1 (SAYSD1), mRNA. [Source:RefSeq mRNA;Acc:NM_001038225]
Bos taurus ubiquitin-conjugating enzyme E2M (UBE2M), mRNA. [Source:RefSeq mRNA;Acc:NM_001083503]
Bos taurus Morf4 family associated protein 1 (MRFAP1), mRNA. [Source:RefSeq mRNA;Acc:NM_001035344]
Bos taurus dysbindin (dystrobrevin binding protein 1) domain containing 2 (DBNDD2), mRNA. [Source:RefSeq mRNA;Acc:NM_001130748]
Uncharacterized protein [Source:UniProtKB/TrEMBL;Acc:G3MYG8]
Uncharacterized protein [Source:UniProtKB/TrEMBL;Acc:G3X7N7]
Bos taurus F-box protein 44 (FBXO44), mRNA. [Source:RefSeq mRNA;Acc:NM_001101295]
Bos taurus translocase of outer mitochondrial membrane 5 homolog (yeast) (TOMM5), mRNA. [Source:RefSeq mRNA;Acc:NM_001143740]
Bos taurus gap junction protein, beta 3, 31kDa (GJB3), mRNA. [Source:RefSeq mRNA;Acc:NM_001104995]
Uncharacterized protein [Source:UniProtKB/TrEMBL;Acc:F1MLV6]
zinc finger and BTB domain containing 42 [Source:HGNC Symbol;Acc:HGNC:32550]
oxoeicosanoid (OXE) receptor 1 [Source:HGNC Symbol;Acc:HGNC:24884]
ADP-ribosylation factor GTPase-activating protein 2; ARFGAP2 protein [Source:UniProtKB/TrEMBL;Acc:A6QR32]
Uncharacterized protein [Source:UniProtKB/TrEMBL;Acc:F1N6X9]

Uncharacterized protein [Source:UniProtKB/TrEMBL;Acc:G3MZ13]
Bos taurus ATPase, H+ transporting, lysosomal 16kDa, V0 subunit c (ATP6V0C), mRNA. [Source:RefSeq mRNA;Acc:NM_001017954]
Uncharacterized protein [Source:UniProtKB/TrEMBL;Acc:G3MZB2]
Bos taurus ATPase, Na+/K+ transporting, beta 2 polypeptide (ATP1B2), mRNA. [Source:RefSeq mRNA;Acc:NM_174677]

Bos taurus solute carrier family 35, member C1 (SLC35C1), mRNA. [Source:RefSeq mRNA;Acc:NM_001101210]

Bos taurus jun proto-oncogene (JUN), mRNA. [Source:RefSeq mRNA;Acc:NM_001077827]
Bos taurus LIM homeobox 1 (LHX1), mRNA. [Source:RefSeq mRNA;Acc:NM_001105447]
Ig-like V-type domain-containing protein FAM187A [Source:UniProtKB/Swiss-Prot;Acc:A7E3C4]
Bos taurus XK, Kell blood group complex subunit-related family, member 7 (XKR7), mRNA. [Source:RefSeq mRNA;Acc:NM_001205845]
Bos taurus late endosomal/lysosomal adaptor, MAPK and MTOR activator 1 (LAMTOR1), mRNA. [Source:RefSeq mRNA;Acc:NM_001034769]
Bos taurus zinc finger protein 593 (ZNF593), mRNA. [Source:RefSeq mRNA;Acc:NM_001192186]
Bos taurus cyclin D2 (CCND2), mRNA. [Source:RefSeq mRNA;Acc:NM_001076372]
Uncharacterized protein [Source:UniProtKB/TrEMBL;Acc:E1B8I0]
Bos taurus cell cycle exit and neuronal differentiation 1 (CEND1), mRNA. [Source:RefSeq mRNA;Acc:NM_001080222]
glutamate receptor, ionotropic, N-methyl D-aspartate 2C [Source:HGNC Symbol;Acc:HGNC:4587]
Bos taurus claudin 19 (CLDN19), mRNA. [Source:RefSeq mRNA;Acc:NM_001101084]
olfactory receptor, family 4, subfamily Q, member 3 [Source:HGNC Symbol;Acc:HGNC:15426]
7SK RNA [Source:RFAM;Acc:RF00100]
hypermethylated in cancer 2 [Source:HGNC Symbol;Acc:HGNC:18595]
Uncharacterized protein [Source:UniProtKB/TrEMBL;Acc:E1BJN5]
60S ribosomal protein L18a [Source:UniProtKB/TrEMBL;Acc:F1MHJ6]
Bos taurus hepatic leukemia factor (HLF), mRNA. [Source:RefSeq mRNA;Acc:NM_001192437]
forkhead box N1 [Source:HGNC Symbol;Acc:HGNC:12765]
Olfactory receptor [Source:UniProtKB/TrEMBL;Acc:F1MVH2]
Bos taurus arginine and glutamate rich 1 (ARGLU1), mRNA. [Source:RefSeq mRNA;Acc:NM_001038190]
hydroxypyruvate isomerase (putative) [Source:HGNC Symbol;Acc:HGNC:26948]
Bos taurus galactose-3-O-sulfotransferase 3 (GAL3ST3), mRNA. [Source:RefSeq mRNA;Acc:NM_001075840]
Bos taurus transcription factor 21 (TCF21), mRNA. [Source:RefSeq mRNA;Acc:NM_001014899]
tumor necrosis factor receptor superfamily, member 13B [Source:HGNC Symbol;Acc:HGNC:18153]
Uncharacterized protein [Source:UniProtKB/TrEMBL;Acc:E1BJN0]
Bos taurus capping protein (actin filament), gelsolin-like (CAPG), mRNA. [Source:RefSeq mRNA;Acc:NM_178574]
Bos taurus leukocyte receptor cluster (LRC) member 8 (LENG8), mRNA. [Source:RefSeq mRNA;Acc:NM_001102100]

Uncharacterized protein [Source:UniProtKB/TrEMBL;Acc:E1BB12]
Uncharacterized protein [Source:UniProtKB/TrEMBL;Acc:G3MWY4]
7SK RNA [Source:RFAM;Acc:RF00100]
chondroitin polymerizing factor [Source:HGNC Symbol;Acc:HGNC:24291]
Bos taurus peptidoglycan recognition protein 1 (PGLYRP1), mRNA. [Source:RefSeq mRNA;Acc:NM_174573]
Uncharacterized protein [Source:UniProtKB/TrEMBL;Acc:F1N6T9]
synaptopodin [Source:HGNC Symbol;Acc:HGNC:30672]
Olfactory receptor [Source:UniProtKB/TrEMBL;Acc:F1N2Y1]
Bos taurus solute carrier family 35, member B2 (SLC35B2), mRNA. [Source:RefSeq mRNA;Acc:NM_001076965]
zinc finger and BTB domain containing 4 [Source:HGNC Symbol;Acc:HGNC:23847]
Bos taurus gap junction protein, delta 2, 36kDa (GJD2), mRNA. [Source:RefSeq mRNA;Acc:NM_174683]

Uncharacterized protein [Source:UniProtKB/TrEMBL;Acc:E1BBW6]

Histone H2B [Source:UniProtKB/TrEMBL;Acc:G3MXG7]
Bos taurus acetylcholinesterase (ACHE), mRNA. [Source:RefSeq mRNA;Acc:NM_001076220]

Bos taurus ribosomal protein S10 (RPS10), mRNA. [Source:RefSeq mRNA;Acc:NM_001034716]
Bos taurus insulin-like growth factor binding protein 1 (IGFBP1), mRNA. [Source:RefSeq mRNA;Acc:NM_174554]
ripply transcriptional repressor 3 [Source:HGNC Symbol;Acc:HGNC:3047]
ladybird homeobox 1 [Source:HGNC Symbol;Acc:HGNC:16960]
Bos taurus msh homeobox 2 (MSX2), mRNA. [Source:RefSeq mRNA;Acc:NM_001079614]
Bos taurus UTP14, U3 small nucleolar ribonucleoprotein, homolog A (yeast) (UTP14A), mRNA. [Source:RefSeq mRNA;Acc:NM_001100302]
Bos taurus growth factor, augmenter of liver regeneration (GFER), mRNA. [Source:RefSeq mRNA;Acc:NM_001193188]
Bos taurus cytochrome b5 type B (outer mitochondrial membrane) (CYB5B), mRNA. [Source:RefSeq mRNA;Acc:NM_001163782]
Bos taurus tripartite motif containing 8 (TRIM8), mRNA. [Source:RefSeq mRNA;Acc:NM_001191476]
Bos taurus T-box 2 (TBX2), mRNA. [Source:RefSeq mRNA;Acc:NM_001191443]
Bos taurus mitochondrial ribosomal protein L17 (MRPL17), mRNA. [Source:RefSeq mRNA;Acc:NM_001034274]
lipocalin 15 [Source:HGNC Symbol;Acc:HGNC:33777]
Uncharacterized protein [Source:UniProtKB/TrEMBL;Acc:G3MZS8]

Bos taurus G protein-coupled receptor 62 (GPR62), mRNA. [Source:RefSeq mRNA;Acc:NM_001101238]
hypermethylated in cancer 1 [Source:HGNC Symbol;Acc:HGNC:4909]
Bos taurus ribosomal protein L17 (RPL17), mRNA. [Source:RefSeq mRNA;Acc:NM_001034459]
Bos taurus calmodulin-like 5 (CALML5), mRNA. [Source:RefSeq mRNA;Acc:NM_001098049]
Bos taurus glycine N-methyltransferase (GNMT), mRNA. [Source:RefSeq mRNA;Acc:NM_001206116]
Bos taurus protease, serine, 23 (PRSS23), mRNA. [Source:RefSeq mRNA;Acc:NM_001080306]
Bos taurus MID1 interacting protein 1 (gastrulation specific G12 homolog (zebrafish)) (MID1IP1), mRNA. [Source:RefSeq mRNA;Acc:NM_001076383]
zinc finger and BTB domain containing 2 [Source:HGNC Symbol;Acc:HGNC:20868]
Bos taurus tubulin polymerization-promoting protein family member 3 (TPPP3), mRNA. [Source:RefSeq mRNA;Acc:NM_001034774]
Bos taurus ubiquitin-like 4A (UBL4A), mRNA. [Source:RefSeq mRNA;Acc:NM_001034214]
sphingosine-1-phosphate receptor 5 [Source:HGNC Symbol;Acc:HGNC:14299]
Bos taurus myeloid-associated differentiation marker-like 2 (MYADML2), mRNA. [Source:RefSeq mRNA;Acc:NM_001076948]
Bos taurus serine/arginine-rich splicing factor 9 (SRSF9), mRNA. [Source:RefSeq mRNA;Acc:NM_001083398]

Bos taurus prolactin releasing hormone receptor (PRLHR), mRNA. [Source:RefSeq mRNA;Acc:NM_001030300]
zinc finger protein 316 [Source:HGNC Symbol;Acc:HGNC:13843]
Bos taurus potassium channel, subfamily K, member 15 (KCNK15), mRNA. [Source:RefSeq mRNA;Acc:NM_001206546]
Bos taurus endozepine-like peptide 2 (DBIL5), mRNA. [Source:RefSeq mRNA;Acc:NM_178322]
leucine rich repeat containing 14B [Source:HGNC Symbol;Acc:HGNC:37268]
Uncharacterized protein [Source:UniProtKB/TrEMBL;Acc:F1MV90]
transmembrane protein 74B [Source:HGNC Symbol;Acc:HGNC:15893]
Bos taurus mitochondrial ribosomal protein S24 (MRPS24), mRNA. [Source:RefSeq mRNA;Acc:NM_001046601]
Uncharacterized protein [Source:UniProtKB/TrEMBL;Acc:G5E5F7]
Uncharacterized protein [Source:UniProtKB/TrEMBL;Acc:F1MKJ7]
ataxin 1 [Source:HGNC Symbol;Acc:HGNC:10548]
Bos taurus zygote arrest 1 (ZAR1), mRNA. [Source:RefSeq mRNA;Acc:NM_001076203]
Bos taurus adenylosuccinate synthase like 1 (ADSSL1), mRNA. [Source:RefSeq mRNA;Acc:NM_001099192]
proline-rich basic protein 1 [Source:HGNC Symbol;Acc:HGNC:41906]
ankyrin repeat and SOCS box containing 10 [Source:HGNC Symbol;Acc:HGNC:17185]
family with sequence similarity 222, member B [Source:HGNC Symbol;Acc:HGNC:25563]

Bos taurus metallo-beta-lactamase domain containing 1 (MBLAC1), mRNA. [Source:RefSeq mRNA;Acc:NM_001075212]
glycolipid transfer protein domain containing 2 [Source:HGNC Symbol;Acc:HGNC:33756]
Uncharacterized protein [Source:UniProtKB/TrEMBL;Acc:G3N270]
Bos taurus inhibitor of DNA binding 1, dominant negative helix-loop-helix protein (ID1), mRNA. [Source:RefSeq mRNA;Acc:NM_001097568]
Peptidyl-prolyl cis-trans isomerase [Source:UniProtKB/TrEMBL;Acc:F1MUA4]
Bos taurus non-classical MHC class I antigen (BOLA-NC1), mRNA. [Source:RefSeq mRNA;Acc:NM_001105616]
Bos taurus potassium voltage-gated channel, Shab-related subfamily, member 2 (KCNB2), mRNA. [Source:RefSeq mRNA;Acc:NM_001024563]
Bos taurus G protein-coupled receptor 183 (GPR183), mRNA. [Source:RefSeq mRNA;Acc:NM_001105442]
G protein-coupled receptor 50 [Source:HGNC Symbol;Acc:HGNC:4506]
Bos taurus ring finger protein 183 (RNF183), mRNA. [Source:RefSeq mRNA;Acc:NM_001035422]
chromosome 6 open reading frame 201 [Source:HGNC Symbol;Acc:HGNC:21620]
keratin 16 [Source:HGNC Symbol;Acc:HGNC:6423]
KLF6 protein; Uncharacterized protein [Source:UniProtKB/TrEMBL;Acc:I6L9I4]
Bos taurus peptide YY (PYY), mRNA. [Source:RefSeq mRNA;Acc:NM_001040587]
Bos taurus alanine-glyoxylate aminotransferase (AGXT), mRNA. [Source:RefSeq mRNA;Acc:NM_001102355]
Bos taurus G protein-coupled receptor 65 (GPR65), mRNA. [Source:RefSeq mRNA;Acc:NM_001101303]
chromosome 16 open reading frame 46 [Source:HGNC Symbol;Acc:HGNC:26525]
Adenosine receptor A2 [Source:UniProtKB/TrEMBL;Acc:E1BKU0]
fms-related tyrosine kinase 3 ligand precursor [Source:RefSeq peptide;Acc:NP_851373]
epididymal sperm binding protein 1 [Source:HGNC Symbol;Acc:HGNC:14417]
Bos taurus endoplasmic reticulum aminopeptidase 2 (ERAP2), mRNA. [Source:RefSeq mRNA;Acc:NM_001075628]
Uncharacterized protein [Source:UniProtKB/TrEMBL;Acc:M0QVZ2]
Pim-3 proto-oncogene, serine/threonine kinase [Source:HGNC Symbol;Acc:HGNC:19310]
inositol 1,4,5-trisphosphate receptor interacting protein-like 2 [Source:HGNC Symbol;Acc:HGNC:27257]
dual specificity phosphatase 22 [Source:HGNC Symbol;Acc:HGNC:16077]
Bos taurus v-ets erythroblastosis virus E26 oncogene homolog 2 (avian) (ETS2), mRNA. [Source:RefSeq mRNA;Acc:NM_001080214]
Bos taurus chromosome 5 open reading frame, human C12orf5 (C5H12orf5), mRNA. [Source:RefSeq mRNA;Acc:NM_001076370]
Olfactory receptor [Source:UniProtKB/TrEMBL;Acc:E1BL96]
Uncharacterized protein [Source:UniProtKB/TrEMBL;Acc:F1N0Z7]

tumor necrosis factor receptor superfamily, member 8 [Source:HGNC Symbol;Acc:HGNC:11923]
glycoprotein V (platelet) [Source:HGNC Symbol;Acc:HGNC:4443]
Bos taurus single-strand-selective monofunctional uracil-DNA glycosylase 1 (SMUG1), mRNA. [Source:RefSeq mRNA;Acc:NM_001014958]
Uncharacterized protein [Source:UniProtKB/TrEMBL;Acc:F1MC69]
glutamate receptor, metabotropic 2 [Source:HGNC Symbol;Acc:HGNC:4594]
INO80 complex subunit E [Source:HGNC Symbol;Acc:HGNC:26905]
Bos taurus mannose-binding lectin (protein C) 2, soluble (MBL2), mRNA. [Source:RefSeq mRNA;Acc:NM_174107]
Uncharacterized protein [Source:UniProtKB/TrEMBL;Acc:G3N103]
Bos taurus GATA zinc finger domain containing 1 (GATAD1), mRNA. [Source:RefSeq mRNA;Acc:NM_001081623]
collagen, type XVIII, alpha 1 [Source:HGNC Symbol;Acc:HGNC:2195]
bifunctional polynucleotide phosphatase/kinase [Source:RefSeq peptide;Acc:NP_001076991]
urotensin 2 receptor [Source:HGNC Symbol;Acc:HGNC:4468]
Bos taurus calcium/calmodulin-dependent protein kinase kinase 1, alpha (CAMKK1), mRNA. [Source:RefSeq mRNA;Acc:NM_001192251]
Bos taurus neurotrophin receptor associated death domain (NRADD), mRNA. [Source:RefSeq mRNA;Acc:NM_001076398]
Uncharacterized protein [Source:UniProtKB/TrEMBL;Acc:E1BB83]
Uncharacterized protein [Source:UniProtKB/TrEMBL;Acc:E1BLI4]
Bos taurus chromosome 5 open reading frame, human C12orf44 (C5H12orf44), mRNA. [Source:RefSeq mRNA;Acc:NM_001046134]
Bos taurus acid phosphatase, testicular (ACPT), mRNA. [Source:RefSeq mRNA;Acc:NM_001206967]
Bos taurus alpha-2-fucosyltransferase Sec1 (SEC1), mRNA. [Source:RefSeq mRNA;Acc:NM_001130745]
Bos taurus GS homeobox 2 (GSX2), mRNA. [Source:RefSeq mRNA;Acc:NM_001192200]

Bos taurus NK6 homeobox 1 (NKX6-1), mRNA. [Source:RefSeq mRNA;Acc:NM_001206238]
Bos taurus hydroxysteroid (17-beta) dehydrogenase 2 (HSD17B2), mRNA. [Source:RefSeq mRNA;Acc:NM_001075726]
Bos taurus desmin (DES), mRNA. [Source:RefSeq mRNA;Acc:NM_001081575]
7SK RNA [Source:RFAM;Acc:RF00100]
wingless-type MMTV integration site family, member 11 [Source:HGNC Symbol;Acc:HGNC:12776]
somatostatin receptor 1 [Source:HGNC Symbol;Acc:HGNC:11330]
Bos taurus CD7 molecule (CD7), mRNA. [Source:RefSeq mRNA;Acc:NM_001098922]
Olfactory receptor [Source:UniProtKB/TrEMBL;Acc:F1MBK2]
Bos taurus sigma non-opioid intracellular receptor 1 (SIGMAR1), mRNA. [Source:RefSeq mRNA;Acc:NM_001034655]

Bos taurus CDC42 effector protein (Rho GTPase binding) 4 (CDC42EP4), mRNA. [Source:RefSeq mRNA;Acc:NM_001046471]
runt-related transcription factor 1 [Source:HGNC Symbol;Acc:HGNC:10471]
Bos taurus LYR motif containing 4 (LYRM4), mRNA. [Source:RefSeq mRNA;Acc:NM_001076306]
7SK RNA [Source:RFAM;Acc:RF00100]
Bos taurus integrin, beta 7 (ITGB7), mRNA. [Source:RefSeq mRNA;Acc:NM_001105365]
Olfactory receptor [Source:UniProtKB/TrEMBL;Acc:E1B8L1]
Uncharacterized protein [Source:UniProtKB/TrEMBL;Acc:F1MN26]
Uncharacterized protein [Source:UniProtKB/TrEMBL;Acc:G3N239]
paired-like homeobox 2b [Source:HGNC Symbol;Acc:HGNC:9143]
Bos taurus actin-like 9 (ACTL9), mRNA. [Source:RefSeq mRNA;Acc:NM_001038569]
unc-45 homolog B (C. elegans) [Source:HGNC Symbol;Acc:HGNC:14304]
Bos taurus obscurin, cytoskeletal calmodulin and titin-interacting RhoGEF (OBSCN), mRNA. [Source:RefSeq mRNA;Acc:NM_001102196]
immunoglobulin lambda variable 5-52 [Source:HGNC Symbol;Acc:HGNC:5926]
phospholipase A2, group XIIB [Source:HGNC Symbol;Acc:HGNC:18555]
Uncharacterized protein [Source:UniProtKB/TrEMBL;Acc:G3N378]
Bos taurus secretoglobin, family 1A, member 1 (uteroglobin) (SCGB1A1), mRNA. [Source:RefSeq mRNA;Acc:NM_001076976]
potassium channel tetramerization domain containing 14 [Source:HGNC Symbol;Acc:HGNC:23295]
glycoprotein VI (platelet) [Source:HGNC Symbol;Acc:HGNC:14388]
Uncharacterized protein [Source:UniProtKB/TrEMBL;Acc:G3MWM3]
Bos taurus retinol dehydrogenase 10 (all-trans) (RDH10), mRNA. [Source:RefSeq mRNA;Acc:NM_174734]
Bos taurus a disintegrin and metallopeptidase domain 1b (ADAM1B), mRNA. [Source:RefSeq mRNA;Acc:NM_001206471]

Myb-related transcription factor, partner of profilin [Source:HGNC Symbol;Acc:HGNC:20178]
cytochrome P450, family 26, subfamily C, polypeptide 1 [Source:HGNC Symbol;Acc:HGNC:20577]
Bos taurus forkhead box A1 (FOXA1), mRNA. [Source:RefSeq mRNA;Acc:NM_001206029]
Bos taurus actin-related protein T2 (ACTRT2), mRNA. [Source:RefSeq mRNA;Acc:NM_001038115]
Bos taurus cytochrome P450, family 8, subfamily B, polypeptide 1 (CYP8B1), mRNA. [Source:RefSeq mRNA;Acc:NM_001076139]
potassium voltage-gated channel, shaker-related subfamily, member 7 [Source:HGNC Symbol;Acc:HGNC:6226]
Bos taurus sestrin 2 (SESN2), mRNA. [Source:RefSeq mRNA;Acc:NM_001024507]
NADH-ubiquinone oxidoreductase chain 6 [Source:UniProtKB/Swiss-Prot;Acc:P03924]

Bos taurus uroplakin 3B-like (UPK3BL), mRNA. [Source:RefSeq mRNA;Acc:NM_001168011]
Bos taurus chemokine (C-X-C motif) ligand 14 (CXCL14), mRNA. [Source:RefSeq mRNA;Acc:NM_001034410]
Uncharacterized protein [Source:UniProtKB/TrEMBL;Acc:E1BII3]
toll-like receptor adaptor molecule 1 [Source:HGNC Symbol;Acc:HGNC:18348]
Bos taurus endonuclease/exonuclease/phosphatase family domain containing 1 (EEPD1), mRNA. [Source:RefSeq mRNA;Acc:NM_001034409]
Bos taurus 24-dehydrocholesterol reductase (DHCR24), mRNA. [Source:RefSeq mRNA;Acc:NM_001103276]
Uncharacterized protein [Source:UniProtKB/TrEMBL;Acc:G3MWR9]
Olfactory receptor [Source:UniProtKB/TrEMBL;Acc:E1BAX9]
5'-nucleotidase [Source:UniProtKB/TrEMBL;Acc:F1MQM4]
olfactory receptor, family 10, subfamily P, member 1 [Source:HGNC Symbol;Acc:HGNC:15378]
Bos taurus lin-7 homolog B (C. elegans) (LIN7B), mRNA. [Source:RefSeq mRNA;Acc:NM_001046577]
prostaglandin D2 receptor 2 [Source:HGNC Symbol;Acc:HGNC:4502]
COMM domain containing 6 [Source:HGNC Symbol;Acc:HGNC:24015]

Uncharacterized protein [Source:UniProtKB/TrEMBL;Acc:G3N2B5]
Bos taurus Kruppel-like factor 15 (KLF15), mRNA. [Source:RefSeq mRNA;Acc:NM_001082425]
Bos taurus histone cluster 3, H2a (HIST3H2A), mRNA. [Source:RefSeq mRNA;Acc:NM_001098097]
Bos taurus G protein-coupled receptor 4 (GPR4), mRNA. [Source:RefSeq mRNA;Acc:NM_001075583]
zinc finger protein 503 [Source:HGNC Symbol;Acc:HGNC:23589]
Uncharacterized protein [Source:UniProtKB/TrEMBL;Acc:G3MZC3]
zinc finger and BTB domain containing 14 [Source:HGNC Symbol;Acc:HGNC:12860]
Bos taurus N-acetyltransferase 14 (GCN5-related, putative) (NAT14), mRNA. [Source:RefSeq mRNA;Acc:NM_001037245]
Bos taurus immediate early response 3 (IER3), mRNA. [Source:RefSeq mRNA;Acc:NM_001075202]
dorsal root ganglia homeobox [Source:HGNC Symbol;Acc:HGNC:21536]
Bos taurus psoriasis susceptibility 1 candidate 2 (PSORS1C2), mRNA. [Source:RefSeq mRNA;Acc:NM_001080374]
Uncharacterized protein [Source:UniProtKB/TrEMBL;Acc:G3MY94]
family with sequence similarity 124A [Source:HGNC Symbol;Acc:HGNC:26413]
Bos taurus ribosomal protein L27a (RPL27A), mRNA. [Source:RefSeq mRNA;Acc:NM_001024471]
potassium inwardly-rectifying channel, subfamily J, member 5 [Source:HGNC Symbol;Acc:HGNC:6266]

zinc finger and BTB domain containing 12 [Source:HGNC Symbol;Acc:HGNC:19066]
Bos taurus xin actin-binding repeat containing 1 (XIRP1), mRNA. [Source:RefSeq mRNA;Acc:NM_001245928]
interferon induced transmembrane protein 10 [Source:HGNC Symbol;Acc:HGNC:40022]
Bos taurus exocyst complex component 8 (EXOC8), mRNA. [Source:RefSeq mRNA;Acc:NM_001098112]
Bos taurus glycosylphosphatidylinositol anchored molecule like protein (GML), mRNA. [Source:RefSeq mRNA;Acc:NM_001077025]
Bos taurus Cbp/p300-interacting transactivator, with Glu/Asp-rich carboxy-terminal domain, 4 (CITED4), mRNA. [Source:RefSeq mRNA;Acc:NM_001080726]
Myb/SANT-like DNA-binding domain containing 1 [Source:HGNC Symbol;Acc:HGNC:33741]
cleavage stimulation factor, 3' pre-RNA, subunit 2, 64kDa, tau variant [Source:HGNC Symbol;Acc:HGNC:17086]
apolipoprotein L, 6 [Source:HGNC Symbol;Acc:HGNC:14870]
Bos taurus crystallin, beta B1 (CRYBB1), mRNA. [Source:RefSeq mRNA;Acc:NM_174526]
Uncharacterized protein [Source:UniProtKB/TrEMBL;Acc:G5E6C6]
7SK RNA [Source:RFAM;Acc:RF00100]
Bos taurus nucleotide binding protein 2 (NUBP2), mRNA. [Source:RefSeq mRNA;Acc:NM_001034505]
chromosome 19 open reading frame 68 [Source:HGNC Symbol;Acc:HGNC:34495]
transmembrane protein, adipocyte asscociated 1 [Source:HGNC Symbol;Acc:HGNC:30413]
Bos taurus solute carrier family 25, member 34 (SLC25A34), mRNA. [Source:RefSeq mRNA;Acc:NM_001034497]
nicotinate phosphoribosyltransferase [Source:HGNC Symbol;Acc:HGNC:30450]
Bos taurus cathelicidin 2 (CATHL2), mRNA. [Source:RefSeq mRNA;Acc:NM_174826]
inositol 1,4,5-trisphosphate receptor interacting protein-like 1 [Source:HGNC Symbol;Acc:HGNC:29371]
Bos taurus chemokine (C-X-C motif) receptor 3 (CXCR3), mRNA. [Source:RefSeq mRNA;Acc:NM_001011673]
zinc finger and BTB domain containing 34 [Source:HGNC Symbol;Acc:HGNC:31446]
solute carrier family 25 (mitochondrial carrier; phosphate carrier), member 23 [Source:HGNC Symbol;Acc:HGNC:19375]
macrophage expressed 1 [Source:HGNC Symbol;Acc:HGNC:29619]
Bos taurus neurocalcin delta (NCALD), mRNA. [Source:RefSeq mRNA;Acc:NM_174398]
Bos taurus zinc finger, matrin-type 5 (ZMAT5), mRNA. [Source:RefSeq mRNA;Acc:NM_001038210]
Bos taurus hyaluronan synthase 3 (HAS3), mRNA. [Source:RefSeq mRNA;Acc:NM_001192867]
Bos taurus dipeptidyl-peptidase 7 (DPP7), mRNA. [Source:RefSeq mRNA;Acc:NM_001105043]
Bos taurus pannexin 2 (PANX2), mRNA. [Source:RefSeq mRNA;Acc:NM_001102225]
B-cell CLL/lymphoma 9-like [Source:HGNC Symbol;Acc:HGNC:23688]
Uncharacterized protein [Source:UniProtKB/TrEMBL;Acc:F1N5B0]

paxillin [Source:HGNC Symbol;Acc:HGNC:9718]
Bos taurus adenosine A1 receptor (ADORA1), mRNA. [Source:RefSeq mRNA;Acc:NM_174497]
family with sequence similarity 46, member A [Source:HGNC Symbol;Acc:HGNC:18345]
Bos taurus 4-hydroxyphenylpyruvate dioxygenase-like (HPDL), mRNA. [Source:RefSeq mRNA;Acc:NM_001099371]
Olfactory receptor [Source:UniProtKB/TrEMBL;Acc:E1BBR8]
HtrA serine peptidase 3 [Source:HGNC Symbol;Acc:HGNC:30406]
Uncharacterized protein [Source:UniProtKB/TrEMBL;Acc:G3N3P7]
leucine rich repeat containing 15 [Source:HGNC Symbol;Acc:HGNC:20818]
Bos taurus chromatin modifying protein 1A (CHMP1A), mRNA. [Source:RefSeq mRNA;Acc:NM_001191260]
Bos taurus CKLF-like MARVEL transmembrane domain containing 3 (CMTM3), mRNA. [Source:RefSeq mRNA;Acc:NM_001099399]
Bos taurus ribosomal protein L35a (RPL35A), mRNA. [Source:RefSeq mRNA;Acc:NM_001025321]
Bos taurus GLIS family zinc finger 2 (GLIS2), mRNA. [Source:RefSeq mRNA;Acc:NM_001205948]
dual specificity phosphatase 21 [Source:HGNC Symbol;Acc:HGNC:20476]
Bos taurus carbohydrate (keratan sulfate Gal-6) sulfotransferase 1 (CHST1), mRNA. [Source:RefSeq mRNA;Acc:NM_001083648]
Bos taurus yippee-like 5 (Drosophila) (YPEL5), mRNA. [Source:RefSeq mRNA;Acc:NM_001079793]
Olfactory receptor [Source:UniProtKB/TrEMBL;Acc:E1BDG1]
Bos taurus polymerase (DNA-directed), delta 4 (POLD4), mRNA. [Source:RefSeq mRNA;Acc:NM_001076481]
Bos taurus BCL2-associated athanogene 5 (BAG5), mRNA. [Source:RefSeq mRNA;Acc:NM_001040544]

F-box and leucine-rich repeat protein 7 [Source:HGNC Symbol;Acc:HGNC:13604]
chromosome 1 open reading frame 122 [Source:HGNC Symbol;Acc:HGNC:24789]
Bos taurus ring finger protein 186 (RNF186), mRNA. [Source:RefSeq mRNA;Acc:NM_001035311]
Bos taurus translin (TSN), mRNA. [Source:RefSeq mRNA;Acc:NM_001075422]
phosphatidylinositol glycan anchor biosynthesis, class Z [Source:HGNC Symbol;Acc:HGNC:30596]
Bos taurus ribonucleoprotein, PTB-binding 1 (RAVER1), mRNA. [Source:RefSeq mRNA;Acc:NM_001103108]
Bos taurus integrator complex subunit 5 (INTS5), mRNA. [Source:RefSeq mRNA;Acc:NM_001098026]
Bos taurus snail homolog 1 (Drosophila) (SNAI1), mRNA. [Source:RefSeq mRNA;Acc:NM_001112708]
Bos taurus myelin protein zero (MPZ), mRNA. [Source:RefSeq mRNA;Acc:NM_001079507]
zinc finger protein 296 [Source:HGNC Symbol;Acc:HGNC:15981]
Bos taurus actin-like 7A (ACTL7A), mRNA. [Source:RefSeq mRNA;Acc:NM_001038570]

Bos taurus aldo-keto reductase family 7, member A2 (aflatoxin aldehyde reductase) (AKR7A2), mRNA. [Source:RefSeq mRNA;Acc:NM_001101949]
Bos taurus YY1 transcription factor (YY1), mRNA. [Source:RefSeq mRNA;Acc:NM_001098081]
Bos taurus guanine nucleotide binding protein (G protein), gamma 7 (GNG7), mRNA. [Source:RefSeq mRNA;Acc:NM_001113314]
leucine rich repeat and Ig domain containing 4 [Source:HGNC Symbol;Acc:HGNC:31814]
7SK RNA [Source:RFAM;Acc:RF00100]
7SK RNA [Source:RFAM;Acc:RF00100]
chondroadherin-like [Source:HGNC Symbol;Acc:HGNC:25165]
Bos taurus heat shock transcription factor 1 (HSF1), mRNA. [Source:RefSeq mRNA;Acc:NM_001076809]
Bos taurus homeobox A5 (HOXA5), mRNA. [Source:RefSeq mRNA;Acc:NM_001077098]
family with sequence similarity 71, member B [Source:HGNC Symbol;Acc:HGNC:28397]

iroquois homeobox 5 [Source:HGNC Symbol;Acc:HGNC:14361]
ankyrin repeat and SOCS box containing 13 [Source:HGNC Symbol;Acc:HGNC:19765]
sarcalumenin [Source:HGNC Symbol;Acc:HGNC:11295]
chromosome 12 open reading frame 74 [Source:HGNC Symbol;Acc:HGNC:27887]
Olfactory receptor [Source:UniProtKB/TrEMBL;Acc:G3N0E9]
cell division cycle 34 [Source:HGNC Symbol;Acc:HGNC:1734]
Uncharacterized protein [Source:UniProtKB/TrEMBL;Acc:F1MPB8]
Bos taurus ORAI calcium release-activated calcium modulator 1 (ORAI1), mRNA. [Source:RefSeq mRNA;Acc:NM_001099002]
Uncharacterized protein [Source:UniProtKB/TrEMBL;Acc:G3N206]
Olfactory receptor [Source:UniProtKB/TrEMBL;Acc:G3N2E3]
Uncharacterized protein [Source:UniProtKB/TrEMBL;Acc:G5E6D1]
TP53 target 5 [Source:HGNC Symbol;Acc:HGNC:15856]
leucine zipper protein 1 [Source:HGNC Symbol;Acc:HGNC:14985]
Olfactory receptor [Source:UniProtKB/TrEMBL;Acc:E1BMS8]
chemokine (C-C motif) receptor 3 [Source:HGNC Symbol;Acc:HGNC:1604]
Bos taurus calicin (CCIN), mRNA. [Source:RefSeq mRNA;Acc:NM_174005]
Bos taurus tumor protein p63 regulated 1-like (TPRG1L), mRNA. [Source:RefSeq mRNA;Acc:NM_001076418]
Bos taurus jumonji domain containing 5 (KDM8), mRNA. [Source:RefSeq mRNA;Acc:NM_001075339]
Bos taurus ribosomal protein L36a-like (RPL36AL), mRNA. [Source:RefSeq mRNA;Acc:NM_001079508]

Bos taurus SH3-domain binding protein 5 (BTK-associated) (SH3BP5), mRNA. [Source:RefSeq mRNA;Acc:NM_001206288]
Bos taurus signal recognition particle 14kDa (homologous Alu RNA binding protein) (SRP14), mRNA. [Source:RefSeq mRNA;Acc:NM_001100350]
Bos taurus purinergic receptor P2Y, G-protein coupled, 11 (P2RY11), mRNA. [Source:RefSeq mRNA;Acc:NM_001204449]
Wilms tumor 1 interacting protein [Source:HGNC Symbol;Acc:HGNC:20964]
Uncharacterized protein [Source:UniProtKB/TrEMBL;Acc:G3N2L8]
Bos taurus myeloma overexpressed 2 (MYEOV2), mRNA. [Source:RefSeq mRNA;Acc:NM_001195051]
vacuolar protein sorting 51 homolog (S. cerevisiae) [Source:HGNC Symbol;Acc:HGNC:1172]
Uncharacterized protein [Source:UniProtKB/TrEMBL;Acc:E1BDZ1]
Bos taurus forkhead box C2 (MFH-1, mesenchyme forkhead 1) (FOXC2), mRNA. [Source:RefSeq mRNA;Acc:NM_001193072]
Bos taurus pyruvate kinase, muscle (PKM), mRNA. [Source:RefSeq mRNA;Acc:NM_001205727]
Bos taurus MHC class II antigen DS beta (DSB), mRNA. [Source:RefSeq mRNA;Acc:NM_001083785]
Bos taurus golgi phosphoprotein 3-like (GOLPH3L), mRNA. [Source:RefSeq mRNA;Acc:NM_001099052]
Bos taurus ankyrin repeat domain 65 (ANKRD65), mRNA. [Source:RefSeq mRNA;Acc:NM_001102349]

Olfactory receptor [Source:UniProtKB/TrEMBL;Acc:F1MV21]
cysteine-serine-rich nuclear protein 1 [Source:HGNC Symbol;Acc:HGNC:14300]
Bos taurus phospholipase A2, group XV (PLA2G15), mRNA. [Source:RefSeq mRNA;Acc:NM_174560]
Bos taurus interleukin 1 family, member 10 (theta) (IL1F10), mRNA. [Source:RefSeq mRNA;Acc:NM_001193085]
Bos taurus homeobox C4 (HOXC4), mRNA. [Source:RefSeq mRNA;Acc:NM_001102373]
Bos taurus ribosomal protein S27-like (RPS27L), mRNA. [Source:RefSeq mRNA;Acc:NM_001040578]
Bos taurus progestin and adipoQ receptor family member IV (PAQR4), mRNA. [Source:RefSeq mRNA;Acc:NM_001098897]
Bos taurus small nuclear ribonucleoprotein polypeptide E (SNRPE), mRNA. [Source:RefSeq mRNA;Acc:NM_001083459]
olfactory receptor, family 6, subfamily K, member 2 [Source:HGNC Symbol;Acc:HGNC:15029]
Uncharacterized protein [Source:UniProtKB/TrEMBL;Acc:G3N312]
Bos taurus myocyte enhancer factor 2D (MEF2D), mRNA. [Source:RefSeq mRNA;Acc:NM_001205178]
7SK RNA [Source:RFAM;Acc:RF00100]
cytochrome b5 domain containing 2 [Source:HGNC Symbol;Acc:HGNC:28471]
Bos taurus CASP2 and RIPK1 domain containing adaptor with death domain (CRADD), mRNA. [Source:RefSeq mRNA;Acc:NM_001083773]
Uncharacterized protein [Source:UniProtKB/TrEMBL;Acc:G5E6Q3]
interferon regulatory factor 2 binding protein 1 [Source:HGNC Symbol;Acc:HGNC:21728]

Uncharacterized protein; WDR81 protein [Source:UniProtKB/TrEMBL;Acc:A7Z049]
Bos taurus protein kinase C, delta binding protein (PRKCDBP), mRNA. [Source:RefSeq mRNA;Acc:NM_001083401]
zinc finger and BTB domain containing 32 [Source:HGNC Symbol;Acc:HGNC:16763]
tripartite motif containing 42 [Source:HGNC Symbol;Acc:HGNC:19014]
zinc and ring finger 3 [Source:HGNC Symbol;Acc:HGNC:18126]
Bos taurus allograft inflammatory factor 1-like (AIF1L), mRNA. [Source:RefSeq mRNA;Acc:NM_001078079]
Bos taurus potassium channel tetramerisation domain containing 5 (KCTD5), mRNA. [Source:RefSeq mRNA;Acc:NM_001103341]
zinc finger protein 358 [Source:HGNC Symbol;Acc:HGNC:16838]
Bos taurus ADP-ribosylhydrolase like 2 (ADPRHL2), mRNA. [Source:RefSeq mRNA;Acc:NM_001035340]
Uncharacterized protein [Source:UniProtKB/TrEMBL;Acc:F1N0E2]
vestigial-like family member 3 [Source:HGNC Symbol;Acc:HGNC:24327]
Uncharacterized protein [Source:UniProtKB/TrEMBL;Acc:F1MSM7]
Bos taurus ribonuclease, RNase A family, 1 (pancreatic) (RNASE1), mRNA. [Source:RefSeq mRNA;Acc:NM_181810]
Bos taurus melanocortin 4 receptor (MC4R), mRNA. [Source:RefSeq mRNA;Acc:NM_174110]
Bos taurus phosphodiesterase 6G, cGMP-specific, rod, gamma (PDE6G), mRNA. [Source:RefSeq mRNA;Acc:NM_174421]
amyloid beta (A4) precursor protein-binding, family B, member 3 [Source:HGNC Symbol;Acc:HGNC:20708]
Bos taurus SLX1 structure-specific endonuclease subunit homolog A (S. cerevisiae) (SLX1A), mRNA. [Source:RefSeq mRNA;Acc:NM_001037456]

Bos taurus brain ribonuclease (BRB), mRNA. [Source:RefSeq mRNA;Acc:NM_173891]
dickkopf WNT signaling pathway inhibitor 4 [Source:HGNC Symbol;Acc:HGNC:2894]
Kruppel-like factor 3 (basic) [Source:HGNC Symbol;Acc:HGNC:16516]
Bos taurus phosphatidylinositol-specific phospholipase C, X domain containing 1 (PLCXD1), mRNA. [Source:RefSeq mRNA;Acc:NM_001105044]
Bos taurus solute carrier family 25 (mitochondrial carrier; adenine nucleotide translocator), member 6 (SLC25A6), mRNA. [Source:RefSeq mRNA;Acc:NM_17466
granzyme M (lymphocyte met-ase 1) [Source:HGNC Symbol;Acc:HGNC:4712]
Bos taurus estrogen-related receptor alpha (ESRRA), mRNA. [Source:RefSeq mRNA;Acc:NM_001191373]
ArfGAP with GTPase domain, ankyrin repeat and PH domain 3 [Source:HGNC Symbol;Acc:HGNC:16923]
Bos taurus syntaxin 6 (STX6), mRNA. [Source:RefSeq mRNA;Acc:NM_001082431]
TEN1 CST complex subunit [Source:HGNC Symbol;Acc:HGNC:37242]
Bos taurus complement component 1, q subcomponent, A chain (C1QA), mRNA. [Source:RefSeq mRNA;Acc:NM_001014945]
Bos taurus microtubule-associated protein 1 light chain 3 alpha (MAP1LC3A), mRNA. [Source:RefSeq mRNA;Acc:NM_001046175]

Bos taurus cytochrome c oxidase subunit IV isoform 1 (COX4I1), mRNA. [Source:RefSeq mRNA;Acc:NM_001001439]
Bos taurus COMM domain containing 5 (COMMD5), mRNA. [Source:RefSeq mRNA;Acc:NM_001014850]
forkhead box B1 [Source:HGNC Symbol;Acc:HGNC:3799]
Bos taurus NHP2 ribonucleoprotein homolog (yeast) (NHP2), mRNA. [Source:RefSeq mRNA;Acc:NM_001015626]
Olfactory receptor [Source:UniProtKB/TrEMBL;Acc:G3N3W2]
Bos taurus fibrosin (FBRS), mRNA. [Source:RefSeq mRNA;Acc:NM_001105002]
coagulation factor XII (Hageman factor) [Source:HGNC Symbol;Acc:HGNC:3530]
Bos taurus ybeY metallopeptidase (putative) (YBEY), mRNA. [Source:RefSeq mRNA;Acc:NM_001077120]
Uncharacterized protein [Source:UniProtKB/TrEMBL;Acc:G5E6L7]
Olfactory receptor [Source:UniProtKB/TrEMBL;Acc:G3MYP0]
Bos taurus spermatid associated (SPERT), mRNA. [Source:RefSeq mRNA;Acc:NM_001102297]
brain-enriched guanylate kinase-associated [Source:HGNC Symbol;Acc:HGNC:24163]
leucine rich repeat neuronal 4 [Source:HGNC Symbol;Acc:HGNC:16208]
Bos taurus VAMP (vesicle-associated membrane protein)-associated protein B and C (VAPB), mRNA. [Source:RefSeq mRNA;Acc:NM_001082423]
Bos taurus chromosome 25 open reading frame, human C16orf91 (C25H16orf91), mRNA. [Source:RefSeq mRNA;Acc:NM_001076512]
Bos taurus FK506 binding protein 11, 19 kDa (FKBP11), mRNA. [Source:RefSeq mRNA;Acc:NM_001045932]
matrix metallopeptidase 11 (stromelysin 3) [Source:HGNC Symbol;Acc:HGNC:7157]
Uncharacterized protein [Source:UniProtKB/TrEMBL;Acc:G5E638]
Uncharacterized protein [Source:UniProtKB/TrEMBL;Acc:E1BG91]
Bos taurus processing of precursor 7, ribonuclease P/MRP subunit (S. cerevisiae) (POP7), mRNA. [Source:RefSeq mRNA;Acc:NM_001076482]
Bos taurus chromosome 1 open reading frame, human C21orf33 (C1H21orf33), mRNA. [Source:RefSeq mRNA;Acc:NM_001034463]

Bos taurus chymotrypsin-like elastase family, member 3B (CELA3B), mRNA. [Source:RefSeq mRNA;Acc:NM_001166518]
Bos taurus solute carrier family 43, member 3 (SLC43A3), mRNA. [Source:RefSeq mRNA;Acc:NM_001038126]
Bos taurus histone H2B (H2B), mRNA. [Source:RefSeq mRNA;Acc:NM_001105643]
Bos taurus high mobility group box 4 (HMGB4), mRNA. [Source:RefSeq mRNA;Acc:NM_001040562]
Bos taurus G protein-coupled receptor 17 (GPR17), mRNA. [Source:RefSeq mRNA;Acc:NM_001082470]
Bos taurus mannosidase, beta A, lysosomal-like (MANBAL), mRNA. [Source:RefSeq mRNA;Acc:NM_001114861]
Bos taurus CKLF-like MARVEL transmembrane domain containing 5 (CMTM5), mRNA. [Source:RefSeq mRNA;Acc:NM_001075434]
Uncharacterized protein [Source:UniProtKB/TrEMBL;Acc:E1B966]

Bos taurus ras homolog gene family, member F (in filopodia) (RHOF), mRNA. [Source:RefSeq mRNA;Acc:NM_001040602]
Bos taurus small integral membrane protein 5 (SMIM5), mRNA. [Source:RefSeq mRNA;Acc:NM_001163000]
protein phosphatase 1, regulatory subunit 3F [Source:HGNC Symbol;Acc:HGNC:14944]
Bos taurus F-box protein 39 (FBXO39), mRNA. [Source:RefSeq mRNA;Acc:NM_001037613]
glycoprotein Ib (platelet), alpha polypeptide [Source:HGNC Symbol;Acc:HGNC:4439]
transmembrane epididymal protein 1 [Source:HGNC Symbol;Acc:HGNC:30233]
Bos taurus guanylate cyclase activator 1A (retina) (GUCA1A), mRNA. [Source:RefSeq mRNA;Acc:NM_174546]
lipoma HMGIC fusion partner-like 5 [Source:HGNC Symbol;Acc:HGNC:21253]
Bos taurus proteasome (prosome, macropain) subunit, beta type, 8 (large multifunctional peptidase 7) (PSMB8), mRNA. [Source:RefSeq mRNA;Acc:NM_00104[illegible]
Olfactory receptor [Source:UniProtKB/TrEMBL;Acc:G5E676]
Bos taurus SPC24, NDC80 kinetochore complex component, homolog (S. cerevisiae) (SPC24), mRNA. [Source:RefSeq mRNA;Acc:NM_001075391]
Bos taurus TSR3, 20S rRNA accumulation, homolog (S. cerevisiae) (TSR3), mRNA. [Source:RefSeq mRNA;Acc:NM_001098911]
Bos taurus exosome component 3 (EXOSC3), mRNA. [Source:RefSeq mRNA;Acc:NM_001035397]
sodium channel, non-voltage-gated 1, delta subunit [Source:HGNC Symbol;Acc:HGNC:10601]
Uncharacterized protein [Source:UniProtKB/TrEMBL;Acc:E1BLD0]
RUN and SH3 domain containing 2 [Source:HGNC Symbol;Acc:HGNC:23625]
Bos taurus zinc finger protein 784 (ZNF784), mRNA. [Source:RefSeq mRNA;Acc:NM_001099731]
Bos taurus ATP-binding cassette, sub-family B (MDR/TAP), member 8 (ABCB8), mRNA. [Source:RefSeq mRNA;Acc:NM_001075526]
Uncharacterized protein [Source:UniProtKB/TrEMBL;Acc:F1N1Y5]
tumor protein p53 inducible protein 11 [Source:HGNC Symbol;Acc:HGNC:16842]
zinc finger protein 408 [Source:HGNC Symbol;Acc:HGNC:20041]

outer dense fiber of sperm tails 3-like 2 [Source:HGNC Symbol;Acc:HGNC:26841]
phospholipase A2 inhibitor and LY6/PLAUR domain containing [Source:HGNC Symbol;Acc:HGNC:44206]
Bos taurus dual specificity phosphatase 7 (DUSP7), mRNA. [Source:RefSeq mRNA;Acc:NM_001101294]
Zic family member 5 [Source:HGNC Symbol;Acc:HGNC:20322]
cyclic nucleotide gated channel alpha 4 [Source:HGNC Symbol;Acc:HGNC:2152]
matrilin 1, cartilage matrix protein [Source:HGNC Symbol;Acc:HGNC:6907]
G protein-coupled receptor 135 [Source:HGNC Symbol;Acc:HGNC:19991]
zinc finger, MYND-type containing 15 [Source:HGNC Symbol;Acc:HGNC:20997]

Bos taurus homeobox B3 (HOXB3), mRNA. [Source:RefSeq mRNA;Acc:NM_001099174]
sosondowah ankyrin repeat domain family member A [Source:HGNC Symbol;Acc:HGNC:27033]
Uncharacterized protein [Source:UniProtKB/TrEMBL;Acc:G3N2L0]
Bos taurus synaptogyrin 2 (SYNGR2), mRNA. [Source:RefSeq mRNA;Acc:NM_001100358]
Bos taurus POU class 4 homeobox 3 (POU4F3), mRNA. [Source:RefSeq mRNA;Acc:NM_001192035]
opioid receptor, delta 1 [Source:HGNC Symbol;Acc:HGNC:8153]
Bos taurus actin, beta (ACTB), mRNA. [Source:RefSeq mRNA;Acc:NM_173979]
Bos taurus pre-B-cell leukemia homeobox 3 (PBX3), mRNA. [Source:RefSeq mRNA;Acc:NM_001046453]
Bos taurus neuralized homolog 2 (Drosophila) (NEURL2), mRNA. [Source:RefSeq mRNA;Acc:NM_001192636]
Bos taurus sirtuin 7 (SIRT7), mRNA. [Source:RefSeq mRNA;Acc:NM_001075217]
Olfactory receptor [Source:UniProtKB/TrEMBL;Acc:M0QVY8]
Bos taurus homeobox B1 (HOXB1), mRNA. [Source:RefSeq mRNA;Acc:NM_001205954]
Bos taurus histone cluster 1, H2bk (HIST1H2BN), mRNA. [Source:RefSeq mRNA;Acc:NM_001078063]
pleiomorphic adenoma gene 1 [Source:HGNC Symbol;Acc:HGNC:9045]
Uncharacterized protein [Source:UniProtKB/TrEMBL;Acc:G1K1P6]
A kinase (PRKA) anchor protein 1 [Source:HGNC Symbol;Acc:HGNC:367]
Uncharacterized protein [Source:UniProtKB/TrEMBL;Acc:G3MWY7]

Bos taurus melanocortin 5 receptor (MC5R), mRNA. [Source:RefSeq mRNA;Acc:NM_001015542]
actin-related protein T1 [Source:HGNC Symbol;Acc:HGNC:24027]
Olfactory receptor [Source:UniProtKB/TrEMBL;Acc:G3N317]
Bos taurus nerve growth factor (beta polypeptide) (NGF), mRNA. [Source:RefSeq mRNA;Acc:NM_001099362]
Bos taurus neuropeptide Y receptor Y2 (NPY2R), mRNA. [Source:RefSeq mRNA;Acc:NM_174401]
Uncharacterized protein [Source:UniProtKB/TrEMBL;Acc:G3N1H5]
Uncharacterized protein [Source:UniProtKB/TrEMBL;Acc:G3N2M0]
Bos taurus TOX high mobility group box family member 2 (TOX2), transcript variant 1, mRNA. [Source:RefSeq mRNA;Acc:NM_001206688]
leucine rich repeat containing 29 [Source:HGNC Symbol;Acc:HGNC:13605]
estrogen-related receptor gamma [Source:HGNC Symbol;Acc:HGNC:3474]
Bos taurus ADNP homeobox 2 (ADNP2), mRNA. [Source:RefSeq mRNA;Acc:NM_001101840]
Bos taurus uncharacterized protein MGC134105 (MGC134105), mRNA. [Source:RefSeq mRNA;Acc:NM_001077031]

leucine rich repeat and Ig domain containing 3 [Source:HGNC Symbol;Acc:HGNC:21206]
keratin associated protein 24-1 [Source:HGNC Symbol;Acc:HGNC:33902]
Bos taurus binder of sperm 5 (BSP5), mRNA. [Source:RefSeq mRNA;Acc:NM_174842]

chemokine (C-C motif) ligand 1 [Source:HGNC Symbol;Acc:HGNC:10609]
Uncharacterized protein [Source:UniProtKB/TrEMBL;Acc:G3N0E4]
testis-specific serine kinase 2 [Source:HGNC Symbol;Acc:HGNC:11401]
Bos taurus insulin-like 3 (Leydig cell) (INSL3), mRNA. [Source:RefSeq mRNA;Acc:NM_174365]
potassium channel, subfamily K, member 3 [Source:HGNC Symbol;Acc:HGNC:6278]
Bos taurus stratifin (SFN), mRNA. [Source:RefSeq mRNA;Acc:NM_001075912]
Bos taurus thymidine kinase 1, soluble (TK1), mRNA. [Source:RefSeq mRNA;Acc:NM_001097572]
NIM1 serine/threonine protein kinase [Source:HGNC Symbol;Acc:HGNC:28646]
Uncharacterized protein [Source:UniProtKB/TrEMBL;Acc:E1BIJ9]
Bos taurus zinc finger protein 639 (ZNF639), mRNA. [Source:RefSeq mRNA;Acc:NM_001099023]
Bos taurus GDP-D-glucose phosphorylase 1 (GDPGP1), mRNA. [Source:RefSeq mRNA;Acc:NM_001015629]
Bos taurus receptor (G protein-coupled) activity modifying protein 3 (RAMP3), mRNA. [Source:RefSeq mRNA;Acc:NM_001083505]
Bos taurus proteasome (prosome, macropain) subunit, beta type, 11 (PSMB11), mRNA. [Source:RefSeq mRNA;Acc:NM_001099386]
Uncharacterized protein [Source:UniProtKB/TrEMBL;Acc:E1BLT1]
Bos taurus blocked early in transport 1 homolog (S. cerevisiae)-like (BET1L), mRNA. [Source:RefSeq mRNA;Acc:NM_001034362]
RAB22A, member RAS oncogene family [Source:HGNC Symbol;Acc:HGNC:9764]
Bos taurus transmembrane protein 79 (TMEM79), mRNA. [Source:RefSeq mRNA;Acc:NM_001014920]
Bos taurus prepronociceptin (PNOC), mRNA. [Source:RefSeq mRNA;Acc:NM_174150]

Bos taurus nudix (nucleoside diphosphate linked moiety X)-type motif 11 (NUDT11), mRNA. [Source:RefSeq mRNA;Acc:NM_001101296]
Bos taurus surfactant associated 2 (SFTA2), mRNA. [Source:RefSeq mRNA;Acc:NM_001114523]
ubiquitin specific peptidase 49 [Source:HGNC Symbol;Acc:HGNC:20078]
40S ribosomal protein S6 [Source:UniProtKB/TrEMBL;Acc:E1B8K9]
zinc finger protein 219 [Source:HGNC Symbol;Acc:HGNC:13011]
Bos taurus histone H3-like (H3F3C), mRNA. [Source:RefSeq mRNA;Acc:NM_001076191]

Bos taurus solute carrier family 3 (activators of dibasic and neutral amino acid transport), member 2 (SLC3A2), mRNA. [Source:RefSeq mRNA;Acc:NM_0010244

TBC1 domain family, member 17 [Source:HGNC Symbol;Acc:HGNC:25699]
Bos taurus protein phosphatase 1, regulatory subunit 16A (PPP1R16A), mRNA. [Source:RefSeq mRNA;Acc:NM_001193251]
Bos taurus NmrA-like family domain containing 1 (NMRAL1), mRNA. [Source:RefSeq mRNA;Acc:NM_001076032]
Olfactory receptor [Source:UniProtKB/TrEMBL;Acc:E1B831]
Bos taurus Ras association (RalGDS/AF-6) domain family member 3 (RASSF3), mRNA. [Source:RefSeq mRNA;Acc:NM_001192886]
Bos taurus progestin and adipoQ receptor family member VII (PAQR7), mRNA. [Source:RefSeq mRNA;Acc:NM_001038553]
Bos taurus potassium inwardly-rectifying channel, subfamily J, member 11 (KCNJ11), mRNA. [Source:RefSeq mRNA;Acc:NM_001081598]

Bos taurus voltage-dependent anion channel 1 (VDAC1), mRNA. [Source:RefSeq mRNA;Acc:NM_174485]
5-hydroxymethylcytosine (hmC) binding, ES cell-specific [Source:HGNC Symbol;Acc:HGNC:24446]
prickle homolog 2 (Drosophila) [Source:HGNC Symbol;Acc:HGNC:20340]
Bos taurus protein FAM229B (FAM229B), mRNA. [Source:RefSeq mRNA;Acc:NM_001195067]
Bos taurus RAS (RAD and GEM)-like GTP-binding 1 (REM1), mRNA. [Source:RefSeq mRNA;Acc:NM_001046001]
Bos taurus plasmalemma vesicle associated protein (PLVAP), mRNA. [Source:RefSeq mRNA;Acc:NM_001035353]

Bos taurus RAB5C, member RAS oncogene family (RAB5C), mRNA. [Source:RefSeq mRNA;Acc:NM_001034743]
Bos taurus RAB21, member RAS oncogene family (RAB21), mRNA. [Source:RefSeq mRNA;Acc:NM_001075317]
kelch-like protein 22 [Source:RefSeq peptide;Acc:NP_001192702]
Bos taurus ankyrin repeat and SOCS box containing 16 (ASB16), mRNA. [Source:RefSeq mRNA;Acc:NM_001101188]
Bos taurus L-gulono-gamma-lactone oxidase (GULO), mRNA. [Source:RefSeq mRNA;Acc:NM_001034043]
Bos taurus asparaginase like 1 (ASRGL1), mRNA. [Source:RefSeq mRNA;Acc:NM_001077035]
Bos taurus eukaryotic translation initiation factor 1A domain containing (EIF1AD), mRNA. [Source:RefSeq mRNA;Acc:NM_001076393]
Bos taurus dual specificity phosphatase 26 (putative) (DUSP26), mRNA. [Source:RefSeq mRNA;Acc:NM_001076004]
proteasome (prosome, macropain) assembly chaperone 4 [Source:HGNC Symbol;Acc:HGNC:21108]
phosphoglycerate mutase family member 5 [Source:HGNC Symbol;Acc:HGNC:28763]
Uncharacterized protein [Source:UniProtKB/TrEMBL;Acc:E1BJ84]
T-cell acute lymphocytic leukemia 1 [Source:HGNC Symbol;Acc:HGNC:11556]
splA/ryanodine receptor domain and SOCS box containing 4 [Source:HGNC Symbol;Acc:HGNC:30630]

Uncharacterized protein [Source:UniProtKB/TrEMBL;Acc:G3X7U9]
gem (nuclear organelle) associated protein 4 [Source:HGNC Symbol;Acc:HGNC:15717]
Uncharacterized protein [Source:UniProtKB/TrEMBL;Acc:E1BCY7]
collectin-43 precursor [Source:RefSeq peptide;Acc:NP_001002237]
Bos taurus leucine rich repeat containing 47 (LRRC47), mRNA. [Source:RefSeq mRNA;Acc:NM_001102298]
C25H7ORF50 protein; Uncharacterized protein [Source:UniProtKB/TrEMBL;Acc:A8R461]
G protein-coupled receptor 12 [Source:HGNC Symbol;Acc:HGNC:4466]
olfactory receptor, family 5, subfamily AU, member 1 [Source:HGNC Symbol;Acc:HGNC:15362]
zinc finger protein 48 [Source:HGNC Symbol;Acc:HGNC:13114]
olfactory receptor, family 1, subfamily M, member 1 [Source:HGNC Symbol;Acc:HGNC:8220]

Uncharacterized protein [Source:UniProtKB/TrEMBL;Acc:E1B7Z5]
radial spoke head 6 homolog A (Chlamydomonas) [Source:HGNC Symbol;Acc:HGNC:14241]
Bos taurus follistatin-like 3 (secreted glycoprotein) (FSTL3), mRNA. [Source:RefSeq mRNA;Acc:NM_001075710]
gap junction protein, alpha 8, 50kDa [Source:HGNC Symbol;Acc:HGNC:4281]
Bos taurus thrombomodulin (THBD), mRNA. [Source:RefSeq mRNA;Acc:NM_001166522]
Bos taurus prolactin regulatory element binding (PREB), mRNA. [Source:RefSeq mRNA;Acc:NM_001076875]
Bos taurus C-type lectin domain family 14, member A (CLEC14A), mRNA. [Source:RefSeq mRNA;Acc:NM_001077890]

Bos taurus spermatogenesis associated 2-like (SPATA2L), mRNA. [Source:RefSeq mRNA;Acc:NM_001076502]
Bos taurus inhibin, beta E (INHBE), mRNA. [Source:RefSeq mRNA;Acc:NM_001205842]
Bos taurus gastrin-releasing peptide (GRP), mRNA. [Source:RefSeq mRNA;Acc:NM_001101239]
Uncharacterized protein [Source:UniProtKB/TrEMBL;Acc:F1MPZ1]
proline/arginine-rich end leucine-rich repeat protein [Source:HGNC Symbol;Acc:HGNC:9357]
sialidase 2 (cytosolic sialidase) [Source:HGNC Symbol;Acc:HGNC:7759]
Bos taurus chemokine-like receptor 1 (CMKLR1), mRNA. [Source:RefSeq mRNA;Acc:NM_001145235]
Bos taurus BTB (POZ) domain containing 19 (BTBD19), mRNA. [Source:RefSeq mRNA;Acc:NM_001101241]
ets variant 4 [Source:HGNC Symbol;Acc:HGNC:3493]
Bos taurus ADP-ribosyltransferase 5 (ART5), mRNA. [Source:RefSeq mRNA;Acc:NM_001076515]
Uncharacterized protein [Source:UniProtKB/TrEMBL;Acc:G3N0G2]

Bos taurus cytokine induced apoptosis inhibitor 1 (CIAPIN1), mRNA. [Source:RefSeq mRNA;Acc:NM_001015659]

ankyrin repeat domain 34C [Source:HGNC Symbol;Acc:HGNC:33888]
Bos taurus amyloid beta (A4) precursor protein-binding, family A, member 3 (APBA3), mRNA. [Source:RefSeq mRNA;Acc:NM_001034414]
Uncharacterized protein [Source:UniProtKB/TrEMBL;Acc:E1BF33]
Uncharacterized protein [Source:UniProtKB/TrEMBL;Acc:G3N234]
cytochrome oxidase assembly 1 [Source:RefSeq peptide;Acc:NP_001029428]
Uncharacterized protein [Source:UniProtKB/TrEMBL;Acc:F1MDZ4]
mannosyl (beta-1,4-)-glycoprotein beta-1,4-N-acetylglucosaminyltransferase [Source:HGNC Symbol;Acc:HGNC:7046]
interferon regulatory factor 2 binding protein-like [Source:HGNC Symbol;Acc:HGNC:14282]

headcase homolog (Drosophila) [Source:HGNC Symbol;Acc:HGNC:21041]
Bos taurus zinc finger protein 689 (ZNF689), mRNA. [Source:RefSeq mRNA;Acc:NM_001083746]
Bos taurus 6-phosphogluconolactonase (PGLS), mRNA. [Source:RefSeq mRNA;Acc:NM_001038580]
olfactory receptor, family 8, subfamily B, member 8 [Source:HGNC Symbol;Acc:HGNC:8477]
Bos taurus Smad nuclear interacting protein 1 (SNIP1), mRNA. [Source:RefSeq mRNA;Acc:NM_001205924]
zinc finger family member 783 [Source:HGNC Symbol;Acc:HGNC:27222]
Bos taurus chemokine (C-X-C motif) receptor 6 (CXCR6), mRNA. [Source:RefSeq mRNA;Acc:NM_001014859]
chemokine (C-C motif) ligand 16 [Source:HGNC Symbol;Acc:HGNC:10614]
xeroderma pigmentosum, complementation group C [Source:HGNC Symbol;Acc:HGNC:12816]
Bos taurus defensin, beta 1 (DEFB1), mRNA. [Source:RefSeq mRNA;Acc:NM_175703]
N-terminal Xaa-Pro-Lys N-methyltransferase 1 [Source:HGNC Symbol;Acc:HGNC:23373]
transmembrane protein 255B [Source:HGNC Symbol;Acc:HGNC:28297]
Bos taurus dolichyl pyrophosphate phosphatase 1 (DOLPP1), mRNA. [Source:RefSeq mRNA;Acc:NM_001192041]
Bos taurus adenosine A3 receptor (ADORA3), transcript variant 1, mRNA. [Source:RefSeq mRNA;Acc:NM_001104611]
Bos taurus dehydrogenase/reductase (SDR family) member 11 (DHRS11), mRNA. [Source:RefSeq mRNA;Acc:NM_001035088]
Bos taurus stanniocalcin 2 (STC2), mRNA. [Source:RefSeq mRNA;Acc:NM_001192745]
Uncharacterized protein [Source:UniProtKB/TrEMBL;Acc:F1MR48]
Uncharacterized protein [Source:UniProtKB/TrEMBL;Acc:G5E625]
Bos taurus nestin (NES), mRNA. [Source:RefSeq mRNA;Acc:NM_001206591]

Uncharacterized protein [Source:UniProtKB/TrEMBL;Acc:F1MGN8]
Bos taurus phospholysine phosphohistidine inorganic pyrophosphate phosphatase (LHPP), mRNA. [Source:RefSeq mRNA;Acc:NM_001080912]
olfactory receptor, family 1, subfamily K, member 1 [Source:HGNC Symbol;Acc:HGNC:8212]
Bos taurus family with sequence similarity 57, member A (FAM57A), mRNA. [Source:RefSeq mRNA;Acc:NM_001081516]
Olfactory receptor [Source:UniProtKB/TrEMBL;Acc:G3MYN9]

leucine rich repeat containing 10B [Source:HGNC Symbol;Acc:HGNC:37215]
olfactory receptor, family 6, subfamily B, member 1 [Source:HGNC Symbol;Acc:HGNC:8354]
Bos taurus TIMP metallopeptidase inhibitor 1 (TIMP1), mRNA. [Source:RefSeq mRNA;Acc:NM_174471]
Bos taurus pyruvate dehydrogenase (lipoamide) alpha 2 (PDHA2), mRNA. [Source:RefSeq mRNA;Acc:NM_001077071]
Ferritin [Source:UniProtKB/TrEMBL;Acc:E1B888]

Bos taurus zinc finger protein 703 (ZNF703), mRNA. [Source:RefSeq mRNA;Acc:NM_001191480]
Bos taurus nerve growth factor receptor (NGFR), mRNA. [Source:RefSeq mRNA;Acc:NM_001102478]

Olfactory receptor [Source:UniProtKB/TrEMBL;Acc:E1BMU7]
Bos taurus SUMO1 activating enzyme subunit 1 (SAE1), mRNA. [Source:RefSeq mRNA;Acc:NM_001081711]
5-hydroxytryptamine (serotonin) receptor 1E, G protein-coupled [Source:HGNC Symbol;Acc:HGNC:5291]
Bos taurus gem (nuclear organelle) associated protein 6 (GEMIN6), mRNA. [Source:RefSeq mRNA;Acc:NM_001046278]
Bos taurus solute carrier family 30 (zinc transporter), member 3 (SLC30A3), mRNA. [Source:RefSeq mRNA;Acc:NM_001076843]
Bos taurus zinc finger protein 36, C3H type, homolog (mouse) (ZFP36), mRNA. [Source:RefSeq mRNA;Acc:NM_174493]
Bos taurus zinc finger protein 274 (ZNF274), mRNA. [Source:RefSeq mRNA;Acc:NM_001101153]

Uncharacterized protein [Source:UniProtKB/TrEMBL;Acc:G3N1S6]
Bos taurus collectin sub-family member 11 (COLEC11), mRNA. [Source:RefSeq mRNA;Acc:NM_001076303]
Bos taurus Purkinje cell protein 4 like 1 (PCP4L1), mRNA. [Source:RefSeq mRNA;Acc:NM_001110445]
N-acetyltransferase 6 (GCN5-related) [Source:HGNC Symbol;Acc:HGNC:30252]
fibronectin leucine rich transmembrane protein 2 [Source:HGNC Symbol;Acc:HGNC:3761]
Bos taurus coiled-coil domain containing 3 (CCDC3), mRNA. [Source:RefSeq mRNA;Acc:NM_001172375]
Bos taurus major facilitator superfamily domain containing 6-like (MFSD6L), mRNA. [Source:RefSeq mRNA;Acc:NM_001102523]

Bos taurus cystatin B (stefin B) (CSTB), mRNA. [Source:RefSeq mRNA;Acc:NM_001037461]
Bos taurus transmembrane protein 100 (TMEM100), mRNA. [Source:RefSeq mRNA;Acc:NM_001046522]
Bos taurus NCK adaptor protein 2 (NCK2), mRNA. [Source:RefSeq mRNA;Acc:NM_001099036]
Bos taurus transducer of ERBB2, 1 (TOB1), transcript variant 1, mRNA. [Source:RefSeq mRNA;Acc:NM_001077075]
Bos taurus carboxypeptidase N, polypeptide 2 (CPN2), mRNA. [Source:RefSeq mRNA;Acc:NM_001101232]
Bos taurus translocase of inner mitochondrial membrane domain containing 1 (TIMMDC1), mRNA. [Source:RefSeq mRNA;Acc:NM_001080235]
Bos taurus ubiquitin-conjugating enzyme E2D 2 (UBE2D2), mRNA. [Source:RefSeq mRNA;Acc:NM_001046496]
zinc finger protein 839 [Source:HGNC Symbol;Acc:HGNC:20345]
G protein-coupled receptor 153 [Source:HGNC Symbol;Acc:HGNC:23618]
Bos taurus LysM, putative peptidoglycan-binding, domain containing 4 (LYSMD4), mRNA. [Source:RefSeq mRNA;Acc:NM_001081528]
Olfactory receptor [Source:UniProtKB/TrEMBL;Acc:F1MYE1]
Bos taurus transmembrane protein 158 (TMEM158), mRNA. [Source:RefSeq mRNA;Acc:NM_001081626]
Uncharacterized protein [Source:UniProtKB/TrEMBL;Acc:E1BG46]
Bos taurus inositol-trisphosphate 3-kinase A (ITPKA), mRNA. [Source:RefSeq mRNA;Acc:NM_001083519]
Bos taurus potassium channel tetramerisation domain containing 11 (KCTD11), mRNA. [Source:RefSeq mRNA;Acc:NM_001035421]
Bos taurus DnaJ (Hsp40) homolog, subfamily C, member 5 gamma (DNAJC5G), mRNA. [Source:RefSeq mRNA;Acc:NM_001076429]
solute carrier family 26 (anion exchanger), member 1 [Source:HGNC Symbol;Acc:HGNC:10993]
Bos taurus mitochondrial E3 ubiquitin protein ligase 1 (MUL1), mRNA. [Source:RefSeq mRNA;Acc:NM_001192182]
testis-specific serine kinase 4 [Source:HGNC Symbol;Acc:HGNC:19825]
Bos taurus fat storage-inducing transmembrane protein 1 (FITM1), mRNA. [Source:RefSeq mRNA;Acc:NM_001105351]
Bos taurus lysozyme (LOC781146), mRNA. [Source:RefSeq mRNA;Acc:NM_001080336]
Bos taurus ORM1-like 2 (S. cerevisiae) (ORMDL2), mRNA. [Source:RefSeq mRNA;Acc:NM_001014931]
podocalyxin-like 2 [Source:HGNC Symbol;Acc:HGNC:17936]
Bos taurus hepatoma derived growth factor-like 1 (HDGFL1), mRNA. [Source:RefSeq mRNA;Acc:NM_001001136]
Uncharacterized protein [Source:UniProtKB/TrEMBL;Acc:F1MP07]
Bos taurus UDP-Gal:betaGlcNAc beta 1,3-galactosyltransferase, polypeptide 4 (B3GALT4), mRNA. [Source:RefSeq mRNA;Acc:NM_001077097]
Olfactory receptor [Source:UniProtKB/TrEMBL;Acc:F1MB62]
Bos taurus interferon regulatory factor 5 (IRF5), mRNA. [Source:RefSeq mRNA;Acc:NM_001035465]
Uncharacterized protein [Source:UniProtKB/TrEMBL;Acc:F1MIN9]
Bos taurus frizzled-related protein (FRZB), mRNA. [Source:RefSeq mRNA;Acc:NM_174059]

Cytochrome c oxidase subunit 6A, mitochondrial [Source:UniProtKB/TrEMBL;Acc:F1MBC7]
Olfactory receptor [Source:UniProtKB/TrEMBL;Acc:F1MUB3]
prostate transmembrane protein, androgen induced 1 [Source:HGNC Symbol;Acc:HGNC:14107]
Uncharacterized protein [Source:UniProtKB/TrEMBL;Acc:E1BJS0]
chemokine (C motif) receptor 1 [Source:HGNC Symbol;Acc:HGNC:1625]
Uncharacterized protein [Source:UniProtKB/TrEMBL;Acc:E1BF78]
basic leucine zipper transcription factor, ATF-like 3 [Source:HGNC Symbol;Acc:HGNC:28915]
Bos taurus LIM homeobox 5 (LHX5), mRNA. [Source:RefSeq mRNA;Acc:NM_001102061]
Fc fragment of IgG binding protein [Source:HGNC Symbol;Acc:HGNC:13572]
Bos taurus zinc finger, C4H2 domain containing (ZC4H2), mRNA. [Source:RefSeq mRNA;Acc:NM_001078111]
calcium/calmodulin-dependent protein kinase IG [Source:HGNC Symbol;Acc:HGNC:14585]
family with sequence similarity 217, member B [Source:HGNC Symbol;Acc:HGNC:16170]
lysophosphatidic acid receptor 3 [Source:HGNC Symbol;Acc:HGNC:14298]
Bos taurus heterogeneous nuclear ribonucleoprotein F (HNRNPF), mRNA. [Source:RefSeq mRNA;Acc:NM_001014860]
high mobility group AT-hook 2 [Source:HGNC Symbol;Acc:HGNC:5009]
Bos taurus regakine 1 (LOC504773), mRNA. [Source:RefSeq mRNA;Acc:NM_001034220]
Ribosomal protein [Source:UniProtKB/TrEMBL;Acc:G3X7N2]
Bos taurus interferon alpha G (IFNAG), mRNA. [Source:RefSeq mRNA;Acc:NM_001172040]
Bos taurus retrotransposon-like 1 (RTL1), mRNA. [Source:RefSeq mRNA;Acc:NM_001195020]
Bos taurus NLR family, pyrin domain containing 12 (NLRP12), mRNA. [Source:RefSeq mRNA;Acc:NM_001192551]
Bos taurus cold shock domain containing C2, RNA binding (CSDC2), mRNA. [Source:RefSeq mRNA;Acc:NM_001076877]
Bos taurus malonyl-CoA decarboxylase (MLYCD), mRNA. [Source:RefSeq mRNA;Acc:NM_001098946]
junctophilin 2 [Source:HGNC Symbol;Acc:HGNC:14202]
Bos taurus PR domain containing 13 (PRDM13), mRNA. [Source:RefSeq mRNA;Acc:NM_001193010]
G protein-coupled receptor 45 [Source:HGNC Symbol;Acc:HGNC:4503]
Bos taurus FtsJ RNA methyltransferase homolog 1 (E. coli) (FTSJ1), mRNA. [Source:RefSeq mRNA;Acc:NM_001077934]
slingshot protein phosphatase 3 [Source:HGNC Symbol;Acc:HGNC:30581]
poliovirus receptor related immunoglobulin domain containing [Source:HGNC Symbol;Acc:HGNC:32190]
Uncharacterized protein [Source:UniProtKB/TrEMBL;Acc:G3N3K7]

Bos taurus chromosome 15 open reading frame, human C11orf88 (C15H11orf88), mRNA. [Source:RefSeq mRNA;Acc:NM_001077028]
Bos taurus complement component 1, q subcomponent, B chain (C1QB), mRNA. [Source:RefSeq mRNA;Acc:NM_001046599]
G protein-coupled receptor 152 [Source:HGNC Symbol;Acc:HGNC:23622]
Bos taurus thyroid hormone receptor interactor 6 (TRIP6), mRNA. [Source:RefSeq mRNA;Acc:NM_001035469]
Bos taurus TSPY-like 1 (TSPYL1), mRNA. [Source:RefSeq mRNA;Acc:NM_001076111]
SH3 domain and tetratricopeptide repeats 1 [Source:HGNC Symbol;Acc:HGNC:26009]
Bos taurus apelin receptor (APLNR), mRNA. [Source:RefSeq mRNA;Acc:NM_001102524]
Bos taurus SPT2, Suppressor of Ty, domain containing 1 (S. cerevisiae) (SPTY2D1), mRNA. [Source:RefSeq mRNA;Acc:NM_001205738]
Bos taurus phosphatidic acid phosphatase type 2 domain containing 3 (PPAPDC3), mRNA. [Source:RefSeq mRNA;Acc:NM_001078118]
Bos taurus v-myc myelocytomatosis viral oncogene homolog (avian) (MYC), mRNA. [Source:RefSeq mRNA;Acc:NM_001046074]
Bos taurus LSM6 homolog, U6 small nuclear RNA associated (S. cerevisiae) (LSM6), mRNA. [Source:RefSeq mRNA;Acc:NM_001114519]
family with sequence similarity 195, member B [Source:HGNC Symbol;Acc:HGNC:28007]
sorting nexin 19 [Source:HGNC Symbol;Acc:HGNC:21532]
Bos taurus solute carrier family 39 (zinc transporter), member 1 (SLC39A1), mRNA. [Source:RefSeq mRNA;Acc:NM_001035381]
olfactory receptor, family 5, subfamily AK, member 2 [Source:HGNC Symbol;Acc:HGNC:15251]
Bos taurus solute carrier family 46 (folate transporter), member 1 (SLC46A1), mRNA. [Source:RefSeq mRNA;Acc:NM_001079585]
Bos taurus mediator complex subunit 26 (MED26), mRNA. [Source:RefSeq mRNA;Acc:NM_001098888]
Uncharacterized protein [Source:UniProtKB/TrEMBL;Acc:F1MQJ8]
zinc finger CCCH-type containing 12B [Source:HGNC Symbol;Acc:HGNC:17407]

Bos taurus iron-sulfur cluster scaffold homolog (E. coli) (ISCU), mRNA. [Source:RefSeq mRNA;Acc:NM_001075683]
Olfactory receptor [Source:UniProtKB/TrEMBL;Acc:G3MZ48]
VANGL planar cell polarity protein 1 [Source:HGNC Symbol;Acc:HGNC:15512]
fucosyltransferase 2 (secretor status included) [Source:HGNC Symbol;Acc:HGNC:4013]
Bos taurus peripherin 2 (retinal degeneration, slow) (PRPH2), mRNA. [Source:RefSeq mRNA;Acc:NM_001166487]
Bos taurus chromosome 28 open reading frame, human C10orf10 (C28H10orf10), mRNA. [Source:RefSeq mRNA;Acc:NM_001046515]
cyclin D3 [Source:HGNC Symbol;Acc:HGNC:1585]
Bos taurus neuronatin (NNAT), transcript variant 1, mRNA. [Source:RefSeq mRNA;Acc:NM_001201324]
Bos taurus histone H2B variant PT15 (LOC404073), mRNA. [Source:RefSeq mRNA;Acc:NM_205774]
Bos taurus hippocalcin-like 1 (HPCAL1), mRNA. [Source:RefSeq mRNA;Acc:NM_001098964]

Bos taurus dual specificity phosphatase 13 (DUSP13), mRNA. [Source:RefSeq mRNA;Acc:NM_001163944]
phakinin [Source:RefSeq peptide;Acc:NP_776673]

unc-5 homolog A (C. elegans) [Source:HGNC Symbol;Acc:HGNC:12567]
WAP four-disulfide core domain 3 [Source:HGNC Symbol;Acc:HGNC:15957]
Uncharacterized protein [Source:UniProtKB/TrEMBL;Acc:G5E6T4]
teashirt zinc finger homeobox 1 [Source:HGNC Symbol;Acc:HGNC:10669]
popeye domain containing 3 [Source:HGNC Symbol;Acc:HGNC:17649]
Bos taurus protein phosphatase 1, regulatory subunit 3B (PPP1R3B), mRNA. [Source:RefSeq mRNA;Acc:NM_001103247]
olfactory receptor, family 4, subfamily N, member 5 [Source:HGNC Symbol;Acc:HGNC:15358]
acyl-CoA thioesterase 8 [Source:HGNC Symbol;Acc:HGNC:15919]
Bos taurus small nuclear ribonucleoprotein 35kDa (U11/U12) (SNRNP35), mRNA. [Source:RefSeq mRNA;Acc:NM_001075556]
peptidoglycan recognition protein 3 [Source:HGNC Symbol;Acc:HGNC:30014]
Bos taurus coiled-coil domain containing 89 (CCDC89), mRNA. [Source:RefSeq mRNA;Acc:NM_001046432]
TATA-box binding protein N-terminal domain splice variant; Uncharacterized protein [Source:UniProtKB/TrEMBL;Acc:C0LUL3]
Bos taurus zinc finger and SCAN domain containing 2 (ZSCAN2), mRNA. [Source:RefSeq mRNA;Acc:NM_001101190]
Bos taurus eukaryotic translation initiation factor 3, subunit J (EIF3J), mRNA. [Source:RefSeq mRNA;Acc:NM_001076143]
chromosome 1 open reading frame 216 [Source:HGNC Symbol;Acc:HGNC:26800]
olfactory receptor, family 3, subfamily A, member 1 [Source:HGNC Symbol;Acc:HGNC:8282]

Bos taurus outer dense fiber of sperm tails 3-like 1 (ODF3L1), mRNA. [Source:RefSeq mRNA;Acc:NM_001075193]
Bos taurus methyltransferase like 21C (METTL21C), mRNA. [Source:RefSeq mRNA;Acc:NM_001100383]
Bos taurus IBA57, iron-sulfur cluster assembly homolog (S. cerevisiae) (IBA57), mRNA. [Source:RefSeq mRNA;Acc:NM_001205580]
Bos taurus tubulin, beta class I (TUBB), mRNA. [Source:RefSeq mRNA;Acc:NM_001046549]
Bos taurus Parkinson disease 7 domain containing 1 (PDDC1), mRNA. [Source:RefSeq mRNA;Acc:NM_001046147]
ring finger protein 187 [Source:HGNC Symbol;Acc:HGNC:27146]
Bos taurus family with sequence similarity 71, member A (FAM71A), mRNA. [Source:RefSeq mRNA;Acc:NM_001099391]
Bos taurus chloride intracellular channel 3 (CLIC3), mRNA. [Source:RefSeq mRNA;Acc:NM_001100301]
Olfactory receptor [Source:UniProtKB/TrEMBL;Acc:F1MI77]
transmembrane protein 177 [Source:HGNC Symbol;Acc:HGNC:28143]

ripply transcriptional repressor 1 [Source:HGNC Symbol;Acc:HGNC:25117]
Bos taurus TAR DNA binding protein (TARDBP), mRNA. [Source:RefSeq mRNA;Acc:NM_001046485]
Uncharacterized protein [Source:UniProtKB/TrEMBL;Acc:G3N3W8]
Bos taurus complement component 1, q subcomponent, C chain (C1QC), mRNA. [Source:RefSeq mRNA;Acc:NM_001206396]
Bos taurus OTU domain, ubiquitin aldehyde binding 1 (OTUB1), mRNA. [Source:RefSeq mRNA;Acc:NM_001034719]
Olfactory receptor [Source:UniProtKB/TrEMBL;Acc:G3N0S3]
Bos taurus homeobox D4 (HOXD4), mRNA. [Source:RefSeq mRNA;Acc:NM_001101087]
CD58 protein; Uncharacterized protein [Source:UniProtKB/TrEMBL;Acc:A7YW37]
family with sequence similarity 64, member A [Source:HGNC Symbol;Acc:HGNC:25483]
Bos taurus NADH dehydrogenase (ubiquinone) 1 alpha subcomplex, 4-like 2 (NDUFA4L2), mRNA. [Source:RefSeq mRNA;Acc:NM_001113305]
double C2-like domain-containing protein gamma [Source:RefSeq peptide;Acc:NP_001069967]
olfactory receptor, family 2, subfamily T, member 1 [Source:HGNC Symbol;Acc:HGNC:8277]
Bos taurus potassium inwardly-rectifying channel, subfamily J, member 16 (KCNJ16), mRNA. [Source:RefSeq mRNA;Acc:NM_001076294]
zinc finger protein 319 [Source:HGNC Symbol;Acc:HGNC:13644]
Bos taurus interferon alpha H (IFNAH), mRNA. [Source:RefSeq mRNA;Acc:NM_001172041]
Bos taurus olfactory receptor-like (LOC519282), mRNA. [Source:RefSeq mRNA;Acc:NM_001206765]
Bos taurus crystallin, gamma S (CRYGS), mRNA. [Source:RefSeq mRNA;Acc:NM_174292]
Bos taurus prostaglandin I2 (prostacyclin) synthase (PTGIS), mRNA. [Source:RefSeq mRNA;Acc:NM_174444]
Bos taurus N(alpha)-acetyltransferase 11, NatA catalytic subunit (NAA11), mRNA. [Source:RefSeq mRNA;Acc:NM_001206411]
Olfactory receptor [Source:UniProtKB/TrEMBL;Acc:G5E5Y2]
Bos taurus Dexi homolog (mouse) (DEXI), mRNA. [Source:RefSeq mRNA;Acc:NM_001191169]
Bos taurus neuro-oncological ventral antigen 2 (NOVA2), mRNA. [Source:RefSeq mRNA;Acc:NM_001206109]
Bos taurus mesoderm development candidate 1 (MESDC1), mRNA. [Source:RefSeq mRNA;Acc:NM_001101258]

Bos taurus chromosome 2 open reading frame, human C1orf172 (C2H1orf172), mRNA. [Source:RefSeq mRNA;Acc:NM_001099103]

Uncharacterized protein [Source:UniProtKB/TrEMBL;Acc:G3MZ23]
Uncharacterized protein [Source:UniProtKB/TrEMBL;Acc:G3X765]
Bos taurus acid phosphatase 6, lysophosphatidic (ACP6), mRNA. [Source:RefSeq mRNA;Acc:NM_001099373]

uridine phosphorylase 1 [Source:HGNC Symbol;Acc:HGNC:12576]
F-box and leucine-rich repeat protein 18 [Source:HGNC Symbol;Acc:HGNC:21874]
potassium inwardly-rectifying channel, subfamily J, member 9 [Source:HGNC Symbol;Acc:HGNC:6270]
zeta-chain (TCR) associated protein kinase 70kDa [Source:HGNC Symbol;Acc:HGNC:12858]
Bos taurus cysteine-rich, DPF motif domain containing 1 (CDPF1), mRNA. [Source:RefSeq mRNA;Acc:NM_001076093]
Uncharacterized protein [Source:UniProtKB/TrEMBL;Acc:E1BJ58]
leucine rich repeat and Ig domain containing 1 [Source:HGNC Symbol;Acc:HGNC:21205]
alpha-kinase 3 [Source:HGNC Symbol;Acc:HGNC:17574]
Bos taurus torsin family 3, member A (TOR3A), mRNA. [Source:RefSeq mRNA;Acc:NM_001080376]
Bos taurus transmembrane protein 185B (TMEM185B), mRNA. [Source:RefSeq mRNA;Acc:NM_001077968]
Bos taurus growth hormone 1 (GH1), mRNA. [Source:RefSeq mRNA;Acc:NM_180996]
Bos taurus vacuolar protein sorting 18 homolog (S. cerevisiae) (VPS18), mRNA. [Source:RefSeq mRNA;Acc:NM_001100380]

Uncharacterized protein [Source:UniProtKB/TrEMBL;Acc:E1BI74]
Bos taurus zinc finger, CCHC domain containing 17 (ZCCHC17), mRNA. [Source:RefSeq mRNA;Acc:NM_001034652]
Bos taurus uroplakin 3B (UPK3B), mRNA. [Source:RefSeq mRNA;Acc:NM_001012672]
Olfactory receptor [Source:UniProtKB/TrEMBL;Acc:E1BMU9]
promyelocytic leukemia [Source:HGNC Symbol;Acc:HGNC:9113]

Bos taurus glutamate-ammonia ligase (GLUL), mRNA. [Source:RefSeq mRNA;Acc:NM_001040474]
insulin receptor substrate 4 [Source:HGNC Symbol;Acc:HGNC:6128]
olfactory receptor, family 6, subfamily A, member 2 [Source:HGNC Symbol;Acc:HGNC:15301]
Bos taurus family with sequence similarity 57, member B (FAM57B), mRNA. [Source:RefSeq mRNA;Acc:NM_001105465]
Bos taurus stathmin-like 4 (STMN4), mRNA. [Source:RefSeq mRNA;Acc:NM_001075580]
transcription factor 20 (AR1) [Source:HGNC Symbol;Acc:HGNC:11631]
Bos taurus Fc gamma 2 receptor (LOC407171), mRNA. [Source:RefSeq mRNA;Acc:NM_001001138]
Olfactory receptor [Source:UniProtKB/TrEMBL;Acc:F1MW42]
G protein-coupled receptor 132 [Source:HGNC Symbol;Acc:HGNC:17482]
chromosome 2 open reading frame 57 [Source:HGNC Symbol;Acc:HGNC:28563]
Bos taurus phosphoribosyl pyrophosphate synthetase-associated protein 1 (PRPSAP1), mRNA. [Source:RefSeq mRNA;Acc:NM_001076857]

Bos taurus hippocalcin (HPCA), mRNA. [Source:RefSeq mRNA;Acc:NM_001025328]
Bos taurus FBJ murine osteosarcoma viral oncogene homolog B (FOSB), mRNA. [Source:RefSeq mRNA;Acc:NM_001102248]
Uncharacterized protein C8orf59 homolog [Source:UniProtKB/Swiss-Prot;Acc:A8YXZ5]
Bos taurus ataxin 7-like 2 (ATXN7L2), mRNA. [Source:RefSeq mRNA;Acc:NM_001102255]
Bos taurus ribosomal protein L15 (RPL15), mRNA. [Source:RefSeq mRNA;Acc:NM_001077866]
Bos taurus ATPase family, AAA domain containing 3A (ATAD3A), mRNA. [Source:RefSeq mRNA;Acc:NM_001105462]
Bos taurus aldolase A, fructose-bisphosphate (ALDOA), mRNA. [Source:RefSeq mRNA;Acc:NM_001101915]
Bos taurus polymerase (DNA-directed), delta interacting protein 2 (POLDIP2), mRNA. [Source:RefSeq mRNA;Acc:NM_001098103]
chromosome 17 open reading frame 97 [Source:HGNC Symbol;Acc:HGNC:33800]
growth factor independent 1B transcription repressor [Source:HGNC Symbol;Acc:HGNC:4238]
cell adhesion molecule 4 [Source:HGNC Symbol;Acc:HGNC:30825]
potassium voltage-gated channel, shaker-related subfamily, member 6 [Source:HGNC Symbol;Acc:HGNC:6225]
CD300e molecule [Source:HGNC Symbol;Acc:HGNC:28874]
Bos taurus phosphodiesterase 12 (PDE12), mRNA. [Source:RefSeq mRNA;Acc:NM_001076133]
Bos taurus bromodomain containing 3 (BRD3), mRNA. [Source:RefSeq mRNA;Acc:NM_001076873]
Bos taurus chemokine (C-X-C motif) receptor 2 (CXCR2), mRNA. [Source:RefSeq mRNA;Acc:NM_001101285]
Bos taurus GTPase, IMAP family member 5 (GIMAP5), mRNA. [Source:RefSeq mRNA;Acc:NM_001079769]
Bos taurus GTPase, IMAP family member 7 (GIMAP7), mRNA. [Source:RefSeq mRNA;Acc:NM_001046308]
G protein-coupled receptor 151 [Source:HGNC Symbol;Acc:HGNC:23624]
Bos taurus transmembrane protein 225-like (LOC513111), mRNA. [Source:RefSeq mRNA;Acc:NM_001098021]
Bos taurus tyrosinase (oculocutaneous albinism IA) (TYR), mRNA. [Source:RefSeq mRNA;Acc:NM_181001]

Bos taurus plasminogen activator, urokinase receptor (PLAUR), mRNA. [Source:RefSeq mRNA;Acc:NM_174423]
Bos taurus ureidopropionase, beta (UPB1), mRNA. [Source:RefSeq mRNA;Acc:NM_001101050]
Bos taurus ring finger protein 19B (RNF19B), mRNA. [Source:RefSeq mRNA;Acc:NM_001205662]
Bos taurus chromosome 7 open reading frame, human C19orf66 (C7H19orf66), mRNA. [Source:RefSeq mRNA;Acc:NM_001038181]
HCLS1 binding protein 3 [Source:HGNC Symbol;Acc:HGNC:24979]
potassium voltage-gated channel, shaker-related subfamily, member 1 (episodic ataxia with myokymia) [Source:HGNC Symbol;Acc:HGNC:6218]
Bos taurus tetratricopeptide repeat domain 9C (TTC9C), mRNA. [Source:RefSeq mRNA;Acc:NM_001083792]
KIAA1328 [Source:HGNC Symbol;Acc:HGNC:29248]

cyclin and CBS domain divalent metal cation transport mediator 3 [Source:HGNC Symbol;Acc:HGNC:104]
Bos taurus MOB kinase activator 3A (MOB3A), mRNA. [Source:RefSeq mRNA;Acc:NM_001034229]
family with sequence similarity 46, member C [Source:HGNC Symbol;Acc:HGNC:24712]
Bos taurus LY6/PLAUR domain containing 3 (LYPD3), mRNA. [Source:RefSeq mRNA;Acc:NM_001083419]
Bos taurus ribonuclease, RNase A family, 13 (non-active) (RNASE13), mRNA. [Source:RefSeq mRNA;Acc:NM_001105426]
T cell receptor alpha variable 29/delta variable 5 (gene/pseudogene) [Source:HGNC Symbol;Acc:HGNC:12127]
Uncharacterized protein [Source:UniProtKB/TrEMBL;Acc:G3N051]
Bos taurus protocadherin 17 (PCDH17), mRNA. [Source:RefSeq mRNA;Acc:NM_001205680]
7SK RNA [Source:RFAM;Acc:RF00100]
Bos taurus death-associated protein (DAP), mRNA. [Source:RefSeq mRNA;Acc:NM_001078058]
teashirt zinc finger homeobox 3 [Source:HGNC Symbol;Acc:HGNC:30700]
forkhead box F1 [Source:HGNC Symbol;Acc:HGNC:3809]
synemin, intermediate filament protein [Source:HGNC Symbol;Acc:HGNC:24466]
Bos taurus gap junction protein, beta 6, 30kDa (GJB6), transcript variant 2, mRNA. [Source:RefSeq mRNA;Acc:NM_001160177]
Bos taurus small EDRK-rich factor 1A (telomeric) (SERF1A), mRNA. [Source:RefSeq mRNA;Acc:NM_001113301]
Bos taurus voltage-dependent anion channel 2 (VDAC2), mRNA. [Source:RefSeq mRNA;Acc:NM_174486]
protocadherin beta 1 [Source:HGNC Symbol;Acc:HGNC:8680]
Uncharacterized protein [Source:UniProtKB/TrEMBL;Acc:F1N6J8]
Bos taurus neurofilament, medium polypeptide (NEFM), mRNA. [Source:RefSeq mRNA;Acc:NM_001244145]
Uncharacterized protein [Source:UniProtKB/TrEMBL;Acc:G5E516]
Bos taurus dermokine (DMKN), mRNA. [Source:RefSeq mRNA;Acc:NM_001082463]
Bos taurus GTPase, IMAP family member 7 (GIMAP7), mRNA. [Source:RefSeq mRNA;Acc:NM_001034771]
pleiomorphic adenoma gene-like 2 [Source:HGNC Symbol;Acc:HGNC:9047]
Bos taurus otolin 1 (OTOL1), mRNA. [Source:RefSeq mRNA;Acc:NM_001192604]
Olfactory receptor [Source:UniProtKB/TrEMBL;Acc:E1BHY7]
short chain dehydrogenase/reductase family 42E, member 2 [Source:HGNC Symbol;Acc:HGNC:35414]
zinc finger protein 469 [Source:HGNC Symbol;Acc:HGNC:23216]

Uncharacterized protein [Source:UniProtKB/TrEMBL;Acc:G3X776]
Bos taurus sialidase 3 (membrane sialidase) (NEU3), mRNA. [Source:RefSeq mRNA;Acc:NM_174122]

polymerase (RNA) III (DNA directed) polypeptide H (22.9kD) [Source:HGNC Symbol;Acc:HGNC:30349]
Uncharacterized protein [Source:UniProtKB/TrEMBL;Acc:G3MYC9]
Uncharacterized protein [Source:UniProtKB/TrEMBL;Acc:F1MGM3]
Bos taurus ring-box 1, E3 ubiquitin protein ligase (RBX1), mRNA. [Source:RefSeq mRNA;Acc:NM_001046241]
Bos taurus neugrin, neurite outgrowth associated (NGRN), transcript variant 2, mRNA. [Source:RefSeq mRNA;Acc:NM_001045992]
Bos taurus synaptopodin 2 (SYNPO2), mRNA. [Source:RefSeq mRNA;Acc:NM_001083751]

interactor of little elongator complex ELL subunit 1 [Source:HGNC Symbol;Acc:HGNC:29154]
Olfactory receptor [Source:UniProtKB/TrEMBL;Acc:E1BD07]
solute carrier family 10, member 5 [Source:HGNC Symbol;Acc:HGNC:22981]
solute carrier family 29 (equilibrative nucleoside transporter), member 4 [Source:HGNC Symbol;Acc:HGNC:23097]
chromosome 9 open reading frame 69 [Source:HGNC Symbol;Acc:HGNC:31009]
Bos taurus PHD finger protein 23 (PHF23), mRNA. [Source:RefSeq mRNA;Acc:NM_001024570]
stomatin (EPB72)-like 1 [Source:HGNC Symbol;Acc:HGNC:14560]
zinc finger and BTB domain containing 46 [Source:HGNC Symbol;Acc:HGNC:16094]
Bos taurus transmembrane and coiled-coil domains 2 (TMCO2), mRNA. [Source:RefSeq mRNA;Acc:NM_001077077]

Bos taurus tyrosyl-tRNA synthetase 2, mitochondrial (YARS2), mRNA. [Source:RefSeq mRNA;Acc:NM_001098088]
Bos taurus succinate dehydrogenase complex assembly factor 2 (SDHAF2), mRNA. [Source:RefSeq mRNA;Acc:NM_001077045]
Bos taurus intercellular adhesion molecule 3 (ICAM3), mRNA. [Source:RefSeq mRNA;Acc:NM_174349]
Uncharacterized protein [Source:UniProtKB/TrEMBL;Acc:E1BHK9]
Olfactory receptor [Source:UniProtKB/TrEMBL;Acc:F1MN82]
Bos taurus SIX homeobox 3 (SIX3), mRNA. [Source:RefSeq mRNA;Acc:NM_001193124]
zinc finger and BTB domain containing 3 [Source:HGNC Symbol;Acc:HGNC:22918]
Bos taurus DEP domain containing MTOR-interacting protein (DEPTOR), mRNA. [Source:RefSeq mRNA;Acc:NM_001102312]
carbohydrate (chondroitin 4) sulfotransferase 11 [Source:HGNC Symbol;Acc:HGNC:17422]
Bos taurus EGF-like-domain, multiple 8 (EGFL8), mRNA. [Source:RefSeq mRNA;Acc:NM_001099187]
Bos taurus calcium channel, voltage-dependent, gamma subunit 2 (CACNG2), mRNA. [Source:RefSeq mRNA;Acc:NM_001077832]
Bos taurus EH-domain containing 2 (EHD2), mRNA. [Source:RefSeq mRNA;Acc:NM_001046431]
Bos taurus glycosyltransferase-like domain containing 2 (GTDC2), mRNA. [Source:RefSeq mRNA;Acc:NM_001011682]

Uncharacterized protein [Source:UniProtKB/TrEMBL;Acc:E1B9H9]
olfactory receptor, family 9, subfamily Q, member 2 [Source:HGNC Symbol;Acc:HGNC:15328]
Bos taurus prostaglandin E synthase 2 (PTGES2), mRNA. [Source:RefSeq mRNA;Acc:NM_001166554]
Uncharacterized protein [Source:UniProtKB/TrEMBL;Acc:G8JL04]

ADP-ribosylation factor-like 14 [Source:HGNC Symbol;Acc:HGNC:22974]
Bos taurus vestigial like 2 (Drosophila) (VGLL2), mRNA. [Source:RefSeq mRNA;Acc:NM_001078032]
Bos taurus bitter taste receptor Bota-T2R10C (T2R10C), mRNA. [Source:RefSeq mRNA;Acc:NM_001046631]
Bos taurus cytochrome c oxidase subunit VIIa polypeptide 2 (liver) (COX7A2), mRNA. [Source:RefSeq mRNA;Acc:NM_175807]
Bos taurus uridine monophosphate synthetase (UMPS), mRNA. [Source:RefSeq mRNA;Acc:NM_177508]
NADH-ubiquinone oxidoreductase chain 3 [Source:UniProtKB/Swiss-Prot;Acc:P03898]
Bos taurus angiopoietin 2 (ANGPT2), mRNA. [Source:RefSeq mRNA;Acc:NM_001098855]
ribosome binding factor A (putative) [Source:HGNC Symbol;Acc:HGNC:26120]
zinc finger protein 446 [Source:HGNC Symbol;Acc:HGNC:21036]
Bos taurus synapse differentiation inducing 1 (SYNDIG1), mRNA. [Source:RefSeq mRNA;Acc:NM_001076263]
casein kinase 1, epsilon [Source:HGNC Symbol;Acc:HGNC:2453]
Bos taurus CD8a molecule (CD8A), mRNA. [Source:RefSeq mRNA;Acc:NM_174015]
Bos taurus phosphomannomutase 1 (PMM1), mRNA. [Source:RefSeq mRNA;Acc:NM_001076083]
gastric intrinsic factor (vitamin B synthesis) [Source:HGNC Symbol;Acc:HGNC:4268]
Bos taurus WAS protein family, member 2 (WASF2), mRNA. [Source:RefSeq mRNA;Acc:NM_001081511]
Olfactory receptor [Source:UniProtKB/TrEMBL;Acc:E1B851]
transcription factor AP-2 delta (activating enhancer binding protein 2 delta) [Source:HGNC Symbol;Acc:HGNC:15581]
Bos taurus mitochondrial ribosomal protein S16 (MRPS16), mRNA. [Source:RefSeq mRNA;Acc:NM_001075474]
GABA(A) receptor-associated protein-like 2 [Source:HGNC Symbol;Acc:HGNC:13291]
Bos taurus ras homolog gene family, member D (RHOD), mRNA. [Source:RefSeq mRNA;Acc:NM_001192338]
Olfactory receptor [Source:UniProtKB/TrEMBL;Acc:F1MEE2]
Bos taurus immunoglobulin superfamily, member 8 (IGSF8), mRNA. [Source:RefSeq mRNA;Acc:NM_001082439]
arginyl aminopeptidase (aminopeptidase B)-like 1 [Source:HGNC Symbol;Acc:HGNC:10079]
tumor necrosis factor (ligand) superfamily, member 14 [Source:HGNC Symbol;Acc:HGNC:11930]
SIX1 protein; Uncharacterized protein [Source:UniProtKB/TrEMBL;Acc:Q08DI4]

glutamic pyruvate transaminase (alanine aminotransferase) 2 [Source:HGNC Symbol;Acc:HGNC:18062]
Bos taurus transducer of ERBB2, 2 (TOB2), mRNA. [Source:RefSeq mRNA;Acc:NM_001083387]
Bos taurus chromosome 19 open reading frame, human C17orf98 (C19H17orf98), mRNA. [Source:RefSeq mRNA;Acc:NM_001206258]
Olfactory receptor [Source:UniProtKB/TrEMBL;Acc:F1MHZ6]
Bos taurus centrin, EF-hand protein, 1 (CETN1), mRNA. [Source:RefSeq mRNA;Acc:NM_001079506]
coiled-coil domain containing 6 [Source:HGNC Symbol;Acc:HGNC:18782]
retinoid X receptor, alpha [Source:HGNC Symbol;Acc:HGNC:10477]
Bos taurus protein phosphatase 1, regulatory (inhibitor) subunit 14C (PPP1R14C), mRNA. [Source:RefSeq mRNA;Acc:NM_001078068]
Bos taurus spermatogenesis and centriole associated 1-like (SPATC1L), mRNA. [Source:RefSeq mRNA;Acc:NM_001038677]
zinc finger protein 786 [Source:HGNC Symbol;Acc:HGNC:21806]
Bos taurus chromosome 25 open reading frame, human C16orf5 (CDIP1), mRNA. [Source:RefSeq mRNA;Acc:NM_001014892]
Bos taurus MHC class II antigen (BLA-DQB), mRNA. [Source:RefSeq mRNA;Acc:NM_001034668]
Bos taurus potassium voltage-gated channel, shaker-related subfamily, member 5 (KCNA5), mRNA. [Source:RefSeq mRNA;Acc:NM_001015552]
Uncharacterized protein [Source:UniProtKB/TrEMBL;Acc:G3N1K6]
chromosome 12 open reading frame 49 [Source:HGNC Symbol;Acc:HGNC:26128]
Bos taurus small nuclear RNA activating complex, polypeptide 2, 45kDa (SNAPC2), mRNA. [Source:RefSeq mRNA;Acc:NM_001034516]
Bos taurus coiled-coil domain containing 96 (CCDC96), mRNA. [Source:RefSeq mRNA;Acc:NM_001103314]
growth differentiation factor 11 [Source:HGNC Symbol;Acc:HGNC:4216]
Uncharacterized protein [Source:UniProtKB/TrEMBL;Acc:F1MNW5]
Williams-Beuren syndrome chromosome region 28 [Source:HGNC Symbol;Acc:HGNC:23018]
potassium channel, subfamily K, member 18 [Source:HGNC Symbol;Acc:HGNC:19439]
G patch domain containing 8 [Source:HGNC Symbol;Acc:HGNC:29066]
Bos taurus chemokine (C-X-C motif) receptor 7 (CXCR7), mRNA. [Source:RefSeq mRNA;Acc:NM_001098381]
Bos taurus hepatitis A virus cellular receptor 1 (HAVCR1), mRNA. [Source:RefSeq mRNA;Acc:NM_001046523]
Bos taurus olfactory receptor, family 4, subfamily D, member 9 (OR4D9), mRNA. [Source:RefSeq mRNA;Acc:NM_001206742]
Uncharacterized protein [Source:UniProtKB/TrEMBL;Acc:F1N2X5]
COMM domain containing 9 [Source:HGNC Symbol;Acc:HGNC:25014]
SH2B adaptor protein 3 [Source:HGNC Symbol;Acc:HGNC:29605]
Bos taurus ESX homeobox 1 (ESX1), mRNA. [Source:RefSeq mRNA;Acc:NM_001079624]
solute carrier family 25 (mitochondrial iron transporter), member 37 [Source:HGNC Symbol;Acc:HGNC:29786]

microseminoprotein, beta- [Source:HGNC Symbol;Acc:HGNC:7372]
homeobox C10 [Source:HGNC Symbol;Acc:HGNC:5122]
Uncharacterized protein [Source:UniProtKB/TrEMBL;Acc:G3N0R0]
Uncharacterized protein [Source:UniProtKB/TrEMBL;Acc:F1MKC3]
Bos taurus non-metastatic cells 4, protein expressed in (NME4), mRNA. [Source:RefSeq mRNA;Acc:NM_001077906]
Bos taurus zinc finger protein 740 (ZNF740), mRNA. [Source:RefSeq mRNA;Acc:NM_001103252]
spalt-like transcription factor 4 [Source:HGNC Symbol;Acc:HGNC:15924]
Bos taurus bitter taste receptor Bota-T2R12 (T2R12), mRNA. [Source:RefSeq mRNA;Acc:NM_001046630]
Bos taurus TIMELESS interacting protein (TIPIN), mRNA. [Source:RefSeq mRNA;Acc:NM_001105336]
Bos taurus casein kinase I beta (CSNK1B), mRNA. [Source:RefSeq mRNA;Acc:NM_001098159]
Bos taurus lectin, galactoside-binding, soluble, 1 (LGALS1), mRNA. [Source:RefSeq mRNA;Acc:NM_175782]
Uncharacterized protein [Source:UniProtKB/TrEMBL;Acc:G3MWM0]

Olfactory receptor [Source:UniProtKB/TrEMBL;Acc:G5E5G0]
Bos taurus opsin 1 (cone pigments), short-wave-sensitive (OPN1SW), mRNA. [Source:RefSeq mRNA;Acc:NM_174567]
chromosome 11 open reading frame 54 [Source:HGNC Symbol;Acc:HGNC:30204]
ubiquitin specific peptidase 27, X-linked [Source:HGNC Symbol;Acc:HGNC:13486]
Uncharacterized protein [Source:UniProtKB/TrEMBL;Acc:E1BPF7]
neuralized E3 ubiquitin protein ligase 1 [Source:HGNC Symbol;Acc:HGNC:7761]
Olfactory receptor [Source:UniProtKB/TrEMBL;Acc:E1BEK5]
heat shock 70kDa protein 6 (HSP70B') [Source:HGNC Symbol;Acc:HGNC:5239]
Bos taurus actin related protein 2/3 complex, subunit 4, 20kDa (ARPC4), mRNA. [Source:RefSeq mRNA;Acc:NM_001076163]
Olfactory receptor [Source:UniProtKB/TrEMBL;Acc:G3X864]
Olfactory receptor [Source:UniProtKB/TrEMBL;Acc:F1N1U2]
Bos taurus zinc finger protein 385A (ZNF385A), mRNA. [Source:RefSeq mRNA;Acc:NM_001206001]

Uncharacterized protein [Source:UniProtKB/TrEMBL;Acc:G3MXP3]
Uncharacterized protein [Source:UniProtKB/TrEMBL;Acc:G3MXJ7]
microfibrillar-associated protein 3-like [Source:HGNC Symbol;Acc:HGNC:29083]
RNA methyltransferase like 1 [Source:HGNC Symbol;Acc:HGNC:18485]

Bos taurus inhibitor of DNA binding 3, dominant negative helix-loop-helix protein (ID3), mRNA. [Source:RefSeq mRNA;Acc:NM_001014950]
rhabdoid tumor deletion region gene 1 [Source:HGNC Symbol;Acc:HGNC:13437]
Bos taurus sulfiredoxin 1 (SRXN1), mRNA. [Source:RefSeq mRNA;Acc:NM_001205988]
Bos taurus chemokine (C-C motif) receptor 1 (CCR1), mRNA. [Source:RefSeq mRNA;Acc:NM_001077839]
Bos taurus transcription factor 23 (TCF23), mRNA. [Source:RefSeq mRNA;Acc:NM_001038216]

Bos taurus Hermansky-Pudlak syndrome 6 (HPS6), mRNA. [Source:RefSeq mRNA;Acc:NM_001081586]
Bos taurus calcium homeostasis modulator 2 (CALHM2), mRNA. [Source:RefSeq mRNA;Acc:NM_001046420]
lymphocyte antigen 6 complex, locus H [Source:HGNC Symbol;Acc:HGNC:6728]
glutaredoxin, cysteine rich 1 [Source:HGNC Symbol;Acc:HGNC:31673]
family with sequence similarity 98, member C [Source:HGNC Symbol;Acc:HGNC:27119]

Olfactory receptor [Source:UniProtKB/TrEMBL;Acc:G3MWV2]
involucrin [Source:HGNC Symbol;Acc:HGNC:6187]
Uncharacterized protein [Source:UniProtKB/TrEMBL;Acc:F1N0A5]
Bos taurus collagen, type X, alpha 1 (COL10A1), mRNA. [Source:RefSeq mRNA;Acc:NM_174634]
HIG1 domain family, member 1D; Uncharacterized protein [Source:UniProtKB/TrEMBL;Acc:Q1LZ70]
CUGBP, Elav-like family member 5 [Source:HGNC Symbol;Acc:HGNC:14058]

Bos taurus family with sequence similarity 155, member A (FAM155A), mRNA. [Source:RefSeq mRNA;Acc:NM_001083776]
Olfactory receptor [Source:UniProtKB/TrEMBL;Acc:E1BE74]
Uncharacterized protein [Source:UniProtKB/TrEMBL;Acc:G3MZA5]
Uncharacterized protein [Source:UniProtKB/TrEMBL;Acc:G3X8G9]
Bos taurus thyroid stimulating hormone, beta (TSHB), mRNA. [Source:RefSeq mRNA;Acc:NM_174205]
Bos taurus fibroblast growth factor 1 (acidic) (FGF1), mRNA. [Source:RefSeq mRNA;Acc:NM_174055]
Uncharacterized protein [Source:UniProtKB/TrEMBL;Acc:E1B8M2]
Olfactory receptor [Source:UniProtKB/TrEMBL;Acc:E1BBH9]
G protein-coupled receptor 83 [Source:HGNC Symbol;Acc:HGNC:4523]
widely interspaced zinc finger motifs [Source:HGNC Symbol;Acc:HGNC:30917]
Bos taurus trophoblast Kunitz domain protein 4 (TKDP4), mRNA. [Source:RefSeq mRNA;Acc:NM_205775]

PR domain containing 2, with ZNF domain [Source:HGNC Symbol;Acc:HGNC:9347]
chromosome 2 open reading frame 71 [Source:HGNC Symbol;Acc:HGNC:34383]
Bos taurus RAB33A, member RAS oncogene family (RAB33A), mRNA. [Source:RefSeq mRNA;Acc:NM_001192993]
DBF4 zinc finger B [Source:HGNC Symbol;Acc:HGNC:17883]

Bos taurus potassium channel regulator (KCNRG), mRNA. [Source:RefSeq mRNA;Acc:NM_205808]

Bos taurus intraflagellar transport 27 homolog (Chlamydomonas) (IFT27), mRNA. [Source:RefSeq mRNA;Acc:NM_001076445]
Olfactory receptor [Source:UniProtKB/TrEMBL;Acc:G5E6G0]
Uncharacterized protein [Source:UniProtKB/TrEMBL;Acc:E1B9B3]
olfactory receptor, family 56, subfamily A, member 3 [Source:HGNC Symbol;Acc:HGNC:14786]
Olfactory receptor [Source:UniProtKB/TrEMBL;Acc:G3X8B2]
Bos taurus coagulation factor X (F10), mRNA. [Source:RefSeq mRNA;Acc:NM_001080213]
Bos taurus 2',3'-cyclic nucleotide 3' phosphodiesterase (CNP), mRNA. [Source:RefSeq mRNA;Acc:NM_180993]
Bos taurus lectin, galactoside-binding, soluble, 3 binding protein (LGALS3BP), mRNA. [Source:RefSeq mRNA;Acc:NM_001046316]
homeobox C6 [Source:HGNC Symbol;Acc:HGNC:5128]
Bos taurus TIMP metallopeptidase inhibitor 3 (TIMP3), mRNA. [Source:RefSeq mRNA;Acc:NM_174473]
Olfactory receptor [Source:UniProtKB/TrEMBL;Acc:E1B6X7]
Bos taurus dehydrogenase/reductase (SDR family) member 13 (DHRS13), mRNA. [Source:RefSeq mRNA;Acc:NM_001075687]
Bos taurus ATP synthase, H+ transporting, mitochondrial F0 complex, subunit C2 (subunit 9) (ATP5G2), mRNA. [Source:RefSeq mRNA;Acc:NM_176613]
Uncharacterized protein [Source:UniProtKB/TrEMBL;Acc:F1N689]
TOX high mobility group box family member 3 [Source:HGNC Symbol;Acc:HGNC:11972]
single-stranded DNA binding protein 1, mitochondrial [Source:HGNC Symbol;Acc:HGNC:11317]
Uncharacterized protein [Source:UniProtKB/TrEMBL;Acc:F1MFF4]
Bos taurus F-box protein 34 (FBXO34), mRNA. [Source:RefSeq mRNA;Acc:NM_001081611]
Bos taurus myotubularin 1 (MTM1), mRNA. [Source:RefSeq mRNA;Acc:NM_001206425]
Bos taurus keratin 27 (KRT27), mRNA. [Source:RefSeq mRNA;Acc:NM_001075815]
Uncharacterized protein [Source:UniProtKB/TrEMBL;Acc:E1B795]

Bos taurus F-box and leucine-rich repeat protein 14 (FBXL14), mRNA. [Source:RefSeq mRNA;Acc:NM_001075947]

Uncharacterized protein [Source:UniProtKB/TrEMBL;Acc:E1B953]
Bos taurus tumor suppressing subtransferable candidate 1 (TSSC1), mRNA. [Source:RefSeq mRNA;Acc:NM_001191328]
Bos taurus tubulin folding cofactor B (TBCB), mRNA. [Source:RefSeq mRNA;Acc:NM_001014939]
Bos taurus peptidyl-tRNA hydrolase 2 (PTRH2), mRNA. [Source:RefSeq mRNA;Acc:NM_001034519]
carbohydrate (N-acetylglucosamine-6-O) sulfotransferase 2 [Source:HGNC Symbol;Acc:HGNC:1970]
Bos taurus RAS, dexamethasone-induced 1 (RASD1), mRNA. [Source:RefSeq mRNA;Acc:NM_001206261]

tripartite motif containing 58 [Source:HGNC Symbol;Acc:HGNC:24150]
repulsive guidance molecule family member a [Source:HGNC Symbol;Acc:HGNC:30308]
ubiquilin 4 [Source:HGNC Symbol;Acc:HGNC:1237]
Uncharacterized protein [Source:UniProtKB/TrEMBL;Acc:F1MV46]
zinc finger and BTB domain containing 39 [Source:HGNC Symbol;Acc:HGNC:29014]
Bos taurus activating transcription factor 4 (tax-responsive enhancer element B67) (ATF4), mRNA. [Source:RefSeq mRNA;Acc:NM_001034342]

Bos taurus DnaJ (Hsp40) homolog, subfamily A, member 1 (DNAJA1), mRNA. [Source:RefSeq mRNA;Acc:NM_001015637]
carcinoembryonic antigen-related cell adhesion molecule 18 [Source:HGNC Symbol;Acc:HGNC:31949]
ankyrin repeat domain 34A [Source:HGNC Symbol;Acc:HGNC:27639]
Olfactory receptor [Source:UniProtKB/TrEMBL;Acc:F1MDY7]
dual specificity phosphatase 27 (putative) [Source:HGNC Symbol;Acc:HGNC:25034]
Olfactory receptor [Source:UniProtKB/TrEMBL;Acc:G5E6Q1]
Bos taurus aldehyde dehydrogenase 3 family, member A1 (ALDH3A1), mRNA. [Source:RefSeq mRNA;Acc:NM_001166513]
Bos taurus transmembrane protein 25 (TMEM25), mRNA. [Source:RefSeq mRNA;Acc:NM_001083639]
Bos taurus aquaporin 2 (collecting duct) (AQP2), mRNA. [Source:RefSeq mRNA;Acc:NM_001101199]

Bos taurus cerebellar degeneration-related protein 2, 62kDa (CDR2), mRNA. [Source:RefSeq mRNA;Acc:NM_001046383]
Bos taurus potassium inwardly-rectifying channel, subfamily J, member 15 (KCNJ15), mRNA. [Source:RefSeq mRNA;Acc:NM_001099018]
Bos taurus TELO2 interacting protein 2 (TTI2), mRNA. [Source:RefSeq mRNA;Acc:NM_001099069]
Bos taurus RASD family, member 2 (RASD2), mRNA. [Source:RefSeq mRNA;Acc:NM_001081594]
spalt-like transcription factor 1 [Source:HGNC Symbol;Acc:HGNC:10524]
C1q and tumor necrosis factor related protein 6 [Source:HGNC Symbol;Acc:HGNC:14343]

Bos taurus chromosome 29 open reading frame, human C11orf48 (C29H11orf48), mRNA. [Source:RefSeq mRNA;Acc:NM_001038581]
Bos taurus nuclear receptor subfamily 1, group H, member 3 (NR1H3), mRNA. [Source:RefSeq mRNA;Acc:NM_001014861]
Bos taurus frizzled family receptor 1 (FZD1), mRNA. [Source:RefSeq mRNA;Acc:NM_001101048]
Bos taurus zinc finger protein 576 (ZNF576), mRNA. [Source:RefSeq mRNA;Acc:NM_001076433]
Bos taurus toll-like receptor 9 (TLR9), mRNA. [Source:RefSeq mRNA;Acc:NM_183081]
chromosome 3 open reading frame 62 [Source:HGNC Symbol;Acc:HGNC:24771]
SET binding protein 1 [Source:HGNC Symbol;Acc:HGNC:15573]
phosphatidylinositol glycan anchor biosynthesis, class V [Source:HGNC Symbol;Acc:HGNC:26031]
Uncharacterized protein [Source:UniProtKB/TrEMBL;Acc:F6Q2H9]
motilin receptor [Source:HGNC Symbol;Acc:HGNC:4495]
melanoma antigen family B, 10 [Source:HGNC Symbol;Acc:HGNC:25377]
60S ribosomal protein L30 [Source:UniProtKB/TrEMBL;Acc:E1B8L7]
Bos taurus sulfotransferase family 4A, member 1 (SULT4A1), mRNA. [Source:RefSeq mRNA;Acc:NM_001076518]
CD248 molecule, endosialin [Source:HGNC Symbol;Acc:HGNC:18219]
Bos taurus sal-like 2 (Drosophila) (SALL2), mRNA. [Source:RefSeq mRNA;Acc:NM_001102112]
zinc finger protein 575 [Source:RefSeq peptide;Acc:NP_001035593]
7SK RNA [Source:RFAM;Acc:RF00100]
Bos taurus leucine rich repeat containing 33 (LRRC33), mRNA. [Source:RefSeq mRNA;Acc:NM_001034391]
F-box and WD repeat domain containing 5 [Source:HGNC Symbol;Acc:HGNC:13613]
olfactory receptor, family 51, subfamily D, member 1 [Source:HGNC Symbol;Acc:HGNC:15193]
Uncharacterized protein [Source:UniProtKB/TrEMBL;Acc:G3MXU5]
Bos taurus ST6 beta-galactosamide alpha-2,6-sialyltranferase 2 (ST6GAL2), mRNA. [Source:RefSeq mRNA;Acc:NM_001008668]
Uncharacterized protein [Source:UniProtKB/TrEMBL;Acc:F1N6G6]
Bos taurus NADH dehydrogenase (ubiquinone) 1 alpha subcomplex, 12 (NDUFA12), mRNA. [Source:RefSeq mRNA;Acc:NM_174306]
Ferritin [Source:UniProtKB/TrEMBL;Acc:F1MMT9]
Bos taurus olfactory receptor 491-like (LOC783210), mRNA. [Source:RefSeq mRNA;Acc:NM_001206666]
Bos taurus mesoderm development candidate 2 (MESDC2), mRNA. [Source:RefSeq mRNA;Acc:NM_001034469]
Bos taurus chemokine (C-C motif) ligand 3 (CCL3), mRNA. [Source:RefSeq mRNA;Acc:NM_174511]
zinc finger, C2HC-type containing 1C [Source:HGNC Symbol;Acc:HGNC:20354]

small G protein signaling modulator 2 [Source:HGNC Symbol;Acc:HGNC:29026]
zinc finger, CCHC domain containing 24 [Source:HGNC Symbol;Acc:HGNC:26911]
Bos taurus integral membrane protein 2C (ITM2C), mRNA. [Source:RefSeq mRNA;Acc:NM_001081538]
chromosome 9 open reading frame 114 [Source:HGNC Symbol;Acc:HGNC:26933]
chromosome 2 open reading frame 49 [Source:HGNC Symbol;Acc:HGNC:28772]
Bos taurus keratin 82 (KRT82), mRNA. [Source:RefSeq mRNA;Acc:NM_001082616]
peroxisomal biogenesis factor 2 [Source:HGNC Symbol;Acc:HGNC:9717]
Bos taurus deleted in primary ciliary dyskinesia homolog (mouse) (DPCD), mRNA. [Source:RefSeq mRNA;Acc:NM_001075745]
Olfactory receptor [Source:UniProtKB/TrEMBL;Acc:G3N0H0]
Uncharacterized protein [Source:UniProtKB/TrEMBL;Acc:E1B959]
Bos taurus torsin family 1, member A (torsin A) (TOR1A), mRNA. [Source:RefSeq mRNA;Acc:NM_001099058]
large tumor suppressor kinase 2 [Source:HGNC Symbol;Acc:HGNC:6515]
Bos taurus ets variant 3-like (ETV3L), mRNA. [Source:RefSeq mRNA;Acc:NM_001192587]
Bos taurus amiloride binding protein 1 (amine oxidase (copper-containing)) (ABP1), mRNA. [Source:RefSeq mRNA;Acc:NM_001034361]
Bos taurus paternally expressed 10 (PEG10), transcript variant 2, mRNA. [Source:RefSeq mRNA;Acc:NM_001109815]
coiled-coil domain containing 166 [Source:HGNC Symbol;Acc:HGNC:41910]
Bos taurus eukaryotic translation elongation factor 1 gamma (EEF1G), mRNA. [Source:RefSeq mRNA;Acc:NM_001040487]
Bos taurus ring finger protein 182 (RNF182), mRNA. [Source:RefSeq mRNA;Acc:NM_001077938]
Bos taurus leucine rich repeat containing 42 (LRRC42), mRNA. [Source:RefSeq mRNA;Acc:NM_001046386]
Bos taurus lysine-rich coiled-coil 1 (KRCC1), mRNA. [Source:RefSeq mRNA;Acc:NM_001075303]
Bos taurus Thy-1 cell surface antigen (THY1), mRNA. [Source:RefSeq mRNA;Acc:NM_001034765]
zinc finger and BTB domain containing 7B [Source:HGNC Symbol;Acc:HGNC:18668]
chromosome 5 open reading frame 52 [Source:HGNC Symbol;Acc:HGNC:35121]

Olfactory receptor [Source:UniProtKB/TrEMBL;Acc:F1MRB3]
Uncharacterized protein [Source:UniProtKB/TrEMBL;Acc:F1MWS7]
solute carrier family 8 (sodium/lithium/calcium exchanger), member B1 [Source:HGNC Symbol;Acc:HGNC:26175]
Bos taurus glycine cleavage system protein H (aminomethyl carrier) (GCSH), mRNA. [Source:RefSeq mRNA;Acc:NM_174844]
Bos taurus guanylate cyclase activator 1B (retina) (GUCA1B), mRNA. [Source:RefSeq mRNA;Acc:NM_174786]
nesprin-4 [Source:RefSeq peptide;Acc:NP_001230216]

repulsive guidance molecule family member b [Source:HGNC Symbol;Acc:HGNC:26896]
forkhead box I3 [Source:HGNC Symbol;Acc:HGNC:35123]
Bos taurus heterogeneous nuclear ribonucleoprotein H2 (H') (HNRNPH2), mRNA. [Source:RefSeq mRNA;Acc:NM_001076008]
protein kinase, AMP-activated, beta 2 non-catalytic subunit [Source:HGNC Symbol;Acc:HGNC:9379]
Olfactory receptor [Source:UniProtKB/TrEMBL;Acc:E1BAQ7]
Bos taurus RRS1 ribosome biogenesis regulator homolog (S. cerevisiae) (RRS1), mRNA. [Source:RefSeq mRNA;Acc:NM_001075481]
Bos taurus gem (nuclear organelle) associated protein 7 (GEMIN7), mRNA. [Source:RefSeq mRNA;Acc:NM_001076489]
Bos taurus ubiquitin-conjugating enzyme E2L 6 (UBE2L6), mRNA. [Source:RefSeq mRNA;Acc:NM_001098917]
Bos taurus CDC42 effector protein (Rho GTPase binding) 2 (CDC42EP2), mRNA. [Source:RefSeq mRNA;Acc:NM_001075341]
paraneoplastic Ma antigen family-like 2 [Source:HGNC Symbol;Acc:HGNC:29206]
UBX domain protein 10 [Source:HGNC Symbol;Acc:HGNC:26354]
Uncharacterized protein [Source:UniProtKB/TrEMBL;Acc:E1BH62]
Bos taurus natural cytotoxicity triggering receptor 1 (NCR1), mRNA. [Source:RefSeq mRNA;Acc:NM_183365]
Bos taurus apoptosis-inducing factor, mitochondrion-associated, 3 (AIFM3), mRNA. [Source:RefSeq mRNA;Acc:NM_001046281]

Bos taurus parathyroid hormone-like hormone (PTHLH), mRNA. [Source:RefSeq mRNA;Acc:NM_174753]
Ferritin [Source:UniProtKB/TrEMBL;Acc:E1BAM4]
Bos taurus exostoses (multiple)-like 1 (EXTL1), mRNA. [Source:RefSeq mRNA;Acc:NM_001100337]
olfactory receptor, family 56, subfamily B, member 4 [Source:HGNC Symbol;Acc:HGNC:15248]
olfactory receptor, family 56, subfamily A, member 1 [Source:HGNC Symbol;Acc:HGNC:14781]
radial spoke head 1 homolog (Chlamydomonas) [Source:HGNC Symbol;Acc:HGNC:12371]
Bos taurus THAP domain containing 4 (THAP4), mRNA. [Source:RefSeq mRNA;Acc:NM_001038669]
Bos taurus fibroblast growth factor receptor substrate 3 (FRS3), mRNA. [Source:RefSeq mRNA;Acc:NM_001098918]

Bos taurus vestigial like 4 (Drosophila) (VGLL4), mRNA. [Source:RefSeq mRNA;Acc:NM_001099128]
spermatogenesis associated 2 [Source:HGNC Symbol;Acc:HGNC:14681]
Bos taurus transcription factor AP-2 beta (activating enhancer binding protein 2 beta) (TFAP2B), mRNA. [Source:RefSeq mRNA;Acc:NM_001076247]
Bos taurus family with sequence similarity 78, member A (FAM78A), mRNA. [Source:RefSeq mRNA;Acc:NM_001038508]
Elongation factor 1-alpha [Source:UniProtKB/TrEMBL;Acc:E1BPF4]
Uncharacterized protein [Source:UniProtKB/TrEMBL;Acc:E1BEW4]

calcium-sensing receptor [Source:HGNC Symbol;Acc:HGNC:1514]
trace amine associated receptor 9 (gene/pseudogene) [Source:HGNC Symbol;Acc:HGNC:20977]
sperm acrosome associated 1 [Source:HGNC Symbol;Acc:HGNC:14967]
homeobox A11 [Source:HGNC Symbol;Acc:HGNC:5101]
Bos taurus coiled-coil-helix-coiled-coil-helix domain containing 2 (CHCHD2), mRNA. [Source:RefSeq mRNA;Acc:NM_001034746]
Bos taurus ribonuclease H2, subunit A (RNASEH2A), mRNA. [Source:RefSeq mRNA;Acc:NM_001038534]
Uncharacterized protein [Source:UniProtKB/TrEMBL;Acc:E1B8G4]
glucocorticoid induced transcript 1 [Source:HGNC Symbol;Acc:HGNC:18713]
Bos taurus CKLF-like MARVEL transmembrane domain containing 4 (CMTM4), mRNA. [Source:RefSeq mRNA;Acc:NM_001083749]

Fermitin family homolog 3 [Source:UniProtKB/TrEMBL;Acc:F1MMJ5]
Bos taurus coiled-coil domain containing 137 (CCDC137), mRNA. [Source:RefSeq mRNA;Acc:NM_001075501]
Bos taurus LanC lantibiotic synthetase component C-like 3 (bacterial) (LANCL3), mRNA. [Source:RefSeq mRNA;Acc:NM_001206408]
Bos taurus bladder cancer associated protein (BLCAP), mRNA. [Source:RefSeq mRNA;Acc:NM_174250]
Uncharacterized protein [Source:UniProtKB/TrEMBL;Acc:G3N2D4]
Bos taurus phosphoribosyl pyrophosphate synthetase 1-like 1 (PRPS1L1), mRNA. [Source:RefSeq mRNA;Acc:NM_001102483]
Bos taurus trefoil factor 3 (intestinal) (TFF3), mRNA. [Source:RefSeq mRNA;Acc:NM_001114509]
Uncharacterized protein [Source:UniProtKB/TrEMBL;Acc:F1MKF9]
Uncharacterized protein [Source:UniProtKB/TrEMBL;Acc:F1N1D9]
Bos taurus phospholipase C-like 2 (PLCL2), mRNA. [Source:RefSeq mRNA;Acc:NM_001192255]
Bos taurus calcium channel flower domain containing 1 (CACFD1), mRNA. [Source:RefSeq mRNA;Acc:NM_001080263]
BCL2-like 13 (apoptosis facilitator) [Source:HGNC Symbol;Acc:HGNC:17164]
receptor (chemosensory) transporter protein 4 [Source:HGNC Symbol;Acc:HGNC:23992]
Uncharacterized protein [Source:UniProtKB/TrEMBL;Acc:E1BCK8]
biotinidase [Source:HGNC Symbol;Acc:HGNC:1122]
BCL2-associated athanogene 3 [Source:HGNC Symbol;Acc:HGNC:939]
Bos taurus tRNA splicing endonuclease 34 homolog (S. cerevisiae) (TSEN34), mRNA. [Source:RefSeq mRNA;Acc:NM_001034251]
Uncharacterized protein [Source:UniProtKB/TrEMBL;Acc:F1MGV7]

carbohydrate (N-acetylgalactosamine 4-sulfate 6-O) sulfotransferase 15 [Source:HGNC Symbol;Acc:HGNC:18137]
Bos taurus decapping enzyme, scavenger (DCPS), mRNA. [Source:RefSeq mRNA;Acc:NM_174695]
Bos taurus solute carrier family 35 (UDP-galactose transporter), member A2 (SLC35A2), mRNA. [Source:RefSeq mRNA;Acc:NM_176640]

Bos taurus ferredoxin reductase (FDXR), mRNA. [Source:RefSeq mRNA;Acc:NM_174691]
Bos taurus rhomboid domain containing 1 (RHBDD1), mRNA. [Source:RefSeq mRNA;Acc:NM_001077995]

Bos taurus neurofilament, light polypeptide (NEFL), mRNA. [Source:RefSeq mRNA;Acc:NM_174121]
Bos taurus E2F transcription factor 1 (E2F1), mRNA. [Source:RefSeq mRNA;Acc:NM_001206079]
Bos taurus transmembrane protein 39B (TMEM39B), mRNA. [Source:RefSeq mRNA;Acc:NM_001075658]
Bos taurus olfactomedin-like 3 (OLFML3), mRNA. [Source:RefSeq mRNA;Acc:NM_001075197]
GTP binding protein 3 (mitochondrial) [Source:HGNC Symbol;Acc:HGNC:14880]
piggyBac transposable element derived 2 [Source:HGNC Symbol;Acc:HGNC:19399]
Bos taurus creatine kinase, muscle (CKM), mRNA. [Source:RefSeq mRNA;Acc:NM_174773]
Bos taurus keratin 18 (KRT18), mRNA. [Source:RefSeq mRNA;Acc:NM_001192095]
Bos taurus carbohydrate (N-acetylgalactosamine 4-0) sulfotransferase 14 (CHST14), mRNA. [Source:RefSeq mRNA;Acc:NM_001098012]
Bos taurus brain expressed, X-linked 5 (BEX5), mRNA. [Source:RefSeq mRNA;Acc:NM_001075735]
Olfactory receptor [Source:UniProtKB/TrEMBL;Acc:F1MPX7]
Bos taurus pipecolic acid oxidase (PIPOX), mRNA. [Source:RefSeq mRNA;Acc:NM_001014878]
Bos taurus Cbp/p300-interacting transactivator, with Glu/Asp-rich carboxy-terminal domain, 1 (CITED1), mRNA. [Source:RefSeq mRNA;Acc:NM_174518]
ST8 alpha-N-acetyl-neuraminide alpha-2,8-sialyltransferase 4 [Source:HGNC Symbol;Acc:HGNC:10871]
Uncharacterized protein [Source:UniProtKB/TrEMBL;Acc:G3N2C0]
ZMYM6 neighbor [Source:HGNC Symbol;Acc:HGNC:40021]
Bos taurus chromodomain protein, Y-like 2 (CDYL2), mRNA. [Source:RefSeq mRNA;Acc:NM_001163785]
Uncharacterized protein [Source:UniProtKB/TrEMBL;Acc:G3X844]
Bos taurus cholinergic receptor, muscarinic 1 (CHRM1), mRNA. [Source:RefSeq mRNA;Acc:NM_001244609]

Bos taurus nerve growth factor receptor (TNFRSF16) associated protein 1 (NGFRAP1), mRNA. [Source:RefSeq mRNA;Acc:NM_001163777]
Bos taurus one cut homeobox 1 (ONECUT1), mRNA. [Source:RefSeq mRNA;Acc:NM_001191161]
Bos taurus glucose-6-phosphatase, catalytic, 2 (G6PC2), mRNA. [Source:RefSeq mRNA;Acc:NM_001192046]

Uncharacterized protein [Source:UniProtKB/TrEMBL;Acc:E1BLE9]
Uncharacterized protein [Source:UniProtKB/TrEMBL;Acc:F1ML34]
nudix (nucleoside diphosphate linked moiety X)-type motif 18 [Source:HGNC Symbol;Acc:HGNC:26194]

ACD protein; Uncharacterized protein [Source:UniProtKB/TrEMBL;Acc:A6QQ10]
chromosome 12 open reading frame 60 [Source:HGNC Symbol;Acc:HGNC:28726]
RNA binding motif protein 15 [Source:HGNC Symbol;Acc:HGNC:14959]
zinc finger and BTB domain containing 22 [Source:HGNC Symbol;Acc:HGNC:13085]
Bos taurus proline rich 14 (PRR14), mRNA. [Source:RefSeq mRNA;Acc:NM_001035396]
Bos taurus colony stimulating factor 3 (granulocyte) (CSF3), mRNA. [Source:RefSeq mRNA;Acc:NM_174028]
zinc finger protein 599 [Source:HGNC Symbol;Acc:HGNC:26408]
Bos taurus endothelin 2 (EDN2), mRNA. [Source:RefSeq mRNA;Acc:NM_175714]
Bos taurus myotubularin related protein 9-like (MTMR9), mRNA. [Source:RefSeq mRNA;Acc:NM_001046256]
Bos taurus chromosome X open reading frame, human CXorf36 (CXHXorf36), mRNA. [Source:RefSeq mRNA;Acc:NM_001035495]
Bos taurus sparc/osteonectin, cwcv and kazal-like domains proteoglycan (testican) 1 (SPOCK1), mRNA. [Source:RefSeq mRNA;Acc:NM_001075500]
cornichon family AMPA receptor auxiliary protein 3 [Source:HGNC Symbol;Acc:HGNC:26802]

Bos taurus GATS protein-like 3 (GATSL3), mRNA. [Source:RefSeq mRNA;Acc:NM_001081514]
lipocalin 2 [Source:HGNC Symbol;Acc:HGNC:6526]
Bos taurus potassium voltage-gated channel, delayed-rectifier, subfamily S, member 3 (KCNS3), mRNA. [Source:RefSeq mRNA;Acc:NM_001013606]
olfactory receptor, family 2, subfamily V, member 1 [Source:HGNC Symbol;Acc:HGNC:8280]
Bos taurus solute carrier family 25, member 33 (SLC25A33), mRNA. [Source:RefSeq mRNA;Acc:NM_001076002]
Bos taurus mastermind-like 2 (Drosophila) (MAML2), mRNA. [Source:RefSeq mRNA;Acc:NM_001098050]
TSC22 domain family, member 2 [Source:HGNC Symbol;Acc:HGNC:29095]
Uncharacterized protein [Source:UniProtKB/TrEMBL;Acc:F1MJM2]
Olfactory receptor [Source:UniProtKB/TrEMBL;Acc:F1MH86]
Uncharacterized protein [Source:UniProtKB/TrEMBL;Acc:F1N4J0]
Bos taurus peripheral myelin protein 22 (PMP22), mRNA. [Source:RefSeq mRNA;Acc:NM_001101156]
Bos taurus transient receptor potential cation channel, subfamily C, member 4 (TRPC4), mRNA. [Source:RefSeq mRNA;Acc:NM_174478]
Bos taurus coactosin-like 1 (Dictyostelium) (COTL1), mRNA. [Source:RefSeq mRNA;Acc:NM_001046593]

Bos taurus fibroblast growth factor binding protein 1 (FGFBP1), mRNA. [Source:RefSeq mRNA;Acc:NM_174337]
Bos taurus WD repeat domain 20 (WDR20), mRNA. [Source:RefSeq mRNA;Acc:NM_001191156]
Bos taurus nuclear transcription factor Y, beta (NFYB), mRNA. [Source:RefSeq mRNA;Acc:NM_001079786]
Bos taurus profilin 1 (PFN1), mRNA. [Source:RefSeq mRNA;Acc:NM_001015592]
RAB34, member RAS oncogene family [Source:HGNC Symbol;Acc:HGNC:16519]
Bos taurus neuroligin 1 (NLGN1), mRNA. [Source:RefSeq mRNA;Acc:NM_001205973]
Aldehyde dehydrogenase X, mitochondrial [Source:UniProtKB/Swiss-Prot;Acc:P52476]

Bos taurus transducin (beta)-like 3 (TBL3), mRNA. [Source:RefSeq mRNA;Acc:NM_001046619]
Bos taurus TP53 regulating kinase (TP53RK), mRNA. [Source:RefSeq mRNA;Acc:NM_001099180]
Bos taurus selenoprotein O (SELO), mRNA. [Source:RefSeq mRNA;Acc:NM_001163193]
Rho guanine nucleotide exchange factor (GEF) 19 [Source:HGNC Symbol;Acc:HGNC:26604]
Bos taurus integrin-binding sialoprotein (IBSP), mRNA. [Source:RefSeq mRNA;Acc:NM_174084]
Bos taurus v-maf musculoaponeurotic fibrosarcoma oncogene homolog G (avian) (MAFG), mRNA. [Source:RefSeq mRNA;Acc:NM_001098980]
Bos taurus homeobox C11 (HOXC11), mRNA. [Source:RefSeq mRNA;Acc:NM_001192873]
methyl-CpG binding domain protein 2 [Source:HGNC Symbol;Acc:HGNC:6917]
Bos taurus family with sequence similarity 193, member B (FAM193B), mRNA. [Source:RefSeq mRNA;Acc:NM_001046433]
Olfactory receptor [Source:UniProtKB/TrEMBL;Acc:F1MNK9]
Bos taurus uncharacterized LOC618367 (LOC618367), mRNA. [Source:RefSeq mRNA;Acc:NM_001105009]
sosondowah ankyrin repeat domain family member B [Source:HGNC Symbol;Acc:HGNC:32958]
Bos taurus cadherin 3, type 1, P-cadherin (placental) (CDH3), mRNA. [Source:RefSeq mRNA;Acc:NM_001244605]
Bos taurus cytochrome P450, family 26, subfamily B, polypeptide 1 (CYP26B1), mRNA. [Source:RefSeq mRNA;Acc:NM_001192793]
Olfactory receptor [Source:UniProtKB/TrEMBL;Acc:F1MFV6]
Bos taurus adhesion molecule with Ig-like domain 1 (AMIGO1), mRNA. [Source:RefSeq mRNA;Acc:NM_001101119]
dermatan sulfate epimerase-like [Source:HGNC Symbol;Acc:HGNC:18144]
Bos taurus cell growth regulator with EF-hand domain 1 (CGREF1), mRNA. [Source:RefSeq mRNA;Acc:NM_001045977]
Uncharacterized protein [Source:UniProtKB/TrEMBL;Acc:F1MER5]
olfactory receptor, family 51, subfamily T, member 1 [Source:HGNC Symbol;Acc:HGNC:15205]
NK2 homeobox 1 [Source:HGNC Symbol;Acc:HGNC:11825]
Uncharacterized protein [Source:UniProtKB/TrEMBL;Acc:G3MYI7]

UPF0730 protein LINC00643 homolog [Source:UniProtKB/Swiss-Prot;Acc:A6QPD8]
Bos taurus protein phosphatase 1, regulatory subunit 18 (PPP1R18), mRNA. [Source:RefSeq mRNA;Acc:NM_001076919]
Bos taurus solute carrier family 9 (sodium/hydrogen exchanger), member 1 (SLC9A1), mRNA. [Source:RefSeq mRNA;Acc:NM_174833]
Bos taurus DMRT-like family A1 (DMRTA1), mRNA. [Source:RefSeq mRNA;Acc:NM_001192640]
Bos taurus leucine-rich alpha-2-glycoprotein 1 (LRG1), mRNA. [Source:RefSeq mRNA;Acc:NM_001046177]
membrane-spanning 4-domains, subfamily A, member 10 [Source:HGNC Symbol;Acc:HGNC:13368]
Uncharacterized protein [Source:UniProtKB/TrEMBL;Acc:G3MXS3]
Bos taurus within bgcn homolog (Drosophila) (WIBG), mRNA. [Source:RefSeq mRNA;Acc:NM_001102344]
Bos taurus olfactory receptor, family 4, subfamily D, member 5 (OR4D5), mRNA. [Source:RefSeq mRNA;Acc:NM_001206740]
chromosome 16 open reading frame 59 [Source:HGNC Symbol;Acc:HGNC:25849]
Olfactory receptor [Source:UniProtKB/TrEMBL;Acc:G3MYI1]
Bos taurus uncharacterized LOC524771 (LOC524771), mRNA. [Source:RefSeq mRNA;Acc:NM_001098057]
Bos taurus eukaryotic translation initiation factor 4E binding protein 2 (EIF4EBP2), mRNA. [Source:RefSeq mRNA;Acc:NM_001191149]
Uncharacterized protein [Source:UniProtKB/TrEMBL;Acc:G3MXW8]
Olfactory receptor [Source:UniProtKB/TrEMBL;Acc:G3N0P7]
dehydrogenase/reductase (SDR family) member 7B [Source:HGNC Symbol;Acc:HGNC:24547]
Uncharacterized protein [Source:UniProtKB/TrEMBL;Acc:G5E574]

Bos taurus actin, alpha 1, skeletal muscle (ACTA1), mRNA. [Source:RefSeq mRNA;Acc:NM_174225]
Bos taurus SWAP switching B-cell complex 70kDa subunit (SWAP70), mRNA. [Source:RefSeq mRNA;Acc:NM_001080297]

transmembrane protein 192 [Source:HGNC Symbol;Acc:HGNC:26775]
leucine zipper, putative tumor suppressor 2 [Source:HGNC Symbol;Acc:HGNC:29381]
Rieske (Fe-S) domain containing [Source:HGNC Symbol;Acc:HGNC:29587]
Bos taurus calmodulin 3 (phosphorylase kinase, delta) (CALM3), mRNA. [Source:RefSeq mRNA;Acc:NM_001046249]
Bos taurus connective tissue growth factor (CTGF), mRNA. [Source:RefSeq mRNA;Acc:NM_174030]

Bos taurus COX19 cytochrome c oxidase assembly homolog (S. cerevisiae) (COX19), mRNA. [Source:RefSeq mRNA;Acc:NM_001109966]
Bos taurus ProSAPiP1 protein (PROSAPIP1), mRNA. [Source:RefSeq mRNA;Acc:NM_001191200]
Uncharacterized protein [Source:UniProtKB/TrEMBL;Acc:G5E5D7]

Bos taurus DET1 and DDB1 associated 1 (DDA1), mRNA. [Source:RefSeq mRNA;Acc:NM_001015583]
Bos taurus HIV-1 Tat specific factor 1 (HTATSF1), mRNA. [Source:RefSeq mRNA;Acc:NM_001206414]
SLAM family member 9 [Source:HGNC Symbol;Acc:HGNC:18430]
myosin light chain kinase family, member 4 [Source:HGNC Symbol;Acc:HGNC:27972]
Guanine nucleotide-binding protein subunit gamma [Source:UniProtKB/TrEMBL;Acc:G3MYD3]
purinergic receptor P2Y, G-protein coupled, 2 [Source:HGNC Symbol;Acc:HGNC:8541]
Bos taurus HMP19 protein (HMP19), mRNA. [Source:RefSeq mRNA;Acc:NM_001076296]
Bos taurus olfactory receptor 52N4-like (LOC100847801), mRNA. [Source:RefSeq mRNA;Acc:NM_001255967]
zinc finger protein 623 [Source:HGNC Symbol;Acc:HGNC:29084]
neuronal differentiation 4 [Source:HGNC Symbol;Acc:HGNC:13802]
Bos taurus interleukin 36 receptor antagonist (IL36RN), mRNA. [Source:RefSeq mRNA;Acc:NM_001075775]
Uncharacterized protein [Source:UniProtKB/TrEMBL;Acc:G3MZI0]
cytochrome c oxidase subunit I [Source:RefSeq peptide;Acc:YP_209207]
Bos taurus T-cell leukemia translocation altered gene (TCTA), mRNA. [Source:RefSeq mRNA;Acc:NM_001034789]
Bos taurus signal peptidase complex subunit 1 homolog (S. cerevisiae) (SPCS1), mRNA. [Source:RefSeq mRNA;Acc:NM_001034404]
Uncharacterized protein [Source:UniProtKB/TrEMBL;Acc:G3N1P6]
5-hydroxytryptamine (serotonin) receptor 1D, G protein-coupled [Source:HGNC Symbol;Acc:HGNC:5289]
zinc finger protein 438 [Source:HGNC Symbol;Acc:HGNC:21029]
kelch-like family member 21 [Source:HGNC Symbol;Acc:HGNC:29041]
Uncharacterized protein [Source:UniProtKB/TrEMBL;Acc:G3N223]
Olfactory receptor [Source:UniProtKB/TrEMBL;Acc:E1BPA2]
leiomodin 2 (cardiac) [Source:HGNC Symbol;Acc:HGNC:6648]
Bos taurus ASF1 anti-silencing function 1 homolog A (S. cerevisiae) (ASF1A), mRNA. [Source:RefSeq mRNA;Acc:NM_001076493]
Spi-1 proto-oncogene [Source:HGNC Symbol;Acc:HGNC:11241]
Olfactory receptor [Source:UniProtKB/TrEMBL;Acc:F1N452]
zinc finger protein 772 [Source:HGNC Symbol;Acc:HGNC:33106]
C1H21ORF7 protein; Uncharacterized protein [Source:UniProtKB/TrEMBL;Acc:Q0VC45]
Bos taurus tubulin, alpha 8 (TUBA8), mRNA. [Source:RefSeq mRNA;Acc:NM_001077095]
Uncharacterized protein [Source:UniProtKB/TrEMBL;Acc:G5E6I0]
sex comb on midleg-like 1 (Drosophila) [Source:HGNC Symbol;Acc:HGNC:10580]

Olfactory receptor [Source:UniProtKB/TrEMBL;Acc:G3X815]
Bos taurus olfactory receptor Olr1242-like (LOC787625), mRNA. [Source:RefSeq mRNA;Acc:NM_001206685]
Bos taurus sodium channel, voltage-gated, type III, beta (SCN3B), mRNA. [Source:RefSeq mRNA;Acc:NM_001046495]
CMT1A duplicated region transcript 4 [Source:HGNC Symbol;Acc:HGNC:14383]
Bos taurus transducin (beta)-like 2 (TBL2), mRNA. [Source:RefSeq mRNA;Acc:NM_001075535]
Bos taurus chromosome 25 open reading frame, human C16orf82 (C25H16orf82), mRNA. [Source:RefSeq mRNA;Acc:NM_001145758]
Bos taurus interferon regulatory factor 6 (IRF6), mRNA. [Source:RefSeq mRNA;Acc:NM_001076934]
Bos taurus eukaryotic elongation factor, selenocysteine-tRNA-specific (EEFSEC), mRNA. [Source:RefSeq mRNA;Acc:NM_001083464]
Bos taurus PARK2 co-regulated (PACRG), mRNA. [Source:RefSeq mRNA;Acc:NM_001077027]
Olfactory receptor [Source:UniProtKB/TrEMBL;Acc:F1MGG9]
A kinase (PRKA) anchor protein 12 [Source:HGNC Symbol;Acc:HGNC:370]
Uncharacterized protein [Source:UniProtKB/TrEMBL;Acc:G3N281]

zinc finger and BTB domain containing 7C [Source:HGNC Symbol;Acc:HGNC:31700]
potassium voltage-gated channel, subfamily G, member 1 [Source:HGNC Symbol;Acc:HGNC:6248]
Bos taurus cholinergic receptor, muscarinic 5 (CHRM5), mRNA. [Source:RefSeq mRNA;Acc:NM_001244611]
retrotransposon gag domain containing 1 [Source:HGNC Symbol;Acc:HGNC:29245]
olfactory receptor, family 11, subfamily H, member 4 [Source:HGNC Symbol;Acc:HGNC:15347]
Uncharacterized protein [Source:UniProtKB/TrEMBL;Acc:E1BNS4]
forkhead box O3 [Source:HGNC Symbol;Acc:HGNC:3821]
Bos taurus serine/arginine-rich splicing factor 4 (SRSF4), mRNA. [Source:RefSeq mRNA;Acc:NM_001101231]
coiled-coil domain containing 82 [Source:HGNC Symbol;Acc:HGNC:26282]
Uncharacterized protein [Source:UniProtKB/TrEMBL;Acc:G3N353]
Bos taurus abhydrolase domain containing 8 (ABHD8), mRNA. [Source:RefSeq mRNA;Acc:NM_001078123]
Bos taurus lactamase, beta (LACTB), mRNA. [Source:RefSeq mRNA;Acc:NM_001099133]
Bos taurus progestin and adipoQ receptor family member VIII (PAQR8), mRNA. [Source:RefSeq mRNA;Acc:NM_001101135]
Bos taurus transmembrane emp24 domain trafficking protein 2 (TMED2), mRNA. [Source:RefSeq mRNA;Acc:NM_001159818]
Bos taurus pannexin 1 (PANX1), mRNA. [Source:RefSeq mRNA;Acc:NM_001245925]
Bos taurus ethylmalonic encephalopathy 1 (ETHE1), mRNA. [Source:RefSeq mRNA;Acc:NM_001034344]
Bos taurus glycerophosphodiester phosphodiesterase 1 (GDE1), mRNA. [Source:RefSeq mRNA;Acc:NM_001034686]

agmatine ureohydrolase (agmatinase) [Source:HGNC Symbol;Acc:HGNC:18407]
Bos taurus lysyl oxidase-like 1 (LOXL1), mRNA. [Source:RefSeq mRNA;Acc:NM_174383]
Bos taurus uncoupling protein 1 (mitochondrial, proton carrier) (UCP1), nuclear gene encoding mitochondrial protein, mRNA. [Source:RefSeq mRNA;Acc:NM_0(
Bos taurus ecotropic viral integration site 2B (EVI2B), mRNA. [Source:RefSeq mRNA;Acc:NM_001099166]
Bos taurus homocysteine-inducible, endoplasmic reticulum stress-inducible, ubiquitin-like domain member 1 (HERPUD1), mRNA. [Source:RefSeq mRNA;Acc:NM
dual specificity protein phosphatase 3 [Source:RefSeq peptide;Acc:NP_001069842]
transmembrane and immunoglobulin domain containing 2 [Source:HGNC Symbol;Acc:HGNC:28324]
Bos taurus protein HP-20 homolog (MGC137014), mRNA. [Source:RefSeq mRNA;Acc:NM_001046584]
Olfactory receptor [Source:UniProtKB/TrEMBL;Acc:F1MJ40]
chromosome 21 open reading frame 62 [Source:HGNC Symbol;Acc:HGNC:1305]

Uncharacterized protein [Source:UniProtKB/TrEMBL;Acc:E1BLF3]
repetin [Source:HGNC Symbol;Acc:HGNC:26809]

Uncharacterized protein [Source:UniProtKB/TrEMBL;Acc:E1B8F8]
Uncharacterized protein [Source:UniProtKB/TrEMBL;Acc:F1MGM9]
proline rich 30 [Source:HGNC Symbol;Acc:HGNC:28677]
Uncharacterized protein [Source:UniProtKB/TrEMBL;Acc:G3N2X4]
Bos taurus activating transcription factor 3 (ATF3), mRNA. [Source:RefSeq mRNA;Acc:NM_001046193]
stearoyl-CoA desaturase (delta-9-desaturase) [Source:HGNC Symbol;Acc:HGNC:10571]
Bos taurus LIM domain only 2 (rhombotin-like 1) (LMO2), mRNA. [Source:RefSeq mRNA;Acc:NM_001076352]
Bos taurus HSPA (heat shock 70kDa) binding protein, cytoplasmic cochaperone 1 (HSPBP1), mRNA. [Source:RefSeq mRNA;Acc:NM_001046131]
purine-rich element binding protein G [Source:HGNC Symbol;Acc:HGNC:17930]
Bos taurus GID complex subunit 8 homolog (S. cerevisiae) (GID8), mRNA. [Source:RefSeq mRNA;Acc:NM_001037587]
Uncharacterized protein [Source:UniProtKB/TrEMBL;Acc:F1MZQ7]
proline dehydrogenase (oxidase) 2 [Source:HGNC Symbol;Acc:HGNC:17325]
Bos taurus thyrotropin-releasing hormone receptor (TRHR), mRNA. [Source:RefSeq mRNA;Acc:NM_174203]
Bos taurus urocortin 3 (stresscopin) (UCN3), mRNA. [Source:RefSeq mRNA;Acc:NM_001076527]
Bos taurus LIM homeobox 4 (LHX4), mRNA. [Source:RefSeq mRNA;Acc:NM_001192785]

Bos taurus serine/threonine kinase 40 (STK40), mRNA. [Source:RefSeq mRNA;Acc:NM_001075727]
transmembrane protein 170B [Source:HGNC Symbol;Acc:HGNC:34244]
Bos taurus tetratricopeptide repeat domain 15 (TTC15), mRNA. [Source:RefSeq mRNA;Acc:NM_001102290]
Bos taurus leucine rich repeat neuronal 2 (LRRN2), mRNA. [Source:RefSeq mRNA;Acc:NM_001076971]
trace amine associated receptor 5 [Source:HGNC Symbol;Acc:HGNC:30236]
Uncharacterized protein [Source:UniProtKB/TrEMBL;Acc:E1BLY1]
Uncharacterized protein [Source:UniProtKB/TrEMBL;Acc:G3X679]
Bos taurus pyruvate dehyrogenase phosphatase catalytic subunit 1 (PDP1), mRNA. [Source:RefSeq mRNA;Acc:NM_001206353]
Bos taurus catenin (cadherin-associated protein), alpha 2 (CTNNA2), mRNA. [Source:RefSeq mRNA;Acc:NM_001102110]
doublecortin-like kinase 2 [Source:HGNC Symbol;Acc:HGNC:19002]
Bos taurus aminoacyl tRNA synthetase complex-interacting multifunctional protein 2 (AIMP2), mRNA. [Source:RefSeq mRNA;Acc:NM_001075813]
Uncharacterized protein [Source:UniProtKB/TrEMBL;Acc:E1BMY1]
Bos taurus free fatty acid receptor 3 (FFAR3), mRNA. [Source:RefSeq mRNA;Acc:NM_001145233]

Bos taurus CD14 molecule (CD14), mRNA. [Source:RefSeq mRNA;Acc:NM_174008]
Bos taurus FERM domain containing 8 (FRMD8), mRNA. [Source:RefSeq mRNA;Acc:NM_001098985]
Bos taurus SCO cytochrome oxidase deficient homolog 1 (yeast) (SCO1), mRNA. [Source:RefSeq mRNA;Acc:NM_001080243]

Bos taurus target of myb1 (chicken) (TOM1), mRNA. [Source:RefSeq mRNA;Acc:NM_001035015]
protein quaking [Source:RefSeq peptide;Acc:NP_001007818]
Bos taurus transmembrane protein 161A (TMEM161A), mRNA. [Source:RefSeq mRNA;Acc:NM_001098144]
Bos taurus heart and neural crest derivatives expressed 1 (HAND1), mRNA. [Source:RefSeq mRNA;Acc:NM_001075761]
Bos taurus guanine nucleotide binding protein (G protein), q polypeptide (GNAQ), mRNA. [Source:RefSeq mRNA;Acc:NM_001110002]
histamine receptor H4 [Source:HGNC Symbol;Acc:HGNC:17383]
reticulon 4 receptor-like 1 [Source:HGNC Symbol;Acc:HGNC:21329]
Olfactory receptor [Source:UniProtKB/TrEMBL;Acc:G5E6K0]
Olfactory receptor [Source:UniProtKB/TrEMBL;Acc:E1BM68]
Uncharacterized protein [Source:UniProtKB/TrEMBL;Acc:F1MSX2]
Bos taurus growth arrest and DNA-damage-inducible, beta (GADD45B), mRNA. [Source:RefSeq mRNA;Acc:NM_001040604]
peptide deformylase (mitochondrial) [Source:HGNC Symbol;Acc:HGNC:30012]

Bos taurus vasodilator-stimulated phosphoprotein (VASP), mRNA. [Source:RefSeq mRNA;Acc:NM_001038110]
Olfactory receptor [Source:UniProtKB/TrEMBL;Acc:F1MDS8]
Bos taurus solute carrier family 25, member 41 (SLC25A41), mRNA. [Source:RefSeq mRNA;Acc:NM_001075841]
Bos taurus growth differentiation factor 2 (GDF2), mRNA. [Source:RefSeq mRNA;Acc:NM_001192420]
pyruvate dehyrogenase phosphatase catalytic subunit 2 [Source:HGNC Symbol;Acc:HGNC:30263]
Bos taurus coronin 6 (CORO6), mRNA. [Source:RefSeq mRNA;Acc:NM_001102282]
chromosome 3 open reading frame 38 [Source:HGNC Symbol;Acc:HGNC:28384]
Bos taurus peptidylprolyl isomerase A (cyclophilin A) (PPIA), mRNA. [Source:RefSeq mRNA;Acc:NM_178320]
aspartate beta-hydroxylase domain containing 2 [Source:HGNC Symbol;Acc:HGNC:30437]
Uncharacterized protein [Source:UniProtKB/TrEMBL;Acc:F1MKA7]
zinc finger protein 774 [Source:RefSeq peptide;Acc:NP_001096795]
Bos taurus complexin 3 (CPLX3), mRNA. [Source:RefSeq mRNA;Acc:NM_001077889]
Bos taurus leucine-rich repeat, immunoglobulin-like and transmembrane domains 1 (LRIT1), mRNA. [Source:RefSeq mRNA;Acc:NM_001192115]
Uncharacterized protein [Source:UniProtKB/TrEMBL;Acc:F1N6H5]
zinc finger and AT hook domain containing [Source:HGNC Symbol;Acc:HGNC:19899]
Olfactory receptor [Source:UniProtKB/TrEMBL;Acc:G3X889]
Bos taurus chromosome 16 open reading frame, human C1orf116 (C16H1orf116), mRNA. [Source:RefSeq mRNA;Acc:NM_001098464]
Bos taurus interferon-induced protein with tetratricopeptide repeats 3 (IFIT3), mRNA. [Source:RefSeq mRNA;Acc:NM_001075414]
Bos taurus yippee-like 3 (Drosophila) (YPEL3), mRNA. [Source:RefSeq mRNA;Acc:NM_001205409]
Bos taurus ribosomal protein L22-like 1 (RPL22L1), mRNA. [Source:RefSeq mRNA;Acc:NM_001098131]
Bos taurus EF-hand domain family, member D2 (EFHD2), mRNA. [Source:RefSeq mRNA;Acc:NM_001103245]
Uncharacterized protein [Source:UniProtKB/TrEMBL;Acc:G3X8C2]
Bos taurus uncharacterized LOC781895 (LOC781895), mRNA. [Source:RefSeq mRNA;Acc:NM_001144075]
Olfactory receptor [Source:UniProtKB/TrEMBL;Acc:F1MSB0]
Bos taurus coiled-coil domain containing 115 (CCDC115), mRNA. [Source:RefSeq mRNA;Acc:NM_001034444]
Bos taurus popeye domain containing 2 (POPDC2), mRNA. [Source:RefSeq mRNA;Acc:NM_001099112]
Bos taurus processing of precursor 5, ribonuclease P/MRP subunit (S. cerevisiae) (POP5), mRNA. [Source:RefSeq mRNA;Acc:NM_001075312]
Bos taurus mesenchyme homeobox 2 (MEOX2), mRNA. [Source:RefSeq mRNA;Acc:NM_001098045]
Bos taurus RAB15, member RAS onocogene family (RAB15), mRNA. [Source:RefSeq mRNA;Acc:NM_001046538]
kelch-like family member 32 [Source:HGNC Symbol;Acc:HGNC:21221]

Bos taurus CD300 molecule-like family member b (CD300LB), mRNA. [Source:RefSeq mRNA;Acc:NM_001105023]
SLP adaptor and CSK interacting membrane protein [Source:HGNC Symbol;Acc:HGNC:33504]
Bos taurus chromosome 29 open reading frame, human C11orf84 (C29H11orf84), mRNA. [Source:RefSeq mRNA;Acc:NM_001205765]
ankyrin repeat and LEM domain containing 2 [Source:HGNC Symbol;Acc:HGNC:29101]
Uncharacterized protein [Source:UniProtKB/TrEMBL;Acc:G3MZA1]
neurexophilin and PC-esterase domain family, member 2 [Source:HGNC Symbol;Acc:HGNC:26331]
Bos taurus vesicle transport through interaction with t-SNAREs homolog 1B (yeast) (VTI1B), mRNA. [Source:RefSeq mRNA;Acc:NM_001079647]
Bos taurus transmembrane protein 251 (TMEM251), mRNA. [Source:RefSeq mRNA;Acc:NM_001077132]

chromosome 17 open reading frame 64 [Source:HGNC Symbol;Acc:HGNC:26990]
POU class 3 homeobox 4 [Source:HGNC Symbol;Acc:HGNC:9217]
Uncharacterized protein [Source:UniProtKB/TrEMBL;Acc:G5E6S6]
Bos taurus melanocortin 2 receptor (adrenocorticotropic hormone) (MC2R), mRNA. [Source:RefSeq mRNA;Acc:NM_174109]
Bos taurus protein phosphatase 1, regulatory (inhibitor) subunit 11 (PPP1R11), mRNA. [Source:RefSeq mRNA;Acc:NM_001100295]
TELO2 interacting protein 1 [Source:HGNC Symbol;Acc:HGNC:29029]
solute carrier family 45, member 4 [Source:HGNC Symbol;Acc:HGNC:29196]
Bos taurus roundabout homolog 4, magic roundabout (Drosophila) (ROBO4), mRNA. [Source:RefSeq mRNA;Acc:NM_001099191]
translocation associated membrane protein 1-like 1 [Source:HGNC Symbol;Acc:HGNC:28371]

RAB7A, member RAS oncogene family [Source:HGNC Symbol;Acc:HGNC:9788]

LOC529047 protein; Uncharacterized protein [Source:UniProtKB/TrEMBL;Acc:Q2T9Y2]
Bos taurus betaine--homocysteine S-methyltransferase (BHMT), mRNA. [Source:RefSeq mRNA;Acc:NM_001011679]
Bos taurus neurotensin (NTS), mRNA. [Source:RefSeq mRNA;Acc:NM_173945]
solute carrier family 25, member 46 [Source:HGNC Symbol;Acc:HGNC:25198]

Uncharacterized protein [Source:UniProtKB/TrEMBL;Acc:E1BK70]
Bos taurus sodium channel, voltage-gated, type II, beta (SCN2B), mRNA. [Source:RefSeq mRNA;Acc:NM_001192074]

Uncharacterized protein [Source:UniProtKB/TrEMBL;Acc:G3N3A8]
tubby like protein 3 [Source:HGNC Symbol;Acc:HGNC:12425]
fidgetin-like 1 [Source:HGNC Symbol;Acc:HGNC:13286]

Uncharacterized protein [Source:UniProtKB/TrEMBL;Acc:G3MZQ7]
CD93 molecule [Source:HGNC Symbol;Acc:HGNC:15855]

Bos taurus chromosome 16 open reading frame, human C1orf65 (C16H1orf65), mRNA. [Source:RefSeq mRNA;Acc:NM_001076193]
High mobility group protein B3 [Source:UniProtKB/TrEMBL;Acc:F1N1P2]
Bos taurus 2,4-dienoyl CoA reductase 2, peroxisomal (DECR2), mRNA. [Source:RefSeq mRNA;Acc:NM_001078122]
Uncharacterized protein [Source:UniProtKB/TrEMBL;Acc:G3N1L0]
Uncharacterized protein [Source:UniProtKB/TrEMBL;Acc:G3N0U6]
Bos taurus phosphatidylethanolamine binding protein 1 (PEBP1), mRNA. [Source:RefSeq mRNA;Acc:NM_001033623]
Bos taurus armadillo repeat containing, X-linked 1 (ARMCX1), mRNA. [Source:RefSeq mRNA;Acc:NM_001075167]
Bos taurus ST6 (alpha-N-acetyl-neuraminyl-2,3-beta-galactosyl-1, 3)-N-acetylgalactosaminide alpha-2,6-sialyltransferase 5 (ST6GALNAC5), mRNA. [Source:RefSe
Olfactory receptor [Source:UniProtKB/TrEMBL;Acc:G3MZA3]
Bos taurus potassium voltage-gated channel, shaker-related subfamily, beta member 2 (KCNAB2), transcript variant 2, mRNA. [Source:RefSeq mRNA;Acc:NM_0
Bos taurus ARP2 actin-related protein 2 homolog (yeast) (ACTR2), mRNA. [Source:RefSeq mRNA;Acc:NM_001102213]
Bos taurus suppressor of variegation 3-9 homolog 1 (Drosophila) (SUV39H1), mRNA. [Source:RefSeq mRNA;Acc:NM_001046264]
Bos taurus phosphatidylinositol glycan anchor biosynthesis, class C (PIGC), mRNA. [Source:RefSeq mRNA;Acc:NM_001034383]
Bos taurus keratin associated protein 12-2 (KRTAP12-2), mRNA. [Source:RefSeq mRNA;Acc:NM_001080351]
Uncharacterized protein [Source:UniProtKB/TrEMBL;Acc:F6QD35]
Bos taurus SRY (sex determining region Y)-box 2 (SOX2), mRNA. [Source:RefSeq mRNA;Acc:NM_001105463]
phosphatidylinositol-5-phosphate 4-kinase, type II, alpha [Source:HGNC Symbol;Acc:HGNC:8997]
Bos taurus CD79a molecule, immunoglobulin-associated alpha (CD79A), mRNA. [Source:RefSeq mRNA;Acc:NM_174266]

chromosome 19 open reading frame 26 [Source:HGNC Symbol;Acc:HGNC:28617]
olfactory receptor, family 8, subfamily S, member 1 [Source:HGNC Symbol;Acc:HGNC:19628]
REC8 meiotic recombination protein [Source:HGNC Symbol;Acc:HGNC:16879]
Bos taurus EF-hand calcium binding domain 1 (EFCAB1), mRNA. [Source:RefSeq mRNA;Acc:NM_001037585]

chromosome 7 open reading frame 25 [Source:HGNC Symbol;Acc:HGNC:21703]
Bos taurus major histocompatibility complex, class II, DM alpha (BOLA-DMA), mRNA. [Source:RefSeq mRNA;Acc:NM_001012674]

Bos taurus CD52 molecule (CD52), mRNA. [Source:RefSeq mRNA;Acc:NM_001198996]
Bos taurus neurexophilin 3 (NXPH3), mRNA. [Source:RefSeq mRNA;Acc:NM_001192824]
Bos taurus Cbp/p300-interacting transactivator, with Glu/Asp-rich carboxy-terminal domain, 2 (CITED2), mRNA. [Source:RefSeq mRNA;Acc:NM_001075819]
Uncharacterized protein [Source:UniProtKB/TrEMBL;Acc:G3N1R2]
Bos taurus 5-hydroxytryptamine (serotonin) receptor 1F (HTR1F), mRNA. [Source:RefSeq mRNA;Acc:NM_001206605]
Bos taurus tissue specific transplantation antigen P35B (TSTA3), mRNA. [Source:RefSeq mRNA;Acc:NM_001046139]
Bos taurus chemokine (C-C motif) ligand 11 (CCL11), mRNA. [Source:RefSeq mRNA;Acc:NM_205773]
Bos taurus MARVEL domain containing 1 (MARVELD1), mRNA. [Source:RefSeq mRNA;Acc:NM_001101262]
Uncharacterized protein [Source:UniProtKB/TrEMBL;Acc:F1MSG1]
mitochondrial elongation factor 1 [Source:HGNC Symbol;Acc:HGNC:25979]
lymphocyte antigen 6 complex, locus K [Source:HGNC Symbol;Acc:HGNC:24225]
Bos taurus claudin 2 (CLDN2), mRNA. [Source:RefSeq mRNA;Acc:NM_205781]
ZFP62 zinc finger protein [Source:HGNC Symbol;Acc:HGNC:23241]
chromosome 1 open reading frame 85 [Source:HGNC Symbol;Acc:HGNC:29436]
Uncharacterized protein [Source:UniProtKB/TrEMBL;Acc:G3X802]
Bos taurus ribosomal protein L35 (RPL35), mRNA. [Source:RefSeq mRNA;Acc:NM_001034495]
olfactory receptor, family 1, subfamily L, member 1 [Source:HGNC Symbol;Acc:HGNC:8213]
solute carrier family 16, member 12 [Source:HGNC Symbol;Acc:HGNC:23094]
Bos taurus fibroblast growth factor 20 (FGF20), mRNA. [Source:RefSeq mRNA;Acc:NM_001192165]
Bos taurus FEZ family zinc finger 2 (FEZF2), mRNA. [Source:RefSeq mRNA;Acc:NM_001038198]
Bos taurus S100 calcium binding protein B (S100B), mRNA. [Source:RefSeq mRNA;Acc:NM_001034555]
Bos taurus G protein-coupled receptor 18 (GPR18), mRNA. [Source:RefSeq mRNA;Acc:NM_001034689]
Bos taurus G protein-coupled receptor 37 (endothelin receptor type B-like) (GPR37), mRNA. [Source:RefSeq mRNA;Acc:NM_001075969]
Olfactory receptor [Source:UniProtKB/TrEMBL;Acc:F1MWJ6]
Uncharacterized protein [Source:UniProtKB/TrEMBL;Acc:E1BEW7]
Bos taurus Ras association (RalGDS/AF-6) domain family (N-terminal) member 9 (RASSF9), mRNA. [Source:RefSeq mRNA;Acc:NM_001193083]
Bos taurus p53 and DNA-damage regulated 1 (PDRG1), mRNA. [Source:RefSeq mRNA;Acc:NM_001078115]

Uncharacterized protein [Source:UniProtKB/TrEMBL;Acc:E1BJL6]
testis expressed 37 [Source:HGNC Symbol;Acc:HGNC:26341]
Bos taurus histone cluster 2, H2be (HIST2H2BE), mRNA. [Source:RefSeq mRNA;Acc:NM_001099384]

Ribosomal protein [Source:UniProtKB/TrEMBL;Acc:E1BKD0]
pseudouridylate synthase 3 [Source:HGNC Symbol;Acc:HGNC:25461]
Bos taurus angiotensin II receptor, type 1 (AGTR1), mRNA. [Source:RefSeq mRNA;Acc:NM_174233]
Olfactory receptor [Source:UniProtKB/TrEMBL;Acc:G3X837]
Bos taurus UPF0552 protein C15orf38 homolog (C21H15orf38), mRNA. [Source:RefSeq mRNA;Acc:NM_001076304]
arylsulfatase family, member H [Source:HGNC Symbol;Acc:HGNC:32488]
male-specific lethal 2 homolog (Drosophila) [Source:HGNC Symbol;Acc:HGNC:25544]
Bos taurus nitric oxide associated 1 (NOA1), mRNA. [Source:RefSeq mRNA;Acc:NM_001038188]
Olfactory receptor [Source:UniProtKB/TrEMBL;Acc:G3N0C8]
Uncharacterized protein [Source:UniProtKB/TrEMBL;Acc:F1MM74]
zinc finger protein 777 [Source:HGNC Symbol;Acc:HGNC:22213]
Bos taurus opioid receptor, mu 1 (OPRM1), mRNA. [Source:RefSeq mRNA;Acc:NM_174408]
UDP-Gal:betaGlcNAc beta 1,4- galactosyltransferase, polypeptide 5 [Source:HGNC Symbol;Acc:HGNC:928]
Uncharacterized protein [Source:UniProtKB/TrEMBL;Acc:G3N0F2]
atlastin GTPase 2 [Source:HGNC Symbol;Acc:HGNC:24047]
kelch-like family member 10 [Source:HGNC Symbol;Acc:HGNC:18829]
Bos taurus SERTA domain containing 3 (SERTAD3), mRNA. [Source:RefSeq mRNA;Acc:NM_001077008]
Bos taurus inositol polyphosphate-1-phosphatase (INPP1), mRNA. [Source:RefSeq mRNA;Acc:NM_174364]
Uncharacterized protein [Source:UniProtKB/TrEMBL;Acc:F1MU03]
Bos taurus potassium channel, subfamily K, member 9 (KCNK9), mRNA. [Source:RefSeq mRNA;Acc:NM_001206824]
Bos taurus ribosomal protein L4 (RPL4), mRNA. [Source:RefSeq mRNA;Acc:NM_001014894]
zinc finger and BTB domain containing 38 [Source:HGNC Symbol;Acc:HGNC:26636]
Bos taurus hypocretin (orexin) receptor 1 (HCRTR1), mRNA. [Source:RefSeq mRNA;Acc:NM_001048182]
family with sequence similarity 155, member B [Source:HGNC Symbol;Acc:HGNC:30701]
Bos taurus myocilin, trabecular meshwork inducible glucocorticoid response (MYOC), mRNA. [Source:RefSeq mRNA;Acc:NM_174118]

Bos taurus ATPase family, AAA domain containing 1 (ATAD1), mRNA. [Source:RefSeq mRNA;Acc:NM_001205581]
Bos taurus G protein-coupled receptor 115 (GPR115), mRNA. [Source:RefSeq mRNA;Acc:NM_001143875]
Olfactory receptor [Source:UniProtKB/TrEMBL;Acc:G3MXW1]
Chymotrypsinogen A Chymotrypsin A chain A Chymotrypsin A chain B Chymotrypsin A chain C [Source:UniProtKB/Swiss-Prot;Acc:P00766]
Bos taurus sorting nexin 8 (SNX8), mRNA. [Source:RefSeq mRNA;Acc:NM_001046533]
Bos taurus myeloid-associated differentiation marker-like (LOC508153), mRNA. [Source:RefSeq mRNA;Acc:NM_001100322]
Bos taurus necdin homolog (mouse) (NDN), mRNA. [Source:RefSeq mRNA;Acc:NM_001014982]
Bos taurus heparan sulfate (glucosamine) 3-O-sulfotransferase 1 (HS3ST1), mRNA. [Source:RefSeq mRNA;Acc:NM_001076122]
Uncharacterized protein [Source:UniProtKB/TrEMBL;Acc:F1N235]
Uncharacterized protein [Source:UniProtKB/TrEMBL;Acc:G3N388]
Olfactory receptor [Source:UniProtKB/TrEMBL;Acc:E1BH63]
Uncharacterized protein [Source:UniProtKB/TrEMBL;Acc:G3N200]
Bos taurus tRNA methyltransferase 11-2 homolog (S. cerevisiae) (TRMT112), mRNA. [Source:RefSeq mRNA;Acc:NM_001045981]
C-type lectin domain family 5, member A [Source:HGNC Symbol;Acc:HGNC:2054]
Olfactory receptor [Source:UniProtKB/TrEMBL;Acc:G3N036]
Uncharacterized protein [Source:UniProtKB/TrEMBL;Acc:M0QW45]
Bos taurus G protein-coupled receptor 84 (GPR84), mRNA. [Source:RefSeq mRNA;Acc:NM_001038568]
Bos taurus spermidine/spermine N1-acetyltransferase 1 (SAT1), mRNA. [Source:RefSeq mRNA;Acc:NM_001034333]
pleckstrin homology domain containing, family D (with coiled-coil domains) member 1 [Source:HGNC Symbol;Acc:HGNC:20148]
Uncharacterized protein [Source:UniProtKB/TrEMBL;Acc:F1MIZ6]
olfactory receptor, family 11, subfamily L, member 1 [Source:HGNC Symbol;Acc:HGNC:14998]
pyroglutamyl-peptidase I [Source:HGNC Symbol;Acc:HGNC:13568]
Bos taurus low density lipoprotein receptor-related protein 10 (LRP10), mRNA. [Source:RefSeq mRNA;Acc:NM_001100371]
Bos taurus family with sequence similarity 105, member A (FAM105A), mRNA. [Source:RefSeq mRNA;Acc:NM_001102171]
chromosome 10 open reading frame 71 [Source:HGNC Symbol;Acc:HGNC:26973]
ankyrin repeat domain 50 [Source:HGNC Symbol;Acc:HGNC:29223]
chromosome 15 open reading frame 59 [Source:HGNC Symbol;Acc:HGNC:33753]
Uncharacterized protein [Source:UniProtKB/TrEMBL;Acc:G3X6Q6]
Bos taurus nuclear receptor subfamily 1, group I, member 2 (NR1I2), mRNA. [Source:RefSeq mRNA;Acc:NM_001103226]
Olfactory receptor [Source:UniProtKB/TrEMBL;Acc:E1B8X0]

Bos taurus mediator complex subunit 20 (MED20), mRNA. [Source:RefSeq mRNA;Acc:NM_001103293]
Bos taurus UDP-GlcNAc:betaGal beta-1,3-N-acetylglucosaminyltransferase 2 (B3GNT2), mRNA. [Source:RefSeq mRNA;Acc:NM_001102497]
Bos taurus lectin, galactoside-binding, soluble, 2 (LGALS2), transcript variant 2, mRNA. [Source:RefSeq mRNA;Acc:NM_001075494]
Bos taurus receptor accessory protein 6 (REEP6), mRNA. [Source:RefSeq mRNA;Acc:NM_001038135]
protocadherin 12 [Source:HGNC Symbol;Acc:HGNC:8657]
solute carrier family 34 (type II sodium/phosphate contransporter), member 3 [Source:HGNC Symbol;Acc:HGNC:20305]
Bos taurus NADH dehydrogenase (ubiquinone) 1 alpha subcomplex, assembly factor 4 (NDUFAF4), mRNA. [Source:RefSeq mRNA;Acc:NM_001083516]
Bos taurus glutamate-rich 2 (ERICH2), mRNA. [Source:RefSeq mRNA;Acc:NM_001102286]

Olfactory receptor [Source:UniProtKB/TrEMBL;Acc:G3N2U4]
Uncharacterized protein [Source:UniProtKB/TrEMBL;Acc:G3N2R7]
Bos taurus prolactin-related protein VI (PRP6), mRNA. [Source:RefSeq mRNA;Acc:NM_205778]
Uncharacterized protein [Source:UniProtKB/TrEMBL;Acc:F1N1B2]
Bos taurus B-cell CLL/lymphoma 7B (BCL7B), mRNA. [Source:RefSeq mRNA;Acc:NM_001034775]

Bos taurus Rho family GTPase 1 (RND1), mRNA. [Source:RefSeq mRNA;Acc:NM_001046016]
dual oxidase maturation factor 2 [Source:HGNC Symbol;Acc:HGNC:32698]
Bos taurus poly(A) binding protein interacting protein 1 (PAIP1), mRNA. [Source:RefSeq mRNA;Acc:NM_001104971]
spermatogenesis associated 16 [Source:HGNC Symbol;Acc:HGNC:29935]
zinc finger, matrin-type 1 [Source:HGNC Symbol;Acc:HGNC:29377]
Bos taurus prostaglandin-endoperoxide synthase 1 (prostaglandin G/H synthase and cyclooxygenase) (PTGS1), mRNA. [Source:RefSeq mRNA;Acc:NM_0011053[illegible]
Bos taurus kelch repeat and BTB (POZ) domain containing 6 (KBTBD6), mRNA. [Source:RefSeq mRNA;Acc:NM_001205540]
olfactory receptor, family 4, subfamily Q, member 2 (gene/pseudogene) [Source:HGNC Symbol;Acc:HGNC:15359]
Olfactory receptor [Source:UniProtKB/TrEMBL;Acc:E1BB61]
Bos taurus ring finger protein 122 (RNF122), mRNA. [Source:RefSeq mRNA;Acc:NM_001101920]
Uncharacterized protein [Source:UniProtKB/TrEMBL;Acc:G3N0H5]
chromosome 4 open reading frame 19 [Source:HGNC Symbol;Acc:HGNC:25618]
immunoglobulin superfamily, member 3 [Source:HGNC Symbol;Acc:HGNC:5950]
Bos taurus YTH domain family, member 2 (YTHDF2), mRNA. [Source:RefSeq mRNA;Acc:NM_001076253]
Olfactory receptor [Source:UniProtKB/TrEMBL;Acc:G5E5H8]

Bos taurus solute carrier family 25 (mitochondrial carrier; phosphate carrier), member 3-like (LOC616319), mRNA. [Source:RefSeq mRNA;Acc:NM_001101254]
Bos taurus prune homolog (Drosophila) (PRUNE), mRNA. [Source:RefSeq mRNA;Acc:NM_001035352]
Bos taurus N-terminal EF-hand calcium binding protein 2 (NECAB2), mRNA. [Source:RefSeq mRNA;Acc:NM_001098977]
claudin 25 [Source:HGNC Symbol;Acc:HGNC:37218]
Bos taurus GTPase, IMAP family member 4 (GIMAP4), mRNA. [Source:RefSeq mRNA;Acc:NM_001046060]
Bos taurus thioredoxin 2 (TXN2), mRNA. [Source:RefSeq mRNA;Acc:NM_174208]
Bos taurus salt-inducible kinase 2 (SIK2), mRNA. [Source:RefSeq mRNA;Acc:NM_001192277]
Bos taurus thromboxane A2 receptor (TBXA2R), mRNA. [Source:RefSeq mRNA;Acc:NM_001167919]
Bos taurus Sp2 transcription factor (SP2), mRNA. [Source:RefSeq mRNA;Acc:NM_001015654]
Uncharacterized protein [Source:UniProtKB/TrEMBL;Acc:F1MNP8]
Uncharacterized protein [Source:UniProtKB/TrEMBL;Acc:G8JKX9]
Bos taurus family with sequence similarity 131, member B (FAM131B), mRNA. [Source:RefSeq mRNA;Acc:NM_001078072]
Bos taurus DDB1 and CUL4 associated factor 16 (DCAF16), mRNA. [Source:RefSeq mRNA;Acc:NM_001078147]
Bos taurus transmembrane protease, serine 2 (TMPRSS2), mRNA. [Source:RefSeq mRNA;Acc:NM_001081585]
Bos taurus glycoprotein A33 (transmembrane) (GPA33), mRNA. [Source:RefSeq mRNA;Acc:NM_001035330]
iroquois homeobox 2 [Source:HGNC Symbol;Acc:HGNC:14359]
Bos taurus regulator of chromosome condensation 2 (RCC2), mRNA. [Source:RefSeq mRNA;Acc:NM_001101911]
Bos taurus leiomodin 1 (smooth muscle) (LMOD1), mRNA. [Source:RefSeq mRNA;Acc:NM_001083715]
Bos taurus matrix metallopeptidase 2 (gelatinase A, 72kDa gelatinase, 72kDa type IV collagenase) (MMP2), mRNA. [Source:RefSeq mRNA;Acc:NM_174745]
GDNF-inducible zinc finger protein 1 [Source:HGNC Symbol;Acc:HGNC:15808]
glucosaminyl (N-acetyl) transferase 1, core 2 [Source:HGNC Symbol;Acc:HGNC:4203]
olfactory receptor, family 2, subfamily M, member 4 [Source:HGNC Symbol;Acc:HGNC:8270]
Bos taurus transgelin (TAGLN), mRNA. [Source:RefSeq mRNA;Acc:NM_001046149]
protein kinase C, delta [Source:HGNC Symbol;Acc:HGNC:9399]
Bos taurus glucose-fructose oxidoreductase domain containing 2 (GFOD2), mRNA. [Source:RefSeq mRNA;Acc:NM_001014863]
chromosome 20 open reading frame 173 [Source:HGNC Symbol;Acc:HGNC:16166]
Bos taurus NOP2/Sun domain family, member 5 (NSUN5), mRNA. [Source:RefSeq mRNA;Acc:NM_001102303]
Olfactory receptor [Source:UniProtKB/TrEMBL;Acc:G3N012]
UDP-N-acetylglucosamine pyrophosphorylase 1 like 1 [Source:HGNC Symbol;Acc:HGNC:28082]
FEZ family zinc finger 1 [Source:HGNC Symbol;Acc:HGNC:22788]

RAN guanine nucleotide release factor [Source:HGNC Symbol;Acc:HGNC:17679]
Bos taurus tripartite motif containing 10 (TRIM10), mRNA. [Source:RefSeq mRNA;Acc:NM_001015607]
Uncharacterized protein [Source:UniProtKB/TrEMBL;Acc:G3N0M5]
zinc finger and SCAN domain containing 23 [Source:HGNC Symbol;Acc:HGNC:21193]
Bos taurus glucuronic acid epimerase (GLCE), mRNA. [Source:RefSeq mRNA;Acc:NM_174070]
BTG family, member 2 [Source:HGNC Symbol;Acc:HGNC:1131]
Bos taurus ADP-ribosyltransferase 5-like (LOC407148), mRNA. [Source:RefSeq mRNA;Acc:NM_001104964]
Guanine nucleotide-binding protein G(I)/G(S)/G(O) subunit gamma-11 [Source:UniProtKB/Swiss-Prot;Acc:Q5E9F0]
Uncharacterized protein [Source:UniProtKB/TrEMBL;Acc:G3MWK3]
Bos taurus phosphatidylinositol glycan anchor biosynthesis, class P (PIGP), mRNA. [Source:RefSeq mRNA;Acc:NM_001077022]
Bos taurus CSRP2 binding protein (CSRP2BP), mRNA. [Source:RefSeq mRNA;Acc:NM_001206055]
Olfactory receptor [Source:UniProtKB/TrEMBL;Acc:E1BF80]
G protein-coupled receptor 176 [Source:HGNC Symbol;Acc:HGNC:32370]
Olfactory receptor [Source:UniProtKB/TrEMBL;Acc:F1N7S1]
Bos taurus potassium inwardly-rectifying channel, subfamily J, member 14 (KCNJ14), mRNA. [Source:RefSeq mRNA;Acc:NM_001206021]
transmembrane protein 64 [Source:HGNC Symbol;Acc:HGNC:25441]
Olfactory receptor [Source:UniProtKB/TrEMBL;Acc:G3MZ81]
kelch-like family member 42 [Source:HGNC Symbol;Acc:HGNC:29252]
Bos taurus chromosome 3 open reading frame, human C1orf182 (TSACC), mRNA. [Source:RefSeq mRNA;Acc:NM_001076989]

synaptotagmin XVII [Source:HGNC Symbol;Acc:HGNC:24119]
teashirt zinc finger homeobox 2 [Source:HGNC Symbol;Acc:HGNC:13010]
NACHT and WD repeat domain containing 2 [Source:HGNC Symbol;Acc:HGNC:29229]

WAS/WASL interacting protein family, member 2 [Source:HGNC Symbol;Acc:HGNC:30923]
Bos taurus ubiquitin-conjugating enzyme E2, J1, U (UBE2J1), mRNA. [Source:RefSeq mRNA;Acc:NM_001206186]
Bos taurus myeloid-associated differentiation marker-like (LOC512150), mRNA. [Source:RefSeq mRNA;Acc:NM_001104975]
recombination activating gene 1 [Source:HGNC Symbol;Acc:HGNC:9831]
RIB43A domain with coiled-coils 1 [Source:HGNC Symbol;Acc:HGNC:26537]

Bos taurus coiled-coil domain containing 51 (CCDC51), mRNA. [Source:RefSeq mRNA;Acc:NM_001015619]

Uncharacterized protein [Source:UniProtKB/TrEMBL;Acc:E1BJ53]
U-box domain containing 5 [Source:HGNC Symbol;Acc:HGNC:17777]
Bos taurus toll-like receptor adaptor molecule 2 (TICAM2), mRNA. [Source:RefSeq mRNA;Acc:NM_001046456]
Bos taurus RMI2, RecQ mediated genome instability 2, homolog (S. cerevisiae) (RMI2), mRNA. [Source:RefSeq mRNA;Acc:NM_001099720]

Uncharacterized protein [Source:UniProtKB/TrEMBL;Acc:G3MWL6]
Bos taurus family with sequence similarity 181, member B (FAM181B), mRNA. [Source:RefSeq mRNA;Acc:NM_001101223]
Olfactory receptor [Source:UniProtKB/TrEMBL;Acc:E1B9Z3]
Bos taurus FXYD domain containing ion transport regulator 6 (FXYD6), mRNA. [Source:RefSeq mRNA;Acc:NM_001034399]
Bos taurus chromosome 5 open reading frame, human C12orf68 (C5H12orf68), mRNA. [Source:RefSeq mRNA;Acc:NM_001078073]

Uncharacterized protein [Source:UniProtKB/TrEMBL;Acc:G3MYX6]
Bos taurus heat shock protein, alpha-crystallin-related, B6 (HSPB6), mRNA. [Source:RefSeq mRNA;Acc:NM_001076027]
potassium intermediate/small conductance calcium-activated channel, subfamily N, member 3 [Source:HGNC Symbol;Acc:HGNC:6292]
Bos taurus transient receptor potential cation channel, subfamily C, member 5 (TRPC5), mRNA. [Source:RefSeq mRNA;Acc:NM_001166574]
Bos taurus dendrin (DDN), mRNA. [Source:RefSeq mRNA;Acc:NM_001102301]
Bos taurus proteasome (prosome, macropain) subunit, beta type, 2 (PSMB2), mRNA. [Source:RefSeq mRNA;Acc:NM_001015615]
Bos taurus phosphomannomutase 2 (PMM2), mRNA. [Source:RefSeq mRNA;Acc:NM_001035095]
Uncharacterized protein [Source:UniProtKB/TrEMBL;Acc:G5E5S2]
ELAV like neuron-specific RNA binding protein 3 [Source:HGNC Symbol;Acc:HGNC:3314]
Bos taurus mago-nashi homolog B (Drosophila) (MAGOHB), mRNA. [Source:RefSeq mRNA;Acc:NM_001076252]
Bos taurus LFNG O-fucosylpeptide 3-beta-N-acetylglucosaminyltransferase (LFNG), mRNA. [Source:RefSeq mRNA;Acc:NM_001046222]
Bos taurus natriuretic peptide A (NPPA), mRNA. [Source:RefSeq mRNA;Acc:NM_174124]
Bos taurus zinc finger protein 419 (ZNF419), mRNA. [Source:RefSeq mRNA;Acc:NM_001101932]
Bos taurus guanine nucleotide binding protein (G protein), alpha 14 (GNA14), mRNA. [Source:RefSeq mRNA;Acc:NM_174323]
dystrophia myotonica, WD repeat containing [Source:HGNC Symbol;Acc:HGNC:2936]
Bos taurus zinc finger and BTB domain containing 8A (ZBTB8A), mRNA. [Source:RefSeq mRNA;Acc:NM_001076264]
Bos taurus GRB2-binding adaptor protein, transmembrane (GAPT), mRNA. [Source:RefSeq mRNA;Acc:NM_001099397]

Bos taurus THAP domain containing 10 (THAP10), mRNA. [Source:RefSeq mRNA;Acc:NM_001080741]
Bos taurus brain-derived neurotrophic factor (BDNF), mRNA. [Source:RefSeq mRNA;Acc:NM_001046607]
Bos taurus coiled-coil domain containing 102A (CCDC102A), mRNA. [Source:RefSeq mRNA;Acc:NM_001077915]
Bos taurus potassium voltage-gated channel, Shaw-related subfamily, member 1 (KCNC1), transcript variant A, mRNA. [Source:RefSeq mRNA;Acc:NM_0011132[illegible]
Bos taurus DEAD (Asp-Glu-Ala-Asp) box polypeptide 17 (DDX17), mRNA. [Source:RefSeq mRNA;Acc:NM_001101993]
Protein NKG7 [Source:UniProtKB/Swiss-Prot;Acc:Q2KJ11]
Olfactory receptor [Source:UniProtKB/TrEMBL;Acc:G5E6M9]
Bos taurus interleukin 12A (natural killer cell stimulatory factor 1, cytotoxic lymphocyte maturation factor 1, p35) (IL12A), mRNA. [Source:RefSeq mRNA;Acc:N[illegible]
Bos taurus A kinase (PRKA) anchor protein 2 (AKAP2), mRNA. [Source:RefSeq mRNA;Acc:NM_001193034]
Bos taurus olfactory receptor, family 4, subfamily X, member 1 (OR4X1), mRNA. [Source:RefSeq mRNA;Acc:NM_001075450]
Uncharacterized protein [Source:UniProtKB/TrEMBL;Acc:E1BIF8]
Bos taurus neutrophilic granule protein-like (NGP), mRNA. [Source:RefSeq mRNA;Acc:NM_001105468]
ataxin 1-like [Source:HGNC Symbol;Acc:HGNC:33279]
triggering receptor expressed on myeloid cells-like 1 [Source:HGNC Symbol;Acc:HGNC:20434]
Bos taurus TBC1 domain family, member 10B (TBC1D10B), mRNA. [Source:RefSeq mRNA;Acc:NM_001205771]
Olfactory receptor [Source:UniProtKB/TrEMBL;Acc:F1MN81]
Bos taurus tetraspanin 15 (TSPAN15), mRNA. [Source:RefSeq mRNA;Acc:NM_001075829]
G protein-coupled receptor 15 [Source:HGNC Symbol;Acc:HGNC:4469]
Bos taurus transmembrane protein 218 (TMEM218), mRNA. [Source:RefSeq mRNA;Acc:NM_001099159]
Bos taurus dual specificity phosphatase 6 (DUSP6), mRNA. [Source:RefSeq mRNA;Acc:NM_001046195]
Bos taurus family with sequence similarity 160, member A2 (FAM160A2), mRNA. [Source:RefSeq mRNA;Acc:NM_001102491]
Olfactory receptor [Source:UniProtKB/TrEMBL;Acc:F1MPY1]
Bos taurus interleukin 18 (interferon-gamma-inducing factor) (IL18), mRNA. [Source:RefSeq mRNA;Acc:NM_174091]
DnaJ (Hsp40) homolog, subfamily C, member 28 [Source:HGNC Symbol;Acc:HGNC:1297]
guanine nucleotide-binding protein G(t) subunit alpha-3 [Source:RefSeq peptide;Acc:NP_001103452]
Uncharacterized protein [Source:UniProtKB/TrEMBL;Acc:G5E5G7]
Bos taurus CGRP receptor component (CRCP), mRNA. [Source:RefSeq mRNA;Acc:NM_001077870]
bradykinin receptor B1 [Source:HGNC Symbol;Acc:HGNC:1029]
Bos taurus interferon, gamma-inducible protein 30 (IFI30), mRNA. [Source:RefSeq mRNA;Acc:NM_001101251]
dopamine receptor D2 [Source:HGNC Symbol;Acc:HGNC:3023]

Bos taurus transmembrane protein 81 (TMEM81), mRNA. [Source:RefSeq mRNA;Acc:NM_001075465]
filamin A, alpha [Source:HGNC Symbol;Acc:HGNC:3754]
Bos taurus LSM10, U7 small nuclear RNA associated (LSM10), mRNA. [Source:RefSeq mRNA;Acc:NM_001083781]
Bos taurus empty spiracles homeobox 2 (EMX2), mRNA. [Source:RefSeq mRNA;Acc:NM_001075845]
Uncharacterized protein [Source:UniProtKB/TrEMBL;Acc:E1BI37]
Bos taurus SMT3 suppressor of mif two 3 homolog 3 (S. cerevisiae) (SUMO3), mRNA. [Source:RefSeq mRNA;Acc:NM_001076449]
zinc finger CCCH-type containing 13 [Source:HGNC Symbol;Acc:HGNC:20368]
Olfactory receptor [Source:UniProtKB/TrEMBL;Acc:G3X792]
amine oxidase, copper containing 2 (retina-specific) [Source:HGNC Symbol;Acc:HGNC:549]
Olfactory receptor [Source:UniProtKB/TrEMBL;Acc:F1MZS2]
Olfactory receptor [Source:UniProtKB/TrEMBL;Acc:G3N2H0]

Bos taurus adrenergic, alpha-1D-, receptor (ADRA1D), mRNA. [Source:RefSeq mRNA;Acc:NM_001205684]
chromosome 11 open reading frame 24 [Source:HGNC Symbol;Acc:HGNC:1174]
doublesex and mab-3 related transcription factor 2 [Source:HGNC Symbol;Acc:HGNC:2935]
Bos taurus high mobility group nucleosomal binding domain 2 (HMGN2), mRNA. [Source:RefSeq mRNA;Acc:NM_001098945]
amyloid beta (A4) precursor-like protein 1 [Source:HGNC Symbol;Acc:HGNC:597]
Bos taurus acyl-CoA oxidase 3, pristanoyl (ACOX3), mRNA. [Source:RefSeq mRNA;Acc:NM_001103236]
Bos taurus toll-like receptor 5 (TLR5), mRNA. [Source:RefSeq mRNA;Acc:NM_001040501]
Bos taurus necdin-like 2 (NDNL2), mRNA. [Source:RefSeq mRNA;Acc:NM_001078080]
Bos taurus golgi phosphoprotein 3 (coat-protein) (GOLPH3), mRNA. [Source:RefSeq mRNA;Acc:NM_001075948]
egl nine homolog 2 [Source:RefSeq peptide;Acc:NP_001095663]
metaxin 1 [Source:HGNC Symbol;Acc:HGNC:7504]
Uncharacterized protein [Source:UniProtKB/TrEMBL;Acc:F1MNR0]
Fas ligand (TNF superfamily, member 6) [Source:HGNC Symbol;Acc:HGNC:11936]
Bos taurus Kruppel-like factor 4 (gut) (KLF4), mRNA. [Source:RefSeq mRNA;Acc:NM_001105385]
Bos taurus ring finger protein 146 (RNF146B), mRNA. [Source:RefSeq mRNA;Acc:NM_001076980]
Uncharacterized protein [Source:UniProtKB/TrEMBL;Acc:E1BDL8]
Uncharacterized protein [Source:UniProtKB/TrEMBL;Acc:F1MYD1]
GPRIN family member 3 [Source:HGNC Symbol;Acc:HGNC:27733]

Bos taurus lymphocyte transmembrane adaptor 1 (LAX1), mRNA. [Source:RefSeq mRNA;Acc:NM_001015674]
ubiquitin specific peptidase 30 [Source:HGNC Symbol;Acc:HGNC:20065]
Bos taurus adrenergic, beta-2-, receptor, surface (ADRB2), mRNA. [Source:RefSeq mRNA;Acc:NM_174231]
Bos taurus zinc finger protein 507 (ZNF507), mRNA. [Source:RefSeq mRNA;Acc:NM_001206198]
tektin-5 [Source:RefSeq peptide;Acc:NP_001039724]
Bos taurus CDC42 effector protein (Rho GTPase binding) 1 (CDC42EP1), mRNA. [Source:RefSeq mRNA;Acc:NM_001075495]
coiled-coil domain containing 87 [Source:HGNC Symbol;Acc:HGNC:25579]
YLP motif containing 1 [Source:HGNC Symbol;Acc:HGNC:17798]
Uncharacterized protein [Source:UniProtKB/TrEMBL;Acc:G3N1M8]
Bos taurus 5-hydroxytryptamine (serotonin) receptor 3C, ionotropic (HTR3C), mRNA. [Source:RefSeq mRNA;Acc:NM_001192508]
Bos taurus coenzyme Q2 homolog, prenyltransferase (yeast) (COQ2), mRNA. [Source:RefSeq mRNA;Acc:NM_001045893]
bromodomain containing 1 [Source:HGNC Symbol;Acc:HGNC:1102]
Bos taurus interleukin 1 receptor antagonist (IL1RN), mRNA. [Source:RefSeq mRNA;Acc:NM_174357]
Bos taurus ornithine decarboxylase antizyme 2 (OAZ2), mRNA. [Source:RefSeq mRNA;Acc:NM_001172576]
Olfactory receptor [Source:UniProtKB/TrEMBL;Acc:G3N209]

Bos taurus STE20-related kinase adaptor alpha (STRADA), mRNA. [Source:RefSeq mRNA;Acc:NM_001015603]
Uterine milk protein [Source:UniProtKB/Swiss-Prot;Acc:P46201]
Bos taurus zinc finger protein 238 (ZBTB18), mRNA. [Source:RefSeq mRNA;Acc:NM_001078016]
Bos taurus immunoglobulin superfamily, member 10 (IGSF10), mRNA. [Source:RefSeq mRNA;Acc:NM_001206785]
chromosome 1 open reading frame 68 [Source:HGNC Symbol;Acc:HGNC:29468]
Bos taurus peroxisome proliferator-activated receptor alpha (PPARA), mRNA. [Source:RefSeq mRNA;Acc:NM_001034036]
Olfactory receptor [Source:UniProtKB/TrEMBL;Acc:F1MBY4]
FK506 binding protein like [Source:HGNC Symbol;Acc:HGNC:13949]
Bos taurus gap junction protein, beta 2, 26kDa (GJB2), mRNA. [Source:RefSeq mRNA;Acc:NM_001083637]
testis expressed 28 [Source:HGNC Symbol;Acc:HGNC:2563]
Bos taurus chromosome 10 open reading frame, human C14orf93 (C10H14orf93), mRNA. [Source:RefSeq mRNA;Acc:NM_001038049]
cat eye syndrome chromosome region, candidate 1 [Source:HGNC Symbol;Acc:HGNC:1839]
leucine rich repeat transmembrane neuronal 4 [Source:HGNC Symbol;Acc:HGNC:19411]

APC membrane recruitment protein 1 [Source:HGNC Symbol;Acc:HGNC:26837]
Bos taurus free fatty acid receptor 2 (FFAR2), mRNA. [Source:RefSeq mRNA;Acc:NM_001163784]

Bos taurus solute carrier family 25, member 44 (SLC25A44), mRNA. [Source:RefSeq mRNA;Acc:NM_001077857]
Olfactory receptor [Source:UniProtKB/TrEMBL;Acc:F1MDI3]
Bos taurus keratin associated protein 1-1 (KRTAP1-1), mRNA. [Source:RefSeq mRNA;Acc:NM_001105369]
PDX1 C-terminal inhibiting factor 1 [Source:HGNC Symbol;Acc:HGNC:16200]
Bos taurus zinc finger, CCHC domain containing 12 (ZCCHC12), mRNA. [Source:RefSeq mRNA;Acc:NM_001076197]
Uncharacterized protein [Source:UniProtKB/TrEMBL;Acc:G3X7F7]
mitochondrial genome maintenance exonuclease 1 [Source:HGNC Symbol;Acc:HGNC:16205]

Bos taurus mitochondrial ribosomal protein S33 (MRPS33), mRNA. [Source:RefSeq mRNA;Acc:NM_001037605]
Bos taurus uncharacterized LOC516719 (MGC137708), transcript variant 1, mRNA. [Source:RefSeq mRNA;Acc:NM_001046227]
Bos taurus protein serine kinase H1 (PSKH1), mRNA. [Source:RefSeq mRNA;Acc:NM_001075413]
Bos taurus cholinergic receptor, nicotinic, beta 4 (CHRNB4), mRNA. [Source:RefSeq mRNA;Acc:NM_174517]
Bos taurus uncharacterized LOC788205 (LOC788205), mRNA. [Source:RefSeq mRNA;Acc:NM_001105644]
protocadherin gamma subfamily A, 5 [Source:HGNC Symbol;Acc:HGNC:8703]

TGF-beta activated kinase 1/MAP3K7 binding protein 3 [Source:HGNC Symbol;Acc:HGNC:30681]
Bos taurus zinc finger protein 502 (ZNF502), mRNA. [Source:RefSeq mRNA;Acc:NM_001103101]
adaptor-related protein complex 3, mu 2 subunit [Source:HGNC Symbol;Acc:HGNC:570]
Bos taurus high mobility group nucleosome binding domain 1 (HMGN1), mRNA. [Source:RefSeq mRNA;Acc:NM_001034772]
SH3-domain binding protein 4 [Source:HGNC Symbol;Acc:HGNC:10826]
Uncharacterized protein [Source:UniProtKB/TrEMBL;Acc:F1MR94]
SLIT and NTRK-like family, member 5 [Source:HGNC Symbol;Acc:HGNC:20295]

chordin-like 1 [Source:HGNC Symbol;Acc:HGNC:29861]
Bos taurus chemokine (C-C motif) ligand 25 (CCL25), mRNA. [Source:RefSeq mRNA;Acc:NM_001046569]

OTU deubiquitinase, ubiquitin aldehyde binding 2 [Source:HGNC Symbol;Acc:HGNC:20351]

Bos taurus retinol binding protein 3, interstitial (RBP3), mRNA. [Source:RefSeq mRNA;Acc:NM_174164]
Bos taurus chromosome 5 open reading frame, human C12orf32 (RHNO1), mRNA. [Source:RefSeq mRNA;Acc:NM_001075631]
family with sequence similarity 115, member C [Source:HGNC Symbol;Acc:HGNC:26878]
Bos taurus deltex homolog 2 (Drosophila) (DTX2), mRNA. [Source:RefSeq mRNA;Acc:NM_001017945]
Uncharacterized protein [Source:UniProtKB/TrEMBL;Acc:E1B8V7]
Bos taurus solute carrier family 7 (amino acid transporter light chain, y+L system), member 7 (SLC7A7), mRNA. [Source:RefSeq mRNA;Acc:NM_001075151]
Bos taurus solute carrier family 38, member 7 (SLC38A7), mRNA. [Source:RefSeq mRNA;Acc:NM_001100355]
Uncharacterized protein [Source:UniProtKB/TrEMBL;Acc:G3MYB7]
Bos taurus chromosome 16 open reading frame, human C1orf86 (FAAP20), mRNA. [Source:RefSeq mRNA;Acc:NM_001166564]
Bos taurus aurora kinase A (AURKA), mRNA. [Source:RefSeq mRNA;Acc:NM_001038028]
Bos taurus armadillo repeat containing, X-linked 6 (ARMCX6), mRNA. [Source:RefSeq mRNA;Acc:NM_001078125]
UTP3, small subunit (SSU) processome component, homolog (S. cerevisiae) [Source:HGNC Symbol;Acc:HGNC:24477]
Olfactory receptor [Source:UniProtKB/TrEMBL;Acc:G3X896]
Bos taurus cadherin 15, type 1, M-cadherin (myotubule) (CDH15), mRNA. [Source:RefSeq mRNA;Acc:NM_001102066]
forkhead box P3 [Source:HGNC Symbol;Acc:HGNC:6106]
interleukin 17F [Source:HGNC Symbol;Acc:HGNC:16404]

Bos taurus ankyrin repeat domain 13A (ANKRD13A), mRNA. [Source:RefSeq mRNA;Acc:NM_001205709]
Olfactory receptor [Source:UniProtKB/TrEMBL;Acc:F1MGW3]
Bos taurus limb bud and heart development homolog (mouse) (LBH), mRNA. [Source:RefSeq mRNA;Acc:NM_001099152]
protocadherin beta 6 [Source:HGNC Symbol;Acc:HGNC:8691]
peptidyl arginine deiminase, type III [Source:HGNC Symbol;Acc:HGNC:18337]
Bos taurus ankyrin repeat domain 61 (ANKRD61), mRNA. [Source:RefSeq mRNA;Acc:NM_001077072]
solute carrier family 25, member 53 [Source:HGNC Symbol;Acc:HGNC:31894]
oncoprotein-induced transcript 3 protein precursor [Source:RefSeq peptide;Acc:NP_001039529]
Uncharacterized protein [Source:UniProtKB/TrEMBL;Acc:F1MPW8]
Olfactory receptor [Source:UniProtKB/TrEMBL;Acc:G3MWN2]
Olfactory receptor [Source:UniProtKB/TrEMBL;Acc:E1BMZ6]
Uncharacterized protein [Source:UniProtKB/TrEMBL;Acc:G3N0C9]
Uncharacterized protein [Source:UniProtKB/TrEMBL;Acc:F1MY03]

Bos taurus family with sequence similarity 96, member A (FAM96A), mRNA. [Source:RefSeq mRNA;Acc:NM_001035110]
chromosome 14 open reading frame 79 [Source:HGNC Symbol;Acc:HGNC:20126]
Bos taurus melanoma antigen family B, 16 (MAGEB16), mRNA. [Source:RefSeq mRNA;Acc:NM_001105399]

Olfactory receptor [Source:UniProtKB/TrEMBL;Acc:F1MBC8]
Bos taurus C-C chemokine receptor type 1-like (LOC529196), mRNA. [Source:RefSeq mRNA;Acc:NM_001075921]
keratin 20 [Source:HGNC Symbol;Acc:HGNC:20412]
KAL1 protein; Uncharacterized protein [Source:UniProtKB/TrEMBL;Acc:A2VE88]
Bos taurus spermatogenic leucine zipper 1 (SPZ1), mRNA. [Source:RefSeq mRNA;Acc:NM_001040540]
Bos taurus transcription factor binding to IGHM enhancer 3 (TFE3), mRNA. [Source:RefSeq mRNA;Acc:NM_001075811]
Uncharacterized protein [Source:UniProtKB/TrEMBL;Acc:G3MXD7]
Bos taurus chromosome 3 open reading frame, human C1orf189 (C3H1orf189), mRNA. [Source:RefSeq mRNA;Acc:NM_001038026]
Bos taurus gastrin-releasing peptide receptor (GRPR), mRNA. [Source:RefSeq mRNA;Acc:NM_001192739]
acyl-CoA dehydrogenase, C-2 to C-3 short chain [Source:HGNC Symbol;Acc:HGNC:90]
Uncharacterized protein [Source:UniProtKB/TrEMBL;Acc:F1MX22]
Olfactory receptor [Source:UniProtKB/TrEMBL;Acc:F1MJ91]

Bos taurus type 1 protein phosphatase inhibitor-like (LOC520057), mRNA. [Source:RefSeq mRNA;Acc:NM_001079599]
Bos taurus extracellular matrix protein 2, female organ and adipocyte specific (ECM2), mRNA. [Source:RefSeq mRNA;Acc:NM_001034597]
Uncharacterized protein [Source:UniProtKB/TrEMBL;Acc:G3X6H8]
fibronectin leucine rich transmembrane protein 1 [Source:HGNC Symbol;Acc:HGNC:3760]
Olfactory receptor [Source:UniProtKB/TrEMBL;Acc:E1BN36]
Bos taurus exostoses (multiple)-like 3 (EXTL3), mRNA. [Source:RefSeq mRNA;Acc:NM_001082473]
Bos taurus UDP-N-acetyl-alpha-D-galactosamine:polypeptide N-acetylgalactosaminyltransferase 13 (GalNAc-T13) (GALNT13), mRNA. [Source:RefSeq mRNA;Acc
Bos taurus snail homolog 2 (Drosophila) (SNAI2), mRNA. [Source:RefSeq mRNA;Acc:NM_001034538]

amyloid beta (A4) precursor protein-binding, family A, member 1 [Source:HGNC Symbol;Acc:HGNC:578]
Bos taurus NFKB activating protein-like (NKAPL), mRNA. [Source:RefSeq mRNA;Acc:NM_001102498]

olfactory receptor, family 2, subfamily AE, member 1 [Source:HGNC Symbol;Acc:HGNC:15087]

Bos taurus zinc finger and BTB domain containing 43 (ZBTB43), mRNA. [Source:RefSeq mRNA;Acc:NM_001024503]
Olfactory receptor [Source:UniProtKB/TrEMBL;Acc:E1B807]
E3 ubiquitin-protein ligase [Source:UniProtKB/TrEMBL;Acc:G3X7V0]
SERTA domain containing 4 [Source:HGNC Symbol;Acc:HGNC:25236]
KH domain containing, RNA binding, signal transduction associated 2 [Source:HGNC Symbol;Acc:HGNC:18114]
Bos taurus REST corepressor 2 (RCOR2), mRNA. [Source:RefSeq mRNA;Acc:NM_001206075]
Olfactory receptor [Source:UniProtKB/TrEMBL;Acc:E1BDK4]
Bos taurus BPI fold containing family A, member 3 (BPIFA3), mRNA. [Source:RefSeq mRNA;Acc:NM_001040535]
Bos taurus CDP-diacylglycerol--inositol 3-phosphatidyltransferase (CDIPT), mRNA. [Source:RefSeq mRNA;Acc:NM_001034482]
Bos taurus heat shock 70kDa protein 1-like (HSPA1L), mRNA. [Source:RefSeq mRNA;Acc:NM_001167895]
Bos taurus par-6 partitioning defective 6 homolog beta (C. elegans) (PARD6B), mRNA. [Source:RefSeq mRNA;Acc:NM_001098104]
Bos taurus family with sequence similarity 72, member A (FAM72A), mRNA. [Source:RefSeq mRNA;Acc:NM_001101263]
Bos taurus chromosome 8 open reading frame, human C9orf85 (C8H9orf85), mRNA. [Source:RefSeq mRNA;Acc:NM_001101235]
zinc finger and SCAN domain containing 9 [Source:HGNC Symbol;Acc:HGNC:12984]
homeobox D3 [Source:HGNC Symbol;Acc:HGNC:5137]
Bos taurus glutamate receptor, ionotropic, N-methyl D-aspartate-associated protein 1 (glutamate binding) (GRINA), mRNA. [Source:RefSeq mRNA;Acc:NM_001[illegible]
Bos taurus serine peptidase inhibitor-like, with Kunitz and WAP domains 1 (eppin) (SPINLW1), mRNA. [Source:RefSeq mRNA;Acc:NM_001077062]
Bos taurus xylulokinase homolog (H. influenzae) (XYLB), mRNA. [Source:RefSeq mRNA;Acc:NM_001034483]
major facilitator superfamily domain containing 9 [Source:HGNC Symbol;Acc:HGNC:28158]
Bos taurus nuclear receptor interacting protein 3 (NRIP3), mRNA. [Source:RefSeq mRNA;Acc:NM_001102218]
Uncharacterized protein [Source:UniProtKB/TrEMBL;Acc:E1B924]
Bos taurus angiotensin II receptor-associated protein (AGTRAP), mRNA. [Source:RefSeq mRNA;Acc:NM_001075363]
Uncharacterized protein [Source:UniProtKB/TrEMBL;Acc:G3MZQ9]
Uncharacterized protein [Source:UniProtKB/TrEMBL;Acc:E1BB66]
Bos taurus lipase, hormone-sensitive (LIPE), mRNA. [Source:RefSeq mRNA;Acc:NM_001080220]
Bos taurus IGF-like family receptor 1 (IGFLR1), mRNA. [Source:RefSeq mRNA;Acc:NM_001099167]
Olfactory receptor [Source:UniProtKB/TrEMBL;Acc:E1BJ39]
Bos taurus G protein-coupled receptor kinase 1 (GRK1), mRNA. [Source:RefSeq mRNA;Acc:NM_174173]

Olfactory receptor [Source:UniProtKB/TrEMBL;Acc:G3N2R4]

Bos taurus DIRAS family, GTP-binding RAS-like 3 (DIRAS3), mRNA. [Source:RefSeq mRNA;Acc:NM_001034215]
Uncharacterized protein [Source:UniProtKB/TrEMBL;Acc:F1MJD3]
Bos taurus 2,3-bisphosphoglycerate mutase (BPGM), mRNA. [Source:RefSeq mRNA;Acc:NM_001035402]
Bos taurus keratin associated protein 11-1 (KRTAP11-1), mRNA. [Source:RefSeq mRNA;Acc:NM_001080740]
pleckstrin and Sec7 domain containing 4 [Source:HGNC Symbol;Acc:HGNC:19096]
Olfactory receptor [Source:UniProtKB/TrEMBL;Acc:F1MS80]
Bos taurus papillary renal cell carcinoma (translocation-associated) (PRCC), mRNA. [Source:RefSeq mRNA;Acc:NM_001045961]
midline 1 [Source:HGNC Symbol;Acc:HGNC:7095]
olfactory receptor, family 2, subfamily C, member 1 [Source:HGNC Symbol;Acc:HGNC:8242]
zinc finger protein 157 [Source:HGNC Symbol;Acc:HGNC:12942]
Prothymosin alpha [Source:UniProtKB/TrEMBL;Acc:Q2LDY9]
Bos taurus apolipoprotein B mRNA editing enzyme, catalytic polypeptide-like 2 (APOBEC2), mRNA. [Source:RefSeq mRNA;Acc:NM_001034355]
Uncharacterized protein [Source:UniProtKB/TrEMBL;Acc:F1MIK9]
Bos taurus PILR alpha associated neural protein (PIANP), mRNA. [Source:RefSeq mRNA;Acc:NM_001083767]
Bos taurus neuron-derived neurotrophic factor (NDNF), mRNA. [Source:RefSeq mRNA;Acc:NM_001205858]

potassium channel, subfamily V, member 2 [Source:HGNC Symbol;Acc:HGNC:19698]
transmembrane protein 200A [Source:HGNC Symbol;Acc:HGNC:21075]
zinc finger and BTB domain containing 24 [Source:HGNC Symbol;Acc:HGNC:21143]
Bos taurus hydrolethalus syndrome 1 (HYLS1), mRNA. [Source:RefSeq mRNA;Acc:NM_001046439]
regulatory solute carrier protein, family 1, member 1 [Source:HGNC Symbol;Acc:HGNC:10458]
Uncharacterized protein [Source:UniProtKB/TrEMBL;Acc:E1BA77]
Bos taurus claudin 4 (CLDN4), mRNA. [Source:RefSeq mRNA;Acc:NM_001014391]

Bos taurus retinal G protein coupled receptor (RGR), mRNA. [Source:RefSeq mRNA;Acc:NM_175775]
Uncharacterized protein [Source:UniProtKB/TrEMBL;Acc:F1MEU8]
Olfactory receptor [Source:UniProtKB/TrEMBL;Acc:F1MEY7]

Bos taurus eukaryotic translation initiation factor 2B, subunit 1 alpha, 26kDa (EIF2B1), mRNA. [Source:RefSeq mRNA;Acc:NM_001075743]
Uncharacterized protein [Source:UniProtKB/TrEMBL;Acc:E1BCH9]
Bos taurus mitogen-activated protein kinase kinase kinase 8 (MAP3K8), mRNA. [Source:RefSeq mRNA;Acc:NM_001099071]
chromosome 17 open reading frame 67 [Source:HGNC Symbol;Acc:HGNC:27900]
Bos taurus phosducin-like (PDCL), mRNA. [Source:RefSeq mRNA;Acc:NM_174759]
Bos taurus gametocyte specific factor 1-like (GTSF1L), mRNA. [Source:RefSeq mRNA;Acc:NM_001079601]
Bos taurus high mobility group 20A (HMG20A), mRNA. [Source:RefSeq mRNA;Acc:NM_001075637]
myelin basic protein [Source:HGNC Symbol;Acc:HGNC:6925]
Bos taurus corneodesmosin (CDSN), mRNA. [Source:RefSeq mRNA;Acc:NM_001077950]
Uncharacterized protein [Source:UniProtKB/TrEMBL;Acc:F1MUL6]
Bos taurus cyclin B1 interacting protein 1, E3 ubiquitin protein ligase (CCNB1IP1), mRNA. [Source:RefSeq mRNA;Acc:NM_001046565]
Bos taurus family with sequence similarity 108, member C1 (ABHD17C), mRNA. [Source:RefSeq mRNA;Acc:NM_001099014]
Bos taurus lysozyme G-like 2 (LYG2), mRNA. [Source:RefSeq mRNA;Acc:NM_001099076]
Uncharacterized protein [Source:UniProtKB/TrEMBL;Acc:F1MXG3]
interferon-induced protein with tetratricopeptide repeats 2 [Source:HGNC Symbol;Acc:HGNC:5409]
Bos taurus major histocompatibility complex, class II, DM beta (BOLA-DMB), mRNA. [Source:RefSeq mRNA;Acc:NM_001040481]
Bos taurus protein phosphatase 1, regulatory subunit 37 (PPP1R37), mRNA. [Source:RefSeq mRNA;Acc:NM_001105379]
Bos taurus ubiquitin domain containing 2 (UBTD2), mRNA. [Source:RefSeq mRNA;Acc:NM_001015679]
Olfactory receptor [Source:UniProtKB/TrEMBL;Acc:G3N325]
Uncharacterized protein [Source:UniProtKB/TrEMBL;Acc:F1MX97]
Uncharacterized protein [Source:UniProtKB/TrEMBL;Acc:G5E6C7]
gap junction protein, alpha 10, 62kDa [Source:HGNC Symbol;Acc:HGNC:16995]
olfactory receptor, family 51, subfamily F, member 2 [Source:HGNC Symbol;Acc:HGNC:15197]
transmembrane protein 132E [Source:HGNC Symbol;Acc:HGNC:26991]
NFAT activating protein with ITAM motif 1 [Source:HGNC Symbol;Acc:HGNC:29872]
Bos taurus heme binding protein 1 (HEBP1), mRNA. [Source:RefSeq mRNA;Acc:NM_001075985]
Bos taurus VMA21 vacuolar H+-ATPase homolog (S. cerevisiae) (VMA21), mRNA. [Source:RefSeq mRNA;Acc:NM_001081616]
leucine rich repeat containing 69 [Source:HGNC Symbol;Acc:HGNC:34303]
Bos taurus hyaluronan and proteoglycan link protein 3 (HAPLN3), mRNA. [Source:RefSeq mRNA;Acc:NM_001098981]

Bos taurus testis derived transcript (3 LIM domains) (TES), mRNA. [Source:RefSeq mRNA;Acc:NM_001046390]
ATPase, H+ transporting, lysosomal 56/58kDa, V1 subunit B1 [Source:HGNC Symbol;Acc:HGNC:853]
Uncharacterized protein [Source:UniProtKB/TrEMBL;Acc:F1MHA7]
Bos taurus oxidoreductase-like domain containing 1 (OXLD1), mRNA. [Source:RefSeq mRNA;Acc:NM_001105635]
Olfactory receptor [Source:UniProtKB/TrEMBL;Acc:F1N5J4]
Bos taurus potassium channel tetramerisation domain containing 7 (KCTD7), mRNA. [Source:RefSeq mRNA;Acc:NM_001102545]
interleukin 15 receptor, alpha [Source:HGNC Symbol;Acc:HGNC:5978]
Bos taurus solute carrier family 16, member 6 (monocarboxylic acid transporter 7) (SLC16A6), mRNA. [Source:RefSeq mRNA;Acc:NM_001192672]
ectonucleotide pyrophosphatase/phosphodiesterase 7 [Source:HGNC Symbol;Acc:HGNC:23764]
olfactory receptor, family 13, subfamily A, member 1 [Source:HGNC Symbol;Acc:HGNC:14772]
Bos taurus sulfatase modifying factor 1 (SUMF1), mRNA. [Source:RefSeq mRNA;Acc:NM_001076076]
Bos taurus DnaJ (Hsp40) homolog, subfamily B, member 1 (DNAJB1), mRNA. [Source:RefSeq mRNA;Acc:NM_001033763]
Olfactory receptor [Source:UniProtKB/TrEMBL;Acc:G3N0L8]
Bos taurus transketolase-like 2 (TKTL2), mRNA. [Source:RefSeq mRNA;Acc:NM_001046025]
Uncharacterized protein [Source:UniProtKB/TrEMBL;Acc:F1N7Q3]
potassium voltage-gated channel, shaker-related subfamily, member 10 [Source:HGNC Symbol;Acc:HGNC:6219]
Uncharacterized protein [Source:UniProtKB/TrEMBL;Acc:F1MGT7]
ubiquitin specific peptidase 46 [Source:HGNC Symbol;Acc:HGNC:20075]
sema domain, transmembrane domain (TM), and cytoplasmic domain, (semaphorin) 6B [Source:HGNC Symbol;Acc:HGNC:10739]
Uncharacterized protein [Source:UniProtKB/TrEMBL;Acc:F1MKG8]
muscleblind-like splicing regulator 3 [Source:HGNC Symbol;Acc:HGNC:20564]
Bos taurus growth differentiation factor 9 (GDF9), mRNA. [Source:RefSeq mRNA;Acc:NM_174681]
Bos taurus proteasome maturation protein (POMP), mRNA. [Source:RefSeq mRNA;Acc:NM_001034376]
zinc finger protein 140 [Source:HGNC Symbol;Acc:HGNC:12925]
calcyphosine-like [Source:HGNC Symbol;Acc:HGNC:28375]
Bos taurus methyltransferase like 15 (METTL15), mRNA. [Source:RefSeq mRNA;Acc:NM_001079782]
leucine rich repeat containing 66 [Source:HGNC Symbol;Acc:HGNC:34299]
Bos taurus steroid receptor RNA activator 1 (SRA1), mRNA. [Source:RefSeq mRNA;Acc:NM_001079642]
pre-mRNA processing factor 38B [Source:HGNC Symbol;Acc:HGNC:25512]
aquaporin 5 [Source:HGNC Symbol;Acc:HGNC:638]

Bos taurus carnitine palmitoyltransferase 1B (muscle) (CPT1B), mRNA. [Source:RefSeq mRNA;Acc:NM_001034349]
synaptotagmin III [Source:HGNC Symbol;Acc:HGNC:11511]

protein KIAA0415 homolog [Source:RefSeq peptide;Acc:NP_001192287]
APC membrane recruitment protein 3 [Source:HGNC Symbol;Acc:HGNC:26771]
Olfactory receptor [Source:UniProtKB/TrEMBL;Acc:M0QW35]

Olfactory receptor [Source:UniProtKB/TrEMBL;Acc:F1MJH5]
thyroid hormone receptor interactor 11 [Source:HGNC Symbol;Acc:HGNC:12305]
SLIT and NTRK-like family, member 2 [Source:HGNC Symbol;Acc:HGNC:13449]
Bos taurus keratin 6A (KRT6A), mRNA. [Source:RefSeq mRNA;Acc:NM_001083510]
Uncharacterized protein [Source:UniProtKB/TrEMBL;Acc:G3X6J6]
Uncharacterized protein [Source:UniProtKB/TrEMBL;Acc:E1BMK2]
Bos taurus sialophorin (SPN), mRNA. [Source:RefSeq mRNA;Acc:NM_001103102]
Bos taurus BARX homeobox 2 (BARX2), mRNA. [Source:RefSeq mRNA;Acc:NM_001101266]
Bos taurus PDZ domain containing 11 (PDZD11), mRNA. [Source:RefSeq mRNA;Acc:NM_001037603]
Bos taurus chromosome 8 open reading frame, human C9orf23 (RPP25L), mRNA. [Source:RefSeq mRNA;Acc:NM_001046111]
Uncharacterized protein [Source:UniProtKB/TrEMBL;Acc:F1MI80]
chromosome 10 open reading frame 82 [Source:HGNC Symbol;Acc:HGNC:28500]
Bos taurus apolipoprotein L, 3 (APOL3), mRNA. [Source:RefSeq mRNA;Acc:NM_001100297]
Bos taurus major facilitator superfamily domain containing 10 (MFSD10), mRNA. [Source:RefSeq mRNA;Acc:NM_001075304]
Bos taurus epilepsy, progressive myoclonus type 2A, Lafora disease (laforin) (EPM2A), mRNA. [Source:RefSeq mRNA;Acc:NM_001099709]
Bos taurus synaptogyrin 3 (SYNGR3), mRNA. [Source:RefSeq mRNA;Acc:NM_001082464]
Uncharacterized protein [Source:UniProtKB/TrEMBL;Acc:G5E5T4]
plexin A1 [Source:HGNC Symbol;Acc:HGNC:9099]
Bos taurus solute carrier family 7 (orphan transporter), member 14 (SLC7A14), mRNA. [Source:RefSeq mRNA;Acc:NM_001077992]
Uncharacterized protein [Source:UniProtKB/TrEMBL;Acc:F1N7K4]

Bos taurus taste receptor, type 2, member 1 (TAS2R1), mRNA. [Source:RefSeq mRNA;Acc:NM_001046636]
Bos taurus nuclear receptor subfamily 1, group D, member 1 (NR1D1), mRNA. [Source:RefSeq mRNA;Acc:NM_001078100]

Bos taurus phosphoserine phosphatase (PSPH), mRNA. [Source:RefSeq mRNA;Acc:NM_001046355]
Bos taurus DEAD (Asp-Glu-Ala-Asp) box polypeptide 28 (DDX28), mRNA. [Source:RefSeq mRNA;Acc:NM_001105422]

Bos taurus sprouty homolog 1, antagonist of FGF signaling (Drosophila) (SPRY1), mRNA. [Source:RefSeq mRNA;Acc:NM_001099366]

Bos taurus eukaryotic translation elongation factor 1 alpha 2 (EEF1A2), mRNA. [Source:RefSeq mRNA;Acc:NM_001037464]
B-cell CLL/lymphoma 6, member B [Source:HGNC Symbol;Acc:HGNC:1002]
tet methylcytosine dioxygenase 3 [Source:HGNC Symbol;Acc:HGNC:28313]
Bos taurus aarF domain containing kinase 3 (ADCK3), mRNA. [Source:RefSeq mRNA;Acc:NM_001046419]
Bos taurus DNA-damage-inducible transcript 3 (DDIT3), mRNA. [Source:RefSeq mRNA;Acc:NM_001078163]
Bos taurus complexin 2 (CPLX2), mRNA. [Source:RefSeq mRNA;Acc:NM_174283]
Bos taurus fatty acid binding protein 7, brain (FABP7), mRNA. [Source:RefSeq mRNA;Acc:NM_001078162]
Bos taurus testis-specific kinase 1 (TESK1), mRNA. [Source:RefSeq mRNA;Acc:NM_001172227]
platelet derived growth factor C [Source:HGNC Symbol;Acc:HGNC:8801]
mastermind-like 1 (Drosophila) [Source:HGNC Symbol;Acc:HGNC:13632]
ring finger protein 139 [Source:HGNC Symbol;Acc:HGNC:17023]
Bos taurus cytochrome b, ascorbate dependent 3 (CYBASC3), mRNA. [Source:RefSeq mRNA;Acc:NM_001099149]
Bos taurus kelch-like 18 (Drosophila) (KLHL18), mRNA. [Source:RefSeq mRNA;Acc:NM_001102043]

Bos taurus SRY (sex determining region Y)-box 5 (SOX5), mRNA. [Source:RefSeq mRNA;Acc:NM_001083471]

Uncharacterized protein [Source:UniProtKB/TrEMBL;Acc:G3MY57]
Bos taurus placenta-specific 9 (PLAC9), mRNA. [Source:RefSeq mRNA;Acc:NM_001038521]
Uncharacterized protein [Source:UniProtKB/TrEMBL;Acc:G3MYR7]
Olfactory receptor [Source:UniProtKB/TrEMBL;Acc:F1MVW8]
Bos taurus TRM1 tRNA methyltransferase 1 homolog (S. cerevisiae) (TRMT1), mRNA. [Source:RefSeq mRNA;Acc:NM_001206374]
interferon, epsilon [Source:HGNC Symbol;Acc:HGNC:18163]
Uncharacterized protein [Source:UniProtKB/TrEMBL;Acc:G3N1I1]
Bos taurus hippocalcin like 4 (HPCAL4), mRNA. [Source:RefSeq mRNA;Acc:NM_001099382]

Bos taurus olfactory receptor, family 10, subfamily H, member 1 (OR10H1), mRNA. [Source:RefSeq mRNA;Acc:NM_001077876]
Bos taurus cholinergic receptor, nicotinic, alpha 7 (CHRNA7), mRNA. [Source:RefSeq mRNA;Acc:NM_174515]
Bos taurus transmembrane protein 129 (TMEM129), mRNA. [Source:RefSeq mRNA;Acc:NM_001076864]
Olfactory receptor [Source:UniProtKB/TrEMBL;Acc:G3MXS4]
olfactory receptor, family 6, subfamily P, member 1 [Source:HGNC Symbol;Acc:HGNC:15036]
aminopeptidase B [Source:RefSeq peptide;Acc:NP_001091032]
Uncharacterized protein [Source:UniProtKB/TrEMBL;Acc:F1MTG6]
Bos taurus SprT-like N-terminal domain (SPRTN), mRNA. [Source:RefSeq mRNA;Acc:NM_001098082]
Bos taurus cholesterol 25-hydroxylase (CH25H), mRNA. [Source:RefSeq mRNA;Acc:NM_001075243]
ring finger protein 26 [Source:HGNC Symbol;Acc:HGNC:14646]
Bos taurus serum/glucocorticoid regulated kinase 1 (SGK1), mRNA. [Source:RefSeq mRNA;Acc:NM_001102033]
theg spermatid protein [Source:HGNC Symbol;Acc:HGNC:13706]
Uncharacterized protein [Source:UniProtKB/TrEMBL;Acc:E1BA89]
cadherin 7, type 2 [Source:HGNC Symbol;Acc:HGNC:1766]
Bos taurus calcitonin (CT), mRNA. [Source:RefSeq mRNA;Acc:NM_001143737]
Bos taurus hairy/enhancer-of-split related with YRPW motif-like (HEYL), mRNA. [Source:RefSeq mRNA;Acc:NM_001024565]
Bos taurus transmembrane protein 41A (TMEM41A), mRNA. [Source:RefSeq mRNA;Acc:NM_001075200]
Bos taurus gap junction protein, gamma 1, 45kDa (GJC1), mRNA. [Source:RefSeq mRNA;Acc:NM_001046076]
Bos taurus chloride intracellular channel 1 (CLIC1), mRNA. [Source:RefSeq mRNA;Acc:NM_001015608]
Olfactory receptor [Source:UniProtKB/TrEMBL;Acc:F1MUG0]
Uncharacterized protein [Source:UniProtKB/TrEMBL;Acc:G3N299]
Bos taurus isthmin 1 homolog (zebrafish) (ISM1), mRNA. [Source:RefSeq mRNA;Acc:NM_001163935]
proline rich 14-like [Source:HGNC Symbol;Acc:HGNC:28738]
Bos taurus ELOVL fatty acid elongase 1 (ELOVL1), mRNA. [Source:RefSeq mRNA;Acc:NM_001034703]
Bos taurus bestrophin 1 (BEST1), mRNA. [Source:RefSeq mRNA;Acc:NM_001080245]
Bos taurus chloride channel, nucleotide-sensitive, 1A (CLNS1A), mRNA. [Source:RefSeq mRNA;Acc:NM_001076300]
Bos taurus phosphate cytidylyltransferase 2, ethanolamine (PCYT2), mRNA. [Source:RefSeq mRNA;Acc:NM_001035089]
proline rich Gla (G-carboxyglutamic acid) 4 (transmembrane) [Source:HGNC Symbol;Acc:HGNC:30799]
Olfactory receptor [Source:UniProtKB/TrEMBL;Acc:G3N0N3]
Bos taurus uncharacterized LOC511175 (MGC157237), mRNA. [Source:RefSeq mRNA;Acc:NM_001083667]

Uncharacterized protein [Source:UniProtKB/TrEMBL;Acc:F1N0N9]
Bos taurus CNDP dipeptidase 2 (metallopeptidase M20 family) (CNDP2), mRNA. [Source:RefSeq mRNA;Acc:NM_001035108]
Bos taurus non-metastatic cells 7, protein expressed in (nucleoside-diphosphate kinase) (NME7), mRNA. [Source:RefSeq mRNA;Acc:NM_001015656]
protocadherin alpha 3 [Source:HGNC Symbol;Acc:HGNC:8669]
Uncharacterized protein [Source:UniProtKB/TrEMBL;Acc:E1BPF9]
Uncharacterized protein [Source:UniProtKB/TrEMBL;Acc:G5E6D5]
Bos taurus asialoglycoprotein receptor 1 (ASGR1), mRNA. [Source:RefSeq mRNA;Acc:NM_001037590]
protein C (inactivator of coagulation factors Va and VIIIa) [Source:HGNC Symbol;Acc:HGNC:9451]
Bos taurus chromosome 28 open reading frame, human C10orf58 (FAM213A), mRNA. [Source:RefSeq mRNA;Acc:NM_001034599]
Bos taurus leucine zipper, putative tumor suppressor 1 (LZTS1), mRNA. [Source:RefSeq mRNA;Acc:NM_001192287]
olfactory receptor, family 6, subfamily C, member 76 [Source:HGNC Symbol;Acc:HGNC:31305]
Bos taurus zinc finger, AN1-type domain 3 (ZFAND3), mRNA. [Source:RefSeq mRNA;Acc:NM_001102145]
Bos taurus NOP2/Sun domain family, member 4 (NSUN4), mRNA. [Source:RefSeq mRNA;Acc:NM_001191381]
gap junction protein, alpha 5, 40kDa [Source:HGNC Symbol;Acc:HGNC:4279]
Bos taurus myeloid cell leukemia sequence 1 (BCL2-related) (MCL1), mRNA. [Source:RefSeq mRNA;Acc:NM_001099206]
Bos taurus protein phosphatase 1, regulatory subunit 8 (PPP1R8), mRNA. [Source:RefSeq mRNA;Acc:NM_174582]
Bos taurus sulfatase modifying factor 2 (SUMF2), mRNA. [Source:RefSeq mRNA;Acc:NM_001014881]
SPATA31 subfamily D, member 1 [Source:HGNC Symbol;Acc:HGNC:37283]
nucleoporin 50kDa [Source:HGNC Symbol;Acc:HGNC:8065]
Bos taurus sequestosome 1 (SQSTM1), mRNA. [Source:RefSeq mRNA;Acc:NM_176641]
Olfactory receptor [Source:UniProtKB/TrEMBL;Acc:F1MT23]
synapse differentiation inducing 1-like [Source:HGNC Symbol;Acc:HGNC:32388]
Bos taurus transmembrane protein 184C (TMEM184C), mRNA. [Source:RefSeq mRNA;Acc:NM_001075180]
Bos taurus chibby homolog 1 (Drosophila) (CBY1), mRNA. [Source:RefSeq mRNA;Acc:NM_174739]

transmembrane protein 132C [Source:HGNC Symbol;Acc:HGNC:25436]
family with sequence similarity 83, member H [Source:HGNC Symbol;Acc:HGNC:24797]
Uncharacterized protein [Source:UniProtKB/TrEMBL;Acc:G3N0T8]
leucine rich repeat and fibronectin type III domain containing 2 [Source:HGNC Symbol;Acc:HGNC:21226]
chromosome 10 open reading frame 12 [Source:HGNC Symbol;Acc:HGNC:23420]

Bos taurus ribonuclease, RNase A family, 1 (pancreatic) (RNASE1), mRNA. [Source:RefSeq mRNA;Acc:NM_001014386]
stromal interaction molecule 1 [Source:HGNC Symbol;Acc:HGNC:11386]
pyrimidinergic receptor P2Y, G-protein coupled, 6 [Source:HGNC Symbol;Acc:HGNC:8543]
deltex 1, E3 ubiquitin ligase [Source:HGNC Symbol;Acc:HGNC:3060]
Bos taurus leiomodin 3 (fetal) (LMOD3), mRNA. [Source:RefSeq mRNA;Acc:NM_001100332]
Uncharacterized protein [Source:UniProtKB/TrEMBL;Acc:G3N232]
Bos taurus zinc finger, CCHC domain containing 3 (ZCCHC3), mRNA. [Source:RefSeq mRNA;Acc:NM_001080234]
Olfactory receptor [Source:UniProtKB/TrEMBL;Acc:G3X8B5]
Uncharacterized protein [Source:UniProtKB/TrEMBL;Acc:G5E581]
Bos taurus zinc finger protein 367 (ZNF367), mRNA. [Source:RefSeq mRNA;Acc:NM_001206033]
Olfactory receptor [Source:UniProtKB/TrEMBL;Acc:E1BBT1]
signal peptide peptidase like 2C [Source:HGNC Symbol;Acc:HGNC:28902]
Uncharacterized protein [Source:UniProtKB/TrEMBL;Acc:F1MUP8]
threonine synthase-like 1 (S. cerevisiae) [Source:HGNC Symbol;Acc:HGNC:26160]
Bos taurus msh homeobox 1 (MSX1), mRNA. [Source:RefSeq mRNA;Acc:NM_174798]
Bos taurus tripartite motif containing 32 (TRIM32), mRNA. [Source:RefSeq mRNA;Acc:NM_001075824]
HOXA9 protein; Uncharacterized protein [Source:UniProtKB/TrEMBL;Acc:A7Z086]
KIAA1024 [Source:HGNC Symbol;Acc:HGNC:29172]
Microsomal glutathione S-transferase 3 [Source:UniProtKB/Swiss-Prot;Acc:Q3T100]
SLIT and NTRK-like family, member 4 [Source:HGNC Symbol;Acc:HGNC:23502]
Bos taurus retinol dehydrogenase 13 (all-trans/9-cis) (RDH13), mRNA. [Source:RefSeq mRNA;Acc:NM_001075345]
zinc finger protein 79 [Source:HGNC Symbol;Acc:HGNC:13153]
potassium voltage-gated channel, Shaw-related subfamily, member 4 [Source:HGNC Symbol;Acc:HGNC:6236]
Bos taurus myosin regulatory light chain interacting protein (MYLIP), mRNA. [Source:RefSeq mRNA;Acc:NM_001102253]
Bos taurus protocadherin 1 (PCDH1), mRNA. [Source:RefSeq mRNA;Acc:NM_001083655]
olfactory receptor, family 5, subfamily D, member 14 [Source:HGNC Symbol;Acc:HGNC:15281]
Bos taurus tubulin, alpha 4a (TUBA4A), mRNA. [Source:RefSeq mRNA;Acc:NM_001078158]
immunoresponsive 1 homolog (mouse) [Source:HGNC Symbol;Acc:HGNC:33904]
Bos taurus deoxyribonuclease I (DNASE1), mRNA. [Source:RefSeq mRNA;Acc:NM_174534]
Bos taurus adrenergic, alpha-2B-, receptor (ADRA2B), mRNA. [Source:RefSeq mRNA;Acc:NM_001206628]

Uncharacterized protein [Source:UniProtKB/TrEMBL;Acc:G3N3N5]
lysophosphatidic acid receptor 2 [Source:HGNC Symbol;Acc:HGNC:3168]
Uncharacterized protein [Source:UniProtKB/TrEMBL;Acc:G3X7L2]

Bos taurus fucosyltransferase 10 (alpha (1,3) fucosyltransferase) (FUT10), mRNA. [Source:RefSeq mRNA;Acc:NM_182987]
Bos taurus eukaryotic translation initiation factor 4E family member 2 (EIF4E2), transcript variant 1, mRNA. [Source:RefSeq mRNA;Acc:NM_001075795]
Bos taurus lactate dehydrogenase A (LDHA), mRNA. [Source:RefSeq mRNA;Acc:NM_174099]
Bos taurus forkhead box A3 (FOXA3), mRNA. [Source:RefSeq mRNA;Acc:NM_001033119]
Bos taurus prostate androgen-regulated mucin-like protein 1 (PARM1), mRNA. [Source:RefSeq mRNA;Acc:NM_001075771]
Bos taurus tumor suppressor candidate 5 (TUSC5), mRNA. [Source:RefSeq mRNA;Acc:NM_001083707]
Bos taurus mannose-6-phosphate receptor (cation dependent) (M6PR), mRNA. [Source:RefSeq mRNA;Acc:NM_175779]
Bos taurus uncharacterized LOC100125913 (LOC100125913), mRNA. [Source:RefSeq mRNA;Acc:NM_001105484]
Bos taurus nuclear factor of kappa light polypeptide gene enhancer in B-cells inhibitor, epsilon (NFKBIE), mRNA. [Source:RefSeq mRNA;Acc:NM_001130746]
Bos taurus aprataxin (APTX), mRNA. [Source:RefSeq mRNA;Acc:NM_182654]
Bos taurus odd-skipped related 2 (Drosophila) (OSR2), mRNA. [Source:RefSeq mRNA;Acc:NM_001034328]
Olfactory receptor [Source:UniProtKB/TrEMBL;Acc:E1BLN3]
Olfactory receptor [Source:UniProtKB/TrEMBL;Acc:F1MSP0]
fibroblast growth factor 7 [Source:HGNC Symbol;Acc:HGNC:3685]
Bos taurus succinate dehydrogenase complex, subunit B, iron sulfur (Ip) (SDHB), mRNA. [Source:RefSeq mRNA;Acc:NM_001040483]
Uncharacterized protein [Source:UniProtKB/TrEMBL;Acc:E1BIA6]
zinc finger protein 365 [Source:HGNC Symbol;Acc:HGNC:18194]
family with sequence similarity 131, member A [Source:HGNC Symbol;Acc:HGNC:28308]
Bos taurus clarin 3 (CLRN3), mRNA. [Source:RefSeq mRNA;Acc:NM_001105504]
Bos taurus loss of heterozygosity, 12, chromosomal region 1 (LOH12CR1), mRNA. [Source:RefSeq mRNA;Acc:NM_001076355]
olfactory receptor, family 9, subfamily I, member 1 [Source:HGNC Symbol;Acc:HGNC:14718]
Bos taurus melanophilin (MLPH), mRNA. [Source:RefSeq mRNA;Acc:NM_001081597]
phosphatidylinositol glycan anchor biosynthesis, class O [Source:HGNC Symbol;Acc:HGNC:23215]
zinc finger protein 677 [Source:HGNC Symbol;Acc:HGNC:28730]
Bos taurus retinol dehydrogenase 8 (all-trans) (RDH8), mRNA. [Source:RefSeq mRNA;Acc:NM_174167]
Bos taurus mitochondrial ribosomal protein L44 (MRPL44), mRNA. [Source:RefSeq mRNA;Acc:NM_001046321]

Bos taurus otopetrin 3 (OTOP3), mRNA. [Source:RefSeq mRNA;Acc:NM_001256572]
TAP binding protein-like [Source:HGNC Symbol;Acc:HGNC:30683]
Bos taurus IQ motif and Sec7 domain 1 (IQSEC1), mRNA. [Source:RefSeq mRNA;Acc:NM_001206814]
Bos taurus ring finger protein 145 (RNF145), mRNA. [Source:RefSeq mRNA;Acc:NM_001102168]
Uncharacterized protein [Source:UniProtKB/TrEMBL;Acc:E1BD90]
Bos taurus cell adhesion molecule 1 (CADM1), mRNA. [Source:RefSeq mRNA;Acc:NM_001038558]
Uncharacterized protein [Source:UniProtKB/TrEMBL;Acc:G3MYL4]
Bos taurus family with sequence similarity 167, member A (FAM167A), mRNA. [Source:RefSeq mRNA;Acc:NM_001076494]
Bos taurus family with sequence similarity 192, member A (FAM192A), mRNA. [Source:RefSeq mRNA;Acc:NM_001014944]
Bos taurus dickkopf homolog 2 (Xenopus laevis) (DKK2), mRNA. [Source:RefSeq mRNA;Acc:NM_001082615]
Uncharacterized protein [Source:UniProtKB/TrEMBL;Acc:G3N1I7]
sprouty homolog 3 (Drosophila) [Source:HGNC Symbol;Acc:HGNC:11271]
Olfactory receptor [Source:UniProtKB/TrEMBL;Acc:F1N6I0]
Bos taurus ovo-like 2 (Drosophila) (OVOL2), mRNA. [Source:RefSeq mRNA;Acc:NM_001206007]
Bos taurus nephroblastoma overexpressed gene (NOV), mRNA. [Source:RefSeq mRNA;Acc:NM_001102382]
Olfactory receptor [Source:UniProtKB/TrEMBL;Acc:F1MRJ0]
Membrane cofactor protein [Source:UniProtKB/TrEMBL;Acc:F1MLR1]
Uncharacterized protein [Source:UniProtKB/TrEMBL;Acc:G5E6N0]
Bos taurus adenylate cyclase 9 (ADCY9), mRNA. [Source:RefSeq mRNA;Acc:NM_001205917]
Olfactory receptor [Source:UniProtKB/TrEMBL;Acc:G5E6F4]
Bos taurus trafficking protein particle complex 4 (TRAPPC4), mRNA. [Source:RefSeq mRNA;Acc:NM_001191204]
Bos taurus glutaredoxin (thioltransferase) (GLRX), mRNA. [Source:RefSeq mRNA;Acc:NM_001037604]
Uncharacterized protein [Source:UniProtKB/TrEMBL;Acc:G3MX76]
Bos taurus prosaposin-like 1 (PSAPL1), mRNA. [Source:RefSeq mRNA;Acc:NM_001103267]
chromosome 17 open reading frame 47 [Source:HGNC Symbol;Acc:HGNC:26844]
Bos taurus complement component 3a receptor 1 (C3AR1), mRNA. [Source:RefSeq mRNA;Acc:NM_001083752]
Bos taurus Kruppel-like factor 11 (KLF11), mRNA. [Source:RefSeq mRNA;Acc:NM_001190301]
Bos taurus poly (ADP-ribose) polymerase family, member 16 (PARP16), mRNA. [Source:RefSeq mRNA;Acc:NM_001083379]
Bos taurus zinc finger protein 263 (ZNF263), mRNA. [Source:RefSeq mRNA;Acc:NM_001205513]
opioid growth factor receptor [Source:HGNC Symbol;Acc:HGNC:15768]

Bos taurus leucine rich repeat containing 3B (LRRC3B), mRNA. [Source:RefSeq mRNA;Acc:NM_001099395]

Bos taurus zinc finger and BTB domain containing 9 (ZBTB9), mRNA. [Source:RefSeq mRNA;Acc:NM_001191462]
Bos taurus UDP-GlcNAc:betaGal beta-1,3-N-acetylglucosaminyltransferase 3 (B3GNT3), mRNA. [Source:RefSeq mRNA;Acc:NM_001098473]
Uncharacterized protein [Source:UniProtKB/TrEMBL;Acc:F1N263]
homeobox A1 [Source:HGNC Symbol;Acc:HGNC:5099]
pecanex-like 4 (Drosophila) [Source:HGNC Symbol;Acc:HGNC:20349]
Bos taurus sterile alpha motif domain containing 4A (SAMD4A), mRNA. [Source:RefSeq mRNA;Acc:NM_001206853]
HEAT repeat containing 4 [Source:HGNC Symbol;Acc:HGNC:16761]
Taste receptor type 2 [Source:UniProtKB/TrEMBL;Acc:E1BAN6]
Olfactory receptor [Source:UniProtKB/TrEMBL;Acc:G3N2M3]
Bos taurus solute carrier family 27 (fatty acid transporter), member 6 (SLC27A6), mRNA. [Source:RefSeq mRNA;Acc:NM_001101169]
Olfactory receptor [Source:UniProtKB/TrEMBL;Acc:F1MHB2]
Bos taurus ephrin-A3 (EFNA3), mRNA. [Source:RefSeq mRNA;Acc:NM_001105429]
Bos taurus ST8 alpha-N-acetyl-neuraminide alpha-2,8-sialyltransferase 1 (ST8SIA1), mRNA. [Source:RefSeq mRNA;Acc:NM_001032299]
ataxin 7 [Source:HGNC Symbol;Acc:HGNC:10560]
Bos taurus TSC22 domain family, member 1 (TSC22D1), mRNA. [Source:RefSeq mRNA;Acc:NM_001034377]
CoA synthase [Source:HGNC Symbol;Acc:HGNC:29932]
Bos taurus secreted frizzled-related protein 5 (SFRP5), mRNA. [Source:RefSeq mRNA;Acc:NM_174461]
mastermind-like 3 (Drosophila) [Source:HGNC Symbol;Acc:HGNC:16272]
PDZ domain containing 7; Uncharacterized protein [Source:UniProtKB/TrEMBL;Acc:Q0IIL8]
Olfactory receptor [Source:UniProtKB/TrEMBL;Acc:F1MI00]
Bos taurus sperm equatorial segment protein 1 (SPESP1), mRNA. [Source:RefSeq mRNA;Acc:NM_001079773]
Uncharacterized protein [Source:UniProtKB/TrEMBL;Acc:F1MV78]
olfactory receptor, family 5, subfamily A, member 1 [Source:HGNC Symbol;Acc:HGNC:8319]
Uncharacterized protein [Source:UniProtKB/TrEMBL;Acc:F1N4X1]
Uncharacterized protein [Source:UniProtKB/TrEMBL;Acc:G3N2K0]

distal-less homeobox 6 [Source:HGNC Symbol;Acc:HGNC:2919]
Bos taurus 3-oxoacyl-ACP synthase, mitochondrial (OXSM), mRNA. [Source:RefSeq mRNA;Acc:NM_001075624]

Bos taurus phosphoprotein enriched in astrocytes 15 (PEA15), mRNA. [Source:RefSeq mRNA;Acc:NM_001075456]
Bos taurus HRAS-like suppressor (HRASLS), mRNA. [Source:RefSeq mRNA;Acc:NM_001076225]
Uncharacterized protein [Source:UniProtKB/TrEMBL;Acc:G3N267]
immunoglobulin heavy constant mu [Source:HGNC Symbol;Acc:HGNC:5541]
Bos taurus Rho GTPase activating protein 35 (ARHGAP35), mRNA. [Source:RefSeq mRNA;Acc:NM_001192398]
Bos taurus serine/arginine-rich splicing factor 2 (SRSF2), mRNA. [Source:RefSeq mRNA;Acc:NM_001034318]
olfactory receptor, family 5, subfamily J, member 2 [Source:HGNC Symbol;Acc:HGNC:19612]
Uncharacterized protein [Source:UniProtKB/TrEMBL;Acc:G3X7N1]
Bos taurus hydroxysteroid (11-beta) dehydrogenase 2 (HSD11B2), mRNA. [Source:RefSeq mRNA;Acc:NM_174642]
Uncharacterized protein [Source:UniProtKB/TrEMBL;Acc:G3MXY8]
immunoglobulin superfamily, member 11 [Source:HGNC Symbol;Acc:HGNC:16669]
syntaxin binding protein 2 [Source:HGNC Symbol;Acc:HGNC:11445]
Bos taurus DnaJ (Hsp40) homolog, subfamily C, member 30 (DNAJC30), mRNA. [Source:RefSeq mRNA;Acc:NM_001080917]
LOC520778 protein; Uncharacterized protein [Source:UniProtKB/TrEMBL;Acc:Q32LK0]
zinc finger, SWIM-type containing 5 [Source:HGNC Symbol;Acc:HGNC:29299]
RIC3 acetylcholine receptor chaperone [Source:HGNC Symbol;Acc:HGNC:30338]

heterogeneous nuclear ribonucleoprotein H3 (2H9) [Source:HGNC Symbol;Acc:HGNC:5043]
Bos taurus cancer/testis antigen family 147, member B1 (CT47B1), mRNA. [Source:RefSeq mRNA;Acc:NM_001099202]
Bos taurus trace amine associated receptor 2 (TAAR2), mRNA. [Source:RefSeq mRNA;Acc:NM_001191222]

Uncharacterized protein [Source:UniProtKB/TrEMBL;Acc:E1BGP6]
G protein-coupled receptor 119 [Source:HGNC Symbol;Acc:HGNC:19060]
Bos taurus rhophilin associated tail protein 1 (ROPN1), mRNA. [Source:RefSeq mRNA;Acc:NM_001075900]
protocadherin alpha 11 [Source:HGNC Symbol;Acc:HGNC:8665]
Bos taurus WD repeat domain 38 (WDR38), mRNA. [Source:RefSeq mRNA;Acc:NM_001163946]
Bos taurus tyrosylprotein sulfotransferase 1 (TPST1), mRNA. [Source:RefSeq mRNA;Acc:NM_001077912]
Bos taurus ADP-ribosylation factor related protein 1 (ARFRP1), mRNA. [Source:RefSeq mRNA;Acc:NM_001037607]
Bos taurus chromosome 25 open reading frame, human C16orf88 (KNOP1), mRNA. [Source:RefSeq mRNA;Acc:NM_001166606]
Uncharacterized protein [Source:UniProtKB/TrEMBL;Acc:G3MYS5]

Bos taurus ferritin, heavy polypeptide 1 (FTH1), mRNA. [Source:RefSeq mRNA;Acc:NM_174062]
Olfactory receptor [Source:UniProtKB/TrEMBL;Acc:E1BFI7]
Bos taurus family with sequence similarity 102, member A (FAM102A), mRNA. [Source:RefSeq mRNA;Acc:NM_001193094]
DEAD (Asp-Glu-Ala-Asp) box polypeptide 49 [Source:HGNC Symbol;Acc:HGNC:18684]
potassium inwardly-rectifying channel, subfamily J, member 1 [Source:HGNC Symbol;Acc:HGNC:6255]

Bos taurus chromosome 27 open reading frame, human C8orf4 (C27H8orf4), mRNA. [Source:RefSeq mRNA;Acc:NM_001035490]
family with sequence similarity 187, member B [Source:HGNC Symbol;Acc:HGNC:26366]
Bos taurus keratin 15 (KRT15), mRNA. [Source:RefSeq mRNA;Acc:NM_001130747]
Bos taurus heat shock protein 90kDa alpha (cytosolic), class B member 1 (HSP90AB1), mRNA. [Source:RefSeq mRNA;Acc:NM_001079637]

Uncharacterized protein [Source:UniProtKB/TrEMBL;Acc:G3MZJ5]
brevican [Source:HGNC Symbol;Acc:HGNC:23059]
Bos taurus ubiquitin-like with PHD and ring finger domains 1 (UHRF1), mRNA. [Source:RefSeq mRNA;Acc:NM_001103098]
Olfactory receptor [Source:UniProtKB/TrEMBL;Acc:F1MGN5]
Bos taurus CDGSH iron sulfur domain 1 (CISD1), mRNA. [Source:RefSeq mRNA;Acc:NM_001034057]

Bos taurus peptidylprolyl isomerase H (cyclophilin H) (PPIH), mRNA. [Source:RefSeq mRNA;Acc:NM_001075618]
Bos taurus UDP-N-acetyl-alpha-D-galactosamine:polypeptide N-acetylgalactosaminyltransferase 5 (GalNAc-T5) (GALNT5), mRNA. [Source:RefSeq mRNA;Acc:NM
PDZ domain containing ring finger 4 [Source:HGNC Symbol;Acc:HGNC:30552]
Bos taurus platelet-activating factor receptor (PTAFR), mRNA. [Source:RefSeq mRNA;Acc:NM_001040538]
Uncharacterized protein [Source:UniProtKB/TrEMBL;Acc:G3N307]
NADH dehydrogenase subunit 5 [Source:RefSeq peptide;Acc:YP_209215]
Bos taurus homeobox A3 (HOXA3), mRNA. [Source:RefSeq mRNA;Acc:NM_001076825]
Olfactory receptor [Source:UniProtKB/TrEMBL;Acc:E1B9J0]
Olfactory receptor [Source:UniProtKB/TrEMBL;Acc:G3N3Q1]
prolylcarboxypeptidase (angiotensinase C) [Source:HGNC Symbol;Acc:HGNC:9344]
protocadherin beta 16 [Source:HGNC Symbol;Acc:HGNC:14546]

Bos taurus RNA binding motif protein 48 (RBM48), mRNA. [Source:RefSeq mRNA;Acc:NM_001101854]

Bos taurus proenkephalin (PENK), mRNA. [Source:RefSeq mRNA;Acc:NM_174141]
Bos taurus tribbles homolog 1 (Drosophila) (TRIB1), mRNA. [Source:RefSeq mRNA;Acc:NM_001101105]
Olfactory receptor [Source:UniProtKB/TrEMBL;Acc:F1MGH4]
Bos taurus pyridoxal (pyridoxine, vitamin B6) phosphatase (PDXP), mRNA. [Source:RefSeq mRNA;Acc:NM_001035035]
gasdermin B [Source:HGNC Symbol;Acc:HGNC:23690]
Bos taurus neuromedin B receptor (NMBR), mRNA. [Source:RefSeq mRNA;Acc:NM_001205710]
Bos taurus emopamil binding protein (sterol isomerase) (EBP), mRNA. [Source:RefSeq mRNA;Acc:NM_001034500]
Bos taurus zinc finger protein 862 (ZNF862), mRNA. [Source:RefSeq mRNA;Acc:NM_001191280]
Olfactory receptor [Source:UniProtKB/TrEMBL;Acc:E1BAE5]
Bos taurus splicing factor 3B, 14 kDa subunit (SF3B14), mRNA. [Source:RefSeq mRNA;Acc:NM_001046017]
Bos taurus cannabinoid receptor interacting protein 1 (CNRIP1), mRNA. [Source:RefSeq mRNA;Acc:NM_001076183]

Bos taurus ectonucleoside triphosphate diphosphohydrolase 2 (ENTPD2), mRNA. [Source:RefSeq mRNA;Acc:NM_001105648]
Uncharacterized protein [Source:UniProtKB/TrEMBL;Acc:G3N020]
Uncharacterized protein [Source:UniProtKB/TrEMBL;Acc:E1BA28]
Bos taurus protein HP-25 homolog 1 (LOC511240), mRNA. [Source:RefSeq mRNA;Acc:NM_001046080]
caudal type homeobox 4 [Source:HGNC Symbol;Acc:HGNC:1808]
membrane magnesium transporter 1 [Source:HGNC Symbol;Acc:HGNC:28100]
mixed lineage kinase domain-like [Source:HGNC Symbol;Acc:HGNC:26617]
Bos taurus Myb/SANT-like DNA-binding domain containing 3 (MSANTD3), mRNA. [Source:RefSeq mRNA;Acc:NM_001076019]
Bos taurus dopamine receptor D5 (DRD5), mRNA. [Source:RefSeq mRNA;Acc:NM_001206629]
Uncharacterized protein [Source:UniProtKB/TrEMBL;Acc:F1N186]
AT rich interactive domain 5B (MRF1-like) [Source:HGNC Symbol;Acc:HGNC:17362]
Bos taurus Bardet-Biedl syndrome 12 (BBS12), mRNA. [Source:RefSeq mRNA;Acc:NM_001100375]
Bos taurus Ts translation elongation factor, mitochondrial (TSFM), mRNA. [Source:RefSeq mRNA;Acc:NM_174204]
Uncharacterized protein [Source:UniProtKB/TrEMBL;Acc:G3N2U8]
Bos taurus ADP-ribosylarginine hydrolase (ADPRH), mRNA. [Source:RefSeq mRNA;Acc:NM_001038554]
Bos taurus uncharacterized LOC507581 (MGC142702), transcript variant 1, mRNA. [Source:RefSeq mRNA;Acc:NM_001076813]
potassium voltage-gated channel, subfamily G, member 3 [Source:HGNC Symbol;Acc:HGNC:18306]
Bos taurus myosin, light chain 12B, regulatory (MYL12B), mRNA. [Source:RefSeq mRNA;Acc:NM_001083764]

Bos taurus PR domain containing 8 (PRDM8), mRNA. [Source:RefSeq mRNA;Acc:NM_001103232]
Bos taurus ninjurin 1 (NINJ1), mRNA. [Source:RefSeq mRNA;Acc:NM_001038082]
transcription factor-like 5 (basic helix-loop-helix) [Source:HGNC Symbol;Acc:HGNC:11646]
Olfactory receptor [Source:UniProtKB/TrEMBL;Acc:G3N1S5]
CUGBP, Elav-like family member 2 [Source:HGNC Symbol;Acc:HGNC:2550]
Bos taurus ataxin 7-like 3B (ATXN7L3B), mRNA. [Source:RefSeq mRNA;Acc:NM_001078161]
Bos taurus ring finger protein 11 (RNF11), mRNA. [Source:RefSeq mRNA;Acc:NM_001077953]
Bos taurus T-box 18 (TBX18), mRNA. [Source:RefSeq mRNA;Acc:NM_001192462]
Uncharacterized protein [Source:UniProtKB/TrEMBL;Acc:G3N1J6]
Bos taurus mal, T-cell differentiation protein (MAL), mRNA. [Source:RefSeq mRNA;Acc:NM_001075428]
Olfactory receptor [Source:UniProtKB/TrEMBL;Acc:G3N1Z5]
chromosome 1 open reading frame 110 [Source:HGNC Symbol;Acc:HGNC:28736]
transmembrane emp24-like trafficking protein 10 (yeast) [Source:HGNC Symbol;Acc:HGNC:16998]
GDNF family receptor alpha like [Source:HGNC Symbol;Acc:HGNC:32789]
Bos taurus general transcription factor IIF subunit 2-like (LOC511494), mRNA. [Source:RefSeq mRNA;Acc:NM_001100339]
Bos taurus upregulator of cell proliferation (URGCP), mRNA. [Source:RefSeq mRNA;Acc:NM_001015513]

Bos taurus UDP glycosyltransferase 3 family, polypeptide A2 (UGT3A2), mRNA. [Source:RefSeq mRNA;Acc:NM_001076087]
Bos taurus proline rich 19 (PRR19), mRNA. [Source:RefSeq mRNA;Acc:NM_001075635]

tetratricopeptide repeat domain 12 [Source:HGNC Symbol;Acc:HGNC:23700]
Uncharacterized protein [Source:UniProtKB/TrEMBL;Acc:F1MKV5]

nucleoredoxin-like 2 [Source:HGNC Symbol;Acc:HGNC:30482]
MAX dimerization protein 1 [Source:HGNC Symbol;Acc:HGNC:6761]
F-box and leucine-rich repeat protein 12 [Source:HGNC Symbol;Acc:HGNC:13611]
histamine receptor H2 [Source:HGNC Symbol;Acc:HGNC:5183]
Olfactory receptor [Source:UniProtKB/TrEMBL;Acc:E1BLH3]
Bos taurus CD1a molecule (CD1A), mRNA. [Source:RefSeq mRNA;Acc:NM_001105456]
Bos taurus PHD finger protein 5A (PHF5A), mRNA. [Source:RefSeq mRNA;Acc:NM_001083382]

Bos taurus Williams-Beuren syndrome chromosome region 17 (WBSCR17), mRNA. [Source:RefSeq mRNA;Acc:NM_001192067]
SWI/SNF related, matrix associated, actin dependent regulator of chromatin, subfamily d, member 3; Uncharacterized protein [Source:UniProtKB/TrEMBL;Acc:
Bos taurus chromosome 10 open reading frame, human C15orf23 (C10H15orf23), mRNA. [Source:RefSeq mRNA;Acc:NM_001038055]
arrestin domain containing 5 [Source:HGNC Symbol;Acc:HGNC:31407]
Bos taurus cyclin-dependent kinase inhibitor 2B (p15, inhibits CDK4) (CDKN2B), mRNA. [Source:RefSeq mRNA;Acc:NM_001075894]
Bos taurus SEC14-like 2 (S. cerevisiae) (SEC14L2), mRNA. [Source:RefSeq mRNA;Acc:NM_177943]
Bos taurus EH-domain containing 3 (EHD3), mRNA. [Source:RefSeq mRNA;Acc:NM_001098004]
Bos taurus tetratricopeptide repeat domain 30A (TTC30A), mRNA. [Source:RefSeq mRNA;Acc:NM_001081716]
Bos taurus keratin 75 (KRT75), mRNA. [Source:RefSeq mRNA;Acc:NM_001076917]
Bos taurus pelota homolog (Drosophila) (PELO), mRNA. [Source:RefSeq mRNA;Acc:NM_001034566]

Bos taurus prostaglandin D2 receptor (DP) (PTGDR), mRNA. [Source:RefSeq mRNA;Acc:NM_001098034]
Bos taurus GTP-binding protein 8 (putative) (GTPBP8), mRNA. [Source:RefSeq mRNA;Acc:NM_001075828]
Bos taurus nudix (nucleoside diphosphate linked moiety X)-type motif 7 (NUDT7), mRNA. [Source:RefSeq mRNA;Acc:NM_001109788]
Bos taurus interleukin 8 receptor, beta (CXCR2), mRNA. [Source:RefSeq mRNA;Acc:NM_174360]
Bos taurus ubiquitin C (UBC), mRNA. [Source:RefSeq mRNA;Acc:NM_001206307]
Bos taurus synaptophysin-like 2 (SYPL2), mRNA. [Source:RefSeq mRNA;Acc:NM_001075676]
ring finger protein 125, E3 ubiquitin protein ligase [Source:HGNC Symbol;Acc:HGNC:21150]
Olfactory receptor [Source:UniProtKB/TrEMBL;Acc:G3N226]
Uncharacterized protein [Source:UniProtKB/TrEMBL;Acc:G3N2F8]
Bos taurus apolipoprotein L domain containing 1 (APOLD1), mRNA. [Source:RefSeq mRNA;Acc:NM_001101180]
tumor necrosis factor (ligand) superfamily, member 18 [Source:HGNC Symbol;Acc:HGNC:11932]
Bos taurus pleckstrin homology domain containing, family O member 1 (PLEKHO1), mRNA. [Source:RefSeq mRNA;Acc:NM_001083797]
Bos taurus BRF2, subunit of RNA polymerase III transcription initiation factor, BRF1-like (BRF2), mRNA. [Source:RefSeq mRNA;Acc:NM_001015582]
Bos taurus family with sequence similarity 122A (FAM122A), mRNA. [Source:RefSeq mRNA;Acc:NM_001101196]
Bos taurus N(alpha)-acetyltransferase 60, NatF catalytic subunit (NAA60), mRNA. [Source:RefSeq mRNA;Acc:NM_001075649]
Bos taurus tripartite motif containing 52 (TRIM52), mRNA. [Source:RefSeq mRNA;Acc:NM_001078024]
Bos taurus recoverin (RCVRN), mRNA. [Source:RefSeq mRNA;Acc:NM_174165]
Uncharacterized protein [Source:UniProtKB/TrEMBL;Acc:E1BFA2]
Bos taurus BCL2/adenovirus E1B 19kD interacting protein like (BNIPL), mRNA. [Source:RefSeq mRNA;Acc:NM_001079621]

CTD (carboxy-terminal domain, RNA polymerase II, polypeptide A) small phosphatase-like [Source:HGNC Symbol;Acc:HGNC:16890]
Bos taurus ribosomal protein S6 (RPS6), mRNA. [Source:RefSeq mRNA;Acc:NM_001015548]
Uncharacterized protein [Source:UniProtKB/TrEMBL;Acc:G3MZY1]
Bos taurus dual specificity phosphatase 18 (DUSP18), mRNA. [Source:RefSeq mRNA;Acc:NM_001034259]
Uncharacterized protein [Source:UniProtKB/TrEMBL;Acc:F1MIL7]
Bos taurus dolichol kinase (DOLK), mRNA. [Source:RefSeq mRNA;Acc:NM_001035365]
olfactory receptor, family 10, subfamily G, member 6 [Source:HGNC Symbol;Acc:HGNC:14836]
chromosome 17 open reading frame 70 [Source:HGNC Symbol;Acc:HGNC:26171]
Olfactory receptor [Source:UniProtKB/TrEMBL;Acc:G3MWY2]
Uncharacterized protein [Source:UniProtKB/TrEMBL;Acc:G3N2H8]
Bos taurus hairy/enhancer-of-split related with YRPW motif 2 (HEY2), mRNA. [Source:RefSeq mRNA;Acc:NM_001192055]
glycogenin 2 [Source:HGNC Symbol;Acc:HGNC:4700]
Bos taurus ethanolaminephosphotransferase 1 (CDP-ethanolamine-specific) (EPT1), mRNA. [Source:RefSeq mRNA;Acc:NM_001075257]
Olfactory receptor [Source:UniProtKB/TrEMBL;Acc:E1B9C7]
Bos taurus RAP2B, member of RAS oncogene family (RAP2B), mRNA. [Source:RefSeq mRNA;Acc:NM_001102531]
Bos taurus Fc fragment of IgG, low affinity IIIa, receptor (CD16a) (FCGR3A), transcript variant 1, mRNA. [Source:RefSeq mRNA;Acc:NM_001077402]
Bos taurus transmembrane protein 194A (TMEM194A), mRNA. [Source:RefSeq mRNA;Acc:NM_001102161]
Uncharacterized protein [Source:UniProtKB/TrEMBL;Acc:F1MT39]

Bos taurus RIB43A domain with coiled-coils 2 (RIBC2), mRNA. [Source:RefSeq mRNA;Acc:NM_001038191]
Olfactory receptor [Source:UniProtKB/TrEMBL;Acc:F1MQY6]

Bos taurus nudix (nucleoside diphosphate linked moiety X)-type motif 3 (NUDT3), mRNA. [Source:RefSeq mRNA;Acc:NM_001082466]
Olfactory receptor [Source:UniProtKB/TrEMBL;Acc:F1N435]

zinc finger protein 169 [Source:HGNC Symbol;Acc:HGNC:12957]
Bos taurus D-tyrosyl-tRNA deacylase 1 homolog (S. cerevisiae) (DTD1), mRNA. [Source:RefSeq mRNA;Acc:NM_001038104]

Bos taurus citrate lyase beta like (CLYBL), mRNA. [Source:RefSeq mRNA;Acc:NM_001080296]
Bos taurus reticulocalbin 1, EF-hand calcium binding domain (RCN1), mRNA. [Source:RefSeq mRNA;Acc:NM_001103221]

Palmitoyltransferase [Source:UniProtKB/TrEMBL;Acc:F1MVC6]
TNFAIP3 interacting protein 3 [Source:HGNC Symbol;Acc:HGNC:19315]
Bos taurus HSPB (heat shock 27kDa) associated protein 1 (HSPBAP1), mRNA. [Source:RefSeq mRNA;Acc:NM_001014911]
Bos taurus chemokine (C-X-C motif) ligand 2 (CXCL2), mRNA. [Source:RefSeq mRNA;Acc:NM_174299]
Bos taurus serpin peptidase inhibitor, clade B (ovalbumin), member 5 (SERPINB5), mRNA. [Source:RefSeq mRNA;Acc:NM_001098080]
Bos taurus protein tyrosine phosphatase, non-receptor type 7 (PTPN7), mRNA. [Source:RefSeq mRNA;Acc:NM_001040576]
chromosome 14 open reading frame 119 [Source:HGNC Symbol;Acc:HGNC:20270]
Olfactory receptor [Source:UniProtKB/TrEMBL;Acc:E1BA90]
Bos taurus pyroglutamylated RFamide peptide receptor (QRFPR), mRNA. [Source:RefSeq mRNA;Acc:NM_001192681]

solute carrier family 8 (sodium/calcium exchanger), member 2 [Source:HGNC Symbol;Acc:HGNC:11069]
cyclin-dependent kinase-like 4 [Source:HGNC Symbol;Acc:HGNC:19287]

Bos taurus tsukushi small leucine rich proteoglycan homolog (Xenopus laevis) (TSKU), mRNA. [Source:RefSeq mRNA;Acc:NM_001103268]
olfactory receptor, family 2, subfamily A, member 12 [Source:HGNC Symbol;Acc:HGNC:15082]
zinc finger protein 449 [Source:HGNC Symbol;Acc:HGNC:21039]

Bos taurus leukemia inhibitory factor (cholinergic differentiation factor) (LIF), mRNA. [Source:RefSeq mRNA;Acc:NM_173931]
zinc finger protein 536 [Source:HGNC Symbol;Acc:HGNC:29025]
Bos taurus fibronectin type III domain containing 5 (FNDC5), mRNA. [Source:RefSeq mRNA;Acc:NM_001105421]
Bos taurus RAB32, member RAS oncogene family (RAB32), mRNA. [Source:RefSeq mRNA;Acc:NM_001205950]
serpin peptidase inhibitor, clade B (ovalbumin), member 13 [Source:HGNC Symbol;Acc:HGNC:8944]
zinc finger protein 583 [Source:HGNC Symbol;Acc:HGNC:26427]
Uncharacterized protein [Source:UniProtKB/TrEMBL;Acc:G3N1Y9]
Bos taurus leucine rich repeat containing 17 (LRRC17), mRNA. [Source:RefSeq mRNA;Acc:NM_001078150]
Bos taurus transcriptional adaptor 1 (TADA1), mRNA. [Source:RefSeq mRNA;Acc:NM_001101106]
Bos taurus vitronectin (VTN), mRNA. [Source:RefSeq mRNA;Acc:NM_001035050]
Bos taurus ribophorin I-like (LOC539818), mRNA. [Source:RefSeq mRNA;Acc:NM_001102521]

Uncharacterized protein [Source:UniProtKB/TrEMBL;Acc:G3N254]
Olfactory receptor [Source:UniProtKB/TrEMBL;Acc:G3X8E5]
Olfactory receptor [Source:UniProtKB/TrEMBL;Acc:E1B8U0]
interferon-induced protein 44 [Source:HGNC Symbol;Acc:HGNC:16938]
zinc finger protein 77 [Source:HGNC Symbol;Acc:HGNC:13150]
Uncharacterized protein [Source:UniProtKB/TrEMBL;Acc:F1N0P3]
Bos taurus dimethylarginine dimethylaminohydrolase 1 (DDAH1), mRNA. [Source:RefSeq mRNA;Acc:NM_001102201]

Bos taurus THAP domain containing 11 (THAP11), mRNA. [Source:RefSeq mRNA;Acc:NM_001104994]
Olfactory receptor [Source:UniProtKB/TrEMBL;Acc:F1MAW6]
phosphatidylinositol-5-phosphate 4-kinase, type II, beta [Source:HGNC Symbol;Acc:HGNC:8998]

Uncharacterized protein [Source:UniProtKB/TrEMBL;Acc:F1MG54]
Bos taurus klotho beta (KLB), mRNA. [Source:RefSeq mRNA;Acc:NM_001205326]
Bos taurus transmembrane protein 120A (TMEM120A), mRNA. [Source:RefSeq mRNA;Acc:NM_001079600]
Bos taurus cortexin 3 (CTXN3), mRNA. [Source:RefSeq mRNA;Acc:NM_001105486]
Bos taurus fat storage-inducing transmembrane protein 2 (FITM2), mRNA. [Source:RefSeq mRNA;Acc:NM_001103095]
Bos taurus polymerase (DNA directed), eta (POLH), mRNA. [Source:RefSeq mRNA;Acc:NM_001034450]
Bos taurus ribosomal protein SA (RPSA), mRNA. [Source:RefSeq mRNA;Acc:NM_174379]
chemokine (C-C motif) ligand 24 [Source:HGNC Symbol;Acc:HGNC:10623]

extracellular leucine-rich repeat and fibronectin type III domain containing 2 [Source:HGNC Symbol;Acc:HGNC:29396]
Bos taurus xylosylprotein beta 1,4-galactosyltransferase, polypeptide 7 (galactosyltransferase I) (B4GALT7), mRNA. [Source:RefSeq mRNA;Acc:NM_001075321]
Bos taurus odd-skipped related 1 (Drosophila) (OSR1), mRNA. [Source:RefSeq mRNA;Acc:NM_001076916]
Bos taurus ephrin-A2 (EFNA2), mRNA. [Source:RefSeq mRNA;Acc:NM_001105000]
polycomb group ring finger 2 [Source:HGNC Symbol;Acc:HGNC:12929]
Bos taurus CDC42 small effector 2 (CDC42SE2), mRNA. [Source:RefSeq mRNA;Acc:NM_001102537]
Bos taurus solute carrier family 16, member 3 (monocarboxylic acid transporter 4) (SLC16A3), mRNA. [Source:RefSeq mRNA;Acc:NM_001109980]
Bos taurus zinc finger protein 410 (ZNF410), mRNA. [Source:RefSeq mRNA;Acc:NM_001034609]
Bos taurus chromosome 9 open reading frame, human C6orf120 (C9H6orf120), mRNA. [Source:RefSeq mRNA;Acc:NM_001103331]

Bos taurus stathmin-like 2 (STMN2), mRNA. [Source:RefSeq mRNA;Acc:NM_001034622]
Bos taurus G protein-coupled receptor, family C, group 5, member A (GPRC5A), mRNA. [Source:RefSeq mRNA;Acc:NM_001034515]
pleiomorphic adenoma gene-like 1 [Source:HGNC Symbol;Acc:HGNC:9046]
Bos taurus POM121 membrane glycoprotein-like 2 (POM121L2), mRNA. [Source:RefSeq mRNA;Acc:NM_001162927]
EF-hand calcium binding domain 12 [Source:HGNC Symbol;Acc:HGNC:28061]

t-complex 11 family, X-linked 2 [Source:HGNC Symbol;Acc:HGNC:48335]
Bos taurus smg-8 homolog, nonsense mediated mRNA decay factor (C. elegans) (SMG8), mRNA. [Source:RefSeq mRNA;Acc:NM_001080286]
Uncharacterized protein [Source:UniProtKB/TrEMBL;Acc:M0QW26]
Bos taurus v-maf musculoaponeurotic fibrosarcoma oncogene homolog F (avian) (MAFF), mRNA. [Source:RefSeq mRNA;Acc:NM_001103300]
Bos taurus ATG14 autophagy related 14 homolog (S. cerevisiae) (ATG14), mRNA. [Source:RefSeq mRNA;Acc:NM_001192099]
Bos taurus STAM binding protein (STAMBP), mRNA. [Source:RefSeq mRNA;Acc:NM_001075971]
Bos taurus density-regulated protein (DENR), mRNA. [Source:RefSeq mRNA;Acc:NM_001046528]
Bos taurus tumor necrosis factor, alpha-induced protein 8-like 1 (TNFAIP8L1), mRNA. [Source:RefSeq mRNA;Acc:NM_001103270]
Bos taurus cholinergic receptor, muscarinic 3 (CHRM3), mRNA. [Source:RefSeq mRNA;Acc:NM_174270]
nudix (nucleoside diphosphate linked moiety X)-type motif 5 [Source:HGNC Symbol;Acc:HGNC:8052]
Uncharacterized protein [Source:UniProtKB/TrEMBL;Acc:G3X701]
Uncharacterized protein [Source:UniProtKB/TrEMBL;Acc:G3MX18]
Uncharacterized protein [Source:UniProtKB/TrEMBL;Acc:F1N7M9]

Bos taurus coiled-coil domain containing 111 (CCDC111), mRNA. [Source:RefSeq mRNA;Acc:NM_001075488]
Bos taurus translocase of inner mitochondrial membrane 22 homolog (yeast) (TIMM22), mRNA. [Source:RefSeq mRNA;Acc:NM_001034498]
C-type lectin domain family 4, member D [Source:HGNC Symbol;Acc:HGNC:14554]
Bos taurus netrin G2 (NTNG2), mRNA. [Source:RefSeq mRNA;Acc:NM_001099025]
transcription elongation factor A (SII) N-terminal and central domain containing 2 [Source:HGNC Symbol;Acc:HGNC:26494]
Bos taurus coenzyme Q5 homolog, methyltransferase (S. cerevisiae) (COQ5), mRNA. [Source:RefSeq mRNA;Acc:NM_001075984]
Bos taurus staufen, RNA binding protein, homolog 2 (Drosophila) (STAU2), mRNA. [Source:RefSeq mRNA;Acc:NM_001103313]
Bos taurus armadillo repeat containing, X-linked 2 (ARMCX2), mRNA. [Source:RefSeq mRNA;Acc:NM_001076960]
Bos taurus debranching enzyme homolog 1 (S. cerevisiae) (DBR1), mRNA. [Source:RefSeq mRNA;Acc:NM_001080290]
Bos taurus ermin, ERM-like protein (ERMN), mRNA. [Source:RefSeq mRNA;Acc:NM_001034799]

LysM, putative peptidoglycan-binding, domain containing 3 [Source:HGNC Symbol;Acc:HGNC:26969]
Bos taurus keratin 74 (KRT74), mRNA. [Source:RefSeq mRNA;Acc:NM_001083447]
testis expressed 35 [Source:HGNC Symbol;Acc:HGNC:25366]

Bos taurus ubiquitin-conjugating enzyme E2R 2 (UBE2R2), mRNA. [Source:RefSeq mRNA;Acc:NM_001206110]
Uncharacterized protein [Source:UniProtKB/TrEMBL;Acc:G3MW04]
Olfactory receptor [Source:UniProtKB/TrEMBL;Acc:G3X6K2]
RNA pseudouridylate synthase domain containing 2 [Source:HGNC Symbol;Acc:HGNC:24180]
H2A histone family, member Z [Source:HGNC Symbol;Acc:HGNC:4741]
Bos taurus dentin matrix acidic phosphoprotein 1 (DMP1), mRNA. [Source:RefSeq mRNA;Acc:NM_174038]
Bos taurus cystatin A (stefin A) (CSTA), mRNA. [Source:RefSeq mRNA;Acc:NM_001167824]
Bos taurus dual specificity phosphatase 19 (DUSP19), mRNA. [Source:RefSeq mRNA;Acc:NM_001098878]
Bos taurus lactalbumin, alpha- (LALBA), mRNA. [Source:RefSeq mRNA;Acc:NM_174378]
Bos taurus solute carrier family 31 (copper transporters), member 1 (SLC31A1), mRNA. [Source:RefSeq mRNA;Acc:NM_001100381]
Bos taurus ADP-ribosylation factor-like 4D (ARL4D), mRNA. [Source:RefSeq mRNA;Acc:NM_001076135]
Bos taurus potassium channel tetramerisation domain containing 18 (KCTD18), mRNA. [Source:RefSeq mRNA;Acc:NM_001046514]
Uncharacterized protein [Source:UniProtKB/TrEMBL;Acc:G3N004]
Bos taurus RAB23, member RAS oncogene family (RAB23), mRNA. [Source:RefSeq mRNA;Acc:NM_001206906]
Bos taurus protein phosphatase 6, regulatory subunit 1 (PPP6R1), mRNA. [Source:RefSeq mRNA;Acc:NM_001114507]
neuronal differentiation 1 [Source:HGNC Symbol;Acc:HGNC:7762]
Smith-Magenis syndrome chromosome region, candidate 8 [Source:HGNC Symbol;Acc:HGNC:17921]

coilin [Source:HGNC Symbol;Acc:HGNC:2184]
Bos taurus vacuolar protein sorting 26 homolog B (S. pombe) (VPS26B), mRNA. [Source:RefSeq mRNA;Acc:NM_001192958]
Uncharacterized protein [Source:UniProtKB/TrEMBL;Acc:F1MVJ8]
Bos taurus chromosome 5 open reading frame, human C12orf23 (C5H12orf23), mRNA. [Source:RefSeq mRNA;Acc:NM_001034673]
patched domain-containing protein C6orf138 homolog [Source:RefSeq peptide;Acc:NP_001095613]
Bos taurus paraneoplastic antigen MA1 (PNMA1), mRNA. [Source:RefSeq mRNA;Acc:NM_001104991]
mannosidase, alpha, class 1A, member 1 [Source:HGNC Symbol;Acc:HGNC:6821]
Uncharacterized protein [Source:UniProtKB/TrEMBL;Acc:G5E6P8]

Bos taurus zinc finger protein 750 (ZNF750), mRNA. [Source:RefSeq mRNA;Acc:NM_001081533]
Bos taurus neurexophilin and PC-esterase domain family, member 4 (NXPE4), mRNA. [Source:RefSeq mRNA;Acc:NM_001034503]
Bos taurus zinc finger protein 135 (ZNF135), mRNA. [Source:RefSeq mRNA;Acc:NM_001076816]
chromosome 3 open reading frame 70 [Source:HGNC Symbol;Acc:HGNC:33731]
olfactory receptor, family 10, subfamily D, member 3 (non-functional) [Source:HGNC Symbol;Acc:HGNC:8168]
Olfactory receptor [Source:UniProtKB/TrEMBL;Acc:G3MYH6]
Bos taurus eukaryotic translation initiation factor 4A2 (EIF4A2), mRNA. [Source:RefSeq mRNA;Acc:NM_001034044]
potassium voltage-gated channel, Shal-related subfamily, member 3 [Source:HGNC Symbol;Acc:HGNC:6239]
Bos taurus molybdenum cofactor synthesis 2 (MOCS2), mRNA. [Source:RefSeq mRNA;Acc:NM_001014867]
Bos taurus transmembrane emp24 protein transport domain containing 4 (TMED4), mRNA. [Source:RefSeq mRNA;Acc:NM_001206433]
Uncharacterized protein [Source:UniProtKB/TrEMBL;Acc:M0QW28]
Bos taurus neurensin 2 (NRSN2), mRNA. [Source:RefSeq mRNA;Acc:NM_001101269]
Uncharacterized protein [Source:UniProtKB/TrEMBL;Acc:F1MRG4]

PC4 and SFRS1 interacting protein 1 [Source:HGNC Symbol;Acc:HGNC:9527]
Bos taurus cytochrome c, testis (CYCT), mRNA. [Source:RefSeq mRNA;Acc:NM_001077963]
Bos taurus distal-less homeobox 4 (DLX4), mRNA. [Source:RefSeq mRNA;Acc:NM_001098041]
potassium inwardly-rectifying channel, subfamily J, member 6 [Source:HGNC Symbol;Acc:HGNC:6267]
Bos taurus RAB1A, member RAS oncogene family (RAB1A), mRNA. [Source:RefSeq mRNA;Acc:NM_001033628]
Bos taurus chromosome 24 open reading frame, human C18orf19 (FAM210A), mRNA. [Source:RefSeq mRNA;Acc:NM_001080259]
serine/threonine kinase-like domain containing 1 [Source:HGNC Symbol;Acc:HGNC:28669]
Olfactory receptor [Source:UniProtKB/TrEMBL;Acc:G3MZY6]
Uncharacterized protein [Source:UniProtKB/TrEMBL;Acc:G3N0T2]
Bos taurus ELOVL fatty acid elongase 7 (ELOVL7), mRNA. [Source:RefSeq mRNA;Acc:NM_001078042]
Bos taurus germ cell associated 1 (GSG1), mRNA. [Source:RefSeq mRNA;Acc:NM_001075933]
Bos taurus mitochondrial calcium uptake 1 (MICU1), mRNA. [Source:RefSeq mRNA;Acc:NM_001075338]
Alpha-endosulfine [Source:UniProtKB/Swiss-Prot;Acc:P68210]
Bos taurus translocase of outer mitochondrial membrane 20 homolog (yeast) (TOMM20), mRNA. [Source:RefSeq mRNA;Acc:NM_001099183]
Olfactory receptor [Source:UniProtKB/TrEMBL;Acc:G3N298]
Bos taurus heat-responsive protein 12 (HRSP12), mRNA. [Source:RefSeq mRNA;Acc:NM_001034208]

heat shock transcription factor family member 5 [Source:HGNC Symbol;Acc:HGNC:26862]
nudE neurodevelopment protein 1-like 1 [Source:HGNC Symbol;Acc:HGNC:17620]
Bos taurus NOP16 nucleolar protein homolog (yeast) (NOP16), mRNA. [Source:RefSeq mRNA;Acc:NM_001014856]
Uncharacterized protein [Source:UniProtKB/TrEMBL;Acc:G3N357]
Bos taurus cytochrome P450, family 51, subfamily A, polypeptide 1 (CYP51A1), mRNA. [Source:RefSeq mRNA;Acc:NM_001025319]
Bos taurus serpin peptidase inhibitor, clade A (alpha-1 antiproteinase, antitrypsin), member 5 (SERPINA5), mRNA. [Source:RefSeq mRNA;Acc:NM_176646]
zinc finger and BTB domain containing 25 [Source:HGNC Symbol;Acc:HGNC:13112]
Bos taurus Nedd4 family interacting protein 2 (NDFIP2), mRNA. [Source:RefSeq mRNA;Acc:NM_001205720]
Uncharacterized protein [Source:UniProtKB/TrEMBL;Acc:E1BFC9]
Bos taurus kelch-like ECH-associated protein 1 (KEAP1), mRNA. [Source:RefSeq mRNA;Acc:NM_001101142]

Bos taurus methyltransferase like 14 (METTL14), mRNA. [Source:RefSeq mRNA;Acc:NM_001083714]
Bos taurus TMEM9 domain family, member B (TMEM9B), mRNA. [Source:RefSeq mRNA;Acc:NM_001034507]
Olfactory receptor [Source:UniProtKB/TrEMBL;Acc:E1BNP4]
Uncharacterized protein [Source:UniProtKB/TrEMBL;Acc:E1BNJ6]
Bos taurus ferritin mitochondrial (FTMT), mRNA. [Source:RefSeq mRNA;Acc:NM_001076190]
Bos taurus dihydroxyacetone kinase 2 homolog (S. cerevisiae) (DAK), mRNA. [Source:RefSeq mRNA;Acc:NM_001024524]
nuclear receptor subfamily 6, group A, member 1 [Source:HGNC Symbol;Acc:HGNC:7985]
Olfactory receptor [Source:UniProtKB/TrEMBL;Acc:E1BEX9]
SLIT and NTRK-like family, member 1 [Source:HGNC Symbol;Acc:HGNC:20297]
Bos taurus CWF19-like 1, cell cycle control (S. pombe) (CWF19L1), mRNA. [Source:RefSeq mRNA;Acc:NM_001192085]
Bos taurus EPH receptor B4 (EPHB4), mRNA. [Source:RefSeq mRNA;Acc:NM_001206268]
Bos taurus PHD finger protein 11 (PHF11), mRNA. [Source:RefSeq mRNA;Acc:NM_001046144]
Uncharacterized protein [Source:UniProtKB/TrEMBL;Acc:G5E5B1]
Bos taurus protein phosphatase, Mg2+/Mn2+ dependent, 1L (PPM1L), mRNA. [Source:RefSeq mRNA;Acc:NM_001099118]

Bos taurus CTF8, chromosome transmission fidelity factor 8 homolog (S. cerevisiae) (CHTF8), mRNA. [Source:RefSeq mRNA;Acc:NM_001114969]

transmembrane protein 130 [Source:HGNC Symbol;Acc:HGNC:25429]

Uncharacterized protein [Source:UniProtKB/TrEMBL;Acc:G3X8C0]
Bos taurus serpin peptidase inhibitor, clade B (ovalbumin), member 6 (SERPINB6), mRNA. [Source:RefSeq mRNA;Acc:NM_174789]
RNA binding motif protein 12B [Source:HGNC Symbol;Acc:HGNC:32310]
Bos taurus peroxisomal membrane protein 4, 24kDa (PXMP4), mRNA. [Source:RefSeq mRNA;Acc:NM_001099163]
Bos taurus sepiapterin reductase (7,8-dihydrobiopterin:NADP+ oxidoreductase) (SPR), mRNA. [Source:RefSeq mRNA;Acc:NM_001076003]
Bos taurus myristoylated alanine-rich protein kinase C substrate (MARCKS), mRNA. [Source:RefSeq mRNA;Acc:NM_001076276]

Olfactory receptor [Source:UniProtKB/TrEMBL;Acc:F1N5W3]
Bos taurus dopamine receptor D1 (DRD1), mRNA. [Source:RefSeq mRNA;Acc:NM_174042]
Bos taurus lipoyltransferase 1 (LIPT1), mRNA. [Source:RefSeq mRNA;Acc:NM_174795]
Protein IMPACT [Source:UniProtKB/Swiss-Prot;Acc:A7YY45]
Uncharacterized protein [Source:UniProtKB/TrEMBL;Acc:F1MMF2]
Bos taurus histidine decarboxylase (HDC), mRNA. [Source:RefSeq mRNA;Acc:NM_001024551]
Bos taurus Wiskott-Aldrich syndrome-like (WASL), mRNA. [Source:RefSeq mRNA;Acc:NM_174219]

nucleosome assembly protein 1-like 1 [Source:HGNC Symbol;Acc:HGNC:7637]
Bos taurus leucine carboxyl methyltransferase 1 (LCMT1), mRNA. [Source:RefSeq mRNA;Acc:NM_001076488]
Bos taurus THAP domain containing 9 (THAP9), mRNA. [Source:RefSeq mRNA;Acc:NM_001244183]
Bos taurus high mobility group box 3 (HMGB3), transcript variant 1, mRNA. [Source:RefSeq mRNA;Acc:NM_001076285]
CD177 molecule [Source:HGNC Symbol;Acc:HGNC:30072]
Bos taurus phospholipase A2, group IIF (PLA2G2F), mRNA. [Source:RefSeq mRNA;Acc:NM_001102522]
Bos taurus microtubule-associated protein 1 light chain 3 beta (MAP1LC3B), mRNA. [Source:RefSeq mRNA;Acc:NM_001001169]
Bos taurus pro-melanin-concentrating hormone (PMCH), mRNA. [Source:RefSeq mRNA;Acc:NM_001080240]
Uncharacterized protein [Source:UniProtKB/TrEMBL;Acc:G3X800]
chemokine (C-X3-C motif) ligand 1 [Source:HGNC Symbol;Acc:HGNC:10647]

Uncharacterized protein [Source:UniProtKB/TrEMBL;Acc:F1ME51]
Bos taurus RAB40C, member RAS oncogene family (RAB40C), mRNA. [Source:RefSeq mRNA;Acc:NM_001081545]
calcium/calmodulin-dependent protein kinase ID [Source:HGNC Symbol;Acc:HGNC:19341]
Uncharacterized protein [Source:UniProtKB/TrEMBL;Acc:M0QW38]

Bos taurus interferon regulatory factor 2 (IRF2), mRNA. [Source:RefSeq mRNA;Acc:NM_001205793]
Bos taurus chromosome 10 open reading frame, human C15orf57 (C10H15orf57), mRNA. [Source:RefSeq mRNA;Acc:NM_001075286]
Bos taurus DnaJ (Hsp40) homolog, subfamily C, member 18 (DNAJC18), mRNA. [Source:RefSeq mRNA;Acc:NM_001015649]
Uncharacterized protein [Source:UniProtKB/TrEMBL;Acc:G3N2M9]
Bos taurus guanylate cyclase 1, soluble, alpha 3 (GUCY1A3), mRNA. [Source:RefSeq mRNA;Acc:NM_175778]
Uncharacterized protein [Source:UniProtKB/TrEMBL;Acc:G3X8F1]

potassium voltage-gated channel, subfamily H (eag-related), member 8 [Source:HGNC Symbol;Acc:HGNC:18864]
death-associated protein kinase 2 [Source:HGNC Symbol;Acc:HGNC:2675]
Bos taurus transcription elongation factor A (SII)-like 1 (TCEAL1), mRNA. [Source:RefSeq mRNA;Acc:NM_001045960]
Bos taurus cell cycle associated protein 1 (CAPRIN1), mRNA. [Source:RefSeq mRNA;Acc:NM_001076062]
Bos taurus glucose-6-phosphatase, catalytic subunit (G6PC), mRNA. [Source:RefSeq mRNA;Acc:NM_001076124]
Uncharacterized protein [Source:UniProtKB/TrEMBL;Acc:G3X8G7]
Uncharacterized protein [Source:UniProtKB/TrEMBL;Acc:F1N4G1]
Olfactory receptor [Source:UniProtKB/TrEMBL;Acc:F1MZ29]
Bos taurus neurexophilin 2 (NXPH2), mRNA. [Source:RefSeq mRNA;Acc:NM_174406]
Bos taurus methionine sulfoxide reductase A (MSRA), mRNA. [Source:RefSeq mRNA;Acc:NM_174114]
potassium voltage-gated channel, shaker-related subfamily, member 3 [Source:HGNC Symbol;Acc:HGNC:6221]
Olfactory receptor [Source:UniProtKB/TrEMBL;Acc:E1BH00]
Uncharacterized protein [Source:UniProtKB/TrEMBL;Acc:G3MWS6]
coiled-coil domain containing 169 [Source:HGNC Symbol;Acc:HGNC:34361]
Bos taurus melanoma antigen family F, 1 (MAGEF1), mRNA. [Source:RefSeq mRNA;Acc:NM_001102049]
Uncharacterized protein [Source:UniProtKB/TrEMBL;Acc:E1BM69]
Bos taurus leucine-rich, glioma inactivated 1 (LGI1), mRNA. [Source:RefSeq mRNA;Acc:NM_001046591]
Uncharacterized protein [Source:UniProtKB/TrEMBL;Acc:G3MZ92]
Bos taurus HtrA serine peptidase 2 (HTRA2), mRNA. [Source:RefSeq mRNA;Acc:NM_001077956]
Bos taurus protocadherin gamma subfamily A, 2 (PCDHGA2), mRNA. [Source:RefSeq mRNA;Acc:NM_001099368]
Bos taurus sorting nexin 33 (SNX33), mRNA. [Source:RefSeq mRNA;Acc:NM_001205702]
arginyltransferase 1 [Source:HGNC Symbol;Acc:HGNC:782]

Bos taurus homeobox D10 (HOXD10), mRNA. [Source:RefSeq mRNA;Acc:NM_001099105]

Bos taurus SCAN domain containing 1-like (LOC515042), mRNA. [Source:RefSeq mRNA;Acc:NM_001105370]
Bos taurus RAD23 homolog A (S. cerevisiae) (RAD23A), mRNA. [Source:RefSeq mRNA;Acc:NM_001082614]
Uncharacterized protein [Source:UniProtKB/TrEMBL;Acc:E1BIS7]
protocadherin beta 14 [Source:HGNC Symbol;Acc:HGNC:8685]
family with sequence similarity 83, member C [Source:HGNC Symbol;Acc:HGNC:16121]
zinc finger protein 235 [Source:HGNC Symbol;Acc:HGNC:12866]
T cell receptor beta variable 29-1 [Source:HGNC Symbol;Acc:HGNC:12210]

Bos taurus pleckstrin homology domain containing, family G (with RhoGef domain) member 1 (PLEKHG1), mRNA. [Source:RefSeq mRNA;Acc:NM_001205921]
Uncharacterized protein [Source:UniProtKB/TrEMBL;Acc:F1N3N1]
Uncharacterized protein [Source:UniProtKB/TrEMBL;Acc:D0PWG1]
Bos taurus lectin, galactoside-binding, soluble, 9 (LGALS9), transcript variant 2, mRNA. [Source:RefSeq mRNA;Acc:NM_001015570]
Olfactory receptor [Source:UniProtKB/TrEMBL;Acc:G5E5H3]
A kinase (PRKA) anchor protein 3 [Source:HGNC Symbol;Acc:HGNC:373]
interleukin 27 [Source:HGNC Symbol;Acc:HGNC:19157]
Bos taurus immunoglobulin (CD79A) binding protein 1-like (LOC534155), mRNA. [Source:RefSeq mRNA;Acc:NM_001104988]
Ras association (RalGDS/AF-6) domain family member 1 [Source:HGNC Symbol;Acc:HGNC:9882]
RWD domain containing 2A [Source:HGNC Symbol;Acc:HGNC:21385]
Bos taurus proline rich 15-like (PRR15L), mRNA. [Source:RefSeq mRNA;Acc:NM_001034794]
Olfactory receptor [Source:UniProtKB/TrEMBL;Acc:G3N1T0]
solute carrier organic anion transporter family, member 4A1 [Source:HGNC Symbol;Acc:HGNC:10953]
Bos taurus family with sequence similarity 89, member A (FAM89A), mRNA. [Source:RefSeq mRNA;Acc:NM_001101257]
Bos taurus serine incorporator 1 (SERINC1), mRNA. [Source:RefSeq mRNA;Acc:NM_001035427]
Bos taurus tumor necrosis factor (ligand) superfamily, member 11 (TNFSF11), mRNA. [Source:RefSeq mRNA;Acc:NM_001205770]
Olfactory receptor [Source:UniProtKB/TrEMBL;Acc:G3N306]
Uncharacterized protein [Source:UniProtKB/TrEMBL;Acc:G3N3D7]
Bos taurus ubiquitin-conjugating enzyme E2H (UBE2H), mRNA. [Source:RefSeq mRNA;Acc:NM_001037614]
Bos taurus ELOVL fatty acid elongase 6 (ELOVL6), mRNA. [Source:RefSeq mRNA;Acc:NM_001102155]

Bos taurus SIL1 homolog, endoplasmic reticulum chaperone (S. cerevisiae) (SIL1), mRNA. [Source:RefSeq mRNA;Acc:NM_001075808]
Bos taurus tubulin, delta 1 (TUBD1), mRNA. [Source:RefSeq mRNA;Acc:NM_001075470]
Bos taurus adhesion molecule with Ig-like domain 2 (AMIGO2), mRNA. [Source:RefSeq mRNA;Acc:NM_001205786]
Bos taurus peroxisomal biogenesis factor 19 (PEX19), mRNA. [Source:RefSeq mRNA;Acc:NM_001034540]
Olfactory receptor [Source:UniProtKB/TrEMBL;Acc:G5E673]
junctophilin 4 [Source:HGNC Symbol;Acc:HGNC:20156]
Uncharacterized protein [Source:UniProtKB/TrEMBL;Acc:G3N2W4]
Bos taurus chromosome 23 open reading frame, human C6orf141 (C23H6orf141), mRNA. [Source:RefSeq mRNA;Acc:NM_001145653]
Bos taurus chromosome 13 open reading frame, human C20orf111 (C13H20orf111), mRNA. [Source:RefSeq mRNA;Acc:NM_001015568]
Bos taurus phosphorylated adaptor for RNA export (PHAX), mRNA. [Source:RefSeq mRNA;Acc:NM_001166562]
family with sequence similarity 126, member A [Source:HGNC Symbol;Acc:HGNC:24587]
Olfactory receptor [Source:UniProtKB/TrEMBL;Acc:F1MRS9]
phosphofurin acidic cluster sorting protein 2 [Source:HGNC Symbol;Acc:HGNC:23794]
junctophilin 1 [Source:HGNC Symbol;Acc:HGNC:14201]
thymus, brain and testes associated [Source:HGNC Symbol;Acc:HGNC:23511]
Olfactory receptor [Source:UniProtKB/TrEMBL;Acc:G3N379]
Bos taurus endoplasmic reticulum protein 29 (ERP29), mRNA. [Source:RefSeq mRNA;Acc:NM_001076271]
Bos taurus lactate dehydrogenase D (LDHD), mRNA. [Source:RefSeq mRNA;Acc:NM_001075443]
Uncharacterized protein [Source:UniProtKB/TrEMBL;Acc:E1B8V8]
Bos taurus fibronectin type III domain containing 8 (FNDC8), mRNA. [Source:RefSeq mRNA;Acc:NM_001076444]
filamin A interacting protein 1-like [Source:HGNC Symbol;Acc:HGNC:24589]
olfactory receptor, family 4, subfamily K, member 1 [Source:HGNC Symbol;Acc:HGNC:14726]
Olfactory receptor [Source:UniProtKB/TrEMBL;Acc:G3N044]
Bos taurus PIH1 domain containing 3 (PIH1D3), mRNA. [Source:RefSeq mRNA;Acc:NM_001046029]
synaptotagmin binding, cytoplasmic RNA interacting protein [Source:HGNC Symbol;Acc:HGNC:16918]
Bos taurus glutaminyl-peptide cyclotransferase-like (QPCTL), mRNA. [Source:RefSeq mRNA;Acc:NM_001075940]
Bos taurus prion protein (PRNP), transcript variant 2, mRNA. [Source:RefSeq mRNA;Acc:NM_181015]
SLIT and NTRK-like family, member 3 [Source:HGNC Symbol;Acc:HGNC:23501]
Olfactory receptor [Source:UniProtKB/TrEMBL;Acc:E1BN84]
Bos taurus chemokine (C-X-C motif) ligand 12 (CXCL12), mRNA. [Source:RefSeq mRNA;Acc:NM_001113174]

tRNA wybutosine-synthesizing protein 1 homolog [Source:RefSeq peptide;Acc:NP_001179682]
Bos taurus radical S-adenosyl methionine domain containing 2 (RSAD2), mRNA. [Source:RefSeq mRNA;Acc:NM_001045941]
Bos taurus SPC25, NDC80 kinetochore complex component, homolog (S. cerevisiae) (SPC25), mRNA. [Source:RefSeq mRNA;Acc:NM_001034230]
Bos taurus potassium voltage-gated channel, subfamily H (eag-related), member 5 (KCNH5), mRNA. [Source:RefSeq mRNA;Acc:NM_001192989]
Bos taurus dynein, axonemal, assembly factor 1 (DNAAF1), mRNA. [Source:RefSeq mRNA;Acc:NM_001035345]
Bos taurus protein-L-isoaspartate (D-aspartate) O-methyltransferase domain containing 1 (PCMTD1), mRNA. [Source:RefSeq mRNA;Acc:NM_001081526]
Bos taurus ectodermal-neural cortex 1 (with BTB-like domain) (ENC1), mRNA. [Source:RefSeq mRNA;Acc:NM_001078067]
Bos taurus NRDE-2, necessary for RNA interference, domain containing (NRDE2), mRNA. [Source:RefSeq mRNA;Acc:NM_001206507]

FAM47E-STBD1 readthrough [Source:HGNC Symbol;Acc:HGNC:44667]
Olfactory receptor [Source:UniProtKB/TrEMBL;Acc:F1MCY6]
Bos taurus E2F-associated phosphoprotein (EAPP), mRNA. [Source:RefSeq mRNA;Acc:NM_001075800]
Bos taurus fucosyltransferase 9 (alpha (1,3) fucosyltransferase) (FUT9), mRNA. [Source:RefSeq mRNA;Acc:NM_174735]
protocadherin 9 [Source:HGNC Symbol;Acc:HGNC:8661]
Uncharacterized protein [Source:UniProtKB/TrEMBL;Acc:F1N6Y4]
potassium voltage-gated channel, shaker-related subfamily, member 2 [Source:HGNC Symbol;Acc:HGNC:6220]
Bos taurus ribonuclease P/MRP 14kDa subunit (RPP14), mRNA. [Source:RefSeq mRNA;Acc:NM_001077924]
Olfactory receptor [Source:UniProtKB/TrEMBL;Acc:G3N2T0]
signal recognition particle receptor, B subunit [Source:HGNC Symbol;Acc:HGNC:24085]
Bos taurus homeobox B4 (HOXB4), mRNA. [Source:RefSeq mRNA;Acc:NM_001078114]
Uncharacterized protein [Source:UniProtKB/TrEMBL;Acc:G3MWQ1]
Bos taurus membrane-spanning 4-domains, subfamily A, member 1 (MS4A1), mRNA. [Source:RefSeq mRNA;Acc:NM_001077854]
Bos taurus ER degradation enhancer, mannosidase alpha-like 1 (EDEM1), mRNA. [Source:RefSeq mRNA;Acc:NM_001103092]
ankyrin repeat domain 34B [Source:HGNC Symbol;Acc:HGNC:33736]
Bos taurus major histocompatibility complex, class I (LOC512672), mRNA. [Source:RefSeq mRNA;Acc:NM_001076841]
Bos taurus centrosomal protein 19kDa (CEP19), mRNA. [Source:RefSeq mRNA;Acc:NM_001099129]
Olfactory receptor [Source:UniProtKB/TrEMBL;Acc:G3MYJ5]
Bos taurus centromere protein H (CENPH), mRNA. [Source:RefSeq mRNA;Acc:NM_001034236]

KIAA2026 [Source:HGNC Symbol;Acc:HGNC:23378]

Bos taurus single-pass membrane protein with coiled-coil domains 3 (SMCO3), mRNA. [Source:RefSeq mRNA;Acc:NM_001206296]
Bos taurus haloacid dehalogenase-like hydrolase domain containing 3 (HDHD3), mRNA. [Source:RefSeq mRNA;Acc:NM_001014896]
Bos taurus nudix (nucleoside diphosphate linked moiety X)-type motif 13 (NUDT13), mRNA. [Source:RefSeq mRNA;Acc:NM_001045905]
family with sequence similarity 170, member A [Source:HGNC Symbol;Acc:HGNC:27963]
Uncharacterized protein [Source:UniProtKB/TrEMBL;Acc:G3N289]
Bos taurus mitogen-activated protein kinase 7 (MAPK7), mRNA. [Source:RefSeq mRNA;Acc:NM_001099080]
integrin, beta-like 1 (with EGF-like repeat domains) [Source:HGNC Symbol;Acc:HGNC:6164]
Bos taurus high mobility group box 2 (HMGB2), mRNA. [Source:RefSeq mRNA;Acc:NM_001037616]
Bos taurus solute carrier organic anion transporter family, member 2A1 (SLCO2A1), mRNA. [Source:RefSeq mRNA;Acc:NM_174829]
Bos taurus sphingomyelin phosphodiesterase 4, neutral membrane (neutral sphingomyelinase-3) (SMPD4), mRNA. [Source:RefSeq mRNA;Acc:NM_001205602]
Bos taurus small integral membrane protein 13 (SMIM13), mRNA. [Source:RefSeq mRNA;Acc:NM_001135578]
Uncharacterized protein [Source:UniProtKB/TrEMBL;Acc:F1MP77]
Bos taurus coiled-coil domain containing 80 (CCDC80), mRNA. [Source:RefSeq mRNA;Acc:NM_001098982]
Olfactory receptor [Source:UniProtKB/TrEMBL;Acc:F1N7S5]
coiled-coil domain containing 28A [Source:HGNC Symbol;Acc:HGNC:21098]
Bos taurus monoacylglycerol O-acyltransferase 1 (MOGAT1), mRNA. [Source:RefSeq mRNA;Acc:NM_001001153]
Bos taurus X-box binding protein 1 (XBP1), transcript variant 1, mRNA. [Source:RefSeq mRNA;Acc:NM_001034727]
Uncharacterized protein [Source:UniProtKB/TrEMBL;Acc:G3X7A1]
Uncharacterized protein [Source:UniProtKB/TrEMBL;Acc:F1MGN4]
Bos taurus peroxisomal biogenesis factor 10 (PEX10), mRNA. [Source:RefSeq mRNA;Acc:NM_001076333]
Bos taurus mitogen-activated protein kinase kinase kinase 7 (MAP3K7), mRNA. [Source:RefSeq mRNA;Acc:NM_001081595]
Uncharacterized protein [Source:UniProtKB/TrEMBL;Acc:E1BGM4]
Bos taurus TOX high mobility group box family member 4 (TOX4), mRNA. [Source:RefSeq mRNA;Acc:NM_001075707]
Bos taurus CD74 molecule, major histocompatibility complex, class II invariant chain (CD74), mRNA. [Source:RefSeq mRNA;Acc:NM_001034735]
Uncharacterized protein [Source:UniProtKB/TrEMBL;Acc:F1ML48]
Bos taurus ubiquitin-like 4B (UBL4B), mRNA. [Source:RefSeq mRNA;Acc:NM_001080734]
Bos taurus solute carrier family 35, member A4 (SLC35A4), mRNA. [Source:RefSeq mRNA;Acc:NM_001079612]
Olfactory receptor [Source:UniProtKB/TrEMBL;Acc:G3MZ74]
cleavage and polyadenylation specific factor 7, 59kDa [Source:HGNC Symbol;Acc:HGNC:30098]

Bos taurus RNA (guanine-9-) methyltransferase domain containing 2 (TRMT10A), mRNA. [Source:RefSeq mRNA;Acc:NM_001035477]
Uncharacterized protein [Source:UniProtKB/TrEMBL;Acc:E1B7X2]
Bos taurus TSPY-like 4 (TSPYL4), mRNA. [Source:RefSeq mRNA;Acc:NM_001101064]
Bos taurus transcription factor B1, mitochondrial (TFB1M), transcript variant 2, mRNA. [Source:RefSeq mRNA;Acc:NM_001076896]

Bos taurus ArfGAP with coiled-coil, ankyrin repeat and PH domains 2 (ACAP2), mRNA. [Source:RefSeq mRNA;Acc:NM_001105337]
Uncharacterized protein [Source:UniProtKB/TrEMBL;Acc:F1MZI6]
Bos taurus pannexin 3 (PANX3), mRNA. [Source:RefSeq mRNA;Acc:NM_001144084]

Bos taurus PDZ domain containing 3 (PDZD3), mRNA. [Source:RefSeq mRNA;Acc:NM_001034664]
Bos taurus LSM12 homolog (S. cerevisiae) (LSM12), mRNA. [Source:RefSeq mRNA;Acc:NM_001076098]
Bos taurus core 1 synthase, glycoprotein-N-acetylgalactosamine 3-beta-galactosyltransferase, 1 (C1GALT1), mRNA. [Source:RefSeq mRNA;Acc:NM_001076160]
40S ribosomal protein S3a [Source:UniProtKB/TrEMBL;Acc:G3N262]
Olfactory receptor [Source:UniProtKB/TrEMBL;Acc:E1BA55]
armadillo repeat containing, X-linked 3 [Source:HGNC Symbol;Acc:HGNC:24065]

Bos taurus kelch-like 9 (Drosophila) (KLHL9), mRNA. [Source:RefSeq mRNA;Acc:NM_001076956]
Bos taurus GLI pathogenesis-related 1 (GLIPR1), mRNA. [Source:RefSeq mRNA;Acc:NM_001076984]
Bos taurus interferon-induced protein with tetratricopeptide repeats 5 (IFIT5), mRNA. [Source:RefSeq mRNA;Acc:NM_001075698]
transmembrane protein 132B [Source:HGNC Symbol;Acc:HGNC:29397]
intracisternal A particle-promoted polypeptide [Source:HGNC Symbol;Acc:HGNC:6108]
Uncharacterized protein [Source:UniProtKB/TrEMBL;Acc:F1MJW1]
Bos taurus E2F transcription factor 6 (E2F6), mRNA. [Source:RefSeq mRNA;Acc:NM_001076848]

taste receptor, type 2, member 38 [Source:HGNC Symbol;Acc:HGNC:9584]
Uncharacterized protein [Source:UniProtKB/TrEMBL;Acc:G3X840]
histone cluster 2, H4a [Source:HGNC Symbol;Acc:HGNC:4794]
protocadherin gamma subfamily B, 1 [Source:HGNC Symbol;Acc:HGNC:8708]
Uncharacterized protein [Source:UniProtKB/TrEMBL;Acc:F1MUK9]
Uncharacterized protein [Source:UniProtKB/TrEMBL;Acc:E1BQ26]

zinc finger and BTB domain containing 37 [Source:HGNC Symbol;Acc:HGNC:28365]
Bos taurus platelet-derived growth factor receptor-like (PDGFRL), mRNA. [Source:RefSeq mRNA;Acc:NM_001035301]
Bos taurus Meis homeobox 2 (MEIS2), mRNA. [Source:RefSeq mRNA;Acc:NM_001191269]
Bos taurus deoxyribonuclease I-like 2 (DNASE1L2), mRNA. [Source:RefSeq mRNA;Acc:NM_001105019]
leucine rich repeat containing 8 family, member B [Source:HGNC Symbol;Acc:HGNC:30692]
chromosome 9 open reading frame 153 [Source:HGNC Symbol;Acc:HGNC:31456]
Bos taurus BCL2-associated athanogene (BAG1), mRNA. [Source:RefSeq mRNA;Acc:NM_001076291]
Bos taurus partner of NOB1 homolog (S. cerevisiae) (PNO1), mRNA. [Source:RefSeq mRNA;Acc:NM_183085]
Bos taurus protein phosphatase, Mg2+/Mn2+ dependent, 1K (PPM1K), mRNA. [Source:RefSeq mRNA;Acc:NM_001046474]
fidgetin [Source:HGNC Symbol;Acc:HGNC:13285]
Bos taurus basic leucine zipper nuclear factor 1 (BLZF1), mRNA. [Source:RefSeq mRNA;Acc:NM_001045899]

Bos taurus small integral membrane protein 20 (SMIM20), mRNA. [Source:RefSeq mRNA;Acc:NM_001145428]

Bos taurus hydroxysteroid (11-beta) dehydrogenase 1 (HSD11B1), mRNA. [Source:RefSeq mRNA;Acc:NM_001123032]
LYN proto-oncogene, Src family tyrosine kinase [Source:HGNC Symbol;Acc:HGNC:6735]
Bos taurus fin bud initiation factor homolog (zebrafish) (FIBIN), mRNA. [Source:RefSeq mRNA;Acc:NM_001015541]
Bos taurus HNF1 homeobox A (HNF1A), mRNA. [Source:RefSeq mRNA;Acc:NM_001192524]
Olfactory receptor [Source:UniProtKB/TrEMBL;Acc:G3X868]
ubiquitin specific peptidase 53 [Source:HGNC Symbol;Acc:HGNC:29255]
zinc finger protein 605 [Source:HGNC Symbol;Acc:HGNC:28068]
Uncharacterized protein [Source:UniProtKB/TrEMBL;Acc:F1ME60]
probable polyprenol reductase [Source:RefSeq peptide;Acc:NP_001193019]
Bos taurus myeloid-associated differentiation marker (MYADM), mRNA. [Source:RefSeq mRNA;Acc:NM_001075252]
Bos taurus solute carrier family 25 (mitochondrial carrier; peroxisomal membrane protein, 34kDa), member 17 (SLC25A17), mRNA. [Source:RefSeq mRNA;Acc:N
SET domain containing (lysine methyltransferase) 7 [Source:HGNC Symbol;Acc:HGNC:30412]
Uncharacterized protein [Source:UniProtKB/TrEMBL;Acc:F1MRL5]
Bos taurus protein phosphatase 2, catalytic subunit, alpha isozyme (PPP2CA), mRNA. [Source:RefSeq mRNA;Acc:NM_181031]
Bos taurus coiled-coil domain containing 86 (CCDC86), mRNA. [Source:RefSeq mRNA;Acc:NM_001038574]
olfactory receptor, family 10, subfamily G, member 3 [Source:HGNC Symbol;Acc:HGNC:8171]

T-box 5 [Source:HGNC Symbol;Acc:HGNC:11604]
Bos taurus phosphotyrosine interaction domain containing 1 (PID1), mRNA. [Source:RefSeq mRNA;Acc:NM_001079584]
Bos taurus C1GALT1-specific chaperone 1 (C1GALT1C1), mRNA. [Source:RefSeq mRNA;Acc:NM_001034575]
Bos taurus amyloid P component, serum (APCS), mRNA. [Source:RefSeq mRNA;Acc:NM_001034466]
Bos taurus glycerophosphodiester phosphodiesterase domain containing 5 (GDPD5), mRNA. [Source:RefSeq mRNA;Acc:NM_001101141]
ring finger protein 175 [Source:HGNC Symbol;Acc:HGNC:27735]
Bos taurus haptoglobin (HP), mRNA. [Source:RefSeq mRNA;Acc:NM_001040470]
Olfactory receptor [Source:UniProtKB/TrEMBL;Acc:F1MVE3]
Bos taurus LIM and SH3 protein 1 (LASP1), mRNA. [Source:RefSeq mRNA;Acc:NM_001035394]
solute carrier family 35, member F3 [Source:HGNC Symbol;Acc:HGNC:23616]
trafficking protein particle complex 3 [Source:HGNC Symbol;Acc:HGNC:19942]
Bos taurus ARP8 actin-related protein 8 homolog (yeast) (ACTR8), mRNA. [Source:RefSeq mRNA;Acc:NM_001076361]
Bos taurus unc-50 homolog (C. elegans) (UNC50), mRNA. [Source:RefSeq mRNA;Acc:NM_001014921]
Bos taurus interleukin 22 receptor, alpha 1 (IL22RA1), mRNA. [Source:RefSeq mRNA;Acc:NM_001034311]
Uncharacterized protein [Source:UniProtKB/TrEMBL;Acc:F1N0V5]
Bos taurus DnaJ (Hsp40) homolog, subfamily A, member 4 (DNAJA4), mRNA. [Source:RefSeq mRNA;Acc:NM_001102120]
Bos taurus chaperonin containing TCP1, subunit 2 (beta) (CCT2), mRNA. [Source:RefSeq mRNA;Acc:NM_001034239]
Olfactory receptor [Source:UniProtKB/TrEMBL;Acc:M0QVY4]
Bos taurus CD34 molecule (CD34), mRNA. [Source:RefSeq mRNA;Acc:NM_174009]
Bos taurus major histocompatibility complex, class II, DQ beta (BOLA-DQB), mRNA. [Source:RefSeq mRNA;Acc:NM_001012676]
Bos taurus glucosaminyl (N-acetyl) transferase 2, I-branching enzyme (I blood group) (GCNT2), mRNA. [Source:RefSeq mRNA;Acc:NM_001101227]
Bos taurus oxidoreductase NAD-binding domain containing 1 (OXNAD1), mRNA. [Source:RefSeq mRNA;Acc:NM_001035090]
Uncharacterized protein [Source:UniProtKB/TrEMBL;Acc:G3N1H7]
olfactory receptor, family 1, subfamily L, member 3 [Source:HGNC Symbol;Acc:HGNC:8215]

G protein-coupled receptor 141 [Source:HGNC Symbol;Acc:HGNC:19997]
KIAA0895 [Source:HGNC Symbol;Acc:HGNC:22206]
Bos taurus adaptor-related protein complex 5, sigma 1 subunit (AP5S1), mRNA. [Source:RefSeq mRNA;Acc:NM_001034811]
Bos taurus neurotrophin 3 (NTF3), mRNA. [Source:RefSeq mRNA;Acc:NM_001077988]
Olfactory receptor [Source:UniProtKB/TrEMBL;Acc:G3MZZ2]

Bos taurus protein S (alpha) (PROS1), mRNA. [Source:RefSeq mRNA;Acc:NM_174438]
Bos taurus chromosome 28 open reading frame, human C10orf57 (TMEM254), mRNA. [Source:RefSeq mRNA;Acc:NM_001075228]
Olfactory receptor [Source:UniProtKB/TrEMBL;Acc:E1BFG5]
Uncharacterized protein [Source:UniProtKB/TrEMBL;Acc:F1MG99]
NHS-like 2 [Source:HGNC Symbol;Acc:HGNC:33737]
Uncharacterized protein [Source:UniProtKB/TrEMBL;Acc:E1BG03]
pseudopodium-enriched atypical kinase 1 [Source:HGNC Symbol;Acc:HGNC:29431]
Olfactory receptor [Source:UniProtKB/TrEMBL;Acc:G3MYT3]
Bos taurus mariner-like transposase (LOC534913), mRNA. [Source:RefSeq mRNA;Acc:NM_001083726]
F-box protein 40 [Source:HGNC Symbol;Acc:HGNC:29816]
Bos taurus B lymphoid tyrosine kinase (BLK), mRNA. [Source:RefSeq mRNA;Acc:NM_001075968]
RAD51 paralog B [Source:HGNC Symbol;Acc:HGNC:9822]

chromosome X open reading frame 65 [Source:HGNC Symbol;Acc:HGNC:33713]
Bos taurus acyl-CoA thioesterase 4 (ACOT4), mRNA. [Source:RefSeq mRNA;Acc:NM_001098941]
olfactory receptor, family 52, subfamily K, member 1 [Source:HGNC Symbol;Acc:HGNC:15222]
Bos taurus tyrosylprotein sulfotransferase 2 (TPST2), mRNA. [Source:RefSeq mRNA;Acc:NM_001035429]
Olfactory receptor [Source:UniProtKB/TrEMBL;Acc:G3MXZ1]
hypoxanthine phosphoribosyltransferase 1 [Source:HGNC Symbol;Acc:HGNC:5157]
Olfactory receptor [Source:UniProtKB/TrEMBL;Acc:F1MBA9]
Uncharacterized protein [Source:UniProtKB/TrEMBL;Acc:G3MZJ6]
prokineticin receptor 1 [Source:HGNC Symbol;Acc:HGNC:4524]

Uncharacterized protein [Source:UniProtKB/TrEMBL;Acc:G5E5C1]
Bos taurus prostaglandin-endoperoxide synthase 2 (prostaglandin G/H synthase and cyclooxygenase) (PTGS2), mRNA. [Source:RefSeq mRNA;Acc:NM_174445]
Bos taurus GIPC PDZ domain containing family, member 2 (GIPC2), mRNA. [Source:RefSeq mRNA;Acc:NM_001080279]
Bos taurus ribulose-5-phosphate-3-epimerase (RPE), mRNA. [Source:RefSeq mRNA;Acc:NM_001098079]
Bos taurus cAMP responsive element modulator (CREM), mRNA. [Source:RefSeq mRNA;Acc:NM_001034710]
metastasis associated in colon cancer 1 [Source:HGNC Symbol;Acc:HGNC:30215]
UDP glucuronosyltransferase 2 family, polypeptide A3 [Source:HGNC Symbol;Acc:HGNC:28528]

Bos taurus insulin induced gene 1 (INSIG1), mRNA. [Source:RefSeq mRNA;Acc:NM_001077909]
Bos taurus ATG12 autophagy related 12 homolog (S. cerevisiae) (ATG12), mRNA. [Source:RefSeq mRNA;Acc:NM_001076982]
Bos taurus Kruppel-like factor 10 (KLF10), mRNA. [Source:RefSeq mRNA;Acc:NM_001168462]
RAN binding protein 6 [Source:HGNC Symbol;Acc:HGNC:9851]
zinc fingers and homeoboxes 3 [Source:HGNC Symbol;Acc:HGNC:15935]
Bos taurus H3 histone, family 3A (H3F3A), mRNA. [Source:RefSeq mRNA;Acc:NM_001014389]
Olfactory receptor [Source:UniProtKB/TrEMBL;Acc:F1MLM1]
Bos taurus EF-hand domain family, member D1 (EFHD1), mRNA. [Source:RefSeq mRNA;Acc:NM_001075832]
Bos taurus DIRAS family, GTP-binding RAS-like 2 (DIRAS2), mRNA. [Source:RefSeq mRNA;Acc:NM_001192064]
Bos taurus cytochrome c, somatic (CYCS), mRNA. [Source:RefSeq mRNA;Acc:NM_001046061]
leucine-rich single-pass membrane protein 1 [Source:HGNC Symbol;Acc:HGNC:22036]
methyltransferase like 6 [Source:HGNC Symbol;Acc:HGNC:28343]
Bos taurus POU class 2 associating factor 1 (POU2AF1), mRNA. [Source:RefSeq mRNA;Acc:NM_001075915]
Olfactory receptor [Source:UniProtKB/TrEMBL;Acc:G3X8G3]
BR serine/threonine kinase 1 [Source:HGNC Symbol;Acc:HGNC:18994]
Bos taurus C-reactive protein, pentraxin-related (CRP), mRNA. [Source:RefSeq mRNA;Acc:NM_001144097]
Bos taurus ADP-ribosylation factor 1 (ARF1), mRNA. [Source:RefSeq mRNA;Acc:NM_176653]
Bos taurus zinc finger protein 350 (ZNF350), mRNA. [Source:RefSeq mRNA;Acc:NM_001075708]
Uncharacterized protein [Source:UniProtKB/TrEMBL;Acc:G3N2D3]
Bos taurus kelch repeat and BTB (POZ) domain containing 2 (KBTBD2), mRNA. [Source:RefSeq mRNA;Acc:NM_001102013]
Bos taurus TRAF3 interacting protein 2 (TRAF3IP2), mRNA. [Source:RefSeq mRNA;Acc:NM_001035483]

Bos taurus leucine rich repeat containing 8 family, member D (LRRC8D), mRNA. [Source:RefSeq mRNA;Acc:NM_001083741]
Bos taurus asparagine-linked glycosylation 2, alpha-1,3-mannosyltransferase homolog (S. cerevisiae) (ALG2), mRNA. [Source:RefSeq mRNA;Acc:NM_001083491
Bos taurus solute carrier family 16, member 1 (monocarboxylic acid transporter 1) (SLC16A1), mRNA. [Source:RefSeq mRNA;Acc:NM_001037319]
olfactory receptor, family 2, subfamily A, member 5 [Source:HGNC Symbol;Acc:HGNC:8232]
Bos taurus nuclear cap binding protein subunit 2, 20kDa (NCBP2), mRNA. [Source:RefSeq mRNA;Acc:NM_001034396]
Olfactory receptor [Source:UniProtKB/TrEMBL;Acc:G5E5X6]
Bos taurus defects in morphology 1 homolog (S. cerevisiae) (EXO5), mRNA. [Source:RefSeq mRNA;Acc:NM_001081608]
Bos taurus tripartite motif containing 38 (TRIM38), mRNA. [Source:RefSeq mRNA;Acc:NM_001034574]

Bos taurus adenosine A2b receptor (ADORA2B), mRNA. [Source:RefSeq mRNA;Acc:NM_001075925]
Bos taurus methyltransferase like 5 (METTL5), mRNA. [Source:RefSeq mRNA;Acc:NM_001099135]
Bos taurus recombination activating gene 2 (RAG2), mRNA. [Source:RefSeq mRNA;Acc:NM_001082469]
sprouty-related, EVH1 domain containing 1 [Source:HGNC Symbol;Acc:HGNC:20249]
Bos taurus tripartite motif family-like 1 (TRIML1), mRNA. [Source:RefSeq mRNA;Acc:NM_001205868]
Bos taurus vacuolar protein sorting 24 homolog (S. cerevisiae) (CHMP3), mRNA. [Source:RefSeq mRNA;Acc:NM_001035051]
Olfactory receptor [Source:UniProtKB/TrEMBL;Acc:F1MPD7]
Bos taurus hydroxysteroid (17-beta) dehydrogenase 13 (HSD17B13), mRNA. [Source:RefSeq mRNA;Acc:NM_001046616]
cryptochrome-1 [Source:RefSeq peptide;Acc:NP_001098885]
Bos taurus chromosome 8 open reading frame, human C9orf64 (C8H9orf64), mRNA. [Source:RefSeq mRNA;Acc:NM_001076978]
Bos taurus metallo-beta-lactamase domain containing 2 (MBLAC2), mRNA. [Source:RefSeq mRNA;Acc:NM_001099079]
short coiled-coil protein [Source:HGNC Symbol;Acc:HGNC:20335]
Olfactory receptor [Source:UniProtKB/TrEMBL;Acc:G3N003]
Bos taurus ferrochelatase (FECH), mRNA. [Source:RefSeq mRNA;Acc:NM_174054]
Olfactory receptor [Source:UniProtKB/TrEMBL;Acc:G3MY88]
formiminotransferase cyclodeaminase N-terminal like [Source:HGNC Symbol;Acc:HGNC:48661]
family with sequence similarity 26, member F [Source:HGNC Symbol;Acc:HGNC:33391]
Uncharacterized protein [Source:UniProtKB/TrEMBL;Acc:E1BCT0]

microtubule-associated protein 2 [Source:RefSeq peptide;Acc:NP_001192736]
transmembrane protein 104 [Source:HGNC Symbol;Acc:HGNC:25984]

protease, serine, 46 [Source:HGNC Symbol;Acc:HGNC:37325]
Bos taurus IKAROS family zinc finger 5 (Pegasus) (IKZF5), mRNA. [Source:RefSeq mRNA;Acc:NM_001083713]
tet methylcytosine dioxygenase 2 [Source:HGNC Symbol;Acc:HGNC:25941]
Bos taurus potassium channel, subfamily K, member 17 (KCNK17), mRNA. [Source:RefSeq mRNA;Acc:NM_174558]
Bos taurus TBC1 domain family, member 7 (TBC1D7), mRNA. [Source:RefSeq mRNA;Acc:NM_001015643]
Uncharacterized protein [Source:UniProtKB/TrEMBL;Acc:F1MKH4]
Bos taurus olfactory receptor, family 52, subfamily J, member 3-like (LOC788703), mRNA. [Source:RefSeq mRNA;Acc:NM_001206686]
Uncharacterized protein [Source:UniProtKB/TrEMBL;Acc:G5E6E0]

TSC22 domain family, member 1 [Source:HGNC Symbol;Acc:HGNC:16826]
galactosidase, alpha [Source:HGNC Symbol;Acc:HGNC:4296]
Bos taurus breast cancer metastasis-suppressor 1-like (BRMS1L), mRNA. [Source:RefSeq mRNA;Acc:NM_001083428]
Bos taurus KDEL (Lys-Asp-Glu-Leu) endoplasmic reticulum protein retention receptor 2 (KDELR2), mRNA. [Source:RefSeq mRNA;Acc:NM_001079779]
olfactory receptor, family 2, subfamily B, member 11 [Source:HGNC Symbol;Acc:HGNC:31249]
von Willebrand factor A domain containing 2 [Source:HGNC Symbol;Acc:HGNC:24709]
Bos taurus serpin peptidase inhibitor, clade H (heat shock protein 47), member 1, (collagen binding protein 1) (SERPINH1), mRNA. [Source:RefSeq mRNA;Acc:NI

Olfactory receptor [Source:UniProtKB/TrEMBL;Acc:G5E6H8]
NIP7, nucleolar pre-rRNA processing protein [Source:HGNC Symbol;Acc:HGNC:24328]
Bos taurus olfactory receptor 701-like (LOC788572), mRNA. [Source:RefSeq mRNA;Acc:NM_001206773]
Bos taurus toll-like receptor 2 (TLR2), mRNA. [Source:RefSeq mRNA;Acc:NM_174197]
mitogen-activated protein kinase 6 [Source:HGNC Symbol;Acc:HGNC:6879]
Bos taurus chemokine (C-C motif) receptor 2 (CCR2), mRNA. [Source:RefSeq mRNA;Acc:NM_001194959]
Bos taurus adrenergic, alpha-1A-, receptor (ADRA1A), mRNA. [Source:RefSeq mRNA;Acc:NM_174498]
Uncharacterized protein [Source:UniProtKB/TrEMBL;Acc:G5E5J4]

uracil phosphoribosyltransferase (FUR1) homolog (S. cerevisiae) [Source:HGNC Symbol;Acc:HGNC:28334]
Bos taurus S100P binding protein (S100PBP), mRNA. [Source:RefSeq mRNA;Acc:NM_001034687]
protocadherin 18 [Source:HGNC Symbol;Acc:HGNC:14268]
chromosome 6 open reading frame 136 [Source:HGNC Symbol;Acc:HGNC:21301]
Bos taurus carbohydrate sulfotransferase 10 (CHST10), mRNA. [Source:RefSeq mRNA;Acc:NM_001098011]
Forkhead box protein O1 [Source:UniProtKB/Swiss-Prot;Acc:E1BPQ1]
Bos taurus cappuccino homolog (mouse) (CNO), mRNA. [Source:RefSeq mRNA;Acc:NM_001080731]
Bos taurus poly(rC) binding protein 4 (PCBP4), mRNA. [Source:RefSeq mRNA;Acc:NM_001075283]
Bos taurus SET domain containing (lysine methyltransferase) 8 (SETD8), mRNA. [Source:RefSeq mRNA;Acc:NM_001046330]
Olfactory receptor [Source:UniProtKB/TrEMBL;Acc:G3MYJ6]
Bos taurus MAD2 mitotic arrest deficient-like 1 (yeast) (MAD2L1), mRNA. [Source:RefSeq mRNA;Acc:NM_001191513]
Bos taurus programmed cell death 6 (PDCD6), mRNA. [Source:RefSeq mRNA;Acc:NM_001105497]
Bos taurus secretory carrier membrane protein 2 (SCAMP2), mRNA. [Source:RefSeq mRNA;Acc:NM_001102170]

Bos taurus chromosome 4 open reading frame, human C7orf62 (C4H7orf62), mRNA. [Source:RefSeq mRNA;Acc:NM_001205417]
Uncharacterized protein [Source:UniProtKB/TrEMBL;Acc:F1MQY7]

Bos taurus leucine rich repeat containing 10 (LRRC10), mRNA. [Source:RefSeq mRNA;Acc:NM_001075757]

Olfactory receptor [Source:UniProtKB/TrEMBL;Acc:F1MC97]
Bos taurus myelin protein zero-like 1 (MPZL1), mRNA. [Source:RefSeq mRNA;Acc:NM_001076158]
Uncharacterized protein [Source:UniProtKB/TrEMBL;Acc:F1MFK0]
Bos taurus guanine nucleotide binding protein (G protein), beta 5 (GNB5), mRNA. [Source:RefSeq mRNA;Acc:NM_001099070]
Bos taurus serine/arginine-rich splicing factor 1 (SRSF1), mRNA. [Source:RefSeq mRNA;Acc:NM_001076394]
TGF-beta activated kinase 1/MAP3K7 binding protein 2 [Source:HGNC Symbol;Acc:HGNC:17075]
Olfactory receptor [Source:UniProtKB/TrEMBL;Acc:G5E603]
somatostatin receptor 2 [Source:HGNC Symbol;Acc:HGNC:11331]
Uncharacterized protein [Source:UniProtKB/TrEMBL;Acc:G3MYV5]
Bos taurus MTERF domain containing 2 (MTERFD2), mRNA. [Source:RefSeq mRNA;Acc:NM_001244226]
Uncharacterized protein [Source:UniProtKB/TrEMBL;Acc:G3MZN8]
Bos taurus gap junction protein, beta 1, 32kDa (GJB1), mRNA. [Source:RefSeq mRNA;Acc:NM_174069]
Bos taurus 5-hydroxytryptamine (serotonin) receptor 2B (HTR2B), mRNA. [Source:RefSeq mRNA;Acc:NM_001205448]
Bos taurus F-box protein 3 (FBXO3), mRNA. [Source:RefSeq mRNA;Acc:NM_001099062]
Uncharacterized protein [Source:UniProtKB/TrEMBL;Acc:G3N0V4]
Bos taurus lysosomal-associated membrane protein 1 (LAMP1), mRNA. [Source:RefSeq mRNA;Acc:NM_001075124]
ligase IV, DNA, ATP-dependent [Source:HGNC Symbol;Acc:HGNC:6601]
Olfactory receptor [Source:UniProtKB/TrEMBL;Acc:G3MY66]
olfactory receptor, family 52, subfamily B, member 2 [Source:HGNC Symbol;Acc:HGNC:15207]
Uncharacterized protein [Source:UniProtKB/TrEMBL;Acc:E1B9E7]

ribosomal protein S6 kinase, 90kDa, polypeptide 2 [Source:HGNC Symbol;Acc:HGNC:10431]
Bos taurus LUC7-like 3 (S. cerevisiae) (LUC7L3), mRNA. [Source:RefSeq mRNA;Acc:NM_001034684]
Bos taurus SEC11 homolog A (S. cerevisiae) (SEC11A), mRNA. [Source:RefSeq mRNA;Acc:NM_174465]
Bos taurus coenzyme Q9 homolog (S. cerevisiae) (COQ9), mRNA. [Source:RefSeq mRNA;Acc:NM_001046302]

Bos taurus melanocortin 2 receptor accessory protein 2 (MRAP2), mRNA. [Source:RefSeq mRNA;Acc:NM_001099393]
Uncharacterized protein [Source:UniProtKB/TrEMBL;Acc:F1MV71]
F-box and WD repeat domain containing 7, E3 ubiquitin protein ligase [Source:HGNC Symbol;Acc:HGNC:16712]
Olfactory receptor [Source:UniProtKB/TrEMBL;Acc:F1N253]
Bos taurus THAP domain containing 6 (THAP6), mRNA. [Source:RefSeq mRNA;Acc:NM_001103290]
Uncharacterized protein [Source:UniProtKB/TrEMBL;Acc:F1N1H6]
olfactory receptor, family 6, subfamily Y, member 1 [Source:HGNC Symbol;Acc:HGNC:14823]
Bos taurus peptidase inhibitor 16 (PI16), mRNA. [Source:RefSeq mRNA;Acc:NM_001024487]
Bos taurus uncharacterized LOC782781 (LOC782781), mRNA. [Source:RefSeq mRNA;Acc:NM_001168605]
zinc finger E-box binding homeobox 1 [Source:HGNC Symbol;Acc:HGNC:11642]
Bos taurus ring finger protein 135 (RNF135), mRNA. [Source:RefSeq mRNA;Acc:NM_001098936]
tropomodulin 2 (neuronal) [Source:HGNC Symbol;Acc:HGNC:11872]
Uncharacterized protein [Source:UniProtKB/TrEMBL;Acc:F1MYC4]
Bos taurus procollagen C-endopeptidase enhancer 2 (PCOLCE2), mRNA. [Source:RefSeq mRNA;Acc:NM_001075629]

Bos taurus secreted and transmembrane protein 1A (SECTM1A), mRNA. [Source:RefSeq mRNA;Acc:NM_001076529]
retinoblastoma binding protein 9 [Source:HGNC Symbol;Acc:HGNC:9892]
Bos taurus 5'-nucleotidase, ecto (CD73) (NT5E), mRNA. [Source:RefSeq mRNA;Acc:NM_174129]
zinc finger protein 114 [Source:HGNC Symbol;Acc:HGNC:12894]
centrosomal protein 57kDa [Source:HGNC Symbol;Acc:HGNC:30794]
olfactory receptor, family 2, subfamily K, member 2 [Source:HGNC Symbol;Acc:HGNC:8264]
signal recognition particle 72kDa [Source:HGNC Symbol;Acc:HGNC:11303]
Bos taurus interleukin-1 receptor-associated kinase 1 binding protein 1 (IRAK1BP1), mRNA. [Source:RefSeq mRNA;Acc:NM_001110093]
HIG1 domain family, member 1D; Uncharacterized protein [Source:UniProtKB/TrEMBL;Acc:Q1LZ70]
solute carrier family 9, subfamily A (NHE2, cation proton antiporter 2), member 2 [Source:HGNC Symbol;Acc:HGNC:11072]
Bos taurus chemokine (C-C motif) ligand 8 (CCL8), mRNA. [Source:RefSeq mRNA;Acc:NM_174007]
adenosine deaminase, RNA-specific, B1 [Source:HGNC Symbol;Acc:HGNC:226]
glucosaminyl (N-acetyl) transferase family member 7 [Source:HGNC Symbol;Acc:HGNC:16099]
Bos taurus pyridoxamine 5'-phosphate oxidase (PNPO), mRNA. [Source:RefSeq mRNA;Acc:NM_001014907]
Bos taurus uncharacterized LOC512219 (MGC138914), mRNA. [Source:RefSeq mRNA;Acc:NM_001083673]

Uncharacterized protein [Source:UniProtKB/TrEMBL;Acc:E1BPI2]
transmembrane protein 71 [Source:HGNC Symbol;Acc:HGNC:26572]
Bos taurus poly(rC) binding protein 1 (PCBP1), mRNA. [Source:RefSeq mRNA;Acc:NM_001015565]
Bos taurus suppressor of variegation 3-9 homolog 2 (Drosophila) (SUV39H2), mRNA. [Source:RefSeq mRNA;Acc:NM_001037479]
Bos taurus complement factor I (CFI), mRNA. [Source:RefSeq mRNA;Acc:NM_001038096]
Bos taurus LYR motif containing 5 (LYRM5), mRNA. [Source:RefSeq mRNA;Acc:NM_001114860]
Bos taurus ubiquinol-cytochrome c reductase, Rieske iron-sulfur polypeptide 1 (UQCRFS1), mRNA. [Source:RefSeq mRNA;Acc:NM_174813]
chromosome 2 open reading frame 47 [Source:HGNC Symbol;Acc:HGNC:26198]
zinc finger, BED-type containing 6 [Source:HGNC Symbol;Acc:HGNC:33273]
Bos taurus acetyl-CoA acetyltransferase 2 (ACAT2), mRNA. [Source:RefSeq mRNA;Acc:NM_001075549]
olfactory receptor, family 6, subfamily C, member 2 [Source:HGNC Symbol;Acc:HGNC:15436]
Bos taurus centromere protein A (CENPA), mRNA. [Source:RefSeq mRNA;Acc:NM_001205380]
Bos taurus chromosome 14 open reading frame, human C8orf47 (C14H8orf47), mRNA. [Source:RefSeq mRNA;Acc:NM_001077844]

Bos taurus cyclin Y (CCNY), mRNA. [Source:RefSeq mRNA;Acc:NM_001102268]
Bos taurus multiple inositol-polyphosphate phosphatase 1 (MINPP1), mRNA. [Source:RefSeq mRNA;Acc:NM_001038575]
Olfactory receptor [Source:UniProtKB/TrEMBL;Acc:G3MXL4]
Bos taurus UBA domain containing 2 (UBAC2), mRNA. [Source:RefSeq mRNA;Acc:NM_001103344]
prodynorphin [Source:HGNC Symbol;Acc:HGNC:8820]
topoisomerase I binding, arginine/serine-rich, E3 ubiquitin protein ligase [Source:HGNC Symbol;Acc:HGNC:21653]
Bos taurus c-fos induced growth factor (vascular endothelial growth factor D) (FIGF), mRNA. [Source:RefSeq mRNA;Acc:NM_001101043]
Bos taurus carbohydrate (N-acetylgalactosamine 4-0) sulfotransferase 9 (CHST9), mRNA. [Source:RefSeq mRNA;Acc:NM_001075879]

Uncharacterized protein [Source:UniProtKB/TrEMBL;Acc:F1MTR6]
Bos taurus peptidase (mitochondrial processing) beta (PMPCB), mRNA. [Source:RefSeq mRNA;Acc:NM_001034613]
Uncharacterized protein [Source:UniProtKB/TrEMBL;Acc:G3MZT7]
oxoglutarate (alpha-ketoglutarate) receptor 1 [Source:HGNC Symbol;Acc:HGNC:4531]
hormonally up-regulated Neu-associated kinase [Source:HGNC Symbol;Acc:HGNC:13326]
Bos taurus retinoic acid early transcript 1G (RAET1G), mRNA. [Source:RefSeq mRNA;Acc:NM_205785]
olfactory receptor, family 4, subfamily S, member 2 [Source:HGNC Symbol;Acc:HGNC:15183]

Bos taurus phosducin (PDC), mRNA. [Source:RefSeq mRNA;Acc:NM_001166527]
Uncharacterized protein [Source:UniProtKB/TrEMBL;Acc:G3X8A8]
Regulator of G-protein signaling 4 [Source:UniProtKB/Swiss-Prot;Acc:Q29RM9]
olfactory receptor, family 6, subfamily N, member 2 [Source:HGNC Symbol;Acc:HGNC:15035]
Bos taurus proteasome (prosome, macropain) assembly chaperone 2 (PSMG2), mRNA. [Source:RefSeq mRNA;Acc:NM_001046365]
Bos taurus ubiquilin 3 (UBQLN3), mRNA. [Source:RefSeq mRNA;Acc:NM_001038583]
A kinase (PRKA) anchor protein 17A [Source:HGNC Symbol;Acc:HGNC:18783]
SLX4 interacting protein [Source:HGNC Symbol;Acc:HGNC:16225]
Uncharacterized protein [Source:UniProtKB/TrEMBL;Acc:F1MKE7]
Bos taurus cyclin H (CCNH), mRNA. [Source:RefSeq mRNA;Acc:NM_001079788]
PWWP domain containing 2A [Source:HGNC Symbol;Acc:HGNC:29406]

Bos taurus plasma glutamate carboxypeptidase (CPQ), mRNA. [Source:RefSeq mRNA;Acc:NM_001076248]
Bos taurus serpin peptidase inhibitor, clade E (nexin, plasminogen activator inhibitor type 1), member 3 (SERPINE3), mRNA. [Source:RefSeq mRNA;Acc:NM_001
Uncharacterized protein [Source:UniProtKB/TrEMBL;Acc:G5E5C9]
Olfactory receptor [Source:UniProtKB/TrEMBL;Acc:F1MRC1]
Bos taurus bone morphogenetic protein 15 (BMP15), mRNA. [Source:RefSeq mRNA;Acc:NM_001031752]
Bos taurus microtubule-associated protein 1B (MAP1B), mRNA. [Source:RefSeq mRNA;Acc:NM_001206119]

phospholipase C, eta 1 [Source:HGNC Symbol;Acc:HGNC:29185]
protocadherin alpha 5 [Source:HGNC Symbol;Acc:HGNC:8671]
nephrosis 2, idiopathic, steroid-resistant (podocin) [Source:HGNC Symbol;Acc:HGNC:13394]
Bos taurus pancreatic polypeptide receptor 1 (PPYR1), mRNA. [Source:RefSeq mRNA;Acc:NM_001205579]
Bos taurus DnaJ (Hsp40) homolog, subfamily C , member 25 (DNAJC25), mRNA. [Source:RefSeq mRNA;Acc:NM_001204995]
Bos taurus RAB10, member RAS oncogene family (RAB10), mRNA. [Source:RefSeq mRNA;Acc:NM_001101288]
Bos taurus ubiquitin-conjugating enzyme E2 variant 1 (UBE2V1), mRNA. [Source:RefSeq mRNA;Acc:NM_001035497]
Bos taurus G protein-coupled receptor 68 (GPR68), mRNA. [Source:RefSeq mRNA;Acc:NM_174329]
Bos taurus chromosome 18 open reading frame, human C19orf12 (C18H19orf12), mRNA. [Source:RefSeq mRNA;Acc:NM_001076892]
G protein-coupled receptor 1 [Source:HGNC Symbol;Acc:HGNC:4463]
Bos taurus prolactin-related protein VIII (PRP8), mRNA. [Source:RefSeq mRNA;Acc:NM_001038200]

Olfactory receptor [Source:UniProtKB/TrEMBL;Acc:E1BD18]
olfactory receptor, family 51, subfamily Q, member 1 (gene/pseudogene) [Source:HGNC Symbol;Acc:HGNC:14851]
AHNAK nucleoprotein [Source:HGNC Symbol;Acc:HGNC:347]
Bos taurus MAK16 homolog (S. cerevisiae) (MAK16), mRNA. [Source:RefSeq mRNA;Acc:NM_001075170]
Bos taurus MyoD family inhibitor domain containing (MDFIC), mRNA. [Source:RefSeq mRNA;Acc:NM_001101102]
Olfactory receptor [Source:UniProtKB/TrEMBL;Acc:G3N185]
forkhead box I1 [Source:HGNC Symbol;Acc:HGNC:3815]
Olfactory receptor [Source:UniProtKB/TrEMBL;Acc:G3X7M6]
Uncharacterized protein [Source:UniProtKB/TrEMBL;Acc:M0QVY2]
Bos taurus chromosome 9 open reading frame, human C6orf211 (C9H6orf211), mRNA. [Source:RefSeq mRNA;Acc:NM_001083499]
centrobin, centrosomal BRCA2 interacting protein [Source:HGNC Symbol;Acc:HGNC:29616]
olfactory receptor, family 13, subfamily C, member 8 [Source:HGNC Symbol;Acc:HGNC:15103]
Bos taurus inducible T-cell co-stimulator (ICOS), mRNA. [Source:RefSeq mRNA;Acc:NM_001034275]
Bos taurus RB-associated KRAB zinc finger (RBAK), mRNA. [Source:RefSeq mRNA;Acc:NM_001191256]
RNA binding motif protein 46 [Source:HGNC Symbol;Acc:HGNC:28401]
Bos taurus polymerase (DNA directed), epsilon 3 (p17 subunit) (POLE3), mRNA. [Source:RefSeq mRNA;Acc:NM_001034390]

Bos taurus neuritin 1 (NRN1), mRNA. [Source:RefSeq mRNA;Acc:NM_001046438]
Olfactory receptor [Source:UniProtKB/TrEMBL;Acc:F1N1E8]
Uncharacterized protein [Source:UniProtKB/TrEMBL;Acc:G5E5I5]
Bos taurus homeobox B9 (HOXB9), mRNA. [Source:RefSeq mRNA;Acc:NM_001191186]
Uncharacterized protein [Source:UniProtKB/TrEMBL;Acc:G3MXH6]
Olfactory receptor [Source:UniProtKB/TrEMBL;Acc:G3MZD9]
olfactory receptor, family 6, subfamily N, member 1 [Source:HGNC Symbol;Acc:HGNC:15034]
dual-specificity tyrosine-(Y)-phosphorylation regulated kinase 2 [Source:HGNC Symbol;Acc:HGNC:3093]
UDP-GlcNAc:betaGal beta-1,3-N-acetylglucosaminyltransferase 5 [Source:HGNC Symbol;Acc:HGNC:15684]
Bos taurus adiponectin receptor 2 (ADIPOR2), mRNA. [Source:RefSeq mRNA;Acc:NM_001040499]
Bos taurus small nuclear ribonucleoprotein polypeptide A' (SNRPA1), mRNA. [Source:RefSeq mRNA;Acc:NM_001098948]
fucosyltransferase 11 (alpha (1,3) fucosyltransferase) [Source:HGNC Symbol;Acc:HGNC:19233]
olfactory receptor, family 4, subfamily L, member 1 [Source:HGNC Symbol;Acc:HGNC:15356]

olfactory receptor, family 4, subfamily E, member 2 [Source:HGNC Symbol;Acc:HGNC:8297]
Bos taurus amylase, alpha 2B (pancreatic) (AMY2B), mRNA. [Source:RefSeq mRNA;Acc:NM_001035016]
olfactory receptor, family 51, subfamily M, member 1 [Source:HGNC Symbol;Acc:HGNC:14847]
Olfactory receptor [Source:UniProtKB/TrEMBL;Acc:F1MV48]
Olfactory receptor [Source:UniProtKB/TrEMBL;Acc:G3N0A2]
solute carrier family 8 (sodium/calcium exchanger), member 3 [Source:HGNC Symbol;Acc:HGNC:11070]
cannabinoid receptor 1 (brain) [Source:HGNC Symbol;Acc:HGNC:2159]
Uncharacterized protein [Source:UniProtKB/TrEMBL;Acc:F1N0F0]
G protein regulated inducer of neurite outgrowth 1 [Source:HGNC Symbol;Acc:HGNC:24835]

Bos taurus STEAP family member 2, metalloreductase (STEAP2), mRNA. [Source:RefSeq mRNA;Acc:NM_001077847]
V-set and transmembrane domain containing 2A [Source:HGNC Symbol;Acc:HGNC:28499]
guanine nucleotide binding protein (G protein), alpha activating activity polypeptide, olfactory type [Source:HGNC Symbol;Acc:HGNC:4388]
Olfactory receptor [Source:UniProtKB/TrEMBL;Acc:F1MKY5]
olfactory receptor, family 52, subfamily N, member 1 [Source:HGNC Symbol;Acc:HGNC:14853]
Bos taurus pituitary tumor-transforming 1 (PTTG1), mRNA. [Source:RefSeq mRNA;Acc:NM_001034310]
Bos taurus zinc finger protein 2 homolog (mouse) (ZFP2), mRNA. [Source:RefSeq mRNA;Acc:NM_001098146]
Bos taurus WD repeat domain 91 (WDR91), mRNA. [Source:RefSeq mRNA;Acc:NM_001046483]
Olfactory receptor [Source:UniProtKB/TrEMBL;Acc:G3MZ39]
SECIS binding protein 2 [Source:HGNC Symbol;Acc:HGNC:30972]
Uncharacterized protein [Source:UniProtKB/TrEMBL;Acc:G3X7X1]
Olfactory receptor [Source:UniProtKB/TrEMBL;Acc:F1MNC8]
Uncharacterized protein [Source:UniProtKB/TrEMBL;Acc:F1N075]
Bos taurus leucine rich repeat containing 18 (LRRC18), mRNA. [Source:RefSeq mRNA;Acc:NM_001077058]
Hyaluronidase [Source:UniProtKB/TrEMBL;Acc:E1BBU8]
solute carrier family 12 (potassium/chloride transporter), member 7 [Source:HGNC Symbol;Acc:HGNC:10915]
Bos taurus collagen, type VIII, alpha 1 (COL8A1), mRNA. [Source:RefSeq mRNA;Acc:NM_001101176]
Bos taurus Y box binding protein 1 (YBX1), mRNA. [Source:RefSeq mRNA;Acc:NM_174815]
transglutaminase 6 [Source:HGNC Symbol;Acc:HGNC:16255]

Bos taurus G protein-coupled receptor 182 (GPR182), mRNA. [Source:RefSeq mRNA;Acc:NM_001206417]
Uncharacterized protein [Source:UniProtKB/TrEMBL;Acc:G3N109]

Bos taurus histidine-rich glycoprotein (HRG), mRNA. [Source:RefSeq mRNA;Acc:NM_173919]
Uncharacterized protein [Source:UniProtKB/TrEMBL;Acc:G3MYS0]
Uncharacterized protein [Source:UniProtKB/TrEMBL;Acc:F1MPQ4]
Bos taurus progesterone receptor membrane component 2 (PGRMC2), mRNA. [Source:RefSeq mRNA;Acc:NM_001099060]
olfactory receptor, family 52, subfamily E, member 2 [Source:HGNC Symbol;Acc:HGNC:14769]
Olfactory receptor [Source:UniProtKB/TrEMBL;Acc:E1BBA4]
Bos taurus peptidase D (PEPD), mRNA. [Source:RefSeq mRNA;Acc:NM_001080318]
mucin 12, cell surface associated [Source:HGNC Symbol;Acc:HGNC:7510]
Bos taurus zinc finger protein 2 (ZNF2), mRNA. [Source:RefSeq mRNA;Acc:NM_001046446]
Olfactory receptor [Source:UniProtKB/TrEMBL;Acc:E1BBY4]
transient receptor potential cation channel, subfamily V, member 6 [Source:HGNC Symbol;Acc:HGNC:14006]

NFKB repressing factor [Source:HGNC Symbol;Acc:HGNC:19374]
olfactory receptor, family 1, subfamily J, member 2 [Source:HGNC Symbol;Acc:HGNC:8209]
Bos taurus transketolase (TKT), mRNA. [Source:RefSeq mRNA;Acc:NM_001003906]
dentin sialophosphoprotein [Source:HGNC Symbol;Acc:HGNC:3054]
Bos taurus keratin associated protein 13-1 (KRTAP13-1), mRNA. [Source:RefSeq mRNA;Acc:NM_001080350]
Olfactory receptor [Source:UniProtKB/TrEMBL;Acc:F1N341]
Bos taurus FK506 binding protein 4, 59kDa (FKBP4), mRNA. [Source:RefSeq mRNA;Acc:NM_001034322]
olfactory receptor, family 6, subfamily C, member 75 [Source:HGNC Symbol;Acc:HGNC:31304]
Bos taurus pygopus homolog 2 (Drosophila) (PYGO2), mRNA. [Source:RefSeq mRNA;Acc:NM_001102242]
Olfactory receptor [Source:UniProtKB/TrEMBL;Acc:G3N3Y0]
Olfactory receptor [Source:UniProtKB/TrEMBL;Acc:F1MDP9]
Olfactory receptor [Source:UniProtKB/TrEMBL;Acc:E1BF36]
Bos taurus syntaxin 18 (STX18), mRNA. [Source:RefSeq mRNA;Acc:NM_001099719]
Bos taurus ribonuclease, RNase A family, k6 (RNASE6), mRNA. [Source:RefSeq mRNA;Acc:NM_174594]
Bos taurus secreted frizzled-related protein 1 (SFRP1), mRNA. [Source:RefSeq mRNA;Acc:NM_174460]

Uncharacterized protein [Source:UniProtKB/TrEMBL;Acc:G3X6Z9]
KIAA2022 [Source:HGNC Symbol;Acc:HGNC:29433]
Bos taurus keratin 79 (KRT79), mRNA. [Source:RefSeq mRNA;Acc:NM_001075348]
NIMA-related kinase 7 [Source:HGNC Symbol;Acc:HGNC:13386]
olfactory receptor, family 4, subfamily C, member 5 [Source:HGNC Symbol;Acc:HGNC:14702]
C-type lectin domain family 4, member A [Source:HGNC Symbol;Acc:HGNC:13257]
Bos taurus isovaleryl-CoA dehydrogenase (IVD), mRNA. [Source:RefSeq mRNA;Acc:NM_001034382]
Uncharacterized protein [Source:UniProtKB/TrEMBL;Acc:G3N296]
F-box protein 30 [Source:HGNC Symbol;Acc:HGNC:15600]
Bos taurus dual specificity phosphatase 14 (DUSP14), mRNA. [Source:RefSeq mRNA;Acc:NM_001075308]
Bos taurus REST corepressor 3 (RCOR3), mRNA. [Source:RefSeq mRNA;Acc:NM_001192733]
nuclear factor of activated T-cells 5, tonicity-responsive [Source:HGNC Symbol;Acc:HGNC:7774]
armadillo repeat containing, X-linked 5 [Source:HGNC Symbol;Acc:HGNC:25772]
Olfactory receptor [Source:UniProtKB/TrEMBL;Acc:G5E6A8]
olfactory receptor, family 14, subfamily J, member 1 [Source:HGNC Symbol;Acc:HGNC:13971]
Uncharacterized protein [Source:UniProtKB/TrEMBL;Acc:G3MY54]
karyopherin alpha 5 (importin alpha 6) [Source:HGNC Symbol;Acc:HGNC:6398]

Yip1 domain family, member 4 [Source:HGNC Symbol;Acc:HGNC:28145]
Bos taurus gamma-glutamylcyclotransferase (GGCT), mRNA. [Source:RefSeq mRNA;Acc:NM_001037610]
Olfactory receptor [Source:UniProtKB/TrEMBL;Acc:G3N3D1]
Bos taurus chromosome 17 open reading frame, human C4orf46 (C17H4orf46), mRNA. [Source:RefSeq mRNA;Acc:NM_001078113]
Olfactory receptor [Source:UniProtKB/TrEMBL;Acc:F1N2W1]
Bos taurus phosphatidic acid phosphatase type 2A (PPAP2A), mRNA. [Source:RefSeq mRNA;Acc:NM_001080329]
Uncharacterized protein [Source:UniProtKB/TrEMBL;Acc:E1BBK5]
Bos taurus mitochondrial ribosomal protein L34 (MRPL34), mRNA. [Source:RefSeq mRNA;Acc:NM_001110187]
chromosome 16 open reading frame 54 [Source:HGNC Symbol;Acc:HGNC:26649]
Bos taurus transmembrane protein 51 (TMEM51), mRNA. [Source:RefSeq mRNA;Acc:NM_001098975]
Bos taurus beta-1,3-N-acetylgalactosaminyltransferase 2 (B3GALNT2), mRNA. [Source:RefSeq mRNA;Acc:NM_001103343]

Uncharacterized protein [Source:UniProtKB/TrEMBL;Acc:F1MH62]

Olfactory receptor [Source:UniProtKB/TrEMBL;Acc:G3MXI1]
Olfactory receptor [Source:UniProtKB/TrEMBL;Acc:F1MFE3]
coiled-coil domain containing 181 [Source:HGNC Symbol;Acc:HGNC:28051]
SH2 domain containing 4B [Source:HGNC Symbol;Acc:HGNC:31440]
Uncharacterized protein [Source:UniProtKB/TrEMBL;Acc:F1N7L2]
Bos taurus methyl-CpG binding domain protein 3-like 1 (MBD3L1), mRNA. [Source:RefSeq mRNA;Acc:NM_001077086]
Bos taurus hyaluronan synthase 2 (HAS2), mRNA. [Source:RefSeq mRNA;Acc:NM_174079]
Bos taurus tocopherol (alpha) transfer protein-like (TTPAL), mRNA. [Source:RefSeq mRNA;Acc:NM_001205931]
Olfactory receptor [Source:UniProtKB/TrEMBL;Acc:G3MX41]
eukaryotic translation elongation factor 1 alpha 1 [Source:HGNC Symbol;Acc:HGNC:3189]
Uncharacterized protein [Source:UniProtKB/TrEMBL;Acc:G3N283]
cleavage and polyadenylation specific factor 6, 68kDa [Source:HGNC Symbol;Acc:HGNC:13871]
Olfactory receptor [Source:UniProtKB/TrEMBL;Acc:E1BMY3]
NADH dehydrogenase subunit 1 [Source:RefSeq peptide;Acc:YP_209205]
Uncharacterized protein [Source:UniProtKB/TrEMBL;Acc:E1BHC4]
additional sex combs like transcriptional regulator 2 [Source:HGNC Symbol;Acc:HGNC:23805]
Bos taurus four and a half LIM domains 5 (FHL5), mRNA. [Source:RefSeq mRNA;Acc:NM_001076379]
Bos taurus Nanog homeobox (NANOG), mRNA. [Source:RefSeq mRNA;Acc:NM_001025344]
Olfactory receptor [Source:UniProtKB/TrEMBL;Acc:G5E656]
Bos taurus calcium modulating ligand (CAMLG), mRNA. [Source:RefSeq mRNA;Acc:NM_001037625]
olfactory receptor, family 11, subfamily H, member 6 [Source:HGNC Symbol;Acc:HGNC:15349]
Rho GTPase activating protein 44 [Source:HGNC Symbol;Acc:HGNC:29096]
Bos taurus family with sequence similarity 210, member B (FAM210B), mRNA. [Source:RefSeq mRNA;Acc:NM_001080278]

Bos taurus excision repair cross-complementing rodent repair deficiency, complementation group 4 (ERCC4), mRNA. [Source:RefSeq mRNA;Acc:NM_00120536[illegible]
Uncharacterized protein [Source:UniProtKB/TrEMBL;Acc:E1BCM6]
Bos taurus sterol O-acyltransferase 1 (SOAT1), mRNA. [Source:RefSeq mRNA;Acc:NM_001034206]

kinase suppressor of ras 1 [Source:HGNC Symbol;Acc:HGNC:6465]
chromosome alignment maintaining phosphoprotein 1 [Source:HGNC Symbol;Acc:HGNC:20311]
Bos taurus ERO1-like (S. cerevisiae) (ERO1L), mRNA. [Source:RefSeq mRNA;Acc:NM_001103348]
transforming growth factor, beta receptor associated protein 1 [Source:HGNC Symbol;Acc:HGNC:16836]
Bos taurus fibrinogen gamma chain (FGG), mRNA. [Source:RefSeq mRNA;Acc:NM_173911]
Amine oxidase [Source:UniProtKB/TrEMBL;Acc:E1BJN3]
olfactory receptor, family 2, subfamily Y, member 1 [Source:HGNC Symbol;Acc:HGNC:14837]
Bos taurus integrin, beta 2 (complement component 3 receptor 3 and 4 subunit) (ITGB2), mRNA. [Source:RefSeq mRNA;Acc:NM_175781]
exocyst complex component 5 [Source:HGNC Symbol;Acc:HGNC:10696]
Olfactory receptor [Source:UniProtKB/TrEMBL;Acc:F1N046]
Bos taurus family with sequence similarity 171, member A1 (FAM171A1), mRNA. [Source:RefSeq mRNA;Acc:NM_001102180]
Bos taurus cadherin-related family member 5 (CDHR5), mRNA. [Source:RefSeq mRNA;Acc:NM_001103326]
Bos taurus RAB3C, member RAS oncogene family (RAB3C), mRNA. [Source:RefSeq mRNA;Acc:NM_176873]
olfactomedin 3 [Source:HGNC Symbol;Acc:HGNC:17990]
Uncharacterized protein [Source:UniProtKB/TrEMBL;Acc:G3N3K8]
Bos taurus myogenic factor 5 (MYF5), mRNA. [Source:RefSeq mRNA;Acc:NM_174116]

Olfactory receptor [Source:UniProtKB/TrEMBL;Acc:G3N2Y2]
beta-1,3-N-acetylgalactosaminyltransferase 1 (globoside blood group) [Source:HGNC Symbol;Acc:HGNC:918]
Uncharacterized protein [Source:UniProtKB/TrEMBL;Acc:G3MZP4]
Uncharacterized protein [Source:UniProtKB/TrEMBL;Acc:G3N367]
Bos taurus growth arrest-specific 2 like 2 (GAS2L2), mRNA. [Source:RefSeq mRNA;Acc:NM_001205642]
Olfactory receptor [Source:UniProtKB/TrEMBL;Acc:F1N5P9]
Bos taurus UDP-N-acetyl-alpha-D-galactosamine:polypeptide N-acetylgalactosaminyltransferase-like 6 (GALNTL6), mRNA. [Source:RefSeq mRNA;Acc:NM_0011[illegible]
Bos taurus patched domain containing 1 (PTCHD1), mRNA. [Source:RefSeq mRNA;Acc:NM_001192881]
Uncharacterized protein [Source:UniProtKB/TrEMBL;Acc:F1N4U0]
zinc finger protein, FOG family member 2 [Source:HGNC Symbol;Acc:HGNC:16700]
chromosome 2 open reading frame 78 [Source:HGNC Symbol;Acc:HGNC:34349]
Bos taurus uncharacterized LOC539014 (C14H8orf76), mRNA. [Source:RefSeq mRNA;Acc:NM_001205614]
Bos taurus BCDIN3 domain containing (BCDIN3D), mRNA. [Source:RefSeq mRNA;Acc:NM_001075216]

delta/notch-like EGF repeat containing [Source:HGNC Symbol;Acc:HGNC:24456]
Uncharacterized protein [Source:UniProtKB/TrEMBL;Acc:F1MTL2]
Olfactory receptor [Source:UniProtKB/TrEMBL;Acc:E1BHR1]
Bos taurus chromosome 9 open reading frame, human C6orf203 (C9H6orf203), mRNA. [Source:RefSeq mRNA;Acc:NM_001102267]
Bos taurus nuclear speckle splicing regulatory protein 1 (NSRP1), mRNA. [Source:RefSeq mRNA;Acc:NM_001046462]
Bos taurus interferon regulatory factor 8 (IRF8), mRNA. [Source:RefSeq mRNA;Acc:NM_001083769]
Olfactory receptor [Source:UniProtKB/TrEMBL;Acc:G3MYR2]
Olfactory receptor [Source:UniProtKB/TrEMBL;Acc:G3N1D7]
Uncharacterized protein [Source:UniProtKB/TrEMBL;Acc:E1BJW9]
KIAA1919 [Source:HGNC Symbol;Acc:HGNC:21053]
Bos taurus glutamic-oxaloacetic transaminase 1-like 1 (GOT1L1), mRNA. [Source:RefSeq mRNA;Acc:NM_001038058]
Olfactory receptor [Source:UniProtKB/TrEMBL;Acc:E1BCA6]
epoxide hydrolase 3 [Source:HGNC Symbol;Acc:HGNC:23760]
Bos taurus chromosome 12 open reading frame, human C13orf30 (FAM216B), mRNA. [Source:RefSeq mRNA;Acc:NM_001076315]
Bos taurus secretogranin II (SCG2), mRNA. [Source:RefSeq mRNA;Acc:NM_174176]

RFK protein; Uncharacterized protein [Source:UniProtKB/TrEMBL;Acc:Q3SZP4]
Bos taurus polymerase (RNA) II (DNA directed) polypeptide M (POLR2M), mRNA. [Source:RefSeq mRNA;Acc:NM_001105477]
Olfactory receptor [Source:UniProtKB/TrEMBL;Acc:F1MWC9]
Bos taurus solute carrier family 5 (choline transporter), member 7 (SLC5A7), mRNA. [Source:RefSeq mRNA;Acc:NM_001046116]
influenza virus NS1A-binding protein [Source:RefSeq peptide;Acc:NP_001039649]
Olfactory receptor [Source:UniProtKB/TrEMBL;Acc:E1BHQ3]
Olfactory receptor [Source:UniProtKB/TrEMBL;Acc:M0QW42]
Bos taurus protein phosphatase 1, regulatory subunit 3A (PPP1R3A), mRNA. [Source:RefSeq mRNA;Acc:NM_001205440]
Bos taurus family with sequence similarity 162, member B (FAM162B), mRNA. [Source:RefSeq mRNA;Acc:NM_001101289]
transmembrane and ubiquitin-like domain containing 2 [Source:HGNC Symbol;Acc:HGNC:28459]
Olfactory receptor [Source:UniProtKB/TrEMBL;Acc:F1N7V9]
Bos taurus transmembrane protein 19 (TMEM19), mRNA. [Source:RefSeq mRNA;Acc:NM_001101184]
lysophosphatidic acid receptor 1 [Source:HGNC Symbol;Acc:HGNC:3166]
Olfactory receptor [Source:UniProtKB/TrEMBL;Acc:E1BGX0]

Bos taurus small membrane A-kinase anchor protein (C2H2orf88), mRNA. [Source:RefSeq mRNA;Acc:NM_001076991]
Uncharacterized protein [Source:UniProtKB/TrEMBL;Acc:F1MIP2]
Olfactory receptor [Source:UniProtKB/TrEMBL;Acc:G3N169]
Bos taurus anaphase promoting complex subunit 16 (ANAPC16), mRNA. [Source:RefSeq mRNA;Acc:NM_001014960]
Bos taurus vesicle-associated membrane protein 3 (cellubrevin) (VAMP3), mRNA. [Source:RefSeq mRNA;Acc:NM_001045915]

taste receptor, type 2, member 40 [Source:HGNC Symbol;Acc:HGNC:18885]
Olfactory receptor [Source:UniProtKB/TrEMBL;Acc:E1BPD1]
Olfactory receptor [Source:UniProtKB/TrEMBL;Acc:G5E672]
Bos taurus mitochondrial ribosomal protein L53 (MRPL53), mRNA. [Source:RefSeq mRNA;Acc:NM_001045926]
Olfactory receptor [Source:UniProtKB/TrEMBL;Acc:F1MVU3]
chromosome 21 open reading frame 140 [Source:HGNC Symbol;Acc:HGNC:39602]
Bos taurus suppressor of var1, 3-like 1 (S. cerevisiae) (SUPV3L1), mRNA. [Source:RefSeq mRNA;Acc:NM_001101862]
Uncharacterized protein [Source:UniProtKB/TrEMBL;Acc:G5E6D8]
Olfactory receptor [Source:UniProtKB/TrEMBL;Acc:G3N0G8]
olfactory receptor, family 52, subfamily E, member 5 [Source:HGNC Symbol;Acc:HGNC:15214]
Bos taurus HOP homeobox (HOPX), mRNA. [Source:RefSeq mRNA;Acc:NM_174097]
olfactory receptor, family 51, subfamily E, member 2 [Source:HGNC Symbol;Acc:HGNC:15195]
Bos taurus secreted frizzled-related protein 4 (SFRP4), mRNA. [Source:RefSeq mRNA;Acc:NM_001075764]
CTTNBP2 N-terminal like [Source:HGNC Symbol;Acc:HGNC:25330]
Bos taurus phosphoribosyl pyrophosphate synthetase 1 (PRPS1), mRNA. [Source:RefSeq mRNA;Acc:NM_001046189]
Bos taurus cancer susceptibility candidate 4 (CASC4), mRNA. [Source:RefSeq mRNA;Acc:NM_001081737]
Uncharacterized protein [Source:UniProtKB/TrEMBL;Acc:F1MP06]
survival motor neuron domain containing 1 [Source:HGNC Symbol;Acc:HGNC:16900]
Bos taurus ATPase, H+ transporting, lysosomal accessory protein 1 (ATP6AP1), mRNA. [Source:RefSeq mRNA;Acc:NM_175806]
Olfactory receptor [Source:UniProtKB/TrEMBL;Acc:F1N5Q9]
Olfactory receptor [Source:UniProtKB/TrEMBL;Acc:G3MXT7]

Bos taurus glutathione synthetase (GSS), mRNA. [Source:RefSeq mRNA;Acc:NM_001015630]

Bos taurus methionyl aminopeptidase 1 (METAP1), mRNA. [Source:RefSeq mRNA;Acc:NM_001102037]
xenotropic and polytropic retrovirus receptor 1 [Source:HGNC Symbol;Acc:HGNC:12827]
Bos taurus prion protein 2 (dublet) (PRND), mRNA. [Source:RefSeq mRNA;Acc:NM_174158]

Bos taurus SEC14 and spectrin domains 1 (SESTD1), mRNA. [Source:RefSeq mRNA;Acc:NM_001191250]
Uncharacterized protein [Source:UniProtKB/TrEMBL;Acc:E1BI89]
Bos taurus suppressor of Ty 7 (S. cerevisiae)-like (SUPT7L), mRNA. [Source:RefSeq mRNA;Acc:NM_001076150]
glutamate decarboxylase-like 1 [Source:HGNC Symbol;Acc:HGNC:27949]
Bos taurus TBC1 domain family, member 14 (TBC1D14), mRNA. [Source:RefSeq mRNA;Acc:NM_001099176]
zinc finger protein 436 [Source:HGNC Symbol;Acc:HGNC:20814]
syntabulin (syntaxin-interacting) [Source:HGNC Symbol;Acc:HGNC:26011]
Olfactory receptor [Source:UniProtKB/TrEMBL;Acc:E1BEN1]
Bos taurus neuromedin U receptor 2 (NMUR2), mRNA. [Source:RefSeq mRNA;Acc:NM_001035048]

sulfatase 1 [Source:HGNC Symbol;Acc:HGNC:20391]
interleukin 37 [Source:HGNC Symbol;Acc:HGNC:15563]
Bos taurus UBX domain protein 7 (UBXN7), mRNA. [Source:RefSeq mRNA;Acc:NM_001206764]
Olfactory receptor [Source:UniProtKB/TrEMBL;Acc:E1BP70]
Bos taurus histidine ammonia-lyase (HAL), mRNA. [Source:RefSeq mRNA;Acc:NM_001105440]
Bos taurus leucine rich transmembrane and 0-methyltransferase domain containing (LRTOMT), mRNA. [Source:RefSeq mRNA;Acc:NM_001024545]
zinc finger protein 791 [Source:HGNC Symbol;Acc:HGNC:26895]
Uncharacterized protein [Source:UniProtKB/TrEMBL;Acc:F1MCQ6]
Cyclic GMP-AMP synthase [Source:UniProtKB/Swiss-Prot;Acc:E1BGN7]
Bos taurus myostatin (MSTN), mRNA. [Source:RefSeq mRNA;Acc:NM_001001525]
Bos taurus nescient helix loop helix 2 (NHLH2), mRNA. [Source:RefSeq mRNA;Acc:NM_001110003]
olfactory receptor, family 1, subfamily Q, member 1 [Source:HGNC Symbol;Acc:HGNC:8223]
Uncharacterized protein [Source:UniProtKB/TrEMBL;Acc:E1BCE3]
Bos taurus leucine rich repeat transmembrane neuronal 2 (LRRTM2), mRNA. [Source:RefSeq mRNA;Acc:NM_001099377]

Olfactory receptor [Source:UniProtKB/TrEMBL;Acc:F1MZK3]

zinc finger protein 518A [Source:HGNC Symbol;Acc:HGNC:29009]
ribosomal protein S6 kinase, 52kDa, polypeptide 1 [Source:HGNC Symbol;Acc:HGNC:10439]
Uncharacterized protein [Source:UniProtKB/TrEMBL;Acc:F1MCA2]
Bos taurus TGFB-induced factor homeobox 1 (TGIF1), mRNA. [Source:RefSeq mRNA;Acc:NM_001128499]
Bos taurus transmembrane protein 68 (TMEM68), mRNA. [Source:RefSeq mRNA;Acc:NM_001076009]
Bos taurus phospholipase A2, group XIIA (PLA2G12A), mRNA. [Source:RefSeq mRNA;Acc:NM_001098063]
Bos taurus chromosome 4 open reading frame, human C7orf41 (C4H7orf41), mRNA. [Source:RefSeq mRNA;Acc:NM_001101246]
Uncharacterized protein [Source:UniProtKB/TrEMBL;Acc:F1ME27]
Bos taurus LIM domain only 4 (LMO4), mRNA. [Source:RefSeq mRNA;Acc:NM_001034751]
Trimethyllysine dioxygenase, mitochondrial [Source:UniProtKB/Swiss-Prot;Acc:Q0VC74]

Olfactory receptor [Source:UniProtKB/TrEMBL;Acc:F1MKN0]
zinc finger protein 493 [Source:HGNC Symbol;Acc:HGNC:23708]
zinc finger protein 814 [Source:HGNC Symbol;Acc:HGNC:33258]
Bos taurus transmembrane protein 242 (TMEM242), mRNA. [Source:RefSeq mRNA;Acc:NM_001046228]
Bos taurus protein-L-isoaspartate (D-aspartate) O-methyltransferase (PCMT1), mRNA. [Source:RefSeq mRNA;Acc:NM_001079617]
Uncharacterized protein [Source:UniProtKB/TrEMBL;Acc:F1MB05]
Bos taurus general transcription factor IIH, polypeptide 5 (GTF2H5), mRNA. [Source:RefSeq mRNA;Acc:NM_001040537]
Olfactory receptor [Source:UniProtKB/TrEMBL;Acc:G3MXW0]
Olfactory receptor [Source:UniProtKB/TrEMBL;Acc:E1BFA3]
Olfactory receptor [Source:UniProtKB/TrEMBL;Acc:G3N112]
Bos taurus ovo-like 1(Drosophila) (OVOL1), mRNA. [Source:RefSeq mRNA;Acc:NM_001081521]
Olfactory receptor [Source:UniProtKB/TrEMBL;Acc:F1MR72]
Bos taurus sclerostin domain containing 1 (SOSTDC1), mRNA. [Source:RefSeq mRNA;Acc:NM_001046265]
Olfactory receptor [Source:UniProtKB/TrEMBL;Acc:G3N0T4]
missing oocyte, meiosis regulator, homolog (Drosophila) [Source:HGNC Symbol;Acc:HGNC:21905]
Bos taurus HESX homeobox 1 (HESX1), mRNA. [Source:RefSeq mRNA;Acc:NM_001191140]
aspartoacylase [Source:HGNC Symbol;Acc:HGNC:756]
Uncharacterized protein [Source:UniProtKB/TrEMBL;Acc:F1N2T9]
Olfactory receptor [Source:UniProtKB/TrEMBL;Acc:F1N2Y0]

Olfactory receptor [Source:UniProtKB/TrEMBL;Acc:E1BBC7]
Uncharacterized protein [Source:UniProtKB/TrEMBL;Acc:G3N160]
solute carrier family 23, member 3 [Source:HGNC Symbol;Acc:HGNC:20601]
Bos taurus Crm, cramped-like (Drosophila) (CRAMP1L), mRNA. [Source:RefSeq mRNA;Acc:NM_001205874]
Bos taurus putative small membrane protein NID67 (MGC128424), mRNA. [Source:RefSeq mRNA;Acc:NM_001244119]
ankyrin repeat and EF-hand domain containing 1 [Source:HGNC Symbol;Acc:HGNC:15803]
Bos taurus glycosylation-dependent cell adhesion molecule 1 (GLYCAM1), mRNA. [Source:RefSeq mRNA;Acc:NM_174828]
Bos taurus THAP domain containing 5 (THAP5), mRNA. [Source:RefSeq mRNA;Acc:NM_001076339]
Olfactory receptor [Source:UniProtKB/TrEMBL;Acc:E1BCM5]
olfactory receptor, family 13, subfamily H, member 1 [Source:HGNC Symbol;Acc:HGNC:14755]
Olfactory receptor [Source:UniProtKB/TrEMBL;Acc:F1N0M4]
Bos taurus haloacid dehalogenase-like hydrolase domain containing 1 (HDHD1), mRNA. [Source:RefSeq mRNA;Acc:NM_001046595]
Uncharacterized protein [Source:UniProtKB/TrEMBL;Acc:F1MUY5]
Bos taurus 3-hydroxymethyl-3-methylglutaryl-CoA lyase (HMGCL), mRNA. [Source:RefSeq mRNA;Acc:NM_001075132]
Olfactory receptor [Source:UniProtKB/TrEMBL;Acc:G3X8B6]
Bos taurus olfactory receptor, family 12, subfamily D, member 2 (OR12D2), mRNA. [Source:RefSeq mRNA;Acc:NM_001098382]
Bos taurus collectin sub-family member 12 (COLEC12), mRNA. [Source:RefSeq mRNA;Acc:NM_001101843]

Bos taurus zinc finger, SWIM-type containing 7 (ZSWIM7), mRNA. [Source:RefSeq mRNA;Acc:NM_001197136]
Bos taurus coproporphyrinogen oxidase (CPOX), mRNA. [Source:RefSeq mRNA;Acc:NM_001192393]
homeobox protein Hox-A10 [Source:RefSeq peptide;Acc:NP_001098487]
Bos taurus coagulation factor II (thrombin) receptor (F2R), mRNA. [Source:RefSeq mRNA;Acc:NM_001103097]
Uncharacterized protein [Source:UniProtKB/TrEMBL;Acc:E1BA19]
Olfactory receptor [Source:UniProtKB/TrEMBL;Acc:F1MU72]
Bos taurus RAB35, member RAS oncogene family (RAB35), mRNA. [Source:RefSeq mRNA;Acc:NM_001098127]
Bos taurus membrane-associated ring finger (C3HC4) 5 (MARCH5), mRNA. [Source:RefSeq mRNA;Acc:NM_001034715]
Bos taurus G1 to S phase transition 1 (GSPT1), mRNA. [Source:RefSeq mRNA;Acc:NM_001098071]
Bos taurus prokineticin receptor 2 (PROKR2), mRNA. [Source:RefSeq mRNA;Acc:NM_174640]
Bos taurus phosphoribosylglycinamide formyltransferase, phosphoribosylglycinamide synthetase, phosphoribosylaminoimidazole synthetase (GART), mRNA. [S
catenin (cadherin-associated protein), alpha 3 [Source:HGNC Symbol;Acc:HGNC:2511]

Olfactory receptor [Source:UniProtKB/TrEMBL;Acc:F1N7L1]
Bos taurus ankyrin repeat and SOCS box containing 4 (ASB4), mRNA. [Source:RefSeq mRNA;Acc:NM_001192864]
PML-RARA regulated adaptor molecule 1 [Source:HGNC Symbol;Acc:HGNC:30091]
Bos taurus chromosome 5 open reading frame, human C22orf28 (C5H22orf28), mRNA. [Source:RefSeq mRNA;Acc:NM_001015631]
Bos taurus solute carrier family 1 (neuronal/epithelial high affinity glutamate transporter, system Xag), member 1 (SLC1A1), mRNA. [Source:RefSeq mRNA;Acc:N
Bos taurus growth differentiation factor 10 (GDF10), mRNA. [Source:RefSeq mRNA;Acc:NM_001076167]
sialic acid acetylesterase [Source:HGNC Symbol;Acc:HGNC:18187]
olfactory receptor, family 10, subfamily K, member 2 [Source:HGNC Symbol;Acc:HGNC:14826]
Bos taurus taste receptor, type 2, member 3 (TAS2R3), mRNA. [Source:RefSeq mRNA;Acc:NM_001046632]
Bos taurus fibromodulin (FMOD), mRNA. [Source:RefSeq mRNA;Acc:NM_174058]
Bos taurus RAB33B, member RAS oncogene family (RAB33B), mRNA. [Source:RefSeq mRNA;Acc:NM_001083440]
Uncharacterized protein [Source:UniProtKB/TrEMBL;Acc:G3N3N8]
Uncharacterized protein [Source:UniProtKB/TrEMBL;Acc:G5E6D7]
tudor domain containing 6 [Source:HGNC Symbol;Acc:HGNC:21339]
Olfactory receptor [Source:UniProtKB/TrEMBL;Acc:G3MXW3]
Uncharacterized protein [Source:UniProtKB/TrEMBL;Acc:G3N2A6]
Bos taurus DPH3, KTI11 homolog (S. cerevisiae) (DPH3), mRNA. [Source:RefSeq mRNA;Acc:NM_001113299]
family with sequence similarity 186, member A [Source:HGNC Symbol;Acc:HGNC:26980]
poliovirus receptor-related 3 [Source:HGNC Symbol;Acc:HGNC:17664]
Bos taurus kelch domain containing 10 (KLHDC10), mRNA. [Source:RefSeq mRNA;Acc:NM_001075227]
Olfactory receptor [Source:UniProtKB/TrEMBL;Acc:G3MX96]
POZ (BTB) and AT hook containing zinc finger 1 [Source:HGNC Symbol;Acc:HGNC:13071]
Uncharacterized protein [Source:UniProtKB/TrEMBL;Acc:G3MY91]
myeloid leukemia factor 2 [Source:HGNC Symbol;Acc:HGNC:7126]
Olfactory receptor [Source:UniProtKB/TrEMBL;Acc:G3N2V6]
G protein-coupled receptor 63 [Source:HGNC Symbol;Acc:HGNC:13302]
keratinocyte proline-rich protein [Source:HGNC Symbol;Acc:HGNC:31823]
Olfactory receptor [Source:UniProtKB/TrEMBL;Acc:E1B700]
Bos taurus ADP-ribosylation factor GTPase activating protein 3 (ARFGAP3), mRNA. [Source:RefSeq mRNA;Acc:NM_001075974]
toll-like receptor 6 [Source:HGNC Symbol;Acc:HGNC:16711]

Bos taurus PDZ binding kinase (PBK), mRNA. [Source:RefSeq mRNA;Acc:NM_001102178]
ankyrin repeat and BTB (POZ) domain containing 2 [Source:HGNC Symbol;Acc:HGNC:23842]
general transcription factor IIIC, polypeptide 6, alpha 35kDa [Source:HGNC Symbol;Acc:HGNC:20872]
Uncharacterized protein [Source:UniProtKB/TrEMBL;Acc:G3N2H9]
Olfactory receptor [Source:UniProtKB/TrEMBL;Acc:E1B7B2]
Olfactory receptor [Source:UniProtKB/TrEMBL;Acc:G3MWT3]
Bos taurus basic transcription factor 3-like 4 (BTF3L4), mRNA. [Source:RefSeq mRNA;Acc:NM_001098106]
syntrophin, gamma 1 [Source:HGNC Symbol;Acc:HGNC:13740]
RNA polymerase II subunit A C-terminal domain phosphatase SSU72 [Source:UniProtKB/Swiss-Prot;Acc:Q17QI2]
Bos taurus inhibitor of DNA binding 2, dominant negative helix-loop-helix protein (ID2), mRNA. [Source:RefSeq mRNA;Acc:NM_001034231]
Bos taurus calmodulin 1 (phosphorylase kinase, delta) (CALM1), mRNA. [Source:RefSeq mRNA;Acc:NM_001242572]
olfactory receptor, family 6, subfamily M, member 1 [Source:HGNC Symbol;Acc:HGNC:14711]
Uncharacterized protein [Source:UniProtKB/TrEMBL;Acc:F1N1Y3]
FOS-like antigen 2 [Source:HGNC Symbol;Acc:HGNC:3798]
Uncharacterized protein [Source:UniProtKB/TrEMBL;Acc:E1BGV6]
olfactory receptor, family 8, subfamily D, member 4 [Source:HGNC Symbol;Acc:HGNC:14840]
transcription factor A, mitochondrial [Source:HGNC Symbol;Acc:HGNC:11741]
Bos taurus SMAD family member 1 (SMAD1), mRNA. [Source:RefSeq mRNA;Acc:NM_001076223]
Bos taurus transmembrane protein 50B (TMEM50B), mRNA. [Source:RefSeq mRNA;Acc:NM_001034786]
zinc finger and BTB domain containing 1 [Source:HGNC Symbol;Acc:HGNC:20259]
Uncharacterized protein [Source:UniProtKB/TrEMBL;Acc:F1MMG4]
Uncharacterized protein [Source:UniProtKB/TrEMBL;Acc:G5E6C9]
Uncharacterized protein [Source:UniProtKB/TrEMBL;Acc:G5E5W3]
olfactory receptor, family 10, subfamily R, member 2 [Source:HGNC Symbol;Acc:HGNC:14820]
Olfactory receptor [Source:UniProtKB/TrEMBL;Acc:E1BER0]
Uncharacterized protein [Source:UniProtKB/TrEMBL;Acc:E1BBA3]
Bos taurus FtsJ RNA methyltransferase homolog 2 (E. coli) (FTSJ2), mRNA. [Source:RefSeq mRNA;Acc:NM_001191258]
Bos taurus solute carrier family 2 (facilitated glucose/fructose transporter), member 5 (SLC2A5), mRNA. [Source:RefSeq mRNA;Acc:NM_001101042]
Uncharacterized protein [Source:UniProtKB/TrEMBL;Acc:G3X796]
Olfactory receptor [Source:UniProtKB/TrEMBL;Acc:E1BN41]

vomeronasal 1 receptor 4 [Source:HGNC Symbol;Acc:HGNC:19871]
olfactory receptor, family 7, subfamily D, member 2 [Source:HGNC Symbol;Acc:HGNC:8378]
Bos taurus G protein-coupled receptor 77 (GPR77), mRNA. [Source:RefSeq mRNA;Acc:NM_001077947]
Bos taurus chromosome 1 open reading frame, human C21orf91 (C1H21orf91), mRNA. [Source:RefSeq mRNA;Acc:NM_001037617]
Bos taurus ankyrin repeat and MYND domain containing 2 (ANKMY2), mRNA. [Source:RefSeq mRNA;Acc:NM_001075384]
Bos taurus C-terminal binding protein 2 (CTBP2), mRNA. [Source:RefSeq mRNA;Acc:NM_175712]
Bos taurus inositol hexakisphosphate kinase 3 (IP6K3), mRNA. [Source:RefSeq mRNA;Acc:NM_001040542]
Bos taurus proteolipid protein 1 (PLP1), mRNA. [Source:RefSeq mRNA;Acc:NM_174149]

Bos taurus amyloid beta (A4) precursor protein (APP), mRNA. [Source:RefSeq mRNA;Acc:NM_001076796]
Bos taurus C1q and tumor necrosis factor related protein 1 (C1QTNF1), mRNA. [Source:RefSeq mRNA;Acc:NM_001083409]
zinc finger protein 281 [Source:HGNC Symbol;Acc:HGNC:13075]

regulatory factor X, 7 [Source:HGNC Symbol;Acc:HGNC:25777]
Bos taurus jagunal homolog 1 (Drosophila) (JAGN1), mRNA. [Source:RefSeq mRNA;Acc:NM_001046450]
Olfactory receptor [Source:UniProtKB/TrEMBL;Acc:F1MQS6]
Uncharacterized protein [Source:UniProtKB/TrEMBL;Acc:G3X880]
Bos taurus serpin peptidase inhibitor, clade B (ovalbumin), member 2 (SERPINB2), mRNA. [Source:RefSeq mRNA;Acc:NM_001192079]
Bos taurus PRA1 domain family, member 2 (PRAF2), mRNA. [Source:RefSeq mRNA;Acc:NM_001046009]
Uncharacterized protein [Source:UniProtKB/TrEMBL;Acc:G5E6G9]
Uncharacterized protein [Source:UniProtKB/TrEMBL;Acc:G5E6A1]
Bos taurus tubulin folding cofactor C (TBCC), mRNA. [Source:RefSeq mRNA;Acc:NM_001075709]
Bos taurus lysophospholipase-like 1 (LYPLAL1), mRNA. [Source:RefSeq mRNA;Acc:NM_001099059]
hyaluronoglucosaminidase 4 [Source:HGNC Symbol;Acc:HGNC:5323]
Bos taurus N-ethylmaleimide-sensitive factor attachment protein, beta (NAPB), mRNA. [Source:RefSeq mRNA;Acc:NM_001046233]
Bos taurus mitochondrial translation optimization 1 homolog (S. cerevisiae) (MTO1), mRNA. [Source:RefSeq mRNA;Acc:NM_001076069]
Olfactory receptor [Source:UniProtKB/TrEMBL;Acc:E1BG17]
lactate dehydrogenase C [Source:HGNC Symbol;Acc:HGNC:6544]
Bos taurus butyrophilin family member (LOC525599), mRNA. [Source:RefSeq mRNA;Acc:NM_001081723]
Bos taurus sprouty homolog 2 (Drosophila) (SPRY2), mRNA. [Source:RefSeq mRNA;Acc:NM_001076147]

Bos taurus melanin-concentrating hormone receptor 1 (MCHR1), mRNA. [Source:RefSeq mRNA;Acc:NM_001103292]
Olfactory receptor [Source:UniProtKB/TrEMBL;Acc:F1N3H9]
zinc finger protein 12 [Source:HGNC Symbol;Acc:HGNC:12902]
Bos taurus MFNG O-fucosylpeptide 3-beta-N-acetylglucosaminyltransferase (MFNG), mRNA. [Source:RefSeq mRNA;Acc:NM_001101051]

Bos taurus abhydrolase domain containing 5 (ABHD5), mRNA. [Source:RefSeq mRNA;Acc:NM_001076063]
argonaute RISC catalytic component 4 [Source:HGNC Symbol;Acc:HGNC:18424]
myotubularin related protein 6 [Source:HGNC Symbol;Acc:HGNC:7453]
Bos taurus claudin 12 (CLDN12), mRNA. [Source:RefSeq mRNA;Acc:NM_001076123]
Bos taurus zinc finger protein 45 (ZNF45), mRNA. [Source:RefSeq mRNA;Acc:NM_001076213]
TROAP protein; Uncharacterized protein [Source:UniProtKB/TrEMBL;Acc:Q08DB3]
Bos taurus N-acetylneuraminic acid phosphatase (NANP), mRNA. [Source:RefSeq mRNA;Acc:NM_001105377]
Bos taurus RAP1B, member of RAS oncogene family (RAP1B), mRNA. [Source:RefSeq mRNA;Acc:NM_175824]
Bos taurus methylmalonic aciduria (cobalamin deficiency) cblD type, with homocystinuria (MMADHC), mRNA. [Source:RefSeq mRNA;Acc:NM_001034541]
THUMP domain containing 2 [Source:HGNC Symbol;Acc:HGNC:14890]
Olfactory receptor [Source:UniProtKB/TrEMBL;Acc:G3N3C8]
Bos taurus ubiquitin-conjugating enzyme E2 variant 2 (UBE2V2), mRNA. [Source:RefSeq mRNA;Acc:NM_001034042]
olfactory receptor, family 2, subfamily D, member 3 [Source:HGNC Symbol;Acc:HGNC:15146]
coiled-coil domain containing 14 [Source:HGNC Symbol;Acc:HGNC:25766]
Bos taurus deoxycytidine kinase (DCK), mRNA. [Source:RefSeq mRNA;Acc:NM_001034573]
Bos taurus zinc finger CCCH-type containing 15 (ZC3H15), mRNA. [Source:RefSeq mRNA;Acc:NM_001046394]
Uncharacterized protein [Source:UniProtKB/TrEMBL;Acc:G3X758]
Bos taurus thioredoxin-related transmembrane protein 3 (TMX3), mRNA. [Source:RefSeq mRNA;Acc:NM_001102289]
CDKN2A interacting protein [Source:HGNC Symbol;Acc:HGNC:24325]
olfactory receptor, family 4, subfamily K, member 15 [Source:HGNC Symbol;Acc:HGNC:15353]
sterile alpha motif domain containing 15 [Source:HGNC Symbol;Acc:HGNC:18631]
DnaJ homolog subfamily B member 4 [Source:UniProtKB/Swiss-Prot;Acc:Q2KIT4]
Olfactory receptor [Source:UniProtKB/TrEMBL;Acc:F1MJK8]
olfactory receptor, family 5, subfamily M, member 11 [Source:HGNC Symbol;Acc:HGNC:15291]
Bos taurus UDP glucuronosyltransferase 2 family, polypeptide B10 (UGT2B10), mRNA. [Source:RefSeq mRNA;Acc:NM_001046354]

G protein-coupled receptor 174 [Source:HGNC Symbol;Acc:HGNC:30245]
KIAA2018 [Source:HGNC Symbol;Acc:HGNC:30494]
Bos taurus androgen-induced 1 (AIG1), mRNA. [Source:RefSeq mRNA;Acc:NM_001076326]

Uncharacterized protein [Source:UniProtKB/TrEMBL;Acc:E1B9Z0]
Bos taurus dihydrouridine synthase 4-like (S. cerevisiae) (DUS4L), mRNA. [Source:RefSeq mRNA;Acc:NM_001101937]
Uncharacterized protein [Source:UniProtKB/TrEMBL;Acc:G5E6R7]
Bos taurus ELOVL fatty acid elongase 3 (ELOVL3), mRNA. [Source:RefSeq mRNA;Acc:NM_001192306]
Bos taurus apolipoprotein O (APOO), mRNA. [Source:RefSeq mRNA;Acc:NM_001075639]
Bos taurus methylsterol monooxygenase 1 (MSMO1), mRNA. [Source:RefSeq mRNA;Acc:NM_001098863]
Olfactory receptor [Source:UniProtKB/TrEMBL;Acc:G3N039]
Bos taurus mediator complex subunit 4 (MED4), mRNA. [Source:RefSeq mRNA;Acc:NM_001034486]
Uncharacterized protein [Source:UniProtKB/TrEMBL;Acc:F1MCD9]
Bos taurus N-acyl phosphatidylethanolamine phospholipase D (NAPEPLD), mRNA. [Source:RefSeq mRNA;Acc:NM_001015680]
lengsin, lens protein with glutamine synthetase domain [Source:HGNC Symbol;Acc:HGNC:21016]
Bos taurus cytochrome P450, family 4, subfamily B, polypeptide 1 (CYP4B1), mRNA. [Source:RefSeq mRNA;Acc:NM_001076202]
Bos taurus family with sequence similarity 26, member D (FAM26D), mRNA. [Source:RefSeq mRNA;Acc:NM_001101133]
Olfactory receptor [Source:UniProtKB/TrEMBL;Acc:E1BB68]
absent in melanoma 1 [Source:HGNC Symbol;Acc:HGNC:356]
keratin 9 [Source:HGNC Symbol;Acc:HGNC:6447]
protein-kinase, interferon-inducible double stranded RNA dependent inhibitor, repressor of (P58 repressor) [Source:HGNC Symbol;Acc:HGNC:9440]
Dab, reelin signal transducer, homolog 1 (Drosophila) [Source:HGNC Symbol;Acc:HGNC:2661]
XK, Kell blood group complex subunit-related family, member 9 [Source:HGNC Symbol;Acc:HGNC:20937]
brain and acute leukemia, cytoplasmic [Source:HGNC Symbol;Acc:HGNC:14333]
Uncharacterized protein [Source:UniProtKB/TrEMBL;Acc:F1MNY9]
Olfactory receptor [Source:UniProtKB/TrEMBL;Acc:M0QW44]
Bos taurus caspase 3, apoptosis-related cysteine peptidase (CASP3), mRNA. [Source:RefSeq mRNA;Acc:NM_001077840]
Bos taurus integrin beta 3 binding protein (beta3-endonexin) (ITGB3BP), mRNA. [Source:RefSeq mRNA;Acc:NM_001079619]
Uncharacterized protein [Source:UniProtKB/TrEMBL;Acc:G3MZA9]
zinc finger protein 268 [Source:HGNC Symbol;Acc:HGNC:13061]

Bos taurus GSK3B interacting protein (GSKIP), mRNA. [Source:RefSeq mRNA;Acc:NM_001078056]
zinc finger, SWIM-type containing 1 [Source:HGNC Symbol;Acc:HGNC:16155]

Wolfram syndrome 1 (wolframin) [Source:HGNC Symbol;Acc:HGNC:12762]
Olfactory receptor [Source:UniProtKB/TrEMBL;Acc:F1N6K2]
Rho guanine nucleotide exchange factor (GEF) 26 [Source:HGNC Symbol;Acc:HGNC:24490]
Bos taurus atlastin GTPase 3 (ATL3), mRNA. [Source:RefSeq mRNA;Acc:NM_001098978]
chromosome 9 open reading frame 72 [Source:HGNC Symbol;Acc:HGNC:28337]

Uncharacterized protein [Source:UniProtKB/TrEMBL;Acc:G3MZ15]
Olfactory receptor [Source:UniProtKB/TrEMBL;Acc:E1BCB5]
membrane bound O-acyltransferase domain containing 1 [Source:HGNC Symbol;Acc:HGNC:21579]
Olfactory receptor [Source:UniProtKB/TrEMBL;Acc:G3N2C6]

Bos taurus ring finger protein 34 (RNF34), mRNA. [Source:RefSeq mRNA;Acc:NM_001014858]
Bos taurus ras homolog gene family, member U (RHOU), mRNA. [Source:RefSeq mRNA;Acc:NM_001098147]
Bos taurus ataxin 3 (ATXN3), mRNA. [Source:RefSeq mRNA;Acc:NM_001099216]
Bos taurus trace amine associated receptor 6 (TAAR6), mRNA. [Source:RefSeq mRNA;Acc:NM_001206565]
Uncharacterized protein [Source:UniProtKB/TrEMBL;Acc:F1MQU6]
Bos taurus excision repair cross-complementing rodent repair deficiency, complementation group 6-like (ERCC6L), mRNA. [Source:RefSeq mRNA;Acc:NM_0011
Olfactory receptor [Source:UniProtKB/TrEMBL;Acc:G3X855]
Olfactory receptor [Source:UniProtKB/TrEMBL;Acc:E1BMX4]
Bos taurus phosphoglycerate mutase 1 (brain) (PGAM1), mRNA. [Source:RefSeq mRNA;Acc:NM_001034054]
Bos taurus ankyrin repeat domain 49 (ANKRD49), mRNA. [Source:RefSeq mRNA;Acc:NM_001014965]
ST3 beta-galactoside alpha-2,3-sialyltransferase 5 [Source:HGNC Symbol;Acc:HGNC:10872]
carboxymethylenebutenolidase homolog (Pseudomonas) [Source:HGNC Symbol;Acc:HGNC:25090]
Bos taurus hydroxy-delta-5-steroid dehydrogenase, 3 beta- and steroid delta-isomerase 1 (HSD3B1), mRNA. [Source:RefSeq mRNA;Acc:NM_174343]
Bos taurus suppression of tumorigenicity 13 (colon carcinoma) (Hsp70 interacting protein) (ST13), mRNA. [Source:RefSeq mRNA;Acc:NM_001101926]
Cytochrome b [Source:UniProtKB/Swiss-Prot;Acc:P00157]
Olfactory receptor [Source:UniProtKB/TrEMBL;Acc:G3MXT5]

Bos taurus inhibin, beta A (INHBA), mRNA. [Source:RefSeq mRNA;Acc:NM_174363]
Uncharacterized protein [Source:UniProtKB/TrEMBL;Acc:F1MKU6]
Bos taurus EP300 interacting inhibitor of differentiation 2 (EID2), mRNA. [Source:RefSeq mRNA;Acc:NM_001078049]
Olfactory receptor [Source:UniProtKB/TrEMBL;Acc:E1B6Y6]
Bos taurus golgi SNAP receptor complex member 2 (GOSR2), mRNA. [Source:RefSeq mRNA;Acc:NM_001098885]
E74-like factor 4 (ets domain transcription factor) [Source:HGNC Symbol;Acc:HGNC:3319]
Olfactory receptor [Source:UniProtKB/TrEMBL;Acc:E1B6Y4]
Bos taurus 5-hydroxytryptamine (serotonin) receptor 5A (HTR5A), mRNA. [Source:RefSeq mRNA;Acc:NM_001192842]
siah E3 ubiquitin protein ligase 1 [Source:HGNC Symbol;Acc:HGNC:10857]

Bos taurus ubiquitin-like domain containing CTD phosphatase 1 (UBLCP1), mRNA. [Source:RefSeq mRNA;Acc:NM_001045994]
Bos taurus ariadne homolog, ubiquitin-conjugating enzyme E2 binding protein, 1 (Drosophila) (ARIH1), mRNA. [Source:RefSeq mRNA;Acc:NM_001081714]
Bos taurus fat mass and obesity associated (FTO), mRNA. [Source:RefSeq mRNA;Acc:NM_001098142]
Olfactory receptor [Source:UniProtKB/TrEMBL;Acc:E1BNR4]
Olfactory receptor [Source:UniProtKB/TrEMBL;Acc:G3N0Y0]
Bos taurus retinol dehydrogenase 14 (all-trans/9-cis/11-cis) (RDH14), mRNA. [Source:RefSeq mRNA;Acc:NM_001075233]
Bos taurus vacuolar protein sorting 4 homolog A (S. cerevisiae) (VPS4A), mRNA. [Source:RefSeq mRNA;Acc:NM_001046615]
Bos taurus transformer 2 beta homolog (Drosophila) (TRA2B), mRNA. [Source:RefSeq mRNA;Acc:NM_001034776]
Bos taurus hippocampus abundant transcript 1 (HIAT1), mRNA. [Source:RefSeq mRNA;Acc:NM_001102038]
ubiquilin-like [Source:HGNC Symbol;Acc:HGNC:28294]
Bos taurus transmembrane protein 66 (TMEM66), mRNA. [Source:RefSeq mRNA;Acc:NM_001076856]
Olfactory receptor [Source:UniProtKB/TrEMBL;Acc:G3X7C6]
tRNA-yW synthesizing protein 5 [Source:HGNC Symbol;Acc:HGNC:26754]
Cbl proto-oncogene, E3 ubiquitin protein ligase [Source:HGNC Symbol;Acc:HGNC:1541]
Olfactory receptor [Source:UniProtKB/TrEMBL;Acc:G3MYQ7]
Olfactory receptor [Source:UniProtKB/TrEMBL;Acc:G3MXP7]
Uncharacterized protein [Source:UniProtKB/TrEMBL;Acc:G3MZ47]
Bos taurus hepatic and glial cell adhesion molecule (HEPACAM), mRNA. [Source:RefSeq mRNA;Acc:NM_001031759]
Bos taurus chromosome 9 open reading frame, human C6orf118 (C9H6orf118), mRNA. [Source:RefSeq mRNA;Acc:NM_001046213]
Bos taurus family with sequence similarity 109, member B (FAM109B), mRNA. [Source:RefSeq mRNA;Acc:NM_001045936]

Bos taurus oligodendrocyte myelin glycoprotein (OMG), mRNA. [Source:RefSeq mRNA;Acc:NM_001077524]
NFKB activating protein [Source:HGNC Symbol;Acc:HGNC:29873]
Bos taurus chromosome 27 open reading frame, human C8orf48 (C27H8orf48), mRNA. [Source:RefSeq mRNA;Acc:NM_001145540]
Olfactory receptor [Source:UniProtKB/TrEMBL;Acc:E1B865]
zinc finger and SCAN domain containing 4 [Source:HGNC Symbol;Acc:HGNC:23709]
Bos taurus G protein-coupled receptor 139 (GPR139), mRNA. [Source:RefSeq mRNA;Acc:NM_001192765]
SNF related kinase [Source:HGNC Symbol;Acc:HGNC:30598]
Bos taurus twinfilin, actin-binding protein, homolog 1 (Drosophila) (TWF1), mRNA. [Source:RefSeq mRNA;Acc:NM_001025320]
Bos taurus progestin and adipoQ receptor family member III (PAQR3), mRNA. [Source:RefSeq mRNA;Acc:NM_001206068]
Bos taurus MAX-like protein X (MLX), mRNA. [Source:RefSeq mRNA;Acc:NM_001035061]
Olfactory receptor [Source:UniProtKB/TrEMBL;Acc:F1MMU0]
Bos taurus lipoma HMGIC fusion partner-like 2 (LHFPL2), mRNA. [Source:RefSeq mRNA;Acc:NM_001099151]
Bos taurus structure specific recognition protein 1 (SSRP1), mRNA. [Source:RefSeq mRNA;Acc:NM_001101041]
Uncharacterized protein [Source:UniProtKB/TrEMBL;Acc:F1MYU9]
zinc finger protein 558 [Source:HGNC Symbol;Acc:HGNC:26422]
Bos taurus kelch-like 23 (Drosophila) (KLHL23), mRNA. [Source:RefSeq mRNA;Acc:NM_001205507]
Olfactory receptor [Source:UniProtKB/TrEMBL;Acc:G5E6H0]
Bos taurus actin related protein M1 (ARPM1), mRNA. [Source:RefSeq mRNA;Acc:NM_001038673]
nitrilase family, member 2 [Source:HGNC Symbol;Acc:HGNC:29878]
Bos taurus capping protein (actin filament) muscle Z-line, alpha 3 (CAPZA3), mRNA. [Source:RefSeq mRNA;Acc:NM_001040561]
tetratricopeptide repeat domain 39A [Source:HGNC Symbol;Acc:HGNC:18657]
Bos taurus hematopoietically expressed homeobox (HHEX), mRNA. [Source:RefSeq mRNA;Acc:NM_001105424]
MGC127538 protein; Uncharacterized protein [Source:UniProtKB/TrEMBL;Acc:Q3ZC86]
Bos taurus phosducin-like 3 (PDCL3), mRNA. [Source:RefSeq mRNA;Acc:NM_001075645]

Bos taurus WD repeat domain 43 (WDR43), mRNA. [Source:RefSeq mRNA;Acc:NM_001205598]
Olfactory receptor [Source:UniProtKB/TrEMBL;Acc:F1MT57]
Uncharacterized protein [Source:UniProtKB/TrEMBL;Acc:E1BHB6]
Bos taurus wingless-type MMTV integration site family, member 16 (WNT16), mRNA. [Source:RefSeq mRNA;Acc:NM_001014949]
ubiquitin specific peptidase like 1 [Source:HGNC Symbol;Acc:HGNC:20294]

Bos taurus myeloid-associated differentiation marker-like (LOC512440), mRNA. [Source:RefSeq mRNA;Acc:NM_001101956]
Olfactory receptor [Source:UniProtKB/TrEMBL;Acc:F1MVJ4]
Bos taurus cysteine-serine-rich nuclear protein 3 (CSRNP3), mRNA. [Source:RefSeq mRNA;Acc:NM_001076244]
chromosome 12 open reading frame 71 [Source:HGNC Symbol;Acc:HGNC:34452]
Olfactory receptor [Source:UniProtKB/TrEMBL;Acc:F1MRF0]
Olfactory receptor [Source:UniProtKB/TrEMBL;Acc:G5E5S1]
Olfactory receptor [Source:UniProtKB/TrEMBL;Acc:G5E559]
Mediterranean fever [Source:HGNC Symbol;Acc:HGNC:6998]
Bos taurus trafficking protein particle complex 2-like (TRAPPC2L), mRNA. [Source:RefSeq mRNA;Acc:NM_001098920]

PCF11 cleavage and polyadenylation factor subunit [Source:HGNC Symbol;Acc:HGNC:30097]
Bos taurus zinc finger protein 36, C3H type-like 1 (ZFP36L1), mRNA. [Source:RefSeq mRNA;Acc:NM_001101234]
Bos taurus PDZ domain containing 9 (PDZD9), mRNA. [Source:RefSeq mRNA;Acc:NM_001076094]
Bos taurus zinc finger, DHHC-type containing 21 (ZDHHC21), mRNA. [Source:RefSeq mRNA;Acc:NM_001081534]
LIM domain and actin binding 1 [Source:HGNC Symbol;Acc:HGNC:24636]
Bos taurus claudin 11 (CLDN11), mRNA. [Source:RefSeq mRNA;Acc:NM_001035055]
Bos taurus stonin 1 (STON1), mRNA. [Source:RefSeq mRNA;Acc:NM_001204851]

Uncharacterized protein [Source:UniProtKB/TrEMBL;Acc:G3MY44]
Bos taurus methionyl-tRNA synthetase 2, mitochondrial (MARS2), mRNA. [Source:RefSeq mRNA;Acc:NM_001098971]
Olfactory receptor [Source:UniProtKB/TrEMBL;Acc:G3X7E4]
Bos taurus X-ray repair complementing defective repair in Chinese hamster cells 2 (XRCC2), mRNA. [Source:RefSeq mRNA;Acc:NM_001102354]
protocadherin 7 [Source:HGNC Symbol;Acc:HGNC:8659]
Uncharacterized protein [Source:UniProtKB/TrEMBL;Acc:G3N057]
Bos taurus RAS-like, family 11, member B (RASL11B), mRNA. [Source:RefSeq mRNA;Acc:NM_001015635]
Bos taurus RNA polymerase II associated protein 2 (RPAP2), mRNA. [Source:RefSeq mRNA;Acc:NM_001103266]
family with sequence similarity 107, member B [Source:HGNC Symbol;Acc:HGNC:23726]
protein phosphatase, Mg2+/Mn2+ dependent, 1D [Source:HGNC Symbol;Acc:HGNC:9277]
Bos taurus MOB kinase activator 3B (MOB3B), mRNA. [Source:RefSeq mRNA;Acc:NM_001046491]
Olfactory receptor [Source:UniProtKB/TrEMBL;Acc:G3MWH9]

Bos taurus dermatan sulfate epimerase (DSE), mRNA. [Source:RefSeq mRNA;Acc:NM_001113232]
Bos taurus desmocollin 3 (DSC3), mRNA. [Source:RefSeq mRNA;Acc:NM_001178103]
chromosome 20 open reading frame 96 [Source:HGNC Symbol;Acc:HGNC:16227]
formin binding protein 1-like [Source:HGNC Symbol;Acc:HGNC:20851]
chromosome 2 open reading frame 44 [Source:HGNC Symbol;Acc:HGNC:26157]
Bos taurus solute carrier family 13 (sodium-dependent dicarboxylate transporter), member 2 (SLC13A2), mRNA. [Source:RefSeq mRNA;Acc:NM_001034234]
Uncharacterized protein [Source:UniProtKB/TrEMBL;Acc:F1MEF8]
Uncharacterized protein [Source:UniProtKB/TrEMBL;Acc:G3MX36]
olfactory receptor, family 6, subfamily F, member 1 [Source:HGNC Symbol;Acc:HGNC:15027]
Bos taurus gamma-aminobutyric acid (GABA) A receptor, pi (GABRP), mRNA. [Source:RefSeq mRNA;Acc:NM_001015618]
olfactory receptor, family 2, subfamily AK, member 2 [Source:HGNC Symbol;Acc:HGNC:19569]
pro-platelet basic protein (chemokine (C-X-C motif) ligand 7) [Source:HGNC Symbol;Acc:HGNC:9240]
Bos taurus c-mer proto-oncogene tyrosine kinase (MERTK), mRNA. [Source:RefSeq mRNA;Acc:NM_001192024]
chitinase 3-like 2 [Source:HGNC Symbol;Acc:HGNC:1933]
Uncharacterized protein [Source:UniProtKB/TrEMBL;Acc:F1MQZ3]
Bos taurus DNA-damage regulated autophagy modulator 1 (DRAM1), mRNA. [Source:RefSeq mRNA;Acc:NM_001031767]
olfactory receptor, family 6, subfamily C, member 1 [Source:HGNC Symbol;Acc:HGNC:8355]
Olfactory receptor [Source:UniProtKB/TrEMBL;Acc:G3MYH7]
interleukin 1 receptor, type I [Source:HGNC Symbol;Acc:HGNC:5993]
t-complex 11, testis-specific-like 1 [Source:HGNC Symbol;Acc:HGNC:25655]
protocadherin beta 5 [Source:HGNC Symbol;Acc:HGNC:8690]
Bos taurus SPRY domain containing 4 (SPRYD4), mRNA. [Source:RefSeq mRNA;Acc:NM_001076173]
Olfactory receptor [Source:UniProtKB/TrEMBL;Acc:E1BB60]
olfactory receptor, family 4, subfamily F, member 15 [Source:HGNC Symbol;Acc:HGNC:15078]
Bos taurus myocyte enhancer factor 2A (MEF2A), mRNA. [Source:RefSeq mRNA;Acc:NM_001083638]
Bos taurus endonuclease domain containing 1 (ENDOD1), mRNA. [Source:RefSeq mRNA;Acc:NM_001102519]
Bos taurus chromosome 9 open reading frame, human CXorf21 (C9HXorf21), mRNA. [Source:RefSeq mRNA;Acc:NM_001038537]
Uncharacterized protein [Source:UniProtKB/TrEMBL;Acc:F1MII6]
Bos taurus muscleblind-like 2 (Drosophila) (MBNL2), mRNA. [Source:RefSeq mRNA;Acc:NM_001099710]
Uncharacterized protein [Source:UniProtKB/TrEMBL;Acc:G3MXT1]

Olfactory receptor [Source:UniProtKB/TrEMBL;Acc:G3MZK5]
Bos taurus heat shock 22kDa protein 8 (HSPB8), mRNA. [Source:RefSeq mRNA;Acc:NM_001014955]
v-crk avian sarcoma virus CT10 oncogene homolog [Source:HGNC Symbol;Acc:HGNC:2362]
family with sequence similarity 111, member A [Source:HGNC Symbol;Acc:HGNC:24725]
Uncharacterized protein [Source:UniProtKB/TrEMBL;Acc:G3N0Q3]
Bos taurus Ras-related GTP binding A (RRAGA), mRNA. [Source:RefSeq mRNA;Acc:NM_001035499]
zinc finger protein 292 [Source:HGNC Symbol;Acc:HGNC:18410]
Uncharacterized protein [Source:UniProtKB/TrEMBL;Acc:F1MN95]
Uncharacterized protein [Source:UniProtKB/TrEMBL;Acc:F1N3X9]
Bos taurus pallidin homolog (mouse) (BLOC1S6), mRNA. [Source:RefSeq mRNA;Acc:NM_001076325]
Bos taurus CD274 molecule (CD274), mRNA. [Source:RefSeq mRNA;Acc:NM_001163412]
Bos taurus family with sequence similarity 175, member B (FAM175B), mRNA. [Source:RefSeq mRNA;Acc:NM_001166587]
Bos taurus CWC15 spliceosome-associated protein homolog (S. cerevisiae) (CWC15), mRNA. [Source:RefSeq mRNA;Acc:NM_001046399]
Bos taurus glial cells missing homolog 1 (Drosophila) (GCM1), transcript variant 2, mRNA. [Source:RefSeq mRNA;Acc:NM_001192105]
low density lipoprotein receptor-related protein 12 [Source:HGNC Symbol;Acc:HGNC:31708]
Olfactory receptor [Source:UniProtKB/TrEMBL;Acc:F1MKF7]
protocadherin beta 4 [Source:HGNC Symbol;Acc:HGNC:8689]
Olfactory receptor [Source:UniProtKB/TrEMBL;Acc:F1N481]
Bos taurus sulfotransferase family, cytosolic, 1C, member 4 (SULT1C4), mRNA. [Source:RefSeq mRNA;Acc:NM_001076097]
Bos taurus actin-binding Rho activating protein (ABRA), mRNA. [Source:RefSeq mRNA;Acc:NM_001192234]
Bos taurus prostaglandin F receptor (FP) (PTGFR), mRNA. [Source:RefSeq mRNA;Acc:NM_181025]
Bos taurus interleukin-1 receptor-associated kinase 3 (IRAK3), mRNA. [Source:RefSeq mRNA;Acc:NM_001190299]
sel-1 suppressor of lin-12-like 2 (C. elegans) [Source:HGNC Symbol;Acc:HGNC:15897]
serine/arginine-rich splicing factor 6 [Source:HGNC Symbol;Acc:HGNC:10788]
Bos taurus acetyl-CoA acyltransferase 2 (ACAA2), mRNA. [Source:RefSeq mRNA;Acc:NM_001035342]

establishment of sister chromatid cohesion N-acetyltransferase 1 [Source:HGNC Symbol;Acc:HGNC:24645]

Short transient receptor potential channel 6 [Source:UniProtKB/Swiss-Prot;Acc:Q9MYW0]

Bos taurus wingless-type MMTV integration site family member 2 (WNT2), mRNA. [Source:RefSeq mRNA;Acc:NM_001013001]
coiled-coil domain containing 102B [Source:HGNC Symbol;Acc:HGNC:26295]

Bos taurus family with sequence similarity 82, member A2 (RMDN3), mRNA. [Source:RefSeq mRNA;Acc:NM_001075679]
Bos taurus 5'-nucleotidase, cytosolic III (NT5C3), mRNA. [Source:RefSeq mRNA;Acc:NM_001037597]
Uncharacterized protein [Source:UniProtKB/TrEMBL;Acc:M0QW15]
Olfactory receptor [Source:UniProtKB/TrEMBL;Acc:F1MMR1]
Bos taurus hypoxia inducible factor 1, alpha subunit (basic helix-loop-helix transcription factor) (HIF1A), mRNA. [Source:RefSeq mRNA;Acc:NM_174339]
Bos taurus menage a trois homolog 1, cyclin H assembly factor (Xenopus laevis) (MNAT1), mRNA. [Source:RefSeq mRNA;Acc:NM_001105409]
zinc finger, CCHC domain containing 14 [Source:HGNC Symbol;Acc:HGNC:24134]
tyrosyl-DNA phosphodiesterase 1 [Source:HGNC Symbol;Acc:HGNC:18884]

retinitis pigmentosa 1 (autosomal dominant) [Source:HGNC Symbol;Acc:HGNC:10263]
Bos taurus major histocompatibility complex, class II, DRB3 (BOLA-DRB3), mRNA. [Source:RefSeq mRNA;Acc:NM_001012680]
Uncharacterized protein [Source:UniProtKB/TrEMBL;Acc:F1MP72]
Bos taurus zinc finger protein 177 (ZNF177), mRNA. [Source:RefSeq mRNA;Acc:NM_001102550]
Bos taurus follistatin-like 5 (FSTL5), mRNA. [Source:RefSeq mRNA;Acc:NM_001206863]
Bos taurus CCR4-NOT transcription complex, subunit 6-like (CNOT6L), mRNA. [Source:RefSeq mRNA;Acc:NM_001191523]

glyoxalase domain containing 4 [Source:HGNC Symbol;Acc:HGNC:14111]
small ArfGAP 1 [Source:HGNC Symbol;Acc:HGNC:19651]
ADAM metallopeptidase domain 20 [Source:HGNC Symbol;Acc:HGNC:199]
Bos taurus olfactory receptor-like (LOC504623), mRNA. [Source:RefSeq mRNA;Acc:NM_001206756]
Bos taurus protocadherin 20 (PCDH20), mRNA. [Source:RefSeq mRNA;Acc:NM_001206231]
Uncharacterized protein [Source:UniProtKB/TrEMBL;Acc:F1N7H9]
Bos taurus egl nine homolog 3 (C. elegans) (EGLN3), mRNA. [Source:RefSeq mRNA;Acc:NM_001101164]
Bos taurus TRM5 tRNA methyltransferase 5 homolog (S. cerevisiae) (TRMT5), mRNA. [Source:RefSeq mRNA;Acc:NM_001034565]
Olfactory receptor [Source:UniProtKB/TrEMBL;Acc:F1MNP1]
YTH domain family, member 3 [Source:HGNC Symbol;Acc:HGNC:26465]
Bos taurus F-box protein 5 (FBXO5), mRNA. [Source:RefSeq mRNA;Acc:NM_001082435]

Uncharacterized protein [Source:UniProtKB/TrEMBL;Acc:F1MMM9]
Uncharacterized protein [Source:UniProtKB/TrEMBL;Acc:F1N7P3]
Bos taurus pregnancy-associated glycoprotein 2 (PAG2), mRNA. [Source:RefSeq mRNA;Acc:NM_176614]
transmembrane protein 132D [Source:HGNC Symbol;Acc:HGNC:29411]
family with sequence similarity 208, member B [Source:HGNC Symbol;Acc:HGNC:23484]
Uncharacterized protein [Source:UniProtKB/TrEMBL;Acc:G3MWZ0]
Bos taurus phosphatase, orphan 2 (PHOSPHO2), mRNA. [Source:RefSeq mRNA;Acc:NM_001045965]

Bos taurus katanin p80 subunit B-like 1 (KATNBL1), mRNA. [Source:RefSeq mRNA;Acc:NM_001199064]
Nik related kinase [Source:HGNC Symbol;Acc:HGNC:25391]
interleukin 10 receptor, alpha [Source:HGNC Symbol;Acc:HGNC:5964]
kelch-like family member 8 [Source:HGNC Symbol;Acc:HGNC:18644]
zona pellucida-like domain containing 1 [Source:HGNC Symbol;Acc:HGNC:27022]

solute carrier family 7 (cationic amino acid transporter, y+ system), member 2 [Source:HGNC Symbol;Acc:HGNC:11060]
Bos taurus 6-pyruvoyltetrahydropterin synthase (PTS), mRNA. [Source:RefSeq mRNA;Acc:NM_001113537]
Bos taurus CCR4-NOT transcription complex, subunit 6 (CNOT6), mRNA. [Source:RefSeq mRNA;Acc:NM_001101161]
speckle-type POZ protein-like [Source:HGNC Symbol;Acc:HGNC:27934]
Uncharacterized protein [Source:UniProtKB/TrEMBL;Acc:F1MDB0]
chromosome 2 open reading frame 16 [Source:HGNC Symbol;Acc:HGNC:25275]
DEP domain containing 1 [Source:HGNC Symbol;Acc:HGNC:22949]
PDLIM1 interacting kinase 1 like [Source:HGNC Symbol;Acc:HGNC:18981]
CD180 molecule [Source:HGNC Symbol;Acc:HGNC:6726]
Bos taurus eukaryotic translation initiation factor 3, subunit M (EIF3M), mRNA. [Source:RefSeq mRNA;Acc:NM_001035312]
Uncharacterized protein [Source:UniProtKB/TrEMBL;Acc:F1MW05]
Uncharacterized protein [Source:UniProtKB/TrEMBL;Acc:G3N0G7]
Olfactory receptor [Source:UniProtKB/TrEMBL;Acc:F1MHI8]
Bos taurus CREB/ATF bZIP transcription factor (CREBZF), mRNA. [Source:RefSeq mRNA;Acc:NM_001099088]
Bos taurus fibrinogen-like 2 (FGL2), mRNA. [Source:RefSeq mRNA;Acc:NM_001046097]
Bos taurus spindlin family, member 2 (SPIN2), mRNA. [Source:RefSeq mRNA;Acc:NM_001079770]

Bos taurus solute carrier family 30 (zinc transporter), member 5 (SLC30A5), mRNA. [Source:RefSeq mRNA;Acc:NM_001192174]
Taste receptor type 2 [Source:UniProtKB/TrEMBL;Acc:F1MP17]
interferon-induced protein with tetratricopeptide repeats 1 [Source:HGNC Symbol;Acc:HGNC:5407]
Bos taurus proline-rich nuclear receptor coactivator 2 (PNRC2), mRNA. [Source:RefSeq mRNA;Acc:NM_001076957]
zinc finger, FYVE domain containing 9 [Source:HGNC Symbol;Acc:HGNC:6775]
Bos taurus ATPase, H+ transporting, lysosomal 34kDa, V1 subunit D (ATP6V1D), transcript variant 1, mRNA. [Source:RefSeq mRNA;Acc:NM_001075141]
Bos taurus zinc finger protein 474 (ZNF474), transcript variant 1, mRNA. [Source:RefSeq mRNA;Acc:NM_001105012]
Bos taurus PNMA-like 1 (PNMAL1), mRNA. [Source:RefSeq mRNA;Acc:NM_001102512]
Kruppel-like factor 8 [Source:HGNC Symbol;Acc:HGNC:6351]
Bos taurus zinc finger protein 286A (ZNF286A), mRNA. [Source:RefSeq mRNA;Acc:NM_001110443]
Uncharacterized protein [Source:UniProtKB/TrEMBL;Acc:F1MY70]
Bos taurus serpin peptidase inhibitor, clade B (ovalbumin), member 10 (SERPINB10), mRNA. [Source:RefSeq mRNA;Acc:NM_001098925]
Bos taurus chloride intracellular channel 4 (CLIC4), mRNA. [Source:RefSeq mRNA;Acc:NM_001080218]
ring finger protein 115 [Source:HGNC Symbol;Acc:HGNC:18154]
xin actin-binding repeat containing 2 [Source:HGNC Symbol;Acc:HGNC:14303]
Bos taurus cullin-associated and neddylation-dissociated 1 (CAND1), mRNA. [Source:RefSeq mRNA;Acc:NM_001101173]
Bos taurus RAB28, member RAS oncogene family (RAB28), mRNA. [Source:RefSeq mRNA;Acc:NM_001034634]
Olfactory receptor [Source:UniProtKB/TrEMBL;Acc:F1MNY6]

Bos taurus phytanoyl-CoA 2-hydroxylase interacting protein-like (PHYHIPL), mRNA. [Source:RefSeq mRNA;Acc:NM_001079798]
Bos taurus tetratricopeptide repeat domain 4 (TTC4), mRNA. [Source:RefSeq mRNA;Acc:NM_001015554]
Bos taurus tRNA methyltransferase 12 homolog (S. cerevisiae) (TRMT12), mRNA. [Source:RefSeq mRNA;Acc:NM_001024552]
Bos taurus dyskeratosis congenita 1, dyskerin (DKC1), mRNA. [Source:RefSeq mRNA;Acc:NM_001105395]
Uncharacterized protein [Source:UniProtKB/TrEMBL;Acc:G3MZ27]

Olfactory receptor [Source:UniProtKB/TrEMBL;Acc:E1BMX6]
Bos taurus activating transcription factor 1 (ATF1), mRNA. [Source:RefSeq mRNA;Acc:NM_001075289]
pancreatic lipase-related protein 3 [Source:HGNC Symbol;Acc:HGNC:23492]
Bos taurus taste receptor, type 2, member 4 (TAS2R4), mRNA. [Source:RefSeq mRNA;Acc:NM_001046635]
olfactory receptor, family 13, subfamily C, member 3 [Source:HGNC Symbol;Acc:HGNC:14704]

Bos taurus bromodomain, testis-specific (BRDT), mRNA. [Source:RefSeq mRNA;Acc:NM_001075852]
Bos taurus SET domain without mariner transposase fusion (SETMAR), mRNA. [Source:RefSeq mRNA;Acc:NM_001075508]
Bos taurus GTPase, IMAP family member 7 (GIMAP7), mRNA. [Source:RefSeq mRNA;Acc:NM_001080257]
Bos taurus visinin-like 1 (VSNL1), mRNA. [Source:RefSeq mRNA;Acc:NM_174490]
Bos taurus guanine nucleotide binding protein (G protein), alpha inhibiting activity polypeptide 1 (GNAI1), mRNA. [Source:RefSeq mRNA;Acc:NM_174324]
Bos taurus blocked early in transport 1 homolog (S. cerevisiae) (BET1), mRNA. [Source:RefSeq mRNA;Acc:NM_001099157]
Bos taurus acetyl-CoA acetyltransferase 1 (ACAT1), mRNA. [Source:RefSeq mRNA;Acc:NM_001046075]
Olfactory receptor [Source:UniProtKB/TrEMBL;Acc:G5E6B7]
intraflagellar transport protein 52 homolog [Source:RefSeq peptide;Acc:NP_001033190]
Bos taurus zinc finger protein 280A (ZNF280A), mRNA. [Source:RefSeq mRNA;Acc:NM_001192483]
serglycin [Source:HGNC Symbol;Acc:HGNC:9361]
Bos taurus pentraxin 3, long (PTX3), mRNA. [Source:RefSeq mRNA;Acc:NM_001076259]
Bos taurus RNA terminal phosphate cyclase-like 1 (RCL1), mRNA. [Source:RefSeq mRNA;Acc:NM_001077131]
zinc finger, CCHC domain containing 8 [Source:HGNC Symbol;Acc:HGNC:25265]
Uncharacterized protein [Source:UniProtKB/TrEMBL;Acc:G3N159]
Bos taurus lysyl oxidase (LOX), mRNA. [Source:RefSeq mRNA;Acc:NM_173932]
Bos taurus Ras suppressor protein 1 (RSU1), mRNA. [Source:RefSeq mRNA;Acc:NM_001040601]
Bos taurus proline-rich nuclear receptor coactivator 1 (PNRC1), mRNA. [Source:RefSeq mRNA;Acc:NM_001034308]
Bos taurus pyruvate dehydrogenase kinase, isozyme 4 (PDK4), mRNA. [Source:RefSeq mRNA;Acc:NM_001101883]
centrosomal protein 97kDa [Source:HGNC Symbol;Acc:HGNC:26244]
Bos taurus HIG1 domain family, member 1D (HIGD1D), mRNA. [Source:RefSeq mRNA;Acc:NM_001077114]
valosin containing protein lysine (K) methyltransferase [Source:HGNC Symbol;Acc:HGNC:20352]
Olfactory receptor [Source:UniProtKB/TrEMBL;Acc:G3N2V9]
Bos taurus dynein, light chain, Tctex-type 3 (DYNLT3), mRNA. [Source:RefSeq mRNA;Acc:NM_001101220]
Bos taurus mitochondrial fission regulator 1 (MTFR1), mRNA. [Source:RefSeq mRNA;Acc:NM_001035412]
Bos taurus chemokine (C-X-C motif) receptor 4 (CXCR4), mRNA. [Source:RefSeq mRNA;Acc:NM_174301]
Bos taurus zinc finger protein LOC509810 (LOC509810), mRNA. [Source:RefSeq mRNA;Acc:NM_001077894]
kinesin family member 24 [Source:HGNC Symbol;Acc:HGNC:19916]
Bos taurus X-linked inhibitor of apoptosis (XIAP), mRNA. [Source:RefSeq mRNA;Acc:NM_001205592]
olfactory receptor, family 4, subfamily K, member 2 [Source:HGNC Symbol;Acc:HGNC:14728]

Bos taurus proteasome (prosome, macropain) subunit, alpha type, 1 (PSMA1), mRNA. [Source:RefSeq mRNA;Acc:NM_001035310]
cadherin 26 [Source:HGNC Symbol;Acc:HGNC:15902]
ventricular zone expressed PH domain-containing 1 [Source:HGNC Symbol;Acc:HGNC:25735]
Bos taurus leucine rich repeat containing 58 (LRRC58), mRNA. [Source:RefSeq mRNA;Acc:NM_001206760]
Bos taurus KRR1, small subunit (SSU) processome component, homolog (yeast) (KRR1), mRNA. [Source:RefSeq mRNA;Acc:NM_001037819]
Uncharacterized protein [Source:UniProtKB/TrEMBL;Acc:G3N2E1]
cation channel, sperm associated 3 [Source:HGNC Symbol;Acc:HGNC:20819]
Bos taurus retinyl ester hydrolase type 1 (BREH1), mRNA. [Source:RefSeq mRNA;Acc:NM_001012287]

ectonucleoside triphosphate diphosphohydrolase 7 [Source:HGNC Symbol;Acc:HGNC:19745]
Bos taurus peptidylprolyl isomerase domain and WD repeat containing 1 (PPWD1), mRNA. [Source:RefSeq mRNA;Acc:NM_001046231]
Bos taurus inositol monophosphatase domain containing 1 (IMPAD1), mRNA. [Source:RefSeq mRNA;Acc:NM_001046335]
muscleblind-like splicing regulator 1 [Source:HGNC Symbol;Acc:HGNC:6923]
Bos taurus chromosome 15 open reading frame, human C11orf46 (ARL14EP), mRNA. [Source:RefSeq mRNA;Acc:NM_001031761]
Uncharacterized protein [Source:UniProtKB/TrEMBL;Acc:G3X6P7]
Bos taurus corticotropin releasing hormone binding protein (CRHBP), mRNA. [Source:RefSeq mRNA;Acc:NM_001101202]
Bos taurus immediate early response 3 interacting protein 1 (IER3IP1), mRNA. [Source:RefSeq mRNA;Acc:NM_001113320]
Bos taurus family with sequence similarity 136, member A (FAM136A), mRNA. [Source:RefSeq mRNA;Acc:NM_001046588]
Bos taurus peroxisomal biogenesis factor 26 (PEX26), mRNA. [Source:RefSeq mRNA;Acc:NM_001101172]
Uncharacterized protein [Source:UniProtKB/TrEMBL;Acc:F1MXY5]
Bos taurus phosphopantothenoylcysteine synthetase (PPCS), mRNA. [Source:RefSeq mRNA;Acc:NM_001102228]
Uncharacterized protein [Source:UniProtKB/TrEMBL;Acc:G3X6F2]
RAD1 checkpoint DNA exonuclease [Source:HGNC Symbol;Acc:HGNC:9806]
tryptophan hydroxylase 2 [Source:HGNC Symbol;Acc:HGNC:20692]
zinc finger protein 570 [Source:HGNC Symbol;Acc:HGNC:26416]
Bos taurus chemokine (C-X-C motif) ligand 10 (CXCL10), mRNA. [Source:RefSeq mRNA;Acc:NM_001046551]
Bos taurus thioredoxin domain containing 12 (endoplasmic reticulum) (TXNDC12), mRNA. [Source:RefSeq mRNA;Acc:NM_001015536]
centrosomal protein 63kDa [Source:HGNC Symbol;Acc:HGNC:25815]
Bos taurus BCL2-associated athanogene 2 (BAG2), mRNA. [Source:RefSeq mRNA;Acc:NM_001034264]
chromosome 4 open reading frame 47 [Source:HGNC Symbol;Acc:HGNC:34346]

Bos taurus alpha- and gamma-adaptin binding protein (AAGAB), mRNA. [Source:RefSeq mRNA;Acc:NM_001098896]
Olfactory receptor [Source:UniProtKB/TrEMBL;Acc:F1ME32]
Olfactory receptor [Source:UniProtKB/TrEMBL;Acc:G5E5E3]
Bos taurus internexin neuronal intermediate filament protein, alpha (INA), mRNA. [Source:RefSeq mRNA;Acc:NM_001075958]
Bos taurus serine/threonine kinase 4 (STK4), mRNA. [Source:RefSeq mRNA;Acc:NM_001015602]
Bos taurus BRX1, biogenesis of ribosomes, homolog (S. cerevisiae) (BRIX1), mRNA. [Source:RefSeq mRNA;Acc:NM_001034529]
Olfactory receptor [Source:UniProtKB/TrEMBL;Acc:F1MY99]
Uncharacterized protein [Source:UniProtKB/TrEMBL;Acc:G3N202]
abhydrolase domain containing 3 [Source:HGNC Symbol;Acc:HGNC:18718]
cadherin 8, type 2 precursor [Source:RefSeq peptide;Acc:NP_001030416]
leucine rich repeat and Ig domain containing 2 [Source:HGNC Symbol;Acc:HGNC:21207]
Uncharacterized protein [Source:UniProtKB/TrEMBL;Acc:F1MLM3]
zinc finger and BTB domain containing 26 [Source:HGNC Symbol;Acc:HGNC:23383]
Uncharacterized protein [Source:UniProtKB/TrEMBL;Acc:M0QW31]
Bos taurus coagulation factor XI (F11), mRNA. [Source:RefSeq mRNA;Acc:NM_001008665]
Uncharacterized protein [Source:UniProtKB/TrEMBL;Acc:E1BJ94]
keratin, type I cytoskeletal 24 [Source:RefSeq peptide;Acc:NP_001192390]
Bos taurus NGFI-A binding protein 1 (EGR1 binding protein 1) (NAB1), mRNA. [Source:RefSeq mRNA;Acc:NM_001102039]
Bos taurus ATPase, H+ transporting, lysosomal 38kDa, V0 subunit d2 (ATP6V0D2), mRNA. [Source:RefSeq mRNA;Acc:NM_001046101]
sodium/hydrogen exchanger-like domain-containing protein 1 [Source:RefSeq peptide;Acc:NP_001071360]
Olfactory receptor [Source:UniProtKB/TrEMBL;Acc:G3MZU9]
Bos taurus chromosome 19 open reading frame, human C17orf42 (C19H17orf42), mRNA. [Source:RefSeq mRNA;Acc:NM_001168010]
nuclear factor, erythroid 2-like 2 [Source:HGNC Symbol;Acc:HGNC:7782]
Bos taurus CD48 molecule (CD48), mRNA. [Source:RefSeq mRNA;Acc:NM_001046002]
Bos taurus armadillo repeat containing 1 (ARMC1), mRNA. [Source:RefSeq mRNA;Acc:NM_001015594]
Bos taurus myelin protein zero-like 3 (MPZL3), mRNA. [Source:RefSeq mRNA;Acc:NM_001098051]
chromosome 17 open reading frame 104 [Source:HGNC Symbol;Acc:HGNC:26670]
Bos taurus serine/threonine kinase 38 like (STK38L), mRNA. [Source:RefSeq mRNA;Acc:NM_001101092]
Bos taurus podoplanin (PDPN), mRNA. [Source:RefSeq mRNA;Acc:NM_001033120]
Bos taurus zinc finger, C3HC-type containing 1 (ZC3HC1), mRNA. [Source:RefSeq mRNA;Acc:NM_001034591]

protein-O-mannose kinase [Source:HGNC Symbol;Acc:HGNC:26267]
storkhead box 1 [Source:HGNC Symbol;Acc:HGNC:23508]
Bos taurus asunder, spermatogenesis regulator homolog (Drosphila) (ASUN), mRNA. [Source:RefSeq mRNA;Acc:NM_001046499]
Uncharacterized protein [Source:UniProtKB/TrEMBL;Acc:M0QW27]
Bos taurus synovial sarcoma translocation, chromosome 18 (SS18), mRNA. [Source:RefSeq mRNA;Acc:NM_001098122]
leucine rich repeat neuronal 3 [Source:HGNC Symbol;Acc:HGNC:17200]
STEAP family member 4 [Source:HGNC Symbol;Acc:HGNC:21923]
Uncharacterized protein [Source:UniProtKB/TrEMBL;Acc:G5E5Y3]

Bos taurus proteasome (prosome, macropain) 26S subunit, non-ATPase, 14 (PSMD14), mRNA. [Source:RefSeq mRNA;Acc:NM_001076067]
Bos taurus muscle-related coiled-coil protein (MURC), mRNA. [Source:RefSeq mRNA;Acc:NM_001103264]
chromosome 10 open reading frame 90 [Source:HGNC Symbol;Acc:HGNC:26563]
Bos taurus protein kinase, interferon-inducible double stranded RNA dependent activator (PRKRA), mRNA. [Source:RefSeq mRNA;Acc:NM_001045870]
Bos taurus protein phosphatase 2, regulatory subunit B', alpha (PPP2R5A), mRNA. [Source:RefSeq mRNA;Acc:NM_001081728]
Uncharacterized protein [Source:UniProtKB/TrEMBL;Acc:F1MYB0]
Bos taurus transcription factor Dp-1 (TFDP1), mRNA. [Source:RefSeq mRNA;Acc:NM_001076029]
Bos taurus transmembrane protein 30A (TMEM30A), mRNA. [Source:RefSeq mRNA;Acc:NM_001075223]
Bos taurus UTP23, small subunit (SSU) processome component, homolog (yeast) (UTP23), mRNA. [Source:RefSeq mRNA;Acc:NM_001076039]
glutamine and serine rich 1 [Source:HGNC Symbol;Acc:HGNC:26154]
Uncharacterized protein [Source:UniProtKB/TrEMBL;Acc:G5E694]
kinesin family member 3A [Source:HGNC Symbol;Acc:HGNC:6319]
Bos taurus ectonucleotide pyrophosphatase/phosphodiesterase 4 (putative) (ENPP4), mRNA. [Source:RefSeq mRNA;Acc:NM_001081535]
Bos taurus serpin peptidase inhibitor, clade A (alpha-1 antiproteinase, antitrypsin), member 12 (SERPINA12), mRNA. [Source:RefSeq mRNA;Acc:NM_001077517
ubiquilin 2 [Source:HGNC Symbol;Acc:HGNC:12509]
suppressor of cytokine signaling 5 [Source:HGNC Symbol;Acc:HGNC:16852]
Bos taurus nucleoside-diphosphate kinase NBR-A (LOC574091), mRNA. [Source:RefSeq mRNA;Acc:NM_001025347]
Bos taurus Bardet-Biedl syndrome 5 (BBS5), mRNA. [Source:RefSeq mRNA;Acc:NM_001101132]
Olfactory receptor [Source:UniProtKB/TrEMBL;Acc:G5E6F8]
Bos taurus Rho family GTPase 3 (RND3), mRNA. [Source:RefSeq mRNA;Acc:NM_001191158]
Bos taurus SAM and SH3 domain containing 3 (SASH3), mRNA. [Source:RefSeq mRNA;Acc:NM_001077998]

Bos taurus uncharacterized protein MGC134040 (MGC134040), transcript variant 1, mRNA. [Source:RefSeq mRNA;Acc:NM_001077037]
dynamin 3 [Source:HGNC Symbol;Acc:HGNC:29125]
centrosomal protein 83kDa [Source:HGNC Symbol;Acc:HGNC:17966]
Bos taurus mal, T-cell differentiation protein 2 (MAL2), mRNA. [Source:RefSeq mRNA;Acc:NM_001081719]
Bos taurus NADH dehydrogenase (ubiquinone) 1 alpha subcomplex, assembly factor 1 (NDUFAF1), mRNA. [Source:RefSeq mRNA;Acc:NM_001206074]
Bos taurus tetratricopeptide repeat domain 29 (TTC29), mRNA. [Source:RefSeq mRNA;Acc:NM_001099073]
cadherin 10, type 2 (T2-cadherin) [Source:HGNC Symbol;Acc:HGNC:1749]
Bos taurus late endosomal/lysosomal adaptor, MAPK and MTOR activator 3 (LAMTOR3), mRNA. [Source:RefSeq mRNA;Acc:NM_001075982]
Bos taurus shisa homolog 2 (Xenopus laevis) (SHISA2), mRNA. [Source:RefSeq mRNA;Acc:NM_001101265]
Bos taurus pyruvate dehydrogenase complex, component X (PDHX), mRNA. [Source:RefSeq mRNA;Acc:NM_001075751]
Bos taurus MKI67 (FHA domain) interacting nucleolar phosphoprotein (MKI67IP), mRNA. [Source:RefSeq mRNA;Acc:NM_001034354]
Bos taurus DnaJ (Hsp40) homolog, subfamily C, member 12 (DNAJC12), mRNA. [Source:RefSeq mRNA;Acc:NM_174096]
Bos taurus recombination signal binding protein for immunoglobulin kappa J region (RBPJ), mRNA. [Source:RefSeq mRNA;Acc:NM_001077001]
protocadherin gamma subfamily A, 3 [Source:HGNC Symbol;Acc:HGNC:8701]
Uncharacterized protein [Source:UniProtKB/TrEMBL;Acc:G3N2S5]
ring finger protein 217 [Source:HGNC Symbol;Acc:HGNC:21487]
Bos taurus zinc finger and SCAN domain containing 21 (ZSCAN21), mRNA. [Source:RefSeq mRNA;Acc:NM_001035368]
protein phosphatase 1, regulatory subunit 15B [Source:HGNC Symbol;Acc:HGNC:14951]
Uncharacterized protein [Source:UniProtKB/TrEMBL;Acc:G3MY70]
Uncharacterized protein [Source:UniProtKB/TrEMBL;Acc:G5E6K2]
topoisomerase (DNA) II binding protein 1 [Source:HGNC Symbol;Acc:HGNC:17008]
Bos taurus glucocorticoid modulatory element binding protein 1 (GMEB1), mRNA. [Source:RefSeq mRNA;Acc:NM_001046475]
Bos taurus actin related protein 2/3 complex, subunit 2, 34kDa (ARPC2), mRNA. [Source:RefSeq mRNA;Acc:NM_001034713]
Bos taurus nicotinamide phosphoribosyltransferase (NAMPT), mRNA. [Source:RefSeq mRNA;Acc:NM_001244141]
armadillo repeat containing 10 [Source:HGNC Symbol;Acc:HGNC:21706]
Bos taurus BCL2/adenovirus E1B 19kDa interacting protein 3-like (BNIP3L), mRNA. [Source:RefSeq mRNA;Acc:NM_001034614]
Bos taurus ELL associated factor 2 (EAF2), mRNA. [Source:RefSeq mRNA;Acc:NM_001046507]
Bos taurus UDP-Gal:betaGlcNAc beta 1,3-galactosyltransferase, polypeptide 2 (B3GALT2), mRNA. [Source:RefSeq mRNA;Acc:NM_001076188]
Uncharacterized protein [Source:UniProtKB/TrEMBL;Acc:E1BMG0]
zinc fingers and homeoboxes 2 [Source:HGNC Symbol;Acc:HGNC:18513]

Bos taurus phosphatidylcholine transfer protein (PCTP), mRNA. [Source:RefSeq mRNA;Acc:NM_174835]
Uncharacterized protein [Source:UniProtKB/TrEMBL;Acc:E1BEY4]
Sp4 transcription factor [Source:HGNC Symbol;Acc:HGNC:11209]
Bos taurus guanine nucleotide binding protein (G protein), gamma 4 (GNG4), mRNA. [Source:RefSeq mRNA;Acc:NM_001103324]
Uncharacterized protein [Source:UniProtKB/TrEMBL;Acc:G3N2J8]
Bos taurus pleckstrin homology domain containing, family F (with FYVE domain) member 2 (PLEKHF2), mRNA. [Source:RefSeq mRNA;Acc:NM_001098150]
Bos taurus CCR4-NOT transcription complex, subunit 7 (CNOT7), mRNA. [Source:RefSeq mRNA;Acc:NM_001034312]
Bos taurus guanylate binding protein 4 (GBP4), mRNA. [Source:RefSeq mRNA;Acc:NM_001102261]
Bos taurus calsyntenin 3 (CLSTN3), mRNA. [Source:RefSeq mRNA;Acc:NM_001075425]
amine oxidase, copper containing 3 [Source:HGNC Symbol;Acc:HGNC:550]
Bos taurus family with sequence similarity 135, member B (FAM135B), mRNA. [Source:RefSeq mRNA;Acc:NM_001078091]
Uncharacterized protein [Source:UniProtKB/TrEMBL;Acc:G5E6A5]
protocadherin gamma subfamily A, 7 [Source:HGNC Symbol;Acc:HGNC:8705]
Bos taurus chromosome 21 open reading frame, human C15orf40 (C21H15orf40), mRNA. [Source:RefSeq mRNA;Acc:NM_001075386]
heterogeneous nuclear ribonucleoprotein L-like [Source:HGNC Symbol;Acc:HGNC:25127]
zinc finger protein 572 [Source:HGNC Symbol;Acc:HGNC:26758]
Uncharacterized protein [Source:UniProtKB/TrEMBL;Acc:G3MWU5]
Bos taurus dCMP deaminase (DCTD), mRNA. [Source:RefSeq mRNA;Acc:NM_001101292]
dynactin 4 (p62) [Source:HGNC Symbol;Acc:HGNC:15518]
Bos taurus poly (ADP-ribose) polymerase family, member 11 (PARP11), mRNA. [Source:RefSeq mRNA;Acc:NM_001082452]
Bos taurus immunoglobulin-like domain containing receptor 1 (ILDR1), mRNA. [Source:RefSeq mRNA;Acc:NM_001102214]
Bos taurus signal-regulatory protein delta (SIRPD), mRNA. [Source:RefSeq mRNA;Acc:NM_001075887]
Uncharacterized protein [Source:UniProtKB/TrEMBL;Acc:F1MHU6]
synaptotagmin XVI [Source:HGNC Symbol;Acc:HGNC:23142]
Bos taurus casein beta (CSN2), mRNA. [Source:RefSeq mRNA;Acc:NM_181008]
Uncharacterized protein [Source:UniProtKB/TrEMBL;Acc:G3N1M2]
Bos taurus acyl-CoA binding domain containing 7 (ACBD7), mRNA. [Source:RefSeq mRNA;Acc:NM_001113297]
DENN/MADD domain containing 6A [Source:HGNC Symbol;Acc:HGNC:26635]
Bos taurus acyl-CoA synthetase short-chain family member 3 (ACSS3), mRNA. [Source:RefSeq mRNA;Acc:NM_001102137]
Bos taurus exosome component 9 (EXOSC9), mRNA. [Source:RefSeq mRNA;Acc:NM_001035053]

Bos taurus klotho (KL), mRNA. [Source:RefSeq mRNA;Acc:NM_001191195]
Bos taurus fatty acid desaturase 2 (FADS2), mRNA. [Source:RefSeq mRNA;Acc:NM_001083444]
Uncharacterized protein [Source:UniProtKB/TrEMBL;Acc:F1MQX8]
Olfactory receptor [Source:UniProtKB/TrEMBL;Acc:F1MKD2]
Bos taurus MANSC domain containing 1 (MANSC1), mRNA. [Source:RefSeq mRNA;Acc:NM_001075592]
Bos taurus T cell receptor associated transmembrane adaptor 1 (TRAT1), mRNA. [Source:RefSeq mRNA;Acc:NM_001163943]
Bos taurus high mobility group nucleosomal binding domain 4 (HMGN4), mRNA. [Source:RefSeq mRNA;Acc:NM_001046547]
dual-specificity tyrosine-(Y)-phosphorylation regulated kinase 3 [Source:HGNC Symbol;Acc:HGNC:3094]
phosphoinositide-3-kinase adaptor protein 1 [Source:HGNC Symbol;Acc:HGNC:30034]
G protein-coupled receptor 22 [Source:HGNC Symbol;Acc:HGNC:4477]
Bos taurus leucine rich repeat containing 49 (LRRC49), mRNA. [Source:RefSeq mRNA;Acc:NM_001100343]
Bos taurus MAP3K12 binding inhibitory protein 1 (MBIP), mRNA. [Source:RefSeq mRNA;Acc:NM_001077518]
orthodenticle homeobox 2 [Source:HGNC Symbol;Acc:HGNC:8522]
Bos taurus chromosome 7 open reading frame, human C5orf24 (C7H5orf24), mRNA. [Source:RefSeq mRNA;Acc:NM_001076110]
Bos taurus ribosomal L1 domain containing 1 (RSL1D1), mRNA. [Source:RefSeq mRNA;Acc:NM_001014876]
cylicin, basic protein of sperm head cytoskeleton 1 [Source:HGNC Symbol;Acc:HGNC:2582]
Bos taurus A kinase (PRKA) anchor protein 7 (AKAP7), mRNA. [Source:RefSeq mRNA;Acc:NM_001102266]
Bos taurus protease, serine, 35 (PRSS35), mRNA. [Source:RefSeq mRNA;Acc:NM_001035457]
Bos taurus Myb/SANT-like DNA-binding domain containing 4 with coiled-coils (MSANTD4), mRNA. [Source:RefSeq mRNA;Acc:NM_001046078]
caspase recruitment domain family, member 6 [Source:HGNC Symbol;Acc:HGNC:16394]
group-specific component (vitamin D binding protein) [Source:HGNC Symbol;Acc:HGNC:4187]
STE20-like kinase [Source:HGNC Symbol;Acc:HGNC:11088]
protein kinase C, theta [Source:HGNC Symbol;Acc:HGNC:9410]
JRK-like [Source:HGNC Symbol;Acc:HGNC:6200]
tigger transposable element derived 4 [Source:HGNC Symbol;Acc:HGNC:18335]
Bos taurus interferon (alpha, beta and omega) receptor 1 (IFNAR1), mRNA. [Source:RefSeq mRNA;Acc:NM_174552]

F-box and leucine-rich repeat protein 17 [Source:HGNC Symbol;Acc:HGNC:13615]
Bos taurus septin 11 (SEPT11), mRNA. [Source:RefSeq mRNA;Acc:NM_001082447]
Bos taurus leucine rich repeat containing 39 (LRRC39), mRNA. [Source:RefSeq mRNA;Acc:NM_001076425]

zinc finger protein 239 [Source:HGNC Symbol;Acc:HGNC:13031]
Bos taurus potassium channel tetramerisation domain containing 6 (KCTD6), mRNA. [Source:RefSeq mRNA;Acc:NM_001101224]
family with sequence similarity 198, member B [Source:HGNC Symbol;Acc:HGNC:25312]
LysM, putative peptidoglycan-binding, domain containing 1 [Source:HGNC Symbol;Acc:HGNC:32070]
Bos taurus transmembrane 4 L six family member 18 (TM4SF18), transcript variant 1, mRNA. [Source:RefSeq mRNA;Acc:NM_001184724]
Bos taurus transmembrane protein 231 (TMEM231), mRNA. [Source:RefSeq mRNA;Acc:NM_001101078]
arylacetamide deacetylase-like 2 [Source:HGNC Symbol;Acc:HGNC:24427]
Bos taurus transcription factor AP-2 alpha (activating enhancer binding protein 2 alpha) (TFAP2A), mRNA. [Source:RefSeq mRNA;Acc:NM_001080228]
ring finger protein, LIM domain interacting [Source:HGNC Symbol;Acc:HGNC:13429]
Bos taurus SEC62 homolog (S. cerevisiae) (SEC62), mRNA. [Source:RefSeq mRNA;Acc:NM_001205604]
Bos taurus solute carrier family 16, member 7 (monocarboxylic acid transporter 2) (SLC16A7), mRNA. [Source:RefSeq mRNA;Acc:NM_001076336]

cytochrome P450, family 7, subfamily B, polypeptide 1 [Source:HGNC Symbol;Acc:HGNC:2652]
Bos taurus malate dehydrogenase 1, NAD (soluble) (MDH1), mRNA. [Source:RefSeq mRNA;Acc:NM_001034628]
Bos taurus transcription factor B2, mitochondrial (TFB2M), mRNA. [Source:RefSeq mRNA;Acc:NM_001038127]
Bos taurus phosphoribosyl pyrophosphate amidotransferase (PPAT), mRNA. [Source:RefSeq mRNA;Acc:NM_001102375]
CDP-diacylglycerol synthase (phosphatidate cytidylyltransferase) 1 [Source:HGNC Symbol;Acc:HGNC:1800]
Bos taurus adenylate kinase 4 (AK4), mRNA. [Source:RefSeq mRNA;Acc:NM_001077933]
SUMO1/sentrin specific peptidase 5 [Source:HGNC Symbol;Acc:HGNC:28407]

Bos taurus serine/threonine protein kinase MST4 (MST4), mRNA. [Source:RefSeq mRNA;Acc:NM_001163786]
YY1 associated factor 2 [Source:HGNC Symbol;Acc:HGNC:17363]
NLR family, pyrin domain containing 14 [Source:HGNC Symbol;Acc:HGNC:22939]
Bos taurus trafficking protein particle complex 6B (TRAPPC6B), mRNA. [Source:RefSeq mRNA;Acc:NM_001038134]
Bos taurus hydroxysteroid (17-beta) dehydrogenase 3 (HSD17B3), mRNA. [Source:RefSeq mRNA;Acc:NM_001076439]
Bos taurus phytanoyl-CoA 2-hydroxylase (PHYH), mRNA. [Source:RefSeq mRNA;Acc:NM_174142]
Bos taurus histone cluster 1, H2bn (HIST1H2BB), mRNA. [Source:RefSeq mRNA;Acc:NM_001081742]
spermatogenesis associated 6 [Source:HGNC Symbol;Acc:HGNC:18309]
Bos taurus tRNA-histidine guanylyltransferase 1-like (S. cerevisiae) (THG1L), mRNA. [Source:RefSeq mRNA;Acc:NM_001080233]
Bos taurus churchill domain containing 1 (CHURC1), mRNA. [Source:RefSeq mRNA;Acc:NM_001046581]

Uncharacterized protein [Source:UniProtKB/TrEMBL;Acc:F1N0I8]
phosphatidylinositol-4-phosphate 3-kinase, catalytic subunit type 2 alpha [Source:HGNC Symbol;Acc:HGNC:8971]
Bos taurus glypican 3 (GPC3), mRNA. [Source:RefSeq mRNA;Acc:NM_001035463]
Bos taurus solute carrier family 6 (neutral amino acid transporter), member 15 (SLC6A15), mRNA. [Source:RefSeq mRNA;Acc:NM_181023]
Bos taurus lin-7 homolog C (C. elegans) (LIN7C), mRNA. [Source:RefSeq mRNA;Acc:NM_001075431]
Bos taurus taspase, threonine aspartase, 1 (TASP1), mRNA. [Source:RefSeq mRNA;Acc:NM_001034577]
Bos taurus general transcription factor IIH, polypeptide 3, 34kDa (GTF2H3), mRNA. [Source:RefSeq mRNA;Acc:NM_001079589]
Bos taurus NOP10 ribonucleoprotein homolog (yeast) (NOP10), mRNA. [Source:RefSeq mRNA;Acc:NM_001114521]
Uncharacterized protein [Source:UniProtKB/TrEMBL;Acc:E1BHD0]
G protein-coupled receptor 149 [Source:HGNC Symbol;Acc:HGNC:23627]
3-hydroxy-3-methylglutaryl-CoA synthase 1 (soluble) [Source:HGNC Symbol;Acc:HGNC:5007]
Bos taurus bifunctional apoptosis regulator (BFAR), mRNA. [Source:RefSeq mRNA;Acc:NM_001098869]
Bos taurus aquaporin 4 (AQP4), mRNA. [Source:RefSeq mRNA;Acc:NM_181003]
junction mediating and regulatory protein, p53 cofactor [Source:HGNC Symbol;Acc:HGNC:28916]
Bos taurus glycerol kinase 2 (GK2), mRNA. [Source:RefSeq mRNA;Acc:NM_001256551]
SMEK homolog 2, suppressor of mek1 (Dictyostelium) [Source:HGNC Symbol;Acc:HGNC:29267]
Olfactory receptor [Source:UniProtKB/TrEMBL;Acc:F1N0J4]
Bos taurus synaptic vesicle glycoprotein 2B (SV2B), mRNA. [Source:RefSeq mRNA;Acc:NM_001083448]
Uncharacterized protein [Source:UniProtKB/TrEMBL;Acc:E1B9Z4]
Bos taurus ring finger protein 38 (RNF38), mRNA. [Source:RefSeq mRNA;Acc:NM_001034581]
KAT8 regulatory NSL complex subunit 1-like [Source:HGNC Symbol;Acc:HGNC:26310]
Bos taurus cathepsin S (CTSS), mRNA. [Source:RefSeq mRNA;Acc:NM_001033615]
Bos taurus L-3-hydroxyproline dehydratase (trans-) (L3HYPDH), mRNA. [Source:RefSeq mRNA;Acc:NM_001034386]
Uncharacterized protein [Source:UniProtKB/TrEMBL;Acc:G5E5U4]
Uncharacterized protein [Source:UniProtKB/TrEMBL;Acc:G5E5P0]
Bos taurus amidohydrolase domain containing 1 (AMDHD1), mRNA. [Source:RefSeq mRNA;Acc:NM_001098872]
Bos taurus coagulation factor II (thrombin) receptor-like 2 (F2RL2), mRNA. [Source:RefSeq mRNA;Acc:NM_001038533]
Bos taurus GTPase, IMAP family member 7 (GIMAP7), mRNA. [Source:RefSeq mRNA;Acc:NM_001105034]
Bos taurus integrator complex subunit 12 (INTS12), mRNA. [Source:RefSeq mRNA;Acc:NM_001075753]
Uncharacterized protein [Source:UniProtKB/TrEMBL;Acc:M0QW22]

Bos taurus spindle and kinetochore associated complex subunit 3 (SKA3), mRNA. [Source:RefSeq mRNA;Acc:NM_001110067]
Bos taurus zinc finger protein 484 (ZNF484), mRNA. [Source:RefSeq mRNA;Acc:NM_001192027]
Bos taurus EP300 interacting inhibitor of differentiation 1 (EID1), mRNA. [Source:RefSeq mRNA;Acc:NM_001098377]
Bos taurus TP53 regulated inhibitor of apoptosis 1 (TRIAP1), mRNA. [Source:RefSeq mRNA;Acc:NM_001079776]
Uncharacterized protein [Source:UniProtKB/TrEMBL;Acc:G3MXF8]
Bos taurus endoplasmic reticulum protein 27 (ERP27), mRNA. [Source:RefSeq mRNA;Acc:NM_001038041]
Bos taurus palmdelphin (PALMD), mRNA. [Source:RefSeq mRNA;Acc:NM_001035079]
ESF1, nucleolar pre-rRNA processing protein, homolog (S. cerevisiae) [Source:HGNC Symbol;Acc:HGNC:15898]
Uncharacterized protein [Source:UniProtKB/TrEMBL;Acc:G5E6C5]
Bos taurus peptidase inhibitor 3, skin-derived (SKALP) (PI3), mRNA. [Source:RefSeq mRNA;Acc:NM_001080353]
inhibitor of growth family, member 3 [Source:HGNC Symbol;Acc:HGNC:14587]
Bos taurus neuropeptide Y receptor Y1 (NPY1R), mRNA. [Source:RefSeq mRNA;Acc:NM_001045898]
Bos taurus zinc finger protein, X-linked (ZFX), mRNA. [Source:RefSeq mRNA;Acc:NM_177490]
zinc finger and BTB domain containing 41 [Source:HGNC Symbol;Acc:HGNC:24819]
trichohyalin-like 1 [Source:HGNC Symbol;Acc:HGNC:31796]
Bos taurus phospholipase A2 receptor 1, 180kDa (PLA2R1), mRNA. [Source:RefSeq mRNA;Acc:NM_175051]
Bos taurus oligonucleotide/oligosaccharide-binding fold containing 2A (NABP1), mRNA. [Source:RefSeq mRNA;Acc:NM_001098124]
Bos taurus down-regulator of transcription 1, TBP-binding (negative cofactor 2) (DR1), mRNA. [Source:RefSeq mRNA;Acc:NM_001046519]
Bos taurus chemokine (C-C motif) receptor 5 (CCR5), mRNA. [Source:RefSeq mRNA;Acc:NM_001011672]
Bos taurus developmental pluripotency associated 3 (DPPA3), transcript variant 1, mRNA. [Source:RefSeq mRNA;Acc:NM_001111109]
Bos taurus zinc finger and BTB domain containing 33 (ZBTB33), mRNA. [Source:RefSeq mRNA;Acc:NM_001098157]
transmembrane protein 199 [Source:HGNC Symbol;Acc:HGNC:18085]
Bos taurus cell growth regulator with ring finger domain 1 (CGRRF1), mRNA. [Source:RefSeq mRNA;Acc:NM_001192807]
Bos taurus regulator of calcineurin 1 (RCAN1), mRNA. [Source:RefSeq mRNA;Acc:NM_001034679]
fibronectin leucine rich transmembrane protein 3 [Source:HGNC Symbol;Acc:HGNC:3762]
Bos taurus gamma-aminobutyric acid (GABA) A receptor, alpha 6 (GABRA6), mRNA. [Source:RefSeq mRNA;Acc:NM_001144085]
ferredoxin-fold anticodon binding domain containing 1 [Source:HGNC Symbol;Acc:HGNC:25110]
Bos taurus GINS complex subunit 3 (Psf3 homolog) (GINS3), mRNA. [Source:RefSeq mRNA;Acc:NM_001076136]
Bos taurus mitochondrial ribosomal protein L1 (MRPL1), mRNA. [Source:RefSeq mRNA;Acc:NM_001101844]
Bos taurus ARP6 actin-related protein 6 homolog (yeast) (ACTR6), mRNA. [Source:RefSeq mRNA;Acc:NM_001099124]

Bos taurus calcium-binding protein, spermatid-specific 1 (CABS1), mRNA. [Source:RefSeq mRNA;Acc:NM_001040539]
maestro [Source:HGNC Symbol;Acc:HGNC:24121]
Bos taurus kelch repeat and BTB (POZ) domain containing 4 (KBTBD4), mRNA. [Source:RefSeq mRNA;Acc:NM_001076463]
Bos taurus peptidase domain containing associated with muscle regeneration 1 (PAMR1), mRNA. [Source:RefSeq mRNA;Acc:NM_001015591]
solute carrier family 12 (sodium/potassium/chloride transporter), member 2 [Source:HGNC Symbol;Acc:HGNC:10911]
Uncharacterized protein [Source:UniProtKB/TrEMBL;Acc:E1B7I3]
Bos taurus glycoprotein galactosyltransferase alpha 1, 3-like (LOC527409), mRNA. [Source:RefSeq mRNA;Acc:NM_001077974]
Bos taurus osteopetrosis associated transmembrane protein 1 (OSTM1), mRNA. [Source:RefSeq mRNA;Acc:NM_001075776]
SLIT and NTRK-like family, member 6 [Source:HGNC Symbol;Acc:HGNC:23503]
Bos taurus transmembrane protein 22 (TMEM22), mRNA. [Source:RefSeq mRNA;Acc:NM_001075524]
RAD51 associated protein 2 [Source:HGNC Symbol;Acc:HGNC:34417]
Bos taurus cholinergic receptor, muscarinic 2 (CHRM2), mRNA. [Source:RefSeq mRNA;Acc:NM_001080733]
Bos taurus transmembrane protein 106A (TMEM106A), mRNA. [Source:RefSeq mRNA;Acc:NM_001014870]
enkurin, TRPC channel interacting protein [Source:HGNC Symbol;Acc:HGNC:28388]
Bos taurus neutral cholesterol ester hydrolase 1 (NCEH1), mRNA. [Source:RefSeq mRNA;Acc:NM_001123034]
Bos taurus RCD1 required for cell differentiation1 homolog (S. pombe) (RQCD1), mRNA. [Source:RefSeq mRNA;Acc:NM_001101167]
Uncharacterized protein [Source:UniProtKB/TrEMBL;Acc:E1BKY8]
pygopus family PHD finger 1 [Source:HGNC Symbol;Acc:HGNC:30256]
Bos taurus cytochrome P450, family 2, subfamily C, polypeptide 18 (CYP2C18), mRNA. [Source:RefSeq mRNA;Acc:NM_001076051]
exostosin-like glycosyltransferase 2 [Source:HGNC Symbol;Acc:HGNC:3516]
Bos taurus kelch-like 7 (Drosophila) (KLHL7), mRNA. [Source:RefSeq mRNA;Acc:NM_001076037]
Olfactory receptor [Source:UniProtKB/TrEMBL;Acc:E1BD33]
ras homolog family member H [Source:HGNC Symbol;Acc:HGNC:686]
Zic family member 2 [Source:HGNC Symbol;Acc:HGNC:12873]
biogenesis of lysosomal organelles complex-1, subunit 5, muted [Source:HGNC Symbol;Acc:HGNC:18561]
sterile alpha motif domain containing 9 [Source:HGNC Symbol;Acc:HGNC:1348]
Bos taurus chondroitin sulfate synthase 1 (CHSY1), mRNA. [Source:RefSeq mRNA;Acc:NM_001191157]
Bos taurus G protein-coupled receptor 52 (GPR52), mRNA. [Source:RefSeq mRNA;Acc:NM_001100304]
Bos taurus polymerase (RNA) I polypeptide B, 128kDa (POLR1B), mRNA. [Source:RefSeq mRNA;Acc:NM_001100292]
kynureninase [Source:HGNC Symbol;Acc:HGNC:6469]

EF-hand calcium binding domain 3 [Source:HGNC Symbol;Acc:HGNC:26379]

C-type lectin-like 1 [Source:HGNC Symbol;Acc:HGNC:24462]
Rho GTPase activating protein 20 [Source:HGNC Symbol;Acc:HGNC:18357]
Bos taurus 5-hydroxytryptamine (serotonin) receptor 2A (HTR2A), mRNA. [Source:RefSeq mRNA;Acc:NM_001001157]
Bos taurus nuclear receptor binding factor 2 (NRBF2), mRNA. [Source:RefSeq mRNA;Acc:NM_001078131]
Bos taurus NudC domain containing 1 (NUDCD1), mRNA. [Source:RefSeq mRNA;Acc:NM_001076880]
KIAA1210 [Source:HGNC Symbol;Acc:HGNC:29218]
Bos taurus DEAD (Asp-Glu-Ala-Asp) box polypeptide 10 (DDX10), mRNA. [Source:RefSeq mRNA;Acc:NM_001076881]
Bos taurus PDZ and LIM domain 5 (PDLIM5), mRNA. [Source:RefSeq mRNA;Acc:NM_001077444]
Bos taurus regulator of G-protein signaling 7 binding protein (RGS7BP), mRNA. [Source:RefSeq mRNA;Acc:NM_001076930]
Bos taurus mannosyl (alpha-1,3-)-glycoprotein beta-1,4-N-acetylglucosaminyltransferase, isozyme C (putative) (MGAT4C), mRNA. [Source:RefSeq mRNA;Acc:N[illegible]
Bos taurus adenylate cyclase activating polypeptide 1 (pituitary) (ADCYAP1), mRNA. [Source:RefSeq mRNA;Acc:NM_001046555]
Uncharacterized protein [Source:UniProtKB/TrEMBL;Acc:F1N4W2]
Bos taurus DnaJ (Hsp40) homolog, subfamily C, member 22 (DNAJC22), mRNA. [Source:RefSeq mRNA;Acc:NM_001075701]
Bos taurus ankyrin repeat and SOCS box containing 15 (ASB15), mRNA. [Source:RefSeq mRNA;Acc:NM_174687]
Bos taurus F-box and leucine-rich repeat protein 3 (FBXL3), mRNA. [Source:RefSeq mRNA;Acc:NM_001102246]
ADAM metallopeptidase domain 21 [Source:HGNC Symbol;Acc:HGNC:200]
family with sequence similarity 169, member A [Source:HGNC Symbol;Acc:HGNC:29138]
Bos taurus zinc fingers and homeoboxes 1 (ZHX1), mRNA. [Source:RefSeq mRNA;Acc:NM_001110095]
Bos taurus uncharacterized LOC783682 (MGC152301), mRNA. [Source:RefSeq mRNA;Acc:NM_001099190]
maelstrom spermatogenic transposon silencer [Source:HGNC Symbol;Acc:HGNC:25929]
Bos taurus acyl-CoA thioesterase 9 (ACOT9), mRNA. [Source:RefSeq mRNA;Acc:NM_001034560]
Bos taurus APEX nuclease (apurinic/apyrimidinic endonuclease) 2 (APEX2), mRNA. [Source:RefSeq mRNA;Acc:NM_001015577]

zinc finger protein 132 [Source:HGNC Symbol;Acc:HGNC:12916]
Bos taurus UTP14, U3 small nucleolar ribonucleoprotein, homolog A (yeast) (UTP14A), mRNA. [Source:RefSeq mRNA;Acc:NM_001034241]
Uncharacterized protein [Source:UniProtKB/TrEMBL;Acc:F1N614]
Bos taurus translocase of outer mitochondrial membrane 70 homolog A (S. cerevisiae) (TOMM70A), mRNA. [Source:RefSeq mRNA;Acc:NM_001075328]
Bos taurus JNK1/MAPK8-associated membrane protein (JKAMP), mRNA. [Source:RefSeq mRNA;Acc:NM_001080247]

Bos taurus serpin peptidase inhibitor, clade A (alpha-1 antiproteinase, antitrypsin), member 7 (SERPINA7), mRNA. [Source:RefSeq mRNA;Acc:NM_174668]
Bos taurus coagulation factor XIII, B polypeptide (F13B), mRNA. [Source:RefSeq mRNA;Acc:NM_001038529]
Bos taurus osteocrin (OSTN), mRNA. [Source:RefSeq mRNA;Acc:NM_001098935]
Bos taurus interleukin 2 receptor, alpha (IL2RA), mRNA. [Source:RefSeq mRNA;Acc:NM_174358]
cyclin and CBS domain divalent metal cation transport mediator 2 [Source:HGNC Symbol;Acc:HGNC:103]
Bos taurus zinc finger protein 200 (ZNF200), mRNA. [Source:RefSeq mRNA;Acc:NM_001083700]
Bos taurus SUB1 homolog (S. cerevisiae) (SUB1), mRNA. [Source:RefSeq mRNA;Acc:NM_001105407]
Bos taurus sorting nexin 7 (SNX7), mRNA. [Source:RefSeq mRNA;Acc:NM_001163937]
Bos taurus RAP2C, member of RAS oncogene family (RAP2C), mRNA. [Source:RefSeq mRNA;Acc:NM_001075700]
filamin A interacting protein 1 [Source:HGNC Symbol;Acc:HGNC:21015]
zinc finger protein 614 [Source:HGNC Symbol;Acc:HGNC:24722]
Bos taurus ring finger protein 111 (RNF111), mRNA. [Source:RefSeq mRNA;Acc:NM_001102239]
Bos taurus bol, boule-like (Drosophila) (BOLL), transcript variant 1, mRNA. [Source:RefSeq mRNA;Acc:NM_001102115]
Wilms tumor 1 associated protein [Source:HGNC Symbol;Acc:HGNC:16846]
Bos taurus melanoma antigen family E, 2 (MAGEE2), mRNA. [Source:RefSeq mRNA;Acc:NM_001076876]
Bos taurus transmembrane protein 67 (TMEM67), mRNA. [Source:RefSeq mRNA;Acc:NM_001205299]
Bos taurus RAB8B, member RAS oncogene family (RAB8B), mRNA. [Source:RefSeq mRNA;Acc:NM_001076317]
Bos taurus cysteine-rich secretory protein 2 (CRISP2), mRNA. [Source:RefSeq mRNA;Acc:NM_001038089]
Bos taurus threonyl-tRNA synthetase-like 2 (TARSL2), mRNA. [Source:RefSeq mRNA;Acc:NM_001102089]
Rho GTPase activating protein 24 [Source:HGNC Symbol;Acc:HGNC:25361]
Bos taurus transmembrane protein 35 (TMEM35), mRNA. [Source:RefSeq mRNA;Acc:NM_001014940]
Uncharacterized protein [Source:UniProtKB/TrEMBL;Acc:E1BGE4]
Bos taurus keratin 10 (KRT10), mRNA. [Source:RefSeq mRNA;Acc:NM_174377]
phosphodiesterase 4D, cAMP-specific [Source:HGNC Symbol;Acc:HGNC:8783]
Uncharacterized protein [Source:UniProtKB/TrEMBL;Acc:E1BA88]
Bos taurus hyaluronan binding protein 4 (HABP4), mRNA. [Source:RefSeq mRNA;Acc:NM_001081523]
Bos taurus phospholipid scramblase 4 (PLSCR4), mRNA. [Source:RefSeq mRNA;Acc:NM_001081732]
Bos taurus OCIA domain containing 2 (OCIAD2), mRNA. [Source:RefSeq mRNA;Acc:NM_001034258]
Bos taurus RE1-silencing transcription factor (REST), mRNA. [Source:RefSeq mRNA;Acc:NM_001101880]
nipsnap homolog 3A (C. elegans) [Source:HGNC Symbol;Acc:HGNC:23619]

protein FAM84B [Source:RefSeq peptide;Acc:NP_001099102]
Bos taurus peripheral myelin protein 2 (PMP2), mRNA. [Source:RefSeq mRNA;Acc:NM_001075239]
Bos taurus DTW domain containing 1 (DTWD1), mRNA. [Source:RefSeq mRNA;Acc:NM_001098120]
N(alpha)-acetyltransferase 15, NatA auxiliary subunit [Source:HGNC Symbol;Acc:HGNC:30782]
Bos taurus thymocyte selection associated (THEMIS), mRNA. [Source:RefSeq mRNA;Acc:NM_001098116]
Bos taurus uncharacterized LOC507942 (MGC152281), mRNA. [Source:RefSeq mRNA;Acc:NM_001101891]
Dimethylaniline monooxygenase [N-oxide-forming] [Source:UniProtKB/TrEMBL;Acc:F1N007]
Bos taurus protein kinase (cAMP-dependent, catalytic) inhibitor alpha (PKIA), mRNA. [Source:RefSeq mRNA;Acc:NM_001037620]
Rho GTPase activating protein 5 [Source:HGNC Symbol;Acc:HGNC:675]
Bos taurus Bardet-Biedl syndrome 2 (BBS2), mRNA. [Source:RefSeq mRNA;Acc:NM_001038160]
membrane protein, palmitoylated 6 (MAGUK p55 subfamily member 6) [Source:HGNC Symbol;Acc:HGNC:18167]
Bos taurus family with sequence similarity 81, member B (FAM81B), mRNA. [Source:RefSeq mRNA;Acc:NM_001075688]
Bos taurus SH3 domain binding glutamic acid-rich protein like (SH3BGRL), mRNA. [Source:RefSeq mRNA;Acc:NM_001035440]
Bos taurus ADP-dependent glucokinase (ADPGK), mRNA. [Source:RefSeq mRNA;Acc:NM_001082438]
solute carrier family 30 (zinc transporter), member 9 [Source:HGNC Symbol;Acc:HGNC:1329]
ring finger protein 103 [Source:HGNC Symbol;Acc:HGNC:12859]
Bos taurus glutamate receptor, metabotropic 3 (GRM3), mRNA. [Source:RefSeq mRNA;Acc:NM_001098123]
Bos taurus peroxisomal biogenesis factor 13 (PEX13), mRNA. [Source:RefSeq mRNA;Acc:NM_001076212]
Bos taurus ENTH domain containing 1 (ENTHD1), mRNA. [Source:RefSeq mRNA;Acc:NM_001205542]
Bos taurus WD repeat domain 26 (WDR26), mRNA. [Source:RefSeq mRNA;Acc:NM_001077059]
matrix metallopeptidase 27 [Source:HGNC Symbol;Acc:HGNC:14250]
Bos taurus amyotrophic lateral sclerosis 2 (juvenile) chromosome region, candidate 8 (ALS2CR8), mRNA. [Source:RefSeq mRNA;Acc:NM_001032294]

Bos taurus protein phosphatase 2, regulatory subunit B'', gamma (PPP2R3C), mRNA. [Source:RefSeq mRNA;Acc:NM_001015645]
Bos taurus GrpE-like 2, mitochondrial (E. coli) (GRPEL2), mRNA. [Source:RefSeq mRNA;Acc:NM_001075674]
Bos taurus BMP and activin membrane-bound inhibitor homolog (Xenopus laevis) (BAMBI), mRNA. [Source:RefSeq mRNA;Acc:NM_001046309]
Bos taurus TAF5 RNA polymerase II, TATA box binding protein (TBP)-associated factor, 100kDa (TAF5), mRNA. [Source:RefSeq mRNA;Acc:NM_001191478]
alpha-kinase 1 [Source:HGNC Symbol;Acc:HGNC:20917]
Uncharacterized protein [Source:UniProtKB/TrEMBL;Acc:G3MWL2]
Bos taurus family with sequence similarity 151, member B (FAM151B), mRNA. [Source:RefSeq mRNA;Acc:NM_001099713]

Olfactory receptor [Source:UniProtKB/TrEMBL;Acc:F1N2C4]
Bos taurus syntaxin 12 (STX12), mRNA. [Source:RefSeq mRNA;Acc:NM_001101053]
Bos taurus kelch-like 28 (Drosophila) (KLHL28), mRNA. [Source:RefSeq mRNA;Acc:NM_001099030]
Bos taurus claudin 8 (CLDN8), mRNA. [Source:RefSeq mRNA;Acc:NM_001098096]
Uncharacterized protein [Source:UniProtKB/TrEMBL;Acc:F1MHR7]
Bos taurus tetratricopeptide repeat domain 30B (TTC30B), mRNA. [Source:RefSeq mRNA;Acc:NM_001099091]
Bos taurus small integral membrane protein 10 (SMIM10), mRNA. [Source:RefSeq mRNA;Acc:NM_001163442]
Bos taurus methyltransferase like 7A (METTL7A), mRNA. [Source:RefSeq mRNA;Acc:NM_001035439]
Bos taurus coxsackie virus and adenovirus receptor (CXADR), mRNA. [Source:RefSeq mRNA;Acc:NM_174298]
Bos taurus profilin 2 (PFN2), mRNA. [Source:RefSeq mRNA;Acc:NM_001128197]
Bos taurus protocadherin beta 11 (PCDHB11), mRNA. [Source:RefSeq mRNA;Acc:NM_001103248]
Bos taurus zona pellucida binding protein 2 (ZPBP2), mRNA. [Source:RefSeq mRNA;Acc:NM_001075661]
zinc finger protein 354C [Source:HGNC Symbol;Acc:HGNC:16736]
Bos taurus prenyl (decaprenyl) diphosphate synthase, subunit 2 (PDSS2), mRNA. [Source:RefSeq mRNA;Acc:NM_001100385]
Bos taurus elongation factor, RNA polymerase II, 2 (ELL2), mRNA. [Source:RefSeq mRNA;Acc:NM_001191146]
Bos taurus zinc finger, CW type with PWWP domain 1 (ZCWPW1), mRNA. [Source:RefSeq mRNA;Acc:NM_001076853]
Bos taurus G protein-coupled receptor 183 (GPR183), mRNA. [Source:RefSeq mRNA;Acc:NM_001046472]
Bos taurus selenoprotein T (SELT), mRNA. [Source:RefSeq mRNA;Acc:NM_001103103]
Bos taurus adenosine kinase (ADK), mRNA. [Source:RefSeq mRNA;Acc:NM_001076066]
Bos taurus chromosome 10 open reading frame, human C15orf41 (C10H15orf41), mRNA. [Source:RefSeq mRNA;Acc:NM_001015668]
Uncharacterized protein [Source:UniProtKB/TrEMBL;Acc:F1MX34]
Bos taurus STE20-related kinase adaptor beta (STRADB), mRNA. [Source:RefSeq mRNA;Acc:NM_001192081]
Cation channel sperm-associated protein subunit delta [Source:UniProtKB/Swiss-Prot;Acc:E1B9E5]
Bos taurus heterogeneous nuclear ribonucleoprotein U-like 1 (HNRNPUL1), mRNA. [Source:RefSeq mRNA;Acc:NM_001083466]
dpy-19-like 2 (C. elegans) [Source:HGNC Symbol;Acc:HGNC:19414]
Bos taurus capping protein (actin filament) muscle Z-line, alpha 2 (CAPZA2), mRNA. [Source:RefSeq mRNA;Acc:NM_001012998]
required for meiotic nuclear division 1 homolog (S. cerevisiae) [Source:HGNC Symbol;Acc:HGNC:21176]
Bos taurus cysteinyl leukotriene receptor 2 (CYSLTR2), mRNA. [Source:RefSeq mRNA;Acc:NM_001102240]
Bos taurus cytochrome P450, family 2, subfamily R, polypeptide 1 (CYP2R1), mRNA. [Source:RefSeq mRNA;Acc:NM_001076267]
fucose-1-phosphate guanylyltransferase [Source:HGNC Symbol;Acc:HGNC:3825]

Bos taurus leucine rich repeat transmembrane neuronal 3 (LRRTM3), mRNA. [Source:RefSeq mRNA;Acc:NM_001103283]
Bos taurus CD69 molecule (CD69), mRNA. [Source:RefSeq mRNA;Acc:NM_174014]
potassium channel tetramerization domain containing 3 [Source:HGNC Symbol;Acc:HGNC:21305]
Bos taurus regulation of nuclear pre-mRNA domain containing 1A (RPRD1A), mRNA. [Source:RefSeq mRNA;Acc:NM_001075156]
mucolipin 2 [Source:HGNC Symbol;Acc:HGNC:13357]
Bos taurus inositol(myo)-1(or 4)-monophosphatase 1 (IMPA1), mRNA. [Source:RefSeq mRNA;Acc:NM_174361]
Bos taurus cornichon homolog (Drosophila) (CNIH), mRNA. [Source:RefSeq mRNA;Acc:NM_001034739]
Uncharacterized protein [Source:UniProtKB/TrEMBL;Acc:G5E5A4]
Bos taurus glucosamine-6-phosphate deaminase 2 (GNPDA2), mRNA. [Source:RefSeq mRNA;Acc:NM_001075356]
choroideremia-like (Rab escort protein 2) [Source:HGNC Symbol;Acc:HGNC:1941]
Olfactory receptor [Source:UniProtKB/TrEMBL;Acc:F1MFK2]
Bos taurus angiopoietin-like 5 (ANGPTL5), mRNA. [Source:RefSeq mRNA;Acc:NM_001128069]
asparagine synthetase domain containing 1 [Source:HGNC Symbol;Acc:HGNC:24910]
Bos taurus chromosome 7 open reading frame, human C5orf15 (C7H5orf15), mRNA. [Source:RefSeq mRNA;Acc:NM_001035299]
cellular repressor of E1A-stimulated genes 1 [Source:HGNC Symbol;Acc:HGNC:2351]
Bos taurus phosphorylase, glycogen, liver (PYGL), mRNA. [Source:RefSeq mRNA;Acc:NM_001075203]
Bos taurus uncharacterized LOC781988 (LOC781988), mRNA. [Source:RefSeq mRNA;Acc:NM_001101281]
family with sequence similarity 46, member D [Source:HGNC Symbol;Acc:HGNC:28399]
Bos taurus UV radiation resistance associated gene (UVRAG), mRNA. [Source:RefSeq mRNA;Acc:NM_001193026]
Bos taurus serpin peptidase inhibitor, clade I (neuroserpin), member 1 (SERPINI1), mRNA. [Source:RefSeq mRNA;Acc:NM_001192238]
Bos taurus prostaglandin E receptor 2 (subtype EP2), 53kDa (PTGER2), mRNA. [Source:RefSeq mRNA;Acc:NM_174588]
PCNA-interacting partner [Source:UniProtKB/Swiss-Prot;Acc:F1MF21]
Bos taurus arrestin domain containing 3 (ARRDC3), mRNA. [Source:RefSeq mRNA;Acc:NM_001076257]
Bos taurus coiled-coil domain containing 127 (CCDC127), mRNA. [Source:RefSeq mRNA;Acc:NM_001103318]
Bos taurus purinergic receptor P2Y, G-protein coupled, 13 (P2RY13), mRNA. [Source:RefSeq mRNA;Acc:NM_001098152]
Bos taurus ADP-ribosylation-like factor 6 interacting protein 6 (ARL6IP6), mRNA. [Source:RefSeq mRNA;Acc:NM_001034281]
stromal interaction molecule 2 [Source:HGNC Symbol;Acc:HGNC:19205]
Bos taurus trans-2,3-enoyl-CoA reductase-like (TECRL), mRNA. [Source:RefSeq mRNA;Acc:NM_001080324]
bicaudal D homolog 1 (Drosophila) [Source:HGNC Symbol;Acc:HGNC:1049]
Bos taurus CD302 molecule (CD302), mRNA. [Source:RefSeq mRNA;Acc:NM_001110191]

Olfactory receptor [Source:UniProtKB/TrEMBL;Acc:E1BF26]
Bos taurus B-cell CLL/lymphoma 10 (BCL10), mRNA. [Source:RefSeq mRNA;Acc:NM_001078028]
Bos taurus phospholamban (PLN), mRNA. [Source:RefSeq mRNA;Acc:NM_001103319]
Bos taurus POU class 2 homeobox 1 (POU2F1), mRNA. [Source:RefSeq mRNA;Acc:NM_001075582]
Bos taurus vasoactive intestinal peptide (VIP), mRNA. [Source:RefSeq mRNA;Acc:NM_173970]
Bos taurus ligand dependent nuclear receptor corepressor-like (LCORL), mRNA. [Source:RefSeq mRNA;Acc:NM_001192357]
Bos taurus RNA (guanine-9-) methyltransferase domain containing 1 (TRMT10C), mRNA. [Source:RefSeq mRNA;Acc:NM_001046323]
chromosome 3 open reading frame 17 [Source:HGNC Symbol;Acc:HGNC:24496]
Bos taurus ecotropic viral integration site 2A (EVI2A), mRNA. [Source:RefSeq mRNA;Acc:NM_001034568]

zinc finger, FYVE domain containing 16 [Source:HGNC Symbol;Acc:HGNC:20756]
Bos taurus family with sequence similarity 188, member A (FAM188A), mRNA. [Source:RefSeq mRNA;Acc:NM_001076107]
Bos taurus dihydrofolate reductase (DHFR), mRNA. [Source:RefSeq mRNA;Acc:NM_001077883]
ubiquitin specific peptidase 14 (tRNA-guanine transglycosylase) [Source:HGNC Symbol;Acc:HGNC:12612]
Bos taurus tumor necrosis factor receptor superfamily, member 21 (TNFRSF21), mRNA. [Source:RefSeq mRNA;Acc:NM_001076911]
Bos taurus gamma-aminobutyric acid (GABA) A receptor, alpha 1 (GABRA1), mRNA. [Source:RefSeq mRNA;Acc:NM_001191119]
Bos taurus sphingomyelin phosphodiesterase, acid-like 3A (SMPDL3A), mRNA. [Source:RefSeq mRNA;Acc:NM_001035019]
Bos taurus transmembrane protein 215 (TMEM215), mRNA. [Source:RefSeq mRNA;Acc:NM_001101071]
Bos taurus solute carrier family 38, member 9 (SLC38A9), mRNA. [Source:RefSeq mRNA;Acc:NM_001102163]
Bos taurus Yip1 domain family, member 6 (YIPF6), mRNA. [Source:RefSeq mRNA;Acc:NM_001101206]
Uncharacterized protein [Source:UniProtKB/TrEMBL;Acc:F1N446]
glucosaminyl (N-acetyl) transferase 4, core 2 [Source:HGNC Symbol;Acc:HGNC:17973]
neurofilament, heavy polypeptide [Source:HGNC Symbol;Acc:HGNC:7737]
A kinase (PRKA) anchor protein 11 [Source:HGNC Symbol;Acc:HGNC:369]
Bos taurus KIAA1279 ortholog (KIAA1279), mRNA. [Source:RefSeq mRNA;Acc:NM_001034559]
Bos taurus fukutin related protein (FKRP), mRNA. [Source:RefSeq mRNA;Acc:NM_001099714]
Bos taurus ubiquitin-conjugating enzyme E2W (putative) (UBE2W), mRNA. [Source:RefSeq mRNA;Acc:NM_001099122]
Bos taurus ankyrin repeat domain 40 (ANKRD40), mRNA. [Source:RefSeq mRNA;Acc:NM_001101109]
zinc finger protein 317 [Source:HGNC Symbol;Acc:HGNC:13507]
coiled-coil domain containing 110 [Source:HGNC Symbol;Acc:HGNC:28504]

Bos taurus oligonucleotide/oligosaccharide-binding fold containing 1 (OBFC1), mRNA. [Source:RefSeq mRNA;Acc:NM_001076849]
zinc finger, CCHC domain containing 5 [Source:HGNC Symbol;Acc:HGNC:22997]
Bos taurus catenin (cadherin-associated protein), alpha-like 1 (CTNNAL1), mRNA. [Source:RefSeq mRNA;Acc:NM_001191534]
Bos taurus POC1 centriolar protein homolog B (Chlamydomonas) (POC1B), mRNA. [Source:RefSeq mRNA;Acc:NM_001099031]
pseudouridylate synthase 10 [Source:HGNC Symbol;Acc:HGNC:26505]
lymphocyte cytosolic protein 2 (SH2 domain containing leukocyte protein of 76kDa) [Source:HGNC Symbol;Acc:HGNC:6529]
Bos taurus protein phosphatase 2, regulatory subunit B, alpha (PPP2R2A), mRNA. [Source:RefSeq mRNA;Acc:NM_001191533]
Bos taurus leucine rich repeat and fibronectin type III domain containing 5 (LRFN5), mRNA. [Source:RefSeq mRNA;Acc:NM_001081610]
family with sequence similarity 161, member A [Source:HGNC Symbol;Acc:HGNC:25808]
KIAA0825 [Source:HGNC Symbol;Acc:HGNC:28532]
Bos taurus kallikrein-related peptidase 10 (KLK10), mRNA. [Source:RefSeq mRNA;Acc:NM_001075890]
Bos taurus proliferating cell nuclear antigen (PCNA), mRNA. [Source:RefSeq mRNA;Acc:NM_001034494]
FGFR1OP N-terminal like [Source:HGNC Symbol;Acc:HGNC:26435]
Bos taurus chondroitin sulfate synthase 3 (CHSY3), mRNA. [Source:RefSeq mRNA;Acc:NM_001192652]
Bos taurus platelet derived growth factor D (PDGFD), mRNA. [Source:RefSeq mRNA;Acc:NM_001083706]
Uncharacterized protein [Source:UniProtKB/TrEMBL;Acc:G3MZ11]
Bos taurus nei endonuclease VIII-like 3 (E. coli) (NEIL3), mRNA. [Source:RefSeq mRNA;Acc:NM_001034490]
Bos taurus sulfotransferase family 1E, estrogen-preferring, member 1 (SULT1E1), mRNA. [Source:RefSeq mRNA;Acc:NM_177488]
Bos taurus 5'-nucleotidase, cytosolic II (NT5C2), mRNA. [Source:RefSeq mRNA;Acc:NM_174405]
Bos taurus fibroblast growth factor 13 (FGF13), mRNA. [Source:RefSeq mRNA;Acc:NM_001098892]
Uncharacterized protein [Source:UniProtKB/TrEMBL;Acc:G3X8F3]
zinc finger with UFM1-specific peptidase domain [Source:HGNC Symbol;Acc:HGNC:21224]
Bos taurus cysteine-rich secretory protein LCCL domain containing 1 (CRISPLD1), mRNA. [Source:RefSeq mRNA;Acc:NM_001101165]
Bos taurus RecQ protein-like (DNA helicase Q1-like) (RECQL), mRNA. [Source:RefSeq mRNA;Acc:NM_001077991]
Bos taurus GINS complex subunit 1 (Psf1 homolog) (GINS1), mRNA. [Source:RefSeq mRNA;Acc:NM_001083701]
Bos taurus helicase (DNA) B (HELB), mRNA. [Source:RefSeq mRNA;Acc:NM_001206182]
Bos taurus squalene epoxidase (SQLE), mRNA. [Source:RefSeq mRNA;Acc:NM_001098061]
Bos taurus scavenger receptor class B, member 2 (SCARB2), mRNA. [Source:RefSeq mRNA;Acc:NM_001102153]
ankyrin repeat domain 11 [Source:HGNC Symbol;Acc:HGNC:21316]
Bos taurus suppressor of cytokine signaling 4 (SOCS4), mRNA. [Source:RefSeq mRNA;Acc:NM_001076218]

Bos taurus F-box and leucine-rich repeat protein 5 (FBXL5), mRNA. [Source:RefSeq mRNA;Acc:NM_001082440]
Bos taurus N-myristoyltransferase 2 (NMT2), mRNA. [Source:RefSeq mRNA;Acc:NM_174456]
programmed cell death 6 interacting protein [Source:HGNC Symbol;Acc:HGNC:8766]
laccase (multicopper oxidoreductase) domain containing 1 [Source:HGNC Symbol;Acc:HGNC:26789]
Bos taurus v-myb myeloblastosis viral oncogene homolog (avian)-like 1 (MYBL1), mRNA. [Source:RefSeq mRNA;Acc:NM_176635]
Bos taurus poly(A) binding protein, cytoplasmic 1 (PABPC1), mRNA. [Source:RefSeq mRNA;Acc:NM_174568]
TTLL11 protein; Uncharacterized protein [Source:UniProtKB/TrEMBL;Acc:A7E2Z5]
Bos taurus zinc finger and BTB domain containing 6 (ZBTB6), mRNA. [Source:RefSeq mRNA;Acc:NM_001076207]
cannabinoid receptor 2 (macrophage) [Source:HGNC Symbol;Acc:HGNC:2160]
Bos taurus RNA binding motif protein 11 (RBM11), mRNA. [Source:RefSeq mRNA;Acc:NM_001040567]
Bos taurus cyclin G1 (CCNG1), mRNA. [Source:RefSeq mRNA;Acc:NM_001013364]
Bos taurus transmembrane protein 173 (TMEM173), mRNA. [Source:RefSeq mRNA;Acc:NM_001046357]
Bos taurus solute carrier family 17 (sodium phosphate), member 2 (SLC17A2), mRNA. [Source:RefSeq mRNA;Acc:NM_001038118]
Uncharacterized protein [Source:UniProtKB/TrEMBL;Acc:F1MLH2]
cytochrome P450, family 2, subfamily B, polypeptide 6 [Source:HGNC Symbol;Acc:HGNC:2615]
Bos taurus oxidized low density lipoprotein (lectin-like) receptor 1 (OLR1), mRNA. [Source:RefSeq mRNA;Acc:NM_174132]
nuclear receptor binding protein 2 [Source:HGNC Symbol;Acc:HGNC:19339]
Bos taurus transient receptor potential cation channel, subfamily C, member 1 (TRPC1), mRNA. [Source:RefSeq mRNA;Acc:NM_174476]
Bos taurus transient receptor potential cation channel, subfamily C, member 3 (TRPC3), mRNA. [Source:RefSeq mRNA;Acc:NM_001104960]
MIS18 binding protein 1 [Source:HGNC Symbol;Acc:HGNC:20190]
Bos taurus tetraspanin 12 (TSPAN12), mRNA. [Source:RefSeq mRNA;Acc:NM_001046512]
TBC1 domain family, member 23 [Source:HGNC Symbol;Acc:HGNC:25622]
Bos taurus toll-like receptor 3 (TLR3), mRNA. [Source:RefSeq mRNA;Acc:NM_001008664]
melanoma associated antigen (mutated) 1-like 1 [Source:HGNC Symbol;Acc:HGNC:26583]
Bos taurus synaptophysin-like 1 (SYPL1), mRNA. [Source:RefSeq mRNA;Acc:NM_001110088]
Bos taurus leptin (LEP), mRNA. [Source:RefSeq mRNA;Acc:NM_173928]
Bos taurus ankyrin repeat domain 46 (ANKRD46), mRNA. [Source:RefSeq mRNA;Acc:NM_001034619]
Uncharacterized protein [Source:UniProtKB/TrEMBL;Acc:F1MV44]
Bardet-Biedl syndrome 10 [Source:HGNC Symbol;Acc:HGNC:26291]
Bos taurus vascular cell adhesion molecule 1 (VCAM1), mRNA. [Source:RefSeq mRNA;Acc:NM_174484]

Bos taurus tumor necrosis factor, alpha-induced protein 8 (TNFAIP8), mRNA. [Source:RefSeq mRNA;Acc:NM_001083711]
Bos taurus GTF2I repeat domain containing 2 (GTF2IRD2), mRNA. [Source:RefSeq mRNA;Acc:NM_001083745]
Uncharacterized protein [Source:UniProtKB/TrEMBL;Acc:F1MFB3]
hepatocyte nuclear factor 4, gamma [Source:HGNC Symbol;Acc:HGNC:5026]
Uncharacterized protein [Source:UniProtKB/TrEMBL;Acc:E1B992]
Bos taurus threonyl-tRNA synthetase (TARS), mRNA. [Source:RefSeq mRNA;Acc:NM_001034370]
Bos taurus vesicle transport through interaction with t-SNAREs homolog 1A (yeast) (VTI1A), mRNA. [Source:RefSeq mRNA;Acc:NM_001079792]
cyclin T2 [Source:HGNC Symbol;Acc:HGNC:1600]
Bos taurus CD38 molecule (CD38), mRNA. [Source:RefSeq mRNA;Acc:NM_175798]
Bos taurus multiple coagulation factor deficiency 2 (MCFD2), mRNA. [Source:RefSeq mRNA;Acc:NM_001040595]
Bos taurus PRP18 pre-mRNA processing factor 18 homolog (S. cerevisiae) (PRPF18), mRNA. [Source:RefSeq mRNA;Acc:NM_001075323]
phosphatidylinositol-specific phospholipase C, X domain containing 3 [Source:HGNC Symbol;Acc:HGNC:31822]
cell cycle progression 1 [Source:HGNC Symbol;Acc:HGNC:24227]
Bos taurus toll-like receptor 7 (TLR7), mRNA. [Source:RefSeq mRNA;Acc:NM_001033761]
Bos taurus protein tyrosine phosphatase-like A domain containing 2 (PTPLAD2), mRNA. [Source:RefSeq mRNA;Acc:NM_001076522]
Bos taurus protein kinase, AMP-activated, alpha 1 catalytic subunit (PRKAA1), mRNA. [Source:RefSeq mRNA;Acc:NM_001109802]
Bos taurus polycomb group ring finger 5 (PCGF5), mRNA. [Source:RefSeq mRNA;Acc:NM_001077980]
Bos taurus histamine N-methyltransferase (HNMT), mRNA. [Source:RefSeq mRNA;Acc:NM_001035434]
Leber congenital amaurosis 5 [Source:HGNC Symbol;Acc:HGNC:31923]
zinc finger protein 800 [Source:HGNC Symbol;Acc:HGNC:27267]
Bos taurus dual adaptor of phosphotyrosine and 3-phosphoinositides (DAPP1), mRNA. [Source:RefSeq mRNA;Acc:NM_001076891]
Bos taurus eukaryotic translation initiation factor 2A, 65kDa (EIF2A), mRNA. [Source:RefSeq mRNA;Acc:NM_001102283]
patatin-like phospholipase domain containing 8 [Source:HGNC Symbol;Acc:HGNC:28900]
Bos taurus ArfGAP with FG repeats 1 (AGFG1), mRNA. [Source:RefSeq mRNA;Acc:NM_001038171]
ankyrin and armadillo repeat containing [Source:HGNC Symbol;Acc:HGNC:26350]
Bos taurus DEAD (Asp-Glu-Ala-Asp) box polypeptide 50 (DDX50), mRNA. [Source:RefSeq mRNA;Acc:NM_001206053]
ATP-binding cassette, sub-family B (MDR/TAP), member 7 [Source:HGNC Symbol;Acc:HGNC:48]
CREB3 regulatory factor [Source:HGNC Symbol;Acc:HGNC:24050]
leucine rich repeat containing 40 [Source:HGNC Symbol;Acc:HGNC:26004]
Bos taurus peroxisomal biogenesis factor 11 alpha (PEX11A), mRNA. [Source:RefSeq mRNA;Acc:NM_001075721]

TBCC domain containing 1 [Source:HGNC Symbol;Acc:HGNC:25546]
MDM2 proto-oncogene, E3 ubiquitin protein ligase [Source:HGNC Symbol;Acc:HGNC:6973]
Bos taurus signal sequence receptor, alpha (SSR1), mRNA. [Source:RefSeq mRNA;Acc:NM_001102122]
leucine-rich repeats and death domain containing 1 [Source:HGNC Symbol;Acc:HGNC:34300]
Bos taurus uncharacterized protein 100125388 (LOC100125388), mRNA. [Source:RefSeq mRNA;Acc:NM_001105028]
Sp3 transcription factor [Source:HGNC Symbol;Acc:HGNC:11208]
Bos taurus cell division cycle 25 homolog C (S. pombe) (CDC25C), mRNA. [Source:RefSeq mRNA;Acc:NM_001097996]
Bos taurus membrane protein, palmitoylated 7 (MAGUK p55 subfamily member 7) (MPP7), mRNA. [Source:RefSeq mRNA;Acc:NM_001100347]
Bos taurus leucine rich repeat containing 8 family, member C (LRRC8C), mRNA. [Source:RefSeq mRNA;Acc:NM_001099039]
Bos taurus cyclic nucleotide gated channel alpha 3 (CNGA3), mRNA. [Source:RefSeq mRNA;Acc:NM_174279]
Bos taurus serine/threonine kinase 3 (STK3), mRNA. [Source:RefSeq mRNA;Acc:NM_001079607]
Bos taurus chromosome 14 open reading frame, human C8orf34 (C14H8orf34), mRNA. [Source:RefSeq mRNA;Acc:NM_001199131]
Bos taurus FERM domain containing 6 (FRMD6), mRNA. [Source:RefSeq mRNA;Acc:NM_001102133]
Bos taurus claudin 16 (CLDN16), mRNA. [Source:RefSeq mRNA;Acc:NM_174519]
Uncharacterized protein [Source:UniProtKB/TrEMBL;Acc:E1B8N1]
F-box/LRR-repeat protein 20 [Source:UniProtKB/Swiss-Prot;Acc:Q58DG6]
Bos taurus gypsy retrotransposon integrase 1 (GIN1), mRNA. [Source:RefSeq mRNA;Acc:NM_001024473]
Bos taurus chemokine (C-C motif) receptor 4 (CCR4), mRNA. [Source:RefSeq mRNA;Acc:NM_001100293]
Bos taurus histone cluster 1, H1d (HIST1H1D), mRNA. [Source:RefSeq mRNA;Acc:NM_001101066]
Bos taurus neuroepithelial cell transforming 1 (NET1), mRNA. [Source:RefSeq mRNA;Acc:NM_001034296]
Bos taurus chromosome 15 open reading frame, human C11orf58 (C15H11orf58), mRNA. [Source:RefSeq mRNA;Acc:NM_001035474]
kelch repeat and BTB (POZ) domain containing 3 [Source:HGNC Symbol;Acc:HGNC:22934]
Bos taurus ring finger protein, LIM domain interacting (RLIM), mRNA. [Source:RefSeq mRNA;Acc:NM_001099728]
Bos taurus protein tyrosine phosphatase, receptor type, R (PTPRR), transcript variant 2, mRNA. [Source:RefSeq mRNA;Acc:NM_001113261]
NOP2/Sun domain family, member 7 [Source:HGNC Symbol;Acc:HGNC:25857]
v-myb avian myeloblastosis viral oncogene homolog [Source:HGNC Symbol;Acc:HGNC:7545]
carboxypeptidase D [Source:HGNC Symbol;Acc:HGNC:2301]
T cell activation inhibitor, mitochondrial [Source:HGNC Symbol;Acc:HGNC:25241]
Bos taurus family with sequence similarity 108, member B1 (FAM108B1), mRNA. [Source:RefSeq mRNA;Acc:NM_001101278]
Bos taurus PL-5283 protein (PL-5283), mRNA. [Source:RefSeq mRNA;Acc:NM_001130930]

zinc finger protein 585A [Source:HGNC Symbol;Acc:HGNC:26305]
cancer susceptibility candidate 1 [Source:HGNC Symbol;Acc:HGNC:29599]
Bos taurus keratin 222 (KRT222), mRNA. [Source:RefSeq mRNA;Acc:NM_001046037]
ring finger protein 128, E3 ubiquitin protein ligase [Source:HGNC Symbol;Acc:HGNC:21153]
solute carrier family 39, member 9 [Source:HGNC Symbol;Acc:HGNC:20182]
Bos taurus ankyrin repeat and SOCS box containing 3 (ASB3), mRNA. [Source:RefSeq mRNA;Acc:NM_001076927]
Bos taurus solute carrier family 35 (UDP-N-acetylglucosamine (UDP-GlcNAc) transporter), member A3 (SLC35A3), mRNA. [Source:RefSeq mRNA;Acc:NM_00110

Bos taurus vacuolar protein sorting 26 homolog A (S. pombe) (VPS26A), mRNA. [Source:RefSeq mRNA;Acc:NM_001075455]
Bos taurus transmembrane protein 136 (TMEM136), mRNA. [Source:RefSeq mRNA;Acc:NM_001075806]
Bos taurus ubiquitin-like 3 (UBL3), mRNA. [Source:RefSeq mRNA;Acc:NM_001038144]
fibrous sheath CABYR binding protein [Source:HGNC Symbol;Acc:HGNC:20494]
Bos taurus kelch-like 13 (Drosophila) (KLHL13), mRNA. [Source:RefSeq mRNA;Acc:NM_001102511]
Bos taurus microtubule associated serine/threonine kinase-like (MASTL), mRNA. [Source:RefSeq mRNA;Acc:NM_001113765]
Bos taurus zinc finger protein 189 (ZNF189), mRNA. [Source:RefSeq mRNA;Acc:NM_001099113]
Bos taurus ubiquitin specific peptidase 15 (USP15), mRNA. [Source:RefSeq mRNA;Acc:NM_001046430]
FK506 binding protein 14, 22 kDa [Source:HGNC Symbol;Acc:HGNC:18625]
Bos taurus RNA binding motif protein 43 (RBM43), mRNA. [Source:RefSeq mRNA;Acc:NM_001099168]
Bos taurus deleted in azoospermia-like (DAZL), mRNA. [Source:RefSeq mRNA;Acc:NM_001081725]
Bos taurus KIAA1383 ortholog (MAP10), mRNA. [Source:RefSeq mRNA;Acc:NM_001098148]
A kinase (PRKA) anchor protein 9 [Source:HGNC Symbol;Acc:HGNC:379]
zinc finger protein 569 [Source:HGNC Symbol;Acc:HGNC:24737]
Bos taurus flavin containing monooxygenase 2 (non-functional) (FMO2), transcript variant 1, mRNA. [Source:RefSeq mRNA;Acc:NM_001163274]
Bos taurus calcium binding protein 39 (CAB39), mRNA. [Source:RefSeq mRNA;Acc:NM_001046087]
Fanconi anemia, complementation group B [Source:HGNC Symbol;Acc:HGNC:3583]
Bos taurus L-2-hydroxyglutarate dehydrogenase (L2HGDH), mRNA. [Source:RefSeq mRNA;Acc:NM_001101090]
ubiquitin specific peptidase 26 [Source:HGNC Symbol;Acc:HGNC:13485]
Bos taurus nucleolar protein 4 (NOL4), transcript variant 1, mRNA. [Source:RefSeq mRNA;Acc:NM_001170532]
zinc finger protein 568 [Source:HGNC Symbol;Acc:HGNC:25392]

SWI/SNF related, matrix associated, actin dependent regulator of chromatin, subfamily a, member 1 [Source:HGNC Symbol;Acc:HGNC:11097]
zinc finger protein 770 [Source:HGNC Symbol;Acc:HGNC:26061]
Bos taurus transmembrane protein 41B (TMEM41B), mRNA. [Source:RefSeq mRNA;Acc:NM_001099156]
Bos taurus solute carrier family 35, member A5 (SLC35A5), mRNA. [Source:RefSeq mRNA;Acc:NM_001076025]
Bos taurus N-acetyltransferase 1 (arylamine N-acetyltransferase) (NAT1), mRNA. [Source:RefSeq mRNA;Acc:NM_001075572]
Bos taurus non-SMC condensin I complex, subunit G (NCAPG), mRNA. [Source:RefSeq mRNA;Acc:NM_001102376]
THAP domain containing, apoptosis associated protein 2 [Source:HGNC Symbol;Acc:HGNC:20854]
Bos taurus spindle assembly 6 homolog (C. elegans) (SASS6), mRNA. [Source:RefSeq mRNA;Acc:NM_001192026]
Bos taurus angiopoietin-like 1 (ANGPTL1), mRNA. [Source:RefSeq mRNA;Acc:NM_001075426]
Bos taurus cleavage stimulation factor, 3' pre-RNA, subunit 1, 50kDa (CSTF1), mRNA. [Source:RefSeq mRNA;Acc:NM_001077865]
Uncharacterized protein [Source:UniProtKB/TrEMBL;Acc:G3X6V5]
Bos taurus NHL repeat containing 2 (NHLRC2), mRNA. [Source:RefSeq mRNA;Acc:NM_001083723]
Bos taurus phosphatidylinositol-4,5-bisphosphate 3-kinase, catalytic subunit alpha (PIK3CA), mRNA. [Source:RefSeq mRNA;Acc:NM_174574]
Bos taurus nucleoporin 43kDa (NUP43), mRNA. [Source:RefSeq mRNA;Acc:NM_001103096]
Uncharacterized protein [Source:UniProtKB/TrEMBL;Acc:E1BJM8]
Bos taurus twisted gastrulation homolog 1 (Drosophila) (TWSG1), mRNA. [Source:RefSeq mRNA;Acc:NM_001076089]
HECT domain and ankyrin repeat containing E3 ubiquitin protein ligase 1 [Source:HGNC Symbol;Acc:HGNC:21033]
chromosome X open reading frame 22 [Source:HGNC Symbol;Acc:HGNC:28546]
interferon induced with helicase C domain 1 [Source:HGNC Symbol;Acc:HGNC:18873]
BUB1 mitotic checkpoint serine/threonine kinase B [Source:HGNC Symbol;Acc:HGNC:1149]
Bos taurus membrane-spanning 4-domains, subfamily A, member 14 (MS4A14), mRNA. [Source:RefSeq mRNA;Acc:NM_001076541]
Bos taurus family with sequence similarity 124B (FAM124B), mRNA. [Source:RefSeq mRNA;Acc:NM_001101870]
Uncharacterized protein [Source:UniProtKB/TrEMBL;Acc:F1MV65]
Bos taurus fem-1 homolog c (C. elegans) (FEM1C), mRNA. [Source:RefSeq mRNA;Acc:NM_001101219]
Bos taurus cysteine-rich secretory protein 3 (CRISP3), mRNA. [Source:RefSeq mRNA;Acc:NM_001079635]
testis expressed 15 [Source:HGNC Symbol;Acc:HGNC:11738]
Bos taurus solute carrier family 17 (sodium-dependent inorganic phosphate cotransporter), member 6 (SLC17A6), mRNA. [Source:RefSeq mRNA;Acc:NM_0011
Bos taurus solute carrier family 1 (glial high affinity glutamate transporter), member 3 (SLC1A3), mRNA. [Source:RefSeq mRNA;Acc:NM_174600]
Bos taurus nudix (nucleoside diphosphate linked moiety X)-type motif 12 (NUDT12), mRNA. [Source:RefSeq mRNA;Acc:NM_001046608]
Bos taurus ATPase family, AAA domain containing 2 (ATAD2), mRNA. [Source:RefSeq mRNA;Acc:NM_001099020]

homeodomain interacting protein kinase 3 [Source:HGNC Symbol;Acc:HGNC:4915]
zinc finger protein 148 [Source:HGNC Symbol;Acc:HGNC:12933]
Bos taurus transmembrane protein 168 (TMEM168), mRNA. [Source:RefSeq mRNA;Acc:NM_001078031]
Bos taurus zinc finger family member 674 (ZNF674), mRNA. [Source:RefSeq mRNA;Acc:NM_001102010]
Uncharacterized protein [Source:UniProtKB/TrEMBL;Acc:F1MGF6]
Bos taurus cleavage and polyadenylation specific factor 2, 100kDa (CPSF2), mRNA. [Source:RefSeq mRNA;Acc:NM_175808]
activating transcription factor 7 interacting protein [Source:HGNC Symbol;Acc:HGNC:20092]
zinc finger, MYM-type 1 [Source:HGNC Symbol;Acc:HGNC:26253]
zinc finger protein 804A [Source:HGNC Symbol;Acc:HGNC:21711]
Bos taurus glia maturation factor, beta (GMFB), mRNA. [Source:RefSeq mRNA;Acc:NM_001101237]
Bos taurus v-yes-1 Yamaguchi sarcoma viral oncogene homolog 1 (YES1), mRNA. [Source:RefSeq mRNA;Acc:NM_001101060]
low density lipoprotein receptor-related protein 1B [Source:HGNC Symbol;Acc:HGNC:6693]
Bos taurus soc-2 suppressor of clear homolog (C. elegans) (SHOC2), mRNA. [Source:RefSeq mRNA;Acc:NM_001101943]
chromosome 9 open reading frame 84 [Source:HGNC Symbol;Acc:HGNC:26535]
Bos taurus SEC24 family, member A (S. cerevisiae) (SEC24A), mRNA. [Source:RefSeq mRNA;Acc:NM_001101849]
IGF-like family member 1 [Source:HGNC Symbol;Acc:HGNC:24093]
RAD52 motif containing 1 [Source:HGNC Symbol;Acc:HGNC:19950]
Bos taurus stromal cell-derived factor 2-like 1 (SDF2L1), mRNA. [Source:RefSeq mRNA;Acc:NM_001035323]
Bos taurus GLI pathogenesis-related 2 (GLIPR2), mRNA. [Source:RefSeq mRNA;Acc:NM_001076112]
centriole, cilia and spindle-associated protein [Source:HGNC Symbol;Acc:HGNC:29578]

39S ribosomal protein L23, mitochondrial [Source:RefSeq peptide;Acc:NP_001071352]
family with sequence similarity 69, member C [Source:HGNC Symbol;Acc:HGNC:31729]
neuronal pentraxin receptor [Source:HGNC Symbol;Acc:HGNC:7954]
Bos taurus UDP-glucose pyrophosphorylase 2 (UGP2), mRNA. [Source:RefSeq mRNA;Acc:NM_174212]
Bos taurus vacuolar protein sorting 54 homolog (S. cerevisiae) (VPS54), mRNA. [Source:RefSeq mRNA;Acc:NM_001105460]
Olfactory receptor [Source:UniProtKB/TrEMBL;Acc:F1MRN4]

Bos taurus fibroblast growth factor 18 (FGF18), mRNA. [Source:RefSeq mRNA;Acc:NM_001076007]
Bos taurus NFU1 iron-sulfur cluster scaffold homolog (S. cerevisiae) (NFU1), mRNA. [Source:RefSeq mRNA;Acc:NM_001046566]

Bos taurus leucine rich repeat and fibronectin type III domain containing 3 (LRFN3), mRNA. [Source:RefSeq mRNA;Acc:NM_001076959]
Bos taurus lectin, galactoside-binding-like (LGALSL), mRNA. [Source:RefSeq mRNA;Acc:NM_001205831]
Bos taurus gonadotropin-releasing hormone 1 (luteinizing-releasing hormone) (GNRH1), mRNA. [Source:RefSeq mRNA;Acc:NM_001078137]
Uncharacterized protein [Source:UniProtKB/TrEMBL;Acc:F1MUM6]

lipoma HMGIC fusion partner-like 3 [Source:HGNC Symbol;Acc:HGNC:6589]
Bos taurus ATPase, H+ transporting, lysosomal 13kDa, V1 subunit G1 (ATP6V1G1), mRNA. [Source:RefSeq mRNA;Acc:NM_174245]
olfactory receptor, family 51, subfamily E, member 1 [Source:HGNC Symbol;Acc:HGNC:15194]
Bos taurus tyrosine 3-monooxygenase/tryptophan 5-monooxygenase activation protein, zeta polypeptide (YWHAZ), mRNA. [Source:RefSeq mRNA;Acc:NM_174
Bos taurus heme binding protein 2 (HEBP2), mRNA. [Source:RefSeq mRNA;Acc:NM_001192213]
NHS-like 1 [Source:HGNC Symbol;Acc:HGNC:21021]
Bos taurus protein FAM228A (FAM228A), mRNA. [Source:RefSeq mRNA;Acc:NM_001077061]
meiosis-specific nuclear structural 1 [Source:HGNC Symbol;Acc:HGNC:29636]
interleukin 5 receptor, alpha [Source:HGNC Symbol;Acc:HGNC:6017]
Uncharacterized protein [Source:UniProtKB/TrEMBL;Acc:F1MGT2]
meiotic nuclear divisions 1 homolog (S. cerevisiae) [Source:HGNC Symbol;Acc:HGNC:24839]
Bos taurus GUF1 GTPase homolog (S. cerevisiae) (GUF1), mRNA. [Source:RefSeq mRNA;Acc:NM_001102067]
Bos taurus zinc finger protein 330 (ZNF330), mRNA. [Source:RefSeq mRNA;Acc:NM_001038157]
Bos taurus glycerol kinase 2-like (LOC538702), mRNA. [Source:RefSeq mRNA;Acc:NM_001046437]
Bos taurus microfibrillar associated protein 5 (MFAP5), mRNA. [Source:RefSeq mRNA;Acc:NM_174386]
Bos taurus isochorismatase domain containing 1 (ISOC1), mRNA. [Source:RefSeq mRNA;Acc:NM_001101213]
Bos taurus sperm acrosome associated 3 (SPACA3), mRNA. [Source:RefSeq mRNA;Acc:NM_001105007]
Bos taurus ras homolog gene family, member G (rho G) (RHOG), mRNA. [Source:RefSeq mRNA;Acc:NM_001080305]

Bos taurus golgi transport 1B (GOLT1B), mRNA. [Source:RefSeq mRNA;Acc:NM_001046215]
sphingosine-1-phosphate receptor 4 [Source:HGNC Symbol;Acc:HGNC:3170]

zinc finger protein 212 [Source:HGNC Symbol;Acc:HGNC:13004]
Bos taurus sarcospan (Kras oncogene-associated gene) (SSPN), mRNA. [Source:RefSeq mRNA;Acc:NM_001193005]
beta-site APP-cleaving enzyme 2 [Source:HGNC Symbol;Acc:HGNC:934]

nanos homolog 3 (Drosophila) [Source:HGNC Symbol;Acc:HGNC:22048]
Bos taurus RNA binding motif protein 4B (RBM4B), mRNA. [Source:RefSeq mRNA;Acc:NM_001045928]
60S ribosomal protein L17 [Source:UniProtKB/Swiss-Prot;Acc:Q3T025]

Bos taurus TAR (HIV-1) RNA binding protein 2 (TARBP2), mRNA. [Source:RefSeq mRNA;Acc:NM_001075678]
Bos taurus heat shock 27kDa protein 2 (HSPB2), mRNA. [Source:RefSeq mRNA;Acc:NM_001098380]
Bos taurus hyaluronoglucosaminidase 1 (HYAL1), mRNA. [Source:RefSeq mRNA;Acc:NM_001017941]
Uncharacterized protein [Source:UniProtKB/TrEMBL;Acc:M0QVY1]
Bos taurus syntrophin, alpha 1 (dystrophin-associated protein A1, 59kDa, acidic component) (SNTA1), mRNA. [Source:RefSeq mRNA;Acc:NM_001075898]
Bos taurus otoraplin (OTOR), mRNA. [Source:RefSeq mRNA;Acc:NM_001037484]
leucine rich repeat containing 28 [Source:HGNC Symbol;Acc:HGNC:28355]
Bos taurus coiled-coil domain containing 104 (CCDC104), mRNA. [Source:RefSeq mRNA;Acc:NM_001034453]
Bos taurus ATP synthase, H+ transporting, mitochondrial F1 complex, delta subunit (ATP5D), mRNA. [Source:RefSeq mRNA;Acc:NM_176670]
Bos taurus phospholipid transfer protein (PLTP), mRNA. [Source:RefSeq mRNA;Acc:NM_001035027]
Uncharacterized protein [Source:UniProtKB/TrEMBL;Acc:G3MXR5]
Bos taurus myeloid differentiation primary response gene (88) (MYD88), mRNA. [Source:RefSeq mRNA;Acc:NM_001014382]
Uncharacterized protein [Source:UniProtKB/TrEMBL;Acc:F1MS03]
calcium channel, voltage-dependent, gamma subunit 4 [Source:HGNC Symbol;Acc:HGNC:1408]
Bos taurus cystatin C (CST3), mRNA. [Source:RefSeq mRNA;Acc:NM_174029]
chromosome 16 open reading frame 90 [Source:HGNC Symbol;Acc:HGNC:34455]
minichromosome maintenance domain containing 2 [Source:HGNC Symbol;Acc:HGNC:26368]
chromatin licensing and DNA replication factor 1 [Source:HGNC Symbol;Acc:HGNC:24576]
Bos taurus adenine phosphoribosyltransferase (APRT), mRNA. [Source:RefSeq mRNA;Acc:NM_001025334]
S100 calcium binding protein A5 [Source:HGNC Symbol;Acc:HGNC:10495]
mirror-image polydactyly 1 [Source:HGNC Symbol;Acc:HGNC:21460]
Bos taurus phosphoglucomutase 3 (PGM3), mRNA. [Source:RefSeq mRNA;Acc:NM_001045922]
WNT1 inducible signaling pathway protein 1 [Source:HGNC Symbol;Acc:HGNC:12769]
Bos taurus cathepsin L1 (CTSL1), mRNA. [Source:RefSeq mRNA;Acc:NM_001083686]
olfactory receptor, family 1, subfamily J, member 1 [Source:HGNC Symbol;Acc:HGNC:8208]
ring finger protein 169 [Source:HGNC Symbol;Acc:HGNC:26961]

Bos taurus BCL2-related protein A1 (BCL2A1), mRNA. [Source:RefSeq mRNA;Acc:NM_001037100]
Bos taurus membrane bound O-acyltransferase domain containing 4 (MBOAT4), mRNA. [Source:RefSeq mRNA;Acc:NM_001192257]
Bos taurus dynactin 6 (DCTN6), mRNA. [Source:RefSeq mRNA;Acc:NM_001075638]
Uncharacterized protein [Source:UniProtKB/TrEMBL;Acc:F1MWG5]
transforming growth factor, alpha [Source:HGNC Symbol;Acc:HGNC:11765]
Bos taurus lymphatic vessel endothelial hyaluronan receptor 1 (LYVE1), mRNA. [Source:RefSeq mRNA;Acc:NM_205815]
Bos taurus IQ motif containing F1 (IQCF1), mRNA. [Source:RefSeq mRNA;Acc:NM_001075773]
Bos taurus transmembrane emp24 protein transport domain containing 3 (TMED3), mRNA. [Source:RefSeq mRNA;Acc:NM_001081609]
Bos taurus COX14 cytochrome c oxidase assembly homolog (S. cerevisiae) (COX14), mRNA. [Source:RefSeq mRNA;Acc:NM_001195064]
Bos taurus guanine nucleotide binding protein (G protein), gamma 11 (GNG11), mRNA. [Source:RefSeq mRNA;Acc:NM_001024523]
G protein-coupled receptor 137C [Source:HGNC Symbol;Acc:HGNC:25445]
Bos taurus calpain 6 (CAPN6), mRNA. [Source:RefSeq mRNA;Acc:NM_001192231]
Bos taurus Tax1 (human T-cell leukemia virus type I) binding protein 3 (TAX1BP3), mRNA. [Source:RefSeq mRNA;Acc:NM_001034474]
Bos taurus keratin 8 (KRT8), mRNA. [Source:RefSeq mRNA;Acc:NM_001033610]

Uncharacterized protein [Source:UniProtKB/TrEMBL;Acc:F1MTY1]
centrosomal protein 131kDa [Source:HGNC Symbol;Acc:HGNC:29511]
chromosome 8 open reading frame 33 [Source:HGNC Symbol;Acc:HGNC:26104]
Bos taurus glycoprotein integral membrane 1 (GINM1), mRNA. [Source:RefSeq mRNA;Acc:NM_001038509]
Uncharacterized protein [Source:UniProtKB/TrEMBL;Acc:F1MK50]
Bos taurus transmembrane protein 17 (TMEM17), mRNA. [Source:RefSeq mRNA;Acc:NM_001083433]
Bos taurus hydroxycarboxylic acid receptor 1 (HCAR1), mRNA. [Source:RefSeq mRNA;Acc:NM_001145234]
Bos taurus ubiquinol-cytochrome c reductase, complex III subunit X (UQCR10), mRNA. [Source:RefSeq mRNA;Acc:NM_001113723]
Bos taurus homeobox C12 (HOXC12), mRNA. [Source:RefSeq mRNA;Acc:NM_001193221]
Bos taurus RAB2A, member RAS oncogene family (RAB2A), mRNA. [Source:RefSeq mRNA;Acc:NM_001075354]
DnaJ (Hsp40) homolog, subfamily C, member 9 [Source:HGNC Symbol;Acc:HGNC:19123]
Janus kinase and microtubule interacting protein 3 [Source:HGNC Symbol;Acc:HGNC:23523]
Bos taurus doublecortin domain containing 1 (DCDC1), mRNA. [Source:RefSeq mRNA;Acc:NM_001076434]
spermatogenesis associated 25 [Source:HGNC Symbol;Acc:HGNC:16158]
Uncharacterized protein [Source:UniProtKB/TrEMBL;Acc:F1MTH2]

Bos taurus keratin associated protein 10-2 (KRTAP10-2), mRNA. [Source:RefSeq mRNA;Acc:NM_001110186]
Bos taurus uncharacterized protein MGC137053 (MGC137053), mRNA. [Source:RefSeq mRNA;Acc:NM_001077085]
Bos taurus mitochondrial ribosomal protein L32 (MRPL32), mRNA. [Source:RefSeq mRNA;Acc:NM_001038679]
Bos taurus bone morphogenetic protein 10 (BMP10), mRNA. [Source:RefSeq mRNA;Acc:NM_001192118]

Bos taurus zinc finger protein 280B (ZNF280B), mRNA. [Source:RefSeq mRNA;Acc:NM_001077935]
solute carrier family 25, member 43 [Source:HGNC Symbol;Acc:HGNC:30557]
small nuclear ribonucleoprotein polypeptides B and B1 [Source:HGNC Symbol;Acc:HGNC:11153]
Bos taurus leucine zipper protein 2 (LUZP2), mRNA. [Source:RefSeq mRNA;Acc:NM_001102336]
Bos taurus parathyroid hormone 2 (PTH2), mRNA. [Source:RefSeq mRNA;Acc:NM_001206281]
coiled-coil domain containing 173 [Source:HGNC Symbol;Acc:HGNC:25064]
Bos taurus FK506 binding protein 7 (FKBP7), mRNA. [Source:RefSeq mRNA;Acc:NM_001100330]
pleckstrin homology domain containing, family A (phosphoinositide binding specific) member 3 [Source:HGNC Symbol;Acc:HGNC:14338]
Bos taurus calcyclin binding protein (CACYBP), mRNA. [Source:RefSeq mRNA;Acc:NM_001034809]
Bos taurus mitochondrial ribosomal protein S14 (MRPS14), mRNA. [Source:RefSeq mRNA;Acc:NM_001003902]
NADH dehydrogenase (ubiquinone) complex I, assembly factor 7 [Source:HGNC Symbol;Acc:HGNC:28816]
small nuclear ribonucleoprotein 48kDa (U11/U12) [Source:HGNC Symbol;Acc:HGNC:21368]
solute carrier family 12, member 9 [Source:HGNC Symbol;Acc:HGNC:17435]
N(alpha)-acetyltransferase 20, NatB catalytic subunit [Source:HGNC Symbol;Acc:HGNC:15908]
transducin-like enhancer of split 6 [Source:HGNC Symbol;Acc:HGNC:30788]
Bos taurus ST3 beta-galactoside alpha-2,3-sialyltransferase 1 (ST3GAL1), mRNA. [Source:RefSeq mRNA;Acc:NM_001097983]
Bos taurus leucine rich repeat neuronal 1 (LRRN1), mRNA. [Source:RefSeq mRNA;Acc:NM_001080738]
Uncharacterized protein [Source:UniProtKB/TrEMBL;Acc:G3X7H4]

NK3 homeobox 1 [Source:HGNC Symbol;Acc:HGNC:7838]
Bos taurus adrenergic, beta-1-, receptor (ADRB1), mRNA. [Source:RefSeq mRNA;Acc:NM_194266]
Bos taurus leukocyte cell-derived chemotaxin 2 (LECT2), mRNA. [Source:RefSeq mRNA;Acc:NM_174380]
Bos taurus mitochondrial ribosomal protein L18 (MRPL18), mRNA. [Source:RefSeq mRNA;Acc:NM_001034608]
Bos taurus mitochondrial ribosomal protein S28 (MRPS28), mRNA. [Source:RefSeq mRNA;Acc:NM_001046401]
Bos taurus rhodopsin (RHO), mRNA. [Source:RefSeq mRNA;Acc:NM_001014890]

Bos taurus RIKEN cDNA 4832428D23-like (METTL21E), mRNA. [Source:RefSeq mRNA;Acc:NM_001014922]
COMM domain containing 8 [Source:HGNC Symbol;Acc:HGNC:26036]
Uncharacterized protein [Source:UniProtKB/TrEMBL;Acc:E1B6Y8]
UDP-GlcNAc:betaGal beta-1,3-N-acetylglucosaminyltransferase 7 [Source:HGNC Symbol;Acc:HGNC:18811]
Bos taurus ribosomal protein S12 (RPS12), mRNA. [Source:RefSeq mRNA;Acc:NM_001014387]
Bos taurus nicotinamide nucleotide adenylyltransferase 1 (NMNAT1), mRNA. [Source:RefSeq mRNA;Acc:NM_001075834]
Bos taurus C1q and tumor necrosis factor related protein 9 (C1QTNF9), mRNA. [Source:RefSeq mRNA;Acc:NM_001075846]
C1q and tumor necrosis factor related protein 2 [Source:HGNC Symbol;Acc:HGNC:14325]
RAD21-like 1 (S. pombe) [Source:HGNC Symbol;Acc:HGNC:16271]
WD repeat domain 73 [Source:HGNC Symbol;Acc:HGNC:25928]
potassium channel tetramerization domain containing 12 [Source:HGNC Symbol;Acc:HGNC:14678]
Bos taurus gamma-aminobutyric acid (GABA) receptor, rho 3 (GABRR3), mRNA. [Source:RefSeq mRNA;Acc:NM_001192519]
Bos taurus abhydrolase domain containing 12 (ABHD12), mRNA. [Source:RefSeq mRNA;Acc:NM_001078116]
Bos taurus high mobility group nucleosomal binding domain 3 (HMGN3), mRNA. [Source:RefSeq mRNA;Acc:NM_001034504]
Uncharacterized protein [Source:UniProtKB/TrEMBL;Acc:F1MPN4]
neuralized E3 ubiquitin protein ligase 1B [Source:HGNC Symbol;Acc:HGNC:35422]
Uncharacterized protein [Source:UniProtKB/TrEMBL;Acc:F1N3X5]
Bos taurus ABRA C-terminal like (ABRACL), mRNA. [Source:RefSeq mRNA;Acc:NM_001166561]
Bos taurus histamine receptor H1 (HRH1), mRNA. [Source:RefSeq mRNA;Acc:NM_174083]
alpha-1,4-N-acetylglucosaminyltransferase [Source:HGNC Symbol;Acc:HGNC:17968]
homeobox A7 [Source:HGNC Symbol;Acc:HGNC:5108]
coiled-coil domain-containing protein 90A, mitochondrial [Source:RefSeq peptide;Acc:NP_001095779]
Uncharacterized protein [Source:UniProtKB/TrEMBL;Acc:F1N3I2]

Bos taurus PPPDE peptidase domain containing 1 (PPPDE1), mRNA. [Source:RefSeq mRNA;Acc:NM_001100384]
Uncharacterized protein [Source:UniProtKB/TrEMBL;Acc:F1MNE2]
B-cell CLL/lymphoma 6 [Source:HGNC Symbol;Acc:HGNC:1001]
Bos taurus ankyrin repeat and SOCS box containing 11 (ASB11), mRNA. [Source:RefSeq mRNA;Acc:NM_001034413]
Bos taurus calcitonin-related polypeptide beta (CALCB), transcript variant 1, mRNA. [Source:RefSeq mRNA;Acc:NM_001134662]
ribosomal RNA adenine dimethylase domain containing 1 [Source:HGNC Symbol;Acc:HGNC:24273]

Uncharacterized protein [Source:UniProtKB/TrEMBL;Acc:F1MN20]
Bos taurus BRI3 binding protein (BRI3BP), mRNA. [Source:RefSeq mRNA;Acc:NM_001099087]
Bos taurus peptidylprolyl isomerase C (cyclophilin C) (PPIC), mRNA. [Source:RefSeq mRNA;Acc:NM_001076910]
Bos taurus deiodinase, iodothyronine, type II (DIO2), mRNA. [Source:RefSeq mRNA;Acc:NM_001010992]
Bos taurus mitogen-activated protein kinase kinase 6 (MAP2K6), mRNA. [Source:RefSeq mRNA;Acc:NM_001034045]
Bos taurus cutA divalent cation tolerance homolog (E. coli) (CUTA), mRNA. [Source:RefSeq mRNA;Acc:NM_001075379]
Bos taurus wingless-type MMTV integration site family, member 7A (WNT7A), mRNA. [Source:RefSeq mRNA;Acc:NM_001192788]

Bos taurus leucine rich repeat containing 52 (LRRC52), mRNA. [Source:RefSeq mRNA;Acc:NM_001206099]

Bos taurus CKLF-like MARVEL transmembrane domain containing 8 (CMTM8), mRNA. [Source:RefSeq mRNA;Acc:NM_001046358]
outer dense fiber of sperm tails 3 [Source:HGNC Symbol;Acc:HGNC:19905]
Bos taurus lumican (LUM), mRNA. [Source:RefSeq mRNA;Acc:NM_173934]
peroxisomal biogenesis factor 3 [Source:HGNC Symbol;Acc:HGNC:8858]
Bos taurus fucosidase, alpha-L- 2, plasma (FUCA2), mRNA. [Source:RefSeq mRNA;Acc:NM_001205818]
Olfactory receptor [Source:UniProtKB/TrEMBL;Acc:E1BLY7]
Bos taurus mitochondrial ribosomal protein S12 (MRPS12), mRNA. [Source:RefSeq mRNA;Acc:NM_001077101]
chromosome 19 open reading frame 70 [Source:HGNC Symbol;Acc:HGNC:33702]
Bos taurus synaptotagmin IV (SYT4), mRNA. [Source:RefSeq mRNA;Acc:NM_001098108]
Bos taurus IQ motif containing D (IQCD), mRNA. [Source:RefSeq mRNA;Acc:NM_001075606]
Bos taurus lactamase, beta 2 (LACTB2), mRNA. [Source:RefSeq mRNA;Acc:NM_001076045]
solute carrier family 25, member 42 [Source:HGNC Symbol;Acc:HGNC:28380]
Sp6 transcription factor [Source:HGNC Symbol;Acc:HGNC:14530]
Bos taurus UL16-binding protein 11 (ULBP11), mRNA. [Source:RefSeq mRNA;Acc:NM_001168610]
Bos taurus docking protein 4 (DOK4), mRNA. [Source:RefSeq mRNA;Acc:NM_001083786]

Serine/threonine-protein phosphatase 2A 55 kDa regulatory subunit B beta isoform [Source:UniProtKB/Swiss-Prot;Acc:Q5E9Q7]
vestigial-like family member 1 [Source:HGNC Symbol;Acc:HGNC:20985]
Bos taurus chromosome 19 open reading frame, human C17orf79 (COPRS), mRNA. [Source:RefSeq mRNA;Acc:NM_001098894]
protein FAM122B [Source:RefSeq peptide;Acc:NP_001077263]

Bos taurus apolipoprotein H (beta-2-glycoprotein I) (APOH), mRNA. [Source:RefSeq mRNA;Acc:NM_173992]
zinc finger protein 652 [Source:HGNC Symbol;Acc:HGNC:29147]
Bos taurus A kinase (PRKA) interacting protein 1 (AKIP1), mRNA. [Source:RefSeq mRNA;Acc:NM_001034203]
transmembrane protease, serine 11D [Source:HGNC Symbol;Acc:HGNC:24059]
Bos taurus RAD23 homolog B (S. cerevisiae) (RAD23B), mRNA. [Source:RefSeq mRNA;Acc:NM_001046310]
Bos taurus carnitine O-acetyltransferase (CRAT), mRNA. [Source:RefSeq mRNA;Acc:NM_001075587]
Bos taurus CDC28 protein kinase regulatory subunit 2 (CKS2), mRNA. [Source:RefSeq mRNA;Acc:NM_001113319]
Bos taurus tRNA methyltransferase 11 homolog (S. cerevisiae) (TRMT11), mRNA. [Source:RefSeq mRNA;Acc:NM_001080273]
protein phosphatase 1, regulatory subunit 17 [Source:HGNC Symbol;Acc:HGNC:16973]
Bos taurus src-related kinase lacking C-terminal regulatory tyrosine and N-terminal myristylation sites (SRMS), mRNA. [Source:RefSeq mRNA;Acc:NM_0011925[illegible]
Bos taurus translocase of inner mitochondrial membrane 8 homolog B (yeast) (TIMM8B), mRNA. [Source:RefSeq mRNA;Acc:NM_001037629]
Uncharacterized protein [Source:UniProtKB/TrEMBL;Acc:F1N1I0]
major facilitator superfamily domain containing 2B [Source:HGNC Symbol;Acc:HGNC:37207]
Bos taurus cytochrome c oxidase subunit VIII-H (heart/muscle) (COX8B), mRNA. [Source:RefSeq mRNA;Acc:NM_001114517]
Bos taurus transcription factor AP-2 gamma (activating enhancer binding protein 2 gamma) (TFAP2C), mRNA. [Source:RefSeq mRNA;Acc:NM_001075509]
Bos taurus MHC class I heavy chain (BOLA), transcript variant 1, mRNA. [Source:RefSeq mRNA;Acc:NM_001038518]
interleukin 36, beta [Source:HGNC Symbol;Acc:HGNC:15564]
Bos taurus cleavage and polyadenylation specific factor 4, 30kDa (CPSF4), mRNA. [Source:RefSeq mRNA;Acc:NM_173942]
Bos taurus ATP synthase, H+ transporting, mitochondrial Fo complex, subunit F2 (ATP5J2), mRNA. [Source:RefSeq mRNA;Acc:NM_001113719]
insulin induced gene 2 [Source:HGNC Symbol;Acc:HGNC:20452]
Bos taurus cyclin-dependent kinase 2 associated protein 2 (CDK2AP2), mRNA. [Source:RefSeq mRNA;Acc:NM_001035320]
family with sequence similarity 109, member A [Source:HGNC Symbol;Acc:HGNC:26509]
Bos taurus SGT1, suppressor of G2 allele of SKP1 (S. cerevisiae) (SUGT1), mRNA. [Source:RefSeq mRNA;Acc:NM_001046203]
Bos taurus interleukin 3 (colony-stimulating factor, multiple) (IL3), mRNA. [Source:RefSeq mRNA;Acc:NM_173920]
Bos taurus DTW domain containing 2 (DTWD2), mRNA. [Source:RefSeq mRNA;Acc:NM_001206125]
Bos taurus interleukin 17A (IL17A), mRNA. [Source:RefSeq mRNA;Acc:NM_001008412]
Bos taurus KH domain containing, RNA binding, signal transduction associated 3 (KHDRBS3), mRNA. [Source:RefSeq mRNA;Acc:NM_001083512]
COX17 cytochrome c oxidase copper chaperone [Source:HGNC Symbol;Acc:HGNC:2264]
chromosome 16 open reading frame 89 [Source:HGNC Symbol;Acc:HGNC:28687]

myocardial zonula adherens protein [Source:HGNC Symbol;Acc:HGNC:43444]
Bos taurus transcription elongation factor B (SIII), polypeptide 1 (15kDa, elongin C) (TCEB1), mRNA. [Source:RefSeq mRNA;Acc:NM_001046493]
Bos taurus YEATS domain containing 4 (YEATS4), mRNA. [Source:RefSeq mRNA;Acc:NM_001076365]
Bos taurus protein tyrosine phosphatase type IVA, member 1 (PTP4A1), mRNA. [Source:RefSeq mRNA;Acc:NM_001206124]
Bos taurus frizzled family receptor 7 (FZD7), mRNA. [Source:RefSeq mRNA;Acc:NM_001144091]
Uncharacterized protein [Source:UniProtKB/TrEMBL;Acc:F1N3T3]
dopachrome tautomerase [Source:HGNC Symbol;Acc:HGNC:2709]
non imprinted in Prader-Willi/Angelman syndrome 1 [Source:HGNC Symbol;Acc:HGNC:17043]
Bos taurus lectin, galactoside-binding, soluble, 3 (LGALS3), mRNA. [Source:RefSeq mRNA;Acc:NM_001102341]
Bos taurus LSM5 homolog, U6 small nuclear RNA associated (S. cerevisiae) (LSM5), mRNA. [Source:RefSeq mRNA;Acc:NM_001046553]
ORAI calcium release-activated calcium modulator 3 [Source:HGNC Symbol;Acc:HGNC:28185]

Uncharacterized protein [Source:UniProtKB/TrEMBL;Acc:F1N4Z6]
Bos taurus nucleobindin 1 (NUCB1), mRNA. [Source:RefSeq mRNA;Acc:NM_001075199]
Bos taurus solute carrier family 35, member E4 (SLC35E4), mRNA. [Source:RefSeq mRNA;Acc:NM_001205905]
Bos taurus neuropeptide FF-amide peptide precursor (NPFF), mRNA. [Source:RefSeq mRNA;Acc:NM_174123]
malonyl CoA:ACP acyltransferase (mitochondrial) [Source:HGNC Symbol;Acc:HGNC:29622]
Bos taurus proteasome (prosome, macropain) 26S subunit, non-ATPase, 12 (PSMD12), mRNA. [Source:RefSeq mRNA;Acc:NM_001046428]
Bos taurus abhydrolase domain containing 13 (ABHD13), mRNA. [Source:RefSeq mRNA;Acc:NM_001101089]
pancreatic polypeptide [Source:HGNC Symbol;Acc:HGNC:9327]

chromosome 19 open reading frame 44 [Source:HGNC Symbol;Acc:HGNC:26141]
mitochondrial ribosomal protein L47 [Source:HGNC Symbol;Acc:HGNC:16652]
Bos taurus NADH dehydrogenase (ubiquinone) 1 beta subcomplex, 5, 16kDa (NDUFB5), mRNA. [Source:RefSeq mRNA;Acc:NM_176656]
Bos taurus family with sequence similarity 173, member A (FAM173A), mRNA. [Source:RefSeq mRNA;Acc:NM_001195829]
Bos taurus ribosomal protein S28 (RPS28), mRNA. [Source:RefSeq mRNA;Acc:NM_001025316]
KN motif and ankyrin repeat domains 3 [Source:HGNC Symbol;Acc:HGNC:24796]
coiled-coil domain containing 78 [Source:HGNC Symbol;Acc:HGNC:14153]
Bos taurus MAD1 mitotic arrest deficient-like 1 (yeast) (MAD1L1), mRNA. [Source:RefSeq mRNA;Acc:NM_001102044]
nudix (nucleoside diphosphate linked moiety X)-type motif 1 [Source:HGNC Symbol;Acc:HGNC:8048]

Bos taurus hairy and enhancer of split 5 (Drosophila) (HES5), mRNA. [Source:RefSeq mRNA;Acc:NM_001105467]
Bos taurus nuclear prelamin A recognition factor-like (NARFL), mRNA. [Source:RefSeq mRNA;Acc:NM_001083411]
synaptonemal complex protein 3 [Source:HGNC Symbol;Acc:HGNC:18130]
Bos taurus ELOVL fatty acid elongase 2 (ELOVL2), mRNA. [Source:RefSeq mRNA;Acc:NM_001083517]
CUE domain containing 2 [Source:HGNC Symbol;Acc:HGNC:28352]
Bos taurus guanine nucleotide binding protein (G protein), gamma 3 (GNG3), mRNA. [Source:RefSeq mRNA;Acc:NM_174073]
Bos taurus coiled-coil domain containing 130 (CCDC130), mRNA. [Source:RefSeq mRNA;Acc:NM_001076344]
Bos taurus methylthioribose-1-phosphate isomerase homolog (S. cerevisiae) (MRI1), mRNA. [Source:RefSeq mRNA;Acc:NM_001046387]
Bos taurus chromosome 7 open reading frame, human C19orf53 (C7H19orf53), mRNA. [Source:RefSeq mRNA;Acc:NM_001076345]
Bos taurus eukaryotic translation elongation factor 1 epsilon 1 (EEF1E1), mRNA. [Source:RefSeq mRNA;Acc:NM_001040599]
Bos taurus PDZK1 interacting protein 1 (PDZK1IP1), mRNA. [Source:RefSeq mRNA;Acc:NM_001046521]
Bos taurus mitochondrial ribosomal protein L19 (MRPL19), mRNA. [Source:RefSeq mRNA;Acc:NM_001046068]
Bos taurus myo-inositol oxygenase (MIOX), mRNA. [Source:RefSeq mRNA;Acc:NM_001101065]
Uncharacterized protein [Source:UniProtKB/TrEMBL;Acc:F1MM67]
Bos taurus myozenin 2 (MYOZ2), mRNA. [Source:RefSeq mRNA;Acc:NM_001015676]
Bos taurus chromosome 17 open reading frame, human C4orf49 (MGARP), mRNA. [Source:RefSeq mRNA;Acc:NM_001166611]
Bos taurus transmembrane and immunoglobulin domain containing 1 (TMIGD1), mRNA. [Source:RefSeq mRNA;Acc:NM_001035036]
Bos taurus Fanconi anemia, complementation group M (FANCM), mRNA. [Source:RefSeq mRNA;Acc:NM_001104976]
Bos taurus RUN and FYVE domain containing 2 (RUFY2), mRNA. [Source:RefSeq mRNA;Acc:NM_001206707]

Bos taurus SAM domain, SH3 domain and nuclear localization signals 1 (SAMSN1), mRNA. [Source:RefSeq mRNA;Acc:NM_001035404]
Bos taurus zinc finger, RAN-binding domain containing 2 (ZRANB2), mRNA. [Source:RefSeq mRNA;Acc:NM_001105346]
septin 9 [Source:HGNC Symbol;Acc:HGNC:7323]
Bos taurus transmembrane protein 115 (TMEM115), mRNA. [Source:RefSeq mRNA;Acc:NM_001083461]
zinc finger, DHHC-type containing 7 [Source:HGNC Symbol;Acc:HGNC:18459]
Uncharacterized protein [Source:UniProtKB/TrEMBL;Acc:E1BH99]
cytoplasmic dynein 1 intermediate chain 2 [Source:RefSeq peptide;Acc:NP_001069351]
Bos taurus fidgetin-like 2 (FIGNL2), mRNA. [Source:RefSeq mRNA;Acc:NM_001214905]
Bos taurus guanine nucleotide binding protein (G protein), gamma transducing activity polypeptide 1 (GNGT1), mRNA. [Source:RefSeq mRNA;Acc:NM_174327]
coiled-coil domain containing 7 [Source:HGNC Symbol;Acc:HGNC:26533]

Bos taurus mitochondrial ribosomal protein L42 (MRPL42), mRNA. [Source:RefSeq mRNA;Acc:NM_001037622]
Bos taurus magnesium-dependent phosphatase 1 (MDP-1), transcript variant 1, mRNA. [Source:RefSeq mRNA;Acc:NM_001164221]
Bos taurus HORMA domain containing 1 (HORMAD1), mRNA. [Source:RefSeq mRNA;Acc:NM_001046305]
Bos taurus chromosome 25 open reading frame, human C16orf13 (C25H16orf13), mRNA. [Source:RefSeq mRNA;Acc:NM_001038106]
phosphodiesterase 1C, calmodulin-dependent 70kDa [Source:HGNC Symbol;Acc:HGNC:8776]

Bos taurus osteoclast stimulating factor 1 (OSTF1), mRNA. [Source:RefSeq mRNA;Acc:NM_174409]
V-set and transmembrane domain containing 5 [Source:HGNC Symbol;Acc:HGNC:34443]
Uncharacterized protein [Source:UniProtKB/TrEMBL;Acc:F1MG16]
Ras-related protein Rab-27B [Source:UniProtKB/Swiss-Prot;Acc:Q8HZJ5]
Bos taurus proteasome (prosome, macropain) subunit, alpha type, 3 (PSMA3), mRNA. [Source:RefSeq mRNA;Acc:NM_001034235]
calpain 10 [Source:HGNC Symbol;Acc:HGNC:1477]
Bos taurus meiosis expressed gene 1 homolog (mouse) (MEIG1), transcript variant 1, mRNA. [Source:RefSeq mRNA;Acc:NM_001114524]
lamin-B1 [Source:RefSeq peptide;Acc:NP_001096765]
tumor necrosis factor (ligand) superfamily, member 4 [Source:HGNC Symbol;Acc:HGNC:11934]
Bos taurus atonal homolog 1 (Drosophila) (ATOH1), mRNA. [Source:RefSeq mRNA;Acc:NM_001098099]
Bos taurus zinc finger protein 787 (ZNF787), mRNA. [Source:RefSeq mRNA;Acc:NM_001206139]
protein kinase, AMP-activated, gamma 2 non-catalytic subunit [Source:HGNC Symbol;Acc:HGNC:9386]
unc-5 homolog C (C. elegans)-like [Source:HGNC Symbol;Acc:HGNC:21203]
amelotin [Source:HGNC Symbol;Acc:HGNC:33188]
bestrophin 3 [Source:HGNC Symbol;Acc:HGNC:17105]
paired related homeobox 2 [Source:HGNC Symbol;Acc:HGNC:21338]
Bos taurus solute carrier family 2 (facilitated glucose transporter), member 10 (SLC2A10), mRNA. [Source:RefSeq mRNA;Acc:NM_001192439]
CD244 molecule, natural killer cell receptor 2B4 [Source:HGNC Symbol;Acc:HGNC:18171]
Uncharacterized protein [Source:UniProtKB/TrEMBL;Acc:F1MUX8]
Bos taurus 5'-nucleotidase, cytosolic IA (NT5C1A), mRNA. [Source:RefSeq mRNA;Acc:NM_001205546]
leucine rich transmembrane and O-methyltransferase domain containing [Source:HGNC Symbol;Acc:HGNC:25033]
Uncharacterized protein [Source:UniProtKB/TrEMBL;Acc:E1BDR1]
crystallin, gamma N [Source:HGNC Symbol;Acc:HGNC:20458]
Bos taurus cathelicidin antimicrobial peptide (CAMP), mRNA. [Source:RefSeq mRNA;Acc:NM_174831]

Olfactory receptor [Source:UniProtKB/TrEMBL;Acc:M0QW10]
Bos taurus guanine nucleotide binding protein (G protein), gamma 2 (GNG2), mRNA. [Source:RefSeq mRNA;Acc:NM_174072]
Bos taurus N(alpha)-acetyltransferase 40, NatD catalytic subunit, homolog (S. cerevisiae) (NAA40), mRNA. [Source:RefSeq mRNA;Acc:NM_001099004]
Bos taurus RWD domain containing 4 (RWDD4), mRNA. [Source:RefSeq mRNA;Acc:NM_001076824]
ribosomal protein, large, P2 [Source:HGNC Symbol;Acc:HGNC:10377]
olfactory receptor, family 6, subfamily K, member 3 [Source:HGNC Symbol;Acc:HGNC:15030]
eukaryotic translation initiation factor 4E family member 1B [Source:HGNC Symbol;Acc:HGNC:33179]
Bos taurus integral membrane protein 2B (ITM2B), mRNA. [Source:RefSeq mRNA;Acc:NM_001035093]
THUMP domain containing 3 [Source:HGNC Symbol;Acc:HGNC:24493]
AVL9 homolog (S. cerevisiase) [Source:HGNC Symbol;Acc:HGNC:28994]

Bos taurus crystallin, alpha A (CRYAA), mRNA. [Source:RefSeq mRNA;Acc:NM_174289]
IQ motif containing J [Source:HGNC Symbol;Acc:HGNC:32406]
Bos taurus interferon, alpha-inducible protein 27-like 2 (IFI27L2), mRNA. [Source:RefSeq mRNA;Acc:NM_001076061]
UDP-Gal:betaGlcNAc beta 1,3-galactosyltransferase, polypeptide 1 [Source:HGNC Symbol;Acc:HGNC:916]
Bos taurus receptor (chemosensory) transporter protein 2 (RTP2), mRNA. [Source:RefSeq mRNA;Acc:NM_001192316]
bifunctional 3'-phosphoadenosine 5'-phosphosulfate synthase 2 [Source:RefSeq peptide;Acc:NP_001069543]

Bos taurus Duffy blood group, chemokine receptor (DARC), mRNA. [Source:RefSeq mRNA;Acc:NM_001015634]
Bos taurus ribosomal protein L3 (RPL3), mRNA. [Source:RefSeq mRNA;Acc:NM_174715]
Bos taurus lens intrinsic membrane protein 2, 19kDa (LIM2), mRNA. [Source:RefSeq mRNA;Acc:NM_174102]
Uncharacterized protein [Source:UniProtKB/TrEMBL;Acc:F1MGV4]
Bos taurus chromosome 8 open reading frame, human C9orf152 (C8H9orf152), mRNA. [Source:RefSeq mRNA;Acc:NM_001206177]
T-cell surface glycoprotein CD4 precursor [Source:RefSeq peptide;Acc:NP_001096695]

Bos taurus tubulin folding cofactor A (TBCA), mRNA. [Source:RefSeq mRNA;Acc:NM_175803]
nuclear receptor subfamily 1, group H, member 2 [Source:HGNC Symbol;Acc:HGNC:7965]
Uncharacterized protein [Source:UniProtKB/TrEMBL;Acc:E1BGB8]
Bos taurus golgin, RAB6-interacting (GORAB), mRNA. [Source:RefSeq mRNA;Acc:NM_001167897]
neural cell adhesion molecule 2 [Source:HGNC Symbol;Acc:HGNC:7657]

Bos taurus neutrophil cytosolic factor 1 (NCF1), mRNA. [Source:RefSeq mRNA;Acc:NM_174119]
Bos taurus mitochondrial ribosomal protein 63 (MRP63), mRNA. [Source:RefSeq mRNA;Acc:NM_001034787]
Uncharacterized protein [Source:UniProtKB/TrEMBL;Acc:G5E552]
Bos taurus defensin, beta 119 (DEFB119), mRNA. [Source:RefSeq mRNA;Acc:NM_001101661]
synaptotagmin XII [Source:HGNC Symbol;Acc:HGNC:18381]
Uncharacterized protein [Source:UniProtKB/TrEMBL;Acc:G5E666]
Bos taurus phosphatidylinositol-4-phosphate 5-kinase-like 1 (PIP5KL1), mRNA. [Source:RefSeq mRNA;Acc:NM_001075847]
Bos taurus dolichyl-phosphate mannosyltransferase polypeptide 2, regulatory subunit (DPM2), mRNA. [Source:RefSeq mRNA;Acc:NM_001046268]
Uncharacterized protein [Source:UniProtKB/TrEMBL;Acc:E1BDW5]
Bos taurus chromosome 3 open reading frame, human C1orf123 (C3H1orf123), mRNA. [Source:RefSeq mRNA;Acc:NM_001038130]
Uncharacterized protein [Source:UniProtKB/TrEMBL;Acc:G5E557]

Bos taurus DDB1 and CUL4 associated factor 7 (DCAF7), mRNA. [Source:RefSeq mRNA;Acc:NM_001038125]
Bos taurus chromosome 18 open reading frame, human C16orf87 (C18H16orf87), mRNA. [Source:RefSeq mRNA;Acc:NM_001078045]
Bos taurus decorin (DCN), mRNA. [Source:RefSeq mRNA;Acc:NM_173906]
Bos taurus pleckstrin homology domain containing, family A (phosphoinositide binding specific) member 2 (PLEKHA2), mRNA. [Source:RefSeq mRNA;Acc:NM_0[illegible]
Bos taurus dolichyl-phosphate mannosyltransferase polypeptide 3 (DPM3), mRNA. [Source:RefSeq mRNA;Acc:NM_001034360]
Bos taurus TATA box binding protein (TBP)-associated factor, RNA polymerase I, D, 41kDa (TAF1D), mRNA. [Source:RefSeq mRNA;Acc:NM_001037586]
Bos taurus glutathione S-transferase pi 1 (GSTP1), mRNA. [Source:RefSeq mRNA;Acc:NM_177516]
ZFP36 ring finger protein-like 2 [Source:HGNC Symbol;Acc:HGNC:1108]
Uncharacterized protein [Source:UniProtKB/TrEMBL;Acc:E1BHY4]
serine/threonine kinase 35 [Source:HGNC Symbol;Acc:HGNC:16254]
KIAA1644 [Source:HGNC Symbol;Acc:HGNC:29335]
Uncharacterized protein [Source:UniProtKB/TrEMBL;Acc:E1BHH2]
olfactory receptor, family 51, subfamily I, member 1 [Source:HGNC Symbol;Acc:HGNC:15200]
Bos taurus COMM domain containing 3 (COMMD3), mRNA. [Source:RefSeq mRNA;Acc:NM_001076999]
ciliary neurotrophic factor [Source:HGNC Symbol;Acc:HGNC:2169]
Bos taurus cationic trypsin (LOC780933), mRNA. [Source:RefSeq mRNA;Acc:NM_001113727]
Bos taurus peroxiredoxin 1 (PRDX1), mRNA. [Source:RefSeq mRNA;Acc:NM_174431]
Bos taurus transmembrane protein 128 (TMEM128), mRNA. [Source:RefSeq mRNA;Acc:NM_001034454]

Uncharacterized protein [Source:UniProtKB/TrEMBL;Acc:G5E562]
Olfactory receptor [Source:UniProtKB/TrEMBL;Acc:F1MUS4]
neurotensin receptor 2 [Source:HGNC Symbol;Acc:HGNC:8040]
Bos taurus vasohibin 2 (VASH2), mRNA. [Source:RefSeq mRNA;Acc:NM_001079611]
Bos taurus GRB2-related adaptor protein (GRAP), mRNA. [Source:RefSeq mRNA;Acc:NM_001102173]
Bos taurus family with sequence similarity 89, member B (FAM89B), mRNA. [Source:RefSeq mRNA;Acc:NM_001075517]
Uncharacterized protein [Source:UniProtKB/TrEMBL;Acc:E1BCG6]
Bos taurus transmembrane 4 L six family member 5 (TM4SF5), mRNA. [Source:RefSeq mRNA;Acc:NM_001046102]

Bos taurus akirin 2 (AKIRIN2), mRNA. [Source:RefSeq mRNA;Acc:NM_001110087]
complement component 1, q subcomponent-like 2 [Source:HGNC Symbol;Acc:HGNC:24181]
Bos taurus tektin 3 (TEKT3), mRNA. [Source:RefSeq mRNA;Acc:NM_001099019]
microfibrillar-associated protein 2 [Source:HGNC Symbol;Acc:HGNC:7033]
insulin-like 5 [Source:HGNC Symbol;Acc:HGNC:6088]
KIAA0247 [Source:HGNC Symbol;Acc:HGNC:19956]
Uncharacterized protein [Source:UniProtKB/TrEMBL;Acc:E1BIQ3]
ER membrane protein complex subunit 3 [Source:RefSeq peptide;Acc:NP_001030228]
retinitis pigmentosa GTPase regulator [Source:HGNC Symbol;Acc:HGNC:10295]
Bos taurus cytochrome b-245, alpha polypeptide (CYBA), mRNA. [Source:RefSeq mRNA;Acc:NM_174034]

Bos taurus family with sequence similarity 125, member A (MVB12A), mRNA. [Source:RefSeq mRNA;Acc:NM_001034283]
transglutaminase 1 [Source:HGNC Symbol;Acc:HGNC:11777]
Bos taurus G protein-coupled bile acid receptor 1 (GPBAR1), mRNA. [Source:RefSeq mRNA;Acc:NM_175049]
Olfactory receptor [Source:UniProtKB/TrEMBL;Acc:G5E565]
Uncharacterized protein [Source:UniProtKB/TrEMBL;Acc:E1BGR6]
Bos taurus phosphatidylinositol-5-phosphate 4-kinase, type II, gamma (PIP4K2C), mRNA. [Source:RefSeq mRNA;Acc:NM_001076196]
Bos taurus F-box and leucine-rich repeat protein 2 (FBXL2), mRNA. [Source:RefSeq mRNA;Acc:NM_001099153]
Bos taurus sarcoglycan, alpha (50kDa dystrophin-associated glycoprotein) (SGCA), mRNA. [Source:RefSeq mRNA;Acc:NM_001013402]
Uncharacterized protein [Source:UniProtKB/TrEMBL;Acc:E1BGA3]
Bos taurus transmembrane protein 220 (TMEM220), mRNA. [Source:RefSeq mRNA;Acc:NM_001192043]

chromosome 20 open reading frame 144 [Source:HGNC Symbol;Acc:HGNC:16137]
Bos taurus CXXC finger protein 5 (CXXC5), mRNA. [Source:RefSeq mRNA;Acc:NM_001038176]
Uncharacterized protein [Source:UniProtKB/TrEMBL;Acc:F1MFP0]
Bos taurus insulin-like growth factor binding protein 3 (IGFBP3), mRNA. [Source:RefSeq mRNA;Acc:NM_174556]
deltex 4, E3 ubiquitin ligase [Source:HGNC Symbol;Acc:HGNC:29151]

Bos taurus poly(A) polymerase alpha (PAPOLA), mRNA. [Source:RefSeq mRNA;Acc:NM_176647]
protein phosphatase 2C-like domain containing 1 [Source:HGNC Symbol;Acc:HGNC:28406]
Bos taurus metastasis associated 1 family, member 3 (MTA3), mRNA. [Source:RefSeq mRNA;Acc:NM_001101964]
Bos taurus isopentenyl-diphosphate delta isomerase 1 (IDI1), mRNA. [Source:RefSeq mRNA;Acc:NM_001075659]

Tubulin beta-2B chain [Source:UniProtKB/Swiss-Prot;Acc:Q6B856]
family with sequence similarity 166, member A [Source:HGNC Symbol;Acc:HGNC:33818]
Bos taurus PRKR interacting protein 1 (IL11 inducible) (PRKRIP1), mRNA. [Source:RefSeq mRNA;Acc:NM_001079641]
Bos taurus guanidinoacetate N-methyltransferase (GAMT), mRNA. [Source:RefSeq mRNA;Acc:NM_001038544]
Bos taurus aminolevulinate, delta-, synthase 1 (ALAS1), mRNA. [Source:RefSeq mRNA;Acc:NM_001101154]
HemK methyltransferase family member 1 [Source:HGNC Symbol;Acc:HGNC:24923]
chromosome 8 open reading frame 74 [Source:HGNC Symbol;Acc:HGNC:32296]
Bos taurus secretory leukocyte peptidase inhibitor (SLPI), mRNA. [Source:RefSeq mRNA;Acc:NM_001098865]
Uncharacterized protein [Source:UniProtKB/TrEMBL;Acc:G3X6E4]
Bos taurus secretoglobin, family 3A, member 2 (SCGB3A2), mRNA. [Source:RefSeq mRNA;Acc:NM_001205460]
SIX homeobox 2 [Source:HGNC Symbol;Acc:HGNC:10888]
Bos taurus UBX domain protein 8 (UBXN8), mRNA. [Source:RefSeq mRNA;Acc:NM_001038672]
jumonji domain containing 4 [Source:HGNC Symbol;Acc:HGNC:25724]
UDP-GlcNAc:betaGal beta-1,3-N-acetylglucosaminyltransferase 4 [Source:HGNC Symbol;Acc:HGNC:15683]
Bos taurus diablo, IAP-binding mitochondrial protein (DIABLO), mRNA. [Source:RefSeq mRNA;Acc:NM_001045882]
Bos taurus vacuolar protein sorting 33 homolog A (S. cerevisiae) (VPS33A), mRNA. [Source:RefSeq mRNA;Acc:NM_001046049]
leucine rich repeat containing 55 [Source:HGNC Symbol;Acc:HGNC:32324]
Uncharacterized protein [Source:UniProtKB/TrEMBL;Acc:F1MSX6]
Bos taurus retinoic acid receptor responder (tazarotene induced) 2 (RARRES2), mRNA. [Source:RefSeq mRNA;Acc:NM_001046020]

Uncharacterized protein [Source:UniProtKB/TrEMBL;Acc:G3X6E7]
Bos taurus aminoacyl tRNA synthetase complex-interacting multifunctional protein 1 (AIMP1), mRNA. [Source:RefSeq mRNA;Acc:NM_001035018]
Bos taurus spondin 2, extracellular matrix protein (SPON2), mRNA. [Source:RefSeq mRNA;Acc:NM_001046157]
coiled-coil domain containing 148 [Source:HGNC Symbol;Acc:HGNC:25191]
trafficking protein particle complex 5 [Source:HGNC Symbol;Acc:HGNC:23067]
Bos taurus TLH29 protein precursor-like (ISG12(B)), mRNA. [Source:RefSeq mRNA;Acc:NM_001076457]
cystatin F (leukocystatin) [Source:HGNC Symbol;Acc:HGNC:2479]
Ellis van Creveld syndrome [Source:HGNC Symbol;Acc:HGNC:3497]
homeobox D13 [Source:HGNC Symbol;Acc:HGNC:5136]
Bos taurus ADP-ribosylation factor-like 3 (ARL3), mRNA. [Source:RefSeq mRNA;Acc:NM_001038567]
Bos taurus sideroflexin 2 (SFXN2), mRNA. [Source:RefSeq mRNA;Acc:NM_001034446]
Bos taurus RELT-like 1 (RELL1), mRNA. [Source:RefSeq mRNA;Acc:NM_001078098]
mitochondrial ribosomal protein L21 [Source:HGNC Symbol;Acc:HGNC:14479]
threonine synthase-like 2 (S. cerevisiae) [Source:HGNC Symbol;Acc:HGNC:25602]
Olfactory receptor [Source:UniProtKB/TrEMBL;Acc:E1B938]
interleukin 23, alpha subunit p19 [Source:HGNC Symbol;Acc:HGNC:15488]
tumor-associated calcium signal transducer 2 [Source:HGNC Symbol;Acc:HGNC:11530]
Bos taurus protein kinase (cAMP-dependent, catalytic) inhibitor beta (PKIB), mRNA. [Source:RefSeq mRNA;Acc:NM_001114518]
Bos taurus IMP2 inner mitochondrial membrane peptidase-like (S. cerevisiae) (IMMP2L), mRNA. [Source:RefSeq mRNA;Acc:NM_001077093]
Bos taurus slowmo homolog 2 (Drosophila) (SLMO2), mRNA. [Source:RefSeq mRNA;Acc:NM_001035476]
IQ motif and ubiquitin domain containing [Source:HGNC Symbol;Acc:HGNC:21995]
musashi RNA-binding protein 2 [Source:HGNC Symbol;Acc:HGNC:18585]
transmembrane protease, serine 11F [Source:HGNC Symbol;Acc:HGNC:29994]
Bos taurus solute carrier family 30, member 10 (SLC30A10), mRNA. [Source:RefSeq mRNA;Acc:NM_001192180]

Bos taurus phospholipase A2, group IIE (PLA2G2E), mRNA. [Source:RefSeq mRNA;Acc:NM_001192086]
Bos taurus asparagine-linked glycosylation 14 homolog (S. cerevisiae) (ALG14), mRNA. [Source:RefSeq mRNA;Acc:NM_001077863]
Bos taurus growth differentiation factor 5 (GDF5), mRNA. [Source:RefSeq mRNA;Acc:NM_001192273]

Uncharacterized protein [Source:UniProtKB/TrEMBL;Acc:G5E566]

Bos taurus transmembrane protein 45A (TMEM45A), mRNA. [Source:RefSeq mRNA;Acc:NM_001075405]
BARX homeobox 1 [Source:HGNC Symbol;Acc:HGNC:955]
Bos taurus dynein, light chain, Tctex-type 1 (DYNLT1), mRNA. [Source:RefSeq mRNA;Acc:NM_174620]
Bos taurus cyclin-dependent kinase 5, regulatory subunit 1 (p35) (CDK5R1), mRNA. [Source:RefSeq mRNA;Acc:NM_174512]
probable E3 ubiquitin-protein ligase makorin-2 [Source:RefSeq peptide;Acc:NP_001039454]
Bos taurus potassium channel, subfamily K, member 1 (KCNK1), mRNA. [Source:RefSeq mRNA;Acc:NM_001075207]
LSM7 homolog, U6 small nuclear RNA associated (S. cerevisiae) [Source:HGNC Symbol;Acc:HGNC:20470]

FGFR1 oncogene partner 2 [Source:HGNC Symbol;Acc:HGNC:23098]
Bos taurus lysophosphatidic acid receptor 5 (LPAR5), mRNA. [Source:RefSeq mRNA;Acc:NM_001034304]
thioredoxin domain containing 9 [Source:HGNC Symbol;Acc:HGNC:24110]

Bos taurus C1D nuclear receptor corepressor (C1D), mRNA. [Source:RefSeq mRNA;Acc:NM_001037448]
Bos taurus GLI pathogenesis-related 1 like 1 (GLIPR1L1), mRNA. [Source:RefSeq mRNA;Acc:NM_001076357]
Bos taurus UDP glycosyltransferase 8 (UGT8), mRNA. [Source:RefSeq mRNA;Acc:NM_001083635]
leucine-rich repeat LGI family, member 3 [Source:HGNC Symbol;Acc:HGNC:18711]
Bos taurus membrane-spanning 4-domains, subfamily A, member 5 (MS4A5), mRNA. [Source:RefSeq mRNA;Acc:NM_001078146]
Uncharacterized protein [Source:UniProtKB/TrEMBL;Acc:F1MU59]
Bos taurus matrix metallopeptidase 7 (matrilysin, uterine) (MMP7), mRNA. [Source:RefSeq mRNA;Acc:NM_001075130]
Bos taurus transgelin 3 (TAGLN3), mRNA. [Source:RefSeq mRNA;Acc:NM_001034499]
Bos taurus abhydrolase domain containing 10 (ABHD10), mRNA. [Source:RefSeq mRNA;Acc:NM_001015606]
Bos taurus coiled-coil domain containing 124 (CCDC124), mRNA. [Source:RefSeq mRNA;Acc:NM_001038523]
Uncharacterized protein [Source:UniProtKB/TrEMBL;Acc:F1MTZ8]
Bos taurus non-metastatic cells 1, protein (NM23A) expressed in (NME1), transcript variant 2, mRNA. [Source:RefSeq mRNA;Acc:NM_205777]
fibrillarin-like 1 [Source:HGNC Symbol;Acc:HGNC:35458]
coenzyme Q4 [Source:HGNC Symbol;Acc:HGNC:19693]
Olfactory receptor [Source:UniProtKB/TrEMBL;Acc:E1BHP8]

Uncharacterized protein [Source:UniProtKB/TrEMBL;Acc:G5E549]

mast cell-expressed membrane protein 1 [Source:HGNC Symbol;Acc:HGNC:27291]
Bos taurus resistin (RETN), mRNA. [Source:RefSeq mRNA;Acc:NM_183362]
Uncharacterized protein [Source:UniProtKB/TrEMBL;Acc:F1MFT8]
Bos taurus V-set and transmembrane domain containing 2 like (VSTM2L), mRNA. [Source:RefSeq mRNA;Acc:NM_001077976]
Uncharacterized protein [Source:UniProtKB/TrEMBL;Acc:G3X6G6]

Bos taurus dual specificity phosphatase 23 (DUSP23), mRNA. [Source:RefSeq mRNA;Acc:NM_001082609]
Bos taurus protein tyrosine phosphatase, receptor type, C-associated protein (PTPRCAP), mRNA. [Source:RefSeq mRNA;Acc:NM_001046618]
Bos taurus calcium binding protein 4 (CABP4), mRNA. [Source:RefSeq mRNA;Acc:NM_174256]
Bos taurus meteorin, glial cell differentiation regulator-like (METRNL), mRNA. [Source:RefSeq mRNA;Acc:NM_001206050]
Bos taurus ameloblastin (enamel matrix protein) (AMBN), mRNA. [Source:RefSeq mRNA;Acc:NM_173988]
tRNA methyltransferase 44 homolog (S. cerevisiae) [Source:HGNC Symbol;Acc:HGNC:26653]
Bos taurus integrin alpha FG-GAP repeat containing 1 (ITFG1), mRNA. [Source:RefSeq mRNA;Acc:NM_001038532]
olfactory receptor, family 4, subfamily K, member 14 [Source:HGNC Symbol;Acc:HGNC:15352]
Bos taurus mitochondrial ribosomal protein L35 (MRPL35), mRNA. [Source:RefSeq mRNA;Acc:NM_001192391]
Bos taurus ADP-ribosylation factor-like 2 binding protein (ARL2BP), mRNA. [Source:RefSeq mRNA;Acc:NM_001076274]
olfactomedin-like 2A [Source:HGNC Symbol;Acc:HGNC:27270]
Bos taurus solute carrier family 24, member 5 (SLC24A5), mRNA. [Source:RefSeq mRNA;Acc:NM_001128509]
transmembrane protein 230 [Source:HGNC Symbol;Acc:HGNC:15876]
Bos taurus keratin 19 (KRT19), mRNA. [Source:RefSeq mRNA;Acc:NM_001015600]
Bos taurus cyclin E2 (CCNE2), mRNA. [Source:RefSeq mRNA;Acc:NM_001015665]
Bos taurus cytochrome c oxidase subunit VIIIA (ubiquitous) (COX8A), mRNA. [Source:RefSeq mRNA;Acc:NM_174024]
Uncharacterized protein [Source:UniProtKB/TrEMBL;Acc:F1MJ44]
Bos taurus leucine rich repeat protein 1 (LRR1), mRNA. [Source:RefSeq mRNA;Acc:NM_001105025]
chromosome 5 open reading frame 51 [Source:HGNC Symbol;Acc:HGNC:27750]
Bos taurus Sec61 alpha 1 subunit (S. cerevisiae) (SEC61A1), mRNA. [Source:RefSeq mRNA;Acc:NM_001040504]
Bos taurus cyclin A2 (CCNA2), mRNA. [Source:RefSeq mRNA;Acc:NM_001075123]
Bos taurus ELK4, ETS-domain protein (SRF accessory protein 1) (ELK4), mRNA. [Source:RefSeq mRNA;Acc:NM_001081612]
protein tyrosine phosphatase, receptor type, N polypeptide 2 [Source:HGNC Symbol;Acc:HGNC:9677]
Bos taurus NTF2-like export factor 1 (NXT1), mRNA. [Source:RefSeq mRNA;Acc:NM_001075805]

Bos taurus family with sequence similarity 206, member A (FAM206A), mRNA. [Source:RefSeq mRNA;Acc:NM_001045956]

Bos taurus double C2-like domains, alpha (DOC2A), mRNA. [Source:RefSeq mRNA;Acc:NM_001083709]
Bos taurus calcium and integrin binding family member 4 (CIB4), mRNA. [Source:RefSeq mRNA;Acc:NM_001076455]
Bos taurus synapsin I (SYN1), mRNA. [Source:RefSeq mRNA;Acc:NM_174191]
Bos taurus gastric inhibitory polypeptide (GIP), mRNA. [Source:RefSeq mRNA;Acc:NM_001166605]
Bos taurus dehydrogenase/reductase (SDR family) member 7C (DHRS7C), mRNA. [Source:RefSeq mRNA;Acc:NM_001075545]
Uncharacterized protein [Source:UniProtKB/TrEMBL;Acc:F1MEI3]
glucagon-like peptide 2 receptor [Source:HGNC Symbol;Acc:HGNC:4325]
Bos taurus family with sequence similarity 204, member A (FAM204A), mRNA. [Source:RefSeq mRNA;Acc:NM_001035052]
Bos taurus tryptophanyl tRNA synthetase 2, mitochondrial (WARS2), mRNA. [Source:RefSeq mRNA;Acc:NM_001034582]
Bos taurus zinc finger, HIT-type containing 2 (ZNHIT2), mRNA. [Source:RefSeq mRNA;Acc:NM_001040560]

pre-B lymphocyte 3 [Source:HGNC Symbol;Acc:HGNC:12710]
Uncharacterized protein [Source:UniProtKB/TrEMBL;Acc:F1MNJ9]
pellino E3 ubiquitin protein ligase family member 2 [Source:HGNC Symbol;Acc:HGNC:8828]
Bos taurus ribosomal protein L37 (RPL37), mRNA. [Source:RefSeq mRNA;Acc:NM_001078132]
Neuronal pentraxin-like protein C16orf38-like; Uncharacterized protein [Source:UniProtKB/TrEMBL;Acc:E1B8G2]
WD repeat containing planar cell polarity effector [Source:HGNC Symbol;Acc:HGNC:28027]
thymidine kinase 2, mitochondrial [Source:HGNC Symbol;Acc:HGNC:11831]
Bos taurus PIH1 domain containing 2 (PIH1D2), mRNA. [Source:RefSeq mRNA;Acc:NM_001038189]
chondroitin sulfate N-acetylgalactosaminyltransferase 2 [Source:HGNC Symbol;Acc:HGNC:24292]
Uncharacterized protein [Source:UniProtKB/TrEMBL;Acc:F1MMB4]
glial cell derived neurotrophic factor [Source:HGNC Symbol;Acc:HGNC:4232]
Bos taurus major histocompatibility complex, class I, A (BOLA-A), mRNA. [Source:RefSeq mRNA;Acc:NM_001040554]
mevalonate kinase [Source:HGNC Symbol;Acc:HGNC:7530]
Bos taurus chromosome 11 open reading frame, human C9orf9 (C11H9orf9), mRNA. [Source:RefSeq mRNA;Acc:NM_001101193]
Bos taurus SCP2 sterol-binding domain containing 1 (SCP2D1), mRNA. [Source:RefSeq mRNA;Acc:NM_001040507]
chromosome 3 open reading frame 33 [Source:HGNC Symbol;Acc:HGNC:26434]
Uncharacterized protein [Source:UniProtKB/TrEMBL;Acc:E1BA06]

phosphodiesterase 3B, cGMP-inhibited [Source:HGNC Symbol;Acc:HGNC:8779]
family with sequence similarity 221, member B [Source:HGNC Symbol;Acc:HGNC:30762]
short stature homeobox 2 [Source:HGNC Symbol;Acc:HGNC:10854]
Uncharacterized protein [Source:UniProtKB/TrEMBL;Acc:E1BMX8]
RAS-like, family 11, member A [Source:HGNC Symbol;Acc:HGNC:23802]

Bos taurus kelch-like 2, Mayven (Drosophila) (KLHL2), mRNA. [Source:RefSeq mRNA;Acc:NM_001102160]
Bos taurus zinc finger, CCHC domain containing 9 (ZCCHC9), mRNA. [Source:RefSeq mRNA;Acc:NM_001101055]
Bos taurus endothelin receptor type B (EDNRB), mRNA. [Source:RefSeq mRNA;Acc:NM_174309]
Bos taurus thyrotropin-releasing hormone (TRH), mRNA. [Source:RefSeq mRNA;Acc:NM_001101221]
Uncharacterized protein [Source:UniProtKB/TrEMBL;Acc:E1BB19]
keratinocyte differentiation-associated protein [Source:HGNC Symbol;Acc:HGNC:16313]
Bos taurus myoglobin (MB), mRNA. [Source:RefSeq mRNA;Acc:NM_173881]
Bos taurus secretion regulating guanine nucleotide exchange factor (SERGEF), mRNA. [Source:RefSeq mRNA;Acc:NM_001076021]
Bos taurus glucosamine-phosphate N-acetyltransferase 1 (GNPNAT1), mRNA. [Source:RefSeq mRNA;Acc:NM_001075559]
Bos taurus crystallin, beta A1 (CRYBA1), mRNA. [Source:RefSeq mRNA;Acc:NM_174523]
Bos taurus glia maturation factor, gamma (GMFG), mRNA. [Source:RefSeq mRNA;Acc:NM_001024537]
Uncharacterized protein [Source:UniProtKB/TrEMBL;Acc:E1BLQ8]
Bos taurus XK, Kell blood group complex subunit-related family, member 6 (XKR6), mRNA. [Source:RefSeq mRNA;Acc:NM_001192331]
Autophagy protein 5 [Source:UniProtKB/Swiss-Prot;Acc:Q3MQ24]
CDC-like kinase 1 [Source:HGNC Symbol;Acc:HGNC:2068]
Uncharacterized protein [Source:UniProtKB/TrEMBL;Acc:G8JKW3]
Bos taurus peptidylprolyl isomerase (cyclophilin)-like 3 (PPIL3), mRNA. [Source:RefSeq mRNA;Acc:NM_001206564]
Bos taurus LIM and cysteine-rich domains 1 (LMCD1), mRNA. [Source:RefSeq mRNA;Acc:NM_001076222]
Bos taurus sperm mitochondria-associated cysteine-rich protein (SMCP), mRNA. [Source:RefSeq mRNA;Acc:NM_001008417]
Bos taurus solute carrier family 35 (UDP-glucuronic acid/UDP-N-acetylgalactosamine dual transporter), member D1 (SLC35D1), mRNA. [Source:RefSeq mRNA;Ac
Bos taurus polymerase (RNA) II (DNA directed) polypeptide J, 13.3kDa (POLR2J), mRNA. [Source:RefSeq mRNA;Acc:NM_001037483]
melanin-concentrating hormone receptor 2 [Source:HGNC Symbol;Acc:HGNC:20867]
Bos taurus cytochrome P450, family 3, subfamily A, polypeptide 5 (CYP3A5), mRNA. [Source:RefSeq mRNA;Acc:NM_001075888]
Bos taurus bridging integrator 3 (BIN3), mRNA. [Source:RefSeq mRNA;Acc:NM_001075299]

Bos taurus transmembrane protein 135 (TMEM135), mRNA. [Source:RefSeq mRNA;Acc:NM_001035348]
Bos taurus prolyl 4-hydroxylase, transmembrane (endoplasmic reticulum) (P4HTM), mRNA. [Source:RefSeq mRNA;Acc:NM_001076115]
Uncharacterized protein [Source:UniProtKB/TrEMBL;Acc:G3MYE2]
zona pellucida binding protein [Source:HGNC Symbol;Acc:HGNC:15662]
tumor protein p53 inducible nuclear protein 2 [Source:HGNC Symbol;Acc:HGNC:16104]
phospholipid scramblase family member 5 [Source:RefSeq peptide;Acc:NP_001179464]
Bos taurus HMG-box transcription factor 1 (HBP1), mRNA. [Source:RefSeq mRNA;Acc:NM_001046196]
LIM homeobox 6 [Source:HGNC Symbol;Acc:HGNC:21735]
gastric inhibitory polypeptide receptor [Source:HGNC Symbol;Acc:HGNC:4271]
Bos taurus chromobox homolog 3 (CBX3), mRNA. [Source:RefSeq mRNA;Acc:NM_001101198]
Bos taurus homeobox B7 (HOXB7), mRNA. [Source:RefSeq mRNA;Acc:NM_174342]

Bos taurus protein inhibitor of activated STAT, 1 (PIAS1), mRNA. [Source:RefSeq mRNA;Acc:NM_001075396]
Bos taurus SCAN domain containing 1 (SCAND1), mRNA. [Source:RefSeq mRNA;Acc:NM_001079591]
Bos taurus C-type lectin domain family 11, member A (CLEC11A), mRNA. [Source:RefSeq mRNA;Acc:NM_001098054]
Bos taurus ribonuclease H2, subunit C (RNASEH2C), mRNA. [Source:RefSeq mRNA;Acc:NM_001045921]

Bos taurus dom-3 homolog Z (C. elegans) (DOM3Z), mRNA. [Source:RefSeq mRNA;Acc:NM_001014872]
Insulin-like growth factor-binding protein 2 [Source:UniProtKB/TrEMBL;Acc:F1N2P8]
Olfactory receptor [Source:UniProtKB/TrEMBL;Acc:F1N2G3]
Bos taurus chemokine (C-X-C motif) ligand 11 (CXCL11), mRNA. [Source:RefSeq mRNA;Acc:NM_001113173]
homeobox C9 [Source:HGNC Symbol;Acc:HGNC:5130]
solute carrier family 41, member 3 [Source:HGNC Symbol;Acc:HGNC:31046]
Bos taurus lipopolysaccharide-induced TNF factor (LITAF), mRNA. [Source:RefSeq mRNA;Acc:NM_001046252]
Olfactory receptor [Source:UniProtKB/TrEMBL;Acc:E1BI40]
Bos taurus LSM2 homolog, U6 small nuclear RNA associated (S. cerevisiae) (LSM2), mRNA. [Source:RefSeq mRNA;Acc:NM_001101177]
Bos taurus POU class 4 homeobox 2 (POU4F2), mRNA. [Source:RefSeq mRNA;Acc:NM_001192952]
Bos taurus asparagine-linked glycosylation 3, alpha-1,3- mannosyltransferase homolog (S. cerevisiae) (ALG3), mRNA. [Source:RefSeq mRNA;Acc:NM_00108351
Bos taurus thymosin beta 10 (TMSB10), mRNA. [Source:RefSeq mRNA;Acc:NM_174623]
Bos taurus calcium/calmodulin-dependent protein kinase II inhibitor 2 (CAMK2N2), mRNA. [Source:RefSeq mRNA;Acc:NM_001114514]

Bos taurus solute carrier family 39 (zinc transporter), member 8 (SLC39A8), mRNA. [Source:RefSeq mRNA;Acc:NM_001205630]
Bos taurus mitochondrial ribosomal protein S2 (MRPS2), mRNA. [Source:RefSeq mRNA;Acc:NM_001098877]
Bos taurus nudix (nucleoside diphosphate linked moiety X)-type motif 6 (NUDT6), mRNA. [Source:RefSeq mRNA;Acc:NM_001105650]
neuron-specific protein family member 1 [Source:RefSeq peptide;Acc:NP_001071425]
Bos taurus chromosome 26 open reading frame, human C10orf125 (FUOM), mRNA. [Source:RefSeq mRNA;Acc:NM_001076278]
gap junction protein, beta 5, 31.1kDa [Source:HGNC Symbol;Acc:HGNC:4287]

indoleamine 2,3-dioxygenase 2 [Source:HGNC Symbol;Acc:HGNC:27269]
cysteine/tyrosine-rich 1 [Source:HGNC Symbol;Acc:HGNC:16274]
Bos taurus survival of motor neuron 1, telomeric (SMN1), mRNA. [Source:RefSeq mRNA;Acc:NM_175701]
zinc finger, DHHC-type containing 24 [Source:HGNC Symbol;Acc:HGNC:27387]
Bos taurus phospholipase A2, group IIA (platelets, synovial fluid) (PLA2G2A), mRNA. [Source:RefSeq mRNA;Acc:NM_001025324]
Bos taurus intraflagellar transport 57 homolog (Chlamydomonas) (IFT57), mRNA. [Source:RefSeq mRNA;Acc:NM_001105403]
uroporphyrinogen III synthase [Source:HGNC Symbol;Acc:HGNC:12592]
Bos taurus tripartite motif containing 50 (TRIM50), mRNA. [Source:RefSeq mRNA;Acc:NM_001206256]
protein prenyltransferase alpha subunit repeat containing 1 [Source:HGNC Symbol;Acc:HGNC:30449]
Bos taurus FIG4 homolog, SAC1 lipid phosphatase domain containing (S. cerevisiae) (FIG4), mRNA. [Source:RefSeq mRNA;Acc:NM_001076014]
ethanolamine kinase 1 [Source:HGNC Symbol;Acc:HGNC:24649]
Bos taurus REX2, RNA exonuclease 2 homolog (S. cerevisiae) (REXO2), mRNA. [Source:RefSeq mRNA;Acc:NM_001081735]
family with sequence similarity 219, member B [Source:HGNC Symbol;Acc:HGNC:24695]
ankyrin repeat and SOCS box containing 17 [Source:HGNC Symbol;Acc:HGNC:19769]
Uncharacterized protein [Source:UniProtKB/TrEMBL;Acc:E1BMR5]
Olfactory receptor [Source:UniProtKB/TrEMBL;Acc:F1N1L2]

Bos taurus NADH dehydrogenase (ubiquinone) 1 beta subcomplex, 6, 17kDa (NDUFB6), mRNA. [Source:RefSeq mRNA;Acc:NM_175787]
Bos taurus par-6 partitioning defective 6 homolog gamma (C. elegans) (PARD6G), mRNA. [Source:RefSeq mRNA;Acc:NM_001098100]
T cell receptor alpha variable 8-1 [Source:HGNC Symbol;Acc:HGNC:12146]
Bos taurus chromosome 10 open reading frame, human C15orf43 (C10H15orf43), mRNA. [Source:RefSeq mRNA;Acc:NM_001077078]
Bos taurus radial spoke 3 homolog (Chlamydomonas) (RSPH3), mRNA. [Source:RefSeq mRNA;Acc:NM_001109799]
G protein-coupled receptor 123 [Source:HGNC Symbol;Acc:HGNC:13838]

Bos taurus chromosome 23 open reading frame, human C6orf130 (OARD1), mRNA. [Source:RefSeq mRNA;Acc:NM_001077113]
Bos taurus cerebellin 2 precursor (CBLN2), mRNA. [Source:RefSeq mRNA;Acc:NM_001098919]
SFT2 domain containing 3 [Source:HGNC Symbol;Acc:HGNC:28767]
Bos taurus leucine aminopeptidase 3 (LAP3), mRNA. [Source:RefSeq mRNA;Acc:NM_174098]
Bos taurus CCR4 carbon catabolite repression 4-like (S. cerevisiae) (CCRN4L), mRNA. [Source:RefSeq mRNA;Acc:NM_001082454]
Bos taurus ATPase, H+ transporting V0 subunit e2 (ATP6V0E2), mRNA. [Source:RefSeq mRNA;Acc:NM_001097574]
Bos taurus immunoglobulin superfamily containing leucine-rich repeat (ISLR), mRNA. [Source:RefSeq mRNA;Acc:NM_001080729]
Bos taurus AHA1, activator of heat shock 90kDa protein ATPase homolog 2 (yeast) (AHSA2), mRNA. [Source:RefSeq mRNA;Acc:NM_001102134]
Bos taurus NADH dehydrogenase (ubiquinone) 1 alpha subcomplex, 1, 7.5kDa (NDUFA1), mRNA. [Source:RefSeq mRNA;Acc:NM_175794]
chromosome 10 open reading frame 128 [Source:HGNC Symbol;Acc:HGNC:27274]

Dephospho-CoA kinase domain-containing protein [Source:UniProtKB/Swiss-Prot;Acc:Q3ZBS0]
Bos taurus hexamethylene bis-acetamide inducible 1 (HEXIM1), mRNA. [Source:RefSeq mRNA;Acc:NM_001076181]
Bos taurus transmembrane protein 230 (TMEM230), mRNA. [Source:RefSeq mRNA;Acc:NM_001014930]
Bos taurus calcyphosine (CAPS), mRNA. [Source:RefSeq mRNA;Acc:NM_001076424]
retbindin [Source:HGNC Symbol;Acc:HGNC:30310]
protein phosphatase, Mg2+/Mn2+ dependent, 1J [Source:HGNC Symbol;Acc:HGNC:20785]
Bos taurus Kruppel-like factor 1 (erythroid) (KLF1), mRNA. [Source:RefSeq mRNA;Acc:NM_001080359]
Bos taurus family with sequence similarity 104, member A (FAM104A), mRNA. [Source:RefSeq mRNA;Acc:NM_001035460]
Uncharacterized protein [Source:UniProtKB/TrEMBL;Acc:F1N0R1]
Bos taurus NHP2 non-histone chromosome protein 2-like 1 (S. cerevisiae) (NHP2L1), mRNA. [Source:RefSeq mRNA;Acc:NM_001077004]
Bos taurus multiple C2 domains, transmembrane 1 (MCTP1), mRNA. [Source:RefSeq mRNA;Acc:NM_001104987]
Tctex1 domain containing 4 [Source:HGNC Symbol;Acc:HGNC:32315]

Bos taurus mitochondrial ribosomal protein S9 (MRPS9), mRNA. [Source:RefSeq mRNA;Acc:NM_001035332]
bone marrow stromal cell antigen 1 [Source:HGNC Symbol;Acc:HGNC:1118]
Bos taurus heparan sulfate 6-O-sulfotransferase 1 (HS6ST1), mRNA. [Source:RefSeq mRNA;Acc:NM_001192491]
potassium inwardly-rectifying channel, subfamily J, member 3 [Source:HGNC Symbol;Acc:HGNC:6264]
DnaJ (Hsp40) homolog, subfamily B, member 8 [Source:HGNC Symbol;Acc:HGNC:23699]
Bos taurus microfibrillar-associated protein 4 (MFAP4), mRNA. [Source:RefSeq mRNA;Acc:NM_001080217]

FOS-like antigen 1 [Source:HGNC Symbol;Acc:HGNC:13718]
Bos taurus forkhead box I2 (FOXI2), mRNA. [Source:RefSeq mRNA;Acc:NM_001192949]
Bos taurus WD repeat domain 86 (WDR86), mRNA. [Source:RefSeq mRNA;Acc:NM_001191339]
Bos taurus guanine nucleotide binding protein (G protein), gamma 12-like (LOC529425), mRNA. [Source:RefSeq mRNA;Acc:NM_001195624]
Bos taurus LIM and senescent cell antigen-like domains 2 (LIMS2), mRNA. [Source:RefSeq mRNA;Acc:NM_001130751]
Bos taurus uromodulin (UMOD), mRNA. [Source:RefSeq mRNA;Acc:NM_174213]
Uncharacterized protein [Source:UniProtKB/TrEMBL;Acc:F1N717]
Bos taurus inhibitor of kappa light polypeptide gene enhancer in B-cells, kinase gamma (IKBKG), mRNA. [Source:RefSeq mRNA;Acc:NM_174354]
Bos taurus placenta-specific 1 (PLAC1), mRNA. [Source:RefSeq mRNA;Acc:NM_001077057]

Bos taurus adenylate kinase 1 (AK1), mRNA. [Source:RefSeq mRNA;Acc:NM_001013582]
Bos taurus hydroxysteroid (17-beta) dehydrogenase 11 (HSD17B11), mRNA. [Source:RefSeq mRNA;Acc:NM_001046286]
v-myc avian myelocytomatosis viral oncogene lung carcinoma derived homolog [Source:HGNC Symbol;Acc:HGNC:7555]
Non-histone chromosomal protein HMG-14 [Source:UniProtKB/TrEMBL;Acc:E1BC56]
solute carrier family 35, member F4 [Source:HGNC Symbol;Acc:HGNC:19845]
Bos taurus ATPase inhibitory factor 1 (ATPIF1), mRNA. [Source:RefSeq mRNA;Acc:NM_175816]
Bos taurus interleukin 1 receptor, type II (IL1R2), mRNA. [Source:RefSeq mRNA;Acc:NM_001046210]
Bos taurus thioredoxin-like 4B (TXNL4B), mRNA. [Source:RefSeq mRNA;Acc:NM_001014897]

Bos taurus required for meiotic nuclear division 5 homolog B (S. cerevisiae) (RMND5B), mRNA. [Source:RefSeq mRNA;Acc:NM_001075706]
Bos taurus F-box protein 45 (FBXO45), mRNA. [Source:RefSeq mRNA;Acc:NM_001206758]
Bos taurus NADH dehydrogenase (ubiquinone) 1, alpha/beta subcomplex, 1, 8kDa (NDUFAB1), mRNA. [Source:RefSeq mRNA;Acc:NM_001040488]

translocase of outer mitochondrial membrane 7 homolog (yeast) [Source:HGNC Symbol;Acc:HGNC:21648]
Bos taurus chromosome 16 open reading frame, human C1orf115 (C16H1orf115), mRNA. [Source:RefSeq mRNA;Acc:NM_001205383]
leucine rich repeat containing 34 [Source:HGNC Symbol;Acc:HGNC:28408]
Bos taurus nuclear transport factor 2 (NUTF2), mRNA. [Source:RefSeq mRNA;Acc:NM_001037624]
Olfactory receptor [Source:UniProtKB/TrEMBL;Acc:F1MGZ3]
Bos taurus troponin I type 3 (cardiac) (TNNI3), mRNA. [Source:RefSeq mRNA;Acc:NM_001040517]
zinc finger protein 354A [Source:HGNC Symbol;Acc:HGNC:11628]

Neurogenin 3; Uncharacterized protein [Source:UniProtKB/TrEMBL;Acc:F1MGY3]
actin-like 8 [Source:HGNC Symbol;Acc:HGNC:24018]
Bos taurus ATP synthase, H+ transporting, mitochondrial Fo complex, subunit B1 (ATP5F1), mRNA. [Source:RefSeq mRNA;Acc:NM_001038501]
PR domain containing 11 [Source:HGNC Symbol;Acc:HGNC:13996]
Bos taurus lipase, gastric (LIPF), mRNA. [Source:RefSeq mRNA;Acc:NM_174103]
alpha hemoglobin stabilizing protein [Source:HGNC Symbol;Acc:HGNC:18075]
Bos taurus epithelial cell adhesion molecule (EPCAM), mRNA. [Source:RefSeq mRNA;Acc:NM_001035290]
Bos taurus ribosomal protein S9 (RPS9), mRNA. [Source:RefSeq mRNA;Acc:NM_001101152]
Uncharacterized protein [Source:UniProtKB/TrEMBL;Acc:E1BI55]

T cell receptor alpha variable 3 (gene/pseudogene) [Source:HGNC Symbol;Acc:HGNC:12128]
Bos taurus superoxide dismutase 2, mitochondrial (SOD2), mRNA. [Source:RefSeq mRNA;Acc:NM_201527]
Bos taurus prenyl (decaprenyl) diphosphate synthase, subunit 1 (PDSS1), mRNA. [Source:RefSeq mRNA;Acc:NM_001100331]

signal-regulatory protein beta 2 [Source:HGNC Symbol;Acc:HGNC:16247]
Ras homolog enriched in brain like 1 [Source:HGNC Symbol;Acc:HGNC:21166]
spermatogenesis and oogenesis specific basic helix-loop-helix 1 [Source:HGNC Symbol;Acc:HGNC:27845]
Uncharacterized protein [Source:UniProtKB/TrEMBL;Acc:F1MI10]
Bos taurus SEC22 vesicle trafficking protein homolog C (S. cerevisiae) (SEC22C), mRNA. [Source:RefSeq mRNA;Acc:NM_001046546]
Bos taurus caspase recruitment domain family, member 9 (CARD9), mRNA. [Source:RefSeq mRNA;Acc:NM_001077111]
ADP-ribosylation factor-like 8A [Source:HGNC Symbol;Acc:HGNC:25192]
Bos taurus sperm flagellar 1 (SPEF1), mRNA. [Source:RefSeq mRNA;Acc:NM_001034422]
Actin-related protein 2/3 complex subunit 2 [Source:UniProtKB/TrEMBL;Acc:F1MEL0]
family with sequence similarity 101, member B [Source:HGNC Symbol;Acc:HGNC:28705]
Olfactory receptor [Source:UniProtKB/TrEMBL;Acc:G5E5K6]
signal peptidase complex subunit 2 homolog (S. cerevisiae) [Source:HGNC Symbol;Acc:HGNC:28962]
Bos taurus protein arginine methyltransferase 1 (PRMT1), mRNA. [Source:RefSeq mRNA;Acc:NM_001015624]
regulator of chromosome condensation (RCC1) and BTB (POZ) domain containing protein 2 [Source:HGNC Symbol;Acc:HGNC:1914]
Uncharacterized protein [Source:UniProtKB/TrEMBL;Acc:F1MCW1]
Bos taurus insulin-like 6 (INSL6), mRNA. [Source:RefSeq mRNA;Acc:NM_001077521]

TRAF family member-associated NFKB activator [Source:HGNC Symbol;Acc:HGNC:11562]
Bos taurus prolactin-induced protein (PIP), mRNA. [Source:RefSeq mRNA;Acc:NM_001080913]
RAN binding protein 1 [Source:HGNC Symbol;Acc:HGNC:9847]
Bos taurus F-box protein 8 (FBXO8), mRNA. [Source:RefSeq mRNA;Acc:NM_001015667]
Bos taurus TWIST neighbor (TWISTNB), mRNA. [Source:RefSeq mRNA;Acc:NM_001105622]
Bos taurus protein phosphatase 1, regulatory (inhibitor) subunit 2 (PPP1R2), mRNA. [Source:RefSeq mRNA;Acc:NM_001035392]

paired box 1 [Source:HGNC Symbol;Acc:HGNC:8615]
Bos taurus D site of albumin promoter (albumin D-box) binding protein (DBP), mRNA. [Source:RefSeq mRNA;Acc:NM_001037445]
orthodenticle homeobox 1 [Source:HGNC Symbol;Acc:HGNC:8521]
Bos taurus chorionic somatomammotropin hormone 2 (CSH2), transcript variant 1, mRNA. [Source:RefSeq mRNA;Acc:NM_181007]
Bos taurus mitochondrial ribosomal protein L13 (MRPL13), mRNA. [Source:RefSeq mRNA;Acc:NM_001034805]
Bos taurus TLC domain containing 1 (TLCD1), mRNA. [Source:RefSeq mRNA;Acc:NM_001105042]
Bos taurus keratin 35 (KRT35), mRNA. [Source:RefSeq mRNA;Acc:NM_001076073]

Josephin domain containing 2 [Source:HGNC Symbol;Acc:HGNC:28853]
leucine rich repeat containing 4B [Source:HGNC Symbol;Acc:HGNC:25042]
Bos taurus UTP15, U3 small nucleolar ribonucleoprotein, homolog (S. cerevisiae) (UTP15), mRNA. [Source:RefSeq mRNA;Acc:NM_001101128]
Bos taurus acylphosphatase 2, muscle type (ACYP2), mRNA. [Source:RefSeq mRNA;Acc:NM_001113230]
Bos taurus interferon, kappa (IFNK), mRNA. [Source:RefSeq mRNA;Acc:NM_001206423]
chromosome 2 open reading frame 73 [Source:HGNC Symbol;Acc:HGNC:26861]
Bos taurus neurexophilin 1 (NXPH1), mRNA. [Source:RefSeq mRNA;Acc:NM_001034762]
Bos taurus zinc finger protein 432 (ZNF432), mRNA. [Source:RefSeq mRNA;Acc:NM_001192232]
60S ribosomal protein L12 [Source:UniProtKB/Swiss-Prot;Acc:P61284]
NLR family, pyrin domain containing 8 [Source:HGNC Symbol;Acc:HGNC:22940]
thyroid hormone receptor interactor 13 [Source:HGNC Symbol;Acc:HGNC:12307]
PHD finger protein 21B [Source:HGNC Symbol;Acc:HGNC:25161]
Bos taurus hydroxysteroid (17-beta) dehydrogenase 4 (HSD17B4), mRNA. [Source:RefSeq mRNA;Acc:NM_001007809]
Bos taurus CD55 molecule, decay accelerating factor for complement (Cromer blood group) (CD55), mRNA. [Source:RefSeq mRNA;Acc:NM_001030303]
Bos taurus alcohol dehydrogenase 6 (class V) (ADH6), mRNA. [Source:RefSeq mRNA;Acc:NM_001075843]

thymidylate synthetase [Source:HGNC Symbol;Acc:HGNC:12441]
ribosomal protein L9 [Source:HGNC Symbol;Acc:HGNC:10369]

calcium homeostasis modulator 1 [Source:HGNC Symbol;Acc:HGNC:23494]
claudin 23 [Source:HGNC Symbol;Acc:HGNC:17591]
glycoprotein hormone alpha 2 [Source:HGNC Symbol;Acc:HGNC:18054]
Bos taurus abhydrolase domain containing 1 (ABHD1), mRNA. [Source:RefSeq mRNA;Acc:NM_001035094]
spermatogenesis associated 32 [Source:HGNC Symbol;Acc:HGNC:26349]
Bos taurus trafficking protein particle complex 10 (TRAPPC10), mRNA. [Source:RefSeq mRNA;Acc:NM_001205885]
Bos taurus POC5 centriolar protein homolog (Chlamydomonas) (POC5), mRNA. [Source:RefSeq mRNA;Acc:NM_001102158]
Bos taurus regenerating islet-derived 3 gamma (REG3G), mRNA. [Source:RefSeq mRNA;Acc:NM_001024534]
Uncharacterized protein [Source:UniProtKB/TrEMBL;Acc:M0QVZ5]
carboxypeptidase, vitellogenic-like [Source:HGNC Symbol;Acc:HGNC:14399]
Bos taurus arginine vasopressin receptor 1A (AVPR1A), mRNA. [Source:RefSeq mRNA;Acc:NM_001104990]
Bos taurus epithelial membrane protein 2 (EMP2), mRNA. [Source:RefSeq mRNA;Acc:NM_001075324]
Bos taurus ring finger and CHY zinc finger domain containing 1 (RCHY1), mRNA. [Source:RefSeq mRNA;Acc:NM_001083754]
Bos taurus chemokine (C-C motif) ligand 5 (CCL5), mRNA. [Source:RefSeq mRNA;Acc:NM_175827]
guanylate cyclase activator 2A (guanylin) [Source:HGNC Symbol;Acc:HGNC:4682]
Bos taurus cytochrome c oxidase subunit VIb polypeptide 2 (testis) (COX6B2), mRNA. [Source:RefSeq mRNA;Acc:NM_001012684]
Bos taurus spermatogenesis associated 9 (SPATA9), mRNA. [Source:RefSeq mRNA;Acc:NM_001034736]
WD repeat domain 25 [Source:HGNC Symbol;Acc:HGNC:21064]
Uncharacterized protein [Source:UniProtKB/TrEMBL;Acc:G3X6N1]
Bos taurus tumor necrosis factor, alpha-induced protein 6 (TNFAIP6), mRNA. [Source:RefSeq mRNA;Acc:NM_001007813]
Olfactory receptor [Source:UniProtKB/TrEMBL;Acc:F1MWB2]
Bos taurus triggering receptor expressed on myeloid cells 2 (TREM2), mRNA. [Source:RefSeq mRNA;Acc:NM_001079580]
Uncharacterized protein [Source:UniProtKB/TrEMBL;Acc:F1MVX4]

Uncharacterized protein [Source:UniProtKB/TrEMBL;Acc:E1BG39]
Uncharacterized protein [Source:UniProtKB/TrEMBL;Acc:E1BDS9]
olfactory receptor, family 10, subfamily T, member 2 [Source:HGNC Symbol;Acc:HGNC:14816]

Bos taurus CD209 molecule (CD209), mRNA. [Source:RefSeq mRNA;Acc:NM_001145756]
splicing regulatory glutamine/lysine-rich protein 1 [Source:HGNC Symbol;Acc:HGNC:17882]
leucine rich repeat containing 4C [Source:HGNC Symbol;Acc:HGNC:29317]
TPSB1 protein; Tryptase alpha/beta 1; Uncharacterized protein [Source:UniProtKB/TrEMBL;Acc:A6QQ05]
SET domain, bifurcated 2 [Source:HGNC Symbol;Acc:HGNC:20263]
Bos taurus ATP synthase, H+ transporting, mitochondrial Fo complex, subunit G (ATP5L), mRNA. [Source:RefSeq mRNA;Acc:NM_001024550]
Bos taurus hematopoietic cell signal transducer (HCST), mRNA. [Source:RefSeq mRNA;Acc:NM_001076977]
Bos taurus TYRO protein tyrosine kinase binding protein (TYROBP), mRNA. [Source:RefSeq mRNA;Acc:NM_174627]
Uncharacterized protein [Source:UniProtKB/TrEMBL;Acc:G3X6N6]
Bos taurus COMM domain containing 4 (COMMD4), mRNA. [Source:RefSeq mRNA;Acc:NM_001040597]
ST8 alpha-N-acetyl-neuraminide alpha-2,8-sialyltransferase 3 [Source:HGNC Symbol;Acc:HGNC:14269]
Uncharacterized protein [Source:UniProtKB/TrEMBL;Acc:G3MYJ3]

Bos taurus EH-domain containing 1 (EHD1), mRNA. [Source:RefSeq mRNA;Acc:NM_001015578]
Bos taurus LSM3 homolog, U6 small nuclear RNA associated (S. cerevisiae) (LSM3), mRNA. [Source:RefSeq mRNA;Acc:NM_001037487]
Bos taurus secretory carrier membrane protein 1 (SCAMP1), mRNA. [Source:RefSeq mRNA;Acc:NM_001076054]
CAP-GLY domain containing linker protein family, member 4 [Source:HGNC Symbol;Acc:HGNC:26108]
Bos taurus chromosome 10 open reading frame, human C15orf24 (EMC7), mRNA. [Source:RefSeq mRNA;Acc:NM_001099143]
Bos taurus enhancer of yellow 2 homolog (Drosophila) (ENY2), mRNA. [Source:RefSeq mRNA;Acc:NM_001034747]
Bos taurus interferon-induced protein 35 (IFI35), mRNA. [Source:RefSeq mRNA;Acc:NM_001075462]
Bos taurus melan-A (MLANA), mRNA. [Source:RefSeq mRNA;Acc:NM_001193135]
Bos taurus chromosome 20 open reading frame, human C5orf34 (C20H5orf34), mRNA. [Source:RefSeq mRNA;Acc:NM_001083426]
XK, Kell blood group complex subunit-related family, member 5 [Source:HGNC Symbol;Acc:HGNC:20782]

ZFP90 zinc finger protein [Source:HGNC Symbol;Acc:HGNC:23329]

tripartite motif containing 69 [Source:HGNC Symbol;Acc:HGNC:17857]
tripartite motif containing 15 [Source:HGNC Symbol;Acc:HGNC:16284]
Bos taurus casein kinase 1, epsilon (CSNK1E), mRNA. [Source:RefSeq mRNA;Acc:NM_001078109]

Bos taurus calcium channel, voltage-dependent, gamma subunit 1 (CACNG1), mRNA. [Source:RefSeq mRNA;Acc:NM_001080337]
matrix extracellular phosphoglycoprotein [Source:HGNC Symbol;Acc:HGNC:13361]
Bos taurus ATPase, H+ transporting, lysosomal 14kDa, V1 subunit F (ATP6V1F), mRNA. [Source:RefSeq mRNA;Acc:NM_001045869]
Bos taurus synaptojanin 2 binding protein (SYNJ2BP), mRNA. [Source:RefSeq mRNA;Acc:NM_001035355]
BRO1 domain and CAAX motif containing [Source:HGNC Symbol;Acc:HGNC:26512]
chromosome 7 open reading frame 60 [Source:HGNC Symbol;Acc:HGNC:26475]
nuclear receptor interacting protein 2 [Source:HGNC Symbol;Acc:HGNC:23078]
Bos taurus transmembrane emp24 protein transport domain containing 6 (TMED6), mRNA. [Source:RefSeq mRNA;Acc:NM_001075988]
Bos taurus cathepsin D (CTSD), mRNA. [Source:RefSeq mRNA;Acc:NM_001166521]
Bos taurus THAP domain containing, apoptosis associated protein 1 (THAP1), mRNA. [Source:RefSeq mRNA;Acc:NM_001034648]
phosphatidylinositol glycan anchor biosynthesis, class A [Source:HGNC Symbol;Acc:HGNC:8957]
carbohydrate (N-acetylglucosamine 6-O) sulfotransferase 7 [Source:HGNC Symbol;Acc:HGNC:13817]
Bos taurus zinc finger protein 414 (ZNF414), mRNA. [Source:RefSeq mRNA;Acc:NM_001038175]
mohawk homeobox [Source:HGNC Symbol;Acc:HGNC:23729]

solute carrier family 45, member 3 [Source:HGNC Symbol;Acc:HGNC:8642]
kinesin family member 25 [Source:HGNC Symbol;Acc:HGNC:6390]
Bos taurus casein alpha s1 (CSN1S1), mRNA. [Source:RefSeq mRNA;Acc:NM_181029]
ribonuclease P/MRP 38kDa subunit [Source:HGNC Symbol;Acc:HGNC:30329]
Bos taurus dolichyl-phosphate mannosyltransferase polypeptide 1, catalytic subunit (DPM1), mRNA. [Source:RefSeq mRNA;Acc:NM_001076013]
Bos taurus pescadillo homolog 1, containing BRCT domain (zebrafish) (PES1), mRNA. [Source:RefSeq mRNA;Acc:NM_001192300]

Bos taurus ubiquitin A-52 residue ribosomal protein fusion product 1 (UBA52), mRNA. [Source:RefSeq mRNA;Acc:NM_001076363]
chromosome 19 open reading frame 60 [Source:HGNC Symbol;Acc:HGNC:26098]
Bos taurus midkine (neurite growth-promoting factor 2) (MDK), mRNA. [Source:RefSeq mRNA;Acc:NM_173935]
Bos taurus zinc finger and BTB domain containing 5 (ZBTB5), mRNA. [Source:RefSeq mRNA;Acc:NM_001077937]
Bos taurus polycystic kidney disease 2-like 2 (PKD2L2), mRNA. [Source:RefSeq mRNA;Acc:NM_001038552]
3-hydroxyisobutyryl-CoA hydrolase [Source:HGNC Symbol;Acc:HGNC:4908]

Bos taurus major facilitator superfamily domain containing 3 (MFSD3), mRNA. [Source:RefSeq mRNA;Acc:NM_001101905]
OTU deubiquitinase 6A [Source:HGNC Symbol;Acc:HGNC:32312]
Bos taurus embryonic ectoderm development (EED), mRNA. [Source:RefSeq mRNA;Acc:NM_001040494]
Uncharacterized protein [Source:UniProtKB/TrEMBL;Acc:G3MZ10]
interleukin 24 [Source:HGNC Symbol;Acc:HGNC:11346]
Bos taurus heparan sulfate (glucosamine) 3-O-sulfotransferase 3B1 (HS3ST3B1), mRNA. [Source:RefSeq mRNA;Acc:NM_001193139]
Rho GTPase activating protein 22 [Source:HGNC Symbol;Acc:HGNC:30320]

Bos taurus proopiomelanocortin (POMC), mRNA. [Source:RefSeq mRNA;Acc:NM_174151]
cytochrome b reductase 1 [Source:HGNC Symbol;Acc:HGNC:20797]

forkhead box J1 [Source:HGNC Symbol;Acc:HGNC:3816]
pantothenate kinase 2 [Source:HGNC Symbol;Acc:HGNC:15894]
HCK proto-oncogene, Src family tyrosine kinase [Source:HGNC Symbol;Acc:HGNC:4840]
Bos taurus mitochondrial ribosomal protein S17 (MRPS17), mRNA. [Source:RefSeq mRNA;Acc:NM_001024500]
Bos taurus charged multivesicular body protein 2B (CHMP2B), mRNA. [Source:RefSeq mRNA;Acc:NM_001035472]
Uncharacterized protein [Source:UniProtKB/TrEMBL;Acc:E1BNA0]
zinc finger protein 775 [Source:HGNC Symbol;Acc:HGNC:28501]
Bos taurus 5-hydroxytryptamine (serotonin) receptor 6 (HTR6), mRNA. [Source:RefSeq mRNA;Acc:NM_001205717]
Uncharacterized protein [Source:UniProtKB/TrEMBL;Acc:F1N742]
transmembrane and coiled-coil domains 4 [Source:HGNC Symbol;Acc:HGNC:27393]
Bos taurus phospholipase D family, member 6 (PLD6), mRNA. [Source:RefSeq mRNA;Acc:NM_001271990]
Bos taurus leucine rich repeat containing 2 (LRRC2), mRNA. [Source:RefSeq mRNA;Acc:NM_001080311]
Bos taurus oxytocin, prepropeptide (OXT), mRNA. [Source:RefSeq mRNA;Acc:NM_176855]
Bos taurus arginine vasopressin (AVP), mRNA. [Source:RefSeq mRNA;Acc:NM_176854]
zinc finger protein 382 [Source:HGNC Symbol;Acc:HGNC:17409]
Bos taurus cysteine-rich secretory protein 1 (CRISP1), mRNA. [Source:RefSeq mRNA;Acc:NM_001206259]
Bos taurus endothelin 1 (EDN1), mRNA. [Source:RefSeq mRNA;Acc:NM_181010]
Bos taurus retinol binding protein 5, cellular (RBP5), mRNA. [Source:RefSeq mRNA;Acc:NM_001101192]
Bos taurus SRA stem-loop interacting RNA binding protein (SLIRP), mRNA. [Source:RefSeq mRNA;Acc:NM_001037485]

Bos taurus serine/threonine kinase 17a (STK17A), mRNA. [Source:RefSeq mRNA;Acc:NM_001083422]
Bos taurus peptidylprolyl isomerase (cyclophilin)-like 6 (PPIL6), mRNA. [Source:RefSeq mRNA;Acc:NM_001046537]
Bos taurus LIM domain containing 2 (LIMD2), mRNA. [Source:RefSeq mRNA;Acc:NM_001040512]
Bos taurus Sad1 and UNC84 domain containing 3 (SUN3), mRNA. [Source:RefSeq mRNA;Acc:NM_001076084]
coiled-coil domain containing 68 [Source:HGNC Symbol;Acc:HGNC:24350]
chromosome 18 open reading frame 54 [Source:HGNC Symbol;Acc:HGNC:13796]
chromosome 16 open reading frame 52 [Source:HGNC Symbol;Acc:HGNC:27087]
Bos taurus phosphorylase kinase, gamma 1 (muscle) (PHKG1), mRNA. [Source:RefSeq mRNA;Acc:NM_001046486]
erythropoietin receptor [Source:HGNC Symbol;Acc:HGNC:3416]
chromosome 9 open reading frame 41 [Source:HGNC Symbol;Acc:HGNC:23435]
Bos taurus RNA binding motif protein 7 (RBM7), mRNA. [Source:RefSeq mRNA;Acc:NM_001034487]
Bos taurus family with sequence similarity 86, member A (FAM86A), mRNA. [Source:RefSeq mRNA;Acc:NM_001163938]
Bos taurus S100 calcium binding protein A7 (S100A7), mRNA. [Source:RefSeq mRNA;Acc:NM_174596]
Uncharacterized protein [Source:UniProtKB/TrEMBL;Acc:F1MHE8]
Bos taurus transmembrane protein 187 (TMEM187), mRNA. [Source:RefSeq mRNA;Acc:NM_001075355]
Bos taurus protein C receptor, endothelial (PROCR), mRNA. [Source:RefSeq mRNA;Acc:NM_174437]
Olfactory receptor [Source:UniProtKB/TrEMBL;Acc:F1MXM2]
Bos taurus aryl hydrocarbon receptor interacting protein-like 1 (AIPL1), mRNA. [Source:RefSeq mRNA;Acc:NM_174234]

Uncharacterized protein [Source:UniProtKB/TrEMBL;Acc:E1BFU3]
Bos taurus cytohesin 1 interacting protein (CYTIP), mRNA. [Source:RefSeq mRNA;Acc:NM_001102243]
Bos taurus transmembrane protein 54 (TMEM54), mRNA. [Source:RefSeq mRNA;Acc:NM_001034364]
Bos taurus ATX1 antioxidant protein 1 homolog (yeast) (ATOX1), mRNA. [Source:RefSeq mRNA;Acc:NM_001130758]
Bos taurus mitochondrial ribosomal protein L48 (MRPL48), mRNA. [Source:RefSeq mRNA;Acc:NM_001046563]
Bos taurus paraoxonase 2 (PON2), mRNA. [Source:RefSeq mRNA;Acc:NM_001013588]
Uncharacterized protein [Source:UniProtKB/TrEMBL;Acc:F1N107]
Bos taurus RNA binding motif protein 8A (RBM8A), mRNA. [Source:RefSeq mRNA;Acc:NM_001035324]
Bos taurus solute carrier family 25, member 47 (SLC25A47), mRNA. [Source:RefSeq mRNA;Acc:NM_001205829]
Bos taurus HtrA serine peptidase 1 (HTRA1), mRNA. [Source:RefSeq mRNA;Acc:NM_001244233]
Bos taurus zinc finger protein 555 (ZNF555), mRNA. [Source:RefSeq mRNA;Acc:NM_001082449]

Bos taurus three prime repair exonuclease 1 (TREX1), mRNA. [Source:RefSeq mRNA;Acc:NM_174475]

Bos taurus glycine C-acetyltransferase (GCAT), mRNA. [Source:RefSeq mRNA;Acc:NM_001075134]
ADP-ribosylation factor interacting protein 1 [Source:HGNC Symbol;Acc:HGNC:21496]
Olfactory receptor [Source:UniProtKB/TrEMBL;Acc:F1MYP5]
Bos taurus vacuolar protein sorting 37 homolog D (S. cerevisiae) (VPS37D), mRNA. [Source:RefSeq mRNA;Acc:NM_001205718]
Uncharacterized protein [Source:UniProtKB/TrEMBL;Acc:E1BN88]
potassium channel, subfamily V, member 1 [Source:HGNC Symbol;Acc:HGNC:18861]
Uncharacterized protein [Source:UniProtKB/TrEMBL;Acc:F1MKI0]
Uncharacterized protein [Source:UniProtKB/TrEMBL;Acc:F1MEB6]
solute carrier family 36 (proton/amino acid symporter), member 4 [Source:HGNC Symbol;Acc:HGNC:19660]
family with sequence similarity 219, member A [Source:HGNC Symbol;Acc:HGNC:19920]
Bos taurus energy homeostasis associated (ENHO), mRNA. [Source:RefSeq mRNA;Acc:NM_001163653]
microsomal glutathione S-transferase 1 [Source:HGNC Symbol;Acc:HGNC:7061]
purinergic receptor P2Y, G-protein coupled, 10 [Source:HGNC Symbol;Acc:HGNC:19906]
Bos taurus LSM4 homolog, U6 small nuclear RNA associated (S. cerevisiae) (LSM4), mRNA. [Source:RefSeq mRNA;Acc:NM_001035436]
paladin [Source:RefSeq peptide;Acc:NP_001193533]
Bos taurus glutathione S-transferase, theta 3 (GSTT3), mRNA. [Source:RefSeq mRNA;Acc:NM_001075738]
Bos taurus periphilin 1 (PPHLN1), mRNA. [Source:RefSeq mRNA;Acc:NM_001075972]
Uncharacterized protein [Source:UniProtKB/TrEMBL;Acc:F1MYN0]
WW domain binding protein 2 [Source:HGNC Symbol;Acc:HGNC:12738]
tripartite motif family-like 2 [Source:HGNC Symbol;Acc:HGNC:26378]
Bos taurus polymerase (RNA) I polypeptide D, 16kDa (POLR1D), mRNA. [Source:RefSeq mRNA;Acc:NM_001076144]
Uncharacterized protein [Source:UniProtKB/TrEMBL;Acc:E1BF89]
Bos taurus ubiquinol-cytochrome c reductase, complex III subunit XI (UQCR11), mRNA. [Source:RefSeq mRNA;Acc:NM_174215]
Bos taurus BCL2-related ovarian killer (BOK), mRNA. [Source:RefSeq mRNA;Acc:NM_001098868]
Bos taurus Sjogren syndrome antigen B (autoantigen La) (SSB), mRNA. [Source:RefSeq mRNA;Acc:NM_176665]
collagen and calcium binding EGF domains 1 [Source:HGNC Symbol;Acc:HGNC:29426]
neuronal calcium sensor 1 [Source:HGNC Symbol;Acc:HGNC:3953]
Bos taurus vasohibin 1 (VASH1), mRNA. [Source:RefSeq mRNA;Acc:NM_001206803]

oligodendrocyte transcription factor 3 [Source:HGNC Symbol;Acc:HGNC:18003]
NME/NM23 family member 5 [Source:HGNC Symbol;Acc:HGNC:7853]
Bos taurus uncharacterized protein CXorf64 homolog (CXHXorf64), mRNA. [Source:RefSeq mRNA;Acc:NM_001099188]
Bos taurus protease-associated domain containing 1 (PRADC1), mRNA. [Source:RefSeq mRNA;Acc:NM_001100338]
Bos taurus carbohydrate (chondroitin 4) sulfotransferase 13 (CHST13), mRNA. [Source:RefSeq mRNA;Acc:NM_001102079]
5-hydroxytryptamine (serotonin) receptor 7, adenylate cyclase-coupled [Source:HGNC Symbol;Acc:HGNC:5302]
Bos taurus high mobility group 20B (HMG20B), mRNA. [Source:RefSeq mRNA;Acc:NM_001038054]
Bos taurus F-box and leucine-rich repeat protein 22 (FBXL22), mRNA. [Source:RefSeq mRNA;Acc:NM_001046156]
Bos taurus apolipoprotein M (APOM), mRNA. [Source:RefSeq mRNA;Acc:NM_001034257]
galanin receptor 3 [Source:HGNC Symbol;Acc:HGNC:4134]
Uncharacterized protein [Source:UniProtKB/TrEMBL;Acc:F1MYQ4]
Bos taurus lymphocyte antigen 96 (LY96), mRNA. [Source:RefSeq mRNA;Acc:NM_001046517]
Bos taurus potassium voltage-gated channel, delayed-rectifier, subfamily S, member 1 (KCNS1), mRNA. [Source:RefSeq mRNA;Acc:NM_001205884]
olfactory receptor, family 52, subfamily W, member 1 [Source:HGNC Symbol;Acc:HGNC:15239]

Bos taurus bridging integrator 2 (BIN2), mRNA. [Source:RefSeq mRNA;Acc:NM_001083435]
Bos taurus gastrulation brain homeobox 2 (GBX2), mRNA. [Source:RefSeq mRNA;Acc:NM_001192145]
somatostatin receptor 3 [Source:HGNC Symbol;Acc:HGNC:11332]
transmembrane protein 98 [Source:HGNC Symbol;Acc:HGNC:24529]
Bos taurus serine dehydratase-like (SDSL), mRNA. [Source:RefSeq mRNA;Acc:NM_001098995]
Bos taurus mitochondrial ribosomal protein L50 (MRPL50), mRNA. [Source:RefSeq mRNA;Acc:NM_001046180]
Bos taurus pancreatic progenitor cell differentiation and proliferation factor homolog (zebrafish) (PPDPF), mRNA. [Source:RefSeq mRNA;Acc:NM_001075294]

transmembrane and tetratricopeptide repeat containing 3 [Source:HGNC Symbol;Acc:HGNC:26899]
olfactory receptor, family 4, subfamily K, member 5 [Source:HGNC Symbol;Acc:HGNC:14745]
zinc finger protein 699 [Source:HGNC Symbol;Acc:HGNC:24750]
Uncharacterized protein [Source:UniProtKB/TrEMBL;Acc:F1MXA6]
Bos taurus ATG3 autophagy related 3 homolog (S. cerevisiae) (ATG3), mRNA. [Source:RefSeq mRNA;Acc:NM_001075364]
Bos taurus guanine nucleotide binding protein (G protein), gamma 10 (GNG10), mRNA. [Source:RefSeq mRNA;Acc:NM_001114512]
Bos taurus stress-associated endoplasmic reticulum protein 1 (SERP1), mRNA. [Source:RefSeq mRNA;Acc:NM_001114083]

Bos taurus THO complex 7 homolog (Drosophila) (THOC7), mRNA. [Source:RefSeq mRNA;Acc:NM_001040514]
Bos taurus translocation associated membrane protein 2 (TRAM2), mRNA. [Source:RefSeq mRNA;Acc:NM_001193150]
Bos taurus fasciculation and elongation protein zeta 1 (zygin I) (FEZ1), mRNA. [Source:RefSeq mRNA;Acc:NM_001024522]
low density lipoprotein receptor class A domain containing 4 [Source:HGNC Symbol;Acc:HGNC:1224]
RNA (guanine-7-) methyltransferase [Source:HGNC Symbol;Acc:HGNC:10075]
Bos taurus calcium/calmodulin-dependent protein kinase II inhibitor 1 (CAMK2N1), mRNA. [Source:RefSeq mRNA;Acc:NM_001114520]
Bos taurus solute carrier family 15, member 4 (SLC15A4), mRNA. [Source:RefSeq mRNA;Acc:NM_001101927]
Bos taurus hydroxysteroid (17-beta) dehydrogenase 14 (HSD17B14), mRNA. [Source:RefSeq mRNA;Acc:NM_174701]
inositol polyphosphate-5-phosphatase, 40kDa [Source:HGNC Symbol;Acc:HGNC:6076]
Bos taurus presequence translocase-associated motor 16 homolog (S. cerevisiae) (PAM16), mRNA. [Source:RefSeq mRNA;Acc:NM_001002893]
Bos taurus forkhead box S1 (FOXS1), mRNA. [Source:RefSeq mRNA;Acc:NM_001099716]
Bos taurus ubiquitin-conjugating enzyme E2S (UBE2S), mRNA. [Source:RefSeq mRNA;Acc:NM_001076472]
Bos taurus apolipoprotein A-II (APOA2), mRNA. [Source:RefSeq mRNA;Acc:NM_001045916]

fibulin 7 [Source:HGNC Symbol;Acc:HGNC:26740]
Ras-related C3 botulinum toxin substrate 1 [Source:UniProtKB/Swiss-Prot;Acc:P62998]
Bos taurus dpy-30 homolog (C. elegans) (DPY30), mRNA. [Source:RefSeq mRNA;Acc:NM_001077916]
Bos taurus SUZ RNA binding domain containing 1 (SZRD1), transcript variant 1, mRNA. [Source:RefSeq mRNA;Acc:NM_001114608]
Bos taurus killer cell lectin-like receptor subfamily A, member 1 (KLRA1), mRNA. [Source:RefSeq mRNA;Acc:NM_174376]

Olfactory receptor [Source:UniProtKB/TrEMBL;Acc:G3X858]
Bos taurus mitochondrial translational release factor 1-like (MTRF1L), mRNA. [Source:RefSeq mRNA;Acc:NM_001046378]

Bos taurus degenerative spermatocyte homolog 2, lipid desaturase (Drosophila) (DEGS2), mRNA. [Source:RefSeq mRNA;Acc:NM_001076251]
Bos taurus programmed cell death 5 (PDCD5), mRNA. [Source:RefSeq mRNA;Acc:NM_001046024]
Bos taurus RER1 retention in endoplasmic reticulum 1 homolog (S. cerevisiae) (RER1), mRNA. [Source:RefSeq mRNA;Acc:NM_001105492]
tudor domain containing 3 [Source:HGNC Symbol;Acc:HGNC:20612]
archaelysin family metallopeptidase 2 [Source:HGNC Symbol;Acc:HGNC:28041]
Bos taurus uncharacterized LOC510904 (LOC510904), mRNA. [Source:RefSeq mRNA;Acc:NM_001103240]
Bos taurus Ras-related GTP binding C (RRAGC), mRNA. [Source:RefSeq mRNA;Acc:NM_001076456]

Bos taurus tetratricopeptide repeat domain 5 (TTC5), mRNA. [Source:RefSeq mRNA;Acc:NM_001075464]
poly (ADP-ribose) polymerase 2 [Source:HGNC Symbol;Acc:HGNC:272]
E2F transcription factor 5, p130-binding [Source:HGNC Symbol;Acc:HGNC:3119]
mbt domain containing 1 [Source:HGNC Symbol;Acc:HGNC:19866]
Bos taurus KIAA1704 ortholog (KIAA1704), mRNA. [Source:RefSeq mRNA;Acc:NM_001034580]
Bos taurus zinc finger, AN1-type domain 5 (ZFAND5), mRNA. [Source:RefSeq mRNA;Acc:NM_001101045]
Bos taurus polyamine-modulated factor 1 (PMF1), mRNA. [Source:RefSeq mRNA;Acc:NM_001038212]
protein arginine methyltransferase 8 [Source:HGNC Symbol;Acc:HGNC:5188]
Uncharacterized protein [Source:UniProtKB/TrEMBL;Acc:E1BE54]
Bos taurus family with sequence similarity 159, member B (FAM159B), mRNA. [Source:RefSeq mRNA;Acc:NM_001100373]
Bos taurus pirin (iron-binding nuclear protein) (PIR), mRNA. [Source:RefSeq mRNA;Acc:NM_001102358]
Cytochrome b-c1 complex subunit 8 [Source:UniProtKB/Swiss-Prot;Acc:P13271]
Bos taurus transmembrane protein 223 (TMEM223), mRNA. [Source:RefSeq mRNA;Acc:NM_001099721]
Uncharacterized protein [Source:UniProtKB/TrEMBL;Acc:F1MDX9]
Bos taurus NADH dehydrogenase (ubiquinone) 1 alpha subcomplex, 6, 14kDa (NDUFA6), mRNA. [Source:RefSeq mRNA;Acc:NM_175791]

wingless-type MMTV integration site family, member 7B [Source:HGNC Symbol;Acc:HGNC:12787]
C-type lectin domain family 9, member A [Source:HGNC Symbol;Acc:HGNC:26705]

Bos taurus zinc finger CCCH-type containing 10 (ZC3H10), mRNA. [Source:RefSeq mRNA;Acc:NM_001097993]
T cell receptor beta variable 30 (gene/pseudogene) [Source:HGNC Symbol;Acc:HGNC:12214]
chromosome 19 open reading frame 80 [Source:HGNC Symbol;Acc:HGNC:24933]
Bos taurus SH3 domain binding glutamic acid-rich protein like 3 (SH3BGRL3), mRNA. [Source:RefSeq mRNA;Acc:NM_001034763]
lipocalin 1 [Source:HGNC Symbol;Acc:HGNC:6525]
WD repeat domain 27 [Source:HGNC Symbol;Acc:HGNC:21248]
serine peptidase inhibitor, Kunitz type 4 [Source:HGNC Symbol;Acc:HGNC:16130]
A kinase (PRKA) anchor protein 5 [Source:HGNC Symbol;Acc:HGNC:375]
Uncharacterized protein [Source:UniProtKB/TrEMBL;Acc:F6PS28]
Bos taurus chromosome 3 open reading frame, human C1orf185 (C3H1orf185), mRNA. [Source:RefSeq mRNA;Acc:NM_001077079]

TBC1 domain family, member 12 [Source:HGNC Symbol;Acc:HGNC:29082]
interleukin 25 [Source:HGNC Symbol;Acc:HGNC:13765]
Bos taurus fructose-1,6-bisphosphatase 1 (FBP1), mRNA. [Source:RefSeq mRNA;Acc:NM_001034447]
Bos taurus xeroderma pigmentosum, complementation group A (XPA), mRNA. [Source:RefSeq mRNA;Acc:NM_001038681]
Bos taurus leukotriene C4 synthase (LTC4S), mRNA. [Source:RefSeq mRNA;Acc:NM_001046098]
Bos taurus retina and anterior neural fold homeobox 2 (RAX2), mRNA. [Source:RefSeq mRNA;Acc:NM_182653]
Uncharacterized protein [Source:UniProtKB/TrEMBL;Acc:E1B7Y5]
proline rich Gla (G-carboxyglutamic acid) 3 (transmembrane) [Source:HGNC Symbol;Acc:HGNC:30798]
Bos taurus chemokine (C-X-C motif) ligand 6 (granulocyte chemotactic protein 2) (CXCL6), mRNA. [Source:RefSeq mRNA;Acc:NM_174300]
scleraxis basic helix-loop-helix transcription factor [Source:HGNC Symbol;Acc:HGNC:32322]

Bos taurus ankyrin repeat and sterile alpha motif domain containing 4B (ANKS4B), mRNA. [Source:RefSeq mRNA;Acc:NM_001075192]
Bos taurus G protein-coupled receptor 171 (GPR171), mRNA. [Source:RefSeq mRNA;Acc:NM_001077002]
chromosome 14 open reading frame 105 [Source:HGNC Symbol;Acc:HGNC:20189]
KDEL (Lys-Asp-Glu-Leu) endoplasmic reticulum protein retention receptor 3 [Source:HGNC Symbol;Acc:HGNC:6306]

Bos taurus Spi-C transcription factor (Spi-1/PU.1 related) (SPIC), mRNA. [Source:RefSeq mRNA;Acc:NM_001076421]
Bos taurus NADH dehydrogenase (ubiquinone) Fe-S protein 6, 13kDa (NADH-coenzyme Q reductase) (NDUFS6), mRNA. [Source:RefSeq mRNA;Acc:NM_175810]
Bos taurus WDYHV motif containing 1 (WDYHV1), mRNA. [Source:RefSeq mRNA;Acc:NM_001035091]
Bos taurus phosphoglycolate phosphatase (PGP), mRNA. [Source:RefSeq mRNA;Acc:NM_001038172]
chromosome 2 open reading frame 69 [Source:HGNC Symbol;Acc:HGNC:26799]
Bos taurus centromere protein M (CENPM), mRNA. [Source:RefSeq mRNA;Acc:NM_001038686]
Bos taurus solute carrier family 6 (neurotransmitter transporter, creatine), member 8 (SLC6A8), mRNA. [Source:RefSeq mRNA;Acc:NM_174611]
Bos taurus enoyl-CoA delta isomerase 1 (ECI1), mRNA. [Source:RefSeq mRNA;Acc:NM_001046396]
Bos taurus X-ray repair complementing defective repair in Chinese hamster cells 3 (XRCC3), mRNA. [Source:RefSeq mRNA;Acc:NM_001078117]
pre-B-cell leukemia homeobox 4 [Source:HGNC Symbol;Acc:HGNC:13403]

calcium activated nucleotidase 1 [Source:HGNC Symbol;Acc:HGNC:19721]
Olfactory receptor [Source:UniProtKB/TrEMBL;Acc:F1MSZ3]

Bos taurus chromosome 16 open reading frame, human C1orf227 (C16H1orf227), mRNA. [Source:RefSeq mRNA;Acc:NM_001077024]

granzyme B (granzyme 2, cytotoxic T-lymphocyte-associated serine esterase 1) [Source:HGNC Symbol;Acc:HGNC:4709]
Bos taurus receptor accessory protein 2 (REEP2), mRNA. [Source:RefSeq mRNA;Acc:NM_001046325]
Bos taurus flotillin 2 (FLOT2), mRNA. [Source:RefSeq mRNA;Acc:NM_001035466]
Bos taurus hydroxyacyl-CoA dehydrogenase/3-ketoacyl-CoA thiolase/enoyl-CoA hydratase (trifunctional protein), beta subunit (HADHB), mRNA. [Source:RefSeq
Bos taurus solute carrier family 35, member G1 (SLC35G1), mRNA. [Source:RefSeq mRNA;Acc:NM_001076470]
Bos taurus apolipoprotein E (APOE), mRNA. [Source:RefSeq mRNA;Acc:NM_173991]
mitochondrial transcription termination factor 2 [Source:HGNC Symbol;Acc:HGNC:30779]
annexin A3 [Source:HGNC Symbol;Acc:HGNC:541]
Bos taurus tumor protein, translationally-controlled 1 (TPT1), mRNA. [Source:RefSeq mRNA;Acc:NM_001014388]
Uncharacterized protein [Source:UniProtKB/TrEMBL;Acc:F1MQ19]
collagen, type VIII, alpha 2 [Source:HGNC Symbol;Acc:HGNC:2216]
Bos taurus golgi transport 1A (GOLT1A), mRNA. [Source:RefSeq mRNA;Acc:NM_001075361]
Bos taurus GrpE-like 1, mitochondrial (E. coli) (GRPEL1), mRNA. [Source:RefSeq mRNA;Acc:NM_001034501]
Bos taurus polymerase (RNA) II (DNA directed) polypeptide D (POLR2D), mRNA. [Source:RefSeq mRNA;Acc:NM_001076458]
LIM homeobox transcription factor 1, beta [Source:HGNC Symbol;Acc:HGNC:6654]
Bos taurus NADH dehydrogenase (ubiquinone) Fe-S protein 5, 15kDa (NADH-coenzyme Q reductase) (NDUFS5), mRNA. [Source:RefSeq mRNA;Acc:NM_176652]
Bos taurus leukotriene B4 receptor (LTB4R), mRNA. [Source:RefSeq mRNA;Acc:NM_001034738]
chloride intracellular channel 5 [Source:HGNC Symbol;Acc:HGNC:13517]
fukutin [Source:HGNC Symbol;Acc:HGNC:3622]
Uncharacterized protein [Source:UniProtKB/TrEMBL;Acc:F6R9K7]
Uncharacterized protein [Source:UniProtKB/TrEMBL;Acc:E1BBG0]
epiregulin [Source:HGNC Symbol;Acc:HGNC:3443]
NK2 homeobox 2 [Source:HGNC Symbol;Acc:HGNC:7835]
par-3 family cell polarity regulator beta [Source:HGNC Symbol;Acc:HGNC:14446]
Bos taurus general transcription factor IIA, 2, 12kDa (GTF2A2), mRNA. [Source:RefSeq mRNA;Acc:NM_001037619]
Bos taurus transaldolase 1 (TALDO1), mRNA. [Source:RefSeq mRNA;Acc:NM_001035283]

Bos taurus vacuolar protein sorting 37 homolog A (S. cerevisiae) (VPS37A), mRNA. [Source:RefSeq mRNA;Acc:NM_001046161]
Bos taurus penta-EF-hand domain containing 1 (PEF1), mRNA. [Source:RefSeq mRNA;Acc:NM_001097992]
Bos taurus CREB regulated transcription coactivator 1 (CRTC1), mRNA. [Source:RefSeq mRNA;Acc:NM_001206319]
Bos taurus tRNA selenocysteine 1 associated protein 1 (TRNAU1AP), mRNA. [Source:RefSeq mRNA;Acc:NM_001075967]
Bos taurus thioredoxin domain containing 16 (TXNDC16), mRNA. [Source:RefSeq mRNA;Acc:NM_001205394]
Bos taurus goosecoid homeobox (GSC), mRNA. [Source:RefSeq mRNA;Acc:NM_001192386]
pantothenate kinase 1 [Source:HGNC Symbol;Acc:HGNC:8598]
deoxyribonuclease II beta [Source:HGNC Symbol;Acc:HGNC:28875]
Bos taurus lymphocyte-specific protein 1 (LSP1), mRNA. [Source:RefSeq mRNA;Acc:NM_001075374]
copine IV [Source:HGNC Symbol;Acc:HGNC:2317]
Uncharacterized protein [Source:UniProtKB/TrEMBL;Acc:F1N1W8]
ubiquinol-cytochrome c reductase complex assembly factor 3 [Source:HGNC Symbol;Acc:HGNC:34399]
tumor necrosis factor, alpha-induced protein 8-like 3 [Source:HGNC Symbol;Acc:HGNC:20620]
olfactory receptor, family 9, subfamily G, member 1 [Source:HGNC Symbol;Acc:HGNC:15319]
Bos taurus egl nine homolog 1 (C. elegans) (EGLN1), mRNA. [Source:RefSeq mRNA;Acc:NM_001206046]
Mitochondrial import inner membrane translocase subunit Tim9 [Source:UniProtKB/Swiss-Prot;Acc:Q2KIV2]
Bos taurus cyclin-dependent kinase 3 (CDK3), mRNA. [Source:RefSeq mRNA;Acc:NM_001099178]
Bos taurus islet amyloid polypeptide (IAPP), mRNA. [Source:RefSeq mRNA;Acc:NM_001195038]
Bos taurus cytochrome P450, family 1, subfamily B, polypeptide 1 (CYP1B1), mRNA. [Source:RefSeq mRNA;Acc:NM_001192294]
Bos taurus myosin phosphatase Rho interacting protein (MPRIP), mRNA. [Source:RefSeq mRNA;Acc:NM_001105627]
Bos taurus thioredoxin domain containing 8 (spermatozoa) (TXNDC8), mRNA. [Source:RefSeq mRNA;Acc:NM_001078160]
G protein-coupled receptor 33 (gene/pseudogene) [Source:HGNC Symbol;Acc:HGNC:4489]

eukaryotic translation initiation factor 5A2 [Source:HGNC Symbol;Acc:HGNC:3301]
Bos taurus prokineticin 1 (PROK1), mRNA. [Source:RefSeq mRNA;Acc:NM_001034018]
interleukin 17B [Source:HGNC Symbol;Acc:HGNC:5982]
acyl-CoA dehydrogenase, long chain [Source:HGNC Symbol;Acc:HGNC:88]
APH1B gamma secretase subunit [Source:HGNC Symbol;Acc:HGNC:24080]
V-set and immunoglobulin domain containing 8 [Source:HGNC Symbol;Acc:HGNC:32063]
Bos taurus ephrin-A4 (EFNA4), mRNA. [Source:RefSeq mRNA;Acc:NM_001101249]

RNA-binding protein 44 [Source:UniProtKB/Swiss-Prot;Acc:E1BC15]
leucine rich repeat containing 31 [Source:HGNC Symbol;Acc:HGNC:26261]
Uncharacterized protein [Source:UniProtKB/TrEMBL;Acc:F1MB63]
epithelial discoidin domain-containing receptor 1 precursor [Source:RefSeq peptide;Acc:NP_001069480]
Bos taurus pancreatic and duodenal homeobox 1 (PDX1), mRNA. [Source:RefSeq mRNA;Acc:NM_001192136]
BicC family RNA binding protein 1 [Source:HGNC Symbol;Acc:HGNC:19351]
Bos taurus leucine rich repeat containing 4 (LRRC4), mRNA. [Source:RefSeq mRNA;Acc:NM_001014938]

Bos taurus prefoldin subunit 6 (PFDN6), mRNA. [Source:RefSeq mRNA;Acc:NM_001075421]

Uncharacterized protein [Source:UniProtKB/TrEMBL;Acc:G3MXD6]
Bos taurus HIG1 hypoxia inducible domain family, member 2A (HIGD2A), mRNA. [Source:RefSeq mRNA;Acc:NM_001077861]
Bos taurus clathrin, light chain B (CLTB), mRNA. [Source:RefSeq mRNA;Acc:NM_174277]
Bos taurus kelch repeat and BTB (POZ) domain containing 10 (KBTBD10), mRNA. [Source:RefSeq mRNA;Acc:NM_001083377]
Opa interacting protein 5 [Source:HGNC Symbol;Acc:HGNC:20300]
tubulin polyglutamylase complex subunit 1 [Source:HGNC Symbol;Acc:HGNC:25058]
Bos taurus activator of basal transcription 1 (ABT1), mRNA. [Source:RefSeq mRNA;Acc:NM_001076152]
Bos taurus replication factor C (activator 1) 3, 38kDa (RFC3), mRNA. [Source:RefSeq mRNA;Acc:NM_001038547]
protein phosphatase, Mg2+/Mn2+ dependent, 1E [Source:HGNC Symbol;Acc:HGNC:19322]
Bos taurus spermadhesin 1 (SPADH1), mRNA. [Source:RefSeq mRNA;Acc:NM_174616]
Bos taurus chromosome 26 open reading frame, human C10orf120 (C26H10orf120), mRNA. [Source:RefSeq mRNA;Acc:NM_001076476]
solute carrier family 23 (ascorbic acid transporter), member 1 [Source:HGNC Symbol;Acc:HGNC:10974]
Uncharacterized protein [Source:UniProtKB/TrEMBL;Acc:E1B9D4]
Bos taurus deiodinase, iodothyronine, type I (DIO1), mRNA. [Source:RefSeq mRNA;Acc:NM_001122593]
Bos taurus alkaline ceramidase 2 (ACER2), mRNA. [Source:RefSeq mRNA;Acc:NM_001192775]
Transcription factor COE2 [Source:UniProtKB/TrEMBL;Acc:F1N3R1]
Bos taurus forkhead box N4 (FOXN4), mRNA. [Source:RefSeq mRNA;Acc:NM_001205815]
ankyrin repeat domain 23 [Source:HGNC Symbol;Acc:HGNC:24470]
basic helix-loop-helix family, member e23 [Source:HGNC Symbol;Acc:HGNC:16093]
Bos taurus growth hormone secretagogue receptor (GHSR), mRNA. [Source:RefSeq mRNA;Acc:NM_001143736]

armadillo repeat containing 12 [Source:HGNC Symbol;Acc:HGNC:21099]
Bos taurus troponin I type 2 (skeletal, fast) (TNNI2), mRNA. [Source:RefSeq mRNA;Acc:NM_001192094]
A kinase (PRKA) anchor protein 14 [Source:HGNC Symbol;Acc:HGNC:24061]
ankyrin repeat domain 9 [Source:HGNC Symbol;Acc:HGNC:20096]
Bos taurus follicle stimulating hormone, beta polypeptide (FSHB), mRNA. [Source:RefSeq mRNA;Acc:NM_174060]
Bos taurus NK3 homeobox 2 (NKX3-2), mRNA. [Source:RefSeq mRNA;Acc:NM_001193133]
Bos taurus solute carrier family 30 (zinc transporter), member 6 (SLC30A6), mRNA. [Source:RefSeq mRNA;Acc:NM_001075766]
Bos taurus solute carrier family 30 (zinc transporter), member 1 (SLC30A1), mRNA. [Source:RefSeq mRNA;Acc:NM_001205893]
Bos taurus RAB9A, member RAS oncogene family (RAB9A), mRNA. [Source:RefSeq mRNA;Acc:NM_001075538]
cysteine-serine-rich nuclear protein 2 [Source:HGNC Symbol;Acc:HGNC:16006]
neuropeptide FF receptor 1 [Source:HGNC Symbol;Acc:HGNC:17425]
Bos taurus chromosome 2 open reading frame, human C1orf64 (C2H1orf64), mRNA. [Source:RefSeq mRNA;Acc:NM_001205359]
Bos taurus chromosome X open reading frame, human CXorf58 (CXHXorf58), mRNA. [Source:RefSeq mRNA;Acc:NM_001102023]
heat shock 27kDa protein family, member 7 (cardiovascular) [Source:HGNC Symbol;Acc:HGNC:5249]
Bos taurus chloride intracellular channel 2 (CLIC2), mRNA. [Source:RefSeq mRNA;Acc:NM_001081727]
nucleoporin 54kDa [Source:RefSeq peptide;Acc:NP_001068753]
Bos taurus histidine triad nucleotide binding protein 1 (HINT1), mRNA. [Source:RefSeq mRNA;Acc:NM_175812]
Bos taurus StAR-related lipid transfer (START) domain containing 5 (STARD5), mRNA. [Source:RefSeq mRNA;Acc:NM_001080265]
Bos taurus chromosome 28 open reading frame, human C10orf107 (C28H10orf107), transcript variant 2, mRNA. [Source:RefSeq mRNA;Acc:NM_001105618]
Uncharacterized protein [Source:UniProtKB/TrEMBL;Acc:E1BM31]
calcium and integrin binding family member 2 [Source:HGNC Symbol;Acc:HGNC:24579]
CDKN2A interacting protein N-terminal like [Source:HGNC Symbol;Acc:HGNC:30545]
Bos taurus transthyretin (TTR), mRNA. [Source:RefSeq mRNA;Acc:NM_173967]
Bos taurus cyclin-dependent kinase 2 interacting protein (CINP), mRNA. [Source:RefSeq mRNA;Acc:NM_001099125]
carbohydrate (chondroitin 6) sulfotransferase 3 [Source:HGNC Symbol;Acc:HGNC:1971]
Bos taurus mitochondrial ribosomal protein S36 (MRPS36), mRNA. [Source:RefSeq mRNA;Acc:NM_001035438]
Bos taurus gametogenetin binding protein 1 (GGNBP1), mRNA. [Source:RefSeq mRNA;Acc:NM_001098993]
Bos taurus cyclin-dependent kinase inhibitor 2C (p18, inhibits CDK4) (CDKN2C), mRNA. [Source:RefSeq mRNA;Acc:NM_001101054]
Bos taurus T-cell leukemia homeobox 1 (TLX1), mRNA. [Source:RefSeq mRNA;Acc:NM_001192057]
Bos taurus ERGIC and golgi 2 (ERGIC2), mRNA. [Source:RefSeq mRNA;Acc:NM_001075563]

Tctex1 domain containing 1 [Source:HGNC Symbol;Acc:HGNC:26882]

Bos taurus progestin and adipoQ receptor family member IX (PAQR9), mRNA. [Source:RefSeq mRNA;Acc:NM_001077951]
Uncharacterized protein [Source:UniProtKB/TrEMBL;Acc:E1BNV9]
gastrulation brain homeobox 1 [Source:HGNC Symbol;Acc:HGNC:4185]
Bos taurus coiled-coil domain containing 83 (CCDC83), mRNA. [Source:RefSeq mRNA;Acc:NM_001038218]
Bos taurus adaptor-related protein complex 1, sigma 3 subunit (AP1S3), mRNA. [Source:RefSeq mRNA;Acc:NM_001103296]
Bos taurus unconventional SNARE in the ER 1 homolog (S. cerevisiae) (USE1), mRNA. [Source:RefSeq mRNA;Acc:NM_001075584]
nuclear receptor subfamily 2, group F, member 6 [Source:HGNC Symbol;Acc:HGNC:7977]
Bos taurus MORN repeat containing 4 (MORN4), mRNA. [Source:RefSeq mRNA;Acc:NM_001035450]
Bos taurus coiled-coil domain containing 58 (CCDC58), mRNA. [Source:RefSeq mRNA;Acc:NM_001083389]
Bos taurus butyrylcholinesterase (BCHE), mRNA. [Source:RefSeq mRNA;Acc:NM_001076906]
Uncharacterized protein [Source:UniProtKB/TrEMBL;Acc:G5E5E7]
Bos taurus NADH dehydrogenase (ubiquinone) 1 alpha subcomplex, 4, 9kDa (NDUFA4), mRNA. [Source:RefSeq mRNA;Acc:NM_175820]

cytokine-like 1 [Source:HGNC Symbol;Acc:HGNC:24435]
Olfactory receptor [Source:UniProtKB/TrEMBL;Acc:F1N236]
inositol polyphosphate multikinase [Source:HGNC Symbol;Acc:HGNC:20739]
chromosome 9 open reading frame 89 [Source:HGNC Symbol;Acc:HGNC:28148]
Bos taurus eukaryotic translation initiation factor 3, subunit K (EIF3K), mRNA. [Source:RefSeq mRNA;Acc:NM_001034489]
Bos taurus THAP domain containing, apoptosis associated protein 3 (THAP3), mRNA. [Source:RefSeq mRNA;Acc:NM_001075347]
Bos taurus syntaxin 11 (STX11), mRNA. [Source:RefSeq mRNA;Acc:NM_001205736]
olfactory receptor, family 2, subfamily Z, member 1 [Source:HGNC Symbol;Acc:HGNC:15391]
Bos taurus eukaryotic translation initiation factor 6 (EIF6), mRNA. [Source:RefSeq mRNA;Acc:NM_174830]
Bos taurus zinc finger and BTB domain containing 16 (ZBTB16), mRNA. [Source:RefSeq mRNA;Acc:NM_001037476]
Bos taurus clarin 1 (CLRN1), mRNA. [Source:RefSeq mRNA;Acc:NM_001192101]
sorting nexin 31 [Source:HGNC Symbol;Acc:HGNC:28605]
family with sequence similarity 133, member A [Source:HGNC Symbol;Acc:HGNC:26748]

NK2 homeobox 4 [Source:HGNC Symbol;Acc:HGNC:7837]

family with sequence similarity 58, member A [Source:HGNC Symbol;Acc:HGNC:28434]
ecotropic viral integration site 5 [Source:HGNC Symbol;Acc:HGNC:3501]
Bos taurus lin-7 homolog A (C. elegans) (LIN7A), mRNA. [Source:RefSeq mRNA;Acc:NM_001079602]

proline rich 7 (synaptic) [Source:HGNC Symbol;Acc:HGNC:28130]
Bos taurus signaling threshold regulating transmembrane adaptor 1 (SIT1), mRNA. [Source:RefSeq mRNA;Acc:NM_001206418]
Bos taurus coiled-coil domain containing 107 (CCDC107), mRNA. [Source:RefSeq mRNA;Acc:NM_001075681]
Bos taurus serpin peptidase inhibitor, clade I (pancpin), member 2 (SERPINI2), mRNA. [Source:RefSeq mRNA;Acc:NM_001102054]
Bos taurus histidine triad nucleotide binding protein 2 (HINT2), mRNA. [Source:RefSeq mRNA;Acc:NM_174340]
Bos taurus kelch-like 11 (Drosophila) (KLHL11), mRNA. [Source:RefSeq mRNA;Acc:NM_001205574]
basic leucine zipper transcription factor, ATF-like 2 [Source:HGNC Symbol;Acc:HGNC:25163]
Kazal-type serine peptidase inhibitor domain 1 [Source:HGNC Symbol;Acc:HGNC:25460]
Olfactory receptor [Source:UniProtKB/TrEMBL;Acc:F1MQN3]
5', 3'-nucleotidase, cytosolic [Source:HGNC Symbol;Acc:HGNC:17144]
Bos taurus solute carrier family 22, member 31 (SLC22A31), mRNA. [Source:RefSeq mRNA;Acc:NM_001101901]

Bos taurus surfeit 6 (SURF6), mRNA. [Source:RefSeq mRNA;Acc:NM_001075529]
serine/threonine kinase 32C [Source:HGNC Symbol;Acc:HGNC:21332]
Ectonucleotide pyrophosphatase/phosphodiesterase family member 6 [Source:UniProtKB/Swiss-Prot;Acc:F1N5C8]
RFNG O-fucosylpeptide 3-beta-N-acetylglucosaminyltransferase [Source:HGNC Symbol;Acc:HGNC:9974]
Bos taurus serine incorporator 2 (SERINC2), mRNA. [Source:RefSeq mRNA;Acc:NM_001035285]
Bos taurus RAS protein activator like 3 (RASAL3), mRNA. [Source:RefSeq mRNA;Acc:NM_001102236]
Bos taurus oligosaccharyltransferase complex subunit (OSTC), mRNA. [Source:RefSeq mRNA;Acc:NM_001077092]
fibroblast growth factor 21 [Source:HGNC Symbol;Acc:HGNC:3678]
tyrosine 3-monooxygenase/tryptophan 5-monooxygenase activation protein, eta [Source:HGNC Symbol;Acc:HGNC:12853]
Bos taurus coiled-coil-helix-coiled-coil-helix domain containing 5 (CHCHD5), mRNA. [Source:RefSeq mRNA;Acc:NM_001037601]
Uncharacterized protein [Source:UniProtKB/TrEMBL;Acc:F1N7E4]
Bos taurus SRY (sex determining region Y)-box 18 (SOX18), mRNA. [Source:RefSeq mRNA;Acc:NM_001075789]
Bos taurus thyroid hormone responsive (THRSP), mRNA. [Source:RefSeq mRNA;Acc:NM_001040533]

leukocyte receptor cluster (LRC) member 9 [Source:HGNC Symbol;Acc:HGNC:16306]
ectonucleoside triphosphate diphosphohydrolase 3 [Source:HGNC Symbol;Acc:HGNC:3365]
Uncharacterized protein [Source:UniProtKB/TrEMBL;Acc:E1BHM9]
Bos taurus hydroxysteroid (17-beta) dehydrogenase 6 homolog (mouse) (HSD17B6), mRNA. [Source:RefSeq mRNA;Acc:NM_001035395]
glutamate receptor, ionotropic kainate 5 [Source:RefSeq peptide;Acc:NP_001039889]
Bos taurus Ewing tumor-associated antigen 1 (ETAA1), mRNA. [Source:RefSeq mRNA;Acc:NM_001075128]
Bos taurus phenylethanolamine N-methyltransferase (PNMT), mRNA. [Source:RefSeq mRNA;Acc:NM_177505]
Uncharacterized protein [Source:UniProtKB/TrEMBL;Acc:E1BMY8]
Bos taurus complement component 1, q subcomponent-like 1 (C1QL1), mRNA. [Source:RefSeq mRNA;Acc:NM_001205862]

FIC domain containing [Source:HGNC Symbol;Acc:HGNC:18416]
Bos taurus brain protein 44-like protein 2 (LOC520682), mRNA. [Source:RefSeq mRNA;Acc:NM_001075809]
Bos taurus DIM1 dimethyladenosine transferase 1 homolog (S. cerevisiae) (DIMT1), mRNA. [Source:RefSeq mRNA;Acc:NM_001046036]
gamma-aminobutyric acid (GABA) A receptor, alpha 2 [Source:HGNC Symbol;Acc:HGNC:4076]
[truncated: 370,056 more chars]
